# Supplementary material for: Optimized workplace risk mitigation measures for SARS-CoV-2 in 2022
Source: Sci Rep. 2023 Feb 16;13:2779. doi: 10.1038/s41598-023-29087-w (PMC9933026; doi:10.1038/s41598-023-29087-w)
Supplement: Supplementary file 1 — Supplementary Information. [file 41598_2023_29087_MOESM1_ESM.pdf]

Table 3. Results of 133,056 Parameter Iterating Simulations

Community Infection Rate (New Daily Cases Per 100,000)	Variant	Non Pharmaceutical Intervention	Size Of Production	Test Name	Test Frequency	Vaccination Efficacy At Preventing Transmission	Vaccination Coverage (%)	Scale Of Testing	Number Of Tests Per Person Per Week	Community Acquired Infections Per Person Per Week	Workplace Acquired Infections Per Person Per Week	Total Acquired Infections Per Person Per Week	Symptomatic Workforce Per Person Per Week	Infections Detected Per Person Per Week	False Positive Tests Per Person Per Week	Number Of Tests 60 Persons 8 Weeks	Community Acquired Infections 60 Persons 8 Weeks	Workplace Acquired Infections 60 Persons 8 Weeks	Total Acquired Infections 60 Persons 8 Weeks	Symptomatic Workforce 60 Persons 8 Weeks	Infections Detected 60 Persons 8 Weeks	False Positive Tests 60 Persons 8 Weeks	Number Of Tests 200 Persons 24 Weeks	Community Acquired Infections 200 Persons 24 Weeks	Workplace Acquired Infections 200 Persons 24 Weeks	Total Acquired Infections 200 Persons 24 Weeks	Symptomatic Workforce 200 Persons 24 Weeks	Infections Detected 200 Persons 24 Weeks	False Positive Tests 200 Persons 24 Weeks
150	original	0.6	60x8 weeks	PCR90	Daily	NOVAC	0	All	4.910	0.010	0.003	0.013	0.001	0.013	0.005	2356.976	4.976	1.305	6.281	0.380	6.145	2.271	23570	49.760	13.050	62.810	3.800	61.450	22.710
150	original	0.6	60x8 weeks	PCR90	Daily	NOVAC	0	All	4.910	0.010	0.003	0.013	0.001	0.013	0.005	2356.976	4.976	1.305	6.281	0.380	6.145	2.271	23570	49.760	13.050	62.810	3.800	61.450	22.710
150	original	0.6	60x8 weeks	PCR90	Daily	NOVAC	0	All	4.910	0.010	0.003	0.013	0.001	0.013	0.005	2356.976	4.976	1.305	6.281	0.380	6.145	2.271	23570	49.760	13.050	62.810	3.800	61.450	22.710
150	original	0.6	60x8 weeks	PCR90	Daily	NOVAC	0	All	4.910	0.010	0.003	0.013	0.001	0.013	0.005	2356.976	4.976	1.305	6.281	0.380	6.145	2.271	23570	49.760	13.050	62.810	3.800	61.450	22.710
150	original	0.6	60x8 weeks	PCR90	Daily	NOVAC	0	All	4.910	0.010	0.003	0.013	0.001	0.013	0.005	2356.976	4.976	1.305	6.281	0.380	6.145	2.271	23570	49.760	13.050	62.810	3.800	61.450	22.710
150	original	0.6	60x8 weeks	PCR90	Daily	NOVAC	0	All	4.910	0.010	0.003	0.013	0.001	0.013	0.005	2356.976	4.976	1.305	6.281	0.380	6.145	2.271	23570	49.760	13.050	62.810	3.800	61.450	22.710
150	original	1	60x8 weeks	PCR90	Daily	NOVAC	0	All	4.895	0.010	0.006	0.015	0.001	0.015	0.005	2349.708	4.616	2.673	7.289	0.496	7.151	2.214	23497	46.160	26.730	72.890	4.960	71.510	22.140
150	original	1	60x8 weeks	PCR90	Daily	NOVAC	0	All	4.895	0.010	0.006	0.015	0.001	0.015	0.005	2349.708	4.616	2.673	7.289	0.496	7.151	2.214	23497	46.160	26.730	72.890	4.960	71.510	22.140
150	original	1	60x8 weeks	PCR90	Daily	NOVAC	0	All	4.895	0.010	0.006	0.015	0.001	0.015	0.005	2349.708	4.616	2.673	7.289	0.496	7.151	2.214	23497	46.160	26.730	72.890	4.960	71.510	22.140
150	original	1.2	60x8 weeks	PCR90	Daily	NOVAC	0	All	4.880	0.010	0.007	0.017	0.001	0.017	0.005	2342.247	4.742	3.561	8.303	0.559	8.209	2.183	23422	47.420	35.610	83.030	5.590	82.090	21.830
150	original	1.2	60x8 weeks	PCR90	Daily	NOVAC	0	All	4.880	0.010	0.007	0.017	0.001	0.017	0.005	2342.247	4.742	3.561	8.303	0.559	8.209	2.183	23422	47.420	35.610	83.030	5.590	82.090	21.830
150	original	1.2	60x8 weeks	PCR90	Daily	NOVAC	0	All	4.880	0.010	0.007	0.017	0.001	0.017	0.005	2342.247	4.742	3.561	8.303	0.559	8.209	2.183	23422	47.420	35.610	83.030	5.590	82.090	21.830
150	alpha	0.6	60x8 weeks	PCR90	Daily	NOVAC	0	All	4.882	0.010	0.007	0.016	0.001	0.017	0.005	2343.541	4.621	3.233	7.854	0.569	7.968	2.233	23435	46.210	32.330	78.540	5.690	79.680	22.330
150	alpha	0.6	60x8 weeks	PCR90	Daily	NOVAC	0	All	4.882	0.010	0.007	0.016	0.001	0.017	0.005	2343.541	4.621	3.233	7.854	0.569	7.968	2.233	23435	46.210	32.330	78.540	5.690	79.680	22.330
150	alpha	0.6	60x8 weeks	PCR90	Daily	NOVAC	0	All	4.882	0.010	0.007	0.016	0.001	0.017	0.005	2343.541	4.621	3.233	7.854	0.569	7.968	2.233	23435	46.210	32.330	78.540	5.690	79.680	22.330
150	alpha	1	60x8 weeks	PCR90	Daily	NOVAC	0	All	4.811	0.009	0.017	0.027	0.002	0.027	0.005	2309.115	4.554	8.175	12.729	0.867	12.879	2.169	23091	45.540	81.750	127.290	8.670	128.790	21.690
150	alpha	1	60x8 weeks	PCR90	Daily	NOVAC	0	All	4.811	0.009	0.017	0.027	0.002	0.027	0.005	2309.115	4.554	8.175	12.729	0.867	12.879	2.169	23091	45.540	81.750	127.290	8.670	128.790	21.690
150	alpha	1	60x8 weeks	PCR90	Daily	NOVAC	0	All	4.811	0.009	0.017	0.027	0.002	0.027	0.005	2309.115	4.554	8.175	12.729	0.867	12.879	2.169	23091	45.540	81.750	127.290	8.670	128.790	21.690
150	alpha	1.2	60x8 weeks	PCR90	Daily	NOVAC	0	All	4.742	0.009	0.027	0.036	0.002	0.036	0.004	2275.999	4.304	12.858	17.162	1.190	17.484	2.016	22760	43.040	128.580	171.620	11.900	174.840	20.160
150	alpha	1.2	60x8 weeks	PCR90	Daily	NOVAC	0	All	4.742	0.009	0.027	0.036	0.002	0.036	0.004	2275.999	4.304	12.858	17.162	1.190	17.484	2.016	22760	43.040	128.580	171.620	11.900	174.840	20.160
150	alpha	1.2	60x8 weeks	PCR90	Daily	NOVAC	0	All	4.742	0.009	0.027	0.036	0.002	0.036	0.004	2275.999	4.304	12.858	17.162	1.190	17.484	2.016	22760	43.040	128.580	171.620	11.900	174.840	20.160
150	delta	0.6	60x8 weeks	PCR90	Daily	NOVAC	0	All	4.685	0.009	0.035	0.044	0.005	0.043	0.004	2248.658	4.229	16.756	20.985	2.181	20.513	1.995	22487	42.290	167.560	209.850	21.810	205.130	19.950
150	delta	0.6	60x8 weeks	PCR90	Daily	NOVAC	0	All	4.685	0.009	0.035	0.044	0.005	0.043	0.004	2248.658	4.229	16.756	20.985	2.181	20.513	1.995	22487	42.290	167.560	209.850	21.810	205.130	19.950
150	delta	0.6	60x8 weeks	PCR90	Daily	NOVAC	0	All	4.685	0.009	0.035	0.044	0.005	0.043	0.004	2248.658	4.229	16.756	20.985	2.181	20.513	1.995	22487	42.290	167.560	209.850	21.810	205.130	19.950
150	delta	1	60x8 weeks	PCR90	Daily	NOVAC	0	All	4.257	0.006	0.092	0.097	0.010	0.097	0.003	2043.506	2.798	43.966	46.764	4.990	46.579	1.331	20435	27.980	439.660	467.640	49.900	465.790	13.310
150	delta	1	60x8 weeks	PCR90	Daily	NOVAC	0	All	4.257	0.006	0.092	0.097	0.010	0.097	0.003	2043.506	2.798	43.966	46.764	4.990	46.579	1.331	20435	27.980	439.660	467.640	49.900	465.790	13.310
150	delta	1	60x8 weeks	PCR90	Daily	NOVAC	0	All	4.257	0.006	0.092	0.097	0.010	0.097	0.003	2043.506	2.798	43.966	46.764	4.990	46.579	1.331	20435	27.980	439.660	467.640	49.900	465.790	13.310
150	delta	1.2	60x8 weeks	PCR90	Daily	NOVAC	0	All	4.129	0.005	0.107	0.112	0.012	0.112	0.002	1981.981	2.440	51.367	53.807	5.875	53.904	1.153	19820	24.400	513.670	538.070	58.750	539.040	11.530
150	delta	1.2	60x8 weeks	PCR90	Daily	NOVAC	0	All	4.129	0.005	0.107	0.112	0.012	0.112	0.002	1981.981	2.440	51.367	53.807	5.875	53.904	1.153	19820	24.400	513.670	538.070	58.750	539.040	11.530
150	delta	1.2	60x8 weeks	PCR90	Daily	NOVAC	0	All	4.129	0.005	0.107	0.112	0.012	0.112	0.002	1981.981	2.440	51.367	53.807	5.875	53.904	1.153	19820	24.400	513.670	538.070	58.750	539.040	11.530
150	original	0.6	60x8 weeks	PCR90	Daily	VAC95	25	All	4.937	0.008	0.001	0.009	0.001	0.009	0.005	2369.886	3.684	0.699	4.383	0.300	4.251	2.273	23699	36.840	6.990	43.830	3.000	42.510	22.730
150	original	0.6	60x8 weeks	PCR90	Daily	VAC95	25	All	4.937	0.008	0.001	0.009	0.001	0.009	0.005	2369.886	3.684	0.699	4.383	0.300	4.251	2.273	23699	36.840	6.990	43.830	3.000	42.510	22.730
150	original	0.6	60x8 weeks	PCR90	Daily	VAC95	25	All	4.937	0.008	0.001	0.009	0.001	0.009	0.005	2369.886	3.684	0.699	4.383	0.300	4.251	2.273	23699	36.840	6.990	43.830	3.000	42.510	22.730
150	original	1	60x8 weeks	PCR90	Daily	VAC95	25	All	4.928	0.008	0.003	0.011	0.001	0.010	0.005	2365.331	3.692	1.396	5.088	0.360	4.881	2.258	23653	36.920	13.960	50.880	3.600	48.810	22.580
150	original	1	60x8 weeks	PCR90	Daily	VAC95	25	All	4.928	0.008	0.003	0.011	0.001	0.010	0.005	2365.331	3.692	1.396	5.088	0.360	4.881	2.258	23653	36.920	13.960	50.880	3.600	48.810	22.580
150	original	1	60x8 weeks	PCR90	Daily	VAC95	25	All	4.928	0.008	0.003	0.011	0.001	0.010	0.005	2365.331	3.692	1.396	5.088	0.360	4.881	2.258	23653	36.920	13.960	50.880	3.600	48.810	22.580
150	original	1.2	60x8 weeks	PCR90	Daily	VAC95	25	All	4.922	0.008	0.004	0.012	0.001	0.011	0.005	2362.402	3.781	1.752	5.533	0.393	5.264	2.322	23624	37.810	17.520	55.330	3.930	52.640	23.220
150	original	1.2	60x8 weeks	PCR90	Daily	VAC95	25	All	4.922	0.008	0.004	0.012	0.001	0.011	0.005	2362.402	3.781	1.752	5.533	0.393	5.264	2.322	23624	37.810	17.520	55.330	3.930	52.640	23.220
150	original	1.2	60x8 weeks	PCR90	Daily	VAC95	25	All	4.922	0.008	0.004	0.012	0.001	0.011	0.005	2362.402	3.781	1.752	5.533	0.393	5.264	2.322	23624	37.810	17.520	55.330	3.930	52.640	23.220
150	alpha	0.6	60x8 weeks	PCR90	Daily	VAC95	25	All	4.923	0.008	0.003	0.011	0.001	0.011	0.005	2363.103	3.647	1.588	5.235	0.381	5.229	2.326	23631	36.470	15.880	52.350	3.810	52.290	23.260
150	alpha	0.6	60x8 weeks	PCR90	Daily	VAC95	25	All	4.923	0.008	0.003	0.011	0.001	0.011	0.005	2363.103	3.647	1.588	5.235	0.381	5.229	2.326	23631	36.470	15.880	52.350	3.810	52.290	23.260
150	alpha	0.6	60x8 weeks	PCR90	Daily	VAC95	25	All	4.923	0.008	0.003	0.011	0.001	0.011	0.005	2363.103	3.647	1.588	5.235	0.381	5.229	2.326	23631	36.470	15.880	52.350	3.810	52.290	23.260
150	alpha	1	60x8 weeks	PCR90	Daily	VAC95	25	All	4.894	0.008	0.007	0.015	0.001	0.015	0.005	2349.335	3.652	3.463	7.115	0.501	7.201	2.236	23493	36.520	34.630	71.150	5.010	72.010	22.360
150	alpha	1	60x8 weeks	PCR90	Daily	VAC95	25	All	4.894	0.008	0.007	0.015	0.001	0.015	0.005	2349.335	3.652	3.463	7.115	0.501	7.201	2.236	23493	36.520	34.630	71.150	5.010	72.010	22.360
150	alpha	1	60x8 weeks	PCR90	Daily	VAC95	25	All	4.894	0.008	0.007	0.015	0.001	0.015	0.005	2349.335	3.652	3.463	7.115	0.501	7.201	2.236	23493	36.520	34.630	71.150	5.010	72.010	22.360
150	alpha	1.2	60x8 weeks	PCR90	Daily	VAC95	25	All	4.875	0.007	0.010	0.018	0.001	0.018	0.005	2340.043	3.497	4.977	8.474	0.598	8.519	2.250	23400	34.970	49.770	84.740	5.980	85.190	22.500
150	alpha	1.2	60x8 weeks	PCR90	Daily	VAC95	25	All	4.875	0.007	0.010	0.018	0.001	0.018	0.005	2340.043	3.497	4.977	8.474	0.598	8.519	2.250	23400	34.970	49.770	84.740	5.980	85.190	22.500
150	alpha	1.2	60x8 weeks	PCR90	Daily	VAC95	25	All	4.875	0.007	0.010	0.018	0.001	0.018	0.005	2340.043	3.497	4.977	8.474	0.598	8.519	2.250	23400	34.970	49.770	84.740	5.980	85.190	22.500
150	delta	0.6	60x8 weeks	PCR90	Daily	VAC95	25	All	4.856	0.007	0.013	0.021	0.002	0.020	0.005	2330.945	3.579	6.289	9.868	0.976	9.598	2.166	23309	35.790	62.890	98.680	9.760	95.980	21.660
150	delta	0.6	60x8 weeks	PCR90	Daily	VAC95	25	All	4.856	0.007	0.013	0.021	0.002	0.020	0.005	2330.945	3.579	6.289	9.868	0.976	9.598	2.166	23309	35.790	62.890	98.680	9.760	95.980	21.660
150	delta	0.6	60x8 weeks	PCR90	Daily	VAC95	25	All	4.856	0.007	0.013	0.021	0.002	0.020	0.005	2330.945	3.579	6.289	9.868	0.976	9.598	2.166	23309	35.790	62.890	98.680	9.760	95.980	21.660
150	delta	1	60x8 weeks	PCR90	Daily	VAC95	25	All	4.653	0.006	0.041	0.047	0.005	0.047	0.004	2233.422	3.077	19.684	22.761	2.289	22.413	1.875	22334	30.770	196.840	227.610	22.890	224.130	18.750
150	delta	1	60x8 weeks	PCR90	Daily	VAC95	25	All	4.653	0.006	0.041	0.047	0.005	0.047	0.004	2233.422	3.077	19.684	22.761	2.289	22.413	1.875	22334	30.770	196.840	227.610	22.890	224.130	18.750
150	delta	1	60x8 weeks	PCR90	Daily	VAC95	25	All	4.653	0.006	0.041	0.047	0.005	0.047	0.004	2233.422	3.077	19.684	22.761	2.289	22.413	1.875	22334	30.770	196.840	227.610	22.890	224.130	18.750
150	delta	1.2	60x8 weeks	PCR90	Daily	VAC95	25	All	4.514	0.006	0.060	0.065	0.007	0.065	0.004	2166.825	2.650	28.754	31.404	3.177	31.081	1.726	21668	26.500	287.540	314.040	31.770	310.810	17.260
150	delta	1.2	60x8 weeks	PCR90	Daily	VAC95	25	All	4.514	0.006	0.060	0.065	0.007	0.065	0.004	2166.825	2.650	28.754	31.404	3.177	31.081	1.726	21668	26.500	287.540	314.040	31.770	310.810	17.260
150	delta	1.2	60x8 weeks	PCR90	Daily	VAC95	25	All	4.514	0.006	0.060	0.065	0.007	0.065	0.004	2166.825	2.650	28.754	31.404	3.177	31.081	1.726	21668	26.500	287.540	314.040	31.770	310.810	17.260
150	original	0.6	60x8 weeks	PCR90	Daily	VAC95	50	All	4.960	0.006	0.001	0.006	0.000	0.006	0.005	2380.844	2.643	0.288	2.931	0.233	2.684	2.295	23808	26.430	2.880	29.310	2.330	26.840	22.950

150	original	0.6	60x8 weeks	PCR90	Daily	VAC95	50	All	4.960	0.006	0.001	0.006	0.000	0.006	0.005	2380.844	2.643	0.288	2.931	0.233	2.684	2.295	23808	26.430	2.880	29.310	2.330	26.840	22.950
150	original	0.6	60x8 weeks	PCR90	Daily	VAC95	50	All	4.960	0.006	0.001	0.006	0.000	0.006	0.005	2380.844	2.643	0.288	2.931	0.233	2.684	2.295	23808	26.430	2.880	29.310	2.330	26.840	22.950
150	original	1	60x8 weeks	PCR90	Daily	VAC95	50	All	4.958	0.005	0.001	0.006	0.001	0.006	0.005	2379.786	2.560	0.537	3.097	0.266	2.838	2.339	23798	25.600	5.370	30.970	2.660	28.380	23.390
150	original	1	60x8 weeks	PCR90	Daily	VAC95	50	All	4.958	0.005	0.001	0.006	0.001	0.006	0.005	2379.786	2.560	0.537	3.097	0.266	2.838	2.339	23798	25.600	5.370	30.970	2.660	28.380	23.390
150	original	1	60x8 weeks	PCR90	Daily	VAC95	50	All	4.958	0.005	0.001	0.006	0.001	0.006	0.005	2379.786	2.560	0.537	3.097	0.266	2.838	2.339	23798	25.600	5.370	30.970	2.660	28.380	23.390
150	original	1.2	60x8 weeks	PCR90	Daily	VAC95	50	All	4.955	0.005	0.001	0.007	0.001	0.006	0.005	2378.283	2.531	0.715	3.246	0.271	3.033	2.242	23783	25.310	7.150	32.460	2.710	30.330	22.420
150	original	1.2	60x8 weeks	PCR90	Daily	VAC95	50	All	4.955	0.005	0.001	0.007	0.001	0.006	0.005	2378.283	2.531	0.715	3.246	0.271	3.033	2.242	23783	25.310	7.150	32.460	2.710	30.330	22.420
150	original	1.2	60x8 weeks	PCR90	Daily	VAC95	50	All	4.955	0.005	0.001	0.007	0.001	0.006	0.005	2378.283	2.531	0.715	3.246	0.271	3.033	2.242	23783	25.310	7.150	32.460	2.710	30.330	22.420
150	alpha	0.6	60x8 weeks	PCR90	Daily	VAC95	50	All	4.952	0.005	0.001	0.007	0.000	0.007	0.005	2376.831	2.624	0.670	3.294	0.230	3.296	2.371	23768	26.240	6.700	32.940	2.300	32.960	23.710
150	alpha	0.6	60x8 weeks	PCR90	Daily	VAC95	50	All	4.952	0.005	0.001	0.007	0.000	0.007	0.005	2376.831	2.624	0.670	3.294	0.230	3.296	2.371	23768	26.240	6.700	32.940	2.300	32.960	23.710
150	alpha	0.6	60x8 weeks	PCR90	Daily	VAC95	50	All	4.952	0.005	0.001	0.007	0.000	0.007	0.005	2376.831	2.624	0.670	3.294	0.230	3.296	2.371	23768	26.240	6.700	32.940	2.300	32.960	23.710
150	alpha	1	60x8 weeks	PCR90	Daily	VAC95	50	All	4.941	0.005	0.003	0.008	0.001	0.008	0.005	2371.826	2.604	1.415	4.019	0.312	3.977	2.234	23718	26.040	14.150	40.190	3.120	39.770	22.340
150	alpha	1	60x8 weeks	PCR90	Daily	VAC95	50	All	4.941	0.005	0.003	0.008	0.001	0.008	0.005	2371.826	2.604	1.415	4.019	0.312	3.977	2.234	23718	26.040	14.150	40.190	3.120	39.770	22.340
150	alpha	1	60x8 weeks	PCR90	Daily	VAC95	50	All	4.941	0.005	0.003	0.008	0.001	0.008	0.005	2371.826	2.604	1.415	4.019	0.312	3.977	2.234	23718	26.040	14.150	40.190	3.120	39.770	22.340
150	alpha	1.2	60x8 weeks	PCR90	Daily	VAC95	50	All	4.935	0.006	0.004	0.009	0.001	0.009	0.005	2368.579	2.685	1.817	4.502	0.273	4.544	2.273	23686	26.850	18.170	45.020	2.730	45.440	22.730
150	alpha	1.2	60x8 weeks	PCR90	Daily	VAC95	50	All	4.935	0.006	0.004	0.009	0.001	0.009	0.005	2368.579	2.685	1.817	4.502	0.273	4.544	2.273	23686	26.850	18.170	45.020	2.730	45.440	22.730
150	alpha	1.2	60x8 weeks	PCR90	Daily	VAC95	50	All	4.935	0.006	0.004	0.009	0.001	0.009	0.005	2368.579	2.685	1.817	4.502	0.273	4.544	2.273	23686	26.850	18.170	45.020	2.730	45.440	22.730
150	delta	0.6	60x8 weeks	PCR90	Daily	VAC95	50	All	4.926	0.006	0.005	0.011	0.001	0.010	0.005	2364.598	2.739	2.334	5.073	0.480	4.891	2.293	23646	27.390	23.340	50.730	4.800	48.910	22.930
150	delta	0.6	60x8 weeks	PCR90	Daily	VAC95	50	All	4.926	0.006	0.005	0.011	0.001	0.010	0.005	2364.598	2.739	2.334	5.073	0.480	4.891	2.293	23646	27.390	23.340	50.730	4.800	48.910	22.930
150	delta	0.6	60x8 weeks	PCR90	Daily	VAC95	50	All	4.926	0.006	0.005	0.011	0.001	0.010	0.005	2364.598	2.739	2.334	5.073	0.480	4.891	2.293	23646	27.390	23.340	50.730	4.800	48.910	22.930
150	delta	1	60x8 weeks	PCR90	Daily	VAC95	50	All	4.875	0.005	0.012	0.018	0.002	0.017	0.005	2340.138	2.562	5.852	8.414	0.829	8.160	2.222	23401	25.620	58.520	84.140	8.290	81.600	22.220
150	delta	1	60x8 weeks	PCR90	Daily	VAC95	50	All	4.875	0.005	0.012	0.018	0.002	0.017	0.005	2340.138	2.562	5.852	8.414	0.829	8.160	2.222	23401	25.620	58.520	84.140	8.290	81.600	22.220
150	delta	1	60x8 weeks	PCR90	Daily	VAC95	50	All	4.875	0.005	0.012	0.018	0.002	0.017	0.005	2340.138	2.562	5.852	8.414	0.829	8.160	2.222	23401	25.620	58.520	84.140	8.290	81.600	22.220
150	delta	1.2	60x8 weeks	PCR90	Daily	VAC95	50	All	4.839	0.005	0.018	0.023	0.002	0.022	0.005	2322.864	2.456	8.411	10.867	1.106	10.574	2.220	23229	24.560	84.110	108.670	11.060	105.740	22.200
150	delta	1.2	60x8 weeks	PCR90	Daily	VAC95	50	All	4.839	0.005	0.018	0.023	0.002	0.022	0.005	2322.864	2.456	8.411	10.867	1.106	10.574	2.220	23229	24.560	84.110	108.670	11.060	105.740	22.200
150	delta	1.2	60x8 weeks	PCR90	Daily	VAC95	50	All	4.839	0.005	0.018	0.023	0.002	0.022	0.005	2322.864	2.456	8.411	10.867	1.106	10.574	2.220	23229	24.560	84.110	108.670	11.060	105.740	22.200
150	original	0.6	60x8 weeks	PCR90	Daily	VAC95	75	All	4.980	0.003	0.000	0.003	0.000	0.003	0.005	2390.402	1.446	0.087	1.533	0.167	1.296	2.422	23904	14.460	0.870	15.330	1.670	12.960	24.220
150	original	0.6	60x8 weeks	PCR90	Daily	VAC95	75	All	4.980	0.003	0.000	0.003	0.000	0.003	0.005	2390.402	1.446	0.087	1.533	0.167	1.296	2.422	23904	14.460	0.870	15.330	1.670	12.960	24.220
150	original	0.6	60x8 weeks	PCR90	Daily	VAC95	75	All	4.980	0.003	0.000	0.003	0.000	0.003	0.005	2390.402	1.446	0.087	1.533	0.167	1.296	2.422	23904	14.460	0.870	15.330	1.670	12.960	24.220
150	original	1	60x8 weeks	PCR90	Daily	VAC95	75	All	4.980	0.003	0.000	0.003	0.000	0.003	0.005	2390.264	1.430	0.118	1.548	0.135	1.337	2.356	23903	14.300	1.180	15.480	1.350	13.370	23.560
150	original	1	60x8 weeks	PCR90	Daily	VAC95	75	All	4.980	0.003	0.000	0.003	0.000	0.003	0.005	2390.264	1.430	0.118	1.548	0.135	1.337	2.356	23903	14.300	1.180	15.480	1.350	13.370	23.560
150	original	1	60x8 weeks	PCR90	Daily	VAC95	75	All	4.980	0.003	0.000	0.003	0.000	0.003	0.005	2390.264	1.430	0.118	1.548	0.135	1.337	2.356	23903	14.300	1.180	15.480	1.350	13.370	23.560
150	original	1.2	60x8 weeks	PCR90	Daily	VAC95	75	All	4.978	0.003	0.000	0.003	0.000	0.003	0.005	2389.601	1.475	0.201	1.676	0.203	1.362	2.382	23896	14.750	2.010	16.760	2.030	13.620	23.820
150	original	1.2	60x8 weeks	PCR90	Daily	VAC95	75	All	4.978	0.003	0.000	0.003	0.000	0.003	0.005	2389.601	1.475	0.201	1.676	0.203	1.362	2.382	23896	14.750	2.010	16.760	2.030	13.620	23.820
150	original	1.2	60x8 weeks	PCR90	Daily	VAC95	75	All	4.978	0.003	0.000	0.003	0.000	0.003	0.005	2389.601	1.475	0.201	1.676	0.203	1.362	2.382	23896	14.750	2.010	16.760	2.030	13.620	23.820
150	alpha	0.6	60x8 weeks	PCR90	Daily	VAC95	75	All	4.975	0.003	0.000	0.004	0.000	0.004	0.005	2387.824	1.537	0.211	1.748	0.115	1.739	2.359	23878	15.370	2.110	17.480	1.150	17.390	23.590
150	alpha	0.6	60x8 weeks	PCR90	Daily	VAC95	75	All	4.975	0.003	0.000	0.004	0.000	0.004	0.005	2387.824	1.537	0.211	1.748	0.115	1.739	2.359	23878	15.370	2.110	17.480	1.150	17.390	23.590
150	alpha	0.6	60x8 weeks	PCR90	Daily	VAC95	75	All	4.975	0.003	0.000	0.004	0.000	0.004	0.005	2387.824	1.537	0.211	1.748	0.115	1.739	2.359	23878	15.370	2.110	17.480	1.150	17.390	23.590
150	alpha	1	60x8 weeks	PCR90	Daily	VAC95	75	All	4.972	0.003	0.001	0.004	0.000	0.004	0.005	2386.666	1.491	0.385	1.876	0.142	1.887	2.347	23867	14.910	3.850	18.760	1.420	18.870	23.470
150	alpha	1	60x8 weeks	PCR90	Daily	VAC95	75	All	4.972	0.003	0.001	0.004	0.000	0.004	0.005	2386.666	1.491	0.385	1.876	0.142	1.887	2.347	23867	14.910	3.850	18.760	1.420	18.870	23.470
150	alpha	1	60x8 weeks	PCR90	Daily	VAC95	75	All	4.972	0.003	0.001	0.004	0.000	0.004	0.005	2386.666	1.491	0.385	1.876	0.142	1.887	2.347	23867	14.910	3.850	18.760	1.420	18.870	23.470
150	alpha	1.2	60x8 weeks	PCR90	Daily	VAC95	75	All	4.971	0.003	0.001	0.004	0.000	0.004	0.005	2386.206	1.576	0.443	2.019	0.143	1.962	2.366	23862	15.760	4.430	20.190	1.430	19.620	23.660
150	alpha	1.2	60x8 weeks	PCR90	Daily	VAC95	75	All	4.971	0.003	0.001	0.004	0.000	0.004	0.005	2386.206	1.576	0.443	2.019	0.143	1.962	2.366	23862	15.760	4.430	20.190	1.430	19.620	23.660
150	alpha	1.2	60x8 weeks	PCR90	Daily	VAC95	75	All	4.971	0.003	0.001	0.004	0.000	0.004	0.005	2386.206	1.576	0.443	2.019	0.143	1.962	2.366	23862	15.760	4.430	20.190	1.430	19.620	23.660
150	delta	0.6	60x8 weeks	PCR90	Daily	VAC95	75	All	4.967	0.003	0.001	0.005	0.000	0.005	0.005	2384.082	1.660	0.597	2.257	0.217	2.188	2.380	23841	16.600	5.970	22.570	2.170	21.880	23.800
150	delta	0.6	60x8 weeks	PCR90	Daily	VAC95	75	All	4.967	0.003	0.001	0.005	0.000	0.005	0.005	2384.082	1.660	0.597	2.257	0.217	2.188	2.380	23841	16.600	5.970	22.570	2.170	21.880	23.800
150	delta	0.6	60x8 weeks	PCR90	Daily	VAC95	75	All	4.967	0.003	0.001	0.005	0.000	0.005	0.005	2384.082	1.660	0.597	2.257	0.217	2.188	2.380	23841	16.600	5.970	22.570	2.170	21.880	23.800
150	delta	1	60x8 weeks	PCR90	Daily	VAC95	75	All	4.954	0.004	0.003	0.007	0.001	0.006	0.005	2377.733	1.795	1.388	3.183	0.306	3.070	2.321	23777	17.950	13.880	31.830	3.060	30.700	23.210
150	delta	1	60x8 weeks	PCR90	Daily	VAC95	75	All	4.954	0.004	0.003	0.007	0.001	0.006	0.005	2377.733	1.795	1.388	3.183	0.306	3.070	2.321	23777	17.950	13.880	31.830	3.060	30.700	23.210
150	delta	1	60x8 weeks	PCR90	Daily	VAC95	75	All	4.954	0.004	0.003	0.007	0.001	0.006	0.005	2377.733	1.795	1.388	3.183	0.306	3.070	2.321	23777	17.950	13.880	31.830	3.060	30.700	23.210
150	delta	1.2	60x8 weeks	PCR90	Daily	VAC95	75	All	4.950	0.004	0.004	0.007	0.001	0.007	0.005	2376.059	1.687	1.785	3.472	0.337	3.344	2.327	23761	16.870	17.850	34.720	3.370	33.440	23.270
150	delta	1.2	60x8 weeks	PCR90	Daily	VAC95	75	All	4.950	0.004	0.004	0.007	0.001	0.007	0.005	2376.059	1.687	1.785	3.472	0.337	3.344	2.327	23761	16.870	17.850	34.720	3.370	33.440	23.270
150	delta	1.2	60x8 weeks	PCR90	Daily	VAC95	75	All	4.950	0.004	0.004	0.007	0.001	0.007	0.005	2376.059	1.687	1.785	3.472	0.337	3.344	2.327	23761	16.870	17.850	34.720	3.370	33.440	23.270
150	original	0.6	60x8 weeks	PCR90	Daily	VAC95	100	All	4.998	0.001	0.000	0.001	0.000	0.000	0.005	2399.011	0.333	0.000	0.333	0.077	0.075	2.371	23990	3.330	0.000	3.330	0.770	0.750	23.710
150	original	0.6	60x8 weeks	PCR90	Daily	VAC95	100	All	4.998	0.001	0.000	0.001	0.000	0.000	0.005	2399.011	0.333	0.000	0.333	0.077	0.075	2.371	23990	3.330	0.000	3.330	0.770	0.750	23.710
150	original	0.6	60x8 weeks	PCR90	Daily	VAC95	100	All	4.998	0.001	0.000	0.001	0.000	0.000	0.005	2399.011	0.333	0.000	0.333	0.077	0.075	2.371	23990	3.330	0.000	3.330	0.770	0.750	23.710
150	original	1	60x8 weeks	PCR90	Daily	VAC95	100	All	4.998	0.001	0.000	0.001	0.000	0.000	0.005	2398.902	0.372	0.003	0.375	0.105	0.070	2.453	23989	3.720	0.030	3.750	1.050	0.700	24.530
150	original	1	60x8 weeks	PCR90	Daily	VAC95	100	All	4.998	0.001	0.000	0.001	0.000	0.000	0.005	2398.902	0.372	0.003	0.375	0.105	0.070	2.453	23989	3.720	0.030	3.750	1.050	0.700	24.530
150	original	1	60x8 weeks	PCR90	Daily	VAC95	100	All	4.998	0.001	0.000	0.001	0.000	0.000	0.005	2398.902	0.372	0.003	0.375	0.105	0.070	2.453	23989	3.720	0.030	3.750	1.050	0.700	24.530
150	original	1.2	60x8 weeks	PCR90	Daily	VAC95	100	All	4.997	0.001	0.000	0.001	0.000	0.000	0.005	2398.789	0.368	0.001	0.369	0.104	0.080	2.407	23988	3.680	0.010	3.690	1.040	0.800	24.070
150	original	1.2	60x8 weeks	PCR90	Daily	VAC95	100	All	4.997	0.001	0.000	0.001	0.000	0.000	0.005	2398.789	0.368	0.001	0.369	0.104	0.080	2.407	23988	3.680	0.010	3.690	1.040	0.800	24.070
150	original	1.2	60x8 weeks	PCR90	Daily	VAC95	100	All	4.997	0.001	0.000	0.001	0.000	0.000	0.005	2398.789	0.368	0.001	0.369	0.104	0.080	2.407	23988	3.680	0.010	3.690	1.040	0.800	24.070
150	alpha	0.6	60x8 weeks	PCR90	Daily	VAC95	100	All	4.993	0.001	0.000	0.001	0.000	0.001	0.005	2396.715	0.469	0.011	0.480	0.030	0.469	2.414	23967	4.690	0.110	4.800	0.300	4.690	24.140
150	alpha	0.6	60x8 weeks	PCR90	Daily	VAC95	100	All	4.993	0.001	0.000	0.001	0.000	0.001	0.005	2396.715	0.469	0.011	0.480	0.030	0.469	2.414	23967	4.690	0.110	4.800	0.300	4.690	24.140
150	alpha	0.6	60x8 weeks	PCR90	Daily	VAC95	100	All	4.993	0.001	0.000	0.001	0.000	0.001	0.005	2396.715	0.469	0.011	0.480	0.030	0.469	2.414	23967	4.690	0.110	4.800	0.300	4.690	24.140
150	alpha	1	60x8 weeks	PCR90	Daily	VAC95	100	All	4.993	0.001	0.000	0.001	0.000	0.001	0.005	2396.719	0.466	0.014	0.480	0.019	0.482	2.460	23967	4.660	0.140	4.800	0.190	4.820	24.600
150	alpha	1	60x8 weeks	PCR90	Daily	VAC95	100	All	4.993	0.001	0.000	0.001	0.000	0.001	0.005	2396.719	0.466	0.014	0.480	0.019	0.482	2.460	23967	4.660	0.140	4.800	0.190	4.820	24.600
150	alpha	1	60x8 weeks	PCR90	Daily	VAC95	100	All	4.993	0.001	0.000	0.001	0.000	0.001	0.005	2396.719	0.466	0.014	0.480	0.019	0.482	2.460	23967	4.660	0.140	4.800	0.190	4.820	24.600
150	alpha	1.2	60x8 weeks	PCR90	Daily	VAC95	100	All	4.993	0.001	0.000	0.001	0.000	0.001	0.005	2396.809	0.452	0.021	0.473	0.028	0.459	2.394	23968	4.520	0.210	4.730	0.280	4.590	23.940
150	alpha	1.2	60x8 weeks	PCR90	Daily	VAC95	100	All	4.993	0.001	0.000	0.001	0.000	0.001	0.005	2396.809	0.452	0.021	0.473	0.028	0.459	2.394	23968	4.520	0.210	4.730	0.280	4.590	23.940
150	alpha	1.2	60x8 weeks	PCR90	Daily	VAC95	100	All	4.993	0.001	0.000	0.001	0.000	0.001	0.005	2396.809	0.452	0.021	0.473	0.028	0.459	2.394	23968	4.520	0.210	4.730	0.280	4.590	23.940

150	delta	0.6	60x8 weeks	PCR90	Daily	VAC95	100	All	4.989	0.001	0.000	0.002	0.000	0.001	0.005	2394.929	0.704	0.036	0.740	0.063	0.699	2.325	23949	7.040	0.360	7.400	0.630	6.990	23.250
150	delta	0.6	60x8 weeks	PCR90	Daily	VAC95	100	All	4.989	0.001	0.000	0.002	0.000	0.001	0.005	2394.929	0.704	0.036	0.740	0.063	0.699	2.325	23949	7.040	0.360	7.400	0.630	6.990	23.250
150	delta	0.6	60x8 weeks	PCR90	Daily	VAC95	100	All	4.989	0.001	0.000	0.002	0.000	0.001	0.005	2394.929	0.704	0.036	0.740	0.063	0.699	2.325	23949	7.040	0.360	7.400	0.630	6.990	23.250
150	delta	1	60x8 weeks	PCR90	Daily	VAC95	100	All	4.988	0.002	0.000	0.002	0.000	0.002	0.005	2394.431	0.726	0.085	0.811	0.057	0.794	2.348	23944	7.260	0.850	8.110	0.570	7.940	23.480
150	delta	1	60x8 weeks	PCR90	Daily	VAC95	100	All	4.988	0.002	0.000	0.002	0.000	0.002	0.005	2394.431	0.726	0.085	0.811	0.057	0.794	2.348	23944	7.260	0.850	8.110	0.570	7.940	23.480
150	delta	1	60x8 weeks	PCR90	Daily	VAC95	100	All	4.988	0.002	0.000	0.002	0.000	0.002	0.005	2394.431	0.726	0.085	0.811	0.057	0.794	2.348	23944	7.260	0.850	8.110	0.570	7.940	23.480
150	delta	1.2	60x8 weeks	PCR90	Daily	VAC95	100	All	4.988	0.002	0.000	0.002	0.000	0.002	0.005	2394.260	0.722	0.113	0.835	0.079	0.788	2.378	23943	7.220	1.130	8.350	0.790	7.880	23.780
150	delta	1.2	60x8 weeks	PCR90	Daily	VAC95	100	All	4.988	0.002	0.000	0.002	0.000	0.002	0.005	2394.260	0.722	0.113	0.835	0.079	0.788	2.378	23943	7.220	1.130	8.350	0.790	7.880	23.780
150	delta	1.2	60x8 weeks	PCR90	Daily	VAC95	100	All	4.988	0.002	0.000	0.002	0.000	0.002	0.005	2394.260	0.722	0.113	0.835	0.079	0.788	2.378	23943	7.220	1.130	8.350	0.790	7.880	23.780
150	original	0.6	60x8 weeks	PCR90	Daily	VAC70	25	All	4.933	0.009	0.002	0.010	0.001	0.009	0.005	2368.051	4.091	0.812	4.903	0.466	4.400	2.346	23681	40.910	8.120	49.030	4.660	44.000	23.460
150	original	0.6	60x8 weeks	PCR90	Daily	VAC70	25	All	4.933	0.009	0.002	0.010	0.001	0.009	0.005	2368.051	4.091	0.812	4.903	0.466	4.400	2.346	23681	40.910	8.120	49.030	4.660	44.000	23.460
150	original	0.6	60x8 weeks	PCR90	Daily	VAC70	25	All	4.933	0.009	0.002	0.010	0.001	0.009	0.005	2368.051	4.091	0.812	4.903	0.466	4.400	2.346	23681	40.910	8.120	49.030	4.660	44.000	23.460
150	original	1	60x8 weeks	PCR90	Daily	VAC70	25	All	4.925	0.008	0.003	0.011	0.001	0.010	0.005	2364.027	3.951	1.535	5.486	0.525	4.975	2.287	23640	39.510	15.350	54.860	5.250	49.750	22.870
150	original	1	60x8 weeks	PCR90	Daily	VAC70	25	All	4.925	0.008	0.003	0.011	0.001	0.010	0.005	2364.027	3.951	1.535	5.486	0.525	4.975	2.287	23640	39.510	15.350	54.860	5.250	49.750	22.870
150	original	1	60x8 weeks	PCR90	Daily	VAC70	25	All	4.925	0.008	0.003	0.011	0.001	0.010	0.005	2364.027	3.951	1.535	5.486	0.525	4.975	2.287	23640	39.510	15.350	54.860	5.250	49.750	22.870
150	original	1.2	60x8 weeks	PCR90	Daily	VAC70	25	All	4.919	0.008	0.004	0.012	0.001	0.011	0.005	2361.003	3.992	1.896	5.888	0.580	5.309	2.245	23610	39.920	18.960	58.880	5.800	53.090	22.450
150	original	1.2	60x8 weeks	PCR90	Daily	VAC70	25	All	4.919	0.008	0.004	0.012	0.001	0.011	0.005	2361.003	3.992	1.896	5.888	0.580	5.309	2.245	23610	39.920	18.960	58.880	5.800	53.090	22.450
150	original	1.2	60x8 weeks	PCR90	Daily	VAC70	25	All	4.919	0.008	0.004	0.012	0.001	0.011	0.005	2361.003	3.992	1.896	5.888	0.580	5.309	2.245	23610	39.920	18.960	58.880	5.800	53.090	22.450
150	alpha	0.6	60x8 weeks	PCR90	Daily	VAC70	25	All	4.914	0.008	0.004	0.012	0.001	0.012	0.005	2358.727	3.968	1.835	5.803	0.400	5.829	2.252	23587	39.680	18.350	58.030	4.000	58.290	22.520
150	alpha	0.6	60x8 weeks	PCR90	Daily	VAC70	25	All	4.914	0.008	0.004	0.012	0.001	0.012	0.005	2358.727	3.968	1.835	5.803	0.400	5.829	2.252	23587	39.680	18.350	58.030	4.000	58.290	22.520
150	alpha	0.6	60x8 weeks	PCR90	Daily	VAC70	25	All	4.914	0.008	0.004	0.012	0.001	0.012	0.005	2358.727	3.968	1.835	5.803	0.400	5.829	2.252	23587	39.680	18.350	58.030	4.000	58.290	22.520
150	alpha	1	60x8 weeks	PCR90	Daily	VAC70	25	All	4.877	0.008	0.009	0.017	0.001	0.017	0.005	2340.721	3.871	4.434	8.305	0.584	8.382	2.195	23407	38.710	44.340	83.050	5.840	83.820	21.950
150	alpha	1	60x8 weeks	PCR90	Daily	VAC70	25	All	4.877	0.008	0.009	0.017	0.001	0.017	0.005	2340.721	3.871	4.434	8.305	0.584	8.382	2.195	23407	38.710	44.340	83.050	5.840	83.820	21.950
150	alpha	1	60x8 weeks	PCR90	Daily	VAC70	25	All	4.877	0.008	0.009	0.017	0.001	0.017	0.005	2340.721	3.871	4.434	8.305	0.584	8.382	2.195	23407	38.710	44.340	83.050	5.840	83.820	21.950
150	alpha	1.2	60x8 weeks	PCR90	Daily	VAC70	25	All	4.855	0.008	0.012	0.020	0.001	0.021	0.005	2330.436	3.816	5.937	9.753	0.678	9.892	2.191	23304	38.160	59.370	97.530	6.780	98.920	21.910
150	alpha	1.2	60x8 weeks	PCR90	Daily	VAC70	25	All	4.855	0.008	0.012	0.020	0.001	0.021	0.005	2330.436	3.816	5.937	9.753	0.678	9.892	2.191	23304	38.160	59.370	97.530	6.780	98.920	21.910
150	alpha	1.2	60x8 weeks	PCR90	Daily	VAC70	25	All	4.855	0.008	0.012	0.020	0.001	0.021	0.005	2330.436	3.816	5.937	9.753	0.678	9.892	2.191	23304	38.160	59.370	97.530	6.780	98.920	21.910
150	delta	0.6	60x8 weeks	PCR90	Daily	VAC70	25	All	4.827	0.008	0.017	0.025	0.002	0.024	0.005	2317.030	3.858	7.968	11.826	1.156	11.481	2.244	23170	38.580	79.680	118.260	11.560	114.810	22.440
150	delta	0.6	60x8 weeks	PCR90	Daily	VAC70	25	All	4.827	0.008	0.017	0.025	0.002	0.024	0.005	2317.030	3.858	7.968	11.826	1.156	11.481	2.244	23170	38.580	79.680	118.260	11.560	114.810	22.440
150	delta	0.6	60x8 weeks	PCR90	Daily	VAC70	25	All	4.827	0.008	0.017	0.025	0.002	0.024	0.005	2317.030	3.858	7.968	11.826	1.156	11.481	2.244	23170	38.580	79.680	118.260	11.560	114.810	22.440
150	delta	1	60x8 weeks	PCR90	Daily	VAC70	25	All	4.559	0.007	0.054	0.060	0.006	0.059	0.004	2188.218	3.231	25.708	28.939	2.969	28.394	1.899	21882	32.310	257.080	289.390	29.690	283.940	18.990
150	delta	1	60x8 weeks	PCR90	Daily	VAC70	25	All	4.559	0.007	0.054	0.060	0.006	0.059	0.004	2188.218	3.231	25.708	28.939	2.969	28.394	1.899	21882	32.310	257.080	289.390	29.690	283.940	18.990
150	delta	1	60x8 weeks	PCR90	Daily	VAC70	25	All	4.559	0.007	0.054	0.060	0.006	0.059	0.004	2188.218	3.231	25.708	28.939	2.969	28.394	1.899	21882	32.310	257.080	289.390	29.690	283.940	18.990
150	delta	1.2	60x8 weeks	PCR90	Daily	VAC70	25	All	4.380	0.006	0.078	0.084	0.009	0.082	0.003	2102.291	2.755	37.337	40.092	4.170	39.515	1.507	21023	27.550	373.370	400.920	41.700	395.150	15.070
150	delta	1.2	60x8 weeks	PCR90	Daily	VAC70	25	All	4.380	0.006	0.078	0.084	0.009	0.082	0.003	2102.291	2.755	37.337	40.092	4.170	39.515	1.507	21023	27.550	373.370	400.920	41.700	395.150	15.070
150	delta	1.2	60x8 weeks	PCR90	Daily	VAC70	25	All	4.380	0.006	0.078	0.084	0.009	0.082	0.003	2102.291	2.755	37.337	40.092	4.170	39.515	1.507	21023	27.550	373.370	400.920	41.700	395.150	15.070
150	original	0.6	60x8 weeks	PCR90	Daily	VAC70	50	All	4.955	0.007	0.001	0.008	0.001	0.006	0.005	2378.195	3.228	0.379	3.607	0.497	2.866	2.291	23782	32.280	3.790	36.070	4.970	28.660	22.910
150	original	0.6	60x8 weeks	PCR90	Daily	VAC70	50	All	4.955	0.007	0.001	0.008	0.001	0.006	0.005	2378.195	3.228	0.379	3.607	0.497	2.866	2.291	23782	32.280	3.790	36.070	4.970	28.660	22.910
150	original	0.6	60x8 weeks	PCR90	Daily	VAC70	50	All	4.955	0.007	0.001	0.008	0.001	0.006	0.005	2378.195	3.228	0.379	3.607	0.497	2.866	2.291	23782	32.280	3.790	36.070	4.970	28.660	22.910
150	original	1	60x8 weeks	PCR90	Daily	VAC70	50	All	4.950	0.007	0.002	0.008	0.001	0.006	0.005	2376.137	3.244	0.724	3.968	0.569	3.084	2.363	23761	32.440	7.240	39.680	5.690	30.840	23.630
150	original	1	60x8 weeks	PCR90	Daily	VAC70	50	All	4.950	0.007	0.002	0.008	0.001	0.006	0.005	2376.137	3.244	0.724	3.968	0.569	3.084	2.363	23761	32.440	7.240	39.680	5.690	30.840	23.630
150	original	1	60x8 weeks	PCR90	Daily	VAC70	50	All	4.950	0.007	0.002	0.008	0.001	0.006	0.005	2376.137	3.244	0.724	3.968	0.569	3.084	2.363	23761	32.440	7.240	39.680	5.690	30.840	23.630
150	original	1.2	60x8 weeks	PCR90	Daily	VAC70	50	All	4.949	0.006	0.002	0.008	0.001	0.007	0.005	2375.292	3.097	0.914	4.011	0.565	3.170	2.269	23753	30.970	9.140	40.110	5.650	31.700	22.690
150	original	1.2	60x8 weeks	PCR90	Daily	VAC70	50	All	4.949	0.006	0.002	0.008	0.001	0.007	0.005	2375.292	3.097	0.914	4.011	0.565	3.170	2.269	23753	30.970	9.140	40.110	5.650	31.700	22.690
150	original	1.2	60x8 weeks	PCR90	Daily	VAC70	50	All	4.949	0.006	0.002	0.008	0.001	0.007	0.005	2375.292	3.097	0.914	4.011	0.565	3.170	2.269	23753	30.970	9.140	40.110	5.650	31.700	22.690
150	alpha	0.6	60x8 weeks	PCR90	Daily	VAC70	50	All	4.938	0.007	0.002	0.009	0.001	0.009	0.005	2370.421	3.177	1.005	4.182	0.286	4.193	2.281	23704	31.770	10.050	41.820	2.860	41.930	22.810
150	alpha	0.6	60x8 weeks	PCR90	Daily	VAC70	50	All	4.938	0.007	0.002	0.009	0.001	0.009	0.005	2370.421	3.177	1.005	4.182	0.286	4.193	2.281	23704	31.770	10.050	41.820	2.860	41.930	22.810
150	alpha	0.6	60x8 weeks	PCR90	Daily	VAC70	50	All	4.938	0.007	0.002	0.009	0.001	0.009	0.005	2370.421	3.177	1.005	4.182	0.286	4.193	2.281	23704	31.770	10.050	41.820	2.860	41.930	22.810
150	alpha	1	60x8 weeks	PCR90	Daily	VAC70	50	All	4.925	0.006	0.004	0.011	0.001	0.011	0.005	2364.168	3.098	1.976	5.074	0.320	5.102	2.351	23642	30.980	19.760	50.740	3.200	51.020	23.510
150	alpha	1	60x8 weeks	PCR90	Daily	VAC70	50	All	4.925	0.006	0.004	0.011	0.001	0.011	0.005	2364.168	3.098	1.976	5.074	0.320	5.102	2.351	23642	30.980	19.760	50.740	3.200	51.020	23.510
150	alpha	1	60x8 weeks	PCR90	Daily	VAC70	50	All	4.925	0.006	0.004	0.011	0.001	0.011	0.005	2364.168	3.098	1.976	5.074	0.320	5.102	2.351	23642	30.980	19.760	50.740	3.200	51.020	23.510
150	alpha	1.2	60x8 weeks	PCR90	Daily	VAC70	50	All	4.914	0.007	0.006	0.012	0.001	0.012	0.005	2358.532	3.126	2.759	5.885	0.439	5.834	2.258	23585	31.260	27.590	58.850	4.390	58.340	22.580
150	alpha	1.2	60x8 weeks	PCR90	Daily	VAC70	50	All	4.914	0.007	0.006	0.012	0.001	0.012	0.005	2358.532	3.126	2.759	5.885	0.439	5.834	2.258	23585	31.260	27.590	58.850	4.390	58.340	22.580
150	alpha	1.2	60x8 weeks	PCR90	Daily	VAC70	50	All	4.914	0.007	0.006	0.012	0.001	0.012	0.005	2358.532	3.126	2.759	5.885	0.439	5.834	2.258	23585	31.260	27.590	58.850	4.390	58.340	22.580
150	delta	0.6	60x8 weeks	PCR90	Daily	VAC70	50	All	4.888	0.007	0.008	0.016	0.001	0.015	0.005	2346.304	3.486	4.077	7.563	0.715	7.383	2.216	23463	34.860	40.770	75.630	7.150	73.830	22.160
150	delta	0.6	60x8 weeks	PCR90	Daily	VAC70	50	All	4.888	0.007	0.008	0.016	0.001	0.015	0.005	2346.304	3.486	4.077	7.563	0.715	7.383	2.216	23463	34.860	40.770	75.630	7.150	73.830	22.160
150	delta	0.6	60x8 weeks	PCR90	Daily	VAC70	50	All	4.888	0.007	0.008	0.016	0.001	0.015	0.005	2346.304	3.486	4.077	7.563	0.715	7.383	2.216	23463	34.860	40.770	75.630	7.150	73.830	22.160
150	delta	1	60x8 weeks	PCR90	Daily	VAC70	50	All	4.795	0.006	0.023	0.029	0.003	0.028	0.004	2301.423	2.982	10.967	13.949	1.421	13.482	2.112	23014	29.820	109.670	139.490	14.210	134.820	21.120
150	delta	1	60x8 weeks	PCR90	Daily	VAC70	50	All	4.795	0.006	0.023	0.029	0.003	0.028	0.004	2301.423	2.982	10.967	13.949	1.421	13.482	2.112	23014	29.820	109.670	139.490	14.210	134.820	21.120
150	delta	1	60x8 weeks	PCR90	Daily	VAC70	50	All	4.795	0.006	0.023	0.029	0.003	0.028	0.004	2301.423	2.982	10.967	13.949	1.421	13.482	2.112	23014	29.820	109.670	139.490	14.210	134.820	21.120
150	delta	1.2	60x8 weeks	PCR90	Daily	VAC70	50	All	4.685	0.006	0.037	0.044	0.004	0.043	0.004	2249.002	2.951	17.980	20.931	2.072	20.546	1.978	22490	29.510	179.800	209.310	20.720	205.460	19.780
150	delta	1.2	60x8 weeks	PCR90	Daily	VAC70	50	All	4.685	0.006	0.037	0.044	0.004	0.043	0.004	2249.002	2.951	17.980	20.931	2.072	20.546	1.978	22490	29.510	179.800	209.310	20.720	205.460	19.780
150	delta	1.2	60x8 weeks	PCR90	Daily	VAC70	50	All	4.685	0.006	0.037	0.044	0.004	0.043	0.004	2249.002	2.951	17.980	20.931	2.072	20.546	1.978	22490	29.510	179.800	209.310	20.720	205.460	19.780
150	original	0.6	60x8 weeks	PCR90	Daily	VAC70	75	All	4.972	0.005	0.000	0.005	0.001	0.003	0.005	2386.381	2.443	0.168	2.611	0.515	1.576	2.343	23864	24.430	1.680	26.110	5.150	15.760	23.430
150	original	0.6	60x8 weeks	PCR90	Daily	VAC70	75	All	4.972	0.005	0.000	0.005	0.001	0.003	0.005	2386.381	2.443	0.168	2.611	0.515	1.576	2.343	23864	24.430	1.680	26.110	5.150	15.760	23.430
150	original	0.6	60x8 weeks	PCR90	Daily	VAC70	75	All	4.972	0.005	0.000	0.005	0.001	0.003	0.005	2386.381	2.443	0.168	2.611	0.515	1.576	2.343	23864	24.430	1.680	26.110	5.150	15.760	23.430
150	original	1	60x8 weeks	PCR90	Daily	VAC70	75	All	4.970	0.005	0.001	0.006	0.001	0.003	0.005	2385.745	2.432	0.275	2.707	0.624	1.576	2.285	23857	24.320	2.750	27.070	6.240	15.760	22.850
150	original	1	60x8 weeks	PCR90	Daily	VAC70	75	All	4.970	0.005	0.001	0.006	0.001	0.003	0.005	2385.745	2.432	0.275	2.707	0.624	1.576	2.285	23857	24.320	2.750	27.070	6.240	15.760	22.850
150	original	1	60x8 weeks	PCR90	Daily	VAC70	75	All	4.970	0.005	0.001	0.006	0.001	0.003	0.005	2385.745	2.432	0.275	2.707	0.624	1.576	2.285	23857	24.320	2.750	27.070	6.240	15.760	22.850
150	original	1.2	60x8 weeks	PCR90	Daily	VAC70	75	All	4.970	0.005	0.001	0.006	0.001	0.003	0.005	2385.707	2.418	0.350	2.768	0.637	1.595	2.241	23857	24.180	3.500	27.680	6.370	15.950	22.410
150	original	1.2	60x8 weeks	PCR90	Daily	VAC70	75	All	4.970	0.005	0.001	0.006	0.001	0.003	0.005	2385.707	2.418	0.350	2.768	0.637	1.595	2.241	23857	24.180	3.500	27.680	6.370	15.950	22.410

150	original	1.2	60x8 weeks	PCR90	Daily	VAC70	75	All	4.970	0.005	0.001	0.006	0.001	0.003	0.005	2385.707	2.418	0.350	2.768	0.637	1.595	2.241	23857	24.180	3.500	27.680	6.370	15.950	22.410
150	alpha	0.6	60x8 weeks	PCR90	Daily	VAC70	75	All	4.958	0.005	0.001	0.006	0.000	0.006	0.005	2379.927	2.384	0.434	2.818	0.201	2.807	2.472	23799	23.840	4.340	28.180	2.010	28.070	24.720
150	alpha	0.6	60x8 weeks	PCR90	Daily	VAC70	75	All	4.958	0.005	0.001	0.006	0.000	0.006	0.005	2379.927	2.384	0.434	2.818	0.201	2.807	2.472	23799	23.840	4.340	28.180	2.010	28.070	24.720
150	alpha	0.6	60x8 weeks	PCR90	Daily	VAC70	75	All	4.958	0.005	0.001	0.006	0.000	0.006	0.005	2379.927	2.384	0.434	2.818	0.201	2.807	2.472	23799	23.840	4.340	28.180	2.010	28.070	24.720
150	alpha	1	60x8 weeks	PCR90	Daily	VAC70	75	All	4.953	0.005	0.002	0.007	0.001	0.007	0.005	2377.529	2.442	0.792	3.234	0.260	3.172	2.439	23775	24.420	7.920	32.340	2.600	31.720	24.390
150	alpha	1	60x8 weeks	PCR90	Daily	VAC70	75	All	4.953	0.005	0.002	0.007	0.001	0.007	0.005	2377.529	2.442	0.792	3.234	0.260	3.172	2.439	23775	24.420	7.920	32.340	2.600	31.720	24.390
150	alpha	1	60x8 weeks	PCR90	Daily	VAC70	75	All	4.953	0.005	0.002	0.007	0.001	0.007	0.005	2377.529	2.442	0.792	3.234	0.260	3.172	2.439	23775	24.420	7.920	32.340	2.600	31.720	24.390
150	alpha	1.2	60x8 weeks	PCR90	Daily	VAC70	75	All	4.950	0.005	0.002	0.007	0.001	0.007	0.005	2375.936	2.409	1.077	3.486	0.259	3.406	2.346	23759	24.090	10.770	34.860	2.590	34.060	23.460
150	alpha	1.2	60x8 weeks	PCR90	Daily	VAC70	75	All	4.950	0.005	0.002	0.007	0.001	0.007	0.005	2375.936	2.409	1.077	3.486	0.259	3.406	2.346	23759	24.090	10.770	34.860	2.590	34.060	23.460
150	alpha	1.2	60x8 weeks	PCR90	Daily	VAC70	75	All	4.950	0.005	0.002	0.007	0.001	0.007	0.005	2375.936	2.409	1.077	3.486	0.259	3.406	2.346	23759	24.090	10.770	34.860	2.590	34.060	23.460
150	delta	0.6	60x8 weeks	PCR90	Daily	VAC70	75	All	4.935	0.006	0.003	0.009	0.001	0.009	0.005	2368.599	2.787	1.677	4.464	0.432	4.332	2.299	23686	27.870	16.770	44.640	4.320	43.320	22.990
150	delta	0.6	60x8 weeks	PCR90	Daily	VAC70	75	All	4.935	0.006	0.003	0.009	0.001	0.009	0.005	2368.599	2.787	1.677	4.464	0.432	4.332	2.299	23686	27.870	16.770	44.640	4.320	43.320	22.990
150	delta	0.6	60x8 weeks	PCR90	Daily	VAC70	75	All	4.935	0.006	0.003	0.009	0.001	0.009	0.005	2368.599	2.787	1.677	4.464	0.432	4.332	2.299	23686	27.870	16.770	44.640	4.320	43.320	22.990
150	delta	1	60x8 weeks	PCR90	Daily	VAC70	75	All	4.912	0.005	0.007	0.013	0.001	0.012	0.005	2357.711	2.587	3.518	6.105	0.606	5.854	2.256	23577	25.870	35.180	61.050	6.060	58.540	22.560
150	delta	1	60x8 weeks	PCR90	Daily	VAC70	75	All	4.912	0.005	0.007	0.013	0.001	0.012	0.005	2357.711	2.587	3.518	6.105	0.606	5.854	2.256	23577	25.870	35.180	61.050	6.060	58.540	22.560
150	delta	1	60x8 weeks	PCR90	Daily	VAC70	75	All	4.912	0.005	0.007	0.013	0.001	0.012	0.005	2357.711	2.587	3.518	6.105	0.606	5.854	2.256	23577	25.870	35.180	61.050	6.060	58.540	22.560
150	delta	1.2	60x8 weeks	PCR90	Daily	VAC70	75	All	4.882	0.005	0.011	0.017	0.002	0.016	0.005	2343.353	2.615	5.474	8.089	0.751	7.905	2.282	23434	26.150	54.740	80.890	7.510	79.050	22.820
150	delta	1.2	60x8 weeks	PCR90	Daily	VAC70	75	All	4.882	0.005	0.011	0.017	0.002	0.016	0.005	2343.353	2.615	5.474	8.089	0.751	7.905	2.282	23434	26.150	54.740	80.890	7.510	79.050	22.820
150	delta	1.2	60x8 weeks	PCR90	Daily	VAC70	75	All	4.882	0.005	0.011	0.017	0.002	0.016	0.005	2343.353	2.615	5.474	8.089	0.751	7.905	2.282	23434	26.150	54.740	80.890	7.510	79.050	22.820
150	original	0.6	60x8 weeks	PCR90	Daily	VAC70	100	All	4.987	0.003	0.000	0.003	0.001	0.001	0.005	2393.714	1.617	0.015	1.632	0.607	0.360	2.404	23937	16.170	0.150	16.320	6.070	3.600	24.040
150	original	0.6	60x8 weeks	PCR90	Daily	VAC70	100	All	4.987	0.003	0.000	0.003	0.001	0.001	0.005	2393.714	1.617	0.015	1.632	0.607	0.360	2.404	23937	16.170	0.150	16.320	6.070	3.600	24.040
150	original	0.6	60x8 weeks	PCR90	Daily	VAC70	100	All	4.987	0.003	0.000	0.003	0.001	0.001	0.005	2393.714	1.617	0.015	1.632	0.607	0.360	2.404	23937	16.170	0.150	16.320	6.070	3.600	24.040
150	original	1	60x8 weeks	PCR90	Daily	VAC70	100	All	4.987	0.003	0.000	0.003	0.001	0.001	0.005	2393.611	1.659	0.014	1.673	0.625	0.361	2.384	23936	16.590	0.140	16.730	6.250	3.610	23.840
150	original	1	60x8 weeks	PCR90	Daily	VAC70	100	All	4.987	0.003	0.000	0.003	0.001	0.001	0.005	2393.611	1.659	0.014	1.673	0.625	0.361	2.384	23936	16.590	0.140	16.730	6.250	3.610	23.840
150	original	1	60x8 weeks	PCR90	Daily	VAC70	100	All	4.987	0.003	0.000	0.003	0.001	0.001	0.005	2393.611	1.659	0.014	1.673	0.625	0.361	2.384	23936	16.590	0.140	16.730	6.250	3.610	23.840
150	original	1.2	60x8 weeks	PCR90	Daily	VAC70	100	All	4.987	0.003	0.000	0.003	0.001	0.001	0.005	2393.570	1.620	0.030	1.650	0.620	0.366	2.354	23936	16.200	0.300	16.500	6.200	3.660	23.540
150	original	1.2	60x8 weeks	PCR90	Daily	VAC70	100	All	4.987	0.003	0.000	0.003	0.001	0.001	0.005	2393.570	1.620	0.030	1.650	0.620	0.366	2.354	23936	16.200	0.300	16.500	6.200	3.660	23.540
150	original	1.2	60x8 weeks	PCR90	Daily	VAC70	100	All	4.987	0.003	0.000	0.003	0.001	0.001	0.005	2393.570	1.620	0.030	1.650	0.620	0.366	2.354	23936	16.200	0.300	16.500	6.200	3.660	23.540
150	alpha	0.6	60x8 weeks	PCR90	Daily	VAC70	100	All	4.975	0.003	0.000	0.004	0.000	0.004	0.005	2388.003	1.609	0.122	1.731	0.126	1.690	2.416	23880	16.090	1.220	17.310	1.260	16.900	24.160
150	alpha	0.6	60x8 weeks	PCR90	Daily	VAC70	100	All	4.975	0.003	0.000	0.004	0.000	0.004	0.005	2388.003	1.609	0.122	1.731	0.126	1.690	2.416	23880	16.090	1.220	17.310	1.260	16.900	24.160
150	alpha	0.6	60x8 weeks	PCR90	Daily	VAC70	100	All	4.975	0.003	0.000	0.004	0.000	0.004	0.005	2388.003	1.609	0.122	1.731	0.126	1.690	2.416	23880	16.090	1.220	17.310	1.260	16.900	24.160
150	alpha	1	60x8 weeks	PCR90	Daily	VAC70	100	All	4.974	0.003	0.000	0.004	0.000	0.004	0.005	2387.750	1.569	0.218	1.787	0.135	1.731	2.332	23878	15.690	2.180	17.870	1.350	17.310	23.320
150	alpha	1	60x8 weeks	PCR90	Daily	VAC70	100	All	4.974	0.003	0.000	0.004	0.000	0.004	0.005	2387.750	1.569	0.218	1.787	0.135	1.731	2.332	23878	15.690	2.180	17.870	1.350	17.310	23.320
150	alpha	1	60x8 weeks	PCR90	Daily	VAC70	100	All	4.974	0.003	0.000	0.004	0.000	0.004	0.005	2387.750	1.569	0.218	1.787	0.135	1.731	2.332	23878	15.690	2.180	17.870	1.350	17.310	23.320
150	alpha	1.2	60x8 weeks	PCR90	Daily	VAC70	100	All	4.974	0.003	0.001	0.004	0.000	0.004	0.005	2387.476	1.518	0.269	1.787	0.141	1.743	2.369	23875	15.180	2.690	17.870	1.410	17.430	23.690
150	alpha	1.2	60x8 weeks	PCR90	Daily	VAC70	100	All	4.974	0.003	0.001	0.004	0.000	0.004	0.005	2387.476	1.518	0.269	1.787	0.141	1.743	2.369	23875	15.180	2.690	17.870	1.410	17.430	23.690
150	alpha	1.2	60x8 weeks	PCR90	Daily	VAC70	100	All	4.974	0.003	0.001	0.004	0.000	0.004	0.005	2387.476	1.518	0.269	1.787	0.141	1.743	2.369	23875	15.180	2.690	17.870	1.410	17.430	23.690
150	delta	0.6	60x8 weeks	PCR90	Daily	VAC70	100	All	4.963	0.004	0.001	0.005	0.001	0.005	0.005	2382.194	2.051	0.517	2.568	0.280	2.410	2.324	23822	20.510	5.170	25.680	2.800	24.100	23.240
150	delta	0.6	60x8 weeks	PCR90	Daily	VAC70	100	All	4.963	0.004	0.001	0.005	0.001	0.005	0.005	2382.194	2.051	0.517	2.568	0.280	2.410	2.324	23822	20.510	5.170	25.680	2.800	24.100	23.240
150	delta	0.6	60x8 weeks	PCR90	Daily	VAC70	100	All	4.963	0.004	0.001	0.005	0.001	0.005	0.005	2382.194	2.051	0.517	2.568	0.280	2.410	2.324	23822	20.510	5.170	25.680	2.800	24.100	23.240
150	delta	1	60x8 weeks	PCR90	Daily	VAC70	100	All	4.958	0.004	0.002	0.006	0.001	0.006	0.005	2379.882	2.028	0.922	2.950	0.284	2.806	2.388	23799	20.280	9.220	29.500	2.840	28.060	23.880
150	delta	1	60x8 weeks	PCR90	Daily	VAC70	100	All	4.958	0.004	0.002	0.006	0.001	0.006	0.005	2379.882	2.028	0.922	2.950	0.284	2.806	2.388	23799	20.280	9.220	29.500	2.840	28.060	23.880
150	delta	1	60x8 weeks	PCR90	Daily	VAC70	100	All	4.958	0.004	0.002	0.006	0.001	0.006	0.005	2379.882	2.028	0.922	2.950	0.284	2.806	2.388	23799	20.280	9.220	29.500	2.840	28.060	23.880
150	delta	1.2	60x8 weeks	PCR90	Daily	VAC70	100	All	4.950	0.004	0.003	0.007	0.001	0.007	0.005	2376.227	2.045	1.390	3.435	0.358	3.277	2.270	23762	20.450	13.900	34.350	3.580	32.770	22.700
150	delta	1.2	60x8 weeks	PCR90	Daily	VAC70	100	All	4.950	0.004	0.003	0.007	0.001	0.007	0.005	2376.227	2.045	1.390	3.435	0.358	3.277	2.270	23762	20.450	13.900	34.350	3.580	32.770	22.700
150	delta	1.2	60x8 weeks	PCR90	Daily	VAC70	100	All	4.950	0.004	0.003	0.007	0.001	0.007	0.005	2376.227	2.045	1.390	3.435	0.358	3.277	2.270	23762	20.450	13.900	34.350	3.580	32.770	22.700
150	original	0.6	60x8 weeks	PCR90	Daily	VAC50	25	All	4.933	0.009	0.002	0.010	0.001	0.009	0.005	2367.943	4.158	0.858	5.016	0.569	4.287	2.294	23679	41.580	8.580	50.160	5.690	42.870	22.940
150	original	0.6	60x8 weeks	PCR90	Daily	VAC50	25	All	4.933	0.009	0.002	0.010	0.001	0.009	0.005	2367.943	4.158	0.858	5.016	0.569	4.287	2.294	23679	41.580	8.580	50.160	5.690	42.870	22.940
150	original	0.6	60x8 weeks	PCR90	Daily	VAC50	25	All	4.933	0.009	0.002	0.010	0.001	0.009	0.005	2367.943	4.158	0.858	5.016	0.569	4.287	2.294	23679	41.580	8.580	50.160	5.690	42.870	22.940
150	original	1	60x8 weeks	PCR90	Daily	VAC50	25	All	4.920	0.009	0.004	0.012	0.001	0.011	0.005	2361.488	4.240	1.744	5.984	0.691	5.179	2.347	23615	42.400	17.440	59.840	6.910	51.790	23.470
150	original	1	60x8 weeks	PCR90	Daily	VAC50	25	All	4.920	0.009	0.004	0.012	0.001	0.011	0.005	2361.488	4.240	1.744	5.984	0.691	5.179	2.347	23615	42.400	17.440	59.840	6.910	51.790	23.470
150	original	1	60x8 weeks	PCR90	Daily	VAC50	25	All	4.920	0.009	0.004	0.012	0.001	0.011	0.005	2361.488	4.240	1.744	5.984	0.691	5.179	2.347	23615	42.400	17.440	59.840	6.910	51.790	23.470
150	original	1.2	60x8 weeks	PCR90	Daily	VAC50	25	All	4.916	0.009	0.004	0.013	0.001	0.011	0.005	2359.689	4.222	2.113	6.335	0.715	5.452	2.287	23597	42.220	21.130	63.350	7.150	54.520	22.870
150	original	1.2	60x8 weeks	PCR90	Daily	VAC50	25	All	4.916	0.009	0.004	0.013	0.001	0.011	0.005	2359.689	4.222	2.113	6.335	0.715	5.452	2.287	23597	42.220	21.130	63.350	7.150	54.520	22.870
150	original	1.2	60x8 weeks	PCR90	Daily	VAC50	25	All	4.916	0.009	0.004	0.013	0.001	0.011	0.005	2359.689	4.222	2.113	6.335	0.715	5.452	2.287	23597	42.220	21.130	63.350	7.150	54.520	22.870
150	alpha	0.6	60x8 weeks	PCR90	Daily	VAC50	25	All	4.907	0.009	0.004	0.013	0.001	0.013	0.005	2355.251	4.268	2.120	6.388	0.459	6.372	2.199	23553	42.680	21.200	63.880	4.590	63.720	21.990
150	alpha	0.6	60x8 weeks	PCR90	Daily	VAC50	25	All	4.907	0.009	0.004	0.013	0.001	0.013	0.005	2355.251	4.268	2.120	6.388	0.459	6.372	2.199	23553	42.680	21.200	63.880	4.590	63.720	21.990
150	alpha	0.6	60x8 weeks	PCR90	Daily	VAC50	25	All	4.907	0.009	0.004	0.013	0.001	0.013	0.005	2355.251	4.268	2.120	6.388	0.459	6.372	2.199	23553	42.680	21.200	63.880	4.590	63.720	21.990
150	alpha	1	60x8 weeks	PCR90	Daily	VAC50	25	All	4.867	0.009	0.011	0.019	0.001	0.019	0.005	2336.114	4.119	5.052	9.171	0.649	9.161	2.276	23361	41.190	50.520	91.710	6.490	91.610	22.760
150	alpha	1	60x8 weeks	PCR90	Daily	VAC50	25	All	4.867	0.009	0.011	0.019	0.001	0.019	0.005	2336.114	4.119	5.052	9.171	0.649	9.161	2.276	23361	41.190	50.520	91.710	6.490	91.610	22.760
150	alpha	1	60x8 weeks	PCR90	Daily	VAC50	25	All	4.867	0.009	0.011	0.019	0.001	0.019	0.005	2336.114	4.119	5.052	9.171	0.649	9.161	2.276	23361	41.190	50.520	91.710	6.490	91.610	22.760
150	alpha	1.2	60x8 weeks	PCR90	Daily	VAC50	25	All	4.843	0.008	0.014	0.022	0.002	0.022	0.005	2324.808	3.970	6.651	10.621	0.763	10.683	2.174	23248	39.700	66.510	106.210	7.630	106.830	21.740
150	alpha	1.2	60x8 weeks	PCR90	Daily	VAC50	25	All	4.843	0.008	0.014	0.022	0.002	0.022	0.005	2324.808	3.970	6.651	10.621	0.763	10.683	2.174	23248	39.700	66.510	106.210	7.630	106.830	21.740
150	alpha	1.2	60x8 weeks	PCR90	Daily	VAC50	25	All	4.843	0.008	0.014	0.022	0.002	0.022	0.005	2324.808	3.970	6.651	10.621	0.763	10.683	2.174	23248	39.700	66.510	106.210	7.630	106.830	21.740
150	delta	0.6	60x8 weeks	PCR90	Daily	VAC50	25	All	4.808	0.008	0.019	0.027	0.003	0.026	0.005	2307.749	4.074	8.944	13.018	1.292	12.654	2.173	23077	40.740	89.440	130.180	12.920	126.540	21.730
150	delta	0.6	60x8 weeks	PCR90	Daily	VAC50	25	All	4.808	0.008	0.019	0.027	0.003	0.026	0.005	2307.749	4.074	8.944	13.018	1.292	12.654	2.173	23077	40.740	89.440	130.180	12.920	126.540	21.730
150	delta	0.6	60x8 weeks	PCR90	Daily	VAC50	25	All	4.808	0.008	0.019	0.027	0.003	0.026	0.005	2307.749	4.074	8.944	13.018	1.292	12.654	2.173	23077	40.740	89.440	130.180	12.920	126.540	21.730
150	delta	1	60x8 weeks	PCR90	Daily	VAC50	25	All	4.449	0.007	0.068	0.074	0.008	0.073	0.003	2135.678	3.231	32.477	35.708	3.699	35.221	1.550	21357	32.310	324.770	357.080	36.990	352.210	15.500
150	delta	1	60x8 weeks	PCR90	Daily	VAC50	25	All	4.449	0.007	0.068	0.074	0.008	0.073	0.003	2135.678	3.231	32.477	35.708	3.699	35.221	1.550	21357	32.310	324.770	357.080	36.990	352.210	15.500
150	delta	1	60x8 weeks	PCR90	Daily	VAC50	25	All	4.449	0.007	0.068	0.074	0.008	0.073	0.003	2135.678	3.231	32.477	35.708	3.699	35.221	1.550	21357	32.310	324.770	357.080	36.990	352.210	15.500
150	delta	1.2	60x8 weeks	PCR90	Daily	VAC50	25	All	4.308	0.006	0.086	0.092	0.010	0.091	0.003	2067.947	2.799	41.351	44.150	4.736	43.673	1.439	20679	27.990	413.510	441.500	47.360	436.730	14.390

150	delta	1.2	60x8 weeks	PCR90	Daily	VAC50	25	All	4.308	0.006	0.086	0.092	0.010	0.091	0.003	2067.947	2.799	41.351	44.150	4.736	43.673	1.439	20679	27.990	413.510	441.500	47.360	436.730	14.390
150	delta	1.2	60x8 weeks	PCR90	Daily	VAC50	25	All	4.308	0.006	0.086	0.092	0.010	0.091	0.003	2067.947	2.799	41.351	44.150	4.736	43.673	1.439	20679	27.990	413.510	441.500	47.360	436.730	14.390
150	original	0.6	60x8 weeks	PCR90	Daily	VAC50	50	All	4.950	0.008	0.001	0.009	0.001	0.006	0.005	2375.870	3.641	0.465	4.106	0.690	2.971	2.276	23759	36.410	4.650	41.060	6.900	29.710	22.760
150	original	0.6	60x8 weeks	PCR90	Daily	VAC50	50	All	4.950	0.008	0.001	0.009	0.001	0.006	0.005	2375.870	3.641	0.465	4.106	0.690	2.971	2.276	23759	36.410	4.650	41.060	6.900	29.710	22.760
150	original	0.6	60x8 weeks	PCR90	Daily	VAC50	50	All	4.950	0.008	0.001	0.009	0.001	0.006	0.005	2375.870	3.641	0.465	4.106	0.690	2.971	2.276	23759	36.410	4.650	41.060	6.900	29.710	22.760
150	original	1	60x8 weeks	PCR90	Daily	VAC50	50	All	4.944	0.008	0.002	0.010	0.002	0.007	0.005	2373.248	3.699	0.872	4.571	0.794	3.260	2.252	23732	36.990	8.720	45.710	7.940	32.600	22.520
150	original	1	60x8 weeks	PCR90	Daily	VAC50	50	All	4.944	0.008	0.002	0.010	0.002	0.007	0.005	2373.248	3.699	0.872	4.571	0.794	3.260	2.252	23732	36.990	8.720	45.710	7.940	32.600	22.520
150	original	1	60x8 weeks	PCR90	Daily	VAC50	50	All	4.944	0.008	0.002	0.010	0.002	0.007	0.005	2373.248	3.699	0.872	4.571	0.794	3.260	2.252	23732	36.990	8.720	45.710	7.940	32.600	22.520
150	original	1.2	60x8 weeks	PCR90	Daily	VAC50	50	All	4.941	0.008	0.002	0.010	0.002	0.007	0.005	2371.839	3.654	1.168	4.822	0.886	3.419	2.316	23718	36.540	11.680	48.220	8.860	34.190	23.160
150	original	1.2	60x8 weeks	PCR90	Daily	VAC50	50	All	4.941	0.008	0.002	0.010	0.002	0.007	0.005	2371.839	3.654	1.168	4.822	0.886	3.419	2.316	23718	36.540	11.680	48.220	8.860	34.190	23.160
150	original	1.2	60x8 weeks	PCR90	Daily	VAC50	50	All	4.941	0.008	0.002	0.010	0.002	0.007	0.005	2371.839	3.654	1.168	4.822	0.886	3.419	2.316	23718	36.540	11.680	48.220	8.860	34.190	23.160
150	alpha	0.6	60x8 weeks	PCR90	Daily	VAC50	50	All	4.926	0.007	0.003	0.010	0.001	0.010	0.005	2364.632	3.599	1.375	4.974	0.367	4.967	2.357	23646	35.990	13.750	49.740	3.670	49.670	23.570
150	alpha	0.6	60x8 weeks	PCR90	Daily	VAC50	50	All	4.926	0.007	0.003	0.010	0.001	0.010	0.005	2364.632	3.599	1.375	4.974	0.367	4.967	2.357	23646	35.990	13.750	49.740	3.670	49.670	23.570
150	alpha	0.6	60x8 weeks	PCR90	Daily	VAC50	50	All	4.926	0.007	0.003	0.010	0.001	0.010	0.005	2364.632	3.599	1.375	4.974	0.367	4.967	2.357	23646	35.990	13.750	49.740	3.670	49.670	23.570
150	alpha	1	60x8 weeks	PCR90	Daily	VAC50	50	All	4.905	0.008	0.006	0.013	0.001	0.013	0.005	2354.553	3.624	2.845	6.469	0.500	6.398	2.292	23546	36.240	28.450	64.690	5.000	63.980	22.920
150	alpha	1	60x8 weeks	PCR90	Daily	VAC50	50	All	4.905	0.008	0.006	0.013	0.001	0.013	0.005	2354.553	3.624	2.845	6.469	0.500	6.398	2.292	23546	36.240	28.450	64.690	5.000	63.980	22.920
150	alpha	1	60x8 weeks	PCR90	Daily	VAC50	50	All	4.905	0.008	0.006	0.013	0.001	0.013	0.005	2354.553	3.624	2.845	6.469	0.500	6.398	2.292	23546	36.240	28.450	64.690	5.000	63.980	22.920
150	alpha	1.2	60x8 weeks	PCR90	Daily	VAC50	50	All	4.895	0.007	0.007	0.015	0.001	0.015	0.005	2349.383	3.584	3.532	7.116	0.490	7.151	2.331	23494	35.840	35.320	71.160	4.900	71.510	23.310
150	alpha	1.2	60x8 weeks	PCR90	Daily	VAC50	50	All	4.895	0.007	0.007	0.015	0.001	0.015	0.005	2349.383	3.584	3.532	7.116	0.490	7.151	2.331	23494	35.840	35.320	71.160	4.900	71.510	23.310
150	alpha	1.2	60x8 weeks	PCR90	Daily	VAC50	50	All	4.895	0.007	0.007	0.015	0.001	0.015	0.005	2349.383	3.584	3.532	7.116	0.490	7.151	2.331	23494	35.840	35.320	71.160	4.900	71.510	23.310
150	delta	0.6	60x8 weeks	PCR90	Daily	VAC50	50	All	4.861	0.008	0.012	0.020	0.002	0.019	0.005	2333.212	3.913	5.592	9.505	0.911	9.228	2.187	23332	39.130	55.920	95.050	9.110	92.280	21.870
150	delta	0.6	60x8 weeks	PCR90	Daily	VAC50	50	All	4.861	0.008	0.012	0.020	0.002	0.019	0.005	2333.212	3.913	5.592	9.505	0.911	9.228	2.187	23332	39.130	55.920	95.050	9.110	92.280	21.870
150	delta	0.6	60x8 weeks	PCR90	Daily	VAC50	50	All	4.861	0.008	0.012	0.020	0.002	0.019	0.005	2333.212	3.913	5.592	9.505	0.911	9.228	2.187	23332	39.130	55.920	95.050	9.110	92.280	21.870
150	delta	1	60x8 weeks	PCR90	Daily	VAC50	50	All	4.681	0.007	0.037	0.044	0.005	0.043	0.004	2247.092	3.440	17.822	21.262	2.177	20.735	1.952	22471	34.400	178.220	212.620	21.770	207.350	19.520
150	delta	1	60x8 weeks	PCR90	Daily	VAC50	50	All	4.681	0.007	0.037	0.044	0.005	0.043	0.004	2247.092	3.440	17.822	21.262	2.177	20.735	1.952	22471	34.400	178.220	212.620	21.770	207.350	19.520
150	delta	1	60x8 weeks	PCR90	Daily	VAC50	50	All	4.681	0.007	0.037	0.044	0.005	0.043	0.004	2247.092	3.440	17.822	21.262	2.177	20.735	1.952	22471	34.400	178.220	212.620	21.770	207.350	19.520
150	delta	1.2	60x8 weeks	PCR90	Daily	VAC50	50	All	4.522	0.006	0.059	0.065	0.007	0.064	0.004	2170.330	3.040	28.306	31.346	3.349	30.674	1.861	21703	30.400	283.060	313.460	33.490	306.740	18.610
150	delta	1.2	60x8 weeks	PCR90	Daily	VAC50	50	All	4.522	0.006	0.059	0.065	0.007	0.064	0.004	2170.330	3.040	28.306	31.346	3.349	30.674	1.861	21703	30.400	283.060	313.460	33.490	306.740	18.610
150	delta	1.2	60x8 weeks	PCR90	Daily	VAC50	50	All	4.522	0.006	0.059	0.065	0.007	0.064	0.004	2170.330	3.040	28.306	31.346	3.349	30.674	1.861	21703	30.400	283.060	313.460	33.490	306.740	18.610
150	original	0.6	60x8 weeks	PCR90	Daily	VAC50	75	All	4.964	0.007	0.000	0.007	0.002	0.004	0.005	2382.580	3.153	0.238	3.391	0.880	1.758	2.312	23826	31.530	2.380	33.910	8.800	17.580	23.120
150	original	0.6	60x8 weeks	PCR90	Daily	VAC50	75	All	4.964	0.007	0.000	0.007	0.002	0.004	0.005	2382.580	3.153	0.238	3.391	0.880	1.758	2.312	23826	31.530	2.380	33.910	8.800	17.580	23.120
150	original	0.6	60x8 weeks	PCR90	Daily	VAC50	75	All	4.964	0.007	0.000	0.007	0.002	0.004	0.005	2382.580	3.153	0.238	3.391	0.880	1.758	2.312	23826	31.530	2.380	33.910	8.800	17.580	23.120
150	original	1	60x8 weeks	PCR90	Daily	VAC50	75	All	4.963	0.007	0.001	0.007	0.002	0.004	0.005	2382.169	3.204	0.317	3.521	0.922	1.795	2.358	23822	32.040	3.170	35.210	9.220	17.950	23.580
150	original	1	60x8 weeks	PCR90	Daily	VAC50	75	All	4.963	0.007	0.001	0.007	0.002	0.004	0.005	2382.169	3.204	0.317	3.521	0.922	1.795	2.358	23822	32.040	3.170	35.210	9.220	17.950	23.580
150	original	1	60x8 weeks	PCR90	Daily	VAC50	75	All	4.963	0.007	0.001	0.007	0.002	0.004	0.005	2382.169	3.204	0.317	3.521	0.922	1.795	2.358	23822	32.040	3.170	35.210	9.220	17.950	23.580
150	original	1.2	60x8 weeks	PCR90	Daily	VAC50	75	All	4.963	0.006	0.001	0.007	0.002	0.004	0.005	2382.107	3.062	0.425	3.487	0.974	1.745	2.295	23821	30.620	4.250	34.870	9.740	17.450	22.950
150	original	1.2	60x8 weeks	PCR90	Daily	VAC50	75	All	4.963	0.006	0.001	0.007	0.002	0.004	0.005	2382.107	3.062	0.425	3.487	0.974	1.745	2.295	23821	30.620	4.250	34.870	9.740	17.450	22.950
150	original	1.2	60x8 weeks	PCR90	Daily	VAC50	75	All	4.963	0.006	0.001	0.007	0.002	0.004	0.005	2382.107	3.062	0.425	3.487	0.974	1.745	2.295	23821	30.620	4.250	34.870	9.740	17.450	22.950
150	alpha	0.6	60x8 weeks	PCR90	Daily	VAC50	75	All	4.946	0.006	0.001	0.008	0.001	0.008	0.005	2374.193	2.993	0.709	3.702	0.261	3.645	2.278	23742	29.930	7.090	37.020	2.610	36.450	22.780
150	alpha	0.6	60x8 weeks	PCR90	Daily	VAC50	75	All	4.946	0.006	0.001	0.008	0.001	0.008	0.005	2374.193	2.993	0.709	3.702	0.261	3.645	2.278	23742	29.930	7.090	37.020	2.610	36.450	22.780
150	alpha	0.6	60x8 weeks	PCR90	Daily	VAC50	75	All	4.946	0.006	0.001	0.008	0.001	0.008	0.005	2374.193	2.993	0.709	3.702	0.261	3.645	2.278	23742	29.930	7.090	37.020	2.610	36.450	22.780
150	alpha	1	60x8 weeks	PCR90	Daily	VAC50	75	All	4.935	0.006	0.003	0.009	0.001	0.009	0.005	2368.819	3.032	1.441	4.473	0.338	4.392	2.233	23688	30.320	14.410	44.730	3.380	43.920	22.330
150	alpha	1	60x8 weeks	PCR90	Daily	VAC50	75	All	4.935	0.006	0.003	0.009	0.001	0.009	0.005	2368.819	3.032	1.441	4.473	0.338	4.392	2.233	23688	30.320	14.410	44.730	3.380	43.920	22.330
150	alpha	1	60x8 weeks	PCR90	Daily	VAC50	75	All	4.935	0.006	0.003	0.009	0.001	0.009	0.005	2368.819	3.032	1.441	4.473	0.338	4.392	2.233	23688	30.320	14.410	44.730	3.380	43.920	22.330
150	alpha	1.2	60x8 weeks	PCR90	Daily	VAC50	75	All	4.931	0.006	0.004	0.010	0.001	0.010	0.005	2366.916	3.061	1.742	4.803	0.337	4.733	2.327	23669	30.610	17.420	48.030	3.370	47.330	23.270
150	alpha	1.2	60x8 weeks	PCR90	Daily	VAC50	75	All	4.931	0.006	0.004	0.010	0.001	0.010	0.005	2366.916	3.061	1.742	4.803	0.337	4.733	2.327	23669	30.610	17.420	48.030	3.370	47.330	23.270
150	alpha	1.2	60x8 weeks	PCR90	Daily	VAC50	75	All	4.931	0.006	0.004	0.010	0.001	0.010	0.005	2366.916	3.061	1.742	4.803	0.337	4.733	2.327	23669	30.610	17.420	48.030	3.370	47.330	23.270
150	delta	0.6	60x8 weeks	PCR90	Daily	VAC50	75	All	4.901	0.008	0.006	0.014	0.001	0.013	0.005	2352.586	3.626	3.064	6.690	0.717	6.437	2.202	23526	36.260	30.640	66.900	7.170	64.370	22.020
150	delta	0.6	60x8 weeks	PCR90	Daily	VAC50	75	All	4.901	0.008	0.006	0.014	0.001	0.013	0.005	2352.586	3.626	3.064	6.690	0.717	6.437	2.202	23526	36.260	30.640	66.900	7.170	64.370	22.020
150	delta	0.6	60x8 weeks	PCR90	Daily	VAC50	75	All	4.901	0.008	0.006	0.014	0.001	0.013	0.005	2352.586	3.626	3.064	6.690	0.717	6.437	2.202	23526	36.260	30.640	66.900	7.170	64.370	22.020
150	delta	1	60x8 weeks	PCR90	Daily	VAC50	75	All	4.825	0.007	0.017	0.025	0.003	0.024	0.005	2315.880	3.477	8.392	11.869	1.210	11.458	2.177	23159	34.770	83.920	118.690	12.100	114.580	21.770
150	delta	1	60x8 weeks	PCR90	Daily	VAC50	75	All	4.825	0.007	0.017	0.025	0.003	0.024	0.005	2315.880	3.477	8.392	11.869	1.210	11.458	2.177	23159	34.770	83.920	118.690	12.100	114.580	21.770
150	delta	1	60x8 weeks	PCR90	Daily	VAC50	75	All	4.825	0.007	0.017	0.025	0.003	0.024	0.005	2315.880	3.477	8.392	11.869	1.210	11.458	2.177	23159	34.770	83.920	118.690	12.100	114.580	21.770
150	delta	1.2	60x8 weeks	PCR90	Daily	VAC50	75	All	4.763	0.007	0.026	0.033	0.004	0.032	0.004	2286.416	3.285	12.686	15.971	1.684	15.423	2.066	22864	32.850	126.860	159.710	16.840	154.230	20.660
150	delta	1.2	60x8 weeks	PCR90	Daily	VAC50	75	All	4.763	0.007	0.026	0.033	0.004	0.032	0.004	2286.416	3.285	12.686	15.971	1.684	15.423	2.066	22864	32.850	126.860	159.710	16.840	154.230	20.660
150	delta	1.2	60x8 weeks	PCR90	Daily	VAC50	75	All	4.763	0.007	0.026	0.033	0.004	0.032	0.004	2286.416	3.285	12.686	15.971	1.684	15.423	2.066	22864	32.850	126.860	159.710	16.840	154.230	20.660
150	original	0.6	60x8 weeks	PCR90	Daily	VAC50	100	All	4.979	0.005	0.000	0.006	0.002	0.001	0.005	2389.772	2.612	0.035	2.647	1.003	0.581	2.353	23898	26.120	0.350	26.470	10.030	5.810	23.530
150	original	0.6	60x8 weeks	PCR90	Daily	VAC50	100	All	4.979	0.005	0.000	0.006	0.002	0.001	0.005	2389.772	2.612	0.035	2.647	1.003	0.581	2.353	23898	26.120	0.350	26.470	10.030	5.810	23.530
150	original	0.6	60x8 weeks	PCR90	Daily	VAC50	100	All	4.979	0.005	0.000	0.006	0.002	0.001	0.005	2389.772	2.612	0.035	2.647	1.003	0.581	2.353	23898	26.120	0.350	26.470	10.030	5.810	23.530
150	original	1	60x8 weeks	PCR90	Daily	VAC50	100	All	4.978	0.006	0.000	0.006	0.002	0.001	0.005	2389.368	2.653	0.045	2.698	1.012	0.629	2.342	23894	26.530	0.450	26.980	10.120	6.290	23.420
150	original	1	60x8 weeks	PCR90	Daily	VAC50	100	All	4.978	0.006	0.000	0.006	0.002	0.001	0.005	2389.368	2.653	0.045	2.698	1.012	0.629	2.342	23894	26.530	0.450	26.980	10.120	6.290	23.420
150	original	1	60x8 weeks	PCR90	Daily	VAC50	100	All	4.978	0.006	0.000	0.006	0.002	0.001	0.005	2389.368	2.653	0.045	2.698	1.012	0.629	2.342	23894	26.530	0.450	26.980	10.120	6.290	23.420
150	original	1.2	60x8 weeks	PCR90	Daily	VAC50	100	All	4.980	0.005	0.000	0.005	0.002	0.001	0.005	2390.167	2.469	0.055	2.524	0.950	0.569	2.393	23902	24.690	0.550	25.240	9.500	5.690	23.930
150	original	1.2	60x8 weeks	PCR90	Daily	VAC50	100	All	4.980	0.005	0.000	0.005	0.002	0.001	0.005	2390.167	2.469	0.055	2.524	0.950	0.569	2.393	23902	24.690	0.550	25.240	9.500	5.690	23.930
150	original	1.2	60x8 weeks	PCR90	Daily	VAC50	100	All	4.980	0.005	0.000	0.005	0.002	0.001	0.005	2390.167	2.469	0.055	2.524	0.950	0.569	2.393	23902	24.690	0.550	25.240	9.500	5.690	23.930
150	alpha	0.6	60x8 weeks	PCR90	Daily	VAC50	100	All	4.958	0.005	0.001	0.006	0.001	0.006	0.005	2380.042	2.527	0.329	2.856	0.250	2.768	2.300	23800	25.270	3.290	28.560	2.500	27.680	23.000
150	alpha	0.6	60x8 weeks	PCR90	Daily	VAC50	100	All	4.958	0.005	0.001	0.006	0.001	0.006	0.005	2380.042	2.527	0.329	2.856	0.250	2.768	2.300	23800	25.270	3.290	28.560	2.500	27.680	23.000
150	alpha	0.6	60x8 weeks	PCR90	Daily	VAC50	100	All	4.958	0.005	0.001	0.006	0.001	0.006	0.005	2380.042	2.527	0.329	2.856	0.250	2.768	2.300	23800	25.270	3.290	28.560	2.500	27.680	23.000
150	alpha	1	60x8 weeks	PCR90	Daily	VAC50	100	All	4.954	0.005	0.001	0.007	0.001	0.006	0.005	2377.969	2.482	0.667	3.149	0.252	3.073	2.412	23780	24.820	6.670	31.490	2.520	30.730	24.120
150	alpha	1	60x8 weeks	PCR90	Daily	VAC50	100	All	4.954	0.005	0.001	0.007	0.001	0.006	0.005	2377.969	2.482	0.667	3.149	0.252	3.073	2.412	23780	24.820	6.670	31.490	2.520	30.730	24.120
150	alpha	1	60x8 weeks	PCR90	Daily	VAC50	100	All	4.954	0.005	0.001	0.007	0.001	0.006	0.005	2377.969	2.482	0.667	3.149	0.252	3.073	2.412	23780	24.820	6.670	31.490	2.520	30.730	24.120

150	alpha	1.2	60x8 weeks	PCR90	Daily	VAC50	100	All	4.953	0.005	0.002	0.007	0.001	0.007	0.005	2377.228	2.512	0.795	3.307	0.264	3.208	2.382	23772	25.120	7.950	33.070	2.640	32.080	23.820
150	alpha	1.2	60x8 weeks	PCR90	Daily	VAC50	100	All	4.953	0.005	0.002	0.007	0.001	0.007	0.005	2377.228	2.512	0.795	3.307	0.264	3.208	2.382	23772	25.120	7.950	33.070	2.640	32.080	23.820
150	alpha	1.2	60x8 weeks	PCR90	Daily	VAC50	100	All	4.953	0.005	0.002	0.007	0.001	0.007	0.005	2377.228	2.512	0.795	3.307	0.264	3.208	2.382	23772	25.120	7.950	33.070	2.640	32.080	23.820
150	delta	0.6	60x8 weeks	PCR90	Daily	VAC50	100	All	4.928	0.007	0.003	0.010	0.001	0.010	0.005	2365.611	3.339	1.570	4.909	0.518	4.713	2.337	23656	33.390	15.700	49.090	5.180	47.130	23.370
150	delta	0.6	60x8 weeks	PCR90	Daily	VAC50	100	All	4.928	0.007	0.003	0.010	0.001	0.010	0.005	2365.611	3.339	1.570	4.909	0.518	4.713	2.337	23656	33.390	15.700	49.090	5.180	47.130	23.370
150	delta	0.6	60x8 weeks	PCR90	Daily	VAC50	100	All	4.928	0.007	0.003	0.010	0.001	0.010	0.005	2365.611	3.339	1.570	4.909	0.518	4.713	2.337	23656	33.390	15.700	49.090	5.180	47.130	23.370
150	delta	1	60x8 weeks	PCR90	Daily	VAC50	100	All	4.899	0.007	0.008	0.015	0.001	0.014	0.005	2351.446	3.337	3.624	6.961	0.676	6.717	2.311	23514	33.370	36.240	69.610	6.760	67.170	23.110
150	delta	1	60x8 weeks	PCR90	Daily	VAC50	100	All	4.899	0.007	0.008	0.015	0.001	0.014	0.005	2351.446	3.337	3.624	6.961	0.676	6.717	2.311	23514	33.370	36.240	69.610	6.760	67.170	23.110
150	delta	1	60x8 weeks	PCR90	Daily	VAC50	100	All	4.899	0.007	0.008	0.015	0.001	0.014	0.005	2351.446	3.337	3.624	6.961	0.676	6.717	2.311	23514	33.370	36.240	69.610	6.760	67.170	23.110
150	delta	1.2	60x8 weeks	PCR90	Daily	VAC50	100	All	4.876	0.007	0.011	0.018	0.002	0.017	0.005	2340.353	3.266	5.169	8.435	0.896	8.114	2.236	23404	32.660	51.690	84.350	8.960	81.140	22.360
150	delta	1.2	60x8 weeks	PCR90	Daily	VAC50	100	All	4.876	0.007	0.011	0.018	0.002	0.017	0.005	2340.353	3.266	5.169	8.435	0.896	8.114	2.236	23404	32.660	51.690	84.350	8.960	81.140	22.360
150	delta	1.2	60x8 weeks	PCR90	Daily	VAC50	100	All	4.876	0.007	0.011	0.018	0.002	0.017	0.005	2340.353	3.266	5.169	8.435	0.896	8.114	2.236	23404	32.660	51.690	84.350	8.960	81.140	22.360
150	original	0.6	60x8 weeks	PCR90	MWF	NOVAC	0	All	2.950	0.010	0.003	0.013	0.001	0.012	0.003	1416.239	4.817	1.650	6.467	0.658	5.961	1.303	14162	48.170	16.500	64.670	6.580	59.610	13.030
150	original	0.6	60x8 weeks	PCR90	MWF	NOVAC	0	All	2.950	0.010	0.003	0.013	0.001	0.012	0.003	1416.239	4.817	1.650	6.467	0.658	5.961	1.303	14162	48.170	16.500	64.670	6.580	59.610	13.030
150	original	0.6	60x8 weeks	PCR90	MWF	NOVAC	0	All	2.950	0.010	0.003	0.013	0.001	0.012	0.003	1416.239	4.817	1.650	6.467	0.658	5.961	1.303	14162	48.170	16.500	64.670	6.580	59.610	13.030
150	original	1	60x8 weeks	PCR90	MWF	NOVAC	0	All	2.934	0.010	0.008	0.018	0.002	0.016	0.003	1408.153	4.715	3.689	8.404	0.879	7.903	1.334	14082	47.150	36.890	84.040	8.790	79.030	13.340
150	original	1	60x8 weeks	PCR90	MWF	NOVAC	0	All	2.934	0.010	0.008	0.018	0.002	0.016	0.003	1408.153	4.715	3.689	8.404	0.879	7.903	1.334	14082	47.150	36.890	84.040	8.790	79.030	13.340
150	original	1	60x8 weeks	PCR90	MWF	NOVAC	0	All	2.934	0.010	0.008	0.018	0.002	0.016	0.003	1408.153	4.715	3.689	8.404	0.879	7.903	1.334	14082	47.150	36.890	84.040	8.790	79.030	13.340
150	original	1.2	60x8 weeks	PCR90	MWF	NOVAC	0	All	2.925	0.010	0.011	0.020	0.002	0.019	0.003	1403.952	4.620	5.104	9.724	0.983	9.052	1.309	14040	46.200	51.040	97.240	9.830	90.520	13.090
150	original	1.2	60x8 weeks	PCR90	MWF	NOVAC	0	All	2.925	0.010	0.011	0.020	0.002	0.019	0.003	1403.952	4.620	5.104	9.724	0.983	9.052	1.309	14040	46.200	51.040	97.240	9.830	90.520	13.090
150	original	1.2	60x8 weeks	PCR90	MWF	NOVAC	0	All	2.925	0.010	0.011	0.020	0.002	0.019	0.003	1403.952	4.620	5.104	9.724	0.983	9.052	1.309	14040	46.200	51.040	97.240	9.830	90.520	13.090
150	alpha	0.6	60x8 weeks	PCR90	MWF	NOVAC	0	All	2.923	0.010	0.010	0.020	0.002	0.019	0.003	1403.118	4.719	4.867	9.586	1.072	9.115	1.330	14031	47.190	48.670	95.860	10.720	91.150	13.300
150	alpha	0.6	60x8 weeks	PCR90	MWF	NOVAC	0	All	2.923	0.010	0.010	0.020	0.002	0.019	0.003	1403.118	4.719	4.867	9.586	1.072	9.115	1.330	14031	47.190	48.670	95.860	10.720	91.150	13.300
150	alpha	0.6	60x8 weeks	PCR90	MWF	NOVAC	0	All	2.923	0.010	0.010	0.020	0.002	0.019	0.003	1403.118	4.719	4.867	9.586	1.072	9.115	1.330	14031	47.190	48.670	95.860	10.720	91.150	13.300
150	alpha	1	60x8 weeks	PCR90	MWF	NOVAC	0	All	2.856	0.009	0.028	0.037	0.004	0.035	0.003	1371.018	4.286	13.556	17.842	2.022	16.932	1.246	13710	42.860	135.560	178.420	20.220	169.320	12.460
150	alpha	1	60x8 weeks	PCR90	MWF	NOVAC	0	All	2.856	0.009	0.028	0.037	0.004	0.035	0.003	1371.018	4.286	13.556	17.842	2.022	16.932	1.246	13710	42.860	135.560	178.420	20.220	169.320	12.460
150	alpha	1	60x8 weeks	PCR90	MWF	NOVAC	0	All	2.856	0.009	0.028	0.037	0.004	0.035	0.003	1371.018	4.286	13.556	17.842	2.022	16.932	1.246	13710	42.860	135.560	178.420	20.220	169.320	12.460
150	alpha	1.2	60x8 weeks	PCR90	MWF	NOVAC	0	All	2.793	0.008	0.044	0.053	0.006	0.050	0.002	1340.857	4.039	21.189	25.228	2.900	24.147	1.112	13409	40.390	211.890	252.280	29.000	241.470	11.120
150	alpha	1.2	60x8 weeks	PCR90	MWF	NOVAC	0	All	2.793	0.008	0.044	0.053	0.006	0.050	0.002	1340.857	4.039	21.189	25.228	2.900	24.147	1.112	13409	40.390	211.890	252.280	29.000	241.470	11.120
150	alpha	1.2	60x8 weeks	PCR90	MWF	NOVAC	0	All	2.793	0.008	0.044	0.053	0.006	0.050	0.002	1340.857	4.039	21.189	25.228	2.900	24.147	1.112	13409	40.390	211.890	252.280	29.000	241.470	11.120
150	delta	0.6	60x8 weeks	PCR90	MWF	NOVAC	0	All	2.736	0.008	0.059	0.066	0.011	0.060	0.002	1313.366	3.722	28.187	31.909	5.313	28.773	1.024	13134	37.220	281.870	319.090	53.130	287.730	10.240
150	delta	0.6	60x8 weeks	PCR90	MWF	NOVAC	0	All	2.736	0.008	0.059	0.066	0.011	0.060	0.002	1313.366	3.722	28.187	31.909	5.313	28.773	1.024	13134	37.220	281.870	319.090	53.130	287.730	10.240
150	delta	0.6	60x8 weeks	PCR90	MWF	NOVAC	0	All	2.736	0.008	0.059	0.066	0.011	0.060	0.002	1313.366	3.722	28.187	31.909	5.313	28.773	1.024	13134	37.220	281.870	319.090	53.130	287.730	10.240
150	delta	1	60x8 weeks	PCR90	MWF	NOVAC	0	All	2.511	0.005	0.109	0.114	0.020	0.105	0.001	1205.326	2.324	52.337	54.661	9.499	50.597	0.616	12053	23.240	523.370	546.610	94.990	505.970	6.160
150	delta	1	60x8 weeks	PCR90	MWF	NOVAC	0	All	2.511	0.005	0.109	0.114	0.020	0.105	0.001	1205.326	2.324	52.337	54.661	9.499	50.597	0.616	12053	23.240	523.370	546.610	94.990	505.970	6.160
150	delta	1	60x8 weeks	PCR90	MWF	NOVAC	0	All	2.511	0.005	0.109	0.114	0.020	0.105	0.001	1205.326	2.324	52.337	54.661	9.499	50.597	0.616	12053	23.240	523.370	546.610	94.990	505.970	6.160
150	delta	1.2	60x8 weeks	PCR90	MWF	NOVAC	0	All	2.479	0.004	0.116	0.121	0.021	0.112	0.001	1190.037	2.069	55.890	57.959	10.211	53.820	0.600	11900	20.690	558.900	579.590	102.110	538.200	6.000
150	delta	1.2	60x8 weeks	PCR90	MWF	NOVAC	0	All	2.479	0.004	0.116	0.121	0.021	0.112	0.001	1190.037	2.069	55.890	57.959	10.211	53.820	0.600	11900	20.690	558.900	579.590	102.110	538.200	6.000
150	delta	1.2	60x8 weeks	PCR90	MWF	NOVAC	0	All	2.479	0.004	0.116	0.121	0.021	0.112	0.001	1190.037	2.069	55.890	57.959	10.211	53.820	0.600	11900	20.690	558.900	579.590	102.110	538.200	6.000
150	original	0.6	60x8 weeks	PCR90	MWF	VAC95	25	All	2.965	0.008	0.002	0.010	0.001	0.009	0.003	1423.245	3.709	0.893	4.602	0.468	4.210	1.317	14232	37.090	8.930	46.020	4.680	42.100	13.170
150	original	0.6	60x8 weeks	PCR90	MWF	VAC95	25	All	2.965	0.008	0.002	0.010	0.001	0.009	0.003	1423.245	3.709	0.893	4.602	0.468	4.210	1.317	14232	37.090	8.930	46.020	4.680	42.100	13.170
150	original	0.6	60x8 weeks	PCR90	MWF	VAC95	25	All	2.965	0.008	0.002	0.010	0.001	0.009	0.003	1423.245	3.709	0.893	4.602	0.468	4.210	1.317	14232	37.090	8.930	46.020	4.680	42.100	13.170
150	original	1	60x8 weeks	PCR90	MWF	VAC95	25	All	2.959	0.008	0.004	0.011	0.001	0.010	0.003	1420.208	3.705	1.789	5.494	0.548	4.989	1.377	14202	37.050	17.890	54.940	5.480	49.890	13.770
150	original	1	60x8 weeks	PCR90	MWF	VAC95	25	All	2.959	0.008	0.004	0.011	0.001	0.010	0.003	1420.208	3.705	1.789	5.494	0.548	4.989	1.377	14202	37.050	17.890	54.940	5.480	49.890	13.770
150	original	1	60x8 weeks	PCR90	MWF	VAC95	25	All	2.959	0.008	0.004	0.011	0.001	0.010	0.003	1420.208	3.705	1.789	5.494	0.548	4.989	1.377	14202	37.050	17.890	54.940	5.480	49.890	13.770
150	original	1.2	60x8 weeks	PCR90	MWF	VAC95	25	All	2.954	0.008	0.005	0.013	0.001	0.012	0.003	1417.733	3.683	2.393	6.076	0.617	5.567	1.444	14177	36.830	23.930	60.760	6.170	55.670	14.440
150	original	1.2	60x8 weeks	PCR90	MWF	VAC95	25	All	2.954	0.008	0.005	0.013	0.001	0.012	0.003	1417.733	3.683	2.393	6.076	0.617	5.567	1.444	14177	36.830	23.930	60.760	6.170	55.670	14.440
150	original	1.2	60x8 weeks	PCR90	MWF	VAC95	25	All	2.954	0.008	0.005	0.013	0.001	0.012	0.003	1417.733	3.683	2.393	6.076	0.617	5.567	1.444	14177	36.830	23.930	60.760	6.170	55.670	14.440
150	alpha	0.6	60x8 weeks	PCR90	MWF	VAC95	25	All	2.953	0.008	0.005	0.012	0.001	0.012	0.003	1417.483	3.663	2.303	5.966	0.619	5.648	1.373	14175	36.630	23.030	59.660	6.190	56.480	13.730
150	alpha	0.6	60x8 weeks	PCR90	MWF	VAC95	25	All	2.953	0.008	0.005	0.012	0.001	0.012	0.003	1417.483	3.663	2.303	5.966	0.619	5.648	1.373	14175	36.630	23.030	59.660	6.190	56.480	13.730
150	alpha	0.6	60x8 weeks	PCR90	MWF	VAC95	25	All	2.953	0.008	0.005	0.012	0.001	0.012	0.003	1417.483	3.663	2.303	5.966	0.619	5.648	1.373	14175	36.630	23.030	59.660	6.190	56.480	13.730
150	alpha	1	60x8 weeks	PCR90	MWF	VAC95	25	All	2.928	0.007	0.011	0.019	0.002	0.018	0.003	1405.654	3.534	5.486	9.020	0.947	8.549	1.388	14057	35.340	54.860	90.200	9.470	85.490	13.880
150	alpha	1	60x8 weeks	PCR90	MWF	VAC95	25	All	2.928	0.007	0.011	0.019	0.002	0.018	0.003	1405.654	3.534	5.486	9.020	0.947	8.549	1.388	14057	35.340	54.860	90.200	9.470	85.490	13.880
150	alpha	1	60x8 weeks	PCR90	MWF	VAC95	25	All	2.928	0.007	0.011	0.019	0.002	0.018	0.003	1405.654	3.534	5.486	9.020	0.947	8.549	1.388	14057	35.340	54.860	90.200	9.470	85.490	13.880
150	alpha	1.2	60x8 weeks	PCR90	MWF	VAC95	25	All	2.909	0.007	0.017	0.024	0.003	0.023	0.003	1396.165	3.418	7.941	11.359	1.280	10.820	1.365	13962	34.180	79.410	113.590	12.800	108.200	13.650
150	alpha	1.2	60x8 weeks	PCR90	MWF	VAC95	25	All	2.909	0.007	0.017	0.024	0.003	0.023	0.003	1396.165	3.418	7.941	11.359	1.280	10.820	1.365	13962	34.180	79.410	113.590	12.800	108.200	13.650
150	alpha	1.2	60x8 weeks	PCR90	MWF	VAC95	25	All	2.909	0.007	0.017	0.024	0.003	0.023	0.003	1396.165	3.418	7.941	11.359	1.280	10.820	1.365	13962	34.180	79.410	113.590	12.800	108.200	13.650
150	delta	0.6	60x8 weeks	PCR90	MWF	VAC95	25	All	2.885	0.007	0.022	0.030	0.005	0.026	0.003	1384.895	3.442	10.752	14.194	2.383	12.638	1.291	13849	34.420	107.520	141.940	23.830	126.380	12.910
150	delta	0.6	60x8 weeks	PCR90	MWF	VAC95	25	All	2.885	0.007	0.022	0.030	0.005	0.026	0.003	1384.895	3.442	10.752	14.194	2.383	12.638	1.291	13849	34.420	107.520	141.940	23.830	126.380	12.910
150	delta	0.6	60x8 weeks	PCR90	MWF	VAC95	25	All	2.885	0.007	0.022	0.030	0.005	0.026	0.003	1384.895	3.442	10.752	14.194	2.383	12.638	1.291	13849	34.420	107.520	141.940	23.830	126.380	12.910
150	delta	1	60x8 weeks	PCR90	MWF	VAC95	25	All	2.722	0.005	0.063	0.069	0.012	0.062	0.002	1306.357	2.607	30.412	33.019	5.579	29.868	0.994	13064	26.070	304.120	330.190	55.790	298.680	9.940
150	delta	1	60x8 weeks	PCR90	MWF	VAC95	25	All	2.722	0.005	0.063	0.069	0.012	0.062	0.002	1306.357	2.607	30.412	33.019	5.579	29.868	0.994	13064	26.070	304.120	330.190	55.790	298.680	9.940
150	delta	1	60x8 weeks	PCR90	MWF	VAC95	25	All	2.722	0.005	0.063	0.069	0.012	0.062	0.002	1306.357	2.607	30.412	33.019	5.579	29.868	0.994	13064	26.070	304.120	330.190	55.790	298.680	9.940
150	delta	1.2	60x8 weeks	PCR90	MWF	VAC95	25	All	2.652	0.005	0.080	0.085	0.014	0.078	0.002	1272.975	2.270	38.437	40.707	6.805	37.213	0.877	12730	22.700	384.370	407.070	68.050	372.130	8.770
150	delta	1.2	60x8 weeks	PCR90	MWF	VAC95	25	All	2.652	0.005	0.080	0.085	0.014	0.078	0.002	1272.975	2.270	38.437	40.707	6.805	37.213	0.877	12730	22.700	384.370	407.070	68.050	372.130	8.770
150	delta	1.2	60x8 weeks	PCR90	MWF	VAC95	25	All	2.652	0.005	0.080	0.085	0.014	0.078	0.002	1272.975	2.270	38.437	40.707	6.805	37.213	0.877	12730	22.700	384.370	407.070	68.050	372.130	8.770
150	original	0.6	60x8 weeks	PCR90	MWF	VAC95	50	All	2.978	0.005	0.001	0.006	0.001	0.005	0.003	1429.366	2.579	0.414	2.993	0.347	2.608	1.405	14294	25.790	4.140	29.930	3.470	26.080	14.050
150	original	0.6	60x8 weeks	PCR90	MWF	VAC95	50	All	2.978	0.005	0.001	0.006	0.001	0.005	0.003	1429.366	2.579	0.414	2.993	0.347	2.608	1.405	14294	25.790	4.140	29.930	3.470	26.080	14.050
150	original	0.6	60x8 weeks	PCR90	MWF	VAC95	50	All	2.978	0.005	0.001	0.006	0.001	0.005	0.003	1429.366	2.579	0.414	2.993	0.347	2.608	1.405	14294	25.790	4.140	29.930	3.470	26.080	14.050
150	original	1	60x8 weeks	PCR90	MWF	VAC95	50	All	2.976	0.005	0.002	0.007	0.001	0.006	0.003	1428.460	2.553	0.729	3.282	0.389	2.811	1.364	14285	25.530	7.290	32.820	3.890	28.110	13.640
150	original	1	60x8 weeks	PCR90	MWF	VAC95	50	All	2.976	0.005	0.002	0.007	0.001	0.006	0.003	1428.460	2.553	0.729	3.282	0.389	2.811	1.364	14285	25.530	7.290	32.820	3.890	28.110	13.640

150	original	1	60x8 weeks	PCR90	MWF	VAC95	50	All	2.976	0.005	0.002	0.007	0.001	0.006	0.003	1428.460	2.553	0.729	3.282	0.389	2.811	1.364	14285	25.530	7.290	32.820	3.890	28.110	13.640
150	original	1.2	60x8 weeks	PCR90	MWF	VAC95	50	All	2.975	0.005	0.002	0.007	0.001	0.006	0.003	1428.100	2.501	0.824	3.325	0.362	2.918	1.329	14281	25.010	8.240	33.250	3.620	29.180	13.290
150	original	1.2	60x8 weeks	PCR90	MWF	VAC95	50	All	2.975	0.005	0.002	0.007	0.001	0.006	0.003	1428.100	2.501	0.824	3.325	0.362	2.918	1.329	14281	25.010	8.240	33.250	3.620	29.180	13.290
150	original	1.2	60x8 weeks	PCR90	MWF	VAC95	50	All	2.975	0.005	0.002	0.007	0.001	0.006	0.003	1428.100	2.501	0.824	3.325	0.362	2.918	1.329	14281	25.010	8.240	33.250	3.620	29.180	13.290
150	alpha	0.6	60x8 weeks	PCR90	MWF	VAC95	50	All	2.972	0.005	0.002	0.007	0.001	0.007	0.003	1426.714	2.603	0.932	3.535	0.384	3.330	1.365	14267	26.030	9.320	35.350	3.840	33.300	13.650
150	alpha	0.6	60x8 weeks	PCR90	MWF	VAC95	50	All	2.972	0.005	0.002	0.007	0.001	0.007	0.003	1426.714	2.603	0.932	3.535	0.384	3.330	1.365	14267	26.030	9.320	35.350	3.840	33.300	13.650
150	alpha	0.6	60x8 weeks	PCR90	MWF	VAC95	50	All	2.972	0.005	0.002	0.007	0.001	0.007	0.003	1426.714	2.603	0.932	3.535	0.384	3.330	1.365	14267	26.030	9.320	35.350	3.840	33.300	13.650
150	alpha	1	60x8 weeks	PCR90	MWF	VAC95	50	All	2.963	0.005	0.004	0.010	0.001	0.009	0.003	1422.442	2.621	1.968	4.589	0.465	4.400	1.384	14224	26.210	19.680	45.890	4.650	44.000	13.840
150	alpha	1	60x8 weeks	PCR90	MWF	VAC95	50	All	2.963	0.005	0.004	0.010	0.001	0.009	0.003	1422.442	2.621	1.968	4.589	0.465	4.400	1.384	14224	26.210	19.680	45.890	4.650	44.000	13.840
150	alpha	1	60x8 weeks	PCR90	MWF	VAC95	50	All	2.963	0.005	0.004	0.010	0.001	0.009	0.003	1422.442	2.621	1.968	4.589	0.465	4.400	1.384	14224	26.210	19.680	45.890	4.650	44.000	13.840
150	alpha	1.2	60x8 weeks	PCR90	MWF	VAC95	50	All	2.960	0.005	0.005	0.010	0.001	0.010	0.003	1420.719	2.547	2.492	5.039	0.518	4.784	1.362	14207	25.470	24.920	50.390	5.180	47.840	13.620
150	alpha	1.2	60x8 weeks	PCR90	MWF	VAC95	50	All	2.960	0.005	0.005	0.010	0.001	0.010	0.003	1420.719	2.547	2.492	5.039	0.518	4.784	1.362	14207	25.470	24.920	50.390	5.180	47.840	13.620
150	alpha	1.2	60x8 weeks	PCR90	MWF	VAC95	50	All	2.960	0.005	0.005	0.010	0.001	0.010	0.003	1420.719	2.547	2.492	5.039	0.518	4.784	1.362	14207	25.470	24.920	50.390	5.180	47.840	13.620
150	delta	0.6	60x8 weeks	PCR90	MWF	VAC95	50	All	2.952	0.005	0.007	0.012	0.002	0.011	0.003	1417.189	2.626	3.335	5.961	0.953	5.308	1.370	14172	26.260	33.350	59.610	9.530	53.080	13.700
150	delta	0.6	60x8 weeks	PCR90	MWF	VAC95	50	All	2.952	0.005	0.007	0.012	0.002	0.011	0.003	1417.189	2.626	3.335	5.961	0.953	5.308	1.370	14172	26.260	33.350	59.610	9.530	53.080	13.700
150	delta	0.6	60x8 weeks	PCR90	MWF	VAC95	50	All	2.952	0.005	0.007	0.012	0.002	0.011	0.003	1417.189	2.626	3.335	5.961	0.953	5.308	1.370	14172	26.260	33.350	59.610	9.530	53.080	13.700
150	delta	1	60x8 weeks	PCR90	MWF	VAC95	50	All	2.901	0.005	0.020	0.026	0.004	0.023	0.003	1392.689	2.472	9.803	12.275	1.939	11.052	1.334	13927	24.720	98.030	122.750	19.390	110.520	13.340
150	delta	1	60x8 weeks	PCR90	MWF	VAC95	50	All	2.901	0.005	0.020	0.026	0.004	0.023	0.003	1392.689	2.472	9.803	12.275	1.939	11.052	1.334	13927	24.720	98.030	122.750	19.390	110.520	13.340
150	delta	1	60x8 weeks	PCR90	MWF	VAC95	50	All	2.901	0.005	0.020	0.026	0.004	0.023	0.003	1392.689	2.472	9.803	12.275	1.939	11.052	1.334	13927	24.720	98.030	122.750	19.390	110.520	13.340
150	delta	1.2	60x8 weeks	PCR90	MWF	VAC95	50	All	2.856	0.005	0.031	0.036	0.006	0.033	0.003	1371.039	2.363	15.119	17.482	2.810	15.800	1.263	13710	23.630	151.190	174.820	28.100	158.000	12.630
150	delta	1.2	60x8 weeks	PCR90	MWF	VAC95	50	All	2.856	0.005	0.031	0.036	0.006	0.033	0.003	1371.039	2.363	15.119	17.482	2.810	15.800	1.263	13710	23.630	151.190	174.820	28.100	158.000	12.630
150	delta	1.2	60x8 weeks	PCR90	MWF	VAC95	50	All	2.856	0.005	0.031	0.036	0.006	0.033	0.003	1371.039	2.363	15.119	17.482	2.810	15.800	1.263	13710	23.630	151.190	174.820	28.100	158.000	12.630
150	original	0.6	60x8 weeks	PCR90	MWF	VAC95	75	All	2.989	0.003	0.000	0.003	0.000	0.003	0.003	1434.848	1.431	0.095	1.526	0.215	1.209	1.453	14348	14.310	0.950	15.260	2.150	12.090	14.530
150	original	0.6	60x8 weeks	PCR90	MWF	VAC95	75	All	2.989	0.003	0.000	0.003	0.000	0.003	0.003	1434.848	1.431	0.095	1.526	0.215	1.209	1.453	14348	14.310	0.950	15.260	2.150	12.090	14.530
150	original	0.6	60x8 weeks	PCR90	MWF	VAC95	75	All	2.989	0.003	0.000	0.003	0.000	0.003	0.003	1434.848	1.431	0.095	1.526	0.215	1.209	1.453	14348	14.310	0.950	15.260	2.150	12.090	14.530
150	original	1	60x8 weeks	PCR90	MWF	VAC95	75	All	2.988	0.003	0.000	0.004	0.001	0.003	0.003	1434.234	1.488	0.214	1.702	0.255	1.338	1.393	14342	14.880	2.140	17.020	2.550	13.380	13.930
150	original	1	60x8 weeks	PCR90	MWF	VAC95	75	All	2.988	0.003	0.000	0.004	0.001	0.003	0.003	1434.234	1.488	0.214	1.702	0.255	1.338	1.393	14342	14.880	2.140	17.020	2.550	13.380	13.930
150	original	1	60x8 weeks	PCR90	MWF	VAC95	75	All	2.988	0.003	0.000	0.004	0.001	0.003	0.003	1434.234	1.488	0.214	1.702	0.255	1.338	1.393	14342	14.880	2.140	17.020	2.550	13.380	13.930
150	original	1.2	60x8 weeks	PCR90	MWF	VAC95	75	All	2.989	0.003	0.000	0.003	0.001	0.003	0.003	1434.492	1.478	0.198	1.676	0.241	1.292	1.411	14345	14.780	1.980	16.760	2.410	12.920	14.110
150	original	1.2	60x8 weeks	PCR90	MWF	VAC95	75	All	2.989	0.003	0.000	0.003	0.001	0.003	0.003	1434.492	1.478	0.198	1.676	0.241	1.292	1.411	14345	14.780	1.980	16.760	2.410	12.920	14.110
150	original	1.2	60x8 weeks	PCR90	MWF	VAC95	75	All	2.989	0.003	0.000	0.003	0.001	0.003	0.003	1434.492	1.478	0.198	1.676	0.241	1.292	1.411	14345	14.780	1.980	16.760	2.410	12.920	14.110
150	alpha	0.6	60x8 weeks	PCR90	MWF	VAC95	75	All	2.986	0.003	0.001	0.004	0.000	0.003	0.003	1433.394	1.531	0.264	1.795	0.180	1.678	1.397	14334	15.310	2.640	17.950	1.800	16.780	13.970
150	alpha	0.6	60x8 weeks	PCR90	MWF	VAC95	75	All	2.986	0.003	0.001	0.004	0.000	0.003	0.003	1433.394	1.531	0.264	1.795	0.180	1.678	1.397	14334	15.310	2.640	17.950	1.800	16.780	13.970
150	alpha	0.6	60x8 weeks	PCR90	MWF	VAC95	75	All	2.986	0.003	0.001	0.004	0.000	0.003	0.003	1433.394	1.531	0.264	1.795	0.180	1.678	1.397	14334	15.310	2.640	17.950	1.800	16.780	13.970
150	alpha	1	60x8 weeks	PCR90	MWF	VAC95	75	All	2.984	0.003	0.001	0.004	0.000	0.004	0.003	1432.471	1.508	0.500	2.008	0.207	1.894	1.460	14325	15.080	5.000	20.080	2.070	18.940	14.600
150	alpha	1	60x8 weeks	PCR90	MWF	VAC95	75	All	2.984	0.003	0.001	0.004	0.000	0.004	0.003	1432.471	1.508	0.500	2.008	0.207	1.894	1.460	14325	15.080	5.000	20.080	2.070	18.940	14.600
150	alpha	1	60x8 weeks	PCR90	MWF	VAC95	75	All	2.984	0.003	0.001	0.004	0.000	0.004	0.003	1432.471	1.508	0.500	2.008	0.207	1.894	1.460	14325	15.080	5.000	20.080	2.070	18.940	14.600
150	alpha	1.2	60x8 weeks	PCR90	MWF	VAC95	75	All	2.984	0.003	0.001	0.004	0.000	0.004	0.003	1432.254	1.488	0.612	2.100	0.231	1.949	1.444	14323	14.880	6.120	21.000	2.310	19.490	14.440
150	alpha	1.2	60x8 weeks	PCR90	MWF	VAC95	75	All	2.984	0.003	0.001	0.004	0.000	0.004	0.003	1432.254	1.488	0.612	2.100	0.231	1.949	1.444	14323	14.880	6.120	21.000	2.310	19.490	14.440
150	alpha	1.2	60x8 weeks	PCR90	MWF	VAC95	75	All	2.984	0.003	0.001	0.004	0.000	0.004	0.003	1432.254	1.488	0.612	2.100	0.231	1.949	1.444	14323	14.880	6.120	21.000	2.310	19.490	14.440
150	delta	0.6	60x8 weeks	PCR90	MWF	VAC95	75	All	2.980	0.004	0.002	0.005	0.001	0.005	0.003	1430.197	1.760	0.854	2.614	0.399	2.330	1.451	14302	17.600	8.540	26.140	3.990	23.300	14.510
150	delta	0.6	60x8 weeks	PCR90	MWF	VAC95	75	All	2.980	0.004	0.002	0.005	0.001	0.005	0.003	1430.197	1.760	0.854	2.614	0.399	2.330	1.451	14302	17.600	8.540	26.140	3.990	23.300	14.510
150	delta	0.6	60x8 weeks	PCR90	MWF	VAC95	75	All	2.980	0.004	0.002	0.005	0.001	0.005	0.003	1430.197	1.760	0.854	2.614	0.399	2.330	1.451	14302	17.600	8.540	26.140	3.990	23.300	14.510
150	delta	1	60x8 weeks	PCR90	MWF	VAC95	75	All	2.973	0.003	0.004	0.007	0.001	0.006	0.003	1427.086	1.595	1.844	3.439	0.504	3.055	1.367	14271	15.950	18.440	34.390	5.040	30.550	13.670
150	delta	1	60x8 weeks	PCR90	MWF	VAC95	75	All	2.973	0.003	0.004	0.007	0.001	0.006	0.003	1427.086	1.595	1.844	3.439	0.504	3.055	1.367	14271	15.950	18.440	34.390	5.040	30.550	13.670
150	delta	1	60x8 weeks	PCR90	MWF	VAC95	75	All	2.973	0.003	0.004	0.007	0.001	0.006	0.003	1427.086	1.595	1.844	3.439	0.504	3.055	1.367	14271	15.950	18.440	34.390	5.040	30.550	13.670
150	delta	1.2	60x8 weeks	PCR90	MWF	VAC95	75	All	2.965	0.004	0.006	0.009	0.001	0.008	0.003	1423.072	1.723	2.714	4.437	0.683	3.983	1.272	14231	17.230	27.140	44.370	6.830	39.830	12.720
150	delta	1.2	60x8 weeks	PCR90	MWF	VAC95	75	All	2.965	0.004	0.006	0.009	0.001	0.008	0.003	1423.072	1.723	2.714	4.437	0.683	3.983	1.272	14231	17.230	27.140	44.370	6.830	39.830	12.720
150	delta	1.2	60x8 weeks	PCR90	MWF	VAC95	75	All	2.965	0.004	0.006	0.009	0.001	0.008	0.003	1423.072	1.723	2.714	4.437	0.683	3.983	1.272	14231	17.230	27.140	44.370	6.830	39.830	12.720
150	original	0.6	60x8 weeks	PCR90	MWF	VAC95	100	All	2.999	0.001	0.000	0.001	0.000	0.000	0.003	1439.382	0.328	0.001	0.329	0.117	0.039	1.469	14394	3.280	0.010	3.290	1.170	0.390	14.690
150	original	0.6	60x8 weeks	PCR90	MWF	VAC95	100	All	2.999	0.001	0.000	0.001	0.000	0.000	0.003	1439.382	0.328	0.001	0.329	0.117	0.039	1.469	14394	3.280	0.010	3.290	1.170	0.390	14.690
150	original	0.6	60x8 weeks	PCR90	MWF	VAC95	100	All	2.999	0.001	0.000	0.001	0.000	0.000	0.003	1439.382	0.328	0.001	0.329	0.117	0.039	1.469	14394	3.280	0.010	3.290	1.170	0.390	14.690
150	original	1	60x8 weeks	PCR90	MWF	VAC95	100	All	2.999	0.001	0.000	0.001	0.000	0.000	0.003	1439.376	0.388	0.001	0.389	0.108	0.056	1.392	14394	3.880	0.010	3.890	1.080	0.560	13.920
150	original	1	60x8 weeks	PCR90	MWF	VAC95	100	All	2.999	0.001	0.000	0.001	0.000	0.000	0.003	1439.376	0.388	0.001	0.389	0.108	0.056	1.392	14394	3.880	0.010	3.890	1.080	0.560	13.920
150	original	1	60x8 weeks	PCR90	MWF	VAC95	100	All	2.999	0.001	0.000	0.001	0.000	0.000	0.003	1439.376	0.388	0.001	0.389	0.108	0.056	1.392	14394	3.880	0.010	3.890	1.080	0.560	13.920
150	original	1.2	60x8 weeks	PCR90	MWF	VAC95	100	All	2.999	0.001	0.000	0.001	0.000	0.000	0.003	1439.457	0.345	0.001	0.346	0.100	0.045	1.408	14395	3.450	0.010	3.460	1.000	0.450	14.080
150	original	1.2	60x8 weeks	PCR90	MWF	VAC95	100	All	2.999	0.001	0.000	0.001	0.000	0.000	0.003	1439.457	0.345	0.001	0.346	0.100	0.045	1.408	14395	3.450	0.010	3.460	1.000	0.450	14.080
150	original	1.2	60x8 weeks	PCR90	MWF	VAC95	100	All	2.999	0.001	0.000	0.001	0.000	0.000	0.003	1439.457	0.345	0.001	0.346	0.100	0.045	1.408	14395	3.450	0.010	3.460	1.000	0.450	14.080
150	alpha	0.6	60x8 weeks	PCR90	MWF	VAC95	100	All	2.996	0.001	0.000	0.001	0.000	0.001	0.003	1438.277	0.469	0.013	0.482	0.053	0.431	1.427	14383	4.690	0.130	4.820	0.530	4.310	14.270
150	alpha	0.6	60x8 weeks	PCR90	MWF	VAC95	100	All	2.996	0.001	0.000	0.001	0.000	0.001	0.003	1438.277	0.469	0.013	0.482	0.053	0.431	1.427	14383	4.690	0.130	4.820	0.530	4.310	14.270
150	alpha	0.6	60x8 weeks	PCR90	MWF	VAC95	100	All	2.996	0.001	0.000	0.001	0.000	0.001	0.003	1438.277	0.469	0.013	0.482	0.053	0.431	1.427	14383	4.690	0.130	4.820	0.530	4.310	14.270
150	alpha	1	60x8 weeks	PCR90	MWF	VAC95	100	All	2.996	0.001	0.000	0.001	0.000	0.001	0.003	1438.264	0.443	0.019	0.462	0.051	0.431	1.385	14383	4.430	0.190	4.620	0.510	4.310	13.850
150	alpha	1	60x8 weeks	PCR90	MWF	VAC95	100	All	2.996	0.001	0.000	0.001	0.000	0.001	0.003	1438.264	0.443	0.019	0.462	0.051	0.431	1.385	14383	4.430	0.190	4.620	0.510	4.310	13.850
150	alpha	1	60x8 weeks	PCR90	MWF	VAC95	100	All	2.996	0.001	0.000	0.001	0.000	0.001	0.003	1438.264	0.443	0.019	0.462	0.051	0.431	1.385	14383	4.430	0.190	4.620	0.510	4.310	13.850
150	alpha	1.2	60x8 weeks	PCR90	MWF	VAC95	100	All	2.996	0.001	0.000	0.001	0.000	0.001	0.003	1438.117	0.482	0.030	0.512	0.046	0.481	1.401	14381	4.820	0.300	5.120	0.460	4.810	14.010
150	alpha	1.2	60x8 weeks	PCR90	MWF	VAC95	100	All	2.996	0.001	0.000	0.001	0.000	0.001	0.003	1438.117	0.482	0.030	0.512	0.046	0.481	1.401	14381	4.820	0.300	5.120	0.460	4.810	14.010
150	alpha	1.2	60x8 weeks	PCR90	MWF	VAC95	100	All	2.996	0.001	0.000	0.001	0.000	0.001	0.003	1438.117	0.482	0.030	0.512	0.046	0.481	1.401	14381	4.820	0.300	5.120	0.460	4.810	14.010
150	delta	0.6	60x8 weeks	PCR90	MWF	VAC95	100	All	2.994	0.001	0.000	0.002	0.000	0.001	0.003	1437.243	0.683	0.065	0.748	0.112	0.656	1.465	14372	6.830	0.650	7.480	1.120	6.560	14.650
150	delta	0.6	60x8 weeks	PCR90	MWF	VAC95	100	All	2.994	0.001	0.000	0.002	0.000	0.001	0.003	1437.243	0.683	0.065	0.748	0.112	0.656	1.465	14372	6.830	0.650	7.480	1.120	6.560	14.650
150	delta	0.6	60x8 weeks	PCR90	MWF	VAC95	100	All	2.994	0.001	0.000	0.002	0.000	0.001	0.003	1437.243	0.683	0.065	0.748	0.112	0.656	1.465	14372	6.830	0.650	7.480	1.120	6.560	14.650
150	delta	1	60x8 weeks	PCR90	MWF	VAC95	100	All	2.994	0.002	0.000	0.002	0.000	0.002	0.003	1436.905	0.725	0.119	0.844	0.105	0.744	1.397	14369	7.250	1.190	8.440	1.050	7.440	13.970

150	delta	1	60x8 weeks	PCR90	MWF	VAC95	100	All	2.994	0.002	0.000	0.002	0.000	0.002	0.003	1436.905	0.725	0.119	0.844	0.105	0.744	1.397	14369	7.250	1.190	8.440	1.050	7.440	13.970
150	delta	1	60x8 weeks	PCR90	MWF	VAC95	100	All	2.994	0.002	0.000	0.002	0.000	0.002	0.003	1436.905	0.725	0.119	0.844	0.105	0.744	1.397	14369	7.250	1.190	8.440	1.050	7.440	13.970
150	delta	1.2	60x8 weeks	PCR90	MWF	VAC95	100	All	2.993	0.001	0.000	0.002	0.000	0.002	0.003	1436.753	0.718	0.172	0.890	0.109	0.799	1.488	14368	7.180	1.720	8.900	1.090	7.990	14.880
150	delta	1.2	60x8 weeks	PCR90	MWF	VAC95	100	All	2.993	0.001	0.000	0.002	0.000	0.002	0.003	1436.753	0.718	0.172	0.890	0.109	0.799	1.488	14368	7.180	1.720	8.900	1.090	7.990	14.880
150	delta	1.2	60x8 weeks	PCR90	MWF	VAC95	100	All	2.993	0.001	0.000	0.002	0.000	0.002	0.003	1436.753	0.718	0.172	0.890	0.109	0.799	1.488	14368	7.180	1.720	8.900	1.090	7.990	14.880
150	original	0.6	60x8 weeks	PCR90	MWF	VAC70	25	All	2.964	0.008	0.002	0.010	0.001	0.009	0.003	1422.594	4.011	0.952	4.963	0.641	4.211	1.385	14226	40.110	9.520	49.630	6.410	42.110	13.850
150	original	0.6	60x8 weeks	PCR90	MWF	VAC70	25	All	2.964	0.008	0.002	0.010	0.001	0.009	0.003	1422.594	4.011	0.952	4.963	0.641	4.211	1.385	14226	40.110	9.520	49.630	6.410	42.110	13.850
150	original	0.6	60x8 weeks	PCR90	MWF	VAC70	25	All	2.964	0.008	0.002	0.010	0.001	0.009	0.003	1422.594	4.011	0.952	4.963	0.641	4.211	1.385	14226	40.110	9.520	49.630	6.410	42.110	13.850
150	original	1	60x8 weeks	PCR90	MWF	VAC70	25	All	2.955	0.008	0.004	0.013	0.002	0.011	0.003	1418.421	3.945	2.082	6.027	0.748	5.193	1.407	14184	39.450	20.820	60.270	7.480	51.930	14.070
150	original	1	60x8 weeks	PCR90	MWF	VAC70	25	All	2.955	0.008	0.004	0.013	0.002	0.011	0.003	1418.421	3.945	2.082	6.027	0.748	5.193	1.407	14184	39.450	20.820	60.270	7.480	51.930	14.070
150	original	1	60x8 weeks	PCR90	MWF	VAC70	25	All	2.955	0.008	0.004	0.013	0.002	0.011	0.003	1418.421	3.945	2.082	6.027	0.748	5.193	1.407	14184	39.450	20.820	60.270	7.480	51.930	14.070
150	original	1.2	60x8 weeks	PCR90	MWF	VAC70	25	All	2.952	0.008	0.005	0.014	0.002	0.011	0.003	1417.188	3.969	2.511	6.480	0.838	5.471	1.299	14172	39.690	25.110	64.800	8.380	54.710	12.990
150	original	1.2	60x8 weeks	PCR90	MWF	VAC70	25	All	2.952	0.008	0.005	0.014	0.002	0.011	0.003	1417.188	3.969	2.511	6.480	0.838	5.471	1.299	14172	39.690	25.110	64.800	8.380	54.710	12.990
150	original	1.2	60x8 weeks	PCR90	MWF	VAC70	25	All	2.952	0.008	0.005	0.014	0.002	0.011	0.003	1417.188	3.969	2.511	6.480	0.838	5.471	1.299	14172	39.690	25.110	64.800	8.380	54.710	12.990
150	alpha	0.6	60x8 weeks	PCR90	MWF	VAC70	25	All	2.949	0.008	0.005	0.014	0.002	0.013	0.003	1415.369	3.903	2.590	6.493	0.749	6.114	1.380	14154	39.030	25.900	64.930	7.490	61.140	13.800
150	alpha	0.6	60x8 weeks	PCR90	MWF	VAC70	25	All	2.949	0.008	0.005	0.014	0.002	0.013	0.003	1415.369	3.903	2.590	6.493	0.749	6.114	1.380	14154	39.030	25.900	64.930	7.490	61.140	13.800
150	alpha	0.6	60x8 weeks	PCR90	MWF	VAC70	25	All	2.949	0.008	0.005	0.014	0.002	0.013	0.003	1415.369	3.903	2.590	6.493	0.749	6.114	1.380	14154	39.030	25.900	64.930	7.490	61.140	13.800
150	alpha	1	60x8 weeks	PCR90	MWF	VAC70	25	All	2.914	0.008	0.014	0.022	0.002	0.021	0.003	1398.936	3.784	6.860	10.644	1.181	10.142	1.296	13989	37.840	68.600	106.440	11.810	101.420	12.960
150	alpha	1	60x8 weeks	PCR90	MWF	VAC70	25	All	2.914	0.008	0.014	0.022	0.002	0.021	0.003	1398.936	3.784	6.860	10.644	1.181	10.142	1.296	13989	37.840	68.600	106.440	11.810	101.420	12.960
150	alpha	1	60x8 weeks	PCR90	MWF	VAC70	25	All	2.914	0.008	0.014	0.022	0.002	0.021	0.003	1398.936	3.784	6.860	10.644	1.181	10.142	1.296	13989	37.840	68.600	106.440	11.810	101.420	12.960
150	alpha	1.2	60x8 weeks	PCR90	MWF	VAC70	25	All	2.891	0.008	0.020	0.028	0.003	0.027	0.003	1387.750	3.670	9.769	13.439	1.574	12.732	1.267	13878	36.700	97.690	134.390	15.740	127.320	12.670
150	alpha	1.2	60x8 weeks	PCR90	MWF	VAC70	25	All	2.891	0.008	0.020	0.028	0.003	0.027	0.003	1387.750	3.670	9.769	13.439	1.574	12.732	1.267	13878	36.700	97.690	134.390	15.740	127.320	12.670
150	alpha	1.2	60x8 weeks	PCR90	MWF	VAC70	25	All	2.891	0.008	0.020	0.028	0.003	0.027	0.003	1387.750	3.670	9.769	13.439	1.574	12.732	1.267	13878	36.700	97.690	134.390	15.740	127.320	12.670
150	delta	0.6	60x8 weeks	PCR90	MWF	VAC70	25	All	2.854	0.008	0.030	0.037	0.006	0.034	0.003	1369.992	3.728	14.219	17.947	2.888	16.134	1.237	13700	37.280	142.190	179.470	28.880	161.340	12.370
150	delta	0.6	60x8 weeks	PCR90	MWF	VAC70	25	All	2.854	0.008	0.030	0.037	0.006	0.034	0.003	1369.992	3.728	14.219	17.947	2.888	16.134	1.237	13700	37.280	142.190	179.470	28.880	161.340	12.370
150	delta	0.6	60x8 weeks	PCR90	MWF	VAC70	25	All	2.854	0.008	0.030	0.037	0.006	0.034	0.003	1369.992	3.728	14.219	17.947	2.888	16.134	1.237	13700	37.280	142.190	179.470	28.880	161.340	12.370
150	delta	1	60x8 weeks	PCR90	MWF	VAC70	25	All	2.648	0.006	0.081	0.086	0.015	0.078	0.002	1270.912	2.669	38.759	41.428	7.155	37.516	0.912	12709	26.690	387.590	414.280	71.550	375.160	9.120
150	delta	1	60x8 weeks	PCR90	MWF	VAC70	25	All	2.648	0.006	0.081	0.086	0.015	0.078	0.002	1270.912	2.669	38.759	41.428	7.155	37.516	0.912	12709	26.690	387.590	414.280	71.550	375.160	9.120
150	delta	1	60x8 weeks	PCR90	MWF	VAC70	25	All	2.648	0.006	0.081	0.086	0.015	0.078	0.002	1270.912	2.669	38.759	41.428	7.155	37.516	0.912	12709	26.690	387.590	414.280	71.550	375.160	9.120
150	delta	1.2	60x8 weeks	PCR90	MWF	VAC70	25	All	2.573	0.005	0.097	0.102	0.017	0.094	0.002	1235.049	2.249	46.751	49.000	8.398	44.949	0.765	12350	22.490	467.510	490.000	83.980	449.490	7.650
150	delta	1.2	60x8 weeks	PCR90	MWF	VAC70	25	All	2.573	0.005	0.097	0.102	0.017	0.094	0.002	1235.049	2.249	46.751	49.000	8.398	44.949	0.765	12350	22.490	467.510	490.000	83.980	449.490	7.650
150	delta	1.2	60x8 weeks	PCR90	MWF	VAC70	25	All	2.573	0.005	0.097	0.102	0.017	0.094	0.002	1235.049	2.249	46.751	49.000	8.398	44.949	0.765	12350	22.490	467.510	490.000	83.980	449.490	7.650
150	original	0.6	60x8 weeks	PCR90	MWF	VAC70	50	All	2.974	0.007	0.001	0.008	0.001	0.006	0.003	1427.396	3.240	0.543	3.783	0.685	2.769	1.374	14274	32.400	5.430	37.830	6.850	27.690	13.740
150	original	0.6	60x8 weeks	PCR90	MWF	VAC70	50	All	2.974	0.007	0.001	0.008	0.001	0.006	0.003	1427.396	3.240	0.543	3.783	0.685	2.769	1.374	14274	32.400	5.430	37.830	6.850	27.690	13.740
150	original	0.6	60x8 weeks	PCR90	MWF	VAC70	50	All	2.974	0.007	0.001	0.008	0.001	0.006	0.003	1427.396	3.240	0.543	3.783	0.685	2.769	1.374	14274	32.400	5.430	37.830	6.850	27.690	13.740
150	original	1	60x8 weeks	PCR90	MWF	VAC70	50	All	2.971	0.007	0.002	0.009	0.002	0.006	0.003	1426.140	3.200	0.937	4.137	0.754	3.016	1.310	14261	32.000	9.370	41.370	7.540	30.160	13.100
150	original	1	60x8 weeks	PCR90	MWF	VAC70	50	All	2.971	0.007	0.002	0.009	0.002	0.006	0.003	1426.140	3.200	0.937	4.137	0.754	3.016	1.310	14261	32.000	9.370	41.370	7.540	30.160	13.100
150	original	1	60x8 weeks	PCR90	MWF	VAC70	50	All	2.971	0.007	0.002	0.009	0.002	0.006	0.003	1426.140	3.200	0.937	4.137	0.754	3.016	1.310	14261	32.000	9.370	41.370	7.540	30.160	13.100
150	original	1.2	60x8 weeks	PCR90	MWF	VAC70	50	All	2.970	0.007	0.002	0.009	0.002	0.007	0.003	1425.521	3.237	1.160	4.397	0.834	3.136	1.419	14255	32.370	11.600	43.970	8.340	31.360	14.190
150	original	1.2	60x8 weeks	PCR90	MWF	VAC70	50	All	2.970	0.007	0.002	0.009	0.002	0.007	0.003	1425.521	3.237	1.160	4.397	0.834	3.136	1.419	14255	32.370	11.600	43.970	8.340	31.360	14.190
150	original	1.2	60x8 weeks	PCR90	MWF	VAC70	50	All	2.970	0.007	0.002	0.009	0.002	0.007	0.003	1425.521	3.237	1.160	4.397	0.834	3.136	1.419	14255	32.370	11.600	43.970	8.340	31.360	14.190
150	alpha	0.6	60x8 weeks	PCR90	MWF	VAC70	50	All	2.966	0.006	0.003	0.009	0.001	0.009	0.003	1423.447	3.103	1.253	4.356	0.475	4.112	1.441	14234	31.030	12.530	43.560	4.750	41.120	14.410
150	alpha	0.6	60x8 weeks	PCR90	MWF	VAC70	50	All	2.966	0.006	0.003	0.009	0.001	0.009	0.003	1423.447	3.103	1.253	4.356	0.475	4.112	1.441	14234	31.030	12.530	43.560	4.750	41.120	14.410
150	alpha	0.6	60x8 weeks	PCR90	MWF	VAC70	50	All	2.966	0.006	0.003	0.009	0.001	0.009	0.003	1423.447	3.103	1.253	4.356	0.475	4.112	1.441	14234	31.030	12.530	43.560	4.750	41.120	14.410
150	alpha	1	60x8 weeks	PCR90	MWF	VAC70	50	All	2.953	0.006	0.006	0.012	0.001	0.012	0.003	1417.208	3.083	2.889	5.972	0.684	5.616	1.330	14172	30.830	28.890	59.720	6.840	56.160	13.300
150	alpha	1	60x8 weeks	PCR90	MWF	VAC70	50	All	2.953	0.006	0.006	0.012	0.001	0.012	0.003	1417.208	3.083	2.889	5.972	0.684	5.616	1.330	14172	30.830	28.890	59.720	6.840	56.160	13.300
150	alpha	1	60x8 weeks	PCR90	MWF	VAC70	50	All	2.953	0.006	0.006	0.012	0.001	0.012	0.003	1417.208	3.083	2.889	5.972	0.684	5.616	1.330	14172	30.830	28.890	59.720	6.840	56.160	13.300
150	alpha	1.2	60x8 weeks	PCR90	MWF	VAC70	50	All	2.942	0.007	0.009	0.015	0.002	0.014	0.003	1412.321	3.122	4.209	7.331	0.823	6.843	1.353	14123	31.220	42.090	73.310	8.230	68.430	13.530
150	alpha	1.2	60x8 weeks	PCR90	MWF	VAC70	50	All	2.942	0.007	0.009	0.015	0.002	0.014	0.003	1412.321	3.122	4.209	7.331	0.823	6.843	1.353	14123	31.220	42.090	73.310	8.230	68.430	13.530
150	alpha	1.2	60x8 weeks	PCR90	MWF	VAC70	50	All	2.942	0.007	0.009	0.015	0.002	0.014	0.003	1412.321	3.122	4.209	7.331	0.823	6.843	1.353	14123	31.220	42.090	73.310	8.230	68.430	13.530
150	delta	0.6	60x8 weeks	PCR90	MWF	VAC70	50	All	2.925	0.007	0.013	0.019	0.003	0.017	0.003	1404.039	3.142	6.054	9.196	1.501	8.273	1.349	14040	31.420	60.540	91.960	15.010	82.730	13.490
150	delta	0.6	60x8 weeks	PCR90	MWF	VAC70	50	All	2.925	0.007	0.013	0.019	0.003	0.017	0.003	1404.039	3.142	6.054	9.196	1.501	8.273	1.349	14040	31.420	60.540	91.960	15.010	82.730	13.490
150	delta	0.6	60x8 weeks	PCR90	MWF	VAC70	50	All	2.925	0.007	0.013	0.019	0.003	0.017	0.003	1404.039	3.142	6.054	9.196	1.501	8.273	1.349	14040	31.420	60.540	91.960	15.010	82.730	13.490
150	delta	1	60x8 weeks	PCR90	MWF	VAC70	50	All	2.812	0.006	0.042	0.048	0.008	0.043	0.002	1349.527	2.829	20.085	22.914	3.757	20.574	1.170	13495	28.290	200.850	229.140	37.570	205.740	11.700
150	delta	1	60x8 weeks	PCR90	MWF	VAC70	50	All	2.812	0.006	0.042	0.048	0.008	0.043	0.002	1349.527	2.829	20.085	22.914	3.757	20.574	1.170	13495	28.290	200.850	229.140	37.570	205.740	11.700
150	delta	1	60x8 weeks	PCR90	MWF	VAC70	50	All	2.812	0.006	0.042	0.048	0.008	0.043	0.002	1349.527	2.829	20.085	22.914	3.757	20.574	1.170	13495	28.290	200.850	229.140	37.570	205.740	11.700
150	delta	1.2	60x8 weeks	PCR90	MWF	VAC70	50	All	2.737	0.005	0.061	0.066	0.011	0.060	0.002	1313.593	2.577	29.087	31.664	5.279	28.609	1.030	13136	25.770	290.870	316.640	52.790	286.090	10.300
150	delta	1.2	60x8 weeks	PCR90	MWF	VAC70	50	All	2.737	0.005	0.061	0.066	0.011	0.060	0.002	1313.593	2.577	29.087	31.664	5.279	28.609	1.030	13136	25.770	290.870	316.640	52.790	286.090	10.300
150	delta	1.2	60x8 weeks	PCR90	MWF	VAC70	50	All	2.737	0.005	0.061	0.066	0.011	0.060	0.002	1313.593	2.577	29.087	31.664	5.279	28.609	1.030	13136	25.770	290.870	316.640	52.790	286.090	10.300
150	original	0.6	60x8 weeks	PCR90	MWF	VAC70	75	All	2.984	0.005	0.000	0.006	0.001	0.003	0.003	1432.237	2.466	0.183	2.649	0.699	1.428	1.415	14322	24.660	1.830	26.490	6.990	14.280	14.150
150	original	0.6	60x8 weeks	PCR90	MWF	VAC70	75	All	2.984	0.005	0.000	0.006	0.001	0.003	0.003	1432.237	2.466	0.183	2.649	0.699	1.428	1.415	14322	24.660	1.830	26.490	6.990	14.280	14.150
150	original	0.6	60x8 weeks	PCR90	MWF	VAC70	75	All	2.984	0.005	0.000	0.006	0.001	0.003	0.003	1432.237	2.466	0.183	2.649	0.699	1.428	1.415	14322	24.660	1.830	26.490	6.990	14.280	14.150
150	original	1	60x8 weeks	PCR90	MWF	VAC70	75	All	2.983	0.005	0.001	0.006	0.001	0.003	0.003	1432.005	2.420	0.315	2.735	0.684	1.483	1.342	14320	24.200	3.150	27.350	6.840	14.830	13.420
150	original	1	60x8 weeks	PCR90	MWF	VAC70	75	All	2.983	0.005	0.001	0.006	0.001	0.003	0.003	1432.005	2.420	0.315	2.735	0.684	1.483	1.342	14320	24.200	3.150	27.350	6.840	14.830	13.420
150	original	1	60x8 weeks	PCR90	MWF	VAC70	75	All	2.983	0.005	0.001	0.006	0.001	0.003	0.003	1432.005	2.420	0.315	2.735	0.684	1.483	1.342	14320	24.200	3.150	27.350	6.840	14.830	13.420
150	original	1.2	60x8 weeks	PCR90	MWF	VAC70	75	All	2.983	0.005	0.001	0.006	0.002	0.003	0.003	1431.725	2.404	0.399	2.803	0.730	1.503	1.361	14317	24.040	3.990	28.030	7.300	15.030	13.610
150	original	1.2	60x8 weeks	PCR90	MWF	VAC70	75	All	2.983	0.005	0.001	0.006	0.002	0.003	0.003	1431.725	2.404	0.399	2.803	0.730	1.503	1.361	14317	24.040	3.990	28.030	7.300	15.030	13.610
150	original	1.2	60x8 weeks	PCR90	MWF	VAC70	75	All	2.983	0.005	0.001	0.006	0.002	0.003	0.003	1431.725	2.404	0.399	2.803	0.730	1.503	1.361	14317	24.040	3.990	28.030	7.300	15.030	13.610
150	alpha	0.6	60x8 weeks	PCR90	MWF	VAC70	75	All	2.976	0.005	0.001	0.006	0.001	0.006	0.003	1428.709	2.445	0.619	3.064	0.323	2.827	1.378	14287	24.450	6.190	30.640	3.230	28.270	13.780
150	alpha	0.6	60x8 weeks	PCR90	MWF	VAC70	75	All	2.976	0.005	0.001	0.006	0.001	0.006	0.003	1428.709	2.445	0.619	3.064	0.323	2.827	1.378	14287	24.450	6.190	30.640	3.230	28.270	13.780
150	alpha	0.6	60x8 weeks	PCR90	MWF	VAC70	75	All	2.976	0.005	0.001	0.006	0.001	0.006	0.003	1428.709	2.445	0.619	3.064	0.323	2.827	1.378	14287	24.450	6.190	30.640	3.230	28.270	13.780

150	alpha	1	60x8 weeks	PCR90	MWF	VAC70	75	All	2.972	0.005	0.002	0.007	0.001	0.007	0.003	1426.790	2.379	1.166	3.545	0.432	3.276	1.412	14268	23.790	11.660	35.450	4.320	32.760	14.120
150	alpha	1	60x8 weeks	PCR90	MWF	VAC70	75	All	2.972	0.005	0.002	0.007	0.001	0.007	0.003	1426.790	2.379	1.166	3.545	0.432	3.276	1.412	14268	23.790	11.660	35.450	4.320	32.760	14.120
150	alpha	1	60x8 weeks	PCR90	MWF	VAC70	75	All	2.972	0.005	0.002	0.007	0.001	0.007	0.003	1426.790	2.379	1.166	3.545	0.432	3.276	1.412	14268	23.790	11.660	35.450	4.320	32.760	14.120
150	alpha	1.2	60x8 weeks	PCR90	MWF	VAC70	75	All	2.970	0.005	0.003	0.008	0.001	0.008	0.003	1425.448	2.413	1.530	3.943	0.451	3.632	1.373	14254	24.130	15.300	39.430	4.510	36.320	13.730
150	alpha	1.2	60x8 weeks	PCR90	MWF	VAC70	75	All	2.970	0.005	0.003	0.008	0.001	0.008	0.003	1425.448	2.413	1.530	3.943	0.451	3.632	1.373	14254	24.130	15.300	39.430	4.510	36.320	13.730
150	alpha	1.2	60x8 weeks	PCR90	MWF	VAC70	75	All	2.970	0.005	0.003	0.008	0.001	0.008	0.003	1425.448	2.413	1.530	3.943	0.451	3.632	1.373	14254	24.130	15.300	39.430	4.510	36.320	13.730
150	delta	0.6	60x8 weeks	PCR90	MWF	VAC70	75	All	2.962	0.006	0.004	0.010	0.002	0.009	0.003	1421.524	2.695	2.148	4.843	0.747	4.323	1.399	14215	26.950	21.480	48.430	7.470	43.230	13.990
150	delta	0.6	60x8 weeks	PCR90	MWF	VAC70	75	All	2.962	0.006	0.004	0.010	0.002	0.009	0.003	1421.524	2.695	2.148	4.843	0.747	4.323	1.399	14215	26.950	21.480	48.430	7.470	43.230	13.990
150	delta	0.6	60x8 weeks	PCR90	MWF	VAC70	75	All	2.962	0.006	0.004	0.010	0.002	0.009	0.003	1421.524	2.695	2.148	4.843	0.747	4.323	1.399	14215	26.950	21.480	48.430	7.470	43.230	13.990
150	delta	1	60x8 weeks	PCR90	MWF	VAC70	75	All	2.933	0.005	0.012	0.018	0.003	0.016	0.003	1408.025	2.604	5.940	8.544	1.320	7.576	1.384	14080	26.040	59.400	85.440	13.200	75.760	13.840
150	delta	1	60x8 weeks	PCR90	MWF	VAC70	75	All	2.933	0.005	0.012	0.018	0.003	0.016	0.003	1408.025	2.604	5.940	8.544	1.320	7.576	1.384	14080	26.040	59.400	85.440	13.200	75.760	13.840
150	delta	1	60x8 weeks	PCR90	MWF	VAC70	75	All	2.933	0.005	0.012	0.018	0.003	0.016	0.003	1408.025	2.604	5.940	8.544	1.320	7.576	1.384	14080	26.040	59.400	85.440	13.200	75.760	13.840
150	delta	1.2	60x8 weeks	PCR90	MWF	VAC70	75	All	2.900	0.005	0.021	0.026	0.004	0.023	0.003	1392.021	2.518	10.135	12.653	2.008	11.224	1.242	13920	25.180	101.350	126.530	20.080	112.240	12.420
150	delta	1.2	60x8 weeks	PCR90	MWF	VAC70	75	All	2.900	0.005	0.021	0.026	0.004	0.023	0.003	1392.021	2.518	10.135	12.653	2.008	11.224	1.242	13920	25.180	101.350	126.530	20.080	112.240	12.420
150	delta	1.2	60x8 weeks	PCR90	MWF	VAC70	75	All	2.900	0.005	0.021	0.026	0.004	0.023	0.003	1392.021	2.518	10.135	12.653	2.008	11.224	1.242	13920	25.180	101.350	126.530	20.080	112.240	12.420
150	original	0.6	60x8 weeks	PCR90	MWF	VAC70	100	All	2.993	0.003	0.000	0.003	0.001	0.000	0.003	1436.626	1.562	0.005	1.567	0.661	0.218	1.396	14366	15.620	0.050	15.670	6.610	2.180	13.960
150	original	0.6	60x8 weeks	PCR90	MWF	VAC70	100	All	2.993	0.003	0.000	0.003	0.001	0.000	0.003	1436.626	1.562	0.005	1.567	0.661	0.218	1.396	14366	15.620	0.050	15.670	6.610	2.180	13.960
150	original	0.6	60x8 weeks	PCR90	MWF	VAC70	100	All	2.993	0.003	0.000	0.003	0.001	0.000	0.003	1436.626	1.562	0.005	1.567	0.661	0.218	1.396	14366	15.620	0.050	15.670	6.610	2.180	13.960
150	original	1	60x8 weeks	PCR90	MWF	VAC70	100	All	2.992	0.003	0.000	0.004	0.001	0.001	0.003	1436.349	1.653	0.027	1.680	0.695	0.271	1.453	14363	16.530	0.270	16.800	6.950	2.710	14.530
150	original	1	60x8 weeks	PCR90	MWF	VAC70	100	All	2.992	0.003	0.000	0.004	0.001	0.001	0.003	1436.349	1.653	0.027	1.680	0.695	0.271	1.453	14363	16.530	0.270	16.800	6.950	2.710	14.530
150	original	1	60x8 weeks	PCR90	MWF	VAC70	100	All	2.992	0.003	0.000	0.004	0.001	0.001	0.003	1436.349	1.653	0.027	1.680	0.695	0.271	1.453	14363	16.530	0.270	16.800	6.950	2.710	14.530
150	original	1.2	60x8 weeks	PCR90	MWF	VAC70	100	All	2.993	0.003	0.000	0.003	0.001	0.001	0.003	1436.455	1.634	0.014	1.648	0.691	0.240	1.416	14365	16.340	0.140	16.480	6.910	2.400	14.160
150	original	1.2	60x8 weeks	PCR90	MWF	VAC70	100	All	2.993	0.003	0.000	0.003	0.001	0.001	0.003	1436.455	1.634	0.014	1.648	0.691	0.240	1.416	14365	16.340	0.140	16.480	6.910	2.400	14.160
150	original	1.2	60x8 weeks	PCR90	MWF	VAC70	100	All	2.993	0.003	0.000	0.003	0.001	0.001	0.003	1436.455	1.634	0.014	1.648	0.691	0.240	1.416	14365	16.340	0.140	16.480	6.910	2.400	14.160
150	alpha	0.6	60x8 weeks	PCR90	MWF	VAC70	100	All	2.987	0.003	0.000	0.003	0.000	0.003	0.003	1433.751	1.545	0.134	1.679	0.211	1.526	1.364	14338	15.450	1.340	16.790	2.110	15.260	13.640
150	alpha	0.6	60x8 weeks	PCR90	MWF	VAC70	100	All	2.987	0.003	0.000	0.003	0.000	0.003	0.003	1433.751	1.545	0.134	1.679	0.211	1.526	1.364	14338	15.450	1.340	16.790	2.110	15.260	13.640
150	alpha	0.6	60x8 weeks	PCR90	MWF	VAC70	100	All	2.987	0.003	0.000	0.003	0.000	0.003	0.003	1433.751	1.545	0.134	1.679	0.211	1.526	1.364	14338	15.450	1.340	16.790	2.110	15.260	13.640
150	alpha	1	60x8 weeks	PCR90	MWF	VAC70	100	All	2.985	0.004	0.001	0.004	0.000	0.004	0.003	1432.716	1.686	0.282	1.968	0.225	1.814	1.428	14327	16.860	2.820	19.680	2.250	18.140	14.280
150	alpha	1	60x8 weeks	PCR90	MWF	VAC70	100	All	2.985	0.004	0.001	0.004	0.000	0.004	0.003	1432.716	1.686	0.282	1.968	0.225	1.814	1.428	14327	16.860	2.820	19.680	2.250	18.140	14.280
150	alpha	1	60x8 weeks	PCR90	MWF	VAC70	100	All	2.985	0.004	0.001	0.004	0.000	0.004	0.003	1432.716	1.686	0.282	1.968	0.225	1.814	1.428	14327	16.860	2.820	19.680	2.250	18.140	14.280
150	alpha	1.2	60x8 weeks	PCR90	MWF	VAC70	100	All	2.985	0.003	0.001	0.004	0.000	0.004	0.003	1432.763	1.579	0.376	1.955	0.231	1.786	1.399	14328	15.790	3.760	19.550	2.310	17.860	13.990
150	alpha	1.2	60x8 weeks	PCR90	MWF	VAC70	100	All	2.985	0.003	0.001	0.004	0.000	0.004	0.003	1432.763	1.579	0.376	1.955	0.231	1.786	1.399	14328	15.790	3.760	19.550	2.310	17.860	13.990
150	alpha	1.2	60x8 weeks	PCR90	MWF	VAC70	100	All	2.985	0.003	0.001	0.004	0.000	0.004	0.003	1432.763	1.579	0.376	1.955	0.231	1.786	1.399	14328	15.790	3.760	19.550	2.310	17.860	13.990
150	delta	0.6	60x8 weeks	PCR90	MWF	VAC70	100	All	2.979	0.004	0.001	0.006	0.001	0.005	0.003	1429.850	2.052	0.704	2.756	0.428	2.430	1.404	14299	20.520	7.040	27.560	4.280	24.300	14.040
150	delta	0.6	60x8 weeks	PCR90	MWF	VAC70	100	All	2.979	0.004	0.001	0.006	0.001	0.005	0.003	1429.850	2.052	0.704	2.756	0.428	2.430	1.404	14299	20.520	7.040	27.560	4.280	24.300	14.040
150	delta	0.6	60x8 weeks	PCR90	MWF	VAC70	100	All	2.979	0.004	0.001	0.006	0.001	0.005	0.003	1429.850	2.052	0.704	2.756	0.428	2.430	1.404	14299	20.520	7.040	27.560	4.280	24.300	14.040
150	delta	1	60x8 weeks	PCR90	MWF	VAC70	100	All	2.973	0.004	0.003	0.007	0.001	0.006	0.003	1427.109	1.999	1.435	3.434	0.479	3.088	1.374	14271	19.990	14.350	34.340	4.790	30.880	13.740
150	delta	1	60x8 weeks	PCR90	MWF	VAC70	100	All	2.973	0.004	0.003	0.007	0.001	0.006	0.003	1427.109	1.999	1.435	3.434	0.479	3.088	1.374	14271	19.990	14.350	34.340	4.790	30.880	13.740
150	delta	1	60x8 weeks	PCR90	MWF	VAC70	100	All	2.973	0.004	0.003	0.007	0.001	0.006	0.003	1427.109	1.999	1.435	3.434	0.479	3.088	1.374	14271	19.990	14.350	34.340	4.790	30.880	13.740
150	delta	1.2	60x8 weeks	PCR90	MWF	VAC70	100	All	2.967	0.004	0.004	0.009	0.001	0.008	0.003	1424.312	2.074	2.118	4.192	0.651	3.682	1.432	14243	20.740	21.180	41.920	6.510	36.820	14.320
150	delta	1.2	60x8 weeks	PCR90	MWF	VAC70	100	All	2.967	0.004	0.004	0.009	0.001	0.008	0.003	1424.312	2.074	2.118	4.192	0.651	3.682	1.432	14243	20.740	21.180	41.920	6.510	36.820	14.320
150	delta	1.2	60x8 weeks	PCR90	MWF	VAC70	100	All	2.967	0.004	0.004	0.009	0.001	0.008	0.003	1424.312	2.074	2.118	4.192	0.651	3.682	1.432	14243	20.740	21.180	41.920	6.510	36.820	14.320
150	original	0.6	60x8 weeks	PCR90	MWF	VAC50	25	All	2.961	0.009	0.002	0.011	0.002	0.009	0.003	1421.343	4.285	1.081	5.366	0.785	4.383	1.398	14213	42.850	10.810	53.660	7.850	43.830	13.980
150	original	0.6	60x8 weeks	PCR90	MWF	VAC50	25	All	2.961	0.009	0.002	0.011	0.002	0.009	0.003	1421.343	4.285	1.081	5.366	0.785	4.383	1.398	14213	42.850	10.810	53.660	7.850	43.830	13.980
150	original	0.6	60x8 weeks	PCR90	MWF	VAC50	25	All	2.961	0.009	0.002	0.011	0.002	0.009	0.003	1421.343	4.285	1.081	5.366	0.785	4.383	1.398	14213	42.850	10.810	53.660	7.850	43.830	13.980
150	original	1	60x8 weeks	PCR90	MWF	VAC50	25	All	2.954	0.009	0.004	0.013	0.002	0.011	0.003	1417.869	4.268	2.080	6.348	0.945	5.154	1.379	14179	42.680	20.800	63.480	9.450	51.540	13.790
150	original	1	60x8 weeks	PCR90	MWF	VAC50	25	All	2.954	0.009	0.004	0.013	0.002	0.011	0.003	1417.869	4.268	2.080	6.348	0.945	5.154	1.379	14179	42.680	20.800	63.480	9.450	51.540	13.790
150	original	1	60x8 weeks	PCR90	MWF	VAC50	25	All	2.954	0.009	0.004	0.013	0.002	0.011	0.003	1417.869	4.268	2.080	6.348	0.945	5.154	1.379	14179	42.680	20.800	63.480	9.450	51.540	13.790
150	original	1.2	60x8 weeks	PCR90	MWF	VAC50	25	All	2.951	0.009	0.006	0.014	0.002	0.011	0.003	1416.314	4.095	2.703	6.798	1.072	5.452	1.324	14163	40.950	27.030	67.980	10.720	54.520	13.240
150	original	1.2	60x8 weeks	PCR90	MWF	VAC50	25	All	2.951	0.009	0.006	0.014	0.002	0.011	0.003	1416.314	4.095	2.703	6.798	1.072	5.452	1.324	14163	40.950	27.030	67.980	10.720	54.520	13.240
150	original	1.2	60x8 weeks	PCR90	MWF	VAC50	25	All	2.951	0.009	0.006	0.014	0.002	0.011	0.003	1416.314	4.095	2.703	6.798	1.072	5.452	1.324	14163	40.950	27.030	67.980	10.720	54.520	13.240
150	alpha	0.6	60x8 weeks	PCR90	MWF	VAC50	25	All	2.943	0.009	0.006	0.015	0.002	0.014	0.003	1412.840	4.156	2.988	7.144	0.780	6.779	1.376	14128	41.560	29.880	71.440	7.800	67.790	13.760
150	alpha	0.6	60x8 weeks	PCR90	MWF	VAC50	25	All	2.943	0.009	0.006	0.015	0.002	0.014	0.003	1412.840	4.156	2.988	7.144	0.780	6.779	1.376	14128	41.560	29.880	71.440	7.800	67.790	13.760
150	alpha	0.6	60x8 weeks	PCR90	MWF	VAC50	25	All	2.943	0.009	0.006	0.015	0.002	0.014	0.003	1412.840	4.156	2.988	7.144	0.780	6.779	1.376	14128	41.560	29.880	71.440	7.800	67.790	13.760
150	alpha	1	60x8 weeks	PCR90	MWF	VAC50	25	All	2.906	0.008	0.017	0.025	0.003	0.023	0.003	1394.895	3.972	7.946	11.918	1.323	11.232	1.323	13949	39.720	79.460	119.180	13.230	112.320	13.230
150	alpha	1	60x8 weeks	PCR90	MWF	VAC50	25	All	2.906	0.008	0.017	0.025	0.003	0.023	0.003	1394.895	3.972	7.946	11.918	1.323	11.232	1.323	13949	39.720	79.460	119.180	13.230	112.320	13.230
150	alpha	1	60x8 weeks	PCR90	MWF	VAC50	25	All	2.906	0.008	0.017	0.025	0.003	0.023	0.003	1394.895	3.972	7.946	11.918	1.323	11.232	1.323	13949	39.720	79.460	119.180	13.230	112.320	13.230
150	alpha	1.2	60x8 weeks	PCR90	MWF	VAC50	25	All	2.874	0.008	0.025	0.033	0.004	0.031	0.003	1379.496	3.795	11.891	15.686	1.862	14.786	1.266	13795	37.950	118.910	156.860	18.620	147.860	12.660
150	alpha	1.2	60x8 weeks	PCR90	MWF	VAC50	25	All	2.874	0.008	0.025	0.033	0.004	0.031	0.003	1379.496	3.795	11.891	15.686	1.862	14.786	1.266	13795	37.950	118.910	156.860	18.620	147.860	12.660
150	alpha	1.2	60x8 weeks	PCR90	MWF	VAC50	25	All	2.874	0.008	0.025	0.033	0.004	0.031	0.003	1379.496	3.795	11.891	15.686	1.862	14.786	1.266	13795	37.950	118.910	156.860	18.620	147.860	12.660
150	delta	0.6	60x8 weeks	PCR90	MWF	VAC50	25	All	2.831	0.008	0.036	0.044	0.007	0.039	0.002	1359.010	3.843	17.070	20.913	3.497	18.658	1.187	13590	38.430	170.700	209.130	34.970	186.580	11.870
150	delta	0.6	60x8 weeks	PCR90	MWF	VAC50	25	All	2.831	0.008	0.036	0.044	0.007	0.039	0.002	1359.010	3.843	17.070	20.913	3.497	18.658	1.187	13590	38.430	170.700	209.130	34.970	186.580	11.870
150	delta	0.6	60x8 weeks	PCR90	MWF	VAC50	25	All	2.831	0.008	0.036	0.044	0.007	0.039	0.002	1359.010	3.843	17.070	20.913	3.497	18.658	1.187	13590	38.430	170.700	209.130	34.970	186.580	11.870
150	delta	1	60x8 weeks	PCR90	MWF	VAC50	25	All	2.585	0.005	0.095	0.100	0.017	0.091	0.002	1240.870	2.637	45.466	48.103	8.296	43.915	0.840	12409	26.370	454.660	481.030	82.960	439.150	8.400
150	delta	1	60x8 weeks	PCR90	MWF	VAC50	25	All	2.585	0.005	0.095	0.100	0.017	0.091	0.002	1240.870	2.637	45.466	48.103	8.296	43.915	0.840	12409	26.370	454.660	481.030	82.960	439.150	8.400
150	delta	1	60x8 weeks	PCR90	MWF	VAC50	25	All	2.585	0.005	0.095	0.100	0.017	0.091	0.002	1240.870	2.637	45.466	48.103	8.296	43.915	0.840	12409	26.370	454.660	481.030	82.960	439.150	8.400
150	delta	1.2	60x8 weeks	PCR90	MWF	VAC50	25	All	2.536	0.005	0.106	0.111	0.019	0.102	0.002	1217.144	2.212	51.056	53.268	9.324	48.726	0.738	12171	22.120	510.560	532.680	93.240	487.260	7.380
150	delta	1.2	60x8 weeks	PCR90	MWF	VAC50	25	All	2.536	0.005	0.106	0.111	0.019	0.102	0.002	1217.144	2.212	51.056	53.268	9.324	48.726	0.738	12171	22.120	510.560	532.680	93.240	487.260	7.380
150	delta	1.2	60x8 weeks	PCR90	MWF	VAC50	25	All	2.536	0.005	0.106	0.111	0.019	0.102	0.002	1217.144	2.212	51.056	53.268	9.324	48.726	0.738	12171	22.120	510.560	532.680	93.240	487.260	7.380
150	original	0.6	60x8 weeks	PCR90	MWF	VAC50	50	All	2.972	0.007	0.001	0.009	0.002	0.006	0.003	1426.392	3.590	0.589	4.179	0.960	2.753	1.388	14264	35.900	5.890	41.790	9.600	27.530	13.880
150	original	0.6	60x8 weeks	PCR90	MWF	VAC50	50	All	2.972	0.007	0.001	0.009	0.002	0.006	0.003	1426.392	3.590	0.589	4.179	0.960	2.753	1.388	14264	35.900	5.890	41.790	9.600	27.530	13.880

150	original	0.6	60x8 weeks	PCR90	MWF	VAC50	50	All	2.972	0.007	0.001	0.009	0.002	0.006	0.003	1426.392	3.590	0.589	4.179	0.960	2.753	1.388	14264	35.900	5.890	41.790	9.600	27.530	13.880
150	original	1	60x8 weeks	PCR90	MWF	VAC50	50	All	2.969	0.008	0.002	0.010	0.002	0.006	0.003	1425.281	3.675	1.010	4.685	0.937	3.094	1.377	14253	36.750	10.100	46.850	9.370	30.940	13.770
150	original	1	60x8 weeks	PCR90	MWF	VAC50	50	All	2.969	0.008	0.002	0.010	0.002	0.006	0.003	1425.281	3.675	1.010	4.685	0.937	3.094	1.377	14253	36.750	10.100	46.850	9.370	30.940	13.770
150	original	1	60x8 weeks	PCR90	MWF	VAC50	50	All	2.969	0.008	0.002	0.010	0.002	0.006	0.003	1425.281	3.675	1.010	4.685	0.937	3.094	1.377	14253	36.750	10.100	46.850	9.370	30.940	13.770
150	original	1.2	60x8 weeks	PCR90	MWF	VAC50	50	All	2.966	0.008	0.003	0.011	0.002	0.007	0.003	1423.596	3.614	1.456	5.070	1.155	3.305	1.329	14236	36.140	14.560	50.700	11.550	33.050	13.290
150	original	1.2	60x8 weeks	PCR90	MWF	VAC50	50	All	2.966	0.008	0.003	0.011	0.002	0.007	0.003	1423.596	3.614	1.456	5.070	1.155	3.305	1.329	14236	36.140	14.560	50.700	11.550	33.050	13.290
150	original	1.2	60x8 weeks	PCR90	MWF	VAC50	50	All	2.966	0.008	0.003	0.011	0.002	0.007	0.003	1423.596	3.614	1.456	5.070	1.155	3.305	1.329	14236	36.140	14.560	50.700	11.550	33.050	13.290
150	alpha	0.6	60x8 weeks	PCR90	MWF	VAC50	50	All	2.959	0.007	0.003	0.011	0.001	0.010	0.003	1420.386	3.571	1.664	5.235	0.624	4.843	1.333	14204	35.710	16.640	52.350	6.240	48.430	13.330
150	alpha	0.6	60x8 weeks	PCR90	MWF	VAC50	50	All	2.959	0.007	0.003	0.011	0.001	0.010	0.003	1420.386	3.571	1.664	5.235	0.624	4.843	1.333	14204	35.710	16.640	52.350	6.240	48.430	13.330
150	alpha	0.6	60x8 weeks	PCR90	MWF	VAC50	50	All	2.959	0.007	0.003	0.011	0.001	0.010	0.003	1420.386	3.571	1.664	5.235	0.624	4.843	1.333	14204	35.710	16.640	52.350	6.240	48.430	13.330
150	alpha	1	60x8 weeks	PCR90	MWF	VAC50	50	All	2.937	0.007	0.009	0.017	0.002	0.016	0.003	1409.999	3.598	4.346	7.944	0.890	7.446	1.268	14100	35.980	43.460	79.440	8.900	74.460	12.680
150	alpha	1	60x8 weeks	PCR90	MWF	VAC50	50	All	2.937	0.007	0.009	0.017	0.002	0.016	0.003	1409.999	3.598	4.346	7.944	0.890	7.446	1.268	14100	35.980	43.460	79.440	8.900	74.460	12.680
150	alpha	1	60x8 weeks	PCR90	MWF	VAC50	50	All	2.937	0.007	0.009	0.017	0.002	0.016	0.003	1409.999	3.598	4.346	7.944	0.890	7.446	1.268	14100	35.980	43.460	79.440	8.900	74.460	12.680
150	alpha	1.2	60x8 weeks	PCR90	MWF	VAC50	50	All	2.926	0.007	0.012	0.019	0.002	0.018	0.003	1404.434	3.555	5.649	9.204	1.055	8.729	1.299	14044	35.550	56.490	92.040	10.550	87.290	12.990
150	alpha	1.2	60x8 weeks	PCR90	MWF	VAC50	50	All	2.926	0.007	0.012	0.019	0.002	0.018	0.003	1404.434	3.555	5.649	9.204	1.055	8.729	1.299	14044	35.550	56.490	92.040	10.550	87.290	12.990
150	alpha	1.2	60x8 weeks	PCR90	MWF	VAC50	50	All	2.926	0.007	0.012	0.019	0.002	0.018	0.003	1404.434	3.555	5.649	9.204	1.055	8.729	1.299	14044	35.550	56.490	92.040	10.550	87.290	12.990
150	delta	0.6	60x8 weeks	PCR90	MWF	VAC50	50	All	2.898	0.008	0.019	0.027	0.004	0.024	0.003	1391.013	3.785	8.988	12.773	2.100	11.368	1.312	13910	37.850	89.880	127.730	21.000	113.680	13.120
150	delta	0.6	60x8 weeks	PCR90	MWF	VAC50	50	All	2.898	0.008	0.019	0.027	0.004	0.024	0.003	1391.013	3.785	8.988	12.773	2.100	11.368	1.312	13910	37.850	89.880	127.730	21.000	113.680	13.120
150	delta	0.6	60x8 weeks	PCR90	MWF	VAC50	50	All	2.898	0.008	0.019	0.027	0.004	0.024	0.003	1391.013	3.785	8.988	12.773	2.100	11.368	1.312	13910	37.850	89.880	127.730	21.000	113.680	13.120
150	delta	1	60x8 weeks	PCR90	MWF	VAC50	50	All	2.718	0.006	0.064	0.071	0.012	0.064	0.002	1304.636	3.097	30.853	33.950	5.830	30.502	1.006	13046	30.970	308.530	339.500	58.300	305.020	10.060
150	delta	1	60x8 weeks	PCR90	MWF	VAC50	50	All	2.718	0.006	0.064	0.071	0.012	0.064	0.002	1304.636	3.097	30.853	33.950	5.830	30.502	1.006	13046	30.970	308.530	339.500	58.300	305.020	10.060
150	delta	1	60x8 weeks	PCR90	MWF	VAC50	50	All	2.718	0.006	0.064	0.071	0.012	0.064	0.002	1304.636	3.097	30.853	33.950	5.830	30.502	1.006	13046	30.970	308.530	339.500	58.300	305.020	10.060
150	delta	1.2	60x8 weeks	PCR90	MWF	VAC50	50	All	2.620	0.005	0.087	0.093	0.016	0.084	0.002	1257.413	2.632	41.999	44.631	7.784	40.410	0.890	12574	26.320	419.990	446.310	77.840	404.100	8.900
150	delta	1.2	60x8 weeks	PCR90	MWF	VAC50	50	All	2.620	0.005	0.087	0.093	0.016	0.084	0.002	1257.413	2.632	41.999	44.631	7.784	40.410	0.890	12574	26.320	419.990	446.310	77.840	404.100	8.900
150	delta	1.2	60x8 weeks	PCR90	MWF	VAC50	50	All	2.620	0.005	0.087	0.093	0.016	0.084	0.002	1257.413	2.632	41.999	44.631	7.784	40.410	0.890	12574	26.320	419.990	446.310	77.840	404.100	8.900
150	original	0.6	60x8 weeks	PCR90	MWF	VAC50	75	All	2.980	0.007	0.001	0.007	0.002	0.003	0.003	1430.217	3.191	0.295	3.486	1.016	1.613	1.437	14302	31.910	2.950	34.860	10.160	16.130	14.370
150	original	0.6	60x8 weeks	PCR90	MWF	VAC50	75	All	2.980	0.007	0.001	0.007	0.002	0.003	0.003	1430.217	3.191	0.295	3.486	1.016	1.613	1.437	14302	31.910	2.950	34.860	10.160	16.130	14.370
150	original	0.6	60x8 weeks	PCR90	MWF	VAC50	75	All	2.980	0.007	0.001	0.007	0.002	0.003	0.003	1430.217	3.191	0.295	3.486	1.016	1.613	1.437	14302	31.910	2.950	34.860	10.160	16.130	14.370
150	original	1	60x8 weeks	PCR90	MWF	VAC50	75	All	2.979	0.006	0.001	0.007	0.002	0.003	0.003	1430.132	3.110	0.401	3.511	1.077	1.595	1.360	14301	31.100	4.010	35.110	10.770	15.950	13.600
150	original	1	60x8 weeks	PCR90	MWF	VAC50	75	All	2.979	0.006	0.001	0.007	0.002	0.003	0.003	1430.132	3.110	0.401	3.511	1.077	1.595	1.360	14301	31.100	4.010	35.110	10.770	15.950	13.600
150	original	1	60x8 weeks	PCR90	MWF	VAC50	75	All	2.979	0.006	0.001	0.007	0.002	0.003	0.003	1430.132	3.110	0.401	3.511	1.077	1.595	1.360	14301	31.100	4.010	35.110	10.770	15.950	13.600
150	original	1.2	60x8 weeks	PCR90	MWF	VAC50	75	All	2.979	0.007	0.001	0.008	0.002	0.003	0.003	1429.708	3.183	0.575	3.758	1.103	1.675	1.329	14297	31.830	5.750	37.580	11.030	16.750	13.290
150	original	1.2	60x8 weeks	PCR90	MWF	VAC50	75	All	2.979	0.007	0.001	0.008	0.002	0.003	0.003	1429.708	3.183	0.575	3.758	1.103	1.675	1.329	14297	31.830	5.750	37.580	11.030	16.750	13.290
150	original	1.2	60x8 weeks	PCR90	MWF	VAC50	75	All	2.979	0.007	0.001	0.008	0.002	0.003	0.003	1429.708	3.183	0.575	3.758	1.103	1.675	1.329	14297	31.830	5.750	37.580	11.030	16.750	13.290
150	alpha	0.6	60x8 weeks	PCR90	MWF	VAC50	75	All	2.969	0.006	0.002	0.008	0.001	0.008	0.003	1425.026	3.084	0.934	4.018	0.470	3.698	1.383	14250	30.840	9.340	40.180	4.700	36.980	13.830
150	alpha	0.6	60x8 weeks	PCR90	MWF	VAC50	75	All	2.969	0.006	0.002	0.008	0.001	0.008	0.003	1425.026	3.084	0.934	4.018	0.470	3.698	1.383	14250	30.840	9.340	40.180	4.700	36.980	13.830
150	alpha	0.6	60x8 weeks	PCR90	MWF	VAC50	75	All	2.969	0.006	0.002	0.008	0.001	0.008	0.003	1425.026	3.084	0.934	4.018	0.470	3.698	1.383	14250	30.840	9.340	40.180	4.700	36.980	13.830
150	alpha	1	60x8 weeks	PCR90	MWF	VAC50	75	All	2.961	0.006	0.004	0.010	0.001	0.010	0.003	1421.444	3.032	1.922	4.954	0.581	4.560	1.339	14214	30.320	19.220	49.540	5.810	45.600	13.390
150	alpha	1	60x8 weeks	PCR90	MWF	VAC50	75	All	2.961	0.006	0.004	0.010	0.001	0.010	0.003	1421.444	3.032	1.922	4.954	0.581	4.560	1.339	14214	30.320	19.220	49.540	5.810	45.600	13.390
150	alpha	1	60x8 weeks	PCR90	MWF	VAC50	75	All	2.961	0.006	0.004	0.010	0.001	0.010	0.003	1421.444	3.032	1.922	4.954	0.581	4.560	1.339	14214	30.320	19.220	49.540	5.810	45.600	13.390
150	alpha	1.2	60x8 weeks	PCR90	MWF	VAC50	75	All	2.957	0.006	0.005	0.012	0.001	0.011	0.003	1419.273	3.086	2.443	5.529	0.649	5.086	1.367	14193	30.860	24.430	55.290	6.490	50.860	13.670
150	alpha	1.2	60x8 weeks	PCR90	MWF	VAC50	75	All	2.957	0.006	0.005	0.012	0.001	0.011	0.003	1419.273	3.086	2.443	5.529	0.649	5.086	1.367	14193	30.860	24.430	55.290	6.490	50.860	13.670
150	alpha	1.2	60x8 weeks	PCR90	MWF	VAC50	75	All	2.957	0.006	0.005	0.012	0.001	0.011	0.003	1419.273	3.086	2.443	5.529	0.649	5.086	1.367	14193	30.860	24.430	55.290	6.490	50.860	13.670
150	delta	0.6	60x8 weeks	PCR90	MWF	VAC50	75	All	2.937	0.007	0.009	0.016	0.003	0.015	0.003	1409.881	3.563	4.350	7.913	1.267	7.046	1.329	14099	35.630	43.500	79.130	12.670	70.460	13.290
150	delta	0.6	60x8 weeks	PCR90	MWF	VAC50	75	All	2.937	0.007	0.009	0.016	0.003	0.015	0.003	1409.881	3.563	4.350	7.913	1.267	7.046	1.329	14099	35.630	43.500	79.130	12.670	70.460	13.290
150	delta	0.6	60x8 weeks	PCR90	MWF	VAC50	75	All	2.937	0.007	0.009	0.016	0.003	0.015	0.003	1409.881	3.563	4.350	7.913	1.267	7.046	1.329	14099	35.630	43.500	79.130	12.670	70.460	13.290
150	delta	1	60x8 weeks	PCR90	MWF	VAC50	75	All	2.857	0.007	0.030	0.037	0.006	0.033	0.003	1371.530	3.216	14.469	17.685	2.894	15.778	1.239	13715	32.160	144.690	176.850	28.940	157.780	12.390
150	delta	1	60x8 weeks	PCR90	MWF	VAC50	75	All	2.857	0.007	0.030	0.037	0.006	0.033	0.003	1371.530	3.216	14.469	17.685	2.894	15.778	1.239	13715	32.160	144.690	176.850	28.940	157.780	12.390
150	delta	1	60x8 weeks	PCR90	MWF	VAC50	75	All	2.857	0.007	0.030	0.037	0.006	0.033	0.003	1371.530	3.216	14.469	17.685	2.894	15.778	1.239	13715	32.160	144.690	176.850	28.940	157.780	12.390
150	delta	1.2	60x8 weeks	PCR90	MWF	VAC50	75	All	2.778	0.006	0.051	0.057	0.010	0.050	0.002	1333.233	3.013	24.361	27.374	4.789	24.158	1.141	13332	30.130	243.610	273.740	47.890	241.580	11.410
150	delta	1.2	60x8 weeks	PCR90	MWF	VAC50	75	All	2.778	0.006	0.051	0.057	0.010	0.050	0.002	1333.233	3.013	24.361	27.374	4.789	24.158	1.141	13332	30.130	243.610	273.740	47.890	241.580	11.410
150	delta	1.2	60x8 weeks	PCR90	MWF	VAC50	75	All	2.778	0.006	0.051	0.057	0.010	0.050	0.002	1333.233	3.013	24.361	27.374	4.789	24.158	1.141	13332	30.130	243.610	273.740	47.890	241.580	11.410
150	original	0.6	60x8 weeks	PCR90	MWF	VAC50	100	All	2.988	0.005	0.000	0.005	0.002	0.001	0.003	1434.003	2.607	0.029	2.636	1.176	0.393	1.392	14340	26.070	0.290	26.360	11.760	3.930	13.920
150	original	0.6	60x8 weeks	PCR90	MWF	VAC50	100	All	2.988	0.005	0.000	0.005	0.002	0.001	0.003	1434.003	2.607	0.029	2.636	1.176	0.393	1.392	14340	26.070	0.290	26.360	11.760	3.930	13.920
150	original	0.6	60x8 weeks	PCR90	MWF	VAC50	100	All	2.988	0.005	0.000	0.005	0.002	0.001	0.003	1434.003	2.607	0.029	2.636	1.176	0.393	1.392	14340	26.070	0.290	26.360	11.760	3.930	13.920
150	original	1	60x8 weeks	PCR90	MWF	VAC50	100	All	2.988	0.005	0.000	0.005	0.002	0.001	0.003	1434.206	2.591	0.047	2.638	1.153	0.383	1.388	14342	25.910	0.470	26.380	11.530	3.830	13.880
150	original	1	60x8 weeks	PCR90	MWF	VAC50	100	All	2.988	0.005	0.000	0.005	0.002	0.001	0.003	1434.206	2.591	0.047	2.638	1.153	0.383	1.388	14342	25.910	0.470	26.380	11.530	3.830	13.880
150	original	1	60x8 weeks	PCR90	MWF	VAC50	100	All	2.988	0.005	0.000	0.005	0.002	0.001	0.003	1434.206	2.591	0.047	2.638	1.153	0.383	1.388	14342	25.910	0.470	26.380	11.530	3.830	13.880
150	original	1.2	60x8 weeks	PCR90	MWF	VAC50	100	All	2.988	0.005	0.000	0.005	0.002	0.001	0.003	1434.427	2.515	0.071	2.586	1.109	0.372	1.372	14344	25.150	0.710	25.860	11.090	3.720	13.720
150	original	1.2	60x8 weeks	PCR90	MWF	VAC50	100	All	2.988	0.005	0.000	0.005	0.002	0.001	0.003	1434.427	2.515	0.071	2.586	1.109	0.372	1.372	14344	25.150	0.710	25.860	11.090	3.720	13.720
150	original	1.2	60x8 weeks	PCR90	MWF	VAC50	100	All	2.988	0.005	0.000	0.005	0.002	0.001	0.003	1434.427	2.515	0.071	2.586	1.109	0.372	1.372	14344	25.150	0.710	25.860	11.090	3.720	13.720
150	alpha	0.6	60x8 weeks	PCR90	MWF	VAC50	100	All	2.977	0.005	0.001	0.006	0.001	0.006	0.003	1428.936	2.538	0.434	2.972	0.355	2.703	1.352	14289	25.380	4.340	29.720	3.550	27.030	13.520
150	alpha	0.6	60x8 weeks	PCR90	MWF	VAC50	100	All	2.977	0.005	0.001	0.006	0.001	0.006	0.003	1428.936	2.538	0.434	2.972	0.355	2.703	1.352	14289	25.380	4.340	29.720	3.550	27.030	13.520
150	alpha	0.6	60x8 weeks	PCR90	MWF	VAC50	100	All	2.977	0.005	0.001	0.006	0.001	0.006	0.003	1428.936	2.538	0.434	2.972	0.355	2.703	1.352	14289	25.380	4.340	29.720	3.550	27.030	13.520
150	alpha	1	60x8 weeks	PCR90	MWF	VAC50	100	All	2.975	0.005	0.002	0.007	0.001	0.006	0.003	1428.214	2.461	0.757	3.218	0.412	2.897	1.432	14282	24.610	7.570	32.180	4.120	28.970	14.320
150	alpha	1	60x8 weeks	PCR90	MWF	VAC50	100	All	2.975	0.005	0.002	0.007	0.001	0.006	0.003	1428.214	2.461	0.757	3.218	0.412	2.897	1.432	14282	24.610	7.570	32.180	4.120	28.970	14.320
150	alpha	1	60x8 weeks	PCR90	MWF	VAC50	100	All	2.975	0.005	0.002	0.007	0.001	0.006	0.003	1428.214	2.461	0.757	3.218	0.412	2.897	1.432	14282	24.610	7.570	32.180	4.120	28.970	14.320
150	alpha	1.2	60x8 weeks	PCR90	MWF	VAC50	100	All	2.972	0.005	0.002	0.007	0.001	0.007	0.003	1426.672	2.492	1.036	3.528	0.433	3.228	1.365	14267	24.920	10.360	35.280	4.330	32.280	13.650
150	alpha	1.2	60x8 weeks	PCR90	MWF	VAC50	100	All	2.972	0.005	0.002	0.007	0.001	0.007	0.003	1426.672	2.492	1.036	3.528	0.433	3.228	1.365	14267	24.920	10.360	35.280	4.330	32.280	13.650
150	alpha	1.2	60x8 weeks	PCR90	MWF	VAC50	100	All	2.972	0.005	0.002	0.007	0.001	0.007	0.003	1426.672	2.492	1.036	3.528	0.433	3.228	1.365	14267	24.920	10.360	35.280	4.330	32.280	13.650
150	delta	0.6	60x8 weeks	PCR90	MWF	VAC50	100	All	2.958	0.007	0.004	0.011	0.002	0.010	0.003	1419.865	3.232	2.124	5.356	0.836	4.712	1.406	14199	32.320	21.240	53.560	8.360	47.120	14.060

150	delta	0.6	60x8 weeks	PCR90	MWF	VAC50	100	All	2.958	0.007	0.004	0.011	0.002	0.010	0.003	1419.865	3.232	2.124	5.356	0.836	4.712	1.406	14199	32.320	21.240	53.560	8.360	47.120	14.060
150	delta	0.6	60x8 weeks	PCR90	MWF	VAC50	100	All	2.958	0.007	0.004	0.011	0.002	0.010	0.003	1419.865	3.232	2.124	5.356	0.836	4.712	1.406	14199	32.320	21.240	53.560	8.360	47.120	14.060
150	delta	1	60x8 weeks	PCR90	MWF	VAC50	100	All	2.930	0.007	0.012	0.019	0.003	0.017	0.003	1406.221	3.258	5.754	9.012	1.408	8.010	1.384	14062	32.580	57.540	90.120	14.080	80.100	13.840
150	delta	1	60x8 weeks	PCR90	MWF	VAC50	100	All	2.930	0.007	0.012	0.019	0.003	0.017	0.003	1406.221	3.258	5.754	9.012	1.408	8.010	1.384	14062	32.580	57.540	90.120	14.080	80.100	13.840
150	delta	1	60x8 weeks	PCR90	MWF	VAC50	100	All	2.930	0.007	0.012	0.019	0.003	0.017	0.003	1406.221	3.258	5.754	9.012	1.408	8.010	1.384	14062	32.580	57.540	90.120	14.080	80.100	13.840
150	delta	1.2	60x8 weeks	PCR90	MWF	VAC50	100	All	2.901	0.006	0.019	0.025	0.004	0.023	0.003	1392.287	3.063	9.063	12.126	1.977	10.924	1.266	13923	30.630	90.630	121.260	19.770	109.240	12.660
150	delta	1.2	60x8 weeks	PCR90	MWF	VAC50	100	All	2.901	0.006	0.019	0.025	0.004	0.023	0.003	1392.287	3.063	9.063	12.126	1.977	10.924	1.266	13923	30.630	90.630	121.260	19.770	109.240	12.660
150	delta	1.2	60x8 weeks	PCR90	MWF	VAC50	100	All	2.901	0.006	0.019	0.025	0.004	0.023	0.003	1392.287	3.063	9.063	12.126	1.977	10.924	1.266	13923	30.630	90.630	121.260	19.770	109.240	12.660
150	original	0.6	60x8 weeks	PCR90	M	NOVAC	0	All	0.986	0.010	0.006	0.016	0.005	0.008	0.001	473.221	4.723	2.999	7.722	2.578	3.624	0.452	4732	47.230	29.990	77.220	25.780	36.240	4.520
150	original	0.6	60x8 weeks	PCR90	M	NOVAC	0	All	0.986	0.010	0.006	0.016	0.005	0.008	0.001	473.221	4.723	2.999	7.722	2.578	3.624	0.452	4732	47.230	29.990	77.220	25.780	36.240	4.520
150	original	0.6	60x8 weeks	PCR90	M	NOVAC	0	All	0.986	0.010	0.006	0.016	0.005	0.008	0.001	473.221	4.723	2.999	7.722	2.578	3.624	0.452	4732	47.230	29.990	77.220	25.780	36.240	4.520
150	original	1	60x8 weeks	PCR90	M	NOVAC	0	All	0.978	0.009	0.016	0.025	0.009	0.012	0.001	469.290	4.544	7.555	12.099	4.112	5.647	0.437	4693	45.440	75.550	120.990	41.120	56.470	4.370
150	original	1	60x8 weeks	PCR90	M	NOVAC	0	All	0.978	0.009	0.016	0.025	0.009	0.012	0.001	469.290	4.544	7.555	12.099	4.112	5.647	0.437	4693	45.440	75.550	120.990	41.120	56.470	4.370
150	original	1	60x8 weeks	PCR90	M	NOVAC	0	All	0.978	0.009	0.016	0.025	0.009	0.012	0.001	469.290	4.544	7.555	12.099	4.112	5.647	0.437	4693	45.440	75.550	120.990	41.120	56.470	4.370
150	original	1.2	60x8 weeks	PCR90	M	NOVAC	0	All	0.972	0.009	0.023	0.032	0.011	0.015	0.001	466.599	4.366	10.881	15.247	5.169	7.118	0.476	4666	43.660	108.810	152.470	51.690	71.180	4.760
150	original	1.2	60x8 weeks	PCR90	M	NOVAC	0	All	0.972	0.009	0.023	0.032	0.011	0.015	0.001	466.599	4.366	10.881	15.247	5.169	7.118	0.476	4666	43.660	108.810	152.470	51.690	71.180	4.760
150	original	1.2	60x8 weeks	PCR90	M	NOVAC	0	All	0.972	0.009	0.023	0.032	0.011	0.015	0.001	466.599	4.366	10.881	15.247	5.169	7.118	0.476	4666	43.660	108.810	152.470	51.690	71.180	4.760
150	alpha	0.6	60x8 weeks	PCR90	M	NOVAC	0	All	0.968	0.009	0.024	0.033	0.013	0.016	0.001	464.462	4.511	11.531	16.042	6.261	7.881	0.402	4645	45.110	115.310	160.420	62.610	78.810	4.020
150	alpha	0.6	60x8 weeks	PCR90	M	NOVAC	0	All	0.968	0.009	0.024	0.033	0.013	0.016	0.001	464.462	4.511	11.531	16.042	6.261	7.881	0.402	4645	45.110	115.310	160.420	62.610	78.810	4.020
150	alpha	0.6	60x8 weeks	PCR90	M	NOVAC	0	All	0.968	0.009	0.024	0.033	0.013	0.016	0.001	464.462	4.511	11.531	16.042	6.261	7.881	0.402	4645	45.110	115.310	160.420	62.610	78.810	4.020
150	alpha	1	60x8 weeks	PCR90	M	NOVAC	0	All	0.919	0.007	0.073	0.081	0.032	0.040	0.001	440.969	3.443	35.208	38.651	15.388	19.437	0.336	4410	34.430	352.080	386.510	153.880	194.370	3.360
150	alpha	1	60x8 weeks	PCR90	M	NOVAC	0	All	0.919	0.007	0.073	0.081	0.032	0.040	0.001	440.969	3.443	35.208	38.651	15.388	19.437	0.336	4410	34.430	352.080	386.510	153.880	194.370	3.360
150	alpha	1	60x8 weeks	PCR90	M	NOVAC	0	All	0.919	0.007	0.073	0.081	0.032	0.040	0.001	440.969	3.443	35.208	38.651	15.388	19.437	0.336	4410	34.430	352.080	386.510	153.880	194.370	3.360
150	alpha	1.2	60x8 weeks	PCR90	M	NOVAC	0	All	0.893	0.006	0.094	0.100	0.041	0.052	0.001	428.440	2.863	44.925	47.788	19.534	24.743	0.283	4284	28.630	449.250	477.880	195.340	247.430	2.830
150	alpha	1.2	60x8 weeks	PCR90	M	NOVAC	0	All	0.893	0.006	0.094	0.100	0.041	0.052	0.001	428.440	2.863	44.925	47.788	19.534	24.743	0.283	4284	28.630	449.250	477.880	195.340	247.430	2.830
150	alpha	1.2	60x8 weeks	PCR90	M	NOVAC	0	All	0.893	0.006	0.094	0.100	0.041	0.052	0.001	428.440	2.863	44.925	47.788	19.534	24.743	0.283	4284	28.630	449.250	477.880	195.340	247.430	2.830
150	delta	0.6	60x8 weeks	PCR90	M	NOVAC	0	All	0.879	0.006	0.103	0.108	0.049	0.053	0.001	421.841	2.683	49.246	51.929	23.338	25.437	0.268	4218	26.830	492.460	519.290	233.380	254.370	2.680
150	delta	0.6	60x8 weeks	PCR90	M	NOVAC	0	All	0.879	0.006	0.103	0.108	0.049	0.053	0.001	421.841	2.683	49.246	51.929	23.338	25.437	0.268	4218	26.830	492.460	519.290	233.380	254.370	2.680
150	delta	0.6	60x8 weeks	PCR90	M	NOVAC	0	All	0.879	0.006	0.103	0.108	0.049	0.053	0.001	421.841	2.683	49.246	51.929	23.338	25.437	0.268	4218	26.830	492.460	519.290	233.380	254.370	2.680
150	delta	1	60x8 weeks	PCR90	M	NOVAC	0	All	0.858	0.004	0.117	0.121	0.056	0.060	0.000	412.001	1.928	56.366	58.294	26.705	28.742	0.212	4120	19.280	563.660	582.940	267.050	287.420	2.120
150	delta	1	60x8 weeks	PCR90	M	NOVAC	0	All	0.858	0.004	0.117	0.121	0.056	0.060	0.000	412.001	1.928	56.366	58.294	26.705	28.742	0.212	4120	19.280	563.660	582.940	267.050	287.420	2.120
150	delta	1	60x8 weeks	PCR90	M	NOVAC	0	All	0.858	0.004	0.117	0.121	0.056	0.060	0.000	412.001	1.928	56.366	58.294	26.705	28.742	0.212	4120	19.280	563.660	582.940	267.050	287.420	2.120
150	delta	1.2	60x8 weeks	PCR90	M	NOVAC	0	All	0.856	0.004	0.118	0.121	0.055	0.061	0.000	411.079	1.767	56.551	58.318	26.570	29.103	0.166	4111	17.670	565.510	583.180	265.700	291.030	1.660
150	delta	1.2	60x8 weeks	PCR90	M	NOVAC	0	All	0.856	0.004	0.118	0.121	0.055	0.061	0.000	411.079	1.767	56.551	58.318	26.570	29.103	0.166	4111	17.670	565.510	583.180	265.700	291.030	1.660
150	delta	1.2	60x8 weeks	PCR90	M	NOVAC	0	All	0.856	0.004	0.118	0.121	0.055	0.061	0.000	411.079	1.767	56.551	58.318	26.570	29.103	0.166	4111	17.670	565.510	583.180	265.700	291.030	1.660
150	original	0.6	60x8 weeks	PCR90	M	VAC95	25	All	0.990	0.008	0.003	0.011	0.004	0.005	0.001	475.348	3.692	1.630	5.322	1.758	2.465	0.468	4753	36.920	16.300	53.220	17.580	24.650	4.680
150	original	0.6	60x8 weeks	PCR90	M	VAC95	25	All	0.990	0.008	0.003	0.011	0.004	0.005	0.001	475.348	3.692	1.630	5.322	1.758	2.465	0.468	4753	36.920	16.300	53.220	17.580	24.650	4.680
150	original	0.6	60x8 weeks	PCR90	M	VAC95	25	All	0.990	0.008	0.003	0.011	0.004	0.005	0.001	475.348	3.692	1.630	5.322	1.758	2.465	0.468	4753	36.920	16.300	53.220	17.580	24.650	4.680
150	original	1	60x8 weeks	PCR90	M	VAC95	25	All	0.988	0.008	0.007	0.014	0.005	0.007	0.001	474.196	3.610	3.201	6.811	2.267	3.153	0.442	4742	36.100	32.010	68.110	22.670	31.530	4.420
150	original	1	60x8 weeks	PCR90	M	VAC95	25	All	0.988	0.008	0.007	0.014	0.005	0.007	0.001	474.196	3.610	3.201	6.811	2.267	3.153	0.442	4742	36.100	32.010	68.110	22.670	31.530	4.420
150	original	1	60x8 weeks	PCR90	M	VAC95	25	All	0.988	0.008	0.007	0.014	0.005	0.007	0.001	474.196	3.610	3.201	6.811	2.267	3.153	0.442	4742	36.100	32.010	68.110	22.670	31.530	4.420
150	original	1.2	60x8 weeks	PCR90	M	VAC95	25	All	0.986	0.007	0.009	0.017	0.006	0.008	0.001	473.169	3.558	4.496	8.054	2.676	3.647	0.412	4732	35.580	44.960	80.540	26.760	36.470	4.120
150	original	1.2	60x8 weeks	PCR90	M	VAC95	25	All	0.986	0.007	0.009	0.017	0.006	0.008	0.001	473.169	3.558	4.496	8.054	2.676	3.647	0.412	4732	35.580	44.960	80.540	26.760	36.470	4.120
150	original	1.2	60x8 weeks	PCR90	M	VAC95	25	All	0.986	0.007	0.009	0.017	0.006	0.008	0.001	473.169	3.558	4.496	8.054	2.676	3.647	0.412	4732	35.580	44.960	80.540	26.760	36.470	4.120
150	alpha	0.6	60x8 weeks	PCR90	M	VAC95	25	All	0.983	0.007	0.010	0.018	0.007	0.009	0.001	471.970	3.566	4.946	8.512	3.264	4.127	0.439	4720	35.660	49.460	85.120	32.640	41.270	4.390
150	alpha	0.6	60x8 weeks	PCR90	M	VAC95	25	All	0.983	0.007	0.010	0.018	0.007	0.009	0.001	471.970	3.566	4.946	8.512	3.264	4.127	0.439	4720	35.660	49.460	85.120	32.640	41.270	4.390
150	alpha	0.6	60x8 weeks	PCR90	M	VAC95	25	All	0.983	0.007	0.010	0.018	0.007	0.009	0.001	471.970	3.566	4.946	8.512	3.264	4.127	0.439	4720	35.660	49.460	85.120	32.640	41.270	4.390
150	alpha	1	60x8 weeks	PCR90	M	VAC95	25	All	0.965	0.007	0.030	0.036	0.014	0.018	0.001	462.963	3.196	14.168	17.364	6.740	8.596	0.369	4630	31.960	141.680	173.640	67.400	85.960	3.690
150	alpha	1	60x8 weeks	PCR90	M	VAC95	25	All	0.965	0.007	0.030	0.036	0.014	0.018	0.001	462.963	3.196	14.168	17.364	6.740	8.596	0.369	4630	31.960	141.680	173.640	67.400	85.960	3.690
150	alpha	1	60x8 weeks	PCR90	M	VAC95	25	All	0.965	0.007	0.030	0.036	0.014	0.018	0.001	462.963	3.196	14.168	17.364	6.740	8.596	0.369	4630	31.960	141.680	173.640	67.400	85.960	3.690
150	alpha	1.2	60x8 weeks	PCR90	M	VAC95	25	All	0.949	0.006	0.044	0.050	0.020	0.025	0.001	455.722	2.940	21.208	24.148	9.465	12.075	0.361	4557	29.400	212.080	241.480	94.650	120.750	3.610
150	alpha	1.2	60x8 weeks	PCR90	M	VAC95	25	All	0.949	0.006	0.044	0.050	0.020	0.025	0.001	455.722	2.940	21.208	24.148	9.465	12.075	0.361	4557	29.400	212.080	241.480	94.650	120.750	3.610
150	alpha	1.2	60x8 weeks	PCR90	M	VAC95	25	All	0.949	0.006	0.044	0.050	0.020	0.025	0.001	455.722	2.940	21.208	24.148	9.465	12.075	0.361	4557	29.400	212.080	241.480	94.650	120.750	3.610
150	delta	0.6	60x8 weeks	PCR90	M	VAC95	25	All	0.940	0.006	0.052	0.058	0.025	0.028	0.001	451.429	2.839	25.171	28.010	11.974	13.331	0.392	4514	28.390	251.710	280.100	119.740	133.310	3.920
150	delta	0.6	60x8 weeks	PCR90	M	VAC95	25	All	0.940	0.006	0.052	0.058	0.025	0.028	0.001	451.429	2.839	25.171	28.010	11.974	13.331	0.392	4514	28.390	251.710	280.100	119.740	133.310	3.920
150	delta	0.6	60x8 weeks	PCR90	M	VAC95	25	All	0.940	0.006	0.052	0.058	0.025	0.028	0.001	451.429	2.839	25.171	28.010	11.974	13.331	0.392	4514	28.390	251.710	280.100	119.740	133.310	3.920
150	delta	1	60x8 weeks	PCR90	M	VAC95	25	All	0.895	0.004	0.092	0.096	0.042	0.047	0.001	429.478	1.995	44.047	46.042	20.201	22.452	0.276	4295	19.950	440.470	460.420	202.010	224.520	2.760
150	delta	1	60x8 weeks	PCR90	M	VAC95	25	All	0.895	0.004	0.092	0.096	0.042	0.047	0.001	429.478	1.995	44.047	46.042	20.201	22.452	0.276	4295	19.950	440.470	460.420	202.010	224.520	2.760
150	delta	1	60x8 weeks	PCR90	M	VAC95	25	All	0.895	0.004	0.092	0.096	0.042	0.047	0.001	429.478	1.995	44.047	46.042	20.201	22.452	0.276	4295	19.950	440.470	460.420	202.010	224.520	2.760
150	delta	1.2	60x8 weeks	PCR90	M	VAC95	25	All	0.886	0.004	0.098	0.102	0.045	0.051	0.001	425.218	1.771	47.222	48.993	21.450	24.393	0.261	4252	17.710	472.220	489.930	214.500	243.930	2.610
150	delta	1.2	60x8 weeks	PCR90	M	VAC95	25	All	0.886	0.004	0.098	0.102	0.045	0.051	0.001	425.218	1.771	47.222	48.993	21.450	24.393	0.261	4252	17.710	472.220	489.930	214.500	243.930	2.610
150	delta	1.2	60x8 weeks	PCR90	M	VAC95	25	All	0.886	0.004	0.098	0.102	0.045	0.051	0.001	425.218	1.771	47.222	48.993	21.450	24.393	0.261	4252	17.710	472.220	489.930	214.500	243.930	2.610
150	original	0.6	60x8 weeks	PCR90	M	VAC95	50	All	0.995	0.005	0.001	0.007	0.002	0.003	0.001	477.405	2.578	0.595	3.173	1.056	1.334	0.533	4774	25.780	5.950	31.730	10.560	13.340	5.330
150	original	0.6	60x8 weeks	PCR90	M	VAC95	50	All	0.995	0.005	0.001	0.007	0.002	0.003	0.001	477.405	2.578	0.595	3.173	1.056	1.334	0.533	4774	25.780	5.950	31.730	10.560	13.340	5.330
150	original	0.6	60x8 weeks	PCR90	M	VAC95	50	All	0.995	0.005	0.001	0.007	0.002	0.003	0.001	477.405	2.578	0.595	3.173	1.056	1.334	0.533	4774	25.780	5.950	31.730	10.560	13.340	5.330
150	original	1	60x8 weeks	PCR90	M	VAC95	50	All	0.994	0.005	0.002	0.008	0.003	0.003	0.001	476.977	2.502	1.118	3.620	1.200	1.578	0.476	4770	25.020	11.180	36.200	12.000	15.780	4.760
150	original	1	60x8 weeks	PCR90	M	VAC95	50	All	0.994	0.005	0.002	0.008	0.003	0.003	0.001	476.977	2.502	1.118	3.620	1.200	1.578	0.476	4770	25.020	11.180	36.200	12.000	15.780	4.760
150	original	1	60x8 weeks	PCR90	M	VAC95	50	All	0.994	0.005	0.002	0.008	0.003	0.003	0.001	476.977	2.502	1.118	3.620	1.200	1.578	0.476	4770	25.020	11.180	36.200	12.000	15.780	4.760
150	original	1.2	60x8 weeks	PCR90	M	VAC95	50	All	0.993	0.005	0.003	0.008	0.003	0.004	0.001	476.598	2.518	1.464	3.982	1.329	1.744	0.438	4766	25.180	14.640	39.820	13.290	17.440	4.380
150	original	1.2	60x8 weeks	PCR90	M	VAC95	50	All	0.993	0.005	0.003	0.008	0.003	0.004	0.001	476.598	2.518	1.464	3.982	1.329	1.744	0.438	4766	25.180	14.640	39.820	13.290	17.440	4.380
150	original	1.2	60x8 weeks	PCR90	M	VAC95	50	All	0.993	0.005	0.003	0.008	0.003	0.004	0.001	476.598	2.518	1.464	3.982	1.329	1.744	0.438	4766	25.180	14.640	39.820	13.290	17.440	4.380

150	alpha	0.6	60x8 weeks	PCR90	M	VAC95	50	All	0.991	0.005	0.004	0.009	0.003	0.004	0.001	475.882	2.569	1.767	4.336	1.661	2.085	0.455	4759	25.690	17.670	43.360	16.610	20.850	4.550
150	alpha	0.6	60x8 weeks	PCR90	M	VAC95	50	All	0.991	0.005	0.004	0.009	0.003	0.004	0.001	475.882	2.569	1.767	4.336	1.661	2.085	0.455	4759	25.690	17.670	43.360	16.610	20.850	4.550
150	alpha	0.6	60x8 weeks	PCR90	M	VAC95	50	All	0.991	0.005	0.004	0.009	0.003	0.004	0.001	475.882	2.569	1.767	4.336	1.661	2.085	0.455	4759	25.690	17.670	43.360	16.610	20.850	4.550
150	alpha	1	60x8 weeks	PCR90	M	VAC95	50	All	0.987	0.005	0.008	0.014	0.005	0.007	0.001	473.616	2.566	4.068	6.634	2.555	3.204	0.481	4736	25.660	40.680	66.340	25.550	32.040	4.810
150	alpha	1	60x8 weeks	PCR90	M	VAC95	50	All	0.987	0.005	0.008	0.014	0.005	0.007	0.001	473.616	2.566	4.068	6.634	2.555	3.204	0.481	4736	25.660	40.680	66.340	25.550	32.040	4.810
150	alpha	1	60x8 weeks	PCR90	M	VAC95	50	All	0.987	0.005	0.008	0.014	0.005	0.007	0.001	473.616	2.566	4.068	6.634	2.555	3.204	0.481	4736	25.660	40.680	66.340	25.550	32.040	4.810
150	alpha	1.2	60x8 weeks	PCR90	M	VAC95	50	All	0.984	0.005	0.012	0.017	0.006	0.008	0.001	472.467	2.472	5.710	8.182	3.034	4.005	0.433	4725	24.720	57.100	81.820	30.340	40.050	4.330
150	alpha	1.2	60x8 weeks	PCR90	M	VAC95	50	All	0.984	0.005	0.012	0.017	0.006	0.008	0.001	472.467	2.472	5.710	8.182	3.034	4.005	0.433	4725	24.720	57.100	81.820	30.340	40.050	4.330
150	alpha	1.2	60x8 weeks	PCR90	M	VAC95	50	All	0.984	0.005	0.012	0.017	0.006	0.008	0.001	472.467	2.472	5.710	8.182	3.034	4.005	0.433	4725	24.720	57.100	81.820	30.340	40.050	4.330
150	delta	0.6	60x8 weeks	PCR90	M	VAC95	50	All	0.979	0.005	0.016	0.022	0.009	0.010	0.001	469.832	2.545	7.889	10.434	4.299	4.890	0.443	4698	25.450	78.890	104.340	42.990	48.900	4.430
150	delta	0.6	60x8 weeks	PCR90	M	VAC95	50	All	0.979	0.005	0.016	0.022	0.009	0.010	0.001	469.832	2.545	7.889	10.434	4.299	4.890	0.443	4698	25.450	78.890	104.340	42.990	48.900	4.430
150	delta	0.6	60x8 weeks	PCR90	M	VAC95	50	All	0.979	0.005	0.016	0.022	0.009	0.010	0.001	469.832	2.545	7.889	10.434	4.299	4.890	0.443	4698	25.450	78.890	104.340	42.990	48.900	4.430
150	delta	1	60x8 weeks	PCR90	M	VAC95	50	All	0.949	0.004	0.046	0.050	0.021	0.024	0.001	455.479	2.125	22.049	24.174	10.193	11.454	0.355	4555	21.250	220.490	241.740	101.930	114.540	3.550
150	delta	1	60x8 weeks	PCR90	M	VAC95	50	All	0.949	0.004	0.046	0.050	0.021	0.024	0.001	455.479	2.125	22.049	24.174	10.193	11.454	0.355	4555	21.250	220.490	241.740	101.930	114.540	3.550
150	delta	1	60x8 weeks	PCR90	M	VAC95	50	All	0.949	0.004	0.046	0.050	0.021	0.024	0.001	455.479	2.125	22.049	24.174	10.193	11.454	0.355	4555	21.250	220.490	241.740	101.930	114.540	3.550
150	delta	1.2	60x8 weeks	PCR90	M	VAC95	50	All	0.935	0.004	0.059	0.063	0.026	0.030	0.001	448.895	1.885	28.375	30.260	12.682	14.606	0.341	4489	18.850	283.750	302.600	126.820	146.060	3.410
150	delta	1.2	60x8 weeks	PCR90	M	VAC95	50	All	0.935	0.004	0.059	0.063	0.026	0.030	0.001	448.895	1.885	28.375	30.260	12.682	14.606	0.341	4489	18.850	283.750	302.600	126.820	146.060	3.410
150	delta	1.2	60x8 weeks	PCR90	M	VAC95	50	All	0.935	0.004	0.059	0.063	0.026	0.030	0.001	448.895	1.885	28.375	30.260	12.682	14.606	0.341	4489	18.850	283.750	302.600	126.820	146.060	3.410
150	original	0.6	60x8 weeks	PCR90	M	VAC95	75	All	0.997	0.003	0.000	0.004	0.001	0.001	0.001	478.648	1.539	0.165	1.704	0.572	0.670	0.428	4786	15.390	1.650	17.040	5.720	6.700	4.280
150	original	0.6	60x8 weeks	PCR90	M	VAC95	75	All	0.997	0.003	0.000	0.004	0.001	0.001	0.001	478.648	1.539	0.165	1.704	0.572	0.670	0.428	4786	15.390	1.650	17.040	5.720	6.700	4.280
150	original	0.6	60x8 weeks	PCR90	M	VAC95	75	All	0.997	0.003	0.000	0.004	0.001	0.001	0.001	478.648	1.539	0.165	1.704	0.572	0.670	0.428	4786	15.390	1.650	17.040	5.720	6.700	4.280
150	original	1	60x8 weeks	PCR90	M	VAC95	75	All	0.997	0.003	0.001	0.004	0.001	0.001	0.001	478.604	1.431	0.284	1.715	0.601	0.651	0.452	4786	14.310	2.840	17.150	6.010	6.510	4.520
150	original	1	60x8 weeks	PCR90	M	VAC95	75	All	0.997	0.003	0.001	0.004	0.001	0.001	0.001	478.604	1.431	0.284	1.715	0.601	0.651	0.452	4786	14.310	2.840	17.150	6.010	6.510	4.520
150	original	1	60x8 weeks	PCR90	M	VAC95	75	All	0.997	0.003	0.001	0.004	0.001	0.001	0.001	478.604	1.431	0.284	1.715	0.601	0.651	0.452	4786	14.310	2.840	17.150	6.010	6.510	4.520
150	original	1.2	60x8 weeks	PCR90	M	VAC95	75	All	0.997	0.003	0.001	0.004	0.001	0.001	0.001	478.535	1.480	0.325	1.805	0.623	0.700	0.472	4785	14.800	3.250	18.050	6.230	7.000	4.720
150	original	1.2	60x8 weeks	PCR90	M	VAC95	75	All	0.997	0.003	0.001	0.004	0.001	0.001	0.001	478.535	1.480	0.325	1.805	0.623	0.700	0.472	4785	14.800	3.250	18.050	6.230	7.000	4.720
150	original	1.2	60x8 weeks	PCR90	M	VAC95	75	All	0.997	0.003	0.001	0.004	0.001	0.001	0.001	478.535	1.480	0.325	1.805	0.623	0.700	0.472	4785	14.800	3.250	18.050	6.230	7.000	4.720
150	alpha	0.6	60x8 weeks	PCR90	M	VAC95	75	All	0.996	0.003	0.001	0.004	0.002	0.002	0.001	478.169	1.549	0.396	1.945	0.727	0.940	0.511	4782	15.490	3.960	19.450	7.270	9.400	5.110
150	alpha	0.6	60x8 weeks	PCR90	M	VAC95	75	All	0.996	0.003	0.001	0.004	0.002	0.002	0.001	478.169	1.549	0.396	1.945	0.727	0.940	0.511	4782	15.490	3.960	19.450	7.270	9.400	5.110
150	alpha	0.6	60x8 weeks	PCR90	M	VAC95	75	All	0.996	0.003	0.001	0.004	0.002	0.002	0.001	478.169	1.549	0.396	1.945	0.727	0.940	0.511	4782	15.490	3.960	19.450	7.270	9.400	5.110
150	alpha	1	60x8 weeks	PCR90	M	VAC95	75	All	0.995	0.003	0.002	0.005	0.002	0.003	0.001	477.682	1.575	0.942	2.517	0.896	1.262	0.462	4777	15.750	9.420	25.170	8.960	12.620	4.620
150	alpha	1	60x8 weeks	PCR90	M	VAC95	75	All	0.995	0.003	0.002	0.005	0.002	0.003	0.001	477.682	1.575	0.942	2.517	0.896	1.262	0.462	4777	15.750	9.420	25.170	8.960	12.620	4.620
150	alpha	1	60x8 weeks	PCR90	M	VAC95	75	All	0.995	0.003	0.002	0.005	0.002	0.003	0.001	477.682	1.575	0.942	2.517	0.896	1.262	0.462	4777	15.750	9.420	25.170	8.960	12.620	4.620
150	alpha	1.2	60x8 weeks	PCR90	M	VAC95	75	All	0.995	0.003	0.002	0.005	0.002	0.003	0.001	477.562	1.507	1.094	2.601	0.941	1.295	0.476	4776	15.070	10.940	26.010	9.410	12.950	4.760
150	alpha	1.2	60x8 weeks	PCR90	M	VAC95	75	All	0.995	0.003	0.002	0.005	0.002	0.003	0.001	477.562	1.507	1.094	2.601	0.941	1.295	0.476	4776	15.070	10.940	26.010	9.410	12.950	4.760
150	alpha	1.2	60x8 weeks	PCR90	M	VAC95	75	All	0.995	0.003	0.002	0.005	0.002	0.003	0.001	477.562	1.507	1.094	2.601	0.941	1.295	0.476	4776	15.070	10.940	26.010	9.410	12.950	4.760
150	delta	0.6	60x8 weeks	PCR90	M	VAC95	75	All	0.994	0.003	0.003	0.007	0.003	0.003	0.001	477.002	1.669	1.525	3.194	1.290	1.485	0.468	4770	16.690	15.250	31.940	12.900	14.850	4.680
150	delta	0.6	60x8 weeks	PCR90	M	VAC95	75	All	0.994	0.003	0.003	0.007	0.003	0.003	0.001	477.002	1.669	1.525	3.194	1.290	1.485	0.468	4770	16.690	15.250	31.940	12.900	14.850	4.680
150	delta	0.6	60x8 weeks	PCR90	M	VAC95	75	All	0.994	0.003	0.003	0.007	0.003	0.003	0.001	477.002	1.669	1.525	3.194	1.290	1.485	0.468	4770	16.690	15.250	31.940	12.900	14.850	4.680
150	delta	1	60x8 weeks	PCR90	M	VAC95	75	All	0.989	0.003	0.008	0.012	0.005	0.005	0.001	474.827	1.636	3.890	5.526	2.178	2.556	0.442	4748	16.360	38.900	55.260	21.780	25.560	4.420
150	delta	1	60x8 weeks	PCR90	M	VAC95	75	All	0.989	0.003	0.008	0.012	0.005	0.005	0.001	474.827	1.636	3.890	5.526	2.178	2.556	0.442	4748	16.360	38.900	55.260	21.780	25.560	4.420
150	delta	1	60x8 weeks	PCR90	M	VAC95	75	All	0.989	0.003	0.008	0.012	0.005	0.005	0.001	474.827	1.636	3.890	5.526	2.178	2.556	0.442	4748	16.360	38.900	55.260	21.780	25.560	4.420
150	delta	1.2	60x8 weeks	PCR90	M	VAC95	75	All	0.985	0.003	0.012	0.015	0.006	0.007	0.001	472.948	1.582	5.762	7.344	2.923	3.434	0.439	4729	15.820	57.620	73.440	29.230	34.340	4.390
150	delta	1.2	60x8 weeks	PCR90	M	VAC95	75	All	0.985	0.003	0.012	0.015	0.006	0.007	0.001	472.948	1.582	5.762	7.344	2.923	3.434	0.439	4729	15.820	57.620	73.440	29.230	34.340	4.390
150	delta	1.2	60x8 weeks	PCR90	M	VAC95	75	All	0.985	0.003	0.012	0.015	0.006	0.007	0.001	472.948	1.582	5.762	7.344	2.923	3.434	0.439	4729	15.820	57.620	73.440	29.230	34.340	4.390
150	original	0.6	60x8 weeks	PCR90	M	VAC95	100	All	1.000	0.001	0.000	0.001	0.000	0.000	0.001	479.824	0.361	0.001	0.362	0.124	0.013	0.487	4798	3.610	0.010	3.620	1.240	0.130	4.870
150	original	0.6	60x8 weeks	PCR90	M	VAC95	100	All	1.000	0.001	0.000	0.001	0.000	0.000	0.001	479.824	0.361	0.001	0.362	0.124	0.013	0.487	4798	3.610	0.010	3.620	1.240	0.130	4.870
150	original	0.6	60x8 weeks	PCR90	M	VAC95	100	All	1.000	0.001	0.000	0.001	0.000	0.000	0.001	479.824	0.361	0.001	0.362	0.124	0.013	0.487	4798	3.610	0.010	3.620	1.240	0.130	4.870
150	original	1	60x8 weeks	PCR90	M	VAC95	100	All	1.000	0.001	0.000	0.001	0.000	0.000	0.001	479.804	0.379	0.002	0.381	0.138	0.014	0.481	4798	3.790	0.020	3.810	1.380	0.140	4.810
150	original	1	60x8 weeks	PCR90	M	VAC95	100	All	1.000	0.001	0.000	0.001	0.000	0.000	0.001	479.804	0.379	0.002	0.381	0.138	0.014	0.481	4798	3.790	0.020	3.810	1.380	0.140	4.810
150	original	1	60x8 weeks	PCR90	M	VAC95	100	All	1.000	0.001	0.000	0.001	0.000	0.000	0.001	479.804	0.379	0.002	0.381	0.138	0.014	0.481	4798	3.790	0.020	3.810	1.380	0.140	4.810
150	original	1.2	60x8 weeks	PCR90	M	VAC95	100	All	1.000	0.001	0.000	0.001	0.000	0.000	0.001	479.796	0.386	0.000	0.386	0.143	0.020	0.464	4798	3.860	0.000	3.860	1.430	0.200	4.640
150	original	1.2	60x8 weeks	PCR90	M	VAC95	100	All	1.000	0.001	0.000	0.001	0.000	0.000	0.001	479.796	0.386	0.000	0.386	0.143	0.020	0.464	4798	3.860	0.000	3.860	1.430	0.200	4.640
150	original	1.2	60x8 weeks	PCR90	M	VAC95	100	All	1.000	0.001	0.000	0.001	0.000	0.000	0.001	479.796	0.386	0.000	0.386	0.143	0.020	0.464	4798	3.860	0.000	3.860	1.430	0.200	4.640
150	alpha	0.6	60x8 weeks	PCR90	M	VAC95	100	All	0.999	0.001	0.000	0.001	0.000	0.001	0.001	479.566	0.466	0.019	0.485	0.159	0.248	0.480	4796	4.660	0.190	4.850	1.590	2.480	4.800
150	alpha	0.6	60x8 weeks	PCR90	M	VAC95	100	All	0.999	0.001	0.000	0.001	0.000	0.001	0.001	479.566	0.466	0.019	0.485	0.159	0.248	0.480	4796	4.660	0.190	4.850	1.590	2.480	4.800
150	alpha	0.6	60x8 weeks	PCR90	M	VAC95	100	All	0.999	0.001	0.000	0.001	0.000	0.001	0.001	479.566	0.466	0.019	0.485	0.159	0.248	0.480	4796	4.660	0.190	4.850	1.590	2.480	4.800
150	alpha	1	60x8 weeks	PCR90	M	VAC95	100	All	0.999	0.001	0.000	0.001	0.000	0.000	0.001	479.560	0.441	0.039	0.480	0.167	0.226	0.500	4796	4.410	0.390	4.800	1.670	2.260	5.000
150	alpha	1	60x8 weeks	PCR90	M	VAC95	100	All	0.999	0.001	0.000	0.001	0.000	0.000	0.001	479.560	0.441	0.039	0.480	0.167	0.226	0.500	4796	4.410	0.390	4.800	1.670	2.260	5.000
150	alpha	1	60x8 weeks	PCR90	M	VAC95	100	All	0.999	0.001	0.000	0.001	0.000	0.000	0.001	479.560	0.441	0.039	0.480	0.167	0.226	0.500	4796	4.410	0.390	4.800	1.670	2.260	5.000
150	alpha	1.2	60x8 weeks	PCR90	M	VAC95	100	All	0.999	0.001	0.000	0.001	0.000	0.001	0.001	479.514	0.500	0.044	0.544	0.178	0.251	0.461	4795	5.000	0.440	5.440	1.780	2.510	4.610
150	alpha	1.2	60x8 weeks	PCR90	M	VAC95	100	All	0.999	0.001	0.000	0.001	0.000	0.001	0.001	479.514	0.500	0.044	0.544	0.178	0.251	0.461	4795	5.000	0.440	5.440	1.780	2.510	4.610
150	alpha	1.2	60x8 weeks	PCR90	M	VAC95	100	All	0.999	0.001	0.000	0.001	0.000	0.001	0.001	479.514	0.500	0.044	0.544	0.178	0.251	0.461	4795	5.000	0.440	5.440	1.780	2.510	4.610
150	delta	0.6	60x8 weeks	PCR90	M	VAC95	100	All	0.998	0.002	0.000	0.002	0.001	0.001	0.001	479.211	0.765	0.101	0.866	0.321	0.409	0.477	4792	7.650	1.010	8.660	3.210	4.090	4.770
150	delta	0.6	60x8 weeks	PCR90	M	VAC95	100	All	0.998	0.002	0.000	0.002	0.001	0.001	0.001	479.211	0.765	0.101	0.866	0.321	0.409	0.477	4792	7.650	1.010	8.660	3.210	4.090	4.770
150	delta	0.6	60x8 weeks	PCR90	M	VAC95	100	All	0.998	0.002	0.000	0.002	0.001	0.001	0.001	479.211	0.765	0.101	0.866	0.321	0.409	0.477	4792	7.650	1.010	8.660	3.210	4.090	4.770
150	delta	1	60x8 weeks	PCR90	M	VAC95	100	All	0.998	0.002	0.001	0.002	0.001	0.001	0.001	479.045	0.790	0.252	1.042	0.368	0.522	0.488	4790	7.900	2.520	10.420	3.680	5.220	4.880
150	delta	1	60x8 weeks	PCR90	M	VAC95	100	All	0.998	0.002	0.001	0.002	0.001	0.001	0.001	479.045	0.790	0.252	1.042	0.368	0.522	0.488	4790	7.900	2.520	10.420	3.680	5.220	4.880
150	delta	1	60x8 weeks	PCR90	M	VAC95	100	All	0.998	0.002	0.001	0.002	0.001	0.001	0.001	479.045	0.790	0.252	1.042	0.368	0.522	0.488	4790	7.900	2.520	10.420	3.680	5.220	4.880
150	delta	1.2	60x8 weeks	PCR90	M	VAC95	100	All	0.998	0.001	0.001	0.002	0.001	0.001	0.001	479.111	0.691	0.282	0.973	0.330	0.486	0.466	4791	6.910	2.820	9.730	3.300	4.860	4.660
150	delta	1.2	60x8 weeks	PCR90	M	VAC95	100	All	0.998	0.001	0.001	0.002	0.001	0.001	0.001	479.111	0.691	0.282	0.973	0.330	0.486	0.466	4791	6.910	2.820	9.730	3.300	4.860	4.660

150	delta	1.2	60x8 weeks	PCR90	M	VAC95	100	All	0.998	0.001	0.001	0.002	0.001	0.001	0.001	479.111	0.691	0.282	0.973	0.330	0.486	0.466	4791	6.910	2.820	9.730	3.300	4.860	4.660
150	original	0.6	60x8 weeks	PCR90	M	VAC70	25	All	0.990	0.008	0.004	0.012	0.004	0.005	0.001	475.200	3.963	1.727	5.690	1.957	2.451	0.501	4752	39.630	17.270	56.900	19.570	24.510	5.010
150	original	0.6	60x8 weeks	PCR90	M	VAC70	25	All	0.990	0.008	0.004	0.012	0.004	0.005	0.001	475.200	3.963	1.727	5.690	1.957	2.451	0.501	4752	39.630	17.270	56.900	19.570	24.510	5.010
150	original	0.6	60x8 weeks	PCR90	M	VAC70	25	All	0.990	0.008	0.004	0.012	0.004	0.005	0.001	475.200	3.963	1.727	5.690	1.957	2.451	0.501	4752	39.630	17.270	56.900	19.570	24.510	5.010
150	original	1	60x8 weeks	PCR90	M	VAC70	25	All	0.987	0.008	0.007	0.015	0.005	0.006	0.001	473.908	3.826	3.397	7.223	2.521	3.087	0.455	4739	38.260	33.970	72.230	25.210	30.870	4.550
150	original	1	60x8 weeks	PCR90	M	VAC70	25	All	0.987	0.008	0.007	0.015	0.005	0.006	0.001	473.908	3.826	3.397	7.223	2.521	3.087	0.455	4739	38.260	33.970	72.230	25.210	30.870	4.550
150	original	1	60x8 weeks	PCR90	M	VAC70	25	All	0.987	0.008	0.007	0.015	0.005	0.006	0.001	473.908	3.826	3.397	7.223	2.521	3.087	0.455	4739	38.260	33.970	72.230	25.210	30.870	4.550
150	original	1.2	60x8 weeks	PCR90	M	VAC70	25	All	0.984	0.008	0.010	0.018	0.007	0.008	0.001	472.508	3.846	4.972	8.818	3.150	3.724	0.457	4725	38.460	49.720	88.180	31.500	37.240	4.570
150	original	1.2	60x8 weeks	PCR90	M	VAC70	25	All	0.984	0.008	0.010	0.018	0.007	0.008	0.001	472.508	3.846	4.972	8.818	3.150	3.724	0.457	4725	38.460	49.720	88.180	31.500	37.240	4.570
150	original	1.2	60x8 weeks	PCR90	M	VAC70	25	All	0.984	0.008	0.010	0.018	0.007	0.008	0.001	472.508	3.846	4.972	8.818	3.150	3.724	0.457	4725	38.460	49.720	88.180	31.500	37.240	4.570
150	alpha	0.6	60x8 weeks	PCR90	M	VAC70	25	All	0.981	0.008	0.012	0.020	0.008	0.009	0.001	471.061	3.865	5.534	9.399	3.617	4.530	0.449	4711	38.650	55.340	93.990	36.170	45.300	4.490
150	alpha	0.6	60x8 weeks	PCR90	M	VAC70	25	All	0.981	0.008	0.012	0.020	0.008	0.009	0.001	471.061	3.865	5.534	9.399	3.617	4.530	0.449	4711	38.650	55.340	93.990	36.170	45.300	4.490
150	alpha	0.6	60x8 weeks	PCR90	M	VAC70	25	All	0.981	0.008	0.012	0.020	0.008	0.009	0.001	471.061	3.865	5.534	9.399	3.617	4.530	0.449	4711	38.650	55.340	93.990	36.170	45.300	4.490
150	alpha	1	60x8 weeks	PCR90	M	VAC70	25	All	0.956	0.007	0.039	0.046	0.018	0.023	0.001	458.689	3.368	18.941	22.309	8.558	10.937	0.392	4587	33.680	189.410	223.090	85.580	109.370	3.920
150	alpha	1	60x8 weeks	PCR90	M	VAC70	25	All	0.956	0.007	0.039	0.046	0.018	0.023	0.001	458.689	3.368	18.941	22.309	8.558	10.937	0.392	4587	33.680	189.410	223.090	85.580	109.370	3.920
150	alpha	1	60x8 weeks	PCR90	M	VAC70	25	All	0.956	0.007	0.039	0.046	0.018	0.023	0.001	458.689	3.368	18.941	22.309	8.558	10.937	0.392	4587	33.680	189.410	223.090	85.580	109.370	3.920
150	alpha	1.2	60x8 weeks	PCR90	M	VAC70	25	All	0.939	0.006	0.055	0.061	0.024	0.030	0.001	450.809	3.054	26.194	29.248	11.569	14.527	0.367	4508	30.540	261.940	292.480	115.690	145.270	3.670
150	alpha	1.2	60x8 weeks	PCR90	M	VAC70	25	All	0.939	0.006	0.055	0.061	0.024	0.030	0.001	450.809	3.054	26.194	29.248	11.569	14.527	0.367	4508	30.540	261.940	292.480	115.690	145.270	3.670
150	alpha	1.2	60x8 weeks	PCR90	M	VAC70	25	All	0.939	0.006	0.055	0.061	0.024	0.030	0.001	450.809	3.054	26.194	29.248	11.569	14.527	0.367	4508	30.540	261.940	292.480	115.690	145.270	3.670
150	delta	0.6	60x8 weeks	PCR90	M	VAC70	25	All	0.923	0.006	0.067	0.073	0.032	0.035	0.001	442.915	2.978	32.277	35.255	15.483	16.656	0.315	4429	29.780	322.770	352.550	154.830	166.560	3.150
150	delta	0.6	60x8 weeks	PCR90	M	VAC70	25	All	0.923	0.006	0.067	0.073	0.032	0.035	0.001	442.915	2.978	32.277	35.255	15.483	16.656	0.315	4429	29.780	322.770	352.550	154.830	166.560	3.150
150	delta	0.6	60x8 weeks	PCR90	M	VAC70	25	All	0.923	0.006	0.067	0.073	0.032	0.035	0.001	442.915	2.978	32.277	35.255	15.483	16.656	0.315	4429	29.780	322.770	352.550	154.830	166.560	3.150
150	delta	1	60x8 weeks	PCR90	M	VAC70	25	All	0.876	0.004	0.107	0.111	0.049	0.055	0.001	420.256	2.025	51.471	53.496	23.750	26.313	0.269	4203	20.250	514.710	534.960	237.500	263.130	2.690
150	delta	1	60x8 weeks	PCR90	M	VAC70	25	All	0.876	0.004	0.107	0.111	0.049	0.055	0.001	420.256	2.025	51.471	53.496	23.750	26.313	0.269	4203	20.250	514.710	534.960	237.500	263.130	2.690
150	delta	1	60x8 weeks	PCR90	M	VAC70	25	All	0.876	0.004	0.107	0.111	0.049	0.055	0.001	420.256	2.025	51.471	53.496	23.750	26.313	0.269	4203	20.250	514.710	534.960	237.500	263.130	2.690
150	delta	1.2	60x8 weeks	PCR90	M	VAC70	25	All	0.868	0.004	0.111	0.115	0.051	0.057	0.001	416.641	1.834	53.461	55.295	24.636	27.507	0.240	4166	18.340	534.610	552.950	246.360	275.070	2.400
150	delta	1.2	60x8 weeks	PCR90	M	VAC70	25	All	0.868	0.004	0.111	0.115	0.051	0.057	0.001	416.641	1.834	53.461	55.295	24.636	27.507	0.240	4166	18.340	534.610	552.950	246.360	275.070	2.400
150	delta	1.2	60x8 weeks	PCR90	M	VAC70	25	All	0.868	0.004	0.111	0.115	0.051	0.057	0.001	416.641	1.834	53.461	55.295	24.636	27.507	0.240	4166	18.340	534.610	552.950	246.360	275.070	2.400
150	original	0.6	60x8 weeks	PCR90	M	VAC70	50	All	0.993	0.007	0.002	0.008	0.003	0.003	0.001	476.797	3.221	0.801	4.022	1.462	1.420	0.498	4768	32.210	8.010	40.220	14.620	14.200	4.980
150	original	0.6	60x8 weeks	PCR90	M	VAC70	50	All	0.993	0.007	0.002	0.008	0.003	0.003	0.001	476.797	3.221	0.801	4.022	1.462	1.420	0.498	4768	32.210	8.010	40.220	14.620	14.200	4.980
150	original	0.6	60x8 weeks	PCR90	M	VAC70	50	All	0.993	0.007	0.002	0.008	0.003	0.003	0.001	476.797	3.221	0.801	4.022	1.462	1.420	0.498	4768	32.210	8.010	40.220	14.620	14.200	4.980
150	original	1	60x8 weeks	PCR90	M	VAC70	50	All	0.992	0.007	0.003	0.010	0.004	0.003	0.001	476.257	3.138	1.524	4.662	1.719	1.647	0.454	4763	31.380	15.240	46.620	17.190	16.470	4.540
150	original	1	60x8 weeks	PCR90	M	VAC70	50	All	0.992	0.007	0.003	0.010	0.004	0.003	0.001	476.257	3.138	1.524	4.662	1.719	1.647	0.454	4763	31.380	15.240	46.620	17.190	16.470	4.540
150	original	1	60x8 weeks	PCR90	M	VAC70	50	All	0.992	0.007	0.003	0.010	0.004	0.003	0.001	476.257	3.138	1.524	4.662	1.719	1.647	0.454	4763	31.380	15.240	46.620	17.190	16.470	4.540
150	original	1.2	60x8 weeks	PCR90	M	VAC70	50	All	0.991	0.007	0.004	0.011	0.004	0.004	0.001	475.815	3.216	2.097	5.313	1.940	1.839	0.488	4758	32.160	20.970	53.130	19.400	18.390	4.880
150	original	1.2	60x8 weeks	PCR90	M	VAC70	50	All	0.991	0.007	0.004	0.011	0.004	0.004	0.001	475.815	3.216	2.097	5.313	1.940	1.839	0.488	4758	32.160	20.970	53.130	19.400	18.390	4.880
150	original	1.2	60x8 weeks	PCR90	M	VAC70	50	All	0.991	0.007	0.004	0.011	0.004	0.004	0.001	475.815	3.216	2.097	5.313	1.940	1.839	0.488	4758	32.160	20.970	53.130	19.400	18.390	4.880
150	alpha	0.6	60x8 weeks	PCR90	M	VAC70	50	All	0.989	0.007	0.005	0.012	0.004	0.006	0.001	474.594	3.133	2.549	5.682	2.151	2.755	0.495	4746	31.330	25.490	56.820	21.510	27.550	4.950
150	alpha	0.6	60x8 weeks	PCR90	M	VAC70	50	All	0.989	0.007	0.005	0.012	0.004	0.006	0.001	474.594	3.133	2.549	5.682	2.151	2.755	0.495	4746	31.330	25.490	56.820	21.510	27.550	4.950
150	alpha	0.6	60x8 weeks	PCR90	M	VAC70	50	All	0.989	0.007	0.005	0.012	0.004	0.006	0.001	474.594	3.133	2.549	5.682	2.151	2.755	0.495	4746	31.330	25.490	56.820	21.510	27.550	4.950
150	alpha	1	60x8 weeks	PCR90	M	VAC70	50	All	0.981	0.006	0.014	0.020	0.007	0.010	0.001	470.970	2.893	6.653	9.546	3.562	4.697	0.452	4710	28.930	66.530	95.460	35.620	46.970	4.520
150	alpha	1	60x8 weeks	PCR90	M	VAC70	50	All	0.981	0.006	0.014	0.020	0.007	0.010	0.001	470.970	2.893	6.653	9.546	3.562	4.697	0.452	4710	28.930	66.530	95.460	35.620	46.970	4.520
150	alpha	1	60x8 weeks	PCR90	M	VAC70	50	All	0.981	0.006	0.014	0.020	0.007	0.010	0.001	470.970	2.893	6.653	9.546	3.562	4.697	0.452	4710	28.930	66.530	95.460	35.620	46.970	4.520
150	alpha	1.2	60x8 weeks	PCR90	M	VAC70	50	All	0.976	0.006	0.020	0.026	0.010	0.013	0.001	468.277	2.879	9.594	12.473	4.756	6.071	0.452	4683	28.790	95.940	124.730	47.560	60.710	4.520
150	alpha	1.2	60x8 weeks	PCR90	M	VAC70	50	All	0.976	0.006	0.020	0.026	0.010	0.013	0.001	468.277	2.879	9.594	12.473	4.756	6.071	0.452	4683	28.790	95.940	124.730	47.560	60.710	4.520
150	alpha	1.2	60x8 weeks	PCR90	M	VAC70	50	All	0.976	0.006	0.020	0.026	0.010	0.013	0.001	468.277	2.879	9.594	12.473	4.756	6.071	0.452	4683	28.790	95.940	124.730	47.560	60.710	4.520
150	delta	0.6	60x8 weeks	PCR90	M	VAC70	50	All	0.964	0.006	0.030	0.036	0.016	0.016	0.001	462.727	2.950	14.260	17.210	7.489	7.893	0.457	4627	29.500	142.600	172.100	74.890	78.930	4.570
150	delta	0.6	60x8 weeks	PCR90	M	VAC70	50	All	0.964	0.006	0.030	0.036	0.016	0.016	0.001	462.727	2.950	14.260	17.210	7.489	7.893	0.457	4627	29.500	142.600	172.100	74.890	78.930	4.570
150	delta	0.6	60x8 weeks	PCR90	M	VAC70	50	All	0.964	0.006	0.030	0.036	0.016	0.016	0.001	462.727	2.950	14.260	17.210	7.489	7.893	0.457	4627	29.500	142.600	172.100	74.890	78.930	4.570
150	delta	1	60x8 weeks	PCR90	M	VAC70	50	All	0.914	0.005	0.077	0.082	0.036	0.038	0.001	438.925	2.240	37.165	39.405	17.239	18.447	0.342	4389	22.400	371.650	394.050	172.390	184.470	3.420
150	delta	1	60x8 weeks	PCR90	M	VAC70	50	All	0.914	0.005	0.077	0.082	0.036	0.038	0.001	438.925	2.240	37.165	39.405	17.239	18.447	0.342	4389	22.400	371.650	394.050	172.390	184.470	3.420
150	delta	1	60x8 weeks	PCR90	M	VAC70	50	All	0.914	0.005	0.077	0.082	0.036	0.038	0.001	438.925	2.240	37.165	39.405	17.239	18.447	0.342	4389	22.400	371.650	394.050	172.390	184.470	3.420
150	delta	1.2	60x8 weeks	PCR90	M	VAC70	50	All	0.895	0.004	0.094	0.098	0.043	0.047	0.001	429.396	1.929	45.093	47.022	20.611	22.799	0.289	4294	19.290	450.930	470.220	206.110	227.990	2.890
150	delta	1.2	60x8 weeks	PCR90	M	VAC70	50	All	0.895	0.004	0.094	0.098	0.043	0.047	0.001	429.396	1.929	45.093	47.022	20.611	22.799	0.289	4294	19.290	450.930	470.220	206.110	227.990	2.890
150	delta	1.2	60x8 weeks	PCR90	M	VAC70	50	All	0.895	0.004	0.094	0.098	0.043	0.047	0.001	429.396	1.929	45.093	47.022	20.611	22.799	0.289	4294	19.290	450.930	470.220	206.110	227.990	2.890
150	original	0.6	60x8 weeks	PCR90	M	VAC70	75	All	0.996	0.005	0.001	0.006	0.002	0.001	0.001	477.983	2.435	0.299	2.734	1.117	0.664	0.473	4780	24.350	2.990	27.340	11.170	6.640	4.730
150	original	0.6	60x8 weeks	PCR90	M	VAC70	75	All	0.996	0.005	0.001	0.006	0.002	0.001	0.001	477.983	2.435	0.299	2.734	1.117	0.664	0.473	4780	24.350	2.990	27.340	11.170	6.640	4.730
150	original	0.6	60x8 weeks	PCR90	M	VAC70	75	All	0.996	0.005	0.001	0.006	0.002	0.001	0.001	477.983	2.435	0.299	2.734	1.117	0.664	0.473	4780	24.350	2.990	27.340	11.170	6.640	4.730
150	original	1	60x8 weeks	PCR90	M	VAC70	75	All	0.995	0.005	0.001	0.006	0.003	0.002	0.001	477.747	2.508	0.499	3.007	1.230	0.727	0.446	4777	25.080	4.990	30.070	12.300	7.270	4.460
150	original	1	60x8 weeks	PCR90	M	VAC70	75	All	0.995	0.005	0.001	0.006	0.003	0.002	0.001	477.747	2.508	0.499	3.007	1.230	0.727	0.446	4777	25.080	4.990	30.070	12.300	7.270	4.460
150	original	1	60x8 weeks	PCR90	M	VAC70	75	All	0.995	0.005	0.001	0.006	0.003	0.002	0.001	477.747	2.508	0.499	3.007	1.230	0.727	0.446	4777	25.080	4.990	30.070	12.300	7.270	4.460
150	original	1.2	60x8 weeks	PCR90	M	VAC70	75	All	0.995	0.005	0.001	0.006	0.003	0.002	0.001	477.705	2.414	0.622	3.036	1.247	0.734	0.492	4777	24.140	6.220	30.360	12.470	7.340	4.920
150	original	1.2	60x8 weeks	PCR90	M	VAC70	75	All	0.995	0.005	0.001	0.006	0.003	0.002	0.001	477.705	2.414	0.622	3.036	1.247	0.734	0.492	4777	24.140	6.220	30.360	12.470	7.340	4.920
150	original	1.2	60x8 weeks	PCR90	M	VAC70	75	All	0.995	0.005	0.001	0.006	0.003	0.002	0.001	477.705	2.414	0.622	3.036	1.247	0.734	0.492	4777	24.140	6.220	30.360	12.470	7.340	4.920
150	alpha	0.6	60x8 weeks	PCR90	M	VAC70	75	All	0.993	0.005	0.002	0.007	0.003	0.003	0.001	476.780	2.443	1.000	3.443	1.309	1.661	0.460	4768	24.430	10.000	34.430	13.090	16.610	4.600
150	alpha	0.6	60x8 weeks	PCR90	M	VAC70	75	All	0.993	0.005	0.002	0.007	0.003	0.003	0.001	476.780	2.443	1.000	3.443	1.309	1.661	0.460	4768	24.430	10.000	34.430	13.090	16.610	4.600
150	alpha	0.6	60x8 weeks	PCR90	M	VAC70	75	All	0.993	0.005	0.002	0.007	0.003	0.003	0.001	476.780	2.443	1.000	3.443	1.309	1.661	0.460	4768	24.430	10.000	34.430	13.090	16.610	4.600
150	alpha	1	60x8 weeks	PCR90	M	VAC70	75	All	0.991	0.005	0.005	0.009	0.003	0.005	0.001	475.841	2.366	2.163	4.529	1.660	2.196	0.476	4758	23.660	21.630	45.290	16.600	21.960	4.760
150	alpha	1	60x8 weeks	PCR90	M	VAC70	75	All	0.991	0.005	0.005	0.009	0.003	0.005	0.001	475.841	2.366	2.163	4.529	1.660	2.196	0.476	4758	23.660	21.630	45.290	16.600	21.960	4.760
150	alpha	1	60x8 weeks	PCR90	M	VAC70	75	All	0.991	0.005	0.005	0.009	0.003	0.005	0.001	475.841	2.366	2.163	4.529	1.660	2.196	0.476	4758	23.660	21.630	45.290	16.600	21.960	4.760
150	alpha	1.2	60x8 weeks	PCR90	M	VAC70	75	All	0.990	0.005	0.006	0.011	0.004	0.005	0.001	474.999	2.289	2.972	5.261	2.048	2.427	0.446	4750	22.890	29.720	52.610	20.480	24.270	4.460

150	alpha	1.2	60x8 weeks	PCR90	M	VAC70	75	All	0.990	0.005	0.006	0.011	0.004	0.005	0.001	474.999	2.289	2.972	5.261	2.048	2.427	0.446	4750	22.890	29.720	52.610	20.480	24.270	4.460
150	alpha	1.2	60x8 weeks	PCR90	M	VAC70	75	All	0.990	0.005	0.006	0.011	0.004	0.005	0.001	474.999	2.289	2.972	5.261	2.048	2.427	0.446	4750	22.890	29.720	52.610	20.480	24.270	4.460
150	delta	0.6	60x8 weeks	PCR90	M	VAC70	75	All	0.986	0.006	0.009	0.015	0.006	0.007	0.001	473.055	2.670	4.530	7.200	3.010	3.267	0.424	4731	26.700	45.300	72.000	30.100	32.670	4.240
150	delta	0.6	60x8 weeks	PCR90	M	VAC70	75	All	0.986	0.006	0.009	0.015	0.006	0.007	0.001	473.055	2.670	4.530	7.200	3.010	3.267	0.424	4731	26.700	45.300	72.000	30.100	32.670	4.240
150	delta	0.6	60x8 weeks	PCR90	M	VAC70	75	All	0.986	0.006	0.009	0.015	0.006	0.007	0.001	473.055	2.670	4.530	7.200	3.010	3.267	0.424	4731	26.700	45.300	72.000	30.100	32.670	4.240
150	delta	1	60x8 weeks	PCR90	M	VAC70	75	All	0.963	0.005	0.033	0.038	0.016	0.017	0.001	462.284	2.364	15.850	18.214	7.716	8.345	0.396	4623	23.640	158.500	182.140	77.160	83.450	3.960
150	delta	1	60x8 weeks	PCR90	M	VAC70	75	All	0.963	0.005	0.033	0.038	0.016	0.017	0.001	462.284	2.364	15.850	18.214	7.716	8.345	0.396	4623	23.640	158.500	182.140	77.160	83.450	3.960
150	delta	1	60x8 weeks	PCR90	M	VAC70	75	All	0.963	0.005	0.033	0.038	0.016	0.017	0.001	462.284	2.364	15.850	18.214	7.716	8.345	0.396	4623	23.640	158.500	182.140	77.160	83.450	3.960
150	delta	1.2	60x8 weeks	PCR90	M	VAC70	75	All	0.947	0.004	0.048	0.053	0.023	0.024	0.001	454.581	2.088	23.131	25.219	10.851	11.718	0.371	4546	20.880	231.310	252.190	108.510	117.180	3.710
150	delta	1.2	60x8 weeks	PCR90	M	VAC70	75	All	0.947	0.004	0.048	0.053	0.023	0.024	0.001	454.581	2.088	23.131	25.219	10.851	11.718	0.371	4546	20.880	231.310	252.190	108.510	117.180	3.710
150	delta	1.2	60x8 weeks	PCR90	M	VAC70	75	All	0.947	0.004	0.048	0.053	0.023	0.024	0.001	454.581	2.088	23.131	25.219	10.851	11.718	0.371	4546	20.880	231.310	252.190	108.510	117.180	3.710
150	original	0.6	60x8 weeks	PCR90	M	VAC70	100	All	0.998	0.003	0.000	0.003	0.002	0.000	0.001	479.057	1.582	0.013	1.595	0.721	0.084	0.506	4791	15.820	0.130	15.950	7.210	0.840	5.060
150	original	0.6	60x8 weeks	PCR90	M	VAC70	100	All	0.998	0.003	0.000	0.003	0.002	0.000	0.001	479.057	1.582	0.013	1.595	0.721	0.084	0.506	4791	15.820	0.130	15.950	7.210	0.840	5.060
150	original	0.6	60x8 weeks	PCR90	M	VAC70	100	All	0.998	0.003	0.000	0.003	0.002	0.000	0.001	479.057	1.582	0.013	1.595	0.721	0.084	0.506	4791	15.820	0.130	15.950	7.210	0.840	5.060
150	original	1	60x8 weeks	PCR90	M	VAC70	100	All	0.998	0.003	0.000	0.003	0.002	0.000	0.001	478.954	1.594	0.027	1.621	0.767	0.076	0.465	4790	15.940	0.270	16.210	7.670	0.760	4.650
150	original	1	60x8 weeks	PCR90	M	VAC70	100	All	0.998	0.003	0.000	0.003	0.002	0.000	0.001	478.954	1.594	0.027	1.621	0.767	0.076	0.465	4790	15.940	0.270	16.210	7.670	0.760	4.650
150	original	1	60x8 weeks	PCR90	M	VAC70	100	All	0.998	0.003	0.000	0.003	0.002	0.000	0.001	478.954	1.594	0.027	1.621	0.767	0.076	0.465	4790	15.940	0.270	16.210	7.670	0.760	4.650
150	original	1.2	60x8 weeks	PCR90	M	VAC70	100	All	0.998	0.003	0.000	0.003	0.001	0.000	0.001	479.036	1.558	0.014	1.572	0.708	0.073	0.452	4790	15.580	0.140	15.720	7.080	0.730	4.520
150	original	1.2	60x8 weeks	PCR90	M	VAC70	100	All	0.998	0.003	0.000	0.003	0.001	0.000	0.001	479.036	1.558	0.014	1.572	0.708	0.073	0.452	4790	15.580	0.140	15.720	7.080	0.730	4.520
150	original	1.2	60x8 weeks	PCR90	M	VAC70	100	All	0.998	0.003	0.000	0.003	0.001	0.000	0.001	479.036	1.558	0.014	1.572	0.708	0.073	0.452	4790	15.580	0.140	15.720	7.080	0.730	4.520
150	alpha	0.6	60x8 weeks	PCR90	M	VAC70	100	All	0.996	0.003	0.001	0.004	0.001	0.002	0.001	478.221	1.677	0.271	1.948	0.713	0.936	0.457	4782	16.770	2.710	19.480	7.130	9.360	4.570
150	alpha	0.6	60x8 weeks	PCR90	M	VAC70	100	All	0.996	0.003	0.001	0.004	0.001	0.002	0.001	478.221	1.677	0.271	1.948	0.713	0.936	0.457	4782	16.770	2.710	19.480	7.130	9.360	4.570
150	alpha	0.6	60x8 weeks	PCR90	M	VAC70	100	All	0.996	0.003	0.001	0.004	0.001	0.002	0.001	478.221	1.677	0.271	1.948	0.713	0.936	0.457	4782	16.770	2.710	19.480	7.130	9.360	4.570
150	alpha	1	60x8 weeks	PCR90	M	VAC70	100	All	0.996	0.003	0.001	0.004	0.002	0.002	0.001	478.021	1.589	0.509	2.098	0.812	0.957	0.482	4780	15.890	5.090	20.980	8.120	9.570	4.820
150	alpha	1	60x8 weeks	PCR90	M	VAC70	100	All	0.996	0.003	0.001	0.004	0.002	0.002	0.001	478.021	1.589	0.509	2.098	0.812	0.957	0.482	4780	15.890	5.090	20.980	8.120	9.570	4.820
150	alpha	1	60x8 weeks	PCR90	M	VAC70	100	All	0.996	0.003	0.001	0.004	0.002	0.002	0.001	478.021	1.589	0.509	2.098	0.812	0.957	0.482	4780	15.890	5.090	20.980	8.120	9.570	4.820
150	alpha	1.2	60x8 weeks	PCR90	M	VAC70	100	All	0.996	0.003	0.001	0.004	0.002	0.002	0.001	478.060	1.568	0.538	2.106	0.793	0.964	0.483	4781	15.680	5.380	21.060	7.930	9.640	4.830
150	alpha	1.2	60x8 weeks	PCR90	M	VAC70	100	All	0.996	0.003	0.001	0.004	0.002	0.002	0.001	478.060	1.568	0.538	2.106	0.793	0.964	0.483	4781	15.680	5.380	21.060	7.930	9.640	4.830
150	alpha	1.2	60x8 weeks	PCR90	M	VAC70	100	All	0.996	0.003	0.001	0.004	0.002	0.002	0.001	478.060	1.568	0.538	2.106	0.793	0.964	0.483	4781	15.680	5.380	21.060	7.930	9.640	4.830
150	delta	0.6	60x8 weeks	PCR90	M	VAC70	100	All	0.993	0.004	0.003	0.007	0.003	0.003	0.001	476.817	2.039	1.281	3.320	1.348	1.539	0.437	4768	20.390	12.810	33.200	13.480	15.390	4.370
150	delta	0.6	60x8 weeks	PCR90	M	VAC70	100	All	0.993	0.004	0.003	0.007	0.003	0.003	0.001	476.817	2.039	1.281	3.320	1.348	1.539	0.437	4768	20.390	12.810	33.200	13.480	15.390	4.370
150	delta	0.6	60x8 weeks	PCR90	M	VAC70	100	All	0.993	0.004	0.003	0.007	0.003	0.003	0.001	476.817	2.039	1.281	3.320	1.348	1.539	0.437	4768	20.390	12.810	33.200	13.480	15.390	4.370
150	delta	1	60x8 weeks	PCR90	M	VAC70	100	All	0.990	0.004	0.007	0.011	0.004	0.005	0.001	475.045	1.980	3.151	5.131	2.154	2.289	0.480	4750	19.800	31.510	51.310	21.540	22.890	4.800
150	delta	1	60x8 weeks	PCR90	M	VAC70	100	All	0.990	0.004	0.007	0.011	0.004	0.005	0.001	475.045	1.980	3.151	5.131	2.154	2.289	0.480	4750	19.800	31.510	51.310	21.540	22.890	4.800
150	delta	1	60x8 weeks	PCR90	M	VAC70	100	All	0.990	0.004	0.007	0.011	0.004	0.005	0.001	475.045	1.980	3.151	5.131	2.154	2.289	0.480	4750	19.800	31.510	51.310	21.540	22.890	4.800
150	delta	1.2	60x8 weeks	PCR90	M	VAC70	100	All	0.988	0.004	0.009	0.013	0.005	0.006	0.001	474.017	1.949	4.507	6.456	2.615	2.929	0.450	4740	19.490	45.070	64.560	26.150	29.290	4.500
150	delta	1.2	60x8 weeks	PCR90	M	VAC70	100	All	0.988	0.004	0.009	0.013	0.005	0.006	0.001	474.017	1.949	4.507	6.456	2.615	2.929	0.450	4740	19.490	45.070	64.560	26.150	29.290	4.500
150	delta	1.2	60x8 weeks	PCR90	M	VAC70	100	All	0.988	0.004	0.009	0.013	0.005	0.006	0.001	474.017	1.949	4.507	6.456	2.615	2.929	0.450	4740	19.490	45.070	64.560	26.150	29.290	4.500
150	original	0.6	60x8 weeks	PCR90	M	VAC50	25	All	0.990	0.009	0.004	0.013	0.004	0.005	0.001	475.077	4.261	1.774	6.035	2.098	2.453	0.444	4751	42.610	17.740	60.350	20.980	24.530	4.440
150	original	0.6	60x8 weeks	PCR90	M	VAC50	25	All	0.990	0.009	0.004	0.013	0.004	0.005	0.001	475.077	4.261	1.774	6.035	2.098	2.453	0.444	4751	42.610	17.740	60.350	20.980	24.530	4.440
150	original	0.6	60x8 weeks	PCR90	M	VAC50	25	All	0.990	0.009	0.004	0.013	0.004	0.005	0.001	475.077	4.261	1.774	6.035	2.098	2.453	0.444	4751	42.610	17.740	60.350	20.980	24.530	4.440
150	original	1	60x8 weeks	PCR90	M	VAC50	25	All	0.986	0.008	0.008	0.016	0.006	0.007	0.001	473.367	4.030	3.784	7.814	2.758	3.222	0.446	4734	40.300	37.840	78.140	27.580	32.220	4.460
150	original	1	60x8 weeks	PCR90	M	VAC50	25	All	0.986	0.008	0.008	0.016	0.006	0.007	0.001	473.367	4.030	3.784	7.814	2.758	3.222	0.446	4734	40.300	37.840	78.140	27.580	32.220	4.460
150	original	1	60x8 weeks	PCR90	M	VAC50	25	All	0.986	0.008	0.008	0.016	0.006	0.007	0.001	473.367	4.030	3.784	7.814	2.758	3.222	0.446	4734	40.300	37.840	78.140	27.580	32.220	4.460
150	original	1.2	60x8 weeks	PCR90	M	VAC50	25	All	0.984	0.008	0.011	0.019	0.007	0.008	0.001	472.306	4.031	5.163	9.194	3.352	3.644	0.450	4723	40.310	51.630	91.940	33.520	36.440	4.500
150	original	1.2	60x8 weeks	PCR90	M	VAC50	25	All	0.984	0.008	0.011	0.019	0.007	0.008	0.001	472.306	4.031	5.163	9.194	3.352	3.644	0.450	4723	40.310	51.630	91.940	33.520	36.440	4.500
150	original	1.2	60x8 weeks	PCR90	M	VAC50	25	All	0.984	0.008	0.011	0.019	0.007	0.008	0.001	472.306	4.031	5.163	9.194	3.352	3.644	0.450	4723	40.310	51.630	91.940	33.520	36.440	4.500
150	alpha	0.6	60x8 weeks	PCR90	M	VAC50	25	All	0.979	0.009	0.013	0.022	0.008	0.011	0.001	469.824	4.096	6.427	10.523	4.073	5.152	0.420	4698	40.960	64.270	105.230	40.730	51.520	4.200
150	alpha	0.6	60x8 weeks	PCR90	M	VAC50	25	All	0.979	0.009	0.013	0.022	0.008	0.011	0.001	469.824	4.096	6.427	10.523	4.073	5.152	0.420	4698	40.960	64.270	105.230	40.730	51.520	4.200
150	alpha	0.6	60x8 weeks	PCR90	M	VAC50	25	All	0.979	0.009	0.013	0.022	0.008	0.011	0.001	469.824	4.096	6.427	10.523	4.073	5.152	0.420	4698	40.960	64.270	105.230	40.730	51.520	4.200
150	alpha	1	60x8 weeks	PCR90	M	VAC50	25	All	0.951	0.007	0.042	0.050	0.020	0.024	0.001	456.381	3.584	20.381	23.965	9.429	11.741	0.404	4564	35.840	203.810	239.650	94.290	117.410	4.040
150	alpha	1	60x8 weeks	PCR90	M	VAC50	25	All	0.951	0.007	0.042	0.050	0.020	0.024	0.001	456.381	3.584	20.381	23.965	9.429	11.741	0.404	4564	35.840	203.810	239.650	94.290	117.410	4.040
150	alpha	1	60x8 weeks	PCR90	M	VAC50	25	All	0.951	0.007	0.042	0.050	0.020	0.024	0.001	456.381	3.584	20.381	23.965	9.429	11.741	0.404	4564	35.840	203.810	239.650	94.290	117.410	4.040
150	alpha	1.2	60x8 weeks	PCR90	M	VAC50	25	All	0.931	0.007	0.062	0.069	0.027	0.034	0.001	446.868	3.288	29.931	33.219	13.166	16.475	0.367	4469	32.880	299.310	332.190	131.660	164.750	3.670
150	alpha	1.2	60x8 weeks	PCR90	M	VAC50	25	All	0.931	0.007	0.062	0.069	0.027	0.034	0.001	446.868	3.288	29.931	33.219	13.166	16.475	0.367	4469	32.880	299.310	332.190	131.660	164.750	3.670
150	alpha	1.2	60x8 weeks	PCR90	M	VAC50	25	All	0.931	0.007	0.062	0.069	0.027	0.034	0.001	446.868	3.288	29.931	33.219	13.166	16.475	0.367	4469	32.880	299.310	332.190	131.660	164.750	3.670
150	delta	0.6	60x8 weeks	PCR90	M	VAC50	25	All	0.910	0.006	0.078	0.084	0.037	0.040	0.001	436.850	2.999	37.429	40.428	17.945	19.219	0.306	4369	29.990	374.290	404.280	179.450	192.190	3.060
150	delta	0.6	60x8 weeks	PCR90	M	VAC50	25	All	0.910	0.006	0.078	0.084	0.037	0.040	0.001	436.850	2.999	37.429	40.428	17.945	19.219	0.306	4369	29.990	374.290	404.280	179.450	192.190	3.060
150	delta	0.6	60x8 weeks	PCR90	M	VAC50	25	All	0.910	0.006	0.078	0.084	0.037	0.040	0.001	436.850	2.999	37.429	40.428	17.945	19.219	0.306	4369	29.990	374.290	404.280	179.450	192.190	3.060
150	delta	1	60x8 weeks	PCR90	M	VAC50	25	All	0.866	0.004	0.113	0.118	0.053	0.058	0.000	415.684	2.026	54.455	56.481	25.647	27.607	0.232	4157	20.260	544.550	564.810	256.470	276.070	2.320
150	delta	1	60x8 weeks	PCR90	M	VAC50	25	All	0.866	0.004	0.113	0.118	0.053	0.058	0.000	415.684	2.026	54.455	56.481	25.647	27.607	0.232	4157	20.260	544.550	564.810	256.470	276.070	2.320
150	delta	1	60x8 weeks	PCR90	M	VAC50	25	All	0.866	0.004	0.113	0.118	0.053	0.058	0.000	415.684	2.026	54.455	56.481	25.647	27.607	0.232	4157	20.260	544.550	564.810	256.470	276.070	2.320
150	delta	1.2	60x8 weeks	PCR90	M	VAC50	25	All	0.861	0.004	0.116	0.120	0.055	0.060	0.000	413.096	1.860	55.659	57.519	26.161	28.669	0.217	4131	18.600	556.590	575.190	261.610	286.690	2.170
150	delta	1.2	60x8 weeks	PCR90	M	VAC50	25	All	0.861	0.004	0.116	0.120	0.055	0.060	0.000	413.096	1.860	55.659	57.519	26.161	28.669	0.217	4131	18.600	556.590	575.190	261.610	286.690	2.170
150	delta	1.2	60x8 weeks	PCR90	M	VAC50	25	All	0.861	0.004	0.116	0.120	0.055	0.060	0.000	413.096	1.860	55.659	57.519	26.161	28.669	0.217	4131	18.600	556.590	575.190	261.610	286.690	2.170
150	original	0.6	60x8 weeks	PCR90	M	VAC50	50	All	0.992	0.008	0.002	0.010	0.004	0.003	0.001	476.271	3.747	0.935	4.682	1.856	1.449	0.446	4763	37.470	9.350	46.820	18.560	14.490	4.460
150	original	0.6	60x8 weeks	PCR90	M	VAC50	50	All	0.992	0.008	0.002	0.010	0.004	0.003	0.001	476.271	3.747	0.935	4.682	1.856	1.449	0.446	4763	37.470	9.350	46.820	18.560	14.490	4.460
150	original	0.6	60x8 weeks	PCR90	M	VAC50	50	All	0.992	0.008	0.002	0.010	0.004	0.003	0.001	476.271	3.747	0.935	4.682	1.856	1.449	0.446	4763	37.470	9.350	46.820	18.560	14.490	4.460
150	original	1	60x8 weeks	PCR90	M	VAC50	50	All	0.991	0.008	0.004	0.012	0.005	0.004	0.001	475.659	3.626	1.897	5.523	2.180	1.699	0.479	4757	36.260	18.970	55.230	21.800	16.990	4.790
150	original	1	60x8 weeks	PCR90	M	VAC50	50	All	0.991	0.008	0.004	0.012	0.005	0.004	0.001	475.659	3.626	1.897	5.523	2.180	1.699	0.479	4757	36.260	18.970	55.230	21.800	16.990	4.790
150	original	1	60x8 weeks	PCR90	M	VAC50	50	All	0.991	0.008	0.004	0.012	0.005	0.004	0.001	475.659	3.626	1.897	5.523	2.180	1.699	0.479	4757	36.260	18.970	55.230	21.800	16.990	4.790

150	original	1.2	60x8 weeks	PCR90	M	VAC50	50	All	0.990	0.008	0.005	0.012	0.005	0.004	0.001	475.361	3.612	2.378	5.990	2.324	1.850	0.460	4754	36.120	23.780	59.900	23.240	18.500	4.600
150	original	1.2	60x8 weeks	PCR90	M	VAC50	50	All	0.990	0.008	0.005	0.012	0.005	0.004	0.001	475.361	3.612	2.378	5.990	2.324	1.850	0.460	4754	36.120	23.780	59.900	23.240	18.500	4.600
150	original	1.2	60x8 weeks	PCR90	M	VAC50	50	All	0.990	0.008	0.005	0.012	0.005	0.004	0.001	475.361	3.612	2.378	5.990	2.324	1.850	0.460	4754	36.120	23.780	59.900	23.240	18.500	4.600
150	alpha	0.6	60x8 weeks	PCR90	M	VAC50	50	All	0.986	0.008	0.008	0.015	0.006	0.007	0.001	473.143	3.604	3.611	7.215	2.835	3.374	0.438	4731	36.040	36.110	72.150	28.350	33.740	4.380
150	alpha	0.6	60x8 weeks	PCR90	M	VAC50	50	All	0.986	0.008	0.008	0.015	0.006	0.007	0.001	473.143	3.604	3.611	7.215	2.835	3.374	0.438	4731	36.040	36.110	72.150	28.350	33.740	4.380
150	alpha	0.6	60x8 weeks	PCR90	M	VAC50	50	All	0.986	0.008	0.008	0.015	0.006	0.007	0.001	473.143	3.604	3.611	7.215	2.835	3.374	0.438	4731	36.040	36.110	72.150	28.350	33.740	4.380
150	alpha	1	60x8 weeks	PCR90	M	VAC50	50	All	0.972	0.007	0.022	0.029	0.011	0.014	0.001	466.694	3.387	10.422	13.809	5.457	6.594	0.449	4667	33.870	104.220	138.090	54.570	65.940	4.490
150	alpha	1	60x8 weeks	PCR90	M	VAC50	50	All	0.972	0.007	0.022	0.029	0.011	0.014	0.001	466.694	3.387	10.422	13.809	5.457	6.594	0.449	4667	33.870	104.220	138.090	54.570	65.940	4.490
150	alpha	1	60x8 weeks	PCR90	M	VAC50	50	All	0.972	0.007	0.022	0.029	0.011	0.014	0.001	466.694	3.387	10.422	13.809	5.457	6.594	0.449	4667	33.870	104.220	138.090	54.570	65.940	4.490
150	alpha	1.2	60x8 weeks	PCR90	M	VAC50	50	All	0.963	0.007	0.032	0.038	0.015	0.018	0.001	462.234	3.254	15.173	18.427	7.244	8.868	0.412	4622	32.540	151.730	184.270	72.440	88.680	4.120
150	alpha	1.2	60x8 weeks	PCR90	M	VAC50	50	All	0.963	0.007	0.032	0.038	0.015	0.018	0.001	462.234	3.254	15.173	18.427	7.244	8.868	0.412	4622	32.540	151.730	184.270	72.440	88.680	4.120
150	alpha	1.2	60x8 weeks	PCR90	M	VAC50	50	All	0.963	0.007	0.032	0.038	0.015	0.018	0.001	462.234	3.254	15.173	18.427	7.244	8.868	0.412	4622	32.540	151.730	184.270	72.440	88.680	4.120
150	delta	0.6	60x8 weeks	PCR90	M	VAC50	50	All	0.945	0.007	0.047	0.054	0.024	0.025	0.001	453.595	3.375	22.648	26.023	11.297	11.988	0.383	4536	33.750	226.480	260.230	112.970	119.880	3.830
150	delta	0.6	60x8 weeks	PCR90	M	VAC50	50	All	0.945	0.007	0.047	0.054	0.024	0.025	0.001	453.595	3.375	22.648	26.023	11.297	11.988	0.383	4536	33.750	226.480	260.230	112.970	119.880	3.830
150	delta	0.6	60x8 weeks	PCR90	M	VAC50	50	All	0.945	0.007	0.047	0.054	0.024	0.025	0.001	453.595	3.375	22.648	26.023	11.297	11.988	0.383	4536	33.750	226.480	260.230	112.970	119.880	3.830
150	delta	1	60x8 weeks	PCR90	M	VAC50	50	All	0.883	0.005	0.101	0.106	0.047	0.051	0.001	423.827	2.255	48.426	50.681	22.712	24.494	0.281	4238	22.550	484.260	506.810	227.120	244.940	2.810
150	delta	1	60x8 weeks	PCR90	M	VAC50	50	All	0.883	0.005	0.101	0.106	0.047	0.051	0.001	423.827	2.255	48.426	50.681	22.712	24.494	0.281	4238	22.550	484.260	506.810	227.120	244.940	2.810
150	delta	1	60x8 weeks	PCR90	M	VAC50	50	All	0.883	0.005	0.101	0.106	0.047	0.051	0.001	423.827	2.255	48.426	50.681	22.712	24.494	0.281	4238	22.550	484.260	506.810	227.120	244.940	2.810
150	delta	1.2	60x8 weeks	PCR90	M	VAC50	50	All	0.871	0.004	0.111	0.115	0.052	0.056	0.001	417.883	1.991	53.290	55.281	24.925	26.949	0.252	4179	19.910	532.900	552.810	249.250	269.490	2.520
150	delta	1.2	60x8 weeks	PCR90	M	VAC50	50	All	0.871	0.004	0.111	0.115	0.052	0.056	0.001	417.883	1.991	53.290	55.281	24.925	26.949	0.252	4179	19.910	532.900	552.810	249.250	269.490	2.520
150	delta	1.2	60x8 weeks	PCR90	M	VAC50	50	All	0.871	0.004	0.111	0.115	0.052	0.056	0.001	417.883	1.991	53.290	55.281	24.925	26.949	0.252	4179	19.910	532.900	552.810	249.250	269.490	2.520
150	original	0.6	60x8 weeks	PCR90	M	VAC50	75	All	0.995	0.007	0.001	0.007	0.003	0.001	0.001	477.409	3.131	0.384	3.515	1.526	0.704	0.472	4774	31.310	3.840	35.150	15.260	7.040	4.720
150	original	0.6	60x8 weeks	PCR90	M	VAC50	75	All	0.995	0.007	0.001	0.007	0.003	0.001	0.001	477.409	3.131	0.384	3.515	1.526	0.704	0.472	4774	31.310	3.840	35.150	15.260	7.040	4.720
150	original	0.6	60x8 weeks	PCR90	M	VAC50	75	All	0.995	0.007	0.001	0.007	0.003	0.001	0.001	477.409	3.131	0.384	3.515	1.526	0.704	0.472	4774	31.310	3.840	35.150	15.260	7.040	4.720
150	original	1	60x8 weeks	PCR90	M	VAC50	75	All	0.994	0.007	0.001	0.008	0.003	0.002	0.001	477.234	3.145	0.716	3.861	1.627	0.787	0.485	4772	31.450	7.160	38.610	16.270	7.870	4.850
150	original	1	60x8 weeks	PCR90	M	VAC50	75	All	0.994	0.007	0.001	0.008	0.003	0.002	0.001	477.234	3.145	0.716	3.861	1.627	0.787	0.485	4772	31.450	7.160	38.610	16.270	7.870	4.850
150	original	1	60x8 weeks	PCR90	M	VAC50	75	All	0.994	0.007	0.001	0.008	0.003	0.002	0.001	477.234	3.145	0.716	3.861	1.627	0.787	0.485	4772	31.450	7.160	38.610	16.270	7.870	4.850
150	original	1.2	60x8 weeks	PCR90	M	VAC50	75	All	0.994	0.006	0.002	0.008	0.003	0.002	0.001	477.185	3.096	0.785	3.881	1.633	0.822	0.494	4772	30.960	7.850	38.810	16.330	8.220	4.940
150	original	1.2	60x8 weeks	PCR90	M	VAC50	75	All	0.994	0.006	0.002	0.008	0.003	0.002	0.001	477.185	3.096	0.785	3.881	1.633	0.822	0.494	4772	30.960	7.850	38.810	16.330	8.220	4.940
150	original	1.2	60x8 weeks	PCR90	M	VAC50	75	All	0.994	0.006	0.002	0.008	0.003	0.002	0.001	477.185	3.096	0.785	3.881	1.633	0.822	0.494	4772	30.960	7.850	38.810	16.330	8.220	4.940
150	alpha	0.6	60x8 weeks	PCR90	M	VAC50	75	All	0.991	0.006	0.003	0.010	0.004	0.005	0.001	475.544	3.062	1.663	4.725	1.843	2.212	0.502	4755	30.620	16.630	47.250	18.430	22.120	5.020
150	alpha	0.6	60x8 weeks	PCR90	M	VAC50	75	All	0.991	0.006	0.003	0.010	0.004	0.005	0.001	475.544	3.062	1.663	4.725	1.843	2.212	0.502	4755	30.620	16.630	47.250	18.430	22.120	5.020
150	alpha	0.6	60x8 weeks	PCR90	M	VAC50	75	All	0.991	0.006	0.003	0.010	0.004	0.005	0.001	475.544	3.062	1.663	4.725	1.843	2.212	0.502	4755	30.620	16.630	47.250	18.430	22.120	5.020
150	alpha	1	60x8 weeks	PCR90	M	VAC50	75	All	0.987	0.006	0.008	0.014	0.006	0.007	0.001	473.676	3.013	3.927	6.940	2.648	3.251	0.448	4737	30.130	39.270	69.400	26.480	32.510	4.480
150	alpha	1	60x8 weeks	PCR90	M	VAC50	75	All	0.987	0.006	0.008	0.014	0.006	0.007	0.001	473.676	3.013	3.927	6.940	2.648	3.251	0.448	4737	30.130	39.270	69.400	26.480	32.510	4.480
150	alpha	1	60x8 weeks	PCR90	M	VAC50	75	All	0.987	0.006	0.008	0.014	0.006	0.007	0.001	473.676	3.013	3.927	6.940	2.648	3.251	0.448	4737	30.130	39.270	69.400	26.480	32.510	4.480
150	alpha	1.2	60x8 weeks	PCR90	M	VAC50	75	All	0.983	0.006	0.012	0.018	0.007	0.008	0.001	472.065	2.953	5.655	8.608	3.358	3.967	0.449	4721	29.530	56.550	86.080	33.580	39.670	4.490
150	alpha	1.2	60x8 weeks	PCR90	M	VAC50	75	All	0.983	0.006	0.012	0.018	0.007	0.008	0.001	472.065	2.953	5.655	8.608	3.358	3.967	0.449	4721	29.530	56.550	86.080	33.580	39.670	4.490
150	alpha	1.2	60x8 weeks	PCR90	M	VAC50	75	All	0.983	0.006	0.012	0.018	0.007	0.008	0.001	472.065	2.953	5.655	8.608	3.358	3.967	0.449	4721	29.530	56.550	86.080	33.580	39.670	4.490
150	delta	0.6	60x8 weeks	PCR90	M	VAC50	75	All	0.970	0.007	0.023	0.030	0.013	0.014	0.001	465.536	3.367	11.165	14.532	6.318	6.552	0.447	4655	33.670	111.650	145.320	63.180	65.520	4.470
150	delta	0.6	60x8 weeks	PCR90	M	VAC50	75	All	0.970	0.007	0.023	0.030	0.013	0.014	0.001	465.536	3.367	11.165	14.532	6.318	6.552	0.447	4655	33.670	111.650	145.320	63.180	65.520	4.470
150	delta	0.6	60x8 weeks	PCR90	M	VAC50	75	All	0.970	0.007	0.023	0.030	0.013	0.014	0.001	465.536	3.367	11.165	14.532	6.318	6.552	0.447	4655	33.670	111.650	145.320	63.180	65.520	4.470
150	delta	1	60x8 weeks	PCR90	M	VAC50	75	All	0.921	0.006	0.071	0.077	0.034	0.035	0.001	441.861	2.692	34.082	36.774	16.245	17.008	0.358	4419	26.920	340.820	367.740	162.450	170.080	3.580
150	delta	1	60x8 weeks	PCR90	M	VAC50	75	All	0.921	0.006	0.071	0.077	0.034	0.035	0.001	441.861	2.692	34.082	36.774	16.245	17.008	0.358	4419	26.920	340.820	367.740	162.450	170.080	3.580
150	delta	1	60x8 weeks	PCR90	M	VAC50	75	All	0.921	0.006	0.071	0.077	0.034	0.035	0.001	441.861	2.692	34.082	36.774	16.245	17.008	0.358	4419	26.920	340.820	367.740	162.450	170.080	3.580
150	delta	1.2	60x8 weeks	PCR90	M	VAC50	75	All	0.898	0.005	0.091	0.096	0.043	0.045	0.001	430.872	2.310	43.816	46.126	20.574	21.700	0.314	4309	23.100	438.160	461.260	205.740	217.000	3.140
150	delta	1.2	60x8 weeks	PCR90	M	VAC50	75	All	0.898	0.005	0.091	0.096	0.043	0.045	0.001	430.872	2.310	43.816	46.126	20.574	21.700	0.314	4309	23.100	438.160	461.260	205.740	217.000	3.140
150	delta	1.2	60x8 weeks	PCR90	M	VAC50	75	All	0.898	0.005	0.091	0.096	0.043	0.045	0.001	430.872	2.310	43.816	46.126	20.574	21.700	0.314	4309	23.100	438.160	461.260	205.740	217.000	3.140
150	original	0.6	60x8 weeks	PCR90	M	VAC50	100	All	0.996	0.005	0.000	0.006	0.003	0.000	0.001	478.181	2.618	0.037	2.655	1.309	0.138	0.475	4782	26.180	0.370	26.550	13.090	1.380	4.750
150	original	0.6	60x8 weeks	PCR90	M	VAC50	100	All	0.996	0.005	0.000	0.006	0.003	0.000	0.001	478.181	2.618	0.037	2.655	1.309	0.138	0.475	4782	26.180	0.370	26.550	13.090	1.380	4.750
150	original	0.6	60x8 weeks	PCR90	M	VAC50	100	All	0.996	0.005	0.000	0.006	0.003	0.000	0.001	478.181	2.618	0.037	2.655	1.309	0.138	0.475	4782	26.180	0.370	26.550	13.090	1.380	4.750
150	original	1	60x8 weeks	PCR90	M	VAC50	100	All	0.996	0.005	0.000	0.006	0.003	0.000	0.001	478.274	2.603	0.059	2.662	1.294	0.112	0.477	4783	26.030	0.590	26.620	12.940	1.120	4.770
150	original	1	60x8 weeks	PCR90	M	VAC50	100	All	0.996	0.005	0.000	0.006	0.003	0.000	0.001	478.274	2.603	0.059	2.662	1.294	0.112	0.477	4783	26.030	0.590	26.620	12.940	1.120	4.770
150	original	1	60x8 weeks	PCR90	M	VAC50	100	All	0.996	0.005	0.000	0.006	0.003	0.000	0.001	478.274	2.603	0.059	2.662	1.294	0.112	0.477	4783	26.030	0.590	26.620	12.940	1.120	4.770
150	original	1.2	60x8 weeks	PCR90	M	VAC50	100	All	0.996	0.005	0.000	0.005	0.003	0.000	0.001	478.253	2.521	0.062	2.583	1.274	0.118	0.476	4783	25.210	0.620	25.830	12.740	1.180	4.760
150	original	1.2	60x8 weeks	PCR90	M	VAC50	100	All	0.996	0.005	0.000	0.005	0.003	0.000	0.001	478.253	2.521	0.062	2.583	1.274	0.118	0.476	4783	25.210	0.620	25.830	12.740	1.180	4.760
150	original	1.2	60x8 weeks	PCR90	M	VAC50	100	All	0.996	0.005	0.000	0.005	0.003	0.000	0.001	478.253	2.521	0.062	2.583	1.274	0.118	0.476	4783	25.210	0.620	25.830	12.740	1.180	4.760
150	alpha	0.6	60x8 weeks	PCR90	M	VAC50	100	All	0.994	0.005	0.001	0.007	0.003	0.003	0.001	476.960	2.578	0.680	3.258	1.241	1.515	0.466	4770	25.780	6.800	32.580	12.410	15.150	4.660
150	alpha	0.6	60x8 weeks	PCR90	M	VAC50	100	All	0.994	0.005	0.001	0.007	0.003	0.003	0.001	476.960	2.578	0.680	3.258	1.241	1.515	0.466	4770	25.780	6.800	32.580	12.410	15.150	4.660
150	alpha	0.6	60x8 weeks	PCR90	M	VAC50	100	All	0.994	0.005	0.001	0.007	0.003	0.003	0.001	476.960	2.578	0.680	3.258	1.241	1.515	0.466	4770	25.780	6.800	32.580	12.410	15.150	4.660
150	alpha	1	60x8 weeks	PCR90	M	VAC50	100	All	0.992	0.005	0.003	0.008	0.003	0.004	0.001	476.271	2.550	1.361	3.911	1.572	1.720	0.425	4763	25.500	13.610	39.110	15.720	17.200	4.250
150	alpha	1	60x8 weeks	PCR90	M	VAC50	100	All	0.992	0.005	0.003	0.008	0.003	0.004	0.001	476.271	2.550	1.361	3.911	1.572	1.720	0.425	4763	25.500	13.610	39.110	15.720	17.200	4.250
150	alpha	1	60x8 weeks	PCR90	M	VAC50	100	All	0.992	0.005	0.003	0.008	0.003	0.004	0.001	476.271	2.550	1.361	3.911	1.572	1.720	0.425	4763	25.500	13.610	39.110	15.720	17.200	4.250
150	alpha	1.2	60x8 weeks	PCR90	M	VAC50	100	All	0.991	0.005	0.004	0.009	0.004	0.004	0.001	475.864	2.523	1.917	4.440	1.712	2.069	0.478	4759	25.230	19.170	44.400	17.120	20.690	4.780
150	alpha	1.2	60x8 weeks	PCR90	M	VAC50	100	All	0.991	0.005	0.004	0.009	0.004	0.004	0.001	475.864	2.523	1.917	4.440	1.712	2.069	0.478	4759	25.230	19.170	44.400	17.120	20.690	4.780
150	alpha	1.2	60x8 weeks	PCR90	M	VAC50	100	All	0.991	0.005	0.004	0.009	0.004	0.004	0.001	475.864	2.523	1.917	4.440	1.712	2.069	0.478	4759	25.230	19.170	44.400	17.120	20.690	4.780
150	delta	0.6	60x8 weeks	PCR90	M	VAC50	100	All	0.985	0.007	0.009	0.015	0.006	0.007	0.001	472.879	3.166	4.201	7.367	3.110	3.360	0.449	4729	31.660	42.010	73.670	31.100	33.600	4.490
150	delta	0.6	60x8 weeks	PCR90	M	VAC50	100	All	0.985	0.007	0.009	0.015	0.006	0.007	0.001	472.879	3.166	4.201	7.367	3.110	3.360	0.449	4729	31.660	42.010	73.670	31.100	33.600	4.490
150	delta	0.6	60x8 weeks	PCR90	M	VAC50	100	All	0.985	0.007	0.009	0.015	0.006	0.007	0.001	472.879	3.166	4.201	7.367	3.110	3.360	0.449	4729	31.660	42.010	73.670	31.100	33.600	4.490
150	delta	1	60x8 weeks	PCR90	M	VAC50	100	All	0.964	0.006	0.030	0.036	0.016	0.017	0.001	462.868	2.861	14.565	17.426	7.443	7.940	0.401	4629	28.610	145.650	174.260	74.430	79.400	4.010
150	delta	1	60x8 weeks	PCR90	M	VAC50	100	All	0.964	0.006	0.030	0.036	0.016	0.017	0.001	462.868	2.861	14.565	17.426	7.443	7.940	0.401	4629	28.610	145.650	174.260	74.430	79.400	4.010

150	delta	1	60x8 weeks	PCR90	M	VAC50	100	All	0.964	0.006	0.030	0.036	0.016	0.017	0.001	462.868	2.861	14.565	17.426	7.443	7.940	0.401	4629	28.610	145.650	174.260	74.430	79.400	4.010
150	delta	1.2	60x8 weeks	PCR90	M	VAC50	100	All	0.948	0.006	0.047	0.053	0.023	0.024	0.001	455.183	2.772	22.693	25.465	10.888	11.477	0.345	4552	27.720	226.930	254.650	108.880	114.770	3.450
150	delta	1.2	60x8 weeks	PCR90	M	VAC50	100	All	0.948	0.006	0.047	0.053	0.023	0.024	0.001	455.183	2.772	22.693	25.465	10.888	11.477	0.345	4552	27.720	226.930	254.650	108.880	114.770	3.450
150	delta	1.2	60x8 weeks	PCR90	M	VAC50	100	All	0.948	0.006	0.047	0.053	0.023	0.024	0.001	455.183	2.772	22.693	25.465	10.888	11.477	0.345	4552	27.720	226.930	254.650	108.880	114.770	3.450
150	original	0.6	60x8 weeks	Ag80	Daily	NOVAC	0	All	4.923	0.010	0.001	0.011	0.000	0.011	0.095	2363.099	4.743	0.678	5.421	0.154	5.429	45.837	23631	47.430	6.780	54.210	1.540	54.290	458.370
150	original	0.6	60x8 weeks	Ag80	Daily	NOVAC	0	All	4.923	0.010	0.001	0.011	0.000	0.011	0.095	2363.099	4.743	0.678	5.421	0.154	5.429	45.837	23631	47.430	6.780	54.210	1.540	54.290	458.370
150	original	0.6	60x8 weeks	Ag80	Daily	NOVAC	0	All	4.923	0.010	0.001	0.011	0.000	0.011	0.095	2363.099	4.743	0.678	5.421	0.154	5.429	45.837	23631	47.430	6.780	54.210	1.540	54.290	458.370
150	original	1	60x8 weeks	Ag80	Daily	NOVAC	0	All	4.916	0.010	0.002	0.012	0.000	0.012	0.094	2359.817	4.739	1.157	5.896	0.147	5.926	44.967	23598	47.390	11.570	58.960	1.470	59.260	449.670
150	original	1	60x8 weeks	Ag80	Daily	NOVAC	0	All	4.916	0.010	0.002	0.012	0.000	0.012	0.094	2359.817	4.739	1.157	5.896	0.147	5.926	44.967	23598	47.390	11.570	58.960	1.470	59.260	449.670
150	original	1	60x8 weeks	Ag80	Daily	NOVAC	0	All	4.916	0.010	0.002	0.012	0.000	0.012	0.094	2359.817	4.739	1.157	5.896	0.147	5.926	44.967	23598	47.390	11.570	58.960	1.470	59.260	449.670
150	original	1.2	60x8 weeks	Ag80	Daily	NOVAC	0	All	4.912	0.010	0.003	0.013	0.000	0.013	0.095	2357.577	4.836	1.398	6.234	0.132	6.283	45.407	23576	48.360	13.980	62.340	1.320	62.830	454.070
150	original	1.2	60x8 weeks	Ag80	Daily	NOVAC	0	All	4.912	0.010	0.003	0.013	0.000	0.013	0.095	2357.577	4.836	1.398	6.234	0.132	6.283	45.407	23576	48.360	13.980	62.340	1.320	62.830	454.070
150	original	1.2	60x8 weeks	Ag80	Daily	NOVAC	0	All	4.912	0.010	0.003	0.013	0.000	0.013	0.095	2357.577	4.836	1.398	6.234	0.132	6.283	45.407	23576	48.360	13.980	62.340	1.320	62.830	454.070
150	alpha	0.6	60x8 weeks	Ag80	Daily	NOVAC	0	All	4.912	0.010	0.002	0.013	0.000	0.013	0.095	2357.864	4.889	1.131	6.020	0.128	6.227	45.610	23579	48.890	11.310	60.200	1.280	62.270	456.100
150	alpha	0.6	60x8 weeks	Ag80	Daily	NOVAC	0	All	4.912	0.010	0.002	0.013	0.000	0.013	0.095	2357.864	4.889	1.131	6.020	0.128	6.227	45.610	23579	48.890	11.310	60.200	1.280	62.270	456.100
150	alpha	0.6	60x8 weeks	Ag80	Daily	NOVAC	0	All	4.912	0.010	0.002	0.013	0.000	0.013	0.095	2357.864	4.889	1.131	6.020	0.128	6.227	45.610	23579	48.890	11.310	60.200	1.280	62.270	456.100
150	alpha	1	60x8 weeks	Ag80	Daily	NOVAC	0	All	4.899	0.010	0.004	0.014	0.000	0.015	0.094	2351.321	4.729	2.056	6.785	0.184	7.128	45.183	23513	47.290	20.560	67.850	1.840	71.280	451.830
150	alpha	1	60x8 weeks	Ag80	Daily	NOVAC	0	All	4.899	0.010	0.004	0.014	0.000	0.015	0.094	2351.321	4.729	2.056	6.785	0.184	7.128	45.183	23513	47.290	20.560	67.850	1.840	71.280	451.830
150	alpha	1	60x8 weeks	Ag80	Daily	NOVAC	0	All	4.899	0.010	0.004	0.014	0.000	0.015	0.094	2351.321	4.729	2.056	6.785	0.184	7.128	45.183	23513	47.290	20.560	67.850	1.840	71.280	451.830
150	alpha	1.2	60x8 weeks	Ag80	Daily	NOVAC	0	All	4.891	0.010	0.005	0.015	0.000	0.016	0.094	2347.899	4.710	2.621	7.331	0.147	7.683	45.052	23479	47.100	26.210	73.310	1.470	76.830	450.520
150	alpha	1.2	60x8 weeks	Ag80	Daily	NOVAC	0	All	4.891	0.010	0.005	0.015	0.000	0.016	0.094	2347.899	4.710	2.621	7.331	0.147	7.683	45.052	23479	47.100	26.210	73.310	1.470	76.830	450.520
150	alpha	1.2	60x8 weeks	Ag80	Daily	NOVAC	0	All	4.891	0.010	0.005	0.015	0.000	0.016	0.094	2347.899	4.710	2.621	7.331	0.147	7.683	45.052	23479	47.100	26.210	73.310	1.470	76.830	450.520
150	delta	0.6	60x8 weeks	Ag80	Daily	NOVAC	0	All	4.889	0.010	0.006	0.015	0.000	0.016	0.094	2346.536	4.722	2.711	7.433	0.215	7.689	44.920	23465	47.220	27.110	74.330	2.150	76.890	449.200
150	delta	0.6	60x8 weeks	Ag80	Daily	NOVAC	0	All	4.889	0.010	0.006	0.015	0.000	0.016	0.094	2346.536	4.722	2.711	7.433	0.215	7.689	44.920	23465	47.220	27.110	74.330	2.150	76.890	449.200
150	delta	0.6	60x8 weeks	Ag80	Daily	NOVAC	0	All	4.889	0.010	0.006	0.015	0.000	0.016	0.094	2346.536	4.722	2.711	7.433	0.215	7.689	44.920	23465	47.220	27.110	74.330	2.150	76.890	449.200
150	delta	1	60x8 weeks	Ag80	Daily	NOVAC	0	All	4.849	0.010	0.011	0.021	0.001	0.022	0.092	2327.379	4.581	5.452	10.033	0.284	10.461	43.936	23274	45.810	54.520	100.330	2.840	104.610	439.360
150	delta	1	60x8 weeks	Ag80	Daily	NOVAC	0	All	4.849	0.010	0.011	0.021	0.001	0.022	0.092	2327.379	4.581	5.452	10.033	0.284	10.461	43.936	23274	45.810	54.520	100.330	2.840	104.610	439.360
150	delta	1	60x8 weeks	Ag80	Daily	NOVAC	0	All	4.849	0.010	0.011	0.021	0.001	0.022	0.092	2327.379	4.581	5.452	10.033	0.284	10.461	43.936	23274	45.810	54.520	100.330	2.840	104.610	439.360
150	delta	1.2	60x8 weeks	Ag80	Daily	NOVAC	0	All	4.812	0.009	0.016	0.026	0.001	0.027	0.090	2309.561	4.482	7.860	12.342	0.315	13.052	43.310	23096	44.820	78.600	123.420	3.150	130.520	433.100
150	delta	1.2	60x8 weeks	Ag80	Daily	NOVAC	0	All	4.812	0.009	0.016	0.026	0.001	0.027	0.090	2309.561	4.482	7.860	12.342	0.315	13.052	43.310	23096	44.820	78.600	123.420	3.150	130.520	433.100
150	delta	1.2	60x8 weeks	Ag80	Daily	NOVAC	0	All	4.812	0.009	0.016	0.026	0.001	0.027	0.090	2309.561	4.482	7.860	12.342	0.315	13.052	43.310	23096	44.820	78.600	123.420	3.150	130.520	433.100
150	original	0.6	60x8 weeks	Ag80	Daily	VAC95	25	All	4.945	0.008	0.001	0.008	0.000	0.008	0.097	2373.500	3.691	0.344	4.035	0.117	3.924	46.513	23735	36.910	3.440	40.350	1.170	39.240	465.130
150	original	0.6	60x8 weeks	Ag80	Daily	VAC95	25	All	4.945	0.008	0.001	0.008	0.000	0.008	0.097	2373.500	3.691	0.344	4.035	0.117	3.924	46.513	23735	36.910	3.440	40.350	1.170	39.240	465.130
150	original	0.6	60x8 weeks	Ag80	Daily	VAC95	25	All	4.945	0.008	0.001	0.008	0.000	0.008	0.097	2373.500	3.691	0.344	4.035	0.117	3.924	46.513	23735	36.910	3.440	40.350	1.170	39.240	465.130
150	original	1	60x8 weeks	Ag80	Daily	VAC95	25	All	4.938	0.008	0.001	0.009	0.000	0.009	0.096	2370.144	3.785	0.691	4.476	0.134	4.401	45.945	23701	37.850	6.910	44.760	1.340	44.010	459.450
150	original	1	60x8 weeks	Ag80	Daily	VAC95	25	All	4.938	0.008	0.001	0.009	0.000	0.009	0.096	2370.144	3.785	0.691	4.476	0.134	4.401	45.945	23701	37.850	6.910	44.760	1.340	44.010	459.450
150	original	1	60x8 weeks	Ag80	Daily	VAC95	25	All	4.938	0.008	0.001	0.009	0.000	0.009	0.096	2370.144	3.785	0.691	4.476	0.134	4.401	45.945	23701	37.850	6.910	44.760	1.340	44.010	459.450
150	original	1.2	60x8 weeks	Ag80	Daily	VAC95	25	All	4.936	0.008	0.002	0.009	0.000	0.009	0.096	2369.467	3.700	0.853	4.553	0.150	4.479	46.250	23695	37.000	8.530	45.530	1.500	44.790	462.500
150	original	1.2	60x8 weeks	Ag80	Daily	VAC95	25	All	4.936	0.008	0.002	0.009	0.000	0.009	0.096	2369.467	3.700	0.853	4.553	0.150	4.479	46.250	23695	37.000	8.530	45.530	1.500	44.790	462.500
150	original	1.2	60x8 weeks	Ag80	Daily	VAC95	25	All	4.936	0.008	0.002	0.009	0.000	0.009	0.096	2369.467	3.700	0.853	4.553	0.150	4.479	46.250	23695	37.000	8.530	45.530	1.500	44.790	462.500
150	alpha	0.6	60x8 weeks	Ag80	Daily	VAC95	25	All	4.935	0.008	0.001	0.009	0.000	0.010	0.096	2368.840	3.753	0.677	4.430	0.107	4.587	46.147	23688	37.530	6.770	44.300	1.070	45.870	461.470
150	alpha	0.6	60x8 weeks	Ag80	Daily	VAC95	25	All	4.935	0.008	0.001	0.009	0.000	0.010	0.096	2368.840	3.753	0.677	4.430	0.107	4.587	46.147	23688	37.530	6.770	44.300	1.070	45.870	461.470
150	alpha	0.6	60x8 weeks	Ag80	Daily	VAC95	25	All	4.935	0.008	0.001	0.009	0.000	0.010	0.096	2368.840	3.753	0.677	4.430	0.107	4.587	46.147	23688	37.530	6.770	44.300	1.070	45.870	461.470
150	alpha	1	60x8 weeks	Ag80	Daily	VAC95	25	All	4.928	0.008	0.002	0.010	0.000	0.011	0.097	2365.279	3.716	1.136	4.852	0.119	5.073	46.441	23653	37.160	11.360	48.520	1.190	50.730	464.410
150	alpha	1	60x8 weeks	Ag80	Daily	VAC95	25	All	4.928	0.008	0.002	0.010	0.000	0.011	0.097	2365.279	3.716	1.136	4.852	0.119	5.073	46.441	23653	37.160	11.360	48.520	1.190	50.730	464.410
150	alpha	1	60x8 weeks	Ag80	Daily	VAC95	25	All	4.928	0.008	0.002	0.010	0.000	0.011	0.097	2365.279	3.716	1.136	4.852	0.119	5.073	46.441	23653	37.160	11.360	48.520	1.190	50.730	464.410
150	alpha	1.2	60x8 weeks	Ag80	Daily	VAC95	25	All	4.925	0.008	0.003	0.011	0.000	0.011	0.096	2363.839	3.681	1.413	5.094	0.095	5.348	46.298	23638	36.810	14.130	50.940	0.950	53.480	462.980
150	alpha	1.2	60x8 weeks	Ag80	Daily	VAC95	25	All	4.925	0.008	0.003	0.011	0.000	0.011	0.096	2363.839	3.681	1.413	5.094	0.095	5.348	46.298	23638	36.810	14.130	50.940	0.950	53.480	462.980
150	alpha	1.2	60x8 weeks	Ag80	Daily	VAC95	25	All	4.925	0.008	0.003	0.011	0.000	0.011	0.096	2363.839	3.681	1.413	5.094	0.095	5.348	46.298	23638	36.810	14.130	50.940	0.950	53.480	462.980
150	delta	0.6	60x8 weeks	Ag80	Daily	VAC95	25	All	4.923	0.008	0.003	0.011	0.000	0.011	0.095	2362.845	3.693	1.469	5.162	0.160	5.384	45.751	23628	36.930	14.690	51.620	1.600	53.840	457.510
150	delta	0.6	60x8 weeks	Ag80	Daily	VAC95	25	All	4.923	0.008	0.003	0.011	0.000	0.011	0.095	2362.845	3.693	1.469	5.162	0.160	5.384	45.751	23628	36.930	14.690	51.620	1.600	53.840	457.510
150	delta	0.6	60x8 weeks	Ag80	Daily	VAC95	25	All	4.923	0.008	0.003	0.011	0.000	0.011	0.095	2362.845	3.693	1.469	5.162	0.160	5.384	45.751	23628	36.930	14.690	51.620	1.600	53.840	457.510
150	delta	1	60x8 weeks	Ag80	Daily	VAC95	25	All	4.903	0.008	0.006	0.013	0.000	0.014	0.095	2353.548	3.633	2.824	6.457	0.198	6.716	45.548	23535	36.330	28.240	64.570	1.980	67.160	455.480
150	delta	1	60x8 weeks	Ag80	Daily	VAC95	25	All	4.903	0.008	0.006	0.013	0.000	0.014	0.095	2353.548	3.633	2.824	6.457	0.198	6.716	45.548	23535	36.330	28.240	64.570	1.980	67.160	455.480
150	delta	1	60x8 weeks	Ag80	Daily	VAC95	25	All	4.903	0.008	0.006	0.013	0.000	0.014	0.095	2353.548	3.633	2.824	6.457	0.198	6.716	45.548	23535	36.330	28.240	64.570	1.980	67.160	455.480
150	delta	1.2	60x8 weeks	Ag80	Daily	VAC95	25	All	4.883	0.008	0.008	0.016	0.000	0.017	0.094	2343.942	3.720	4.028	7.748	0.211	8.095	45.037	23439	37.200	40.280	77.480	2.110	80.950	450.370
150	delta	1.2	60x8 weeks	Ag80	Daily	VAC95	25	All	4.883	0.008	0.008	0.016	0.000	0.017	0.094	2343.942	3.720	4.028	7.748	0.211	8.095	45.037	23439	37.200	40.280	77.480	2.110	80.950	450.370
150	delta	1.2	60x8 weeks	Ag80	Daily	VAC95	25	All	4.883	0.008	0.008	0.016	0.000	0.017	0.094	2343.942	3.720	4.028	7.748	0.211	8.095	45.037	23439	37.200	40.280	77.480	2.110	80.950	450.370
150	original	0.6	60x8 weeks	Ag80	Daily	VAC95	50	All	4.962	0.005	0.000	0.006	0.000	0.005	0.099	2381.979	2.603	0.180	2.783	0.137	2.608	47.434	23820	26.030	1.800	27.830	1.370	26.080	474.340
150	original	0.6	60x8 weeks	Ag80	Daily	VAC95	50	All	4.962	0.005	0.000	0.006	0.000	0.005	0.099	2381.979	2.603	0.180	2.783	0.137	2.608	47.434	23820	26.030	1.800	27.830	1.370	26.080	474.340
150	original	0.6	60x8 weeks	Ag80	Daily	VAC95	50	All	4.962	0.005	0.000	0.006	0.000	0.005	0.099	2381.979	2.603	0.180	2.783	0.137	2.608	47.434	23820	26.030	1.800	27.830	1.370	26.080	474.340
150	original	1	60x8 weeks	Ag80	Daily	VAC95	50	All	4.961	0.005	0.001	0.006	0.000	0.006	0.098	2381.151	2.573	0.308	2.881	0.120	2.735	47.247	23812	25.730	3.080	28.810	1.200	27.350	472.470
150	original	1	60x8 weeks	Ag80	Daily	VAC95	50	All	4.961	0.005	0.001	0.006	0.000	0.006	0.098	2381.151	2.573	0.308	2.881	0.120	2.735	47.247	23812	25.730	3.080	28.810	1.200	27.350	472.470
150	original	1	60x8 weeks	Ag80	Daily	VAC95	50	All	4.961	0.005	0.001	0.006	0.000	0.006	0.098	2381.151	2.573	0.308	2.881	0.120	2.735	47.247	23812	25.730	3.080	28.810	1.200	27.350	472.470
150	original	1.2	60x8 weeks	Ag80	Daily	VAC95	50	All	4.959	0.005	0.001	0.006	0.000	0.006	0.098	2380.446	2.600	0.368	2.968	0.105	2.837	47.076	23804	26.000	3.680	29.680	1.050	28.370	470.760
150	original	1.2	60x8 weeks	Ag80	Daily	VAC95	50	All	4.959	0.005	0.001	0.006	0.000	0.006	0.098	2380.446	2.600	0.368	2.968	0.105	2.837	47.076	23804	26.000	3.680	29.680	1.050	28.370	470.760
150	original	1.2	60x8 weeks	Ag80	Daily	VAC95	50	All	4.959	0.005	0.001	0.006	0.000	0.006	0.098	2380.446	2.600	0.368	2.968	0.105	2.837	47.076	23804	26.000	3.680	29.680	1.050	28.370	470.760
150	alpha	0.6	60x8 weeks	Ag80	Daily	VAC95	50	All	4.957	0.005	0.001	0.006	0.000	0.006	0.097	2379.484	2.628	0.284	2.912	0.066	3.039	46.773	23795	26.280	2.840	29.120	0.660	30.390	467.730
150	alpha	0.6	60x8 weeks	Ag80	Daily	VAC95	50	All	4.957	0.005	0.001	0.006	0.000	0.006	0.097	2379.484	2.628	0.284	2.912	0.066	3.039	46.773	23795	26.280	2.840	29.120	0.660	30.390	467.730
150	alpha	0.6	60x8 weeks	Ag80	Daily	VAC95	50	All	4.957	0.005	0.001	0.006	0.000	0.006	0.097	2379.484	2.628	0.284	2.912	0.066	3.039	46.773	23795	26.280	2.840	29.120	0.660	30.390	467.730
150	alpha	1	60x8 weeks	Ag80	Daily	VAC95	50	All	4.953	0.006	0.001	0.007	0.000	0.007	0.098	2377.679	2.643	0.543	3.186	0.075	3.299	46.823	23777	26.430	5.430	31.860	0.750	32.990	468.230

150	alpha	1	60x8 weeks	Ag80	Daily	VAC95	50	All	4.953	0.006	0.001	0.007	0.000	0.007	0.098	2377.679	2.643	0.543	3.186	0.075	3.299	46.823	23777	26.430	5.430	31.860	0.750	32.990	468.230
150	alpha	1	60x8 weeks	Ag80	Daily	VAC95	50	All	4.953	0.006	0.001	0.007	0.000	0.007	0.098	2377.679	2.643	0.543	3.186	0.075	3.299	46.823	23777	26.430	5.430	31.860	0.750	32.990	468.230
150	alpha	1.2	60x8 weeks	Ag80	Daily	VAC95	50	All	4.952	0.005	0.001	0.007	0.000	0.007	0.097	2376.865	2.595	0.657	3.252	0.090	3.369	46.422	23769	25.950	6.570	32.520	0.900	33.690	464.220
150	alpha	1.2	60x8 weeks	Ag80	Daily	VAC95	50	All	4.952	0.005	0.001	0.007	0.000	0.007	0.097	2376.865	2.595	0.657	3.252	0.090	3.369	46.422	23769	25.950	6.570	32.520	0.900	33.690	464.220
150	alpha	1.2	60x8 weeks	Ag80	Daily	VAC95	50	All	4.952	0.005	0.001	0.007	0.000	0.007	0.097	2376.865	2.595	0.657	3.252	0.090	3.369	46.422	23769	25.950	6.570	32.520	0.900	33.690	464.220
150	delta	0.6	60x8 weeks	Ag80	Daily	VAC95	50	All	4.949	0.006	0.001	0.007	0.000	0.007	0.097	2375.423	2.785	0.682	3.467	0.101	3.556	46.398	23754	27.850	6.820	34.670	1.010	35.560	463.980
150	delta	0.6	60x8 weeks	Ag80	Daily	VAC95	50	All	4.949	0.006	0.001	0.007	0.000	0.007	0.097	2375.423	2.785	0.682	3.467	0.101	3.556	46.398	23754	27.850	6.820	34.670	1.010	35.560	463.980
150	delta	0.6	60x8 weeks	Ag80	Daily	VAC95	50	All	4.949	0.006	0.001	0.007	0.000	0.007	0.097	2375.423	2.785	0.682	3.467	0.101	3.556	46.398	23754	27.850	6.820	34.670	1.010	35.560	463.980
150	delta	1	60x8 weeks	Ag80	Daily	VAC95	50	All	4.940	0.006	0.003	0.008	0.000	0.009	0.096	2371.415	2.808	1.207	4.015	0.115	4.162	46.251	23714	28.080	12.070	40.150	1.150	41.620	462.510
150	delta	1	60x8 weeks	Ag80	Daily	VAC95	50	All	4.940	0.006	0.003	0.008	0.000	0.009	0.096	2371.415	2.808	1.207	4.015	0.115	4.162	46.251	23714	28.080	12.070	40.150	1.150	41.620	462.510
150	delta	1	60x8 weeks	Ag80	Daily	VAC95	50	All	4.940	0.006	0.003	0.008	0.000	0.009	0.096	2371.415	2.808	1.207	4.015	0.115	4.162	46.251	23714	28.080	12.070	40.150	1.150	41.620	462.510
150	delta	1.2	60x8 weeks	Ag80	Daily	VAC95	50	All	4.939	0.005	0.003	0.008	0.000	0.009	0.096	2370.630	2.609	1.461	4.070	0.116	4.246	46.156	23706	26.090	14.610	40.700	1.160	42.460	461.560
150	delta	1.2	60x8 weeks	Ag80	Daily	VAC95	50	All	4.939	0.005	0.003	0.008	0.000	0.009	0.096	2370.630	2.609	1.461	4.070	0.116	4.246	46.156	23706	26.090	14.610	40.700	1.160	42.460	461.560
150	delta	1.2	60x8 weeks	Ag80	Daily	VAC95	50	All	4.939	0.005	0.003	0.008	0.000	0.009	0.096	2370.630	2.609	1.461	4.070	0.116	4.246	46.156	23706	26.090	14.610	40.700	1.160	42.460	461.560
150	original	0.6	60x8 weeks	Ag80	Daily	VAC95	75	All	4.981	0.003	0.000	0.003	0.000	0.003	0.099	2390.742	1.464	0.044	1.508	0.095	1.300	47.410	23907	14.640	0.440	15.080	0.950	13.000	474.100
150	original	0.6	60x8 weeks	Ag80	Daily	VAC95	75	All	4.981	0.003	0.000	0.003	0.000	0.003	0.099	2390.742	1.464	0.044	1.508	0.095	1.300	47.410	23907	14.640	0.440	15.080	0.950	13.000	474.100
150	original	0.6	60x8 weeks	Ag80	Daily	VAC95	75	All	4.981	0.003	0.000	0.003	0.000	0.003	0.099	2390.742	1.464	0.044	1.508	0.095	1.300	47.410	23907	14.640	0.440	15.080	0.950	13.000	474.100
150	original	1	60x8 weeks	Ag80	Daily	VAC95	75	All	4.980	0.003	0.000	0.003	0.000	0.003	0.099	2390.449	1.459	0.076	1.535	0.114	1.322	47.382	23904	14.590	0.760	15.350	1.140	13.220	473.820
150	original	1	60x8 weeks	Ag80	Daily	VAC95	75	All	4.980	0.003	0.000	0.003	0.000	0.003	0.099	2390.449	1.459	0.076	1.535	0.114	1.322	47.382	23904	14.590	0.760	15.350	1.140	13.220	473.820
150	original	1	60x8 weeks	Ag80	Daily	VAC95	75	All	4.980	0.003	0.000	0.003	0.000	0.003	0.099	2390.449	1.459	0.076	1.535	0.114	1.322	47.382	23904	14.590	0.760	15.350	1.140	13.220	473.820
150	original	1.2	60x8 weeks	Ag80	Daily	VAC95	75	All	4.980	0.003	0.000	0.003	0.000	0.003	0.099	2390.603	1.492	0.082	1.574	0.114	1.324	47.420	23906	14.920	0.820	15.740	1.140	13.240	474.200
150	original	1.2	60x8 weeks	Ag80	Daily	VAC95	75	All	4.980	0.003	0.000	0.003	0.000	0.003	0.099	2390.603	1.492	0.082	1.574	0.114	1.324	47.420	23906	14.920	0.820	15.740	1.140	13.240	474.200
150	original	1.2	60x8 weeks	Ag80	Daily	VAC95	75	All	4.980	0.003	0.000	0.003	0.000	0.003	0.099	2390.603	1.492	0.082	1.574	0.114	1.324	47.420	23906	14.920	0.820	15.740	1.140	13.240	474.200
150	alpha	0.6	60x8 weeks	Ag80	Daily	VAC95	75	All	4.976	0.003	0.000	0.003	0.000	0.004	0.099	2388.295	1.558	0.103	1.661	0.037	1.727	47.369	23883	15.580	1.030	16.610	0.370	17.270	473.690
150	alpha	0.6	60x8 weeks	Ag80	Daily	VAC95	75	All	4.976	0.003	0.000	0.003	0.000	0.004	0.099	2388.295	1.558	0.103	1.661	0.037	1.727	47.369	23883	15.580	1.030	16.610	0.370	17.270	473.690
150	alpha	0.6	60x8 weeks	Ag80	Daily	VAC95	75	All	4.976	0.003	0.000	0.003	0.000	0.004	0.099	2388.295	1.558	0.103	1.661	0.037	1.727	47.369	23883	15.580	1.030	16.610	0.370	17.270	473.690
150	alpha	1	60x8 weeks	Ag80	Daily	VAC95	75	All	4.975	0.003	0.000	0.004	0.000	0.004	0.098	2387.826	1.566	0.164	1.730	0.039	1.793	47.002	23878	15.660	1.640	17.300	0.390	17.930	470.020
150	alpha	1	60x8 weeks	Ag80	Daily	VAC95	75	All	4.975	0.003	0.000	0.004	0.000	0.004	0.098	2387.826	1.566	0.164	1.730	0.039	1.793	47.002	23878	15.660	1.640	17.300	0.390	17.930	470.020
150	alpha	1	60x8 weeks	Ag80	Daily	VAC95	75	All	4.975	0.003	0.000	0.004	0.000	0.004	0.098	2387.826	1.566	0.164	1.730	0.039	1.793	47.002	23878	15.660	1.640	17.300	0.390	17.930	470.020
150	alpha	1.2	60x8 weeks	Ag80	Daily	VAC95	75	All	4.974	0.003	0.000	0.004	0.000	0.004	0.099	2387.727	1.554	0.179	1.733	0.039	1.816	47.547	23877	15.540	1.790	17.330	0.390	18.160	475.470
150	alpha	1.2	60x8 weeks	Ag80	Daily	VAC95	75	All	4.974	0.003	0.000	0.004	0.000	0.004	0.099	2387.727	1.554	0.179	1.733	0.039	1.816	47.547	23877	15.540	1.790	17.330	0.390	18.160	475.470
150	alpha	1.2	60x8 weeks	Ag80	Daily	VAC95	75	All	4.974	0.003	0.000	0.004	0.000	0.004	0.099	2387.727	1.554	0.179	1.733	0.039	1.816	47.547	23877	15.540	1.790	17.330	0.390	18.160	475.470
150	delta	0.6	60x8 weeks	Ag80	Daily	VAC95	75	All	4.971	0.004	0.000	0.004	0.000	0.004	0.098	2386.303	1.735	0.224	1.959	0.046	2.005	46.950	23863	17.350	2.240	19.590	0.460	20.050	469.500
150	delta	0.6	60x8 weeks	Ag80	Daily	VAC95	75	All	4.971	0.004	0.000	0.004	0.000	0.004	0.098	2386.303	1.735	0.224	1.959	0.046	2.005	46.950	23863	17.350	2.240	19.590	0.460	20.050	469.500
150	delta	0.6	60x8 weeks	Ag80	Daily	VAC95	75	All	4.971	0.004	0.000	0.004	0.000	0.004	0.098	2386.303	1.735	0.224	1.959	0.046	2.005	46.950	23863	17.350	2.240	19.590	0.460	20.050	469.500
150	delta	1	60x8 weeks	Ag80	Daily	VAC95	75	All	4.967	0.004	0.001	0.005	0.000	0.005	0.099	2383.940	1.824	0.440	2.264	0.077	2.319	47.436	23839	18.240	4.400	22.640	0.770	23.190	474.360
150	delta	1	60x8 weeks	Ag80	Daily	VAC95	75	All	4.967	0.004	0.001	0.005	0.000	0.005	0.099	2383.940	1.824	0.440	2.264	0.077	2.319	47.436	23839	18.240	4.400	22.640	0.770	23.190	474.360
150	delta	1	60x8 weeks	Ag80	Daily	VAC95	75	All	4.967	0.004	0.001	0.005	0.000	0.005	0.099	2383.940	1.824	0.440	2.264	0.077	2.319	47.436	23839	18.240	4.400	22.640	0.770	23.190	474.360
150	delta	1.2	60x8 weeks	Ag80	Daily	VAC95	75	All	4.968	0.004	0.001	0.005	0.000	0.005	0.098	2384.627	1.716	0.461	2.177	0.063	2.244	47.164	23846	17.160	4.610	21.770	0.630	22.440	471.640
150	delta	1.2	60x8 weeks	Ag80	Daily	VAC95	75	All	4.968	0.004	0.001	0.005	0.000	0.005	0.098	2384.627	1.716	0.461	2.177	0.063	2.244	47.164	23846	17.160	4.610	21.770	0.630	22.440	471.640
150	delta	1.2	60x8 weeks	Ag80	Daily	VAC95	75	All	4.968	0.004	0.001	0.005	0.000	0.005	0.098	2384.627	1.716	0.461	2.177	0.063	2.244	47.164	23846	17.160	4.610	21.770	0.630	22.440	471.640
150	original	0.6	60x8 weeks	Ag80	Daily	VAC95	100	All	4.998	0.001	0.000	0.001	0.000	0.000	0.100	2398.884	0.337	0.002	0.339	0.105	0.068	48.168	23989	3.370	0.020	3.390	1.050	0.680	481.680
150	original	0.6	60x8 weeks	Ag80	Daily	VAC95	100	All	4.998	0.001	0.000	0.001	0.000	0.000	0.100	2398.884	0.337	0.002	0.339	0.105	0.068	48.168	23989	3.370	0.020	3.390	1.050	0.680	481.680
150	original	0.6	60x8 weeks	Ag80	Daily	VAC95	100	All	4.998	0.001	0.000	0.001	0.000	0.000	0.100	2398.884	0.337	0.002	0.339	0.105	0.068	48.168	23989	3.370	0.020	3.390	1.050	0.680	481.680
150	original	1	60x8 weeks	Ag80	Daily	VAC95	100	All	4.998	0.001	0.000	0.001	0.000	0.000	0.100	2398.863	0.345	0.000	0.345	0.100	0.071	47.872	23989	3.450	0.000	3.450	1.000	0.710	478.720
150	original	1	60x8 weeks	Ag80	Daily	VAC95	100	All	4.998	0.001	0.000	0.001	0.000	0.000	0.100	2398.863	0.345	0.000	0.345	0.100	0.071	47.872	23989	3.450	0.000	3.450	1.000	0.710	478.720
150	original	1	60x8 weeks	Ag80	Daily	VAC95	100	All	4.998	0.001	0.000	0.001	0.000	0.000	0.100	2398.863	0.345	0.000	0.345	0.100	0.071	47.872	23989	3.450	0.000	3.450	1.000	0.710	478.720
150	original	1.2	60x8 weeks	Ag80	Daily	VAC95	100	All	4.998	0.001	0.000	0.001	0.000	0.000	0.099	2398.856	0.353	0.000	0.353	0.096	0.080	47.629	23989	3.530	0.000	3.530	0.960	0.800	476.290
150	original	1.2	60x8 weeks	Ag80	Daily	VAC95	100	All	4.998	0.001	0.000	0.001	0.000	0.000	0.099	2398.856	0.353	0.000	0.353	0.096	0.080	47.629	23989	3.530	0.000	3.530	0.960	0.800	476.290
150	original	1.2	60x8 weeks	Ag80	Daily	VAC95	100	All	4.998	0.001	0.000	0.001	0.000	0.000	0.099	2398.856	0.353	0.000	0.353	0.096	0.080	47.629	23989	3.530	0.000	3.530	0.960	0.800	476.290
150	alpha	0.6	60x8 weeks	Ag80	Daily	VAC95	100	All	4.993	0.001	0.000	0.001	0.000	0.001	0.099	2396.597	0.498	0.002	0.500	0.019	0.484	47.595	23966	4.980	0.020	5.000	0.190	4.840	475.950
150	alpha	0.6	60x8 weeks	Ag80	Daily	VAC95	100	All	4.993	0.001	0.000	0.001	0.000	0.001	0.099	2396.597	0.498	0.002	0.500	0.019	0.484	47.595	23966	4.980	0.020	5.000	0.190	4.840	475.950
150	alpha	0.6	60x8 weeks	Ag80	Daily	VAC95	100	All	4.993	0.001	0.000	0.001	0.000	0.001	0.099	2396.597	0.498	0.002	0.500	0.019	0.484	47.595	23966	4.980	0.020	5.000	0.190	4.840	475.950
150	alpha	1	60x8 weeks	Ag80	Daily	VAC95	100	All	4.993	0.001	0.000	0.001	0.000	0.001	0.100	2396.737	0.471	0.009	0.480	0.012	0.476	47.993	23967	4.710	0.090	4.800	0.120	4.760	479.930
150	alpha	1	60x8 weeks	Ag80	Daily	VAC95	100	All	4.993	0.001	0.000	0.001	0.000	0.001	0.100	2396.737	0.471	0.009	0.480	0.012	0.476	47.993	23967	4.710	0.090	4.800	0.120	4.760	479.930
150	alpha	1	60x8 weeks	Ag80	Daily	VAC95	100	All	4.993	0.001	0.000	0.001	0.000	0.001	0.100	2396.737	0.471	0.009	0.480	0.012	0.476	47.993	23967	4.710	0.090	4.800	0.120	4.760	479.930
150	alpha	1.2	60x8 weeks	Ag80	Daily	VAC95	100	All	4.993	0.001	0.000	0.001	0.000	0.001	0.100	2396.708	0.491	0.008	0.499	0.014	0.487	47.881	23967	4.910	0.080	4.990	0.140	4.870	478.810
150	alpha	1.2	60x8 weeks	Ag80	Daily	VAC95	100	All	4.993	0.001	0.000	0.001	0.000	0.001	0.100	2396.708	0.491	0.008	0.499	0.014	0.487	47.881	23967	4.910	0.080	4.990	0.140	4.870	478.810
150	alpha	1.2	60x8 weeks	Ag80	Daily	VAC95	100	All	4.993	0.001	0.000	0.001	0.000	0.001	0.100	2396.708	0.491	0.008	0.499	0.014	0.487	47.881	23967	4.910	0.080	4.990	0.140	4.870	478.810
150	delta	0.6	60x8 weeks	Ag80	Daily	VAC95	100	All	4.990	0.001	0.000	0.002	0.000	0.001	0.100	2395.082	0.696	0.031	0.727	0.027	0.712	47.821	23951	6.960	0.310	7.270	0.270	7.120	478.210
150	delta	0.6	60x8 weeks	Ag80	Daily	VAC95	100	All	4.990	0.001	0.000	0.002	0.000	0.001	0.100	2395.082	0.696	0.031	0.727	0.027	0.712	47.821	23951	6.960	0.310	7.270	0.270	7.120	478.210
150	delta	0.6	60x8 weeks	Ag80	Daily	VAC95	100	All	4.990	0.001	0.000	0.002	0.000	0.001	0.100	2395.082	0.696	0.031	0.727	0.027	0.712	47.821	23951	6.960	0.310	7.270	0.270	7.120	478.210
150	delta	1	60x8 weeks	Ag80	Daily	VAC95	100	All	4.989	0.002	0.000	0.002	0.000	0.002	0.100	2394.921	0.724	0.036	0.760	0.025	0.738	47.971	23949	7.240	0.360	7.600	0.250	7.380	479.710
150	delta	1	60x8 weeks	Ag80	Daily	VAC95	100	All	4.989	0.002	0.000	0.002	0.000	0.002	0.100	2394.921	0.724	0.036	0.760	0.025	0.738	47.971	23949	7.240	0.360	7.600	0.250	7.380	479.710
150	delta	1	60x8 weeks	Ag80	Daily	VAC95	100	All	4.989	0.002	0.000	0.002	0.000	0.002	0.100	2394.921	0.724	0.036	0.760	0.025	0.738	47.971	23949	7.240	0.360	7.600	0.250	7.380	479.710
150	delta	1.2	60x8 weeks	Ag80	Daily	VAC95	100	All	4.990	0.001	0.000	0.001	0.000	0.001	0.100	2395.187	0.655	0.034	0.689	0.024	0.710	47.826	23952	6.550	0.340	6.890	0.240	7.100	478.260
150	delta	1.2	60x8 weeks	Ag80	Daily	VAC95	100	All	4.990	0.001	0.000	0.001	0.000	0.001	0.100	2395.187	0.655	0.034	0.689	0.024	0.710	47.826	23952	6.550	0.340	6.890	0.240	7.100	478.260
150	delta	1.2	60x8 weeks	Ag80	Daily	VAC95	100	All	4.990	0.001	0.000	0.001	0.000	0.001	0.100	2395.187	0.655	0.034	0.689	0.024	0.710	47.826	23952	6.550	0.340	6.890	0.240	7.100	478.260
150	original	0.6	60x8 weeks	Ag80	Daily	VAC70	25	All	4.940	0.008	0.001	0.009	0.001	0.009	0.096	2371.224	4.010	0.428	4.438	0.256	4.090	45.989	23712	40.100	4.280	44.380	2.560	40.900	459.890
150	original	0.6	60x8 weeks	Ag80	Daily	VAC70	25	All	4.940	0.008	0.001	0.009	0.001	0.009	0.096	2371.224	4.010	0.428	4.438	0.256	4.090	45.989	23712	40.100	4.280	44.380	2.560	40.900	459.890
150	original	0.6	60x8 weeks	Ag80	Daily	VAC70	25	All	4.940	0.008	0.001	0.009	0.001	0.009	0.096	2371.224	4.010	0.428	4.438	0.256	4.090	45.989	23712	40.100	4.280	44.380	2.560	40.900	459.890

150	original	1	60x8 weeks	Ag80	Daily	VAC70	25	All	4.938	0.008	0.001	0.009	0.001	0.009	0.096	2370.346	3.838	0.693	4.531	0.256	4.225	46.295	23703	38.380	6.930	45.310	2.560	42.250	462.950
150	original	1	60x8 weeks	Ag80	Daily	VAC70	25	All	4.938	0.008	0.001	0.009	0.001	0.009	0.096	2370.346	3.838	0.693	4.531	0.256	4.225	46.295	23703	38.380	6.930	45.310	2.560	42.250	462.950
150	original	1	60x8 weeks	Ag80	Daily	VAC70	25	All	4.938	0.008	0.001	0.009	0.001	0.009	0.096	2370.346	3.838	0.693	4.531	0.256	4.225	46.295	23703	38.380	6.930	45.310	2.560	42.250	462.950
150	original	1.2	60x8 weeks	Ag80	Daily	VAC70	25	All	4.934	0.008	0.002	0.010	0.001	0.009	0.096	2368.264	3.927	0.934	4.861	0.302	4.494	46.010	23683	39.270	9.340	48.610	3.020	44.940	460.100
150	original	1.2	60x8 weeks	Ag80	Daily	VAC70	25	All	4.934	0.008	0.002	0.010	0.001	0.009	0.096	2368.264	3.927	0.934	4.861	0.302	4.494	46.010	23683	39.270	9.340	48.610	3.020	44.940	460.100
150	original	1.2	60x8 weeks	Ag80	Daily	VAC70	25	All	4.934	0.008	0.002	0.010	0.001	0.009	0.096	2368.264	3.927	0.934	4.861	0.302	4.494	46.010	23683	39.270	9.340	48.610	3.020	44.940	460.100
150	alpha	0.6	60x8 weeks	Ag80	Daily	VAC70	25	All	4.930	0.008	0.002	0.010	0.000	0.010	0.095	2366.541	4.021	0.749	4.770	0.112	4.932	45.815	23665	40.210	7.490	47.700	1.120	49.320	458.150
150	alpha	0.6	60x8 weeks	Ag80	Daily	VAC70	25	All	4.930	0.008	0.002	0.010	0.000	0.010	0.095	2366.541	4.021	0.749	4.770	0.112	4.932	45.815	23665	40.210	7.490	47.700	1.120	49.320	458.150
150	alpha	0.6	60x8 weeks	Ag80	Daily	VAC70	25	All	4.930	0.008	0.002	0.010	0.000	0.010	0.095	2366.541	4.021	0.749	4.770	0.112	4.932	45.815	23665	40.210	7.490	47.700	1.120	49.320	458.150
150	alpha	1	60x8 weeks	Ag80	Daily	VAC70	25	All	4.924	0.008	0.003	0.011	0.000	0.011	0.096	2363.353	3.924	1.263	5.187	0.119	5.383	45.885	23634	39.240	12.630	51.870	1.190	53.830	458.850
150	alpha	1	60x8 weeks	Ag80	Daily	VAC70	25	All	4.924	0.008	0.003	0.011	0.000	0.011	0.096	2363.353	3.924	1.263	5.187	0.119	5.383	45.885	23634	39.240	12.630	51.870	1.190	53.830	458.850
150	alpha	1	60x8 weeks	Ag80	Daily	VAC70	25	All	4.924	0.008	0.003	0.011	0.000	0.011	0.096	2363.353	3.924	1.263	5.187	0.119	5.383	45.885	23634	39.240	12.630	51.870	1.190	53.830	458.850
150	alpha	1.2	60x8 weeks	Ag80	Daily	VAC70	25	All	4.916	0.008	0.004	0.012	0.000	0.012	0.095	2359.664	4.016	1.720	5.736	0.140	5.948	45.788	23597	40.160	17.200	57.360	1.400	59.480	457.880
150	alpha	1.2	60x8 weeks	Ag80	Daily	VAC70	25	All	4.916	0.008	0.004	0.012	0.000	0.012	0.095	2359.664	4.016	1.720	5.736	0.140	5.948	45.788	23597	40.160	17.200	57.360	1.400	59.480	457.880
150	alpha	1.2	60x8 weeks	Ag80	Daily	VAC70	25	All	4.916	0.008	0.004	0.012	0.000	0.012	0.095	2359.664	4.016	1.720	5.736	0.140	5.948	45.788	23597	40.160	17.200	57.360	1.400	59.480	457.880
150	delta	0.6	60x8 weeks	Ag80	Daily	VAC70	25	All	4.912	0.009	0.004	0.012	0.000	0.013	0.095	2357.982	4.140	1.728	5.868	0.163	6.109	45.371	23580	41.400	17.280	58.680	1.630	61.090	453.710
150	delta	0.6	60x8 weeks	Ag80	Daily	VAC70	25	All	4.912	0.009	0.004	0.012	0.000	0.013	0.095	2357.982	4.140	1.728	5.868	0.163	6.109	45.371	23580	41.400	17.280	58.680	1.630	61.090	453.710
150	delta	0.6	60x8 weeks	Ag80	Daily	VAC70	25	All	4.912	0.009	0.004	0.012	0.000	0.013	0.095	2357.982	4.140	1.728	5.868	0.163	6.109	45.371	23580	41.400	17.280	58.680	1.630	61.090	453.710
150	delta	1	60x8 weeks	Ag80	Daily	VAC70	25	All	4.891	0.008	0.007	0.015	0.000	0.016	0.094	2347.555	4.033	3.336	7.369	0.206	7.606	45.218	23476	40.330	33.360	73.690	2.060	76.060	452.180
150	delta	1	60x8 weeks	Ag80	Daily	VAC70	25	All	4.891	0.008	0.007	0.015	0.000	0.016	0.094	2347.555	4.033	3.336	7.369	0.206	7.606	45.218	23476	40.330	33.360	73.690	2.060	76.060	452.180
150	delta	1	60x8 weeks	Ag80	Daily	VAC70	25	All	4.891	0.008	0.007	0.015	0.000	0.016	0.094	2347.555	4.033	3.336	7.369	0.206	7.606	45.218	23476	40.330	33.360	73.690	2.060	76.060	452.180
150	delta	1.2	60x8 weeks	Ag80	Daily	VAC70	25	All	4.868	0.008	0.010	0.018	0.001	0.019	0.093	2336.490	3.973	4.738	8.711	0.244	9.124	44.412	23365	39.730	47.380	87.110	2.440	91.240	444.120
150	delta	1.2	60x8 weeks	Ag80	Daily	VAC70	25	All	4.868	0.008	0.010	0.018	0.001	0.019	0.093	2336.490	3.973	4.738	8.711	0.244	9.124	44.412	23365	39.730	47.380	87.110	2.440	91.240	444.120
150	delta	1.2	60x8 weeks	Ag80	Daily	VAC70	25	All	4.868	0.008	0.010	0.018	0.001	0.019	0.093	2336.490	3.973	4.738	8.711	0.244	9.124	44.412	23365	39.730	47.380	87.110	2.440	91.240	444.120
150	original	0.6	60x8 weeks	Ag80	Daily	VAC70	50	All	4.955	0.007	0.000	0.007	0.001	0.006	0.097	2378.579	3.309	0.233	3.542	0.380	2.884	46.390	23786	33.090	2.330	35.420	3.800	28.840	463.900
150	original	0.6	60x8 weeks	Ag80	Daily	VAC70	50	All	4.955	0.007	0.000	0.007	0.001	0.006	0.097	2378.579	3.309	0.233	3.542	0.380	2.884	46.390	23786	33.090	2.330	35.420	3.800	28.840	463.900
150	original	0.6	60x8 weeks	Ag80	Daily	VAC70	50	All	4.955	0.007	0.000	0.007	0.001	0.006	0.097	2378.579	3.309	0.233	3.542	0.380	2.884	46.390	23786	33.090	2.330	35.420	3.800	28.840	463.900
150	original	1	60x8 weeks	Ag80	Daily	VAC70	50	All	4.956	0.007	0.001	0.007	0.001	0.006	0.098	2378.765	3.175	0.396	3.571	0.370	2.851	46.858	23788	31.750	3.960	35.710	3.700	28.510	468.580
150	original	1	60x8 weeks	Ag80	Daily	VAC70	50	All	4.956	0.007	0.001	0.007	0.001	0.006	0.098	2378.765	3.175	0.396	3.571	0.370	2.851	46.858	23788	31.750	3.960	35.710	3.700	28.510	468.580
150	original	1	60x8 weeks	Ag80	Daily	VAC70	50	All	4.956	0.007	0.001	0.007	0.001	0.006	0.098	2378.765	3.175	0.396	3.571	0.370	2.851	46.858	23788	31.750	3.960	35.710	3.700	28.510	468.580
150	original	1.2	60x8 weeks	Ag80	Daily	VAC70	50	All	4.954	0.007	0.001	0.008	0.001	0.006	0.096	2377.787	3.217	0.445	3.662	0.438	2.921	46.092	23778	32.170	4.450	36.620	4.380	29.210	460.920
150	original	1.2	60x8 weeks	Ag80	Daily	VAC70	50	All	4.954	0.007	0.001	0.008	0.001	0.006	0.096	2377.787	3.217	0.445	3.662	0.438	2.921	46.092	23778	32.170	4.450	36.620	4.380	29.210	460.920
150	original	1.2	60x8 weeks	Ag80	Daily	VAC70	50	All	4.954	0.007	0.001	0.008	0.001	0.006	0.096	2377.787	3.217	0.445	3.662	0.438	2.921	46.092	23778	32.170	4.450	36.620	4.380	29.210	460.920
150	alpha	0.6	60x8 weeks	Ag80	Daily	VAC70	50	All	4.948	0.007	0.001	0.007	0.000	0.008	0.098	2375.184	3.168	0.423	3.591	0.091	3.659	46.845	23752	31.680	4.230	35.910	0.910	36.590	468.450
150	alpha	0.6	60x8 weeks	Ag80	Daily	VAC70	50	All	4.948	0.007	0.001	0.007	0.000	0.008	0.098	2375.184	3.168	0.423	3.591	0.091	3.659	46.845	23752	31.680	4.230	35.910	0.910	36.590	468.450
150	alpha	0.6	60x8 weeks	Ag80	Daily	VAC70	50	All	4.948	0.007	0.001	0.007	0.000	0.008	0.098	2375.184	3.168	0.423	3.591	0.091	3.659	46.845	23752	31.680	4.230	35.910	0.910	36.590	468.450
150	alpha	1	60x8 weeks	Ag80	Daily	VAC70	50	All	4.940	0.007	0.002	0.008	0.000	0.009	0.096	2371.287	3.249	0.823	4.072	0.105	4.207	46.166	23713	32.490	8.230	40.720	1.050	42.070	461.660
150	alpha	1	60x8 weeks	Ag80	Daily	VAC70	50	All	4.940	0.007	0.002	0.008	0.000	0.009	0.096	2371.287	3.249	0.823	4.072	0.105	4.207	46.166	23713	32.490	8.230	40.720	1.050	42.070	461.660
150	alpha	1	60x8 weeks	Ag80	Daily	VAC70	50	All	4.940	0.007	0.002	0.008	0.000	0.009	0.096	2371.287	3.249	0.823	4.072	0.105	4.207	46.166	23713	32.490	8.230	40.720	1.050	42.070	461.660
150	alpha	1.2	60x8 weeks	Ag80	Daily	VAC70	50	All	4.938	0.007	0.002	0.009	0.000	0.009	0.097	2370.031	3.244	1.041	4.285	0.108	4.401	46.375	23700	32.440	10.410	42.850	1.080	44.010	463.750
150	alpha	1.2	60x8 weeks	Ag80	Daily	VAC70	50	All	4.938	0.007	0.002	0.009	0.000	0.009	0.097	2370.031	3.244	1.041	4.285	0.108	4.401	46.375	23700	32.440	10.410	42.850	1.080	44.010	463.750
150	alpha	1.2	60x8 weeks	Ag80	Daily	VAC70	50	All	4.938	0.007	0.002	0.009	0.000	0.009	0.097	2370.031	3.244	1.041	4.285	0.108	4.401	46.375	23700	32.440	10.410	42.850	1.080	44.010	463.750
150	delta	0.6	60x8 weeks	Ag80	Daily	VAC70	50	All	4.936	0.007	0.002	0.009	0.000	0.009	0.097	2369.086	3.349	1.010	4.359	0.139	4.470	46.475	23691	33.490	10.100	43.590	1.390	44.700	464.750
150	delta	0.6	60x8 weeks	Ag80	Daily	VAC70	50	All	4.936	0.007	0.002	0.009	0.000	0.009	0.097	2369.086	3.349	1.010	4.359	0.139	4.470	46.475	23691	33.490	10.100	43.590	1.390	44.700	464.750
150	delta	0.6	60x8 weeks	Ag80	Daily	VAC70	50	All	4.936	0.007	0.002	0.009	0.000	0.009	0.097	2369.086	3.349	1.010	4.359	0.139	4.470	46.475	23691	33.490	10.100	43.590	1.390	44.700	464.750
150	delta	1	60x8 weeks	Ag80	Daily	VAC70	50	All	4.922	0.007	0.004	0.011	0.000	0.011	0.095	2362.692	3.396	1.911	5.307	0.154	5.481	45.782	23627	33.960	19.110	53.070	1.540	54.810	457.820
150	delta	1	60x8 weeks	Ag80	Daily	VAC70	50	All	4.922	0.007	0.004	0.011	0.000	0.011	0.095	2362.692	3.396	1.911	5.307	0.154	5.481	45.782	23627	33.960	19.110	53.070	1.540	54.810	457.820
150	delta	1	60x8 weeks	Ag80	Daily	VAC70	50	All	4.922	0.007	0.004	0.011	0.000	0.011	0.095	2362.692	3.396	1.911	5.307	0.154	5.481	45.782	23627	33.960	19.110	53.070	1.540	54.810	457.820
150	delta	1.2	60x8 weeks	Ag80	Daily	VAC70	50	All	4.913	0.007	0.005	0.012	0.000	0.013	0.095	2358.194	3.321	2.566	5.887	0.165	6.067	45.441	23582	33.210	25.660	58.870	1.650	60.670	454.410
150	delta	1.2	60x8 weeks	Ag80	Daily	VAC70	50	All	4.913	0.007	0.005	0.012	0.000	0.013	0.095	2358.194	3.321	2.566	5.887	0.165	6.067	45.441	23582	33.210	25.660	58.870	1.650	60.670	454.410
150	delta	1.2	60x8 weeks	Ag80	Daily	VAC70	50	All	4.913	0.007	0.005	0.012	0.000	0.013	0.095	2358.194	3.321	2.566	5.887	0.165	6.067	45.441	23582	33.210	25.660	58.870	1.650	60.670	454.410
150	original	0.6	60x8 weeks	Ag80	Daily	VAC70	75	All	4.973	0.005	0.000	0.005	0.001	0.003	0.098	2387.267	2.404	0.081	2.485	0.489	1.470	47.197	23873	24.040	0.810	24.850	4.890	14.700	471.970
150	original	0.6	60x8 weeks	Ag80	Daily	VAC70	75	All	4.973	0.005	0.000	0.005	0.001	0.003	0.098	2387.267	2.404	0.081	2.485	0.489	1.470	47.197	23873	24.040	0.810	24.850	4.890	14.700	471.970
150	original	0.6	60x8 weeks	Ag80	Daily	VAC70	75	All	4.973	0.005	0.000	0.005	0.001	0.003	0.098	2387.267	2.404	0.081	2.485	0.489	1.470	47.197	23873	24.040	0.810	24.850	4.890	14.700	471.970
150	original	1	60x8 weeks	Ag80	Daily	VAC70	75	All	4.973	0.005	0.000	0.005	0.001	0.003	0.097	2387.059	2.373	0.147	2.520	0.498	1.468	46.706	23871	23.730	1.470	25.200	4.980	14.680	467.060
150	original	1	60x8 weeks	Ag80	Daily	VAC70	75	All	4.973	0.005	0.000	0.005	0.001	0.003	0.097	2387.059	2.373	0.147	2.520	0.498	1.468	46.706	23871	23.730	1.470	25.200	4.980	14.680	467.060
150	original	1	60x8 weeks	Ag80	Daily	VAC70	75	All	4.973	0.005	0.000	0.005	0.001	0.003	0.097	2387.059	2.373	0.147	2.520	0.498	1.468	46.706	23871	23.730	1.470	25.200	4.980	14.680	467.060
150	original	1.2	60x8 weeks	Ag80	Daily	VAC70	75	All	4.972	0.005	0.000	0.005	0.001	0.003	0.098	2386.503	2.455	0.163	2.618	0.488	1.574	46.966	23865	24.550	1.630	26.180	4.880	15.740	469.660
150	original	1.2	60x8 weeks	Ag80	Daily	VAC70	75	All	4.972	0.005	0.000	0.005	0.001	0.003	0.098	2386.503	2.455	0.163	2.618	0.488	1.574	46.966	23865	24.550	1.630	26.180	4.880	15.740	469.660
150	original	1.2	60x8 weeks	Ag80	Daily	VAC70	75	All	4.972	0.005	0.000	0.005	0.001	0.003	0.098	2386.503	2.455	0.163	2.618	0.488	1.574	46.966	23865	24.550	1.630	26.180	4.880	15.740	469.660
150	alpha	0.6	60x8 weeks	Ag80	Daily	VAC70	75	All	4.963	0.005	0.000	0.005	0.000	0.005	0.098	2382.198	2.413	0.184	2.597	0.066	2.616	47.082	23822	24.130	1.840	25.970	0.660	26.160	470.820
150	alpha	0.6	60x8 weeks	Ag80	Daily	VAC70	75	All	4.963	0.005	0.000	0.005	0.000	0.005	0.098	2382.198	2.413	0.184	2.597	0.066	2.616	47.082	23822	24.130	1.840	25.970	0.660	26.160	470.820
150	alpha	0.6	60x8 weeks	Ag80	Daily	VAC70	75	All	4.963	0.005	0.000	0.005	0.000	0.005	0.098	2382.198	2.413	0.184	2.597	0.066	2.616	47.082	23822	24.130	1.840	25.970	0.660	26.160	470.820
150	alpha	1	60x8 weeks	Ag80	Daily	VAC70	75	All	4.961	0.005	0.001	0.006	0.000	0.006	0.098	2381.325	2.365	0.304	2.669	0.078	2.729	47.133	23813	23.650	3.040	26.690	0.780	27.290	471.330
150	alpha	1	60x8 weeks	Ag80	Daily	VAC70	75	All	4.961	0.005	0.001	0.006	0.000	0.006	0.098	2381.325	2.365	0.304	2.669	0.078	2.729	47.133	23813	23.650	3.040	26.690	0.780	27.290	471.330
150	alpha	1	60x8 weeks	Ag80	Daily	VAC70	75	All	4.961	0.005	0.001	0.006	0.000	0.006	0.098	2381.325	2.365	0.304	2.669	0.078	2.729	47.133	23813	23.650	3.040	26.690	0.780	27.290	471.330
150	alpha	1.2	60x8 weeks	Ag80	Daily	VAC70	75	All	4.959	0.005	0.001	0.006	0.000	0.006	0.098	2380.481	2.396	0.447	2.843	0.075	2.876	46.819	23805	23.960	4.470	28.430	0.750	28.760	468.190
150	alpha	1.2	60x8 weeks	Ag80	Daily	VAC70	75	All	4.959	0.005	0.001	0.006	0.000	0.006	0.098	2380.481	2.396	0.447	2.843	0.075	2.876	46.819	23805	23.960	4.470	28.430	0.750	28.760	468.190
150	alpha	1.2	60x8 weeks	Ag80	Daily	VAC70	75	All	4.959	0.005	0.001	0.006	0.000	0.006	0.098	2380.481	2.396	0.447	2.843	0.075	2.876	46.819	23805	23.960	4.470	28.430	0.750	28.760	468.190
150	delta	0.6	60x8 weeks	Ag80	Daily	VAC70	75	All	4.952	0.006	0.001	0.007	0.000	0.007	0.098	2376.815	2.793	0.523	3.316	0.099	3.372	46.838	23768	27.930	5.230	33.160	0.990	33.720	468.380
150	delta	0.6	60x8 weeks	Ag80	Daily	VAC70	75	All	4.952	0.006	0.001	0.007	0.000	0.007	0.098	2376.815	2.793	0.523	3.316	0.099	3.372	46.838	23768	27.930	5.230	33.160	0.990	33.720	468.380

150	delta	0.6	60x8 weeks	Ag80	Daily	VAC70	75	All	4.952	0.006	0.001	0.007	0.000	0.007	0.098	2376.815	2.793	0.523	3.316	0.099	3.372	46.838	23768	27.930	5.230	33.160	0.990	33.720	468.380
150	delta	1	60x8 weeks	Ag80	Daily	VAC70	75	All	4.944	0.006	0.002	0.008	0.000	0.008	0.097	2373.143	2.793	1.041	3.834	0.140	3.876	46.766	23731	27.930	10.410	38.340	1.400	38.760	467.660
150	delta	1	60x8 weeks	Ag80	Daily	VAC70	75	All	4.944	0.006	0.002	0.008	0.000	0.008	0.097	2373.143	2.793	1.041	3.834	0.140	3.876	46.766	23731	27.930	10.410	38.340	1.400	38.760	467.660
150	delta	1	60x8 weeks	Ag80	Daily	VAC70	75	All	4.944	0.006	0.002	0.008	0.000	0.008	0.097	2373.143	2.793	1.041	3.834	0.140	3.876	46.766	23731	27.930	10.410	38.340	1.400	38.760	467.660
150	delta	1.2	60x8 weeks	Ag80	Daily	VAC70	75	All	4.939	0.006	0.003	0.009	0.000	0.009	0.097	2370.956	2.787	1.297	4.084	0.114	4.208	46.502	23710	27.870	12.970	40.840	1.140	42.080	465.020
150	delta	1.2	60x8 weeks	Ag80	Daily	VAC70	75	All	4.939	0.006	0.003	0.009	0.000	0.009	0.097	2370.956	2.787	1.297	4.084	0.114	4.208	46.502	23710	27.870	12.970	40.840	1.140	42.080	465.020
150	delta	1.2	60x8 weeks	Ag80	Daily	VAC70	75	All	4.939	0.006	0.003	0.009	0.000	0.009	0.097	2370.956	2.787	1.297	4.084	0.114	4.208	46.502	23710	27.870	12.970	40.840	1.140	42.080	465.020
150	original	0.6	60x8 weeks	Ag80	Daily	VAC70	100	All	4.987	0.003	0.000	0.003	0.001	0.001	0.098	2393.986	1.634	0.004	1.638	0.603	0.322	47.221	23940	16.340	0.040	16.380	6.030	3.220	472.210
150	original	0.6	60x8 weeks	Ag80	Daily	VAC70	100	All	4.987	0.003	0.000	0.003	0.001	0.001	0.098	2393.986	1.634	0.004	1.638	0.603	0.322	47.221	23940	16.340	0.040	16.380	6.030	3.220	472.210
150	original	0.6	60x8 weeks	Ag80	Daily	VAC70	100	All	4.987	0.003	0.000	0.003	0.001	0.001	0.098	2393.986	1.634	0.004	1.638	0.603	0.322	47.221	23940	16.340	0.040	16.380	6.030	3.220	472.210
150	original	1	60x8 weeks	Ag80	Daily	VAC70	100	All	4.988	0.003	0.000	0.003	0.001	0.001	0.099	2394.199	1.607	0.017	1.624	0.591	0.324	47.462	23942	16.070	0.170	16.240	5.910	3.240	474.620
150	original	1	60x8 weeks	Ag80	Daily	VAC70	100	All	4.988	0.003	0.000	0.003	0.001	0.001	0.099	2394.199	1.607	0.017	1.624	0.591	0.324	47.462	23942	16.070	0.170	16.240	5.910	3.240	474.620
150	original	1	60x8 weeks	Ag80	Daily	VAC70	100	All	4.988	0.003	0.000	0.003	0.001	0.001	0.099	2394.199	1.607	0.017	1.624	0.591	0.324	47.462	23942	16.070	0.170	16.240	5.910	3.240	474.620
150	original	1.2	60x8 weeks	Ag80	Daily	VAC70	100	All	4.987	0.003	0.000	0.003	0.001	0.001	0.098	2393.883	1.640	0.020	1.660	0.603	0.342	46.890	23939	16.400	0.200	16.600	6.030	3.420	468.900
150	original	1.2	60x8 weeks	Ag80	Daily	VAC70	100	All	4.987	0.003	0.000	0.003	0.001	0.001	0.098	2393.883	1.640	0.020	1.660	0.603	0.342	46.890	23939	16.400	0.200	16.600	6.030	3.420	468.900
150	original	1.2	60x8 weeks	Ag80	Daily	VAC70	100	All	4.987	0.003	0.000	0.003	0.001	0.001	0.098	2393.883	1.640	0.020	1.660	0.603	0.342	46.890	23939	16.400	0.200	16.600	6.030	3.420	468.900
150	alpha	0.6	60x8 weeks	Ag80	Daily	VAC70	100	All	4.976	0.003	0.000	0.004	0.000	0.003	0.099	2388.585	1.628	0.073	1.701	0.073	1.663	47.289	23886	16.280	0.730	17.010	0.730	16.630	472.890
150	alpha	0.6	60x8 weeks	Ag80	Daily	VAC70	100	All	4.976	0.003	0.000	0.004	0.000	0.003	0.099	2388.585	1.628	0.073	1.701	0.073	1.663	47.289	23886	16.280	0.730	17.010	0.730	16.630	472.890
150	alpha	0.6	60x8 weeks	Ag80	Daily	VAC70	100	All	4.976	0.003	0.000	0.004	0.000	0.003	0.099	2388.585	1.628	0.073	1.701	0.073	1.663	47.289	23886	16.280	0.730	17.010	0.730	16.630	472.890
150	alpha	1	60x8 weeks	Ag80	Daily	VAC70	100	All	4.976	0.003	0.000	0.004	0.000	0.004	0.099	2388.526	1.591	0.103	1.694	0.048	1.692	47.375	23885	15.910	1.030	16.940	0.480	16.920	473.750
150	alpha	1	60x8 weeks	Ag80	Daily	VAC70	100	All	4.976	0.003	0.000	0.004	0.000	0.004	0.099	2388.526	1.591	0.103	1.694	0.048	1.692	47.375	23885	15.910	1.030	16.940	0.480	16.920	473.750
150	alpha	1	60x8 weeks	Ag80	Daily	VAC70	100	All	4.976	0.003	0.000	0.004	0.000	0.004	0.099	2388.526	1.591	0.103	1.694	0.048	1.692	47.375	23885	15.910	1.030	16.940	0.480	16.920	473.750
150	alpha	1.2	60x8 weeks	Ag80	Daily	VAC70	100	All	4.975	0.003	0.000	0.004	0.000	0.004	0.097	2388.155	1.624	0.142	1.766	0.061	1.744	46.753	23882	16.240	1.420	17.660	0.610	17.440	467.530
150	alpha	1.2	60x8 weeks	Ag80	Daily	VAC70	100	All	4.975	0.003	0.000	0.004	0.000	0.004	0.097	2388.155	1.624	0.142	1.766	0.061	1.744	46.753	23882	16.240	1.420	17.660	0.610	17.440	467.530
150	alpha	1.2	60x8 weeks	Ag80	Daily	VAC70	100	All	4.975	0.003	0.000	0.004	0.000	0.004	0.097	2388.155	1.624	0.142	1.766	0.061	1.744	46.753	23882	16.240	1.420	17.660	0.610	17.440	467.530
150	delta	0.6	60x8 weeks	Ag80	Daily	VAC70	100	All	4.968	0.004	0.000	0.005	0.000	0.005	0.099	2384.545	2.046	0.207	2.253	0.067	2.298	47.361	23845	20.460	2.070	22.530	0.670	22.980	473.610
150	delta	0.6	60x8 weeks	Ag80	Daily	VAC70	100	All	4.968	0.004	0.000	0.005	0.000	0.005	0.099	2384.545	2.046	0.207	2.253	0.067	2.298	47.361	23845	20.460	2.070	22.530	0.670	22.980	473.610
150	delta	0.6	60x8 weeks	Ag80	Daily	VAC70	100	All	4.968	0.004	0.000	0.005	0.000	0.005	0.099	2384.545	2.046	0.207	2.253	0.067	2.298	47.361	23845	20.460	2.070	22.530	0.670	22.980	473.610
150	delta	1	60x8 weeks	Ag80	Daily	VAC70	100	All	4.964	0.004	0.001	0.005	0.000	0.005	0.098	2382.915	2.089	0.354	2.443	0.081	2.485	46.974	23829	20.890	3.540	24.430	0.810	24.850	469.740
150	delta	1	60x8 weeks	Ag80	Daily	VAC70	100	All	4.964	0.004	0.001	0.005	0.000	0.005	0.098	2382.915	2.089	0.354	2.443	0.081	2.485	46.974	23829	20.890	3.540	24.430	0.810	24.850	469.740
150	delta	1	60x8 weeks	Ag80	Daily	VAC70	100	All	4.964	0.004	0.001	0.005	0.000	0.005	0.098	2382.915	2.089	0.354	2.443	0.081	2.485	46.974	23829	20.890	3.540	24.430	0.810	24.850	469.740
150	delta	1.2	60x8 weeks	Ag80	Daily	VAC70	100	All	4.964	0.004	0.001	0.005	0.000	0.005	0.098	2382.857	2.011	0.424	2.435	0.087	2.480	47.271	23829	20.110	4.240	24.350	0.870	24.800	472.710
150	delta	1.2	60x8 weeks	Ag80	Daily	VAC70	100	All	4.964	0.004	0.001	0.005	0.000	0.005	0.098	2382.857	2.011	0.424	2.435	0.087	2.480	47.271	23829	20.110	4.240	24.350	0.870	24.800	472.710
150	delta	1.2	60x8 weeks	Ag80	Daily	VAC70	100	All	4.964	0.004	0.001	0.005	0.000	0.005	0.098	2382.857	2.011	0.424	2.435	0.087	2.480	47.271	23829	20.110	4.240	24.350	0.870	24.800	472.710
150	original	0.6	60x8 weeks	Ag80	Daily	VAC50	25	All	4.937	0.009	0.001	0.010	0.001	0.009	0.096	2369.604	4.314	0.440	4.754	0.364	4.190	46.045	23696	43.140	4.400	47.540	3.640	41.900	460.450
150	original	0.6	60x8 weeks	Ag80	Daily	VAC50	25	All	4.937	0.009	0.001	0.010	0.001	0.009	0.096	2369.604	4.314	0.440	4.754	0.364	4.190	46.045	23696	43.140	4.400	47.540	3.640	41.900	460.450
150	original	0.6	60x8 weeks	Ag80	Daily	VAC50	25	All	4.937	0.009	0.001	0.010	0.001	0.009	0.096	2369.604	4.314	0.440	4.754	0.364	4.190	46.045	23696	43.140	4.400	47.540	3.640	41.900	460.450
150	original	1	60x8 weeks	Ag80	Daily	VAC50	25	All	4.936	0.009	0.001	0.010	0.001	0.009	0.095	2369.295	4.120	0.717	4.837	0.390	4.276	45.688	23693	41.200	7.170	48.370	3.900	42.760	456.880
150	original	1	60x8 weeks	Ag80	Daily	VAC50	25	All	4.936	0.009	0.001	0.010	0.001	0.009	0.095	2369.295	4.120	0.717	4.837	0.390	4.276	45.688	23693	41.200	7.170	48.370	3.900	42.760	456.880
150	original	1	60x8 weeks	Ag80	Daily	VAC50	25	All	4.936	0.009	0.001	0.010	0.001	0.009	0.095	2369.295	4.120	0.717	4.837	0.390	4.276	45.688	23693	41.200	7.170	48.370	3.900	42.760	456.880
150	original	1.2	60x8 weeks	Ag80	Daily	VAC50	25	All	4.931	0.009	0.002	0.011	0.001	0.010	0.095	2366.951	4.181	0.988	5.169	0.406	4.601	45.592	23670	41.810	9.880	51.690	4.060	46.010	455.920
150	original	1.2	60x8 weeks	Ag80	Daily	VAC50	25	All	4.931	0.009	0.002	0.011	0.001	0.010	0.095	2366.951	4.181	0.988	5.169	0.406	4.601	45.592	23670	41.810	9.880	51.690	4.060	46.010	455.920
150	original	1.2	60x8 weeks	Ag80	Daily	VAC50	25	All	4.931	0.009	0.002	0.011	0.001	0.010	0.095	2366.951	4.181	0.988	5.169	0.406	4.601	45.592	23670	41.810	9.880	51.690	4.060	46.010	455.920
150	alpha	0.6	60x8 weeks	Ag80	Daily	VAC50	25	All	4.925	0.009	0.002	0.011	0.000	0.011	0.096	2363.857	4.274	0.805	5.079	0.123	5.291	45.841	23639	42.740	8.050	50.790	1.230	52.910	458.410
150	alpha	0.6	60x8 weeks	Ag80	Daily	VAC50	25	All	4.925	0.009	0.002	0.011	0.000	0.011	0.096	2363.857	4.274	0.805	5.079	0.123	5.291	45.841	23639	42.740	8.050	50.790	1.230	52.910	458.410
150	alpha	0.6	60x8 weeks	Ag80	Daily	VAC50	25	All	4.925	0.009	0.002	0.011	0.000	0.011	0.096	2363.857	4.274	0.805	5.079	0.123	5.291	45.841	23639	42.740	8.050	50.790	1.230	52.910	458.410
150	alpha	1	60x8 weeks	Ag80	Daily	VAC50	25	All	4.916	0.009	0.003	0.012	0.000	0.012	0.096	2359.914	4.303	1.416	5.719	0.137	5.918	45.976	23599	43.030	14.160	57.190	1.370	59.180	459.760
150	alpha	1	60x8 weeks	Ag80	Daily	VAC50	25	All	4.916	0.009	0.003	0.012	0.000	0.012	0.096	2359.914	4.303	1.416	5.719	0.137	5.918	45.976	23599	43.030	14.160	57.190	1.370	59.180	459.760
150	alpha	1	60x8 weeks	Ag80	Daily	VAC50	25	All	4.916	0.009	0.003	0.012	0.000	0.012	0.096	2359.914	4.303	1.416	5.719	0.137	5.918	45.976	23599	43.030	14.160	57.190	1.370	59.180	459.760
150	alpha	1.2	60x8 weeks	Ag80	Daily	VAC50	25	All	4.908	0.009	0.004	0.013	0.000	0.013	0.095	2355.800	4.285	1.902	6.187	0.156	6.464	45.614	23558	42.850	19.020	61.870	1.560	64.640	456.140
150	alpha	1.2	60x8 weeks	Ag80	Daily	VAC50	25	All	4.908	0.009	0.004	0.013	0.000	0.013	0.095	2355.800	4.285	1.902	6.187	0.156	6.464	45.614	23558	42.850	19.020	61.870	1.560	64.640	456.140
150	alpha	1.2	60x8 weeks	Ag80	Daily	VAC50	25	All	4.908	0.009	0.004	0.013	0.000	0.013	0.095	2355.800	4.285	1.902	6.187	0.156	6.464	45.614	23558	42.850	19.020	61.870	1.560	64.640	456.140
150	delta	0.6	60x8 weeks	Ag80	Daily	VAC50	25	All	4.908	0.009	0.004	0.013	0.000	0.013	0.095	2355.735	4.321	1.919	6.240	0.193	6.452	45.705	23557	43.210	19.190	62.400	1.930	64.520	457.050
150	delta	0.6	60x8 weeks	Ag80	Daily	VAC50	25	All	4.908	0.009	0.004	0.013	0.000	0.013	0.095	2355.735	4.321	1.919	6.240	0.193	6.452	45.705	23557	43.210	19.190	62.400	1.930	64.520	457.050
150	delta	0.6	60x8 weeks	Ag80	Daily	VAC50	25	All	4.908	0.009	0.004	0.013	0.000	0.013	0.095	2355.735	4.321	1.919	6.240	0.193	6.452	45.705	23557	43.210	19.190	62.400	1.930	64.520	457.050
150	delta	1	60x8 weeks	Ag80	Daily	VAC50	25	All	4.878	0.009	0.008	0.017	0.000	0.018	0.093	2341.529	4.240	3.896	8.136	0.236	8.459	44.806	23415	42.400	38.960	81.360	2.360	84.590	448.060
150	delta	1	60x8 weeks	Ag80	Daily	VAC50	25	All	4.878	0.009	0.008	0.017	0.000	0.018	0.093	2341.529	4.240	3.896	8.136	0.236	8.459	44.806	23415	42.400	38.960	81.360	2.360	84.590	448.060
150	delta	1	60x8 weeks	Ag80	Daily	VAC50	25	All	4.878	0.009	0.008	0.017	0.000	0.018	0.093	2341.529	4.240	3.896	8.136	0.236	8.459	44.806	23415	42.400	38.960	81.360	2.360	84.590	448.060
150	delta	1.2	60x8 weeks	Ag80	Daily	VAC50	25	All	4.856	0.009	0.011	0.020	0.001	0.021	0.092	2330.891	4.258	5.279	9.537	0.269	9.950	44.114	23309	42.580	52.790	95.370	2.690	99.500	441.140
150	delta	1.2	60x8 weeks	Ag80	Daily	VAC50	25	All	4.856	0.009	0.011	0.020	0.001	0.021	0.092	2330.891	4.258	5.279	9.537	0.269	9.950	44.114	23309	42.580	52.790	95.370	2.690	99.500	441.140
150	delta	1.2	60x8 weeks	Ag80	Daily	VAC50	25	All	4.856	0.009	0.011	0.020	0.001	0.021	0.092	2330.891	4.258	5.279	9.537	0.269	9.950	44.114	23309	42.580	52.790	95.370	2.690	99.500	441.140
150	original	0.6	60x8 weeks	Ag80	Daily	VAC50	50	All	4.952	0.008	0.001	0.008	0.001	0.006	0.096	2377.159	3.724	0.277	4.001	0.595	2.871	46.294	23772	37.240	2.770	40.010	5.950	28.710	462.940
150	original	0.6	60x8 weeks	Ag80	Daily	VAC50	50	All	4.952	0.008	0.001	0.008	0.001	0.006	0.096	2377.159	3.724	0.277	4.001	0.595	2.871	46.294	23772	37.240	2.770	40.010	5.950	28.710	462.940
150	original	0.6	60x8 weeks	Ag80	Daily	VAC50	50	All	4.952	0.008	0.001	0.008	0.001	0.006	0.096	2377.159	3.724	0.277	4.001	0.595	2.871	46.294	23772	37.240	2.770	40.010	5.950	28.710	462.940
150	original	1	60x8 weeks	Ag80	Daily	VAC50	50	All	4.951	0.008	0.001	0.009	0.001	0.006	0.096	2376.387	3.702	0.442	4.144	0.613	2.967	46.183	23764	37.020	4.420	41.440	6.130	29.670	461.830
150	original	1	60x8 weeks	Ag80	Daily	VAC50	50	All	4.951	0.008	0.001	0.009	0.001	0.006	0.096	2376.387	3.702	0.442	4.144	0.613	2.967	46.183	23764	37.020	4.420	41.440	6.130	29.670	461.830
150	original	1	60x8 weeks	Ag80	Daily	VAC50	50	All	4.951	0.008	0.001	0.009	0.001	0.006	0.096	2376.387	3.702	0.442	4.144	0.613	2.967	46.183	23764	37.020	4.420	41.440	6.130	29.670	461.830
150	original	1.2	60x8 weeks	Ag80	Daily	VAC50	50	All	4.950	0.008	0.001	0.009	0.001	0.006	0.096	2376.123	3.647	0.508	4.155	0.631	2.984	46.119	23761	36.470	5.080	41.550	6.310	29.840	461.190
150	original	1.2	60x8 weeks	Ag80	Daily	VAC50	50	All	4.950	0.008	0.001	0.009	0.001	0.006	0.096	2376.123	3.647	0.508	4.155	0.631	2.984	46.119	23761	36.470	5.080	41.550	6.310	29.840	461.190
150	original	1.2	60x8 weeks	Ag80	Daily	VAC50	50	All	4.950	0.008	0.001	0.009	0.001	0.006	0.096	2376.123	3.647	0.508	4.155	0.631	2.984	46.119	23761	36.470	5.080	41.550	6.310	29.840	461.190
150	alpha	0.6	60x8 weeks	Ag80	Daily	VAC50	50	All	4.940	0.008	0.001	0.009	0.000	0.009	0.096	2371.186	3.634	0.480	4.114	0.116	4.225	46.305	23712	36.340	4.800	41.140	1.160	42.250	463.050

150	alpha	0.6	60x8 weeks	Ag80	Daily	VAC50	50	All	4.940	0.008	0.001	0.009	0.000	0.009	0.096	2371.186	3.634	0.480	4.114	0.116	4.225	46.305	23712	36.340	4.800	41.140	1.160	42.250	463.050
150	alpha	0.6	60x8 weeks	Ag80	Daily	VAC50	50	All	4.940	0.008	0.001	0.009	0.000	0.009	0.096	2371.186	3.634	0.480	4.114	0.116	4.225	46.305	23712	36.340	4.800	41.140	1.160	42.250	463.050
150	alpha	1	60x8 weeks	Ag80	Daily	VAC50	50	All	4.931	0.008	0.002	0.010	0.000	0.010	0.096	2366.884	3.778	0.980	4.758	0.139	4.864	46.174	23669	37.780	9.800	47.580	1.390	48.640	461.740
150	alpha	1	60x8 weeks	Ag80	Daily	VAC50	50	All	4.931	0.008	0.002	0.010	0.000	0.010	0.096	2366.884	3.778	0.980	4.758	0.139	4.864	46.174	23669	37.780	9.800	47.580	1.390	48.640	461.740
150	alpha	1	60x8 weeks	Ag80	Daily	VAC50	50	All	4.931	0.008	0.002	0.010	0.000	0.010	0.096	2366.884	3.778	0.980	4.758	0.139	4.864	46.174	23669	37.780	9.800	47.580	1.390	48.640	461.740
150	alpha	1.2	60x8 weeks	Ag80	Daily	VAC50	50	All	4.929	0.008	0.002	0.010	0.000	0.010	0.095	2366.125	3.663	1.150	4.813	0.116	4.980	45.748	23661	36.630	11.500	48.130	1.160	49.800	457.480
150	alpha	1.2	60x8 weeks	Ag80	Daily	VAC50	50	All	4.929	0.008	0.002	0.010	0.000	0.010	0.095	2366.125	3.663	1.150	4.813	0.116	4.980	45.748	23661	36.630	11.500	48.130	1.160	49.800	457.480
150	alpha	1.2	60x8 weeks	Ag80	Daily	VAC50	50	All	4.929	0.008	0.002	0.010	0.000	0.010	0.095	2366.125	3.663	1.150	4.813	0.116	4.980	45.748	23661	36.630	11.500	48.130	1.160	49.800	457.480
150	delta	0.6	60x8 weeks	Ag80	Daily	VAC50	50	All	4.918	0.009	0.003	0.012	0.000	0.012	0.096	2360.419	4.094	1.472	5.566	0.183	5.688	45.856	23604	40.940	14.720	55.660	1.830	56.880	458.560
150	delta	0.6	60x8 weeks	Ag80	Daily	VAC50	50	All	4.918	0.009	0.003	0.012	0.000	0.012	0.096	2360.419	4.094	1.472	5.566	0.183	5.688	45.856	23604	40.940	14.720	55.660	1.830	56.880	458.560
150	delta	0.6	60x8 weeks	Ag80	Daily	VAC50	50	All	4.918	0.009	0.003	0.012	0.000	0.012	0.096	2360.419	4.094	1.472	5.566	0.183	5.688	45.856	23604	40.940	14.720	55.660	1.830	56.880	458.560
150	delta	1	60x8 weeks	Ag80	Daily	VAC50	50	All	4.896	0.008	0.006	0.014	0.000	0.015	0.094	2350.175	3.972	2.925	6.897	0.201	7.195	45.085	23502	39.720	29.250	68.970	2.010	71.950	450.850
150	delta	1	60x8 weeks	Ag80	Daily	VAC50	50	All	4.896	0.008	0.006	0.014	0.000	0.015	0.094	2350.175	3.972	2.925	6.897	0.201	7.195	45.085	23502	39.720	29.250	68.970	2.010	71.950	450.850
150	delta	1	60x8 weeks	Ag80	Daily	VAC50	50	All	4.896	0.008	0.006	0.014	0.000	0.015	0.094	2350.175	3.972	2.925	6.897	0.201	7.195	45.085	23502	39.720	29.250	68.970	2.010	71.950	450.850
150	delta	1.2	60x8 weeks	Ag80	Daily	VAC50	50	All	4.890	0.008	0.007	0.016	0.000	0.016	0.093	2347.021	3.985	3.468	7.453	0.225	7.689	44.630	23470	39.850	34.680	74.530	2.250	76.890	446.300
150	delta	1.2	60x8 weeks	Ag80	Daily	VAC50	50	All	4.890	0.008	0.007	0.016	0.000	0.016	0.093	2347.021	3.985	3.468	7.453	0.225	7.689	44.630	23470	39.850	34.680	74.530	2.250	76.890	446.300
150	delta	1.2	60x8 weeks	Ag80	Daily	VAC50	50	All	4.890	0.008	0.007	0.016	0.000	0.016	0.093	2347.021	3.985	3.468	7.453	0.225	7.689	44.630	23470	39.850	34.680	74.530	2.250	76.890	446.300
150	original	0.6	60x8 weeks	Ag80	Daily	VAC50	75	All	4.967	0.007	0.000	0.007	0.002	0.003	0.097	2383.928	3.139	0.131	3.270	0.793	1.663	46.492	23839	31.390	1.310	32.700	7.930	16.630	464.920
150	original	0.6	60x8 weeks	Ag80	Daily	VAC50	75	All	4.967	0.007	0.000	0.007	0.002	0.003	0.097	2383.928	3.139	0.131	3.270	0.793	1.663	46.492	23839	31.390	1.310	32.700	7.930	16.630	464.920
150	original	0.6	60x8 weeks	Ag80	Daily	VAC50	75	All	4.967	0.007	0.000	0.007	0.002	0.003	0.097	2383.928	3.139	0.131	3.270	0.793	1.663	46.492	23839	31.390	1.310	32.700	7.930	16.630	464.920
150	original	1	60x8 weeks	Ag80	Daily	VAC50	75	All	4.966	0.007	0.000	0.007	0.002	0.003	0.097	2383.525	3.128	0.208	3.336	0.827	1.679	46.667	23835	31.280	2.080	33.360	8.270	16.790	466.670
150	original	1	60x8 weeks	Ag80	Daily	VAC50	75	All	4.966	0.007	0.000	0.007	0.002	0.003	0.097	2383.525	3.128	0.208	3.336	0.827	1.679	46.667	23835	31.280	2.080	33.360	8.270	16.790	466.670
150	original	1	60x8 weeks	Ag80	Daily	VAC50	75	All	4.966	0.007	0.000	0.007	0.002	0.003	0.097	2383.525	3.128	0.208	3.336	0.827	1.679	46.667	23835	31.280	2.080	33.360	8.270	16.790	466.670
150	original	1.2	60x8 weeks	Ag80	Daily	VAC50	75	All	4.964	0.006	0.001	0.007	0.002	0.004	0.097	2382.859	3.105	0.274	3.379	0.866	1.727	46.680	23829	31.050	2.740	33.790	8.660	17.270	466.800
150	original	1.2	60x8 weeks	Ag80	Daily	VAC50	75	All	4.964	0.006	0.001	0.007	0.002	0.004	0.097	2382.859	3.105	0.274	3.379	0.866	1.727	46.680	23829	31.050	2.740	33.790	8.660	17.270	466.800
150	original	1.2	60x8 weeks	Ag80	Daily	VAC50	75	All	4.964	0.006	0.001	0.007	0.002	0.004	0.097	2382.859	3.105	0.274	3.379	0.866	1.727	46.680	23829	31.050	2.740	33.790	8.660	17.270	466.800
150	alpha	0.6	60x8 weeks	Ag80	Daily	VAC50	75	All	4.951	0.006	0.001	0.007	0.000	0.007	0.097	2376.662	3.097	0.281	3.378	0.083	3.420	46.676	23767	30.970	2.810	33.780	0.830	34.200	466.760
150	alpha	0.6	60x8 weeks	Ag80	Daily	VAC50	75	All	4.951	0.006	0.001	0.007	0.000	0.007	0.097	2376.662	3.097	0.281	3.378	0.083	3.420	46.676	23767	30.970	2.810	33.780	0.830	34.200	466.760
150	alpha	0.6	60x8 weeks	Ag80	Daily	VAC50	75	All	4.951	0.006	0.001	0.007	0.000	0.007	0.097	2376.662	3.097	0.281	3.378	0.083	3.420	46.676	23767	30.970	2.810	33.780	0.830	34.200	466.760
150	alpha	1	60x8 weeks	Ag80	Daily	VAC50	75	All	4.947	0.006	0.001	0.008	0.000	0.008	0.097	2374.511	3.053	0.595	3.648	0.129	3.721	46.741	23745	30.530	5.950	36.480	1.290	37.210	467.410
150	alpha	1	60x8 weeks	Ag80	Daily	VAC50	75	All	4.947	0.006	0.001	0.008	0.000	0.008	0.097	2374.511	3.053	0.595	3.648	0.129	3.721	46.741	23745	30.530	5.950	36.480	1.290	37.210	467.410
150	alpha	1	60x8 weeks	Ag80	Daily	VAC50	75	All	4.947	0.006	0.001	0.008	0.000	0.008	0.097	2374.511	3.053	0.595	3.648	0.129	3.721	46.741	23745	30.530	5.950	36.480	1.290	37.210	467.410
150	alpha	1.2	60x8 weeks	Ag80	Daily	VAC50	75	All	4.941	0.007	0.002	0.008	0.000	0.009	0.097	2371.850	3.256	0.747	4.003	0.111	4.123	46.673	23719	32.560	7.470	40.030	1.110	41.230	466.730
150	alpha	1.2	60x8 weeks	Ag80	Daily	VAC50	75	All	4.941	0.007	0.002	0.008	0.000	0.009	0.097	2371.850	3.256	0.747	4.003	0.111	4.123	46.673	23719	32.560	7.470	40.030	1.110	41.230	466.730
150	alpha	1.2	60x8 weeks	Ag80	Daily	VAC50	75	All	4.941	0.007	0.002	0.008	0.000	0.009	0.097	2371.850	3.256	0.747	4.003	0.111	4.123	46.673	23719	32.560	7.470	40.030	1.110	41.230	466.730
150	delta	0.6	60x8 weeks	Ag80	Daily	VAC50	75	All	4.932	0.008	0.002	0.010	0.000	0.010	0.096	2367.149	3.686	0.960	4.646	0.164	4.744	46.297	23671	36.860	9.600	46.460	1.640	47.440	462.970
150	delta	0.6	60x8 weeks	Ag80	Daily	VAC50	75	All	4.932	0.008	0.002	0.010	0.000	0.010	0.096	2367.149	3.686	0.960	4.646	0.164	4.744	46.297	23671	36.860	9.600	46.460	1.640	47.440	462.970
150	delta	0.6	60x8 weeks	Ag80	Daily	VAC50	75	All	4.932	0.008	0.002	0.010	0.000	0.010	0.096	2367.149	3.686	0.960	4.646	0.164	4.744	46.297	23671	36.860	9.600	46.460	1.640	47.440	462.970
150	delta	1	60x8 weeks	Ag80	Daily	VAC50	75	All	4.921	0.008	0.003	0.011	0.000	0.011	0.096	2362.036	3.653	1.649	5.302	0.143	5.508	45.875	23620	36.530	16.490	53.020	1.430	55.080	458.750
150	delta	1	60x8 weeks	Ag80	Daily	VAC50	75	All	4.921	0.008	0.003	0.011	0.000	0.011	0.096	2362.036	3.653	1.649	5.302	0.143	5.508	45.875	23620	36.530	16.490	53.020	1.430	55.080	458.750
150	delta	1	60x8 weeks	Ag80	Daily	VAC50	75	All	4.921	0.008	0.003	0.011	0.000	0.011	0.096	2362.036	3.653	1.649	5.302	0.143	5.508	45.875	23620	36.530	16.490	53.020	1.430	55.080	458.750
150	delta	1.2	60x8 weeks	Ag80	Daily	VAC50	75	All	4.915	0.008	0.004	0.012	0.000	0.012	0.095	2359.293	3.607	2.125	5.732	0.179	5.935	45.718	23593	36.070	21.250	57.320	1.790	59.350	457.180
150	delta	1.2	60x8 weeks	Ag80	Daily	VAC50	75	All	4.915	0.008	0.004	0.012	0.000	0.012	0.095	2359.293	3.607	2.125	5.732	0.179	5.935	45.718	23593	36.070	21.250	57.320	1.790	59.350	457.180
150	delta	1.2	60x8 weeks	Ag80	Daily	VAC50	75	All	4.915	0.008	0.004	0.012	0.000	0.012	0.095	2359.293	3.607	2.125	5.732	0.179	5.935	45.718	23593	36.070	21.250	57.320	1.790	59.350	457.180
150	original	0.6	60x8 weeks	Ag80	Daily	VAC50	100	All	4.980	0.005	0.000	0.005	0.002	0.001	0.098	2390.402	2.533	0.026	2.559	0.982	0.498	46.929	23904	25.330	0.260	25.590	9.820	4.980	469.290
150	original	0.6	60x8 weeks	Ag80	Daily	VAC50	100	All	4.980	0.005	0.000	0.005	0.002	0.001	0.098	2390.402	2.533	0.026	2.559	0.982	0.498	46.929	23904	25.330	0.260	25.590	9.820	4.980	469.290
150	original	0.6	60x8 weeks	Ag80	Daily	VAC50	100	All	4.980	0.005	0.000	0.005	0.002	0.001	0.098	2390.402	2.533	0.026	2.559	0.982	0.498	46.929	23904	25.330	0.260	25.590	9.820	4.980	469.290
150	original	1	60x8 weeks	Ag80	Daily	VAC50	100	All	4.979	0.005	0.000	0.006	0.002	0.001	0.098	2389.813	2.599	0.062	2.661	1.011	0.563	46.823	23898	25.990	0.620	26.610	10.110	5.630	468.230
150	original	1	60x8 weeks	Ag80	Daily	VAC50	100	All	4.979	0.005	0.000	0.006	0.002	0.001	0.098	2389.813	2.599	0.062	2.661	1.011	0.563	46.823	23898	25.990	0.620	26.610	10.110	5.630	468.230
150	original	1	60x8 weeks	Ag80	Daily	VAC50	100	All	4.979	0.005	0.000	0.006	0.002	0.001	0.098	2389.813	2.599	0.062	2.661	1.011	0.563	46.823	23898	25.990	0.620	26.610	10.110	5.630	468.230
150	original	1.2	60x8 weeks	Ag80	Daily	VAC50	100	All	4.979	0.005	0.000	0.006	0.002	0.001	0.098	2389.898	2.631	0.058	2.689	1.045	0.507	47.102	23899	26.310	0.580	26.890	10.450	5.070	471.020
150	original	1.2	60x8 weeks	Ag80	Daily	VAC50	100	All	4.979	0.005	0.000	0.006	0.002	0.001	0.098	2389.898	2.631	0.058	2.689	1.045	0.507	47.102	23899	26.310	0.580	26.890	10.450	5.070	471.020
150	original	1.2	60x8 weeks	Ag80	Daily	VAC50	100	All	4.979	0.005	0.000	0.006	0.002	0.001	0.098	2389.898	2.631	0.058	2.689	1.045	0.507	47.102	23899	26.310	0.580	26.890	10.450	5.070	471.020
150	alpha	0.6	60x8 weeks	Ag80	Daily	VAC50	100	All	4.961	0.005	0.000	0.006	0.000	0.006	0.098	2381.331	2.578	0.175	2.753	0.110	2.722	47.054	23813	25.780	1.750	27.530	1.100	27.220	470.540
150	alpha	0.6	60x8 weeks	Ag80	Daily	VAC50	100	All	4.961	0.005	0.000	0.006	0.000	0.006	0.098	2381.331	2.578	0.175	2.753	0.110	2.722	47.054	23813	25.780	1.750	27.530	1.100	27.220	470.540
150	alpha	0.6	60x8 weeks	Ag80	Daily	VAC50	100	All	4.961	0.005	0.000	0.006	0.000	0.006	0.098	2381.331	2.578	0.175	2.753	0.110	2.722	47.054	23813	25.780	1.750	27.530	1.100	27.220	470.540
150	alpha	1	60x8 weeks	Ag80	Daily	VAC50	100	All	4.960	0.005	0.001	0.006	0.000	0.006	0.097	2380.833	2.513	0.299	2.812	0.119	2.803	46.668	23808	25.130	2.990	28.120	1.190	28.030	466.680
150	alpha	1	60x8 weeks	Ag80	Daily	VAC50	100	All	4.960	0.005	0.001	0.006	0.000	0.006	0.097	2380.833	2.513	0.299	2.812	0.119	2.803	46.668	23808	25.130	2.990	28.120	1.190	28.030	466.680
150	alpha	1	60x8 weeks	Ag80	Daily	VAC50	100	All	4.960	0.005	0.001	0.006	0.000	0.006	0.097	2380.833	2.513	0.299	2.812	0.119	2.803	46.668	23808	25.130	2.990	28.120	1.190	28.030	466.680
150	alpha	1.2	60x8 weeks	Ag80	Daily	VAC50	100	All	4.961	0.005	0.001	0.006	0.000	0.006	0.098	2381.123	2.466	0.339	2.805	0.093	2.787	46.841	23811	24.660	3.390	28.050	0.930	27.870	468.410
150	alpha	1.2	60x8 weeks	Ag80	Daily	VAC50	100	All	4.961	0.005	0.001	0.006	0.000	0.006	0.098	2381.123	2.466	0.339	2.805	0.093	2.787	46.841	23811	24.660	3.390	28.050	0.930	27.870	468.410
150	alpha	1.2	60x8 weeks	Ag80	Daily	VAC50	100	All	4.961	0.005	0.001	0.006	0.000	0.006	0.098	2381.123	2.466	0.339	2.805	0.093	2.787	46.841	23811	24.660	3.390	28.050	0.930	27.870	468.410
150	delta	0.6	60x8 weeks	Ag80	Daily	VAC50	100	All	4.944	0.007	0.001	0.008	0.000	0.008	0.097	2373.289	3.278	0.522	3.800	0.115	3.889	46.570	23733	32.780	5.220	38.000	1.150	38.890	465.700
150	delta	0.6	60x8 weeks	Ag80	Daily	VAC50	100	All	4.944	0.007	0.001	0.008	0.000	0.008	0.097	2373.289	3.278	0.522	3.800	0.115	3.889	46.570	23733	32.780	5.220	38.000	1.150	38.890	465.700
150	delta	0.6	60x8 weeks	Ag80	Daily	VAC50	100	All	4.944	0.007	0.001	0.008	0.000	0.008	0.097	2373.289	3.278	0.522	3.800	0.115	3.889	46.570	23733	32.780	5.220	38.000	1.150	38.890	465.700
150	delta	1	60x8 weeks	Ag80	Daily	VAC50	100	All	4.937	0.007	0.002	0.009	0.000	0.009	0.097	2369.960	3.319	1.006	4.325	0.158	4.360	46.382	23700	33.190	10.060	43.250	1.580	43.600	463.820
150	delta	1	60x8 weeks	Ag80	Daily	VAC50	100	All	4.937	0.007	0.002	0.009	0.000	0.009	0.097	2369.960	3.319	1.006	4.325	0.158	4.360	46.382	23700	33.190	10.060	43.250	1.580	43.600	463.820
150	delta	1	60x8 weeks	Ag80	Daily	VAC50	100	All	4.937	0.007	0.002	0.009	0.000	0.009	0.097	2369.960	3.319	1.006	4.325	0.158	4.360	46.382	23700	33.190	10.060	43.250	1.580	43.600	463.820
150	delta	1.2	60x8 weeks	Ag80	Daily	VAC50	100	All	4.933	0.007	0.003	0.009	0.000	0.010	0.096	2367.840	3.296	1.252	4.548	0.142	4.647	46.166	23678	32.960	12.520	45.480	1.420	46.470	461.660
150	delta	1.2	60x8 weeks	Ag80	Daily	VAC50	100	All	4.933	0.007	0.003	0.009	0.000	0.010	0.096	2367.840	3.296	1.252	4.548	0.142	4.647	46.166	23678	32.960	12.520	45.480	1.420	46.470	461.660
150	delta	1.2	60x8 weeks	Ag80	Daily	VAC50	100	All	4.933	0.007	0.003	0.009	0.000	0.010	0.096	2367.840	3.296	1.252	4.548	0.142	4.647	46.166	23678	32.960	12.520	45.480	1.420	46.470	461.660

150	original	0.6	60x8 weeks	Ag80	MWF	NOVAC	0	All	2.956	0.010	0.002	0.012	0.000	0.012	0.057	1419.005	4.811	0.984	5.795	0.221	5.585	27.492	14190	48.110	9.840	57.950	2.210	55.850	274.920
150	original	0.6	60x8 weeks	Ag80	MWF	NOVAC	0	All	2.956	0.010	0.002	0.012	0.000	0.012	0.057	1419.005	4.811	0.984	5.795	0.221	5.585	27.492	14190	48.110	9.840	57.950	2.210	55.850	274.920
150	original	0.6	60x8 weeks	Ag80	MWF	NOVAC	0	All	2.956	0.010	0.002	0.012	0.000	0.012	0.057	1419.005	4.811	0.984	5.795	0.221	5.585	27.492	14190	48.110	9.840	57.950	2.210	55.850	274.920
150	original	1	60x8 weeks	Ag80	MWF	NOVAC	0	All	2.950	0.010	0.004	0.014	0.001	0.013	0.057	1415.908	4.766	1.896	6.662	0.250	6.391	27.235	14159	47.660	18.960	66.620	2.500	63.910	272.350
150	original	1	60x8 weeks	Ag80	MWF	NOVAC	0	All	2.950	0.010	0.004	0.014	0.001	0.013	0.057	1415.908	4.766	1.896	6.662	0.250	6.391	27.235	14159	47.660	18.960	66.620	2.500	63.910	272.350
150	original	1	60x8 weeks	Ag80	MWF	NOVAC	0	All	2.950	0.010	0.004	0.014	0.001	0.013	0.057	1415.908	4.766	1.896	6.662	0.250	6.391	27.235	14159	47.660	18.960	66.620	2.500	63.910	272.350
150	original	1.2	60x8 weeks	Ag80	MWF	NOVAC	0	All	2.945	0.010	0.005	0.015	0.001	0.014	0.057	1413.419	4.669	2.569	7.238	0.379	6.953	27.395	14134	46.690	25.690	72.380	3.790	69.530	273.950
150	original	1.2	60x8 weeks	Ag80	MWF	NOVAC	0	All	2.945	0.010	0.005	0.015	0.001	0.014	0.057	1413.419	4.669	2.569	7.238	0.379	6.953	27.395	14134	46.690	25.690	72.380	3.790	69.530	273.950
150	original	1.2	60x8 weeks	Ag80	MWF	NOVAC	0	All	2.945	0.010	0.005	0.015	0.001	0.014	0.057	1413.419	4.669	2.569	7.238	0.379	6.953	27.395	14134	46.690	25.690	72.380	3.790	69.530	273.950
150	alpha	0.6	60x8 weeks	Ag80	MWF	NOVAC	0	All	2.948	0.010	0.004	0.014	0.001	0.014	0.057	1414.880	4.650	1.885	6.535	0.266	6.655	27.375	14149	46.500	18.850	65.350	2.660	66.550	273.750
150	alpha	0.6	60x8 weeks	Ag80	MWF	NOVAC	0	All	2.948	0.010	0.004	0.014	0.001	0.014	0.057	1414.880	4.650	1.885	6.535	0.266	6.655	27.375	14149	46.500	18.850	65.350	2.660	66.550	273.750
150	alpha	0.6	60x8 weeks	Ag80	MWF	NOVAC	0	All	2.948	0.010	0.004	0.014	0.001	0.014	0.057	1414.880	4.650	1.885	6.535	0.266	6.655	27.375	14149	46.500	18.850	65.350	2.660	66.550	273.750
150	alpha	1	60x8 weeks	Ag80	MWF	NOVAC	0	All	2.928	0.010	0.009	0.019	0.001	0.019	0.055	1405.499	4.689	4.391	9.080	0.382	9.187	26.563	14055	46.890	43.910	90.800	3.820	91.870	265.630
150	alpha	1	60x8 weeks	Ag80	MWF	NOVAC	0	All	2.928	0.010	0.009	0.019	0.001	0.019	0.055	1405.499	4.689	4.391	9.080	0.382	9.187	26.563	14055	46.890	43.910	90.800	3.820	91.870	265.630
150	alpha	1	60x8 weeks	Ag80	MWF	NOVAC	0	All	2.928	0.010	0.009	0.019	0.001	0.019	0.055	1405.499	4.689	4.391	9.080	0.382	9.187	26.563	14055	46.890	43.910	90.800	3.820	91.870	265.630
150	alpha	1.2	60x8 weeks	Ag80	MWF	NOVAC	0	All	2.914	0.010	0.013	0.023	0.001	0.023	0.055	1398.757	4.571	6.233	10.804	0.479	10.916	26.462	13988	45.710	62.330	108.040	4.790	109.160	264.620
150	alpha	1.2	60x8 weeks	Ag80	MWF	NOVAC	0	All	2.914	0.010	0.013	0.023	0.001	0.023	0.055	1398.757	4.571	6.233	10.804	0.479	10.916	26.462	13988	45.710	62.330	108.040	4.790	109.160	264.620
150	alpha	1.2	60x8 weeks	Ag80	MWF	NOVAC	0	All	2.914	0.010	0.013	0.023	0.001	0.023	0.055	1398.757	4.571	6.233	10.804	0.479	10.916	26.462	13988	45.710	62.330	108.040	4.790	109.160	264.620
150	delta	0.6	60x8 weeks	Ag80	MWF	NOVAC	0	All	2.912	0.009	0.014	0.023	0.001	0.023	0.054	1397.896	4.361	6.578	10.939	0.708	10.837	26.058	13979	43.610	65.780	109.390	7.080	108.370	260.580
150	delta	0.6	60x8 weeks	Ag80	MWF	NOVAC	0	All	2.912	0.009	0.014	0.023	0.001	0.023	0.054	1397.896	4.361	6.578	10.939	0.708	10.837	26.058	13979	43.610	65.780	109.390	7.080	108.370	260.580
150	delta	0.6	60x8 weeks	Ag80	MWF	NOVAC	0	All	2.912	0.009	0.014	0.023	0.001	0.023	0.054	1397.896	4.361	6.578	10.939	0.708	10.837	26.058	13979	43.610	65.780	109.390	7.080	108.370	260.580
150	delta	1	60x8 weeks	Ag80	MWF	NOVAC	0	All	2.770	0.008	0.050	0.058	0.004	0.058	0.046	1329.525	3.875	23.769	27.644	1.932	27.724	21.910	13295	38.750	237.690	276.440	19.320	277.240	219.100
150	delta	1	60x8 weeks	Ag80	MWF	NOVAC	0	All	2.770	0.008	0.050	0.058	0.004	0.058	0.046	1329.525	3.875	23.769	27.644	1.932	27.724	21.910	13295	38.750	237.690	276.440	19.320	277.240	219.100
150	delta	1	60x8 weeks	Ag80	MWF	NOVAC	0	All	2.770	0.008	0.050	0.058	0.004	0.058	0.046	1329.525	3.875	23.769	27.644	1.932	27.724	21.910	13295	38.750	237.690	276.440	19.320	277.240	219.100
150	delta	1.2	60x8 weeks	Ag80	MWF	NOVAC	0	All	2.687	0.007	0.070	0.077	0.006	0.078	0.040	1289.656	3.387	33.760	37.147	2.658	37.241	19.300	12897	33.870	337.600	371.470	26.580	372.410	193.000
150	delta	1.2	60x8 weeks	Ag80	MWF	NOVAC	0	All	2.687	0.007	0.070	0.077	0.006	0.078	0.040	1289.656	3.387	33.760	37.147	2.658	37.241	19.300	12897	33.870	337.600	371.470	26.580	372.410	193.000
150	delta	1.2	60x8 weeks	Ag80	MWF	NOVAC	0	All	2.687	0.007	0.070	0.077	0.006	0.078	0.040	1289.656	3.387	33.760	37.147	2.658	37.241	19.300	12897	33.870	337.600	371.470	26.580	372.410	193.000
150	original	0.6	60x8 weeks	Ag80	MWF	VAC95	25	All	2.969	0.008	0.001	0.009	0.000	0.008	0.058	1425.317	3.658	0.523	4.181	0.191	3.880	27.673	14253	36.580	5.230	41.810	1.910	38.800	276.730
150	original	0.6	60x8 weeks	Ag80	MWF	VAC95	25	All	2.969	0.008	0.001	0.009	0.000	0.008	0.058	1425.317	3.658	0.523	4.181	0.191	3.880	27.673	14253	36.580	5.230	41.810	1.910	38.800	276.730
150	original	0.6	60x8 weeks	Ag80	MWF	VAC95	25	All	2.969	0.008	0.001	0.009	0.000	0.008	0.058	1425.317	3.658	0.523	4.181	0.191	3.880	27.673	14253	36.580	5.230	41.810	1.910	38.800	276.730
150	original	1	60x8 weeks	Ag80	MWF	VAC95	25	All	2.964	0.008	0.002	0.010	0.000	0.009	0.058	1422.956	3.791	0.980	4.771	0.213	4.536	27.692	14230	37.910	9.800	47.710	2.130	45.360	276.920
150	original	1	60x8 weeks	Ag80	MWF	VAC95	25	All	2.964	0.008	0.002	0.010	0.000	0.009	0.058	1422.956	3.791	0.980	4.771	0.213	4.536	27.692	14230	37.910	9.800	47.710	2.130	45.360	276.920
150	original	1	60x8 weeks	Ag80	MWF	VAC95	25	All	2.964	0.008	0.002	0.010	0.000	0.009	0.058	1422.956	3.791	0.980	4.771	0.213	4.536	27.692	14230	37.910	9.800	47.710	2.130	45.360	276.920
150	original	1.2	60x8 weeks	Ag80	MWF	VAC95	25	All	2.965	0.007	0.002	0.010	0.000	0.009	0.058	1423.191	3.555	1.192	4.747	0.209	4.432	27.797	14232	35.550	11.920	47.470	2.090	44.320	277.970
150	original	1.2	60x8 weeks	Ag80	MWF	VAC95	25	All	2.965	0.007	0.002	0.010	0.000	0.009	0.058	1423.191	3.555	1.192	4.747	0.209	4.432	27.797	14232	35.550	11.920	47.470	2.090	44.320	277.970
150	original	1.2	60x8 weeks	Ag80	MWF	VAC95	25	All	2.965	0.007	0.002	0.010	0.000	0.009	0.058	1423.191	3.555	1.192	4.747	0.209	4.432	27.797	14232	35.550	11.920	47.470	2.090	44.320	277.970
150	alpha	0.6	60x8 weeks	Ag80	MWF	VAC95	25	All	2.963	0.008	0.002	0.010	0.000	0.010	0.058	1422.214	3.726	1.030	4.756	0.197	4.740	27.854	14222	37.260	10.300	47.560	1.970	47.400	278.540
150	alpha	0.6	60x8 weeks	Ag80	MWF	VAC95	25	All	2.963	0.008	0.002	0.010	0.000	0.010	0.058	1422.214	3.726	1.030	4.756	0.197	4.740	27.854	14222	37.260	10.300	47.560	1.970	47.400	278.540
150	alpha	0.6	60x8 weeks	Ag80	MWF	VAC95	25	All	2.963	0.008	0.002	0.010	0.000	0.010	0.058	1422.214	3.726	1.030	4.756	0.197	4.740	27.854	14222	37.260	10.300	47.560	1.970	47.400	278.540
150	alpha	1	60x8 weeks	Ag80	MWF	VAC95	25	All	2.954	0.008	0.005	0.012	0.001	0.012	0.058	1417.755	3.694	2.217	5.911	0.272	5.897	27.615	14178	36.940	22.170	59.110	2.720	58.970	276.150
150	alpha	1	60x8 weeks	Ag80	MWF	VAC95	25	All	2.954	0.008	0.005	0.012	0.001	0.012	0.058	1417.755	3.694	2.217	5.911	0.272	5.897	27.615	14178	36.940	22.170	59.110	2.720	58.970	276.150
150	alpha	1	60x8 weeks	Ag80	MWF	VAC95	25	All	2.954	0.008	0.005	0.012	0.001	0.012	0.058	1417.755	3.694	2.217	5.911	0.272	5.897	27.615	14178	36.940	22.170	59.110	2.720	58.970	276.150
150	alpha	1.2	60x8 weeks	Ag80	MWF	VAC95	25	All	2.949	0.008	0.006	0.014	0.001	0.014	0.057	1415.576	3.713	2.849	6.562	0.290	6.524	27.231	14156	37.130	28.490	65.620	2.900	65.240	272.310
150	alpha	1.2	60x8 weeks	Ag80	MWF	VAC95	25	All	2.949	0.008	0.006	0.014	0.001	0.014	0.057	1415.576	3.713	2.849	6.562	0.290	6.524	27.231	14156	37.130	28.490	65.620	2.900	65.240	272.310
150	alpha	1.2	60x8 weeks	Ag80	MWF	VAC95	25	All	2.949	0.008	0.006	0.014	0.001	0.014	0.057	1415.576	3.713	2.849	6.562	0.290	6.524	27.231	14156	37.130	28.490	65.620	2.900	65.240	272.310
150	delta	0.6	60x8 weeks	Ag80	MWF	VAC95	25	All	2.946	0.008	0.007	0.014	0.001	0.014	0.057	1413.913	3.675	3.230	6.905	0.447	6.756	27.237	14139	36.750	32.300	69.050	4.470	67.560	272.370
150	delta	0.6	60x8 weeks	Ag80	MWF	VAC95	25	All	2.946	0.008	0.007	0.014	0.001	0.014	0.057	1413.913	3.675	3.230	6.905	0.447	6.756	27.237	14139	36.750	32.300	69.050	4.470	67.560	272.370
150	delta	0.6	60x8 weeks	Ag80	MWF	VAC95	25	All	2.946	0.008	0.007	0.014	0.001	0.014	0.057	1413.913	3.675	3.230	6.905	0.447	6.756	27.237	14139	36.750	32.300	69.050	4.470	67.560	272.370
150	delta	1	60x8 weeks	Ag80	MWF	VAC95	25	All	2.900	0.007	0.019	0.026	0.002	0.026	0.054	1391.932	3.452	9.017	12.469	0.835	12.300	25.803	13919	34.520	90.170	124.690	8.350	123.000	258.030
150	delta	1	60x8 weeks	Ag80	MWF	VAC95	25	All	2.900	0.007	0.019	0.026	0.002	0.026	0.054	1391.932	3.452	9.017	12.469	0.835	12.300	25.803	13919	34.520	90.170	124.690	8.350	123.000	258.030
150	delta	1	60x8 weeks	Ag80	MWF	VAC95	25	All	2.900	0.007	0.019	0.026	0.002	0.026	0.054	1391.932	3.452	9.017	12.469	0.835	12.300	25.803	13919	34.520	90.170	124.690	8.350	123.000	258.030
150	delta	1.2	60x8 weeks	Ag80	MWF	VAC95	25	All	2.864	0.007	0.029	0.035	0.002	0.035	0.052	1374.520	3.208	13.729	16.937	1.147	16.828	25.156	13745	32.080	137.290	169.370	11.470	168.280	251.560
150	delta	1.2	60x8 weeks	Ag80	MWF	VAC95	25	All	2.864	0.007	0.029	0.035	0.002	0.035	0.052	1374.520	3.208	13.729	16.937	1.147	16.828	25.156	13745	32.080	137.290	169.370	11.470	168.280	251.560
150	delta	1.2	60x8 weeks	Ag80	MWF	VAC95	25	All	2.864	0.007	0.029	0.035	0.002	0.035	0.052	1374.520	3.208	13.729	16.937	1.147	16.828	25.156	13745	32.080	137.290	169.370	11.470	168.280	251.560
150	original	0.6	60x8 weeks	Ag80	MWF	VAC95	50	All	2.980	0.005	0.000	0.006	0.000	0.005	0.058	1430.289	2.636	0.210	2.846	0.167	2.527	27.907	14303	26.360	2.100	28.460	1.670	25.270	279.070
150	original	0.6	60x8 weeks	Ag80	MWF	VAC95	50	All	2.980	0.005	0.000	0.006	0.000	0.005	0.058	1430.289	2.636	0.210	2.846	0.167	2.527	27.907	14303	26.360	2.100	28.460	1.670	25.270	279.070
150	original	0.6	60x8 weeks	Ag80	MWF	VAC95	50	All	2.980	0.005	0.000	0.006	0.000	0.005	0.058	1430.289	2.636	0.210	2.846	0.167	2.527	27.907	14303	26.360	2.100	28.460	1.670	25.270	279.070
150	original	1	60x8 weeks	Ag80	MWF	VAC95	50	All	2.979	0.005	0.001	0.006	0.000	0.006	0.059	1429.691	2.580	0.382	2.962	0.182	2.648	28.350	14297	25.800	3.820	29.620	1.820	26.480	283.500
150	original	1	60x8 weeks	Ag80	MWF	VAC95	50	All	2.979	0.005	0.001	0.006	0.000	0.006	0.059	1429.691	2.580	0.382	2.962	0.182	2.648	28.350	14297	25.800	3.820	29.620	1.820	26.480	283.500
150	original	1	60x8 weeks	Ag80	MWF	VAC95	50	All	2.979	0.005	0.001	0.006	0.000	0.006	0.059	1429.691	2.580	0.382	2.962	0.182	2.648	28.350	14297	25.800	3.820	29.620	1.820	26.480	283.500
150	original	1.2	60x8 weeks	Ag80	MWF	VAC95	50	All	2.978	0.005	0.001	0.006	0.000	0.006	0.058	1429.482	2.547	0.502	3.049	0.183	2.754	27.747	14295	25.470	5.020	30.490	1.830	27.540	277.470
150	original	1.2	60x8 weeks	Ag80	MWF	VAC95	50	All	2.978	0.005	0.001	0.006	0.000	0.006	0.058	1429.482	2.547	0.502	3.049	0.183	2.754	27.747	14295	25.470	5.020	30.490	1.830	27.540	277.470
150	original	1.2	60x8 weeks	Ag80	MWF	VAC95	50	All	2.978	0.005	0.001	0.006	0.000	0.006	0.058	1429.482	2.547	0.502	3.049	0.183	2.754	27.747	14295	25.470	5.020	30.490	1.830	27.540	277.470
150	alpha	0.6	60x8 weeks	Ag80	MWF	VAC95	50	All	2.977	0.005	0.001	0.006	0.000	0.006	0.059	1428.837	2.588	0.406	2.994	0.119	2.982	28.099	14288	25.880	4.060	29.940	1.190	29.820	280.990
150	alpha	0.6	60x8 weeks	Ag80	MWF	VAC95	50	All	2.977	0.005	0.001	0.006	0.000	0.006	0.059	1428.837	2.588	0.406	2.994	0.119	2.982	28.099	14288	25.880	4.060	29.940	1.190	29.820	280.990
150	alpha	0.6	60x8 weeks	Ag80	MWF	VAC95	50	All	2.977	0.005	0.001	0.006	0.000	0.006	0.059	1428.837	2.588	0.406	2.994	0.119	2.982	28.099	14288	25.880	4.060	29.940	1.190	29.820	280.990
150	alpha	1	60x8 weeks	Ag80	MWF	VAC95	50	All	2.973	0.005	0.002	0.007	0.000	0.007	0.059	1427.277	2.553	0.914	3.467	0.148	3.440	28.275	14273	25.530	9.140	34.670	1.480	34.400	282.750
150	alpha	1	60x8 weeks	Ag80	MWF	VAC95	50	All	2.973	0.005	0.002	0.007	0.000	0.007	0.059	1427.277	2.553	0.914	3.467	0.148	3.440	28.275	14273	25.530	9.140	34.670	1.480	34.400	282.750
150	alpha	1	60x8 weeks	Ag80	MWF	VAC95	50	All	2.973	0.005	0.002	0.007	0.000	0.007	0.059	1427.277	2.553	0.914	3.467	0.148	3.440	28.275	14273	25.530	9.140	34.670	1.480	34.400	282.750
150	alpha	1.2	60x8 weeks	Ag80	MWF	VAC95	50	All	2.972	0.005	0.002	0.008	0.000	0.008	0.058	1426.454	2.617	1.069	3.686	0.162	3.627	28.052	14265	26.170	10.690	36.860	1.620	36.270	280.520
150	alpha	1.2	60x8 weeks	Ag80	MWF	VAC95	50	All	2.972	0.005	0.002	0.008	0.000	0.008	0.058	1426.454	2.617	1.069	3.686	0.162	3.627	28.052	14265	26.170	10.690	36.860	1.620	36.270	280.520

150	alpha	1.2	60x8 weeks	Ag80	MWF	VAC95	50	All	2.972	0.005	0.002	0.008	0.000	0.008	0.058	1426.454	2.617	1.069	3.686	0.162	3.627	28.052	14265	26.170	10.690	36.860	1.620	36.270	280.520
150	delta	0.6	60x8 weeks	Ag80	MWF	VAC95	50	All	2.968	0.006	0.003	0.008	0.000	0.008	0.058	1424.853	2.724	1.252	3.976	0.219	3.976	27.827	14249	27.240	12.520	39.760	2.190	39.760	278.270
150	delta	0.6	60x8 weeks	Ag80	MWF	VAC95	50	All	2.968	0.006	0.003	0.008	0.000	0.008	0.058	1424.853	2.724	1.252	3.976	0.219	3.976	27.827	14249	27.240	12.520	39.760	2.190	39.760	278.270
150	delta	0.6	60x8 weeks	Ag80	MWF	VAC95	50	All	2.968	0.006	0.003	0.008	0.000	0.008	0.058	1424.853	2.724	1.252	3.976	0.219	3.976	27.827	14249	27.240	12.520	39.760	2.190	39.760	278.270
150	delta	1	60x8 weeks	Ag80	MWF	VAC95	50	All	2.957	0.005	0.006	0.011	0.001	0.011	0.058	1419.191	2.635	2.845	5.480	0.330	5.417	27.723	14192	26.350	28.450	54.800	3.300	54.170	277.230
150	delta	1	60x8 weeks	Ag80	MWF	VAC95	50	All	2.957	0.005	0.006	0.011	0.001	0.011	0.058	1419.191	2.635	2.845	5.480	0.330	5.417	27.723	14192	26.350	28.450	54.800	3.300	54.170	277.230
150	delta	1	60x8 weeks	Ag80	MWF	VAC95	50	All	2.957	0.005	0.006	0.011	0.001	0.011	0.058	1419.191	2.635	2.845	5.480	0.330	5.417	27.723	14192	26.350	28.450	54.800	3.300	54.170	277.230
150	delta	1.2	60x8 weeks	Ag80	MWF	VAC95	50	All	2.943	0.006	0.009	0.015	0.001	0.015	0.056	1412.705	2.676	4.465	7.141	0.447	7.067	26.889	14127	26.760	44.650	71.410	4.470	70.670	268.890
150	delta	1.2	60x8 weeks	Ag80	MWF	VAC95	50	All	2.943	0.006	0.009	0.015	0.001	0.015	0.056	1412.705	2.676	4.465	7.141	0.447	7.067	26.889	14127	26.760	44.650	71.410	4.470	70.670	268.890
150	delta	1.2	60x8 weeks	Ag80	MWF	VAC95	50	All	2.943	0.006	0.009	0.015	0.001	0.015	0.056	1412.705	2.676	4.465	7.141	0.447	7.067	26.889	14127	26.760	44.650	71.410	4.470	70.670	268.890
150	original	0.6	60x8 weeks	Ag80	MWF	VAC95	75	All	2.990	0.003	0.000	0.003	0.000	0.003	0.060	1435.129	1.439	0.056	1.495	0.130	1.213	28.609	14351	14.390	0.560	14.950	1.300	12.130	286.090
150	original	0.6	60x8 weeks	Ag80	MWF	VAC95	75	All	2.990	0.003	0.000	0.003	0.000	0.003	0.060	1435.129	1.439	0.056	1.495	0.130	1.213	28.609	14351	14.390	0.560	14.950	1.300	12.130	286.090
150	original	0.6	60x8 weeks	Ag80	MWF	VAC95	75	All	2.990	0.003	0.000	0.003	0.000	0.003	0.060	1435.129	1.439	0.056	1.495	0.130	1.213	28.609	14351	14.390	0.560	14.950	1.300	12.130	286.090
150	original	1	60x8 weeks	Ag80	MWF	VAC95	75	All	2.989	0.003	0.000	0.003	0.000	0.003	0.060	1434.763	1.492	0.110	1.602	0.173	1.273	28.580	14348	14.920	1.100	16.020	1.730	12.730	285.800
150	original	1	60x8 weeks	Ag80	MWF	VAC95	75	All	2.989	0.003	0.000	0.003	0.000	0.003	0.060	1434.763	1.492	0.110	1.602	0.173	1.273	28.580	14348	14.920	1.100	16.020	1.730	12.730	285.800
150	original	1	60x8 weeks	Ag80	MWF	VAC95	75	All	2.989	0.003	0.000	0.003	0.000	0.003	0.060	1434.763	1.492	0.110	1.602	0.173	1.273	28.580	14348	14.920	1.100	16.020	1.730	12.730	285.800
150	original	1.2	60x8 weeks	Ag80	MWF	VAC95	75	All	2.989	0.003	0.000	0.004	0.000	0.003	0.059	1434.601	1.537	0.147	1.684	0.140	1.321	28.325	14346	15.370	1.470	16.840	1.400	13.210	283.250
150	original	1.2	60x8 weeks	Ag80	MWF	VAC95	75	All	2.989	0.003	0.000	0.004	0.000	0.003	0.059	1434.601	1.537	0.147	1.684	0.140	1.321	28.325	14346	15.370	1.470	16.840	1.400	13.210	283.250
150	original	1.2	60x8 weeks	Ag80	MWF	VAC95	75	All	2.989	0.003	0.000	0.004	0.000	0.003	0.059	1434.601	1.537	0.147	1.684	0.140	1.321	28.325	14346	15.370	1.470	16.840	1.400	13.210	283.250
150	alpha	0.6	60x8 weeks	Ag80	MWF	VAC95	75	All	2.987	0.003	0.000	0.003	0.000	0.004	0.059	1433.684	1.546	0.130	1.676	0.064	1.684	28.436	14337	15.460	1.300	16.760	0.640	16.840	284.360
150	alpha	0.6	60x8 weeks	Ag80	MWF	VAC95	75	All	2.987	0.003	0.000	0.003	0.000	0.004	0.059	1433.684	1.546	0.130	1.676	0.064	1.684	28.436	14337	15.460	1.300	16.760	0.640	16.840	284.360
150	alpha	0.6	60x8 weeks	Ag80	MWF	VAC95	75	All	2.987	0.003	0.000	0.003	0.000	0.004	0.059	1433.684	1.546	0.130	1.676	0.064	1.684	28.436	14337	15.460	1.300	16.760	0.640	16.840	284.360
150	alpha	1	60x8 weeks	Ag80	MWF	VAC95	75	All	2.986	0.003	0.000	0.004	0.000	0.004	0.059	1433.458	1.548	0.231	1.779	0.070	1.756	28.453	14335	15.480	2.310	17.790	0.700	17.560	284.530
150	alpha	1	60x8 weeks	Ag80	MWF	VAC95	75	All	2.986	0.003	0.000	0.004	0.000	0.004	0.059	1433.458	1.548	0.231	1.779	0.070	1.756	28.453	14335	15.480	2.310	17.790	0.700	17.560	284.530
150	alpha	1	60x8 weeks	Ag80	MWF	VAC95	75	All	2.986	0.003	0.000	0.004	0.000	0.004	0.059	1433.458	1.548	0.231	1.779	0.070	1.756	28.453	14335	15.480	2.310	17.790	0.700	17.560	284.530
150	alpha	1.2	60x8 weeks	Ag80	MWF	VAC95	75	All	2.986	0.003	0.001	0.004	0.000	0.004	0.059	1433.329	1.484	0.307	1.791	0.073	1.767	28.121	14333	14.840	3.070	17.910	0.730	17.670	281.210
150	alpha	1.2	60x8 weeks	Ag80	MWF	VAC95	75	All	2.986	0.003	0.001	0.004	0.000	0.004	0.059	1433.329	1.484	0.307	1.791	0.073	1.767	28.121	14333	14.840	3.070	17.910	0.730	17.670	281.210
150	alpha	1.2	60x8 weeks	Ag80	MWF	VAC95	75	All	2.986	0.003	0.001	0.004	0.000	0.004	0.059	1433.329	1.484	0.307	1.791	0.073	1.767	28.121	14333	14.840	3.070	17.910	0.730	17.670	281.210
150	delta	0.6	60x8 weeks	Ag80	MWF	VAC95	75	All	2.984	0.004	0.001	0.004	0.000	0.004	0.059	1432.252	1.731	0.347	2.078	0.121	2.016	28.468	14323	17.310	3.470	20.780	1.210	20.160	284.680
150	delta	0.6	60x8 weeks	Ag80	MWF	VAC95	75	All	2.984	0.004	0.001	0.004	0.000	0.004	0.059	1432.252	1.731	0.347	2.078	0.121	2.016	28.468	14323	17.310	3.470	20.780	1.210	20.160	284.680
150	delta	0.6	60x8 weeks	Ag80	MWF	VAC95	75	All	2.984	0.004	0.001	0.004	0.000	0.004	0.059	1432.252	1.731	0.347	2.078	0.121	2.016	28.468	14323	17.310	3.470	20.780	1.210	20.160	284.680
150	delta	1	60x8 weeks	Ag80	MWF	VAC95	75	All	2.980	0.004	0.002	0.005	0.000	0.005	0.059	1430.554	1.755	0.766	2.521	0.143	2.476	28.479	14306	17.550	7.660	25.210	1.430	24.760	284.790
150	delta	1	60x8 weeks	Ag80	MWF	VAC95	75	All	2.980	0.004	0.002	0.005	0.000	0.005	0.059	1430.554	1.755	0.766	2.521	0.143	2.476	28.479	14306	17.550	7.660	25.210	1.430	24.760	284.790
150	delta	1	60x8 weeks	Ag80	MWF	VAC95	75	All	2.980	0.004	0.002	0.005	0.000	0.005	0.059	1430.554	1.755	0.766	2.521	0.143	2.476	28.479	14306	17.550	7.660	25.210	1.430	24.760	284.790
150	delta	1.2	60x8 weeks	Ag80	MWF	VAC95	75	All	2.979	0.004	0.002	0.006	0.000	0.006	0.058	1429.847	1.712	0.979	2.691	0.155	2.657	27.938	14298	17.120	9.790	26.910	1.550	26.570	279.380
150	delta	1.2	60x8 weeks	Ag80	MWF	VAC95	75	All	2.979	0.004	0.002	0.006	0.000	0.006	0.058	1429.847	1.712	0.979	2.691	0.155	2.657	27.938	14298	17.120	9.790	26.910	1.550	26.570	279.380
150	delta	1.2	60x8 weeks	Ag80	MWF	VAC95	75	All	2.979	0.004	0.002	0.006	0.000	0.006	0.058	1429.847	1.712	0.979	2.691	0.155	2.657	27.938	14298	17.120	9.790	26.910	1.550	26.570	279.380
150	original	0.6	60x8 weeks	Ag80	MWF	VAC95	100	All	2.999	0.001	0.000	0.001	0.000	0.000	0.059	1439.314	0.383	0.000	0.383	0.133	0.042	28.509	14393	3.830	0.000	3.830	1.330	0.420	285.090
150	original	0.6	60x8 weeks	Ag80	MWF	VAC95	100	All	2.999	0.001	0.000	0.001	0.000	0.000	0.059	1439.314	0.383	0.000	0.383	0.133	0.042	28.509	14393	3.830	0.000	3.830	1.330	0.420	285.090
150	original	0.6	60x8 weeks	Ag80	MWF	VAC95	100	All	2.999	0.001	0.000	0.001	0.000	0.000	0.059	1439.314	0.383	0.000	0.383	0.133	0.042	28.509	14393	3.830	0.000	3.830	1.330	0.420	285.090
150	original	1	60x8 weeks	Ag80	MWF	VAC95	100	All	2.999	0.001	0.000	0.001	0.000	0.000	0.059	1439.414	0.360	0.000	0.360	0.105	0.046	28.534	14394	3.600	0.000	3.600	1.050	0.460	285.340
150	original	1	60x8 weeks	Ag80	MWF	VAC95	100	All	2.999	0.001	0.000	0.001	0.000	0.000	0.059	1439.414	0.360	0.000	0.360	0.105	0.046	28.534	14394	3.600	0.000	3.600	1.050	0.460	285.340
150	original	1	60x8 weeks	Ag80	MWF	VAC95	100	All	2.999	0.001	0.000	0.001	0.000	0.000	0.059	1439.414	0.360	0.000	0.360	0.105	0.046	28.534	14394	3.600	0.000	3.600	1.050	0.460	285.340
150	original	1.2	60x8 weeks	Ag80	MWF	VAC95	100	All	2.999	0.001	0.000	0.001	0.000	0.000	0.060	1439.388	0.342	0.001	0.343	0.122	0.033	28.849	14394	3.420	0.010	3.430	1.220	0.330	288.490
150	original	1.2	60x8 weeks	Ag80	MWF	VAC95	100	All	2.999	0.001	0.000	0.001	0.000	0.000	0.060	1439.388	0.342	0.001	0.343	0.122	0.033	28.849	14394	3.420	0.010	3.430	1.220	0.330	288.490
150	original	1.2	60x8 weeks	Ag80	MWF	VAC95	100	All	2.999	0.001	0.000	0.001	0.000	0.000	0.060	1439.388	0.342	0.001	0.343	0.122	0.033	28.849	14394	3.420	0.010	3.430	1.220	0.330	288.490
150	alpha	0.6	60x8 weeks	Ag80	MWF	VAC95	100	All	2.996	0.001	0.000	0.001	0.000	0.001	0.060	1438.184	0.492	0.004	0.496	0.028	0.472	28.982	14382	4.920	0.040	4.960	0.280	4.720	289.820
150	alpha	0.6	60x8 weeks	Ag80	MWF	VAC95	100	All	2.996	0.001	0.000	0.001	0.000	0.001	0.060	1438.184	0.492	0.004	0.496	0.028	0.472	28.982	14382	4.920	0.040	4.960	0.280	4.720	289.820
150	alpha	0.6	60x8 weeks	Ag80	MWF	VAC95	100	All	2.996	0.001	0.000	0.001	0.000	0.001	0.060	1438.184	0.492	0.004	0.496	0.028	0.472	28.982	14382	4.920	0.040	4.960	0.280	4.720	289.820
150	alpha	1	60x8 weeks	Ag80	MWF	VAC95	100	All	2.997	0.001	0.000	0.001	0.000	0.001	0.060	1438.353	0.431	0.015	0.446	0.026	0.419	28.619	14384	4.310	0.150	4.460	0.260	4.190	286.190
150	alpha	1	60x8 weeks	Ag80	MWF	VAC95	100	All	2.997	0.001	0.000	0.001	0.000	0.001	0.060	1438.353	0.431	0.015	0.446	0.026	0.419	28.619	14384	4.310	0.150	4.460	0.260	4.190	286.190
150	alpha	1	60x8 weeks	Ag80	MWF	VAC95	100	All	2.997	0.001	0.000	0.001	0.000	0.001	0.060	1438.353	0.431	0.015	0.446	0.026	0.419	28.619	14384	4.310	0.150	4.460	0.260	4.190	286.190
150	alpha	1.2	60x8 weeks	Ag80	MWF	VAC95	100	All	2.996	0.001	0.000	0.001	0.000	0.001	0.059	1438.312	0.448	0.019	0.467	0.022	0.443	28.387	14383	4.480	0.190	4.670	0.220	4.430	283.870
150	alpha	1.2	60x8 weeks	Ag80	MWF	VAC95	100	All	2.996	0.001	0.000	0.001	0.000	0.001	0.059	1438.312	0.448	0.019	0.467	0.022	0.443	28.387	14383	4.480	0.190	4.670	0.220	4.430	283.870
150	alpha	1.2	60x8 weeks	Ag80	MWF	VAC95	100	All	2.996	0.001	0.000	0.001	0.000	0.001	0.059	1438.312	0.448	0.019	0.467	0.022	0.443	28.387	14383	4.480	0.190	4.670	0.220	4.430	283.870
150	delta	0.6	60x8 weeks	Ag80	MWF	VAC95	100	All	2.994	0.002	0.000	0.002	0.000	0.002	0.059	1437.137	0.734	0.036	0.770	0.043	0.738	28.331	14371	7.340	0.360	7.700	0.430	7.380	283.310
150	delta	0.6	60x8 weeks	Ag80	MWF	VAC95	100	All	2.994	0.002	0.000	0.002	0.000	0.002	0.059	1437.137	0.734	0.036	0.770	0.043	0.738	28.331	14371	7.340	0.360	7.700	0.430	7.380	283.310
150	delta	0.6	60x8 weeks	Ag80	MWF	VAC95	100	All	2.994	0.002	0.000	0.002	0.000	0.002	0.059	1437.137	0.734	0.036	0.770	0.043	0.738	28.331	14371	7.340	0.360	7.700	0.430	7.380	283.310
150	delta	1	60x8 weeks	Ag80	MWF	VAC95	100	All	2.994	0.002	0.000	0.002	0.000	0.002	0.059	1437.146	0.721	0.061	0.782	0.035	0.762	28.546	14371	7.210	0.610	7.820	0.350	7.620	285.460
150	delta	1	60x8 weeks	Ag80	MWF	VAC95	100	All	2.994	0.002	0.000	0.002	0.000	0.002	0.059	1437.146	0.721	0.061	0.782	0.035	0.762	28.546	14371	7.210	0.610	7.820	0.350	7.620	285.460
150	delta	1	60x8 weeks	Ag80	MWF	VAC95	100	All	2.994	0.002	0.000	0.002	0.000	0.002	0.059	1437.146	0.721	0.061	0.782	0.035	0.762	28.546	14371	7.210	0.610	7.820	0.350	7.620	285.460
150	delta	1.2	60x8 weeks	Ag80	MWF	VAC95	100	All	2.994	0.001	0.000	0.002	0.000	0.002	0.060	1437.014	0.703	0.096	0.799	0.041	0.772	28.745	14370	7.030	0.960	7.990	0.410	7.720	287.450
150	delta	1.2	60x8 weeks	Ag80	MWF	VAC95	100	All	2.994	0.001	0.000	0.002	0.000	0.002	0.060	1437.014	0.703	0.096	0.799	0.041	0.772	28.745	14370	7.030	0.960	7.990	0.410	7.720	287.450
150	delta	1.2	60x8 weeks	Ag80	MWF	VAC95	100	All	2.994	0.001	0.000	0.002	0.000	0.002	0.060	1437.014	0.703	0.096	0.799	0.041	0.772	28.745	14370	7.030	0.960	7.990	0.410	7.720	287.450
150	original	0.6	60x8 weeks	Ag80	MWF	VAC70	25	All	2.967	0.008	0.001	0.010	0.001	0.008	0.058	1424.085	3.986	0.613	4.599	0.362	4.001	27.807	14241	39.860	6.130	45.990	3.620	40.010	278.070
150	original	0.6	60x8 weeks	Ag80	MWF	VAC70	25	All	2.967	0.008	0.001	0.010	0.001	0.008	0.058	1424.085	3.986	0.613	4.599	0.362	4.001	27.807	14241	39.860	6.130	45.990	3.620	40.010	278.070
150	original	0.6	60x8 weeks	Ag80	MWF	VAC70	25	All	2.967	0.008	0.001	0.010	0.001	0.008	0.058	1424.085	3.986	0.613	4.599	0.362	4.001	27.807	14241	39.860	6.130	45.990	3.620	40.010	278.070
150	original	1	60x8 weeks	Ag80	MWF	VAC70	25	All	2.963	0.008	0.002	0.011	0.001	0.009	0.057	1422.372	3.999	1.090	5.089	0.420	4.436	27.540	14224	39.990	10.900	50.890	4.200	44.360	275.400
150	original	1	60x8 weeks	Ag80	MWF	VAC70	25	All	2.963	0.008	0.002	0.011	0.001	0.009	0.057	1422.372	3.999	1.090	5.089	0.420	4.436	27.540	14224	39.990	10.900	50.890	4.200	44.360	275.400
150	original	1	60x8 weeks	Ag80	MWF	VAC70	25	All	2.963	0.008	0.002	0.011	0.001	0.009	0.057	1422.372	3.999	1.090	5.089	0.420	4.436	27.540	14224	39.990	10.900	50.890	4.200	44.360	275.400
150	original	1.2	60x8 weeks	Ag80	MWF	VAC70	25	All	2.962	0.008	0.003	0.011	0.001	0.010	0.057	1421.606	3.986	1.457	5.443	0.439	4.680	27.551	14216	39.860	14.570	54.430	4.390	46.800	275.510

150	original	1.2	60x8 weeks	Ag80	MWF	VAC70	25	All	2.962	0.008	0.003	0.011	0.001	0.010	0.057	1421.606	3.986	1.457	5.443	0.439	4.680	27.551	14216	39.860	14.570	54.430	4.390	46.800	275.510
150	original	1.2	60x8 weeks	Ag80	MWF	VAC70	25	All	2.962	0.008	0.003	0.011	0.001	0.010	0.057	1421.606	3.986	1.457	5.443	0.439	4.680	27.551	14216	39.860	14.570	54.430	4.390	46.800	275.510
150	alpha	0.6	60x8 weeks	Ag80	MWF	VAC70	25	All	2.960	0.008	0.002	0.011	0.000	0.011	0.057	1420.847	3.913	1.154	5.067	0.219	5.054	27.562	14208	39.130	11.540	50.670	2.190	50.540	275.620
150	alpha	0.6	60x8 weeks	Ag80	MWF	VAC70	25	All	2.960	0.008	0.002	0.011	0.000	0.011	0.057	1420.847	3.913	1.154	5.067	0.219	5.054	27.562	14208	39.130	11.540	50.670	2.190	50.540	275.620
150	alpha	0.6	60x8 weeks	Ag80	MWF	VAC70	25	All	2.960	0.008	0.002	0.011	0.000	0.011	0.057	1420.847	3.913	1.154	5.067	0.219	5.054	27.562	14208	39.130	11.540	50.670	2.190	50.540	275.620
150	alpha	1	60x8 weeks	Ag80	MWF	VAC70	25	All	2.949	0.008	0.005	0.013	0.001	0.013	0.057	1415.681	4.006	2.428	6.434	0.281	6.474	27.221	14157	40.060	24.280	64.340	2.810	64.740	272.210
150	alpha	1	60x8 weeks	Ag80	MWF	VAC70	25	All	2.949	0.008	0.005	0.013	0.001	0.013	0.057	1415.681	4.006	2.428	6.434	0.281	6.474	27.221	14157	40.060	24.280	64.340	2.810	64.740	272.210
150	alpha	1	60x8 weeks	Ag80	MWF	VAC70	25	All	2.949	0.008	0.005	0.013	0.001	0.013	0.057	1415.681	4.006	2.428	6.434	0.281	6.474	27.221	14157	40.060	24.280	64.340	2.810	64.740	272.210
150	alpha	1.2	60x8 weeks	Ag80	MWF	VAC70	25	All	2.942	0.008	0.007	0.015	0.001	0.015	0.056	1412.322	3.928	3.503	7.431	0.335	7.414	26.929	14123	39.280	35.030	74.310	3.350	74.140	269.290
150	alpha	1.2	60x8 weeks	Ag80	MWF	VAC70	25	All	2.942	0.008	0.007	0.015	0.001	0.015	0.056	1412.322	3.928	3.503	7.431	0.335	7.414	26.929	14123	39.280	35.030	74.310	3.350	74.140	269.290
150	alpha	1.2	60x8 weeks	Ag80	MWF	VAC70	25	All	2.942	0.008	0.007	0.015	0.001	0.015	0.056	1412.322	3.928	3.503	7.431	0.335	7.414	26.929	14123	39.280	35.030	74.310	3.350	74.140	269.290
150	delta	0.6	60x8 weeks	Ag80	MWF	VAC70	25	All	2.936	0.008	0.008	0.017	0.001	0.016	0.056	1409.343	4.031	3.946	7.977	0.484	7.913	26.790	14093	40.310	39.460	79.770	4.840	79.130	267.900
150	delta	0.6	60x8 weeks	Ag80	MWF	VAC70	25	All	2.936	0.008	0.008	0.017	0.001	0.016	0.056	1409.343	4.031	3.946	7.977	0.484	7.913	26.790	14093	40.310	39.460	79.770	4.840	79.130	267.900
150	delta	0.6	60x8 weeks	Ag80	MWF	VAC70	25	All	2.936	0.008	0.008	0.017	0.001	0.016	0.056	1409.343	4.031	3.946	7.977	0.484	7.913	26.790	14093	40.310	39.460	79.770	4.840	79.130	267.900
150	delta	1	60x8 weeks	Ag80	MWF	VAC70	25	All	2.882	0.008	0.023	0.030	0.002	0.030	0.054	1383.580	3.680	10.873	14.553	0.988	14.438	25.876	13836	36.800	108.730	145.530	9.880	144.380	258.760
150	delta	1	60x8 weeks	Ag80	MWF	VAC70	25	All	2.882	0.008	0.023	0.030	0.002	0.030	0.054	1383.580	3.680	10.873	14.553	0.988	14.438	25.876	13836	36.800	108.730	145.530	9.880	144.380	258.760
150	delta	1	60x8 weeks	Ag80	MWF	VAC70	25	All	2.882	0.008	0.023	0.030	0.002	0.030	0.054	1383.580	3.680	10.873	14.553	0.988	14.438	25.876	13836	36.800	108.730	145.530	9.880	144.380	258.760
150	delta	1.2	60x8 weeks	Ag80	MWF	VAC70	25	All	2.822	0.007	0.038	0.045	0.003	0.045	0.049	1354.647	3.438	18.224	21.662	1.487	21.546	23.668	13546	34.380	182.240	216.620	14.870	215.460	236.680
150	delta	1.2	60x8 weeks	Ag80	MWF	VAC70	25	All	2.822	0.007	0.038	0.045	0.003	0.045	0.049	1354.647	3.438	18.224	21.662	1.487	21.546	23.668	13546	34.380	182.240	216.620	14.870	215.460	236.680
150	delta	1.2	60x8 weeks	Ag80	MWF	VAC70	25	All	2.822	0.007	0.038	0.045	0.003	0.045	0.049	1354.647	3.438	18.224	21.662	1.487	21.546	23.668	13546	34.380	182.240	216.620	14.870	215.460	236.680
150	original	0.6	60x8 weeks	Ag80	MWF	VAC70	50	All	2.976	0.007	0.001	0.007	0.001	0.005	0.058	1428.536	3.295	0.297	3.592	0.509	2.639	27.956	14285	32.950	2.970	35.920	5.090	26.390	279.560
150	original	0.6	60x8 weeks	Ag80	MWF	VAC70	50	All	2.976	0.007	0.001	0.007	0.001	0.005	0.058	1428.536	3.295	0.297	3.592	0.509	2.639	27.956	14285	32.950	2.970	35.920	5.090	26.390	279.560
150	original	0.6	60x8 weeks	Ag80	MWF	VAC70	50	All	2.976	0.007	0.001	0.007	0.001	0.005	0.058	1428.536	3.295	0.297	3.592	0.509	2.639	27.956	14285	32.950	2.970	35.920	5.090	26.390	279.560
150	original	1	60x8 weeks	Ag80	MWF	VAC70	50	All	2.976	0.007	0.001	0.008	0.001	0.006	0.058	1428.248	3.208	0.523	3.731	0.541	2.698	27.739	14282	32.080	5.230	37.310	5.410	26.980	277.390
150	original	1	60x8 weeks	Ag80	MWF	VAC70	50	All	2.976	0.007	0.001	0.008	0.001	0.006	0.058	1428.248	3.208	0.523	3.731	0.541	2.698	27.739	14282	32.080	5.230	37.310	5.410	26.980	277.390
150	original	1	60x8 weeks	Ag80	MWF	VAC70	50	All	2.976	0.007	0.001	0.008	0.001	0.006	0.058	1428.248	3.208	0.523	3.731	0.541	2.698	27.739	14282	32.080	5.230	37.310	5.410	26.980	277.390
150	original	1.2	60x8 weeks	Ag80	MWF	VAC70	50	All	2.973	0.007	0.001	0.008	0.001	0.006	0.058	1427.277	3.238	0.706	3.944	0.543	2.961	27.830	14273	32.380	7.060	39.440	5.430	29.610	278.300
150	original	1.2	60x8 weeks	Ag80	MWF	VAC70	50	All	2.973	0.007	0.001	0.008	0.001	0.006	0.058	1427.277	3.238	0.706	3.944	0.543	2.961	27.830	14273	32.380	7.060	39.440	5.430	29.610	278.300
150	original	1.2	60x8 weeks	Ag80	MWF	VAC70	50	All	2.973	0.007	0.001	0.008	0.001	0.006	0.058	1427.277	3.238	0.706	3.944	0.543	2.961	27.830	14273	32.380	7.060	39.440	5.430	29.610	278.300
150	alpha	0.6	60x8 weeks	Ag80	MWF	VAC70	50	All	2.969	0.007	0.001	0.008	0.000	0.008	0.059	1425.286	3.247	0.716	3.963	0.158	3.953	28.105	14253	32.470	7.160	39.630	1.580	39.530	281.050
150	alpha	0.6	60x8 weeks	Ag80	MWF	VAC70	50	All	2.969	0.007	0.001	0.008	0.000	0.008	0.059	1425.286	3.247	0.716	3.963	0.158	3.953	28.105	14253	32.470	7.160	39.630	1.580	39.530	281.050
150	alpha	0.6	60x8 weeks	Ag80	MWF	VAC70	50	All	2.969	0.007	0.001	0.008	0.000	0.008	0.059	1425.286	3.247	0.716	3.963	0.158	3.953	28.105	14253	32.470	7.160	39.630	1.580	39.530	281.050
150	alpha	1	60x8 weeks	Ag80	MWF	VAC70	50	All	2.964	0.007	0.003	0.009	0.000	0.009	0.057	1422.944	3.245	1.298	4.543	0.206	4.516	27.513	14229	32.450	12.980	45.430	2.060	45.160	275.130
150	alpha	1	60x8 weeks	Ag80	MWF	VAC70	50	All	2.964	0.007	0.003	0.009	0.000	0.009	0.057	1422.944	3.245	1.298	4.543	0.206	4.516	27.513	14229	32.450	12.980	45.430	2.060	45.160	275.130
150	alpha	1	60x8 weeks	Ag80	MWF	VAC70	50	All	2.964	0.007	0.003	0.009	0.000	0.009	0.057	1422.944	3.245	1.298	4.543	0.206	4.516	27.513	14229	32.450	12.980	45.430	2.060	45.160	275.130
150	alpha	1.2	60x8 weeks	Ag80	MWF	VAC70	50	All	2.963	0.007	0.003	0.010	0.000	0.010	0.058	1422.007	3.175	1.625	4.800	0.219	4.747	27.707	14220	31.750	16.250	48.000	2.190	47.470	277.070
150	alpha	1.2	60x8 weeks	Ag80	MWF	VAC70	50	All	2.963	0.007	0.003	0.010	0.000	0.010	0.058	1422.007	3.175	1.625	4.800	0.219	4.747	27.707	14220	31.750	16.250	48.000	2.190	47.470	277.070
150	alpha	1.2	60x8 weeks	Ag80	MWF	VAC70	50	All	2.963	0.007	0.003	0.010	0.000	0.010	0.058	1422.007	3.175	1.625	4.800	0.219	4.747	27.707	14220	31.750	16.250	48.000	2.190	47.470	277.070
150	delta	0.6	60x8 weeks	Ag80	MWF	VAC70	50	All	2.957	0.007	0.004	0.011	0.001	0.011	0.057	1419.483	3.327	2.056	5.383	0.351	5.302	27.590	14195	33.270	20.560	53.830	3.510	53.020	275.900
150	delta	0.6	60x8 weeks	Ag80	MWF	VAC70	50	All	2.957	0.007	0.004	0.011	0.001	0.011	0.057	1419.483	3.327	2.056	5.383	0.351	5.302	27.590	14195	33.270	20.560	53.830	3.510	53.020	275.900
150	delta	0.6	60x8 weeks	Ag80	MWF	VAC70	50	All	2.957	0.007	0.004	0.011	0.001	0.011	0.057	1419.483	3.327	2.056	5.383	0.351	5.302	27.590	14195	33.270	20.560	53.830	3.510	53.020	275.900
150	delta	1	60x8 weeks	Ag80	MWF	VAC70	50	All	2.931	0.007	0.011	0.018	0.001	0.018	0.056	1406.977	3.281	5.369	8.650	0.534	8.544	26.811	14070	32.810	53.690	86.500	5.340	85.440	268.110
150	delta	1	60x8 weeks	Ag80	MWF	VAC70	50	All	2.931	0.007	0.011	0.018	0.001	0.018	0.056	1406.977	3.281	5.369	8.650	0.534	8.544	26.811	14070	32.810	53.690	86.500	5.340	85.440	268.110
150	delta	1	60x8 weeks	Ag80	MWF	VAC70	50	All	2.931	0.007	0.011	0.018	0.001	0.018	0.056	1406.977	3.281	5.369	8.650	0.534	8.544	26.811	14070	32.810	53.690	86.500	5.340	85.440	268.110
150	delta	1.2	60x8 weeks	Ag80	MWF	VAC70	50	All	2.910	0.007	0.016	0.023	0.002	0.023	0.054	1396.929	3.198	7.801	10.999	0.752	10.975	26.003	13969	31.980	78.010	109.990	7.520	109.750	260.030
150	delta	1.2	60x8 weeks	Ag80	MWF	VAC70	50	All	2.910	0.007	0.016	0.023	0.002	0.023	0.054	1396.929	3.198	7.801	10.999	0.752	10.975	26.003	13969	31.980	78.010	109.990	7.520	109.750	260.030
150	delta	1.2	60x8 weeks	Ag80	MWF	VAC70	50	All	2.910	0.007	0.016	0.023	0.002	0.023	0.054	1396.929	3.198	7.801	10.999	0.752	10.975	26.003	13969	31.980	78.010	109.990	7.520	109.750	260.030
150	original	0.6	60x8 weeks	Ag80	MWF	VAC70	75	All	2.985	0.005	0.000	0.005	0.001	0.003	0.059	1432.941	2.394	0.126	2.520	0.583	1.334	28.297	14329	23.940	1.260	25.200	5.830	13.340	282.970
150	original	0.6	60x8 weeks	Ag80	MWF	VAC70	75	All	2.985	0.005	0.000	0.005	0.001	0.003	0.059	1432.941	2.394	0.126	2.520	0.583	1.334	28.297	14329	23.940	1.260	25.200	5.830	13.340	282.970
150	original	0.6	60x8 weeks	Ag80	MWF	VAC70	75	All	2.985	0.005	0.000	0.005	0.001	0.003	0.059	1432.941	2.394	0.126	2.520	0.583	1.334	28.297	14329	23.940	1.260	25.200	5.830	13.340	282.970
150	original	1	60x8 weeks	Ag80	MWF	VAC70	75	All	2.984	0.005	0.000	0.006	0.001	0.003	0.058	1432.339	2.459	0.208	2.667	0.611	1.464	27.950	14323	24.590	2.080	26.670	6.110	14.640	279.500
150	original	1	60x8 weeks	Ag80	MWF	VAC70	75	All	2.984	0.005	0.000	0.006	0.001	0.003	0.058	1432.339	2.459	0.208	2.667	0.611	1.464	27.950	14323	24.590	2.080	26.670	6.110	14.640	279.500
150	original	1	60x8 weeks	Ag80	MWF	VAC70	75	All	2.984	0.005	0.000	0.006	0.001	0.003	0.058	1432.339	2.459	0.208	2.667	0.611	1.464	27.950	14323	24.590	2.080	26.670	6.110	14.640	279.500
150	original	1.2	60x8 weeks	Ag80	MWF	VAC70	75	All	2.984	0.005	0.001	0.006	0.001	0.003	0.059	1432.462	2.443	0.274	2.717	0.633	1.433	28.137	14325	24.430	2.740	27.170	6.330	14.330	281.370
150	original	1.2	60x8 weeks	Ag80	MWF	VAC70	75	All	2.984	0.005	0.001	0.006	0.001	0.003	0.059	1432.462	2.443	0.274	2.717	0.633	1.433	28.137	14325	24.430	2.740	27.170	6.330	14.330	281.370
150	original	1.2	60x8 weeks	Ag80	MWF	VAC70	75	All	2.984	0.005	0.001	0.006	0.001	0.003	0.059	1432.462	2.443	0.274	2.717	0.633	1.433	28.137	14325	24.430	2.740	27.170	6.330	14.330	281.370
150	alpha	0.6	60x8 weeks	Ag80	MWF	VAC70	75	All	2.979	0.005	0.001	0.006	0.000	0.006	0.059	1429.819	2.397	0.305	2.702	0.111	2.704	28.218	14298	23.970	3.050	27.020	1.110	27.040	282.180
150	alpha	0.6	60x8 weeks	Ag80	MWF	VAC70	75	All	2.979	0.005	0.001	0.006	0.000	0.006	0.059	1429.819	2.397	0.305	2.702	0.111	2.704	28.218	14298	23.970	3.050	27.020	1.110	27.040	282.180
150	alpha	0.6	60x8 weeks	Ag80	MWF	VAC70	75	All	2.979	0.005	0.001	0.006	0.000	0.006	0.059	1429.819	2.397	0.305	2.702	0.111	2.704	28.218	14298	23.970	3.050	27.020	1.110	27.040	282.180
150	alpha	1	60x8 weeks	Ag80	MWF	VAC70	75	All	2.978	0.005	0.001	0.006	0.000	0.006	0.059	1429.430	2.316	0.520	2.836	0.136	2.791	28.388	14294	23.160	5.200	28.360	1.360	27.910	283.880
150	alpha	1	60x8 weeks	Ag80	MWF	VAC70	75	All	2.978	0.005	0.001	0.006	0.000	0.006	0.059	1429.430	2.316	0.520	2.836	0.136	2.791	28.388	14294	23.160	5.200	28.360	1.360	27.910	283.880
150	alpha	1	60x8 weeks	Ag80	MWF	VAC70	75	All	2.978	0.005	0.001	0.006	0.000	0.006	0.059	1429.430	2.316	0.520	2.836	0.136	2.791	28.388	14294	23.160	5.200	28.360	1.360	27.910	283.880
150	alpha	1.2	60x8 weeks	Ag80	MWF	VAC70	75	All	2.976	0.005	0.001	0.006	0.000	0.006	0.058	1428.396	2.459	0.649	3.108	0.144	3.062	28.037	14284	24.590	6.490	31.080	1.440	30.620	280.370
150	alpha	1.2	60x8 weeks	Ag80	MWF	VAC70	75	All	2.976	0.005	0.001	0.006	0.000	0.006	0.058	1428.396	2.459	0.649	3.108	0.144	3.062	28.037	14284	24.590	6.490	31.080	1.440	30.620	280.370
150	alpha	1.2	60x8 weeks	Ag80	MWF	VAC70	75	All	2.976	0.005	0.001	0.006	0.000	0.006	0.058	1428.396	2.459	0.649	3.108	0.144	3.062	28.037	14284	24.590	6.490	31.080	1.440	30.620	280.370
150	delta	0.6	60x8 weeks	Ag80	MWF	VAC70	75	All	2.971	0.006	0.002	0.008	0.000	0.008	0.058	1425.898	2.801	0.968	3.769	0.212	3.715	27.803	14259	28.010	9.680	37.690	2.120	37.150	278.030
150	delta	0.6	60x8 weeks	Ag80	MWF	VAC70	75	All	2.971	0.006	0.002	0.008	0.000	0.008	0.058	1425.898	2.801	0.968	3.769	0.212	3.715	27.803	14259	28.010	9.680	37.690	2.120	37.150	278.030
150	delta	0.6	60x8 weeks	Ag80	MWF	VAC70	75	All	2.971	0.006	0.002	0.008	0.000	0.008	0.058	1425.898	2.801	0.968	3.769	0.212	3.715	27.803	14259	28.010	9.680	37.690	2.120	37.150	278.030
150	delta	1	60x8 weeks	Ag80	MWF	VAC70	75	All	2.963	0.006	0.004	0.010	0.001	0.010	0.058	1422.175	2.752	1.940	4.692	0.315	4.595	27.861	14222	27.520	19.400	46.920	3.150	45.950	278.610
150	delta	1	60x8 weeks	Ag80	MWF	VAC70	75	All	2.963	0.006	0.004	0.010	0.001	0.010	0.058	1422.175	2.752	1.940	4.692	0.315	4.595	27.861	14222	27.520	19.400	46.920	3.150	45.950	278.610
150	delta	1	60x8 weeks	Ag80	MWF	VAC70	75	All	2.963	0.006	0.004	0.010	0.001	0.010	0.058	1422.175	2.752	1.940	4.692	0.315	4.595	27.861	14222	27.520	19.400	46.920	3.150	45.950	278.610

150	delta	1.2	60x8 weeks	Ag80	MWF	VAC70	75	All	2.955	0.006	0.006	0.012	0.001	0.012	0.058	1418.278	2.767	2.934	5.701	0.366	5.613	27.679	14183	27.670	29.340	57.010	3.660	56.130	276.790
150	delta	1.2	60x8 weeks	Ag80	MWF	VAC70	75	All	2.955	0.006	0.006	0.012	0.001	0.012	0.058	1418.278	2.767	2.934	5.701	0.366	5.613	27.679	14183	27.670	29.340	57.010	3.660	56.130	276.790
150	delta	1.2	60x8 weeks	Ag80	MWF	VAC70	75	All	2.955	0.006	0.006	0.012	0.001	0.012	0.058	1418.278	2.767	2.934	5.701	0.366	5.613	27.679	14183	27.670	29.340	57.010	3.660	56.130	276.790
150	original	0.6	60x8 weeks	Ag80	MWF	VAC70	100	All	2.993	0.003	0.000	0.003	0.001	0.000	0.059	1436.611	1.659	0.009	1.668	0.671	0.207	28.532	14366	16.590	0.090	16.680	6.710	2.070	285.320
150	original	0.6	60x8 weeks	Ag80	MWF	VAC70	100	All	2.993	0.003	0.000	0.003	0.001	0.000	0.059	1436.611	1.659	0.009	1.668	0.671	0.207	28.532	14366	16.590	0.090	16.680	6.710	2.070	285.320
150	original	0.6	60x8 weeks	Ag80	MWF	VAC70	100	All	2.993	0.003	0.000	0.003	0.001	0.000	0.059	1436.611	1.659	0.009	1.668	0.671	0.207	28.532	14366	16.590	0.090	16.680	6.710	2.070	285.320
150	original	1	60x8 weeks	Ag80	MWF	VAC70	100	All	2.993	0.003	0.000	0.003	0.001	0.000	0.059	1436.551	1.648	0.019	1.667	0.695	0.201	28.152	14366	16.480	0.190	16.670	6.950	2.010	281.520
150	original	1	60x8 weeks	Ag80	MWF	VAC70	100	All	2.993	0.003	0.000	0.003	0.001	0.000	0.059	1436.551	1.648	0.019	1.667	0.695	0.201	28.152	14366	16.480	0.190	16.670	6.950	2.010	281.520
150	original	1	60x8 weeks	Ag80	MWF	VAC70	100	All	2.993	0.003	0.000	0.003	0.001	0.000	0.059	1436.551	1.648	0.019	1.667	0.695	0.201	28.152	14366	16.480	0.190	16.670	6.950	2.010	281.520
150	original	1.2	60x8 weeks	Ag80	MWF	VAC70	100	All	2.993	0.003	0.000	0.003	0.001	0.000	0.059	1436.785	1.593	0.019	1.612	0.657	0.190	28.296	14368	15.930	0.190	16.120	6.570	1.900	282.960
150	original	1.2	60x8 weeks	Ag80	MWF	VAC70	100	All	2.993	0.003	0.000	0.003	0.001	0.000	0.059	1436.785	1.593	0.019	1.612	0.657	0.190	28.296	14368	15.930	0.190	16.120	6.570	1.900	282.960
150	original	1.2	60x8 weeks	Ag80	MWF	VAC70	100	All	2.993	0.003	0.000	0.003	0.001	0.000	0.059	1436.785	1.593	0.019	1.612	0.657	0.190	28.296	14368	15.930	0.190	16.120	6.570	1.900	282.960
150	alpha	0.6	60x8 weeks	Ag80	MWF	VAC70	100	All	2.987	0.003	0.000	0.003	0.000	0.003	0.059	1433.905	1.558	0.103	1.661	0.073	1.614	28.368	14339	15.580	1.030	16.610	0.730	16.140	283.680
150	alpha	0.6	60x8 weeks	Ag80	MWF	VAC70	100	All	2.987	0.003	0.000	0.003	0.000	0.003	0.059	1433.905	1.558	0.103	1.661	0.073	1.614	28.368	14339	15.580	1.030	16.610	0.730	16.140	283.680
150	alpha	0.6	60x8 weeks	Ag80	MWF	VAC70	100	All	2.987	0.003	0.000	0.003	0.000	0.003	0.059	1433.905	1.558	0.103	1.661	0.073	1.614	28.368	14339	15.580	1.030	16.610	0.730	16.140	283.680
150	alpha	1	60x8 weeks	Ag80	MWF	VAC70	100	All	2.987	0.003	0.000	0.004	0.000	0.003	0.059	1433.799	1.573	0.153	1.726	0.092	1.634	28.212	14338	15.730	1.530	17.260	0.920	16.340	282.120
150	alpha	1	60x8 weeks	Ag80	MWF	VAC70	100	All	2.987	0.003	0.000	0.004	0.000	0.003	0.059	1433.799	1.573	0.153	1.726	0.092	1.634	28.212	14338	15.730	1.530	17.260	0.920	16.340	282.120
150	alpha	1	60x8 weeks	Ag80	MWF	VAC70	100	All	2.987	0.003	0.000	0.004	0.000	0.003	0.059	1433.799	1.573	0.153	1.726	0.092	1.634	28.212	14338	15.730	1.530	17.260	0.920	16.340	282.120
150	alpha	1.2	60x8 weeks	Ag80	MWF	VAC70	100	All	2.986	0.003	0.000	0.004	0.000	0.004	0.059	1433.139	1.664	0.237	1.901	0.104	1.802	28.202	14331	16.640	2.370	19.010	1.040	18.020	282.020
150	alpha	1.2	60x8 weeks	Ag80	MWF	VAC70	100	All	2.986	0.003	0.000	0.004	0.000	0.004	0.059	1433.139	1.664	0.237	1.901	0.104	1.802	28.202	14331	16.640	2.370	19.010	1.040	18.020	282.020
150	alpha	1.2	60x8 weeks	Ag80	MWF	VAC70	100	All	2.986	0.003	0.000	0.004	0.000	0.004	0.059	1433.139	1.664	0.237	1.901	0.104	1.802	28.202	14331	16.640	2.370	19.010	1.040	18.020	282.020
150	delta	0.6	60x8 weeks	Ag80	MWF	VAC70	100	All	2.981	0.004	0.001	0.005	0.000	0.005	0.059	1430.819	2.095	0.360	2.455	0.163	2.365	28.318	14308	20.950	3.600	24.550	1.630	23.650	283.180
150	delta	0.6	60x8 weeks	Ag80	MWF	VAC70	100	All	2.981	0.004	0.001	0.005	0.000	0.005	0.059	1430.819	2.095	0.360	2.455	0.163	2.365	28.318	14308	20.950	3.600	24.550	1.630	23.650	283.180
150	delta	0.6	60x8 weeks	Ag80	MWF	VAC70	100	All	2.981	0.004	0.001	0.005	0.000	0.005	0.059	1430.819	2.095	0.360	2.455	0.163	2.365	28.318	14308	20.950	3.600	24.550	1.630	23.650	283.180
150	delta	1	60x8 weeks	Ag80	MWF	VAC70	100	All	2.979	0.004	0.001	0.006	0.000	0.005	0.058	1429.906	2.064	0.622	2.686	0.165	2.624	27.989	14299	20.640	6.220	26.860	1.650	26.240	279.890
150	delta	1	60x8 weeks	Ag80	MWF	VAC70	100	All	2.979	0.004	0.001	0.006	0.000	0.005	0.058	1429.906	2.064	0.622	2.686	0.165	2.624	27.989	14299	20.640	6.220	26.860	1.650	26.240	279.890
150	delta	1	60x8 weeks	Ag80	MWF	VAC70	100	All	2.979	0.004	0.001	0.006	0.000	0.005	0.058	1429.906	2.064	0.622	2.686	0.165	2.624	27.989	14299	20.640	6.220	26.860	1.650	26.240	279.890
150	delta	1.2	60x8 weeks	Ag80	MWF	VAC70	100	All	2.978	0.004	0.002	0.006	0.000	0.006	0.059	1429.223	2.060	0.803	2.863	0.159	2.801	28.199	14292	20.600	8.030	28.630	1.590	28.010	281.990
150	delta	1.2	60x8 weeks	Ag80	MWF	VAC70	100	All	2.978	0.004	0.002	0.006	0.000	0.006	0.059	1429.223	2.060	0.803	2.863	0.159	2.801	28.199	14292	20.600	8.030	28.630	1.590	28.010	281.990
150	delta	1.2	60x8 weeks	Ag80	MWF	VAC70	100	All	2.978	0.004	0.002	0.006	0.000	0.006	0.059	1429.223	2.060	0.803	2.863	0.159	2.801	28.199	14292	20.600	8.030	28.630	1.590	28.010	281.990
150	original	0.6	60x8 weeks	Ag80	MWF	VAC50	25	All	2.965	0.009	0.001	0.010	0.001	0.008	0.057	1423.399	4.211	0.663	4.874	0.492	4.070	27.385	14234	42.110	6.630	48.740	4.920	40.700	273.850
150	original	0.6	60x8 weeks	Ag80	MWF	VAC50	25	All	2.965	0.009	0.001	0.010	0.001	0.008	0.057	1423.399	4.211	0.663	4.874	0.492	4.070	27.385	14234	42.110	6.630	48.740	4.920	40.700	273.850
150	original	0.6	60x8 weeks	Ag80	MWF	VAC50	25	All	2.965	0.009	0.001	0.010	0.001	0.008	0.057	1423.399	4.211	0.663	4.874	0.492	4.070	27.385	14234	42.110	6.630	48.740	4.920	40.700	273.850
150	original	1	60x8 weeks	Ag80	MWF	VAC50	25	All	2.962	0.009	0.002	0.011	0.001	0.009	0.057	1421.689	4.250	1.176	5.426	0.566	4.469	27.336	14217	42.500	11.760	54.260	5.660	44.690	273.360
150	original	1	60x8 weeks	Ag80	MWF	VAC50	25	All	2.962	0.009	0.002	0.011	0.001	0.009	0.057	1421.689	4.250	1.176	5.426	0.566	4.469	27.336	14217	42.500	11.760	54.260	5.660	44.690	273.360
150	original	1	60x8 weeks	Ag80	MWF	VAC50	25	All	2.962	0.009	0.002	0.011	0.001	0.009	0.057	1421.689	4.250	1.176	5.426	0.566	4.469	27.336	14217	42.500	11.760	54.260	5.660	44.690	273.360
150	original	1.2	60x8 weeks	Ag80	MWF	VAC50	25	All	2.957	0.009	0.003	0.012	0.001	0.010	0.057	1419.556	4.390	1.570	5.960	0.585	5.006	27.306	14196	43.900	15.700	59.600	5.850	50.060	273.060
150	original	1.2	60x8 weeks	Ag80	MWF	VAC50	25	All	2.957	0.009	0.003	0.012	0.001	0.010	0.057	1419.556	4.390	1.570	5.960	0.585	5.006	27.306	14196	43.900	15.700	59.600	5.850	50.060	273.060
150	original	1.2	60x8 weeks	Ag80	MWF	VAC50	25	All	2.957	0.009	0.003	0.012	0.001	0.010	0.057	1419.556	4.390	1.570	5.960	0.585	5.006	27.306	14196	43.900	15.700	59.600	5.850	50.060	273.060
150	alpha	0.6	60x8 weeks	Ag80	MWF	VAC50	25	All	2.957	0.009	0.003	0.011	0.001	0.011	0.057	1419.286	4.209	1.310	5.519	0.266	5.484	27.426	14193	42.090	13.100	55.190	2.660	54.840	274.260
150	alpha	0.6	60x8 weeks	Ag80	MWF	VAC50	25	All	2.957	0.009	0.003	0.011	0.001	0.011	0.057	1419.286	4.209	1.310	5.519	0.266	5.484	27.426	14193	42.090	13.100	55.190	2.660	54.840	274.260
150	alpha	0.6	60x8 weeks	Ag80	MWF	VAC50	25	All	2.957	0.009	0.003	0.011	0.001	0.011	0.057	1419.286	4.209	1.310	5.519	0.266	5.484	27.426	14193	42.090	13.100	55.190	2.660	54.840	274.260
150	alpha	1	60x8 weeks	Ag80	MWF	VAC50	25	All	2.944	0.009	0.006	0.015	0.001	0.015	0.057	1413.149	4.215	2.867	7.082	0.334	7.091	27.262	14131	42.150	28.670	70.820	3.340	70.910	272.620
150	alpha	1	60x8 weeks	Ag80	MWF	VAC50	25	All	2.944	0.009	0.006	0.015	0.001	0.015	0.057	1413.149	4.215	2.867	7.082	0.334	7.091	27.262	14131	42.150	28.670	70.820	3.340	70.910	272.620
150	alpha	1	60x8 weeks	Ag80	MWF	VAC50	25	All	2.944	0.009	0.006	0.015	0.001	0.015	0.057	1413.149	4.215	2.867	7.082	0.334	7.091	27.262	14131	42.150	28.670	70.820	3.340	70.910	272.620
150	alpha	1.2	60x8 weeks	Ag80	MWF	VAC50	25	All	2.938	0.008	0.008	0.016	0.001	0.016	0.056	1410.383	4.055	3.791	7.846	0.374	7.808	27.062	14104	40.550	37.910	78.460	3.740	78.080	270.620
150	alpha	1.2	60x8 weeks	Ag80	MWF	VAC50	25	All	2.938	0.008	0.008	0.016	0.001	0.016	0.056	1410.383	4.055	3.791	7.846	0.374	7.808	27.062	14104	40.550	37.910	78.460	3.740	78.080	270.620
150	alpha	1.2	60x8 weeks	Ag80	MWF	VAC50	25	All	2.938	0.008	0.008	0.016	0.001	0.016	0.056	1410.383	4.055	3.791	7.846	0.374	7.808	27.062	14104	40.550	37.910	78.460	3.740	78.080	270.620
150	delta	0.6	60x8 weeks	Ag80	MWF	VAC50	25	All	2.930	0.009	0.009	0.018	0.001	0.018	0.055	1406.409	4.374	4.413	8.787	0.556	8.627	26.603	14064	43.740	44.130	87.870	5.560	86.270	266.030
150	delta	0.6	60x8 weeks	Ag80	MWF	VAC50	25	All	2.930	0.009	0.009	0.018	0.001	0.018	0.055	1406.409	4.374	4.413	8.787	0.556	8.627	26.603	14064	43.740	44.130	87.870	5.560	86.270	266.030
150	delta	0.6	60x8 weeks	Ag80	MWF	VAC50	25	All	2.930	0.009	0.009	0.018	0.001	0.018	0.055	1406.409	4.374	4.413	8.787	0.556	8.627	26.603	14064	43.740	44.130	87.870	5.560	86.270	266.030
150	delta	1	60x8 weeks	Ag80	MWF	VAC50	25	All	2.853	0.008	0.029	0.037	0.002	0.037	0.051	1369.642	3.983	13.839	17.822	1.180	17.859	24.441	13696	39.830	138.390	178.220	11.800	178.590	244.410
150	delta	1	60x8 weeks	Ag80	MWF	VAC50	25	All	2.853	0.008	0.029	0.037	0.002	0.037	0.051	1369.642	3.983	13.839	17.822	1.180	17.859	24.441	13696	39.830	138.390	178.220	11.800	178.590	244.410
150	delta	1	60x8 weeks	Ag80	MWF	VAC50	25	All	2.853	0.008	0.029	0.037	0.002	0.037	0.051	1369.642	3.983	13.839	17.822	1.180	17.859	24.441	13696	39.830	138.390	178.220	11.800	178.590	244.410
150	delta	1.2	60x8 weeks	Ag80	MWF	VAC50	25	All	2.790	0.008	0.046	0.053	0.004	0.053	0.047	1339.106	3.683	21.891	25.574	1.761	25.453	22.611	13391	36.830	218.910	255.740	17.610	254.530	226.110
150	delta	1.2	60x8 weeks	Ag80	MWF	VAC50	25	All	2.790	0.008	0.046	0.053	0.004	0.053	0.047	1339.106	3.683	21.891	25.574	1.761	25.453	22.611	13391	36.830	218.910	255.740	17.610	254.530	226.110
150	delta	1.2	60x8 weeks	Ag80	MWF	VAC50	25	All	2.790	0.008	0.046	0.053	0.004	0.053	0.047	1339.106	3.683	21.891	25.574	1.761	25.453	22.611	13391	36.830	218.910	255.740	17.610	254.530	226.110
150	original	0.6	60x8 weeks	Ag80	MWF	VAC50	50	All	2.974	0.008	0.001	0.008	0.001	0.006	0.058	1427.728	3.682	0.393	4.075	0.699	2.676	27.903	14277	36.820	3.930	40.750	6.990	26.760	279.030
150	original	0.6	60x8 weeks	Ag80	MWF	VAC50	50	All	2.974	0.008	0.001	0.008	0.001	0.006	0.058	1427.728	3.682	0.393	4.075	0.699	2.676	27.903	14277	36.820	3.930	40.750	6.990	26.760	279.030
150	original	0.6	60x8 weeks	Ag80	MWF	VAC50	50	All	2.974	0.008	0.001	0.008	0.001	0.006	0.058	1427.728	3.682	0.393	4.075	0.699	2.676	27.903	14277	36.820	3.930	40.750	6.990	26.760	279.030
150	original	1	60x8 weeks	Ag80	MWF	VAC50	50	All	2.973	0.007	0.001	0.009	0.002	0.006	0.058	1427.198	3.559	0.630	4.189	0.784	2.729	27.854	14272	35.590	6.300	41.890	7.840	27.290	278.540
150	original	1	60x8 weeks	Ag80	MWF	VAC50	50	All	2.973	0.007	0.001	0.009	0.002	0.006	0.058	1427.198	3.559	0.630	4.189	0.784	2.729	27.854	14272	35.590	6.300	41.890	7.840	27.290	278.540
150	original	1	60x8 weeks	Ag80	MWF	VAC50	50	All	2.973	0.007	0.001	0.009	0.002	0.006	0.058	1427.198	3.559	0.630	4.189	0.784	2.729	27.854	14272	35.590	6.300	41.890	7.840	27.290	278.540
150	original	1.2	60x8 weeks	Ag80	MWF	VAC50	50	All	2.971	0.008	0.002	0.009	0.002	0.006	0.058	1426.045	3.714	0.800	4.514	0.840	2.972	27.622	14260	37.140	8.000	45.140	8.400	29.720	276.220
150	original	1.2	60x8 weeks	Ag80	MWF	VAC50	50	All	2.971	0.008	0.002	0.009	0.002	0.006	0.058	1426.045	3.714	0.800	4.514	0.840	2.972	27.622	14260	37.140	8.000	45.140	8.400	29.720	276.220
150	original	1.2	60x8 weeks	Ag80	MWF	VAC50	50	All	2.971	0.008	0.002	0.009	0.002	0.006	0.058	1426.045	3.714	0.800	4.514	0.840	2.972	27.622	14260	37.140	8.000	45.140	8.400	29.720	276.220
150	alpha	0.6	60x8 weeks	Ag80	MWF	VAC50	50	All	2.965	0.008	0.002	0.010	0.000	0.009	0.058	1423.049	3.657	0.930	4.587	0.222	4.497	27.816	14230	36.570	9.300	45.870	2.220	44.970	278.160
150	alpha	0.6	60x8 weeks	Ag80	MWF	VAC50	50	All	2.965	0.008	0.002	0.010	0.000	0.009	0.058	1423.049	3.657	0.930	4.587	0.222	4.497	27.816	14230	36.570	9.300	45.870	2.220	44.970	278.160
150	alpha	0.6	60x8 weeks	Ag80	MWF	VAC50	50	All	2.965	0.008	0.002	0.010	0.000	0.009	0.058	1423.049	3.657	0.930	4.587	0.222	4.497	27.816	14230	36.570	9.300	45.870	2.220	44.970	278.160
150	alpha	1	60x8 weeks	Ag80	MWF	VAC50	50	All	2.958	0.008	0.003	0.011	0.001	0.011	0.058	1420.063	3.645	1.676	5.321	0.274	5.231	27.612	14201	36.450	16.760	53.210	2.740	52.310	276.120
150	alpha	1	60x8 weeks	Ag80	MWF	VAC50	50	All	2.958	0.008	0.003	0.011	0.001	0.011	0.058	1420.063	3.645	1.676	5.321	0.274	5.231	27.612	14201	36.450	16.760	53.210	2.740	52.310	276.120

150	alpha	1	60x8 weeks	Ag80	MWF	VAC50	50	All	2.958	0.008	0.003	0.011	0.001	0.011	0.058	1420.063	3.645	1.676	5.321	0.274	5.231	27.612	14201	36.450	16.760	53.210	2.740	52.310	276.120
150	alpha	1.2	60x8 weeks	Ag80	MWF	VAC50	50	All	2.956	0.007	0.004	0.012	0.001	0.012	0.057	1418.696	3.505	2.105	5.610	0.263	5.574	27.467	14187	35.050	21.050	56.100	2.630	55.740	274.670
150	alpha	1.2	60x8 weeks	Ag80	MWF	VAC50	50	All	2.956	0.007	0.004	0.012	0.001	0.012	0.057	1418.696	3.505	2.105	5.610	0.263	5.574	27.467	14187	35.050	21.050	56.100	2.630	55.740	274.670
150	alpha	1.2	60x8 weeks	Ag80	MWF	VAC50	50	All	2.956	0.007	0.004	0.012	0.001	0.012	0.057	1418.696	3.505	2.105	5.610	0.263	5.574	27.467	14187	35.050	21.050	56.100	2.630	55.740	274.670
150	delta	0.6	60x8 weeks	Ag80	MWF	VAC50	50	All	2.946	0.008	0.006	0.014	0.001	0.014	0.057	1414.269	4.016	2.806	6.822	0.425	6.685	27.217	14143	40.160	28.060	68.220	4.250	66.850	272.170
150	delta	0.6	60x8 weeks	Ag80	MWF	VAC50	50	All	2.946	0.008	0.006	0.014	0.001	0.014	0.057	1414.269	4.016	2.806	6.822	0.425	6.685	27.217	14143	40.160	28.060	68.220	4.250	66.850	272.170
150	delta	0.6	60x8 weeks	Ag80	MWF	VAC50	50	All	2.946	0.008	0.006	0.014	0.001	0.014	0.057	1414.269	4.016	2.806	6.822	0.425	6.685	27.217	14143	40.160	28.060	68.220	4.250	66.850	272.170
150	delta	1	60x8 weeks	Ag80	MWF	VAC50	50	All	2.905	0.008	0.017	0.025	0.002	0.024	0.054	1394.556	3.859	7.928	11.787	0.806	11.624	25.912	13946	38.590	79.280	117.870	8.060	116.240	259.120
150	delta	1	60x8 weeks	Ag80	MWF	VAC50	50	All	2.905	0.008	0.017	0.025	0.002	0.024	0.054	1394.556	3.859	7.928	11.787	0.806	11.624	25.912	13946	38.590	79.280	117.870	8.060	116.240	259.120
150	delta	1	60x8 weeks	Ag80	MWF	VAC50	50	All	2.905	0.008	0.017	0.025	0.002	0.024	0.054	1394.556	3.859	7.928	11.787	0.806	11.624	25.912	13946	38.590	79.280	117.870	8.060	116.240	259.120
150	delta	1.2	60x8 weeks	Ag80	MWF	VAC50	50	All	2.872	0.008	0.025	0.033	0.002	0.033	0.052	1378.456	3.636	12.198	15.834	1.096	15.661	24.824	13785	36.360	121.980	158.340	10.960	156.610	248.240
150	delta	1.2	60x8 weeks	Ag80	MWF	VAC50	50	All	2.872	0.008	0.025	0.033	0.002	0.033	0.052	1378.456	3.636	12.198	15.834	1.096	15.661	24.824	13785	36.360	121.980	158.340	10.960	156.610	248.240
150	delta	1.2	60x8 weeks	Ag80	MWF	VAC50	50	All	2.872	0.008	0.025	0.033	0.002	0.033	0.052	1378.456	3.636	12.198	15.834	1.096	15.661	24.824	13785	36.360	121.980	158.340	10.960	156.610	248.240
150	original	0.6	60x8 weeks	Ag80	MWF	VAC50	75	All	2.981	0.006	0.000	0.007	0.002	0.003	0.058	1431.055	3.077	0.191	3.268	0.916	1.498	28.047	14311	30.770	1.910	32.680	9.160	14.980	280.470
150	original	0.6	60x8 weeks	Ag80	MWF	VAC50	75	All	2.981	0.006	0.000	0.007	0.002	0.003	0.058	1431.055	3.077	0.191	3.268	0.916	1.498	28.047	14311	30.770	1.910	32.680	9.160	14.980	280.470
150	original	0.6	60x8 weeks	Ag80	MWF	VAC50	75	All	2.981	0.006	0.000	0.007	0.002	0.003	0.058	1431.055	3.077	0.191	3.268	0.916	1.498	28.047	14311	30.770	1.910	32.680	9.160	14.980	280.470
150	original	1	60x8 weeks	Ag80	MWF	VAC50	75	All	2.980	0.007	0.001	0.007	0.002	0.003	0.059	1430.427	3.169	0.332	3.501	0.983	1.586	28.133	14304	31.690	3.320	35.010	9.830	15.860	281.330
150	original	1	60x8 weeks	Ag80	MWF	VAC50	75	All	2.980	0.007	0.001	0.007	0.002	0.003	0.059	1430.427	3.169	0.332	3.501	0.983	1.586	28.133	14304	31.690	3.320	35.010	9.830	15.860	281.330
150	original	1	60x8 weeks	Ag80	MWF	VAC50	75	All	2.980	0.007	0.001	0.007	0.002	0.003	0.059	1430.427	3.169	0.332	3.501	0.983	1.586	28.133	14304	31.690	3.320	35.010	9.830	15.860	281.330
150	original	1.2	60x8 weeks	Ag80	MWF	VAC50	75	All	2.979	0.007	0.001	0.008	0.002	0.003	0.058	1430.021	3.219	0.389	3.608	1.059	1.607	27.992	14300	32.190	3.890	36.080	10.590	16.070	279.920
150	original	1.2	60x8 weeks	Ag80	MWF	VAC50	75	All	2.979	0.007	0.001	0.008	0.002	0.003	0.058	1430.021	3.219	0.389	3.608	1.059	1.607	27.992	14300	32.190	3.890	36.080	10.590	16.070	279.920
150	original	1.2	60x8 weeks	Ag80	MWF	VAC50	75	All	2.979	0.007	0.001	0.008	0.002	0.003	0.058	1430.021	3.219	0.389	3.608	1.059	1.607	27.992	14300	32.190	3.890	36.080	10.590	16.070	279.920
150	alpha	0.6	60x8 weeks	Ag80	MWF	VAC50	75	All	2.972	0.006	0.001	0.008	0.000	0.007	0.059	1426.756	3.070	0.530	3.600	0.177	3.499	28.210	14268	30.700	5.300	36.000	1.770	34.990	282.100
150	alpha	0.6	60x8 weeks	Ag80	MWF	VAC50	75	All	2.972	0.006	0.001	0.008	0.000	0.007	0.059	1426.756	3.070	0.530	3.600	0.177	3.499	28.210	14268	30.700	5.300	36.000	1.770	34.990	282.100
150	alpha	0.6	60x8 weeks	Ag80	MWF	VAC50	75	All	2.972	0.006	0.001	0.008	0.000	0.007	0.059	1426.756	3.070	0.530	3.600	0.177	3.499	28.210	14268	30.700	5.300	36.000	1.770	34.990	282.100
150	alpha	1	60x8 weeks	Ag80	MWF	VAC50	75	All	2.968	0.007	0.002	0.009	0.000	0.008	0.059	1424.566	3.176	0.989	4.165	0.222	4.060	28.132	14246	31.760	9.890	41.650	2.220	40.600	281.320
150	alpha	1	60x8 weeks	Ag80	MWF	VAC50	75	All	2.968	0.007	0.002	0.009	0.000	0.008	0.059	1424.566	3.176	0.989	4.165	0.222	4.060	28.132	14246	31.760	9.890	41.650	2.220	40.600	281.320
150	alpha	1	60x8 weeks	Ag80	MWF	VAC50	75	All	2.968	0.007	0.002	0.009	0.000	0.008	0.059	1424.566	3.176	0.989	4.165	0.222	4.060	28.132	14246	31.760	9.890	41.650	2.220	40.600	281.320
150	alpha	1.2	60x8 weeks	Ag80	MWF	VAC50	75	All	2.967	0.006	0.003	0.009	0.000	0.009	0.058	1424.259	3.058	1.208	4.266	0.214	4.170	27.883	14243	30.580	12.080	42.660	2.140	41.700	278.830
150	alpha	1.2	60x8 weeks	Ag80	MWF	VAC50	75	All	2.967	0.006	0.003	0.009	0.000	0.009	0.058	1424.259	3.058	1.208	4.266	0.214	4.170	27.883	14243	30.580	12.080	42.660	2.140	41.700	278.830
150	alpha	1.2	60x8 weeks	Ag80	MWF	VAC50	75	All	2.967	0.006	0.003	0.009	0.000	0.009	0.058	1424.259	3.058	1.208	4.266	0.214	4.170	27.883	14243	30.580	12.080	42.660	2.140	41.700	278.830
150	delta	0.6	60x8 weeks	Ag80	MWF	VAC50	75	All	2.959	0.007	0.003	0.011	0.001	0.011	0.057	1420.186	3.544	1.676	5.220	0.343	5.131	27.530	14202	35.440	16.760	52.200	3.430	51.310	275.300
150	delta	0.6	60x8 weeks	Ag80	MWF	VAC50	75	All	2.959	0.007	0.003	0.011	0.001	0.011	0.057	1420.186	3.544	1.676	5.220	0.343	5.131	27.530	14202	35.440	16.760	52.200	3.430	51.310	275.300
150	delta	0.6	60x8 weeks	Ag80	MWF	VAC50	75	All	2.959	0.007	0.003	0.011	0.001	0.011	0.057	1420.186	3.544	1.676	5.220	0.343	5.131	27.530	14202	35.440	16.760	52.200	3.430	51.310	275.300
150	delta	1	60x8 weeks	Ag80	MWF	VAC50	75	All	2.939	0.008	0.009	0.016	0.001	0.016	0.056	1410.700	3.623	4.160	7.783	0.524	7.571	26.932	14107	36.230	41.600	77.830	5.240	75.710	269.320
150	delta	1	60x8 weeks	Ag80	MWF	VAC50	75	All	2.939	0.008	0.009	0.016	0.001	0.016	0.056	1410.700	3.623	4.160	7.783	0.524	7.571	26.932	14107	36.230	41.600	77.830	5.240	75.710	269.320
150	delta	1	60x8 weeks	Ag80	MWF	VAC50	75	All	2.939	0.008	0.009	0.016	0.001	0.016	0.056	1410.700	3.623	4.160	7.783	0.524	7.571	26.932	14107	36.230	41.600	77.830	5.240	75.710	269.320
150	delta	1.2	60x8 weeks	Ag80	MWF	VAC50	75	All	2.923	0.007	0.013	0.020	0.001	0.020	0.055	1402.851	3.517	6.146	9.663	0.674	9.505	26.531	14029	35.170	61.460	96.630	6.740	95.050	265.310
150	delta	1.2	60x8 weeks	Ag80	MWF	VAC50	75	All	2.923	0.007	0.013	0.020	0.001	0.020	0.055	1402.851	3.517	6.146	9.663	0.674	9.505	26.531	14029	35.170	61.460	96.630	6.740	95.050	265.310
150	delta	1.2	60x8 weeks	Ag80	MWF	VAC50	75	All	2.923	0.007	0.013	0.020	0.001	0.020	0.055	1402.851	3.517	6.146	9.663	0.674	9.505	26.531	14029	35.170	61.460	96.630	6.740	95.050	265.310
150	original	0.6	60x8 weeks	Ag80	MWF	VAC50	100	All	2.989	0.005	0.000	0.005	0.002	0.001	0.058	1434.622	2.547	0.032	2.579	1.086	0.333	27.972	14346	25.470	0.320	25.790	10.860	3.330	279.720
150	original	0.6	60x8 weeks	Ag80	MWF	VAC50	100	All	2.989	0.005	0.000	0.005	0.002	0.001	0.058	1434.622	2.547	0.032	2.579	1.086	0.333	27.972	14346	25.470	0.320	25.790	10.860	3.330	279.720
150	original	0.6	60x8 weeks	Ag80	MWF	VAC50	100	All	2.989	0.005	0.000	0.005	0.002	0.001	0.058	1434.622	2.547	0.032	2.579	1.086	0.333	27.972	14346	25.470	0.320	25.790	10.860	3.330	279.720
150	original	1	60x8 weeks	Ag80	MWF	VAC50	100	All	2.989	0.005	0.000	0.005	0.002	0.001	0.059	1434.493	2.514	0.057	2.571	1.082	0.336	28.274	14345	25.140	0.570	25.710	10.820	3.360	282.740
150	original	1	60x8 weeks	Ag80	MWF	VAC50	100	All	2.989	0.005	0.000	0.005	0.002	0.001	0.059	1434.493	2.514	0.057	2.571	1.082	0.336	28.274	14345	25.140	0.570	25.710	10.820	3.360	282.740
150	original	1	60x8 weeks	Ag80	MWF	VAC50	100	All	2.989	0.005	0.000	0.005	0.002	0.001	0.059	1434.493	2.514	0.057	2.571	1.082	0.336	28.274	14345	25.140	0.570	25.710	10.820	3.360	282.740
150	original	1.2	60x8 weeks	Ag80	MWF	VAC50	100	All	2.988	0.005	0.000	0.006	0.002	0.001	0.059	1434.353	2.609	0.068	2.677	1.131	0.347	28.290	14344	26.090	0.680	26.770	11.310	3.470	282.900
150	original	1.2	60x8 weeks	Ag80	MWF	VAC50	100	All	2.988	0.005	0.000	0.006	0.002	0.001	0.059	1434.353	2.609	0.068	2.677	1.131	0.347	28.290	14344	26.090	0.680	26.770	11.310	3.470	282.900
150	original	1.2	60x8 weeks	Ag80	MWF	VAC50	100	All	2.988	0.005	0.000	0.006	0.002	0.001	0.059	1434.353	2.609	0.068	2.677	1.131	0.347	28.290	14344	26.090	0.680	26.770	11.310	3.470	282.900
150	alpha	0.6	60x8 weeks	Ag80	MWF	VAC50	100	All	2.979	0.005	0.001	0.006	0.000	0.005	0.059	1429.977	2.481	0.261	2.742	0.142	2.630	28.258	14300	24.810	2.610	27.420	1.420	26.300	282.580
150	alpha	0.6	60x8 weeks	Ag80	MWF	VAC50	100	All	2.979	0.005	0.001	0.006	0.000	0.005	0.059	1429.977	2.481	0.261	2.742	0.142	2.630	28.258	14300	24.810	2.610	27.420	1.420	26.300	282.580
150	alpha	0.6	60x8 weeks	Ag80	MWF	VAC50	100	All	2.979	0.005	0.001	0.006	0.000	0.005	0.059	1429.977	2.481	0.261	2.742	0.142	2.630	28.258	14300	24.810	2.610	27.420	1.420	26.300	282.580
150	alpha	1	60x8 weeks	Ag80	MWF	VAC50	100	All	2.978	0.005	0.001	0.006	0.000	0.006	0.058	1429.291	2.466	0.440	2.906	0.177	2.780	27.759	14293	24.660	4.400	29.060	1.770	27.800	277.590
150	alpha	1	60x8 weeks	Ag80	MWF	VAC50	100	All	2.978	0.005	0.001	0.006	0.000	0.006	0.058	1429.291	2.466	0.440	2.906	0.177	2.780	27.759	14293	24.660	4.400	29.060	1.770	27.800	277.590
150	alpha	1	60x8 weeks	Ag80	MWF	VAC50	100	All	2.978	0.005	0.001	0.006	0.000	0.006	0.058	1429.291	2.466	0.440	2.906	0.177	2.780	27.759	14293	24.660	4.400	29.060	1.770	27.800	277.590
150	alpha	1.2	60x8 weeks	Ag80	MWF	VAC50	100	All	2.976	0.005	0.001	0.007	0.000	0.006	0.058	1428.438	2.530	0.621	3.151	0.181	3.030	27.692	14284	25.300	6.210	31.510	1.810	30.300	276.920
150	alpha	1.2	60x8 weeks	Ag80	MWF	VAC50	100	All	2.976	0.005	0.001	0.007	0.000	0.006	0.058	1428.438	2.530	0.621	3.151	0.181	3.030	27.692	14284	25.300	6.210	31.510	1.810	30.300	276.920
150	alpha	1.2	60x8 weeks	Ag80	MWF	VAC50	100	All	2.976	0.005	0.001	0.007	0.000	0.006	0.058	1428.438	2.530	0.621	3.151	0.181	3.030	27.692	14284	25.300	6.210	31.510	1.810	30.300	276.920
150	delta	0.6	60x8 weeks	Ag80	MWF	VAC50	100	All	2.967	0.007	0.002	0.009	0.001	0.009	0.058	1424.218	3.252	0.961	4.213	0.249	4.121	27.845	14242	32.520	9.610	42.130	2.490	41.210	278.450
150	delta	0.6	60x8 weeks	Ag80	MWF	VAC50	100	All	2.967	0.007	0.002	0.009	0.001	0.009	0.058	1424.218	3.252	0.961	4.213	0.249	4.121	27.845	14242	32.520	9.610	42.130	2.490	41.210	278.450
150	delta	0.6	60x8 weeks	Ag80	MWF	VAC50	100	All	2.967	0.007	0.002	0.009	0.001	0.009	0.058	1424.218	3.252	0.961	4.213	0.249	4.121	27.845	14242	32.520	9.610	42.130	2.490	41.210	278.450
150	delta	1	60x8 weeks	Ag80	MWF	VAC50	100	All	2.958	0.007	0.004	0.011	0.001	0.011	0.058	1419.679	3.342	2.049	5.391	0.345	5.311	27.646	14197	33.420	20.490	53.910	3.450	53.110	276.460
150	delta	1	60x8 weeks	Ag80	MWF	VAC50	100	All	2.958	0.007	0.004	0.011	0.001	0.011	0.058	1419.679	3.342	2.049	5.391	0.345	5.311	27.646	14197	33.420	20.490	53.910	3.450	53.110	276.460
150	delta	1	60x8 weeks	Ag80	MWF	VAC50	100	All	2.958	0.007	0.004	0.011	0.001	0.011	0.058	1419.679	3.342	2.049	5.391	0.345	5.311	27.646	14197	33.420	20.490	53.910	3.450	53.110	276.460
150	delta	1.2	60x8 weeks	Ag80	MWF	VAC50	100	All	2.955	0.007	0.005	0.012	0.001	0.012	0.057	1418.409	3.210	2.546	5.756	0.368	5.624	27.383	14184	32.100	25.460	57.560	3.680	56.240	273.830
150	delta	1.2	60x8 weeks	Ag80	MWF	VAC50	100	All	2.955	0.007	0.005	0.012	0.001	0.012	0.057	1418.409	3.210	2.546	5.756	0.368	5.624	27.383	14184	32.100	25.460	57.560	3.680	56.240	273.830
150	delta	1.2	60x8 weeks	Ag80	MWF	VAC50	100	All	2.955	0.007	0.005	0.012	0.001	0.012	0.057	1418.409	3.210	2.546	5.756	0.368	5.624	27.383	14184	32.100	25.460	57.560	3.680	56.240	273.830
150	original	0.6	60x8 weeks	Ag80	M	NOVAC	0	All	0.987	0.010	0.005	0.015	0.004	0.007	0.019	473.963	4.780	2.467	7.247	2.012	3.506	8.906	4740	47.800	24.670	72.470	20.120	35.060	89.060
150	original	0.6	60x8 weeks	Ag80	M	NOVAC	0	All	0.987	0.010	0.005	0.015	0.004	0.007	0.019	473.963	4.780	2.467	7.247	2.012	3.506	8.906	4740	47.800	24.670	72.470	20.120	35.060	89.060
150	original	0.6	60x8 weeks	Ag80	M	NOVAC	0	All	0.987	0.010	0.005	0.015	0.004	0.007	0.019	473.963	4.780	2.467	7.247	2.012	3.506	8.906	4740	47.800	24.670	72.470	20.120	35.060	89.060
150	original	1	60x8 weeks	Ag80	M	NOVAC	0	All	0.981	0.010	0.012	0.022	0.006	0.011	0.018	471.030	4.661	5.952	10.613	2.923	5.257	8.834	4710	46.610	59.520	106.130	29.230	52.570	88.340

150	original	1	60x8 weeks	Ag80	M	NOVAC	0	All	0.981	0.010	0.012	0.022	0.006	0.011	0.018	471.030	4.661	5.952	10.613	2.923	5.257	8.834	4710	46.610	59.520	106.130	29.230	52.570	88.340
150	original	1	60x8 weeks	Ag80	M	NOVAC	0	All	0.981	0.010	0.012	0.022	0.006	0.011	0.018	471.030	4.661	5.952	10.613	2.923	5.257	8.834	4710	46.610	59.520	106.130	29.230	52.570	88.340
150	original	1.2	60x8 weeks	Ag80	M	NOVAC	0	All	0.978	0.009	0.017	0.026	0.007	0.013	0.018	469.266	4.530	8.160	12.690	3.443	6.365	8.728	4693	45.300	81.600	126.900	34.430	63.650	87.280
150	original	1.2	60x8 weeks	Ag80	M	NOVAC	0	All	0.978	0.009	0.017	0.026	0.007	0.013	0.018	469.266	4.530	8.160	12.690	3.443	6.365	8.728	4693	45.300	81.600	126.900	34.430	63.650	87.280
150	original	1.2	60x8 weeks	Ag80	M	NOVAC	0	All	0.978	0.009	0.017	0.026	0.007	0.013	0.018	469.266	4.530	8.160	12.690	3.443	6.365	8.728	4693	45.300	81.600	126.900	34.430	63.650	87.280
150	alpha	0.6	60x8 weeks	Ag80	M	NOVAC	0	All	0.976	0.009	0.016	0.025	0.008	0.015	0.018	468.296	4.509	7.656	12.165	3.616	7.120	8.733	4683	45.090	76.560	121.650	36.160	71.200	87.330
150	alpha	0.6	60x8 weeks	Ag80	M	NOVAC	0	All	0.976	0.009	0.016	0.025	0.008	0.015	0.018	468.296	4.509	7.656	12.165	3.616	7.120	8.733	4683	45.090	76.560	121.650	36.160	71.200	87.330
150	alpha	0.6	60x8 weeks	Ag80	M	NOVAC	0	All	0.976	0.009	0.016	0.025	0.008	0.015	0.018	468.296	4.509	7.656	12.165	3.616	7.120	8.733	4683	45.090	76.560	121.650	36.160	71.200	87.330
150	alpha	1	60x8 weeks	Ag80	M	NOVAC	0	All	0.941	0.008	0.051	0.059	0.018	0.035	0.016	451.699	3.837	24.655	28.492	8.742	16.843	7.476	4517	38.370	246.550	284.920	87.420	168.430	74.760
150	alpha	1	60x8 weeks	Ag80	M	NOVAC	0	All	0.941	0.008	0.051	0.059	0.018	0.035	0.016	451.699	3.837	24.655	28.492	8.742	16.843	7.476	4517	38.370	246.550	284.920	87.420	168.430	74.760
150	alpha	1	60x8 weeks	Ag80	M	NOVAC	0	All	0.941	0.008	0.051	0.059	0.018	0.035	0.016	451.699	3.837	24.655	28.492	8.742	16.843	7.476	4517	38.370	246.550	284.920	87.420	168.430	74.760
150	alpha	1.2	60x8 weeks	Ag80	M	NOVAC	0	All	0.919	0.007	0.071	0.078	0.025	0.047	0.014	441.093	3.346	34.186	37.532	11.779	22.628	6.743	4411	33.460	341.860	375.320	117.790	226.280	67.430
150	alpha	1.2	60x8 weeks	Ag80	M	NOVAC	0	All	0.919	0.007	0.071	0.078	0.025	0.047	0.014	441.093	3.346	34.186	37.532	11.779	22.628	6.743	4411	33.460	341.860	375.320	117.790	226.280	67.430
150	alpha	1.2	60x8 weeks	Ag80	M	NOVAC	0	All	0.919	0.007	0.071	0.078	0.025	0.047	0.014	441.093	3.346	34.186	37.532	11.779	22.628	6.743	4411	33.460	341.860	375.320	117.790	226.280	67.430
150	delta	0.6	60x8 weeks	Ag80	M	NOVAC	0	All	0.914	0.007	0.076	0.083	0.028	0.049	0.014	438.580	3.254	36.355	39.609	13.310	23.300	6.689	4386	32.540	363.550	396.090	133.100	233.000	66.890
150	delta	0.6	60x8 weeks	Ag80	M	NOVAC	0	All	0.914	0.007	0.076	0.083	0.028	0.049	0.014	438.580	3.254	36.355	39.609	13.310	23.300	6.689	4386	32.540	363.550	396.090	133.100	233.000	66.890
150	delta	0.6	60x8 weeks	Ag80	M	NOVAC	0	All	0.914	0.007	0.076	0.083	0.028	0.049	0.014	438.580	3.254	36.355	39.609	13.310	23.300	6.689	4386	32.540	363.550	396.090	133.100	233.000	66.890
150	delta	1	60x8 weeks	Ag80	M	NOVAC	0	All	0.866	0.005	0.112	0.116	0.040	0.071	0.009	415.604	2.197	53.623	55.820	19.318	34.247	4.476	4156	21.970	536.230	558.200	193.180	342.470	44.760
150	delta	1	60x8 weeks	Ag80	M	NOVAC	0	All	0.866	0.005	0.112	0.116	0.040	0.071	0.009	415.604	2.197	53.623	55.820	19.318	34.247	4.476	4156	21.970	536.230	558.200	193.180	342.470	44.760
150	delta	1	60x8 weeks	Ag80	M	NOVAC	0	All	0.866	0.005	0.112	0.116	0.040	0.071	0.009	415.604	2.197	53.623	55.820	19.318	34.247	4.476	4156	21.970	536.230	558.200	193.180	342.470	44.760
150	delta	1.2	60x8 weeks	Ag80	M	NOVAC	0	All	0.861	0.004	0.116	0.121	0.041	0.074	0.009	413.358	2.056	55.815	57.871	19.856	35.715	4.167	4134	20.560	558.150	578.710	198.560	357.150	41.670
150	delta	1.2	60x8 weeks	Ag80	M	NOVAC	0	All	0.861	0.004	0.116	0.121	0.041	0.074	0.009	413.358	2.056	55.815	57.871	19.856	35.715	4.167	4134	20.560	558.150	578.710	198.560	357.150	41.670
150	delta	1.2	60x8 weeks	Ag80	M	NOVAC	0	All	0.861	0.004	0.116	0.121	0.041	0.074	0.009	413.358	2.056	55.815	57.871	19.856	35.715	4.167	4134	20.560	558.150	578.710	198.560	357.150	41.670
150	original	0.6	60x8 weeks	Ag80	M	VAC95	25	All	0.991	0.008	0.003	0.010	0.003	0.005	0.019	475.851	3.708	1.323	5.031	1.398	2.348	9.149	4759	37.080	13.230	50.310	13.980	23.480	91.490
150	original	0.6	60x8 weeks	Ag80	M	VAC95	25	All	0.991	0.008	0.003	0.010	0.003	0.005	0.019	475.851	3.708	1.323	5.031	1.398	2.348	9.149	4759	37.080	13.230	50.310	13.980	23.480	91.490
150	original	0.6	60x8 weeks	Ag80	M	VAC95	25	All	0.991	0.008	0.003	0.010	0.003	0.005	0.019	475.851	3.708	1.323	5.031	1.398	2.348	9.149	4759	37.080	13.230	50.310	13.980	23.480	91.490
150	original	1	60x8 weeks	Ag80	M	VAC95	25	All	0.989	0.008	0.006	0.013	0.004	0.006	0.019	474.869	3.622	2.743	6.365	1.718	2.971	9.171	4749	36.220	27.430	63.650	17.180	29.710	91.710
150	original	1	60x8 weeks	Ag80	M	VAC95	25	All	0.989	0.008	0.006	0.013	0.004	0.006	0.019	474.869	3.622	2.743	6.365	1.718	2.971	9.171	4749	36.220	27.430	63.650	17.180	29.710	91.710
150	original	1	60x8 weeks	Ag80	M	VAC95	25	All	0.989	0.008	0.006	0.013	0.004	0.006	0.019	474.869	3.622	2.743	6.365	1.718	2.971	9.171	4749	36.220	27.430	63.650	17.180	29.710	91.710
150	original	1.2	60x8 weeks	Ag80	M	VAC95	25	All	0.988	0.008	0.008	0.015	0.004	0.007	0.019	474.112	3.632	3.650	7.282	1.984	3.474	9.050	4741	36.320	36.500	72.820	19.840	34.740	90.500
150	original	1.2	60x8 weeks	Ag80	M	VAC95	25	All	0.988	0.008	0.008	0.015	0.004	0.007	0.019	474.112	3.632	3.650	7.282	1.984	3.474	9.050	4741	36.320	36.500	72.820	19.840	34.740	90.500
150	original	1.2	60x8 weeks	Ag80	M	VAC95	25	All	0.988	0.008	0.008	0.015	0.004	0.007	0.019	474.112	3.632	3.650	7.282	1.984	3.474	9.050	4741	36.320	36.500	72.820	19.840	34.740	90.500
150	alpha	0.6	60x8 weeks	Ag80	M	VAC95	25	All	0.986	0.008	0.007	0.015	0.005	0.008	0.019	473.364	3.601	3.470	7.071	2.166	3.973	9.006	4734	36.010	34.700	70.710	21.660	39.730	90.060
150	alpha	0.6	60x8 weeks	Ag80	M	VAC95	25	All	0.986	0.008	0.007	0.015	0.005	0.008	0.019	473.364	3.601	3.470	7.071	2.166	3.973	9.006	4734	36.010	34.700	70.710	21.660	39.730	90.060
150	alpha	0.6	60x8 weeks	Ag80	M	VAC95	25	All	0.986	0.008	0.007	0.015	0.005	0.008	0.019	473.364	3.601	3.470	7.071	2.166	3.973	9.006	4734	36.010	34.700	70.710	21.660	39.730	90.060
150	alpha	1	60x8 weeks	Ag80	M	VAC95	25	All	0.975	0.007	0.019	0.026	0.008	0.015	0.018	467.929	3.432	9.137	12.569	3.769	7.303	8.553	4679	34.320	91.370	125.690	37.690	73.030	85.530
150	alpha	1	60x8 weeks	Ag80	M	VAC95	25	All	0.975	0.007	0.019	0.026	0.008	0.015	0.018	467.929	3.432	9.137	12.569	3.769	7.303	8.553	4679	34.320	91.370	125.690	37.690	73.030	85.530
150	alpha	1	60x8 weeks	Ag80	M	VAC95	25	All	0.975	0.007	0.019	0.026	0.008	0.015	0.018	467.929	3.432	9.137	12.569	3.769	7.303	8.553	4679	34.320	91.370	125.690	37.690	73.030	85.530
150	alpha	1.2	60x8 weeks	Ag80	M	VAC95	25	All	0.965	0.007	0.029	0.036	0.011	0.021	0.018	463.138	3.210	14.071	17.281	5.252	10.189	8.421	4631	32.100	140.710	172.810	52.520	101.890	84.210
150	alpha	1.2	60x8 weeks	Ag80	M	VAC95	25	All	0.965	0.007	0.029	0.036	0.011	0.021	0.018	463.138	3.210	14.071	17.281	5.252	10.189	8.421	4631	32.100	140.710	172.810	52.520	101.890	84.210
150	alpha	1.2	60x8 weeks	Ag80	M	VAC95	25	All	0.965	0.007	0.029	0.036	0.011	0.021	0.018	463.138	3.210	14.071	17.281	5.252	10.189	8.421	4631	32.100	140.710	172.810	52.520	101.890	84.210
150	delta	0.6	60x8 weeks	Ag80	M	VAC95	25	All	0.962	0.007	0.032	0.039	0.012	0.023	0.017	461.611	3.243	15.354	18.597	5.943	10.816	8.194	4616	32.430	153.540	185.970	59.430	108.160	81.940
150	delta	0.6	60x8 weeks	Ag80	M	VAC95	25	All	0.962	0.007	0.032	0.039	0.012	0.023	0.017	461.611	3.243	15.354	18.597	5.943	10.816	8.194	4616	32.430	153.540	185.970	59.430	108.160	81.940
150	delta	0.6	60x8 weeks	Ag80	M	VAC95	25	All	0.962	0.007	0.032	0.039	0.012	0.023	0.017	461.611	3.243	15.354	18.597	5.943	10.816	8.194	4616	32.430	153.540	185.970	59.430	108.160	81.940
150	delta	1	60x8 weeks	Ag80	M	VAC95	25	All	0.914	0.005	0.075	0.080	0.027	0.048	0.013	438.952	2.340	36.128	38.468	12.811	23.081	6.456	4390	23.400	361.280	384.680	128.110	230.810	64.560
150	delta	1	60x8 weeks	Ag80	M	VAC95	25	All	0.914	0.005	0.075	0.080	0.027	0.048	0.013	438.952	2.340	36.128	38.468	12.811	23.081	6.456	4390	23.400	361.280	384.680	128.110	230.810	64.560
150	delta	1	60x8 weeks	Ag80	M	VAC95	25	All	0.914	0.005	0.075	0.080	0.027	0.048	0.013	438.952	2.340	36.128	38.468	12.811	23.081	6.456	4390	23.400	361.280	384.680	128.110	230.810	64.560
150	delta	1.2	60x8 weeks	Ag80	M	VAC95	25	All	0.898	0.004	0.088	0.093	0.031	0.056	0.012	431.020	2.074	42.451	44.525	14.857	26.956	5.769	4310	20.740	424.510	445.250	148.570	269.560	57.690
150	delta	1.2	60x8 weeks	Ag80	M	VAC95	25	All	0.898	0.004	0.088	0.093	0.031	0.056	0.012	431.020	2.074	42.451	44.525	14.857	26.956	5.769	4310	20.740	424.510	445.250	148.570	269.560	57.690
150	delta	1.2	60x8 weeks	Ag80	M	VAC95	25	All	0.898	0.004	0.088	0.093	0.031	0.056	0.012	431.020	2.074	42.451	44.525	14.857	26.956	5.769	4310	20.740	424.510	445.250	148.570	269.560	57.690
150	original	0.6	60x8 weeks	Ag80	M	VAC95	50	All	0.995	0.005	0.001	0.007	0.002	0.003	0.020	477.412	2.584	0.560	3.144	0.909	1.402	9.463	4774	25.840	5.600	31.440	9.090	14.020	94.630
150	original	0.6	60x8 weeks	Ag80	M	VAC95	50	All	0.995	0.005	0.001	0.007	0.002	0.003	0.020	477.412	2.584	0.560	3.144	0.909	1.402	9.463	4774	25.840	5.600	31.440	9.090	14.020	94.630
150	original	0.6	60x8 weeks	Ag80	M	VAC95	50	All	0.995	0.005	0.001	0.007	0.002	0.003	0.020	477.412	2.584	0.560	3.144	0.909	1.402	9.463	4774	25.840	5.600	31.440	9.090	14.020	94.630
150	original	1	60x8 weeks	Ag80	M	VAC95	50	All	0.994	0.005	0.002	0.007	0.002	0.003	0.020	477.167	2.531	0.924	3.455	0.980	1.555	9.463	4772	25.310	9.240	34.550	9.800	15.550	94.630
150	original	1	60x8 weeks	Ag80	M	VAC95	50	All	0.994	0.005	0.002	0.007	0.002	0.003	0.020	477.167	2.531	0.924	3.455	0.980	1.555	9.463	4772	25.310	9.240	34.550	9.800	15.550	94.630
150	original	1	60x8 weeks	Ag80	M	VAC95	50	All	0.994	0.005	0.002	0.007	0.002	0.003	0.020	477.167	2.531	0.924	3.455	0.980	1.555	9.463	4772	25.310	9.240	34.550	9.800	15.550	94.630
150	original	1.2	60x8 weeks	Ag80	M	VAC95	50	All	0.994	0.005	0.003	0.008	0.002	0.003	0.019	477.056	2.531	1.231	3.762	1.086	1.619	9.088	4771	25.310	12.310	37.620	10.860	16.190	90.880
150	original	1.2	60x8 weeks	Ag80	M	VAC95	50	All	0.994	0.005	0.003	0.008	0.002	0.003	0.019	477.056	2.531	1.231	3.762	1.086	1.619	9.088	4771	25.310	12.310	37.620	10.860	16.190	90.880
150	original	1.2	60x8 weeks	Ag80	M	VAC95	50	All	0.994	0.005	0.003	0.008	0.002	0.003	0.019	477.056	2.531	1.231	3.762	1.086	1.619	9.088	4771	25.310	12.310	37.620	10.860	16.190	90.880
150	alpha	0.6	60x8 weeks	Ag80	M	VAC95	50	All	0.992	0.005	0.003	0.008	0.003	0.005	0.019	476.199	2.602	1.404	4.006	1.217	2.226	9.333	4762	26.020	14.040	40.060	12.170	22.260	93.330
150	alpha	0.6	60x8 weeks	Ag80	M	VAC95	50	All	0.992	0.005	0.003	0.008	0.003	0.005	0.019	476.199	2.602	1.404	4.006	1.217	2.226	9.333	4762	26.020	14.040	40.060	12.170	22.260	93.330
150	alpha	0.6	60x8 weeks	Ag80	M	VAC95	50	All	0.992	0.005	0.003	0.008	0.003	0.005	0.019	476.199	2.602	1.404	4.006	1.217	2.226	9.333	4762	26.020	14.040	40.060	12.170	22.260	93.330
150	alpha	1	60x8 weeks	Ag80	M	VAC95	50	All	0.989	0.005	0.007	0.012	0.004	0.007	0.019	474.521	2.592	3.148	5.740	1.696	3.360	9.243	4745	25.920	31.480	57.400	16.960	33.600	92.430
150	alpha	1	60x8 weeks	Ag80	M	VAC95	50	All	0.989	0.005	0.007	0.012	0.004	0.007	0.019	474.521	2.592	3.148	5.740	1.696	3.360	9.243	4745	25.920	31.480	57.400	16.960	33.600	92.430
150	alpha	1	60x8 weeks	Ag80	M	VAC95	50	All	0.989	0.005	0.007	0.012	0.004	0.007	0.019	474.521	2.592	3.148	5.740	1.696	3.360	9.243	4745	25.920	31.480	57.400	16.960	33.600	92.430
150	alpha	1.2	60x8 weeks	Ag80	M	VAC95	50	All	0.988	0.005	0.008	0.013	0.004	0.008	0.019	474.013	2.421	4.030	6.451	1.908	3.666	9.268	4740	24.210	40.300	64.510	19.080	36.660	92.680
150	alpha	1.2	60x8 weeks	Ag80	M	VAC95	50	All	0.988	0.005	0.008	0.013	0.004	0.008	0.019	474.013	2.421	4.030	6.451	1.908	3.666	9.268	4740	24.210	40.300	64.510	19.080	36.660	92.680
150	alpha	1.2	60x8 weeks	Ag80	M	VAC95	50	All	0.988	0.005	0.008	0.013	0.004	0.008	0.019	474.013	2.421	4.030	6.451	1.908	3.666	9.268	4740	24.210	40.300	64.510	19.080	36.660	92.680
150	delta	0.6	60x8 weeks	Ag80	M	VAC95	50	All	0.985	0.006	0.010	0.016	0.005	0.009	0.019	472.601	2.644	4.976	7.620	2.408	4.379	8.912	4726	26.440	49.760	76.200	24.080	43.790	89.120
150	delta	0.6	60x8 weeks	Ag80	M	VAC95	50	All	0.985	0.006	0.010	0.016	0.005	0.009	0.019	472.601	2.644	4.976	7.620	2.408	4.379	8.912	4726	26.440	49.760	76.200	24.080	43.790	89.120
150	delta	0.6	60x8 weeks	Ag80	M	VAC95	50	All	0.985	0.006	0.010	0.016	0.005	0.009	0.019	472.601	2.644	4.976	7.620	2.408	4.379	8.912	4726	26.440	49.760	76.200	24.080	43.790	89.120

150	delta	1	60x8 weeks	Ag80	M	VAC95	50	All	0.965	0.005	0.030	0.035	0.011	0.020	0.017	463.279	2.310	14.315	16.625	5.417	9.632	8.358	4633	23.100	143.150	166.250	54.170	96.320	83.580
150	delta	1	60x8 weeks	Ag80	M	VAC95	50	All	0.965	0.005	0.030	0.035	0.011	0.020	0.017	463.279	2.310	14.315	16.625	5.417	9.632	8.358	4633	23.100	143.150	166.250	54.170	96.320	83.580
150	delta	1	60x8 weeks	Ag80	M	VAC95	50	All	0.965	0.005	0.030	0.035	0.011	0.020	0.017	463.279	2.310	14.315	16.625	5.417	9.632	8.358	4633	23.100	143.150	166.250	54.170	96.320	83.580
150	delta	1.2	60x8 weeks	Ag80	M	VAC95	50	All	0.956	0.004	0.039	0.044	0.014	0.026	0.016	458.680	2.131	18.841	20.972	6.812	12.272	7.819	4587	21.310	188.410	209.720	68.120	122.720	78.190
150	delta	1.2	60x8 weeks	Ag80	M	VAC95	50	All	0.956	0.004	0.039	0.044	0.014	0.026	0.016	458.680	2.131	18.841	20.972	6.812	12.272	7.819	4587	21.310	188.410	209.720	68.120	122.720	78.190
150	delta	1.2	60x8 weeks	Ag80	M	VAC95	50	All	0.956	0.004	0.039	0.044	0.014	0.026	0.016	458.680	2.131	18.841	20.972	6.812	12.272	7.819	4587	21.310	188.410	209.720	68.120	122.720	78.190
150	original	0.6	60x8 weeks	Ag80	M	VAC95	75	All	0.997	0.003	0.000	0.003	0.001	0.001	0.019	478.751	1.454	0.130	1.584	0.463	0.654	9.267	4788	14.540	1.300	15.840	4.630	6.540	92.670
150	original	0.6	60x8 weeks	Ag80	M	VAC95	75	All	0.997	0.003	0.000	0.003	0.001	0.001	0.019	478.751	1.454	0.130	1.584	0.463	0.654	9.267	4788	14.540	1.300	15.840	4.630	6.540	92.670
150	original	0.6	60x8 weeks	Ag80	M	VAC95	75	All	0.997	0.003	0.000	0.003	0.001	0.001	0.019	478.751	1.454	0.130	1.584	0.463	0.654	9.267	4788	14.540	1.300	15.840	4.630	6.540	92.670
150	original	1	60x8 weeks	Ag80	M	VAC95	75	All	0.997	0.003	0.001	0.004	0.001	0.001	0.020	478.714	1.425	0.260	1.685	0.483	0.675	9.431	4787	14.250	2.600	16.850	4.830	6.750	94.310
150	original	1	60x8 weeks	Ag80	M	VAC95	75	All	0.997	0.003	0.001	0.004	0.001	0.001	0.020	478.714	1.425	0.260	1.685	0.483	0.675	9.431	4787	14.250	2.600	16.850	4.830	6.750	94.310
150	original	1	60x8 weeks	Ag80	M	VAC95	75	All	0.997	0.003	0.001	0.004	0.001	0.001	0.020	478.714	1.425	0.260	1.685	0.483	0.675	9.431	4787	14.250	2.600	16.850	4.830	6.750	94.310
150	original	1.2	60x8 weeks	Ag80	M	VAC95	75	All	0.997	0.003	0.001	0.004	0.001	0.001	0.020	478.583	1.486	0.344	1.830	0.561	0.693	9.400	4786	14.860	3.440	18.300	5.610	6.930	94.000
150	original	1.2	60x8 weeks	Ag80	M	VAC95	75	All	0.997	0.003	0.001	0.004	0.001	0.001	0.020	478.583	1.486	0.344	1.830	0.561	0.693	9.400	4786	14.860	3.440	18.300	5.610	6.930	94.000
150	original	1.2	60x8 weeks	Ag80	M	VAC95	75	All	0.997	0.003	0.001	0.004	0.001	0.001	0.020	478.583	1.486	0.344	1.830	0.561	0.693	9.400	4786	14.860	3.440	18.300	5.610	6.930	94.000
150	alpha	0.6	60x8 weeks	Ag80	M	VAC95	75	All	0.996	0.003	0.001	0.004	0.001	0.002	0.020	478.288	1.538	0.336	1.874	0.549	1.002	9.559	4783	15.380	3.360	18.740	5.490	10.020	95.590
150	alpha	0.6	60x8 weeks	Ag80	M	VAC95	75	All	0.996	0.003	0.001	0.004	0.001	0.002	0.020	478.288	1.538	0.336	1.874	0.549	1.002	9.559	4783	15.380	3.360	18.740	5.490	10.020	95.590
150	alpha	0.6	60x8 weeks	Ag80	M	VAC95	75	All	0.996	0.003	0.001	0.004	0.001	0.002	0.020	478.288	1.538	0.336	1.874	0.549	1.002	9.559	4783	15.380	3.360	18.740	5.490	10.020	95.590
150	alpha	1	60x8 weeks	Ag80	M	VAC95	75	All	0.996	0.003	0.001	0.005	0.001	0.003	0.020	477.954	1.563	0.641	2.204	0.629	1.276	9.428	4780	15.630	6.410	22.040	6.290	12.760	94.280
150	alpha	1	60x8 weeks	Ag80	M	VAC95	75	All	0.996	0.003	0.001	0.005	0.001	0.003	0.020	477.954	1.563	0.641	2.204	0.629	1.276	9.428	4780	15.630	6.410	22.040	6.290	12.760	94.280
150	alpha	1	60x8 weeks	Ag80	M	VAC95	75	All	0.996	0.003	0.001	0.005	0.001	0.003	0.020	477.954	1.563	0.641	2.204	0.629	1.276	9.428	4780	15.630	6.410	22.040	6.290	12.760	94.280
150	alpha	1.2	60x8 weeks	Ag80	M	VAC95	75	All	0.995	0.003	0.002	0.005	0.002	0.003	0.020	477.685	1.573	0.968	2.541	0.740	1.381	9.458	4777	15.730	9.680	25.410	7.400	13.810	94.580
150	alpha	1.2	60x8 weeks	Ag80	M	VAC95	75	All	0.995	0.003	0.002	0.005	0.002	0.003	0.020	477.685	1.573	0.968	2.541	0.740	1.381	9.458	4777	15.730	9.680	25.410	7.400	13.810	94.580
150	alpha	1.2	60x8 weeks	Ag80	M	VAC95	75	All	0.995	0.003	0.002	0.005	0.002	0.003	0.020	477.685	1.573	0.968	2.541	0.740	1.381	9.458	4777	15.730	9.680	25.410	7.400	13.810	94.580
150	delta	0.6	60x8 weeks	Ag80	M	VAC95	75	All	0.994	0.003	0.002	0.006	0.002	0.003	0.019	477.354	1.662	1.096	2.758	0.869	1.512	9.329	4774	16.620	10.960	27.580	8.690	15.120	93.290
150	delta	0.6	60x8 weeks	Ag80	M	VAC95	75	All	0.994	0.003	0.002	0.006	0.002	0.003	0.019	477.354	1.662	1.096	2.758	0.869	1.512	9.329	4774	16.620	10.960	27.580	8.690	15.120	93.290
150	delta	0.6	60x8 weeks	Ag80	M	VAC95	75	All	0.994	0.003	0.002	0.006	0.002	0.003	0.019	477.354	1.662	1.096	2.758	0.869	1.512	9.329	4774	16.620	10.960	27.580	8.690	15.120	93.290
150	delta	1	60x8 weeks	Ag80	M	VAC95	75	All	0.992	0.003	0.005	0.009	0.003	0.005	0.020	476.121	1.622	2.608	4.230	1.285	2.382	9.376	4761	16.220	26.080	42.300	12.850	23.820	93.760
150	delta	1	60x8 weeks	Ag80	M	VAC95	75	All	0.992	0.003	0.005	0.009	0.003	0.005	0.020	476.121	1.622	2.608	4.230	1.285	2.382	9.376	4761	16.220	26.080	42.300	12.850	23.820	93.760
150	delta	1	60x8 weeks	Ag80	M	VAC95	75	All	0.992	0.003	0.005	0.009	0.003	0.005	0.020	476.121	1.622	2.608	4.230	1.285	2.382	9.376	4761	16.220	26.080	42.300	12.850	23.820	93.760
150	delta	1.2	60x8 weeks	Ag80	M	VAC95	75	All	0.989	0.003	0.008	0.011	0.003	0.006	0.019	474.827	1.646	3.734	5.380	1.653	3.110	9.167	4748	16.460	37.340	53.800	16.530	31.100	91.670
150	delta	1.2	60x8 weeks	Ag80	M	VAC95	75	All	0.989	0.003	0.008	0.011	0.003	0.006	0.019	474.827	1.646	3.734	5.380	1.653	3.110	9.167	4748	16.460	37.340	53.800	16.530	31.100	91.670
150	delta	1.2	60x8 weeks	Ag80	M	VAC95	75	All	0.989	0.003	0.008	0.011	0.003	0.006	0.019	474.827	1.646	3.734	5.380	1.653	3.110	9.167	4748	16.460	37.340	53.800	16.530	31.100	91.670
150	original	0.6	60x8 weeks	Ag80	M	VAC95	100	All	1.000	0.001	0.000	0.001	0.000	0.000	0.020	479.855	0.341	0.000	0.341	0.118	0.014	9.484	4799	3.410	0.000	3.410	1.180	0.140	94.840
150	original	0.6	60x8 weeks	Ag80	M	VAC95	100	All	1.000	0.001	0.000	0.001	0.000	0.000	0.020	479.855	0.341	0.000	0.341	0.118	0.014	9.484	4799	3.410	0.000	3.410	1.180	0.140	94.840
150	original	0.6	60x8 weeks	Ag80	M	VAC95	100	All	1.000	0.001	0.000	0.001	0.000	0.000	0.020	479.855	0.341	0.000	0.341	0.118	0.014	9.484	4799	3.410	0.000	3.410	1.180	0.140	94.840
150	original	1	60x8 weeks	Ag80	M	VAC95	100	All	1.000	0.001	0.000	0.001	0.000	0.000	0.019	479.833	0.345	0.000	0.345	0.127	0.008	9.351	4798	3.450	0.000	3.450	1.270	0.080	93.510
150	original	1	60x8 weeks	Ag80	M	VAC95	100	All	1.000	0.001	0.000	0.001	0.000	0.000	0.019	479.833	0.345	0.000	0.345	0.127	0.008	9.351	4798	3.450	0.000	3.450	1.270	0.080	93.510
150	original	1	60x8 weeks	Ag80	M	VAC95	100	All	1.000	0.001	0.000	0.001	0.000	0.000	0.019	479.833	0.345	0.000	0.345	0.127	0.008	9.351	4798	3.450	0.000	3.450	1.270	0.080	93.510
150	original	1.2	60x8 weeks	Ag80	M	VAC95	100	All	1.000	0.001	0.000	0.001	0.000	0.000	0.020	479.813	0.358	0.002	0.360	0.132	0.012	9.551	4798	3.580	0.020	3.600	1.320	0.120	95.510
150	original	1.2	60x8 weeks	Ag80	M	VAC95	100	All	1.000	0.001	0.000	0.001	0.000	0.000	0.020	479.813	0.358	0.002	0.360	0.132	0.012	9.551	4798	3.580	0.020	3.600	1.320	0.120	95.510
150	original	1.2	60x8 weeks	Ag80	M	VAC95	100	All	1.000	0.001	0.000	0.001	0.000	0.000	0.020	479.813	0.358	0.002	0.360	0.132	0.012	9.551	4798	3.580	0.020	3.600	1.320	0.120	95.510
150	alpha	0.6	60x8 weeks	Ag80	M	VAC95	100	All	0.999	0.001	0.000	0.001	0.000	0.001	0.020	479.613	0.468	0.015	0.483	0.119	0.246	9.490	4796	4.680	0.150	4.830	1.190	2.460	94.900
150	alpha	0.6	60x8 weeks	Ag80	M	VAC95	100	All	0.999	0.001	0.000	0.001	0.000	0.001	0.020	479.613	0.468	0.015	0.483	0.119	0.246	9.490	4796	4.680	0.150	4.830	1.190	2.460	94.900
150	alpha	0.6	60x8 weeks	Ag80	M	VAC95	100	All	0.999	0.001	0.000	0.001	0.000	0.001	0.020	479.613	0.468	0.015	0.483	0.119	0.246	9.490	4796	4.680	0.150	4.830	1.190	2.460	94.900
150	alpha	1	60x8 weeks	Ag80	M	VAC95	100	All	0.999	0.001	0.000	0.001	0.000	0.001	0.020	479.618	0.432	0.024	0.456	0.117	0.247	9.611	4796	4.320	0.240	4.560	1.170	2.470	96.110
150	alpha	1	60x8 weeks	Ag80	M	VAC95	100	All	0.999	0.001	0.000	0.001	0.000	0.001	0.020	479.618	0.432	0.024	0.456	0.117	0.247	9.611	4796	4.320	0.240	4.560	1.170	2.470	96.110
150	alpha	1	60x8 weeks	Ag80	M	VAC95	100	All	0.999	0.001	0.000	0.001	0.000	0.001	0.020	479.618	0.432	0.024	0.456	0.117	0.247	9.611	4796	4.320	0.240	4.560	1.170	2.470	96.110
150	alpha	1.2	60x8 weeks	Ag80	M	VAC95	100	All	0.999	0.001	0.000	0.001	0.000	0.000	0.020	479.599	0.447	0.025	0.472	0.138	0.234	9.400	4796	4.470	0.250	4.720	1.380	2.340	94.000
150	alpha	1.2	60x8 weeks	Ag80	M	VAC95	100	All	0.999	0.001	0.000	0.001	0.000	0.000	0.020	479.599	0.447	0.025	0.472	0.138	0.234	9.400	4796	4.470	0.250	4.720	1.380	2.340	94.000
150	alpha	1.2	60x8 weeks	Ag80	M	VAC95	100	All	0.999	0.001	0.000	0.001	0.000	0.000	0.020	479.599	0.447	0.025	0.472	0.138	0.234	9.400	4796	4.470	0.250	4.720	1.380	2.340	94.000
150	delta	0.6	60x8 weeks	Ag80	M	VAC95	100	All	0.999	0.002	0.000	0.002	0.000	0.001	0.020	479.283	0.725	0.096	0.821	0.223	0.438	9.457	4793	7.250	0.960	8.210	2.230	4.380	94.570
150	delta	0.6	60x8 weeks	Ag80	M	VAC95	100	All	0.999	0.002	0.000	0.002	0.000	0.001	0.020	479.283	0.725	0.096	0.821	0.223	0.438	9.457	4793	7.250	0.960	8.210	2.230	4.380	94.570
150	delta	0.6	60x8 weeks	Ag80	M	VAC95	100	All	0.999	0.002	0.000	0.002	0.000	0.001	0.020	479.283	0.725	0.096	0.821	0.223	0.438	9.457	4793	7.250	0.960	8.210	2.230	4.380	94.570
150	delta	1	60x8 weeks	Ag80	M	VAC95	100	All	0.998	0.001	0.000	0.002	0.001	0.001	0.020	479.205	0.694	0.173	0.867	0.268	0.474	9.578	4792	6.940	1.730	8.670	2.680	4.740	95.780
150	delta	1	60x8 weeks	Ag80	M	VAC95	100	All	0.998	0.001	0.000	0.002	0.001	0.001	0.020	479.205	0.694	0.173	0.867	0.268	0.474	9.578	4792	6.940	1.730	8.670	2.680	4.740	95.780
150	delta	1	60x8 weeks	Ag80	M	VAC95	100	All	0.998	0.001	0.000	0.002	0.001	0.001	0.020	479.205	0.694	0.173	0.867	0.268	0.474	9.578	4792	6.940	1.730	8.670	2.680	4.740	95.780
150	delta	1.2	60x8 weeks	Ag80	M	VAC95	100	All	0.998	0.002	0.000	0.002	0.001	0.001	0.020	479.211	0.722	0.196	0.918	0.287	0.455	9.566	4792	7.220	1.960	9.180	2.870	4.550	95.660
150	delta	1.2	60x8 weeks	Ag80	M	VAC95	100	All	0.998	0.002	0.000	0.002	0.001	0.001	0.020	479.211	0.722	0.196	0.918	0.287	0.455	9.566	4792	7.220	1.960	9.180	2.870	4.550	95.660
150	delta	1.2	60x8 weeks	Ag80	M	VAC95	100	All	0.998	0.002	0.000	0.002	0.001	0.001	0.020	479.211	0.722	0.196	0.918	0.287	0.455	9.566	4792	7.220	1.960	9.180	2.870	4.550	95.660
150	original	0.6	60x8 weeks	Ag80	M	VAC70	25	All	0.991	0.008	0.003	0.011	0.003	0.005	0.019	475.633	3.961	1.371	5.332	1.579	2.359	9.138	4756	39.610	13.710	53.320	15.790	23.590	91.380
150	original	0.6	60x8 weeks	Ag80	M	VAC70	25	All	0.991	0.008	0.003	0.011	0.003	0.005	0.019	475.633	3.961	1.371	5.332	1.579	2.359	9.138	4756	39.610	13.710	53.320	15.790	23.590	91.380
150	original	0.6	60x8 weeks	Ag80	M	VAC70	25	All	0.991	0.008	0.003	0.011	0.003	0.005	0.019	475.633	3.961	1.371	5.332	1.579	2.359	9.138	4756	39.610	13.710	53.320	15.790	23.590	91.380
150	original	1	60x8 weeks	Ag80	M	VAC70	25	All	0.988	0.008	0.006	0.014	0.004	0.006	0.019	474.368	3.871	3.015	6.886	2.099	2.986	9.256	4744	38.710	30.150	68.860	20.990	29.860	92.560
150	original	1	60x8 weeks	Ag80	M	VAC70	25	All	0.988	0.008	0.006	0.014	0.004	0.006	0.019	474.368	3.871	3.015	6.886	2.099	2.986	9.256	4744	38.710	30.150	68.860	20.990	29.860	92.560
150	original	1	60x8 weeks	Ag80	M	VAC70	25	All	0.988	0.008	0.006	0.014	0.004	0.006	0.019	474.368	3.871	3.015	6.886	2.099	2.986	9.256	4744	38.710	30.150	68.860	20.990	29.860	92.560
150	original	1.2	60x8 weeks	Ag80	M	VAC70	25	All	0.987	0.008	0.008	0.016	0.005	0.007	0.018	473.635	3.888	3.869	7.757	2.284	3.456	8.859	4736	38.880	38.690	77.570	22.840	34.560	88.590
150	original	1.2	60x8 weeks	Ag80	M	VAC70	25	All	0.987	0.008	0.008	0.016	0.005	0.007	0.018	473.635	3.888	3.869	7.757	2.284	3.456	8.859	4736	38.880	38.690	77.570	22.840	34.560	88.590
150	original	1.2	60x8 weeks	Ag80	M	VAC70	25	All	0.987	0.008	0.008	0.016	0.005	0.007	0.018	473.635	3.888	3.869	7.757	2.284	3.456	8.859	4736	38.880	38.690	77.570	22.840	34.560	88.590
150	alpha	0.6	60x8 weeks	Ag80	M	VAC70	25	All	0.984	0.008	0.009	0.017	0.005	0.009	0.018	472.495	3.830	4.107	7.937	2.438	4.496	8.841	4725	38.300	41.070	79.370	24.380	44.960	88.410
150	alpha	0.6	60x8 weeks	Ag80	M	VAC70	25	All	0.984	0.008	0.009	0.017	0.005	0.009	0.018	472.495	3.830	4.107	7.937	2.438	4.496	8.841	4725	38.300	41.070	79.370	24.380	44.960	88.410

150	alpha	0.6	60x8 weeks	Ag80	M	VAC70	25	All	0.984	0.008	0.009	0.017	0.005	0.009	0.018	472.495	3.830	4.107	7.937	2.438	4.496	8.841	4725	38.300	41.070	79.370	24.380	44.960	88.410
150	alpha	1	60x8 weeks	Ag80	M	VAC70	25	All	0.968	0.008	0.025	0.033	0.010	0.019	0.018	464.845	3.627	12.037	15.664	4.745	9.102	8.612	4648	36.270	120.370	156.640	47.450	91.020	86.120
150	alpha	1	60x8 weeks	Ag80	M	VAC70	25	All	0.968	0.008	0.025	0.033	0.010	0.019	0.018	464.845	3.627	12.037	15.664	4.745	9.102	8.612	4648	36.270	120.370	156.640	47.450	91.020	86.120
150	alpha	1	60x8 weeks	Ag80	M	VAC70	25	All	0.968	0.008	0.025	0.033	0.010	0.019	0.018	464.845	3.627	12.037	15.664	4.745	9.102	8.612	4648	36.270	120.370	156.640	47.450	91.020	86.120
150	alpha	1.2	60x8 weeks	Ag80	M	VAC70	25	All	0.957	0.007	0.037	0.044	0.014	0.025	0.017	459.415	3.396	17.559	20.955	6.560	12.144	7.972	4594	33.960	175.590	209.550	65.600	121.440	79.720
150	alpha	1.2	60x8 weeks	Ag80	M	VAC70	25	All	0.957	0.007	0.037	0.044	0.014	0.025	0.017	459.415	3.396	17.559	20.955	6.560	12.144	7.972	4594	33.960	175.590	209.550	65.600	121.440	79.720
150	alpha	1.2	60x8 weeks	Ag80	M	VAC70	25	All	0.957	0.007	0.037	0.044	0.014	0.025	0.017	459.415	3.396	17.559	20.955	6.560	12.144	7.972	4594	33.960	175.590	209.550	65.600	121.440	79.720
150	delta	0.6	60x8 weeks	Ag80	M	VAC70	25	All	0.951	0.007	0.042	0.049	0.017	0.028	0.016	456.260	3.428	20.324	23.752	7.942	13.470	7.880	4563	34.280	203.240	237.520	79.420	134.700	78.800
150	delta	0.6	60x8 weeks	Ag80	M	VAC70	25	All	0.951	0.007	0.042	0.049	0.017	0.028	0.016	456.260	3.428	20.324	23.752	7.942	13.470	7.880	4563	34.280	203.240	237.520	79.420	134.700	78.800
150	delta	0.6	60x8 weeks	Ag80	M	VAC70	25	All	0.951	0.007	0.042	0.049	0.017	0.028	0.016	456.260	3.428	20.324	23.752	7.942	13.470	7.880	4563	34.280	203.240	237.520	79.420	134.700	78.800
150	delta	1	60x8 weeks	Ag80	M	VAC70	25	All	0.892	0.005	0.095	0.100	0.034	0.060	0.012	427.988	2.399	45.531	47.930	16.120	28.831	5.573	4280	23.990	455.310	479.300	161.200	288.310	55.730
150	delta	1	60x8 weeks	Ag80	M	VAC70	25	All	0.892	0.005	0.095	0.100	0.034	0.060	0.012	427.988	2.399	45.531	47.930	16.120	28.831	5.573	4280	23.990	455.310	479.300	161.200	288.310	55.730
150	delta	1	60x8 weeks	Ag80	M	VAC70	25	All	0.892	0.005	0.095	0.100	0.034	0.060	0.012	427.988	2.399	45.531	47.930	16.120	28.831	5.573	4280	23.990	455.310	479.300	161.200	288.310	55.730
150	delta	1.2	60x8 weeks	Ag80	M	VAC70	25	All	0.881	0.004	0.103	0.108	0.036	0.065	0.011	422.713	2.109	49.537	51.646	17.329	31.420	5.094	4227	21.090	495.370	516.460	173.290	314.200	50.940
150	delta	1.2	60x8 weeks	Ag80	M	VAC70	25	All	0.881	0.004	0.103	0.108	0.036	0.065	0.011	422.713	2.109	49.537	51.646	17.329	31.420	5.094	4227	21.090	495.370	516.460	173.290	314.200	50.940
150	delta	1.2	60x8 weeks	Ag80	M	VAC70	25	All	0.881	0.004	0.103	0.108	0.036	0.065	0.011	422.713	2.109	49.537	51.646	17.329	31.420	5.094	4227	21.090	495.370	516.460	173.290	314.200	50.940
150	original	0.6	60x8 weeks	Ag80	M	VAC70	50	All	0.994	0.007	0.001	0.008	0.003	0.003	0.020	476.954	3.219	0.688	3.907	1.259	1.439	9.419	4770	32.190	6.880	39.070	12.590	14.390	94.190
150	original	0.6	60x8 weeks	Ag80	M	VAC70	50	All	0.994	0.007	0.001	0.008	0.003	0.003	0.020	476.954	3.219	0.688	3.907	1.259	1.439	9.419	4770	32.190	6.880	39.070	12.590	14.390	94.190
150	original	0.6	60x8 weeks	Ag80	M	VAC70	50	All	0.994	0.007	0.001	0.008	0.003	0.003	0.020	476.954	3.219	0.688	3.907	1.259	1.439	9.419	4770	32.190	6.880	39.070	12.590	14.390	94.190
150	original	1	60x8 weeks	Ag80	M	VAC70	50	All	0.992	0.007	0.003	0.010	0.003	0.004	0.019	476.376	3.277	1.326	4.603	1.483	1.739	9.307	4764	32.770	13.260	46.030	14.830	17.390	93.070
150	original	1	60x8 weeks	Ag80	M	VAC70	50	All	0.992	0.007	0.003	0.010	0.003	0.004	0.019	476.376	3.277	1.326	4.603	1.483	1.739	9.307	4764	32.770	13.260	46.030	14.830	17.390	93.070
150	original	1	60x8 weeks	Ag80	M	VAC70	50	All	0.992	0.007	0.003	0.010	0.003	0.004	0.019	476.376	3.277	1.326	4.603	1.483	1.739	9.307	4764	32.770	13.260	46.030	14.830	17.390	93.070
150	original	1.2	60x8 weeks	Ag80	M	VAC70	50	All	0.992	0.007	0.004	0.010	0.003	0.004	0.020	476.172	3.187	1.702	4.889	1.631	1.788	9.465	4762	31.870	17.020	48.890	16.310	17.880	94.650
150	original	1.2	60x8 weeks	Ag80	M	VAC70	50	All	0.992	0.007	0.004	0.010	0.003	0.004	0.020	476.172	3.187	1.702	4.889	1.631	1.788	9.465	4762	31.870	17.020	48.890	16.310	17.880	94.650
150	original	1.2	60x8 weeks	Ag80	M	VAC70	50	All	0.992	0.007	0.004	0.010	0.003	0.004	0.020	476.172	3.187	1.702	4.889	1.631	1.788	9.465	4762	31.870	17.020	48.890	16.310	17.880	94.650
150	alpha	0.6	60x8 weeks	Ag80	M	VAC70	50	All	0.990	0.006	0.004	0.010	0.003	0.006	0.020	475.388	3.067	1.920	4.987	1.543	2.718	9.412	4754	30.670	19.200	49.870	15.430	27.180	94.120
150	alpha	0.6	60x8 weeks	Ag80	M	VAC70	50	All	0.990	0.006	0.004	0.010	0.003	0.006	0.020	475.388	3.067	1.920	4.987	1.543	2.718	9.412	4754	30.670	19.200	49.870	15.430	27.180	94.120
150	alpha	0.6	60x8 weeks	Ag80	M	VAC70	50	All	0.990	0.006	0.004	0.010	0.003	0.006	0.020	475.388	3.067	1.920	4.987	1.543	2.718	9.412	4754	30.670	19.200	49.870	15.430	27.180	94.120
150	alpha	1	60x8 weeks	Ag80	M	VAC70	50	All	0.984	0.007	0.010	0.017	0.005	0.009	0.019	472.461	3.178	4.810	7.988	2.397	4.499	8.971	4725	31.780	48.100	79.880	23.970	44.990	89.710
150	alpha	1	60x8 weeks	Ag80	M	VAC70	50	All	0.984	0.007	0.010	0.017	0.005	0.009	0.019	472.461	3.178	4.810	7.988	2.397	4.499	8.971	4725	31.780	48.100	79.880	23.970	44.990	89.710
150	alpha	1	60x8 weeks	Ag80	M	VAC70	50	All	0.984	0.007	0.010	0.017	0.005	0.009	0.019	472.461	3.178	4.810	7.988	2.397	4.499	8.971	4725	31.780	48.100	79.880	23.970	44.990	89.710
150	alpha	1.2	60x8 weeks	Ag80	M	VAC70	50	All	0.980	0.006	0.014	0.020	0.006	0.012	0.018	470.629	2.985	6.777	9.762	2.935	5.558	8.718	4706	29.850	67.770	97.620	29.350	55.580	87.180
150	alpha	1.2	60x8 weeks	Ag80	M	VAC70	50	All	0.980	0.006	0.014	0.020	0.006	0.012	0.018	470.629	2.985	6.777	9.762	2.935	5.558	8.718	4706	29.850	67.770	97.620	29.350	55.580	87.180
150	alpha	1.2	60x8 weeks	Ag80	M	VAC70	50	All	0.980	0.006	0.014	0.020	0.006	0.012	0.018	470.629	2.985	6.777	9.762	2.935	5.558	8.718	4706	29.850	67.770	97.620	29.350	55.580	87.180
150	delta	0.6	60x8 weeks	Ag80	M	VAC70	50	All	0.975	0.006	0.019	0.025	0.008	0.014	0.018	467.975	3.108	8.903	12.011	4.040	6.796	8.662	4680	31.080	89.030	120.110	40.400	67.960	86.620
150	delta	0.6	60x8 weeks	Ag80	M	VAC70	50	All	0.975	0.006	0.019	0.025	0.008	0.014	0.018	467.975	3.108	8.903	12.011	4.040	6.796	8.662	4680	31.080	89.030	120.110	40.400	67.960	86.620
150	delta	0.6	60x8 weeks	Ag80	M	VAC70	50	All	0.975	0.006	0.019	0.025	0.008	0.014	0.018	467.975	3.108	8.903	12.011	4.040	6.796	8.662	4680	31.080	89.030	120.110	40.400	67.960	86.620
150	delta	1	60x8 weeks	Ag80	M	VAC70	50	All	0.938	0.005	0.054	0.060	0.020	0.035	0.015	450.147	2.625	26.065	28.690	9.567	16.876	7.180	4501	26.250	260.650	286.900	95.670	168.760	71.800
150	delta	1	60x8 weeks	Ag80	M	VAC70	50	All	0.938	0.005	0.054	0.060	0.020	0.035	0.015	450.147	2.625	26.065	28.690	9.567	16.876	7.180	4501	26.250	260.650	286.900	95.670	168.760	71.800
150	delta	1	60x8 weeks	Ag80	M	VAC70	50	All	0.938	0.005	0.054	0.060	0.020	0.035	0.015	450.147	2.625	26.065	28.690	9.567	16.876	7.180	4501	26.250	260.650	286.900	95.670	168.760	71.800
150	delta	1.2	60x8 weeks	Ag80	M	VAC70	50	All	0.918	0.005	0.074	0.079	0.026	0.046	0.014	440.817	2.332	35.452	37.784	12.396	22.137	6.760	4408	23.320	354.520	377.840	123.960	221.370	67.600
150	delta	1.2	60x8 weeks	Ag80	M	VAC70	50	All	0.918	0.005	0.074	0.079	0.026	0.046	0.014	440.817	2.332	35.452	37.784	12.396	22.137	6.760	4408	23.320	354.520	377.840	123.960	221.370	67.600
150	delta	1.2	60x8 weeks	Ag80	M	VAC70	50	All	0.918	0.005	0.074	0.079	0.026	0.046	0.014	440.817	2.332	35.452	37.784	12.396	22.137	6.760	4408	23.320	354.520	377.840	123.960	221.370	67.600
150	original	0.6	60x8 weeks	Ag80	M	VAC70	75	All	0.996	0.005	0.001	0.006	0.002	0.001	0.019	478.056	2.451	0.265	2.716	1.012	0.678	9.358	4781	24.510	2.650	27.160	10.120	6.780	93.580
150	original	0.6	60x8 weeks	Ag80	M	VAC70	75	All	0.996	0.005	0.001	0.006	0.002	0.001	0.019	478.056	2.451	0.265	2.716	1.012	0.678	9.358	4781	24.510	2.650	27.160	10.120	6.780	93.580
150	original	0.6	60x8 weeks	Ag80	M	VAC70	75	All	0.996	0.005	0.001	0.006	0.002	0.001	0.019	478.056	2.451	0.265	2.716	1.012	0.678	9.358	4781	24.510	2.650	27.160	10.120	6.780	93.580
150	original	1	60x8 weeks	Ag80	M	VAC70	75	All	0.996	0.005	0.001	0.006	0.002	0.001	0.020	477.860	2.513	0.422	2.935	1.123	0.716	9.443	4779	25.130	4.220	29.350	11.230	7.160	94.430
150	original	1	60x8 weeks	Ag80	M	VAC70	75	All	0.996	0.005	0.001	0.006	0.002	0.001	0.020	477.860	2.513	0.422	2.935	1.123	0.716	9.443	4779	25.130	4.220	29.350	11.230	7.160	94.430
150	original	1	60x8 weeks	Ag80	M	VAC70	75	All	0.996	0.005	0.001	0.006	0.002	0.001	0.020	477.860	2.513	0.422	2.935	1.123	0.716	9.443	4779	25.130	4.220	29.350	11.230	7.160	94.430
150	original	1.2	60x8 weeks	Ag80	M	VAC70	75	All	0.995	0.005	0.001	0.006	0.002	0.002	0.020	477.814	2.384	0.549	2.933	1.127	0.779	9.448	4778	23.840	5.490	29.330	11.270	7.790	94.480
150	original	1.2	60x8 weeks	Ag80	M	VAC70	75	All	0.995	0.005	0.001	0.006	0.002	0.002	0.020	477.814	2.384	0.549	2.933	1.127	0.779	9.448	4778	23.840	5.490	29.330	11.270	7.790	94.480
150	original	1.2	60x8 weeks	Ag80	M	VAC70	75	All	0.995	0.005	0.001	0.006	0.002	0.002	0.020	477.814	2.384	0.549	2.933	1.127	0.779	9.448	4778	23.840	5.490	29.330	11.270	7.790	94.480
150	alpha	0.6	60x8 weeks	Ag80	M	VAC70	75	All	0.994	0.005	0.002	0.006	0.002	0.004	0.019	477.093	2.271	0.834	3.105	0.947	1.694	9.305	4771	22.710	8.340	31.050	9.470	16.940	93.050
150	alpha	0.6	60x8 weeks	Ag80	M	VAC70	75	All	0.994	0.005	0.002	0.006	0.002	0.004	0.019	477.093	2.271	0.834	3.105	0.947	1.694	9.305	4771	22.710	8.340	31.050	9.470	16.940	93.050
150	alpha	0.6	60x8 weeks	Ag80	M	VAC70	75	All	0.994	0.005	0.002	0.006	0.002	0.004	0.019	477.093	2.271	0.834	3.105	0.947	1.694	9.305	4771	22.710	8.340	31.050	9.470	16.940	93.050
150	alpha	1	60x8 weeks	Ag80	M	VAC70	75	All	0.992	0.005	0.004	0.009	0.003	0.005	0.019	476.167	2.413	1.727	4.140	1.283	2.207	9.345	4762	24.130	17.270	41.400	12.830	22.070	93.450
150	alpha	1	60x8 weeks	Ag80	M	VAC70	75	All	0.992	0.005	0.004	0.009	0.003	0.005	0.019	476.167	2.413	1.727	4.140	1.283	2.207	9.345	4762	24.130	17.270	41.400	12.830	22.070	93.450
150	alpha	1	60x8 weeks	Ag80	M	VAC70	75	All	0.992	0.005	0.004	0.009	0.003	0.005	0.019	476.167	2.413	1.727	4.140	1.283	2.207	9.345	4762	24.130	17.270	41.400	12.830	22.070	93.450
150	alpha	1.2	60x8 weeks	Ag80	M	VAC70	75	All	0.991	0.005	0.005	0.010	0.003	0.005	0.019	475.699	2.351	2.268	4.619	1.492	2.445	9.197	4757	23.510	22.680	46.190	14.920	24.450	91.970
150	alpha	1.2	60x8 weeks	Ag80	M	VAC70	75	All	0.991	0.005	0.005	0.010	0.003	0.005	0.019	475.699	2.351	2.268	4.619	1.492	2.445	9.197	4757	23.510	22.680	46.190	14.920	24.450	91.970
150	alpha	1.2	60x8 weeks	Ag80	M	VAC70	75	All	0.991	0.005	0.005	0.010	0.003	0.005	0.019	475.699	2.351	2.268	4.619	1.492	2.445	9.197	4757	23.510	22.680	46.190	14.920	24.450	91.970
150	delta	0.6	60x8 weeks	Ag80	M	VAC70	75	All	0.988	0.006	0.007	0.013	0.004	0.007	0.019	474.045	2.670	3.378	6.048	2.025	3.365	9.118	4740	26.700	33.780	60.480	20.250	33.650	91.180
150	delta	0.6	60x8 weeks	Ag80	M	VAC70	75	All	0.988	0.006	0.007	0.013	0.004	0.007	0.019	474.045	2.670	3.378	6.048	2.025	3.365	9.118	4740	26.700	33.780	60.480	20.250	33.650	91.180
150	delta	0.6	60x8 weeks	Ag80	M	VAC70	75	All	0.988	0.006	0.007	0.013	0.004	0.007	0.019	474.045	2.670	3.378	6.048	2.025	3.365	9.118	4740	26.700	33.780	60.480	20.250	33.650	91.180
150	delta	1	60x8 weeks	Ag80	M	VAC70	75	All	0.975	0.005	0.021	0.026	0.009	0.015	0.018	467.815	2.626	10.053	12.679	4.118	7.142	8.680	4678	26.260	100.530	126.790	41.180	71.420	86.800
150	delta	1	60x8 weeks	Ag80	M	VAC70	75	All	0.975	0.005	0.021	0.026	0.009	0.015	0.018	467.815	2.626	10.053	12.679	4.118	7.142	8.680	4678	26.260	100.530	126.790	41.180	71.420	86.800
150	delta	1	60x8 weeks	Ag80	M	VAC70	75	All	0.975	0.005	0.021	0.026	0.009	0.015	0.018	467.815	2.626	10.053	12.679	4.118	7.142	8.680	4678	26.260	100.530	126.790	41.180	71.420	86.800
150	delta	1.2	60x8 weeks	Ag80	M	VAC70	75	All	0.966	0.005	0.030	0.035	0.011	0.020	0.018	463.736	2.488	14.397	16.885	5.480	9.450	8.422	4637	24.880	143.970	168.850	54.800	94.500	84.220
150	delta	1.2	60x8 weeks	Ag80	M	VAC70	75	All	0.966	0.005	0.030	0.035	0.011	0.020	0.018	463.736	2.488	14.397	16.885	5.480	9.450	8.422	4637	24.880	143.970	168.850	54.800	94.500	84.220
150	delta	1.2	60x8 weeks	Ag80	M	VAC70	75	All	0.966	0.005	0.030	0.035	0.011	0.020	0.018	463.736	2.488	14.397	16.885	5.480	9.450	8.422	4637	24.880	143.970	168.850	54.800	94.500	84.220
150	original	0.6	60x8 weeks	Ag80	M	VAC70	100	All	0.998	0.003	0.000	0.003	0.002	0.000	0.020	478.949	1.653	0.013	1.666	0.782	0.075	9.482	4789	16.530	0.130	16.660	7.820	0.750	94.820

150	original	0.6	60x8 weeks	Ag80	M	VAC70	100	All	0.998	0.003	0.000	0.003	0.002	0.000	0.020	478.949	1.653	0.013	1.666	0.782	0.075	9.482	4789	16.530	0.130	16.660	7.820	0.750	94.820
150	original	0.6	60x8 weeks	Ag80	M	VAC70	100	All	0.998	0.003	0.000	0.003	0.002	0.000	0.020	478.949	1.653	0.013	1.666	0.782	0.075	9.482	4789	16.530	0.130	16.660	7.820	0.750	94.820
150	original	1	60x8 weeks	Ag80	M	VAC70	100	All	0.998	0.003	0.000	0.003	0.002	0.000	0.020	478.989	1.573	0.027	1.600	0.750	0.065	9.579	4790	15.730	0.270	16.000	7.500	0.650	95.790
150	original	1	60x8 weeks	Ag80	M	VAC70	100	All	0.998	0.003	0.000	0.003	0.002	0.000	0.020	478.989	1.573	0.027	1.600	0.750	0.065	9.579	4790	15.730	0.270	16.000	7.500	0.650	95.790
150	original	1	60x8 weeks	Ag80	M	VAC70	100	All	0.998	0.003	0.000	0.003	0.002	0.000	0.020	478.989	1.573	0.027	1.600	0.750	0.065	9.579	4790	15.730	0.270	16.000	7.500	0.650	95.790
150	original	1.2	60x8 weeks	Ag80	M	VAC70	100	All	0.998	0.003	0.000	0.003	0.002	0.000	0.020	479.009	1.627	0.027	1.654	0.738	0.061	9.477	4790	16.270	0.270	16.540	7.380	0.610	94.770
150	original	1.2	60x8 weeks	Ag80	M	VAC70	100	All	0.998	0.003	0.000	0.003	0.002	0.000	0.020	479.009	1.627	0.027	1.654	0.738	0.061	9.477	4790	16.270	0.270	16.540	7.380	0.610	94.770
150	original	1.2	60x8 weeks	Ag80	M	VAC70	100	All	0.998	0.003	0.000	0.003	0.002	0.000	0.020	479.009	1.627	0.027	1.654	0.738	0.061	9.477	4790	16.270	0.270	16.540	7.380	0.610	94.770
150	alpha	0.6	60x8 weeks	Ag80	M	VAC70	100	All	0.997	0.003	0.000	0.004	0.001	0.002	0.020	478.377	1.573	0.233	1.806	0.581	0.893	9.377	4784	15.730	2.330	18.060	5.810	8.930	93.770
150	alpha	0.6	60x8 weeks	Ag80	M	VAC70	100	All	0.997	0.003	0.000	0.004	0.001	0.002	0.020	478.377	1.573	0.233	1.806	0.581	0.893	9.377	4784	15.730	2.330	18.060	5.810	8.930	93.770
150	alpha	0.6	60x8 weeks	Ag80	M	VAC70	100	All	0.997	0.003	0.000	0.004	0.001	0.002	0.020	478.377	1.573	0.233	1.806	0.581	0.893	9.377	4784	15.730	2.330	18.060	5.810	8.930	93.770
150	alpha	1	60x8 weeks	Ag80	M	VAC70	100	All	0.996	0.003	0.001	0.004	0.001	0.002	0.020	478.114	1.634	0.457	2.091	0.674	1.022	9.530	4781	16.340	4.570	20.910	6.740	10.220	95.300
150	alpha	1	60x8 weeks	Ag80	M	VAC70	100	All	0.996	0.003	0.001	0.004	0.001	0.002	0.020	478.114	1.634	0.457	2.091	0.674	1.022	9.530	4781	16.340	4.570	20.910	6.740	10.220	95.300
150	alpha	1	60x8 weeks	Ag80	M	VAC70	100	All	0.996	0.003	0.001	0.004	0.001	0.002	0.020	478.114	1.634	0.457	2.091	0.674	1.022	9.530	4781	16.340	4.570	20.910	6.740	10.220	95.300
150	alpha	1.2	60x8 weeks	Ag80	M	VAC70	100	All	0.996	0.003	0.001	0.004	0.001	0.002	0.020	478.189	1.549	0.485	2.034	0.649	1.006	9.532	4782	15.490	4.850	20.340	6.490	10.060	95.320
150	alpha	1.2	60x8 weeks	Ag80	M	VAC70	100	All	0.996	0.003	0.001	0.004	0.001	0.002	0.020	478.189	1.549	0.485	2.034	0.649	1.006	9.532	4782	15.490	4.850	20.340	6.490	10.060	95.320
150	alpha	1.2	60x8 weeks	Ag80	M	VAC70	100	All	0.996	0.003	0.001	0.004	0.001	0.002	0.020	478.189	1.549	0.485	2.034	0.649	1.006	9.532	4782	15.490	4.850	20.340	6.490	10.060	95.320
150	delta	0.6	60x8 weeks	Ag80	M	VAC70	100	All	0.994	0.004	0.002	0.006	0.002	0.003	0.020	477.186	2.038	0.953	2.991	0.971	1.581	9.403	4772	20.380	9.530	29.910	9.710	15.810	94.030
150	delta	0.6	60x8 weeks	Ag80	M	VAC70	100	All	0.994	0.004	0.002	0.006	0.002	0.003	0.020	477.186	2.038	0.953	2.991	0.971	1.581	9.403	4772	20.380	9.530	29.910	9.710	15.810	94.030
150	delta	0.6	60x8 weeks	Ag80	M	VAC70	100	All	0.994	0.004	0.002	0.006	0.002	0.003	0.020	477.186	2.038	0.953	2.991	0.971	1.581	9.403	4772	20.380	9.530	29.910	9.710	15.810	94.030
150	delta	1	60x8 weeks	Ag80	M	VAC70	100	All	0.992	0.004	0.004	0.009	0.003	0.005	0.020	476.112	2.073	2.096	4.169	1.329	2.267	9.368	4761	20.730	20.960	41.690	13.290	22.670	93.680
150	delta	1	60x8 weeks	Ag80	M	VAC70	100	All	0.992	0.004	0.004	0.009	0.003	0.005	0.020	476.112	2.073	2.096	4.169	1.329	2.267	9.368	4761	20.730	20.960	41.690	13.290	22.670	93.680
150	delta	1	60x8 weeks	Ag80	M	VAC70	100	All	0.992	0.004	0.004	0.009	0.003	0.005	0.020	476.112	2.073	2.096	4.169	1.329	2.267	9.368	4761	20.730	20.960	41.690	13.290	22.670	93.680
150	delta	1.2	60x8 weeks	Ag80	M	VAC70	100	All	0.990	0.004	0.006	0.011	0.003	0.006	0.019	475.207	2.036	3.034	5.070	1.629	2.745	9.059	4752	20.360	30.340	50.700	16.290	27.450	90.590
150	delta	1.2	60x8 weeks	Ag80	M	VAC70	100	All	0.990	0.004	0.006	0.011	0.003	0.006	0.019	475.207	2.036	3.034	5.070	1.629	2.745	9.059	4752	20.360	30.340	50.700	16.290	27.450	90.590
150	delta	1.2	60x8 weeks	Ag80	M	VAC70	100	All	0.990	0.004	0.006	0.011	0.003	0.006	0.019	475.207	2.036	3.034	5.070	1.629	2.745	9.059	4752	20.360	30.340	50.700	16.290	27.450	90.590
150	original	0.6	60x8 weeks	Ag80	M	VAC50	25	All	0.990	0.009	0.003	0.012	0.004	0.005	0.019	475.270	4.202	1.503	5.705	1.797	2.362	9.091	4753	42.020	15.030	57.050	17.970	23.620	90.910
150	original	0.6	60x8 weeks	Ag80	M	VAC50	25	All	0.990	0.009	0.003	0.012	0.004	0.005	0.019	475.270	4.202	1.503	5.705	1.797	2.362	9.091	4753	42.020	15.030	57.050	17.970	23.620	90.910
150	original	0.6	60x8 weeks	Ag80	M	VAC50	25	All	0.990	0.009	0.003	0.012	0.004	0.005	0.019	475.270	4.202	1.503	5.705	1.797	2.362	9.091	4753	42.020	15.030	57.050	17.970	23.620	90.910
150	original	1	60x8 weeks	Ag80	M	VAC50	25	All	0.988	0.009	0.007	0.015	0.005	0.006	0.019	474.073	4.167	3.233	7.400	2.279	3.085	9.045	4741	41.670	32.330	74.000	22.790	30.850	90.450
150	original	1	60x8 weeks	Ag80	M	VAC50	25	All	0.988	0.009	0.007	0.015	0.005	0.006	0.019	474.073	4.167	3.233	7.400	2.279	3.085	9.045	4741	41.670	32.330	74.000	22.790	30.850	90.450
150	original	1	60x8 weeks	Ag80	M	VAC50	25	All	0.988	0.009	0.007	0.015	0.005	0.006	0.019	474.073	4.167	3.233	7.400	2.279	3.085	9.045	4741	41.670	32.330	74.000	22.790	30.850	90.450
150	original	1.2	60x8 weeks	Ag80	M	VAC50	25	All	0.987	0.009	0.008	0.017	0.005	0.007	0.019	473.587	4.097	3.939	8.036	2.462	3.341	9.255	4736	40.970	39.390	80.360	24.620	33.410	92.550
150	original	1.2	60x8 weeks	Ag80	M	VAC50	25	All	0.987	0.009	0.008	0.017	0.005	0.007	0.019	473.587	4.097	3.939	8.036	2.462	3.341	9.255	4736	40.970	39.390	80.360	24.620	33.410	92.550
150	original	1.2	60x8 weeks	Ag80	M	VAC50	25	All	0.987	0.009	0.008	0.017	0.005	0.007	0.019	473.587	4.097	3.939	8.036	2.462	3.341	9.255	4736	40.970	39.390	80.360	24.620	33.410	92.550
150	alpha	0.6	60x8 weeks	Ag80	M	VAC50	25	All	0.983	0.009	0.009	0.018	0.005	0.010	0.018	471.790	4.163	4.433	8.596	2.635	4.835	8.787	4718	41.630	44.330	85.960	26.350	48.350	87.870
150	alpha	0.6	60x8 weeks	Ag80	M	VAC50	25	All	0.983	0.009	0.009	0.018	0.005	0.010	0.018	471.790	4.163	4.433	8.596	2.635	4.835	8.787	4718	41.630	44.330	85.960	26.350	48.350	87.870
150	alpha	0.6	60x8 weeks	Ag80	M	VAC50	25	All	0.983	0.009	0.009	0.018	0.005	0.010	0.018	471.790	4.163	4.433	8.596	2.635	4.835	8.787	4718	41.630	44.330	85.960	26.350	48.350	87.870
150	alpha	1	60x8 weeks	Ag80	M	VAC50	25	All	0.965	0.008	0.028	0.036	0.011	0.021	0.017	463.064	3.811	13.570	17.381	5.390	10.145	8.336	4631	38.110	135.700	173.810	53.900	101.450	83.360
150	alpha	1	60x8 weeks	Ag80	M	VAC50	25	All	0.965	0.008	0.028	0.036	0.011	0.021	0.017	463.064	3.811	13.570	17.381	5.390	10.145	8.336	4631	38.110	135.700	173.810	53.900	101.450	83.360
150	alpha	1	60x8 weeks	Ag80	M	VAC50	25	All	0.965	0.008	0.028	0.036	0.011	0.021	0.017	463.064	3.811	13.570	17.381	5.390	10.145	8.336	4631	38.110	135.700	173.810	53.900	101.450	83.360
150	alpha	1.2	60x8 weeks	Ag80	M	VAC50	25	All	0.953	0.007	0.041	0.049	0.015	0.028	0.016	457.230	3.501	19.876	23.377	7.275	13.419	7.663	4572	35.010	198.760	233.770	72.750	134.190	76.630
150	alpha	1.2	60x8 weeks	Ag80	M	VAC50	25	All	0.953	0.007	0.041	0.049	0.015	0.028	0.016	457.230	3.501	19.876	23.377	7.275	13.419	7.663	4572	35.010	198.760	233.770	72.750	134.190	76.630
150	alpha	1.2	60x8 weeks	Ag80	M	VAC50	25	All	0.953	0.007	0.041	0.049	0.015	0.028	0.016	457.230	3.501	19.876	23.377	7.275	13.419	7.663	4572	35.010	198.760	233.770	72.750	134.190	76.630
150	delta	0.6	60x8 weeks	Ag80	M	VAC50	25	All	0.939	0.007	0.052	0.059	0.020	0.034	0.016	450.938	3.441	24.853	28.294	9.394	16.555	7.533	4509	34.410	248.530	282.940	93.940	165.550	75.330
150	delta	0.6	60x8 weeks	Ag80	M	VAC50	25	All	0.939	0.007	0.052	0.059	0.020	0.034	0.016	450.938	3.441	24.853	28.294	9.394	16.555	7.533	4509	34.410	248.530	282.940	93.940	165.550	75.330
150	delta	0.6	60x8 weeks	Ag80	M	VAC50	25	All	0.939	0.007	0.052	0.059	0.020	0.034	0.016	450.938	3.441	24.853	28.294	9.394	16.555	7.533	4509	34.410	248.530	282.940	93.940	165.550	75.330
150	delta	1	60x8 weeks	Ag80	M	VAC50	25	All	0.881	0.005	0.102	0.108	0.037	0.064	0.011	422.975	2.448	49.161	51.609	17.767	30.780	5.146	4230	24.480	491.610	516.090	177.670	307.800	51.460
150	delta	1	60x8 weeks	Ag80	M	VAC50	25	All	0.881	0.005	0.102	0.108	0.037	0.064	0.011	422.975	2.448	49.161	51.609	17.767	30.780	5.146	4230	24.480	491.610	516.090	177.670	307.800	51.460
150	delta	1	60x8 weeks	Ag80	M	VAC50	25	All	0.881	0.005	0.102	0.108	0.037	0.064	0.011	422.975	2.448	49.161	51.609	17.767	30.780	5.146	4230	24.480	491.610	516.090	177.670	307.800	51.460
150	delta	1.2	60x8 weeks	Ag80	M	VAC50	25	All	0.868	0.004	0.111	0.116	0.040	0.070	0.009	416.713	2.122	53.504	55.626	19.292	33.811	4.530	4167	21.220	535.040	556.260	192.920	338.110	45.300
150	delta	1.2	60x8 weeks	Ag80	M	VAC50	25	All	0.868	0.004	0.111	0.116	0.040	0.070	0.009	416.713	2.122	53.504	55.626	19.292	33.811	4.530	4167	21.220	535.040	556.260	192.920	338.110	45.300
150	delta	1.2	60x8 weeks	Ag80	M	VAC50	25	All	0.868	0.004	0.111	0.116	0.040	0.070	0.009	416.713	2.122	53.504	55.626	19.292	33.811	4.530	4167	21.220	535.040	556.260	192.920	338.110	45.300
150	original	0.6	60x8 weeks	Ag80	M	VAC50	50	All	0.993	0.007	0.002	0.009	0.003	0.003	0.019	476.693	3.562	0.759	4.321	1.489	1.413	9.182	4767	35.620	7.590	43.210	14.890	14.130	91.820
150	original	0.6	60x8 weeks	Ag80	M	VAC50	50	All	0.993	0.007	0.002	0.009	0.003	0.003	0.019	476.693	3.562	0.759	4.321	1.489	1.413	9.182	4767	35.620	7.590	43.210	14.890	14.130	91.820
150	original	0.6	60x8 weeks	Ag80	M	VAC50	50	All	0.993	0.007	0.002	0.009	0.003	0.003	0.019	476.693	3.562	0.759	4.321	1.489	1.413	9.182	4767	35.620	7.590	43.210	14.890	14.130	91.820
150	original	1	60x8 weeks	Ag80	M	VAC50	50	All	0.992	0.008	0.003	0.011	0.004	0.004	0.019	476.064	3.646	1.512	5.158	1.771	1.699	9.005	4761	36.460	15.120	51.580	17.710	16.990	90.050
150	original	1	60x8 weeks	Ag80	M	VAC50	50	All	0.992	0.008	0.003	0.011	0.004	0.004	0.019	476.064	3.646	1.512	5.158	1.771	1.699	9.005	4761	36.460	15.120	51.580	17.710	16.990	90.050
150	original	1	60x8 weeks	Ag80	M	VAC50	50	All	0.992	0.008	0.003	0.011	0.004	0.004	0.019	476.064	3.646	1.512	5.158	1.771	1.699	9.005	4761	36.460	15.120	51.580	17.710	16.990	90.050
150	original	1.2	60x8 weeks	Ag80	M	VAC50	50	All	0.991	0.008	0.004	0.012	0.004	0.004	0.019	475.601	3.744	1.926	5.670	1.958	1.904	9.172	4756	37.440	19.260	56.700	19.580	19.040	91.720
150	original	1.2	60x8 weeks	Ag80	M	VAC50	50	All	0.991	0.008	0.004	0.012	0.004	0.004	0.019	475.601	3.744	1.926	5.670	1.958	1.904	9.172	4756	37.440	19.260	56.700	19.580	19.040	91.720
150	original	1.2	60x8 weeks	Ag80	M	VAC50	50	All	0.991	0.008	0.004	0.012	0.004	0.004	0.019	475.601	3.744	1.926	5.670	1.958	1.904	9.172	4756	37.440	19.260	56.700	19.580	19.040	91.720
150	alpha	0.6	60x8 weeks	Ag80	M	VAC50	50	All	0.988	0.007	0.005	0.013	0.004	0.007	0.019	474.240	3.457	2.639	6.096	1.938	3.304	9.153	4742	34.570	26.390	60.960	19.380	33.040	91.530
150	alpha	0.6	60x8 weeks	Ag80	M	VAC50	50	All	0.988	0.007	0.005	0.013	0.004	0.007	0.019	474.240	3.457	2.639	6.096	1.938	3.304	9.153	4742	34.570	26.390	60.960	19.380	33.040	91.530
150	alpha	0.6	60x8 weeks	Ag80	M	VAC50	50	All	0.988	0.007	0.005	0.013	0.004	0.007	0.019	474.240	3.457	2.639	6.096	1.938	3.304	9.153	4742	34.570	26.390	60.960	19.380	33.040	91.530
150	alpha	1	60x8 weeks	Ag80	M	VAC50	50	All	0.981	0.007	0.013	0.020	0.006	0.011	0.018	470.688	3.406	6.390	9.796	3.038	5.456	8.730	4707	34.060	63.900	97.960	30.380	54.560	87.300
150	alpha	1	60x8 weeks	Ag80	M	VAC50	50	All	0.981	0.007	0.013	0.020	0.006	0.011	0.018	470.688	3.406	6.390	9.796	3.038	5.456	8.730	4707	34.060	63.900	97.960	30.380	54.560	87.300
150	alpha	1	60x8 weeks	Ag80	M	VAC50	50	All	0.981	0.007	0.013	0.020	0.006	0.011	0.018	470.688	3.406	6.390	9.796	3.038	5.456	8.730	4707	34.060	63.900	97.960	30.380	54.560	87.300
150	alpha	1.2	60x8 weeks	Ag80	M	VAC50	50	All	0.974	0.007	0.020	0.027	0.008	0.015	0.018	467.621	3.399	9.634	13.033	4.017	7.343	8.584	4676	33.990	96.340	130.330	40.170	73.430	85.840
150	alpha	1.2	60x8 weeks	Ag80	M	VAC50	50	All	0.974	0.007	0.020	0.027	0.008	0.015	0.018	467.621	3.399	9.634	13.033	4.017	7.343	8.584	4676	33.990	96.340	130.330	40.170	73.430	85.840
150	alpha	1.2	60x8 weeks	Ag80	M	VAC50	50	All	0.974	0.007	0.020	0.027	0.008	0.015	0.018	467.621	3.399	9.634	13.033	4.017	7.343	8.584	4676	33.990	96.340	130.330	40.170	73.430	85.840

150	delta	0.6	60x8 weeks	Ag80	M	VAC50	50	All	0.964	0.007	0.029	0.037	0.012	0.021	0.017	462.563	3.576	13.952	17.528	5.805	9.957	8.323	4626	35.760	139.520	175.280	58.050	99.570	83.230
150	delta	0.6	60x8 weeks	Ag80	M	VAC50	50	All	0.964	0.007	0.029	0.037	0.012	0.021	0.017	462.563	3.576	13.952	17.528	5.805	9.957	8.323	4626	35.760	139.520	175.280	58.050	99.570	83.230
150	delta	0.6	60x8 weeks	Ag80	M	VAC50	50	All	0.964	0.007	0.029	0.037	0.012	0.021	0.017	462.563	3.576	13.952	17.528	5.805	9.957	8.323	4626	35.760	139.520	175.280	58.050	99.570	83.230
150	delta	1	60x8 weeks	Ag80	M	VAC50	50	All	0.908	0.006	0.081	0.087	0.029	0.051	0.013	435.637	2.778	38.936	41.714	14.156	24.476	6.270	4356	27.780	389.360	417.140	141.560	244.760	62.700
150	delta	1	60x8 weeks	Ag80	M	VAC50	50	All	0.908	0.006	0.081	0.087	0.029	0.051	0.013	435.637	2.778	38.936	41.714	14.156	24.476	6.270	4356	27.780	389.360	417.140	141.560	244.760	62.700
150	delta	1	60x8 weeks	Ag80	M	VAC50	50	All	0.908	0.006	0.081	0.087	0.029	0.051	0.013	435.637	2.778	38.936	41.714	14.156	24.476	6.270	4356	27.780	389.360	417.140	141.560	244.760	62.700
150	delta	1.2	60x8 weeks	Ag80	M	VAC50	50	All	0.888	0.005	0.098	0.103	0.035	0.061	0.012	426.077	2.343	46.942	49.285	16.892	29.307	5.541	4261	23.430	469.420	492.850	168.920	293.070	55.410
150	delta	1.2	60x8 weeks	Ag80	M	VAC50	50	All	0.888	0.005	0.098	0.103	0.035	0.061	0.012	426.077	2.343	46.942	49.285	16.892	29.307	5.541	4261	23.430	469.420	492.850	168.920	293.070	55.410
150	delta	1.2	60x8 weeks	Ag80	M	VAC50	50	All	0.888	0.005	0.098	0.103	0.035	0.061	0.012	426.077	2.343	46.942	49.285	16.892	29.307	5.541	4261	23.430	469.420	492.850	168.920	293.070	55.410
150	original	0.6	60x8 weeks	Ag80	M	VAC50	75	All	0.995	0.007	0.001	0.007	0.003	0.001	0.019	477.388	3.182	0.329	3.511	1.467	0.716	9.224	4774	31.820	3.290	35.110	14.670	7.160	92.240
150	original	0.6	60x8 weeks	Ag80	M	VAC50	75	All	0.995	0.007	0.001	0.007	0.003	0.001	0.019	477.388	3.182	0.329	3.511	1.467	0.716	9.224	4774	31.820	3.290	35.110	14.670	7.160	92.240
150	original	0.6	60x8 weeks	Ag80	M	VAC50	75	All	0.995	0.007	0.001	0.007	0.003	0.001	0.019	477.388	3.182	0.329	3.511	1.467	0.716	9.224	4774	31.820	3.290	35.110	14.670	7.160	92.240
150	original	1	60x8 weeks	Ag80	M	VAC50	75	All	0.994	0.006	0.001	0.008	0.003	0.002	0.019	477.343	2.989	0.611	3.600	1.540	0.723	9.351	4773	29.890	6.110	36.000	15.400	7.230	93.510
150	original	1	60x8 weeks	Ag80	M	VAC50	75	All	0.994	0.006	0.001	0.008	0.003	0.002	0.019	477.343	2.989	0.611	3.600	1.540	0.723	9.351	4773	29.890	6.110	36.000	15.400	7.230	93.510
150	original	1	60x8 weeks	Ag80	M	VAC50	75	All	0.994	0.006	0.001	0.008	0.003	0.002	0.019	477.343	2.989	0.611	3.600	1.540	0.723	9.351	4773	29.890	6.110	36.000	15.400	7.230	93.510
150	original	1.2	60x8 weeks	Ag80	M	VAC50	75	All	0.994	0.006	0.002	0.008	0.003	0.002	0.019	477.216	3.090	0.766	3.856	1.586	0.797	9.275	4772	30.900	7.660	38.560	15.860	7.970	92.750
150	original	1.2	60x8 weeks	Ag80	M	VAC50	75	All	0.994	0.006	0.002	0.008	0.003	0.002	0.019	477.216	3.090	0.766	3.856	1.586	0.797	9.275	4772	30.900	7.660	38.560	15.860	7.970	92.750
150	original	1.2	60x8 weeks	Ag80	M	VAC50	75	All	0.994	0.006	0.002	0.008	0.003	0.002	0.019	477.216	3.090	0.766	3.856	1.586	0.797	9.275	4772	30.900	7.660	38.560	15.860	7.970	92.750
150	alpha	0.6	60x8 weeks	Ag80	M	VAC50	75	All	0.991	0.006	0.003	0.009	0.003	0.005	0.019	475.788	3.075	1.366	4.441	1.429	2.324	9.267	4758	30.750	13.660	44.410	14.290	23.240	92.670
150	alpha	0.6	60x8 weeks	Ag80	M	VAC50	75	All	0.991	0.006	0.003	0.009	0.003	0.005	0.019	475.788	3.075	1.366	4.441	1.429	2.324	9.267	4758	30.750	13.660	44.410	14.290	23.240	92.670
150	alpha	0.6	60x8 weeks	Ag80	M	VAC50	75	All	0.991	0.006	0.003	0.009	0.003	0.005	0.019	475.788	3.075	1.366	4.441	1.429	2.324	9.267	4758	30.750	13.660	44.410	14.290	23.240	92.670
150	alpha	1	60x8 weeks	Ag80	M	VAC50	75	All	0.989	0.006	0.006	0.012	0.004	0.007	0.019	474.482	3.014	2.849	5.863	1.897	3.126	9.091	4745	30.140	28.490	58.630	18.970	31.260	90.910
150	alpha	1	60x8 weeks	Ag80	M	VAC50	75	All	0.989	0.006	0.006	0.012	0.004	0.007	0.019	474.482	3.014	2.849	5.863	1.897	3.126	9.091	4745	30.140	28.490	58.630	18.970	31.260	90.910
150	alpha	1	60x8 weeks	Ag80	M	VAC50	75	All	0.989	0.006	0.006	0.012	0.004	0.007	0.019	474.482	3.014	2.849	5.863	1.897	3.126	9.091	4745	30.140	28.490	58.630	18.970	31.260	90.910
150	alpha	1.2	60x8 weeks	Ag80	M	VAC50	75	All	0.986	0.006	0.009	0.015	0.005	0.008	0.019	473.262	3.098	4.313	7.411	2.331	3.930	9.074	4733	30.980	43.130	74.110	23.310	39.300	90.740
150	alpha	1.2	60x8 weeks	Ag80	M	VAC50	75	All	0.986	0.006	0.009	0.015	0.005	0.008	0.019	473.262	3.098	4.313	7.411	2.331	3.930	9.074	4733	30.980	43.130	74.110	23.310	39.300	90.740
150	alpha	1.2	60x8 weeks	Ag80	M	VAC50	75	All	0.986	0.006	0.009	0.015	0.005	0.008	0.019	473.262	3.098	4.313	7.411	2.331	3.930	9.074	4733	30.980	43.130	74.110	23.310	39.300	90.740
150	delta	0.6	60x8 weeks	Ag80	M	VAC50	75	All	0.980	0.007	0.014	0.021	0.007	0.011	0.018	470.340	3.400	6.550	9.950	3.326	5.497	8.677	4703	34.000	65.500	99.500	33.260	54.970	86.770
150	delta	0.6	60x8 weeks	Ag80	M	VAC50	75	All	0.980	0.007	0.014	0.021	0.007	0.011	0.018	470.340	3.400	6.550	9.950	3.326	5.497	8.677	4703	34.000	65.500	99.500	33.260	54.970	86.770
150	delta	0.6	60x8 weeks	Ag80	M	VAC50	75	All	0.980	0.007	0.014	0.021	0.007	0.011	0.018	470.340	3.400	6.550	9.950	3.326	5.497	8.677	4703	34.000	65.500	99.500	33.260	54.970	86.770
150	delta	1	60x8 weeks	Ag80	M	VAC50	75	All	0.945	0.006	0.048	0.054	0.018	0.031	0.015	453.467	3.043	23.021	26.064	8.848	14.711	7.430	4535	30.430	230.210	260.640	88.480	147.110	74.300
150	delta	1	60x8 weeks	Ag80	M	VAC50	75	All	0.945	0.006	0.048	0.054	0.018	0.031	0.015	453.467	3.043	23.021	26.064	8.848	14.711	7.430	4535	30.430	230.210	260.640	88.480	147.110	74.300
150	delta	1	60x8 weeks	Ag80	M	VAC50	75	All	0.945	0.006	0.048	0.054	0.018	0.031	0.015	453.467	3.043	23.021	26.064	8.848	14.711	7.430	4535	30.430	230.210	260.640	88.480	147.110	74.300
150	delta	1.2	60x8 weeks	Ag80	M	VAC50	75	All	0.929	0.006	0.065	0.070	0.024	0.040	0.015	445.909	2.656	31.040	33.696	11.301	19.151	7.009	4459	26.560	310.400	336.960	113.010	191.510	70.090
150	delta	1.2	60x8 weeks	Ag80	M	VAC50	75	All	0.929	0.006	0.065	0.070	0.024	0.040	0.015	445.909	2.656	31.040	33.696	11.301	19.151	7.009	4459	26.560	310.400	336.960	113.010	191.510	70.090
150	delta	1.2	60x8 weeks	Ag80	M	VAC50	75	All	0.929	0.006	0.065	0.070	0.024	0.040	0.015	445.909	2.656	31.040	33.696	11.301	19.151	7.009	4459	26.560	310.400	336.960	113.010	191.510	70.090
150	original	0.6	60x8 weeks	Ag80	M	VAC50	100	All	0.997	0.005	0.000	0.005	0.003	0.000	0.020	478.321	2.560	0.030	2.590	1.264	0.097	9.422	4783	25.600	0.300	25.900	12.640	0.970	94.220
150	original	0.6	60x8 weeks	Ag80	M	VAC50	100	All	0.997	0.005	0.000	0.005	0.003	0.000	0.020	478.321	2.560	0.030	2.590	1.264	0.097	9.422	4783	25.600	0.300	25.900	12.640	0.970	94.220
150	original	0.6	60x8 weeks	Ag80	M	VAC50	100	All	0.997	0.005	0.000	0.005	0.003	0.000	0.020	478.321	2.560	0.030	2.590	1.264	0.097	9.422	4783	25.600	0.300	25.900	12.640	0.970	94.220
150	original	1	60x8 weeks	Ag80	M	VAC50	100	All	0.996	0.005	0.000	0.005	0.003	0.000	0.020	478.214	2.563	0.053	2.616	1.312	0.100	9.536	4782	25.630	0.530	26.160	13.120	1.000	95.360
150	original	1	60x8 weeks	Ag80	M	VAC50	100	All	0.996	0.005	0.000	0.005	0.003	0.000	0.020	478.214	2.563	0.053	2.616	1.312	0.100	9.536	4782	25.630	0.530	26.160	13.120	1.000	95.360
150	original	1	60x8 weeks	Ag80	M	VAC50	100	All	0.996	0.005	0.000	0.005	0.003	0.000	0.020	478.214	2.563	0.053	2.616	1.312	0.100	9.536	4782	25.630	0.530	26.160	13.120	1.000	95.360
150	original	1.2	60x8 weeks	Ag80	M	VAC50	100	All	0.997	0.005	0.000	0.005	0.003	0.000	0.019	478.365	2.471	0.062	2.533	1.224	0.099	9.353	4784	24.710	0.620	25.330	12.240	0.990	93.530
150	original	1.2	60x8 weeks	Ag80	M	VAC50	100	All	0.997	0.005	0.000	0.005	0.003	0.000	0.019	478.365	2.471	0.062	2.533	1.224	0.099	9.353	4784	24.710	0.620	25.330	12.240	0.990	93.530
150	original	1.2	60x8 weeks	Ag80	M	VAC50	100	All	0.997	0.005	0.000	0.005	0.003	0.000	0.019	478.365	2.471	0.062	2.533	1.224	0.099	9.353	4784	24.710	0.620	25.330	12.240	0.990	93.530
150	alpha	0.6	60x8 weeks	Ag80	M	VAC50	100	All	0.994	0.005	0.001	0.006	0.002	0.003	0.019	477.186	2.475	0.566	3.041	0.983	1.576	9.338	4772	24.750	5.660	30.410	9.830	15.760	93.380
150	alpha	0.6	60x8 weeks	Ag80	M	VAC50	100	All	0.994	0.005	0.001	0.006	0.002	0.003	0.019	477.186	2.475	0.566	3.041	0.983	1.576	9.338	4772	24.750	5.660	30.410	9.830	15.760	93.380
150	alpha	0.6	60x8 weeks	Ag80	M	VAC50	100	All	0.994	0.005	0.001	0.006	0.002	0.003	0.019	477.186	2.475	0.566	3.041	0.983	1.576	9.338	4772	24.750	5.660	30.410	9.830	15.760	93.380
150	alpha	1	60x8 weeks	Ag80	M	VAC50	100	All	0.993	0.005	0.003	0.008	0.002	0.004	0.020	476.626	2.442	1.215	3.657	1.188	1.858	9.407	4766	24.420	12.150	36.570	11.880	18.580	94.070
150	alpha	1	60x8 weeks	Ag80	M	VAC50	100	All	0.993	0.005	0.003	0.008	0.002	0.004	0.020	476.626	2.442	1.215	3.657	1.188	1.858	9.407	4766	24.420	12.150	36.570	11.880	18.580	94.070
150	alpha	1	60x8 weeks	Ag80	M	VAC50	100	All	0.993	0.005	0.003	0.008	0.002	0.004	0.020	476.626	2.442	1.215	3.657	1.188	1.858	9.407	4766	24.420	12.150	36.570	11.880	18.580	94.070
150	alpha	1.2	60x8 weeks	Ag80	M	VAC50	100	All	0.992	0.005	0.003	0.008	0.003	0.004	0.019	476.322	2.458	1.484	3.942	1.313	2.002	9.325	4763	24.580	14.840	39.420	13.130	20.020	93.250
150	alpha	1.2	60x8 weeks	Ag80	M	VAC50	100	All	0.992	0.005	0.003	0.008	0.003	0.004	0.019	476.322	2.458	1.484	3.942	1.313	2.002	9.325	4763	24.580	14.840	39.420	13.130	20.020	93.250
150	alpha	1.2	60x8 weeks	Ag80	M	VAC50	100	All	0.992	0.005	0.003	0.008	0.003	0.004	0.019	476.322	2.458	1.484	3.942	1.313	2.002	9.325	4763	24.580	14.840	39.420	13.130	20.020	93.250
150	delta	0.6	60x8 weeks	Ag80	M	VAC50	100	All	0.987	0.007	0.007	0.013	0.005	0.007	0.019	473.575	3.321	3.152	6.473	2.250	3.480	9.149	4736	33.210	31.520	64.730	22.500	34.800	91.490
150	delta	0.6	60x8 weeks	Ag80	M	VAC50	100	All	0.987	0.007	0.007	0.013	0.005	0.007	0.019	473.575	3.321	3.152	6.473	2.250	3.480	9.149	4736	33.210	31.520	64.730	22.500	34.800	91.490
150	delta	0.6	60x8 weeks	Ag80	M	VAC50	100	All	0.987	0.007	0.007	0.013	0.005	0.007	0.019	473.575	3.321	3.152	6.473	2.250	3.480	9.149	4736	33.210	31.520	64.730	22.500	34.800	91.490
150	delta	1	60x8 weeks	Ag80	M	VAC50	100	All	0.975	0.006	0.019	0.025	0.009	0.014	0.018	467.966	3.085	9.096	12.181	4.272	6.643	8.688	4680	30.850	90.960	121.810	42.720	66.430	86.880
150	delta	1	60x8 weeks	Ag80	M	VAC50	100	All	0.975	0.006	0.019	0.025	0.009	0.014	0.018	467.966	3.085	9.096	12.181	4.272	6.643	8.688	4680	30.850	90.960	121.810	42.720	66.430	86.880
150	delta	1	60x8 weeks	Ag80	M	VAC50	100	All	0.975	0.006	0.019	0.025	0.009	0.014	0.018	467.966	3.085	9.096	12.181	4.272	6.643	8.688	4680	30.850	90.960	121.810	42.720	66.430	86.880
150	delta	1.2	60x8 weeks	Ag80	M	VAC50	100	All	0.966	0.006	0.029	0.035	0.011	0.020	0.017	463.714	2.926	13.847	16.773	5.459	9.393	8.314	4637	29.260	138.470	167.730	54.590	93.930	83.140
150	delta	1.2	60x8 weeks	Ag80	M	VAC50	100	All	0.966	0.006	0.029	0.035	0.011	0.020	0.017	463.714	2.926	13.847	16.773	5.459	9.393	8.314	4637	29.260	138.470	167.730	54.590	93.930	83.140
150	delta	1.2	60x8 weeks	Ag80	M	VAC50	100	All	0.966	0.006	0.029	0.035	0.011	0.020	0.017	463.714	2.926	13.847	16.773	5.459	9.393	8.314	4637	29.260	138.470	167.730	54.590	93.930	83.140
150	original	0.6	60x8 weeks	PCR90	Daily	NOVAC	0	Jnvaccinate	4.911	0.010	0.003	0.013	0.001	0.013	0.005	2357.140	4.905	1.309	6.214	0.394	6.083	2.285	23571	49.050	13.090	62.140	3.940	60.830	22.850
150	original	0.6	60x8 weeks	PCR90	Daily	NOVAC	0	Jnvaccinate	4.911	0.010	0.003	0.013	0.001	0.013	0.005	2357.140	4.905	1.309	6.214	0.394	6.083	2.285	23571	49.050	13.090	62.140	3.940	60.830	22.850
150	original	0.6	60x8 weeks	PCR90	Daily	NOVAC	0	Jnvaccinate	4.911	0.010	0.003	0.013	0.001	0.013	0.005	2357.140	4.905	1.309	6.214	0.394	6.083	2.285	23571	49.050	13.090	62.140	3.940	60.830	22.850
150	original	1	60x8 weeks	PCR90	Daily	NOVAC	0	Jnvaccinate	4.892	0.010	0.006	0.015	0.001	0.015	0.004	2348.153	4.709	2.706	7.415	0.484	7.334	2.155	23482	47.090	27.060	74.150	4.840	73.340	21.550
150	original	1	60x8 weeks	PCR90	Daily	NOVAC	0	Jnvaccinate	4.892	0.010	0.006	0.015	0.001	0.015	0.004	2348.153	4.709	2.706	7.415	0.484	7.334	2.155	23482	47.090	27.060	74.150	4.840	73.340	21.550
150	original	1	60x8 weeks	PCR90	Daily	NOVAC	0	Jnvaccinate	4.892	0.010	0.006	0.015	0.001	0.015	0.004	2348.153	4.709	2.706	7.415	0.484	7.334	2.155	23482	47.090	27.060	74.150	4.840	73.340	21.550
150	original	1.2	60x8 weeks	PCR90	Daily	NOVAC	0	Jnvaccinate	4.878	0.010	0.008	0.018	0.001	0.017	0.004	2341.460	4.775	3.633	8.408	0.539	8.281	2.157	23415	47.750	36.330	84.080	5.390	82.810	21.570
150	original	1.2	60x8 weeks	PCR90	Daily	NOVAC	0	Jnvaccinate	4.878	0.010	0.008	0.018	0.001	0.017	0.004	2341.460	4.775	3.633	8.408	0.539	8.281	2.157	23415	47.750	36.330	84.080	5.390	82.810	21.570

150	original	1.2	60x8 weeks	PCR90	Daily	NOVAC	0	Jnvaccinate	4.878	0.010	0.008	0.018	0.001	0.017	0.004	2341.460	4.775	3.633	8.408	0.539	8.281	2.157	23415	47.750	36.330	84.080	5.390	82.810	21.570
150	alpha	0.6	60x8 weeks	PCR90	Daily	NOVAC	0	Jnvaccinate	4.883	0.010	0.007	0.017	0.001	0.016	0.005	2343.645	4.722	3.200	7.922	0.583	7.878	2.162	23436	47.220	32.000	79.220	5.830	78.780	21.620
150	alpha	0.6	60x8 weeks	PCR90	Daily	NOVAC	0	Jnvaccinate	4.883	0.010	0.007	0.017	0.001	0.016	0.005	2343.645	4.722	3.200	7.922	0.583	7.878	2.162	23436	47.220	32.000	79.220	5.830	78.780	21.620
150	alpha	0.6	60x8 weeks	PCR90	Daily	NOVAC	0	Jnvaccinate	4.883	0.010	0.007	0.017	0.001	0.016	0.005	2343.645	4.722	3.200	7.922	0.583	7.878	2.162	23436	47.220	32.000	79.220	5.830	78.780	21.620
150	alpha	1	60x8 weeks	PCR90	Daily	NOVAC	0	Jnvaccinate	4.815	0.010	0.017	0.026	0.002	0.026	0.004	2311.310	4.562	8.083	12.645	0.852	12.688	2.115	23113	45.620	80.830	126.450	8.520	126.880	21.150
150	alpha	1	60x8 weeks	PCR90	Daily	NOVAC	0	Jnvaccinate	4.815	0.010	0.017	0.026	0.002	0.026	0.004	2311.310	4.562	8.083	12.645	0.852	12.688	2.115	23113	45.620	80.830	126.450	8.520	126.880	21.150
150	alpha	1	60x8 weeks	PCR90	Daily	NOVAC	0	Jnvaccinate	4.815	0.010	0.017	0.026	0.002	0.026	0.004	2311.310	4.562	8.083	12.645	0.852	12.688	2.115	23113	45.620	80.830	126.450	8.520	126.880	21.150
150	alpha	1.2	60x8 weeks	PCR90	Daily	NOVAC	0	Jnvaccinate	4.755	0.009	0.025	0.034	0.002	0.035	0.004	2282.631	4.358	12.162	16.520	1.191	16.587	2.114	22826	43.580	121.620	165.200	11.910	165.870	21.140
150	alpha	1.2	60x8 weeks	PCR90	Daily	NOVAC	0	Jnvaccinate	4.755	0.009	0.025	0.034	0.002	0.035	0.004	2282.631	4.358	12.162	16.520	1.191	16.587	2.114	22826	43.580	121.620	165.200	11.910	165.870	21.140
150	alpha	1.2	60x8 weeks	PCR90	Daily	NOVAC	0	Jnvaccinate	4.755	0.009	0.025	0.034	0.002	0.035	0.004	2282.631	4.358	12.162	16.520	1.191	16.587	2.114	22826	43.580	121.620	165.200	11.910	165.870	21.140
150	delta	0.6	60x8 weeks	PCR90	Daily	NOVAC	0	Jnvaccinate	4.693	0.009	0.033	0.042	0.004	0.041	0.004	2252.829	4.169	16.008	20.177	2.031	19.846	1.896	22528	41.690	160.080	201.770	20.310	198.460	18.960
150	delta	0.6	60x8 weeks	PCR90	Daily	NOVAC	0	Jnvaccinate	4.693	0.009	0.033	0.042	0.004	0.041	0.004	2252.829	4.169	16.008	20.177	2.031	19.846	1.896	22528	41.690	160.080	201.770	20.310	198.460	18.960
150	delta	0.6	60x8 weeks	PCR90	Daily	NOVAC	0	Jnvaccinate	4.693	0.009	0.033	0.042	0.004	0.041	0.004	2252.829	4.169	16.008	20.177	2.031	19.846	1.896	22528	41.690	160.080	201.770	20.310	198.460	18.960
150	delta	1	60x8 weeks	PCR90	Daily	NOVAC	0	Jnvaccinate	4.244	0.006	0.093	0.099	0.011	0.099	0.003	2037.095	2.841	44.758	47.599	5.089	47.469	1.325	20371	28.410	447.580	475.990	50.890	474.690	13.250
150	delta	1	60x8 weeks	PCR90	Daily	NOVAC	0	Jnvaccinate	4.244	0.006	0.093	0.099	0.011	0.099	0.003	2037.095	2.841	44.758	47.599	5.089	47.469	1.325	20371	28.410	447.580	475.990	50.890	474.690	13.250
150	delta	1	60x8 weeks	PCR90	Daily	NOVAC	0	Jnvaccinate	4.244	0.006	0.093	0.099	0.011	0.099	0.003	2037.095	2.841	44.758	47.599	5.089	47.469	1.325	20371	28.410	447.580	475.990	50.890	474.690	13.250
150	delta	1.2	60x8 weeks	PCR90	Daily	NOVAC	0	Jnvaccinate	4.121	0.005	0.108	0.113	0.012	0.113	0.002	1977.860	2.371	51.946	54.317	5.885	54.338	1.105	19779	23.710	519.460	543.170	58.850	543.380	11.050
150	delta	1.2	60x8 weeks	PCR90	Daily	NOVAC	0	Jnvaccinate	4.121	0.005	0.108	0.113	0.012	0.113	0.002	1977.860	2.371	51.946	54.317	5.885	54.338	1.105	19779	23.710	519.460	543.170	58.850	543.380	11.050
150	delta	1.2	60x8 weeks	PCR90	Daily	NOVAC	0	Jnvaccinate	4.121	0.005	0.108	0.113	0.012	0.113	0.002	1977.860	2.371	51.946	54.317	5.885	54.338	1.105	19779	23.710	519.460	543.170	58.850	543.380	11.050
150	original	0.6	60x8 weeks	PCR90	Daily	VAC95	25	Jnvaccinate	3.688	0.008	0.002	0.009	0.001	0.009	0.004	1770.057	3.681	0.724	4.405	0.321	4.234	1.748	17701	36.810	7.240	44.050	3.210	42.340	17.480
150	original	0.6	60x8 weeks	PCR90	Daily	VAC95	25	Jnvaccinate	3.688	0.008	0.002	0.009	0.001	0.009	0.004	1770.057	3.681	0.724	4.405	0.321	4.234	1.748	17701	36.810	7.240	44.050	3.210	42.340	17.480
150	original	0.6	60x8 weeks	PCR90	Daily	VAC95	25	Jnvaccinate	3.688	0.008	0.002	0.009	0.001	0.009	0.004	1770.057	3.681	0.724	4.405	0.321	4.234	1.748	17701	36.810	7.240	44.050	3.210	42.340	17.480
150	original	1	60x8 weeks	PCR90	Daily	VAC95	25	Jnvaccinate	3.681	0.008	0.003	0.010	0.001	0.010	0.004	1767.014	3.651	1.339	4.990	0.353	4.734	1.722	17670	36.510	13.390	49.900	3.530	47.340	17.220
150	original	1	60x8 weeks	PCR90	Daily	VAC95	25	Jnvaccinate	3.681	0.008	0.003	0.010	0.001	0.010	0.004	1767.014	3.651	1.339	4.990	0.353	4.734	1.722	17670	36.510	13.390	49.900	3.530	47.340	17.220
150	original	1	60x8 weeks	PCR90	Daily	VAC95	25	Jnvaccinate	3.681	0.008	0.003	0.010	0.001	0.010	0.004	1767.014	3.651	1.339	4.990	0.353	4.734	1.722	17670	36.510	13.390	49.900	3.530	47.340	17.220
150	original	1.2	60x8 weeks	PCR90	Daily	VAC95	25	Jnvaccinate	3.675	0.008	0.004	0.011	0.001	0.011	0.004	1764.009	3.608	1.711	5.319	0.374	5.140	1.724	17640	36.080	17.110	53.190	3.740	51.400	17.240
150	original	1.2	60x8 weeks	PCR90	Daily	VAC95	25	Jnvaccinate	3.675	0.008	0.004	0.011	0.001	0.011	0.004	1764.009	3.608	1.711	5.319	0.374	5.140	1.724	17640	36.080	17.110	53.190	3.740	51.400	17.240
150	original	1.2	60x8 weeks	PCR90	Daily	VAC95	25	Jnvaccinate	3.675	0.008	0.004	0.011	0.001	0.011	0.004	1764.009	3.608	1.711	5.319	0.374	5.140	1.724	17640	36.080	17.110	53.190	3.740	51.400	17.240
150	alpha	0.6	60x8 weeks	PCR90	Daily	VAC95	25	Jnvaccinate	3.672	0.008	0.004	0.011	0.001	0.011	0.003	1762.732	3.717	1.736	5.453	0.444	5.343	1.670	17627	37.170	17.360	54.530	4.440	53.430	16.700
150	alpha	0.6	60x8 weeks	PCR90	Daily	VAC95	25	Jnvaccinate	3.672	0.008	0.004	0.011	0.001	0.011	0.003	1762.732	3.717	1.736	5.453	0.444	5.343	1.670	17627	37.170	17.360	54.530	4.440	53.430	16.700
150	alpha	0.6	60x8 weeks	PCR90	Daily	VAC95	25	Jnvaccinate	3.672	0.008	0.004	0.011	0.001	0.011	0.003	1762.732	3.717	1.736	5.453	0.444	5.343	1.670	17627	37.170	17.360	54.530	4.440	53.430	16.700
150	alpha	1	60x8 weeks	PCR90	Daily	VAC95	25	Jnvaccinate	3.650	0.007	0.007	0.014	0.001	0.014	0.003	1752.024	3.378	3.575	6.953	0.576	6.789	1.608	17520	33.780	35.750	69.530	5.760	67.890	16.080
150	alpha	1	60x8 weeks	PCR90	Daily	VAC95	25	Jnvaccinate	3.650	0.007	0.007	0.014	0.001	0.014	0.003	1752.024	3.378	3.575	6.953	0.576	6.789	1.608	17520	33.780	35.750	69.530	5.760	67.890	16.080
150	alpha	1	60x8 weeks	PCR90	Daily	VAC95	25	Jnvaccinate	3.650	0.007	0.007	0.014	0.001	0.014	0.003	1752.024	3.378	3.575	6.953	0.576	6.789	1.608	17520	33.780	35.750	69.530	5.760	67.890	16.080
150	alpha	1.2	60x8 weeks	PCR90	Daily	VAC95	25	Jnvaccinate	3.625	0.007	0.011	0.018	0.002	0.018	0.003	1739.805	3.599	5.206	8.805	0.782	8.526	1.604	17398	35.990	52.060	88.050	7.820	85.260	16.040
150	alpha	1.2	60x8 weeks	PCR90	Daily	VAC95	25	Jnvaccinate	3.625	0.007	0.011	0.018	0.002	0.018	0.003	1739.805	3.599	5.206	8.805	0.782	8.526	1.604	17398	35.990	52.060	88.050	7.820	85.260	16.040
150	alpha	1.2	60x8 weeks	PCR90	Daily	VAC95	25	Jnvaccinate	3.625	0.007	0.011	0.018	0.002	0.018	0.003	1739.805	3.599	5.206	8.805	0.782	8.526	1.604	17398	35.990	52.060	88.050	7.820	85.260	16.040
150	delta	0.6	60x8 weeks	PCR90	Daily	VAC95	25	Jnvaccinate	3.603	0.007	0.014	0.022	0.003	0.020	0.003	1729.645	3.530	6.882	10.412	1.355	9.595	1.590	17296	35.300	68.820	104.120	13.550	95.950	15.900
150	delta	0.6	60x8 weeks	PCR90	Daily	VAC95	25	Jnvaccinate	3.603	0.007	0.014	0.022	0.003	0.020	0.003	1729.645	3.530	6.882	10.412	1.355	9.595	1.590	17296	35.300	68.820	104.120	13.550	95.950	15.900
150	delta	0.6	60x8 weeks	PCR90	Daily	VAC95	25	Jnvaccinate	3.603	0.007	0.014	0.022	0.003	0.020	0.003	1729.645	3.530	6.882	10.412	1.355	9.595	1.590	17296	35.300	68.820	104.120	13.550	95.950	15.900
150	delta	1	60x8 weeks	PCR90	Daily	VAC95	25	Jnvaccinate	3.397	0.006	0.046	0.052	0.007	0.047	0.003	1630.593	2.945	21.891	24.836	3.427	22.616	1.348	16306	29.450	218.910	248.360	34.270	226.160	13.480
150	delta	1	60x8 weeks	PCR90	Daily	VAC95	25	Jnvaccinate	3.397	0.006	0.046	0.052	0.007	0.047	0.003	1630.593	2.945	21.891	24.836	3.427	22.616	1.348	16306	29.450	218.910	248.360	34.270	226.160	13.480
150	delta	1	60x8 weeks	PCR90	Daily	VAC95	25	Jnvaccinate	3.397	0.006	0.046	0.052	0.007	0.047	0.003	1630.593	2.945	21.891	24.836	3.427	22.616	1.348	16306	29.450	218.910	248.360	34.270	226.160	13.480
150	delta	1.2	60x8 weeks	PCR90	Daily	VAC95	25	Jnvaccinate	3.287	0.005	0.063	0.069	0.010	0.061	0.003	1577.971	2.599	30.324	32.923	4.909	29.424	1.220	15780	25.990	303.240	329.230	49.090	294.240	12.200
150	delta	1.2	60x8 weeks	PCR90	Daily	VAC95	25	Jnvaccinate	3.287	0.005	0.063	0.069	0.010	0.061	0.003	1577.971	2.599	30.324	32.923	4.909	29.424	1.220	15780	25.990	303.240	329.230	49.090	294.240	12.200
150	delta	1.2	60x8 weeks	PCR90	Daily	VAC95	25	Jnvaccinate	3.287	0.005	0.063	0.069	0.010	0.061	0.003	1577.971	2.599	30.324	32.923	4.909	29.424	1.220	15780	25.990	303.240	329.230	49.090	294.240	12.200
150	original	0.6	60x8 weeks	PCR90	Daily	VAC95	50	Jnvaccinate	2.461	0.005	0.001	0.006	0.001	0.006	0.002	1181.325	2.636	0.289	2.925	0.257	2.670	1.155	11813	26.360	2.890	29.250	2.570	26.700	11.550
150	original	0.6	60x8 weeks	PCR90	Daily	VAC95	50	Jnvaccinate	2.461	0.005	0.001	0.006	0.001	0.006	0.002	1181.325	2.636	0.289	2.925	0.257	2.670	1.155	11813	26.360	2.890	29.250	2.570	26.700	11.550
150	original	0.6	60x8 weeks	PCR90	Daily	VAC95	50	Jnvaccinate	2.461	0.005	0.001	0.006	0.001	0.006	0.002	1181.325	2.636	0.289	2.925	0.257	2.670	1.155	11813	26.360	2.890	29.250	2.570	26.700	11.550
150	original	1	60x8 weeks	PCR90	Daily	VAC95	50	Jnvaccinate	2.458	0.005	0.001	0.007	0.001	0.006	0.002	1179.720	2.554	0.579	3.133	0.269	2.892	1.143	11797	25.540	5.790	31.330	2.690	28.920	11.430
150	original	1	60x8 weeks	PCR90	Daily	VAC95	50	Jnvaccinate	2.458	0.005	0.001	0.007	0.001	0.006	0.002	1179.720	2.554	0.579	3.133	0.269	2.892	1.143	11797	25.540	5.790	31.330	2.690	28.920	11.430
150	original	1	60x8 weeks	PCR90	Daily	VAC95	50	Jnvaccinate	2.458	0.005	0.001	0.007	0.001	0.006	0.002	1179.720	2.554	0.579	3.133	0.269	2.892	1.143	11797	25.540	5.790	31.330	2.690	28.920	11.430
150	original	1.2	60x8 weeks	PCR90	Daily	VAC95	50	Jnvaccinate	2.456	0.005	0.001	0.007	0.001	0.006	0.002	1178.677	2.618	0.669	3.287	0.295	3.017	1.158	11787	26.180	6.690	32.870	2.950	30.170	11.580
150	original	1.2	60x8 weeks	PCR90	Daily	VAC95	50	Jnvaccinate	2.456	0.005	0.001	0.007	0.001	0.006	0.002	1178.677	2.618	0.669	3.287	0.295	3.017	1.158	11787	26.180	6.690	32.870	2.950	30.170	11.580
150	original	1.2	60x8 weeks	PCR90	Daily	VAC95	50	Jnvaccinate	2.456	0.005	0.001	0.007	0.001	0.006	0.002	1178.677	2.618	0.669	3.287	0.295	3.017	1.158	11787	26.180	6.690	32.870	2.950	30.170	11.580
150	alpha	0.6	60x8 weeks	PCR90	Daily	VAC95	50	Jnvaccinate	2.457	0.005	0.002	0.007	0.001	0.006	0.002	1179.310	2.468	0.772	3.240	0.353	2.934	1.149	11793	24.680	7.720	32.400	3.530	29.340	11.490
150	alpha	0.6	60x8 weeks	PCR90	Daily	VAC95	50	Jnvaccinate	2.457	0.005	0.002	0.007	0.001	0.006	0.002	1179.310	2.468	0.772	3.240	0.353	2.934	1.149	11793	24.680	7.720	32.400	3.530	29.340	11.490
150	alpha	0.6	60x8 weeks	PCR90	Daily	VAC95	50	Jnvaccinate	2.457	0.005	0.002	0.007	0.001	0.006	0.002	1179.310	2.468	0.772	3.240	0.353	2.934	1.149	11793	24.680	7.720	32.400	3.530	29.340	11.490
150	alpha	1	60x8 weeks	PCR90	Daily	VAC95	50	Jnvaccinate	2.446	0.005	0.003	0.008	0.001	0.008	0.002	1174.060	2.589	1.451	4.040	0.420	3.726	1.107	11741	25.890	14.510	40.400	4.200	37.260	11.070
150	alpha	1	60x8 weeks	PCR90	Daily	VAC95	50	Jnvaccinate	2.446	0.005	0.003	0.008	0.001	0.008	0.002	1174.060	2.589	1.451	4.040	0.420	3.726	1.107	11741	25.890	14.510	40.400	4.200	37.260	11.070
150	alpha	1	60x8 weeks	PCR90	Daily	VAC95	50	Jnvaccinate	2.446	0.005	0.003	0.008	0.001	0.008	0.002	1174.060	2.589	1.451	4.040	0.420	3.726	1.107	11741	25.890	14.510	40.400	4.200	37.260	11.070
150	alpha	1.2	60x8 weeks	PCR90	Daily	VAC95	50	Jnvaccinate	2.440	0.005	0.004	0.009	0.001	0.009	0.002	1171.236	2.554	1.999	4.553	0.508	4.093	1.128	11712	25.540	19.990	45.530	5.080	40.930	11.280
150	alpha	1.2	60x8 weeks	PCR90	Daily	VAC95	50	Jnvaccinate	2.440	0.005	0.004	0.009	0.001	0.009	0.002	1171.236	2.554	1.999	4.553	0.508	4.093	1.128	11712	25.540	19.990	45.530	5.080	40.930	11.280
150	alpha	1.2	60x8 weeks	PCR90	Daily	VAC95	50	Jnvaccinate	2.440	0.005	0.004	0.009	0.001	0.009	0.002	1171.236	2.554	1.999	4.553	0.508	4.093	1.128	11712	25.540	19.990	45.530	5.080	40.930	11.280
150	delta	0.6	60x8 weeks	PCR90	Daily	VAC95	50	Jnvaccinate	2.431	0.006	0.006	0.011	0.002	0.009	0.002	1166.979	2.662	2.744	5.406	0.902	4.506	1.105	11670	26.620	27.440	54.060	9.020	45.060	11.050
150	delta	0.6	60x8 weeks	PCR90	Daily	VAC95	50	Jnvaccinate	2.431	0.006	0.006	0.011	0.002	0.009	0.002	1166.979	2.662	2.744	5.406	0.902	4.506	1.105	11670	26.620	27.440	54.060	9.020	45.060	11.050
150	delta	0.6	60x8 weeks	PCR90	Daily	VAC95	50	Jnvaccinate	2.431	0.006	0.006	0.011	0.002	0.009	0.002	1166.979	2.662	2.744	5.406	0.902	4.506	1.105	11670	26.620	27.440	54.060	9.020	45.060	11.050
150	delta	1	60x8 weeks	PCR90	Daily	VAC95	50	Jnvaccinate	2.371	0.005	0.016	0.021	0.004	0.017	0.002	1138.310	2.577	7.618	10.195	1.777	8.367	1.022	11383	25.770	76.180	101.950	17.770	83.670	10.220
150	delta	1	60x8 weeks	PCR90	Daily	VAC95	50	Jnvaccinate	2.371	0.005	0.016	0.021	0.004	0.017	0.002	1138.310	2.577	7.618	10.195	1.777	8.367	1.022	11383	25.770	76.180	101.950	17.770	83.670	10.220
150	delta	1	60x8 weeks	PCR90	Daily	VAC95	50	Jnvaccinate	2.371	0.005	0.016	0.021	0.004	0.017	0.002	1138.310	2.577	7.618	10.195	1.777	8.367	1.022	11383	25.770	76.180	101.950	17.770	83.670	10.220
150	delta	1.2	60x8 weeks	PCR90	Daily	VAC95	50	Jnvaccinate	2.321	0.005	0.025	0.030	0.006	0.024	0.002	1114.071	2.386	12.095	14.481	2.690	11.522	0.963	11141	23.860	120.950	144.810	26.900	115.220	9.630

150	delta	1.2	60x8 weeks	PCR90	Daily	VAC95	50	Unvaccinate	2.321	0.005	0.025	0.030	0.006	0.024	0.002	1114.071	2.386	12.095	14.481	2.690	11.522	0.963	11141	23.860	120.950	144.810	26.900	115.220	9.630
150	delta	1.2	60x8 weeks	PCR90	Daily	VAC95	50	Unvaccinate	2.321	0.005	0.025	0.030	0.006	0.024	0.002	1114.071	2.386	12.095	14.481	2.690	11.522	0.963	11141	23.860	120.950	144.810	26.900	115.220	9.630
150	original	0.6	60x8 weeks	PCR90	Daily	VAC95	75	Unvaccinate	1.232	0.003	0.000	0.003	0.000	0.003	0.001	591.319	1.393	0.101	1.494	0.171	1.234	0.593	5913	13.930	1.010	14.940	1.710	12.340	5.930
150	original	0.6	60x8 weeks	PCR90	Daily	VAC95	75	Unvaccinate	1.232	0.003	0.000	0.003	0.000	0.003	0.001	591.319	1.393	0.101	1.494	0.171	1.234	0.593	5913	13.930	1.010	14.940	1.710	12.340	5.930
150	original	0.6	60x8 weeks	PCR90	Daily	VAC95	75	Unvaccinate	1.232	0.003	0.000	0.003	0.000	0.003	0.001	591.319	1.393	0.101	1.494	0.171	1.234	0.593	5913	13.930	1.010	14.940	1.710	12.340	5.930
150	original	1	60x8 weeks	PCR90	Daily	VAC95	75	Unvaccinate	1.230	0.003	0.000	0.003	0.000	0.003	0.001	590.414	1.489	0.154	1.643	0.177	1.357	0.577	5904	14.890	1.540	16.430	1.770	13.570	5.770
150	original	1	60x8 weeks	PCR90	Daily	VAC95	75	Unvaccinate	1.230	0.003	0.000	0.003	0.000	0.003	0.001	590.414	1.489	0.154	1.643	0.177	1.357	0.577	5904	14.890	1.540	16.430	1.770	13.570	5.770
150	original	1	60x8 weeks	PCR90	Daily	VAC95	75	Unvaccinate	1.230	0.003	0.000	0.003	0.000	0.003	0.001	590.414	1.489	0.154	1.643	0.177	1.357	0.577	5904	14.890	1.540	16.430	1.770	13.570	5.770
150	original	1.2	60x8 weeks	PCR90	Daily	VAC95	75	Unvaccinate	1.231	0.003	0.000	0.003	0.000	0.003	0.001	590.766	1.465	0.169	1.634	0.223	1.307	0.587	5908	14.650	1.690	16.340	2.230	13.070	5.870
150	original	1.2	60x8 weeks	PCR90	Daily	VAC95	75	Unvaccinate	1.231	0.003	0.000	0.003	0.000	0.003	0.001	590.766	1.465	0.169	1.634	0.223	1.307	0.587	5908	14.650	1.690	16.340	2.230	13.070	5.870
150	original	1.2	60x8 weeks	PCR90	Daily	VAC95	75	Unvaccinate	1.231	0.003	0.000	0.003	0.000	0.003	0.001	590.766	1.465	0.169	1.634	0.223	1.307	0.587	5908	14.650	1.690	16.340	2.230	13.070	5.870
150	alpha	0.6	60x8 weeks	PCR90	Daily	VAC95	75	Unvaccinate	1.229	0.003	0.001	0.004	0.001	0.003	0.001	589.975	1.556	0.241	1.797	0.275	1.419	0.559	5900	15.560	2.410	17.970	2.750	14.190	5.590
150	alpha	0.6	60x8 weeks	PCR90	Daily	VAC95	75	Unvaccinate	1.229	0.003	0.001	0.004	0.001	0.003	0.001	589.975	1.556	0.241	1.797	0.275	1.419	0.559	5900	15.560	2.410	17.970	2.750	14.190	5.590
150	alpha	0.6	60x8 weeks	PCR90	Daily	VAC95	75	Unvaccinate	1.229	0.003	0.001	0.004	0.001	0.003	0.001	589.975	1.556	0.241	1.797	0.275	1.419	0.559	5900	15.560	2.410	17.970	2.750	14.190	5.590
150	alpha	1	60x8 weeks	PCR90	Daily	VAC95	75	Unvaccinate	1.227	0.003	0.001	0.004	0.001	0.003	0.001	589.078	1.505	0.477	1.982	0.318	1.544	0.575	5891	15.050	4.770	19.820	3.180	15.440	5.750
150	alpha	1	60x8 weeks	PCR90	Daily	VAC95	75	Unvaccinate	1.227	0.003	0.001	0.004	0.001	0.003	0.001	589.078	1.505	0.477	1.982	0.318	1.544	0.575	5891	15.050	4.770	19.820	3.180	15.440	5.750
150	alpha	1	60x8 weeks	PCR90	Daily	VAC95	75	Unvaccinate	1.227	0.003	0.001	0.004	0.001	0.003	0.001	589.078	1.505	0.477	1.982	0.318	1.544	0.575	5891	15.050	4.770	19.820	3.180	15.440	5.750
150	alpha	1.2	60x8 weeks	PCR90	Daily	VAC95	75	Unvaccinate	1.226	0.003	0.001	0.004	0.001	0.003	0.001	588.544	1.554	0.562	2.116	0.364	1.622	0.590	5885	15.540	5.620	21.160	3.640	16.220	5.900
150	alpha	1.2	60x8 weeks	PCR90	Daily	VAC95	75	Unvaccinate	1.226	0.003	0.001	0.004	0.001	0.003	0.001	588.544	1.554	0.562	2.116	0.364	1.622	0.590	5885	15.540	5.620	21.160	3.640	16.220	5.900
150	alpha	1.2	60x8 weeks	PCR90	Daily	VAC95	75	Unvaccinate	1.226	0.003	0.001	0.004	0.001	0.003	0.001	588.544	1.554	0.562	2.116	0.364	1.622	0.590	5885	15.540	5.620	21.160	3.640	16.220	5.900
150	delta	0.6	60x8 weeks	PCR90	Daily	VAC95	75	Unvaccinate	1.224	0.004	0.002	0.005	0.001	0.004	0.001	587.545	1.698	0.901	2.599	0.642	1.711	0.575	5875	16.980	9.010	25.990	6.420	17.110	5.750
150	delta	0.6	60x8 weeks	PCR90	Daily	VAC95	75	Unvaccinate	1.224	0.004	0.002	0.005	0.001	0.004	0.001	587.545	1.698	0.901	2.599	0.642	1.711	0.575	5875	16.980	9.010	25.990	6.420	17.110	5.750
150	delta	0.6	60x8 weeks	PCR90	Daily	VAC95	75	Unvaccinate	1.224	0.004	0.002	0.005	0.001	0.004	0.001	587.545	1.698	0.901	2.599	0.642	1.711	0.575	5875	16.980	9.010	25.990	6.420	17.110	5.750
150	delta	1	60x8 weeks	PCR90	Daily	VAC95	75	Unvaccinate	1.215	0.003	0.004	0.007	0.002	0.005	0.001	583.319	1.644	1.887	3.531	0.888	2.254	0.539	5833	16.440	18.870	35.310	8.880	22.540	5.390
150	delta	1	60x8 weeks	PCR90	Daily	VAC95	75	Unvaccinate	1.215	0.003	0.004	0.007	0.002	0.005	0.001	583.319	1.644	1.887	3.531	0.888	2.254	0.539	5833	16.440	18.870	35.310	8.880	22.540	5.390
150	delta	1	60x8 weeks	PCR90	Daily	VAC95	75	Unvaccinate	1.215	0.003	0.004	0.007	0.002	0.005	0.001	583.319	1.644	1.887	3.531	0.888	2.254	0.539	5833	16.440	18.870	35.310	8.880	22.540	5.390
150	delta	1.2	60x8 weeks	PCR90	Daily	VAC95	75	Unvaccinate	1.210	0.003	0.005	0.009	0.002	0.006	0.001	580.652	1.640	2.626	4.266	1.135	2.640	0.497	5807	16.400	26.260	42.660	11.350	26.400	4.970
150	delta	1.2	60x8 weeks	PCR90	Daily	VAC95	75	Unvaccinate	1.210	0.003	0.005	0.009	0.002	0.006	0.001	580.652	1.640	2.626	4.266	1.135	2.640	0.497	5807	16.400	26.260	42.660	11.350	26.400	4.970
150	delta	1.2	60x8 weeks	PCR90	Daily	VAC95	75	Unvaccinate	1.210	0.003	0.005	0.009	0.002	0.006	0.001	580.652	1.640	2.626	4.266	1.135	2.640	0.497	5807	16.400	26.260	42.660	11.350	26.400	4.970
150	original	0.6	60x8 weeks	PCR90	Daily	VAC95	100	Unvaccinate	0.000	0.001	0.000	0.001	0.000	0.000	0.000	0.000	0.345	0.001	0.346	0.135	0.000	0.000	0	3.450	0.010	3.460	1.350	0.000	0.000
150	original	0.6	60x8 weeks	PCR90	Daily	VAC95	100	Unvaccinate	0.000	0.001	0.000	0.001	0.000	0.000	0.000	0.000	0.345	0.001	0.346	0.135	0.000	0.000	0	3.450	0.010	3.460	1.350	0.000	0.000
150	original	0.6	60x8 weeks	PCR90	Daily	VAC95	100	Unvaccinate	0.000	0.001	0.000	0.001	0.000	0.000	0.000	0.000	0.345	0.001	0.346	0.135	0.000	0.000	0	3.450	0.010	3.460	1.350	0.000	0.000
150	original	1	60x8 weeks	PCR90	Daily	VAC95	100	Unvaccinate	0.000	0.001	0.000	0.001	0.000	0.000	0.000	0.000	0.346	0.001	0.347	0.116	0.000	0.000	0	3.460	0.010	3.470	1.160	0.000	0.000
150	original	1	60x8 weeks	PCR90	Daily	VAC95	100	Unvaccinate	0.000	0.001	0.000	0.001	0.000	0.000	0.000	0.000	0.346	0.001	0.347	0.116	0.000	0.000	0	3.460	0.010	3.470	1.160	0.000	0.000
150	original	1	60x8 weeks	PCR90	Daily	VAC95	100	Unvaccinate	0.000	0.001	0.000	0.001	0.000	0.000	0.000	0.000	0.346	0.001	0.347	0.116	0.000	0.000	0	3.460	0.010	3.470	1.160	0.000	0.000
150	original	1.2	60x8 weeks	PCR90	Daily	VAC95	100	Unvaccinate	0.000	0.001	0.000	0.001	0.000	0.000	0.000	0.000	0.361	0.002	0.363	0.140	0.000	0.000	0	3.610	0.020	3.630	1.400	0.000	0.000
150	original	1.2	60x8 weeks	PCR90	Daily	VAC95	100	Unvaccinate	0.000	0.001	0.000	0.001	0.000	0.000	0.000	0.000	0.361	0.002	0.363	0.140	0.000	0.000	0	3.610	0.020	3.630	1.400	0.000	0.000
150	original	1.2	60x8 weeks	PCR90	Daily	VAC95	100	Unvaccinate	0.000	0.001	0.000	0.001	0.000	0.000	0.000	0.000	0.361	0.002	0.363	0.140	0.000	0.000	0	3.610	0.020	3.630	1.400	0.000	0.000
150	alpha	0.6	60x8 weeks	PCR90	Daily	VAC95	100	Unvaccinate	0.000	0.001	0.000	0.001	0.000	0.000	0.000	0.000	0.443	0.018	0.461	0.228	0.000	0.000	0	4.430	0.180	4.610	2.280	0.000	0.000
150	alpha	0.6	60x8 weeks	PCR90	Daily	VAC95	100	Unvaccinate	0.000	0.001	0.000	0.001	0.000	0.000	0.000	0.000	0.443	0.018	0.461	0.228	0.000	0.000	0	4.430	0.180	4.610	2.280	0.000	0.000
150	alpha	0.6	60x8 weeks	PCR90	Daily	VAC95	100	Unvaccinate	0.000	0.001	0.000	0.001	0.000	0.000	0.000	0.000	0.443	0.018	0.461	0.228	0.000	0.000	0	4.430	0.180	4.610	2.280	0.000	0.000
150	alpha	1	60x8 weeks	PCR90	Daily	VAC95	100	Unvaccinate	0.000	0.001	0.000	0.001	0.000	0.000	0.000	0.000	0.456	0.042	0.498	0.225	0.000	0.000	0	4.560	0.420	4.980	2.250	0.000	0.000
150	alpha	1	60x8 weeks	PCR90	Daily	VAC95	100	Unvaccinate	0.000	0.001	0.000	0.001	0.000	0.000	0.000	0.000	0.456	0.042	0.498	0.225	0.000	0.000	0	4.560	0.420	4.980	2.250	0.000	0.000
150	alpha	1	60x8 weeks	PCR90	Daily	VAC95	100	Unvaccinate	0.000	0.001	0.000	0.001	0.000	0.000	0.000	0.000	0.456	0.042	0.498	0.225	0.000	0.000	0	4.560	0.420	4.980	2.250	0.000	0.000
150	alpha	1.2	60x8 weeks	PCR90	Daily	VAC95	100	Unvaccinate	0.000	0.001	0.000	0.001	0.000	0.000	0.000	0.000	0.433	0.045	0.478	0.236	0.000	0.000	0	4.330	0.450	4.780	2.360	0.000	0.000
150	alpha	1.2	60x8 weeks	PCR90	Daily	VAC95	100	Unvaccinate	0.000	0.001	0.000	0.001	0.000	0.000	0.000	0.000	0.433	0.045	0.478	0.236	0.000	0.000	0	4.330	0.450	4.780	2.360	0.000	0.000
150	alpha	1.2	60x8 weeks	PCR90	Daily	VAC95	100	Unvaccinate	0.000	0.001	0.000	0.001	0.000	0.000	0.000	0.000	0.433	0.045	0.478	0.236	0.000	0.000	0	4.330	0.450	4.780	2.360	0.000	0.000
150	delta	0.6	60x8 weeks	PCR90	Daily	VAC95	100	Unvaccinate	0.000	0.002	0.000	0.002	0.001	0.000	0.000	0.000	0.720	0.173	0.893	0.450	0.000	0.000	0	7.200	1.730	8.930	4.500	0.000	0.000
150	delta	0.6	60x8 weeks	PCR90	Daily	VAC95	100	Unvaccinate	0.000	0.002	0.000	0.002	0.001	0.000	0.000	0.000	0.720	0.173	0.893	0.450	0.000	0.000	0	7.200	1.730	8.930	4.500	0.000	0.000
150	delta	0.6	60x8 weeks	PCR90	Daily	VAC95	100	Unvaccinate	0.000	0.002	0.000	0.002	0.001	0.000	0.000	0.000	0.720	0.173	0.893	0.450	0.000	0.000	0	7.200	1.730	8.930	4.500	0.000	0.000
150	delta	1	60x8 weeks	PCR90	Daily	VAC95	100	Unvaccinate	0.000	0.002	0.001	0.002	0.001	0.000	0.000	0.000	0.735	0.260	0.995	0.522	0.000	0.000	0	7.350	2.600	9.950	5.220	0.000	0.000
150	delta	1	60x8 weeks	PCR90	Daily	VAC95	100	Unvaccinate	0.000	0.002	0.001	0.002	0.001	0.000	0.000	0.000	0.735	0.260	0.995	0.522	0.000	0.000	0	7.350	2.600	9.950	5.220	0.000	0.000
150	delta	1	60x8 weeks	PCR90	Daily	VAC95	100	Unvaccinate	0.000	0.002	0.001	0.002	0.001	0.000	0.000	0.000	0.735	0.260	0.995	0.522	0.000	0.000	0	7.350	2.600	9.950	5.220	0.000	0.000
150	delta	1.2	60x8 weeks	PCR90	Daily	VAC95	100	Unvaccinate	0.000	0.002	0.001	0.002	0.001	0.000	0.000	0.000	0.749	0.357	1.106	0.603	0.000	0.000	0	7.490	3.570	11.060	6.030	0.000	0.000
150	delta	1.2	60x8 weeks	PCR90	Daily	VAC95	100	Unvaccinate	0.000	0.002	0.001	0.002	0.001	0.000	0.000	0.000	0.749	0.357	1.106	0.603	0.000	0.000	0	7.490	3.570	11.060	6.030	0.000	0.000
150	delta	1.2	60x8 weeks	PCR90	Daily	VAC95	100	Unvaccinate	0.000	0.002	0.001	0.002	0.001	0.000	0.000	0.000	0.749	0.357	1.106	0.603	0.000	0.000	0	7.490	3.570	11.060	6.030	0.000	0.000
150	original	0.6	60x8 weeks	PCR90	Daily	VAC70	25	Unvaccinate	3.689	0.008	0.002	0.010	0.001	0.009	0.004	1770.555	3.948	0.791	4.739	0.507	4.192	1.715	17706	39.480	7.910	47.390	5.070	41.920	17.150
150	original	0.6	60x8 weeks	PCR90	Daily	VAC70	25	Unvaccinate	3.689	0.008	0.002	0.010	0.001	0.009	0.004	1770.555	3.948	0.791	4.739	0.507	4.192	1.715	17706	39.480	7.910	47.390	5.070	41.920	17.150
150	original	0.6	60x8 weeks	PCR90	Daily	VAC70	25	Unvaccinate	3.689	0.008	0.002	0.010	0.001	0.009	0.004	1770.555	3.948	0.791	4.739	0.507	4.192	1.715	17706	39.480	7.910	47.390	5.070	41.920	17.150
150	original	1	60x8 weeks	PCR90	Daily	VAC70	25	Unvaccinate	3.678	0.008	0.003	0.011	0.001	0.010	0.004	1765.453	3.986	1.516	5.502	0.556	4.914	1.690	17655	39.860	15.160	55.020	5.560	49.140	16.900
150	original	1	60x8 weeks	PCR90	Daily	VAC70	25	Unvaccinate	3.678	0.008	0.003	0.011	0.001	0.010	0.004	1765.453	3.986	1.516	5.502	0.556	4.914	1.690	17655	39.860	15.160	55.020	5.560	49.140	16.900
150	original	1	60x8 weeks	PCR90	Daily	VAC70	25	Unvaccinate	3.678	0.008	0.003	0.011	0.001	0.010	0.004	1765.453	3.986	1.516	5.502	0.556	4.914	1.690	17655	39.860	15.160	55.020	5.560	49.140	16.900
150	original	1.2	60x8 weeks	PCR90	Daily	VAC70	25	Unvaccinate	3.675	0.008	0.004	0.012	0.001	0.011	0.003	1764.098	3.937	1.803	5.740	0.630	5.064	1.673	17641	39.370	18.030	57.400	6.300	50.640	16.730
150	original	1.2	60x8 weeks	PCR90	Daily	VAC70	25	Unvaccinate	3.675	0.008	0.004	0.012	0.001	0.011	0.003	1764.098	3.937	1.803	5.740	0.630	5.064	1.673	17641	39.370	18.030	57.400	6.300	50.640	16.730
150	original	1.2	60x8 weeks	PCR90	Daily	VAC70	25	Unvaccinate	3.675	0.008	0.004	0.012	0.001	0.011	0.003	1764.098	3.937	1.803	5.740	0.630	5.064	1.673	17641	39.370	18.030	57.400	6.300	50.640	16.730
150	alpha	0.6	60x8 weeks	PCR90	Daily	VAC70	25	Unvaccinate	3.671	0.008	0.004	0.013	0.002	0.011	0.004	1762.261	3.880	2.157	6.037	0.728	5.359	1.718	17623	38.800	21.570	60.370	7.280	53.590	17.180
150	alpha	0.6	60x8 weeks	PCR90	Daily	VAC70	25	Unvaccinate	3.671	0.008	0.004	0.013	0.002	0.011	0.004	1762.261	3.880	2.157	6.037	0.728	5.359	1.718	17623	38.800	21.570	60.370	7.280	53.590	17.180
150	alpha	0.6	60x8 weeks	PCR90	Daily	VAC70	25	Unvaccinate	3.671	0.008	0.004	0.013	0.002	0.011	0.004	1762.261	3.880	2.157	6.037	0.728	5.359	1.718	17623	38.800	21.570	60.370	7.280	53.590	17.180
150	alpha	1	60x8 weeks	PCR90	Daily	VAC70	25	Unvaccinate	3.633	0.008	0.010	0.018	0.002	0.017	0.004	1743.777	3.823	5.018	8.841	1.061	7.995	1.684	17438	38.230	50.180	88.410	10.610	79.950	16.840
150	alpha	1	60x8 weeks	PCR90	Daily	VAC70	25	Unvaccinate	3.633	0.008	0.010	0.018	0.002	0.017	0.004	1743.777	3.823	5.018	8.841	1.061	7.995	1.684	17438	38.230	50.180	88.410	10.610	79.950	16.840
150	alpha	1	60x8 weeks	PCR90	Daily	VAC70	25	Unvaccinate	3.633	0.008	0.010	0.018	0.002	0.017	0.004	1743.777	3.823	5.018	8.841	1.061	7.995	1.684	17438	38.230	50.180	88.410	10.610	79.950	16.840

150	alpha	1.2	60x8 weeks	PCR90	Daily	VAC70	25	Invaccinate	3.608	0.008	0.015	0.022	0.003	0.020	0.003	1731.713	3.770	6.998	10.768	1.386	9.743	1.641	17317	37.700	69.980	107.680	13.860	97.430	16.410
150	alpha	1.2	60x8 weeks	PCR90	Daily	VAC70	25	Invaccinate	3.608	0.008	0.015	0.022	0.003	0.020	0.003	1731.713	3.770	6.998	10.768	1.386	9.743	1.641	17317	37.700	69.980	107.680	13.860	97.430	16.410
150	alpha	1.2	60x8 weeks	PCR90	Daily	VAC70	25	Invaccinate	3.608	0.008	0.015	0.022	0.003	0.020	0.003	1731.713	3.770	6.998	10.768	1.386	9.743	1.641	17317	37.700	69.980	107.680	13.860	97.430	16.410
150	delta	0.6	60x8 weeks	PCR90	Daily	VAC70	25	Invaccinate	3.574	0.008	0.020	0.028	0.005	0.024	0.003	1715.680	3.879	9.712	13.591	2.334	11.461	1.513	17157	38.790	97.120	135.910	23.340	114.610	15.130
150	delta	0.6	60x8 weeks	PCR90	Daily	VAC70	25	Invaccinate	3.574	0.008	0.020	0.028	0.005	0.024	0.003	1715.680	3.879	9.712	13.591	2.334	11.461	1.513	17157	38.790	97.120	135.910	23.340	114.610	15.130
150	delta	0.6	60x8 weeks	PCR90	Daily	VAC70	25	Invaccinate	3.574	0.008	0.020	0.028	0.005	0.024	0.003	1715.680	3.879	9.712	13.591	2.334	11.461	1.513	17157	38.790	97.120	135.910	23.340	114.610	15.130
150	delta	1	60x8 weeks	PCR90	Daily	VAC70	25	Invaccinate	3.294	0.006	0.068	0.074	0.015	0.060	0.003	1581.106	2.894	32.776	35.670	7.255	28.961	1.255	15811	28.940	327.760	356.700	72.550	289.610	12.550
150	delta	1	60x8 weeks	PCR90	Daily	VAC70	25	Invaccinate	3.294	0.006	0.068	0.074	0.015	0.060	0.003	1581.106	2.894	32.776	35.670	7.255	28.961	1.255	15811	28.940	327.760	356.700	72.550	289.610	12.550
150	delta	1	60x8 weeks	PCR90	Daily	VAC70	25	Invaccinate	3.294	0.006	0.068	0.074	0.015	0.060	0.003	1581.106	2.894	32.776	35.670	7.255	28.961	1.255	15811	28.940	327.760	356.700	72.550	289.610	12.550
150	delta	1.2	60x8 weeks	PCR90	Daily	VAC70	25	Invaccinate	3.195	0.005	0.086	0.091	0.019	0.073	0.002	1533.629	2.527	41.212	43.739	9.287	34.900	1.066	15336	25.270	412.120	437.390	92.870	349.000	10.660
150	delta	1.2	60x8 weeks	PCR90	Daily	VAC70	25	Invaccinate	3.195	0.005	0.086	0.091	0.019	0.073	0.002	1533.629	2.527	41.212	43.739	9.287	34.900	1.066	15336	25.270	412.120	437.390	92.870	349.000	10.660
150	delta	1.2	60x8 weeks	PCR90	Daily	VAC70	25	Invaccinate	3.195	0.005	0.086	0.091	0.019	0.073	0.002	1533.629	2.527	41.212	43.739	9.287	34.900	1.066	15336	25.270	412.120	437.390	92.870	349.000	10.660
150	original	0.6	60x8 weeks	PCR90	Daily	VAC70	50	Invaccinate	2.462	0.007	0.001	0.007	0.001	0.005	0.002	1181.629	3.187	0.371	3.558	0.615	2.622	1.186	11816	31.870	3.710	35.580	6.150	26.220	11.860
150	original	0.6	60x8 weeks	PCR90	Daily	VAC70	50	Invaccinate	2.462	0.007	0.001	0.007	0.001	0.005	0.002	1181.629	3.187	0.371	3.558	0.615	2.622	1.186	11816	31.870	3.710	35.580	6.150	26.220	11.860
150	original	0.6	60x8 weeks	PCR90	Daily	VAC70	50	Invaccinate	2.462	0.007	0.001	0.007	0.001	0.005	0.002	1181.629	3.187	0.371	3.558	0.615	2.622	1.186	11816	31.870	3.710	35.580	6.150	26.220	11.860
150	original	1	60x8 weeks	PCR90	Daily	VAC70	50	Invaccinate	2.457	0.007	0.002	0.008	0.001	0.006	0.002	1179.144	3.236	0.734	3.970	0.673	2.970	1.163	11791	32.360	7.340	39.700	6.730	29.700	11.630
150	original	1	60x8 weeks	PCR90	Daily	VAC70	50	Invaccinate	2.457	0.007	0.002	0.008	0.001	0.006	0.002	1179.144	3.236	0.734	3.970	0.673	2.970	1.163	11791	32.360	7.340	39.700	6.730	29.700	11.630
150	original	1	60x8 weeks	PCR90	Daily	VAC70	50	Invaccinate	2.457	0.007	0.002	0.008	0.001	0.006	0.002	1179.144	3.236	0.734	3.970	0.673	2.970	1.163	11791	32.360	7.340	39.700	6.730	29.700	11.630
150	original	1.2	60x8 weeks	PCR90	Daily	VAC70	50	Invaccinate	2.456	0.007	0.002	0.009	0.002	0.006	0.002	1179.033	3.233	0.918	4.151	0.780	2.981	1.135	11790	32.330	9.180	41.510	7.800	29.810	11.350
150	original	1.2	60x8 weeks	PCR90	Daily	VAC70	50	Invaccinate	2.456	0.007	0.002	0.009	0.002	0.006	0.002	1179.033	3.233	0.918	4.151	0.780	2.981	1.135	11790	32.330	9.180	41.510	7.800	29.810	11.350
150	original	1.2	60x8 weeks	PCR90	Daily	VAC70	50	Invaccinate	2.456	0.007	0.002	0.009	0.002	0.006	0.002	1179.033	3.233	0.918	4.151	0.780	2.981	1.135	11790	32.330	9.180	41.510	7.800	29.810	11.350
150	alpha	0.6	60x8 weeks	PCR90	Daily	VAC70	50	Invaccinate	2.450	0.007	0.003	0.010	0.002	0.007	0.002	1176.105	3.229	1.356	4.585	0.921	3.436	1.126	11761	32.290	13.560	45.850	9.210	34.360	11.260
150	alpha	0.6	60x8 weeks	PCR90	Daily	VAC70	50	Invaccinate	2.450	0.007	0.003	0.010	0.002	0.007	0.002	1176.105	3.229	1.356	4.585	0.921	3.436	1.126	11761	32.290	13.560	45.850	9.210	34.360	11.260
150	alpha	0.6	60x8 weeks	PCR90	Daily	VAC70	50	Invaccinate	2.450	0.007	0.003	0.010	0.002	0.007	0.002	1176.105	3.229	1.356	4.585	0.921	3.436	1.126	11761	32.290	13.560	45.850	9.210	34.360	11.260
150	alpha	1	60x8 weeks	PCR90	Daily	VAC70	50	Invaccinate	2.435	0.007	0.006	0.012	0.002	0.009	0.002	1168.720	3.192	2.753	5.945	1.197	4.451	1.114	11687	31.920	27.530	59.450	11.970	44.510	11.140
150	alpha	1	60x8 weeks	PCR90	Daily	VAC70	50	Invaccinate	2.435	0.007	0.006	0.012	0.002	0.009	0.002	1168.720	3.192	2.753	5.945	1.197	4.451	1.114	11687	31.920	27.530	59.450	11.970	44.510	11.140
150	alpha	1	60x8 weeks	PCR90	Daily	VAC70	50	Invaccinate	2.435	0.007	0.006	0.012	0.002	0.009	0.002	1168.720	3.192	2.753	5.945	1.197	4.451	1.114	11687	31.920	27.530	59.450	11.970	44.510	11.140
150	alpha	1.2	60x8 weeks	PCR90	Daily	VAC70	50	Invaccinate	2.425	0.006	0.008	0.014	0.003	0.010	0.002	1164.194	3.001	3.786	6.787	1.435	5.026	1.135	11642	30.010	37.860	67.870	14.350	50.260	11.350
150	alpha	1.2	60x8 weeks	PCR90	Daily	VAC70	50	Invaccinate	2.425	0.006	0.008	0.014	0.003	0.010	0.002	1164.194	3.001	3.786	6.787	1.435	5.026	1.135	11642	30.010	37.860	67.870	14.350	50.260	11.350
150	alpha	1.2	60x8 weeks	PCR90	Daily	VAC70	50	Invaccinate	2.425	0.006	0.008	0.014	0.003	0.010	0.002	1164.194	3.001	3.786	6.787	1.435	5.026	1.135	11642	30.010	37.860	67.870	14.350	50.260	11.350
150	delta	0.6	60x8 weeks	PCR90	Daily	VAC70	50	Invaccinate	2.408	0.007	0.012	0.019	0.005	0.013	0.002	1155.604	3.178	5.955	9.133	2.455	6.008	1.113	11556	31.780	59.550	91.330	24.550	60.080	11.130
150	delta	0.6	60x8 weeks	PCR90	Daily	VAC70	50	Invaccinate	2.408	0.007	0.012	0.019	0.005	0.013	0.002	1155.604	3.178	5.955	9.133	2.455	6.008	1.113	11556	31.780	59.550	91.330	24.550	60.080	11.130
150	delta	0.6	60x8 weeks	PCR90	Daily	VAC70	50	Invaccinate	2.408	0.007	0.012	0.019	0.005	0.013	0.002	1155.604	3.178	5.955	9.133	2.455	6.008	1.113	11556	31.780	59.550	91.330	24.550	60.080	11.130
150	delta	1	60x8 weeks	PCR90	Daily	VAC70	50	Invaccinate	2.282	0.006	0.042	0.048	0.014	0.029	0.002	1095.501	2.871	19.953	22.824	6.826	14.057	0.917	10955	28.710	199.530	228.240	68.260	140.570	9.170
150	delta	1	60x8 weeks	PCR90	Daily	VAC70	50	Invaccinate	2.282	0.006	0.042	0.048	0.014	0.029	0.002	1095.501	2.871	19.953	22.824	6.826	14.057	0.917	10955	28.710	199.530	228.240	68.260	140.570	9.170
150	delta	1	60x8 weeks	PCR90	Daily	VAC70	50	Invaccinate	2.282	0.006	0.042	0.048	0.014	0.029	0.002	1095.501	2.871	19.953	22.824	6.826	14.057	0.917	10955	28.710	199.530	228.240	68.260	140.570	9.170
150	delta	1.2	60x8 weeks	PCR90	Daily	VAC70	50	Invaccinate	2.212	0.005	0.060	0.065	0.020	0.039	0.002	1061.715	2.524	28.701	31.225	9.700	18.507	0.817	10617	25.240	287.010	312.250	97.000	185.070	8.170
150	delta	1.2	60x8 weeks	PCR90	Daily	VAC70	50	Invaccinate	2.212	0.005	0.060	0.065	0.020	0.039	0.002	1061.715	2.524	28.701	31.225	9.700	18.507	0.817	10617	25.240	287.010	312.250	97.000	185.070	8.170
150	delta	1.2	60x8 weeks	PCR90	Daily	VAC70	50	Invaccinate	2.212	0.005	0.060	0.065	0.020	0.039	0.002	1061.715	2.524	28.701	31.225	9.700	18.507	0.817	10617	25.240	287.010	312.250	97.000	185.070	8.170
150	original	0.6	60x8 weeks	PCR90	Daily	VAC70	75	Invaccinate	1.231	0.005	0.000	0.006	0.001	0.003	0.001	590.744	2.450	0.190	2.640	0.706	1.300	0.547	5907	24.500	1.900	26.400	7.060	13.000	5.470
150	original	0.6	60x8 weeks	PCR90	Daily	VAC70	75	Invaccinate	1.231	0.005	0.000	0.006	0.001	0.003	0.001	590.744	2.450	0.190	2.640	0.706	1.300	0.547	5907	24.500	1.900	26.400	7.060	13.000	5.470
150	original	0.6	60x8 weeks	PCR90	Daily	VAC70	75	Invaccinate	1.231	0.005	0.000	0.006	0.001	0.003	0.001	590.744	2.450	0.190	2.640	0.706	1.300	0.547	5907	24.500	1.900	26.400	7.060	13.000	5.470
150	original	1	60x8 weeks	PCR90	Daily	VAC70	75	Invaccinate	1.232	0.005	0.001	0.005	0.002	0.003	0.001	591.395	2.353	0.279	2.632	0.749	1.231	0.593	5914	23.530	2.790	26.320	7.490	12.310	5.930
150	original	1	60x8 weeks	PCR90	Daily	VAC70	75	Invaccinate	1.232	0.005	0.001	0.005	0.002	0.003	0.001	591.395	2.353	0.279	2.632	0.749	1.231	0.593	5914	23.530	2.790	26.320	7.490	12.310	5.930
150	original	1	60x8 weeks	PCR90	Daily	VAC70	75	Invaccinate	1.232	0.005	0.001	0.005	0.002	0.003	0.001	591.395	2.353	0.279	2.632	0.749	1.231	0.593	5914	23.530	2.790	26.320	7.490	12.310	5.930
150	original	1.2	60x8 weeks	PCR90	Daily	VAC70	75	Invaccinate	1.232	0.005	0.001	0.006	0.002	0.003	0.001	591.451	2.389	0.291	2.680	0.797	1.214	0.563	5915	23.890	2.910	26.800	7.970	12.140	5.630
150	original	1.2	60x8 weeks	PCR90	Daily	VAC70	75	Invaccinate	1.232	0.005	0.001	0.006	0.002	0.003	0.001	591.451	2.389	0.291	2.680	0.797	1.214	0.563	5915	23.890	2.910	26.800	7.970	12.140	5.630
150	original	1.2	60x8 weeks	PCR90	Daily	VAC70	75	Invaccinate	1.232	0.005	0.001	0.006	0.002	0.003	0.001	591.451	2.389	0.291	2.680	0.797	1.214	0.563	5915	23.890	2.910	26.800	7.970	12.140	5.630
150	alpha	0.6	60x8 weeks	PCR90	Daily	VAC70	75	Invaccinate	1.227	0.005	0.001	0.006	0.002	0.003	0.001	588.954	2.402	0.715	3.117	1.005	1.576	0.568	5890	24.020	7.150	31.170	10.050	15.760	5.680
150	alpha	0.6	60x8 weeks	PCR90	Daily	VAC70	75	Invaccinate	1.227	0.005	0.001	0.006	0.002	0.003	0.001	588.954	2.402	0.715	3.117	1.005	1.576	0.568	5890	24.020	7.150	31.170	10.050	15.760	5.680
150	alpha	0.6	60x8 weeks	PCR90	Daily	VAC70	75	Invaccinate	1.227	0.005	0.001	0.006	0.002	0.003	0.001	588.954	2.402	0.715	3.117	1.005	1.576	0.568	5890	24.020	7.150	31.170	10.050	15.760	5.680
150	alpha	1	60x8 weeks	PCR90	Daily	VAC70	75	Invaccinate	1.222	0.005	0.003	0.008	0.003	0.004	0.001	586.706	2.351	1.441	3.792	1.233	1.907	0.567	5867	23.510	14.410	37.920	12.330	19.070	5.670
150	alpha	1	60x8 weeks	PCR90	Daily	VAC70	75	Invaccinate	1.222	0.005	0.003	0.008	0.003	0.004	0.001	586.706	2.351	1.441	3.792	1.233	1.907	0.567	5867	23.510	14.410	37.920	12.330	19.070	5.670
150	alpha	1	60x8 weeks	PCR90	Daily	VAC70	75	Invaccinate	1.222	0.005	0.003	0.008	0.003	0.004	0.001	586.706	2.351	1.441	3.792	1.233	1.907	0.567	5867	23.510	14.410	37.920	12.330	19.070	5.670
150	alpha	1.2	60x8 weeks	PCR90	Daily	VAC70	75	Invaccinate	1.220	0.005	0.004	0.009	0.003	0.004	0.001	585.483	2.346	1.842	4.188	1.385	2.055	0.559	5855	23.460	18.420	41.880	13.850	20.550	5.590
150	alpha	1.2	60x8 weeks	PCR90	Daily	VAC70	75	Invaccinate	1.220	0.005	0.004	0.009	0.003	0.004	0.001	585.483	2.346	1.842	4.188	1.385	2.055	0.559	5855	23.460	18.420	41.880	13.850	20.550	5.590
150	alpha	1.2	60x8 weeks	PCR90	Daily	VAC70	75	Invaccinate	1.220	0.005	0.004	0.009	0.003	0.004	0.001	585.483	2.346	1.842	4.188	1.385	2.055	0.559	5855	23.460	18.420	41.880	13.850	20.550	5.590
150	delta	0.6	60x8 weeks	PCR90	Daily	VAC70	75	Invaccinate	1.211	0.006	0.007	0.013	0.005	0.005	0.001	581.120	2.812	3.474	6.286	2.466	2.608	0.581	5811	28.120	34.740	62.860	24.660	26.080	5.810
150	delta	0.6	60x8 weeks	PCR90	Daily	VAC70	75	Invaccinate	1.211	0.006	0.007	0.013	0.005	0.005	0.001	581.120	2.812	3.474	6.286	2.466	2.608	0.581	5811	28.120	34.740	62.860	24.660	26.080	5.810
150	delta	0.6	60x8 weeks	PCR90	Daily	VAC70	75	Invaccinate	1.211	0.006	0.007	0.013	0.005	0.005	0.001	581.120	2.812	3.474	6.286	2.466	2.608	0.581	5811	28.120	34.740	62.860	24.660	26.080	5.810
150	delta	1	60x8 weeks	PCR90	Daily	VAC70	75	Invaccinate	1.176	0.005	0.022	0.027	0.011	0.010	0.001	564.449	2.552	10.381	12.933	5.434	4.855	0.469	5644	25.520	103.810	129.330	54.340	48.550	4.690
150	delta	1	60x8 weeks	PCR90	Daily	VAC70	75	Invaccinate	1.176	0.005	0.022	0.027	0.011	0.010	0.001	564.449	2.552	10.381	12.933	5.434	4.855	0.469	5644	25.520	103.810	129.330	54.340	48.550	4.690
150	delta	1	60x8 weeks	PCR90	Daily	VAC70	75	Invaccinate	1.176	0.005	0.022	0.027	0.011	0.010	0.001	564.449	2.552	10.381	12.933	5.434	4.855	0.469	5644	25.520	103.810	129.330	54.340	48.550	4.690
150	delta	1.2	60x8 weeks	PCR90	Daily	VAC70	75	Invaccinate	1.145	0.005	0.035	0.040	0.017	0.014	0.001	549.790	2.348	17.000	19.348	8.379	6.721	0.489	5498	23.480	170.000	193.480	83.790	67.210	4.890
150	delta	1.2	60x8 weeks	PCR90	Daily	VAC70	75	Invaccinate	1.145	0.005	0.035	0.040	0.017	0.014	0.001	549.790	2.348	17.000	19.348	8.379	6.721	0.489	5498	23.480	170.000	193.480	83.790	67.210	4.890
150	delta	1.2	60x8 weeks	PCR90	Daily	VAC70	75	Invaccinate	1.145	0.005	0.035	0.040	0.017	0.014	0.001	549.790	2.348	17.000	19.348	8.379	6.721	0.489	5498	23.480	170.000	193.480	83.790	67.210	4.890
150	original	0.6	60x8 weeks	PCR90	Daily	VAC70	100	Invaccinate	0.000	0.003	0.000	0.003	0.002	0.000	0.000	0.000	1.617	0.012	1.629	0.799	0.000	0.000	0	16.170	0.120	16.290	7.990	0.000	0.000
150	original	0.6	60x8 weeks	PCR90	Daily	VAC70	100	Invaccinate	0.000	0.003	0.000	0.003	0.002	0.000	0.000	0.000	1.617	0.012	1.629	0.799	0.000	0.000	0	16.170	0.120	16.290	7.990	0.000	0.000
150	original	0.6	60x8 weeks	PCR90	Daily	VAC70	100	Invaccinate	0.000	0.003	0.000	0.003	0.002	0.000	0.000	0.000	1.617	0.012	1.629	0.799	0.000	0.000	0	16.170	0.120	16.290	7.990	0.000	0.000
150	original	1	60x8 weeks	PCR90	Daily	VAC70	100	Invaccinate	0.000	0.003	0.000	0.003	0.002	0.000	0.000	0.000	1.661	0.016	1.677	0.848	0.000	0.000	0	16.610	0.160	16.770	8.480	0.000	0.000
150	original	1	60x8 weeks	PCR90	Daily	VAC70	100	Invaccinate	0.000	0.003	0.000	0.003	0.002	0.000	0.000	0.000	1.661	0.016	1.677	0.848	0.000	0.000	0	16.610	0.160	16.770	8.480	0.000	0.000

150	original	1	60x8 weeks	PCR90	Daily	VAC70	100	Jnvaccinate	0.000	0.003	0.000	0.003	0.002	0.000	0.000	0.000	1.661	0.016	1.677	0.848	0.000	0.000	0	16.610	0.160	16.770	8.480	0.000	0.000
150	original	1.2	60x8 weeks	PCR90	Daily	VAC70	100	Jnvaccinate	0.000	0.004	0.000	0.004	0.002	0.000	0.000	0.000	1.715	0.027	1.742	0.834	0.000	0.000	0	17.150	0.270	17.420	8.340	0.000	0.000
150	original	1.2	60x8 weeks	PCR90	Daily	VAC70	100	Jnvaccinate	0.000	0.004	0.000	0.004	0.002	0.000	0.000	0.000	1.715	0.027	1.742	0.834	0.000	0.000	0	17.150	0.270	17.420	8.340	0.000	0.000
150	original	1.2	60x8 weeks	PCR90	Daily	VAC70	100	Jnvaccinate	0.000	0.004	0.000	0.004	0.002	0.000	0.000	0.000	1.715	0.027	1.742	0.834	0.000	0.000	0	17.150	0.270	17.420	8.340	0.000	0.000
150	alpha	0.6	60x8 weeks	PCR90	Daily	VAC70	100	Jnvaccinate	0.000	0.003	0.001	0.004	0.002	0.000	0.000	0.000	1.569	0.316	1.885	1.110	0.000	0.000	0	15.690	3.160	18.850	11.100	0.000	0.000
150	alpha	0.6	60x8 weeks	PCR90	Daily	VAC70	100	Jnvaccinate	0.000	0.003	0.001	0.004	0.002	0.000	0.000	0.000	1.569	0.316	1.885	1.110	0.000	0.000	0	15.690	3.160	18.850	11.100	0.000	0.000
150	alpha	0.6	60x8 weeks	PCR90	Daily	VAC70	100	Jnvaccinate	0.000	0.003	0.001	0.004	0.002	0.000	0.000	0.000	1.569	0.316	1.885	1.110	0.000	0.000	0	15.690	3.160	18.850	11.100	0.000	0.000
150	alpha	1	60x8 weeks	PCR90	Daily	VAC70	100	Jnvaccinate	0.000	0.003	0.001	0.004	0.003	0.000	0.000	0.000	1.547	0.605	2.152	1.288	0.000	0.000	0	15.470	6.050	21.520	12.880	0.000	0.000
150	alpha	1	60x8 weeks	PCR90	Daily	VAC70	100	Jnvaccinate	0.000	0.003	0.001	0.004	0.003	0.000	0.000	0.000	1.547	0.605	2.152	1.288	0.000	0.000	0	15.470	6.050	21.520	12.880	0.000	0.000
150	alpha	1	60x8 weeks	PCR90	Daily	VAC70	100	Jnvaccinate	0.000	0.003	0.001	0.004	0.003	0.000	0.000	0.000	1.547	0.605	2.152	1.288	0.000	0.000	0	15.470	6.050	21.520	12.880	0.000	0.000
150	alpha	1.2	60x8 weeks	PCR90	Daily	VAC70	100	Jnvaccinate	0.000	0.003	0.002	0.005	0.003	0.000	0.000	0.000	1.660	0.779	2.439	1.417	0.000	0.000	0	16.600	7.790	24.390	14.170	0.000	0.000
150	alpha	1.2	60x8 weeks	PCR90	Daily	VAC70	100	Jnvaccinate	0.000	0.003	0.002	0.005	0.003	0.000	0.000	0.000	1.660	0.779	2.439	1.417	0.000	0.000	0	16.600	7.790	24.390	14.170	0.000	0.000
150	alpha	1.2	60x8 weeks	PCR90	Daily	VAC70	100	Jnvaccinate	0.000	0.003	0.002	0.005	0.003	0.000	0.000	0.000	1.660	0.779	2.439	1.417	0.000	0.000	0	16.600	7.790	24.390	14.170	0.000	0.000
150	delta	0.6	60x8 weeks	PCR90	Daily	VAC70	100	Jnvaccinate	0.000	0.004	0.004	0.008	0.005	0.000	0.000	0.000	2.068	1.765	3.833	2.323	0.000	0.000	0	20.680	17.650	38.330	23.230	0.000	0.000
150	delta	0.6	60x8 weeks	PCR90	Daily	VAC70	100	Jnvaccinate	0.000	0.004	0.004	0.008	0.005	0.000	0.000	0.000	2.068	1.765	3.833	2.323	0.000	0.000	0	20.680	17.650	38.330	23.230	0.000	0.000
150	delta	0.6	60x8 weeks	PCR90	Daily	VAC70	100	Jnvaccinate	0.000	0.004	0.004	0.008	0.005	0.000	0.000	0.000	2.068	1.765	3.833	2.323	0.000	0.000	0	20.680	17.650	38.330	23.230	0.000	0.000
150	delta	1	60x8 weeks	PCR90	Daily	VAC70	100	Jnvaccinate	0.000	0.004	0.011	0.015	0.009	0.000	0.000	0.000	1.929	5.074	7.003	4.355	0.000	0.000	0	19.290	50.740	70.030	43.550	0.000	0.000
150	delta	1	60x8 weeks	PCR90	Daily	VAC70	100	Jnvaccinate	0.000	0.004	0.011	0.015	0.009	0.000	0.000	0.000	1.929	5.074	7.003	4.355	0.000	0.000	0	19.290	50.740	70.030	43.550	0.000	0.000
150	delta	1	60x8 weeks	PCR90	Daily	VAC70	100	Jnvaccinate	0.000	0.004	0.011	0.015	0.009	0.000	0.000	0.000	1.929	5.074	7.003	4.355	0.000	0.000	0	19.290	50.740	70.030	43.550	0.000	0.000
150	delta	1.2	60x8 weeks	PCR90	Daily	VAC70	100	Jnvaccinate	0.000	0.004	0.016	0.020	0.012	0.000	0.000	0.000	1.983	7.768	9.751	5.952	0.000	0.000	0	19.830	77.680	97.510	59.520	0.000	0.000
150	delta	1.2	60x8 weeks	PCR90	Daily	VAC70	100	Jnvaccinate	0.000	0.004	0.016	0.020	0.012	0.000	0.000	0.000	1.983	7.768	9.751	5.952	0.000	0.000	0	19.830	77.680	97.510	59.520	0.000	0.000
150	delta	1.2	60x8 weeks	PCR90	Daily	VAC70	100	Jnvaccinate	0.000	0.004	0.016	0.020	0.012	0.000	0.000	0.000	1.983	7.768	9.751	5.952	0.000	0.000	0	19.830	77.680	97.510	59.520	0.000	0.000
150	original	0.6	60x8 weeks	PCR90	Daily	VAC50	25	Jnvaccinate	3.687	0.009	0.002	0.011	0.001	0.009	0.004	1769.677	4.238	0.895	5.133	0.680	4.316	1.748	17697	42.380	8.950	51.330	6.800	43.160	17.480
150	original	0.6	60x8 weeks	PCR90	Daily	VAC50	25	Jnvaccinate	3.687	0.009	0.002	0.011	0.001	0.009	0.004	1769.677	4.238	0.895	5.133	0.680	4.316	1.748	17697	42.380	8.950	51.330	6.800	43.160	17.480
150	original	0.6	60x8 weeks	PCR90	Daily	VAC50	25	Jnvaccinate	3.687	0.009	0.002	0.011	0.001	0.009	0.004	1769.677	4.238	0.895	5.133	0.680	4.316	1.748	17697	42.380	8.950	51.330	6.800	43.160	17.480
150	original	1	60x8 weeks	PCR90	Daily	VAC50	25	Jnvaccinate	3.678	0.009	0.003	0.012	0.002	0.010	0.004	1765.329	4.312	1.637	5.949	0.774	4.934	1.721	17653	43.120	16.370	59.490	7.740	49.340	17.210
150	original	1	60x8 weeks	PCR90	Daily	VAC50	25	Jnvaccinate	3.678	0.009	0.003	0.012	0.002	0.010	0.004	1765.329	4.312	1.637	5.949	0.774	4.934	1.721	17653	43.120	16.370	59.490	7.740	49.340	17.210
150	original	1	60x8 weeks	PCR90	Daily	VAC50	25	Jnvaccinate	3.678	0.009	0.003	0.012	0.002	0.010	0.004	1765.329	4.312	1.637	5.949	0.774	4.934	1.721	17653	43.120	16.370	59.490	7.740	49.340	17.210
150	original	1.2	60x8 weeks	PCR90	Daily	VAC50	25	Jnvaccinate	3.674	0.009	0.004	0.013	0.002	0.011	0.003	1763.540	4.212	2.048	6.260	0.797	5.237	1.659	17635	42.120	20.480	62.600	7.970	52.370	16.590
150	original	1.2	60x8 weeks	PCR90	Daily	VAC50	25	Jnvaccinate	3.674	0.009	0.004	0.013	0.002	0.011	0.003	1763.540	4.212	2.048	6.260	0.797	5.237	1.659	17635	42.120	20.480	62.600	7.970	52.370	16.590
150	original	1.2	60x8 weeks	PCR90	Daily	VAC50	25	Jnvaccinate	3.674	0.009	0.004	0.013	0.002	0.011	0.003	1763.540	4.212	2.048	6.260	0.797	5.237	1.659	17635	42.120	20.480	62.600	7.970	52.370	16.590
150	alpha	0.6	60x8 weeks	PCR90	Daily	VAC50	25	Jnvaccinate	3.669	0.009	0.005	0.013	0.002	0.011	0.004	1760.920	4.134	2.303	6.437	0.969	5.511	1.754	17609	41.340	23.030	64.370	9.690	55.110	17.540
150	alpha	0.6	60x8 weeks	PCR90	Daily	VAC50	25	Jnvaccinate	3.669	0.009	0.005	0.013	0.002	0.011	0.004	1760.920	4.134	2.303	6.437	0.969	5.511	1.754	17609	41.340	23.030	64.370	9.690	55.110	17.540
150	alpha	0.6	60x8 weeks	PCR90	Daily	VAC50	25	Jnvaccinate	3.669	0.009	0.005	0.013	0.002	0.011	0.004	1760.920	4.134	2.303	6.437	0.969	5.511	1.754	17609	41.340	23.030	64.370	9.690	55.110	17.540
150	alpha	1	60x8 weeks	PCR90	Daily	VAC50	25	Jnvaccinate	3.621	0.008	0.013	0.022	0.003	0.018	0.003	1737.872	4.078	6.290	10.368	1.604	8.811	1.581	17379	40.780	62.900	103.680	16.040	88.110	15.810
150	alpha	1	60x8 weeks	PCR90	Daily	VAC50	25	Jnvaccinate	3.621	0.008	0.013	0.022	0.003	0.018	0.003	1737.872	4.078	6.290	10.368	1.604	8.811	1.581	17379	40.780	62.900	103.680	16.040	88.110	15.810
150	alpha	1	60x8 weeks	PCR90	Daily	VAC50	25	Jnvaccinate	3.621	0.008	0.013	0.022	0.003	0.018	0.003	1737.872	4.078	6.290	10.368	1.604	8.811	1.581	17379	40.780	62.900	103.680	16.040	88.110	15.810
150	alpha	1.2	60x8 weeks	PCR90	Daily	VAC50	25	Jnvaccinate	3.589	0.008	0.019	0.027	0.004	0.023	0.003	1722.665	4.002	9.028	13.030	2.093	10.925	1.551	17227	40.020	90.280	130.300	20.930	109.250	15.510
150	alpha	1.2	60x8 weeks	PCR90	Daily	VAC50	25	Jnvaccinate	3.589	0.008	0.019	0.027	0.004	0.023	0.003	1722.665	4.002	9.028	13.030	2.093	10.925	1.551	17227	40.020	90.280	130.300	20.930	109.250	15.510
150	alpha	1.2	60x8 weeks	PCR90	Daily	VAC50	25	Jnvaccinate	3.589	0.008	0.019	0.027	0.004	0.023	0.003	1722.665	4.002	9.028	13.030	2.093	10.925	1.551	17227	40.020	90.280	130.300	20.930	109.250	15.510
150	delta	0.6	60x8 weeks	PCR90	Daily	VAC50	25	Jnvaccinate	3.546	0.008	0.027	0.035	0.008	0.028	0.003	1701.898	3.970	12.982	16.952	3.645	13.226	1.519	17019	39.700	129.820	169.520	36.450	132.260	15.190
150	delta	0.6	60x8 weeks	PCR90	Daily	VAC50	25	Jnvaccinate	3.546	0.008	0.027	0.035	0.008	0.028	0.003	1701.898	3.970	12.982	16.952	3.645	13.226	1.519	17019	39.700	129.820	169.520	36.450	132.260	15.190
150	delta	0.6	60x8 weeks	PCR90	Daily	VAC50	25	Jnvaccinate	3.546	0.008	0.027	0.035	0.008	0.028	0.003	1701.898	3.970	12.982	16.952	3.645	13.226	1.519	17019	39.700	129.820	169.520	36.450	132.260	15.190
150	delta	1	60x8 weeks	PCR90	Daily	VAC50	25	Jnvaccinate	3.228	0.006	0.083	0.090	0.021	0.069	0.002	1549.538	2.916	40.058	42.974	10.013	32.986	1.068	15495	29.160	400.580	429.740	100.130	329.860	10.680
150	delta	1	60x8 weeks	PCR90	Daily	VAC50	25	Jnvaccinate	3.228	0.006	0.083	0.090	0.021	0.069	0.002	1549.538	2.916	40.058	42.974	10.013	32.986	1.068	15495	29.160	400.580	429.740	100.130	329.860	10.680
150	delta	1	60x8 weeks	PCR90	Daily	VAC50	25	Jnvaccinate	3.228	0.006	0.083	0.090	0.021	0.069	0.002	1549.538	2.916	40.058	42.974	10.013	32.986	1.068	15495	29.160	400.580	429.740	100.130	329.860	10.680
150	delta	1.2	60x8 weeks	PCR90	Daily	VAC50	25	Jnvaccinate	3.143	0.005	0.099	0.104	0.025	0.078	0.002	1508.778	2.437	47.385	49.822	12.088	37.670	0.905	15088	24.370	473.850	498.220	120.880	376.700	9.050
150	delta	1.2	60x8 weeks	PCR90	Daily	VAC50	25	Jnvaccinate	3.143	0.005	0.099	0.104	0.025	0.078	0.002	1508.778	2.437	47.385	49.822	12.088	37.670	0.905	15088	24.370	473.850	498.220	120.880	376.700	9.050
150	delta	1.2	60x8 weeks	PCR90	Daily	VAC50	25	Jnvaccinate	3.143	0.005	0.099	0.104	0.025	0.078	0.002	1508.778	2.437	47.385	49.822	12.088	37.670	0.905	15088	24.370	473.850	498.220	120.880	376.700	9.050
150	original	0.6	60x8 weeks	PCR90	Daily	VAC50	50	Jnvaccinate	2.462	0.008	0.001	0.009	0.002	0.005	0.002	1181.899	3.660	0.445	4.105	0.911	2.601	1.151	11819	36.600	4.450	41.050	9.110	26.010	11.510
150	original	0.6	60x8 weeks	PCR90	Daily	VAC50	50	Jnvaccinate	2.462	0.008	0.001	0.009	0.002	0.005	0.002	1181.899	3.660	0.445	4.105	0.911	2.601	1.151	11819	36.600	4.450	41.050	9.110	26.010	11.510
150	original	0.6	60x8 weeks	PCR90	Daily	VAC50	50	Jnvaccinate	2.462	0.008	0.001	0.009	0.002	0.005	0.002	1181.899	3.660	0.445	4.105	0.911	2.601	1.151	11819	36.600	4.450	41.050	9.110	26.010	11.510
150	original	1	60x8 weeks	PCR90	Daily	VAC50	50	Jnvaccinate	2.459	0.008	0.002	0.009	0.002	0.006	0.002	1180.252	3.705	0.839	4.544	1.055	2.823	1.091	11803	37.050	8.390	45.440	10.550	28.230	10.910
150	original	1	60x8 weeks	PCR90	Daily	VAC50	50	Jnvaccinate	2.459	0.008	0.002	0.009	0.002	0.006	0.002	1180.252	3.705	0.839	4.544	1.055	2.823	1.091	11803	37.050	8.390	45.440	10.550	28.230	10.910
150	original	1	60x8 weeks	PCR90	Daily	VAC50	50	Jnvaccinate	2.459	0.008	0.002	0.009	0.002	0.006	0.002	1180.252	3.705	0.839	4.544	1.055	2.823	1.091	11803	37.050	8.390	45.440	10.550	28.230	10.910
150	original	1.2	60x8 weeks	PCR90	Daily	VAC50	50	Jnvaccinate	2.456	0.008	0.002	0.010	0.002	0.006	0.002	1178.644	3.612	1.101	4.713	1.074	3.031	1.164	11786	36.120	11.010	47.130	10.740	30.310	11.640
150	original	1.2	60x8 weeks	PCR90	Daily	VAC50	50	Jnvaccinate	2.456	0.008	0.002	0.010	0.002	0.006	0.002	1178.644	3.612	1.101	4.713	1.074	3.031	1.164	11786	36.120	11.010	47.130	10.740	30.310	11.640
150	original	1.2	60x8 weeks	PCR90	Daily	VAC50	50	Jnvaccinate	2.456	0.008	0.002	0.010	0.002	0.006	0.002	1178.644	3.612	1.101	4.713	1.074	3.031	1.164	11786	36.120	11.010	47.130	10.740	30.310	11.640
150	alpha	0.6	60x8 weeks	PCR90	Daily	VAC50	50	Jnvaccinate	2.449	0.007	0.004	0.011	0.003	0.007	0.002	1175.649	3.562	1.768	5.330	1.375	3.455	1.153	11756	35.620	17.680	53.300	13.750	34.550	11.530
150	alpha	0.6	60x8 weeks	PCR90	Daily	VAC50	50	Jnvaccinate	2.449	0.007	0.004	0.011	0.003	0.007	0.002	1175.649	3.562	1.768	5.330	1.375	3.455	1.153	11756	35.620	17.680	53.300	13.750	34.550	11.530
150	alpha	0.6	60x8 weeks	PCR90	Daily	VAC50	50	Jnvaccinate	2.449	0.007	0.004	0.011	0.003	0.007	0.002	1175.649	3.562	1.768	5.330	1.375	3.455	1.153	11756	35.620	17.680	53.300	13.750	34.550	11.530
150	alpha	1	60x8 weeks	PCR90	Daily	VAC50	50	Jnvaccinate	2.429	0.007	0.008	0.016	0.004	0.010	0.002	1165.932	3.485	4.005	7.490	1.941	4.903	1.123	11659	34.850	40.050	74.900	19.410	49.030	11.230
150	alpha	1	60x8 weeks	PCR90	Daily	VAC50	50	Jnvaccinate	2.429	0.007	0.008	0.016	0.004	0.010	0.002	1165.932	3.485	4.005	7.490	1.941	4.903	1.123	11659	34.850	40.050	74.900	19.410	49.030	11.230
150	alpha	1	60x8 weeks	PCR90	Daily	VAC50	50	Jnvaccinate	2.429	0.007	0.008	0.016	0.004	0.010	0.002	1165.932	3.485	4.005	7.490	1.941	4.903	1.123	11659	34.850	40.050	74.900	19.410	49.030	11.230
150	alpha	1.2	60x8 weeks	PCR90	Daily	VAC50	50	Jnvaccinate	2.407	0.007	0.013	0.021	0.006	0.013	0.002	1155.408	3.525	6.434	9.959	2.719	6.344	1.048	11554	35.250	64.340	99.590	27.190	63.440	10.480
150	alpha	1.2	60x8 weeks	PCR90	Daily	VAC50	50	Jnvaccinate	2.407	0.007	0.013	0.021	0.006	0.013	0.002	1155.408	3.525	6.434	9.959	2.719	6.344	1.048	11554	35.250	64.340	99.590	27.190	63.440	10.480
150	alpha	1.2	60x8 weeks	PCR90	Daily	VAC50	50	Jnvaccinate	2.407	0.007	0.013	0.021	0.006	0.013	0.002	1155.408	3.525	6.434	9.959	2.719	6.344	1.048	11554	35.250	64.340	99.590	27.190	63.440	10.480
150	delta	0.6	60x8 weeks	PCR90	Daily	VAC50	50	Jnvaccinate	2.377	0.008	0.023	0.030	0.010	0.017	0.002	1140.799	3.671	10.834	14.505	4.779	8.063	0.993	11408	36.710	108.340	145.050	47.790	80.630	9.930
150	delta	0.6	60x8 weeks	PCR90	Daily	VAC50	50	Jnvaccinate	2.377	0.008	0.023	0.030	0.010	0.017	0.002	1140.799	3.671	10.834	14.505	4.779	8.063	0.993	11408	36.710	108.340	145.050	47.790	80.630	9.930
150	delta	0.6	60x8 weeks	PCR90	Daily	VAC50	50	Jnvaccinate	2.377	0.008	0.023	0.030	0.010	0.017	0.002	1140.799	3.671	10.834	14.505	4.779	8.063	0.993	11408	36.710	108.340	145.050	47.790	80.630	9.930
150	delta	1	60x8 weeks	PCR90	Daily	VAC50	50	Jnvaccinate	2.198	0.006	0.070	0.076	0.027	0.040	0.002	1054.997	2.918	33.523	36.441	13.100	19.239	0.819	10550	29.180	335.230	364.410	131.000	192.390	8.190

150	delta	1	60x8 weeks	PCR90	Daily	VAC50	50	Jnvaccinate	2.198	0.006	0.070	0.076	0.027	0.040	0.002	1054.997	2.918	33.523	36.441	13.100	19.239	0.819	10550	29.180	335.230	364.410	131.000	192.390	8.190
150	delta	1	60x8 weeks	PCR90	Daily	VAC50	50	Jnvaccinate	2.198	0.006	0.070	0.076	0.027	0.040	0.002	1054.997	2.918	33.523	36.441	13.100	19.239	0.819	10550	29.180	335.230	364.410	131.000	192.390	8.190
150	delta	1.2	60x8 weeks	PCR90	Daily	VAC50	50	Jnvaccinate	2.108	0.005	0.093	0.098	0.037	0.051	0.001	1011.864	2.413	44.572	46.985	17.648	24.308	0.650	10119	24.130	445.720	469.850	176.480	243.080	6.500
150	delta	1.2	60x8 weeks	PCR90	Daily	VAC50	50	Jnvaccinate	2.108	0.005	0.093	0.098	0.037	0.051	0.001	1011.864	2.413	44.572	46.985	17.648	24.308	0.650	10119	24.130	445.720	469.850	176.480	243.080	6.500
150	delta	1.2	60x8 weeks	PCR90	Daily	VAC50	50	Jnvaccinate	2.108	0.005	0.093	0.098	0.037	0.051	0.001	1011.864	2.413	44.572	46.985	17.648	24.308	0.650	10119	24.130	445.720	469.850	176.480	243.080	6.500
150	original	0.6	60x8 weeks	PCR90	Daily	VAC50	75	Jnvaccinate	1.232	0.006	0.000	0.007	0.002	0.003	0.001	591.219	3.102	0.218	3.320	1.142	1.252	0.605	5912	31.020	2.180	33.200	11.420	12.520	6.050
150	original	0.6	60x8 weeks	PCR90	Daily	VAC50	75	Jnvaccinate	1.232	0.006	0.000	0.007	0.002	0.003	0.001	591.219	3.102	0.218	3.320	1.142	1.252	0.605	5912	31.020	2.180	33.200	11.420	12.520	6.050
150	original	0.6	60x8 weeks	PCR90	Daily	VAC50	75	Jnvaccinate	1.232	0.006	0.000	0.007	0.002	0.003	0.001	591.219	3.102	0.218	3.320	1.142	1.252	0.605	5912	31.020	2.180	33.200	11.420	12.520	6.050
150	original	1	60x8 weeks	PCR90	Daily	VAC50	75	Jnvaccinate	1.231	0.007	0.001	0.007	0.003	0.003	0.001	590.942	3.138	0.382	3.520	1.243	1.311	0.570	5909	31.380	3.820	35.200	12.430	13.110	5.700
150	original	1	60x8 weeks	PCR90	Daily	VAC50	75	Jnvaccinate	1.231	0.007	0.001	0.007	0.003	0.003	0.001	590.942	3.138	0.382	3.520	1.243	1.311	0.570	5909	31.380	3.820	35.200	12.430	13.110	5.700
150	original	1	60x8 weeks	PCR90	Daily	VAC50	75	Jnvaccinate	1.231	0.007	0.001	0.007	0.003	0.003	0.001	590.942	3.138	0.382	3.520	1.243	1.311	0.570	5909	31.380	3.820	35.200	12.430	13.110	5.700
150	original	1.2	60x8 weeks	PCR90	Daily	VAC50	75	Jnvaccinate	1.230	0.006	0.001	0.007	0.002	0.003	0.001	590.635	3.094	0.458	3.552	1.169	1.347	0.550	5906	30.940	4.580	35.520	11.690	13.470	5.500
150	original	1.2	60x8 weeks	PCR90	Daily	VAC50	75	Jnvaccinate	1.230	0.006	0.001	0.007	0.002	0.003	0.001	590.635	3.094	0.458	3.552	1.169	1.347	0.550	5906	30.940	4.580	35.520	11.690	13.470	5.500
150	original	1.2	60x8 weeks	PCR90	Daily	VAC50	75	Jnvaccinate	1.230	0.006	0.001	0.007	0.002	0.003	0.001	590.635	3.094	0.458	3.552	1.169	1.347	0.550	5906	30.940	4.580	35.520	11.690	13.470	5.500
150	alpha	0.6	60x8 weeks	PCR90	Daily	VAC50	75	Jnvaccinate	1.225	0.006	0.003	0.009	0.004	0.004	0.001	587.812	3.113	1.317	4.430	1.753	1.728	0.562	5878	31.130	13.170	44.300	17.530	17.280	5.620
150	alpha	0.6	60x8 weeks	PCR90	Daily	VAC50	75	Jnvaccinate	1.225	0.006	0.003	0.009	0.004	0.004	0.001	587.812	3.113	1.317	4.430	1.753	1.728	0.562	5878	31.130	13.170	44.300	17.530	17.280	5.620
150	alpha	0.6	60x8 weeks	PCR90	Daily	VAC50	75	Jnvaccinate	1.225	0.006	0.003	0.009	0.004	0.004	0.001	587.812	3.113	1.317	4.430	1.753	1.728	0.562	5878	31.130	13.170	44.300	17.530	17.280	5.620
150	alpha	1	60x8 weeks	PCR90	Daily	VAC50	75	Jnvaccinate	1.217	0.006	0.006	0.012	0.005	0.005	0.001	584.238	2.970	2.922	5.892	2.416	2.215	0.579	5842	29.700	29.220	58.920	24.160	22.150	5.790
150	alpha	1	60x8 weeks	PCR90	Daily	VAC50	75	Jnvaccinate	1.217	0.006	0.006	0.012	0.005	0.005	0.001	584.238	2.970	2.922	5.892	2.416	2.215	0.579	5842	29.700	29.220	58.920	24.160	22.150	5.790
150	alpha	1	60x8 weeks	PCR90	Daily	VAC50	75	Jnvaccinate	1.217	0.006	0.006	0.012	0.005	0.005	0.001	584.238	2.970	2.922	5.892	2.416	2.215	0.579	5842	29.700	29.220	58.920	24.160	22.150	5.790
150	alpha	1.2	60x8 weeks	PCR90	Daily	VAC50	75	Jnvaccinate	1.210	0.006	0.009	0.015	0.006	0.006	0.001	580.914	3.090	4.194	7.284	3.074	2.680	0.517	5809	30.900	41.940	72.840	30.740	26.800	5.170
150	alpha	1.2	60x8 weeks	PCR90	Daily	VAC50	75	Jnvaccinate	1.210	0.006	0.009	0.015	0.006	0.006	0.001	580.914	3.090	4.194	7.284	3.074	2.680	0.517	5809	30.900	41.940	72.840	30.740	26.800	5.170
150	alpha	1.2	60x8 weeks	PCR90	Daily	VAC50	75	Jnvaccinate	1.210	0.006	0.009	0.015	0.006	0.006	0.001	580.914	3.090	4.194	7.284	3.074	2.680	0.517	5809	30.900	41.940	72.840	30.740	26.800	5.170
150	delta	0.6	60x8 weeks	PCR90	Daily	VAC50	75	Jnvaccinate	1.196	0.007	0.017	0.024	0.012	0.007	0.001	574.307	3.408	8.202	11.610	5.522	3.486	0.516	5743	34.080	82.020	116.100	55.220	34.860	5.160
150	delta	0.6	60x8 weeks	PCR90	Daily	VAC50	75	Jnvaccinate	1.196	0.007	0.017	0.024	0.012	0.007	0.001	574.307	3.408	8.202	11.610	5.522	3.486	0.516	5743	34.080	82.020	116.100	55.220	34.860	5.160
150	delta	0.6	60x8 weeks	PCR90	Daily	VAC50	75	Jnvaccinate	1.196	0.007	0.017	0.024	0.012	0.007	0.001	574.307	3.408	8.202	11.610	5.522	3.486	0.516	5743	34.080	82.020	116.100	55.220	34.860	5.160
150	delta	1	60x8 weeks	PCR90	Daily	VAC50	75	Jnvaccinate	1.113	0.006	0.059	0.065	0.032	0.018	0.001	534.151	2.806	28.345	31.151	15.394	8.703	0.382	5342	28.060	283.450	311.510	153.940	87.030	3.820
150	delta	1	60x8 weeks	PCR90	Daily	VAC50	75	Jnvaccinate	1.113	0.006	0.059	0.065	0.032	0.018	0.001	534.151	2.806	28.345	31.151	15.394	8.703	0.382	5342	28.060	283.450	311.510	153.940	87.030	3.820
150	delta	1	60x8 weeks	PCR90	Daily	VAC50	75	Jnvaccinate	1.113	0.006	0.059	0.065	0.032	0.018	0.001	534.151	2.806	28.345	31.151	15.394	8.703	0.382	5342	28.060	283.450	311.510	153.940	87.030	3.820
150	delta	1.2	60x8 weeks	PCR90	Daily	VAC50	75	Jnvaccinate	1.073	0.005	0.081	0.086	0.044	0.023	0.001	514.891	2.464	38.835	41.299	21.281	11.035	0.383	5149	24.640	388.350	412.990	212.810	110.350	3.830
150	delta	1.2	60x8 weeks	PCR90	Daily	VAC50	75	Jnvaccinate	1.073	0.005	0.081	0.086	0.044	0.023	0.001	514.891	2.464	38.835	41.299	21.281	11.035	0.383	5149	24.640	388.350	412.990	212.810	110.350	3.830
150	delta	1.2	60x8 weeks	PCR90	Daily	VAC50	75	Jnvaccinate	1.073	0.005	0.081	0.086	0.044	0.023	0.001	514.891	2.464	38.835	41.299	21.281	11.035	0.383	5149	24.640	388.350	412.990	212.810	110.350	3.830
150	original	0.6	60x8 weeks	PCR90	Daily	VAC50	100	Jnvaccinate	0.000	0.006	0.000	0.006	0.003	0.000	0.000	0.000	2.667	0.043	2.710	1.401	0.000	0.000	0	26.670	0.430	27.100	14.010	0.000	0.000
150	original	0.6	60x8 weeks	PCR90	Daily	VAC50	100	Jnvaccinate	0.000	0.006	0.000	0.006	0.003	0.000	0.000	0.000	2.667	0.043	2.710	1.401	0.000	0.000	0	26.670	0.430	27.100	14.010	0.000	0.000
150	original	0.6	60x8 weeks	PCR90	Daily	VAC50	100	Jnvaccinate	0.000	0.006	0.000	0.006	0.003	0.000	0.000	0.000	2.667	0.043	2.710	1.401	0.000	0.000	0	26.670	0.430	27.100	14.010	0.000	0.000
150	original	1	60x8 weeks	PCR90	Daily	VAC50	100	Jnvaccinate	0.000	0.005	0.000	0.006	0.003	0.000	0.000	0.000	2.587	0.057	2.644	1.389	0.000	0.000	0	25.870	0.570	26.440	13.890	0.000	0.000
150	original	1	60x8 weeks	PCR90	Daily	VAC50	100	Jnvaccinate	0.000	0.005	0.000	0.006	0.003	0.000	0.000	0.000	2.587	0.057	2.644	1.389	0.000	0.000	0	25.870	0.570	26.440	13.890	0.000	0.000
150	original	1	60x8 weeks	PCR90	Daily	VAC50	100	Jnvaccinate	0.000	0.005	0.000	0.006	0.003	0.000	0.000	0.000	2.587	0.057	2.644	1.389	0.000	0.000	0	25.870	0.570	26.440	13.890	0.000	0.000
150	original	1.2	60x8 weeks	PCR90	Daily	VAC50	100	Jnvaccinate	0.000	0.005	0.000	0.005	0.003	0.000	0.000	0.000	2.539	0.074	2.613	1.387	0.000	0.000	0	25.390	0.740	26.130	13.870	0.000	0.000
150	original	1.2	60x8 weeks	PCR90	Daily	VAC50	100	Jnvaccinate	0.000	0.005	0.000	0.005	0.003	0.000	0.000	0.000	2.539	0.074	2.613	1.387	0.000	0.000	0	25.390	0.740	26.130	13.870	0.000	0.000
150	original	1.2	60x8 weeks	PCR90	Daily	VAC50	100	Jnvaccinate	0.000	0.005	0.000	0.005	0.003	0.000	0.000	0.000	2.539	0.074	2.613	1.387	0.000	0.000	0	25.390	0.740	26.130	13.870	0.000	0.000
150	alpha	0.6	60x8 weeks	PCR90	Daily	VAC50	100	Jnvaccinate	0.000	0.005	0.002	0.007	0.004	0.000	0.000	0.000	2.550	0.953	3.503	2.130	0.000	0.000	0	25.500	9.530	35.030	21.300	0.000	0.000
150	alpha	0.6	60x8 weeks	PCR90	Daily	VAC50	100	Jnvaccinate	0.000	0.005	0.002	0.007	0.004	0.000	0.000	0.000	2.550	0.953	3.503	2.130	0.000	0.000	0	25.500	9.530	35.030	21.300	0.000	0.000
150	alpha	0.6	60x8 weeks	PCR90	Daily	VAC50	100	Jnvaccinate	0.000	0.005	0.002	0.007	0.004	0.000	0.000	0.000	2.550	0.953	3.503	2.130	0.000	0.000	0	25.500	9.530	35.030	21.300	0.000	0.000
150	alpha	1	60x8 weeks	PCR90	Daily	VAC50	100	Jnvaccinate	0.000	0.005	0.004	0.010	0.006	0.000	0.000	0.000	2.611	2.101	4.712	2.916	0.000	0.000	0	26.110	21.010	47.120	29.160	0.000	0.000
150	alpha	1	60x8 weeks	PCR90	Daily	VAC50	100	Jnvaccinate	0.000	0.005	0.004	0.010	0.006	0.000	0.000	0.000	2.611	2.101	4.712	2.916	0.000	0.000	0	26.110	21.010	47.120	29.160	0.000	0.000
150	alpha	1	60x8 weeks	PCR90	Daily	VAC50	100	Jnvaccinate	0.000	0.005	0.004	0.010	0.006	0.000	0.000	0.000	2.611	2.101	4.712	2.916	0.000	0.000	0	26.110	21.010	47.120	29.160	0.000	0.000
150	alpha	1.2	60x8 weeks	PCR90	Daily	VAC50	100	Jnvaccinate	0.000	0.005	0.006	0.011	0.007	0.000	0.000	0.000	2.583	2.812	5.395	3.277	0.000	0.000	0	25.830	28.120	53.950	32.770	0.000	0.000
150	alpha	1.2	60x8 weeks	PCR90	Daily	VAC50	100	Jnvaccinate	0.000	0.005	0.006	0.011	0.007	0.000	0.000	0.000	2.583	2.812	5.395	3.277	0.000	0.000	0	25.830	28.120	53.950	32.770	0.000	0.000
150	alpha	1.2	60x8 weeks	PCR90	Daily	VAC50	100	Jnvaccinate	0.000	0.005	0.006	0.011	0.007	0.000	0.000	0.000	2.583	2.812	5.395	3.277	0.000	0.000	0	25.830	28.120	53.950	32.770	0.000	0.000
150	delta	0.6	60x8 weeks	PCR90	Daily	VAC50	100	Jnvaccinate	0.000	0.007	0.014	0.021	0.014	0.000	0.000	0.000	3.125	6.955	10.080	6.481	0.000	0.000	0	31.250	69.550	100.800	64.810	0.000	0.000
150	delta	0.6	60x8 weeks	PCR90	Daily	VAC50	100	Jnvaccinate	0.000	0.007	0.014	0.021	0.014	0.000	0.000	0.000	3.125	6.955	10.080	6.481	0.000	0.000	0	31.250	69.550	100.800	64.810	0.000	0.000
150	delta	0.6	60x8 weeks	PCR90	Daily	VAC50	100	Jnvaccinate	0.000	0.007	0.014	0.021	0.014	0.000	0.000	0.000	3.125	6.955	10.080	6.481	0.000	0.000	0	31.250	69.550	100.800	64.810	0.000	0.000
150	delta	1	60x8 weeks	PCR90	Daily	VAC50	100	Jnvaccinate	0.000	0.006	0.046	0.052	0.034	0.000	0.000	0.000	2.747	22.300	25.047	16.150	0.000	0.000	0	27.470	223.000	250.470	161.500	0.000	0.000
150	delta	1	60x8 weeks	PCR90	Daily	VAC50	100	Jnvaccinate	0.000	0.006	0.046	0.052	0.034	0.000	0.000	0.000	2.747	22.300	25.047	16.150	0.000	0.000	0	27.470	223.000	250.470	161.500	0.000	0.000
150	delta	1	60x8 weeks	PCR90	Daily	VAC50	100	Jnvaccinate	0.000	0.006	0.046	0.052	0.034	0.000	0.000	0.000	2.747	22.300	25.047	16.150	0.000	0.000	0	27.470	223.000	250.470	161.500	0.000	0.000
150	delta	1.2	60x8 weeks	PCR90	Daily	VAC50	100	Jnvaccinate	0.000	0.005	0.070	0.075	0.049	0.000	0.000	0.000	2.429	33.569	35.998	23.341	0.000	0.000	0	24.290	335.690	359.980	233.410	0.000	0.000
150	delta	1.2	60x8 weeks	PCR90	Daily	VAC50	100	Jnvaccinate	0.000	0.005	0.070	0.075	0.049	0.000	0.000	0.000	2.429	33.569	35.998	23.341	0.000	0.000	0	24.290	335.690	359.980	233.410	0.000	0.000
150	delta	1.2	60x8 weeks	PCR90	Daily	VAC50	100	Jnvaccinate	0.000	0.005	0.070	0.075	0.049	0.000	0.000	0.000	2.429	33.569	35.998	23.341	0.000	0.000	0	24.290	335.690	359.980	233.410	0.000	0.000
150	original	0.6	60x8 weeks	PCR90	MWF	NOVAC	0	Jnvaccinate	2.950	0.010	0.004	0.014	0.001	0.013	0.003	1415.815	4.752	1.751	6.503	0.658	6.040	1.444	14158	47.520	17.510	65.030	6.580	60.400	14.440
150	original	0.6	60x8 weeks	PCR90	MWF	NOVAC	0	Jnvaccinate	2.950	0.010	0.004	0.014	0.001	0.013	0.003	1415.815	4.752	1.751	6.503	0.658	6.040	1.444	14158	47.520	17.510	65.030	6.580	60.400	14.440
150	original	0.6	60x8 weeks	PCR90	MWF	NOVAC	0	Jnvaccinate	2.950	0.010	0.004	0.014	0.001	0.013	0.003	1415.815	4.752	1.751	6.503	0.658	6.040	1.444	14158	47.520	17.510	65.030	6.580	60.400	14.440
150	original	1	60x8 weeks	PCR90	MWF	NOVAC	0	Jnvaccinate	2.935	0.010	0.008	0.017	0.002	0.016	0.003	1409.026	4.720	3.676	8.396	0.902	7.724	1.370	14090	47.200	36.760	83.960	9.020	77.240	13.700
150	original	1	60x8 weeks	PCR90	MWF	NOVAC	0	Jnvaccinate	2.935	0.010	0.008	0.017	0.002	0.016	0.003	1409.026	4.720	3.676	8.396	0.902	7.724	1.370	14090	47.200	36.760	83.960	9.020	77.240	13.700
150	original	1	60x8 weeks	PCR90	MWF	NOVAC	0	Jnvaccinate	2.935	0.010	0.008	0.017	0.002	0.016	0.003	1409.026	4.720	3.676	8.396	0.902	7.724	1.370	14090	47.200	36.760	83.960	9.020	77.240	13.700
150	original	1.2	60x8 weeks	PCR90	MWF	NOVAC	0	Jnvaccinate	2.925	0.010	0.011	0.020	0.002	0.019	0.003	1403.967	4.618	5.041	9.659	0.998	9.026	1.310	14040	46.180	50.410	96.590	9.980	90.260	13.100
150	original	1.2	60x8 weeks	PCR90	MWF	NOVAC	0	Jnvaccinate	2.925	0.010	0.011	0.020	0.002	0.019	0.003	1403.967	4.618	5.041	9.659	0.998	9.026	1.310	14040	46.180	50.410	96.590	9.980	90.260	13.100
150	original	1.2	60x8 weeks	PCR90	MWF	NOVAC	0	Jnvaccinate	2.925	0.010	0.011	0.020	0.002	0.019	0.003	1403.967	4.618	5.041	9.659	0.998	9.026	1.310	14040	46.180	50.410	96.590	9.980	90.260	13.100
150	alpha	0.6	60x8 weeks	PCR90	MWF	NOVAC	0	Jnvaccinate	2.924	0.010	0.010	0.020	0.002	0.019	0.003	1403.586	4.689	4.759	9.448	1.054	9.021	1.335	14036	46.890	47.590	94.480	10.540	90.210	13.350
150	alpha	0.6	60x8 weeks	PCR90	MWF	NOVAC	0	Jnvaccinate	2.924	0.010	0.010	0.020	0.002	0.019	0.003	1403.586	4.689	4.759	9.448	1.054	9.021	1.335	14036	46.890	47.590	94.480	10.540	90.210	13.350
150	alpha	0.6	60x8 weeks	PCR90	MWF	NOVAC	0	Jnvaccinate	2.924	0.010	0.010	0.020	0.002	0.019	0.003	1403.586	4.689	4.759	9.448	1.054	9.021	1.335	14036	46.890	47.590	94.480	10.540	90.210	13.350

150	alpha	1	60x8 weeks	PCR90	MWF	NOVAC	0	Jnvaccinate	2.847	0.009	0.030	0.039	0.004	0.038	0.002	1366.714	4.326	14.537	18.863	2.092	18.065	1.194	13667	43.260	145.370	188.630	20.920	180.650	11.940
150	alpha	1	60x8 weeks	PCR90	MWF	NOVAC	0	Jnvaccinate	2.847	0.009	0.030	0.039	0.004	0.038	0.002	1366.714	4.326	14.537	18.863	2.092	18.065	1.194	13667	43.260	145.370	188.630	20.920	180.650	11.940
150	alpha	1	60x8 weeks	PCR90	MWF	NOVAC	0	Jnvaccinate	2.847	0.009	0.030	0.039	0.004	0.038	0.002	1366.714	4.326	14.537	18.863	2.092	18.065	1.194	13667	43.260	145.370	188.630	20.920	180.650	11.940
150	alpha	1.2	60x8 weeks	PCR90	MWF	NOVAC	0	Jnvaccinate	2.790	0.008	0.045	0.053	0.006	0.051	0.002	1339.365	3.947	21.701	25.648	3.027	24.396	1.120	13394	39.470	217.010	256.480	30.270	243.960	11.200
150	alpha	1.2	60x8 weeks	PCR90	MWF	NOVAC	0	Jnvaccinate	2.790	0.008	0.045	0.053	0.006	0.051	0.002	1339.365	3.947	21.701	25.648	3.027	24.396	1.120	13394	39.470	217.010	256.480	30.270	243.960	11.200
150	alpha	1.2	60x8 weeks	PCR90	MWF	NOVAC	0	Jnvaccinate	2.790	0.008	0.045	0.053	0.006	0.051	0.002	1339.365	3.947	21.701	25.648	3.027	24.396	1.120	13394	39.470	217.010	256.480	30.270	243.960	11.200
150	delta	0.6	60x8 weeks	PCR90	MWF	NOVAC	0	Jnvaccinate	2.732	0.008	0.059	0.067	0.011	0.061	0.002	1311.345	3.649	28.395	32.044	5.215	29.219	1.091	13113	36.490	283.950	320.440	52.150	292.190	10.910
150	delta	0.6	60x8 weeks	PCR90	MWF	NOVAC	0	Jnvaccinate	2.732	0.008	0.059	0.067	0.011	0.061	0.002	1311.345	3.649	28.395	32.044	5.215	29.219	1.091	13113	36.490	283.950	320.440	52.150	292.190	10.910
150	delta	0.6	60x8 weeks	PCR90	MWF	NOVAC	0	Jnvaccinate	2.732	0.008	0.059	0.067	0.011	0.061	0.002	1311.345	3.649	28.395	32.044	5.215	29.219	1.091	13113	36.490	283.950	320.440	52.150	292.190	10.910
150	delta	1	60x8 weeks	PCR90	MWF	NOVAC	0	Jnvaccinate	2.514	0.005	0.110	0.115	0.020	0.106	0.001	1206.554	2.272	52.771	55.043	9.451	50.812	0.616	12066	22.720	527.710	550.430	94.510	508.120	6.160
150	delta	1	60x8 weeks	PCR90	MWF	NOVAC	0	Jnvaccinate	2.514	0.005	0.110	0.115	0.020	0.106	0.001	1206.554	2.272	52.771	55.043	9.451	50.812	0.616	12066	22.720	527.710	550.430	94.510	508.120	6.160
150	delta	1	60x8 weeks	PCR90	MWF	NOVAC	0	Jnvaccinate	2.514	0.005	0.110	0.115	0.020	0.106	0.001	1206.554	2.272	52.771	55.043	9.451	50.812	0.616	12066	22.720	527.710	550.430	94.510	508.120	6.160
150	delta	1.2	60x8 weeks	PCR90	MWF	NOVAC	0	Jnvaccinate	2.483	0.004	0.116	0.120	0.021	0.112	0.001	1191.838	2.068	55.576	57.644	10.062	53.545	0.553	11918	20.680	555.760	576.440	100.620	535.450	5.530
150	delta	1.2	60x8 weeks	PCR90	MWF	NOVAC	0	Jnvaccinate	2.483	0.004	0.116	0.120	0.021	0.112	0.001	1191.838	2.068	55.576	57.644	10.062	53.545	0.553	11918	20.680	555.760	576.440	100.620	535.450	5.530
150	delta	1.2	60x8 weeks	PCR90	MWF	NOVAC	0	Jnvaccinate	2.483	0.004	0.116	0.120	0.021	0.112	0.001	1191.838	2.068	55.576	57.644	10.062	53.545	0.553	11918	20.680	555.760	576.440	100.620	535.450	5.530
150	original	0.6	60x8 weeks	PCR90	MWF	VAC95	25	Jnvaccinate	2.216	0.008	0.002	0.009	0.001	0.009	0.002	1063.615	3.643	0.912	4.555	0.508	4.125	0.975	10636	36.430	9.120	45.550	5.080	41.250	9.750
150	original	0.6	60x8 weeks	PCR90	MWF	VAC95	25	Jnvaccinate	2.216	0.008	0.002	0.009	0.001	0.009	0.002	1063.615	3.643	0.912	4.555	0.508	4.125	0.975	10636	36.430	9.120	45.550	5.080	41.250	9.750
150	original	0.6	60x8 weeks	PCR90	MWF	VAC95	25	Jnvaccinate	2.216	0.008	0.002	0.009	0.001	0.009	0.002	1063.615	3.643	0.912	4.555	0.508	4.125	0.975	10636	36.430	9.120	45.550	5.080	41.250	9.750
150	original	1	60x8 weeks	PCR90	MWF	VAC95	25	Jnvaccinate	2.209	0.008	0.004	0.012	0.001	0.010	0.002	1060.161	3.699	1.847	5.546	0.597	4.996	1.022	10602	36.990	18.470	55.460	5.970	49.960	10.220
150	original	1	60x8 weeks	PCR90	MWF	VAC95	25	Jnvaccinate	2.209	0.008	0.004	0.012	0.001	0.010	0.002	1060.161	3.699	1.847	5.546	0.597	4.996	1.022	10602	36.990	18.470	55.460	5.970	49.960	10.220
150	original	1	60x8 weeks	PCR90	MWF	VAC95	25	Jnvaccinate	2.209	0.008	0.004	0.012	0.001	0.010	0.002	1060.161	3.699	1.847	5.546	0.597	4.996	1.022	10602	36.990	18.470	55.460	5.970	49.960	10.220
150	original	1.2	60x8 weeks	PCR90	MWF	VAC95	25	Jnvaccinate	2.205	0.008	0.005	0.012	0.001	0.011	0.002	1058.451	3.642	2.229	5.871	0.671	5.345	1.063	10585	36.420	22.290	58.710	6.710	53.450	10.630
150	original	1.2	60x8 weeks	PCR90	MWF	VAC95	25	Jnvaccinate	2.205	0.008	0.005	0.012	0.001	0.011	0.002	1058.451	3.642	2.229	5.871	0.671	5.345	1.063	10585	36.420	22.290	58.710	6.710	53.450	10.630
150	original	1.2	60x8 weeks	PCR90	MWF	VAC95	25	Jnvaccinate	2.205	0.008	0.005	0.012	0.001	0.011	0.002	1058.451	3.642	2.229	5.871	0.671	5.345	1.063	10585	36.420	22.290	58.710	6.710	53.450	10.630
150	alpha	0.6	60x8 weeks	PCR90	MWF	VAC95	25	Jnvaccinate	2.203	0.008	0.005	0.013	0.001	0.012	0.002	1057.613	3.632	2.378	6.010	0.700	5.551	0.974	10576	36.320	23.780	60.100	7.000	55.510	9.740
150	alpha	0.6	60x8 weeks	PCR90	MWF	VAC95	25	Jnvaccinate	2.203	0.008	0.005	0.013	0.001	0.012	0.002	1057.613	3.632	2.378	6.010	0.700	5.551	0.974	10576	36.320	23.780	60.100	7.000	55.510	9.740
150	alpha	0.6	60x8 weeks	PCR90	MWF	VAC95	25	Jnvaccinate	2.203	0.008	0.005	0.013	0.001	0.012	0.002	1057.613	3.632	2.378	6.010	0.700	5.551	0.974	10576	36.320	23.780	60.100	7.000	55.510	9.740
150	alpha	1	60x8 weeks	PCR90	MWF	VAC95	25	Jnvaccinate	2.177	0.007	0.012	0.019	0.002	0.018	0.002	1045.153	3.511	5.777	9.288	1.136	8.562	0.968	10452	35.110	57.770	92.880	11.360	85.620	9.680
150	alpha	1	60x8 weeks	PCR90	MWF	VAC95	25	Jnvaccinate	2.177	0.007	0.012	0.019	0.002	0.018	0.002	1045.153	3.511	5.777	9.288	1.136	8.562	0.968	10452	35.110	57.770	92.880	11.360	85.620	9.680
150	alpha	1	60x8 weeks	PCR90	MWF	VAC95	25	Jnvaccinate	2.177	0.007	0.012	0.019	0.002	0.018	0.002	1045.153	3.511	5.777	9.288	1.136	8.562	0.968	10452	35.110	57.770	92.880	11.360	85.620	9.680
150	alpha	1.2	60x8 weeks	PCR90	MWF	VAC95	25	Jnvaccinate	2.159	0.007	0.017	0.024	0.003	0.022	0.002	1036.276	3.404	8.340	11.744	1.460	10.723	0.928	10363	34.040	83.400	117.440	14.600	107.230	9.280
150	alpha	1.2	60x8 weeks	PCR90	MWF	VAC95	25	Jnvaccinate	2.159	0.007	0.017	0.024	0.003	0.022	0.002	1036.276	3.404	8.340	11.744	1.460	10.723	0.928	10363	34.040	83.400	117.440	14.600	107.230	9.280
150	alpha	1.2	60x8 weeks	PCR90	MWF	VAC95	25	Jnvaccinate	2.159	0.007	0.017	0.024	0.003	0.022	0.002	1036.276	3.404	8.340	11.744	1.460	10.723	0.928	10363	34.040	83.400	117.440	14.600	107.230	9.280
150	delta	0.6	60x8 weeks	PCR90	MWF	VAC95	25	Jnvaccinate	2.137	0.007	0.024	0.031	0.006	0.026	0.002	1025.955	3.364	11.396	14.760	2.709	12.543	0.922	10260	33.640	113.960	147.600	27.090	125.430	9.220
150	delta	0.6	60x8 weeks	PCR90	MWF	VAC95	25	Jnvaccinate	2.137	0.007	0.024	0.031	0.006	0.026	0.002	1025.955	3.364	11.396	14.760	2.709	12.543	0.922	10260	33.640	113.960	147.600	27.090	125.430	9.220
150	delta	0.6	60x8 weeks	PCR90	MWF	VAC95	25	Jnvaccinate	2.137	0.007	0.024	0.031	0.006	0.026	0.002	1025.955	3.364	11.396	14.760	2.709	12.543	0.922	10260	33.640	113.960	147.600	27.090	125.430	9.220
150	delta	1	60x8 weeks	PCR90	MWF	VAC95	25	Jnvaccinate	1.983	0.005	0.067	0.072	0.015	0.060	0.001	952.067	2.549	32.152	34.701	7.113	28.591	0.691	9521	25.490	321.520	347.010	71.130	285.910	6.910
150	delta	1	60x8 weeks	PCR90	MWF	VAC95	25	Jnvaccinate	1.983	0.005	0.067	0.072	0.015	0.060	0.001	952.067	2.549	32.152	34.701	7.113	28.591	0.691	9521	25.490	321.520	347.010	71.130	285.910	6.910
150	delta	1	60x8 weeks	PCR90	MWF	VAC95	25	Jnvaccinate	1.983	0.005	0.067	0.072	0.015	0.060	0.001	952.067	2.549	32.152	34.701	7.113	28.591	0.691	9521	25.490	321.520	347.010	71.130	285.910	6.910
150	delta	1.2	60x8 weeks	PCR90	MWF	VAC95	25	Jnvaccinate	1.933	0.005	0.082	0.086	0.019	0.070	0.001	927.774	2.173	39.153	41.326	9.021	33.402	0.555	9278	21.730	391.530	413.260	90.210	334.020	5.550
150	delta	1.2	60x8 weeks	PCR90	MWF	VAC95	25	Jnvaccinate	1.933	0.005	0.082	0.086	0.019	0.070	0.001	927.774	2.173	39.153	41.326	9.021	33.402	0.555	9278	21.730	391.530	413.260	90.210	334.020	5.550
150	delta	1.2	60x8 weeks	PCR90	MWF	VAC95	25	Jnvaccinate	1.933	0.005	0.082	0.086	0.019	0.070	0.001	927.774	2.173	39.153	41.326	9.021	33.402	0.555	9278	21.730	391.530	413.260	90.210	334.020	5.550
150	original	0.6	60x8 weeks	PCR90	MWF	VAC95	50	Jnvaccinate	1.478	0.005	0.001	0.006	0.001	0.005	0.001	709.619	2.585	0.388	2.973	0.328	2.602	0.656	7096	25.850	3.880	29.730	3.280	26.020	6.560
150	original	0.6	60x8 weeks	PCR90	MWF	VAC95	50	Jnvaccinate	1.478	0.005	0.001	0.006	0.001	0.005	0.001	709.619	2.585	0.388	2.973	0.328	2.602	0.656	7096	25.850	3.880	29.730	3.280	26.020	6.560
150	original	0.6	60x8 weeks	PCR90	MWF	VAC95	50	Jnvaccinate	1.478	0.005	0.001	0.006	0.001	0.005	0.001	709.619	2.585	0.388	2.973	0.328	2.602	0.656	7096	25.850	3.880	29.730	3.280	26.020	6.560
150	original	1	60x8 weeks	PCR90	MWF	VAC95	50	Jnvaccinate	1.476	0.005	0.002	0.007	0.001	0.006	0.001	708.655	2.576	0.736	3.312	0.397	2.845	0.663	7087	25.760	7.360	33.120	3.970	28.450	6.630
150	original	1	60x8 weeks	PCR90	MWF	VAC95	50	Jnvaccinate	1.476	0.005	0.002	0.007	0.001	0.006	0.001	708.655	2.576	0.736	3.312	0.397	2.845	0.663	7087	25.760	7.360	33.120	3.970	28.450	6.630
150	original	1	60x8 weeks	PCR90	MWF	VAC95	50	Jnvaccinate	1.476	0.005	0.002	0.007	0.001	0.006	0.001	708.655	2.576	0.736	3.312	0.397	2.845	0.663	7087	25.760	7.360	33.120	3.970	28.450	6.630
150	original	1.2	60x8 weeks	PCR90	MWF	VAC95	50	Jnvaccinate	1.475	0.005	0.002	0.007	0.001	0.006	0.001	708.157	2.600	0.852	3.452	0.430	2.947	0.670	7082	26.000	8.520	34.520	4.300	29.470	6.700
150	original	1.2	60x8 weeks	PCR90	MWF	VAC95	50	Jnvaccinate	1.475	0.005	0.002	0.007	0.001	0.006	0.001	708.157	2.600	0.852	3.452	0.430	2.947	0.670	7082	26.000	8.520	34.520	4.300	29.470	6.700
150	original	1.2	60x8 weeks	PCR90	MWF	VAC95	50	Jnvaccinate	1.475	0.005	0.002	0.007	0.001	0.006	0.001	708.157	2.600	0.852	3.452	0.430	2.947	0.670	7082	26.000	8.520	34.520	4.300	29.470	6.700
150	alpha	0.6	60x8 weeks	PCR90	MWF	VAC95	50	Jnvaccinate	1.474	0.006	0.002	0.008	0.001	0.007	0.001	707.396	2.658	0.969	3.627	0.506	3.137	0.649	7074	26.580	9.690	36.270	5.060	31.370	6.490
150	alpha	0.6	60x8 weeks	PCR90	MWF	VAC95	50	Jnvaccinate	1.474	0.006	0.002	0.008	0.001	0.007	0.001	707.396	2.658	0.969	3.627	0.506	3.137	0.649	7074	26.580	9.690	36.270	5.060	31.370	6.490
150	alpha	0.6	60x8 weeks	PCR90	MWF	VAC95	50	Jnvaccinate	1.474	0.006	0.002	0.008	0.001	0.007	0.001	707.396	2.658	0.969	3.627	0.506	3.137	0.649	7074	26.580	9.690	36.270	5.060	31.370	6.490
150	alpha	1	60x8 weeks	PCR90	MWF	VAC95	50	Jnvaccinate	1.469	0.005	0.004	0.009	0.001	0.008	0.001	704.918	2.460	1.907	4.367	0.634	3.754	0.699	7049	24.600	19.070	43.670	6.340	37.540	6.990
150	alpha	1	60x8 weeks	PCR90	MWF	VAC95	50	Jnvaccinate	1.469	0.005	0.004	0.009	0.001	0.008	0.001	704.918	2.460	1.907	4.367	0.634	3.754	0.699	7049	24.600	19.070	43.670	6.340	37.540	6.990
150	alpha	1	60x8 weeks	PCR90	MWF	VAC95	50	Jnvaccinate	1.469	0.005	0.004	0.009	0.001	0.008	0.001	704.918	2.460	1.907	4.367	0.634	3.754	0.699	7049	24.600	19.070	43.670	6.340	37.540	6.990
150	alpha	1.2	60x8 weeks	PCR90	MWF	VAC95	50	Jnvaccinate	1.463	0.005	0.006	0.011	0.002	0.009	0.001	702.225	2.464	2.718	5.182	0.763	4.400	0.628	7022	24.640	27.180	51.820	7.630	44.000	6.280
150	alpha	1.2	60x8 weeks	PCR90	MWF	VAC95	50	Jnvaccinate	1.463	0.005	0.006	0.011	0.002	0.009	0.001	702.225	2.464	2.718	5.182	0.763	4.400	0.628	7022	24.640	27.180	51.820	7.630	44.000	6.280
150	alpha	1.2	60x8 weeks	PCR90	MWF	VAC95	50	Jnvaccinate	1.463	0.005	0.006	0.011	0.002	0.009	0.001	702.225	2.464	2.718	5.182	0.763	4.400	0.628	7022	24.640	27.180	51.820	7.630	44.000	6.280
150	delta	0.6	60x8 weeks	PCR90	MWF	VAC95	50	Jnvaccinate	1.456	0.005	0.008	0.013	0.003	0.010	0.001	699.070	2.631	3.778	6.409	1.367	4.859	0.674	6991	26.310	37.780	64.090	13.670	48.590	6.740
150	delta	0.6	60x8 weeks	PCR90	MWF	VAC95	50	Jnvaccinate	1.456	0.005	0.008	0.013	0.003	0.010	0.001	699.070	2.631	3.778	6.409	1.367	4.859	0.674	6991	26.310	37.780	64.090	13.670	48.590	6.740
150	delta	0.6	60x8 weeks	PCR90	MWF	VAC95	50	Jnvaccinate	1.456	0.005	0.008	0.013	0.003	0.010	0.001	699.070	2.631	3.778	6.409	1.367	4.859	0.674	6991	26.310	37.780	64.090	13.670	48.590	6.740
150	delta	1	60x8 weeks	PCR90	MWF	VAC95	50	Jnvaccinate	1.408	0.005	0.023	0.028	0.006	0.021	0.001	675.849	2.411	11.179	13.590	3.107	10.149	0.578	6758	24.110	111.790	135.900	31.070	101.490	5.780
150	delta	1	60x8 weeks	PCR90	MWF	VAC95	50	Jnvaccinate	1.408	0.005	0.023	0.028	0.006	0.021	0.001	675.849	2.411	11.179	13.590	3.107	10.149	0.578	6758	24.110	111.790	135.900	31.070	101.490	5.780
150	delta	1	60x8 weeks	PCR90	MWF	VAC95	50	Jnvaccinate	1.408	0.005	0.023	0.028	0.006	0.021	0.001	675.849	2.411	11.179	13.590	3.107	10.149	0.578	6758	24.110	111.790	135.900	31.070	101.490	5.780
150	delta	1.2	60x8 weeks	PCR90	MWF	VAC95	50	Jnvaccinate	1.378	0.005	0.034	0.039	0.009	0.028	0.001	661.596	2.200	16.418	18.618	4.487	13.387	0.523	6616	22.000	164.180	186.180	44.870	133.870	5.230
150	delta	1.2	60x8 weeks	PCR90	MWF	VAC95	50	Jnvaccinate	1.378	0.005	0.034	0.039	0.009	0.028	0.001	661.596	2.200	16.418	18.618	4.487	13.387	0.523	6616	22.000	164.180	186.180	44.870	133.870	5.230
150	delta	1.2	60x8 weeks	PCR90	MWF	VAC95	50	Jnvaccinate	1.378	0.005	0.034	0.039	0.009	0.028	0.001	661.596	2.200	16.418	18.618	4.487	13.387	0.523	6616	22.000	164.180	186.180	44.870	133.870	5.230
150	original	0.6	60x8 weeks	PCR90	MWF	VAC95	75	Jnvaccinate	0.740	0.003	0.000	0.003	0.000	0.002	0.001	355.418	1.398	0.103	1.501	0.211	1.168	0.377	3554	13.980	1.030	15.010	2.110	11.680	3.770
150	original	0.6	60x8 weeks	PCR90	MWF	VAC95	75	Jnvaccinate	0.740	0.003	0.000	0.003	0.000	0.002	0.001	355.418	1.398	0.103	1.501	0.211	1.168	0.377	3554	13.980	1.030	15.010	2.110	11.680	3.770

150	original	0.6	60x8 weeks	PCR90	MWF	VAC95	75	Jnvaccinate	0.740	0.003	0.000	0.003	0.000	0.002	0.001	355.418	1.398	0.103	1.501	0.211	1.168	0.377	3554	13.980	1.030	15.010	2.110	11.680	3.770
150	original	1	60x8 weeks	PCR90	MWF	VAC95	75	Jnvaccinate	0.739	0.003	0.000	0.004	0.000	0.003	0.001	354.528	1.578	0.203	1.781	0.239	1.393	0.333	3545	15.780	2.030	17.810	2.390	13.930	3.330
150	original	1	60x8 weeks	PCR90	MWF	VAC95	75	Jnvaccinate	0.739	0.003	0.000	0.004	0.000	0.003	0.001	354.528	1.578	0.203	1.781	0.239	1.393	0.333	3545	15.780	2.030	17.810	2.390	13.930	3.330
150	original	1	60x8 weeks	PCR90	MWF	VAC95	75	Jnvaccinate	0.739	0.003	0.000	0.004	0.000	0.003	0.001	354.528	1.578	0.203	1.781	0.239	1.393	0.333	3545	15.780	2.030	17.810	2.390	13.930	3.330
150	original	1.2	60x8 weeks	PCR90	MWF	VAC95	75	Jnvaccinate	0.739	0.003	0.000	0.003	0.001	0.003	0.001	354.921	1.453	0.212	1.665	0.256	1.261	0.364	3549	14.530	2.120	16.650	2.560	12.610	3.640
150	original	1.2	60x8 weeks	PCR90	MWF	VAC95	75	Jnvaccinate	0.739	0.003	0.000	0.003	0.001	0.003	0.001	354.921	1.453	0.212	1.665	0.256	1.261	0.364	3549	14.530	2.120	16.650	2.560	12.610	3.640
150	original	1.2	60x8 weeks	PCR90	MWF	VAC95	75	Jnvaccinate	0.739	0.003	0.000	0.003	0.001	0.003	0.001	354.921	1.453	0.212	1.665	0.256	1.261	0.364	3549	14.530	2.120	16.650	2.560	12.610	3.640
150	alpha	0.6	60x8 weeks	PCR90	MWF	VAC95	75	Jnvaccinate	0.738	0.003	0.001	0.004	0.001	0.003	0.001	354.431	1.567	0.283	1.850	0.374	1.374	0.321	3544	15.670	2.830	18.500	3.740	13.740	3.210
150	alpha	0.6	60x8 weeks	PCR90	MWF	VAC95	75	Jnvaccinate	0.738	0.003	0.001	0.004	0.001	0.003	0.001	354.431	1.567	0.283	1.850	0.374	1.374	0.321	3544	15.670	2.830	18.500	3.740	13.740	3.210
150	alpha	0.6	60x8 weeks	PCR90	MWF	VAC95	75	Jnvaccinate	0.738	0.003	0.001	0.004	0.001	0.003	0.001	354.431	1.567	0.283	1.850	0.374	1.374	0.321	3544	15.670	2.830	18.500	3.740	13.740	3.210
150	alpha	1	60x8 weeks	PCR90	MWF	VAC95	75	Jnvaccinate	0.737	0.003	0.001	0.004	0.001	0.003	0.001	353.928	1.536	0.565	2.101	0.379	1.534	0.346	3539	15.360	5.650	21.010	3.790	15.340	3.460
150	alpha	1	60x8 weeks	PCR90	MWF	VAC95	75	Jnvaccinate	0.737	0.003	0.001	0.004	0.001	0.003	0.001	353.928	1.536	0.565	2.101	0.379	1.534	0.346	3539	15.360	5.650	21.010	3.790	15.340	3.460
150	alpha	1	60x8 weeks	PCR90	MWF	VAC95	75	Jnvaccinate	0.737	0.003	0.001	0.004	0.001	0.003	0.001	353.928	1.536	0.565	2.101	0.379	1.534	0.346	3539	15.360	5.650	21.010	3.790	15.340	3.460
150	alpha	1.2	60x8 weeks	PCR90	MWF	VAC95	75	Jnvaccinate	0.737	0.003	0.001	0.004	0.001	0.003	0.001	353.632	1.498	0.642	2.140	0.416	1.589	0.355	3536	14.980	6.420	21.400	4.160	15.890	3.550
150	alpha	1.2	60x8 weeks	PCR90	MWF	VAC95	75	Jnvaccinate	0.737	0.003	0.001	0.004	0.001	0.003	0.001	353.632	1.498	0.642	2.140	0.416	1.589	0.355	3536	14.980	6.420	21.400	4.160	15.890	3.550
150	alpha	1.2	60x8 weeks	PCR90	MWF	VAC95	75	Jnvaccinate	0.737	0.003	0.001	0.004	0.001	0.003	0.001	353.632	1.498	0.642	2.140	0.416	1.589	0.355	3536	14.980	6.420	21.400	4.160	15.890	3.550
150	delta	0.6	60x8 weeks	PCR90	MWF	VAC95	75	Jnvaccinate	0.735	0.004	0.002	0.006	0.002	0.003	0.001	352.928	1.699	1.022	2.721	0.783	1.652	0.342	3529	16.990	10.220	27.210	7.830	16.520	3.420
150	delta	0.6	60x8 weeks	PCR90	MWF	VAC95	75	Jnvaccinate	0.735	0.004	0.002	0.006	0.002	0.003	0.001	352.928	1.699	1.022	2.721	0.783	1.652	0.342	3529	16.990	10.220	27.210	7.830	16.520	3.420
150	delta	0.6	60x8 weeks	PCR90	MWF	VAC95	75	Jnvaccinate	0.735	0.004	0.002	0.006	0.002	0.003	0.001	352.928	1.699	1.022	2.721	0.783	1.652	0.342	3529	16.990	10.220	27.210	7.830	16.520	3.420
150	delta	1	60x8 weeks	PCR90	MWF	VAC95	75	Jnvaccinate	0.728	0.004	0.005	0.009	0.003	0.005	0.001	349.254	1.685	2.585	4.270	1.281	2.478	0.340	3493	16.850	25.850	42.700	12.810	24.780	3.400
150	delta	1	60x8 weeks	PCR90	MWF	VAC95	75	Jnvaccinate	0.728	0.004	0.005	0.009	0.003	0.005	0.001	349.254	1.685	2.585	4.270	1.281	2.478	0.340	3493	16.850	25.850	42.700	12.810	24.780	3.400
150	delta	1	60x8 weeks	PCR90	MWF	VAC95	75	Jnvaccinate	0.728	0.004	0.005	0.009	0.003	0.005	0.001	349.254	1.685	2.585	4.270	1.281	2.478	0.340	3493	16.850	25.850	42.700	12.810	24.780	3.400
150	delta	1.2	60x8 weeks	PCR90	MWF	VAC95	75	Jnvaccinate	0.724	0.003	0.007	0.010	0.003	0.006	0.001	347.530	1.633	3.391	5.024	1.443	2.914	0.321	3475	16.330	33.910	50.240	14.430	29.140	3.210
150	delta	1.2	60x8 weeks	PCR90	MWF	VAC95	75	Jnvaccinate	0.724	0.003	0.007	0.010	0.003	0.006	0.001	347.530	1.633	3.391	5.024	1.443	2.914	0.321	3475	16.330	33.910	50.240	14.430	29.140	3.210
150	delta	1.2	60x8 weeks	PCR90	MWF	VAC95	75	Jnvaccinate	0.724	0.003	0.007	0.010	0.003	0.006	0.001	347.530	1.633	3.391	5.024	1.443	2.914	0.321	3475	16.330	33.910	50.240	14.430	29.140	3.210
150	original	0.6	60x8 weeks	PCR90	MWF	VAC95	100	Jnvaccinate	0.000	0.001	0.000	0.001	0.000	0.000	0.000	0.000	0.330	0.000	0.330	0.123	0.000	0.000	0	3.300	0.000	3.300	1.230	0.000	0.000
150	original	0.6	60x8 weeks	PCR90	MWF	VAC95	100	Jnvaccinate	0.000	0.001	0.000	0.001	0.000	0.000	0.000	0.000	0.330	0.000	0.330	0.123	0.000	0.000	0	3.300	0.000	3.300	1.230	0.000	0.000
150	original	0.6	60x8 weeks	PCR90	MWF	VAC95	100	Jnvaccinate	0.000	0.001	0.000	0.001	0.000	0.000	0.000	0.000	0.330	0.000	0.330	0.123	0.000	0.000	0	3.300	0.000	3.300	1.230	0.000	0.000
150	original	1	60x8 weeks	PCR90	MWF	VAC95	100	Jnvaccinate	0.000	0.001	0.000	0.001	0.000	0.000	0.000	0.000	0.350	0.002	0.352	0.141	0.000	0.000	0	3.500	0.020	3.520	1.410	0.000	0.000
150	original	1	60x8 weeks	PCR90	MWF	VAC95	100	Jnvaccinate	0.000	0.001	0.000	0.001	0.000	0.000	0.000	0.000	0.350	0.002	0.352	0.141	0.000	0.000	0	3.500	0.020	3.520	1.410	0.000	0.000
150	original	1	60x8 weeks	PCR90	MWF	VAC95	100	Jnvaccinate	0.000	0.001	0.000	0.001	0.000	0.000	0.000	0.000	0.350	0.002	0.352	0.141	0.000	0.000	0	3.500	0.020	3.520	1.410	0.000	0.000
150	original	1.2	60x8 weeks	PCR90	MWF	VAC95	100	Jnvaccinate	0.000	0.001	0.000	0.001	0.000	0.000	0.000	0.000	0.360	0.001	0.361	0.144	0.000	0.000	0	3.600	0.010	3.610	1.440	0.000	0.000
150	original	1.2	60x8 weeks	PCR90	MWF	VAC95	100	Jnvaccinate	0.000	0.001	0.000	0.001	0.000	0.000	0.000	0.000	0.360	0.001	0.361	0.144	0.000	0.000	0	3.600	0.010	3.610	1.440	0.000	0.000
150	original	1.2	60x8 weeks	PCR90	MWF	VAC95	100	Jnvaccinate	0.000	0.001	0.000	0.001	0.000	0.000	0.000	0.000	0.360	0.001	0.361	0.144	0.000	0.000	0	3.600	0.010	3.610	1.440	0.000	0.000
150	alpha	0.6	60x8 weeks	PCR90	MWF	VAC95	100	Jnvaccinate	0.000	0.001	0.000	0.001	0.000	0.000	0.000	0.000	0.427	0.021	0.448	0.229	0.000	0.000	0	4.270	0.210	4.480	2.290	0.000	0.000
150	alpha	0.6	60x8 weeks	PCR90	MWF	VAC95	100	Jnvaccinate	0.000	0.001	0.000	0.001	0.000	0.000	0.000	0.000	0.427	0.021	0.448	0.229	0.000	0.000	0	4.270	0.210	4.480	2.290	0.000	0.000
150	alpha	0.6	60x8 weeks	PCR90	MWF	VAC95	100	Jnvaccinate	0.000	0.001	0.000	0.001	0.000	0.000	0.000	0.000	0.427	0.021	0.448	0.229	0.000	0.000	0	4.270	0.210	4.480	2.290	0.000	0.000
150	alpha	1	60x8 weeks	PCR90	MWF	VAC95	100	Jnvaccinate	0.000	0.001	0.000	0.001	0.000	0.000	0.000	0.000	0.469	0.030	0.499	0.226	0.000	0.000	0	4.690	0.300	4.990	2.260	0.000	0.000
150	alpha	1	60x8 weeks	PCR90	MWF	VAC95	100	Jnvaccinate	0.000	0.001	0.000	0.001	0.000	0.000	0.000	0.000	0.469	0.030	0.499	0.226	0.000	0.000	0	4.690	0.300	4.990	2.260	0.000	0.000
150	alpha	1	60x8 weeks	PCR90	MWF	VAC95	100	Jnvaccinate	0.000	0.001	0.000	0.001	0.000	0.000	0.000	0.000	0.469	0.030	0.499	0.226	0.000	0.000	0	4.690	0.300	4.990	2.260	0.000	0.000
150	alpha	1.2	60x8 weeks	PCR90	MWF	VAC95	100	Jnvaccinate	0.000	0.001	0.000	0.001	0.000	0.000	0.000	0.000	0.411	0.035	0.446	0.218	0.000	0.000	0	4.110	0.350	4.460	2.180	0.000	0.000
150	alpha	1.2	60x8 weeks	PCR90	MWF	VAC95	100	Jnvaccinate	0.000	0.001	0.000	0.001	0.000	0.000	0.000	0.000	0.411	0.035	0.446	0.218	0.000	0.000	0	4.110	0.350	4.460	2.180	0.000	0.000
150	alpha	1.2	60x8 weeks	PCR90	MWF	VAC95	100	Jnvaccinate	0.000	0.001	0.000	0.001	0.000	0.000	0.000	0.000	0.411	0.035	0.446	0.218	0.000	0.000	0	4.110	0.350	4.460	2.180	0.000	0.000
150	delta	0.6	60x8 weeks	PCR90	MWF	VAC95	100	Jnvaccinate	0.000	0.001	0.000	0.002	0.001	0.000	0.000	0.000	0.695	0.142	0.837	0.471	0.000	0.000	0	6.950	1.420	8.370	4.710	0.000	0.000
150	delta	0.6	60x8 weeks	PCR90	MWF	VAC95	100	Jnvaccinate	0.000	0.001	0.000	0.002	0.001	0.000	0.000	0.000	0.695	0.142	0.837	0.471	0.000	0.000	0	6.950	1.420	8.370	4.710	0.000	0.000
150	delta	0.6	60x8 weeks	PCR90	MWF	VAC95	100	Jnvaccinate	0.000	0.001	0.000	0.002	0.001	0.000	0.000	0.000	0.695	0.142	0.837	0.471	0.000	0.000	0	6.950	1.420	8.370	4.710	0.000	0.000
150	delta	1	60x8 weeks	PCR90	MWF	VAC95	100	Jnvaccinate	0.000	0.002	0.001	0.002	0.001	0.000	0.000	0.000	0.729	0.244	0.973	0.517	0.000	0.000	0	7.290	2.440	9.730	5.170	0.000	0.000
150	delta	1	60x8 weeks	PCR90	MWF	VAC95	100	Jnvaccinate	0.000	0.002	0.001	0.002	0.001	0.000	0.000	0.000	0.729	0.244	0.973	0.517	0.000	0.000	0	7.290	2.440	9.730	5.170	0.000	0.000
150	delta	1	60x8 weeks	PCR90	MWF	VAC95	100	Jnvaccinate	0.000	0.002	0.001	0.002	0.001	0.000	0.000	0.000	0.729	0.244	0.973	0.517	0.000	0.000	0	7.290	2.440	9.730	5.170	0.000	0.000
150	delta	1.2	60x8 weeks	PCR90	MWF	VAC95	100	Jnvaccinate	0.000	0.001	0.001	0.002	0.001	0.000	0.000	0.000	0.707	0.342	1.049	0.578	0.000	0.000	0	7.070	3.420	10.490	5.780	0.000	0.000
150	delta	1.2	60x8 weeks	PCR90	MWF	VAC95	100	Jnvaccinate	0.000	0.001	0.001	0.002	0.001	0.000	0.000	0.000	0.707	0.342	1.049	0.578	0.000	0.000	0	7.070	3.420	10.490	5.780	0.000	0.000
150	delta	1.2	60x8 weeks	PCR90	MWF	VAC95	100	Jnvaccinate	0.000	0.001	0.001	0.002	0.001	0.000	0.000	0.000	0.707	0.342	1.049	0.578	0.000	0.000	0	7.070	3.420	10.490	5.780	0.000	0.000
150	original	0.6	60x8 weeks	PCR90	MWF	VAC70	25	Jnvaccinate	2.216	0.008	0.002	0.010	0.001	0.009	0.002	1063.558	4.014	1.001	5.015	0.710	4.167	1.036	10636	40.140	10.010	50.150	7.100	41.670	10.360
150	original	0.6	60x8 weeks	PCR90	MWF	VAC70	25	Jnvaccinate	2.216	0.008	0.002	0.010	0.001	0.009	0.002	1063.558	4.014	1.001	5.015	0.710	4.167	1.036	10636	40.140	10.010	50.150	7.100	41.670	10.360
150	original	0.6	60x8 weeks	PCR90	MWF	VAC70	25	Jnvaccinate	2.216	0.008	0.002	0.010	0.001	0.009	0.002	1063.558	4.014	1.001	5.015	0.710	4.167	1.036	10636	40.140	10.010	50.150	7.100	41.670	10.360
150	original	1	60x8 weeks	PCR90	MWF	VAC70	25	Jnvaccinate	2.209	0.008	0.004	0.012	0.002	0.010	0.002	1060.449	3.950	1.918	5.868	0.844	4.850	1.023	10604	39.500	19.180	58.680	8.440	48.500	10.230
150	original	1	60x8 weeks	PCR90	MWF	VAC70	25	Jnvaccinate	2.209	0.008	0.004	0.012	0.002	0.010	0.002	1060.449	3.950	1.918	5.868	0.844	4.850	1.023	10604	39.500	19.180	58.680	8.440	48.500	10.230
150	original	1	60x8 weeks	PCR90	MWF	VAC70	25	Jnvaccinate	2.209	0.008	0.004	0.012	0.002	0.010	0.002	1060.449	3.950	1.918	5.868	0.844	4.850	1.023	10604	39.500	19.180	58.680	8.440	48.500	10.230
150	original	1.2	60x8 weeks	PCR90	MWF	VAC70	25	Jnvaccinate	2.205	0.008	0.005	0.013	0.002	0.011	0.002	1058.415	3.973	2.493	6.466	0.853	5.428	1.030	10584	39.730	24.930	64.660	8.530	54.280	10.300
150	original	1.2	60x8 weeks	PCR90	MWF	VAC70	25	Jnvaccinate	2.205	0.008	0.005	0.013	0.002	0.011	0.002	1058.415	3.973	2.493	6.466	0.853	5.428	1.030	10584	39.730	24.930	64.660	8.530	54.280	10.300
150	original	1.2	60x8 weeks	PCR90	MWF	VAC70	25	Jnvaccinate	2.205	0.008	0.005	0.013	0.002	0.011	0.002	1058.415	3.973	2.493	6.466	0.853	5.428	1.030	10584	39.730	24.930	64.660	8.530	54.280	10.300
150	alpha	0.6	60x8 weeks	PCR90	MWF	VAC70	25	Jnvaccinate	2.202	0.008	0.006	0.014	0.002	0.012	0.002	1056.935	3.978	2.780	6.758	1.108	5.689	1.017	10569	39.780	27.800	67.580	11.080	56.890	10.170
150	alpha	0.6	60x8 weeks	PCR90	MWF	VAC70	25	Jnvaccinate	2.202	0.008	0.006	0.014	0.002	0.012	0.002	1056.935	3.978	2.780	6.758	1.108	5.689	1.017	10569	39.780	27.800	67.580	11.080	56.890	10.170
150	alpha	0.6	60x8 weeks	PCR90	MWF	VAC70	25	Jnvaccinate	2.202	0.008	0.006	0.014	0.002	0.012	0.002	1056.935	3.978	2.780	6.758	1.108	5.689	1.017	10569	39.780	27.800	67.580	11.080	56.890	10.170
150	alpha	1	60x8 weeks	PCR90	MWF	VAC70	25	Jnvaccinate	2.170	0.008	0.015	0.023	0.004	0.020	0.002	1041.651	3.734	7.269	11.003	1.787	9.412	0.930	10417	37.340	72.690	110.030	17.870	94.120	9.300
150	alpha	1	60x8 weeks	PCR90	MWF	VAC70	25	Jnvaccinate	2.170	0.008	0.015	0.023	0.004	0.020	0.002	1041.651	3.734	7.269	11.003	1.787	9.412	0.930	10417	37.340	72.690	110.030	17.870	94.120	9.300
150	alpha	1	60x8 weeks	PCR90	MWF	VAC70	25	Jnvaccinate	2.170	0.008	0.015	0.023	0.004	0.020	0.002	1041.651	3.734	7.269	11.003	1.787	9.412	0.930	10417	37.340	72.690	110.030	17.870	94.120	9.300
150	alpha	1.2	60x8 weeks	PCR90	MWF	VAC70	25	Jnvaccinate	2.144	0.007	0.024	0.031	0.005	0.026	0.002	1029.256	3.567	11.498	15.065	2.618	12.416	0.905	10293	35.670	114.980	150.650	26.180	124.160	9.050
150	alpha	1.2	60x8 weeks	PCR90	MWF	VAC70	25	Jnvaccinate	2.144	0.007	0.024	0.031	0.005	0.026	0.002	1029.256	3.567	11.498	15.065	2.618	12.416	0.905	10293	35.670	114.980	150.650	26.180	124.160	9.050
150	alpha	1.2	60x8 weeks	PCR90	MWF	VAC70	25	Jnvaccinate	2.144	0.007	0.024	0.031	0.005	0.026	0.002	1029.256	3.567	11.498	15.065	2.618	12.416	0.905	10293	35.670	114.980	150.650	26.180	124.160	9.050
150	delta	0.6	60x8 weeks	PCR90	MWF	VAC70	25	Jnvaccinate	2.120	0.007	0.032	0.040	0.009	0.030	0.002	1017.570	3.474	15.486	18.960	4.413	14.460	0.915	10176	34.740	154.860	189.600	44.130	144.600	9.150

150	delta	0.6	60x8 weeks	PCR90	MWF	VAC70	25	Unvaccinate	2.120	0.007	0.032	0.040	0.009	0.030	0.002	1017.570	3.474	15.486	18.960	4.413	14.460	0.915	10176	34.740	154.860	189.600	44.130	144.600	9.150
150	delta	0.6	60x8 weeks	PCR90	MWF	VAC70	25	Unvaccinate	2.120	0.007	0.032	0.040	0.009	0.030	0.002	1017.570	3.474	15.486	18.960	4.413	14.460	0.915	10176	34.740	154.860	189.600	44.130	144.600	9.150
150	delta	1	60x8 weeks	PCR90	MWF	VAC70	25	Unvaccinate	1.935	0.005	0.089	0.094	0.025	0.070	0.001	928.943	2.526	42.769	45.295	11.927	33.418	0.599	9289	25.260	427.690	452.950	119.270	334.180	5.990
150	delta	1	60x8 weeks	PCR90	MWF	VAC70	25	Unvaccinate	1.935	0.005	0.089	0.094	0.025	0.070	0.001	928.943	2.526	42.769	45.295	11.927	33.418	0.599	9289	25.260	427.690	452.950	119.270	334.180	5.990
150	delta	1	60x8 weeks	PCR90	MWF	VAC70	25	Unvaccinate	1.935	0.005	0.089	0.094	0.025	0.070	0.001	928.943	2.526	42.769	45.295	11.927	33.418	0.599	9289	25.260	427.690	452.950	119.270	334.180	5.990
150	delta	1.2	60x8 weeks	PCR90	MWF	VAC70	25	Unvaccinate	1.896	0.004	0.102	0.106	0.029	0.077	0.001	910.249	2.126	48.877	51.003	14.062	36.915	0.522	9102	21.260	488.770	510.030	140.620	369.150	5.220
150	delta	1.2	60x8 weeks	PCR90	MWF	VAC70	25	Unvaccinate	1.896	0.004	0.102	0.106	0.029	0.077	0.001	910.249	2.126	48.877	51.003	14.062	36.915	0.522	9102	21.260	488.770	510.030	140.620	369.150	5.220
150	delta	1.2	60x8 weeks	PCR90	MWF	VAC70	25	Unvaccinate	1.896	0.004	0.102	0.106	0.029	0.077	0.001	910.249	2.126	48.877	51.003	14.062	36.915	0.522	9102	21.260	488.770	510.030	140.620	369.150	5.220
150	original	0.6	60x8 weeks	PCR90	MWF	VAC70	50	Unvaccinate	1.478	0.007	0.001	0.008	0.002	0.005	0.001	709.675	3.231	0.500	3.731	0.725	2.601	0.713	7097	32.310	5.000	37.310	7.250	26.010	7.130
150	original	0.6	60x8 weeks	PCR90	MWF	VAC70	50	Unvaccinate	1.478	0.007	0.001	0.008	0.002	0.005	0.001	709.675	3.231	0.500	3.731	0.725	2.601	0.713	7097	32.310	5.000	37.310	7.250	26.010	7.130
150	original	0.6	60x8 weeks	PCR90	MWF	VAC70	50	Unvaccinate	1.478	0.007	0.001	0.008	0.002	0.005	0.001	709.675	3.231	0.500	3.731	0.725	2.601	0.713	7097	32.310	5.000	37.310	7.250	26.010	7.130
150	original	1	60x8 weeks	PCR90	MWF	VAC70	50	Unvaccinate	1.476	0.007	0.002	0.009	0.002	0.006	0.001	708.396	3.200	0.970	4.170	0.821	2.943	0.689	7084	32.000	9.700	41.700	8.210	29.430	6.890
150	original	1	60x8 weeks	PCR90	MWF	VAC70	50	Unvaccinate	1.476	0.007	0.002	0.009	0.002	0.006	0.001	708.396	3.200	0.970	4.170	0.821	2.943	0.689	7084	32.000	9.700	41.700	8.210	29.430	6.890
150	original	1	60x8 weeks	PCR90	MWF	VAC70	50	Unvaccinate	1.476	0.007	0.002	0.009	0.002	0.006	0.001	708.396	3.200	0.970	4.170	0.821	2.943	0.689	7084	32.000	9.700	41.700	8.210	29.430	6.890
150	original	1.2	60x8 weeks	PCR90	MWF	VAC70	50	Unvaccinate	1.476	0.007	0.002	0.009	0.002	0.006	0.001	708.339	3.146	1.140	4.286	0.921	2.907	0.661	7083	31.460	11.400	42.860	9.210	29.070	6.610
150	original	1.2	60x8 weeks	PCR90	MWF	VAC70	50	Unvaccinate	1.476	0.007	0.002	0.009	0.002	0.006	0.001	708.339	3.146	1.140	4.286	0.921	2.907	0.661	7083	31.460	11.400	42.860	9.210	29.070	6.610
150	original	1.2	60x8 weeks	PCR90	MWF	VAC70	50	Unvaccinate	1.476	0.007	0.002	0.009	0.002	0.006	0.001	708.339	3.146	1.140	4.286	0.921	2.907	0.661	7083	31.460	11.400	42.860	9.210	29.070	6.610
150	alpha	0.6	60x8 weeks	PCR90	MWF	VAC70	50	Unvaccinate	1.472	0.006	0.003	0.010	0.002	0.007	0.001	706.486	3.102	1.610	4.712	1.077	3.347	0.659	7065	31.020	16.100	47.120	10.770	33.470	6.590
150	alpha	0.6	60x8 weeks	PCR90	MWF	VAC70	50	Unvaccinate	1.472	0.006	0.003	0.010	0.002	0.007	0.001	706.486	3.102	1.610	4.712	1.077	3.347	0.659	7065	31.020	16.100	47.120	10.770	33.470	6.590
150	alpha	0.6	60x8 weeks	PCR90	MWF	VAC70	50	Unvaccinate	1.472	0.006	0.003	0.010	0.002	0.007	0.001	706.486	3.102	1.610	4.712	1.077	3.347	0.659	7065	31.020	16.100	47.120	10.770	33.470	6.590
150	alpha	1	60x8 weeks	PCR90	MWF	VAC70	50	Unvaccinate	1.460	0.006	0.008	0.014	0.003	0.010	0.001	700.706	3.066	3.678	6.744	1.578	4.745	0.670	7007	30.660	36.780	67.440	15.780	47.450	6.700
150	alpha	1	60x8 weeks	PCR90	MWF	VAC70	50	Unvaccinate	1.460	0.006	0.008	0.014	0.003	0.010	0.001	700.706	3.066	3.678	6.744	1.578	4.745	0.670	7007	30.660	36.780	67.440	15.780	47.450	6.700
150	alpha	1	60x8 weeks	PCR90	MWF	VAC70	50	Unvaccinate	1.460	0.006	0.008	0.014	0.003	0.010	0.001	700.706	3.066	3.678	6.744	1.578	4.745	0.670	7007	30.660	36.780	67.440	15.780	47.450	6.700
150	alpha	1.2	60x8 weeks	PCR90	MWF	VAC70	50	Unvaccinate	1.453	0.007	0.010	0.017	0.004	0.012	0.001	697.448	3.158	4.900	8.058	1.880	5.611	0.677	6974	31.580	49.000	80.580	18.800	56.110	6.770
150	alpha	1.2	60x8 weeks	PCR90	MWF	VAC70	50	Unvaccinate	1.453	0.007	0.010	0.017	0.004	0.012	0.001	697.448	3.158	4.900	8.058	1.880	5.611	0.677	6974	31.580	49.000	80.580	18.800	56.110	6.770
150	alpha	1.2	60x8 weeks	PCR90	MWF	VAC70	50	Unvaccinate	1.453	0.007	0.010	0.017	0.004	0.012	0.001	697.448	3.158	4.900	8.058	1.880	5.611	0.677	6974	31.580	49.000	80.580	18.800	56.110	6.770
150	delta	0.6	60x8 weeks	PCR90	MWF	VAC70	50	Unvaccinate	1.438	0.007	0.018	0.024	0.007	0.015	0.001	690.128	3.198	8.408	11.606	3.569	7.031	0.646	6901	31.980	84.080	116.060	35.690	70.310	6.460
150	delta	0.6	60x8 weeks	PCR90	MWF	VAC70	50	Unvaccinate	1.438	0.007	0.018	0.024	0.007	0.015	0.001	690.128	3.198	8.408	11.606	3.569	7.031	0.646	6901	31.980	84.080	116.060	35.690	70.310	6.460
150	delta	0.6	60x8 weeks	PCR90	MWF	VAC70	50	Unvaccinate	1.438	0.007	0.018	0.024	0.007	0.015	0.001	690.128	3.198	8.408	11.606	3.569	7.031	0.646	6901	31.980	84.080	116.060	35.690	70.310	6.460
150	delta	1	60x8 weeks	PCR90	MWF	VAC70	50	Unvaccinate	1.349	0.005	0.056	0.062	0.022	0.034	0.001	647.643	2.531	27.060	29.591	10.387	16.426	0.505	6476	25.310	270.600	295.910	103.870	164.260	5.050
150	delta	1	60x8 weeks	PCR90	MWF	VAC70	50	Unvaccinate	1.349	0.005	0.056	0.062	0.022	0.034	0.001	647.643	2.531	27.060	29.591	10.387	16.426	0.505	6476	25.310	270.600	295.910	103.870	164.260	5.050
150	delta	1	60x8 weeks	PCR90	MWF	VAC70	50	Unvaccinate	1.349	0.005	0.056	0.062	0.022	0.034	0.001	647.643	2.531	27.060	29.591	10.387	16.426	0.505	6476	25.310	270.600	295.910	103.870	164.260	5.050
150	delta	1.2	60x8 weeks	PCR90	MWF	VAC70	50	Unvaccinate	1.305	0.005	0.078	0.083	0.030	0.044	0.001	626.416	2.350	37.348	39.698	14.555	20.905	0.428	6264	23.500	373.480	396.980	145.550	209.050	4.280
150	delta	1.2	60x8 weeks	PCR90	MWF	VAC70	50	Unvaccinate	1.305	0.005	0.078	0.083	0.030	0.044	0.001	626.416	2.350	37.348	39.698	14.555	20.905	0.428	6264	23.500	373.480	396.980	145.550	209.050	4.280
150	delta	1.2	60x8 weeks	PCR90	MWF	VAC70	50	Unvaccinate	1.305	0.005	0.078	0.083	0.030	0.044	0.001	626.416	2.350	37.348	39.698	14.555	20.905	0.428	6264	23.500	373.480	396.980	145.550	209.050	4.280
150	original	0.6	60x8 weeks	PCR90	MWF	VAC70	75	Unvaccinate	0.740	0.005	0.000	0.006	0.002	0.002	0.001	355.260	2.466	0.184	2.650	0.751	1.180	0.344	3553	24.660	1.840	26.500	7.510	11.800	3.440
150	original	0.6	60x8 weeks	PCR90	MWF	VAC70	75	Unvaccinate	0.740	0.005	0.000	0.006	0.002	0.002	0.001	355.260	2.466	0.184	2.650	0.751	1.180	0.344	3553	24.660	1.840	26.500	7.510	11.800	3.440
150	original	0.6	60x8 weeks	PCR90	MWF	VAC70	75	Unvaccinate	0.740	0.005	0.000	0.006	0.002	0.002	0.001	355.260	2.466	0.184	2.650	0.751	1.180	0.344	3553	24.660	1.840	26.500	7.510	11.800	3.440
150	original	1	60x8 weeks	PCR90	MWF	VAC70	75	Unvaccinate	0.740	0.005	0.001	0.006	0.002	0.002	0.001	355.154	2.400	0.300	2.700	0.818	1.196	0.338	3552	24.000	3.000	27.000	8.180	11.960	3.380
150	original	1	60x8 weeks	PCR90	MWF	VAC70	75	Unvaccinate	0.740	0.005	0.001	0.006	0.002	0.002	0.001	355.154	2.400	0.300	2.700	0.818	1.196	0.338	3552	24.000	3.000	27.000	8.180	11.960	3.380
150	original	1	60x8 weeks	PCR90	MWF	VAC70	75	Unvaccinate	0.740	0.005	0.001	0.006	0.002	0.002	0.001	355.154	2.400	0.300	2.700	0.818	1.196	0.338	3552	24.000	3.000	27.000	8.180	11.960	3.380
150	original	1.2	60x8 weeks	PCR90	MWF	VAC70	75	Unvaccinate	0.739	0.005	0.001	0.006	0.002	0.003	0.001	354.909	2.383	0.406	2.789	0.816	1.288	0.342	3549	23.830	4.060	27.890	8.160	12.880	3.420
150	original	1.2	60x8 weeks	PCR90	MWF	VAC70	75	Unvaccinate	0.739	0.005	0.001	0.006	0.002	0.003	0.001	354.909	2.383	0.406	2.789	0.816	1.288	0.342	3549	23.830	4.060	27.890	8.160	12.880	3.420
150	original	1.2	60x8 weeks	PCR90	MWF	VAC70	75	Unvaccinate	0.739	0.005	0.001	0.006	0.002	0.003	0.001	354.909	2.383	0.406	2.789	0.816	1.288	0.342	3549	23.830	4.060	27.890	8.160	12.880	3.420
150	alpha	0.6	60x8 weeks	PCR90	MWF	VAC70	75	Unvaccinate	0.737	0.005	0.002	0.007	0.002	0.003	0.001	353.878	2.413	0.864	3.277	1.165	1.518	0.370	3539	24.130	8.640	32.770	11.650	15.180	3.700
150	alpha	0.6	60x8 weeks	PCR90	MWF	VAC70	75	Unvaccinate	0.737	0.005	0.002	0.007	0.002	0.003	0.001	353.878	2.413	0.864	3.277	1.165	1.518	0.370	3539	24.130	8.640	32.770	11.650	15.180	3.700
150	alpha	0.6	60x8 weeks	PCR90	MWF	VAC70	75	Unvaccinate	0.737	0.005	0.002	0.007	0.002	0.003	0.001	353.878	2.413	0.864	3.277	1.165	1.518	0.370	3539	24.130	8.640	32.770	11.650	15.180	3.700
150	alpha	1	60x8 weeks	PCR90	MWF	VAC70	75	Unvaccinate	0.734	0.005	0.004	0.009	0.003	0.004	0.001	352.363	2.427	1.726	4.153	1.466	1.899	0.339	3524	24.270	17.260	41.530	14.660	18.990	3.390
150	alpha	1	60x8 weeks	PCR90	MWF	VAC70	75	Unvaccinate	0.734	0.005	0.004	0.009	0.003	0.004	0.001	352.363	2.427	1.726	4.153	1.466	1.899	0.339	3524	24.270	17.260	41.530	14.660	18.990	3.390
150	alpha	1	60x8 weeks	PCR90	MWF	VAC70	75	Unvaccinate	0.734	0.005	0.004	0.009	0.003	0.004	0.001	352.363	2.427	1.726	4.153	1.466	1.899	0.339	3524	24.270	17.260	41.530	14.660	18.990	3.390
150	alpha	1.2	60x8 weeks	PCR90	MWF	VAC70	75	Unvaccinate	0.732	0.005	0.005	0.010	0.004	0.004	0.001	351.462	2.377	2.277	4.654	1.680	2.103	0.340	3515	23.770	22.770	46.540	16.800	21.030	3.400
150	alpha	1.2	60x8 weeks	PCR90	MWF	VAC70	75	Unvaccinate	0.732	0.005	0.005	0.010	0.004	0.004	0.001	351.462	2.377	2.277	4.654	1.680	2.103	0.340	3515	23.770	22.770	46.540	16.800	21.030	3.400
150	alpha	1.2	60x8 weeks	PCR90	MWF	VAC70	75	Unvaccinate	0.732	0.005	0.005	0.010	0.004	0.004	0.001	351.462	2.377	2.277	4.654	1.680	2.103	0.340	3515	23.770	22.770	46.540	16.800	21.030	3.400
150	delta	0.6	60x8 weeks	PCR90	MWF	VAC70	75	Unvaccinate	0.728	0.006	0.008	0.014	0.006	0.005	0.001	349.444	2.662	3.920	6.582	2.756	2.467	0.341	3494	26.620	39.200	65.820	27.560	24.670	3.410
150	delta	0.6	60x8 weeks	PCR90	MWF	VAC70	75	Unvaccinate	0.728	0.006	0.008	0.014	0.006	0.005	0.001	349.444	2.662	3.920	6.582	2.756	2.467	0.341	3494	26.620	39.200	65.820	27.560	24.670	3.410
150	delta	0.6	60x8 weeks	PCR90	MWF	VAC70	75	Unvaccinate	0.728	0.006	0.008	0.014	0.006	0.005	0.001	349.444	2.662	3.920	6.582	2.756	2.467	0.341	3494	26.620	39.200	65.820	27.560	24.670	3.410
150	delta	1	60x8 weeks	PCR90	MWF	VAC70	75	Unvaccinate	0.704	0.005	0.026	0.031	0.014	0.011	0.001	337.964	2.464	12.616	15.080	6.719	5.098	0.266	3380	24.640	126.160	150.800	67.190	50.980	2.660
150	delta	1	60x8 weeks	PCR90	MWF	VAC70	75	Unvaccinate	0.704	0.005	0.026	0.031	0.014	0.011	0.001	337.964	2.464	12.616	15.080	6.719	5.098	0.266	3380	24.640	126.160	150.800	67.190	50.980	2.660
150	delta	1	60x8 weeks	PCR90	MWF	VAC70	75	Unvaccinate	0.704	0.005	0.026	0.031	0.014	0.011	0.001	337.964	2.464	12.616	15.080	6.719	5.098	0.266	3380	24.640	126.160	150.800	67.190	50.980	2.660
150	delta	1.2	60x8 weeks	PCR90	MWF	VAC70	75	Unvaccinate	0.685	0.005	0.042	0.047	0.022	0.015	0.001	328.632	2.271	20.390	22.661	10.554	7.094	0.276	3286	22.710	203.900	226.610	105.540	70.940	2.760
150	delta	1.2	60x8 weeks	PCR90	MWF	VAC70	75	Unvaccinate	0.685	0.005	0.042	0.047	0.022	0.015	0.001	328.632	2.271	20.390	22.661	10.554	7.094	0.276	3286	22.710	203.900	226.610	105.540	70.940	2.760
150	delta	1.2	60x8 weeks	PCR90	MWF	VAC70	75	Unvaccinate	0.685	0.005	0.042	0.047	0.022	0.015	0.001	328.632	2.271	20.390	22.661	10.554	7.094	0.276	3286	22.710	203.900	226.610	105.540	70.940	2.760
150	original	0.6	60x8 weeks	PCR90	MWF	VAC70	100	Unvaccinate	0.000	0.003	0.000	0.003	0.002	0.000	0.000	0.000	1.621	0.013	1.634	0.792	0.000	0.000	0	16.210	0.130	16.340	7.920	0.000	0.000
150	original	0.6	60x8 weeks	PCR90	MWF	VAC70	100	Unvaccinate	0.000	0.003	0.000	0.003	0.002	0.000	0.000	0.000	1.621	0.013	1.634	0.792	0.000	0.000	0	16.210	0.130	16.340	7.920	0.000	0.000
150	original	0.6	60x8 weeks	PCR90	MWF	VAC70	100	Unvaccinate	0.000	0.003	0.000	0.003	0.002	0.000	0.000	0.000	1.621	0.013	1.634	0.792	0.000	0.000	0	16.210	0.130	16.340	7.920	0.000	0.000
150	original	1	60x8 weeks	PCR90	MWF	VAC70	100	Unvaccinate	0.000	0.003	0.000	0.003	0.002	0.000	0.000	0.000	1.588	0.025	1.613	0.808	0.000	0.000	0	15.880	0.250	16.130	8.080	0.000	0.000
150	original	1	60x8 weeks	PCR90	MWF	VAC70	100	Unvaccinate	0.000	0.003	0.000	0.003	0.002	0.000	0.000	0.000	1.588	0.025	1.613	0.808	0.000	0.000	0	15.880	0.250	16.130	8.080	0.000	0.000
150	original	1	60x8 weeks	PCR90	MWF	VAC70	100	Unvaccinate	0.000	0.003	0.000	0.003	0.002	0.000	0.000	0.000	1.588	0.025	1.613	0.808	0.000	0.000	0	15.880	0.250	16.130	8.080	0.000	0.000
150	original	1.2	60x8 weeks	PCR90	MWF	VAC70	100	Unvaccinate	0.000	0.003	0.000	0.003	0.002	0.000	0.000	0.000	1.603	0.043	1.646	0.802	0.000	0.000	0	16.030	0.430	16.460	8.020	0.000	0.000
150	original	1.2	60x8 weeks	PCR90	MWF	VAC70	100	Unvaccinate	0.000	0.003	0.000	0.003	0.002	0.000	0.000	0.000	1.603	0.043	1.646	0.802	0.000	0.000	0	16.030	0.430	16.460	8.020	0.000	0.000
150	original	1.2	60x8 weeks	PCR90	MWF	VAC70	100	Unvaccinate	0.000	0.003	0.000	0.003	0.002	0.000	0.000	0.000	1.603	0.043	1.646	0.802	0.000	0.000	0	16.030	0.430	16.460	8.020	0.000	0.000

150	alpha	0.6	60x8 weeks	PCR90	MWF	VAC70	100	Unvaccinate	0.000	0.003	0.001	0.004	0.002	0.000	0.000	0.000	1.521	0.314	1.835	1.096	0.000	0.000	0	15.210	3.140	18.350	10.960	0.000	0.000
150	alpha	0.6	60x8 weeks	PCR90	MWF	VAC70	100	Unvaccinate	0.000	0.003	0.001	0.004	0.002	0.000	0.000	0.000	1.521	0.314	1.835	1.096	0.000	0.000	0	15.210	3.140	18.350	10.960	0.000	0.000
150	alpha	0.6	60x8 weeks	PCR90	MWF	VAC70	100	Unvaccinate	0.000	0.003	0.001	0.004	0.002	0.000	0.000	0.000	1.521	0.314	1.835	1.096	0.000	0.000	0	15.210	3.140	18.350	10.960	0.000	0.000
150	alpha	1	60x8 weeks	PCR90	MWF	VAC70	100	Unvaccinate	0.000	0.003	0.001	0.005	0.003	0.000	0.000	0.000	1.596	0.627	2.223	1.299	0.000	0.000	0	15.960	6.270	22.230	12.990	0.000	0.000
150	alpha	1	60x8 weeks	PCR90	MWF	VAC70	100	Unvaccinate	0.000	0.003	0.001	0.005	0.003	0.000	0.000	0.000	1.596	0.627	2.223	1.299	0.000	0.000	0	15.960	6.270	22.230	12.990	0.000	0.000
150	alpha	1	60x8 weeks	PCR90	MWF	VAC70	100	Unvaccinate	0.000	0.003	0.001	0.005	0.003	0.000	0.000	0.000	1.596	0.627	2.223	1.299	0.000	0.000	0	15.960	6.270	22.230	12.990	0.000	0.000
150	alpha	1.2	60x8 weeks	PCR90	MWF	VAC70	100	Unvaccinate	0.000	0.003	0.002	0.005	0.003	0.000	0.000	0.000	1.612	0.882	2.494	1.448	0.000	0.000	0	16.120	8.820	24.940	14.480	0.000	0.000
150	alpha	1.2	60x8 weeks	PCR90	MWF	VAC70	100	Unvaccinate	0.000	0.003	0.002	0.005	0.003	0.000	0.000	0.000	1.612	0.882	2.494	1.448	0.000	0.000	0	16.120	8.820	24.940	14.480	0.000	0.000
150	alpha	1.2	60x8 weeks	PCR90	MWF	VAC70	100	Unvaccinate	0.000	0.003	0.002	0.005	0.003	0.000	0.000	0.000	1.612	0.882	2.494	1.448	0.000	0.000	0	16.120	8.820	24.940	14.480	0.000	0.000
150	delta	0.6	60x8 weeks	PCR90	MWF	VAC70	100	Unvaccinate	0.000	0.004	0.003	0.008	0.005	0.000	0.000	0.000	2.100	1.520	3.620	2.233	0.000	0.000	0	21.000	15.200	36.200	22.330	0.000	0.000
150	delta	0.6	60x8 weeks	PCR90	MWF	VAC70	100	Unvaccinate	0.000	0.004	0.003	0.008	0.005	0.000	0.000	0.000	2.100	1.520	3.620	2.233	0.000	0.000	0	21.000	15.200	36.200	22.330	0.000	0.000
150	delta	0.6	60x8 weeks	PCR90	MWF	VAC70	100	Unvaccinate	0.000	0.004	0.003	0.008	0.005	0.000	0.000	0.000	2.100	1.520	3.620	2.233	0.000	0.000	0	21.000	15.200	36.200	22.330	0.000	0.000
150	delta	1	60x8 weeks	PCR90	MWF	VAC70	100	Unvaccinate	0.000	0.004	0.010	0.014	0.009	0.000	0.000	0.000	2.010	4.651	6.661	4.095	0.000	0.000	0	20.100	46.510	66.610	40.950	0.000	0.000
150	delta	1	60x8 weeks	PCR90	MWF	VAC70	100	Unvaccinate	0.000	0.004	0.010	0.014	0.009	0.000	0.000	0.000	2.010	4.651	6.661	4.095	0.000	0.000	0	20.100	46.510	66.610	40.950	0.000	0.000
150	delta	1	60x8 weeks	PCR90	MWF	VAC70	100	Unvaccinate	0.000	0.004	0.010	0.014	0.009	0.000	0.000	0.000	2.010	4.651	6.661	4.095	0.000	0.000	0	20.100	46.510	66.610	40.950	0.000	0.000
150	delta	1.2	60x8 weeks	PCR90	MWF	VAC70	100	Unvaccinate	0.000	0.004	0.016	0.020	0.012	0.000	0.000	0.000	1.926	7.474	9.400	5.797	0.000	0.000	0	19.260	74.740	94.000	57.970	0.000	0.000
150	delta	1.2	60x8 weeks	PCR90	MWF	VAC70	100	Unvaccinate	0.000	0.004	0.016	0.020	0.012	0.000	0.000	0.000	1.926	7.474	9.400	5.797	0.000	0.000	0	19.260	74.740	94.000	57.970	0.000	0.000
150	delta	1.2	60x8 weeks	PCR90	MWF	VAC70	100	Unvaccinate	0.000	0.004	0.016	0.020	0.012	0.000	0.000	0.000	1.926	7.474	9.400	5.797	0.000	0.000	0	19.260	74.740	94.000	57.970	0.000	0.000
150	original	0.6	60x8 weeks	PCR90	MWF	VAC50	25	Unvaccinate	2.215	0.009	0.002	0.011	0.002	0.009	0.002	1063.349	4.199	1.057	5.256	0.831	4.219	1.001	10633	41.990	10.570	52.560	8.310	42.190	10.010
150	original	0.6	60x8 weeks	PCR90	MWF	VAC50	25	Unvaccinate	2.215	0.009	0.002	0.011	0.002	0.009	0.002	1063.349	4.199	1.057	5.256	0.831	4.219	1.001	10633	41.990	10.570	52.560	8.310	42.190	10.010
150	original	0.6	60x8 weeks	PCR90	MWF	VAC50	25	Unvaccinate	2.215	0.009	0.002	0.011	0.002	0.009	0.002	1063.349	4.199	1.057	5.256	0.831	4.219	1.001	10633	41.990	10.570	52.560	8.310	42.190	10.010
150	original	1	60x8 weeks	PCR90	MWF	VAC50	25	Unvaccinate	2.209	0.009	0.004	0.013	0.002	0.010	0.002	1060.349	4.133	2.122	6.255	0.975	5.016	1.014	10603	41.330	21.220	62.550	9.750	50.160	10.140
150	original	1	60x8 weeks	PCR90	MWF	VAC50	25	Unvaccinate	2.209	0.009	0.004	0.013	0.002	0.010	0.002	1060.349	4.133	2.122	6.255	0.975	5.016	1.014	10603	41.330	21.220	62.550	9.750	50.160	10.140
150	original	1	60x8 weeks	PCR90	MWF	VAC50	25	Unvaccinate	2.209	0.009	0.004	0.013	0.002	0.010	0.002	1060.349	4.133	2.122	6.255	0.975	5.016	1.014	10603	41.330	21.220	62.550	9.750	50.160	10.140
150	original	1.2	60x8 weeks	PCR90	MWF	VAC50	25	Unvaccinate	2.203	0.009	0.006	0.015	0.002	0.012	0.002	1057.572	4.199	2.842	7.041	1.191	5.589	0.989	10576	41.990	28.420	70.410	11.910	55.890	9.890
150	original	1.2	60x8 weeks	PCR90	MWF	VAC50	25	Unvaccinate	2.203	0.009	0.006	0.015	0.002	0.012	0.002	1057.572	4.199	2.842	7.041	1.191	5.589	0.989	10576	41.990	28.420	70.410	11.910	55.890	9.890
150	original	1.2	60x8 weeks	PCR90	MWF	VAC50	25	Unvaccinate	2.203	0.009	0.006	0.015	0.002	0.012	0.002	1057.572	4.199	2.842	7.041	1.191	5.589	0.989	10576	41.990	28.420	70.410	11.910	55.890	9.890
150	alpha	0.6	60x8 weeks	PCR90	MWF	VAC50	25	Unvaccinate	2.201	0.009	0.007	0.015	0.003	0.012	0.002	1056.623	4.087	3.177	7.264	1.319	5.814	1.019	10566	40.870	31.770	72.640	13.190	58.140	10.190
150	alpha	0.6	60x8 weeks	PCR90	MWF	VAC50	25	Unvaccinate	2.201	0.009	0.007	0.015	0.003	0.012	0.002	1056.623	4.087	3.177	7.264	1.319	5.814	1.019	10566	40.870	31.770	72.640	13.190	58.140	10.190
150	alpha	0.6	60x8 weeks	PCR90	MWF	VAC50	25	Unvaccinate	2.201	0.009	0.007	0.015	0.003	0.012	0.002	1056.623	4.087	3.177	7.264	1.319	5.814	1.019	10566	40.870	31.770	72.640	13.190	58.140	10.190
150	alpha	1	60x8 weeks	PCR90	MWF	VAC50	25	Unvaccinate	2.165	0.008	0.018	0.026	0.005	0.021	0.002	1039.182	3.898	8.701	12.599	2.471	9.954	0.952	10392	38.980	87.010	125.990	24.710	99.540	9.520
150	alpha	1	60x8 weeks	PCR90	MWF	VAC50	25	Unvaccinate	2.165	0.008	0.018	0.026	0.005	0.021	0.002	1039.182	3.898	8.701	12.599	2.471	9.954	0.952	10392	38.980	87.010	125.990	24.710	99.540	9.520
150	alpha	1	60x8 weeks	PCR90	MWF	VAC50	25	Unvaccinate	2.165	0.008	0.018	0.026	0.005	0.021	0.002	1039.182	3.898	8.701	12.599	2.471	9.954	0.952	10392	38.980	87.010	125.990	24.710	99.540	9.520
150	alpha	1.2	60x8 weeks	PCR90	MWF	VAC50	25	Unvaccinate	2.130	0.008	0.029	0.037	0.007	0.029	0.002	1022.446	3.784	13.992	17.776	3.541	14.130	0.917	10224	37.840	139.920	177.760	35.410	141.300	9.170
150	alpha	1.2	60x8 weeks	PCR90	MWF	VAC50	25	Unvaccinate	2.130	0.008	0.029	0.037	0.007	0.029	0.002	1022.446	3.784	13.992	17.776	3.541	14.130	0.917	10224	37.840	139.920	177.760	35.410	141.300	9.170
150	alpha	1.2	60x8 weeks	PCR90	MWF	VAC50	25	Unvaccinate	2.130	0.008	0.029	0.037	0.007	0.029	0.002	1022.446	3.784	13.992	17.776	3.541	14.130	0.917	10224	37.840	139.920	177.760	35.410	141.300	9.170
150	delta	0.6	60x8 weeks	PCR90	MWF	VAC50	25	Unvaccinate	2.094	0.008	0.043	0.050	0.013	0.036	0.002	1005.017	3.696	20.429	24.125	6.368	17.237	0.882	10050	36.960	204.290	241.250	63.680	172.370	8.820
150	delta	0.6	60x8 weeks	PCR90	MWF	VAC50	25	Unvaccinate	2.094	0.008	0.043	0.050	0.013	0.036	0.002	1005.017	3.696	20.429	24.125	6.368	17.237	0.882	10050	36.960	204.290	241.250	63.680	172.370	8.820
150	delta	0.6	60x8 weeks	PCR90	MWF	VAC50	25	Unvaccinate	2.094	0.008	0.043	0.050	0.013	0.036	0.002	1005.017	3.696	20.429	24.125	6.368	17.237	0.882	10050	36.960	204.290	241.250	63.680	172.370	8.820
150	delta	1	60x8 weeks	PCR90	MWF	VAC50	25	Unvaccinate	1.917	0.005	0.100	0.105	0.030	0.074	0.001	919.966	2.492	48.003	50.495	14.522	35.356	0.557	9200	24.920	480.030	504.950	145.220	353.560	5.570
150	delta	1	60x8 weeks	PCR90	MWF	VAC50	25	Unvaccinate	1.917	0.005	0.100	0.105	0.030	0.074	0.001	919.966	2.492	48.003	50.495	14.522	35.356	0.557	9200	24.920	480.030	504.950	145.220	353.560	5.570
150	delta	1	60x8 weeks	PCR90	MWF	VAC50	25	Unvaccinate	1.917	0.005	0.100	0.105	0.030	0.074	0.001	919.966	2.492	48.003	50.495	14.522	35.356	0.557	9200	24.920	480.030	504.950	145.220	353.560	5.570
150	delta	1.2	60x8 weeks	PCR90	MWF	VAC50	25	Unvaccinate	1.877	0.005	0.111	0.115	0.034	0.080	0.001	900.745	2.177	53.067	55.244	16.277	38.637	0.496	9007	21.770	530.670	552.440	162.770	386.370	4.960
150	delta	1.2	60x8 weeks	PCR90	MWF	VAC50	25	Unvaccinate	1.877	0.005	0.111	0.115	0.034	0.080	0.001	900.745	2.177	53.067	55.244	16.277	38.637	0.496	9007	21.770	530.670	552.440	162.770	386.370	4.960
150	delta	1.2	60x8 weeks	PCR90	MWF	VAC50	25	Unvaccinate	1.877	0.005	0.111	0.115	0.034	0.080	0.001	900.745	2.177	53.067	55.244	16.277	38.637	0.496	9007	21.770	530.670	552.440	162.770	386.370	4.960
150	original	0.6	60x8 weeks	PCR90	MWF	VAC50	50	Unvaccinate	1.479	0.008	0.001	0.009	0.002	0.005	0.002	709.836	3.658	0.540	4.198	1.025	2.545	0.737	7098	36.580	5.400	41.980	10.250	25.450	7.370
150	original	0.6	60x8 weeks	PCR90	MWF	VAC50	50	Unvaccinate	1.479	0.008	0.001	0.009	0.002	0.005	0.002	709.836	3.658	0.540	4.198	1.025	2.545	0.737	7098	36.580	5.400	41.980	10.250	25.450	7.370
150	original	0.6	60x8 weeks	PCR90	MWF	VAC50	50	Unvaccinate	1.479	0.008	0.001	0.009	0.002	0.005	0.002	709.836	3.658	0.540	4.198	1.025	2.545	0.737	7098	36.580	5.400	41.980	10.250	25.450	7.370
150	original	1	60x8 weeks	PCR90	MWF	VAC50	50	Unvaccinate	1.475	0.008	0.002	0.010	0.002	0.006	0.001	708.231	3.627	1.184	4.811	1.120	2.956	0.668	7082	36.270	11.840	48.110	11.200	29.560	6.680
150	original	1	60x8 weeks	PCR90	MWF	VAC50	50	Unvaccinate	1.475	0.008	0.002	0.010	0.002	0.006	0.001	708.231	3.627	1.184	4.811	1.120	2.956	0.668	7082	36.270	11.840	48.110	11.200	29.560	6.680
150	original	1	60x8 weeks	PCR90	MWF	VAC50	50	Unvaccinate	1.475	0.008	0.002	0.010	0.002	0.006	0.001	708.231	3.627	1.184	4.811	1.120	2.956	0.668	7082	36.270	11.840	48.110	11.200	29.560	6.680
150	original	1.2	60x8 weeks	PCR90	MWF	VAC50	50	Unvaccinate	1.474	0.008	0.003	0.011	0.003	0.006	0.001	707.627	3.665	1.429	5.094	1.246	3.087	0.677	7076	36.650	14.290	50.940	12.460	30.870	6.770
150	original	1.2	60x8 weeks	PCR90	MWF	VAC50	50	Unvaccinate	1.474	0.008	0.003	0.011	0.003	0.006	0.001	707.627	3.665	1.429	5.094	1.246	3.087	0.677	7076	36.650	14.290	50.940	12.460	30.870	6.770
150	original	1.2	60x8 weeks	PCR90	MWF	VAC50	50	Unvaccinate	1.474	0.008	0.003	0.011	0.003	0.006	0.001	707.627	3.665	1.429	5.094	1.246	3.087	0.677	7076	36.650	14.290	50.940	12.460	30.870	6.770
150	alpha	0.6	60x8 weeks	PCR90	MWF	VAC50	50	Unvaccinate	1.469	0.007	0.005	0.012	0.004	0.008	0.001	705.166	3.594	2.362	5.956	1.691	3.678	0.704	7052	35.940	23.620	59.560	16.910	36.780	7.040
150	alpha	0.6	60x8 weeks	PCR90	MWF	VAC50	50	Unvaccinate	1.469	0.007	0.005	0.012	0.004	0.008	0.001	705.166	3.594	2.362	5.956	1.691	3.678	0.704	7052	35.940	23.620	59.560	16.910	36.780	7.040
150	alpha	0.6	60x8 weeks	PCR90	MWF	VAC50	50	Unvaccinate	1.469	0.007	0.005	0.012	0.004	0.008	0.001	705.166	3.594	2.362	5.956	1.691	3.678	0.704	7052	35.940	23.620	59.560	16.910	36.780	7.040
150	alpha	1	60x8 weeks	PCR90	MWF	VAC50	50	Unvaccinate	1.453	0.007	0.012	0.019	0.006	0.012	0.001	697.228	3.560	5.735	9.295	2.742	5.650	0.669	6972	35.600	57.350	92.950	27.420	56.500	6.690
150	alpha	1	60x8 weeks	PCR90	MWF	VAC50	50	Unvaccinate	1.453	0.007	0.012	0.019	0.006	0.012	0.001	697.228	3.560	5.735	9.295	2.742	5.650	0.669	6972	35.600	57.350	92.950	27.420	56.500	6.690
150	alpha	1	60x8 weeks	PCR90	MWF	VAC50	50	Unvaccinate	1.453	0.007	0.012	0.019	0.006	0.012	0.001	697.228	3.560	5.735	9.295	2.742	5.650	0.669	6972	35.600	57.350	92.950	27.420	56.500	6.690
150	alpha	1.2	60x8 weeks	PCR90	MWF	VAC50	50	Unvaccinate	1.443	0.007	0.017	0.024	0.007	0.014	0.001	692.747	3.409	8.127	11.536	3.419	6.851	0.609	6927	34.090	81.270	115.360	34.190	68.510	6.090
150	alpha	1.2	60x8 weeks	PCR90	MWF	VAC50	50	Unvaccinate	1.443	0.007	0.017	0.024	0.007	0.014	0.001	692.747	3.409	8.127	11.536	3.419	6.851	0.609	6927	34.090	81.270	115.360	34.190	68.510	6.090
150	alpha	1.2	60x8 weeks	PCR90	MWF	VAC50	50	Unvaccinate	1.443	0.007	0.017	0.024	0.007	0.014	0.001	692.747	3.409	8.127	11.536	3.419	6.851	0.609	6927	34.090	81.270	115.360	34.190	68.510	6.090
150	delta	0.6	60x8 weeks	PCR90	MWF	VAC50	50	Unvaccinate	1.419	0.008	0.029	0.037	0.014	0.019	0.001	681.137	3.634	14.092	17.726	6.614	8.965	0.612	6811	36.340	140.920	177.260	66.140	89.650	6.120
150	delta	0.6	60x8 weeks	PCR90	MWF	VAC50	50	Unvaccinate	1.419	0.008	0.029	0.037	0.014	0.019	0.001	681.137	3.634	14.092	17.726	6.614	8.965	0.612	6811	36.340	140.920	177.260	66.140	89.650	6.120
150	delta	0.6	60x8 weeks	PCR90	MWF	VAC50	50	Unvaccinate	1.419	0.008	0.029	0.037	0.014	0.019	0.001	681.137	3.634	14.092	17.726	6.614	8.965	0.612	6811	36.340	140.920	177.260	66.140	89.650	6.120
150	delta	1	60x8 weeks	PCR90	MWF	VAC50	50	Unvaccinate	1.297	0.006	0.088	0.093	0.038	0.045	0.001	622.420	2.661	42.114	44.775	18.152	21.507	0.419	6224	26.610	421.140	447.750	181.520	215.070	4.190
150	delta	1	60x8 weeks	PCR90	MWF	VAC50	50	Unvaccinate	1.297	0.006	0.088	0.093	0.038	0.045	0.001	622.420	2.661	42.114	44.775	18.152	21.507	0.419	6224	26.610	421.140	447.750	181.520	215.070	4.190
150	delta	1	60x8 weeks	PCR90	MWF	VAC50	50	Unvaccinate	1.297	0.006	0.088	0.093	0.038	0.045	0.001	622.420	2.661	42.114	44.775	18.152	21.507	0.419	6224	26.610	421.140	447.750	181.520	215.070	4.190
150	delta	1.2	60x8 weeks	PCR90	MWF	VAC50	50	Unvaccinate	1.271	0.005	0.100	0.105	0.043	0.050	0.001	609.996	2.243	48.220	50.463	20.722	24.143	0.338	6100	22.430	482.200	504.630	207.220	241.430	3.380
150	delta	1.2	60x8 weeks	PCR90	MWF	VAC50	50	Unvaccinate	1.271	0.005	0.100	0.105	0.043	0.050	0.001	609.996	2.243	48.220	50.463	20.722	24.143	0.338	6100	22.430	482.200	504.630	207.220	241.430	3.380

150	delta	1.2	60x8 weeks	PCR90	MWF	VAC50	50	Unvaccinate	1.271	0.005	0.100	0.105	0.043	0.050	0.001	609.996	2.243	48.220	50.463	20.722	24.143	0.338	6100	22.430	482.200	504.630	207.220	241.430	3.380
150	original	0.6	60x8 weeks	PCR90	MWF	VAC50	75	Unvaccinate	0.740	0.006	0.001	0.007	0.003	0.002	0.001	355.254	3.115	0.286	3.401	1.221	1.179	0.346	3553	31.150	2.860	34.010	12.210	11.790	3.460
150	original	0.6	60x8 weeks	PCR90	MWF	VAC50	75	Unvaccinate	0.740	0.006	0.001	0.007	0.003	0.002	0.001	355.254	3.115	0.286	3.401	1.221	1.179	0.346	3553	31.150	2.860	34.010	12.210	11.790	3.460
150	original	0.6	60x8 weeks	PCR90	MWF	VAC50	75	Unvaccinate	0.740	0.006	0.001	0.007	0.003	0.002	0.001	355.254	3.115	0.286	3.401	1.221	1.179	0.346	3553	31.150	2.860	34.010	12.210	11.790	3.460
150	original	1	60x8 weeks	PCR90	MWF	VAC50	75	Unvaccinate	0.739	0.007	0.001	0.008	0.003	0.003	0.001	354.709	3.144	0.465	3.609	1.310	1.343	0.369	3547	31.440	4.650	36.090	13.100	13.430	3.690
150	original	1	60x8 weeks	PCR90	MWF	VAC50	75	Unvaccinate	0.739	0.007	0.001	0.008	0.003	0.003	0.001	354.709	3.144	0.465	3.609	1.310	1.343	0.369	3547	31.440	4.650	36.090	13.100	13.430	3.690
150	original	1	60x8 weeks	PCR90	MWF	VAC50	75	Unvaccinate	0.739	0.007	0.001	0.008	0.003	0.003	0.001	354.709	3.144	0.465	3.609	1.310	1.343	0.369	3547	31.440	4.650	36.090	13.100	13.430	3.690
150	original	1.2	60x8 weeks	PCR90	MWF	VAC50	75	Unvaccinate	0.739	0.006	0.001	0.008	0.003	0.003	0.001	354.939	3.109	0.554	3.663	1.290	1.290	0.339	3549	31.090	5.540	36.630	12.900	12.900	3.390
150	original	1.2	60x8 weeks	PCR90	MWF	VAC50	75	Unvaccinate	0.739	0.006	0.001	0.008	0.003	0.003	0.001	354.939	3.109	0.554	3.663	1.290	1.290	0.339	3549	31.090	5.540	36.630	12.900	12.900	3.390
150	original	1.2	60x8 weeks	PCR90	MWF	VAC50	75	Unvaccinate	0.739	0.006	0.001	0.008	0.003	0.003	0.001	354.939	3.109	0.554	3.663	1.290	1.290	0.339	3549	31.090	5.540	36.630	12.900	12.900	3.390
150	alpha	0.6	60x8 weeks	PCR90	MWF	VAC50	75	Unvaccinate	0.735	0.007	0.003	0.010	0.004	0.004	0.001	353.008	3.137	1.495	4.632	1.938	1.726	0.332	3530	31.370	14.950	46.320	19.380	17.260	3.320
150	alpha	0.6	60x8 weeks	PCR90	MWF	VAC50	75	Unvaccinate	0.735	0.007	0.003	0.010	0.004	0.004	0.001	353.008	3.137	1.495	4.632	1.938	1.726	0.332	3530	31.370	14.950	46.320	19.380	17.260	3.320
150	alpha	0.6	60x8 weeks	PCR90	MWF	VAC50	75	Unvaccinate	0.735	0.007	0.003	0.010	0.004	0.004	0.001	353.008	3.137	1.495	4.632	1.938	1.726	0.332	3530	31.370	14.950	46.320	19.380	17.260	3.320
150	alpha	1	60x8 weeks	PCR90	MWF	VAC50	75	Unvaccinate	0.731	0.006	0.007	0.013	0.006	0.005	0.001	351.082	3.055	3.308	6.363	2.646	2.261	0.328	3511	30.550	33.080	63.630	26.460	22.610	3.280
150	alpha	1	60x8 weeks	PCR90	MWF	VAC50	75	Unvaccinate	0.731	0.006	0.007	0.013	0.006	0.005	0.001	351.082	3.055	3.308	6.363	2.646	2.261	0.328	3511	30.550	33.080	63.630	26.460	22.610	3.280
150	alpha	1	60x8 weeks	PCR90	MWF	VAC50	75	Unvaccinate	0.731	0.006	0.007	0.013	0.006	0.005	0.001	351.082	3.055	3.308	6.363	2.646	2.261	0.328	3511	30.550	33.080	63.630	26.460	22.610	3.280
150	alpha	1.2	60x8 weeks	PCR90	MWF	VAC50	75	Unvaccinate	0.727	0.006	0.010	0.016	0.007	0.006	0.001	348.966	2.926	4.740	7.666	3.328	2.724	0.338	3490	29.260	47.400	76.660	33.280	27.240	3.380
150	alpha	1.2	60x8 weeks	PCR90	MWF	VAC50	75	Unvaccinate	0.727	0.006	0.010	0.016	0.007	0.006	0.001	348.966	2.926	4.740	7.666	3.328	2.724	0.338	3490	29.260	47.400	76.660	33.280	27.240	3.380
150	alpha	1.2	60x8 weeks	PCR90	MWF	VAC50	75	Unvaccinate	0.727	0.006	0.010	0.016	0.007	0.006	0.001	348.966	2.926	4.740	7.666	3.328	2.724	0.338	3490	29.260	47.400	76.660	33.280	27.240	3.380
150	delta	0.6	60x8 weeks	PCR90	MWF	VAC50	75	Unvaccinate	0.716	0.007	0.021	0.028	0.014	0.008	0.001	343.691	3.482	10.055	13.537	6.585	3.805	0.301	3437	34.820	100.550	135.370	65.850	38.050	3.010
150	delta	0.6	60x8 weeks	PCR90	MWF	VAC50	75	Unvaccinate	0.716	0.007	0.021	0.028	0.014	0.008	0.001	343.691	3.482	10.055	13.537	6.585	3.805	0.301	3437	34.820	100.550	135.370	65.850	38.050	3.010
150	delta	0.6	60x8 weeks	PCR90	MWF	VAC50	75	Unvaccinate	0.716	0.007	0.021	0.028	0.014	0.008	0.001	343.691	3.482	10.055	13.537	6.585	3.805	0.301	3437	34.820	100.550	135.370	65.850	38.050	3.010
150	delta	1	60x8 weeks	PCR90	MWF	VAC50	75	Unvaccinate	0.668	0.006	0.067	0.072	0.038	0.018	0.000	320.608	2.738	32.032	34.770	18.114	8.827	0.212	3206	27.380	320.320	347.700	181.140	88.270	2.120
150	delta	1	60x8 weeks	PCR90	MWF	VAC50	75	Unvaccinate	0.668	0.006	0.067	0.072	0.038	0.018	0.000	320.608	2.738	32.032	34.770	18.114	8.827	0.212	3206	27.380	320.320	347.700	181.140	88.270	2.120
150	delta	1	60x8 weeks	PCR90	MWF	VAC50	75	Unvaccinate	0.668	0.006	0.067	0.072	0.038	0.018	0.000	320.608	2.738	32.032	34.770	18.114	8.827	0.212	3206	27.380	320.320	347.700	181.140	88.270	2.120
150	delta	1.2	60x8 weeks	PCR90	MWF	VAC50	75	Unvaccinate	0.649	0.005	0.087	0.092	0.048	0.023	0.000	311.700	2.357	41.601	43.958	23.224	10.804	0.233	3117	23.570	416.010	439.580	232.240	108.040	2.330
150	delta	1.2	60x8 weeks	PCR90	MWF	VAC50	75	Unvaccinate	0.649	0.005	0.087	0.092	0.048	0.023	0.000	311.700	2.357	41.601	43.958	23.224	10.804	0.233	3117	23.570	416.010	439.580	232.240	108.040	2.330
150	delta	1.2	60x8 weeks	PCR90	MWF	VAC50	75	Unvaccinate	0.649	0.005	0.087	0.092	0.048	0.023	0.000	311.700	2.357	41.601	43.958	23.224	10.804	0.233	3117	23.570	416.010	439.580	232.240	108.040	2.330
150	original	0.6	60x8 weeks	PCR90	MWF	VAC50	100	Unvaccinate	0.000	0.005	0.000	0.005	0.003	0.000	0.000	0.000	2.588	0.033	2.621	1.367	0.000	0.000	0	25.880	0.330	26.210	13.670	0.000	0.000
150	original	0.6	60x8 weeks	PCR90	MWF	VAC50	100	Unvaccinate	0.000	0.005	0.000	0.005	0.003	0.000	0.000	0.000	2.588	0.033	2.621	1.367	0.000	0.000	0	25.880	0.330	26.210	13.670	0.000	0.000
150	original	0.6	60x8 weeks	PCR90	MWF	VAC50	100	Unvaccinate	0.000	0.005	0.000	0.005	0.003	0.000	0.000	0.000	2.588	0.033	2.621	1.367	0.000	0.000	0	25.880	0.330	26.210	13.670	0.000	0.000
150	original	1	60x8 weeks	PCR90	MWF	VAC50	100	Unvaccinate	0.000	0.005	0.000	0.005	0.003	0.000	0.000	0.000	2.549	0.048	2.597	1.384	0.000	0.000	0	25.490	0.480	25.970	13.840	0.000	0.000
150	original	1	60x8 weeks	PCR90	MWF	VAC50	100	Unvaccinate	0.000	0.005	0.000	0.005	0.003	0.000	0.000	0.000	2.549	0.048	2.597	1.384	0.000	0.000	0	25.490	0.480	25.970	13.840	0.000	0.000
150	original	1	60x8 weeks	PCR90	MWF	VAC50	100	Unvaccinate	0.000	0.005	0.000	0.005	0.003	0.000	0.000	0.000	2.549	0.048	2.597	1.384	0.000	0.000	0	25.490	0.480	25.970	13.840	0.000	0.000
150	original	1.2	60x8 weeks	PCR90	MWF	VAC50	100	Unvaccinate	0.000	0.005	0.000	0.006	0.003	0.000	0.000	0.000	2.615	0.069	2.684	1.418	0.000	0.000	0	26.150	0.690	26.840	14.180	0.000	0.000
150	original	1.2	60x8 weeks	PCR90	MWF	VAC50	100	Unvaccinate	0.000	0.005	0.000	0.006	0.003	0.000	0.000	0.000	2.615	0.069	2.684	1.418	0.000	0.000	0	26.150	0.690	26.840	14.180	0.000	0.000
150	original	1.2	60x8 weeks	PCR90	MWF	VAC50	100	Unvaccinate	0.000	0.005	0.000	0.006	0.003	0.000	0.000	0.000	2.615	0.069	2.684	1.418	0.000	0.000	0	26.150	0.690	26.840	14.180	0.000	0.000
150	alpha	0.6	60x8 weeks	PCR90	MWF	VAC50	100	Unvaccinate	0.000	0.005	0.002	0.007	0.004	0.000	0.000	0.000	2.492	0.909	3.401	2.074	0.000	0.000	0	24.920	9.090	34.010	20.740	0.000	0.000
150	alpha	0.6	60x8 weeks	PCR90	MWF	VAC50	100	Unvaccinate	0.000	0.005	0.002	0.007	0.004	0.000	0.000	0.000	2.492	0.909	3.401	2.074	0.000	0.000	0	24.920	9.090	34.010	20.740	0.000	0.000
150	alpha	0.6	60x8 weeks	PCR90	MWF	VAC50	100	Unvaccinate	0.000	0.005	0.002	0.007	0.004	0.000	0.000	0.000	2.492	0.909	3.401	2.074	0.000	0.000	0	24.920	9.090	34.010	20.740	0.000	0.000
150	alpha	1	60x8 weeks	PCR90	MWF	VAC50	100	Unvaccinate	0.000	0.005	0.004	0.009	0.005	0.000	0.000	0.000	2.363	1.843	4.206	2.588	0.000	0.000	0	23.630	18.430	42.060	25.880	0.000	0.000
150	alpha	1	60x8 weeks	PCR90	MWF	VAC50	100	Unvaccinate	0.000	0.005	0.004	0.009	0.005	0.000	0.000	0.000	2.363	1.843	4.206	2.588	0.000	0.000	0	23.630	18.430	42.060	25.880	0.000	0.000
150	alpha	1	60x8 weeks	PCR90	MWF	VAC50	100	Unvaccinate	0.000	0.005	0.004	0.009	0.005	0.000	0.000	0.000	2.363	1.843	4.206	2.588	0.000	0.000	0	23.630	18.430	42.060	25.880	0.000	0.000
150	alpha	1.2	60x8 weeks	PCR90	MWF	VAC50	100	Unvaccinate	0.000	0.005	0.005	0.011	0.006	0.000	0.000	0.000	2.484	2.637	5.121	3.100	0.000	0.000	0	24.840	26.370	51.210	31.000	0.000	0.000
150	alpha	1.2	60x8 weeks	PCR90	MWF	VAC50	100	Unvaccinate	0.000	0.005	0.005	0.011	0.006	0.000	0.000	0.000	2.484	2.637	5.121	3.100	0.000	0.000	0	24.840	26.370	51.210	31.000	0.000	0.000
150	alpha	1.2	60x8 weeks	PCR90	MWF	VAC50	100	Unvaccinate	0.000	0.005	0.005	0.011	0.006	0.000	0.000	0.000	2.484	2.637	5.121	3.100	0.000	0.000	0	24.840	26.370	51.210	31.000	0.000	0.000
150	delta	0.6	60x8 weeks	PCR90	MWF	VAC50	100	Unvaccinate	0.000	0.007	0.014	0.021	0.013	0.000	0.000	0.000	3.182	6.882	10.064	6.450	0.000	0.000	0	31.820	68.820	100.640	64.500	0.000	0.000
150	delta	0.6	60x8 weeks	PCR90	MWF	VAC50	100	Unvaccinate	0.000	0.007	0.014	0.021	0.013	0.000	0.000	0.000	3.182	6.882	10.064	6.450	0.000	0.000	0	31.820	68.820	100.640	64.500	0.000	0.000
150	delta	0.6	60x8 weeks	PCR90	MWF	VAC50	100	Unvaccinate	0.000	0.007	0.014	0.021	0.013	0.000	0.000	0.000	3.182	6.882	10.064	6.450	0.000	0.000	0	31.820	68.820	100.640	64.500	0.000	0.000
150	delta	1	60x8 weeks	PCR90	MWF	VAC50	100	Unvaccinate	0.000	0.006	0.046	0.052	0.034	0.000	0.000	0.000	2.766	22.309	25.075	16.132	0.000	0.000	0	27.660	223.090	250.750	161.320	0.000	0.000
150	delta	1	60x8 weeks	PCR90	MWF	VAC50	100	Unvaccinate	0.000	0.006	0.046	0.052	0.034	0.000	0.000	0.000	2.766	22.309	25.075	16.132	0.000	0.000	0	27.660	223.090	250.750	161.320	0.000	0.000
150	delta	1	60x8 weeks	PCR90	MWF	VAC50	100	Unvaccinate	0.000	0.006	0.046	0.052	0.034	0.000	0.000	0.000	2.766	22.309	25.075	16.132	0.000	0.000	0	27.660	223.090	250.750	161.320	0.000	0.000
150	delta	1.2	60x8 weeks	PCR90	MWF	VAC50	100	Unvaccinate	0.000	0.005	0.070	0.076	0.049	0.000	0.000	0.000	2.463	33.805	36.268	23.622	0.000	0.000	0	24.630	338.050	362.680	236.220	0.000	0.000
150	delta	1.2	60x8 weeks	PCR90	MWF	VAC50	100	Unvaccinate	0.000	0.005	0.070	0.076	0.049	0.000	0.000	0.000	2.463	33.805	36.268	23.622	0.000	0.000	0	24.630	338.050	362.680	236.220	0.000	0.000
150	delta	1.2	60x8 weeks	PCR90	MWF	VAC50	100	Unvaccinate	0.000	0.005	0.070	0.076	0.049	0.000	0.000	0.000	2.463	33.805	36.268	23.622	0.000	0.000	0	24.630	338.050	362.680	236.220	0.000	0.000
150	original	0.6	60x8 weeks	PCR90	M	NOVAC	0	Unvaccinate	0.986	0.010	0.006	0.016	0.005	0.007	0.001	473.420	4.706	2.924	7.630	2.576	3.522	0.422	4734	47.060	29.240	76.300	25.760	35.220	4.220
150	original	0.6	60x8 weeks	PCR90	M	NOVAC	0	Unvaccinate	0.986	0.010	0.006	0.016	0.005	0.007	0.001	473.420	4.706	2.924	7.630	2.576	3.522	0.422	4734	47.060	29.240	76.300	25.760	35.220	4.220
150	original	0.6	60x8 weeks	PCR90	M	NOVAC	0	Unvaccinate	0.986	0.010	0.006	0.016	0.005	0.007	0.001	473.420	4.706	2.924	7.630	2.576	3.522	0.422	4734	47.060	29.240	76.300	25.760	35.220	4.220
150	original	1	60x8 weeks	PCR90	M	NOVAC	0	Unvaccinate	0.979	0.009	0.015	0.024	0.008	0.011	0.001	470.075	4.354	7.175	11.529	3.866	5.337	0.460	4701	43.540	71.750	115.290	38.660	53.370	4.600
150	original	1	60x8 weeks	PCR90	M	NOVAC	0	Unvaccinate	0.979	0.009	0.015	0.024	0.008	0.011	0.001	470.075	4.354	7.175	11.529	3.866	5.337	0.460	4701	43.540	71.750	115.290	38.660	53.370	4.600
150	original	1	60x8 weeks	PCR90	M	NOVAC	0	Unvaccinate	0.979	0.009	0.015	0.024	0.008	0.011	0.001	470.075	4.354	7.175	11.529	3.866	5.337	0.460	4701	43.540	71.750	115.290	38.660	53.370	4.600
150	original	1.2	60x8 weeks	PCR90	M	NOVAC	0	Unvaccinate	0.972	0.009	0.023	0.032	0.011	0.015	0.001	466.428	4.418	11.094	15.512	5.265	7.211	0.425	4664	44.180	110.940	155.120	52.650	72.110	4.250
150	original	1.2	60x8 weeks	PCR90	M	NOVAC	0	Unvaccinate	0.972	0.009	0.023	0.032	0.011	0.015	0.001	466.428	4.418	11.094	15.512	5.265	7.211	0.425	4664	44.180	110.940	155.120	52.650	72.110	4.250
150	original	1.2	60x8 weeks	PCR90	M	NOVAC	0	Unvaccinate	0.972	0.009	0.023	0.032	0.011	0.015	0.001	466.428	4.418	11.094	15.512	5.265	7.211	0.425	4664	44.180	110.940	155.120	52.650	72.110	4.250
150	alpha	0.6	60x8 weeks	PCR90	M	NOVAC	0	Unvaccinate	0.967	0.009	0.024	0.033	0.013	0.016	0.001	464.265	4.487	11.559	16.046	6.305	7.851	0.411	4643	44.870	115.590	160.460	63.050	78.510	4.110
150	alpha	0.6	60x8 weeks	PCR90	M	NOVAC	0	Unvaccinate	0.967	0.009	0.024	0.033	0.013	0.016	0.001	464.265	4.487	11.559	16.046	6.305	7.851	0.411	4643	44.870	115.590	160.460	63.050	78.510	4.110
150	alpha	0.6	60x8 weeks	PCR90	M	NOVAC	0	Unvaccinate	0.967	0.009	0.024	0.033	0.013	0.016	0.001	464.265	4.487	11.559	16.046	6.305	7.851	0.411	4643	44.870	115.590	160.460	63.050	78.510	4.110
150	alpha	1	60x8 weeks	PCR90	M	NOVAC	0	Unvaccinate	0.916	0.007	0.074	0.081	0.033	0.041	0.001	439.819	3.369	35.718	39.087	15.622	19.919	0.324	4398	33.690	357.180	390.870	156.220	199.190	3.240
150	alpha	1	60x8 weeks	PCR90	M	NOVAC	0	Unvaccinate	0.916	0.007	0.074	0.081	0.033	0.041	0.001	439.819	3.369	35.718	39.087	15.622	19.919	0.324	4398	33.690	357.180	390.870	156.220	199.190	3.240
150	alpha	1	60x8 weeks	PCR90	M	NOVAC	0	Unvaccinate	0.916	0.007	0.074	0.081	0.033	0.041	0.001	439.819	3.369	35.718	39.087	15.622	19.919	0.324	4398	33.690	357.180	390.870	156.220	199.190	3.240
150	alpha	1.2	60x8 weeks	PCR90	M	NOVAC	0	Unvaccinate	0.894	0.006	0.093	0.099	0.040	0.052	0.001	429.260	2.980	44.627	47.607	19.217	24.741	0.287	4293	29.800	446.270	476.070	192.170	247.410	2.870

150	alpha	1.2	60x8 weeks	PCR90	M	NOVAC	0	Unvaccinate	0.894	0.006	0.093	0.099	0.040	0.052	0.001	429.260	2.980	44.627	47.607	19.217	24.741	0.287	4293	29.800	446.270	476.070	192.170	247.410	2.870
150	alpha	1.2	60x8 weeks	PCR90	M	NOVAC	0	Unvaccinate	0.894	0.006	0.093	0.099	0.040	0.052	0.001	429.260	2.980	44.627	47.607	19.217	24.741	0.287	4293	29.800	446.270	476.070	192.170	247.410	2.870
150	delta	0.6	60x8 weeks	PCR90	M	NOVAC	0	Unvaccinate	0.880	0.006	0.102	0.108	0.048	0.053	0.001	422.599	2.672	49.038	51.710	23.163	25.284	0.300	4226	26.720	490.380	517.100	231.630	252.840	3.000
150	delta	0.6	60x8 weeks	PCR90	M	NOVAC	0	Unvaccinate	0.880	0.006	0.102	0.108	0.048	0.053	0.001	422.599	2.672	49.038	51.710	23.163	25.284	0.300	4226	26.720	490.380	517.100	231.630	252.840	3.000
150	delta	0.6	60x8 weeks	PCR90	M	NOVAC	0	Unvaccinate	0.880	0.006	0.102	0.108	0.048	0.053	0.001	422.599	2.672	49.038	51.710	23.163	25.284	0.300	4226	26.720	490.380	517.100	231.630	252.840	3.000
150	delta	1	60x8 weeks	PCR90	M	NOVAC	0	Unvaccinate	0.858	0.004	0.117	0.121	0.055	0.060	0.000	411.945	1.891	56.371	58.262	26.470	29.012	0.184	4119	18.910	563.710	582.620	264.700	290.120	1.840
150	delta	1	60x8 weeks	PCR90	M	NOVAC	0	Unvaccinate	0.858	0.004	0.117	0.121	0.055	0.060	0.000	411.945	1.891	56.371	58.262	26.470	29.012	0.184	4119	18.910	563.710	582.620	264.700	290.120	1.840
150	delta	1	60x8 weeks	PCR90	M	NOVAC	0	Unvaccinate	0.858	0.004	0.117	0.121	0.055	0.060	0.000	411.945	1.891	56.371	58.262	26.470	29.012	0.184	4119	18.910	563.710	582.620	264.700	290.120	1.840
150	delta	1.2	60x8 weeks	PCR90	M	NOVAC	0	Unvaccinate	0.855	0.004	0.119	0.123	0.056	0.061	0.000	410.186	1.766	57.096	58.862	27.117	29.295	0.179	4102	17.660	570.960	588.620	271.170	292.950	1.790
150	delta	1.2	60x8 weeks	PCR90	M	NOVAC	0	Unvaccinate	0.855	0.004	0.119	0.123	0.056	0.061	0.000	410.186	1.766	57.096	58.862	27.117	29.295	0.179	4102	17.660	570.960	588.620	271.170	292.950	1.790
150	delta	1.2	60x8 weeks	PCR90	M	NOVAC	0	Unvaccinate	0.855	0.004	0.119	0.123	0.056	0.061	0.000	410.186	1.766	57.096	58.862	27.117	29.295	0.179	4102	17.660	570.960	588.620	271.170	292.950	1.790
150	original	0.6	60x8 weeks	PCR90	M	VAC95	25	Unvaccinate	0.741	0.008	0.003	0.011	0.004	0.005	0.001	355.609	3.641	1.532	5.173	1.714	2.385	0.315	3556	36.410	15.320	51.730	17.140	23.850	3.150
150	original	0.6	60x8 weeks	PCR90	M	VAC95	25	Unvaccinate	0.741	0.008	0.003	0.011	0.004	0.005	0.001	355.609	3.641	1.532	5.173	1.714	2.385	0.315	3556	36.410	15.320	51.730	17.140	23.850	3.150
150	original	0.6	60x8 weeks	PCR90	M	VAC95	25	Unvaccinate	0.741	0.008	0.003	0.011	0.004	0.005	0.001	355.609	3.641	1.532	5.173	1.714	2.385	0.315	3556	36.410	15.320	51.730	17.140	23.850	3.150
150	original	1	60x8 weeks	PCR90	M	VAC95	25	Unvaccinate	0.737	0.008	0.007	0.015	0.005	0.007	0.001	353.973	3.629	3.360	6.989	2.379	3.184	0.327	3540	36.290	33.600	69.890	23.790	31.840	3.270
150	original	1	60x8 weeks	PCR90	M	VAC95	25	Unvaccinate	0.737	0.008	0.007	0.015	0.005	0.007	0.001	353.973	3.629	3.360	6.989	2.379	3.184	0.327	3540	36.290	33.600	69.890	23.790	31.840	3.270
150	original	1	60x8 weeks	PCR90	M	VAC95	25	Unvaccinate	0.737	0.008	0.007	0.015	0.005	0.007	0.001	353.973	3.629	3.360	6.989	2.379	3.184	0.327	3540	36.290	33.600	69.890	23.790	31.840	3.270
150	original	1.2	60x8 weeks	PCR90	M	VAC95	25	Unvaccinate	0.736	0.007	0.010	0.017	0.006	0.008	0.001	353.124	3.550	4.619	8.169	2.778	3.685	0.334	3531	35.500	46.190	81.690	27.780	36.850	3.340
150	original	1.2	60x8 weeks	PCR90	M	VAC95	25	Unvaccinate	0.736	0.007	0.010	0.017	0.006	0.008	0.001	353.124	3.550	4.619	8.169	2.778	3.685	0.334	3531	35.500	46.190	81.690	27.780	36.850	3.340
150	original	1.2	60x8 weeks	PCR90	M	VAC95	25	Unvaccinate	0.736	0.007	0.010	0.017	0.006	0.008	0.001	353.124	3.550	4.619	8.169	2.778	3.685	0.334	3531	35.500	46.190	81.690	27.780	36.850	3.340
150	alpha	0.6	60x8 weeks	PCR90	M	VAC95	25	Unvaccinate	0.734	0.007	0.010	0.018	0.007	0.008	0.001	352.254	3.599	4.969	8.568	3.256	4.054	0.324	3523	35.990	49.690	85.680	32.560	40.540	3.240
150	alpha	0.6	60x8 weeks	PCR90	M	VAC95	25	Unvaccinate	0.734	0.007	0.010	0.018	0.007	0.008	0.001	352.254	3.599	4.969	8.568	3.256	4.054	0.324	3523	35.990	49.690	85.680	32.560	40.540	3.240
150	alpha	0.6	60x8 weeks	PCR90	M	VAC95	25	Unvaccinate	0.734	0.007	0.010	0.018	0.007	0.008	0.001	352.254	3.599	4.969	8.568	3.256	4.054	0.324	3523	35.990	49.690	85.680	32.560	40.540	3.240
150	alpha	1	60x8 weeks	PCR90	M	VAC95	25	Unvaccinate	0.716	0.007	0.030	0.037	0.014	0.018	0.001	343.703	3.170	14.480	17.650	6.929	8.404	0.279	3437	31.700	144.800	176.500	69.290	84.040	2.790
150	alpha	1	60x8 weeks	PCR90	M	VAC95	25	Unvaccinate	0.716	0.007	0.030	0.037	0.014	0.018	0.001	343.703	3.170	14.480	17.650	6.929	8.404	0.279	3437	31.700	144.800	176.500	69.290	84.040	2.790
150	alpha	1	60x8 weeks	PCR90	M	VAC95	25	Unvaccinate	0.716	0.007	0.030	0.037	0.014	0.018	0.001	343.703	3.170	14.480	17.650	6.929	8.404	0.279	3437	31.700	144.800	176.500	69.290	84.040	2.790
150	alpha	1.2	60x8 weeks	PCR90	M	VAC95	25	Unvaccinate	0.703	0.006	0.043	0.049	0.020	0.024	0.001	337.323	2.892	20.794	23.686	9.449	11.319	0.264	3373	28.920	207.940	236.860	94.490	113.190	2.640
150	alpha	1.2	60x8 weeks	PCR90	M	VAC95	25	Unvaccinate	0.703	0.006	0.043	0.049	0.020	0.024	0.001	337.323	2.892	20.794	23.686	9.449	11.319	0.264	3373	28.920	207.940	236.860	94.490	113.190	2.640
150	alpha	1.2	60x8 weeks	PCR90	M	VAC95	25	Unvaccinate	0.703	0.006	0.043	0.049	0.020	0.024	0.001	337.323	2.892	20.794	23.686	9.449	11.319	0.264	3373	28.920	207.940	236.860	94.490	113.190	2.640
150	delta	0.6	60x8 weeks	PCR90	M	VAC95	25	Unvaccinate	0.692	0.006	0.054	0.060	0.027	0.026	0.001	332.204	2.889	25.817	28.706	12.850	12.573	0.267	3322	28.890	258.170	287.060	128.500	125.730	2.670
150	delta	0.6	60x8 weeks	PCR90	M	VAC95	25	Unvaccinate	0.692	0.006	0.054	0.060	0.027	0.026	0.001	332.204	2.889	25.817	28.706	12.850	12.573	0.267	3322	28.890	258.170	287.060	128.500	125.730	2.670
150	delta	0.6	60x8 weeks	PCR90	M	VAC95	25	Unvaccinate	0.692	0.006	0.054	0.060	0.027	0.026	0.001	332.204	2.889	25.817	28.706	12.850	12.573	0.267	3322	28.890	258.170	287.060	128.500	125.730	2.670
150	delta	1	60x8 weeks	PCR90	M	VAC95	25	Unvaccinate	0.655	0.004	0.093	0.097	0.045	0.041	0.000	314.419	1.953	44.505	46.458	21.735	19.684	0.171	3144	19.530	445.050	464.580	217.350	196.840	1.710
150	delta	1	60x8 weeks	PCR90	M	VAC95	25	Unvaccinate	0.655	0.004	0.093	0.097	0.045	0.041	0.000	314.419	1.953	44.505	46.458	21.735	19.684	0.171	3144	19.530	445.050	464.580	217.350	196.840	1.710
150	delta	1	60x8 weeks	PCR90	M	VAC95	25	Unvaccinate	0.655	0.004	0.093	0.097	0.045	0.041	0.000	314.419	1.953	44.505	46.458	21.735	19.684	0.171	3144	19.530	445.050	464.580	217.350	196.840	1.710
150	delta	1.2	60x8 weeks	PCR90	M	VAC95	25	Unvaccinate	0.650	0.004	0.099	0.103	0.048	0.043	0.000	312.152	1.781	47.495	49.276	22.957	20.700	0.154	3122	17.810	474.950	492.760	229.570	207.000	1.540
150	delta	1.2	60x8 weeks	PCR90	M	VAC95	25	Unvaccinate	0.650	0.004	0.099	0.103	0.048	0.043	0.000	312.152	1.781	47.495	49.276	22.957	20.700	0.154	3122	17.810	474.950	492.760	229.570	207.000	1.540
150	delta	1.2	60x8 weeks	PCR90	M	VAC95	25	Unvaccinate	0.650	0.004	0.099	0.103	0.048	0.043	0.000	312.152	1.781	47.495	49.276	22.957	20.700	0.154	3122	17.810	474.950	492.760	229.570	207.000	1.540
150	original	0.6	60x8 weeks	PCR90	M	VAC95	50	Unvaccinate	0.495	0.005	0.001	0.007	0.002	0.003	0.000	237.436	2.515	0.632	3.147	1.070	1.368	0.228	2374	25.150	6.320	31.470	10.700	13.680	2.280
150	original	0.6	60x8 weeks	PCR90	M	VAC95	50	Unvaccinate	0.495	0.005	0.001	0.007	0.002	0.003	0.000	237.436	2.515	0.632	3.147	1.070	1.368	0.228	2374	25.150	6.320	31.470	10.700	13.680	2.280
150	original	0.6	60x8 weeks	PCR90	M	VAC95	50	Unvaccinate	0.495	0.005	0.001	0.007	0.002	0.003	0.000	237.436	2.515	0.632	3.147	1.070	1.368	0.228	2374	25.150	6.320	31.470	10.700	13.680	2.280
150	original	1	60x8 weeks	PCR90	M	VAC95	50	Unvaccinate	0.494	0.005	0.002	0.008	0.003	0.003	0.000	236.978	2.605	1.129	3.734	1.237	1.649	0.208	2370	26.050	11.290	37.340	12.370	16.490	2.080
150	original	1	60x8 weeks	PCR90	M	VAC95	50	Unvaccinate	0.494	0.005	0.002	0.008	0.003	0.003	0.000	236.978	2.605	1.129	3.734	1.237	1.649	0.208	2370	26.050	11.290	37.340	12.370	16.490	2.080
150	original	1	60x8 weeks	PCR90	M	VAC95	50	Unvaccinate	0.494	0.005	0.002	0.008	0.003	0.003	0.000	236.978	2.605	1.129	3.734	1.237	1.649	0.208	2370	26.050	11.290	37.340	12.370	16.490	2.080
150	original	1.2	60x8 weeks	PCR90	M	VAC95	50	Unvaccinate	0.493	0.005	0.003	0.008	0.003	0.004	0.001	236.715	2.493	1.541	4.034	1.406	1.708	0.247	2367	24.930	15.410	40.340	14.060	17.080	2.470
150	original	1.2	60x8 weeks	PCR90	M	VAC95	50	Unvaccinate	0.493	0.005	0.003	0.008	0.003	0.004	0.001	236.715	2.493	1.541	4.034	1.406	1.708	0.247	2367	24.930	15.410	40.340	14.060	17.080	2.470
150	original	1.2	60x8 weeks	PCR90	M	VAC95	50	Unvaccinate	0.493	0.005	0.003	0.008	0.003	0.004	0.001	236.715	2.493	1.541	4.034	1.406	1.708	0.247	2367	24.930	15.410	40.340	14.060	17.080	2.470
150	alpha	0.6	60x8 weeks	PCR90	M	VAC95	50	Unvaccinate	0.492	0.005	0.004	0.009	0.003	0.004	0.000	236.217	2.603	1.730	4.333	1.665	1.952	0.237	2362	26.030	17.300	43.330	16.650	19.520	2.370
150	alpha	0.6	60x8 weeks	PCR90	M	VAC95	50	Unvaccinate	0.492	0.005	0.004	0.009	0.003	0.004	0.000	236.217	2.603	1.730	4.333	1.665	1.952	0.237	2362	26.030	17.300	43.330	16.650	19.520	2.370
150	alpha	0.6	60x8 weeks	PCR90	M	VAC95	50	Unvaccinate	0.492	0.005	0.004	0.009	0.003	0.004	0.000	236.217	2.603	1.730	4.333	1.665	1.952	0.237	2362	26.030	17.300	43.330	16.650	19.520	2.370
150	alpha	1	60x8 weeks	PCR90	M	VAC95	50	Unvaccinate	0.488	0.005	0.009	0.014	0.006	0.006	0.000	234.076	2.406	4.289	6.695	2.650	2.982	0.227	2341	24.060	42.890	66.950	26.500	29.820	2.270
150	alpha	1	60x8 weeks	PCR90	M	VAC95	50	Unvaccinate	0.488	0.005	0.009	0.014	0.006	0.006	0.000	234.076	2.406	4.289	6.695	2.650	2.982	0.227	2341	24.060	42.890	66.950	26.500	29.820	2.270
150	alpha	1	60x8 weeks	PCR90	M	VAC95	50	Unvaccinate	0.488	0.005	0.009	0.014	0.006	0.006	0.000	234.076	2.406	4.289	6.695	2.650	2.982	0.227	2341	24.060	42.890	66.950	26.500	29.820	2.270
150	alpha	1.2	60x8 weeks	PCR90	M	VAC95	50	Unvaccinate	0.484	0.005	0.013	0.018	0.007	0.008	0.000	232.424	2.475	6.212	8.687	3.509	3.843	0.202	2324	24.750	62.120	86.870	35.090	38.430	2.020
150	alpha	1.2	60x8 weeks	PCR90	M	VAC95	50	Unvaccinate	0.484	0.005	0.013	0.018	0.007	0.008	0.000	232.424	2.475	6.212	8.687	3.509	3.843	0.202	2324	24.750	62.120	86.870	35.090	38.430	2.020
150	alpha	1.2	60x8 weeks	PCR90	M	VAC95	50	Unvaccinate	0.484	0.005	0.013	0.018	0.007	0.008	0.000	232.424	2.475	6.212	8.687	3.509	3.843	0.202	2324	24.750	62.120	86.870	35.090	38.430	2.020
150	delta	0.6	60x8 weeks	PCR90	M	VAC95	50	Unvaccinate	0.481	0.005	0.017	0.022	0.010	0.009	0.000	231.098	2.484	7.974	10.458	4.677	4.174	0.202	2311	24.840	79.740	104.580	46.770	41.740	2.020
150	delta	0.6	60x8 weeks	PCR90	M	VAC95	50	Unvaccinate	0.481	0.005	0.017	0.022	0.010	0.009	0.000	231.098	2.484	7.974	10.458	4.677	4.174	0.202	2311	24.840	79.740	104.580	46.770	41.740	2.020
150	delta	0.6	60x8 weeks	PCR90	M	VAC95	50	Unvaccinate	0.481	0.005	0.017	0.022	0.010	0.009	0.000	231.098	2.484	7.974	10.458	4.677	4.174	0.202	2311	24.840	79.740	104.580	46.770	41.740	2.020
150	delta	1	60x8 weeks	PCR90	M	VAC95	50	Unvaccinate	0.460	0.004	0.046	0.051	0.023	0.018	0.000	220.831	2.044	22.238	24.282	11.168	8.773	0.163	2208	20.440	222.380	242.820	111.680	87.730	1.630
150	delta	1	60x8 weeks	PCR90	M	VAC95	50	Unvaccinate	0.460	0.004	0.046	0.051	0.023	0.018	0.000	220.831	2.044	22.238	24.282	11.168	8.773	0.163	2208	20.440	222.380	242.820	111.680	87.730	1.630
150	delta	1	60x8 weeks	PCR90	M	VAC95	50	Unvaccinate	0.460	0.004	0.046	0.051	0.023	0.018	0.000	220.831	2.044	22.238	24.282	11.168	8.773	0.163	2208	20.440	222.380	242.820	111.680	87.730	1.630
150	delta	1.2	60x8 weeks	PCR90	M	VAC95	50	Unvaccinate	0.451	0.004	0.058	0.062	0.029	0.022	0.000	216.634	1.846	28.070	29.916	13.913	10.534	0.164	2166	18.460	280.700	299.160	139.130	105.340	1.640
150	delta	1.2	60x8 weeks	PCR90	M	VAC95	50	Unvaccinate	0.451	0.004	0.058	0.062	0.029	0.022	0.000	216.634	1.846	28.070	29.916	13.913	10.534	0.164	2166	18.460	280.700	299.160	139.130	105.340	1.640
150	delta	1.2	60x8 weeks	PCR90	M	VAC95	50	Unvaccinate	0.451	0.004	0.058	0.062	0.029	0.022	0.000	216.634	1.846	28.070	29.916	13.913	10.534	0.164	2166	18.460	280.700	299.160	139.130	105.340	1.640
150	original	0.6	60x8 weeks	PCR90	M	VAC95	75	Unvaccinate	0.248	0.003	0.000	0.003	0.001	0.001	0.000	118.919	1.434	0.147	1.581	0.518	0.614	0.114	1189	14.340	1.470	15.810	5.180	6.140	1.140
150	original	0.6	60x8 weeks	PCR90	M	VAC95	75	Unvaccinate	0.248	0.003	0.000	0.003	0.001	0.001	0.000	118.919	1.434	0.147	1.581	0.518	0.614	0.114	1189	14.340	1.470	15.810	5.180	6.140	1.140
150	original	0.6	60x8 weeks	PCR90	M	VAC95	75	Unvaccinate	0.248	0.003	0.000	0.003	0.001	0.001	0.000	118.919	1.434	0.147	1.581	0.518	0.614	0.114	1189	14.340	1.470	15.810	5.180	6.140	1.140
150	original	1	60x8 weeks	PCR90	M	VAC95	75	Unvaccinate	0.248	0.003	0.001	0.004	0.001	0.001	0.000	118.821	1.429	0.266	1.695	0.647	0.568	0.114	1188	14.290	2.660	16.950	6.470	5.680	1.140
150	original	1	60x8 weeks	PCR90	M	VAC95	75	Unvaccinate	0.248	0.003	0.001	0.004	0.001	0.001	0.000	118.821	1.429	0.266	1.695	0.647	0.568	0.114	1188	14.290	2.660	16.950	6.470	5.680	1.140
150	original	1	60x8 weeks	PCR90	M	VAC95	75	Unvaccinate	0.248	0.003	0.001	0.004	0.001	0.001	0.000	118.821	1.429	0.266	1.695	0.647	0.568	0.114	1188	14.290	2.660	16.950	6.470	5.680	1.140

150	original	1.2	60x8 weeks	PCR90	M	VAC95	75	Unvaccinate	0.247	0.003	0.001	0.004	0.001	0.001	0.000	118.715	1.436	0.340	1.776	0.637	0.667	0.094	1187	14.360	3.400	17.760	6.370	6.670	0.940
150	original	1.2	60x8 weeks	PCR90	M	VAC95	75	Unvaccinate	0.247	0.003	0.001	0.004	0.001	0.001	0.000	118.715	1.436	0.340	1.776	0.637	0.667	0.094	1187	14.360	3.400	17.760	6.370	6.670	0.940
150	original	1.2	60x8 weeks	PCR90	M	VAC95	75	Unvaccinate	0.247	0.003	0.001	0.004	0.001	0.001	0.000	118.715	1.436	0.340	1.776	0.637	0.667	0.094	1187	14.360	3.400	17.760	6.370	6.670	0.940
150	alpha	0.6	60x8 weeks	PCR90	M	VAC95	75	Unvaccinate	0.247	0.003	0.001	0.004	0.002	0.002	0.000	118.623	1.495	0.461	1.956	0.757	0.745	0.123	1186	14.950	4.610	19.560	7.570	7.450	1.230
150	alpha	0.6	60x8 weeks	PCR90	M	VAC95	75	Unvaccinate	0.247	0.003	0.001	0.004	0.002	0.002	0.000	118.623	1.495	0.461	1.956	0.757	0.745	0.123	1186	14.950	4.610	19.560	7.570	7.450	1.230
150	alpha	0.6	60x8 weeks	PCR90	M	VAC95	75	Unvaccinate	0.247	0.003	0.001	0.004	0.002	0.002	0.000	118.623	1.495	0.461	1.956	0.757	0.745	0.123	1186	14.950	4.610	19.560	7.570	7.450	1.230
150	alpha	1	60x8 weeks	PCR90	M	VAC95	75	Unvaccinate	0.246	0.003	0.002	0.005	0.002	0.002	0.000	118.200	1.491	0.915	2.406	0.961	0.937	0.115	1182	14.910	9.150	24.060	9.610	9.370	1.150
150	alpha	1	60x8 weeks	PCR90	M	VAC95	75	Unvaccinate	0.246	0.003	0.002	0.005	0.002	0.002	0.000	118.200	1.491	0.915	2.406	0.961	0.937	0.115	1182	14.910	9.150	24.060	9.610	9.370	1.150
150	alpha	1	60x8 weeks	PCR90	M	VAC95	75	Unvaccinate	0.246	0.003	0.002	0.005	0.002	0.002	0.000	118.200	1.491	0.915	2.406	0.961	0.937	0.115	1182	14.910	9.150	24.060	9.610	9.370	1.150
150	alpha	1.2	60x8 weeks	PCR90	M	VAC95	75	Unvaccinate	0.246	0.003	0.002	0.005	0.002	0.002	0.000	118.121	1.540	1.077	2.617	1.044	0.968	0.129	1181	15.400	10.770	26.170	10.440	9.680	1.290
150	alpha	1.2	60x8 weeks	PCR90	M	VAC95	75	Unvaccinate	0.246	0.003	0.002	0.005	0.002	0.002	0.000	118.121	1.540	1.077	2.617	1.044	0.968	0.129	1181	15.400	10.770	26.170	10.440	9.680	1.290
150	alpha	1.2	60x8 weeks	PCR90	M	VAC95	75	Unvaccinate	0.246	0.003	0.002	0.005	0.002	0.002	0.000	118.121	1.540	1.077	2.617	1.044	0.968	0.129	1181	15.400	10.770	26.170	10.440	9.680	1.290
150	delta	0.6	60x8 weeks	PCR90	M	VAC95	75	Unvaccinate	0.245	0.004	0.004	0.007	0.003	0.002	0.000	117.684	1.706	1.807	3.513	1.615	1.075	0.105	1177	17.060	18.070	35.130	16.150	10.750	1.050
150	delta	0.6	60x8 weeks	PCR90	M	VAC95	75	Unvaccinate	0.245	0.004	0.004	0.007	0.003	0.002	0.000	117.684	1.706	1.807	3.513	1.615	1.075	0.105	1177	17.060	18.070	35.130	16.150	10.750	1.050
150	delta	0.6	60x8 weeks	PCR90	M	VAC95	75	Unvaccinate	0.245	0.004	0.004	0.007	0.003	0.002	0.000	117.684	1.706	1.807	3.513	1.615	1.075	0.105	1177	17.060	18.070	35.130	16.150	10.750	1.050
150	delta	1	60x8 weeks	PCR90	M	VAC95	75	Unvaccinate	0.242	0.003	0.010	0.013	0.006	0.004	0.000	115.974	1.623	4.740	6.363	3.003	1.814	0.111	1160	16.230	47.400	63.630	30.030	18.140	1.110
150	delta	1	60x8 weeks	PCR90	M	VAC95	75	Unvaccinate	0.242	0.003	0.010	0.013	0.006	0.004	0.000	115.974	1.623	4.740	6.363	3.003	1.814	0.111	1160	16.230	47.400	63.630	30.030	18.140	1.110
150	delta	1	60x8 weeks	PCR90	M	VAC95	75	Unvaccinate	0.242	0.003	0.010	0.013	0.006	0.004	0.000	115.974	1.623	4.740	6.363	3.003	1.814	0.111	1160	16.230	47.400	63.630	30.030	18.140	1.110
150	delta	1.2	60x8 weeks	PCR90	M	VAC95	75	Unvaccinate	0.239	0.003	0.014	0.017	0.008	0.005	0.000	114.948	1.608	6.781	8.389	3.907	2.389	0.107	1149	16.080	67.810	83.890	39.070	23.890	1.070
150	delta	1.2	60x8 weeks	PCR90	M	VAC95	75	Unvaccinate	0.239	0.003	0.014	0.017	0.008	0.005	0.000	114.948	1.608	6.781	8.389	3.907	2.389	0.107	1149	16.080	67.810	83.890	39.070	23.890	1.070
150	delta	1.2	60x8 weeks	PCR90	M	VAC95	75	Unvaccinate	0.239	0.003	0.014	0.017	0.008	0.005	0.000	114.948	1.608	6.781	8.389	3.907	2.389	0.107	1149	16.080	67.810	83.890	39.070	23.890	1.070
150	original	0.6	60x8 weeks	PCR90	M	VAC95	100	Unvaccinate	0.000	0.001	0.000	0.001	0.000	0.000	0.000	0.000	0.382	0.001	0.383	0.151	0.000	0.000	0	3.820	0.010	3.830	1.510	0.000	0.000
150	original	0.6	60x8 weeks	PCR90	M	VAC95	100	Unvaccinate	0.000	0.001	0.000	0.001	0.000	0.000	0.000	0.000	0.382	0.001	0.383	0.151	0.000	0.000	0	3.820	0.010	3.830	1.510	0.000	0.000
150	original	0.6	60x8 weeks	PCR90	M	VAC95	100	Unvaccinate	0.000	0.001	0.000	0.001	0.000	0.000	0.000	0.000	0.382	0.001	0.383	0.151	0.000	0.000	0	3.820	0.010	3.830	1.510	0.000	0.000
150	original	1	60x8 weeks	PCR90	M	VAC95	100	Unvaccinate	0.000	0.001	0.000	0.001	0.000	0.000	0.000	0.000	0.385	0.000	0.385	0.151	0.000	0.000	0	3.850	0.000	3.850	1.510	0.000	0.000
150	original	1	60x8 weeks	PCR90	M	VAC95	100	Unvaccinate	0.000	0.001	0.000	0.001	0.000	0.000	0.000	0.000	0.385	0.000	0.385	0.151	0.000	0.000	0	3.850	0.000	3.850	1.510	0.000	0.000
150	original	1	60x8 weeks	PCR90	M	VAC95	100	Unvaccinate	0.000	0.001	0.000	0.001	0.000	0.000	0.000	0.000	0.385	0.000	0.385	0.151	0.000	0.000	0	3.850	0.000	3.850	1.510	0.000	0.000
150	original	1.2	60x8 weeks	PCR90	M	VAC95	100	Unvaccinate	0.000	0.001	0.000	0.001	0.000	0.000	0.000	0.000	0.327	0.003	0.330	0.132	0.000	0.000	0	3.270	0.030	3.300	1.320	0.000	0.000
150	original	1.2	60x8 weeks	PCR90	M	VAC95	100	Unvaccinate	0.000	0.001	0.000	0.001	0.000	0.000	0.000	0.000	0.327	0.003	0.330	0.132	0.000	0.000	0	3.270	0.030	3.300	1.320	0.000	0.000
150	original	1.2	60x8 weeks	PCR90	M	VAC95	100	Unvaccinate	0.000	0.001	0.000	0.001	0.000	0.000	0.000	0.000	0.327	0.003	0.330	0.132	0.000	0.000	0	3.270	0.030	3.300	1.320	0.000	0.000
150	alpha	0.6	60x8 weeks	PCR90	M	VAC95	100	Unvaccinate	0.000	0.001	0.000	0.001	0.001	0.000	0.000	0.000	0.463	0.024	0.487	0.244	0.000	0.000	0	4.630	0.240	4.870	2.440	0.000	0.000
150	alpha	0.6	60x8 weeks	PCR90	M	VAC95	100	Unvaccinate	0.000	0.001	0.000	0.001	0.001	0.000	0.000	0.000	0.463	0.024	0.487	0.244	0.000	0.000	0	4.630	0.240	4.870	2.440	0.000	0.000
150	alpha	0.6	60x8 weeks	PCR90	M	VAC95	100	Unvaccinate	0.000	0.001	0.000	0.001	0.001	0.000	0.000	0.000	0.463	0.024	0.487	0.244	0.000	0.000	0	4.630	0.240	4.870	2.440	0.000	0.000
150	alpha	1	60x8 weeks	PCR90	M	VAC95	100	Unvaccinate	0.000	0.001	0.000	0.001	0.001	0.000	0.000	0.000	0.463	0.052	0.515	0.242	0.000	0.000	0	4.630	0.520	5.150	2.420	0.000	0.000
150	alpha	1	60x8 weeks	PCR90	M	VAC95	100	Unvaccinate	0.000	0.001	0.000	0.001	0.001	0.000	0.000	0.000	0.463	0.052	0.515	0.242	0.000	0.000	0	4.630	0.520	5.150	2.420	0.000	0.000
150	alpha	1	60x8 weeks	PCR90	M	VAC95	100	Unvaccinate	0.000	0.001	0.000	0.001	0.001	0.000	0.000	0.000	0.463	0.052	0.515	0.242	0.000	0.000	0	4.630	0.520	5.150	2.420	0.000	0.000
150	alpha	1.2	60x8 weeks	PCR90	M	VAC95	100	Unvaccinate	0.000	0.001	0.000	0.001	0.000	0.000	0.000	0.000	0.441	0.050	0.491	0.236	0.000	0.000	0	4.410	0.500	4.910	2.360	0.000	0.000
150	alpha	1.2	60x8 weeks	PCR90	M	VAC95	100	Unvaccinate	0.000	0.001	0.000	0.001	0.000	0.000	0.000	0.000	0.441	0.050	0.491	0.236	0.000	0.000	0	4.410	0.500	4.910	2.360	0.000	0.000
150	alpha	1.2	60x8 weeks	PCR90	M	VAC95	100	Unvaccinate	0.000	0.001	0.000	0.001	0.000	0.000	0.000	0.000	0.441	0.050	0.491	0.236	0.000	0.000	0	4.410	0.500	4.910	2.360	0.000	0.000
150	delta	0.6	60x8 weeks	PCR90	M	VAC95	100	Unvaccinate	0.000	0.001	0.000	0.002	0.001	0.000	0.000	0.000	0.668	0.143	0.811	0.444	0.000	0.000	0	6.680	1.430	8.110	4.440	0.000	0.000
150	delta	0.6	60x8 weeks	PCR90	M	VAC95	100	Unvaccinate	0.000	0.001	0.000	0.002	0.001	0.000	0.000	0.000	0.668	0.143	0.811	0.444	0.000	0.000	0	6.680	1.430	8.110	4.440	0.000	0.000
150	delta	0.6	60x8 weeks	PCR90	M	VAC95	100	Unvaccinate	0.000	0.001	0.000	0.002	0.001	0.000	0.000	0.000	0.668	0.143	0.811	0.444	0.000	0.000	0	6.680	1.430	8.110	4.440	0.000	0.000
150	delta	1	60x8 weeks	PCR90	M	VAC95	100	Unvaccinate	0.000	0.001	0.000	0.002	0.001	0.000	0.000	0.000	0.693	0.237	0.930	0.487	0.000	0.000	0	6.930	2.370	9.300	4.870	0.000	0.000
150	delta	1	60x8 weeks	PCR90	M	VAC95	100	Unvaccinate	0.000	0.001	0.000	0.002	0.001	0.000	0.000	0.000	0.693	0.237	0.930	0.487	0.000	0.000	0	6.930	2.370	9.300	4.870	0.000	0.000
150	delta	1	60x8 weeks	PCR90	M	VAC95	100	Unvaccinate	0.000	0.001	0.000	0.002	0.001	0.000	0.000	0.000	0.693	0.237	0.930	0.487	0.000	0.000	0	6.930	2.370	9.300	4.870	0.000	0.000
150	delta	1.2	60x8 weeks	PCR90	M	VAC95	100	Unvaccinate	0.000	0.002	0.001	0.002	0.001	0.000	0.000	0.000	0.735	0.365	1.100	0.597	0.000	0.000	0	7.350	3.650	11.000	5.970	0.000	0.000
150	delta	1.2	60x8 weeks	PCR90	M	VAC95	100	Unvaccinate	0.000	0.002	0.001	0.002	0.001	0.000	0.000	0.000	0.735	0.365	1.100	0.597	0.000	0.000	0	7.350	3.650	11.000	5.970	0.000	0.000
150	delta	1.2	60x8 weeks	PCR90	M	VAC95	100	Unvaccinate	0.000	0.002	0.001	0.002	0.001	0.000	0.000	0.000	0.735	0.365	1.100	0.597	0.000	0.000	0	7.350	3.650	11.000	5.970	0.000	0.000
150	original	0.6	60x8 weeks	PCR90	M	VAC70	25	Unvaccinate	0.741	0.008	0.003	0.012	0.004	0.005	0.001	355.624	3.983	1.589	5.572	1.939	2.312	0.334	3556	39.830	15.890	55.720	19.390	23.120	3.340
150	original	0.6	60x8 weeks	PCR90	M	VAC70	25	Unvaccinate	0.741	0.008	0.003	0.012	0.004	0.005	0.001	355.624	3.983	1.589	5.572	1.939	2.312	0.334	3556	39.830	15.890	55.720	19.390	23.120	3.340
150	original	0.6	60x8 weeks	PCR90	M	VAC70	25	Unvaccinate	0.741	0.008	0.003	0.012	0.004	0.005	0.001	355.624	3.983	1.589	5.572	1.939	2.312	0.334	3556	39.830	15.890	55.720	19.390	23.120	3.340
150	original	1	60x8 weeks	PCR90	M	VAC70	25	Unvaccinate	0.737	0.008	0.008	0.016	0.006	0.006	0.001	353.981	3.860	3.772	7.632	2.743	3.109	0.367	3540	38.600	37.720	76.320	27.430	31.090	3.670
150	original	1	60x8 weeks	PCR90	M	VAC70	25	Unvaccinate	0.737	0.008	0.008	0.016	0.006	0.006	0.001	353.981	3.860	3.772	7.632	2.743	3.109	0.367	3540	38.600	37.720	76.320	27.430	31.090	3.670
150	original	1	60x8 weeks	PCR90	M	VAC70	25	Unvaccinate	0.737	0.008	0.008	0.016	0.006	0.006	0.001	353.981	3.860	3.772	7.632	2.743	3.109	0.367	3540	38.600	37.720	76.320	27.430	31.090	3.670
150	original	1.2	60x8 weeks	PCR90	M	VAC70	25	Unvaccinate	0.736	0.008	0.010	0.018	0.006	0.008	0.001	353.273	3.832	4.790	8.622	3.098	3.602	0.347	3533	38.320	47.900	86.220	30.980	36.020	3.470
150	original	1.2	60x8 weeks	PCR90	M	VAC70	25	Unvaccinate	0.736	0.008	0.010	0.018	0.006	0.008	0.001	353.273	3.832	4.790	8.622	3.098	3.602	0.347	3533	38.320	47.900	86.220	30.980	36.020	3.470
150	original	1.2	60x8 weeks	PCR90	M	VAC70	25	Unvaccinate	0.736	0.008	0.010	0.018	0.006	0.008	0.001	353.273	3.832	4.790	8.622	3.098	3.602	0.347	3533	38.320	47.900	86.220	30.980	36.020	3.470
150	alpha	0.6	60x8 weeks	PCR90	M	VAC70	25	Unvaccinate	0.733	0.008	0.012	0.020	0.008	0.009	0.001	351.675	3.846	5.840	9.686	4.003	4.156	0.330	3517	38.460	58.400	96.860	40.030	41.560	3.300
150	alpha	0.6	60x8 weeks	PCR90	M	VAC70	25	Unvaccinate	0.733	0.008	0.012	0.020	0.008	0.009	0.001	351.675	3.846	5.840	9.686	4.003	4.156	0.330	3517	38.460	58.400	96.860	40.030	41.560	3.300
150	alpha	0.6	60x8 weeks	PCR90	M	VAC70	25	Unvaccinate	0.733	0.008	0.012	0.020	0.008	0.009	0.001	351.675	3.846	5.840	9.686	4.003	4.156	0.330	3517	38.460	58.400	96.860	40.030	41.560	3.300
150	alpha	1	60x8 weeks	PCR90	M	VAC70	25	Unvaccinate	0.712	0.007	0.037	0.044	0.019	0.019	0.001	341.840	3.316	17.967	21.283	8.945	9.162	0.288	3418	33.160	179.670	212.830	89.450	91.620	2.880
150	alpha	1	60x8 weeks	PCR90	M	VAC70	25	Unvaccinate	0.712	0.007	0.037	0.044	0.019	0.019	0.001	341.840	3.316	17.967	21.283	8.945	9.162	0.288	3418	33.160	179.670	212.830	89.450	91.620	2.880
150	alpha	1	60x8 weeks	PCR90	M	VAC70	25	Unvaccinate	0.712	0.007	0.037	0.044	0.019	0.019	0.001	341.840	3.316	17.967	21.283	8.945	9.162	0.288	3418	33.160	179.670	212.830	89.450	91.620	2.880
150	alpha	1.2	60x8 weeks	PCR90	M	VAC70	25	Unvaccinate	0.695	0.007	0.056	0.063	0.027	0.027	0.001	333.569	3.189	26.883	30.072	13.020	13.011	0.268	3336	31.890	268.830	300.720	130.200	130.110	2.680
150	alpha	1.2	60x8 weeks	PCR90	M	VAC70	25	Unvaccinate	0.695	0.007	0.056	0.063	0.027	0.027	0.001	333.569	3.189	26.883	30.072	13.020	13.011	0.268	3336	31.890	268.830	300.720	130.200	130.110	2.680
150	alpha	1.2	60x8 weeks	PCR90	M	VAC70	25	Unvaccinate	0.695	0.007	0.056	0.063	0.027	0.027	0.001	333.569	3.189	26.883	30.072	13.020	13.011	0.268	3336	31.890	268.830	300.720	130.200	130.110	2.680
150	delta	0.6	60x8 weeks	PCR90	M	VAC70	25	Unvaccinate	0.687	0.006	0.066	0.073	0.035	0.028	0.001	329.633	2.974	31.894	34.868	16.696	13.527	0.247	3296	29.740	318.940	348.680	166.960	135.270	2.470
150	delta	0.6	60x8 weeks	PCR90	M	VAC70	25	Unvaccinate	0.687	0.006	0.066	0.073	0.035	0.028	0.001	329.633	2.974	31.894	34.868	16.696	13.527	0.247	3296	29.740	318.940	348.680	166.960	135.270	2.470
150	delta	0.6	60x8 weeks	PCR90	M	VAC70	25	Unvaccinate	0.687	0.006	0.066	0.073	0.035	0.028	0.001	329.633	2.974	31.894	34.868	16.696	13.527	0.247	3296	29.740	318.940	348.680	166.960	135.270	2.470
150	delta	1	60x8 weeks	PCR90	M	VAC70	25	Unvaccinate	0.649	0.004	0.109	0.113	0.056	0.043	0.000	311.663	2.020	52.087	54.107	26.972	20.757	0.181	3117	20.200	520.870	541.070	269.720	207.570	1.810
150	delta	1	60x8 weeks	PCR90	M	VAC70	25	Unvaccinate	0.649	0.004	0.109	0.113	0.056	0.043	0.000	311.663	2.020	52.087	54.107	26.972	20.757	0.181	3117	20.200	520.870	541.070	269.720	207.570	1.810

150	delta	1	60x8 weeks	PCR90	M	VAC70	25	Unvaccinate	0.649	0.004	0.109	0.113	0.056	0.043	0.000	311.663	2.020	52.087	54.107	26.972	20.757	0.181	3117	20.200	520.870	541.070	269.720	207.570	1.810
150	delta	1.2	60x8 weeks	PCR90	M	VAC70	25	Unvaccinate	0.645	0.004	0.113	0.117	0.058	0.045	0.000	309.741	1.863	54.239	56.102	28.079	21.487	0.163	3097	18.630	542.390	561.020	280.790	214.870	1.630
150	delta	1.2	60x8 weeks	PCR90	M	VAC70	25	Unvaccinate	0.645	0.004	0.113	0.117	0.058	0.045	0.000	309.741	1.863	54.239	56.102	28.079	21.487	0.163	3097	18.630	542.390	561.020	280.790	214.870	1.630
150	delta	1.2	60x8 weeks	PCR90	M	VAC70	25	Unvaccinate	0.645	0.004	0.113	0.117	0.058	0.045	0.000	309.741	1.863	54.239	56.102	28.079	21.487	0.163	3097	18.630	542.390	561.020	280.790	214.870	1.630
150	original	0.6	60x8 weeks	PCR90	M	VAC70	50	Unvaccinate	0.495	0.007	0.002	0.008	0.003	0.003	0.000	237.447	3.229	0.775	4.004	1.424	1.468	0.230	2374	32.290	7.750	40.040	14.240	14.680	2.300
150	original	0.6	60x8 weeks	PCR90	M	VAC70	50	Unvaccinate	0.495	0.007	0.002	0.008	0.003	0.003	0.000	237.447	3.229	0.775	4.004	1.424	1.468	0.230	2374	32.290	7.750	40.040	14.240	14.680	2.300
150	original	0.6	60x8 weeks	PCR90	M	VAC70	50	Unvaccinate	0.495	0.007	0.002	0.008	0.003	0.003	0.000	237.447	3.229	0.775	4.004	1.424	1.468	0.230	2374	32.290	7.750	40.040	14.240	14.680	2.300
150	original	1	60x8 weeks	PCR90	M	VAC70	50	Unvaccinate	0.494	0.007	0.003	0.010	0.004	0.003	0.000	237.022	3.134	1.550	4.684	1.771	1.589	0.226	2370	31.340	15.500	46.840	17.710	15.890	2.260
150	original	1	60x8 weeks	PCR90	M	VAC70	50	Unvaccinate	0.494	0.007	0.003	0.010	0.004	0.003	0.000	237.022	3.134	1.550	4.684	1.771	1.589	0.226	2370	31.340	15.500	46.840	17.710	15.890	2.260
150	original	1	60x8 weeks	PCR90	M	VAC70	50	Unvaccinate	0.494	0.007	0.003	0.010	0.004	0.003	0.000	237.022	3.134	1.550	4.684	1.771	1.589	0.226	2370	31.340	15.500	46.840	17.710	15.890	2.260
150	original	1.2	60x8 weeks	PCR90	M	VAC70	50	Unvaccinate	0.493	0.006	0.004	0.010	0.004	0.004	0.000	236.803	3.093	1.905	4.998	1.866	1.704	0.208	2368	30.930	19.050	49.980	18.660	17.040	2.080
150	original	1.2	60x8 weeks	PCR90	M	VAC70	50	Unvaccinate	0.493	0.006	0.004	0.010	0.004	0.004	0.000	236.803	3.093	1.905	4.998	1.866	1.704	0.208	2368	30.930	19.050	49.980	18.660	17.040	2.080
150	original	1.2	60x8 weeks	PCR90	M	VAC70	50	Unvaccinate	0.493	0.006	0.004	0.010	0.004	0.004	0.000	236.803	3.093	1.905	4.998	1.866	1.704	0.208	2368	30.930	19.050	49.980	18.660	17.040	2.080
150	alpha	0.6	60x8 weeks	PCR90	M	VAC70	50	Unvaccinate	0.491	0.006	0.006	0.012	0.005	0.004	0.000	235.897	3.113	2.716	5.829	2.548	2.134	0.228	2359	31.130	27.160	58.290	25.480	21.340	2.280
150	alpha	0.6	60x8 weeks	PCR90	M	VAC70	50	Unvaccinate	0.491	0.006	0.006	0.012	0.005	0.004	0.000	235.897	3.113	2.716	5.829	2.548	2.134	0.228	2359	31.130	27.160	58.290	25.480	21.340	2.280
150	alpha	0.6	60x8 weeks	PCR90	M	VAC70	50	Unvaccinate	0.491	0.006	0.006	0.012	0.005	0.004	0.000	235.897	3.113	2.716	5.829	2.548	2.134	0.228	2359	31.130	27.160	58.290	25.480	21.340	2.280
150	alpha	1	60x8 weeks	PCR90	M	VAC70	50	Unvaccinate	0.485	0.006	0.015	0.021	0.010	0.008	0.000	232.781	3.031	7.196	10.227	4.576	3.614	0.210	2328	30.310	71.960	102.270	45.760	36.140	2.100
150	alpha	1	60x8 weeks	PCR90	M	VAC70	50	Unvaccinate	0.485	0.006	0.015	0.021	0.010	0.008	0.000	232.781	3.031	7.196	10.227	4.576	3.614	0.210	2328	30.310	71.960	102.270	45.760	36.140	2.100
150	alpha	1	60x8 weeks	PCR90	M	VAC70	50	Unvaccinate	0.485	0.006	0.015	0.021	0.010	0.008	0.000	232.781	3.031	7.196	10.227	4.576	3.614	0.210	2328	30.310	71.960	102.270	45.760	36.140	2.100
150	alpha	1.2	60x8 weeks	PCR90	M	VAC70	50	Unvaccinate	0.480	0.006	0.023	0.030	0.013	0.010	0.000	230.238	2.911	11.261	14.172	6.384	4.979	0.234	2302	29.110	112.610	141.720	63.840	49.790	2.340
150	alpha	1.2	60x8 weeks	PCR90	M	VAC70	50	Unvaccinate	0.480	0.006	0.023	0.030	0.013	0.010	0.000	230.238	2.911	11.261	14.172	6.384	4.979	0.234	2302	29.110	112.610	141.720	63.840	49.790	2.340
150	alpha	1.2	60x8 weeks	PCR90	M	VAC70	50	Unvaccinate	0.480	0.006	0.023	0.030	0.013	0.010	0.000	230.238	2.911	11.261	14.172	6.384	4.979	0.234	2302	29.110	112.610	141.720	63.840	49.790	2.340
150	delta	0.6	60x8 weeks	PCR90	M	VAC70	50	Unvaccinate	0.474	0.006	0.033	0.039	0.020	0.012	0.000	227.582	2.844	15.796	18.640	9.385	5.581	0.191	2276	28.440	157.960	186.400	93.850	55.810	1.910
150	delta	0.6	60x8 weeks	PCR90	M	VAC70	50	Unvaccinate	0.474	0.006	0.033	0.039	0.020	0.012	0.000	227.582	2.844	15.796	18.640	9.385	5.581	0.191	2276	28.440	157.960	186.400	93.850	55.810	1.910
150	delta	0.6	60x8 weeks	PCR90	M	VAC70	50	Unvaccinate	0.474	0.006	0.033	0.039	0.020	0.012	0.000	227.582	2.844	15.796	18.640	9.385	5.581	0.191	2276	28.440	157.960	186.400	93.850	55.810	1.910
150	delta	1	60x8 weeks	PCR90	M	VAC70	50	Unvaccinate	0.446	0.005	0.084	0.088	0.047	0.024	0.000	213.848	2.239	40.186	42.425	22.444	11.592	0.137	2138	22.390	401.860	424.250	224.440	115.920	1.370
150	delta	1	60x8 weeks	PCR90	M	VAC70	50	Unvaccinate	0.446	0.005	0.084	0.088	0.047	0.024	0.000	213.848	2.239	40.186	42.425	22.444	11.592	0.137	2138	22.390	401.860	424.250	224.440	115.920	1.370
150	delta	1	60x8 weeks	PCR90	M	VAC70	50	Unvaccinate	0.446	0.005	0.084	0.088	0.047	0.024	0.000	213.848	2.239	40.186	42.425	22.444	11.592	0.137	2138	22.390	401.860	424.250	224.440	115.920	1.370
150	delta	1.2	60x8 weeks	PCR90	M	VAC70	50	Unvaccinate	0.440	0.004	0.095	0.099	0.053	0.026	0.000	211.137	1.961	45.764	47.725	25.528	12.550	0.136	2111	19.610	457.640	477.250	255.280	125.500	1.360
150	delta	1.2	60x8 weeks	PCR90	M	VAC70	50	Unvaccinate	0.440	0.004	0.095	0.099	0.053	0.026	0.000	211.137	1.961	45.764	47.725	25.528	12.550	0.136	2111	19.610	457.640	477.250	255.280	125.500	1.360
150	delta	1.2	60x8 weeks	PCR90	M	VAC70	50	Unvaccinate	0.440	0.004	0.095	0.099	0.053	0.026	0.000	211.137	1.961	45.764	47.725	25.528	12.550	0.136	2111	19.610	457.640	477.250	255.280	125.500	1.360
150	original	0.6	60x8 weeks	PCR90	M	VAC70	75	Unvaccinate	0.248	0.005	0.001	0.006	0.002	0.001	0.000	118.900	2.387	0.282	2.669	1.112	0.607	0.093	1189	23.870	2.820	26.690	11.120	6.070	0.930
150	original	0.6	60x8 weeks	PCR90	M	VAC70	75	Unvaccinate	0.248	0.005	0.001	0.006	0.002	0.001	0.000	118.900	2.387	0.282	2.669	1.112	0.607	0.093	1189	23.870	2.820	26.690	11.120	6.070	0.930
150	original	0.6	60x8 weeks	PCR90	M	VAC70	75	Unvaccinate	0.248	0.005	0.001	0.006	0.002	0.001	0.000	118.900	2.387	0.282	2.669	1.112	0.607	0.093	1189	23.870	2.820	26.690	11.120	6.070	0.930
150	original	1	60x8 weeks	PCR90	M	VAC70	75	Unvaccinate	0.247	0.005	0.001	0.006	0.002	0.001	0.000	118.741	2.339	0.523	2.862	1.175	0.700	0.121	1187	23.390	5.230	28.620	11.750	7.000	1.210
150	original	1	60x8 weeks	PCR90	M	VAC70	75	Unvaccinate	0.247	0.005	0.001	0.006	0.002	0.001	0.000	118.741	2.339	0.523	2.862	1.175	0.700	0.121	1187	23.390	5.230	28.620	11.750	7.000	1.210
150	original	1	60x8 weeks	PCR90	M	VAC70	75	Unvaccinate	0.247	0.005	0.001	0.006	0.002	0.001	0.000	118.741	2.339	0.523	2.862	1.175	0.700	0.121	1187	23.390	5.230	28.620	11.750	7.000	1.210
150	original	1.2	60x8 weeks	PCR90	M	VAC70	75	Unvaccinate	0.247	0.005	0.001	0.006	0.003	0.001	0.000	118.735	2.362	0.623	2.985	1.267	0.655	0.129	1187	23.620	6.230	29.850	12.670	6.550	1.290
150	original	1.2	60x8 weeks	PCR90	M	VAC70	75	Unvaccinate	0.247	0.005	0.001	0.006	0.003	0.001	0.000	118.735	2.362	0.623	2.985	1.267	0.655	0.129	1187	23.620	6.230	29.850	12.670	6.550	1.290
150	original	1.2	60x8 weeks	PCR90	M	VAC70	75	Unvaccinate	0.247	0.005	0.001	0.006	0.003	0.001	0.000	118.735	2.362	0.623	2.985	1.267	0.655	0.129	1187	23.620	6.230	29.850	12.670	6.550	1.290
150	alpha	0.6	60x8 weeks	PCR90	M	VAC70	75	Unvaccinate	0.247	0.005	0.002	0.007	0.004	0.002	0.000	118.323	2.394	1.183	3.577	1.765	0.848	0.111	1183	23.940	11.830	35.770	17.650	8.480	1.110
150	alpha	0.6	60x8 weeks	PCR90	M	VAC70	75	Unvaccinate	0.247	0.005	0.002	0.007	0.004	0.002	0.000	118.323	2.394	1.183	3.577	1.765	0.848	0.111	1183	23.940	11.830	35.770	17.650	8.480	1.110
150	alpha	0.6	60x8 weeks	PCR90	M	VAC70	75	Unvaccinate	0.247	0.005	0.002	0.007	0.004	0.002	0.000	118.323	2.394	1.183	3.577	1.765	0.848	0.111	1183	23.940	11.830	35.770	17.650	8.480	1.110
150	alpha	1	60x8 weeks	PCR90	M	VAC70	75	Unvaccinate	0.245	0.005	0.005	0.010	0.005	0.002	0.000	117.794	2.404	2.428	4.832	2.376	1.138	0.105	1178	24.040	24.280	48.320	23.760	11.380	1.050
150	alpha	1	60x8 weeks	PCR90	M	VAC70	75	Unvaccinate	0.245	0.005	0.005	0.010	0.005	0.002	0.000	117.794	2.404	2.428	4.832	2.376	1.138	0.105	1178	24.040	24.280	48.320	23.760	11.380	1.050
150	alpha	1	60x8 weeks	PCR90	M	VAC70	75	Unvaccinate	0.245	0.005	0.005	0.010	0.005	0.002	0.000	117.794	2.404	2.428	4.832	2.376	1.138	0.105	1178	24.040	24.280	48.320	23.760	11.380	1.050
150	alpha	1.2	60x8 weeks	PCR90	M	VAC70	75	Unvaccinate	0.244	0.005	0.007	0.012	0.006	0.003	0.000	117.358	2.314	3.521	5.835	2.949	1.333	0.116	1174	23.140	35.210	58.350	29.490	13.330	1.160
150	alpha	1.2	60x8 weeks	PCR90	M	VAC70	75	Unvaccinate	0.244	0.005	0.007	0.012	0.006	0.003	0.000	117.358	2.314	3.521	5.835	2.949	1.333	0.116	1174	23.140	35.210	58.350	29.490	13.330	1.160
150	alpha	1.2	60x8 weeks	PCR90	M	VAC70	75	Unvaccinate	0.244	0.005	0.007	0.012	0.006	0.003	0.000	117.358	2.314	3.521	5.835	2.949	1.333	0.116	1174	23.140	35.210	58.350	29.490	13.330	1.160
150	delta	0.6	60x8 weeks	PCR90	M	VAC70	75	Unvaccinate	0.243	0.006	0.012	0.018	0.009	0.003	0.000	116.629	2.650	5.759	8.409	4.518	1.533	0.102	1166	26.500	57.590	84.090	45.180	15.330	1.020
150	delta	0.6	60x8 weeks	PCR90	M	VAC70	75	Unvaccinate	0.243	0.006	0.012	0.018	0.009	0.003	0.000	116.629	2.650	5.759	8.409	4.518	1.533	0.102	1166	26.500	57.590	84.090	45.180	15.330	1.020
150	delta	0.6	60x8 weeks	PCR90	M	VAC70	75	Unvaccinate	0.243	0.006	0.012	0.018	0.009	0.003	0.000	116.629	2.650	5.759	8.409	4.518	1.533	0.102	1166	26.500	57.590	84.090	45.180	15.330	1.020
150	delta	1	60x8 weeks	PCR90	M	VAC70	75	Unvaccinate	0.234	0.005	0.041	0.045	0.025	0.007	0.000	112.363	2.294	19.502	21.796	12.140	3.415	0.092	1124	22.940	195.020	217.960	121.400	34.150	0.920
150	delta	1	60x8 weeks	PCR90	M	VAC70	75	Unvaccinate	0.234	0.005	0.041	0.045	0.025	0.007	0.000	112.363	2.294	19.502	21.796	12.140	3.415	0.092	1124	22.940	195.020	217.960	121.400	34.150	0.920
150	delta	1	60x8 weeks	PCR90	M	VAC70	75	Unvaccinate	0.234	0.005	0.041	0.045	0.025	0.007	0.000	112.363	2.294	19.502	21.796	12.140	3.415	0.092	1124	22.940	195.020	217.960	121.400	34.150	0.920
150	delta	1.2	60x8 weeks	PCR90	M	VAC70	75	Unvaccinate	0.229	0.004	0.059	0.063	0.036	0.009	0.000	109.685	2.114	28.306	30.420	17.447	4.538	0.075	1097	21.140	283.060	304.200	174.470	45.380	0.750
150	delta	1.2	60x8 weeks	PCR90	M	VAC70	75	Unvaccinate	0.229	0.004	0.059	0.063	0.036	0.009	0.000	109.685	2.114	28.306	30.420	17.447	4.538	0.075	1097	21.140	283.060	304.200	174.470	45.380	0.750
150	delta	1.2	60x8 weeks	PCR90	M	VAC70	75	Unvaccinate	0.229	0.004	0.059	0.063	0.036	0.009	0.000	109.685	2.114	28.306	30.420	17.447	4.538	0.075	1097	21.140	283.060	304.200	174.470	45.380	0.750
150	original	0.6	60x8 weeks	PCR90	M	VAC70	100	Unvaccinate	0.000	0.003	0.000	0.003	0.002	0.000	0.000	0.000	1.629	0.020	1.649	0.743	0.000	0.000	0	16.290	0.200	16.490	7.430	0.000	0.000
150	original	0.6	60x8 weeks	PCR90	M	VAC70	100	Unvaccinate	0.000	0.003	0.000	0.003	0.002	0.000	0.000	0.000	1.629	0.020	1.649	0.743	0.000	0.000	0	16.290	0.200	16.490	7.430	0.000	0.000
150	original	0.6	60x8 weeks	PCR90	M	VAC70	100	Unvaccinate	0.000	0.003	0.000	0.003	0.002	0.000	0.000	0.000	1.629	0.020	1.649	0.743	0.000	0.000	0	16.290	0.200	16.490	7.430	0.000	0.000
150	original	1	60x8 weeks	PCR90	M	VAC70	100	Unvaccinate	0.000	0.003	0.000	0.004	0.002	0.000	0.000	0.000	1.655	0.026	1.681	0.829	0.000	0.000	0	16.550	0.260	16.810	8.290	0.000	0.000
150	original	1	60x8 weeks	PCR90	M	VAC70	100	Unvaccinate	0.000	0.003	0.000	0.004	0.002	0.000	0.000	0.000	1.655	0.026	1.681	0.829	0.000	0.000	0	16.550	0.260	16.810	8.290	0.000	0.000
150	original	1	60x8 weeks	PCR90	M	VAC70	100	Unvaccinate	0.000	0.003	0.000	0.004	0.002	0.000	0.000	0.000	1.655	0.026	1.681	0.829	0.000	0.000	0	16.550	0.260	16.810	8.290	0.000	0.000
150	original	1.2	60x8 weeks	PCR90	M	VAC70	100	Unvaccinate	0.000	0.003	0.000	0.004	0.002	0.000	0.000	0.000	1.660	0.022	1.682	0.829	0.000	0.000	0	16.600	0.220	16.820	8.290	0.000	0.000
150	original	1.2	60x8 weeks	PCR90	M	VAC70	100	Unvaccinate	0.000	0.003	0.000	0.004	0.002	0.000	0.000	0.000	1.660	0.022	1.682	0.829	0.000	0.000	0	16.600	0.220	16.820	8.290	0.000	0.000
150	original	1.2	60x8 weeks	PCR90	M	VAC70	100	Unvaccinate	0.000	0.003	0.000	0.004	0.002	0.000	0.000	0.000	1.660	0.022	1.682	0.829	0.000	0.000	0	16.600	0.220	16.820	8.290	0.000	0.000
150	alpha	0.6	60x8 weeks	PCR90	M	VAC70	100	Unvaccinate	0.000	0.003	0.001	0.004	0.002	0.000	0.000	0.000	1.591	0.305	1.896	1.149	0.000	0.000	0	15.910	3.050	18.960	11.490	0.000	0.000
150	alpha	0.6	60x8 weeks	PCR90	M	VAC70	100	Unvaccinate	0.000	0.003	0.001	0.004	0.002	0.000	0.000	0.000	1.591	0.305	1.896	1.149	0.000	0.000	0	15.910	3.050	18.960	11.490	0.000	0.000
150	alpha	0.6	60x8 weeks	PCR90	M	VAC70	100	Unvaccinate	0.000	0.003	0.001	0.004	0.002	0.000	0.000	0.000	1.591	0.305	1.896	1.149	0.000	0.000	0	15.910	3.050	18.960	11.490	0.000	0.000
150	alpha	1	60x8 weeks	PCR90	M	VAC70	100	Unvaccinate	0.000	0.003	0.001	0.005	0.003	0.000	0.000	0.000	1.625	0.631	2.256	1.342	0.000	0.000	0	16.250	6.310	22.560	13.420	0.000	0.000

150	alpha	1	60x8 weeks	PCR90	M	VAC70	100	Unvaccinate	0.000	0.003	0.001	0.005	0.003	0.000	0.000	0.000	1.625	0.631	2.256	1.342	0.000	0.000	0	16.250	6.310	22.560	13.420	0.000	0.000
150	alpha	1	60x8 weeks	PCR90	M	VAC70	100	Unvaccinate	0.000	0.003	0.001	0.005	0.003	0.000	0.000	0.000	1.625	0.631	2.256	1.342	0.000	0.000	0	16.250	6.310	22.560	13.420	0.000	0.000
150	alpha	1.2	60x8 weeks	PCR90	M	VAC70	100	Unvaccinate	0.000	0.003	0.002	0.005	0.003	0.000	0.000	0.000	1.578	0.743	2.321	1.323	0.000	0.000	0	15.780	7.430	23.210	13.230	0.000	0.000
150	alpha	1.2	60x8 weeks	PCR90	M	VAC70	100	Unvaccinate	0.000	0.003	0.002	0.005	0.003	0.000	0.000	0.000	1.578	0.743	2.321	1.323	0.000	0.000	0	15.780	7.430	23.210	13.230	0.000	0.000
150	alpha	1.2	60x8 weeks	PCR90	M	VAC70	100	Unvaccinate	0.000	0.003	0.002	0.005	0.003	0.000	0.000	0.000	1.578	0.743	2.321	1.323	0.000	0.000	0	15.780	7.430	23.210	13.230	0.000	0.000
150	delta	0.6	60x8 weeks	PCR90	M	VAC70	100	Unvaccinate	0.000	0.004	0.004	0.008	0.005	0.000	0.000	0.000	2.092	1.774	3.866	2.426	0.000	0.000	0	20.920	17.740	38.660	24.260	0.000	0.000
150	delta	0.6	60x8 weeks	PCR90	M	VAC70	100	Unvaccinate	0.000	0.004	0.004	0.008	0.005	0.000	0.000	0.000	2.092	1.774	3.866	2.426	0.000	0.000	0	20.920	17.740	38.660	24.260	0.000	0.000
150	delta	0.6	60x8 weeks	PCR90	M	VAC70	100	Unvaccinate	0.000	0.004	0.004	0.008	0.005	0.000	0.000	0.000	2.092	1.774	3.866	2.426	0.000	0.000	0	20.920	17.740	38.660	24.260	0.000	0.000
150	delta	1	60x8 weeks	PCR90	M	VAC70	100	Unvaccinate	0.000	0.004	0.010	0.014	0.009	0.000	0.000	0.000	1.964	4.792	6.756	4.154	0.000	0.000	0	19.640	47.920	67.560	41.540	0.000	0.000
150	delta	1	60x8 weeks	PCR90	M	VAC70	100	Unvaccinate	0.000	0.004	0.010	0.014	0.009	0.000	0.000	0.000	1.964	4.792	6.756	4.154	0.000	0.000	0	19.640	47.920	67.560	41.540	0.000	0.000
150	delta	1	60x8 weeks	PCR90	M	VAC70	100	Unvaccinate	0.000	0.004	0.010	0.014	0.009	0.000	0.000	0.000	1.964	4.792	6.756	4.154	0.000	0.000	0	19.640	47.920	67.560	41.540	0.000	0.000
150	delta	1.2	60x8 weeks	PCR90	M	VAC70	100	Unvaccinate	0.000	0.004	0.017	0.021	0.013	0.000	0.000	0.000	1.977	8.239	10.216	6.234	0.000	0.000	0	19.770	82.390	102.160	62.340	0.000	0.000
150	delta	1.2	60x8 weeks	PCR90	M	VAC70	100	Unvaccinate	0.000	0.004	0.017	0.021	0.013	0.000	0.000	0.000	1.977	8.239	10.216	6.234	0.000	0.000	0	19.770	82.390	102.160	62.340	0.000	0.000
150	delta	1.2	60x8 weeks	PCR90	M	VAC70	100	Unvaccinate	0.000	0.004	0.017	0.021	0.013	0.000	0.000	0.000	1.977	8.239	10.216	6.234	0.000	0.000	0	19.770	82.390	102.160	62.340	0.000	0.000
150	original	0.6	60x8 weeks	PCR90	M	VAC50	25	Unvaccinate	0.741	0.009	0.004	0.012	0.004	0.005	0.001	355.698	4.136	1.740	5.876	2.157	2.307	0.318	3557	41.360	17.400	58.760	21.570	23.070	3.180
150	original	0.6	60x8 weeks	PCR90	M	VAC50	25	Unvaccinate	0.741	0.009	0.004	0.012	0.004	0.005	0.001	355.698	4.136	1.740	5.876	2.157	2.307	0.318	3557	41.360	17.400	58.760	21.570	23.070	3.180
150	original	0.6	60x8 weeks	PCR90	M	VAC50	25	Unvaccinate	0.741	0.009	0.004	0.012	0.004	0.005	0.001	355.698	4.136	1.740	5.876	2.157	2.307	0.318	3557	41.360	17.400	58.760	21.570	23.070	3.180
150	original	1	60x8 weeks	PCR90	M	VAC50	25	Unvaccinate	0.738	0.009	0.008	0.016	0.006	0.006	0.001	354.338	4.119	3.732	7.851	2.821	3.067	0.363	3543	41.190	37.320	78.510	28.210	30.670	3.630
150	original	1	60x8 weeks	PCR90	M	VAC50	25	Unvaccinate	0.738	0.009	0.008	0.016	0.006	0.006	0.001	354.338	4.119	3.732	7.851	2.821	3.067	0.363	3543	41.190	37.320	78.510	28.210	30.670	3.630
150	original	1	60x8 weeks	PCR90	M	VAC50	25	Unvaccinate	0.738	0.009	0.008	0.016	0.006	0.006	0.001	354.338	4.119	3.732	7.851	2.821	3.067	0.363	3543	41.190	37.320	78.510	28.210	30.670	3.630
150	original	1.2	60x8 weeks	PCR90	M	VAC50	25	Unvaccinate	0.735	0.008	0.012	0.020	0.008	0.008	0.001	352.792	4.075	5.699	9.774	3.670	3.837	0.304	3528	40.750	56.990	97.740	36.700	38.370	3.040
150	original	1.2	60x8 weeks	PCR90	M	VAC50	25	Unvaccinate	0.735	0.008	0.012	0.020	0.008	0.008	0.001	352.792	4.075	5.699	9.774	3.670	3.837	0.304	3528	40.750	56.990	97.740	36.700	38.370	3.040
150	original	1.2	60x8 weeks	PCR90	M	VAC50	25	Unvaccinate	0.735	0.008	0.012	0.020	0.008	0.008	0.001	352.792	4.075	5.699	9.774	3.670	3.837	0.304	3528	40.750	56.990	97.740	36.700	38.370	3.040
150	alpha	0.6	60x8 weeks	PCR90	M	VAC50	25	Unvaccinate	0.732	0.008	0.014	0.023	0.009	0.009	0.001	351.310	3.981	6.828	10.809	4.514	4.461	0.314	3513	39.810	68.280	108.090	45.140	44.610	3.140
150	alpha	0.6	60x8 weeks	PCR90	M	VAC50	25	Unvaccinate	0.732	0.008	0.014	0.023	0.009	0.009	0.001	351.310	3.981	6.828	10.809	4.514	4.461	0.314	3513	39.810	68.280	108.090	45.140	44.610	3.140
150	alpha	0.6	60x8 weeks	PCR90	M	VAC50	25	Unvaccinate	0.732	0.008	0.014	0.023	0.009	0.009	0.001	351.310	3.981	6.828	10.809	4.514	4.461	0.314	3513	39.810	68.280	108.090	45.140	44.610	3.140
150	alpha	1	60x8 weeks	PCR90	M	VAC50	25	Unvaccinate	0.708	0.007	0.044	0.051	0.022	0.021	0.001	339.893	3.534	21.094	24.628	10.732	10.103	0.270	3399	35.340	210.940	246.280	107.320	101.030	2.700
150	alpha	1	60x8 weeks	PCR90	M	VAC50	25	Unvaccinate	0.708	0.007	0.044	0.051	0.022	0.021	0.001	339.893	3.534	21.094	24.628	10.732	10.103	0.270	3399	35.340	210.940	246.280	107.320	101.030	2.700
150	alpha	1	60x8 weeks	PCR90	M	VAC50	25	Unvaccinate	0.708	0.007	0.044	0.051	0.022	0.021	0.001	339.893	3.534	21.094	24.628	10.732	10.103	0.270	3399	35.340	210.940	246.280	107.320	101.030	2.700
150	alpha	1.2	60x8 weeks	PCR90	M	VAC50	25	Unvaccinate	0.690	0.007	0.066	0.073	0.032	0.030	0.001	331.404	3.172	31.754	34.926	15.568	14.177	0.251	3314	31.720	317.540	349.260	155.680	141.770	2.510
150	alpha	1.2	60x8 weeks	PCR90	M	VAC50	25	Unvaccinate	0.690	0.007	0.066	0.073	0.032	0.030	0.001	331.404	3.172	31.754	34.926	15.568	14.177	0.251	3314	31.720	317.540	349.260	155.680	141.770	2.510
150	alpha	1.2	60x8 weeks	PCR90	M	VAC50	25	Unvaccinate	0.690	0.007	0.066	0.073	0.032	0.030	0.001	331.404	3.172	31.754	34.926	15.568	14.177	0.251	3314	31.720	317.540	349.260	155.680	141.770	2.510
150	delta	0.6	60x8 weeks	PCR90	M	VAC50	25	Unvaccinate	0.679	0.006	0.080	0.086	0.042	0.032	0.000	325.737	2.934	38.231	41.165	20.265	15.247	0.213	3257	29.340	382.310	411.650	202.650	152.470	2.130
150	delta	0.6	60x8 weeks	PCR90	M	VAC50	25	Unvaccinate	0.679	0.006	0.080	0.086	0.042	0.032	0.000	325.737	2.934	38.231	41.165	20.265	15.247	0.213	3257	29.340	382.310	411.650	202.650	152.470	2.130
150	delta	0.6	60x8 weeks	PCR90	M	VAC50	25	Unvaccinate	0.679	0.006	0.080	0.086	0.042	0.032	0.000	325.737	2.934	38.231	41.165	20.265	15.247	0.213	3257	29.340	382.310	411.650	202.650	152.470	2.130
150	delta	1	60x8 weeks	PCR90	M	VAC50	25	Unvaccinate	0.648	0.004	0.113	0.117	0.059	0.044	0.000	311.123	1.980	54.352	56.332	28.530	20.928	0.145	3111	19.800	543.520	563.320	285.300	209.280	1.450
150	delta	1	60x8 weeks	PCR90	M	VAC50	25	Unvaccinate	0.648	0.004	0.113	0.117	0.059	0.044	0.000	311.123	1.980	54.352	56.332	28.530	20.928	0.145	3111	19.800	543.520	563.320	285.300	209.280	1.450
150	delta	1	60x8 weeks	PCR90	M	VAC50	25	Unvaccinate	0.648	0.004	0.113	0.117	0.059	0.044	0.000	311.123	1.980	54.352	56.332	28.530	20.928	0.145	3111	19.800	543.520	563.320	285.300	209.280	1.450
150	delta	1.2	60x8 weeks	PCR90	M	VAC50	25	Unvaccinate	0.646	0.004	0.116	0.120	0.061	0.044	0.000	310.065	1.862	55.573	57.435	29.298	21.257	0.164	3101	18.620	555.730	574.350	292.980	212.570	1.640
150	delta	1.2	60x8 weeks	PCR90	M	VAC50	25	Unvaccinate	0.646	0.004	0.116	0.120	0.061	0.044	0.000	310.065	1.862	55.573	57.435	29.298	21.257	0.164	3101	18.620	555.730	574.350	292.980	212.570	1.640
150	delta	1.2	60x8 weeks	PCR90	M	VAC50	25	Unvaccinate	0.646	0.004	0.116	0.120	0.061	0.044	0.000	310.065	1.862	55.573	57.435	29.298	21.257	0.164	3101	18.620	555.730	574.350	292.980	212.570	1.640
150	original	0.6	60x8 weeks	PCR90	M	VAC50	50	Unvaccinate	0.495	0.008	0.002	0.009	0.004	0.003	0.001	237.369	3.633	0.921	4.554	1.854	1.383	0.249	2374	36.330	9.210	45.540	18.540	13.830	2.490
150	original	0.6	60x8 weeks	PCR90	M	VAC50	50	Unvaccinate	0.495	0.008	0.002	0.009	0.004	0.003	0.001	237.369	3.633	0.921	4.554	1.854	1.383	0.249	2374	36.330	9.210	45.540	18.540	13.830	2.490
150	original	0.6	60x8 weeks	PCR90	M	VAC50	50	Unvaccinate	0.495	0.008	0.002	0.009	0.004	0.003	0.001	237.369	3.633	0.921	4.554	1.854	1.383	0.249	2374	36.330	9.210	45.540	18.540	13.830	2.490
150	original	1	60x8 weeks	PCR90	M	VAC50	50	Unvaccinate	0.494	0.008	0.004	0.011	0.005	0.003	0.000	236.963	3.604	1.863	5.467	2.201	1.639	0.233	2370	36.040	18.630	54.670	22.010	16.390	2.330
150	original	1	60x8 weeks	PCR90	M	VAC50	50	Unvaccinate	0.494	0.008	0.004	0.011	0.005	0.003	0.000	236.963	3.604	1.863	5.467	2.201	1.639	0.233	2370	36.040	18.630	54.670	22.010	16.390	2.330
150	original	1	60x8 weeks	PCR90	M	VAC50	50	Unvaccinate	0.494	0.008	0.004	0.011	0.005	0.003	0.000	236.963	3.604	1.863	5.467	2.201	1.639	0.233	2370	36.040	18.630	54.670	22.010	16.390	2.330
150	original	1.2	60x8 weeks	PCR90	M	VAC50	50	Unvaccinate	0.493	0.007	0.005	0.013	0.005	0.004	0.000	236.664	3.546	2.461	6.007	2.425	1.774	0.221	2367	35.460	24.610	60.070	24.250	17.740	2.210
150	original	1.2	60x8 weeks	PCR90	M	VAC50	50	Unvaccinate	0.493	0.007	0.005	0.013	0.005	0.004	0.000	236.664	3.546	2.461	6.007	2.425	1.774	0.221	2367	35.460	24.610	60.070	24.250	17.740	2.210
150	original	1.2	60x8 weeks	PCR90	M	VAC50	50	Unvaccinate	0.493	0.007	0.005	0.013	0.005	0.004	0.000	236.664	3.546	2.461	6.007	2.425	1.774	0.221	2367	35.460	24.610	60.070	24.250	17.740	2.210
150	alpha	0.6	60x8 weeks	PCR90	M	VAC50	50	Unvaccinate	0.491	0.007	0.008	0.015	0.007	0.005	0.000	235.456	3.529	3.714	7.243	3.368	2.302	0.239	2355	35.290	37.140	72.430	33.680	23.020	2.390
150	alpha	0.6	60x8 weeks	PCR90	M	VAC50	50	Unvaccinate	0.491	0.007	0.008	0.015	0.007	0.005	0.000	235.456	3.529	3.714	7.243	3.368	2.302	0.239	2355	35.290	37.140	72.430	33.680	23.020	2.390
150	alpha	0.6	60x8 weeks	PCR90	M	VAC50	50	Unvaccinate	0.491	0.007	0.008	0.015	0.007	0.005	0.000	235.456	3.529	3.714	7.243	3.368	2.302	0.239	2355	35.290	37.140	72.430	33.680	23.020	2.390
150	alpha	1	60x8 weeks	PCR90	M	VAC50	50	Unvaccinate	0.482	0.007	0.022	0.029	0.014	0.009	0.000	231.568	3.288	10.736	14.024	6.625	4.281	0.198	2316	32.880	107.360	140.240	66.250	42.810	1.980
150	alpha	1	60x8 weeks	PCR90	M	VAC50	50	Unvaccinate	0.482	0.007	0.022	0.029	0.014	0.009	0.000	231.568	3.288	10.736	14.024	6.625	4.281	0.198	2316	32.880	107.360	140.240	66.250	42.810	1.980
150	alpha	1	60x8 weeks	PCR90	M	VAC50	50	Unvaccinate	0.482	0.007	0.022	0.029	0.014	0.009	0.000	231.568	3.288	10.736	14.024	6.625	4.281	0.198	2316	32.880	107.360	140.240	66.250	42.810	1.980
150	alpha	1.2	60x8 weeks	PCR90	M	VAC50	50	Unvaccinate	0.474	0.007	0.036	0.043	0.021	0.013	0.000	227.622	3.127	17.389	20.516	9.898	6.303	0.187	2276	31.270	173.890	205.160	98.980	63.030	1.870
150	alpha	1.2	60x8 weeks	PCR90	M	VAC50	50	Unvaccinate	0.474	0.007	0.036	0.043	0.021	0.013	0.000	227.622	3.127	17.389	20.516	9.898	6.303	0.187	2276	31.270	173.890	205.160	98.980	63.030	1.870
150	alpha	1.2	60x8 weeks	PCR90	M	VAC50	50	Unvaccinate	0.474	0.007	0.036	0.043	0.021	0.013	0.000	227.622	3.127	17.389	20.516	9.898	6.303	0.187	2276	31.270	173.890	205.160	98.980	63.030	1.870
150	delta	0.6	60x8 weeks	PCR90	M	VAC50	50	Unvaccinate	0.467	0.006	0.053	0.059	0.031	0.015	0.000	224.059	3.093	25.227	28.320	15.078	7.274	0.178	2241	30.930	252.270	283.200	150.780	72.740	1.780
150	delta	0.6	60x8 weeks	PCR90	M	VAC50	50	Unvaccinate	0.467	0.006	0.053	0.059	0.031	0.015	0.000	224.059	3.093	25.227	28.320	15.078	7.274	0.178	2241	30.930	252.270	283.200	150.780	72.740	1.780
150	delta	0.6	60x8 weeks	PCR90	M	VAC50	50	Unvaccinate	0.467	0.006	0.053	0.059	0.031	0.015	0.000	224.059	3.093	25.227	28.320	15.078	7.274	0.178	2241	30.930	252.270	283.200	150.780	72.740	1.780
150	delta	1	60x8 weeks	PCR90	M	VAC50	50	Unvaccinate	0.439	0.005	0.102	0.107	0.059	0.026	0.000	210.544	2.209	48.960	51.169	28.499	12.690	0.127	2105	22.090	489.600	511.690	284.990	126.900	1.270
150	delta	1	60x8 weeks	PCR90	M	VAC50	50	Unvaccinate	0.439	0.005	0.102	0.107	0.059	0.026	0.000	210.544	2.209	48.960	51.169	28.499	12.690	0.127	2105	22.090	489.600	511.690	284.990	126.900	1.270
150	delta	1	60x8 weeks	PCR90	M	VAC50	50	Unvaccinate	0.439	0.005	0.102	0.107	0.059	0.026	0.000	210.544	2.209	48.960	51.169	28.499	12.690	0.127	2105	22.090	489.600	511.690	284.990	126.900	1.270
150	delta	1.2	60x8 weeks	PCR90	M	VAC50	50	Unvaccinate	0.432	0.004	0.111	0.115	0.065	0.029	0.000	207.558	1.953	53.142	55.095	31.142	13.734	0.140	2076	19.530	531.420	550.950	311.420	137.340	1.400
150	delta	1.2	60x8 weeks	PCR90	M	VAC50	50	Unvaccinate	0.432	0.004	0.111	0.115	0.065	0.029	0.000	207.558	1.953	53.142	55.095	31.142	13.734	0.140	2076	19.530	531.420	550.950	311.420	137.340	1.400
150	delta	1.2	60x8 weeks	PCR90	M	VAC50	50	Unvaccinate	0.432	0.004	0.111	0.115	0.065	0.029	0.000	207.558	1.953	53.142	55.095	31.142	13.734	0.140	2076	19.530	531.420	550.950	311.420	137.340	1.400
150	original	0.6	60x8 weeks	PCR90	M	VAC50	75	Unvaccinate	0.248	0.006	0.001	0.007	0.003	0.001	0.000	118.930	2.995	0.331	3.326	1.510	0.601	0.105	1189	29.950	3.310	33.260	15.100	6.010	1.050
150	original	0.6	60x8 weeks	PCR90	M	VAC50	75	Unvaccinate	0.248	0.006	0.001	0.007	0.003	0.001	0.000	118.930	2.995	0.331	3.326	1.510	0.601	0.105	1189	29.950	3.310	33.260	15.100	6.010	1.050
150	original	0.6	60x8 weeks	PCR90	M	VAC50	75	Unvaccinate	0.248	0.006	0.001	0.007	0.003	0.001	0.000	118.930	2.995	0.331	3.326	1.510	0.601	0.105	1189	29.950	3.310	33.260	15.100	6.010	1.050

150	original	1	60x8 weeks	PCR90	M	VAC50	75	Invaccinate	0.248	0.007	0.001	0.008	0.004	0.001	0.000	118.817	3.186	0.624	3.810	1.687	0.643	0.123	1188	31.860	6.240	38.100	16.870	6.430	1.230
150	original	1	60x8 weeks	PCR90	M	VAC50	75	Invaccinate	0.248	0.007	0.001	0.008	0.004	0.001	0.000	118.817	3.186	0.624	3.810	1.687	0.643	0.123	1188	31.860	6.240	38.100	16.870	6.430	1.230
150	original	1	60x8 weeks	PCR90	M	VAC50	75	Invaccinate	0.248	0.007	0.001	0.008	0.004	0.001	0.000	118.817	3.186	0.624	3.810	1.687	0.643	0.123	1188	31.860	6.240	38.100	16.870	6.430	1.230
150	original	1.2	60x8 weeks	PCR90	M	VAC50	75	Invaccinate	0.247	0.007	0.002	0.009	0.004	0.002	0.000	118.619	3.217	0.880	4.097	1.839	0.734	0.099	1186	32.170	8.800	40.970	18.390	7.340	0.990
150	original	1.2	60x8 weeks	PCR90	M	VAC50	75	Invaccinate	0.247	0.007	0.002	0.009	0.004	0.002	0.000	118.619	3.217	0.880	4.097	1.839	0.734	0.099	1186	32.170	8.800	40.970	18.390	7.340	0.990
150	original	1.2	60x8 weeks	PCR90	M	VAC50	75	Invaccinate	0.247	0.007	0.002	0.009	0.004	0.002	0.000	118.619	3.217	0.880	4.097	1.839	0.734	0.099	1186	32.170	8.800	40.970	18.390	7.340	0.990
150	alpha	0.6	60x8 weeks	PCR90	M	VAC50	75	Invaccinate	0.246	0.006	0.004	0.010	0.005	0.002	0.000	118.216	3.025	1.907	4.932	2.612	0.863	0.114	1182	30.250	19.070	49.320	26.120	8.630	1.140
150	alpha	0.6	60x8 weeks	PCR90	M	VAC50	75	Invaccinate	0.246	0.006	0.004	0.010	0.005	0.002	0.000	118.216	3.025	1.907	4.932	2.612	0.863	0.114	1182	30.250	19.070	49.320	26.120	8.630	1.140
150	alpha	0.6	60x8 weeks	PCR90	M	VAC50	75	Invaccinate	0.246	0.006	0.004	0.010	0.005	0.002	0.000	118.216	3.025	1.907	4.932	2.612	0.863	0.114	1182	30.250	19.070	49.320	26.120	8.630	1.140
150	alpha	1	60x8 weeks	PCR90	M	VAC50	75	Invaccinate	0.244	0.006	0.010	0.016	0.009	0.003	0.000	117.283	2.900	4.809	7.709	4.154	1.337	0.124	1173	29.000	48.090	77.090	41.540	13.370	1.240
150	alpha	1	60x8 weeks	PCR90	M	VAC50	75	Invaccinate	0.244	0.006	0.010	0.016	0.009	0.003	0.000	117.283	2.900	4.809	7.709	4.154	1.337	0.124	1173	29.000	48.090	77.090	41.540	13.370	1.240
150	alpha	1	60x8 weeks	PCR90	M	VAC50	75	Invaccinate	0.244	0.006	0.010	0.016	0.009	0.003	0.000	117.283	2.900	4.809	7.709	4.154	1.337	0.124	1173	29.000	48.090	77.090	41.540	13.370	1.240
150	alpha	1.2	60x8 weeks	PCR90	M	VAC50	75	Invaccinate	0.242	0.006	0.016	0.022	0.012	0.004	0.000	116.372	2.977	7.479	10.456	5.522	1.851	0.109	1164	29.770	74.790	104.560	55.220	18.510	1.090
150	alpha	1.2	60x8 weeks	PCR90	M	VAC50	75	Invaccinate	0.242	0.006	0.016	0.022	0.012	0.004	0.000	116.372	2.977	7.479	10.456	5.522	1.851	0.109	1164	29.770	74.790	104.560	55.220	18.510	1.090
150	alpha	1.2	60x8 weeks	PCR90	M	VAC50	75	Invaccinate	0.242	0.006	0.016	0.022	0.012	0.004	0.000	116.372	2.977	7.479	10.456	5.522	1.851	0.109	1164	29.770	74.790	104.560	55.220	18.510	1.090
150	delta	0.6	60x8 weeks	PCR90	M	VAC50	75	Invaccinate	0.239	0.007	0.028	0.035	0.020	0.005	0.000	114.930	3.233	13.490	16.723	9.613	2.286	0.098	1149	32.330	134.900	167.230	96.130	22.860	0.980
150	delta	0.6	60x8 weeks	PCR90	M	VAC50	75	Invaccinate	0.239	0.007	0.028	0.035	0.020	0.005	0.000	114.930	3.233	13.490	16.723	9.613	2.286	0.098	1149	32.330	134.900	167.230	96.130	22.860	0.980
150	delta	0.6	60x8 weeks	PCR90	M	VAC50	75	Invaccinate	0.239	0.007	0.028	0.035	0.020	0.005	0.000	114.930	3.233	13.490	16.723	9.613	2.286	0.098	1149	32.330	134.900	167.230	96.130	22.860	0.980
150	delta	1	60x8 weeks	PCR90	M	VAC50	75	Invaccinate	0.225	0.005	0.083	0.088	0.053	0.011	0.000	107.900	2.461	39.953	42.414	25.286	5.412	0.079	1079	24.610	399.530	424.140	252.860	54.120	0.790
150	delta	1	60x8 weeks	PCR90	M	VAC50	75	Invaccinate	0.225	0.005	0.083	0.088	0.053	0.011	0.000	107.900	2.461	39.953	42.414	25.286	5.412	0.079	1079	24.610	399.530	424.140	252.860	54.120	0.790
150	delta	1	60x8 weeks	PCR90	M	VAC50	75	Invaccinate	0.225	0.005	0.083	0.088	0.053	0.011	0.000	107.900	2.461	39.953	42.414	25.286	5.412	0.079	1079	24.610	399.530	424.140	252.860	54.120	0.790
150	delta	1.2	60x8 weeks	PCR90	M	VAC50	75	Invaccinate	0.221	0.005	0.099	0.103	0.063	0.013	0.000	105.844	2.172	47.431	49.603	30.205	6.270	0.072	1058	21.720	474.310	496.030	302.050	62.700	0.720
150	delta	1.2	60x8 weeks	PCR90	M	VAC50	75	Invaccinate	0.221	0.005	0.099	0.103	0.063	0.013	0.000	105.844	2.172	47.431	49.603	30.205	6.270	0.072	1058	21.720	474.310	496.030	302.050	62.700	0.720
150	delta	1.2	60x8 weeks	PCR90	M	VAC50	75	Invaccinate	0.221	0.005	0.099	0.103	0.063	0.013	0.000	105.844	2.172	47.431	49.603	30.205	6.270	0.072	1058	21.720	474.310	496.030	302.050	62.700	0.720
150	original	0.6	60x8 weeks	PCR90	M	VAC50	100	Invaccinate	0.000	0.005	0.000	0.005	0.003	0.000	0.000	0.000	2.576	0.047	2.623	1.330	0.000	0.000	0	25.760	0.470	26.230	13.300	0.000	0.000
150	original	0.6	60x8 weeks	PCR90	M	VAC50	100	Invaccinate	0.000	0.005	0.000	0.005	0.003	0.000	0.000	0.000	2.576	0.047	2.623	1.330	0.000	0.000	0	25.760	0.470	26.230	13.300	0.000	0.000
150	original	0.6	60x8 weeks	PCR90	M	VAC50	100	Invaccinate	0.000	0.005	0.000	0.005	0.003	0.000	0.000	0.000	2.576	0.047	2.623	1.330	0.000	0.000	0	25.760	0.470	26.230	13.300	0.000	0.000
150	original	1	60x8 weeks	PCR90	M	VAC50	100	Invaccinate	0.000	0.005	0.000	0.006	0.003	0.000	0.000	0.000	2.618	0.051	2.669	1.419	0.000	0.000	0	26.180	0.510	26.690	14.190	0.000	0.000
150	original	1	60x8 weeks	PCR90	M	VAC50	100	Invaccinate	0.000	0.005	0.000	0.006	0.003	0.000	0.000	0.000	2.618	0.051	2.669	1.419	0.000	0.000	0	26.180	0.510	26.690	14.190	0.000	0.000
150	original	1	60x8 weeks	PCR90	M	VAC50	100	Invaccinate	0.000	0.005	0.000	0.006	0.003	0.000	0.000	0.000	2.618	0.051	2.669	1.419	0.000	0.000	0	26.180	0.510	26.690	14.190	0.000	0.000
150	original	1.2	60x8 weeks	PCR90	M	VAC50	100	Invaccinate	0.000	0.005	0.000	0.005	0.003	0.000	0.000	0.000	2.538	0.074	2.612	1.343	0.000	0.000	0	25.380	0.740	26.120	13.430	0.000	0.000
150	original	1.2	60x8 weeks	PCR90	M	VAC50	100	Invaccinate	0.000	0.005	0.000	0.005	0.003	0.000	0.000	0.000	2.538	0.074	2.612	1.343	0.000	0.000	0	25.380	0.740	26.120	13.430	0.000	0.000
150	original	1.2	60x8 weeks	PCR90	M	VAC50	100	Invaccinate	0.000	0.005	0.000	0.005	0.003	0.000	0.000	0.000	2.538	0.074	2.612	1.343	0.000	0.000	0	25.380	0.740	26.120	13.430	0.000	0.000
150	alpha	0.6	60x8 weeks	PCR90	M	VAC50	100	Invaccinate	0.000	0.005	0.002	0.007	0.004	0.000	0.000	0.000	2.476	0.857	3.333	2.028	0.000	0.000	0	24.760	8.570	33.330	20.280	0.000	0.000
150	alpha	0.6	60x8 weeks	PCR90	M	VAC50	100	Invaccinate	0.000	0.005	0.002	0.007	0.004	0.000	0.000	0.000	2.476	0.857	3.333	2.028	0.000	0.000	0	24.760	8.570	33.330	20.280	0.000	0.000
150	alpha	0.6	60x8 weeks	PCR90	M	VAC50	100	Invaccinate	0.000	0.005	0.002	0.007	0.004	0.000	0.000	0.000	2.476	0.857	3.333	2.028	0.000	0.000	0	24.760	8.570	33.330	20.280	0.000	0.000
150	alpha	1	60x8 weeks	PCR90	M	VAC50	100	Invaccinate	0.000	0.005	0.004	0.009	0.006	0.000	0.000	0.000	2.567	1.883	4.450	2.699	0.000	0.000	0	25.670	18.830	44.500	26.990	0.000	0.000
150	alpha	1	60x8 weeks	PCR90	M	VAC50	100	Invaccinate	0.000	0.005	0.004	0.009	0.006	0.000	0.000	0.000	2.567	1.883	4.450	2.699	0.000	0.000	0	25.670	18.830	44.500	26.990	0.000	0.000
150	alpha	1	60x8 weeks	PCR90	M	VAC50	100	Invaccinate	0.000	0.005	0.004	0.009	0.006	0.000	0.000	0.000	2.567	1.883	4.450	2.699	0.000	0.000	0	25.670	18.830	44.500	26.990	0.000	0.000
150	alpha	1.2	60x8 weeks	PCR90	M	VAC50	100	Invaccinate	0.000	0.005	0.006	0.011	0.007	0.000	0.000	0.000	2.561	2.697	5.258	3.248	0.000	0.000	0	25.610	26.970	52.580	32.480	0.000	0.000
150	alpha	1.2	60x8 weeks	PCR90	M	VAC50	100	Invaccinate	0.000	0.005	0.006	0.011	0.007	0.000	0.000	0.000	2.561	2.697	5.258	3.248	0.000	0.000	0	25.610	26.970	52.580	32.480	0.000	0.000
150	alpha	1.2	60x8 weeks	PCR90	M	VAC50	100	Invaccinate	0.000	0.005	0.006	0.011	0.007	0.000	0.000	0.000	2.561	2.697	5.258	3.248	0.000	0.000	0	25.610	26.970	52.580	32.480	0.000	0.000
150	delta	0.6	60x8 weeks	PCR90	M	VAC50	100	Invaccinate	0.000	0.007	0.014	0.020	0.013	0.000	0.000	0.000	3.138	6.488	9.626	6.115	0.000	0.000	0	31.380	64.880	96.260	61.150	0.000	0.000
150	delta	0.6	60x8 weeks	PCR90	M	VAC50	100	Invaccinate	0.000	0.007	0.014	0.020	0.013	0.000	0.000	0.000	3.138	6.488	9.626	6.115	0.000	0.000	0	31.380	64.880	96.260	61.150	0.000	0.000
150	delta	0.6	60x8 weeks	PCR90	M	VAC50	100	Invaccinate	0.000	0.007	0.014	0.020	0.013	0.000	0.000	0.000	3.138	6.488	9.626	6.115	0.000	0.000	0	31.380	64.880	96.260	61.150	0.000	0.000
150	delta	1	60x8 weeks	PCR90	M	VAC50	100	Invaccinate	0.000	0.006	0.047	0.053	0.035	0.000	0.000	0.000	2.681	22.788	25.469	16.587	0.000	0.000	0	26.810	227.880	254.690	165.870	0.000	0.000
150	delta	1	60x8 weeks	PCR90	M	VAC50	100	Invaccinate	0.000	0.006	0.047	0.053	0.035	0.000	0.000	0.000	2.681	22.788	25.469	16.587	0.000	0.000	0	26.810	227.880	254.690	165.870	0.000	0.000
150	delta	1	60x8 weeks	PCR90	M	VAC50	100	Invaccinate	0.000	0.006	0.047	0.053	0.035	0.000	0.000	0.000	2.681	22.788	25.469	16.587	0.000	0.000	0	26.810	227.880	254.690	165.870	0.000	0.000
150	delta	1.2	60x8 weeks	PCR90	M	VAC50	100	Invaccinate	0.000	0.005	0.068	0.073	0.047	0.000	0.000	0.000	2.441	32.757	35.198	22.649	0.000	0.000	0	24.410	327.570	351.980	226.490	0.000	0.000
150	delta	1.2	60x8 weeks	PCR90	M	VAC50	100	Invaccinate	0.000	0.005	0.068	0.073	0.047	0.000	0.000	0.000	2.441	32.757	35.198	22.649	0.000	0.000	0	24.410	327.570	351.980	226.490	0.000	0.000
150	delta	1.2	60x8 weeks	PCR90	M	VAC50	100	Invaccinate	0.000	0.005	0.068	0.073	0.047	0.000	0.000	0.000	2.441	32.757	35.198	22.649	0.000	0.000	0	24.410	327.570	351.980	226.490	0.000	0.000
150	original	0.6	60x8 weeks	Ag80	Daily	NOVAC	0	Invaccinate	4.922	0.010	0.001	0.011	0.000	0.011	0.095	2362.738	4.852	0.636	5.488	0.142	5.479	45.762	23627	48.520	6.360	54.880	1.420	54.790	457.620
150	original	0.6	60x8 weeks	Ag80	Daily	NOVAC	0	Invaccinate	4.922	0.010	0.001	0.011	0.000	0.011	0.095	2362.738	4.852	0.636	5.488	0.142	5.479	45.762	23627	48.520	6.360	54.880	1.420	54.790	457.620
150	original	0.6	60x8 weeks	Ag80	Daily	NOVAC	0	Invaccinate	4.922	0.010	0.001	0.011	0.000	0.011	0.095	2362.738	4.852	0.636	5.488	0.142	5.479	45.762	23627	48.520	6.360	54.880	1.420	54.790	457.620
150	original	1	60x8 weeks	Ag80	Daily	NOVAC	0	Invaccinate	4.918	0.010	0.002	0.012	0.000	0.012	0.096	2360.449	4.725	1.082	5.807	0.147	5.823	45.847	23604	47.250	10.820	58.070	1.470	58.230	458.470
150	original	1	60x8 weeks	Ag80	Daily	NOVAC	0	Invaccinate	4.918	0.010	0.002	0.012	0.000	0.012	0.096	2360.449	4.725	1.082	5.807	0.147	5.823	45.847	23604	47.250	10.820	58.070	1.470	58.230	458.470
150	original	1	60x8 weeks	Ag80	Daily	NOVAC	0	Invaccinate	4.918	0.010	0.002	0.012	0.000	0.012	0.096	2360.449	4.725	1.082	5.807	0.147	5.823	45.847	23604	47.250	10.820	58.070	1.470	58.230	458.470
150	original	1.2	60x8 weeks	Ag80	Daily	NOVAC	0	Invaccinate	4.912	0.010	0.003	0.013	0.000	0.013	0.094	2357.654	4.737	1.507	6.244	0.160	6.252	45.337	23577	47.370	15.070	62.440	1.600	62.520	453.370
150	original	1.2	60x8 weeks	Ag80	Daily	NOVAC	0	Invaccinate	4.912	0.010	0.003	0.013	0.000	0.013	0.094	2357.654	4.737	1.507	6.244	0.160	6.252	45.337	23577	47.370	15.070	62.440	1.600	62.520	453.370
150	original	1.2	60x8 weeks	Ag80	Daily	NOVAC	0	Invaccinate	4.912	0.010	0.003	0.013	0.000	0.013	0.094	2357.654	4.737	1.507	6.244	0.160	6.252	45.337	23577	47.370	15.070	62.440	1.600	62.520	453.370
150	alpha	0.6	60x8 weeks	Ag80	Daily	NOVAC	0	Invaccinate	4.913	0.010	0.002	0.012	0.000	0.013	0.095	2358.405	4.867	1.106	5.973	0.146	6.159	45.799	23584	48.670	11.060	59.730	1.460	61.590	457.990
150	alpha	0.6	60x8 weeks	Ag80	Daily	NOVAC	0	Invaccinate	4.913	0.010	0.002	0.012	0.000	0.013	0.095	2358.405	4.867	1.106	5.973	0.146	6.159	45.799	23584	48.670	11.060	59.730	1.460	61.590	457.990
150	alpha	0.6	60x8 weeks	Ag80	Daily	NOVAC	0	Invaccinate	4.913	0.010	0.002	0.012	0.000	0.013	0.095	2358.405	4.867	1.106	5.973	0.146	6.159	45.799	23584	48.670	11.060	59.730	1.460	61.590	457.990
150	alpha	1	60x8 weeks	Ag80	Daily	NOVAC	0	Invaccinate	4.899	0.010	0.004	0.014	0.000	0.015	0.095	2351.287	4.829	2.003	6.832	0.149	7.213	45.397	23513	48.290	20.030	68.320	1.490	72.130	453.970
150	alpha	1	60x8 weeks	Ag80	Daily	NOVAC	0	Invaccinate	4.899	0.010	0.004	0.014	0.000	0.015	0.095	2351.287	4.829	2.003	6.832	0.149	7.213	45.397	23513	48.290	20.030	68.320	1.490	72.130	453.970
150	alpha	1	60x8 weeks	Ag80	Daily	NOVAC	0	Invaccinate	4.899	0.010	0.004	0.014	0.000	0.015	0.095	2351.287	4.829	2.003	6.832	0.149	7.213	45.397	23513	48.290	20.030	68.320	1.490	72.130	453.970
150	alpha	1.2	60x8 weeks	Ag80	Daily	NOVAC	0	Invaccinate	4.891	0.010	0.006	0.015	0.000	0.016	0.094	2347.786	4.621	2.677	7.298	0.182	7.660	45.197	23478	46.210	26.770	72.980	1.820	76.600	451.970
150	alpha	1.2	60x8 weeks	Ag80	Daily	NOVAC	0	Invaccinate	4.891	0.010	0.006	0.015	0.000	0.016	0.094	2347.786	4.621	2.677	7.298	0.182	7.660	45.197	23478	46.210	26.770	72.980	1.820	76.600	451.970
150	alpha	1.2	60x8 weeks	Ag80	Daily	NOVAC	0	Invaccinate	4.891	0.010	0.006	0.015	0.000	0.016	0.094	2347.786	4.621	2.677	7.298	0.182	7.660	45.197	23478	46.210	26.770	72.980	1.820	76.600	451.970
150	delta	0.6	60x8 weeks	Ag80	Daily	NOVAC	0	Invaccinate	4.888	0.010	0.006	0.016	0.000	0.016	0.094	2346.135	4.788	2.717	7.505	0.214	7.783	45.143	23461	47.880	27.170	75.050	2.140	77.830	451.430
150	delta	0.6	60x8 weeks	Ag80	Daily	NOVAC	0	Invaccinate	4.888	0.010	0.006	0.016	0.000	0.016	0.094	2346.135	4.788	2.717	7.505	0.214	7.783	45.143	23461	47.880	27.170	75.050	2.140	77.830	451.430

150	delta	0.6	60x8 weeks	Ag80	Daily	NOVAC	0	Unvaccinate	4.888	0.010	0.006	0.016	0.000	0.016	0.094	2346.135	4.788	2.717	7.505	0.214	7.783	45.143	23461	47.880	27.170	75.050	2.140	77.830	451.430
150	delta	1	60x8 weeks	Ag80	Daily	NOVAC	0	Unvaccinate	4.849	0.010	0.011	0.021	0.001	0.022	0.092	2327.556	4.599	5.385	9.984	0.274	10.497	44.108	23276	45.990	53.850	99.840	2.740	104.970	441.080
150	delta	1	60x8 weeks	Ag80	Daily	NOVAC	0	Unvaccinate	4.849	0.010	0.011	0.021	0.001	0.022	0.092	2327.556	4.599	5.385	9.984	0.274	10.497	44.108	23276	45.990	53.850	99.840	2.740	104.970	441.080
150	delta	1	60x8 weeks	Ag80	Daily	NOVAC	0	Unvaccinate	4.849	0.010	0.011	0.021	0.001	0.022	0.092	2327.556	4.599	5.385	9.984	0.274	10.497	44.108	23276	45.990	53.850	99.840	2.740	104.970	441.080
150	delta	1.2	60x8 weeks	Ag80	Daily	NOVAC	0	Unvaccinate	4.807	0.010	0.017	0.026	0.001	0.028	0.090	2307.555	4.641	7.922	12.563	0.323	13.249	43.010	23076	46.410	79.220	125.630	3.230	132.490	430.100
150	delta	1.2	60x8 weeks	Ag80	Daily	NOVAC	0	Unvaccinate	4.807	0.010	0.017	0.026	0.001	0.028	0.090	2307.555	4.641	7.922	12.563	0.323	13.249	43.010	23076	46.410	79.220	125.630	3.230	132.490	430.100
150	delta	1.2	60x8 weeks	Ag80	Daily	NOVAC	0	Unvaccinate	4.807	0.010	0.017	0.026	0.001	0.028	0.090	2307.555	4.641	7.922	12.563	0.323	13.249	43.010	23076	46.410	79.220	125.630	3.230	132.490	430.100
150	original	0.6	60x8 weeks	Ag80	Daily	VAC95	25	Unvaccinate	3.693	0.008	0.001	0.009	0.000	0.008	0.071	1772.566	3.730	0.420	4.150	0.159	4.042	34.263	17726	37.300	4.200	41.500	1.590	40.420	342.630
150	original	0.6	60x8 weeks	Ag80	Daily	VAC95	25	Unvaccinate	3.693	0.008	0.001	0.009	0.000	0.008	0.071	1772.566	3.730	0.420	4.150	0.159	4.042	34.263	17726	37.300	4.200	41.500	1.590	40.420	342.630
150	original	0.6	60x8 weeks	Ag80	Daily	VAC95	25	Unvaccinate	3.693	0.008	0.001	0.009	0.000	0.008	0.071	1772.566	3.730	0.420	4.150	0.159	4.042	34.263	17726	37.300	4.200	41.500	1.590	40.420	342.630
150	original	1	60x8 weeks	Ag80	Daily	VAC95	25	Unvaccinate	3.688	0.008	0.001	0.009	0.000	0.009	0.071	1770.360	3.783	0.647	4.430	0.130	4.374	34.071	17704	37.830	6.470	44.300	1.300	43.740	340.710
150	original	1	60x8 weeks	Ag80	Daily	VAC95	25	Unvaccinate	3.688	0.008	0.001	0.009	0.000	0.009	0.071	1770.360	3.783	0.647	4.430	0.130	4.374	34.071	17704	37.830	6.470	44.300	1.300	43.740	340.710
150	original	1	60x8 weeks	Ag80	Daily	VAC95	25	Unvaccinate	3.688	0.008	0.001	0.009	0.000	0.009	0.071	1770.360	3.783	0.647	4.430	0.130	4.374	34.071	17704	37.830	6.470	44.300	1.300	43.740	340.710
150	original	1.2	60x8 weeks	Ag80	Daily	VAC95	25	Unvaccinate	3.687	0.008	0.002	0.009	0.000	0.009	0.071	1769.656	3.685	0.868	4.553	0.162	4.474	34.312	17697	36.850	8.680	45.530	1.620	44.740	343.120
150	original	1.2	60x8 weeks	Ag80	Daily	VAC95	25	Unvaccinate	3.687	0.008	0.002	0.009	0.000	0.009	0.071	1769.656	3.685	0.868	4.553	0.162	4.474	34.312	17697	36.850	8.680	45.530	1.620	44.740	343.120
150	original	1.2	60x8 weeks	Ag80	Daily	VAC95	25	Unvaccinate	3.687	0.008	0.002	0.009	0.000	0.009	0.071	1769.656	3.685	0.868	4.553	0.162	4.474	34.312	17697	36.850	8.680	45.530	1.620	44.740	343.120
150	alpha	0.6	60x8 weeks	Ag80	Daily	VAC95	25	Unvaccinate	3.688	0.008	0.001	0.009	0.000	0.009	0.072	1770.048	3.712	0.677	4.389	0.173	4.406	34.437	17700	37.120	6.770	43.890	1.730	44.060	344.370
150	alpha	0.6	60x8 weeks	Ag80	Daily	VAC95	25	Unvaccinate	3.688	0.008	0.001	0.009	0.000	0.009	0.072	1770.048	3.712	0.677	4.389	0.173	4.406	34.437	17700	37.120	6.770	43.890	1.730	44.060	344.370
150	alpha	0.6	60x8 weeks	Ag80	Daily	VAC95	25	Unvaccinate	3.688	0.008	0.001	0.009	0.000	0.009	0.072	1770.048	3.712	0.677	4.389	0.173	4.406	34.437	17700	37.120	6.770	43.890	1.730	44.060	344.370
150	alpha	1	60x8 weeks	Ag80	Daily	VAC95	25	Unvaccinate	3.680	0.008	0.003	0.010	0.000	0.010	0.071	1766.198	3.682	1.271	4.953	0.180	4.975	34.198	17662	36.820	12.710	49.530	1.800	49.750	341.980
150	alpha	1	60x8 weeks	Ag80	Daily	VAC95	25	Unvaccinate	3.680	0.008	0.003	0.010	0.000	0.010	0.071	1766.198	3.682	1.271	4.953	0.180	4.975	34.198	17662	36.820	12.710	49.530	1.800	49.750	341.980
150	alpha	1	60x8 weeks	Ag80	Daily	VAC95	25	Unvaccinate	3.680	0.008	0.003	0.010	0.000	0.010	0.071	1766.198	3.682	1.271	4.953	0.180	4.975	34.198	17662	36.820	12.710	49.530	1.800	49.750	341.980
150	alpha	1.2	60x8 weeks	Ag80	Daily	VAC95	25	Unvaccinate	3.675	0.008	0.003	0.011	0.000	0.011	0.071	1763.868	3.689	1.548	5.237	0.206	5.276	34.226	17639	36.890	15.480	52.370	2.060	52.760	342.260
150	alpha	1.2	60x8 weeks	Ag80	Daily	VAC95	25	Unvaccinate	3.675	0.008	0.003	0.011	0.000	0.011	0.071	1763.868	3.689	1.548	5.237	0.206	5.276	34.226	17639	36.890	15.480	52.370	2.060	52.760	342.260
150	alpha	1.2	60x8 weeks	Ag80	Daily	VAC95	25	Unvaccinate	3.675	0.008	0.003	0.011	0.000	0.011	0.071	1763.868	3.689	1.548	5.237	0.206	5.276	34.226	17639	36.890	15.480	52.370	2.060	52.760	342.260
150	delta	0.6	60x8 weeks	Ag80	Daily	VAC95	25	Unvaccinate	3.671	0.008	0.004	0.012	0.001	0.012	0.071	1762.114	3.817	1.770	5.587	0.278	5.535	33.937	17621	38.170	17.700	55.870	2.780	55.350	339.370
150	delta	0.6	60x8 weeks	Ag80	Daily	VAC95	25	Unvaccinate	3.671	0.008	0.004	0.012	0.001	0.012	0.071	1762.114	3.817	1.770	5.587	0.278	5.535	33.937	17621	38.170	17.700	55.870	2.780	55.350	339.370
150	delta	0.6	60x8 weeks	Ag80	Daily	VAC95	25	Unvaccinate	3.671	0.008	0.004	0.012	0.001	0.012	0.071	1762.114	3.817	1.770	5.587	0.278	5.535	33.937	17621	38.170	17.700	55.870	2.780	55.350	339.370
150	delta	1	60x8 weeks	Ag80	Daily	VAC95	25	Unvaccinate	3.644	0.008	0.008	0.015	0.001	0.015	0.069	1749.139	3.611	3.725	7.336	0.407	7.294	33.196	17491	36.110	37.250	73.360	4.070	72.940	331.960
150	delta	1	60x8 weeks	Ag80	Daily	VAC95	25	Unvaccinate	3.644	0.008	0.008	0.015	0.001	0.015	0.069	1749.139	3.611	3.725	7.336	0.407	7.294	33.196	17491	36.110	37.250	73.360	4.070	72.940	331.960
150	delta	1	60x8 weeks	Ag80	Daily	VAC95	25	Unvaccinate	3.644	0.008	0.008	0.015	0.001	0.015	0.069	1749.139	3.611	3.725	7.336	0.407	7.294	33.196	17491	36.110	37.250	73.360	4.070	72.940	331.960
150	delta	1.2	60x8 weeks	Ag80	Daily	VAC95	25	Unvaccinate	3.629	0.007	0.010	0.018	0.001	0.017	0.068	1742.058	3.594	4.918	8.512	0.451	8.384	32.820	17421	35.940	49.180	85.120	4.510	83.840	328.200
150	delta	1.2	60x8 weeks	Ag80	Daily	VAC95	25	Unvaccinate	3.629	0.007	0.010	0.018	0.001	0.017	0.068	1742.058	3.594	4.918	8.512	0.451	8.384	32.820	17421	35.940	49.180	85.120	4.510	83.840	328.200
150	delta	1.2	60x8 weeks	Ag80	Daily	VAC95	25	Unvaccinate	3.629	0.007	0.010	0.018	0.001	0.017	0.068	1742.058	3.594	4.918	8.512	0.451	8.384	32.820	17421	35.940	49.180	85.120	4.510	83.840	328.200
150	original	0.6	60x8 weeks	Ag80	Daily	VAC95	50	Unvaccinate	2.463	0.005	0.000	0.006	0.000	0.005	0.048	1182.458	2.619	0.179	2.798	0.164	2.584	23.120	11825	26.190	1.790	27.980	1.640	25.840	231.200
150	original	0.6	60x8 weeks	Ag80	Daily	VAC95	50	Unvaccinate	2.463	0.005	0.000	0.006	0.000	0.005	0.048	1182.458	2.619	0.179	2.798	0.164	2.584	23.120	11825	26.190	1.790	27.980	1.640	25.840	231.200
150	original	0.6	60x8 weeks	Ag80	Daily	VAC95	50	Unvaccinate	2.463	0.005	0.000	0.006	0.000	0.005	0.048	1182.458	2.619	0.179	2.798	0.164	2.584	23.120	11825	26.190	1.790	27.980	1.640	25.840	231.200
150	original	1	60x8 weeks	Ag80	Daily	VAC95	50	Unvaccinate	2.462	0.005	0.001	0.006	0.000	0.006	0.048	1181.692	2.625	0.244	2.869	0.140	2.703	22.940	11817	26.250	2.440	28.690	1.400	27.030	229.400
150	original	1	60x8 weeks	Ag80	Daily	VAC95	50	Unvaccinate	2.462	0.005	0.001	0.006	0.000	0.006	0.048	1181.692	2.625	0.244	2.869	0.140	2.703	22.940	11817	26.250	2.440	28.690	1.400	27.030	229.400
150	original	1	60x8 weeks	Ag80	Daily	VAC95	50	Unvaccinate	2.462	0.005	0.001	0.006	0.000	0.006	0.048	1181.692	2.625	0.244	2.869	0.140	2.703	22.940	11817	26.250	2.440	28.690	1.400	27.030	229.400
150	original	1.2	60x8 weeks	Ag80	Daily	VAC95	50	Unvaccinate	2.461	0.005	0.001	0.006	0.000	0.006	0.048	1181.066	2.576	0.376	2.952	0.125	2.769	23.102	11811	25.760	3.760	29.520	1.250	27.690	231.020
150	original	1.2	60x8 weeks	Ag80	Daily	VAC95	50	Unvaccinate	2.461	0.005	0.001	0.006	0.000	0.006	0.048	1181.066	2.576	0.376	2.952	0.125	2.769	23.102	11811	25.760	3.760	29.520	1.250	27.690	231.020
150	original	1.2	60x8 weeks	Ag80	Daily	VAC95	50	Unvaccinate	2.461	0.005	0.001	0.006	0.000	0.006	0.048	1181.066	2.576	0.376	2.952	0.125	2.769	23.102	11811	25.760	3.760	29.520	1.250	27.690	231.020
150	alpha	0.6	60x8 weeks	Ag80	Daily	VAC95	50	Unvaccinate	2.458	0.006	0.001	0.006	0.000	0.006	0.048	1180.016	2.727	0.387	3.114	0.213	2.946	22.831	11800	27.270	3.870	31.140	2.130	29.460	228.310
150	alpha	0.6	60x8 weeks	Ag80	Daily	VAC95	50	Unvaccinate	2.458	0.006	0.001	0.006	0.000	0.006	0.048	1180.016	2.727	0.387	3.114	0.213	2.946	22.831	11800	27.270	3.870	31.140	2.130	29.460	228.310
150	alpha	0.6	60x8 weeks	Ag80	Daily	VAC95	50	Unvaccinate	2.458	0.006	0.001	0.006	0.000	0.006	0.048	1180.016	2.727	0.387	3.114	0.213	2.946	22.831	11800	27.270	3.870	31.140	2.130	29.460	228.310
150	alpha	1	60x8 weeks	Ag80	Daily	VAC95	50	Unvaccinate	2.457	0.005	0.001	0.007	0.000	0.006	0.047	1179.292	2.546	0.620	3.166	0.232	3.023	22.608	11793	25.460	6.200	31.660	2.320	30.230	226.080
150	alpha	1	60x8 weeks	Ag80	Daily	VAC95	50	Unvaccinate	2.457	0.005	0.001	0.007	0.000	0.006	0.047	1179.292	2.546	0.620	3.166	0.232	3.023	22.608	11793	25.460	6.200	31.660	2.320	30.230	226.080
150	alpha	1	60x8 weeks	Ag80	Daily	VAC95	50	Unvaccinate	2.457	0.005	0.001	0.007	0.000	0.006	0.047	1179.292	2.546	0.620	3.166	0.232	3.023	22.608	11793	25.460	6.200	31.660	2.320	30.230	226.080
150	alpha	1.2	60x8 weeks	Ag80	Daily	VAC95	50	Unvaccinate	2.454	0.005	0.002	0.007	0.000	0.007	0.047	1177.787	2.638	0.815	3.453	0.223	3.280	22.733	11778	26.380	8.150	34.530	2.230	32.800	227.330
150	alpha	1.2	60x8 weeks	Ag80	Daily	VAC95	50	Unvaccinate	2.454	0.005	0.002	0.007	0.000	0.007	0.047	1177.787	2.638	0.815	3.453	0.223	3.280	22.733	11778	26.380	8.150	34.530	2.230	32.800	227.330
150	alpha	1.2	60x8 weeks	Ag80	Daily	VAC95	50	Unvaccinate	2.454	0.005	0.002	0.007	0.000	0.007	0.047	1177.787	2.638	0.815	3.453	0.223	3.280	22.733	11778	26.380	8.150	34.530	2.230	32.800	227.330
150	delta	0.6	60x8 weeks	Ag80	Daily	VAC95	50	Unvaccinate	2.454	0.006	0.002	0.007	0.001	0.007	0.048	1177.828	2.663	0.918	3.581	0.379	3.212	23.127	11778	26.630	9.180	35.810	3.790	32.120	231.270
150	delta	0.6	60x8 weeks	Ag80	Daily	VAC95	50	Unvaccinate	2.454	0.006	0.002	0.007	0.001	0.007	0.048	1177.828	2.663	0.918	3.581	0.379	3.212	23.127	11778	26.630	9.180	35.810	3.790	32.120	231.270
150	delta	0.6	60x8 weeks	Ag80	Daily	VAC95	50	Unvaccinate	2.454	0.006	0.002	0.007	0.001	0.007	0.048	1177.828	2.663	0.918	3.581	0.379	3.212	23.127	11778	26.630	9.180	35.810	3.790	32.120	231.270
150	delta	1	60x8 weeks	Ag80	Daily	VAC95	50	Unvaccinate	2.438	0.006	0.004	0.010	0.001	0.009	0.047	1170.241	2.680	2.132	4.812	0.484	4.311	22.412	11702	26.800	21.320	48.120	4.840	43.110	224.120
150	delta	1	60x8 weeks	Ag80	Daily	VAC95	50	Unvaccinate	2.438	0.006	0.004	0.010	0.001	0.009	0.047	1170.241	2.680	2.132	4.812	0.484	4.311	22.412	11702	26.800	21.320	48.120	4.840	43.110	224.120
150	delta	1	60x8 weeks	Ag80	Daily	VAC95	50	Unvaccinate	2.438	0.006	0.004	0.010	0.001	0.009	0.047	1170.241	2.680	2.132	4.812	0.484	4.311	22.412	11702	26.800	21.320	48.120	4.840	43.110	224.120
150	delta	1.2	60x8 weeks	Ag80	Daily	VAC95	50	Unvaccinate	2.428	0.006	0.006	0.012	0.001	0.011	0.046	1165.222	2.770	2.953	5.723	0.581	5.123	22.103	11652	27.700	29.530	57.230	5.810	51.230	221.030
150	delta	1.2	60x8 weeks	Ag80	Daily	VAC95	50	Unvaccinate	2.428	0.006	0.006	0.012	0.001	0.011	0.046	1165.222	2.770	2.953	5.723	0.581	5.123	22.103	11652	27.700	29.530	57.230	5.810	51.230	221.030
150	delta	1.2	60x8 weeks	Ag80	Daily	VAC95	50	Unvaccinate	2.428	0.006	0.006	0.012	0.001	0.011	0.046	1165.222	2.770	2.953	5.723	0.581	5.123	22.103	11652	27.700	29.530	57.230	5.810	51.230	221.030
150	original	0.6	60x8 weeks	Ag80	Daily	VAC95	75	Unvaccinate	1.232	0.003	0.000	0.003	0.000	0.003	0.024	591.308	1.493	0.053	1.546	0.147	1.284	11.522	5913	14.930	0.530	15.460	1.470	12.840	115.220
150	original	0.6	60x8 weeks	Ag80	Daily	VAC95	75	Unvaccinate	1.232	0.003	0.000	0.003	0.000	0.003	0.024	591.308	1.493	0.053	1.546	0.147	1.284	11.522	5913	14.930	0.530	15.460	1.470	12.840	115.220
150	original	0.6	60x8 weeks	Ag80	Daily	VAC95	75	Unvaccinate	1.232	0.003	0.000	0.003	0.000	0.003	0.024	591.308	1.493	0.053	1.546	0.147	1.284	11.522	5913	14.930	0.530	15.460	1.470	12.840	115.220
150	original	1	60x8 weeks	Ag80	Daily	VAC95	75	Unvaccinate	1.233	0.003	0.000	0.003	0.000	0.003	0.024	591.750	1.464	0.067	1.531	0.129	1.232	11.619	5918	14.640	0.670	15.310	1.290	12.320	116.190
150	original	1	60x8 weeks	Ag80	Daily	VAC95	75	Unvaccinate	1.233	0.003	0.000	0.003	0.000	0.003	0.024	591.750	1.464	0.067	1.531	0.129	1.232	11.619	5918	14.640	0.670	15.310	1.290	12.320	116.190
150	original	1	60x8 weeks	Ag80	Daily	VAC95	75	Unvaccinate	1.233	0.003	0.000	0.003	0.000	0.003	0.024	591.750	1.464	0.067	1.531	0.129	1.232	11.619	5918	14.640	0.670	15.310	1.290	12.320	116.190
150	original	1.2	60x8 weeks	Ag80	Daily	VAC95	75	Unvaccinate	1.232	0.003	0.000	0.003	0.000	0.003	0.024	591.324	1.509	0.089	1.598	0.155	1.293	11.424	5913	15.090	0.890	15.980	1.550	12.930	114.240
150	original	1.2	60x8 weeks	Ag80	Daily	VAC95	75	Unvaccinate	1.232	0.003	0.000	0.003	0.000	0.003	0.024	591.324	1.509	0.089	1.598	0.155	1.293	11.424	5913	15.090	0.890	15.980	1.550	12.930	114.240
150	original	1.2	60x8 weeks	Ag80	Daily	VAC95	75	Unvaccinate	1.232	0.003	0.000	0.003	0.000	0.003	0.024	591.324	1.509	0.089	1.598	0.155	1.293	11.424	5913	15.090	0.890	15.980	1.550	12.930	114.240
150	alpha	0.6	60x8 weeks	Ag80	Daily	VAC95	75	Unvaccinate	1.230	0.003	0.000	0.004	0.000	0.003	0.023	590.514	1.598	0.140	1.738	0.216	1.418	11.273	5905	15.980	1.400	17.380	2.160	14.180	112.730

150	alpha	0.6	60x8 weeks	Ag80	Daily	VAC95	75	Unvaccinate	1.230	0.003	0.000	0.004	0.000	0.003	0.023	590.514	1.598	0.140	1.738	0.216	1.418	11.273	5905	15.980	1.400	17.380	2.160	14.180	112.730
150	alpha	0.6	60x8 weeks	Ag80	Daily	VAC95	75	Unvaccinate	1.230	0.003	0.000	0.004	0.000	0.003	0.023	590.514	1.598	0.140	1.738	0.216	1.418	11.273	5905	15.980	1.400	17.380	2.160	14.180	112.730
150	alpha	1	60x8 weeks	Ag80	Daily	VAC95	75	Unvaccinate	1.229	0.003	0.001	0.004	0.000	0.003	0.024	589.957	1.553	0.262	1.815	0.215	1.466	11.461	5900	15.530	2.620	18.150	2.150	14.660	114.610
150	alpha	1	60x8 weeks	Ag80	Daily	VAC95	75	Unvaccinate	1.229	0.003	0.001	0.004	0.000	0.003	0.024	589.957	1.553	0.262	1.815	0.215	1.466	11.461	5900	15.530	2.620	18.150	2.150	14.660	114.610
150	alpha	1	60x8 weeks	Ag80	Daily	VAC95	75	Unvaccinate	1.229	0.003	0.001	0.004	0.000	0.003	0.024	589.957	1.553	0.262	1.815	0.215	1.466	11.461	5900	15.530	2.620	18.150	2.150	14.660	114.610
150	alpha	1.2	60x8 weeks	Ag80	Daily	VAC95	75	Unvaccinate	1.229	0.003	0.001	0.004	0.001	0.003	0.024	590.032	1.526	0.292	1.818	0.242	1.477	11.322	5900	15.260	2.920	18.180	2.420	14.770	113.220
150	alpha	1.2	60x8 weeks	Ag80	Daily	VAC95	75	Unvaccinate	1.229	0.003	0.001	0.004	0.001	0.003	0.024	590.032	1.526	0.292	1.818	0.242	1.477	11.322	5900	15.260	2.920	18.180	2.420	14.770	113.220
150	alpha	1.2	60x8 weeks	Ag80	Daily	VAC95	75	Unvaccinate	1.229	0.003	0.001	0.004	0.001	0.003	0.024	590.032	1.526	0.292	1.818	0.242	1.477	11.322	5900	15.260	2.920	18.180	2.420	14.770	113.220
150	delta	0.6	60x8 weeks	Ag80	Daily	VAC95	75	Unvaccinate	1.228	0.004	0.001	0.005	0.001	0.003	0.024	589.305	1.769	0.514	2.283	0.454	1.559	11.438	5893	17.690	5.140	22.830	4.540	15.590	114.380
150	delta	0.6	60x8 weeks	Ag80	Daily	VAC95	75	Unvaccinate	1.228	0.004	0.001	0.005	0.001	0.003	0.024	589.305	1.769	0.514	2.283	0.454	1.559	11.438	5893	17.690	5.140	22.830	4.540	15.590	114.380
150	delta	0.6	60x8 weeks	Ag80	Daily	VAC95	75	Unvaccinate	1.228	0.004	0.001	0.005	0.001	0.003	0.024	589.305	1.769	0.514	2.283	0.454	1.559	11.438	5893	17.690	5.140	22.830	4.540	15.590	114.380
150	delta	1	60x8 weeks	Ag80	Daily	VAC95	75	Unvaccinate	1.224	0.003	0.002	0.005	0.001	0.004	0.023	587.587	1.672	0.862	2.534	0.485	1.800	11.258	5876	16.720	8.620	25.340	4.850	18.000	112.580
150	delta	1	60x8 weeks	Ag80	Daily	VAC95	75	Unvaccinate	1.224	0.003	0.002	0.005	0.001	0.004	0.023	587.587	1.672	0.862	2.534	0.485	1.800	11.258	5876	16.720	8.620	25.340	4.850	18.000	112.580
150	delta	1	60x8 weeks	Ag80	Daily	VAC95	75	Unvaccinate	1.224	0.003	0.002	0.005	0.001	0.004	0.023	587.587	1.672	0.862	2.534	0.485	1.800	11.258	5876	16.720	8.620	25.340	4.850	18.000	112.580
150	delta	1.2	60x8 weeks	Ag80	Daily	VAC95	75	Unvaccinate	1.221	0.003	0.003	0.006	0.001	0.004	0.023	585.958	1.668	1.202	2.870	0.564	2.030	11.173	5860	16.680	12.020	28.700	5.640	20.300	111.730
150	delta	1.2	60x8 weeks	Ag80	Daily	VAC95	75	Unvaccinate	1.221	0.003	0.003	0.006	0.001	0.004	0.023	585.958	1.668	1.202	2.870	0.564	2.030	11.173	5860	16.680	12.020	28.700	5.640	20.300	111.730
150	delta	1.2	60x8 weeks	Ag80	Daily	VAC95	75	Unvaccinate	1.221	0.003	0.003	0.006	0.001	0.004	0.023	585.958	1.668	1.202	2.870	0.564	2.030	11.173	5860	16.680	12.020	28.700	5.640	20.300	111.730
150	original	0.6	60x8 weeks	Ag80	Daily	VAC95	100	Unvaccinate	0.000	0.001	0.000	0.001	0.000	0.000	0.000	0.000	0.391	0.000	0.391	0.174	0.000	0.000	0	3.910	0.000	3.910	1.740	0.000	0.000
150	original	0.6	60x8 weeks	Ag80	Daily	VAC95	100	Unvaccinate	0.000	0.001	0.000	0.001	0.000	0.000	0.000	0.000	0.391	0.000	0.391	0.174	0.000	0.000	0	3.910	0.000	3.910	1.740	0.000	0.000
150	original	0.6	60x8 weeks	Ag80	Daily	VAC95	100	Unvaccinate	0.000	0.001	0.000	0.001	0.000	0.000	0.000	0.000	0.391	0.000	0.391	0.174	0.000	0.000	0	3.910	0.000	3.910	1.740	0.000	0.000
150	original	1	60x8 weeks	Ag80	Daily	VAC95	100	Unvaccinate	0.000	0.001	0.000	0.001	0.000	0.000	0.000	0.000	0.343	0.001	0.344	0.119	0.000	0.000	0	3.430	0.010	3.440	1.190	0.000	0.000
150	original	1	60x8 weeks	Ag80	Daily	VAC95	100	Unvaccinate	0.000	0.001	0.000	0.001	0.000	0.000	0.000	0.000	0.343	0.001	0.344	0.119	0.000	0.000	0	3.430	0.010	3.440	1.190	0.000	0.000
150	original	1	60x8 weeks	Ag80	Daily	VAC95	100	Unvaccinate	0.000	0.001	0.000	0.001	0.000	0.000	0.000	0.000	0.343	0.001	0.344	0.119	0.000	0.000	0	3.430	0.010	3.440	1.190	0.000	0.000
150	original	1.2	60x8 weeks	Ag80	Daily	VAC95	100	Unvaccinate	0.000	0.001	0.000	0.001	0.000	0.000	0.000	0.000	0.352	0.000	0.352	0.122	0.000	0.000	0	3.520	0.000	3.520	1.220	0.000	0.000
150	original	1.2	60x8 weeks	Ag80	Daily	VAC95	100	Unvaccinate	0.000	0.001	0.000	0.001	0.000	0.000	0.000	0.000	0.352	0.000	0.352	0.122	0.000	0.000	0	3.520	0.000	3.520	1.220	0.000	0.000
150	original	1.2	60x8 weeks	Ag80	Daily	VAC95	100	Unvaccinate	0.000	0.001	0.000	0.001	0.000	0.000	0.000	0.000	0.352	0.000	0.352	0.122	0.000	0.000	0	3.520	0.000	3.520	1.220	0.000	0.000
150	alpha	0.6	60x8 weeks	Ag80	Daily	VAC95	100	Unvaccinate	0.000	0.001	0.000	0.001	0.001	0.000	0.000	0.000	0.475	0.032	0.507	0.249	0.000	0.000	0	4.750	0.320	5.070	2.490	0.000	0.000
150	alpha	0.6	60x8 weeks	Ag80	Daily	VAC95	100	Unvaccinate	0.000	0.001	0.000	0.001	0.001	0.000	0.000	0.000	0.475	0.032	0.507	0.249	0.000	0.000	0	4.750	0.320	5.070	2.490	0.000	0.000
150	alpha	0.6	60x8 weeks	Ag80	Daily	VAC95	100	Unvaccinate	0.000	0.001	0.000	0.001	0.001	0.000	0.000	0.000	0.475	0.032	0.507	0.249	0.000	0.000	0	4.750	0.320	5.070	2.490	0.000	0.000
150	alpha	1	60x8 weeks	Ag80	Daily	VAC95	100	Unvaccinate	0.000	0.001	0.000	0.001	0.000	0.000	0.000	0.000	0.465	0.028	0.493	0.228	0.000	0.000	0	4.650	0.280	4.930	2.280	0.000	0.000
150	alpha	1	60x8 weeks	Ag80	Daily	VAC95	100	Unvaccinate	0.000	0.001	0.000	0.001	0.000	0.000	0.000	0.000	0.465	0.028	0.493	0.228	0.000	0.000	0	4.650	0.280	4.930	2.280	0.000	0.000
150	alpha	1	60x8 weeks	Ag80	Daily	VAC95	100	Unvaccinate	0.000	0.001	0.000	0.001	0.000	0.000	0.000	0.000	0.465	0.028	0.493	0.228	0.000	0.000	0	4.650	0.280	4.930	2.280	0.000	0.000
150	alpha	1.2	60x8 weeks	Ag80	Daily	VAC95	100	Unvaccinate	0.000	0.001	0.000	0.001	0.001	0.000	0.000	0.000	0.430	0.054	0.484	0.251	0.000	0.000	0	4.300	0.540	4.840	2.510	0.000	0.000
150	alpha	1.2	60x8 weeks	Ag80	Daily	VAC95	100	Unvaccinate	0.000	0.001	0.000	0.001	0.001	0.000	0.000	0.000	0.430	0.054	0.484	0.251	0.000	0.000	0	4.300	0.540	4.840	2.510	0.000	0.000
150	alpha	1.2	60x8 weeks	Ag80	Daily	VAC95	100	Unvaccinate	0.000	0.001	0.000	0.001	0.001	0.000	0.000	0.000	0.430	0.054	0.484	0.251	0.000	0.000	0	4.300	0.540	4.840	2.510	0.000	0.000
150	delta	0.6	60x8 weeks	Ag80	Daily	VAC95	100	Unvaccinate	0.000	0.001	0.000	0.002	0.001	0.000	0.000	0.000	0.715	0.159	0.874	0.447	0.000	0.000	0	7.150	1.590	8.740	4.470	0.000	0.000
150	delta	0.6	60x8 weeks	Ag80	Daily	VAC95	100	Unvaccinate	0.000	0.001	0.000	0.002	0.001	0.000	0.000	0.000	0.715	0.159	0.874	0.447	0.000	0.000	0	7.150	1.590	8.740	4.470	0.000	0.000
150	delta	0.6	60x8 weeks	Ag80	Daily	VAC95	100	Unvaccinate	0.000	0.001	0.000	0.002	0.001	0.000	0.000	0.000	0.715	0.159	0.874	0.447	0.000	0.000	0	7.150	1.590	8.740	4.470	0.000	0.000
150	delta	1	60x8 weeks	Ag80	Daily	VAC95	100	Unvaccinate	0.000	0.001	0.001	0.002	0.001	0.000	0.000	0.000	0.688	0.273	0.961	0.492	0.000	0.000	0	6.880	2.730	9.610	4.920	0.000	0.000
150	delta	1	60x8 weeks	Ag80	Daily	VAC95	100	Unvaccinate	0.000	0.001	0.001	0.002	0.001	0.000	0.000	0.000	0.688	0.273	0.961	0.492	0.000	0.000	0	6.880	2.730	9.610	4.920	0.000	0.000
150	delta	1	60x8 weeks	Ag80	Daily	VAC95	100	Unvaccinate	0.000	0.001	0.001	0.002	0.001	0.000	0.000	0.000	0.688	0.273	0.961	0.492	0.000	0.000	0	6.880	2.730	9.610	4.920	0.000	0.000
150	delta	1.2	60x8 weeks	Ag80	Daily	VAC95	100	Unvaccinate	0.000	0.002	0.001	0.002	0.001	0.000	0.000	0.000	0.724	0.347	1.071	0.607	0.000	0.000	0	7.240	3.470	10.710	6.070	0.000	0.000
150	delta	1.2	60x8 weeks	Ag80	Daily	VAC95	100	Unvaccinate	0.000	0.002	0.001	0.002	0.001	0.000	0.000	0.000	0.724	0.347	1.071	0.607	0.000	0.000	0	7.240	3.470	10.710	6.070	0.000	0.000
150	delta	1.2	60x8 weeks	Ag80	Daily	VAC95	100	Unvaccinate	0.000	0.002	0.001	0.002	0.001	0.000	0.000	0.000	0.724	0.347	1.071	0.607	0.000	0.000	0	7.240	3.470	10.710	6.070	0.000	0.000
150	original	0.6	60x8 weeks	Ag80	Daily	VAC70	25	Unvaccinate	3.693	0.008	0.001	0.009	0.001	0.008	0.072	1772.662	4.077	0.426	4.503	0.307	4.074	34.408	17727	40.770	4.260	45.030	3.070	40.740	344.080
150	original	0.6	60x8 weeks	Ag80	Daily	VAC70	25	Unvaccinate	3.693	0.008	0.001	0.009	0.001	0.008	0.072	1772.662	4.077	0.426	4.503	0.307	4.074	34.408	17727	40.770	4.260	45.030	3.070	40.740	344.080
150	original	0.6	60x8 weeks	Ag80	Daily	VAC70	25	Unvaccinate	3.693	0.008	0.001	0.009	0.001	0.008	0.072	1772.662	4.077	0.426	4.503	0.307	4.074	34.408	17727	40.770	4.260	45.030	3.070	40.740	344.080
150	original	1	60x8 weeks	Ag80	Daily	VAC70	25	Unvaccinate	3.688	0.008	0.002	0.010	0.001	0.009	0.071	1770.314	4.005	0.763	4.768	0.329	4.351	34.147	17703	40.050	7.630	47.680	3.290	43.510	341.470
150	original	1	60x8 weeks	Ag80	Daily	VAC70	25	Unvaccinate	3.688	0.008	0.002	0.010	0.001	0.009	0.071	1770.314	4.005	0.763	4.768	0.329	4.351	34.147	17703	40.050	7.630	47.680	3.290	43.510	341.470
150	original	1	60x8 weeks	Ag80	Daily	VAC70	25	Unvaccinate	3.688	0.008	0.002	0.010	0.001	0.009	0.071	1770.314	4.005	0.763	4.768	0.329	4.351	34.147	17703	40.050	7.630	47.680	3.290	43.510	341.470
150	original	1.2	60x8 weeks	Ag80	Daily	VAC70	25	Unvaccinate	3.688	0.008	0.002	0.010	0.001	0.009	0.072	1770.308	4.011	0.883	4.894	0.382	4.373	34.519	17703	40.110	8.830	48.940	3.820	43.730	345.190
150	original	1.2	60x8 weeks	Ag80	Daily	VAC70	25	Unvaccinate	3.688	0.008	0.002	0.010	0.001	0.009	0.072	1770.308	4.011	0.883	4.894	0.382	4.373	34.519	17703	40.110	8.830	48.940	3.820	43.730	345.190
150	original	1.2	60x8 weeks	Ag80	Daily	VAC70	25	Unvaccinate	3.688	0.008	0.002	0.010	0.001	0.009	0.072	1770.308	4.011	0.883	4.894	0.382	4.373	34.519	17703	40.110	8.830	48.940	3.820	43.730	345.190
150	alpha	0.6	60x8 weeks	Ag80	Daily	VAC70	25	Unvaccinate	3.684	0.008	0.002	0.010	0.001	0.010	0.071	1768.499	4.010	0.886	4.896	0.402	4.645	34.066	17685	40.100	8.860	48.960	4.020	46.450	340.660
150	alpha	0.6	60x8 weeks	Ag80	Daily	VAC70	25	Unvaccinate	3.684	0.008	0.002	0.010	0.001	0.010	0.071	1768.499	4.010	0.886	4.896	0.402	4.645	34.066	17685	40.100	8.860	48.960	4.020	46.450	340.660
150	alpha	0.6	60x8 weeks	Ag80	Daily	VAC70	25	Unvaccinate	3.684	0.008	0.002	0.010	0.001	0.010	0.071	1768.499	4.010	0.886	4.896	0.402	4.645	34.066	17685	40.100	8.860	48.960	4.020	46.450	340.660
150	alpha	1	60x8 weeks	Ag80	Daily	VAC70	25	Unvaccinate	3.678	0.008	0.003	0.011	0.001	0.011	0.071	1765.663	3.854	1.548	5.402	0.426	5.085	34.173	17657	38.540	15.480	54.020	4.260	50.850	341.730
150	alpha	1	60x8 weeks	Ag80	Daily	VAC70	25	Unvaccinate	3.678	0.008	0.003	0.011	0.001	0.011	0.071	1765.663	3.854	1.548	5.402	0.426	5.085	34.173	17657	38.540	15.480	54.020	4.260	50.850	341.730
150	alpha	1	60x8 weeks	Ag80	Daily	VAC70	25	Unvaccinate	3.678	0.008	0.003	0.011	0.001	0.011	0.071	1765.663	3.854	1.548	5.402	0.426	5.085	34.173	17657	38.540	15.480	54.020	4.260	50.850	341.730
150	alpha	1.2	60x8 weeks	Ag80	Daily	VAC70	25	Unvaccinate	3.666	0.009	0.005	0.013	0.001	0.012	0.070	1759.661	4.103	2.269	6.372	0.514	5.959	33.520	17597	41.030	22.690	63.720	5.140	59.590	335.200
150	alpha	1.2	60x8 weeks	Ag80	Daily	VAC70	25	Unvaccinate	3.666	0.009	0.005	0.013	0.001	0.012	0.070	1759.661	4.103	2.269	6.372	0.514	5.959	33.520	17597	41.030	22.690	63.720	5.140	59.590	335.200
150	alpha	1.2	60x8 weeks	Ag80	Daily	VAC70	25	Unvaccinate	3.666	0.009	0.005	0.013	0.001	0.012	0.070	1759.661	4.103	2.269	6.372	0.514	5.959	33.520	17597	41.030	22.690	63.720	5.140	59.590	335.200
150	delta	0.6	60x8 weeks	Ag80	Daily	VAC70	25	Unvaccinate	3.664	0.008	0.005	0.014	0.001	0.012	0.070	1758.631	4.067	2.567	6.634	0.710	5.977	33.799	17586	40.670	25.670	66.340	7.100	59.770	337.990
150	delta	0.6	60x8 weeks	Ag80	Daily	VAC70	25	Unvaccinate	3.664	0.008	0.005	0.014	0.001	0.012	0.070	1758.631	4.067	2.567	6.634	0.710	5.977	33.799	17586	40.670	25.670	66.340	7.100	59.770	337.990
150	delta	0.6	60x8 weeks	Ag80	Daily	VAC70	25	Unvaccinate	3.664	0.008	0.005	0.014	0.001	0.012	0.070	1758.631	4.067	2.567	6.634	0.710	5.977	33.799	17586	40.670	25.670	66.340	7.100	59.770	337.990
150	delta	1	60x8 weeks	Ag80	Daily	VAC70	25	Unvaccinate	3.613	0.008	0.013	0.022	0.002	0.020	0.068	1734.353	4.024	6.433	10.457	1.146	9.426	32.593	17344	40.240	64.330	104.570	11.460	94.260	325.930
150	delta	1	60x8 weeks	Ag80	Daily	VAC70	25	Unvaccinate	3.613	0.008	0.013	0.022	0.002	0.020	0.068	1734.353	4.024	6.433	10.457	1.146	9.426	32.593	17344	40.240	64.330	104.570	11.460	94.260	325.930
150	delta	1	60x8 weeks	Ag80	Daily	VAC70	25	Unvaccinate	3.613	0.008	0.013	0.022	0.002	0.020	0.068	1734.353	4.024	6.433	10.457	1.146	9.426	32.593	17344	40.240	64.330	104.570	11.460	94.260	325.930
150	delta	1.2	60x8 weeks	Ag80	Daily	VAC70	25	Unvaccinate	3.570	0.008	0.021	0.029	0.003	0.026	0.065	1713.506	3.883	9.965	13.848	1.598	12.418	31.392	17135	38.830	99.650	138.480	15.980	124.180	313.920
150	delta	1.2	60x8 weeks	Ag80	Daily	VAC70	25	Unvaccinate	3.570	0.008	0.021	0.029	0.003	0.026	0.065	1713.506	3.883	9.965	13.848	1.598	12.418	31.392	17135	38.830	99.650	138.480	15.980	124.180	313.920
150	delta	1.2	60x8 weeks	Ag80	Daily	VAC70	25	Unvaccinate	3.570	0.008	0.021	0.029	0.003	0.026	0.065	1713.506	3.883	9.965	13.848	1.598	12.418	31.392	17135	38.830	99.650	138.480	15.980	124.180	313.920

150	original	0.6	60x8 weeks	Ag80	Daily	VAC70	50	Unvaccinate	2.465	0.007	0.000	0.007	0.001	0.005	0.049	1183.051	3.189	0.229	3.418	0.503	2.501	23.319	11831	31.890	2.290	34.180	5.030	25.010	233.190
150	original	0.6	60x8 weeks	Ag80	Daily	VAC70	50	Unvaccinate	2.465	0.007	0.000	0.007	0.001	0.005	0.049	1183.051	3.189	0.229	3.418	0.503	2.501	23.319	11831	31.890	2.290	34.180	5.030	25.010	233.190
150	original	0.6	60x8 weeks	Ag80	Daily	VAC70	50	Unvaccinate	2.465	0.007	0.000	0.007	0.001	0.005	0.049	1183.051	3.189	0.229	3.418	0.503	2.501	23.319	11831	31.890	2.290	34.180	5.030	25.010	233.190
150	original	1	60x8 weeks	Ag80	Daily	VAC70	50	Unvaccinate	2.461	0.007	0.001	0.008	0.001	0.006	0.048	1181.255	3.223	0.430	3.653	0.537	2.747	22.827	11813	32.230	4.300	36.530	5.370	27.470	228.270
150	original	1	60x8 weeks	Ag80	Daily	VAC70	50	Unvaccinate	2.461	0.007	0.001	0.008	0.001	0.006	0.048	1181.255	3.223	0.430	3.653	0.537	2.747	22.827	11813	32.230	4.300	36.530	5.370	27.470	228.270
150	original	1	60x8 weeks	Ag80	Daily	VAC70	50	Unvaccinate	2.461	0.007	0.001	0.008	0.001	0.006	0.048	1181.255	3.223	0.430	3.653	0.537	2.747	22.827	11813	32.230	4.300	36.530	5.370	27.470	228.270
150	original	1.2	60x8 weeks	Ag80	Daily	VAC70	50	Unvaccinate	2.462	0.007	0.001	0.008	0.001	0.006	0.048	1181.737	3.175	0.434	3.609	0.532	2.677	22.952	11817	31.750	4.340	36.090	5.320	26.770	229.520
150	original	1.2	60x8 weeks	Ag80	Daily	VAC70	50	Unvaccinate	2.462	0.007	0.001	0.008	0.001	0.006	0.048	1181.737	3.175	0.434	3.609	0.532	2.677	22.952	11817	31.750	4.340	36.090	5.320	26.770	229.520
150	original	1.2	60x8 weeks	Ag80	Daily	VAC70	50	Unvaccinate	2.462	0.007	0.001	0.008	0.001	0.006	0.048	1181.737	3.175	0.434	3.609	0.532	2.677	22.952	11817	31.750	4.340	36.090	5.320	26.770	229.520
150	alpha	0.6	60x8 weeks	Ag80	Daily	VAC70	50	Unvaccinate	2.458	0.007	0.001	0.008	0.001	0.006	0.047	1179.999	3.145	0.693	3.838	0.682	2.921	22.789	11800	31.450	6.930	38.380	6.820	29.210	227.890
150	alpha	0.6	60x8 weeks	Ag80	Daily	VAC70	50	Unvaccinate	2.458	0.007	0.001	0.008	0.001	0.006	0.047	1179.999	3.145	0.693	3.838	0.682	2.921	22.789	11800	31.450	6.930	38.380	6.820	29.210	227.890
150	alpha	0.6	60x8 weeks	Ag80	Daily	VAC70	50	Unvaccinate	2.458	0.007	0.001	0.008	0.001	0.006	0.047	1179.999	3.145	0.693	3.838	0.682	2.921	22.789	11800	31.450	6.930	38.380	6.820	29.210	227.890
150	alpha	1	60x8 weeks	Ag80	Daily	VAC70	50	Unvaccinate	2.452	0.006	0.003	0.009	0.002	0.007	0.047	1176.761	3.106	1.411	4.517	0.783	3.450	22.705	11768	31.060	14.110	45.170	7.830	34.500	227.050
150	alpha	1	60x8 weeks	Ag80	Daily	VAC70	50	Unvaccinate	2.452	0.006	0.003	0.009	0.002	0.007	0.047	1176.761	3.106	1.411	4.517	0.783	3.450	22.705	11768	31.060	14.110	45.170	7.830	34.500	227.050
150	alpha	1	60x8 weeks	Ag80	Daily	VAC70	50	Unvaccinate	2.452	0.006	0.003	0.009	0.002	0.007	0.047	1176.761	3.106	1.411	4.517	0.783	3.450	22.705	11768	31.060	14.110	45.170	7.830	34.500	227.050
150	alpha	1.2	60x8 weeks	Ag80	Daily	VAC70	50	Unvaccinate	2.446	0.007	0.004	0.010	0.002	0.008	0.047	1174.003	3.199	1.778	4.977	0.828	3.851	22.477	11740	31.990	17.780	49.770	8.280	38.510	224.770
150	alpha	1.2	60x8 weeks	Ag80	Daily	VAC70	50	Unvaccinate	2.446	0.007	0.004	0.010	0.002	0.008	0.047	1174.003	3.199	1.778	4.977	0.828	3.851	22.477	11740	31.990	17.780	49.770	8.280	38.510	224.770
150	alpha	1.2	60x8 weeks	Ag80	Daily	VAC70	50	Unvaccinate	2.446	0.007	0.004	0.010	0.002	0.008	0.047	1174.003	3.199	1.778	4.977	0.828	3.851	22.477	11740	31.990	17.780	49.770	8.280	38.510	224.770
150	delta	0.6	60x8 weeks	Ag80	Daily	VAC70	50	Unvaccinate	2.438	0.007	0.005	0.013	0.003	0.009	0.047	1170.455	3.462	2.629	6.091	1.309	4.323	22.405	11705	34.620	26.290	60.910	13.090	43.230	224.050
150	delta	0.6	60x8 weeks	Ag80	Daily	VAC70	50	Unvaccinate	2.438	0.007	0.005	0.013	0.003	0.009	0.047	1170.455	3.462	2.629	6.091	1.309	4.323	22.405	11705	34.620	26.290	60.910	13.090	43.230	224.050
150	delta	0.6	60x8 weeks	Ag80	Daily	VAC70	50	Unvaccinate	2.438	0.007	0.005	0.013	0.003	0.009	0.047	1170.455	3.462	2.629	6.091	1.309	4.323	22.405	11705	34.620	26.290	60.910	13.090	43.230	224.050
150	delta	1	60x8 weeks	Ag80	Daily	VAC70	50	Unvaccinate	2.406	0.007	0.013	0.020	0.004	0.014	0.045	1154.761	3.195	6.305	9.500	2.115	6.627	21.374	11548	31.950	63.050	95.000	21.150	66.270	213.740
150	delta	1	60x8 weeks	Ag80	Daily	VAC70	50	Unvaccinate	2.406	0.007	0.013	0.020	0.004	0.014	0.045	1154.761	3.195	6.305	9.500	2.115	6.627	21.374	11548	31.950	63.050	95.000	21.150	66.270	213.740
150	delta	1	60x8 weeks	Ag80	Daily	VAC70	50	Unvaccinate	2.406	0.007	0.013	0.020	0.004	0.014	0.045	1154.761	3.195	6.305	9.500	2.115	6.627	21.374	11548	31.950	63.050	95.000	21.150	66.270	213.740
150	delta	1.2	60x8 weeks	Ag80	Daily	VAC70	50	Unvaccinate	2.363	0.007	0.022	0.028	0.007	0.020	0.043	1134.101	3.251	10.337	13.588	3.122	9.451	20.449	11341	32.510	103.370	135.880	31.220	94.510	204.490
150	delta	1.2	60x8 weeks	Ag80	Daily	VAC70	50	Unvaccinate	2.363	0.007	0.022	0.028	0.007	0.020	0.043	1134.101	3.251	10.337	13.588	3.122	9.451	20.449	11341	32.510	103.370	135.880	31.220	94.510	204.490
150	delta	1.2	60x8 weeks	Ag80	Daily	VAC70	50	Unvaccinate	2.363	0.007	0.022	0.028	0.007	0.020	0.043	1134.101	3.251	10.337	13.588	3.122	9.451	20.449	11341	32.510	103.370	135.880	31.220	94.510	204.490
150	original	0.6	60x8 weeks	Ag80	Daily	VAC70	75	Unvaccinate	1.232	0.005	0.000	0.005	0.001	0.003	0.024	591.572	2.372	0.083	2.455	0.578	1.236	11.636	5916	23.720	0.830	24.550	5.780	12.360	116.360
150	original	0.6	60x8 weeks	Ag80	Daily	VAC70	75	Unvaccinate	1.232	0.005	0.000	0.005	0.001	0.003	0.024	591.572	2.372	0.083	2.455	0.578	1.236	11.636	5916	23.720	0.830	24.550	5.780	12.360	116.360
150	original	0.6	60x8 weeks	Ag80	Daily	VAC70	75	Unvaccinate	1.232	0.005	0.000	0.005	0.001	0.003	0.024	591.572	2.372	0.083	2.455	0.578	1.236	11.636	5916	23.720	0.830	24.550	5.780	12.360	116.360
150	original	1	60x8 weeks	Ag80	Daily	VAC70	75	Unvaccinate	1.233	0.005	0.000	0.005	0.001	0.003	0.024	591.697	2.409	0.156	2.565	0.659	1.230	11.620	5917	24.090	1.560	25.650	6.590	12.300	116.200
150	original	1	60x8 weeks	Ag80	Daily	VAC70	75	Unvaccinate	1.233	0.005	0.000	0.005	0.001	0.003	0.024	591.697	2.409	0.156	2.565	0.659	1.230	11.620	5917	24.090	1.560	25.650	6.590	12.300	116.200
150	original	1	60x8 weeks	Ag80	Daily	VAC70	75	Unvaccinate	1.233	0.005	0.000	0.005	0.001	0.003	0.024	591.697	2.409	0.156	2.565	0.659	1.230	11.620	5917	24.090	1.560	25.650	6.590	12.300	116.200
150	original	1.2	60x8 weeks	Ag80	Daily	VAC70	75	Unvaccinate	1.231	0.005	0.000	0.006	0.001	0.003	0.024	591.117	2.432	0.214	2.646	0.694	1.292	11.532	5911	24.320	2.140	26.460	6.940	12.920	115.320
150	original	1.2	60x8 weeks	Ag80	Daily	VAC70	75	Unvaccinate	1.231	0.005	0.000	0.006	0.001	0.003	0.024	591.117	2.432	0.214	2.646	0.694	1.292	11.532	5911	24.320	2.140	26.460	6.940	12.920	115.320
150	original	1.2	60x8 weeks	Ag80	Daily	VAC70	75	Unvaccinate	1.231	0.005	0.000	0.006	0.001	0.003	0.024	591.117	2.432	0.214	2.646	0.694	1.292	11.532	5911	24.320	2.140	26.460	6.940	12.920	115.320
150	alpha	0.6	60x8 weeks	Ag80	Daily	VAC70	75	Unvaccinate	1.229	0.005	0.001	0.006	0.002	0.003	0.024	589.888	2.355	0.527	2.882	0.941	1.483	11.468	5899	23.550	5.270	28.820	9.410	14.830	114.680
150	alpha	0.6	60x8 weeks	Ag80	Daily	VAC70	75	Unvaccinate	1.229	0.005	0.001	0.006	0.002	0.003	0.024	589.888	2.355	0.527	2.882	0.941	1.483	11.468	5899	23.550	5.270	28.820	9.410	14.830	114.680
150	alpha	0.6	60x8 weeks	Ag80	Daily	VAC70	75	Unvaccinate	1.229	0.005	0.001	0.006	0.002	0.003	0.024	589.888	2.355	0.527	2.882	0.941	1.483	11.468	5899	23.550	5.270	28.820	9.410	14.830	114.680
150	alpha	1	60x8 weeks	Ag80	Daily	VAC70	75	Unvaccinate	1.226	0.005	0.002	0.007	0.002	0.004	0.023	588.330	2.335	0.945	3.280	0.998	1.717	11.222	5883	23.350	9.450	32.800	9.980	17.170	112.220
150	alpha	1	60x8 weeks	Ag80	Daily	VAC70	75	Unvaccinate	1.226	0.005	0.002	0.007	0.002	0.004	0.023	588.330	2.335	0.945	3.280	0.998	1.717	11.222	5883	23.350	9.450	32.800	9.980	17.170	112.220
150	alpha	1	60x8 weeks	Ag80	Daily	VAC70	75	Unvaccinate	1.226	0.005	0.002	0.007	0.002	0.004	0.023	588.330	2.335	0.945	3.280	0.998	1.717	11.222	5883	23.350	9.450	32.800	9.980	17.170	112.220
150	alpha	1.2	60x8 weeks	Ag80	Daily	VAC70	75	Unvaccinate	1.225	0.005	0.003	0.007	0.002	0.004	0.024	587.767	2.358	1.219	3.577	1.084	1.837	11.336	5878	23.580	12.190	35.770	10.840	18.370	113.360
150	alpha	1.2	60x8 weeks	Ag80	Daily	VAC70	75	Unvaccinate	1.225	0.005	0.003	0.007	0.002	0.004	0.024	587.767	2.358	1.219	3.577	1.084	1.837	11.336	5878	23.580	12.190	35.770	10.840	18.370	113.360
150	alpha	1.2	60x8 weeks	Ag80	Daily	VAC70	75	Unvaccinate	1.225	0.005	0.003	0.007	0.002	0.004	0.024	587.767	2.358	1.219	3.577	1.084	1.837	11.336	5878	23.580	12.190	35.770	10.840	18.370	113.360
150	delta	0.6	60x8 weeks	Ag80	Daily	VAC70	75	Unvaccinate	1.220	0.005	0.004	0.010	0.004	0.004	0.023	585.468	2.627	2.102	4.729	1.783	2.099	11.119	5855	26.270	21.020	47.290	17.830	20.990	111.190
150	delta	0.6	60x8 weeks	Ag80	Daily	VAC70	75	Unvaccinate	1.220	0.005	0.004	0.010	0.004	0.004	0.023	585.468	2.627	2.102	4.729	1.783	2.099	11.119	5855	26.270	21.020	47.290	17.830	20.990	111.190
150	delta	0.6	60x8 weeks	Ag80	Daily	VAC70	75	Unvaccinate	1.220	0.005	0.004	0.010	0.004	0.004	0.023	585.468	2.627	2.102	4.729	1.783	2.099	11.119	5855	26.270	21.020	47.290	17.830	20.990	111.190
150	delta	1	60x8 weeks	Ag80	Daily	VAC70	75	Unvaccinate	1.199	0.006	0.012	0.018	0.007	0.007	0.023	575.560	2.672	5.729	8.401	3.166	3.538	10.863	5756	26.720	57.290	84.010	31.660	35.380	108.630
150	delta	1	60x8 weeks	Ag80	Daily	VAC70	75	Unvaccinate	1.199	0.006	0.012	0.018	0.007	0.007	0.023	575.560	2.672	5.729	8.401	3.166	3.538	10.863	5756	26.720	57.290	84.010	31.660	35.380	108.630
150	delta	1	60x8 weeks	Ag80	Daily	VAC70	75	Unvaccinate	1.199	0.006	0.012	0.018	0.007	0.007	0.023	575.560	2.672	5.729	8.401	3.166	3.538	10.863	5756	26.720	57.290	84.010	31.660	35.380	108.630
150	delta	1.2	60x8 weeks	Ag80	Daily	VAC70	75	Unvaccinate	1.182	0.005	0.019	0.024	0.009	0.010	0.022	567.163	2.556	9.017	11.573	4.418	4.779	10.337	5672	25.560	90.170	115.730	44.180	47.790	103.370
150	delta	1.2	60x8 weeks	Ag80	Daily	VAC70	75	Unvaccinate	1.182	0.005	0.019	0.024	0.009	0.010	0.022	567.163	2.556	9.017	11.573	4.418	4.779	10.337	5672	25.560	90.170	115.730	44.180	47.790	103.370
150	delta	1.2	60x8 weeks	Ag80	Daily	VAC70	75	Unvaccinate	1.182	0.005	0.019	0.024	0.009	0.010	0.022	567.163	2.556	9.017	11.573	4.418	4.779	10.337	5672	25.560	90.170	115.730	44.180	47.790	103.370
150	original	0.6	60x8 weeks	Ag80	Daily	VAC70	100	Unvaccinate	0.000	0.003	0.000	0.003	0.002	0.000	0.000	0.000	1.636	0.016	1.652	0.841	0.000	0.000	0	16.360	0.160	16.520	8.410	0.000	0.000
150	original	0.6	60x8 weeks	Ag80	Daily	VAC70	100	Unvaccinate	0.000	0.003	0.000	0.003	0.002	0.000	0.000	0.000	1.636	0.016	1.652	0.841	0.000	0.000	0	16.360	0.160	16.520	8.410	0.000	0.000
150	original	0.6	60x8 weeks	Ag80	Daily	VAC70	100	Unvaccinate	0.000	0.003	0.000	0.003	0.002	0.000	0.000	0.000	1.636	0.016	1.652	0.841	0.000	0.000	0	16.360	0.160	16.520	8.410	0.000	0.000
150	original	1	60x8 weeks	Ag80	Daily	VAC70	100	Unvaccinate	0.000	0.003	0.000	0.003	0.002	0.000	0.000	0.000	1.649	0.018	1.667	0.811	0.000	0.000	0	16.490	0.180	16.670	8.110	0.000	0.000
150	original	1	60x8 weeks	Ag80	Daily	VAC70	100	Unvaccinate	0.000	0.003	0.000	0.003	0.002	0.000	0.000	0.000	1.649	0.018	1.667	0.811	0.000	0.000	0	16.490	0.180	16.670	8.110	0.000	0.000
150	original	1	60x8 weeks	Ag80	Daily	VAC70	100	Unvaccinate	0.000	0.003	0.000	0.003	0.002	0.000	0.000	0.000	1.649	0.018	1.667	0.811	0.000	0.000	0	16.490	0.180	16.670	8.110	0.000	0.000
150	original	1.2	60x8 weeks	Ag80	Daily	VAC70	100	Unvaccinate	0.000	0.003	0.000	0.003	0.002	0.000	0.000	0.000	1.637	0.028	1.665	0.837	0.000	0.000	0	16.370	0.280	16.650	8.370	0.000	0.000
150	original	1.2	60x8 weeks	Ag80	Daily	VAC70	100	Unvaccinate	0.000	0.003	0.000	0.003	0.002	0.000	0.000	0.000	1.637	0.028	1.665	0.837	0.000	0.000	0	16.370	0.280	16.650	8.370	0.000	0.000
150	original	1.2	60x8 weeks	Ag80	Daily	VAC70	100	Unvaccinate	0.000	0.003	0.000	0.003	0.002	0.000	0.000	0.000	1.637	0.028	1.665	0.837	0.000	0.000	0	16.370	0.280	16.650	8.370	0.000	0.000
150	alpha	0.6	60x8 weeks	Ag80	Daily	VAC70	100	Unvaccinate	0.000	0.003	0.001	0.004	0.002	0.000	0.000	0.000	1.604	0.306	1.910	1.146	0.000	0.000	0	16.040	3.060	19.100	11.460	0.000	0.000
150	alpha	0.6	60x8 weeks	Ag80	Daily	VAC70	100	Unvaccinate	0.000	0.003	0.001	0.004	0.002	0.000	0.000	0.000	1.604	0.306	1.910	1.146	0.000	0.000	0	16.040	3.060	19.100	11.460	0.000	0.000
150	alpha	0.6	60x8 weeks	Ag80	Daily	VAC70	100	Unvaccinate	0.000	0.003	0.001	0.004	0.002	0.000	0.000	0.000	1.604	0.306	1.910	1.146	0.000	0.000	0	16.040	3.060	19.100	11.460	0.000	0.000
150	alpha	1	60x8 weeks	Ag80	Daily	VAC70	100	Unvaccinate	0.000	0.003	0.001	0.004	0.003	0.000	0.000	0.000	1.584	0.568	2.152	1.305	0.000	0.000	0	15.840	5.680	21.520	13.050	0.000	0.000
150	alpha	1	60x8 weeks	Ag80	Daily	VAC70	100	Unvaccinate	0.000	0.003	0.001	0.004	0.003	0.000	0.000	0.000	1.584	0.568	2.152	1.305	0.000	0.000	0	15.840	5.680	21.520	13.050	0.000	0.000
150	alpha	1	60x8 weeks	Ag80	Daily	VAC70	100	Unvaccinate	0.000	0.003	0.001	0.004	0.003	0.000	0.000	0.000	1.584	0.568	2.152	1.305	0.000	0.000	0	15.840	5.680	21.520	13.050	0.000	0.000
150	alpha	1.2	60x8 weeks	Ag80	Daily	VAC70	100	Unvaccinate	0.000	0.003	0.002	0.005	0.003	0.000	0.000	0.000	1.580	0.898	2.478	1.411	0.000	0.000	0	15.800	8.980	24.780	14.110	0.000	0.000
150	alpha	1.2	60x8 weeks	Ag80	Daily	VAC70	100	Unvaccinate	0.000	0.003	0.002	0.005	0.003	0.000	0.000	0.000	1.580	0.898	2.478	1.411	0.000	0.000	0	15.800	8.980	24.780	14.110	0.000	0.000

150	alpha	1.2	60x8 weeks	Ag80	Daily	VAC70	100	Unvaccinate	0.000	0.003	0.002	0.005	0.003	0.000	0.000	0.000	1.580	0.898	2.478	1.411	0.000	0.000	0	15.800	8.980	24.780	14.110	0.000	0.000
150	delta	0.6	60x8 weeks	Ag80	Daily	VAC70	100	Unvaccinate	0.000	0.004	0.004	0.008	0.005	0.000	0.000	0.000	2.027	1.734	3.761	2.318	0.000	0.000	0	20.270	17.340	37.610	23.180	0.000	0.000
150	delta	0.6	60x8 weeks	Ag80	Daily	VAC70	100	Unvaccinate	0.000	0.004	0.004	0.008	0.005	0.000	0.000	0.000	2.027	1.734	3.761	2.318	0.000	0.000	0	20.270	17.340	37.610	23.180	0.000	0.000
150	delta	0.6	60x8 weeks	Ag80	Daily	VAC70	100	Unvaccinate	0.000	0.004	0.004	0.008	0.005	0.000	0.000	0.000	2.027	1.734	3.761	2.318	0.000	0.000	0	20.270	17.340	37.610	23.180	0.000	0.000
150	delta	1	60x8 weeks	Ag80	Daily	VAC70	100	Unvaccinate	0.000	0.004	0.010	0.014	0.009	0.000	0.000	0.000	2.002	4.950	6.952	4.348	0.000	0.000	0	20.020	49.500	69.520	43.480	0.000	0.000
150	delta	1	60x8 weeks	Ag80	Daily	VAC70	100	Unvaccinate	0.000	0.004	0.010	0.014	0.009	0.000	0.000	0.000	2.002	4.950	6.952	4.348	0.000	0.000	0	20.020	49.500	69.520	43.480	0.000	0.000
150	delta	1	60x8 weeks	Ag80	Daily	VAC70	100	Unvaccinate	0.000	0.004	0.010	0.014	0.009	0.000	0.000	0.000	2.002	4.950	6.952	4.348	0.000	0.000	0	20.020	49.500	69.520	43.480	0.000	0.000
150	delta	1.2	60x8 weeks	Ag80	Daily	VAC70	100	Unvaccinate	0.000	0.004	0.014	0.018	0.011	0.000	0.000	0.000	1.846	6.687	8.533	5.255	0.000	0.000	0	18.460	66.870	85.330	52.550	0.000	0.000
150	delta	1.2	60x8 weeks	Ag80	Daily	VAC70	100	Unvaccinate	0.000	0.004	0.014	0.018	0.011	0.000	0.000	0.000	1.846	6.687	8.533	5.255	0.000	0.000	0	18.460	66.870	85.330	52.550	0.000	0.000
150	delta	1.2	60x8 weeks	Ag80	Daily	VAC70	100	Unvaccinate	0.000	0.004	0.014	0.018	0.011	0.000	0.000	0.000	1.846	6.687	8.533	5.255	0.000	0.000	0	18.460	66.870	85.330	52.550	0.000	0.000
150	original	0.6	60x8 weeks	Ag80	Daily	VAC50	25	Unvaccinate	3.694	0.009	0.001	0.010	0.001	0.008	0.072	1773.354	4.226	0.433	4.659	0.493	3.942	34.606	17734	42.260	4.330	46.590	4.930	39.420	346.060
150	original	0.6	60x8 weeks	Ag80	Daily	VAC50	25	Unvaccinate	3.694	0.009	0.001	0.010	0.001	0.008	0.072	1773.354	4.226	0.433	4.659	0.493	3.942	34.606	17734	42.260	4.330	46.590	4.930	39.420	346.060
150	original	0.6	60x8 weeks	Ag80	Daily	VAC50	25	Unvaccinate	3.694	0.009	0.001	0.010	0.001	0.008	0.072	1773.354	4.226	0.433	4.659	0.493	3.942	34.606	17734	42.260	4.330	46.590	4.930	39.420	346.060
150	original	1	60x8 weeks	Ag80	Daily	VAC50	25	Unvaccinate	3.689	0.009	0.002	0.011	0.001	0.009	0.072	1770.938	4.275	0.798	5.073	0.471	4.347	34.455	17709	42.750	7.980	50.730	4.710	43.470	344.550
150	original	1	60x8 weeks	Ag80	Daily	VAC50	25	Unvaccinate	3.689	0.009	0.002	0.011	0.001	0.009	0.072	1770.938	4.275	0.798	5.073	0.471	4.347	34.455	17709	42.750	7.980	50.730	4.710	43.470	344.550
150	original	1	60x8 weeks	Ag80	Daily	VAC50	25	Unvaccinate	3.689	0.009	0.002	0.011	0.001	0.009	0.072	1770.938	4.275	0.798	5.073	0.471	4.347	34.455	17709	42.750	7.980	50.730	4.710	43.470	344.550
150	original	1.2	60x8 weeks	Ag80	Daily	VAC50	25	Unvaccinate	3.689	0.009	0.002	0.011	0.001	0.009	0.071	1770.606	4.124	0.963	5.087	0.537	4.339	34.318	17706	41.240	9.630	50.870	5.370	43.390	343.180
150	original	1.2	60x8 weeks	Ag80	Daily	VAC50	25	Unvaccinate	3.689	0.009	0.002	0.011	0.001	0.009	0.071	1770.606	4.124	0.963	5.087	0.537	4.339	34.318	17706	41.240	9.630	50.870	5.370	43.390	343.180
150	original	1.2	60x8 weeks	Ag80	Daily	VAC50	25	Unvaccinate	3.689	0.009	0.002	0.011	0.001	0.009	0.071	1770.606	4.124	0.963	5.087	0.537	4.339	34.318	17706	41.240	9.630	50.870	5.370	43.390	343.180
150	alpha	0.6	60x8 weeks	Ag80	Daily	VAC50	25	Unvaccinate	3.684	0.009	0.002	0.011	0.001	0.010	0.072	1768.397	4.163	1.128	5.291	0.591	4.674	34.403	17684	41.630	11.280	52.910	5.910	46.740	344.030
150	alpha	0.6	60x8 weeks	Ag80	Daily	VAC50	25	Unvaccinate	3.684	0.009	0.002	0.011	0.001	0.010	0.072	1768.397	4.163	1.128	5.291	0.591	4.674	34.403	17684	41.630	11.280	52.910	5.910	46.740	344.030
150	alpha	0.6	60x8 weeks	Ag80	Daily	VAC50	25	Unvaccinate	3.684	0.009	0.002	0.011	0.001	0.010	0.072	1768.397	4.163	1.128	5.291	0.591	4.674	34.403	17684	41.630	11.280	52.910	5.910	46.740	344.030
150	alpha	1	60x8 weeks	Ag80	Daily	VAC50	25	Unvaccinate	3.669	0.009	0.005	0.014	0.002	0.012	0.070	1761.169	4.281	2.204	6.485	0.752	5.699	33.731	17612	42.810	22.040	64.850	7.520	56.990	337.310
150	alpha	1	60x8 weeks	Ag80	Daily	VAC50	25	Unvaccinate	3.669	0.009	0.005	0.014	0.002	0.012	0.070	1761.169	4.281	2.204	6.485	0.752	5.699	33.731	17612	42.810	22.040	64.850	7.520	56.990	337.310
150	alpha	1	60x8 weeks	Ag80	Daily	VAC50	25	Unvaccinate	3.669	0.009	0.005	0.014	0.002	0.012	0.070	1761.169	4.281	2.204	6.485	0.752	5.699	33.731	17612	42.810	22.040	64.850	7.520	56.990	337.310
150	alpha	1.2	60x8 weeks	Ag80	Daily	VAC50	25	Unvaccinate	3.662	0.009	0.006	0.015	0.002	0.013	0.070	1757.577	4.113	2.912	7.025	0.762	6.264	33.694	17576	41.130	29.120	70.250	7.620	62.640	336.940
150	alpha	1.2	60x8 weeks	Ag80	Daily	VAC50	25	Unvaccinate	3.662	0.009	0.006	0.015	0.002	0.013	0.070	1757.577	4.113	2.912	7.025	0.762	6.264	33.694	17576	41.130	29.120	70.250	7.620	62.640	336.940
150	alpha	1.2	60x8 weeks	Ag80	Daily	VAC50	25	Unvaccinate	3.662	0.009	0.006	0.015	0.002	0.013	0.070	1757.577	4.113	2.912	7.025	0.762	6.264	33.694	17576	41.130	29.120	70.250	7.620	62.640	336.940
150	delta	0.6	60x8 weeks	Ag80	Daily	VAC50	25	Unvaccinate	3.655	0.009	0.007	0.016	0.002	0.014	0.070	1754.421	4.360	3.402	7.762	1.138	6.604	33.436	17544	43.600	34.020	77.620	11.380	66.040	334.360
150	delta	0.6	60x8 weeks	Ag80	Daily	VAC50	25	Unvaccinate	3.655	0.009	0.007	0.016	0.002	0.014	0.070	1754.421	4.360	3.402	7.762	1.138	6.604	33.436	17544	43.600	34.020	77.620	11.380	66.040	334.360
150	delta	0.6	60x8 weeks	Ag80	Daily	VAC50	25	Unvaccinate	3.655	0.009	0.007	0.016	0.002	0.014	0.070	1754.421	4.360	3.402	7.762	1.138	6.604	33.436	17544	43.600	34.020	77.620	11.380	66.040	334.360
150	delta	1	60x8 weeks	Ag80	Daily	VAC50	25	Unvaccinate	3.584	0.009	0.020	0.028	0.004	0.024	0.066	1720.326	4.152	9.492	13.644	2.054	11.536	31.569	17203	41.520	94.920	136.440	20.540	115.360	315.690
150	delta	1	60x8 weeks	Ag80	Daily	VAC50	25	Unvaccinate	3.584	0.009	0.020	0.028	0.004	0.024	0.066	1720.326	4.152	9.492	13.644	2.054	11.536	31.569	17203	41.520	94.920	136.440	20.540	115.360	315.690
150	delta	1	60x8 weeks	Ag80	Daily	VAC50	25	Unvaccinate	3.584	0.009	0.020	0.028	0.004	0.024	0.066	1720.326	4.152	9.492	13.644	2.054	11.536	31.569	17203	41.520	94.920	136.440	20.540	115.360	315.690
150	delta	1.2	60x8 weeks	Ag80	Daily	VAC50	25	Unvaccinate	3.522	0.008	0.031	0.039	0.006	0.033	0.063	1690.726	3.889	15.053	18.942	2.942	15.845	30.097	16907	38.890	150.530	189.420	29.420	158.450	300.970
150	delta	1.2	60x8 weeks	Ag80	Daily	VAC50	25	Unvaccinate	3.522	0.008	0.031	0.039	0.006	0.033	0.063	1690.726	3.889	15.053	18.942	2.942	15.845	30.097	16907	38.890	150.530	189.420	29.420	158.450	300.970
150	delta	1.2	60x8 weeks	Ag80	Daily	VAC50	25	Unvaccinate	3.522	0.008	0.031	0.039	0.006	0.033	0.063	1690.726	3.889	15.053	18.942	2.942	15.845	30.097	16907	38.890	150.530	189.420	29.420	158.450	300.970
150	original	0.6	60x8 weeks	Ag80	Daily	VAC50	50	Unvaccinate	2.462	0.008	0.001	0.008	0.002	0.006	0.048	1181.917	3.749	0.283	4.032	0.782	2.671	23.065	11819	37.490	2.830	40.320	7.820	26.710	230.650
150	original	0.6	60x8 weeks	Ag80	Daily	VAC50	50	Unvaccinate	2.462	0.008	0.001	0.008	0.002	0.006	0.048	1181.917	3.749	0.283	4.032	0.782	2.671	23.065	11819	37.490	2.830	40.320	7.820	26.710	230.650
150	original	0.6	60x8 weeks	Ag80	Daily	VAC50	50	Unvaccinate	2.462	0.008	0.001	0.008	0.002	0.006	0.048	1181.917	3.749	0.283	4.032	0.782	2.671	23.065	11819	37.490	2.830	40.320	7.820	26.710	230.650
150	original	1	60x8 weeks	Ag80	Daily	VAC50	50	Unvaccinate	2.461	0.008	0.001	0.009	0.002	0.006	0.047	1181.270	3.744	0.451	4.195	0.812	2.792	22.751	11813	37.440	4.510	41.950	8.120	27.920	227.510
150	original	1	60x8 weeks	Ag80	Daily	VAC50	50	Unvaccinate	2.461	0.008	0.001	0.009	0.002	0.006	0.047	1181.270	3.744	0.451	4.195	0.812	2.792	22.751	11813	37.440	4.510	41.950	8.120	27.920	227.510
150	original	1	60x8 weeks	Ag80	Daily	VAC50	50	Unvaccinate	2.461	0.008	0.001	0.009	0.002	0.006	0.047	1181.270	3.744	0.451	4.195	0.812	2.792	22.751	11813	37.440	4.510	41.950	8.120	27.920	227.510
150	original	1.2	60x8 weeks	Ag80	Daily	VAC50	50	Unvaccinate	2.461	0.008	0.001	0.009	0.002	0.006	0.048	1181.113	3.697	0.539	4.236	0.813	2.786	22.970	11811	36.970	5.390	42.360	8.130	27.860	229.700
150	original	1.2	60x8 weeks	Ag80	Daily	VAC50	50	Unvaccinate	2.461	0.008	0.001	0.009	0.002	0.006	0.048	1181.113	3.697	0.539	4.236	0.813	2.786	22.970	11811	36.970	5.390	42.360	8.130	27.860	229.700
150	original	1.2	60x8 weeks	Ag80	Daily	VAC50	50	Unvaccinate	2.461	0.008	0.001	0.009	0.002	0.006	0.048	1181.113	3.697	0.539	4.236	0.813	2.786	22.970	11811	36.970	5.390	42.360	8.130	27.860	229.700
150	alpha	0.6	60x8 weeks	Ag80	Daily	VAC50	50	Unvaccinate	2.455	0.008	0.002	0.010	0.002	0.007	0.048	1178.249	3.628	1.090	4.718	1.050	3.196	22.874	11782	36.280	10.900	47.180	10.500	31.960	228.740
150	alpha	0.6	60x8 weeks	Ag80	Daily	VAC50	50	Unvaccinate	2.455	0.008	0.002	0.010	0.002	0.007	0.048	1178.249	3.628	1.090	4.718	1.050	3.196	22.874	11782	36.280	10.900	47.180	10.500	31.960	228.740
150	alpha	0.6	60x8 weeks	Ag80	Daily	VAC50	50	Unvaccinate	2.455	0.008	0.002	0.010	0.002	0.007	0.048	1178.249	3.628	1.090	4.718	1.050	3.196	22.874	11782	36.280	10.900	47.180	10.500	31.960	228.740
150	alpha	1	60x8 weeks	Ag80	Daily	VAC50	50	Unvaccinate	2.442	0.008	0.005	0.012	0.003	0.008	0.047	1172.273	3.627	2.284	5.911	1.340	4.069	22.341	11723	36.270	22.840	59.110	13.400	40.690	223.410
150	alpha	1	60x8 weeks	Ag80	Daily	VAC50	50	Unvaccinate	2.442	0.008	0.005	0.012	0.003	0.008	0.047	1172.273	3.627	2.284	5.911	1.340	4.069	22.341	11723	36.270	22.840	59.110	13.400	40.690	223.410
150	alpha	1	60x8 weeks	Ag80	Daily	VAC50	50	Unvaccinate	2.442	0.008	0.005	0.012	0.003	0.008	0.047	1172.273	3.627	2.284	5.911	1.340	4.069	22.341	11723	36.270	22.840	59.110	13.400	40.690	223.410
150	alpha	1.2	60x8 weeks	Ag80	Daily	VAC50	50	Unvaccinate	2.438	0.007	0.006	0.013	0.003	0.009	0.047	1170.456	3.557	2.863	6.420	1.452	4.374	22.423	11705	35.570	28.630	64.200	14.520	43.740	224.230
150	alpha	1.2	60x8 weeks	Ag80	Daily	VAC50	50	Unvaccinate	2.438	0.007	0.006	0.013	0.003	0.009	0.047	1170.456	3.557	2.863	6.420	1.452	4.374	22.423	11705	35.570	28.630	64.200	14.520	43.740	224.230
150	alpha	1.2	60x8 weeks	Ag80	Daily	VAC50	50	Unvaccinate	2.438	0.007	0.006	0.013	0.003	0.009	0.047	1170.456	3.557	2.863	6.420	1.452	4.374	22.423	11705	35.570	28.630	64.200	14.520	43.740	224.230
150	delta	0.6	60x8 weeks	Ag80	Daily	VAC50	50	Unvaccinate	2.427	0.008	0.010	0.018	0.005	0.011	0.046	1164.757	3.873	4.628	8.501	2.402	5.146	22.041	11648	38.730	46.280	85.010	24.020	51.460	220.410
150	delta	0.6	60x8 weeks	Ag80	Daily	VAC50	50	Unvaccinate	2.427	0.008	0.010	0.018	0.005	0.011	0.046	1164.757	3.873	4.628	8.501	2.402	5.146	22.041	11648	38.730	46.280	85.010	24.020	51.460	220.410
150	delta	0.6	60x8 weeks	Ag80	Daily	VAC50	50	Unvaccinate	2.427	0.008	0.010	0.018	0.005	0.011	0.046	1164.757	3.873	4.628	8.501	2.402	5.146	22.041	11648	38.730	46.280	85.010	24.020	51.460	220.410
150	delta	1	60x8 weeks	Ag80	Daily	VAC50	50	Unvaccinate	2.345	0.007	0.030	0.038	0.011	0.022	0.042	1125.768	3.544	14.461	18.005	5.243	10.780	20.345	11258	35.440	144.610	180.050	52.430	107.800	203.450
150	delta	1	60x8 weeks	Ag80	Daily	VAC50	50	Unvaccinate	2.345	0.007	0.030	0.038	0.011	0.022	0.042	1125.768	3.544	14.461	18.005	5.243	10.780	20.345	11258	35.440	144.610	180.050	52.430	107.800	203.450
150	delta	1	60x8 weeks	Ag80	Daily	VAC50	50	Unvaccinate	2.345	0.007	0.030	0.038	0.011	0.022	0.042	1125.768	3.544	14.461	18.005	5.243	10.780	20.345	11258	35.440	144.610	180.050	52.430	107.800	203.450
150	delta	1.2	60x8 weeks	Ag80	Daily	VAC50	50	Unvaccinate	2.279	0.007	0.047	0.054	0.016	0.031	0.038	1094.116	3.361	22.339	25.700	7.883	15.011	18.160	10941	33.610	223.390	257.000	78.830	150.110	181.600
150	delta	1.2	60x8 weeks	Ag80	Daily	VAC50	50	Unvaccinate	2.279	0.007	0.047	0.054	0.016	0.031	0.038	1094.116	3.361	22.339	25.700	7.883	15.011	18.160	10941	33.610	223.390	257.000	78.830	150.110	181.600
150	delta	1.2	60x8 weeks	Ag80	Daily	VAC50	50	Unvaccinate	2.279	0.007	0.047	0.054	0.016	0.031	0.038	1094.116	3.361	22.339	25.700	7.883	15.011	18.160	10941	33.610	223.390	257.000	78.830	150.110	181.600
150	original	0.6	60x8 weeks	Ag80	Daily	VAC50	75	Unvaccinate	1.232	0.007	0.000	0.007	0.002	0.003	0.024	591.270	3.124	0.114	3.238	1.081	1.313	11.651	5913	31.240	1.140	32.380	10.810	13.130	116.510
150	original	0.6	60x8 weeks	Ag80	Daily	VAC50	75	Unvaccinate	1.232	0.007	0.000	0.007	0.002	0.003	0.024	591.270	3.124	0.114	3.238	1.081	1.313	11.651	5913	31.240	1.140	32.380	10.810	13.130	116.510
150	original	0.6	60x8 weeks	Ag80	Daily	VAC50	75	Unvaccinate	1.232	0.007	0.000	0.007	0.002	0.003	0.024	591.270	3.124	0.114	3.238	1.081	1.313	11.651	5913	31.240	1.140	32.380	10.810	13.130	116.510
150	original	1	60x8 weeks	Ag80	Daily	VAC50	75	Unvaccinate	1.232	0.006	0.000	0.007	0.002	0.003	0.024	591.317	3.111	0.224	3.335	1.079	1.295	11.659	5913	31.110	2.240	33.350	10.790	12.950	116.590
150	original	1	60x8 weeks	Ag80	Daily	VAC50	75	Unvaccinate	1.232	0.006	0.000	0.007	0.002	0.003	0.024	591.317	3.111	0.224	3.335	1.079	1.295	11.659	5913	31.110	2.240	33.350	10.790	12.950	116.590
150	original	1	60x8 weeks	Ag80	Daily	VAC50	75	Unvaccinate	1.232	0.006	0.000	0.007	0.002	0.003	0.024	591.317	3.111	0.224	3.335	1.079	1.295	11.659	5913	31.110	2.240	33.350	10.790	12.950	116.590
150	original	1.2	60x8 weeks	Ag80	Daily	VAC50	75	Unvaccinate	1.232	0.006	0.001	0.007	0.002	0.003	0.024	591.443	3.102	0.245	3.347	1.090	1.292	11.365	5914	31.020	2.450	33.470	10.900	12.920	113.650

150	original	1.2	60x8 weeks	Ag80	Daily	VAC50	75	Unvaccinate	1.232	0.006	0.001	0.007	0.002	0.003	0.024	591.443	3.102	0.245	3.347	1.090	1.292	11.365	5914	31.020	2.450	33.470	10.900	12.920	113.650
150	original	1.2	60x8 weeks	Ag80	Daily	VAC50	75	Unvaccinate	1.232	0.006	0.001	0.007	0.002	0.003	0.024	591.443	3.102	0.245	3.347	1.090	1.292	11.365	5914	31.020	2.450	33.470	10.900	12.920	113.650
150	alpha	0.6	60x8 weeks	Ag80	Daily	VAC50	75	Unvaccinate	1.226	0.006	0.002	0.009	0.003	0.003	0.024	588.626	3.117	0.974	4.091	1.577	1.675	11.392	5886	31.170	9.740	40.910	15.770	16.750	113.920
150	alpha	0.6	60x8 weeks	Ag80	Daily	VAC50	75	Unvaccinate	1.226	0.006	0.002	0.009	0.003	0.003	0.024	588.626	3.117	0.974	4.091	1.577	1.675	11.392	5886	31.170	9.740	40.910	15.770	16.750	113.920
150	alpha	0.6	60x8 weeks	Ag80	Daily	VAC50	75	Unvaccinate	1.226	0.006	0.002	0.009	0.003	0.003	0.024	588.626	3.117	0.974	4.091	1.577	1.675	11.392	5886	31.170	9.740	40.910	15.770	16.750	113.920
150	alpha	1	60x8 weeks	Ag80	Daily	VAC50	75	Unvaccinate	1.220	0.006	0.005	0.011	0.004	0.004	0.024	585.482	3.108	2.210	5.318	2.112	2.108	11.298	5855	31.080	22.100	53.180	21.120	21.080	112.980
150	alpha	1	60x8 weeks	Ag80	Daily	VAC50	75	Unvaccinate	1.220	0.006	0.005	0.011	0.004	0.004	0.024	585.482	3.108	2.210	5.318	2.112	2.108	11.298	5855	31.080	22.100	53.180	21.120	21.080	112.980
150	alpha	1	60x8 weeks	Ag80	Daily	VAC50	75	Unvaccinate	1.220	0.006	0.005	0.011	0.004	0.004	0.024	585.482	3.108	2.210	5.318	2.112	2.108	11.298	5855	31.080	22.100	53.180	21.120	21.080	112.980
150	alpha	1.2	60x8 weeks	Ag80	Daily	VAC50	75	Unvaccinate	1.216	0.006	0.006	0.013	0.005	0.005	0.023	583.732	3.094	3.049	6.143	2.425	2.398	11.163	5837	30.940	30.490	61.430	24.250	23.980	111.630
150	alpha	1.2	60x8 weeks	Ag80	Daily	VAC50	75	Unvaccinate	1.216	0.006	0.006	0.013	0.005	0.005	0.023	583.732	3.094	3.049	6.143	2.425	2.398	11.163	5837	30.940	30.490	61.430	24.250	23.980	111.630
150	alpha	1.2	60x8 weeks	Ag80	Daily	VAC50	75	Unvaccinate	1.216	0.006	0.006	0.013	0.005	0.005	0.023	583.732	3.094	3.049	6.143	2.425	2.398	11.163	5837	30.940	30.490	61.430	24.250	23.980	111.630
150	delta	0.6	60x8 weeks	Ag80	Daily	VAC50	75	Unvaccinate	1.206	0.007	0.012	0.020	0.009	0.006	0.023	578.980	3.593	5.834	9.427	4.262	3.026	10.917	5790	35.930	58.340	94.270	42.620	30.260	109.170
150	delta	0.6	60x8 weeks	Ag80	Daily	VAC50	75	Unvaccinate	1.206	0.007	0.012	0.020	0.009	0.006	0.023	578.980	3.593	5.834	9.427	4.262	3.026	10.917	5790	35.930	58.340	94.270	42.620	30.260	109.170
150	delta	0.6	60x8 weeks	Ag80	Daily	VAC50	75	Unvaccinate	1.206	0.007	0.012	0.020	0.009	0.006	0.023	578.980	3.593	5.834	9.427	4.262	3.026	10.917	5790	35.930	58.340	94.270	42.620	30.260	109.170
150	delta	1	60x8 weeks	Ag80	Daily	VAC50	75	Unvaccinate	1.149	0.007	0.040	0.046	0.021	0.015	0.020	551.708	3.220	18.979	22.199	10.169	7.007	9.506	5517	32.200	189.790	221.990	101.690	70.070	95.060
150	delta	1	60x8 weeks	Ag80	Daily	VAC50	75	Unvaccinate	1.149	0.007	0.040	0.046	0.021	0.015	0.020	551.708	3.220	18.979	22.199	10.169	7.007	9.506	5517	32.200	189.790	221.990	101.690	70.070	95.060
150	delta	1	60x8 weeks	Ag80	Daily	VAC50	75	Unvaccinate	1.149	0.007	0.040	0.046	0.021	0.015	0.020	551.708	3.220	18.979	22.199	10.169	7.007	9.506	5517	32.200	189.790	221.990	101.690	70.070	95.060
150	delta	1.2	60x8 weeks	Ag80	Daily	VAC50	75	Unvaccinate	1.108	0.006	0.061	0.067	0.031	0.020	0.017	531.680	2.890	29.178	32.068	15.099	9.660	8.138	5317	28.900	291.780	320.680	150.990	96.600	81.380
150	delta	1.2	60x8 weeks	Ag80	Daily	VAC50	75	Unvaccinate	1.108	0.006	0.061	0.067	0.031	0.020	0.017	531.680	2.890	29.178	32.068	15.099	9.660	8.138	5317	28.900	291.780	320.680	150.990	96.600	81.380
150	delta	1.2	60x8 weeks	Ag80	Daily	VAC50	75	Unvaccinate	1.108	0.006	0.061	0.067	0.031	0.020	0.017	531.680	2.890	29.178	32.068	15.099	9.660	8.138	5317	28.900	291.780	320.680	150.990	96.600	81.380
150	original	0.6	60x8 weeks	Ag80	Daily	VAC50	100	Unvaccinate	0.000	0.005	0.000	0.005	0.003	0.000	0.000	0.000	2.572	0.041	2.613	1.354	0.000	0.000	0	25.720	0.410	26.130	13.540	0.000	0.000
150	original	0.6	60x8 weeks	Ag80	Daily	VAC50	100	Unvaccinate	0.000	0.005	0.000	0.005	0.003	0.000	0.000	0.000	2.572	0.041	2.613	1.354	0.000	0.000	0	25.720	0.410	26.130	13.540	0.000	0.000
150	original	0.6	60x8 weeks	Ag80	Daily	VAC50	100	Unvaccinate	0.000	0.005	0.000	0.005	0.003	0.000	0.000	0.000	2.572	0.041	2.613	1.354	0.000	0.000	0	25.720	0.410	26.130	13.540	0.000	0.000
150	original	1	60x8 weeks	Ag80	Daily	VAC50	100	Unvaccinate	0.000	0.005	0.000	0.005	0.003	0.000	0.000	0.000	2.510	0.070	2.580	1.361	0.000	0.000	0	25.100	0.700	25.800	13.610	0.000	0.000
150	original	1	60x8 weeks	Ag80	Daily	VAC50	100	Unvaccinate	0.000	0.005	0.000	0.005	0.003	0.000	0.000	0.000	2.510	0.070	2.580	1.361	0.000	0.000	0	25.100	0.700	25.800	13.610	0.000	0.000
150	original	1	60x8 weeks	Ag80	Daily	VAC50	100	Unvaccinate	0.000	0.005	0.000	0.005	0.003	0.000	0.000	0.000	2.510	0.070	2.580	1.361	0.000	0.000	0	25.100	0.700	25.800	13.610	0.000	0.000
150	original	1.2	60x8 weeks	Ag80	Daily	VAC50	100	Unvaccinate	0.000	0.005	0.000	0.006	0.003	0.000	0.000	0.000	2.595	0.071	2.666	1.407	0.000	0.000	0	25.950	0.710	26.660	14.070	0.000	0.000
150	original	1.2	60x8 weeks	Ag80	Daily	VAC50	100	Unvaccinate	0.000	0.005	0.000	0.006	0.003	0.000	0.000	0.000	2.595	0.071	2.666	1.407	0.000	0.000	0	25.950	0.710	26.660	14.070	0.000	0.000
150	original	1.2	60x8 weeks	Ag80	Daily	VAC50	100	Unvaccinate	0.000	0.005	0.000	0.006	0.003	0.000	0.000	0.000	2.595	0.071	2.666	1.407	0.000	0.000	0	25.950	0.710	26.660	14.070	0.000	0.000
150	alpha	0.6	60x8 weeks	Ag80	Daily	VAC50	100	Unvaccinate	0.000	0.005	0.002	0.007	0.004	0.000	0.000	0.000	2.430	0.960	3.390	2.055	0.000	0.000	0	24.300	9.600	33.900	20.550	0.000	0.000
150	alpha	0.6	60x8 weeks	Ag80	Daily	VAC50	100	Unvaccinate	0.000	0.005	0.002	0.007	0.004	0.000	0.000	0.000	2.430	0.960	3.390	2.055	0.000	0.000	0	24.300	9.600	33.900	20.550	0.000	0.000
150	alpha	0.6	60x8 weeks	Ag80	Daily	VAC50	100	Unvaccinate	0.000	0.005	0.002	0.007	0.004	0.000	0.000	0.000	2.430	0.960	3.390	2.055	0.000	0.000	0	24.300	9.600	33.900	20.550	0.000	0.000
150	alpha	1	60x8 weeks	Ag80	Daily	VAC50	100	Unvaccinate	0.000	0.005	0.004	0.009	0.006	0.000	0.000	0.000	2.497	1.898	4.395	2.696	0.000	0.000	0	24.970	18.980	43.950	26.960	0.000	0.000
150	alpha	1	60x8 weeks	Ag80	Daily	VAC50	100	Unvaccinate	0.000	0.005	0.004	0.009	0.006	0.000	0.000	0.000	2.497	1.898	4.395	2.696	0.000	0.000	0	24.970	18.980	43.950	26.960	0.000	0.000
150	alpha	1	60x8 weeks	Ag80	Daily	VAC50	100	Unvaccinate	0.000	0.005	0.004	0.009	0.006	0.000	0.000	0.000	2.497	1.898	4.395	2.696	0.000	0.000	0	24.970	18.980	43.950	26.960	0.000	0.000
150	alpha	1.2	60x8 weeks	Ag80	Daily	VAC50	100	Unvaccinate	0.000	0.005	0.005	0.010	0.006	0.000	0.000	0.000	2.459	2.555	5.014	3.069	0.000	0.000	0	24.590	25.550	50.140	30.690	0.000	0.000
150	alpha	1.2	60x8 weeks	Ag80	Daily	VAC50	100	Unvaccinate	0.000	0.005	0.005	0.010	0.006	0.000	0.000	0.000	2.459	2.555	5.014	3.069	0.000	0.000	0	24.590	25.550	50.140	30.690	0.000	0.000
150	alpha	1.2	60x8 weeks	Ag80	Daily	VAC50	100	Unvaccinate	0.000	0.005	0.005	0.010	0.006	0.000	0.000	0.000	2.459	2.555	5.014	3.069	0.000	0.000	0	24.590	25.550	50.140	30.690	0.000	0.000
150	delta	0.6	60x8 weeks	Ag80	Daily	VAC50	100	Unvaccinate	0.000	0.007	0.014	0.021	0.013	0.000	0.000	0.000	3.199	6.857	10.056	6.331	0.000	0.000	0	31.990	68.570	100.560	63.310	0.000	0.000
150	delta	0.6	60x8 weeks	Ag80	Daily	VAC50	100	Unvaccinate	0.000	0.007	0.014	0.021	0.013	0.000	0.000	0.000	3.199	6.857	10.056	6.331	0.000	0.000	0	31.990	68.570	100.560	63.310	0.000	0.000
150	delta	0.6	60x8 weeks	Ag80	Daily	VAC50	100	Unvaccinate	0.000	0.007	0.014	0.021	0.013	0.000	0.000	0.000	3.199	6.857	10.056	6.331	0.000	0.000	0	31.990	68.570	100.560	63.310	0.000	0.000
150	delta	1	60x8 weeks	Ag80	Daily	VAC50	100	Unvaccinate	0.000	0.006	0.048	0.053	0.035	0.000	0.000	0.000	2.760	22.916	25.676	16.634	0.000	0.000	0	27.600	229.160	256.760	166.340	0.000	0.000
150	delta	1	60x8 weeks	Ag80	Daily	VAC50	100	Unvaccinate	0.000	0.006	0.048	0.053	0.035	0.000	0.000	0.000	2.760	22.916	25.676	16.634	0.000	0.000	0	27.600	229.160	256.760	166.340	0.000	0.000
150	delta	1	60x8 weeks	Ag80	Daily	VAC50	100	Unvaccinate	0.000	0.006	0.048	0.053	0.035	0.000	0.000	0.000	2.760	22.916	25.676	16.634	0.000	0.000	0	27.600	229.160	256.760	166.340	0.000	0.000
150	delta	1.2	60x8 weeks	Ag80	Daily	VAC50	100	Unvaccinate	0.000	0.005	0.072	0.077	0.051	0.000	0.000	0.000	2.370	34.636	37.006	24.246	0.000	0.000	0	23.700	346.360	370.060	242.460	0.000	0.000
150	delta	1.2	60x8 weeks	Ag80	Daily	VAC50	100	Unvaccinate	0.000	0.005	0.072	0.077	0.051	0.000	0.000	0.000	2.370	34.636	37.006	24.246	0.000	0.000	0	23.700	346.360	370.060	242.460	0.000	0.000
150	delta	1.2	60x8 weeks	Ag80	Daily	VAC50	100	Unvaccinate	0.000	0.005	0.072	0.077	0.051	0.000	0.000	0.000	2.370	34.636	37.006	24.246	0.000	0.000	0	23.700	346.360	370.060	242.460	0.000	0.000
150	original	0.6	60x8 weeks	Ag80	MWF	NOVAC	0	Unvaccinate	2.957	0.010	0.002	0.012	0.001	0.011	0.057	1419.491	4.745	0.973	5.718	0.255	5.472	27.483	14195	47.450	9.730	57.180	2.550	54.720	274.830
150	original	0.6	60x8 weeks	Ag80	MWF	NOVAC	0	Unvaccinate	2.957	0.010	0.002	0.012	0.001	0.011	0.057	1419.491	4.745	0.973	5.718	0.255	5.472	27.483	14195	47.450	9.730	57.180	2.550	54.720	274.830
150	original	0.6	60x8 weeks	Ag80	MWF	NOVAC	0	Unvaccinate	2.957	0.010	0.002	0.012	0.001	0.011	0.057	1419.491	4.745	0.973	5.718	0.255	5.472	27.483	14195	47.450	9.730	57.180	2.550	54.720	274.830
150	original	1	60x8 weeks	Ag80	MWF	NOVAC	0	Unvaccinate	2.950	0.010	0.004	0.014	0.001	0.013	0.056	1416.073	4.653	1.941	6.594	0.306	6.300	27.040	14161	46.530	19.410	65.940	3.060	63.000	270.400
150	original	1	60x8 weeks	Ag80	MWF	NOVAC	0	Unvaccinate	2.950	0.010	0.004	0.014	0.001	0.013	0.056	1416.073	4.653	1.941	6.594	0.306	6.300	27.040	14161	46.530	19.410	65.940	3.060	63.000	270.400
150	original	1	60x8 weeks	Ag80	MWF	NOVAC	0	Unvaccinate	2.950	0.010	0.004	0.014	0.001	0.013	0.056	1416.073	4.653	1.941	6.594	0.306	6.300	27.040	14161	46.530	19.410	65.940	3.060	63.000	270.400
150	original	1.2	60x8 weeks	Ag80	MWF	NOVAC	0	Unvaccinate	2.945	0.010	0.005	0.015	0.001	0.015	0.057	1413.574	4.772	2.503	7.275	0.294	6.990	27.263	14136	47.720	25.030	72.750	2.940	69.900	272.630
150	original	1.2	60x8 weeks	Ag80	MWF	NOVAC	0	Unvaccinate	2.945	0.010	0.005	0.015	0.001	0.015	0.057	1413.574	4.772	2.503	7.275	0.294	6.990	27.263	14136	47.720	25.030	72.750	2.940	69.900	272.630
150	original	1.2	60x8 weeks	Ag80	MWF	NOVAC	0	Unvaccinate	2.945	0.010	0.005	0.015	0.001	0.015	0.057	1413.574	4.772	2.503	7.275	0.294	6.990	27.263	14136	47.720	25.030	72.750	2.940	69.900	272.630
150	alpha	0.6	60x8 weeks	Ag80	MWF	NOVAC	0	Unvaccinate	2.949	0.010	0.004	0.014	0.001	0.014	0.057	1415.280	4.768	1.778	6.546	0.279	6.560	27.398	14153	47.680	17.780	65.460	2.790	65.600	273.980
150	alpha	0.6	60x8 weeks	Ag80	MWF	NOVAC	0	Unvaccinate	2.949	0.010	0.004	0.014	0.001	0.014	0.057	1415.280	4.768	1.778	6.546	0.279	6.560	27.398	14153	47.680	17.780	65.460	2.790	65.600	273.980
150	alpha	0.6	60x8 weeks	Ag80	MWF	NOVAC	0	Unvaccinate	2.949	0.010	0.004	0.014	0.001	0.014	0.057	1415.280	4.768	1.778	6.546	0.279	6.560	27.398	14153	47.680	17.780	65.460	2.790	65.600	273.980
150	alpha	1	60x8 weeks	Ag80	MWF	NOVAC	0	Unvaccinate	2.930	0.010	0.009	0.019	0.001	0.019	0.056	1406.192	4.561	4.384	8.945	0.390	8.970	26.994	14062	45.610	43.840	89.450	3.900	89.700	269.940
150	alpha	1	60x8 weeks	Ag80	MWF	NOVAC	0	Unvaccinate	2.930	0.010	0.009	0.019	0.001	0.019	0.056	1406.192	4.561	4.384	8.945	0.390	8.970	26.994	14062	45.610	43.840	89.450	3.900	89.700	269.940
150	alpha	1	60x8 weeks	Ag80	MWF	NOVAC	0	Unvaccinate	2.930	0.010	0.009	0.019	0.001	0.019	0.056	1406.192	4.561	4.384	8.945	0.390	8.970	26.994	14062	45.610	43.840	89.450	3.900	89.700	269.940
150	alpha	1.2	60x8 weeks	Ag80	MWF	NOVAC	0	Unvaccinate	2.915	0.010	0.013	0.022	0.001	0.022	0.054	1398.976	4.582	6.083	10.665	0.483	10.798	26.122	13990	45.820	60.830	106.650	4.830	107.980	261.220
150	alpha	1.2	60x8 weeks	Ag80	MWF	NOVAC	0	Unvaccinate	2.915	0.010	0.013	0.022	0.001	0.022	0.054	1398.976	4.582	6.083	10.665	0.483	10.798	26.122	13990	45.820	60.830	106.650	4.830	107.980	261.220
150	alpha	1.2	60x8 weeks	Ag80	MWF	NOVAC	0	Unvaccinate	2.915	0.010	0.013	0.022	0.001	0.022	0.054	1398.976	4.582	6.083	10.665	0.483	10.798	26.122	13990	45.820	60.830	106.650	4.830	107.980	261.220
150	delta	0.6	60x8 weeks	Ag80	MWF	NOVAC	0	Unvaccinate	2.907	0.010	0.014	0.024	0.002	0.024	0.054	1395.559	4.567	6.862	11.429	0.737	11.385	25.959	13956	45.670	68.620	114.290	7.370	113.850	259.590
150	delta	0.6	60x8 weeks	Ag80	MWF	NOVAC	0	Unvaccinate	2.907	0.010	0.014	0.024	0.002	0.024	0.054	1395.559	4.567	6.862	11.429	0.737	11.385	25.959	13956	45.670	68.620	114.290	7.370	113.850	259.590
150	delta	0.6	60x8 weeks	Ag80	MWF	NOVAC	0	Unvaccinate	2.907	0.010	0.014	0.024	0.002	0.024	0.054	1395.559	4.567	6.862	11.429	0.737	11.385	25.959	13956	45.670	68.620	114.290	7.370	113.850	259.590
150	delta	1	60x8 weeks	Ag80	MWF	NOVAC	0	Unvaccinate	2.779	0.008	0.048	0.056	0.004	0.056	0.046	1333.754	3.857	23.053	26.910	1.823	26.871	22.077	13338	38.570	230.530	269.100	18.230	268.710	220.770
150	delta	1	60x8 weeks	Ag80	MWF	NOVAC	0	Unvaccinate	2.779	0.008	0.048	0.056	0.004	0.056	0.046	1333.754	3.857	23.053	26.910	1.823	26.871	22.077	13338	38.570	230.530	269.100	18.230	268.710	220.770
150	delta	1	60x8 weeks	Ag80	MWF	NOVAC	0	Unvaccinate	2.779	0.008	0.048	0.056	0.004	0.056	0.046	1333.754	3.857	23.053	26.910	1.823	26.871	22.077	13338	38.570	230.530	269.100	18.230	268.710	220.770

150	delta	1.2	60x8 weeks	Ag80	MWF	NOVAC	0	Unvaccinate	2.683	0.007	0.071	0.078	0.005	0.079	0.040	1288.030	3.362	34.209	37.571	2.624	37.809	19.172	12880	33.620	342.090	375.710	26.240	378.090	191.720
150	delta	1.2	60x8 weeks	Ag80	MWF	NOVAC	0	Unvaccinate	2.683	0.007	0.071	0.078	0.005	0.079	0.040	1288.030	3.362	34.209	37.571	2.624	37.809	19.172	12880	33.620	342.090	375.710	26.240	378.090	191.720
150	delta	1.2	60x8 weeks	Ag80	MWF	NOVAC	0	Unvaccinate	2.683	0.007	0.071	0.078	0.005	0.079	0.040	1288.030	3.362	34.209	37.571	2.624	37.809	19.172	12880	33.620	342.090	375.710	26.240	378.090	191.720
150	original	0.6	60x8 weeks	Ag80	MWF	VAC95	25	Unvaccinate	2.219	0.008	0.001	0.009	0.000	0.008	0.043	1065.184	3.705	0.524	4.229	0.221	3.917	20.640	10652	37.050	5.240	42.290	2.210	39.170	206.400
150	original	0.6	60x8 weeks	Ag80	MWF	VAC95	25	Unvaccinate	2.219	0.008	0.001	0.009	0.000	0.008	0.043	1065.184	3.705	0.524	4.229	0.221	3.917	20.640	10652	37.050	5.240	42.290	2.210	39.170	206.400
150	original	0.6	60x8 weeks	Ag80	MWF	VAC95	25	Unvaccinate	2.219	0.008	0.001	0.009	0.000	0.008	0.043	1065.184	3.705	0.524	4.229	0.221	3.917	20.640	10652	37.050	5.240	42.290	2.210	39.170	206.400
150	original	1	60x8 weeks	Ag80	MWF	VAC95	25	Unvaccinate	2.215	0.008	0.002	0.010	0.000	0.009	0.043	1063.215	3.818	1.002	4.820	0.239	4.453	20.588	10632	38.180	10.020	48.200	2.390	44.530	205.880
150	original	1	60x8 weeks	Ag80	MWF	VAC95	25	Unvaccinate	2.215	0.008	0.002	0.010	0.000	0.009	0.043	1063.215	3.818	1.002	4.820	0.239	4.453	20.588	10632	38.180	10.020	48.200	2.390	44.530	205.880
150	original	1	60x8 weeks	Ag80	MWF	VAC95	25	Unvaccinate	2.215	0.008	0.002	0.010	0.000	0.009	0.043	1063.215	3.818	1.002	4.820	0.239	4.453	20.588	10632	38.180	10.020	48.200	2.390	44.530	205.880
150	original	1.2	60x8 weeks	Ag80	MWF	VAC95	25	Unvaccinate	2.213	0.008	0.003	0.011	0.001	0.010	0.043	1062.259	3.660	1.389	5.049	0.253	4.714	20.517	10623	36.600	13.890	50.490	2.530	47.140	205.170
150	original	1.2	60x8 weeks	Ag80	MWF	VAC95	25	Unvaccinate	2.213	0.008	0.003	0.011	0.001	0.010	0.043	1062.259	3.660	1.389	5.049	0.253	4.714	20.517	10623	36.600	13.890	50.490	2.530	47.140	205.170
150	original	1.2	60x8 weeks	Ag80	MWF	VAC95	25	Unvaccinate	2.213	0.008	0.003	0.011	0.001	0.010	0.043	1062.259	3.660	1.389	5.049	0.253	4.714	20.517	10623	36.600	13.890	50.490	2.530	47.140	205.170
150	alpha	0.6	60x8 weeks	Ag80	MWF	VAC95	25	Unvaccinate	2.213	0.008	0.002	0.010	0.001	0.010	0.043	1062.432	3.761	1.054	4.815	0.252	4.697	20.586	10624	37.610	10.540	48.150	2.520	46.970	205.860
150	alpha	0.6	60x8 weeks	Ag80	MWF	VAC95	25	Unvaccinate	2.213	0.008	0.002	0.010	0.001	0.010	0.043	1062.432	3.761	1.054	4.815	0.252	4.697	20.586	10624	37.610	10.540	48.150	2.520	46.970	205.860
150	alpha	0.6	60x8 weeks	Ag80	MWF	VAC95	25	Unvaccinate	2.213	0.008	0.002	0.010	0.001	0.010	0.043	1062.432	3.761	1.054	4.815	0.252	4.697	20.586	10624	37.610	10.540	48.150	2.520	46.970	205.860
150	alpha	1	60x8 weeks	Ag80	MWF	VAC95	25	Unvaccinate	2.206	0.008	0.005	0.012	0.001	0.012	0.043	1058.903	3.602	2.244	5.846	0.379	5.630	20.403	10589	36.020	22.440	58.460	3.790	56.300	204.030
150	alpha	1	60x8 weeks	Ag80	MWF	VAC95	25	Unvaccinate	2.206	0.008	0.005	0.012	0.001	0.012	0.043	1058.903	3.602	2.244	5.846	0.379	5.630	20.403	10589	36.020	22.440	58.460	3.790	56.300	204.030
150	alpha	1	60x8 weeks	Ag80	MWF	VAC95	25	Unvaccinate	2.206	0.008	0.005	0.012	0.001	0.012	0.043	1058.903	3.602	2.244	5.846	0.379	5.630	20.403	10589	36.020	22.440	58.460	3.790	56.300	204.030
150	alpha	1.2	60x8 weeks	Ag80	MWF	VAC95	25	Unvaccinate	2.199	0.007	0.006	0.014	0.001	0.013	0.042	1055.749	3.579	2.954	6.533	0.400	6.362	19.920	10557	35.790	29.540	65.330	4.000	63.620	199.200
150	alpha	1.2	60x8 weeks	Ag80	MWF	VAC95	25	Unvaccinate	2.199	0.007	0.006	0.014	0.001	0.013	0.042	1055.749	3.579	2.954	6.533	0.400	6.362	19.920	10557	35.790	29.540	65.330	4.000	63.620	199.200
150	alpha	1.2	60x8 weeks	Ag80	MWF	VAC95	25	Unvaccinate	2.199	0.007	0.006	0.014	0.001	0.013	0.042	1055.749	3.579	2.954	6.533	0.400	6.362	19.920	10557	35.790	29.540	65.330	4.000	63.620	199.200
150	delta	0.6	60x8 weeks	Ag80	MWF	VAC95	25	Unvaccinate	2.195	0.008	0.007	0.015	0.001	0.014	0.042	1053.484	3.720	3.484	7.204	0.588	6.843	19.990	10535	37.200	34.840	72.040	5.880	68.430	199.900
150	delta	0.6	60x8 weeks	Ag80	MWF	VAC95	25	Unvaccinate	2.195	0.008	0.007	0.015	0.001	0.014	0.042	1053.484	3.720	3.484	7.204	0.588	6.843	19.990	10535	37.200	34.840	72.040	5.880	68.430	199.900
150	delta	0.6	60x8 weeks	Ag80	MWF	VAC95	25	Unvaccinate	2.195	0.008	0.007	0.015	0.001	0.014	0.042	1053.484	3.720	3.484	7.204	0.588	6.843	19.990	10535	37.200	34.840	72.040	5.880	68.430	199.900
150	delta	1	60x8 weeks	Ag80	MWF	VAC95	25	Unvaccinate	2.151	0.007	0.020	0.027	0.002	0.025	0.039	1032.534	3.286	9.760	13.046	1.194	12.201	18.573	10325	32.860	97.600	130.460	11.940	122.010	185.730
150	delta	1	60x8 weeks	Ag80	MWF	VAC95	25	Unvaccinate	2.151	0.007	0.020	0.027	0.002	0.025	0.039	1032.534	3.286	9.760	13.046	1.194	12.201	18.573	10325	32.860	97.600	130.460	11.940	122.010	185.730
150	delta	1	60x8 weeks	Ag80	MWF	VAC95	25	Unvaccinate	2.151	0.007	0.020	0.027	0.002	0.025	0.039	1032.534	3.286	9.760	13.046	1.194	12.201	18.573	10325	32.860	97.600	130.460	11.940	122.010	185.730
150	delta	1.2	60x8 weeks	Ag80	MWF	VAC95	25	Unvaccinate	2.109	0.006	0.032	0.039	0.004	0.036	0.036	1012.271	3.113	15.393	18.506	1.896	17.156	17.357	10123	31.130	153.930	185.060	18.960	171.560	173.570
150	delta	1.2	60x8 weeks	Ag80	MWF	VAC95	25	Unvaccinate	2.109	0.006	0.032	0.039	0.004	0.036	0.036	1012.271	3.113	15.393	18.506	1.896	17.156	17.357	10123	31.130	153.930	185.060	18.960	171.560	173.570
150	delta	1.2	60x8 weeks	Ag80	MWF	VAC95	25	Unvaccinate	2.109	0.006	0.032	0.039	0.004	0.036	0.036	1012.271	3.113	15.393	18.506	1.896	17.156	17.357	10123	31.130	153.930	185.060	18.960	171.560	173.570
150	original	0.6	60x8 weeks	Ag80	MWF	VAC95	50	Unvaccinate	1.480	0.005	0.000	0.006	0.000	0.005	0.029	710.469	2.624	0.219	2.843	0.204	2.516	13.721	7105	26.240	2.190	28.430	2.040	25.160	137.210
150	original	0.6	60x8 weeks	Ag80	MWF	VAC95	50	Unvaccinate	1.480	0.005	0.000	0.006	0.000	0.005	0.029	710.469	2.624	0.219	2.843	0.204	2.516	13.721	7105	26.240	2.190	28.430	2.040	25.160	137.210
150	original	0.6	60x8 weeks	Ag80	MWF	VAC95	50	Unvaccinate	1.480	0.005	0.000	0.006	0.000	0.005	0.029	710.469	2.624	0.219	2.843	0.204	2.516	13.721	7105	26.240	2.190	28.430	2.040	25.160	137.210
150	original	1	60x8 weeks	Ag80	MWF	VAC95	50	Unvaccinate	1.479	0.005	0.001	0.006	0.000	0.006	0.028	709.799	2.568	0.433	3.001	0.206	2.700	13.564	7098	25.680	4.330	30.010	2.060	27.000	135.640
150	original	1	60x8 weeks	Ag80	MWF	VAC95	50	Unvaccinate	1.479	0.005	0.001	0.006	0.000	0.006	0.028	709.799	2.568	0.433	3.001	0.206	2.700	13.564	7098	25.680	4.330	30.010	2.060	27.000	135.640
150	original	1	60x8 weeks	Ag80	MWF	VAC95	50	Unvaccinate	1.479	0.005	0.001	0.006	0.000	0.006	0.028	709.799	2.568	0.433	3.001	0.206	2.700	13.564	7098	25.680	4.330	30.010	2.060	27.000	135.640
150	original	1.2	60x8 weeks	Ag80	MWF	VAC95	50	Unvaccinate	1.478	0.005	0.001	0.006	0.000	0.006	0.029	709.321	2.571	0.543	3.114	0.195	2.826	13.875	7093	25.710	5.430	31.140	1.950	28.260	138.750
150	original	1.2	60x8 weeks	Ag80	MWF	VAC95	50	Unvaccinate	1.478	0.005	0.001	0.006	0.000	0.006	0.029	709.321	2.571	0.543	3.114	0.195	2.826	13.875	7093	25.710	5.430	31.140	1.950	28.260	138.750
150	original	1.2	60x8 weeks	Ag80	MWF	VAC95	50	Unvaccinate	1.478	0.005	0.001	0.006	0.000	0.006	0.029	709.321	2.571	0.543	3.114	0.195	2.826	13.875	7093	25.710	5.430	31.140	1.950	28.260	138.750
150	alpha	0.6	60x8 weeks	Ag80	MWF	VAC95	50	Unvaccinate	1.478	0.006	0.001	0.007	0.001	0.006	0.029	709.342	2.656	0.495	3.151	0.258	2.836	13.733	7093	26.560	4.950	31.510	2.580	28.360	137.330
150	alpha	0.6	60x8 weeks	Ag80	MWF	VAC95	50	Unvaccinate	1.478	0.006	0.001	0.007	0.001	0.006	0.029	709.342	2.656	0.495	3.151	0.258	2.836	13.733	7093	26.560	4.950	31.510	2.580	28.360	137.330
150	alpha	0.6	60x8 weeks	Ag80	MWF	VAC95	50	Unvaccinate	1.478	0.006	0.001	0.007	0.001	0.006	0.029	709.342	2.656	0.495	3.151	0.258	2.836	13.733	7093	26.560	4.950	31.510	2.580	28.360	137.330
150	alpha	1	60x8 weeks	Ag80	MWF	VAC95	50	Unvaccinate	1.474	0.005	0.002	0.007	0.001	0.007	0.028	707.629	2.629	0.959	3.588	0.280	3.312	13.504	7076	26.290	9.590	35.880	2.800	33.120	135.040
150	alpha	1	60x8 weeks	Ag80	MWF	VAC95	50	Unvaccinate	1.474	0.005	0.002	0.007	0.001	0.007	0.028	707.629	2.629	0.959	3.588	0.280	3.312	13.504	7076	26.290	9.590	35.880	2.800	33.120	135.040
150	alpha	1	60x8 weeks	Ag80	MWF	VAC95	50	Unvaccinate	1.474	0.005	0.002	0.007	0.001	0.007	0.028	707.629	2.629	0.959	3.588	0.280	3.312	13.504	7076	26.290	9.590	35.880	2.800	33.120	135.040
150	alpha	1.2	60x8 weeks	Ag80	MWF	VAC95	50	Unvaccinate	1.472	0.006	0.003	0.008	0.001	0.008	0.028	706.477	2.668	1.290	3.958	0.306	3.631	13.623	7065	26.680	12.900	39.580	3.060	36.310	136.230
150	alpha	1.2	60x8 weeks	Ag80	MWF	VAC95	50	Unvaccinate	1.472	0.006	0.003	0.008	0.001	0.008	0.028	706.477	2.668	1.290	3.958	0.306	3.631	13.623	7065	26.680	12.900	39.580	3.060	36.310	136.230
150	alpha	1.2	60x8 weeks	Ag80	MWF	VAC95	50	Unvaccinate	1.472	0.006	0.003	0.008	0.001	0.008	0.028	706.477	2.668	1.290	3.958	0.306	3.631	13.623	7065	26.680	12.900	39.580	3.060	36.310	136.230
150	delta	0.6	60x8 weeks	Ag80	MWF	VAC95	50	Unvaccinate	1.471	0.006	0.003	0.009	0.001	0.008	0.028	705.947	2.713	1.610	4.323	0.544	3.678	13.520	7059	27.130	16.100	43.230	5.440	36.780	135.200
150	delta	0.6	60x8 weeks	Ag80	MWF	VAC95	50	Unvaccinate	1.471	0.006	0.003	0.009	0.001	0.008	0.028	705.947	2.713	1.610	4.323	0.544	3.678	13.520	7059	27.130	16.100	43.230	5.440	36.780	135.200
150	delta	0.6	60x8 weeks	Ag80	MWF	VAC95	50	Unvaccinate	1.471	0.006	0.003	0.009	0.001	0.008	0.028	705.947	2.713	1.610	4.323	0.544	3.678	13.520	7059	27.130	16.100	43.230	5.440	36.780	135.200
150	delta	1	60x8 weeks	Ag80	MWF	VAC95	50	Unvaccinate	1.453	0.006	0.009	0.014	0.002	0.012	0.027	697.224	2.649	4.305	6.954	0.959	5.871	12.906	6972	26.490	43.050	69.540	9.590	58.710	129.060
150	delta	1	60x8 weeks	Ag80	MWF	VAC95	50	Unvaccinate	1.453	0.006	0.009	0.014	0.002	0.012	0.027	697.224	2.649	4.305	6.954	0.959	5.871	12.906	6972	26.490	43.050	69.540	9.590	58.710	129.060
150	delta	1	60x8 weeks	Ag80	MWF	VAC95	50	Unvaccinate	1.453	0.006	0.009	0.014	0.002	0.012	0.027	697.224	2.649	4.305	6.954	0.959	5.871	12.906	6972	26.490	43.050	69.540	9.590	58.710	129.060
150	delta	1.2	60x8 weeks	Ag80	MWF	VAC95	50	Unvaccinate	1.444	0.005	0.012	0.017	0.002	0.015	0.027	692.892	2.597	5.774	8.371	1.137	7.007	12.730	6929	25.970	57.740	83.710	11.370	70.070	127.300
150	delta	1.2	60x8 weeks	Ag80	MWF	VAC95	50	Unvaccinate	1.444	0.005	0.012	0.017	0.002	0.015	0.027	692.892	2.597	5.774	8.371	1.137	7.007	12.730	6929	25.970	57.740	83.710	11.370	70.070	127.300
150	delta	1.2	60x8 weeks	Ag80	MWF	VAC95	50	Unvaccinate	1.444	0.005	0.012	0.017	0.002	0.015	0.027	692.892	2.597	5.774	8.371	1.137	7.007	12.730	6929	25.970	57.740	83.710	11.370	70.070	127.300
150	original	0.6	60x8 weeks	Ag80	MWF	VAC95	75	Unvaccinate	0.741	0.003	0.000	0.003	0.000	0.003	0.014	355.443	1.494	0.080	1.574	0.169	1.204	6.904	3554	14.940	0.800	15.740	1.690	12.040	69.040
150	original	0.6	60x8 weeks	Ag80	MWF	VAC95	75	Unvaccinate	0.741	0.003	0.000	0.003	0.000	0.003	0.014	355.443	1.494	0.080	1.574	0.169	1.204	6.904	3554	14.940	0.800	15.740	1.690	12.040	69.040
150	original	0.6	60x8 weeks	Ag80	MWF	VAC95	75	Unvaccinate	0.741	0.003	0.000	0.003	0.000	0.003	0.014	355.443	1.494	0.080	1.574	0.169	1.204	6.904	3554	14.940	0.800	15.740	1.690	12.040	69.040
150	original	1	60x8 weeks	Ag80	MWF	VAC95	75	Unvaccinate	0.741	0.003	0.000	0.003	0.000	0.002	0.015	355.536	1.441	0.086	1.527	0.177	1.175	6.996	3555	14.410	0.860	15.270	1.770	11.750	69.960
150	original	1	60x8 weeks	Ag80	MWF	VAC95	75	Unvaccinate	0.741	0.003	0.000	0.003	0.000	0.002	0.015	355.536	1.441	0.086	1.527	0.177	1.175	6.996	3555	14.410	0.860	15.270	1.770	11.750	69.960
150	original	1	60x8 weeks	Ag80	MWF	VAC95	75	Unvaccinate	0.741	0.003	0.000	0.003	0.000	0.002	0.015	355.536	1.441	0.086	1.527	0.177	1.175	6.996	3555	14.410	0.860	15.270	1.770	11.750	69.960
150	original	1.2	60x8 weeks	Ag80	MWF	VAC95	75	Unvaccinate	0.740	0.003	0.000	0.003	0.000	0.003	0.015	355.096	1.490	0.152	1.642	0.165	1.295	6.966	3551	14.900	1.520	16.420	1.650	12.950	69.660
150	original	1.2	60x8 weeks	Ag80	MWF	VAC95	75	Unvaccinate	0.740	0.003	0.000	0.003	0.000	0.003	0.015	355.096	1.490	0.152	1.642	0.165	1.295	6.966	3551	14.900	1.520	16.420	1.650	12.950	69.660
150	original	1.2	60x8 weeks	Ag80	MWF	VAC95	75	Unvaccinate	0.740	0.003	0.000	0.003	0.000	0.003	0.015	355.096	1.490	0.152	1.642	0.165	1.295	6.966	3551	14.900	1.520	16.420	1.650	12.950	69.660
150	alpha	0.6	60x8 weeks	Ag80	MWF	VAC95	75	Unvaccinate	0.740	0.003	0.000	0.004	0.000	0.003	0.014	355.070	1.490	0.193	1.683	0.222	1.326	6.930	3551	14.900	1.930	16.830	2.220	13.260	69.300
150	alpha	0.6	60x8 weeks	Ag80	MWF	VAC95	75	Unvaccinate	0.740	0.003	0.000	0.004	0.000	0.003	0.014	355.070	1.490	0.193	1.683	0.222	1.326	6.930	3551	14.900	1.930	16.830	2.220	13.260	69.300
150	alpha	0.6	60x8 weeks	Ag80	MWF	VAC95	75	Unvaccinate	0.740	0.003	0.000	0.004	0.000	0.003	0.014	355.070	1.490	0.193	1.683	0.222	1.326	6.930	3551	14.900	1.930	16.830	2.220	13.260	69.300
150	alpha	1	60x8 weeks	Ag80	MWF	VAC95	75	Unvaccinate	0.738	0.003	0.001	0.004	0.001	0.003	0.014	354.266	1.625	0.323	1.948	0.275	1.523	6.819	3543	16.250	3.230	19.480	2.750	15.230	68.190
150	alpha	1	60x8 weeks	Ag80	MWF	VAC95	75	Unvaccinate	0.738	0.003	0.001	0.004	0.001	0.003	0.014	354.266	1.625	0.323	1.948	0.275	1.523	6.819	3543	16.250	3.230	19.480	2.750	15.230	68.190

150	alpha	1	60x8 weeks	Ag80	MWF	VAC95	75	Jnvaccinate	0.738	0.003	0.001	0.004	0.001	0.003	0.014	354.266	1.625	0.323	1.948	0.275	1.523	6.819	3543	16.250	3.230	19.480	2.750	15.230	68.190
150	alpha	1.2	60x8 weeks	Ag80	MWF	VAC95	75	Jnvaccinate	0.738	0.003	0.001	0.004	0.001	0.003	0.014	354.088	1.609	0.420	2.029	0.287	1.577	6.622	3541	16.090	4.200	20.290	2.870	15.770	66.220
150	alpha	1.2	60x8 weeks	Ag80	MWF	VAC95	75	Jnvaccinate	0.738	0.003	0.001	0.004	0.001	0.003	0.014	354.088	1.609	0.420	2.029	0.287	1.577	6.622	3541	16.090	4.200	20.290	2.870	15.770	66.220
150	alpha	1.2	60x8 weeks	Ag80	MWF	VAC95	75	Jnvaccinate	0.738	0.003	0.001	0.004	0.001	0.003	0.014	354.088	1.609	0.420	2.029	0.287	1.577	6.622	3541	16.090	4.200	20.290	2.870	15.770	66.220
150	delta	0.6	60x8 weeks	Ag80	MWF	VAC95	75	Jnvaccinate	0.736	0.004	0.001	0.005	0.001	0.003	0.015	353.498	1.769	0.657	2.426	0.527	1.666	6.983	3535	17.690	6.570	24.260	5.270	16.660	69.830
150	delta	0.6	60x8 weeks	Ag80	MWF	VAC95	75	Jnvaccinate	0.736	0.004	0.001	0.005	0.001	0.003	0.015	353.498	1.769	0.657	2.426	0.527	1.666	6.983	3535	17.690	6.570	24.260	5.270	16.660	69.830
150	delta	0.6	60x8 weeks	Ag80	MWF	VAC95	75	Jnvaccinate	0.736	0.004	0.001	0.005	0.001	0.003	0.015	353.498	1.769	0.657	2.426	0.527	1.666	6.983	3535	17.690	6.570	24.260	5.270	16.660	69.830
150	delta	1	60x8 weeks	Ag80	MWF	VAC95	75	Jnvaccinate	0.734	0.004	0.003	0.006	0.001	0.004	0.014	352.209	1.741	1.339	3.080	0.653	2.035	6.806	3522	17.410	13.390	30.800	6.530	20.350	68.060
150	delta	1	60x8 weeks	Ag80	MWF	VAC95	75	Jnvaccinate	0.734	0.004	0.003	0.006	0.001	0.004	0.014	352.209	1.741	1.339	3.080	0.653	2.035	6.806	3522	17.410	13.390	30.800	6.530	20.350	68.060
150	delta	1	60x8 weeks	Ag80	MWF	VAC95	75	Jnvaccinate	0.734	0.004	0.003	0.006	0.001	0.004	0.014	352.209	1.741	1.339	3.080	0.653	2.035	6.806	3522	17.410	13.390	30.800	6.530	20.350	68.060
150	delta	1.2	60x8 weeks	Ag80	MWF	VAC95	75	Jnvaccinate	0.732	0.003	0.004	0.007	0.002	0.005	0.014	351.234	1.640	1.779	3.419	0.744	2.282	6.603	3512	16.400	17.790	34.190	7.440	22.820	66.030
150	delta	1.2	60x8 weeks	Ag80	MWF	VAC95	75	Jnvaccinate	0.732	0.003	0.004	0.007	0.002	0.005	0.014	351.234	1.640	1.779	3.419	0.744	2.282	6.603	3512	16.400	17.790	34.190	7.440	22.820	66.030
150	delta	1.2	60x8 weeks	Ag80	MWF	VAC95	75	Jnvaccinate	0.732	0.003	0.004	0.007	0.002	0.005	0.014	351.234	1.640	1.779	3.419	0.744	2.282	6.603	3512	16.400	17.790	34.190	7.440	22.820	66.030
150	original	0.6	60x8 weeks	Ag80	MWF	VAC95	100	Jnvaccinate	0.000	0.001	0.000	0.001	0.000	0.000	0.000	0.000	0.348	0.000	0.348	0.136	0.000	0.000	0	3.480	0.000	3.480	1.360	0.000	0.000
150	original	0.6	60x8 weeks	Ag80	MWF	VAC95	100	Jnvaccinate	0.000	0.001	0.000	0.001	0.000	0.000	0.000	0.000	0.348	0.000	0.348	0.136	0.000	0.000	0	3.480	0.000	3.480	1.360	0.000	0.000
150	original	0.6	60x8 weeks	Ag80	MWF	VAC95	100	Jnvaccinate	0.000	0.001	0.000	0.001	0.000	0.000	0.000	0.000	0.348	0.000	0.348	0.136	0.000	0.000	0	3.480	0.000	3.480	1.360	0.000	0.000
150	original	1	60x8 weeks	Ag80	MWF	VAC95	100	Jnvaccinate	0.000	0.001	0.000	0.001	0.000	0.000	0.000	0.000	0.368	0.001	0.369	0.128	0.000	0.000	0	3.680	0.010	3.690	1.280	0.000	0.000
150	original	1	60x8 weeks	Ag80	MWF	VAC95	100	Jnvaccinate	0.000	0.001	0.000	0.001	0.000	0.000	0.000	0.000	0.368	0.001	0.369	0.128	0.000	0.000	0	3.680	0.010	3.690	1.280	0.000	0.000
150	original	1	60x8 weeks	Ag80	MWF	VAC95	100	Jnvaccinate	0.000	0.001	0.000	0.001	0.000	0.000	0.000	0.000	0.368	0.001	0.369	0.128	0.000	0.000	0	3.680	0.010	3.690	1.280	0.000	0.000
150	original	1.2	60x8 weeks	Ag80	MWF	VAC95	100	Jnvaccinate	0.000	0.001	0.000	0.001	0.000	0.000	0.000	0.000	0.317	0.001	0.318	0.109	0.000	0.000	0	3.170	0.010	3.180	1.090	0.000	0.000
150	original	1.2	60x8 weeks	Ag80	MWF	VAC95	100	Jnvaccinate	0.000	0.001	0.000	0.001	0.000	0.000	0.000	0.000	0.317	0.001	0.318	0.109	0.000	0.000	0	3.170	0.010	3.180	1.090	0.000	0.000
150	original	1.2	60x8 weeks	Ag80	MWF	VAC95	100	Jnvaccinate	0.000	0.001	0.000	0.001	0.000	0.000	0.000	0.000	0.317	0.001	0.318	0.109	0.000	0.000	0	3.170	0.010	3.180	1.090	0.000	0.000
150	alpha	0.6	60x8 weeks	Ag80	MWF	VAC95	100	Jnvaccinate	0.000	0.001	0.000	0.001	0.000	0.000	0.000	0.000	0.463	0.025	0.488	0.235	0.000	0.000	0	4.630	0.250	4.880	2.350	0.000	0.000
150	alpha	0.6	60x8 weeks	Ag80	MWF	VAC95	100	Jnvaccinate	0.000	0.001	0.000	0.001	0.000	0.000	0.000	0.000	0.463	0.025	0.488	0.235	0.000	0.000	0	4.630	0.250	4.880	2.350	0.000	0.000
150	alpha	0.6	60x8 weeks	Ag80	MWF	VAC95	100	Jnvaccinate	0.000	0.001	0.000	0.001	0.000	0.000	0.000	0.000	0.463	0.025	0.488	0.235	0.000	0.000	0	4.630	0.250	4.880	2.350	0.000	0.000
150	alpha	1	60x8 weeks	Ag80	MWF	VAC95	100	Jnvaccinate	0.000	0.001	0.000	0.001	0.000	0.000	0.000	0.000	0.424	0.029	0.453	0.228	0.000	0.000	0	4.240	0.290	4.530	2.280	0.000	0.000
150	alpha	1	60x8 weeks	Ag80	MWF	VAC95	100	Jnvaccinate	0.000	0.001	0.000	0.001	0.000	0.000	0.000	0.000	0.424	0.029	0.453	0.228	0.000	0.000	0	4.240	0.290	4.530	2.280	0.000	0.000
150	alpha	1	60x8 weeks	Ag80	MWF	VAC95	100	Jnvaccinate	0.000	0.001	0.000	0.001	0.000	0.000	0.000	0.000	0.424	0.029	0.453	0.228	0.000	0.000	0	4.240	0.290	4.530	2.280	0.000	0.000
150	alpha	1.2	60x8 weeks	Ag80	MWF	VAC95	100	Jnvaccinate	0.000	0.001	0.000	0.001	0.001	0.000	0.000	0.000	0.483	0.064	0.547	0.250	0.000	0.000	0	4.830	0.640	5.470	2.500	0.000	0.000
150	alpha	1.2	60x8 weeks	Ag80	MWF	VAC95	100	Jnvaccinate	0.000	0.001	0.000	0.001	0.001	0.000	0.000	0.000	0.483	0.064	0.547	0.250	0.000	0.000	0	4.830	0.640	5.470	2.500	0.000	0.000
150	alpha	1.2	60x8 weeks	Ag80	MWF	VAC95	100	Jnvaccinate	0.000	0.001	0.000	0.001	0.001	0.000	0.000	0.000	0.483	0.064	0.547	0.250	0.000	0.000	0	4.830	0.640	5.470	2.500	0.000	0.000
150	delta	0.6	60x8 weeks	Ag80	MWF	VAC95	100	Jnvaccinate	0.000	0.002	0.000	0.002	0.001	0.000	0.000	0.000	0.743	0.165	0.908	0.501	0.000	0.000	0	7.430	1.650	9.080	5.010	0.000	0.000
150	delta	0.6	60x8 weeks	Ag80	MWF	VAC95	100	Jnvaccinate	0.000	0.002	0.000	0.002	0.001	0.000	0.000	0.000	0.743	0.165	0.908	0.501	0.000	0.000	0	7.430	1.650	9.080	5.010	0.000	0.000
150	delta	0.6	60x8 weeks	Ag80	MWF	VAC95	100	Jnvaccinate	0.000	0.002	0.000	0.002	0.001	0.000	0.000	0.000	0.743	0.165	0.908	0.501	0.000	0.000	0	7.430	1.650	9.080	5.010	0.000	0.000
150	delta	1	60x8 weeks	Ag80	MWF	VAC95	100	Jnvaccinate	0.000	0.001	0.001	0.002	0.001	0.000	0.000	0.000	0.675	0.252	0.927	0.504	0.000	0.000	0	6.750	2.520	9.270	5.040	0.000	0.000
150	delta	1	60x8 weeks	Ag80	MWF	VAC95	100	Jnvaccinate	0.000	0.001	0.001	0.002	0.001	0.000	0.000	0.000	0.675	0.252	0.927	0.504	0.000	0.000	0	6.750	2.520	9.270	5.040	0.000	0.000
150	delta	1	60x8 weeks	Ag80	MWF	VAC95	100	Jnvaccinate	0.000	0.001	0.001	0.002	0.001	0.000	0.000	0.000	0.675	0.252	0.927	0.504	0.000	0.000	0	6.750	2.520	9.270	5.040	0.000	0.000
150	delta	1.2	60x8 weeks	Ag80	MWF	VAC95	100	Jnvaccinate	0.000	0.001	0.001	0.002	0.001	0.000	0.000	0.000	0.694	0.336	1.030	0.531	0.000	0.000	0	6.940	3.360	10.300	5.310	0.000	0.000
150	delta	1.2	60x8 weeks	Ag80	MWF	VAC95	100	Jnvaccinate	0.000	0.001	0.001	0.002	0.001	0.000	0.000	0.000	0.694	0.336	1.030	0.531	0.000	0.000	0	6.940	3.360	10.300	5.310	0.000	0.000
150	delta	1.2	60x8 weeks	Ag80	MWF	VAC95	100	Jnvaccinate	0.000	0.001	0.001	0.002	0.001	0.000	0.000	0.000	0.694	0.336	1.030	0.531	0.000	0.000	0	6.940	3.360	10.300	5.310	0.000	0.000
150	original	0.6	60x8 weeks	Ag80	MWF	VAC70	25	Jnvaccinate	2.218	0.009	0.001	0.010	0.001	0.009	0.043	1064.475	4.172	0.623	4.795	0.452	4.096	20.500	10645	41.720	6.230	47.950	4.520	40.960	205.000
150	original	0.6	60x8 weeks	Ag80	MWF	VAC70	25	Jnvaccinate	2.218	0.009	0.001	0.010	0.001	0.009	0.043	1064.475	4.172	0.623	4.795	0.452	4.096	20.500	10645	41.720	6.230	47.950	4.520	40.960	205.000
150	original	0.6	60x8 weeks	Ag80	MWF	VAC70	25	Jnvaccinate	2.218	0.009	0.001	0.010	0.001	0.009	0.043	1064.475	4.172	0.623	4.795	0.452	4.096	20.500	10645	41.720	6.230	47.950	4.520	40.960	205.000
150	original	1	60x8 weeks	Ag80	MWF	VAC70	25	Jnvaccinate	2.216	0.008	0.002	0.011	0.001	0.009	0.043	1063.463	4.055	1.009	5.064	0.457	4.368	20.708	10635	40.550	10.090	50.640	4.570	43.680	207.080
150	original	1	60x8 weeks	Ag80	MWF	VAC70	25	Jnvaccinate	2.216	0.008	0.002	0.011	0.001	0.009	0.043	1063.463	4.055	1.009	5.064	0.457	4.368	20.708	10635	40.550	10.090	50.640	4.570	43.680	207.080
150	original	1	60x8 weeks	Ag80	MWF	VAC70	25	Jnvaccinate	2.216	0.008	0.002	0.011	0.001	0.009	0.043	1063.463	4.055	1.009	5.064	0.457	4.368	20.708	10635	40.550	10.090	50.640	4.570	43.680	207.080
150	original	1.2	60x8 weeks	Ag80	MWF	VAC70	25	Jnvaccinate	2.213	0.008	0.003	0.011	0.001	0.010	0.042	1062.099	4.037	1.448	5.485	0.521	4.677	20.116	10621	40.370	14.480	54.850	5.210	46.770	201.160
150	original	1.2	60x8 weeks	Ag80	MWF	VAC70	25	Jnvaccinate	2.213	0.008	0.003	0.011	0.001	0.010	0.042	1062.099	4.037	1.448	5.485	0.521	4.677	20.116	10621	40.370	14.480	54.850	5.210	46.770	201.160
150	original	1.2	60x8 weeks	Ag80	MWF	VAC70	25	Jnvaccinate	2.213	0.008	0.003	0.011	0.001	0.010	0.042	1062.099	4.037	1.448	5.485	0.521	4.677	20.116	10621	40.370	14.480	54.850	5.210	46.770	201.160
150	alpha	0.6	60x8 weeks	Ag80	MWF	VAC70	25	Jnvaccinate	2.212	0.008	0.003	0.011	0.001	0.010	0.042	1061.790	3.968	1.406	5.374	0.522	4.871	20.237	10618	39.680	14.060	53.740	5.220	48.710	202.370
150	alpha	0.6	60x8 weeks	Ag80	MWF	VAC70	25	Jnvaccinate	2.212	0.008	0.003	0.011	0.001	0.010	0.042	1061.790	3.968	1.406	5.374	0.522	4.871	20.237	10618	39.680	14.060	53.740	5.220	48.710	202.370
150	alpha	0.6	60x8 weeks	Ag80	MWF	VAC70	25	Jnvaccinate	2.212	0.008	0.003	0.011	0.001	0.010	0.042	1061.790	3.968	1.406	5.374	0.522	4.871	20.237	10618	39.680	14.060	53.740	5.220	48.710	202.370
150	alpha	1	60x8 weeks	Ag80	MWF	VAC70	25	Jnvaccinate	2.200	0.008	0.006	0.015	0.001	0.013	0.042	1056.217	3.971	3.044	7.015	0.680	6.318	20.039	10562	39.710	30.440	70.150	6.800	63.180	200.390
150	alpha	1	60x8 weeks	Ag80	MWF	VAC70	25	Jnvaccinate	2.200	0.008	0.006	0.015	0.001	0.013	0.042	1056.217	3.971	3.044	7.015	0.680	6.318	20.039	10562	39.710	30.440	70.150	6.800	63.180	200.390
150	alpha	1	60x8 weeks	Ag80	MWF	VAC70	25	Jnvaccinate	2.200	0.008	0.006	0.015	0.001	0.013	0.042	1056.217	3.971	3.044	7.015	0.680	6.318	20.039	10562	39.710	30.440	70.150	6.800	63.180	200.390
150	alpha	1.2	60x8 weeks	Ag80	MWF	VAC70	25	Jnvaccinate	2.192	0.008	0.009	0.017	0.002	0.015	0.041	1052.235	3.908	4.242	8.150	0.842	7.343	19.530	10522	39.080	42.420	81.500	8.420	73.430	195.300
150	alpha	1.2	60x8 weeks	Ag80	MWF	VAC70	25	Jnvaccinate	2.192	0.008	0.009	0.017	0.002	0.015	0.041	1052.235	3.908	4.242	8.150	0.842	7.343	19.530	10522	39.080	42.420	81.500	8.420	73.430	195.300
150	alpha	1.2	60x8 weeks	Ag80	MWF	VAC70	25	Jnvaccinate	2.192	0.008	0.009	0.017	0.002	0.015	0.041	1052.235	3.908	4.242	8.150	0.842	7.343	19.530	10522	39.080	42.420	81.500	8.420	73.430	195.300
150	delta	0.6	60x8 weeks	Ag80	MWF	VAC70	25	Jnvaccinate	2.186	0.008	0.011	0.019	0.003	0.017	0.041	1049.278	3.807	5.505	9.312	1.306	7.921	19.861	10493	38.070	55.050	93.120	13.060	79.210	198.610
150	delta	0.6	60x8 weeks	Ag80	MWF	VAC70	25	Jnvaccinate	2.186	0.008	0.011	0.019	0.003	0.017	0.041	1049.278	3.807	5.505	9.312	1.306	7.921	19.861	10493	38.070	55.050	93.120	13.060	79.210	198.610
150	delta	0.6	60x8 weeks	Ag80	MWF	VAC70	25	Jnvaccinate	2.186	0.008	0.011	0.019	0.003	0.017	0.041	1049.278	3.807	5.505	9.312	1.306	7.921	19.861	10493	38.070	55.050	93.120	13.060	79.210	198.610
150	delta	1	60x8 weeks	Ag80	MWF	VAC70	25	Jnvaccinate	2.114	0.007	0.034	0.041	0.006	0.035	0.037	1014.641	3.527	16.217	19.744	3.008	16.621	17.726	10146	35.270	162.170	197.440	30.080	166.210	177.260
150	delta	1	60x8 weeks	Ag80	MWF	VAC70	25	Jnvaccinate	2.114	0.007	0.034	0.041	0.006	0.035	0.037	1014.641	3.527	16.217	19.744	3.008	16.621	17.726	10146	35.270	162.170	197.440	30.080	166.210	177.260
150	delta	1	60x8 weeks	Ag80	MWF	VAC70	25	Jnvaccinate	2.114	0.007	0.034	0.041	0.006	0.035	0.037	1014.641	3.527	16.217	19.744	3.008	16.621	17.726	10146	35.270	162.170	197.440	30.080	166.210	177.260
150	delta	1.2	60x8 weeks	Ag80	MWF	VAC70	25	Jnvaccinate	2.056	0.007	0.052	0.059	0.010	0.049	0.033	986.719	3.227	25.143	28.370	4.662	23.567	15.983	9867	32.270	251.430	283.700	46.620	235.670	159.830
150	delta	1.2	60x8 weeks	Ag80	MWF	VAC70	25	Jnvaccinate	2.056	0.007	0.052	0.059	0.010	0.049	0.033	986.719	3.227	25.143	28.370	4.662	23.567	15.983	9867	32.270	251.430	283.700	46.620	235.670	159.830
150	delta	1.2	60x8 weeks	Ag80	MWF	VAC70	25	Jnvaccinate	2.056	0.007	0.052	0.059	0.010	0.049	0.033	986.719	3.227	25.143	28.370	4.662	23.567	15.983	9867	32.270	251.430	283.700	46.620	235.670	159.830
150	original	0.6	60x8 weeks	Ag80	MWF	VAC70	50	Jnvaccinate	1.480	0.007	0.001	0.007	0.001	0.005	0.028	710.448	3.200	0.298	3.498	0.544	2.534	13.637	7104	32.000	2.980	34.980	5.440	25.340	136.370
150	original	0.6	60x8 weeks	Ag80	MWF	VAC70	50	Jnvaccinate	1.480	0.007	0.001	0.007	0.001	0.005	0.028	710.448	3.200	0.298	3.498	0.544	2.534	13.637	7104	32.000	2.980	34.980	5.440	25.340	136.370
150	original	0.6	60x8 weeks	Ag80	MWF	VAC70	50	Jnvaccinate	1.480	0.007	0.001	0.007	0.001	0.005	0.028	710.448	3.200	0.298	3.498	0.544	2.534	13.637	7104	32.000	2.980	34.980	5.440	25.340	136.370
150	original	1	60x8 weeks	Ag80	MWF	VAC70	50	Jnvaccinate	1.480	0.007	0.001	0.008	0.001	0.005	0.029	710.254	3.168	0.518	3.686	0.578	2.608	13.753	7103	31.680	5.180	36.860	5.780	26.080	137.530

150	original	1	60x8 weeks	Ag80	MWF	VAC70	50	Unvaccinate	1.480	0.007	0.001	0.008	0.001	0.005	0.029	710.254	3.168	0.518	3.686	0.578	2.608	13.753	7103	31.680	5.180	36.860	5.780	26.080	137.530
150	original	1	60x8 weeks	Ag80	MWF	VAC70	50	Unvaccinate	1.480	0.007	0.001	0.008	0.001	0.005	0.029	710.254	3.168	0.518	3.686	0.578	2.608	13.753	7103	31.680	5.180	36.860	5.780	26.080	137.530
150	original	1.2	60x8 weeks	Ag80	MWF	VAC70	50	Unvaccinate	1.478	0.007	0.001	0.008	0.001	0.006	0.028	709.462	3.252	0.653	3.905	0.638	2.782	13.672	7095	32.520	6.530	39.050	6.380	27.820	136.720
150	original	1.2	60x8 weeks	Ag80	MWF	VAC70	50	Unvaccinate	1.478	0.007	0.001	0.008	0.001	0.006	0.028	709.462	3.252	0.653	3.905	0.638	2.782	13.672	7095	32.520	6.530	39.050	6.380	27.820	136.720
150	original	1.2	60x8 weeks	Ag80	MWF	VAC70	50	Unvaccinate	1.478	0.007	0.001	0.008	0.001	0.006	0.028	709.462	3.252	0.653	3.905	0.638	2.782	13.672	7095	32.520	6.530	39.050	6.380	27.820	136.720
150	alpha	0.6	60x8 weeks	Ag80	MWF	VAC70	50	Unvaccinate	1.476	0.006	0.002	0.008	0.002	0.006	0.029	708.624	3.097	0.910	4.007	0.731	3.016	13.694	7086	30.970	9.100	40.070	7.310	30.160	136.940
150	alpha	0.6	60x8 weeks	Ag80	MWF	VAC70	50	Unvaccinate	1.476	0.006	0.002	0.008	0.002	0.006	0.029	708.624	3.097	0.910	4.007	0.731	3.016	13.694	7086	30.970	9.100	40.070	7.310	30.160	136.940
150	alpha	0.6	60x8 weeks	Ag80	MWF	VAC70	50	Unvaccinate	1.476	0.006	0.002	0.008	0.002	0.006	0.029	708.624	3.097	0.910	4.007	0.731	3.016	13.694	7086	30.970	9.100	40.070	7.310	30.160	136.940
150	alpha	1	60x8 weeks	Ag80	MWF	VAC70	50	Unvaccinate	1.470	0.007	0.004	0.011	0.002	0.008	0.028	705.779	3.174	1.873	5.047	0.947	3.740	13.232	7058	31.740	18.730	50.470	9.470	37.400	132.320
150	alpha	1	60x8 weeks	Ag80	MWF	VAC70	50	Unvaccinate	1.470	0.007	0.004	0.011	0.002	0.008	0.028	705.779	3.174	1.873	5.047	0.947	3.740	13.232	7058	31.740	18.730	50.470	9.470	37.400	132.320
150	alpha	1	60x8 weeks	Ag80	MWF	VAC70	50	Unvaccinate	1.470	0.007	0.004	0.011	0.002	0.008	0.028	705.779	3.174	1.873	5.047	0.947	3.740	13.232	7058	31.740	18.730	50.470	9.470	37.400	132.320
150	alpha	1.2	60x8 weeks	Ag80	MWF	VAC70	50	Unvaccinate	1.465	0.007	0.005	0.012	0.002	0.009	0.028	703.178	3.164	2.626	5.790	1.015	4.455	13.317	7032	31.640	26.260	57.900	10.150	44.550	133.170
150	alpha	1.2	60x8 weeks	Ag80	MWF	VAC70	50	Unvaccinate	1.465	0.007	0.005	0.012	0.002	0.009	0.028	703.178	3.164	2.626	5.790	1.015	4.455	13.317	7032	31.640	26.260	57.900	10.150	44.550	133.170
150	alpha	1.2	60x8 weeks	Ag80	MWF	VAC70	50	Unvaccinate	1.465	0.007	0.005	0.012	0.002	0.009	0.028	703.178	3.164	2.626	5.790	1.015	4.455	13.317	7032	31.640	26.260	57.900	10.150	44.550	133.170
150	delta	0.6	60x8 weeks	Ag80	MWF	VAC70	50	Unvaccinate	1.460	0.007	0.008	0.015	0.003	0.010	0.027	700.884	3.401	3.632	7.033	1.601	4.914	13.161	7009	34.010	36.320	70.330	16.010	49.140	131.610
150	delta	0.6	60x8 weeks	Ag80	MWF	VAC70	50	Unvaccinate	1.460	0.007	0.008	0.015	0.003	0.010	0.027	700.884	3.401	3.632	7.033	1.601	4.914	13.161	7009	34.010	36.320	70.330	16.010	49.140	131.610
150	delta	0.6	60x8 weeks	Ag80	MWF	VAC70	50	Unvaccinate	1.460	0.007	0.008	0.015	0.003	0.010	0.027	700.884	3.401	3.632	7.033	1.601	4.914	13.161	7009	34.010	36.320	70.330	16.010	49.140	131.610
150	delta	1	60x8 weeks	Ag80	MWF	VAC70	50	Unvaccinate	1.421	0.006	0.024	0.031	0.008	0.020	0.026	682.176	3.093	11.562	14.655	3.676	9.719	12.248	6822	30.930	115.620	146.550	36.760	97.190	122.480
150	delta	1	60x8 weeks	Ag80	MWF	VAC70	50	Unvaccinate	1.421	0.006	0.024	0.031	0.008	0.020	0.026	682.176	3.093	11.562	14.655	3.676	9.719	12.248	6822	30.930	115.620	146.550	36.760	97.190	122.480
150	delta	1	60x8 weeks	Ag80	MWF	VAC70	50	Unvaccinate	1.421	0.006	0.024	0.031	0.008	0.020	0.026	682.176	3.093	11.562	14.655	3.676	9.719	12.248	6822	30.930	115.620	146.550	36.760	97.190	122.480
150	delta	1.2	60x8 weeks	Ag80	MWF	VAC70	50	Unvaccinate	1.394	0.006	0.036	0.042	0.011	0.027	0.024	669.070	2.903	17.340	20.243	5.451	12.915	11.380	6691	29.030	173.400	202.430	54.510	129.150	113.800
150	delta	1.2	60x8 weeks	Ag80	MWF	VAC70	50	Unvaccinate	1.394	0.006	0.036	0.042	0.011	0.027	0.024	669.070	2.903	17.340	20.243	5.451	12.915	11.380	6691	29.030	173.400	202.430	54.510	129.150	113.800
150	delta	1.2	60x8 weeks	Ag80	MWF	VAC70	50	Unvaccinate	1.394	0.006	0.036	0.042	0.011	0.027	0.024	669.070	2.903	17.340	20.243	5.451	12.915	11.380	6691	29.030	173.400	202.430	54.510	129.150	113.800
150	original	0.6	60x8 weeks	Ag80	MWF	VAC70	75	Unvaccinate	0.741	0.005	0.000	0.005	0.001	0.003	0.015	355.441	2.445	0.117	2.562	0.683	1.214	7.066	3554	24.450	1.170	25.620	6.830	12.140	70.660
150	original	0.6	60x8 weeks	Ag80	MWF	VAC70	75	Unvaccinate	0.741	0.005	0.000	0.005	0.001	0.003	0.015	355.441	2.445	0.117	2.562	0.683	1.214	7.066	3554	24.450	1.170	25.620	6.830	12.140	70.660
150	original	0.6	60x8 weeks	Ag80	MWF	VAC70	75	Unvaccinate	0.741	0.005	0.000	0.005	0.001	0.003	0.015	355.441	2.445	0.117	2.562	0.683	1.214	7.066	3554	24.450	1.170	25.620	6.830	12.140	70.660
150	original	1	60x8 weeks	Ag80	MWF	VAC70	75	Unvaccinate	0.741	0.005	0.000	0.005	0.002	0.003	0.014	355.527	2.427	0.185	2.612	0.741	1.200	6.813	3555	24.270	1.850	26.120	7.410	12.000	68.130
150	original	1	60x8 weeks	Ag80	MWF	VAC70	75	Unvaccinate	0.741	0.005	0.000	0.005	0.002	0.003	0.014	355.527	2.427	0.185	2.612	0.741	1.200	6.813	3555	24.270	1.850	26.120	7.410	12.000	68.130
150	original	1	60x8 weeks	Ag80	MWF	VAC70	75	Unvaccinate	0.741	0.005	0.000	0.005	0.002	0.003	0.014	355.527	2.427	0.185	2.612	0.741	1.200	6.813	3555	24.270	1.850	26.120	7.410	12.000	68.130
150	original	1.2	60x8 weeks	Ag80	MWF	VAC70	75	Unvaccinate	0.740	0.005	0.000	0.005	0.001	0.003	0.014	355.438	2.329	0.222	2.551	0.664	1.208	6.797	3554	23.290	2.220	25.510	6.640	12.080	67.970
150	original	1.2	60x8 weeks	Ag80	MWF	VAC70	75	Unvaccinate	0.740	0.005	0.000	0.005	0.001	0.003	0.014	355.438	2.329	0.222	2.551	0.664	1.208	6.797	3554	23.290	2.220	25.510	6.640	12.080	67.970
150	original	1.2	60x8 weeks	Ag80	MWF	VAC70	75	Unvaccinate	0.740	0.005	0.000	0.005	0.001	0.003	0.014	355.438	2.329	0.222	2.551	0.664	1.208	6.797	3554	23.290	2.220	25.510	6.640	12.080	67.970
150	alpha	0.6	60x8 weeks	Ag80	MWF	VAC70	75	Unvaccinate	0.738	0.005	0.001	0.006	0.002	0.003	0.014	354.295	2.414	0.604	3.018	0.986	1.511	6.771	3543	24.140	6.040	30.180	9.860	15.110	67.710
150	alpha	0.6	60x8 weeks	Ag80	MWF	VAC70	75	Unvaccinate	0.738	0.005	0.001	0.006	0.002	0.003	0.014	354.295	2.414	0.604	3.018	0.986	1.511	6.771	3543	24.140	6.040	30.180	9.860	15.110	67.710
150	alpha	0.6	60x8 weeks	Ag80	MWF	VAC70	75	Unvaccinate	0.738	0.005	0.001	0.006	0.002	0.003	0.014	354.295	2.414	0.604	3.018	0.986	1.511	6.771	3543	24.140	6.040	30.180	9.860	15.110	67.710
150	alpha	1	60x8 weeks	Ag80	MWF	VAC70	75	Unvaccinate	0.736	0.005	0.003	0.007	0.002	0.004	0.014	353.204	2.323	1.218	3.541	1.089	1.820	6.869	3532	23.230	12.180	35.410	10.890	18.200	68.690
150	alpha	1	60x8 weeks	Ag80	MWF	VAC70	75	Unvaccinate	0.736	0.005	0.003	0.007	0.002	0.004	0.014	353.204	2.323	1.218	3.541	1.089	1.820	6.869	3532	23.230	12.180	35.410	10.890	18.200	68.690
150	alpha	1	60x8 weeks	Ag80	MWF	VAC70	75	Unvaccinate	0.736	0.005	0.003	0.007	0.002	0.004	0.014	353.204	2.323	1.218	3.541	1.089	1.820	6.869	3532	23.230	12.180	35.410	10.890	18.200	68.690
150	alpha	1.2	60x8 weeks	Ag80	MWF	VAC70	75	Unvaccinate	0.735	0.005	0.003	0.008	0.003	0.004	0.014	352.804	2.341	1.594	3.935	1.282	1.899	6.803	3528	23.410	15.940	39.350	12.820	18.990	68.030
150	alpha	1.2	60x8 weeks	Ag80	MWF	VAC70	75	Unvaccinate	0.735	0.005	0.003	0.008	0.003	0.004	0.014	352.804	2.341	1.594	3.935	1.282	1.899	6.803	3528	23.410	15.940	39.350	12.820	18.990	68.030
150	alpha	1.2	60x8 weeks	Ag80	MWF	VAC70	75	Unvaccinate	0.735	0.005	0.003	0.008	0.003	0.004	0.014	352.804	2.341	1.594	3.935	1.282	1.899	6.803	3528	23.410	15.940	39.350	12.820	18.990	68.030
150	delta	0.6	60x8 weeks	Ag80	MWF	VAC70	75	Unvaccinate	0.732	0.006	0.006	0.011	0.004	0.005	0.014	351.130	2.758	2.661	5.419	2.015	2.289	6.587	3511	27.580	26.610	54.190	20.150	22.890	65.870
150	delta	0.6	60x8 weeks	Ag80	MWF	VAC70	75	Unvaccinate	0.732	0.006	0.006	0.011	0.004	0.005	0.014	351.130	2.758	2.661	5.419	2.015	2.289	6.587	3511	27.580	26.610	54.190	20.150	22.890	65.870
150	delta	0.6	60x8 weeks	Ag80	MWF	VAC70	75	Unvaccinate	0.732	0.006	0.006	0.011	0.004	0.005	0.014	351.130	2.758	2.661	5.419	2.015	2.289	6.587	3511	27.580	26.610	54.190	20.150	22.890	65.870
150	delta	1	60x8 weeks	Ag80	MWF	VAC70	75	Unvaccinate	0.718	0.005	0.015	0.021	0.008	0.008	0.013	344.823	2.577	7.334	9.911	3.909	3.943	6.208	3448	25.770	73.340	99.110	39.090	39.430	62.080
150	delta	1	60x8 weeks	Ag80	MWF	VAC70	75	Unvaccinate	0.718	0.005	0.015	0.021	0.008	0.008	0.013	344.823	2.577	7.334	9.911	3.909	3.943	6.208	3448	25.770	73.340	99.110	39.090	39.430	62.080
150	delta	1	60x8 weeks	Ag80	MWF	VAC70	75	Unvaccinate	0.718	0.005	0.015	0.021	0.008	0.008	0.013	344.823	2.577	7.334	9.911	3.909	3.943	6.208	3448	25.770	73.340	99.110	39.090	39.430	62.080
150	delta	1.2	60x8 weeks	Ag80	MWF	VAC70	75	Unvaccinate	0.708	0.005	0.024	0.029	0.012	0.011	0.012	339.656	2.456	11.526	13.982	5.644	5.252	5.999	3397	24.560	115.260	139.820	56.440	52.520	59.990
150	delta	1.2	60x8 weeks	Ag80	MWF	VAC70	75	Unvaccinate	0.708	0.005	0.024	0.029	0.012	0.011	0.012	339.656	2.456	11.526	13.982	5.644	5.252	5.999	3397	24.560	115.260	139.820	56.440	52.520	59.990
150	delta	1.2	60x8 weeks	Ag80	MWF	VAC70	75	Unvaccinate	0.708	0.005	0.024	0.029	0.012	0.011	0.012	339.656	2.456	11.526	13.982	5.644	5.252	5.999	3397	24.560	115.260	139.820	56.440	52.520	59.990
150	original	0.6	60x8 weeks	Ag80	MWF	VAC70	100	Unvaccinate	0.000	0.004	0.000	0.004	0.002	0.000	0.000	0.000	1.701	0.010	1.711	0.834	0.000	0.000	0	17.010	0.100	17.110	8.340	0.000	0.000
150	original	0.6	60x8 weeks	Ag80	MWF	VAC70	100	Unvaccinate	0.000	0.004	0.000	0.004	0.002	0.000	0.000	0.000	1.701	0.010	1.711	0.834	0.000	0.000	0	17.010	0.100	17.110	8.340	0.000	0.000
150	original	0.6	60x8 weeks	Ag80	MWF	VAC70	100	Unvaccinate	0.000	0.004	0.000	0.004	0.002	0.000	0.000	0.000	1.701	0.010	1.711	0.834	0.000	0.000	0	17.010	0.100	17.110	8.340	0.000	0.000
150	original	1	60x8 weeks	Ag80	MWF	VAC70	100	Unvaccinate	0.000	0.004	0.000	0.004	0.002	0.000	0.000	0.000	1.686	0.025	1.711	0.864	0.000	0.000	0	16.860	0.250	17.110	8.640	0.000	0.000
150	original	1	60x8 weeks	Ag80	MWF	VAC70	100	Unvaccinate	0.000	0.004	0.000	0.004	0.002	0.000	0.000	0.000	1.686	0.025	1.711	0.864	0.000	0.000	0	16.860	0.250	17.110	8.640	0.000	0.000
150	original	1	60x8 weeks	Ag80	MWF	VAC70	100	Unvaccinate	0.000	0.004	0.000	0.004	0.002	0.000	0.000	0.000	1.686	0.025	1.711	0.864	0.000	0.000	0	16.860	0.250	17.110	8.640	0.000	0.000
150	original	1.2	60x8 weeks	Ag80	MWF	VAC70	100	Unvaccinate	0.000	0.003	0.000	0.003	0.002	0.000	0.000	0.000	1.637	0.025	1.662	0.803	0.000	0.000	0	16.370	0.250	16.620	8.030	0.000	0.000
150	original	1.2	60x8 weeks	Ag80	MWF	VAC70	100	Unvaccinate	0.000	0.003	0.000	0.003	0.002	0.000	0.000	0.000	1.637	0.025	1.662	0.803	0.000	0.000	0	16.370	0.250	16.620	8.030	0.000	0.000
150	original	1.2	60x8 weeks	Ag80	MWF	VAC70	100	Unvaccinate	0.000	0.003	0.000	0.003	0.002	0.000	0.000	0.000	1.637	0.025	1.662	0.803	0.000	0.000	0	16.370	0.250	16.620	8.030	0.000	0.000
150	alpha	0.6	60x8 weeks	Ag80	MWF	VAC70	100	Unvaccinate	0.000	0.003	0.001	0.004	0.002	0.000	0.000	0.000	1.628	0.333	1.961	1.136	0.000	0.000	0	16.280	3.330	19.610	11.360	0.000	0.000
150	alpha	0.6	60x8 weeks	Ag80	MWF	VAC70	100	Unvaccinate	0.000	0.003	0.001	0.004	0.002	0.000	0.000	0.000	1.628	0.333	1.961	1.136	0.000	0.000	0	16.280	3.330	19.610	11.360	0.000	0.000
150	alpha	0.6	60x8 weeks	Ag80	MWF	VAC70	100	Unvaccinate	0.000	0.003	0.001	0.004	0.002	0.000	0.000	0.000	1.628	0.333	1.961	1.136	0.000	0.000	0	16.280	3.330	19.610	11.360	0.000	0.000
150	alpha	1	60x8 weeks	Ag80	MWF	VAC70	100	Unvaccinate	0.000	0.003	0.001	0.005	0.003	0.000	0.000	0.000	1.566	0.691	2.257	1.317	0.000	0.000	0	15.660	6.910	22.570	13.170	0.000	0.000
150	alpha	1	60x8 weeks	Ag80	MWF	VAC70	100	Unvaccinate	0.000	0.003	0.001	0.005	0.003	0.000	0.000	0.000	1.566	0.691	2.257	1.317	0.000	0.000	0	15.660	6.910	22.570	13.170	0.000	0.000
150	alpha	1	60x8 weeks	Ag80	MWF	VAC70	100	Unvaccinate	0.000	0.003	0.001	0.005	0.003	0.000	0.000	0.000	1.566	0.691	2.257	1.317	0.000	0.000	0	15.660	6.910	22.570	13.170	0.000	0.000
150	alpha	1.2	60x8 weeks	Ag80	MWF	VAC70	100	Unvaccinate	0.000	0.003	0.002	0.005	0.003	0.000	0.000	0.000	1.572	0.778	2.350	1.363	0.000	0.000	0	15.720	7.780	23.500	13.630	0.000	0.000
150	alpha	1.2	60x8 weeks	Ag80	MWF	VAC70	100	Unvaccinate	0.000	0.003	0.002	0.005	0.003	0.000	0.000	0.000	1.572	0.778	2.350	1.363	0.000	0.000	0	15.720	7.780	23.500	13.630	0.000	0.000
150	alpha	1.2	60x8 weeks	Ag80	MWF	VAC70	100	Unvaccinate	0.000	0.003	0.002	0.005	0.003	0.000	0.000	0.000	1.572	0.778	2.350	1.363	0.000	0.000	0	15.720	7.780	23.500	13.630	0.000	0.000
150	delta	0.6	60x8 weeks	Ag80	MWF	VAC70	100	Unvaccinate	0.000	0.004	0.004	0.008	0.005	0.000	0.000	0.000	1.978	1.732	3.710	2.286	0.000	0.000	0	19.780	17.320	37.100	22.860	0.000	0.000
150	delta	0.6	60x8 weeks	Ag80	MWF	VAC70	100	Unvaccinate	0.000	0.004	0.004	0.008	0.005	0.000	0.000	0.000	1.978	1.732	3.710	2.286	0.000	0.000	0	19.780	17.320	37.100	22.860	0.000	0.000
150	delta	0.6	60x8 weeks	Ag80	MWF	VAC70	100	Unvaccinate	0.000	0.004	0.004	0.008	0.005	0.000	0.000	0.000	1.978	1.732	3.710	2.286	0.000	0.000	0	19.780	17.320	37.100	22.860	0.000	0.000

150	delta	1	60x8 weeks	Ag80	MWF	VAC70	100	Unvaccinate	0.000	0.004	0.009	0.014	0.008	0.000	0.000	0.000	1.994	4.508	6.502	4.041	0.000	0.000	0	19.940	45.080	65.020	40.410	0.000	0.000
150	delta	1	60x8 weeks	Ag80	MWF	VAC70	100	Unvaccinate	0.000	0.004	0.009	0.014	0.008	0.000	0.000	0.000	1.994	4.508	6.502	4.041	0.000	0.000	0	19.940	45.080	65.020	40.410	0.000	0.000
150	delta	1	60x8 weeks	Ag80	MWF	VAC70	100	Unvaccinate	0.000	0.004	0.009	0.014	0.008	0.000	0.000	0.000	1.994	4.508	6.502	4.041	0.000	0.000	0	19.940	45.080	65.020	40.410	0.000	0.000
150	delta	1.2	60x8 weeks	Ag80	MWF	VAC70	100	Unvaccinate	0.000	0.004	0.015	0.019	0.012	0.000	0.000	0.000	1.961	7.161	9.122	5.536	0.000	0.000	0	19.610	71.610	91.220	55.360	0.000	0.000
150	delta	1.2	60x8 weeks	Ag80	MWF	VAC70	100	Unvaccinate	0.000	0.004	0.015	0.019	0.012	0.000	0.000	0.000	1.961	7.161	9.122	5.536	0.000	0.000	0	19.610	71.610	91.220	55.360	0.000	0.000
150	delta	1.2	60x8 weeks	Ag80	MWF	VAC70	100	Unvaccinate	0.000	0.004	0.015	0.019	0.012	0.000	0.000	0.000	1.961	7.161	9.122	5.536	0.000	0.000	0	19.610	71.610	91.220	55.360	0.000	0.000
150	original	0.6	60x8 weeks	Ag80	MWF	VAC50	25	Unvaccinate	2.220	0.009	0.001	0.010	0.001	0.008	0.043	1065.400	4.215	0.617	4.832	0.561	3.888	20.535	10654	42.150	6.170	48.320	5.610	38.880	205.350
150	original	0.6	60x8 weeks	Ag80	MWF	VAC50	25	Unvaccinate	2.220	0.009	0.001	0.010	0.001	0.008	0.043	1065.400	4.215	0.617	4.832	0.561	3.888	20.535	10654	42.150	6.170	48.320	5.610	38.880	205.350
150	original	0.6	60x8 weeks	Ag80	MWF	VAC50	25	Unvaccinate	2.220	0.009	0.001	0.010	0.001	0.008	0.043	1065.400	4.215	0.617	4.832	0.561	3.888	20.535	10654	42.150	6.170	48.320	5.610	38.880	205.350
150	original	1	60x8 weeks	Ag80	MWF	VAC50	25	Unvaccinate	2.216	0.009	0.002	0.011	0.001	0.009	0.043	1063.557	4.226	1.156	5.382	0.602	4.374	20.623	10636	42.260	11.560	53.820	6.020	43.740	206.230
150	original	1	60x8 weeks	Ag80	MWF	VAC50	25	Unvaccinate	2.216	0.009	0.002	0.011	0.001	0.009	0.043	1063.557	4.226	1.156	5.382	0.602	4.374	20.623	10636	42.260	11.560	53.820	6.020	43.740	206.230
150	original	1	60x8 weeks	Ag80	MWF	VAC50	25	Unvaccinate	2.216	0.009	0.002	0.011	0.001	0.009	0.043	1063.557	4.226	1.156	5.382	0.602	4.374	20.623	10636	42.260	11.560	53.820	6.020	43.740	206.230
150	original	1.2	60x8 weeks	Ag80	MWF	VAC50	25	Unvaccinate	2.212	0.009	0.003	0.012	0.001	0.010	0.043	1061.977	4.217	1.542	5.759	0.657	4.774	20.552	10620	42.170	15.420	57.590	6.570	47.740	205.520
150	original	1.2	60x8 weeks	Ag80	MWF	VAC50	25	Unvaccinate	2.212	0.009	0.003	0.012	0.001	0.010	0.043	1061.977	4.217	1.542	5.759	0.657	4.774	20.552	10620	42.170	15.420	57.590	6.570	47.740	205.520
150	original	1.2	60x8 weeks	Ag80	MWF	VAC50	25	Unvaccinate	2.212	0.009	0.003	0.012	0.001	0.010	0.043	1061.977	4.217	1.542	5.759	0.657	4.774	20.552	10620	42.170	15.420	57.590	6.570	47.740	205.520
150	alpha	0.6	60x8 weeks	Ag80	MWF	VAC50	25	Unvaccinate	2.209	0.009	0.004	0.012	0.002	0.011	0.042	1060.482	4.198	1.771	5.969	0.745	5.165	20.385	10605	41.980	17.710	59.690	7.450	51.650	203.850
150	alpha	0.6	60x8 weeks	Ag80	MWF	VAC50	25	Unvaccinate	2.209	0.009	0.004	0.012	0.002	0.011	0.042	1060.482	4.198	1.771	5.969	0.745	5.165	20.385	10605	41.980	17.710	59.690	7.450	51.650	203.850
150	alpha	0.6	60x8 weeks	Ag80	MWF	VAC50	25	Unvaccinate	2.209	0.009	0.004	0.012	0.002	0.011	0.042	1060.482	4.198	1.771	5.969	0.745	5.165	20.385	10605	41.980	17.710	59.690	7.450	51.650	203.850
150	alpha	1	60x8 weeks	Ag80	MWF	VAC50	25	Unvaccinate	2.195	0.009	0.008	0.017	0.002	0.015	0.042	1053.813	4.114	3.953	8.067	1.022	6.970	20.206	10538	41.140	39.530	80.670	10.220	69.700	202.060
150	alpha	1	60x8 weeks	Ag80	MWF	VAC50	25	Unvaccinate	2.195	0.009	0.008	0.017	0.002	0.015	0.042	1053.813	4.114	3.953	8.067	1.022	6.970	20.206	10538	41.140	39.530	80.670	10.220	69.700	202.060
150	alpha	1	60x8 weeks	Ag80	MWF	VAC50	25	Unvaccinate	2.195	0.009	0.008	0.017	0.002	0.015	0.042	1053.813	4.114	3.953	8.067	1.022	6.970	20.206	10538	41.140	39.530	80.670	10.220	69.700	202.060
150	alpha	1.2	60x8 weeks	Ag80	MWF	VAC50	25	Unvaccinate	2.189	0.008	0.011	0.019	0.003	0.016	0.041	1050.585	4.044	5.223	9.267	1.314	7.769	19.754	10506	40.440	52.230	92.670	13.140	77.690	197.540
150	alpha	1.2	60x8 weeks	Ag80	MWF	VAC50	25	Unvaccinate	2.189	0.008	0.011	0.019	0.003	0.016	0.041	1050.585	4.044	5.223	9.267	1.314	7.769	19.754	10506	40.440	52.230	92.670	13.140	77.690	197.540
150	alpha	1.2	60x8 weeks	Ag80	MWF	VAC50	25	Unvaccinate	2.189	0.008	0.011	0.019	0.003	0.016	0.041	1050.585	4.044	5.223	9.267	1.314	7.769	19.754	10506	40.440	52.230	92.670	13.140	77.690	197.540
150	delta	0.6	60x8 weeks	Ag80	MWF	VAC50	25	Unvaccinate	2.178	0.009	0.014	0.023	0.004	0.018	0.040	1045.624	4.245	6.744	10.989	1.932	8.794	19.271	10456	42.450	67.440	109.890	19.320	87.940	192.710
150	delta	0.6	60x8 weeks	Ag80	MWF	VAC50	25	Unvaccinate	2.178	0.009	0.014	0.023	0.004	0.018	0.040	1045.624	4.245	6.744	10.989	1.932	8.794	19.271	10456	42.450	67.440	109.890	19.320	87.940	192.710
150	delta	0.6	60x8 weeks	Ag80	MWF	VAC50	25	Unvaccinate	2.178	0.009	0.014	0.023	0.004	0.018	0.040	1045.624	4.245	6.744	10.989	1.932	8.794	19.271	10456	42.450	67.440	109.890	19.320	87.940	192.710
150	delta	1	60x8 weeks	Ag80	MWF	VAC50	25	Unvaccinate	2.077	0.007	0.048	0.055	0.011	0.044	0.034	996.921	3.547	22.963	26.510	5.126	20.906	16.482	9969	35.470	229.630	265.100	51.260	209.060	164.820
150	delta	1	60x8 weeks	Ag80	MWF	VAC50	25	Unvaccinate	2.077	0.007	0.048	0.055	0.011	0.044	0.034	996.921	3.547	22.963	26.510	5.126	20.906	16.482	9969	35.470	229.630	265.100	51.260	209.060	164.820
150	delta	1	60x8 weeks	Ag80	MWF	VAC50	25	Unvaccinate	2.077	0.007	0.048	0.055	0.011	0.044	0.034	996.921	3.547	22.963	26.510	5.126	20.906	16.482	9969	35.470	229.630	265.100	51.260	209.060	164.820
150	delta	1.2	60x8 weeks	Ag80	MWF	VAC50	25	Unvaccinate	2.014	0.007	0.068	0.075	0.015	0.059	0.030	966.741	3.219	32.796	36.015	7.138	28.175	14.471	9667	32.190	327.960	360.150	71.380	281.750	144.710
150	delta	1.2	60x8 weeks	Ag80	MWF	VAC50	25	Unvaccinate	2.014	0.007	0.068	0.075	0.015	0.059	0.030	966.741	3.219	32.796	36.015	7.138	28.175	14.471	9667	32.190	327.960	360.150	71.380	281.750	144.710
150	delta	1.2	60x8 weeks	Ag80	MWF	VAC50	25	Unvaccinate	2.014	0.007	0.068	0.075	0.015	0.059	0.030	966.741	3.219	32.796	36.015	7.138	28.175	14.471	9667	32.190	327.960	360.150	71.380	281.750	144.710
150	original	0.6	60x8 weeks	Ag80	MWF	VAC50	50	Unvaccinate	1.480	0.008	0.001	0.009	0.002	0.005	0.028	710.247	3.724	0.386	4.110	0.856	2.566	13.602	7102	37.240	3.860	41.100	8.560	25.660	136.020
150	original	0.6	60x8 weeks	Ag80	MWF	VAC50	50	Unvaccinate	1.480	0.008	0.001	0.009	0.002	0.005	0.028	710.247	3.724	0.386	4.110	0.856	2.566	13.602	7102	37.240	3.860	41.100	8.560	25.660	136.020
150	original	0.6	60x8 weeks	Ag80	MWF	VAC50	50	Unvaccinate	1.480	0.008	0.001	0.009	0.002	0.005	0.028	710.247	3.724	0.386	4.110	0.856	2.566	13.602	7102	37.240	3.860	41.100	8.560	25.660	136.020
150	original	1	60x8 weeks	Ag80	MWF	VAC50	50	Unvaccinate	1.479	0.008	0.001	0.009	0.002	0.006	0.028	709.841	3.676	0.642	4.318	0.897	2.717	13.573	7098	36.760	6.420	43.180	8.970	27.170	135.730
150	original	1	60x8 weeks	Ag80	MWF	VAC50	50	Unvaccinate	1.479	0.008	0.001	0.009	0.002	0.006	0.028	709.841	3.676	0.642	4.318	0.897	2.717	13.573	7098	36.760	6.420	43.180	8.970	27.170	135.730
150	original	1	60x8 weeks	Ag80	MWF	VAC50	50	Unvaccinate	1.479	0.008	0.001	0.009	0.002	0.006	0.028	709.841	3.676	0.642	4.318	0.897	2.717	13.573	7098	36.760	6.420	43.180	8.970	27.170	135.730
150	original	1.2	60x8 weeks	Ag80	MWF	VAC50	50	Unvaccinate	1.479	0.008	0.002	0.009	0.002	0.006	0.029	709.750	3.686	0.770	4.456	0.903	2.777	13.762	7098	36.860	7.700	44.560	9.030	27.770	137.620
150	original	1.2	60x8 weeks	Ag80	MWF	VAC50	50	Unvaccinate	1.479	0.008	0.002	0.009	0.002	0.006	0.029	709.750	3.686	0.770	4.456	0.903	2.777	13.762	7098	36.860	7.700	44.560	9.030	27.770	137.620
150	original	1.2	60x8 weeks	Ag80	MWF	VAC50	50	Unvaccinate	1.479	0.008	0.002	0.009	0.002	0.006	0.029	709.750	3.686	0.770	4.456	0.903	2.777	13.762	7098	36.860	7.700	44.560	9.030	27.770	137.620
150	alpha	0.6	60x8 weeks	Ag80	MWF	VAC50	50	Unvaccinate	1.474	0.008	0.003	0.011	0.003	0.007	0.028	707.582	3.633	1.471	5.104	1.205	3.317	13.583	7076	36.330	14.710	51.040	12.050	33.170	135.830
150	alpha	0.6	60x8 weeks	Ag80	MWF	VAC50	50	Unvaccinate	1.474	0.008	0.003	0.011	0.003	0.007	0.028	707.582	3.633	1.471	5.104	1.205	3.317	13.583	7076	36.330	14.710	51.040	12.050	33.170	135.830
150	alpha	0.6	60x8 weeks	Ag80	MWF	VAC50	50	Unvaccinate	1.474	0.008	0.003	0.011	0.003	0.007	0.028	707.582	3.633	1.471	5.104	1.205	3.317	13.583	7076	36.330	14.710	51.040	12.050	33.170	135.830
150	alpha	1	60x8 weeks	Ag80	MWF	VAC50	50	Unvaccinate	1.466	0.007	0.006	0.014	0.003	0.009	0.028	703.827	3.587	3.053	6.640	1.648	4.267	13.367	7038	35.870	30.530	66.400	16.480	42.670	133.670
150	alpha	1	60x8 weeks	Ag80	MWF	VAC50	50	Unvaccinate	1.466	0.007	0.006	0.014	0.003	0.009	0.028	703.827	3.587	3.053	6.640	1.648	4.267	13.367	7038	35.870	30.530	66.400	16.480	42.670	133.670
150	alpha	1	60x8 weeks	Ag80	MWF	VAC50	50	Unvaccinate	1.466	0.007	0.006	0.014	0.003	0.009	0.028	703.827	3.587	3.053	6.640	1.648	4.267	13.367	7038	35.870	30.530	66.400	16.480	42.670	133.670
150	alpha	1.2	60x8 weeks	Ag80	MWF	VAC50	50	Unvaccinate	1.461	0.007	0.008	0.016	0.004	0.010	0.028	701.406	3.580	3.948	7.528	1.863	4.914	13.284	7014	35.800	39.480	75.280	18.630	49.140	132.840
150	alpha	1.2	60x8 weeks	Ag80	MWF	VAC50	50	Unvaccinate	1.461	0.007	0.008	0.016	0.004	0.010	0.028	701.406	3.580	3.948	7.528	1.863	4.914	13.284	7014	35.800	39.480	75.280	18.630	49.140	132.840
150	alpha	1.2	60x8 weeks	Ag80	MWF	VAC50	50	Unvaccinate	1.461	0.007	0.008	0.016	0.004	0.010	0.028	701.406	3.580	3.948	7.528	1.863	4.914	13.284	7014	35.800	39.480	75.280	18.630	49.140	132.840
150	delta	0.6	60x8 weeks	Ag80	MWF	VAC50	50	Unvaccinate	1.451	0.008	0.014	0.022	0.007	0.013	0.027	696.444	3.818	6.668	10.486	3.205	6.070	13.114	6964	38.180	66.680	104.860	32.050	60.700	131.140
150	delta	0.6	60x8 weeks	Ag80	MWF	VAC50	50	Unvaccinate	1.451	0.008	0.014	0.022	0.007	0.013	0.027	696.444	3.818	6.668	10.486	3.205	6.070	13.114	6964	38.180	66.680	104.860	32.050	60.700	131.140
150	delta	0.6	60x8 weeks	Ag80	MWF	VAC50	50	Unvaccinate	1.451	0.008	0.014	0.022	0.007	0.013	0.027	696.444	3.818	6.668	10.486	3.205	6.070	13.114	6964	38.180	66.680	104.860	32.050	60.700	131.140
150	delta	1	60x8 weeks	Ag80	MWF	VAC50	50	Unvaccinate	1.379	0.007	0.049	0.055	0.018	0.031	0.022	661.788	3.285	23.350	26.635	8.548	14.812	10.787	6618	32.850	233.500	266.350	85.480	148.120	107.870
150	delta	1	60x8 weeks	Ag80	MWF	VAC50	50	Unvaccinate	1.379	0.007	0.049	0.055	0.018	0.031	0.022	661.788	3.285	23.350	26.635	8.548	14.812	10.787	6618	32.850	233.500	266.350	85.480	148.120	107.870
150	delta	1	60x8 weeks	Ag80	MWF	VAC50	50	Unvaccinate	1.379	0.007	0.049	0.055	0.018	0.031	0.022	661.788	3.285	23.350	26.635	8.548	14.812	10.787	6618	32.850	233.500	266.350	85.480	148.120	107.870
150	delta	1.2	60x8 weeks	Ag80	MWF	VAC50	50	Unvaccinate	1.335	0.006	0.070	0.076	0.026	0.041	0.020	640.646	2.847	33.509	36.356	12.407	19.724	9.547	6406	28.470	335.090	363.560	124.070	197.240	95.470
150	delta	1.2	60x8 weeks	Ag80	MWF	VAC50	50	Unvaccinate	1.335	0.006	0.070	0.076	0.026	0.041	0.020	640.646	2.847	33.509	36.356	12.407	19.724	9.547	6406	28.470	335.090	363.560	124.070	197.240	95.470
150	delta	1.2	60x8 weeks	Ag80	MWF	VAC50	50	Unvaccinate	1.335	0.006	0.070	0.076	0.026	0.041	0.020	640.646	2.847	33.509	36.356	12.407	19.724	9.547	6406	28.470	335.090	363.560	124.070	197.240	95.470
150	original	0.6	60x8 weeks	Ag80	MWF	VAC50	75	Unvaccinate	0.741	0.006	0.000	0.007	0.002	0.003	0.014	355.531	3.109	0.160	3.269	1.070	1.201	6.938	3555	31.090	1.600	32.690	10.700	12.010	69.380
150	original	0.6	60x8 weeks	Ag80	MWF	VAC50	75	Unvaccinate	0.741	0.006	0.000	0.007	0.002	0.003	0.014	355.531	3.109	0.160	3.269	1.070	1.201	6.938	3555	31.090	1.600	32.690	10.700	12.010	69.380
150	original	0.6	60x8 weeks	Ag80	MWF	VAC50	75	Unvaccinate	0.741	0.006	0.000	0.007	0.002	0.003	0.014	355.531	3.109	0.160	3.269	1.070	1.201	6.938	3555	31.090	1.600	32.690	10.700	12.010	69.380
150	original	1	60x8 weeks	Ag80	MWF	VAC50	75	Unvaccinate	0.740	0.007	0.001	0.007	0.002	0.003	0.014	355.168	3.185	0.280	3.465	1.155	1.299	6.844	3552	31.850	2.800	34.650	11.550	12.990	68.440
150	original	1	60x8 weeks	Ag80	MWF	VAC50	75	Unvaccinate	0.740	0.007	0.001	0.007	0.002	0.003	0.014	355.168	3.185	0.280	3.465	1.155	1.299	6.844	3552	31.850	2.800	34.650	11.550	12.990	68.440
150	original	1	60x8 weeks	Ag80	MWF	VAC50	75	Unvaccinate	0.740	0.007	0.001	0.007	0.002	0.003	0.014	355.168	3.185	0.280	3.465	1.155	1.299	6.844	3552	31.850	2.800	34.650	11.550	12.990	68.440
150	original	1.2	60x8 weeks	Ag80	MWF	VAC50	75	Unvaccinate	0.740	0.007	0.001	0.007	0.003	0.003	0.014	355.172	3.157	0.343	3.500	1.220	1.278	6.693	3552	31.570	3.430	35.000	12.200	12.780	66.930
150	original	1.2	60x8 weeks	Ag80	MWF	VAC50	75	Unvaccinate	0.740	0.007	0.001	0.007	0.003	0.003	0.014	355.172	3.157	0.343	3.500	1.220	1.278	6.693	3552	31.570	3.430	35.000	12.200	12.780	66.930
150	original	1.2	60x8 weeks	Ag80	MWF	VAC50	75	Unvaccinate	0.740	0.007	0.001	0.007	0.003	0.003	0.014	355.172	3.157	0.343	3.500	1.220	1.278	6.693	3552	31.570	3.430	35.000	12.200	12.780	66.930
150	alpha	0.6	60x8 weeks	Ag80	MWF	VAC50	75	Unvaccinate	0.737	0.006	0.002	0.009	0.003	0.003	0.014	353.976	3.045	1.110	4.155	1.609	1.621	6.847	3540	30.450	11.100	41.550	16.090	16.210	68.470
150	alpha	0.6	60x8 weeks	Ag80	MWF	VAC50	75	Unvaccinate	0.737	0.006	0.002	0.009	0.003	0.003	0.014	353.976	3.045	1.110	4.155	1.609	1.621	6.847	3540	30.450	11.100	41.550	16.090	16.210	68.470

150	alpha	0.6	60x8 weeks	Ag80	MWF	VAC50	75	Jnvaccinate	0.737	0.006	0.002	0.009	0.003	0.003	0.014	353.976	3.045	1.110	4.155	1.609	1.621	6.847	3540	30.450	11.100	41.550	16.090	16.210	68.470
150	alpha	1	60x8 weeks	Ag80	MWF	VAC50	75	Jnvaccinate	0.733	0.006	0.005	0.012	0.005	0.005	0.014	351.966	3.013	2.508	5.521	2.238	2.163	6.716	3520	30.130	25.080	55.210	22.380	21.630	67.160
150	alpha	1	60x8 weeks	Ag80	MWF	VAC50	75	Jnvaccinate	0.733	0.006	0.005	0.012	0.005	0.005	0.014	351.966	3.013	2.508	5.521	2.238	2.163	6.716	3520	30.130	25.080	55.210	22.380	21.630	67.160
150	alpha	1	60x8 weeks	Ag80	MWF	VAC50	75	Jnvaccinate	0.733	0.006	0.005	0.012	0.005	0.005	0.014	351.966	3.013	2.508	5.521	2.238	2.163	6.716	3520	30.130	25.080	55.210	22.380	21.630	67.160
150	alpha	1.2	60x8 weeks	Ag80	MWF	VAC50	75	Jnvaccinate	0.731	0.006	0.007	0.013	0.005	0.005	0.014	350.978	2.933	3.407	6.340	2.589	2.417	6.582	3510	29.330	34.070	63.400	25.890	24.170	65.820
150	alpha	1.2	60x8 weeks	Ag80	MWF	VAC50	75	Jnvaccinate	0.731	0.006	0.007	0.013	0.005	0.005	0.014	350.978	2.933	3.407	6.340	2.589	2.417	6.582	3510	29.330	34.070	63.400	25.890	24.170	65.820
150	alpha	1.2	60x8 weeks	Ag80	MWF	VAC50	75	Jnvaccinate	0.731	0.006	0.007	0.013	0.005	0.005	0.014	350.978	2.933	3.407	6.340	2.589	2.417	6.582	3510	29.330	34.070	63.400	25.890	24.170	65.820
150	delta	0.6	60x8 weeks	Ag80	MWF	VAC50	75	Jnvaccinate	0.725	0.007	0.013	0.021	0.009	0.007	0.013	347.953	3.439	6.414	9.853	4.427	3.139	6.475	3480	34.390	64.140	98.530	44.270	31.390	64.750
150	delta	0.6	60x8 weeks	Ag80	MWF	VAC50	75	Jnvaccinate	0.725	0.007	0.013	0.021	0.009	0.007	0.013	347.953	3.439	6.414	9.853	4.427	3.139	6.475	3480	34.390	64.140	98.530	44.270	31.390	64.750
150	delta	0.6	60x8 weeks	Ag80	MWF	VAC50	75	Jnvaccinate	0.725	0.007	0.013	0.021	0.009	0.007	0.013	347.953	3.439	6.414	9.853	4.427	3.139	6.475	3480	34.390	64.140	98.530	44.270	31.390	64.750
150	delta	1	60x8 weeks	Ag80	MWF	VAC50	75	Jnvaccinate	0.689	0.006	0.047	0.054	0.025	0.016	0.011	330.480	3.055	22.736	25.791	12.181	7.547	5.382	3305	30.550	227.360	257.910	121.810	75.470	53.820
150	delta	1	60x8 weeks	Ag80	MWF	VAC50	75	Jnvaccinate	0.689	0.006	0.047	0.054	0.025	0.016	0.011	330.480	3.055	22.736	25.791	12.181	7.547	5.382	3305	30.550	227.360	257.910	121.810	75.470	53.820
150	delta	1	60x8 weeks	Ag80	MWF	VAC50	75	Jnvaccinate	0.689	0.006	0.047	0.054	0.025	0.016	0.011	330.480	3.055	22.736	25.791	12.181	7.547	5.382	3305	30.550	227.360	257.910	121.810	75.470	53.820
150	delta	1.2	60x8 weeks	Ag80	MWF	VAC50	75	Jnvaccinate	0.666	0.005	0.070	0.075	0.037	0.021	0.010	319.831	2.590	33.541	36.131	17.586	10.121	4.684	3198	25.900	335.410	361.310	175.860	101.210	46.840
150	delta	1.2	60x8 weeks	Ag80	MWF	VAC50	75	Jnvaccinate	0.666	0.005	0.070	0.075	0.037	0.021	0.010	319.831	2.590	33.541	36.131	17.586	10.121	4.684	3198	25.900	335.410	361.310	175.860	101.210	46.840
150	delta	1.2	60x8 weeks	Ag80	MWF	VAC50	75	Jnvaccinate	0.666	0.005	0.070	0.075	0.037	0.021	0.010	319.831	2.590	33.541	36.131	17.586	10.121	4.684	3198	25.900	335.410	361.310	175.860	101.210	46.840
150	original	0.6	60x8 weeks	Ag80	MWF	VAC50	100	Jnvaccinate	0.000	0.005	0.000	0.005	0.003	0.000	0.000	0.000	2.571	0.036	2.607	1.375	0.000	0.000	0	25.710	0.360	26.070	13.750	0.000	0.000
150	original	0.6	60x8 weeks	Ag80	MWF	VAC50	100	Jnvaccinate	0.000	0.005	0.000	0.005	0.003	0.000	0.000	0.000	2.571	0.036	2.607	1.375	0.000	0.000	0	25.710	0.360	26.070	13.750	0.000	0.000
150	original	0.6	60x8 weeks	Ag80	MWF	VAC50	100	Jnvaccinate	0.000	0.005	0.000	0.005	0.003	0.000	0.000	0.000	2.571	0.036	2.607	1.375	0.000	0.000	0	25.710	0.360	26.070	13.750	0.000	0.000
150	original	1	60x8 weeks	Ag80	MWF	VAC50	100	Jnvaccinate	0.000	0.005	0.000	0.005	0.003	0.000	0.000	0.000	2.575	0.048	2.623	1.322	0.000	0.000	0	25.750	0.480	26.230	13.220	0.000	0.000
150	original	1	60x8 weeks	Ag80	MWF	VAC50	100	Jnvaccinate	0.000	0.005	0.000	0.005	0.003	0.000	0.000	0.000	2.575	0.048	2.623	1.322	0.000	0.000	0	25.750	0.480	26.230	13.220	0.000	0.000
150	original	1	60x8 weeks	Ag80	MWF	VAC50	100	Jnvaccinate	0.000	0.005	0.000	0.005	0.003	0.000	0.000	0.000	2.575	0.048	2.623	1.322	0.000	0.000	0	25.750	0.480	26.230	13.220	0.000	0.000
150	original	1.2	60x8 weeks	Ag80	MWF	VAC50	100	Jnvaccinate	0.000	0.005	0.000	0.005	0.003	0.000	0.000	0.000	2.507	0.065	2.572	1.315	0.000	0.000	0	25.070	0.650	25.720	13.150	0.000	0.000
150	original	1.2	60x8 weeks	Ag80	MWF	VAC50	100	Jnvaccinate	0.000	0.005	0.000	0.005	0.003	0.000	0.000	0.000	2.507	0.065	2.572	1.315	0.000	0.000	0	25.070	0.650	25.720	13.150	0.000	0.000
150	original	1.2	60x8 weeks	Ag80	MWF	VAC50	100	Jnvaccinate	0.000	0.005	0.000	0.005	0.003	0.000	0.000	0.000	2.507	0.065	2.572	1.315	0.000	0.000	0	25.070	0.650	25.720	13.150	0.000	0.000
150	alpha	0.6	60x8 weeks	Ag80	MWF	VAC50	100	Jnvaccinate	0.000	0.005	0.002	0.007	0.004	0.000	0.000	0.000	2.537	0.977	3.514	2.157	0.000	0.000	0	25.370	9.770	35.140	21.570	0.000	0.000
150	alpha	0.6	60x8 weeks	Ag80	MWF	VAC50	100	Jnvaccinate	0.000	0.005	0.002	0.007	0.004	0.000	0.000	0.000	2.537	0.977	3.514	2.157	0.000	0.000	0	25.370	9.770	35.140	21.570	0.000	0.000
150	alpha	0.6	60x8 weeks	Ag80	MWF	VAC50	100	Jnvaccinate	0.000	0.005	0.002	0.007	0.004	0.000	0.000	0.000	2.537	0.977	3.514	2.157	0.000	0.000	0	25.370	9.770	35.140	21.570	0.000	0.000
150	alpha	1	60x8 weeks	Ag80	MWF	VAC50	100	Jnvaccinate	0.000	0.005	0.004	0.009	0.005	0.000	0.000	0.000	2.502	1.812	4.314	2.622	0.000	0.000	0	25.020	18.120	43.140	26.220	0.000	0.000
150	alpha	1	60x8 weeks	Ag80	MWF	VAC50	100	Jnvaccinate	0.000	0.005	0.004	0.009	0.005	0.000	0.000	0.000	2.502	1.812	4.314	2.622	0.000	0.000	0	25.020	18.120	43.140	26.220	0.000	0.000
150	alpha	1	60x8 weeks	Ag80	MWF	VAC50	100	Jnvaccinate	0.000	0.005	0.004	0.009	0.005	0.000	0.000	0.000	2.502	1.812	4.314	2.622	0.000	0.000	0	25.020	18.120	43.140	26.220	0.000	0.000
150	alpha	1.2	60x8 weeks	Ag80	MWF	VAC50	100	Jnvaccinate	0.000	0.005	0.006	0.011	0.007	0.000	0.000	0.000	2.491	2.762	5.253	3.166	0.000	0.000	0	24.910	27.620	52.530	31.660	0.000	0.000
150	alpha	1.2	60x8 weeks	Ag80	MWF	VAC50	100	Jnvaccinate	0.000	0.005	0.006	0.011	0.007	0.000	0.000	0.000	2.491	2.762	5.253	3.166	0.000	0.000	0	24.910	27.620	52.530	31.660	0.000	0.000
150	alpha	1.2	60x8 weeks	Ag80	MWF	VAC50	100	Jnvaccinate	0.000	0.005	0.006	0.011	0.007	0.000	0.000	0.000	2.491	2.762	5.253	3.166	0.000	0.000	0	24.910	27.620	52.530	31.660	0.000	0.000
150	delta	0.6	60x8 weeks	Ag80	MWF	VAC50	100	Jnvaccinate	0.000	0.007	0.014	0.020	0.013	0.000	0.000	0.000	3.178	6.578	9.756	6.282	0.000	0.000	0	31.780	65.780	97.560	62.820	0.000	0.000
150	delta	0.6	60x8 weeks	Ag80	MWF	VAC50	100	Jnvaccinate	0.000	0.007	0.014	0.020	0.013	0.000	0.000	0.000	3.178	6.578	9.756	6.282	0.000	0.000	0	31.780	65.780	97.560	62.820	0.000	0.000
150	delta	0.6	60x8 weeks	Ag80	MWF	VAC50	100	Jnvaccinate	0.000	0.007	0.014	0.020	0.013	0.000	0.000	0.000	3.178	6.578	9.756	6.282	0.000	0.000	0	31.780	65.780	97.560	62.820	0.000	0.000
150	delta	1	60x8 weeks	Ag80	MWF	VAC50	100	Jnvaccinate	0.000	0.006	0.048	0.053	0.034	0.000	0.000	0.000	2.776	22.903	25.679	16.529	0.000	0.000	0	27.760	229.030	256.790	165.290	0.000	0.000
150	delta	1	60x8 weeks	Ag80	MWF	VAC50	100	Jnvaccinate	0.000	0.006	0.048	0.053	0.034	0.000	0.000	0.000	2.776	22.903	25.679	16.529	0.000	0.000	0	27.760	229.030	256.790	165.290	0.000	0.000
150	delta	1	60x8 weeks	Ag80	MWF	VAC50	100	Jnvaccinate	0.000	0.006	0.048	0.053	0.034	0.000	0.000	0.000	2.776	22.903	25.679	16.529	0.000	0.000	0	27.760	229.030	256.790	165.290	0.000	0.000
150	delta	1.2	60x8 weeks	Ag80	MWF	VAC50	100	Jnvaccinate	0.000	0.005	0.069	0.075	0.049	0.000	0.000	0.000	2.461	33.351	35.812	23.459	0.000	0.000	0	24.610	333.510	358.120	234.590	0.000	0.000
150	delta	1.2	60x8 weeks	Ag80	MWF	VAC50	100	Jnvaccinate	0.000	0.005	0.069	0.075	0.049	0.000	0.000	0.000	2.461	33.351	35.812	23.459	0.000	0.000	0	24.610	333.510	358.120	234.590	0.000	0.000
150	delta	1.2	60x8 weeks	Ag80	MWF	VAC50	100	Jnvaccinate	0.000	0.005	0.069	0.075	0.049	0.000	0.000	0.000	2.461	33.351	35.812	23.459	0.000	0.000	0	24.610	333.510	358.120	234.590	0.000	0.000
150	original	0.6	60x8 weeks	Ag80	M	NOVAC	0	Jnvaccinate	0.987	0.010	0.005	0.015	0.004	0.007	0.019	473.957	4.735	2.518	7.253	2.011	3.487	8.993	4740	47.350	25.180	72.530	20.110	34.870	89.930
150	original	0.6	60x8 weeks	Ag80	M	NOVAC	0	Jnvaccinate	0.987	0.010	0.005	0.015	0.004	0.007	0.019	473.957	4.735	2.518	7.253	2.011	3.487	8.993	4740	47.350	25.180	72.530	20.110	34.870	89.930
150	original	0.6	60x8 weeks	Ag80	M	NOVAC	0	Jnvaccinate	0.987	0.010	0.005	0.015	0.004	0.007	0.019	473.957	4.735	2.518	7.253	2.011	3.487	8.993	4740	47.350	25.180	72.530	20.110	34.870	89.930
150	original	1	60x8 weeks	Ag80	M	NOVAC	0	Jnvaccinate	0.981	0.010	0.013	0.022	0.006	0.011	0.018	471.001	4.644	6.059	10.703	2.913	5.284	8.736	4710	46.440	60.590	107.030	29.130	52.840	87.360
150	original	1	60x8 weeks	Ag80	M	NOVAC	0	Jnvaccinate	0.981	0.010	0.013	0.022	0.006	0.011	0.018	471.001	4.644	6.059	10.703	2.913	5.284	8.736	4710	46.440	60.590	107.030	29.130	52.840	87.360
150	original	1	60x8 weeks	Ag80	M	NOVAC	0	Jnvaccinate	0.981	0.010	0.013	0.022	0.006	0.011	0.018	471.001	4.644	6.059	10.703	2.913	5.284	8.736	4710	46.440	60.590	107.030	29.130	52.840	87.360
150	original	1.2	60x8 weeks	Ag80	M	NOVAC	0	Jnvaccinate	0.978	0.010	0.017	0.027	0.007	0.013	0.018	469.269	4.580	8.251	12.831	3.551	6.356	8.589	4693	45.800	82.510	128.310	35.510	63.560	85.890
150	original	1.2	60x8 weeks	Ag80	M	NOVAC	0	Jnvaccinate	0.978	0.010	0.017	0.027	0.007	0.013	0.018	469.269	4.580	8.251	12.831	3.551	6.356	8.589	4693	45.800	82.510	128.310	35.510	63.560	85.890
150	original	1.2	60x8 weeks	Ag80	M	NOVAC	0	Jnvaccinate	0.978	0.010	0.017	0.027	0.007	0.013	0.018	469.269	4.580	8.251	12.831	3.551	6.356	8.589	4693	45.800	82.510	128.310	35.510	63.560	85.890
150	alpha	0.6	60x8 weeks	Ag80	M	NOVAC	0	Jnvaccinate	0.976	0.009	0.016	0.025	0.008	0.014	0.018	468.529	4.511	7.574	12.085	3.613	6.957	8.646	4685	45.110	75.740	120.850	36.130	69.570	86.460
150	alpha	0.6	60x8 weeks	Ag80	M	NOVAC	0	Jnvaccinate	0.976	0.009	0.016	0.025	0.008	0.014	0.018	468.529	4.511	7.574	12.085	3.613	6.957	8.646	4685	45.110	75.740	120.850	36.130	69.570	86.460
150	alpha	0.6	60x8 weeks	Ag80	M	NOVAC	0	Jnvaccinate	0.976	0.009	0.016	0.025	0.008	0.014	0.018	468.529	4.511	7.574	12.085	3.613	6.957	8.646	4685	45.110	75.740	120.850	36.130	69.570	86.460
150	alpha	1	60x8 weeks	Ag80	M	NOVAC	0	Jnvaccinate	0.941	0.008	0.051	0.059	0.018	0.035	0.016	451.699	3.925	24.254	28.179	8.621	16.977	7.514	4517	39.250	242.540	281.790	86.210	169.770	75.140
150	alpha	1	60x8 weeks	Ag80	M	NOVAC	0	Jnvaccinate	0.941	0.008	0.051	0.059	0.018	0.035	0.016	451.699	3.925	24.254	28.179	8.621	16.977	7.514	4517	39.250	242.540	281.790	86.210	169.770	75.140
150	alpha	1	60x8 weeks	Ag80	M	NOVAC	0	Jnvaccinate	0.941	0.008	0.051	0.059	0.018	0.035	0.016	451.699	3.925	24.254	28.179	8.621	16.977	7.514	4517	39.250	242.540	281.790	86.210	169.770	75.140
150	alpha	1.2	60x8 weeks	Ag80	M	NOVAC	0	Jnvaccinate	0.918	0.007	0.073	0.080	0.025	0.049	0.014	440.559	3.450	35.002	38.452	11.904	23.317	6.611	4406	34.500	350.020	384.520	119.040	233.170	66.110
150	alpha	1.2	60x8 weeks	Ag80	M	NOVAC	0	Jnvaccinate	0.918	0.007	0.073	0.080	0.025	0.049	0.014	440.559	3.450	35.002	38.452	11.904	23.317	6.611	4406	34.500	350.020	384.520	119.040	233.170	66.110
150	alpha	1.2	60x8 weeks	Ag80	M	NOVAC	0	Jnvaccinate	0.918	0.007	0.073	0.080	0.025	0.049	0.014	440.559	3.450	35.002	38.452	11.904	23.317	6.611	4406	34.500	350.020	384.520	119.040	233.170	66.110
150	delta	0.6	60x8 weeks	Ag80	M	NOVAC	0	Jnvaccinate	0.912	0.007	0.076	0.083	0.028	0.049	0.014	437.930	3.370	36.525	39.895	13.390	23.585	6.611	4379	33.700	365.250	398.950	133.900	235.850	66.110
150	delta	0.6	60x8 weeks	Ag80	M	NOVAC	0	Jnvaccinate	0.912	0.007	0.076	0.083	0.028	0.049	0.014	437.930	3.370	36.525	39.895	13.390	23.585	6.611	4379	33.700	365.250	398.950	133.900	235.850	66.110
150	delta	0.6	60x8 weeks	Ag80	M	NOVAC	0	Jnvaccinate	0.912	0.007	0.076	0.083	0.028	0.049	0.014	437.930	3.370	36.525	39.895	13.390	23.585	6.611	4379	33.700	365.250	398.950	133.900	235.850	66.110
150	delta	1	60x8 weeks	Ag80	M	NOVAC	0	Jnvaccinate	0.865	0.005	0.113	0.117	0.040	0.072	0.009	415.171	2.182	54.113	56.295	19.346	34.798	4.433	4152	21.820	541.130	562.950	193.460	347.980	44.330
150	delta	1	60x8 weeks	Ag80	M	NOVAC	0	Jnvaccinate	0.865	0.005	0.113	0.117	0.040	0.072	0.009	415.171	2.182	54.113	56.295	19.346	34.798	4.433	4152	21.820	541.130	562.950	193.460	347.980	44.330
150	delta	1	60x8 weeks	Ag80	M	NOVAC	0	Jnvaccinate	0.865	0.005	0.113	0.117	0.040	0.072	0.009	415.171	2.182	54.113	56.295	19.346	34.798	4.433	4152	21.820	541.130	562.950	193.460	347.980	44.330
150	delta	1.2	60x8 weeks	Ag80	M	NOVAC	0	Jnvaccinate	0.860	0.004	0.116	0.120	0.041	0.075	0.008	412.722	1.998	55.598	57.596	19.843	35.766	4.025	4127	19.980	555.980	575.960	198.430	357.660	40.250
150	delta	1.2	60x8 weeks	Ag80	M	NOVAC	0	Jnvaccinate	0.860	0.004	0.116	0.120	0.041	0.075	0.008	412.722	1.998	55.598	57.596	19.843	35.766	4.025	4127	19.980	555.980	575.960	198.430	357.660	40.250
150	delta	1.2	60x8 weeks	Ag80	M	NOVAC	0	Jnvaccinate	0.860	0.004	0.116	0.120	0.041	0.075	0.008	412.722	1.998	55.598	57.596	19.843	35.766	4.025	4127	19.980	555.980	575.960	198.430	357.660	40.250
150	original	0.6	60x8 weeks	Ag80	M	VAC95	25	Jnvaccinate	0.742	0.008	0.003	0.010	0.003	0.005	0.014	355.965	3.669	1.263	4.932	1.427	2.290	6.823	3560	36.690	12.630	49.320	14.270	22.900	68.230

150	original	0.6	60x8 weeks	Ag80	M	VAC95	25	Unvaccinate	0.742	0.008	0.003	0.010	0.003	0.005	0.014	355.965	3.669	1.263	4.932	1.427	2.290	6.823	3560	36.690	12.630	49.320	14.270	22.900	68.230
150	original	0.6	60x8 weeks	Ag80	M	VAC95	25	Unvaccinate	0.742	0.008	0.003	0.010	0.003	0.005	0.014	355.965	3.669	1.263	4.932	1.427	2.290	6.823	3560	36.690	12.630	49.320	14.270	22.900	68.230
150	original	1	60x8 weeks	Ag80	M	VAC95	25	Unvaccinate	0.739	0.008	0.006	0.013	0.004	0.006	0.014	354.685	3.710	2.712	6.422	1.801	3.072	6.828	3547	37.100	27.120	64.220	18.010	30.720	68.280
150	original	1	60x8 weeks	Ag80	M	VAC95	25	Unvaccinate	0.739	0.008	0.006	0.013	0.004	0.006	0.014	354.685	3.710	2.712	6.422	1.801	3.072	6.828	3547	37.100	27.120	64.220	18.010	30.720	68.280
150	original	1	60x8 weeks	Ag80	M	VAC95	25	Unvaccinate	0.739	0.008	0.006	0.013	0.004	0.006	0.014	354.685	3.710	2.712	6.422	1.801	3.072	6.828	3547	37.100	27.120	64.220	18.010	30.720	68.280
150	original	1.2	60x8 weeks	Ag80	M	VAC95	25	Unvaccinate	0.738	0.007	0.007	0.015	0.004	0.007	0.014	354.225	3.492	3.516	7.008	1.905	3.393	6.748	3542	34.920	35.160	70.080	19.050	33.930	67.480
150	original	1.2	60x8 weeks	Ag80	M	VAC95	25	Unvaccinate	0.738	0.007	0.007	0.015	0.004	0.007	0.014	354.225	3.492	3.516	7.008	1.905	3.393	6.748	3542	34.920	35.160	70.080	19.050	33.930	67.480
150	original	1.2	60x8 weeks	Ag80	M	VAC95	25	Unvaccinate	0.738	0.007	0.007	0.015	0.004	0.007	0.014	354.225	3.492	3.516	7.008	1.905	3.393	6.748	3542	34.920	35.160	70.080	19.050	33.930	67.480
150	alpha	0.6	60x8 weeks	Ag80	M	VAC95	25	Unvaccinate	0.736	0.008	0.007	0.015	0.004	0.008	0.014	353.449	3.649	3.465	7.114	2.142	3.980	6.619	3534	36.490	34.650	71.140	21.420	39.800	66.190
150	alpha	0.6	60x8 weeks	Ag80	M	VAC95	25	Unvaccinate	0.736	0.008	0.007	0.015	0.004	0.008	0.014	353.449	3.649	3.465	7.114	2.142	3.980	6.619	3534	36.490	34.650	71.140	21.420	39.800	66.190
150	alpha	0.6	60x8 weeks	Ag80	M	VAC95	25	Unvaccinate	0.736	0.008	0.007	0.015	0.004	0.008	0.014	353.449	3.649	3.465	7.114	2.142	3.980	6.619	3534	36.490	34.650	71.140	21.420	39.800	66.190
150	alpha	1	60x8 weeks	Ag80	M	VAC95	25	Unvaccinate	0.725	0.007	0.019	0.026	0.008	0.015	0.013	347.932	3.389	9.297	12.686	4.007	7.164	6.291	3479	33.890	92.970	126.860	40.070	71.640	62.910
150	alpha	1	60x8 weeks	Ag80	M	VAC95	25	Unvaccinate	0.725	0.007	0.019	0.026	0.008	0.015	0.013	347.932	3.389	9.297	12.686	4.007	7.164	6.291	3479	33.890	92.970	126.860	40.070	71.640	62.910
150	alpha	1	60x8 weeks	Ag80	M	VAC95	25	Unvaccinate	0.725	0.007	0.019	0.026	0.008	0.015	0.013	347.932	3.389	9.297	12.686	4.007	7.164	6.291	3479	33.890	92.970	126.860	40.070	71.640	62.910
150	alpha	1.2	60x8 weeks	Ag80	M	VAC95	25	Unvaccinate	0.715	0.007	0.030	0.037	0.012	0.021	0.012	343.171	3.270	14.608	17.878	5.632	10.028	5.964	3432	32.700	146.080	178.780	56.320	100.280	59.640
150	alpha	1.2	60x8 weeks	Ag80	M	VAC95	25	Unvaccinate	0.715	0.007	0.030	0.037	0.012	0.021	0.012	343.171	3.270	14.608	17.878	5.632	10.028	5.964	3432	32.700	146.080	178.780	56.320	100.280	59.640
150	alpha	1.2	60x8 weeks	Ag80	M	VAC95	25	Unvaccinate	0.715	0.007	0.030	0.037	0.012	0.021	0.012	343.171	3.270	14.608	17.878	5.632	10.028	5.964	3432	32.700	146.080	178.780	56.320	100.280	59.640
150	delta	0.6	60x8 weeks	Ag80	M	VAC95	25	Unvaccinate	0.710	0.007	0.035	0.041	0.014	0.023	0.012	340.850	3.217	16.696	19.913	6.907	10.884	5.811	3409	32.170	166.960	199.130	69.070	108.840	58.110
150	delta	0.6	60x8 weeks	Ag80	M	VAC95	25	Unvaccinate	0.710	0.007	0.035	0.041	0.014	0.023	0.012	340.850	3.217	16.696	19.913	6.907	10.884	5.811	3409	32.170	166.960	199.130	69.070	108.840	58.110
150	delta	0.6	60x8 weeks	Ag80	M	VAC95	25	Unvaccinate	0.710	0.007	0.035	0.041	0.014	0.023	0.012	340.850	3.217	16.696	19.913	6.907	10.884	5.811	3409	32.170	166.960	199.130	69.070	108.840	58.110
150	delta	1	60x8 weeks	Ag80	M	VAC95	25	Unvaccinate	0.672	0.005	0.077	0.081	0.029	0.043	0.009	322.321	2.311	36.779	39.090	14.052	20.843	4.330	3223	23.110	367.790	390.900	140.520	208.430	43.300
150	delta	1	60x8 weeks	Ag80	M	VAC95	25	Unvaccinate	0.672	0.005	0.077	0.081	0.029	0.043	0.009	322.321	2.311	36.779	39.090	14.052	20.843	4.330	3223	23.110	367.790	390.900	140.520	208.430	43.300
150	delta	1	60x8 weeks	Ag80	M	VAC95	25	Unvaccinate	0.672	0.005	0.077	0.081	0.029	0.043	0.009	322.321	2.311	36.779	39.090	14.052	20.843	4.330	3223	23.110	367.790	390.900	140.520	208.430	43.300
150	delta	1.2	60x8 weeks	Ag80	M	VAC95	25	Unvaccinate	0.660	0.004	0.088	0.092	0.034	0.049	0.008	316.920	2.050	42.285	44.335	16.261	23.514	3.832	3169	20.500	422.850	443.350	162.610	235.140	38.320
150	delta	1.2	60x8 weeks	Ag80	M	VAC95	25	Unvaccinate	0.660	0.004	0.088	0.092	0.034	0.049	0.008	316.920	2.050	42.285	44.335	16.261	23.514	3.832	3169	20.500	422.850	443.350	162.610	235.140	38.320
150	delta	1.2	60x8 weeks	Ag80	M	VAC95	25	Unvaccinate	0.660	0.004	0.088	0.092	0.034	0.049	0.008	316.920	2.050	42.285	44.335	16.261	23.514	3.832	3169	20.500	422.850	443.350	162.610	235.140	38.320
150	original	0.6	60x8 weeks	Ag80	M	VAC95	50	Unvaccinate	0.495	0.005	0.001	0.006	0.002	0.003	0.010	237.530	2.545	0.567	3.112	0.918	1.373	4.567	2375	25.450	5.670	31.120	9.180	13.730	45.670
150	original	0.6	60x8 weeks	Ag80	M	VAC95	50	Unvaccinate	0.495	0.005	0.001	0.006	0.002	0.003	0.010	237.530	2.545	0.567	3.112	0.918	1.373	4.567	2375	25.450	5.670	31.120	9.180	13.730	45.670
150	original	0.6	60x8 weeks	Ag80	M	VAC95	50	Unvaccinate	0.495	0.005	0.001	0.006	0.002	0.003	0.010	237.530	2.545	0.567	3.112	0.918	1.373	4.567	2375	25.450	5.670	31.120	9.180	13.730	45.670
150	original	1	60x8 weeks	Ag80	M	VAC95	50	Unvaccinate	0.494	0.005	0.002	0.007	0.002	0.003	0.010	237.270	2.566	1.007	3.573	1.038	1.589	4.655	2373	25.660	10.070	35.730	10.380	15.890	46.550
150	original	1	60x8 weeks	Ag80	M	VAC95	50	Unvaccinate	0.494	0.005	0.002	0.007	0.002	0.003	0.010	237.270	2.566	1.007	3.573	1.038	1.589	4.655	2373	25.660	10.070	35.730	10.380	15.890	46.550
150	original	1	60x8 weeks	Ag80	M	VAC95	50	Unvaccinate	0.494	0.005	0.002	0.007	0.002	0.003	0.010	237.270	2.566	1.007	3.573	1.038	1.589	4.655	2373	25.660	10.070	35.730	10.380	15.890	46.550
150	original	1.2	60x8 weeks	Ag80	M	VAC95	50	Unvaccinate	0.494	0.006	0.003	0.008	0.002	0.004	0.009	236.948	2.643	1.288	3.931	1.136	1.749	4.556	2369	26.430	12.880	39.310	11.360	17.490	45.560
150	original	1.2	60x8 weeks	Ag80	M	VAC95	50	Unvaccinate	0.494	0.006	0.003	0.008	0.002	0.004	0.009	236.948	2.643	1.288	3.931	1.136	1.749	4.556	2369	26.430	12.880	39.310	11.360	17.490	45.560
150	original	1.2	60x8 weeks	Ag80	M	VAC95	50	Unvaccinate	0.494	0.006	0.003	0.008	0.002	0.004	0.009	236.948	2.643	1.288	3.931	1.136	1.749	4.556	2369	26.430	12.880	39.310	11.360	17.490	45.560
150	alpha	0.6	60x8 weeks	Ag80	M	VAC95	50	Unvaccinate	0.493	0.005	0.003	0.008	0.003	0.004	0.009	236.549	2.584	1.458	4.042	1.325	2.034	4.414	2365	25.840	14.580	40.420	13.250	20.340	44.140
150	alpha	0.6	60x8 weeks	Ag80	M	VAC95	50	Unvaccinate	0.493	0.005	0.003	0.008	0.003	0.004	0.009	236.549	2.584	1.458	4.042	1.325	2.034	4.414	2365	25.840	14.580	40.420	13.250	20.340	44.140
150	alpha	0.6	60x8 weeks	Ag80	M	VAC95	50	Unvaccinate	0.493	0.005	0.003	0.008	0.003	0.004	0.009	236.549	2.584	1.458	4.042	1.325	2.034	4.414	2365	25.840	14.580	40.420	13.250	20.340	44.140
150	alpha	1	60x8 weeks	Ag80	M	VAC95	50	Unvaccinate	0.489	0.005	0.007	0.012	0.004	0.006	0.009	234.868	2.512	3.325	5.837	1.913	3.017	4.411	2349	25.120	33.250	58.370	19.130	30.170	44.110
150	alpha	1	60x8 weeks	Ag80	M	VAC95	50	Unvaccinate	0.489	0.005	0.007	0.012	0.004	0.006	0.009	234.868	2.512	3.325	5.837	1.913	3.017	4.411	2349	25.120	33.250	58.370	19.130	30.170	44.110
150	alpha	1	60x8 weeks	Ag80	M	VAC95	50	Unvaccinate	0.489	0.005	0.007	0.012	0.004	0.006	0.009	234.868	2.512	3.325	5.837	1.913	3.017	4.411	2349	25.120	33.250	58.370	19.130	30.170	44.110
150	alpha	1.2	60x8 weeks	Ag80	M	VAC95	50	Unvaccinate	0.487	0.005	0.009	0.014	0.005	0.007	0.009	233.762	2.500	4.390	6.890	2.309	3.592	4.325	2338	25.000	43.900	68.900	23.090	35.920	43.250
150	alpha	1.2	60x8 weeks	Ag80	M	VAC95	50	Unvaccinate	0.487	0.005	0.009	0.014	0.005	0.007	0.009	233.762	2.500	4.390	6.890	2.309	3.592	4.325	2338	25.000	43.900	68.900	23.090	35.920	43.250
150	alpha	1.2	60x8 weeks	Ag80	M	VAC95	50	Unvaccinate	0.487	0.005	0.009	0.014	0.005	0.007	0.009	233.762	2.500	4.390	6.890	2.309	3.592	4.325	2338	25.000	43.900	68.900	23.090	35.920	43.250
150	delta	0.6	60x8 weeks	Ag80	M	VAC95	50	Unvaccinate	0.486	0.005	0.011	0.016	0.006	0.008	0.009	233.291	2.523	5.369	7.892	2.959	3.687	4.430	2333	25.230	53.690	78.920	29.590	36.870	44.300
150	delta	0.6	60x8 weeks	Ag80	M	VAC95	50	Unvaccinate	0.486	0.005	0.011	0.016	0.006	0.008	0.009	233.291	2.523	5.369	7.892	2.959	3.687	4.430	2333	25.230	53.690	78.920	29.590	36.870	44.300
150	delta	0.6	60x8 weeks	Ag80	M	VAC95	50	Unvaccinate	0.486	0.005	0.011	0.016	0.006	0.008	0.009	233.291	2.523	5.369	7.892	2.959	3.687	4.430	2333	25.230	53.690	78.920	29.590	36.870	44.300
150	delta	1	60x8 weeks	Ag80	M	VAC95	50	Unvaccinate	0.468	0.005	0.034	0.039	0.015	0.018	0.008	224.540	2.270	16.475	18.745	7.105	8.709	3.621	2245	22.700	164.750	187.450	71.050	87.090	36.210
150	delta	1	60x8 weeks	Ag80	M	VAC95	50	Unvaccinate	0.468	0.005	0.034	0.039	0.015	0.018	0.008	224.540	2.270	16.475	18.745	7.105	8.709	3.621	2245	22.700	164.750	187.450	71.050	87.090	36.210
150	delta	1	60x8 weeks	Ag80	M	VAC95	50	Unvaccinate	0.468	0.005	0.034	0.039	0.015	0.018	0.008	224.540	2.270	16.475	18.745	7.105	8.709	3.621	2245	22.700	164.750	187.450	71.050	87.090	36.210
150	delta	1.2	60x8 weeks	Ag80	M	VAC95	50	Unvaccinate	0.462	0.004	0.044	0.048	0.018	0.022	0.007	221.592	2.058	21.095	23.153	8.861	10.402	3.372	2216	20.580	210.950	231.530	88.610	104.020	33.720
150	delta	1.2	60x8 weeks	Ag80	M	VAC95	50	Unvaccinate	0.462	0.004	0.044	0.048	0.018	0.022	0.007	221.592	2.058	21.095	23.153	8.861	10.402	3.372	2216	20.580	210.950	231.530	88.610	104.020	33.720
150	delta	1.2	60x8 weeks	Ag80	M	VAC95	50	Unvaccinate	0.462	0.004	0.044	0.048	0.018	0.022	0.007	221.592	2.058	21.095	23.153	8.861	10.402	3.372	2216	20.580	210.950	231.530	88.610	104.020	33.720
150	original	0.6	60x8 weeks	Ag80	M	VAC95	75	Unvaccinate	0.248	0.003	0.000	0.003	0.001	0.001	0.005	118.947	1.455	0.145	1.600	0.486	0.594	2.299	1189	14.550	1.450	16.000	4.860	5.940	22.990
150	original	0.6	60x8 weeks	Ag80	M	VAC95	75	Unvaccinate	0.248	0.003	0.000	0.003	0.001	0.001	0.005	118.947	1.455	0.145	1.600	0.486	0.594	2.299	1189	14.550	1.450	16.000	4.860	5.940	22.990
150	original	0.6	60x8 weeks	Ag80	M	VAC95	75	Unvaccinate	0.248	0.003	0.000	0.003	0.001	0.001	0.005	118.947	1.455	0.145	1.600	0.486	0.594	2.299	1189	14.550	1.450	16.000	4.860	5.940	22.990
150	original	1	60x8 weeks	Ag80	M	VAC95	75	Unvaccinate	0.248	0.003	0.000	0.003	0.001	0.001	0.005	118.833	1.438	0.234	1.672	0.521	0.656	2.285	1188	14.380	2.340	16.720	5.210	6.560	22.850
150	original	1	60x8 weeks	Ag80	M	VAC95	75	Unvaccinate	0.248	0.003	0.000	0.003	0.001	0.001	0.005	118.833	1.438	0.234	1.672	0.521	0.656	2.285	1188	14.380	2.340	16.720	5.210	6.560	22.850
150	original	1	60x8 weeks	Ag80	M	VAC95	75	Unvaccinate	0.248	0.003	0.000	0.003	0.001	0.001	0.005	118.833	1.438	0.234	1.672	0.521	0.656	2.285	1188	14.380	2.340	16.720	5.210	6.560	22.850
150	original	1.2	60x8 weeks	Ag80	M	VAC95	75	Unvaccinate	0.247	0.003	0.001	0.004	0.001	0.001	0.005	118.799	1.475	0.334	1.809	0.549	0.700	2.288	1188	14.750	3.340	18.090	5.490	7.000	22.880
150	original	1.2	60x8 weeks	Ag80	M	VAC95	75	Unvaccinate	0.247	0.003	0.001	0.004	0.001	0.001	0.005	118.799	1.475	0.334	1.809	0.549	0.700	2.288	1188	14.750	3.340	18.090	5.490	7.000	22.880
150	original	1.2	60x8 weeks	Ag80	M	VAC95	75	Unvaccinate	0.247	0.003	0.001	0.004	0.001	0.001	0.005	118.799	1.475	0.334	1.809	0.549	0.700	2.288	1188	14.750	3.340	18.090	5.490	7.000	22.880
150	alpha	0.6	60x8 weeks	Ag80	M	VAC95	75	Unvaccinate	0.247	0.003	0.001	0.004	0.001	0.002	0.005	118.518	1.579	0.399	1.978	0.689	0.848	2.203	1185	15.790	3.990	19.780	6.890	8.480	22.030
150	alpha	0.6	60x8 weeks	Ag80	M	VAC95	75	Unvaccinate	0.247	0.003	0.001	0.004	0.001	0.002	0.005	118.518	1.579	0.399	1.978	0.689	0.848	2.203	1185	15.790	3.990	19.780	6.890	8.480	22.030
150	alpha	0.6	60x8 weeks	Ag80	M	VAC95	75	Unvaccinate	0.247	0.003	0.001	0.004	0.001	0.002	0.005	118.518	1.579	0.399	1.978	0.689	0.848	2.203	1185	15.790	3.990	19.780	6.890	8.480	22.030
150	alpha	1	60x8 weeks	Ag80	M	VAC95	75	Unvaccinate	0.246	0.003	0.002	0.005	0.002	0.002	0.005	118.302	1.505	0.754	2.259	0.817	0.978	2.233	1183	15.050	7.540	22.590	8.170	9.780	22.330
150	alpha	1	60x8 weeks	Ag80	M	VAC95	75	Unvaccinate	0.246	0.003	0.002	0.005	0.002	0.002	0.005	118.302	1.505	0.754	2.259	0.817	0.978	2.233	1183	15.050	7.540	22.590	8.170	9.780	22.330
150	alpha	1	60x8 weeks	Ag80	M	VAC95	75	Unvaccinate	0.246	0.003	0.002	0.005	0.002	0.002	0.005	118.302	1.505	0.754	2.259	0.817	0.978	2.233	1183	15.050	7.540	22.590	8.170	9.780	22.330
150	alpha	1.2	60x8 weeks	Ag80	M	VAC95	75	Unvaccinate	0.246	0.003	0.002	0.005	0.002	0.002	0.005	118.255	1.530	0.874	2.404	0.830	1.032	2.271	1183	15.300	8.740	24.040	8.300	10.320	22.710
150	alpha	1.2	60x8 weeks	Ag80	M	VAC95	75	Unvaccinate	0.246	0.003	0.002	0.005	0.002	0.002	0.005	118.255	1.530	0.874	2.404	0.830	1.032	2.271	1183	15.300	8.740	24.040	8.300	10.320	22.710
150	alpha	1.2	60x8 weeks	Ag80	M	VAC95	75	Unvaccinate	0.246	0.003	0.002	0.005	0.002	0.002	0.005	118.255	1.530	0.874	2.404	0.830	1.032	2.271	1183	15.300	8.740	24.040	8.300	10.320	22.710

150	delta	0.6	60x8 weeks	Ag80	M	VAC95	75	Unvaccinate	0.246	0.004	0.003	0.006	0.003	0.002	0.005	118.008	1.702	1.324	3.026	1.252	1.081	2.232	1180	17.020	13.240	30.260	12.520	10.810	22.320
150	delta	0.6	60x8 weeks	Ag80	M	VAC95	75	Unvaccinate	0.246	0.004	0.003	0.006	0.003	0.002	0.005	118.008	1.702	1.324	3.026	1.252	1.081	2.232	1180	17.020	13.240	30.260	12.520	10.810	22.320
150	delta	0.6	60x8 weeks	Ag80	M	VAC95	75	Unvaccinate	0.246	0.004	0.003	0.006	0.003	0.002	0.005	118.008	1.702	1.324	3.026	1.252	1.081	2.232	1180	17.020	13.240	30.260	12.520	10.810	22.320
150	delta	1	60x8 weeks	Ag80	M	VAC95	75	Unvaccinate	0.243	0.004	0.007	0.011	0.004	0.004	0.005	116.701	1.683	3.549	5.232	2.126	1.847	2.171	1167	16.830	35.490	52.320	21.260	18.470	21.710
150	delta	1	60x8 weeks	Ag80	M	VAC95	75	Unvaccinate	0.243	0.004	0.007	0.011	0.004	0.004	0.005	116.701	1.683	3.549	5.232	2.126	1.847	2.171	1167	16.830	35.490	52.320	21.260	18.470	21.710
150	delta	1	60x8 weeks	Ag80	M	VAC95	75	Unvaccinate	0.243	0.004	0.007	0.011	0.004	0.004	0.005	116.701	1.683	3.549	5.232	2.126	1.847	2.171	1167	16.830	35.490	52.320	21.260	18.470	21.710
150	delta	1.2	60x8 weeks	Ag80	M	VAC95	75	Unvaccinate	0.242	0.003	0.010	0.013	0.006	0.005	0.004	115.979	1.614	4.770	6.384	2.706	2.189	2.114	1160	16.140	47.700	63.840	27.060	21.890	21.140
150	delta	1.2	60x8 weeks	Ag80	M	VAC95	75	Unvaccinate	0.242	0.003	0.010	0.013	0.006	0.005	0.004	115.979	1.614	4.770	6.384	2.706	2.189	2.114	1160	16.140	47.700	63.840	27.060	21.890	21.140
150	delta	1.2	60x8 weeks	Ag80	M	VAC95	75	Unvaccinate	0.242	0.003	0.010	0.013	0.006	0.005	0.004	115.979	1.614	4.770	6.384	2.706	2.189	2.114	1160	16.140	47.700	63.840	27.060	21.890	21.140
150	original	0.6	60x8 weeks	Ag80	M	VAC95	100	Unvaccinate	0.000	0.001	0.000	0.001	0.000	0.000	0.000	0.000	0.348	0.001	0.349	0.127	0.000	0.000	0	3.480	0.010	3.490	1.270	0.000	0.000
150	original	0.6	60x8 weeks	Ag80	M	VAC95	100	Unvaccinate	0.000	0.001	0.000	0.001	0.000	0.000	0.000	0.000	0.348	0.001	0.349	0.127	0.000	0.000	0	3.480	0.010	3.490	1.270	0.000	0.000
150	original	0.6	60x8 weeks	Ag80	M	VAC95	100	Unvaccinate	0.000	0.001	0.000	0.001	0.000	0.000	0.000	0.000	0.348	0.001	0.349	0.127	0.000	0.000	0	3.480	0.010	3.490	1.270	0.000	0.000
150	original	1	60x8 weeks	Ag80	M	VAC95	100	Unvaccinate	0.000	0.001	0.000	0.001	0.000	0.000	0.000	0.000	0.356	0.004	0.360	0.128	0.000	0.000	0	3.560	0.040	3.600	1.280	0.000	0.000
150	original	1	60x8 weeks	Ag80	M	VAC95	100	Unvaccinate	0.000	0.001	0.000	0.001	0.000	0.000	0.000	0.000	0.356	0.004	0.360	0.128	0.000	0.000	0	3.560	0.040	3.600	1.280	0.000	0.000
150	original	1	60x8 weeks	Ag80	M	VAC95	100	Unvaccinate	0.000	0.001	0.000	0.001	0.000	0.000	0.000	0.000	0.356	0.004	0.360	0.128	0.000	0.000	0	3.560	0.040	3.600	1.280	0.000	0.000
150	original	1.2	60x8 weeks	Ag80	M	VAC95	100	Unvaccinate	0.000	0.001	0.000	0.001	0.000	0.000	0.000	0.000	0.369	0.000	0.369	0.154	0.000	0.000	0	3.690	0.000	3.690	1.540	0.000	0.000
150	original	1.2	60x8 weeks	Ag80	M	VAC95	100	Unvaccinate	0.000	0.001	0.000	0.001	0.000	0.000	0.000	0.000	0.369	0.000	0.369	0.154	0.000	0.000	0	3.690	0.000	3.690	1.540	0.000	0.000
150	original	1.2	60x8 weeks	Ag80	M	VAC95	100	Unvaccinate	0.000	0.001	0.000	0.001	0.000	0.000	0.000	0.000	0.369	0.000	0.369	0.154	0.000	0.000	0	3.690	0.000	3.690	1.540	0.000	0.000
150	alpha	0.6	60x8 weeks	Ag80	M	VAC95	100	Unvaccinate	0.000	0.001	0.000	0.001	0.000	0.000	0.000	0.000	0.443	0.021	0.464	0.203	0.000	0.000	0	4.430	0.210	4.640	2.030	0.000	0.000
150	alpha	0.6	60x8 weeks	Ag80	M	VAC95	100	Unvaccinate	0.000	0.001	0.000	0.001	0.000	0.000	0.000	0.000	0.443	0.021	0.464	0.203	0.000	0.000	0	4.430	0.210	4.640	2.030	0.000	0.000
150	alpha	0.6	60x8 weeks	Ag80	M	VAC95	100	Unvaccinate	0.000	0.001	0.000	0.001	0.000	0.000	0.000	0.000	0.443	0.021	0.464	0.203	0.000	0.000	0	4.430	0.210	4.640	2.030	0.000	0.000
150	alpha	1	60x8 weeks	Ag80	M	VAC95	100	Unvaccinate	0.000	0.001	0.000	0.001	0.001	0.000	0.000	0.000	0.462	0.039	0.501	0.243	0.000	0.000	0	4.620	0.390	5.010	2.430	0.000	0.000
150	alpha	1	60x8 weeks	Ag80	M	VAC95	100	Unvaccinate	0.000	0.001	0.000	0.001	0.001	0.000	0.000	0.000	0.462	0.039	0.501	0.243	0.000	0.000	0	4.620	0.390	5.010	2.430	0.000	0.000
150	alpha	1	60x8 weeks	Ag80	M	VAC95	100	Unvaccinate	0.000	0.001	0.000	0.001	0.001	0.000	0.000	0.000	0.462	0.039	0.501	0.243	0.000	0.000	0	4.620	0.390	5.010	2.430	0.000	0.000
150	alpha	1.2	60x8 weeks	Ag80	M	VAC95	100	Unvaccinate	0.000	0.001	0.000	0.001	0.001	0.000	0.000	0.000	0.460	0.051	0.511	0.254	0.000	0.000	0	4.600	0.510	5.110	2.540	0.000	0.000
150	alpha	1.2	60x8 weeks	Ag80	M	VAC95	100	Unvaccinate	0.000	0.001	0.000	0.001	0.001	0.000	0.000	0.000	0.460	0.051	0.511	0.254	0.000	0.000	0	4.600	0.510	5.110	2.540	0.000	0.000
150	alpha	1.2	60x8 weeks	Ag80	M	VAC95	100	Unvaccinate	0.000	0.001	0.000	0.001	0.001	0.000	0.000	0.000	0.460	0.051	0.511	0.254	0.000	0.000	0	4.600	0.510	5.110	2.540	0.000	0.000
150	delta	0.6	60x8 weeks	Ag80	M	VAC95	100	Unvaccinate	0.000	0.002	0.000	0.002	0.001	0.000	0.000	0.000	0.734	0.155	0.889	0.470	0.000	0.000	0	7.340	1.550	8.890	4.700	0.000	0.000
150	delta	0.6	60x8 weeks	Ag80	M	VAC95	100	Unvaccinate	0.000	0.002	0.000	0.002	0.001	0.000	0.000	0.000	0.734	0.155	0.889	0.470	0.000	0.000	0	7.340	1.550	8.890	4.700	0.000	0.000
150	delta	0.6	60x8 weeks	Ag80	M	VAC95	100	Unvaccinate	0.000	0.002	0.000	0.002	0.001	0.000	0.000	0.000	0.734	0.155	0.889	0.470	0.000	0.000	0	7.340	1.550	8.890	4.700	0.000	0.000
150	delta	1	60x8 weeks	Ag80	M	VAC95	100	Unvaccinate	0.000	0.002	0.001	0.002	0.001	0.000	0.000	0.000	0.729	0.264	0.993	0.544	0.000	0.000	0	7.290	2.640	9.930	5.440	0.000	0.000
150	delta	1	60x8 weeks	Ag80	M	VAC95	100	Unvaccinate	0.000	0.002	0.001	0.002	0.001	0.000	0.000	0.000	0.729	0.264	0.993	0.544	0.000	0.000	0	7.290	2.640	9.930	5.440	0.000	0.000
150	delta	1	60x8 weeks	Ag80	M	VAC95	100	Unvaccinate	0.000	0.002	0.001	0.002	0.001	0.000	0.000	0.000	0.729	0.264	0.993	0.544	0.000	0.000	0	7.290	2.640	9.930	5.440	0.000	0.000
150	delta	1.2	60x8 weeks	Ag80	M	VAC95	100	Unvaccinate	0.000	0.001	0.001	0.002	0.001	0.000	0.000	0.000	0.697	0.304	1.001	0.533	0.000	0.000	0	6.970	3.040	10.010	5.330	0.000	0.000
150	delta	1.2	60x8 weeks	Ag80	M	VAC95	100	Unvaccinate	0.000	0.001	0.001	0.002	0.001	0.000	0.000	0.000	0.697	0.304	1.001	0.533	0.000	0.000	0	6.970	3.040	10.010	5.330	0.000	0.000
150	delta	1.2	60x8 weeks	Ag80	M	VAC95	100	Unvaccinate	0.000	0.001	0.001	0.002	0.001	0.000	0.000	0.000	0.697	0.304	1.001	0.533	0.000	0.000	0	6.970	3.040	10.010	5.330	0.000	0.000
150	original	0.6	60x8 weeks	Ag80	M	VAC70	25	Unvaccinate	0.742	0.008	0.003	0.011	0.003	0.005	0.014	355.937	4.011	1.444	5.455	1.641	2.326	6.783	3559	40.110	14.440	54.550	16.410	23.260	67.830
150	original	0.6	60x8 weeks	Ag80	M	VAC70	25	Unvaccinate	0.742	0.008	0.003	0.011	0.003	0.005	0.014	355.937	4.011	1.444	5.455	1.641	2.326	6.783	3559	40.110	14.440	54.550	16.410	23.260	67.830
150	original	0.6	60x8 weeks	Ag80	M	VAC70	25	Unvaccinate	0.742	0.008	0.003	0.011	0.003	0.005	0.014	355.937	4.011	1.444	5.455	1.641	2.326	6.783	3559	40.110	14.440	54.550	16.410	23.260	67.830
150	original	1	60x8 weeks	Ag80	M	VAC70	25	Unvaccinate	0.739	0.008	0.006	0.014	0.004	0.006	0.014	354.943	3.966	2.749	6.715	1.984	2.948	6.791	3549	39.660	27.490	67.150	19.840	29.480	67.910
150	original	1	60x8 weeks	Ag80	M	VAC70	25	Unvaccinate	0.739	0.008	0.006	0.014	0.004	0.006	0.014	354.943	3.966	2.749	6.715	1.984	2.948	6.791	3549	39.660	27.490	67.150	19.840	29.480	67.910
150	original	1	60x8 weeks	Ag80	M	VAC70	25	Unvaccinate	0.739	0.008	0.006	0.014	0.004	0.006	0.014	354.943	3.966	2.749	6.715	1.984	2.948	6.791	3549	39.660	27.490	67.150	19.840	29.480	67.910
150	original	1.2	60x8 weeks	Ag80	M	VAC70	25	Unvaccinate	0.738	0.008	0.008	0.016	0.005	0.007	0.014	354.345	3.963	3.835	7.798	2.270	3.336	6.671	3543	39.630	38.350	77.980	22.700	33.360	66.710
150	original	1.2	60x8 weeks	Ag80	M	VAC70	25	Unvaccinate	0.738	0.008	0.008	0.016	0.005	0.007	0.014	354.345	3.963	3.835	7.798	2.270	3.336	6.671	3543	39.630	38.350	77.980	22.700	33.360	66.710
150	original	1.2	60x8 weeks	Ag80	M	VAC70	25	Unvaccinate	0.738	0.008	0.008	0.016	0.005	0.007	0.014	354.345	3.963	3.835	7.798	2.270	3.336	6.671	3543	39.630	38.350	77.980	22.700	33.360	66.710
150	alpha	0.6	60x8 weeks	Ag80	M	VAC70	25	Unvaccinate	0.735	0.008	0.009	0.017	0.006	0.009	0.014	352.906	3.830	4.358	8.188	2.742	4.185	6.664	3529	38.300	43.580	81.880	27.420	41.850	66.640
150	alpha	0.6	60x8 weeks	Ag80	M	VAC70	25	Unvaccinate	0.735	0.008	0.009	0.017	0.006	0.009	0.014	352.906	3.830	4.358	8.188	2.742	4.185	6.664	3529	38.300	43.580	81.880	27.420	41.850	66.640
150	alpha	0.6	60x8 weeks	Ag80	M	VAC70	25	Unvaccinate	0.735	0.008	0.009	0.017	0.006	0.009	0.014	352.906	3.830	4.358	8.188	2.742	4.185	6.664	3529	38.300	43.580	81.880	27.420	41.850	66.640
150	alpha	1	60x8 weeks	Ag80	M	VAC70	25	Unvaccinate	0.722	0.008	0.025	0.032	0.011	0.017	0.013	346.471	3.606	11.894	15.500	5.349	7.962	6.132	3465	36.060	118.940	155.000	53.490	79.620	61.320
150	alpha	1	60x8 weeks	Ag80	M	VAC70	25	Unvaccinate	0.722	0.008	0.025	0.032	0.011	0.017	0.013	346.471	3.606	11.894	15.500	5.349	7.962	6.132	3465	36.060	118.940	155.000	53.490	79.620	61.320
150	alpha	1	60x8 weeks	Ag80	M	VAC70	25	Unvaccinate	0.722	0.008	0.025	0.032	0.011	0.017	0.013	346.471	3.606	11.894	15.500	5.349	7.962	6.132	3465	36.060	118.940	155.000	53.490	79.620	61.320
150	alpha	1.2	60x8 weeks	Ag80	M	VAC70	25	Unvaccinate	0.711	0.007	0.038	0.045	0.016	0.023	0.012	341.345	3.410	18.326	21.736	7.604	11.003	5.857	3413	34.100	183.260	217.360	76.040	110.030	58.570
150	alpha	1.2	60x8 weeks	Ag80	M	VAC70	25	Unvaccinate	0.711	0.007	0.038	0.045	0.016	0.023	0.012	341.345	3.410	18.326	21.736	7.604	11.003	5.857	3413	34.100	183.260	217.360	76.040	110.030	58.570
150	alpha	1.2	60x8 weeks	Ag80	M	VAC70	25	Unvaccinate	0.711	0.007	0.038	0.045	0.016	0.023	0.012	341.345	3.410	18.326	21.736	7.604	11.003	5.857	3413	34.100	183.260	217.360	76.040	110.030	58.570
150	delta	0.6	60x8 weeks	Ag80	M	VAC70	25	Unvaccinate	0.702	0.007	0.048	0.055	0.021	0.027	0.011	337.183	3.359	23.125	26.484	10.144	12.853	5.515	3372	33.590	231.250	264.840	101.440	128.530	55.150
150	delta	0.6	60x8 weeks	Ag80	M	VAC70	25	Unvaccinate	0.702	0.007	0.048	0.055	0.021	0.027	0.011	337.183	3.359	23.125	26.484	10.144	12.853	5.515	3372	33.590	231.250	264.840	101.440	128.530	55.150
150	delta	0.6	60x8 weeks	Ag80	M	VAC70	25	Unvaccinate	0.702	0.007	0.048	0.055	0.021	0.027	0.011	337.183	3.359	23.125	26.484	10.144	12.853	5.515	3372	33.590	231.250	264.840	101.440	128.530	55.150
150	delta	1	60x8 weeks	Ag80	M	VAC70	25	Unvaccinate	0.660	0.005	0.097	0.102	0.041	0.049	0.008	316.944	2.241	46.584	48.825	19.850	23.308	3.903	3169	22.410	465.840	488.250	198.500	233.080	39.030
150	delta	1	60x8 weeks	Ag80	M	VAC70	25	Unvaccinate	0.660	0.005	0.097	0.102	0.041	0.049	0.008	316.944	2.241	46.584	48.825	19.850	23.308	3.903	3169	22.410	465.840	488.250	198.500	233.080	39.030
150	delta	1	60x8 weeks	Ag80	M	VAC70	25	Unvaccinate	0.660	0.005	0.097	0.102	0.041	0.049	0.008	316.944	2.241	46.584	48.825	19.850	23.308	3.903	3169	22.410	465.840	488.250	198.500	233.080	39.030
150	delta	1.2	60x8 weeks	Ag80	M	VAC70	25	Unvaccinate	0.651	0.004	0.107	0.111	0.047	0.052	0.007	312.592	2.029	51.489	53.518	22.396	25.096	3.433	3126	20.290	514.890	535.180	223.960	250.960	34.330
150	delta	1.2	60x8 weeks	Ag80	M	VAC70	25	Unvaccinate	0.651	0.004	0.107	0.111	0.047	0.052	0.007	312.592	2.029	51.489	53.518	22.396	25.096	3.433	3126	20.290	514.890	535.180	223.960	250.960	34.330
150	delta	1.2	60x8 weeks	Ag80	M	VAC70	25	Unvaccinate	0.651	0.004	0.107	0.111	0.047	0.052	0.007	312.592	2.029	51.489	53.518	22.396	25.096	3.433	3126	20.290	514.890	535.180	223.960	250.960	34.330
150	original	0.6	60x8 weeks	Ag80	M	VAC70	50	Unvaccinate	0.495	0.007	0.001	0.008	0.003	0.003	0.010	237.613	3.191	0.616	3.807	1.242	1.408	4.633	2376	31.910	6.160	38.070	12.420	14.080	46.330
150	original	0.6	60x8 weeks	Ag80	M	VAC70	50	Unvaccinate	0.495	0.007	0.001	0.008	0.003	0.003	0.010	237.613	3.191	0.616	3.807	1.242	1.408	4.633	2376	31.910	6.160	38.070	12.420	14.080	46.330
150	original	0.6	60x8 weeks	Ag80	M	VAC70	50	Unvaccinate	0.495	0.007	0.001	0.008	0.003	0.003	0.010	237.613	3.191	0.616	3.807	1.242	1.408	4.633	2376	31.910	6.160	38.070	12.420	14.080	46.330
150	original	1	60x8 weeks	Ag80	M	VAC70	50	Unvaccinate	0.494	0.007	0.003	0.009	0.003	0.003	0.009	237.233	3.240	1.301	4.541	1.509	1.615	4.502	2372	32.400	13.010	45.410	15.090	16.150	45.020
150	original	1	60x8 weeks	Ag80	M	VAC70	50	Unvaccinate	0.494	0.007	0.003	0.009	0.003	0.003	0.009	237.233	3.240	1.301	4.541	1.509	1.615	4.502	2372	32.400	13.010	45.410	15.090	16.150	45.020
150	original	1	60x8 weeks	Ag80	M	VAC70	50	Unvaccinate	0.494	0.007	0.003	0.009	0.003	0.003	0.009	237.233	3.240	1.301	4.541	1.509	1.615	4.502	2372	32.400	13.010	45.410	15.090	16.150	45.020
150	original	1.2	60x8 weeks	Ag80	M	VAC70	50	Unvaccinate	0.494	0.007	0.003	0.010	0.003	0.004	0.009	237.034	3.209	1.677	4.886	1.654	1.704	4.553	2370	32.090	16.770	48.860	16.540	17.040	45.530
150	original	1.2	60x8 weeks	Ag80	M	VAC70	50	Unvaccinate	0.494	0.007	0.003	0.010	0.003	0.004	0.009	237.034	3.209	1.677	4.886	1.654	1.704	4.553	2370	32.090	16.770	48.860	16.540	17.040	45.530

150	original	1.2	60x8 weeks	Ag80	M	VAC70	50	Unvaccinate	0.494	0.007	0.003	0.010	0.003	0.004	0.009	237.034	3.209	1.677	4.886	1.654	1.704	4.553	2370	32.090	16.770	48.860	16.540	17.040	45.530
150	alpha	0.6	60x8 weeks	Ag80	M	VAC70	50	Unvaccinate	0.492	0.007	0.005	0.011	0.004	0.005	0.009	236.146	3.125	2.191	5.316	2.096	2.170	4.551	2361	31.250	21.910	53.160	20.960	21.700	45.510
150	alpha	0.6	60x8 weeks	Ag80	M	VAC70	50	Unvaccinate	0.492	0.007	0.005	0.011	0.004	0.005	0.009	236.146	3.125	2.191	5.316	2.096	2.170	4.551	2361	31.250	21.910	53.160	20.960	21.700	45.510
150	alpha	0.6	60x8 weeks	Ag80	M	VAC70	50	Unvaccinate	0.492	0.007	0.005	0.011	0.004	0.005	0.009	236.146	3.125	2.191	5.316	2.096	2.170	4.551	2361	31.250	21.910	53.160	20.960	21.700	45.510
150	alpha	1	60x8 weeks	Ag80	M	VAC70	50	Unvaccinate	0.487	0.006	0.012	0.018	0.007	0.008	0.009	233.750	3.102	5.712	8.814	3.374	3.722	4.267	2338	31.020	57.120	88.140	33.740	37.220	42.670
150	alpha	1	60x8 weeks	Ag80	M	VAC70	50	Unvaccinate	0.487	0.006	0.012	0.018	0.007	0.008	0.009	233.750	3.102	5.712	8.814	3.374	3.722	4.267	2338	31.020	57.120	88.140	33.740	37.220	42.670
150	alpha	1	60x8 weeks	Ag80	M	VAC70	50	Unvaccinate	0.487	0.006	0.012	0.018	0.007	0.008	0.009	233.750	3.102	5.712	8.814	3.374	3.722	4.267	2338	31.020	57.120	88.140	33.740	37.220	42.670
150	alpha	1.2	60x8 weeks	Ag80	M	VAC70	50	Unvaccinate	0.484	0.006	0.016	0.023	0.009	0.009	0.009	232.549	3.000	7.851	10.851	4.107	4.541	4.192	2325	30.000	78.510	108.510	41.070	45.410	41.920
150	alpha	1.2	60x8 weeks	Ag80	M	VAC70	50	Unvaccinate	0.484	0.006	0.016	0.023	0.009	0.009	0.009	232.549	3.000	7.851	10.851	4.107	4.541	4.192	2325	30.000	78.510	108.510	41.070	45.410	41.920
150	alpha	1.2	60x8 weeks	Ag80	M	VAC70	50	Unvaccinate	0.484	0.006	0.016	0.023	0.009	0.009	0.009	232.549	3.000	7.851	10.851	4.107	4.541	4.192	2325	30.000	78.510	108.510	41.070	45.410	41.920
150	delta	0.6	60x8 weeks	Ag80	M	VAC70	50	Unvaccinate	0.480	0.006	0.024	0.031	0.013	0.011	0.008	230.189	3.084	11.621	14.705	6.462	5.376	4.006	2302	30.840	116.210	147.050	64.620	53.760	40.060
150	delta	0.6	60x8 weeks	Ag80	M	VAC70	50	Unvaccinate	0.480	0.006	0.024	0.031	0.013	0.011	0.008	230.189	3.084	11.621	14.705	6.462	5.376	4.006	2302	30.840	116.210	147.050	64.620	53.760	40.060
150	delta	0.6	60x8 weeks	Ag80	M	VAC70	50	Unvaccinate	0.480	0.006	0.024	0.031	0.013	0.011	0.008	230.189	3.084	11.621	14.705	6.462	5.376	4.006	2302	30.840	116.210	147.050	64.620	53.760	40.060
150	delta	1	60x8 weeks	Ag80	M	VAC70	50	Unvaccinate	0.455	0.005	0.068	0.074	0.034	0.025	0.007	218.287	2.440	32.878	35.318	16.315	11.984	3.208	2183	24.400	328.780	353.180	163.150	119.840	32.080
150	delta	1	60x8 weeks	Ag80	M	VAC70	50	Unvaccinate	0.455	0.005	0.068	0.074	0.034	0.025	0.007	218.287	2.440	32.878	35.318	16.315	11.984	3.208	2183	24.400	328.780	353.180	163.150	119.840	32.080
150	delta	1	60x8 weeks	Ag80	M	VAC70	50	Unvaccinate	0.455	0.005	0.068	0.074	0.034	0.025	0.007	218.287	2.440	32.878	35.318	16.315	11.984	3.208	2183	24.400	328.780	353.180	163.150	119.840	32.080
150	delta	1.2	60x8 weeks	Ag80	M	VAC70	50	Unvaccinate	0.447	0.004	0.085	0.089	0.042	0.029	0.006	214.326	2.106	40.833	42.939	20.321	13.984	2.771	2143	21.060	408.330	429.390	203.210	139.840	27.710
150	delta	1.2	60x8 weeks	Ag80	M	VAC70	50	Unvaccinate	0.447	0.004	0.085	0.089	0.042	0.029	0.006	214.326	2.106	40.833	42.939	20.321	13.984	2.771	2143	21.060	408.330	429.390	203.210	139.840	27.710
150	delta	1.2	60x8 weeks	Ag80	M	VAC70	50	Unvaccinate	0.447	0.004	0.085	0.089	0.042	0.029	0.006	214.326	2.106	40.833	42.939	20.321	13.984	2.771	2143	21.060	408.330	429.390	203.210	139.840	27.710
150	original	0.6	60x8 weeks	Ag80	M	VAC70	75	Unvaccinate	0.248	0.005	0.001	0.006	0.002	0.001	0.005	118.866	2.433	0.245	2.678	1.058	0.655	2.300	1189	24.330	2.450	26.780	10.580	6.550	23.000
150	original	0.6	60x8 weeks	Ag80	M	VAC70	75	Unvaccinate	0.248	0.005	0.001	0.006	0.002	0.001	0.005	118.866	2.433	0.245	2.678	1.058	0.655	2.300	1189	24.330	2.450	26.780	10.580	6.550	23.000
150	original	0.6	60x8 weeks	Ag80	M	VAC70	75	Unvaccinate	0.248	0.005	0.001	0.006	0.002	0.001	0.005	118.866	2.433	0.245	2.678	1.058	0.655	2.300	1189	24.330	2.450	26.780	10.580	6.550	23.000
150	original	1	60x8 weeks	Ag80	M	VAC70	75	Unvaccinate	0.248	0.005	0.001	0.006	0.002	0.001	0.005	118.903	2.390	0.426	2.816	1.064	0.629	2.296	1189	23.900	4.260	28.160	10.640	6.290	22.960
150	original	1	60x8 weeks	Ag80	M	VAC70	75	Unvaccinate	0.248	0.005	0.001	0.006	0.002	0.001	0.005	118.903	2.390	0.426	2.816	1.064	0.629	2.296	1189	23.900	4.260	28.160	10.640	6.290	22.960
150	original	1	60x8 weeks	Ag80	M	VAC70	75	Unvaccinate	0.248	0.005	0.001	0.006	0.002	0.001	0.005	118.903	2.390	0.426	2.816	1.064	0.629	2.296	1189	23.900	4.260	28.160	10.640	6.290	22.960
150	original	1.2	60x8 weeks	Ag80	M	VAC70	75	Unvaccinate	0.247	0.005	0.001	0.006	0.002	0.001	0.005	118.749	2.414	0.595	3.009	1.166	0.691	2.315	1187	24.140	5.950	30.090	11.660	6.910	23.150
150	original	1.2	60x8 weeks	Ag80	M	VAC70	75	Unvaccinate	0.247	0.005	0.001	0.006	0.002	0.001	0.005	118.749	2.414	0.595	3.009	1.166	0.691	2.315	1187	24.140	5.950	30.090	11.660	6.910	23.150
150	original	1.2	60x8 weeks	Ag80	M	VAC70	75	Unvaccinate	0.247	0.005	0.001	0.006	0.002	0.001	0.005	118.749	2.414	0.595	3.009	1.166	0.691	2.315	1187	24.140	5.950	30.090	11.660	6.910	23.150
150	alpha	0.6	60x8 weeks	Ag80	M	VAC70	75	Unvaccinate	0.247	0.005	0.002	0.007	0.003	0.002	0.005	118.453	2.364	0.905	3.269	1.441	0.917	2.321	1185	23.640	9.050	32.690	14.410	9.170	23.210
150	alpha	0.6	60x8 weeks	Ag80	M	VAC70	75	Unvaccinate	0.247	0.005	0.002	0.007	0.003	0.002	0.005	118.453	2.364	0.905	3.269	1.441	0.917	2.321	1185	23.640	9.050	32.690	14.410	9.170	23.210
150	alpha	0.6	60x8 weeks	Ag80	M	VAC70	75	Unvaccinate	0.247	0.005	0.002	0.007	0.003	0.002	0.005	118.453	2.364	0.905	3.269	1.441	0.917	2.321	1185	23.640	9.050	32.690	14.410	9.170	23.210
150	alpha	1	60x8 weeks	Ag80	M	VAC70	75	Unvaccinate	0.246	0.005	0.005	0.009	0.004	0.002	0.005	117.895	2.338	2.172	4.510	2.093	1.175	2.219	1179	23.380	21.720	45.100	20.930	11.750	22.190
150	alpha	1	60x8 weeks	Ag80	M	VAC70	75	Unvaccinate	0.246	0.005	0.005	0.009	0.004	0.002	0.005	117.895	2.338	2.172	4.510	2.093	1.175	2.219	1179	23.380	21.720	45.100	20.930	11.750	22.190
150	alpha	1	60x8 weeks	Ag80	M	VAC70	75	Unvaccinate	0.246	0.005	0.005	0.009	0.004	0.002	0.005	117.895	2.338	2.172	4.510	2.093	1.175	2.219	1179	23.380	21.720	45.100	20.930	11.750	22.190
150	alpha	1.2	60x8 weeks	Ag80	M	VAC70	75	Unvaccinate	0.245	0.005	0.006	0.011	0.005	0.003	0.005	117.543	2.324	2.904	5.228	2.372	1.397	2.208	1175	23.240	29.040	52.280	23.720	13.970	22.080
150	alpha	1.2	60x8 weeks	Ag80	M	VAC70	75	Unvaccinate	0.245	0.005	0.006	0.011	0.005	0.003	0.005	117.543	2.324	2.904	5.228	2.372	1.397	2.208	1175	23.240	29.040	52.280	23.720	13.970	22.080
150	alpha	1.2	60x8 weeks	Ag80	M	VAC70	75	Unvaccinate	0.245	0.005	0.006	0.011	0.005	0.003	0.005	117.543	2.324	2.904	5.228	2.372	1.397	2.208	1175	23.240	29.040	52.280	23.720	13.970	22.080
150	delta	0.6	60x8 weeks	Ag80	M	VAC70	75	Unvaccinate	0.244	0.005	0.010	0.016	0.008	0.003	0.005	116.947	2.626	4.877	7.503	3.821	1.653	2.176	1169	26.260	48.770	75.030	38.210	16.530	21.760
150	delta	0.6	60x8 weeks	Ag80	M	VAC70	75	Unvaccinate	0.244	0.005	0.010	0.016	0.008	0.003	0.005	116.947	2.626	4.877	7.503	3.821	1.653	2.176	1169	26.260	48.770	75.030	38.210	16.530	21.760
150	delta	0.6	60x8 weeks	Ag80	M	VAC70	75	Unvaccinate	0.244	0.005	0.010	0.016	0.008	0.003	0.005	116.947	2.626	4.877	7.503	3.821	1.653	2.176	1169	26.260	48.770	75.030	38.210	16.530	21.760
150	delta	1	60x8 weeks	Ag80	M	VAC70	75	Unvaccinate	0.236	0.005	0.032	0.037	0.020	0.007	0.004	113.383	2.367	15.599	17.966	9.467	3.551	1.937	1134	23.670	155.990	179.660	94.670	35.510	19.370
150	delta	1	60x8 weeks	Ag80	M	VAC70	75	Unvaccinate	0.236	0.005	0.032	0.037	0.020	0.007	0.004	113.383	2.367	15.599	17.966	9.467	3.551	1.937	1134	23.670	155.990	179.660	94.670	35.510	19.370
150	delta	1	60x8 weeks	Ag80	M	VAC70	75	Unvaccinate	0.236	0.005	0.032	0.037	0.020	0.007	0.004	113.383	2.367	15.599	17.966	9.467	3.551	1.937	1134	23.670	155.990	179.660	94.670	35.510	19.370
150	delta	1.2	60x8 weeks	Ag80	M	VAC70	75	Unvaccinate	0.231	0.005	0.050	0.055	0.029	0.010	0.004	110.890	2.244	24.134	26.378	14.091	4.980	1.741	1109	22.440	241.340	263.780	140.910	49.800	17.410
150	delta	1.2	60x8 weeks	Ag80	M	VAC70	75	Unvaccinate	0.231	0.005	0.050	0.055	0.029	0.010	0.004	110.890	2.244	24.134	26.378	14.091	4.980	1.741	1109	22.440	241.340	263.780	140.910	49.800	17.410
150	delta	1.2	60x8 weeks	Ag80	M	VAC70	75	Unvaccinate	0.231	0.005	0.050	0.055	0.029	0.010	0.004	110.890	2.244	24.134	26.378	14.091	4.980	1.741	1109	22.440	241.340	263.780	140.910	49.800	17.410
150	original	0.6	60x8 weeks	Ag80	M	VAC70	100	Unvaccinate	0.000	0.003	0.000	0.003	0.002	0.000	0.000	0.000	1.620	0.013	1.633	0.808	0.000	0.000	0	16.200	0.130	16.330	8.080	0.000	0.000
150	original	0.6	60x8 weeks	Ag80	M	VAC70	100	Unvaccinate	0.000	0.003	0.000	0.003	0.002	0.000	0.000	0.000	1.620	0.013	1.633	0.808	0.000	0.000	0	16.200	0.130	16.330	8.080	0.000	0.000
150	original	0.6	60x8 weeks	Ag80	M	VAC70	100	Unvaccinate	0.000	0.003	0.000	0.003	0.002	0.000	0.000	0.000	1.620	0.013	1.633	0.808	0.000	0.000	0	16.200	0.130	16.330	8.080	0.000	0.000
150	original	1	60x8 weeks	Ag80	M	VAC70	100	Unvaccinate	0.000	0.003	0.000	0.003	0.002	0.000	0.000	0.000	1.572	0.027	1.599	0.779	0.000	0.000	0	15.720	0.270	15.990	7.790	0.000	0.000
150	original	1	60x8 weeks	Ag80	M	VAC70	100	Unvaccinate	0.000	0.003	0.000	0.003	0.002	0.000	0.000	0.000	1.572	0.027	1.599	0.779	0.000	0.000	0	15.720	0.270	15.990	7.790	0.000	0.000
150	original	1	60x8 weeks	Ag80	M	VAC70	100	Unvaccinate	0.000	0.003	0.000	0.003	0.002	0.000	0.000	0.000	1.572	0.027	1.599	0.779	0.000	0.000	0	15.720	0.270	15.990	7.790	0.000	0.000
150	original	1.2	60x8 weeks	Ag80	M	VAC70	100	Unvaccinate	0.000	0.003	0.000	0.004	0.002	0.000	0.000	0.000	1.679	0.025	1.704	0.837	0.000	0.000	0	16.790	0.250	17.040	8.370	0.000	0.000
150	original	1.2	60x8 weeks	Ag80	M	VAC70	100	Unvaccinate	0.000	0.003	0.000	0.004	0.002	0.000	0.000	0.000	1.679	0.025	1.704	0.837	0.000	0.000	0	16.790	0.250	17.040	8.370	0.000	0.000
150	original	1.2	60x8 weeks	Ag80	M	VAC70	100	Unvaccinate	0.000	0.003	0.000	0.004	0.002	0.000	0.000	0.000	1.679	0.025	1.704	0.837	0.000	0.000	0	16.790	0.250	17.040	8.370	0.000	0.000
150	alpha	0.6	60x8 weeks	Ag80	M	VAC70	100	Unvaccinate	0.000	0.003	0.001	0.004	0.002	0.000	0.000	0.000	1.535	0.324	1.859	1.114	0.000	0.000	0	15.350	3.240	18.590	11.140	0.000	0.000
150	alpha	0.6	60x8 weeks	Ag80	M	VAC70	100	Unvaccinate	0.000	0.003	0.001	0.004	0.002	0.000	0.000	0.000	1.535	0.324	1.859	1.114	0.000	0.000	0	15.350	3.240	18.590	11.140	0.000	0.000
150	alpha	0.6	60x8 weeks	Ag80	M	VAC70	100	Unvaccinate	0.000	0.003	0.001	0.004	0.002	0.000	0.000	0.000	1.535	0.324	1.859	1.114	0.000	0.000	0	15.350	3.240	18.590	11.140	0.000	0.000
150	alpha	1	60x8 weeks	Ag80	M	VAC70	100	Unvaccinate	0.000	0.003	0.001	0.005	0.003	0.000	0.000	0.000	1.641	0.711	2.352	1.355	0.000	0.000	0	16.410	7.110	23.520	13.550	0.000	0.000
150	alpha	1	60x8 weeks	Ag80	M	VAC70	100	Unvaccinate	0.000	0.003	0.001	0.005	0.003	0.000	0.000	0.000	1.641	0.711	2.352	1.355	0.000	0.000	0	16.410	7.110	23.520	13.550	0.000	0.000
150	alpha	1	60x8 weeks	Ag80	M	VAC70	100	Unvaccinate	0.000	0.003	0.001	0.005	0.003	0.000	0.000	0.000	1.641	0.711	2.352	1.355	0.000	0.000	0	16.410	7.110	23.520	13.550	0.000	0.000
150	alpha	1.2	60x8 weeks	Ag80	M	VAC70	100	Unvaccinate	0.000	0.003	0.002	0.005	0.003	0.000	0.000	0.000	1.624	0.802	2.426	1.375	0.000	0.000	0	16.240	8.020	24.260	13.750	0.000	0.000
150	alpha	1.2	60x8 weeks	Ag80	M	VAC70	100	Unvaccinate	0.000	0.003	0.002	0.005	0.003	0.000	0.000	0.000	1.624	0.802	2.426	1.375	0.000	0.000	0	16.240	8.020	24.260	13.750	0.000	0.000
150	alpha	1.2	60x8 weeks	Ag80	M	VAC70	100	Unvaccinate	0.000	0.003	0.002	0.005	0.003	0.000	0.000	0.000	1.624	0.802	2.426	1.375	0.000	0.000	0	16.240	8.020	24.260	13.750	0.000	0.000
150	delta	0.6	60x8 weeks	Ag80	M	VAC70	100	Unvaccinate	0.000	0.004	0.003	0.007	0.004	0.000	0.000	0.000	2.016	1.516	3.532	2.155	0.000	0.000	0	20.160	15.160	35.320	21.550	0.000	0.000
150	delta	0.6	60x8 weeks	Ag80	M	VAC70	100	Unvaccinate	0.000	0.004	0.003	0.007	0.004	0.000	0.000	0.000	2.016	1.516	3.532	2.155	0.000	0.000	0	20.160	15.160	35.320	21.550	0.000	0.000
150	delta	0.6	60x8 weeks	Ag80	M	VAC70	100	Unvaccinate	0.000	0.004	0.003	0.007	0.004	0.000	0.000	0.000	2.016	1.516	3.532	2.155	0.000	0.000	0	20.160	15.160	35.320	21.550	0.000	0.000
150	delta	1	60x8 weeks	Ag80	M	VAC70	100	Unvaccinate	0.000	0.004	0.011	0.015	0.009	0.000	0.000	0.000	2.018	5.389	7.407	4.529	0.000	0.000	0	20.180	53.890	74.070	45.290	0.000	0.000
150	delta	1	60x8 weeks	Ag80	M	VAC70	100	Unvaccinate	0.000	0.004	0.011	0.015	0.009	0.000	0.000	0.000	2.018	5.389	7.407	4.529	0.000	0.000	0	20.180	53.890	74.070	45.290	0.000	0.000
150	delta	1	60x8 weeks	Ag80	M	VAC70	100	Unvaccinate	0.000	0.004	0.011	0.015	0.009	0.000	0.000	0.000	2.018	5.389	7.407	4.529	0.000	0.000	0	20.180	53.890	74.070	45.290	0.000	0.000
150	delta	1.2	60x8 weeks	Ag80	M	VAC70	100	Unvaccinate	0.000	0.004	0.018	0.022	0.013	0.000	0.000	0.000	2.027	8.405	10.432	6.317	0.000	0.000	0	20.270	84.050	104.320	63.170	0.000	0.000

150	delta	1.2	60x8 weeks	Ag80	M	VAC70	100	Jnvaccinate	0.000	0.004	0.018	0.022	0.013	0.000	0.000	0.000	2.027	8.405	10.432	6.317	0.000	0.000	0	20.270	84.050	104.320	63.170	0.000	0.000
150	delta	1.2	60x8 weeks	Ag80	M	VAC70	100	Jnvaccinate	0.000	0.004	0.018	0.022	0.013	0.000	0.000	0.000	2.027	8.405	10.432	6.317	0.000	0.000	0	20.270	84.050	104.320	63.170	0.000	0.000
150	original	0.6	60x8 weeks	Ag80	M	VAC50	25	Jnvaccinate	0.742	0.009	0.003	0.012	0.004	0.005	0.014	355.978	4.205	1.489	5.694	1.789	2.282	6.821	3560	42.050	14.890	56.940	17.890	22.820	68.210
150	original	0.6	60x8 weeks	Ag80	M	VAC50	25	Jnvaccinate	0.742	0.009	0.003	0.012	0.004	0.005	0.014	355.978	4.205	1.489	5.694	1.789	2.282	6.821	3560	42.050	14.890	56.940	17.890	22.820	68.210
150	original	0.6	60x8 weeks	Ag80	M	VAC50	25	Jnvaccinate	0.742	0.009	0.003	0.012	0.004	0.005	0.014	355.978	4.205	1.489	5.694	1.789	2.282	6.821	3560	42.050	14.890	56.940	17.890	22.820	68.210
150	original	1	60x8 weeks	Ag80	M	VAC50	25	Jnvaccinate	0.739	0.009	0.006	0.015	0.005	0.006	0.014	354.800	4.149	3.087	7.236	2.333	2.987	6.807	3548	41.490	30.870	72.360	23.330	29.870	68.070
150	original	1	60x8 weeks	Ag80	M	VAC50	25	Jnvaccinate	0.739	0.009	0.006	0.015	0.005	0.006	0.014	354.800	4.149	3.087	7.236	2.333	2.987	6.807	3548	41.490	30.870	72.360	23.330	29.870	68.070
150	original	1	60x8 weeks	Ag80	M	VAC50	25	Jnvaccinate	0.739	0.009	0.006	0.015	0.005	0.006	0.014	354.800	4.149	3.087	7.236	2.333	2.987	6.807	3548	41.490	30.870	72.360	23.330	29.870	68.070
150	original	1.2	60x8 weeks	Ag80	M	VAC50	25	Jnvaccinate	0.738	0.009	0.009	0.017	0.005	0.007	0.014	354.131	4.089	4.180	8.269	2.606	3.416	6.735	3541	40.890	41.800	82.690	26.060	34.160	67.350
150	original	1.2	60x8 weeks	Ag80	M	VAC50	25	Jnvaccinate	0.738	0.009	0.009	0.017	0.005	0.007	0.014	354.131	4.089	4.180	8.269	2.606	3.416	6.735	3541	40.890	41.800	82.690	26.060	34.160	67.350
150	original	1.2	60x8 weeks	Ag80	M	VAC50	25	Jnvaccinate	0.738	0.009	0.009	0.017	0.005	0.007	0.014	354.131	4.089	4.180	8.269	2.606	3.416	6.735	3541	40.890	41.800	82.690	26.060	34.160	67.350
150	alpha	0.6	60x8 weeks	Ag80	M	VAC50	25	Jnvaccinate	0.735	0.008	0.011	0.019	0.007	0.009	0.014	352.846	3.963	5.059	9.022	3.202	4.310	6.591	3528	39.630	50.590	90.220	32.020	43.100	65.910
150	alpha	0.6	60x8 weeks	Ag80	M	VAC50	25	Jnvaccinate	0.735	0.008	0.011	0.019	0.007	0.009	0.014	352.846	3.963	5.059	9.022	3.202	4.310	6.591	3528	39.630	50.590	90.220	32.020	43.100	65.910
150	alpha	0.6	60x8 weeks	Ag80	M	VAC50	25	Jnvaccinate	0.735	0.008	0.011	0.019	0.007	0.009	0.014	352.846	3.963	5.059	9.022	3.202	4.310	6.591	3528	39.630	50.590	90.220	32.020	43.100	65.910
150	alpha	1	60x8 weeks	Ag80	M	VAC50	25	Jnvaccinate	0.719	0.008	0.031	0.039	0.014	0.019	0.013	345.046	3.716	14.835	18.551	6.696	8.952	6.070	3450	37.160	148.350	185.510	66.960	89.520	60.700
150	alpha	1	60x8 weeks	Ag80	M	VAC50	25	Jnvaccinate	0.719	0.008	0.031	0.039	0.014	0.019	0.013	345.046	3.716	14.835	18.551	6.696	8.952	6.070	3450	37.160	148.350	185.510	66.960	89.520	60.700
150	alpha	1	60x8 weeks	Ag80	M	VAC50	25	Jnvaccinate	0.719	0.008	0.031	0.039	0.014	0.019	0.013	345.046	3.716	14.835	18.551	6.696	8.952	6.070	3450	37.160	148.350	185.510	66.960	89.520	60.700
150	alpha	1.2	60x8 weeks	Ag80	M	VAC50	25	Jnvaccinate	0.706	0.007	0.046	0.054	0.020	0.026	0.012	338.938	3.413	22.269	25.682	9.447	12.491	5.772	3389	34.130	222.690	256.820	94.470	124.910	57.720
150	alpha	1.2	60x8 weeks	Ag80	M	VAC50	25	Jnvaccinate	0.706	0.007	0.046	0.054	0.020	0.026	0.012	338.938	3.413	22.269	25.682	9.447	12.491	5.772	3389	34.130	222.690	256.820	94.470	124.910	57.720
150	alpha	1.2	60x8 weeks	Ag80	M	VAC50	25	Jnvaccinate	0.706	0.007	0.046	0.054	0.020	0.026	0.012	338.938	3.413	22.269	25.682	9.447	12.491	5.772	3389	34.130	222.690	256.820	94.470	124.910	57.720
150	delta	0.6	60x8 weeks	Ag80	M	VAC50	25	Jnvaccinate	0.696	0.007	0.058	0.065	0.027	0.030	0.011	333.934	3.392	27.933	31.325	12.770	14.274	5.236	3339	33.920	279.330	313.250	127.700	142.740	52.360
150	delta	0.6	60x8 weeks	Ag80	M	VAC50	25	Jnvaccinate	0.696	0.007	0.058	0.065	0.027	0.030	0.011	333.934	3.392	27.933	31.325	12.770	14.274	5.236	3339	33.920	279.330	313.250	127.700	142.740	52.360
150	delta	0.6	60x8 weeks	Ag80	M	VAC50	25	Jnvaccinate	0.696	0.007	0.058	0.065	0.027	0.030	0.011	333.934	3.392	27.933	31.325	12.770	14.274	5.236	3339	33.920	279.330	313.250	127.700	142.740	52.360
150	delta	1	60x8 weeks	Ag80	M	VAC50	25	Jnvaccinate	0.653	0.005	0.108	0.113	0.048	0.052	0.007	313.269	2.318	52.062	54.380	23.152	24.987	3.418	3133	23.180	520.620	543.800	231.520	249.870	34.180
150	delta	1	60x8 weeks	Ag80	M	VAC50	25	Jnvaccinate	0.653	0.005	0.108	0.113	0.048	0.052	0.007	313.269	2.318	52.062	54.380	23.152	24.987	3.418	3133	23.180	520.620	543.800	231.520	249.870	34.180
150	delta	1	60x8 weeks	Ag80	M	VAC50	25	Jnvaccinate	0.653	0.005	0.108	0.113	0.048	0.052	0.007	313.269	2.318	52.062	54.380	23.152	24.987	3.418	3133	23.180	520.620	543.800	231.520	249.870	34.180
150	delta	1.2	60x8 weeks	Ag80	M	VAC50	25	Jnvaccinate	0.650	0.004	0.112	0.116	0.050	0.053	0.007	311.828	2.057	53.577	55.634	23.772	25.471	3.293	3118	20.570	535.770	556.340	237.720	254.710	32.930
150	delta	1.2	60x8 weeks	Ag80	M	VAC50	25	Jnvaccinate	0.650	0.004	0.112	0.116	0.050	0.053	0.007	311.828	2.057	53.577	55.634	23.772	25.471	3.293	3118	20.570	535.770	556.340	237.720	254.710	32.930
150	delta	1.2	60x8 weeks	Ag80	M	VAC50	25	Jnvaccinate	0.650	0.004	0.112	0.116	0.050	0.053	0.007	311.828	2.057	53.577	55.634	23.772	25.471	3.293	3118	20.570	535.770	556.340	237.720	254.710	32.930
150	original	0.6	60x8 weeks	Ag80	M	VAC50	50	Jnvaccinate	0.495	0.008	0.002	0.010	0.003	0.003	0.010	237.644	3.718	0.847	4.565	1.615	1.330	4.609	2376	37.180	8.470	45.650	16.150	13.300	46.090
150	original	0.6	60x8 weeks	Ag80	M	VAC50	50	Jnvaccinate	0.495	0.008	0.002	0.010	0.003	0.003	0.010	237.644	3.718	0.847	4.565	1.615	1.330	4.609	2376	37.180	8.470	45.650	16.150	13.300	46.090
150	original	0.6	60x8 weeks	Ag80	M	VAC50	50	Jnvaccinate	0.495	0.008	0.002	0.010	0.003	0.003	0.010	237.644	3.718	0.847	4.565	1.615	1.330	4.609	2376	37.180	8.470	45.650	16.150	13.300	46.090
150	original	1	60x8 weeks	Ag80	M	VAC50	50	Jnvaccinate	0.494	0.008	0.003	0.011	0.004	0.003	0.010	237.246	3.706	1.549	5.255	1.825	1.640	4.574	2372	37.060	15.490	52.550	18.250	16.400	45.740
150	original	1	60x8 weeks	Ag80	M	VAC50	50	Jnvaccinate	0.494	0.008	0.003	0.011	0.004	0.003	0.010	237.246	3.706	1.549	5.255	1.825	1.640	4.574	2372	37.060	15.490	52.550	18.250	16.400	45.740
150	original	1	60x8 weeks	Ag80	M	VAC50	50	Jnvaccinate	0.494	0.008	0.003	0.011	0.004	0.003	0.010	237.246	3.706	1.549	5.255	1.825	1.640	4.574	2372	37.060	15.490	52.550	18.250	16.400	45.740
150	original	1.2	60x8 weeks	Ag80	M	VAC50	50	Jnvaccinate	0.494	0.008	0.004	0.012	0.004	0.004	0.009	236.963	3.691	1.976	5.667	2.044	1.826	4.382	2370	36.910	19.760	56.670	20.440	18.260	43.820
150	original	1.2	60x8 weeks	Ag80	M	VAC50	50	Jnvaccinate	0.494	0.008	0.004	0.012	0.004	0.004	0.009	236.963	3.691	1.976	5.667	2.044	1.826	4.382	2370	36.910	19.760	56.670	20.440	18.260	43.820
150	original	1.2	60x8 weeks	Ag80	M	VAC50	50	Jnvaccinate	0.494	0.008	0.004	0.012	0.004	0.004	0.009	236.963	3.691	1.976	5.667	2.044	1.826	4.382	2370	36.910	19.760	56.670	20.440	18.260	43.820
150	alpha	0.6	60x8 weeks	Ag80	M	VAC50	50	Jnvaccinate	0.491	0.008	0.006	0.014	0.006	0.005	0.009	235.781	3.617	3.025	6.642	2.837	2.399	4.496	2358	36.170	30.250	66.420	28.370	23.990	44.960
150	alpha	0.6	60x8 weeks	Ag80	M	VAC50	50	Jnvaccinate	0.491	0.008	0.006	0.014	0.006	0.005	0.009	235.781	3.617	3.025	6.642	2.837	2.399	4.496	2358	36.170	30.250	66.420	28.370	23.990	44.960
150	alpha	0.6	60x8 weeks	Ag80	M	VAC50	50	Jnvaccinate	0.491	0.008	0.006	0.014	0.006	0.005	0.009	235.781	3.617	3.025	6.642	2.837	2.399	4.496	2358	36.170	30.250	66.420	28.370	23.990	44.960
150	alpha	1	60x8 weeks	Ag80	M	VAC50	50	Jnvaccinate	0.485	0.007	0.018	0.025	0.010	0.009	0.009	232.782	3.411	8.428	11.839	5.001	4.199	4.327	2328	34.110	84.280	118.390	50.010	41.990	43.270
150	alpha	1	60x8 weeks	Ag80	M	VAC50	50	Jnvaccinate	0.485	0.007	0.018	0.025	0.010	0.009	0.009	232.782	3.411	8.428	11.839	5.001	4.199	4.327	2328	34.110	84.280	118.390	50.010	41.990	43.270
150	alpha	1	60x8 weeks	Ag80	M	VAC50	50	Jnvaccinate	0.485	0.007	0.018	0.025	0.010	0.009	0.009	232.782	3.411	8.428	11.839	5.001	4.199	4.327	2328	34.110	84.280	118.390	50.010	41.990	43.270
150	alpha	1.2	60x8 weeks	Ag80	M	VAC50	50	Jnvaccinate	0.480	0.007	0.026	0.033	0.014	0.012	0.009	230.435	3.228	12.486	15.714	6.808	5.593	4.139	2304	32.280	124.860	157.140	68.080	55.930	41.390
150	alpha	1.2	60x8 weeks	Ag80	M	VAC50	50	Jnvaccinate	0.480	0.007	0.026	0.033	0.014	0.012	0.009	230.435	3.228	12.486	15.714	6.808	5.593	4.139	2304	32.280	124.860	157.140	68.080	55.930	41.390
150	alpha	1.2	60x8 weeks	Ag80	M	VAC50	50	Jnvaccinate	0.480	0.007	0.026	0.033	0.014	0.012	0.009	230.435	3.228	12.486	15.714	6.808	5.593	4.139	2304	32.280	124.860	157.140	68.080	55.930	41.390
150	delta	0.6	60x8 weeks	Ag80	M	VAC50	50	Jnvaccinate	0.473	0.007	0.041	0.048	0.023	0.015	0.008	226.959	3.420	19.572	22.992	10.885	7.202	3.856	2270	34.200	195.720	229.920	108.850	72.020	38.560
150	delta	0.6	60x8 weeks	Ag80	M	VAC50	50	Jnvaccinate	0.473	0.007	0.041	0.048	0.023	0.015	0.008	226.959	3.420	19.572	22.992	10.885	7.202	3.856	2270	34.200	195.720	229.920	108.850	72.020	38.560
150	delta	0.6	60x8 weeks	Ag80	M	VAC50	50	Jnvaccinate	0.473	0.007	0.041	0.048	0.023	0.015	0.008	226.959	3.420	19.572	22.992	10.885	7.202	3.856	2270	34.200	195.720	229.920	108.850	72.020	38.560
150	delta	1	60x8 weeks	Ag80	M	VAC50	50	Jnvaccinate	0.444	0.005	0.095	0.100	0.050	0.030	0.006	212.882	2.420	45.417	47.837	23.918	14.370	2.749	2129	24.200	454.170	478.370	239.180	143.700	27.490
150	delta	1	60x8 weeks	Ag80	M	VAC50	50	Jnvaccinate	0.444	0.005	0.095	0.100	0.050	0.030	0.006	212.882	2.420	45.417	47.837	23.918	14.370	2.749	2129	24.200	454.170	478.370	239.180	143.700	27.490
150	delta	1	60x8 weeks	Ag80	M	VAC50	50	Jnvaccinate	0.444	0.005	0.095	0.100	0.050	0.030	0.006	212.882	2.420	45.417	47.837	23.918	14.370	2.749	2129	24.200	454.170	478.370	239.180	143.700	27.490
150	delta	1.2	60x8 weeks	Ag80	M	VAC50	50	Jnvaccinate	0.435	0.005	0.107	0.112	0.057	0.034	0.005	208.649	2.223	51.384	53.607	27.418	16.266	2.407	2086	22.230	513.840	536.070	274.180	162.660	24.070
150	delta	1.2	60x8 weeks	Ag80	M	VAC50	50	Jnvaccinate	0.435	0.005	0.107	0.112	0.057	0.034	0.005	208.649	2.223	51.384	53.607	27.418	16.266	2.407	2086	22.230	513.840	536.070	274.180	162.660	24.070
150	delta	1.2	60x8 weeks	Ag80	M	VAC50	50	Jnvaccinate	0.435	0.005	0.107	0.112	0.057	0.034	0.005	208.649	2.223	51.384	53.607	27.418	16.266	2.407	2086	22.230	513.840	536.070	274.180	162.660	24.070
150	original	0.6	60x8 weeks	Ag80	M	VAC50	75	Jnvaccinate	0.248	0.007	0.001	0.007	0.003	0.001	0.005	118.939	3.187	0.352	3.539	1.531	0.602	2.287	1189	31.870	3.520	35.390	15.310	6.020	22.870
150	original	0.6	60x8 weeks	Ag80	M	VAC50	75	Jnvaccinate	0.248	0.007	0.001	0.007	0.003	0.001	0.005	118.939	3.187	0.352	3.539	1.531	0.602	2.287	1189	31.870	3.520	35.390	15.310	6.020	22.870
150	original	0.6	60x8 weeks	Ag80	M	VAC50	75	Jnvaccinate	0.248	0.007	0.001	0.007	0.003	0.001	0.005	118.939	3.187	0.352	3.539	1.531	0.602	2.287	1189	31.870	3.520	35.390	15.310	6.020	22.870
150	original	1	60x8 weeks	Ag80	M	VAC50	75	Jnvaccinate	0.248	0.007	0.001	0.008	0.003	0.001	0.005	118.815	3.185	0.621	3.806	1.639	0.686	2.337	1188	31.850	6.210	38.060	16.390	6.860	23.370
150	original	1	60x8 weeks	Ag80	M	VAC50	75	Jnvaccinate	0.248	0.007	0.001	0.008	0.003	0.001	0.005	118.815	3.185	0.621	3.806	1.639	0.686	2.337	1188	31.850	6.210	38.060	16.390	6.860	23.370
150	original	1	60x8 weeks	Ag80	M	VAC50	75	Jnvaccinate	0.248	0.007	0.001	0.008	0.003	0.001	0.005	118.815	3.185	0.621	3.806	1.639	0.686	2.337	1188	31.850	6.210	38.060	16.390	6.860	23.370
150	original	1.2	60x8 weeks	Ag80	M	VAC50	75	Jnvaccinate	0.247	0.006	0.001	0.008	0.003	0.001	0.005	118.779	3.107	0.715	3.822	1.619	0.693	2.318	1188	31.070	7.150	38.220	16.190	6.930	23.180
150	original	1.2	60x8 weeks	Ag80	M	VAC50	75	Jnvaccinate	0.247	0.006	0.001	0.008	0.003	0.001	0.005	118.779	3.107	0.715	3.822	1.619	0.693	2.318	1188	31.070	7.150	38.220	16.190	6.930	23.180
150	original	1.2	60x8 weeks	Ag80	M	VAC50	75	Jnvaccinate	0.247	0.006	0.001	0.008	0.003	0.001	0.005	118.779	3.107	0.715	3.822	1.619	0.693	2.318	1188	31.070	7.150	38.220	16.190	6.930	23.180
150	alpha	0.6	60x8 weeks	Ag80	M	VAC50	75	Jnvaccinate	0.246	0.006	0.004	0.010	0.005	0.002	0.005	118.196	3.098	1.734	4.832	2.421	1.054	2.316	1182	30.980	17.340	48.320	24.210	10.540	23.160
150	alpha	0.6	60x8 weeks	Ag80	M	VAC50	75	Jnvaccinate	0.246	0.006	0.004	0.010	0.005	0.002	0.005	118.196	3.098	1.734	4.832	2.421	1.054	2.316	1182	30.980	17.340	48.320	24.210	10.540	23.160
150	alpha	0.6	60x8 weeks	Ag80	M	VAC50	75	Jnvaccinate	0.246	0.006	0.004	0.010	0.005	0.002	0.005	118.196	3.098	1.734	4.832	2.421	1.054	2.316	1182	30.980	17.340	48.320	24.210	10.540	23.160
150	alpha	1	60x8 weeks	Ag80	M	VAC50	75	Jnvaccinate	0.245	0.006	0.009	0.015	0.007	0.003	0.005	117.524	2.877	4.129	7.006	3.576	1.424	2.188	1175	28.770	41.290	70.060	35.760	14.240	21.880
150	alpha	1	60x8 weeks	Ag80	M	VAC50	75	Jnvaccinate	0.245	0.006	0.009	0.015	0.007	0.003	0.005	117.524	2.877	4.129	7.006	3.576	1.424	2.188	1175	28.770	41.290	70.060	35.760	14.240	21.880
150	alpha	1	60x8 weeks	Ag80	M	VAC50	75	Jnvaccinate	0.245	0.006	0.009	0.015	0.007	0.003	0.005	117.524	2.877	4.129	7.006	3.576	1.424	2.188	1175	28.770	41.290	70.060	35.760	14.240	21.880

150	alpha	1.2	60x8 weeks	Ag80	M	VAC50	75	Unvaccinate	0.244	0.006	0.013	0.019	0.009	0.004	0.004	116.904	2.875	6.178	9.053	4.519	1.824	2.142	1169	28.750	61.780	90.530	45.190	18.240	21.420
150	alpha	1.2	60x8 weeks	Ag80	M	VAC50	75	Unvaccinate	0.244	0.006	0.013	0.019	0.009	0.004	0.004	116.904	2.875	6.178	9.053	4.519	1.824	2.142	1169	28.750	61.780	90.530	45.190	18.240	21.420
150	alpha	1.2	60x8 weeks	Ag80	M	VAC50	75	Unvaccinate	0.244	0.006	0.013	0.019	0.009	0.004	0.004	116.904	2.875	6.178	9.053	4.519	1.824	2.142	1169	28.750	61.780	90.530	45.190	18.240	21.420
150	delta	0.6	60x8 weeks	Ag80	M	VAC50	75	Unvaccinate	0.241	0.007	0.024	0.031	0.017	0.005	0.004	115.473	3.355	11.607	14.962	8.133	2.440	2.089	1155	33.550	116.070	149.620	81.330	24.400	20.890
150	delta	0.6	60x8 weeks	Ag80	M	VAC50	75	Unvaccinate	0.241	0.007	0.024	0.031	0.017	0.005	0.004	115.473	3.355	11.607	14.962	8.133	2.440	2.089	1155	33.550	116.070	149.620	81.330	24.400	20.890
150	delta	0.6	60x8 weeks	Ag80	M	VAC50	75	Unvaccinate	0.241	0.007	0.024	0.031	0.017	0.005	0.004	115.473	3.355	11.607	14.962	8.133	2.440	2.089	1155	33.550	116.070	149.620	81.330	24.400	20.890
150	delta	1	60x8 weeks	Ag80	M	VAC50	75	Unvaccinate	0.226	0.005	0.075	0.081	0.047	0.012	0.003	108.618	2.594	36.162	38.756	22.381	5.995	1.568	1086	25.940	361.620	387.560	223.810	59.950	15.680
150	delta	1	60x8 weeks	Ag80	M	VAC50	75	Unvaccinate	0.226	0.005	0.075	0.081	0.047	0.012	0.003	108.618	2.594	36.162	38.756	22.381	5.995	1.568	1086	25.940	361.620	387.560	223.810	59.950	15.680
150	delta	1	60x8 weeks	Ag80	M	VAC50	75	Unvaccinate	0.226	0.005	0.075	0.081	0.047	0.012	0.003	108.618	2.594	36.162	38.756	22.381	5.995	1.568	1086	25.940	361.620	387.560	223.810	59.950	15.680
150	delta	1.2	60x8 weeks	Ag80	M	VAC50	75	Unvaccinate	0.222	0.005	0.093	0.098	0.057	0.015	0.003	106.374	2.225	44.827	47.052	27.518	7.214	1.326	1064	22.250	448.270	470.520	275.180	72.140	13.260
150	delta	1.2	60x8 weeks	Ag80	M	VAC50	75	Unvaccinate	0.222	0.005	0.093	0.098	0.057	0.015	0.003	106.374	2.225	44.827	47.052	27.518	7.214	1.326	1064	22.250	448.270	470.520	275.180	72.140	13.260
150	delta	1.2	60x8 weeks	Ag80	M	VAC50	75	Unvaccinate	0.222	0.005	0.093	0.098	0.057	0.015	0.003	106.374	2.225	44.827	47.052	27.518	7.214	1.326	1064	22.250	448.270	470.520	275.180	72.140	13.260
150	original	0.6	60x8 weeks	Ag80	M	VAC50	100	Unvaccinate	0.000	0.005	0.000	0.005	0.003	0.000	0.000	0.000	2.540	0.048	2.588	1.345	0.000	0.000	0	25.400	0.480	25.880	13.450	0.000	0.000
150	original	0.6	60x8 weeks	Ag80	M	VAC50	100	Unvaccinate	0.000	0.005	0.000	0.005	0.003	0.000	0.000	0.000	2.540	0.048	2.588	1.345	0.000	0.000	0	25.400	0.480	25.880	13.450	0.000	0.000
150	original	0.6	60x8 weeks	Ag80	M	VAC50	100	Unvaccinate	0.000	0.005	0.000	0.005	0.003	0.000	0.000	0.000	2.540	0.048	2.588	1.345	0.000	0.000	0	25.400	0.480	25.880	13.450	0.000	0.000
150	original	1	60x8 weeks	Ag80	M	VAC50	100	Unvaccinate	0.000	0.005	0.000	0.005	0.003	0.000	0.000	0.000	2.484	0.059	2.543	1.311	0.000	0.000	0	24.840	0.590	25.430	13.110	0.000	0.000
150	original	1	60x8 weeks	Ag80	M	VAC50	100	Unvaccinate	0.000	0.005	0.000	0.005	0.003	0.000	0.000	0.000	2.484	0.059	2.543	1.311	0.000	0.000	0	24.840	0.590	25.430	13.110	0.000	0.000
150	original	1	60x8 weeks	Ag80	M	VAC50	100	Unvaccinate	0.000	0.005	0.000	0.005	0.003	0.000	0.000	0.000	2.484	0.059	2.543	1.311	0.000	0.000	0	24.840	0.590	25.430	13.110	0.000	0.000
150	original	1.2	60x8 weeks	Ag80	M	VAC50	100	Unvaccinate	0.000	0.006	0.000	0.006	0.003	0.000	0.000	0.000	2.684	0.067	2.751	1.401	0.000	0.000	0	26.840	0.670	27.510	14.010	0.000	0.000
150	original	1.2	60x8 weeks	Ag80	M	VAC50	100	Unvaccinate	0.000	0.006	0.000	0.006	0.003	0.000	0.000	0.000	2.684	0.067	2.751	1.401	0.000	0.000	0	26.840	0.670	27.510	14.010	0.000	0.000
150	original	1.2	60x8 weeks	Ag80	M	VAC50	100	Unvaccinate	0.000	0.006	0.000	0.006	0.003	0.000	0.000	0.000	2.684	0.067	2.751	1.401	0.000	0.000	0	26.840	0.670	27.510	14.010	0.000	0.000
150	alpha	0.6	60x8 weeks	Ag80	M	VAC50	100	Unvaccinate	0.000	0.005	0.002	0.007	0.004	0.000	0.000	0.000	2.518	0.926	3.444	2.066	0.000	0.000	0	25.180	9.260	34.440	20.660	0.000	0.000
150	alpha	0.6	60x8 weeks	Ag80	M	VAC50	100	Unvaccinate	0.000	0.005	0.002	0.007	0.004	0.000	0.000	0.000	2.518	0.926	3.444	2.066	0.000	0.000	0	25.180	9.260	34.440	20.660	0.000	0.000
150	alpha	0.6	60x8 weeks	Ag80	M	VAC50	100	Unvaccinate	0.000	0.005	0.002	0.007	0.004	0.000	0.000	0.000	2.518	0.926	3.444	2.066	0.000	0.000	0	25.180	9.260	34.440	20.660	0.000	0.000
150	alpha	1	60x8 weeks	Ag80	M	VAC50	100	Unvaccinate	0.000	0.005	0.004	0.010	0.006	0.000	0.000	0.000	2.535	2.096	4.631	2.793	0.000	0.000	0	25.350	20.960	46.310	27.930	0.000	0.000
150	alpha	1	60x8 weeks	Ag80	M	VAC50	100	Unvaccinate	0.000	0.005	0.004	0.010	0.006	0.000	0.000	0.000	2.535	2.096	4.631	2.793	0.000	0.000	0	25.350	20.960	46.310	27.930	0.000	0.000
150	alpha	1	60x8 weeks	Ag80	M	VAC50	100	Unvaccinate	0.000	0.005	0.004	0.010	0.006	0.000	0.000	0.000	2.535	2.096	4.631	2.793	0.000	0.000	0	25.350	20.960	46.310	27.930	0.000	0.000
150	alpha	1.2	60x8 weeks	Ag80	M	VAC50	100	Unvaccinate	0.000	0.005	0.006	0.011	0.007	0.000	0.000	0.000	2.461	2.719	5.180	3.184	0.000	0.000	0	24.610	27.190	51.800	31.840	0.000	0.000
150	alpha	1.2	60x8 weeks	Ag80	M	VAC50	100	Unvaccinate	0.000	0.005	0.006	0.011	0.007	0.000	0.000	0.000	2.461	2.719	5.180	3.184	0.000	0.000	0	24.610	27.190	51.800	31.840	0.000	0.000
150	alpha	1.2	60x8 weeks	Ag80	M	VAC50	100	Unvaccinate	0.000	0.005	0.006	0.011	0.007	0.000	0.000	0.000	2.461	2.719	5.180	3.184	0.000	0.000	0	24.610	27.190	51.800	31.840	0.000	0.000
150	delta	0.6	60x8 weeks	Ag80	M	VAC50	100	Unvaccinate	0.000	0.007	0.014	0.021	0.013	0.000	0.000	0.000	3.348	6.899	10.247	6.462	0.000	0.000	0	33.480	68.990	102.470	64.620	0.000	0.000
150	delta	0.6	60x8 weeks	Ag80	M	VAC50	100	Unvaccinate	0.000	0.007	0.014	0.021	0.013	0.000	0.000	0.000	3.348	6.899	10.247	6.462	0.000	0.000	0	33.480	68.990	102.470	64.620	0.000	0.000
150	delta	0.6	60x8 weeks	Ag80	M	VAC50	100	Unvaccinate	0.000	0.007	0.014	0.021	0.013	0.000	0.000	0.000	3.348	6.899	10.247	6.462	0.000	0.000	0	33.480	68.990	102.470	64.620	0.000	0.000
150	delta	1	60x8 weeks	Ag80	M	VAC50	100	Unvaccinate	0.000	0.006	0.050	0.055	0.036	0.000	0.000	0.000	2.768	23.814	26.582	17.130	0.000	0.000	0	27.680	238.140	265.820	171.300	0.000	0.000
150	delta	1	60x8 weeks	Ag80	M	VAC50	100	Unvaccinate	0.000	0.006	0.050	0.055	0.036	0.000	0.000	0.000	2.768	23.814	26.582	17.130	0.000	0.000	0	27.680	238.140	265.820	171.300	0.000	0.000
150	delta	1	60x8 weeks	Ag80	M	VAC50	100	Unvaccinate	0.000	0.006	0.050	0.055	0.036	0.000	0.000	0.000	2.768	23.814	26.582	17.130	0.000	0.000	0	27.680	238.140	265.820	171.300	0.000	0.000
150	delta	1.2	60x8 weeks	Ag80	M	VAC50	100	Unvaccinate	0.000	0.005	0.070	0.075	0.049	0.000	0.000	0.000	2.445	33.534	35.979	23.613	0.000	0.000	0	24.450	335.340	359.790	236.130	0.000	0.000
150	delta	1.2	60x8 weeks	Ag80	M	VAC50	100	Unvaccinate	0.000	0.005	0.070	0.075	0.049	0.000	0.000	0.000	2.445	33.534	35.979	23.613	0.000	0.000	0	24.450	335.340	359.790	236.130	0.000	0.000
150	delta	1.2	60x8 weeks	Ag80	M	VAC50	100	Unvaccinate	0.000	0.005	0.070	0.075	0.049	0.000	0.000	0.000	2.445	33.534	35.979	23.613	0.000	0.000	0	24.450	335.340	359.790	236.130	0.000	0.000
150	original	0.6	60x8 weeks	NOTEST	NA	NOVAC	0	All	0.000	0.010	0.009	0.018	0.010	0.000	0.000	0.000	4.595	4.192	8.787	4.908	0.000	0.000	0	45.950	41.920	87.870	49.080	0.000	0.000
150	original	0.6	60x8 weeks	NOTEST	NA	NOVAC	0	All	0.000	0.010	0.009	0.018	0.010	0.000	0.000	0.000	4.595	4.192	8.787	4.908	0.000	0.000	0	45.950	41.920	87.870	49.080	0.000	0.000
150	original	0.6	60x8 weeks	NOTEST	NA	NOVAC	0	All	0.000	0.010	0.009	0.018	0.010	0.000	0.000	0.000	4.595	4.192	8.787	4.908	0.000	0.000	0	45.950	41.920	87.870	49.080	0.000	0.000
150	original	1	60x8 weeks	NOTEST	NA	NOVAC	0	All	0.000	0.009	0.023	0.032	0.017	0.000	0.000	0.000	4.414	10.965	15.379	8.390	0.000	0.000	0	44.140	109.650	153.790	83.900	0.000	0.000
150	original	1	60x8 weeks	NOTEST	NA	NOVAC	0	All	0.000	0.009	0.023	0.032	0.017	0.000	0.000	0.000	4.414	10.965	15.379	8.390	0.000	0.000	0	44.140	109.650	153.790	83.900	0.000	0.000
150	original	1	60x8 weeks	NOTEST	NA	NOVAC	0	All	0.000	0.009	0.023	0.032	0.017	0.000	0.000	0.000	4.414	10.965	15.379	8.390	0.000	0.000	0	44.140	109.650	153.790	83.900	0.000	0.000
150	original	1.2	60x8 weeks	NOTEST	NA	NOVAC	0	All	0.000	0.009	0.035	0.043	0.024	0.000	0.000	0.000	4.201	16.674	20.875	11.553	0.000	0.000	0	42.010	166.740	208.750	115.530	0.000	0.000
150	original	1.2	60x8 weeks	NOTEST	NA	NOVAC	0	All	0.000	0.009	0.035	0.043	0.024	0.000	0.000	0.000	4.201	16.674	20.875	11.553	0.000	0.000	0	42.010	166.740	208.750	115.530	0.000	0.000
150	original	1.2	60x8 weeks	NOTEST	NA	NOVAC	0	All	0.000	0.009	0.035	0.043	0.024	0.000	0.000	0.000	4.201	16.674	20.875	11.553	0.000	0.000	0	42.010	166.740	208.750	115.530	0.000	0.000
150	alpha	0.6	60x8 weeks	NOTEST	NA	NOVAC	0	All	0.000	0.009	0.040	0.049	0.031	0.000	0.000	0.000	4.142	19.271	23.413	15.046	0.000	0.000	0	41.420	192.710	234.130	150.460	0.000	0.000
150	alpha	0.6	60x8 weeks	NOTEST	NA	NOVAC	0	All	0.000	0.009	0.040	0.049	0.031	0.000	0.000	0.000	4.142	19.271	23.413	15.046	0.000	0.000	0	41.420	192.710	234.130	150.460	0.000	0.000
150	alpha	0.6	60x8 weeks	NOTEST	NA	NOVAC	0	All	0.000	0.009	0.040	0.049	0.031	0.000	0.000	0.000	4.142	19.271	23.413	15.046	0.000	0.000	0	41.420	192.710	234.130	150.460	0.000	0.000
150	alpha	1	60x8 weeks	NOTEST	NA	NOVAC	0	All	0.000	0.006	0.096	0.102	0.069	0.000	0.000	0.000	2.878	46.008	48.886	33.054	0.000	0.000	0	28.780	460.080	488.860	330.540	0.000	0.000
150	alpha	1	60x8 weeks	NOTEST	NA	NOVAC	0	All	0.000	0.006	0.096	0.102	0.069	0.000	0.000	0.000	2.878	46.008	48.886	33.054	0.000	0.000	0	28.780	460.080	488.860	330.540	0.000	0.000
150	alpha	1	60x8 weeks	NOTEST	NA	NOVAC	0	All	0.000	0.006	0.096	0.102	0.069	0.000	0.000	0.000	2.878	46.008	48.886	33.054	0.000	0.000	0	28.780	460.080	488.860	330.540	0.000	0.000
150	alpha	1.2	60x8 weeks	NOTEST	NA	NOVAC	0	All	0.000	0.005	0.108	0.114	0.078	0.000	0.000	0.000	2.507	52.043	54.550	37.283	0.000	0.000	0	25.070	520.430	545.500	372.830	0.000	0.000
150	alpha	1.2	60x8 weeks	NOTEST	NA	NOVAC	0	All	0.000	0.005	0.108	0.114	0.078	0.000	0.000	0.000	2.507	52.043	54.550	37.283	0.000	0.000	0	25.070	520.430	545.500	372.830	0.000	0.000
150	alpha	1.2	60x8 weeks	NOTEST	NA	NOVAC	0	All	0.000	0.005	0.108	0.114	0.078	0.000	0.000	0.000	2.507	52.043	54.550	37.283	0.000	0.000	0	25.070	520.430	545.500	372.830	0.000	0.000
150	delta	0.6	60x8 weeks	NOTEST	NA	NOVAC	0	All	0.000	0.005	0.111	0.116	0.079	0.000	0.000	0.000	2.355	53.132	55.487	37.989	0.000	0.000	0	23.550	531.320	554.870	379.890	0.000	0.000
150	delta	0.6	60x8 weeks	NOTEST	NA	NOVAC	0	All	0.000	0.005	0.111	0.116	0.079	0.000	0.000	0.000	2.355	53.132	55.487	37.989	0.000	0.000	0	23.550	531.320	554.870	379.890	0.000	0.000
150	delta	0.6	60x8 weeks	NOTEST	NA	NOVAC	0	All	0.000	0.005	0.111	0.116	0.079	0.000	0.000	0.000	2.355	53.132	55.487	37.989	0.000	0.000	0	23.550	531.320	554.870	379.890	0.000	0.000
150	delta	1	60x8 weeks	NOTEST	NA	NOVAC	0	All	0.000	0.004	0.120	0.124	0.086	0.000	0.000	0.000	1.779	57.764	59.543	41.342	0.000	0.000	0	17.790	577.640	595.430	413.420	0.000	0.000
150	delta	1	60x8 weeks	NOTEST	NA	NOVAC	0	All	0.000	0.004	0.120	0.124	0.086	0.000	0.000	0.000	1.779	57.764	59.543	41.342	0.000	0.000	0	17.790	577.640	595.430	413.420	0.000	0.000
150	delta	1	60x8 weeks	NOTEST	NA	NOVAC	0	All	0.000	0.004	0.120	0.124	0.086	0.000	0.000	0.000	1.779	57.764	59.543	41.342	0.000	0.000	0	17.790	577.640	595.430	413.420	0.000	0.000
150	delta	1.2	60x8 weeks	NOTEST	NA	NOVAC	0	All	0.000	0.003	0.121	0.125	0.087	0.000	0.000	0.000	1.650	58.175	59.825	41.660	0.000	0.000	0	16.500	581.750	598.250	416.600	0.000	0.000
150	delta	1.2	60x8 weeks	NOTEST	NA	NOVAC	0	All	0.000	0.003	0.121	0.125	0.087	0.000	0.000	0.000	1.650	58.175	59.825	41.660	0.000	0.000	0	16.500	581.750	598.250	416.600	0.000	0.000
150	delta	1.2	60x8 weeks	NOTEST	NA	NOVAC	0	All	0.000	0.003	0.121	0.125	0.087	0.000	0.000	0.000	1.650	58.175	59.825	41.660	0.000	0.000	0	16.500	581.750	598.250	416.600	0.000	0.000
150	original	0.6	60x8 weeks	NOTEST	NA	VAC95	25	All	0.000	0.007	0.004	0.012	0.006	0.000	0.000	0.000	3.598	2.043	5.641	3.071	0.000	0.000	0	35.980	20.430	56.410	30.710	0.000	0.000
150	original	0.6	60x8 weeks	NOTEST	NA	VAC95	25	All	0.000	0.007	0.004	0.012	0.006	0.000	0.000	0.000	3.598	2.043	5.641	3.071	0.000	0.000	0	35.980	20.430	56.410	30.710	0.000	0.000
150	original	0.6	60x8 weeks	NOTEST	NA	VAC95	25	All	0.000	0.007	0.004	0.012	0.006	0.000	0.000	0.000	3.598	2.043	5.641	3.071	0.000	0.000	0	35.980	20.430	56.410	30.710	0.000	0.000
150	original	1	60x8 weeks	NOTEST	NA	VAC95	25	All	0.000	0.007	0.009	0.016	0.009	0.000	0.000	0.000	3.483	4.400	7.883	4.293	0.000	0.000	0	34.830	44.000	78.830	42.930	0.000	0.000
150	original	1	60x8 weeks	NOTEST	NA	VAC95	25	All	0.000	0.007	0.009	0.016	0.009	0.000	0.000	0.000	3.483	4.400	7.883	4.293	0.000	0.000	0	34.830	44.000	78.830	42.930	0.000	0.000

150	original	1	60x8 weeks	NOTEST	NA	VAC95	25	All	0.000	0.007	0.009	0.016	0.009	0.000	0.000	0.000	3.483	4.400	7.883	4.293	0.000	0.000	0	34.830	44.000	78.830	42.930	0.000	0.000
150	original	1.2	60x8 weeks	NOTEST	NA	VAC95	25	All	0.000	0.007	0.014	0.021	0.012	0.000	0.000	0.000	3.441	6.781	10.222	5.532	0.000	0.000	0	34.410	67.810	102.220	55.320	0.000	0.000
150	original	1.2	60x8 weeks	NOTEST	NA	VAC95	25	All	0.000	0.007	0.014	0.021	0.012	0.000	0.000	0.000	3.441	6.781	10.222	5.532	0.000	0.000	0	34.410	67.810	102.220	55.320	0.000	0.000
150	original	1.2	60x8 weeks	NOTEST	NA	VAC95	25	All	0.000	0.007	0.014	0.021	0.012	0.000	0.000	0.000	3.441	6.781	10.222	5.532	0.000	0.000	0	34.410	67.810	102.220	55.320	0.000	0.000
150	alpha	0.6	60x8 weeks	NOTEST	NA	VAC95	25	All	0.000	0.007	0.016	0.023	0.014	0.000	0.000	0.000	3.502	7.475	10.977	6.876	0.000	0.000	0	35.020	74.750	109.770	68.760	0.000	0.000
150	alpha	0.6	60x8 weeks	NOTEST	NA	VAC95	25	All	0.000	0.007	0.016	0.023	0.014	0.000	0.000	0.000	3.502	7.475	10.977	6.876	0.000	0.000	0	35.020	74.750	109.770	68.760	0.000	0.000
150	alpha	0.6	60x8 weeks	NOTEST	NA	VAC95	25	All	0.000	0.007	0.016	0.023	0.014	0.000	0.000	0.000	3.502	7.475	10.977	6.876	0.000	0.000	0	35.020	74.750	109.770	68.760	0.000	0.000
150	alpha	1	60x8 weeks	NOTEST	NA	VAC95	25	All	0.000	0.006	0.046	0.052	0.034	0.000	0.000	0.000	2.918	22.043	24.961	16.111	0.000	0.000	0	29.180	220.430	249.610	161.110	0.000	0.000
150	alpha	1	60x8 weeks	NOTEST	NA	VAC95	25	All	0.000	0.006	0.046	0.052	0.034	0.000	0.000	0.000	2.918	22.043	24.961	16.111	0.000	0.000	0	29.180	220.430	249.610	161.110	0.000	0.000
150	alpha	1	60x8 weeks	NOTEST	NA	VAC95	25	All	0.000	0.006	0.046	0.052	0.034	0.000	0.000	0.000	2.918	22.043	24.961	16.111	0.000	0.000	0	29.180	220.430	249.610	161.110	0.000	0.000
150	alpha	1.2	60x8 weeks	NOTEST	NA	VAC95	25	All	0.000	0.005	0.061	0.066	0.043	0.000	0.000	0.000	2.542	29.294	31.836	20.559	0.000	0.000	0	25.420	292.940	318.360	205.590	0.000	0.000
150	alpha	1.2	60x8 weeks	NOTEST	NA	VAC95	25	All	0.000	0.005	0.061	0.066	0.043	0.000	0.000	0.000	2.542	29.294	31.836	20.559	0.000	0.000	0	25.420	292.940	318.360	205.590	0.000	0.000
150	alpha	1.2	60x8 weeks	NOTEST	NA	VAC95	25	All	0.000	0.005	0.061	0.066	0.043	0.000	0.000	0.000	2.542	29.294	31.836	20.559	0.000	0.000	0	25.420	292.940	318.360	205.590	0.000	0.000
150	delta	0.6	60x8 weeks	NOTEST	NA	VAC95	25	All	0.000	0.005	0.070	0.076	0.050	0.000	0.000	0.000	2.567	33.681	36.248	23.922	0.000	0.000	0	25.670	336.810	362.480	239.220	0.000	0.000
150	delta	0.6	60x8 weeks	NOTEST	NA	VAC95	25	All	0.000	0.005	0.070	0.076	0.050	0.000	0.000	0.000	2.567	33.681	36.248	23.922	0.000	0.000	0	25.670	336.810	362.480	239.220	0.000	0.000
150	delta	0.6	60x8 weeks	NOTEST	NA	VAC95	25	All	0.000	0.005	0.070	0.076	0.050	0.000	0.000	0.000	2.567	33.681	36.248	23.922	0.000	0.000	0	25.670	336.810	362.480	239.220	0.000	0.000
150	delta	1	60x8 weeks	NOTEST	NA	VAC95	25	All	0.000	0.004	0.098	0.101	0.068	0.000	0.000	0.000	1.865	46.811	48.676	32.661	0.000	0.000	0	18.650	468.110	486.760	326.610	0.000	0.000
150	delta	1	60x8 weeks	NOTEST	NA	VAC95	25	All	0.000	0.004	0.098	0.101	0.068	0.000	0.000	0.000	1.865	46.811	48.676	32.661	0.000	0.000	0	18.650	468.110	486.760	326.610	0.000	0.000
150	delta	1	60x8 weeks	NOTEST	NA	VAC95	25	All	0.000	0.004	0.098	0.101	0.068	0.000	0.000	0.000	1.865	46.811	48.676	32.661	0.000	0.000	0	18.650	468.110	486.760	326.610	0.000	0.000
150	delta	1.2	60x8 weeks	NOTEST	NA	VAC95	25	All	0.000	0.003	0.103	0.106	0.071	0.000	0.000	0.000	1.656	49.278	50.934	34.081	0.000	0.000	0	16.560	492.780	509.340	340.810	0.000	0.000
150	delta	1.2	60x8 weeks	NOTEST	NA	VAC95	25	All	0.000	0.003	0.103	0.106	0.071	0.000	0.000	0.000	1.656	49.278	50.934	34.081	0.000	0.000	0	16.560	492.780	509.340	340.810	0.000	0.000
150	delta	1.2	60x8 weeks	NOTEST	NA	VAC95	25	All	0.000	0.003	0.103	0.106	0.071	0.000	0.000	0.000	1.656	49.278	50.934	34.081	0.000	0.000	0	16.560	492.780	509.340	340.810	0.000	0.000
150	original	0.6	60x8 weeks	NOTEST	NA	VAC95	50	All	0.000	0.005	0.002	0.007	0.004	0.000	0.000	0.000	2.589	0.860	3.449	1.849	0.000	0.000	0	25.890	8.600	34.490	18.490	0.000	0.000
150	original	0.6	60x8 weeks	NOTEST	NA	VAC95	50	All	0.000	0.005	0.002	0.007	0.004	0.000	0.000	0.000	2.589	0.860	3.449	1.849	0.000	0.000	0	25.890	8.600	34.490	18.490	0.000	0.000
150	original	0.6	60x8 weeks	NOTEST	NA	VAC95	50	All	0.000	0.005	0.002	0.007	0.004	0.000	0.000	0.000	2.589	0.860	3.449	1.849	0.000	0.000	0	25.890	8.600	34.490	18.490	0.000	0.000
150	original	1	60x8 weeks	NOTEST	NA	VAC95	50	All	0.000	0.005	0.003	0.009	0.005	0.000	0.000	0.000	2.565	1.587	4.152	2.256	0.000	0.000	0	25.650	15.870	41.520	22.560	0.000	0.000
150	original	1	60x8 weeks	NOTEST	NA	VAC95	50	All	0.000	0.005	0.003	0.009	0.005	0.000	0.000	0.000	2.565	1.587	4.152	2.256	0.000	0.000	0	25.650	15.870	41.520	22.560	0.000	0.000
150	original	1	60x8 weeks	NOTEST	NA	VAC95	50	All	0.000	0.005	0.003	0.009	0.005	0.000	0.000	0.000	2.565	1.587	4.152	2.256	0.000	0.000	0	25.650	15.870	41.520	22.560	0.000	0.000
150	original	1.2	60x8 weeks	NOTEST	NA	VAC95	50	All	0.000	0.005	0.005	0.010	0.005	0.000	0.000	0.000	2.515	2.184	4.699	2.517	0.000	0.000	0	25.150	21.840	46.990	25.170	0.000	0.000
150	original	1.2	60x8 weeks	NOTEST	NA	VAC95	50	All	0.000	0.005	0.005	0.010	0.005	0.000	0.000	0.000	2.515	2.184	4.699	2.517	0.000	0.000	0	25.150	21.840	46.990	25.170	0.000	0.000
150	original	1.2	60x8 weeks	NOTEST	NA	VAC95	50	All	0.000	0.005	0.005	0.010	0.005	0.000	0.000	0.000	2.515	2.184	4.699	2.517	0.000	0.000	0	25.150	21.840	46.990	25.170	0.000	0.000
150	alpha	0.6	60x8 weeks	NOTEST	NA	VAC95	50	All	0.000	0.005	0.005	0.011	0.007	0.000	0.000	0.000	2.567	2.603	5.170	3.183	0.000	0.000	0	25.670	26.030	51.700	31.830	0.000	0.000
150	alpha	0.6	60x8 weeks	NOTEST	NA	VAC95	50	All	0.000	0.005	0.005	0.011	0.007	0.000	0.000	0.000	2.567	2.603	5.170	3.183	0.000	0.000	0	25.670	26.030	51.700	31.830	0.000	0.000
150	alpha	0.6	60x8 weeks	NOTEST	NA	VAC95	50	All	0.000	0.005	0.005	0.011	0.007	0.000	0.000	0.000	2.567	2.603	5.170	3.183	0.000	0.000	0	25.670	26.030	51.700	31.830	0.000	0.000
150	alpha	1	60x8 weeks	NOTEST	NA	VAC95	50	All	0.000	0.005	0.013	0.018	0.011	0.000	0.000	0.000	2.448	6.242	8.690	5.432	0.000	0.000	0	24.480	62.420	86.900	54.320	0.000	0.000
150	alpha	1	60x8 weeks	NOTEST	NA	VAC95	50	All	0.000	0.005	0.013	0.018	0.011	0.000	0.000	0.000	2.448	6.242	8.690	5.432	0.000	0.000	0	24.480	62.420	86.900	54.320	0.000	0.000
150	alpha	1	60x8 weeks	NOTEST	NA	VAC95	50	All	0.000	0.005	0.013	0.018	0.011	0.000	0.000	0.000	2.448	6.242	8.690	5.432	0.000	0.000	0	24.480	62.420	86.900	54.320	0.000	0.000
150	alpha	1.2	60x8 weeks	NOTEST	NA	VAC95	50	All	0.000	0.005	0.020	0.025	0.016	0.000	0.000	0.000	2.358	9.544	11.902	7.444	0.000	0.000	0	23.580	95.440	119.020	74.440	0.000	0.000
150	alpha	1.2	60x8 weeks	NOTEST	NA	VAC95	50	All	0.000	0.005	0.020	0.025	0.016	0.000	0.000	0.000	2.358	9.544	11.902	7.444	0.000	0.000	0	23.580	95.440	119.020	74.440	0.000	0.000
150	alpha	1.2	60x8 weeks	NOTEST	NA	VAC95	50	All	0.000	0.005	0.020	0.025	0.016	0.000	0.000	0.000	2.358	9.544	11.902	7.444	0.000	0.000	0	23.580	95.440	119.020	74.440	0.000	0.000
150	delta	0.6	60x8 weeks	NOTEST	NA	VAC95	50	All	0.000	0.005	0.024	0.029	0.019	0.000	0.000	0.000	2.398	11.697	14.095	8.929	0.000	0.000	0	23.980	116.970	140.950	89.290	0.000	0.000
150	delta	0.6	60x8 weeks	NOTEST	NA	VAC95	50	All	0.000	0.005	0.024	0.029	0.019	0.000	0.000	0.000	2.398	11.697	14.095	8.929	0.000	0.000	0	23.980	116.970	140.950	89.290	0.000	0.000
150	delta	0.6	60x8 weeks	NOTEST	NA	VAC95	50	All	0.000	0.005	0.024	0.029	0.019	0.000	0.000	0.000	2.398	11.697	14.095	8.929	0.000	0.000	0	23.980	116.970	140.950	89.290	0.000	0.000
150	delta	1	60x8 weeks	NOTEST	NA	VAC95	50	All	0.000	0.004	0.058	0.062	0.040	0.000	0.000	0.000	1.828	28.022	29.850	19.268	0.000	0.000	0	18.280	280.220	298.500	192.680	0.000	0.000
150	delta	1	60x8 weeks	NOTEST	NA	VAC95	50	All	0.000	0.004	0.058	0.062	0.040	0.000	0.000	0.000	1.828	28.022	29.850	19.268	0.000	0.000	0	18.280	280.220	298.500	192.680	0.000	0.000
150	delta	1	60x8 weeks	NOTEST	NA	VAC95	50	All	0.000	0.004	0.058	0.062	0.040	0.000	0.000	0.000	1.828	28.022	29.850	19.268	0.000	0.000	0	18.280	280.220	298.500	192.680	0.000	0.000
150	delta	1.2	60x8 weeks	NOTEST	NA	VAC95	50	All	0.000	0.004	0.067	0.071	0.046	0.000	0.000	0.000	1.705	32.378	34.083	21.843	0.000	0.000	0	17.050	323.780	340.830	218.430	0.000	0.000
150	delta	1.2	60x8 weeks	NOTEST	NA	VAC95	50	All	0.000	0.004	0.067	0.071	0.046	0.000	0.000	0.000	1.705	32.378	34.083	21.843	0.000	0.000	0	17.050	323.780	340.830	218.430	0.000	0.000
150	delta	1.2	60x8 weeks	NOTEST	NA	VAC95	50	All	0.000	0.004	0.067	0.071	0.046	0.000	0.000	0.000	1.705	32.378	34.083	21.843	0.000	0.000	0	17.050	323.780	340.830	218.430	0.000	0.000
150	original	0.6	60x8 weeks	NOTEST	NA	VAC95	75	All	0.000	0.003	0.000	0.003	0.002	0.000	0.000	0.000	1.455	0.190	1.645	0.833	0.000	0.000	0	14.550	1.900	16.450	8.330	0.000	0.000
150	original	0.6	60x8 weeks	NOTEST	NA	VAC95	75	All	0.000	0.003	0.000	0.003	0.002	0.000	0.000	0.000	1.455	0.190	1.645	0.833	0.000	0.000	0	14.550	1.900	16.450	8.330	0.000	0.000
150	original	0.6	60x8 weeks	NOTEST	NA	VAC95	75	All	0.000	0.003	0.000	0.003	0.002	0.000	0.000	0.000	1.455	0.190	1.645	0.833	0.000	0.000	0	14.550	1.900	16.450	8.330	0.000	0.000
150	original	1	60x8 weeks	NOTEST	NA	VAC95	75	All	0.000	0.003	0.001	0.004	0.002	0.000	0.000	0.000	1.442	0.377	1.819	0.901	0.000	0.000	0	14.420	3.770	18.190	9.010	0.000	0.000
150	original	1	60x8 weeks	NOTEST	NA	VAC95	75	All	0.000	0.003	0.001	0.004	0.002	0.000	0.000	0.000	1.442	0.377	1.819	0.901	0.000	0.000	0	14.420	3.770	18.190	9.010	0.000	0.000
150	original	1	60x8 weeks	NOTEST	NA	VAC95	75	All	0.000	0.003	0.001	0.004	0.002	0.000	0.000	0.000	1.442	0.377	1.819	0.901	0.000	0.000	0	14.420	3.770	18.190	9.010	0.000	0.000
150	original	1.2	60x8 weeks	NOTEST	NA	VAC95	75	All	0.000	0.003	0.001	0.004	0.002	0.000	0.000	0.000	1.464	0.469	1.933	1.020	0.000	0.000	0	14.640	4.690	19.330	10.200	0.000	0.000
150	original	1.2	60x8 weeks	NOTEST	NA	VAC95	75	All	0.000	0.003	0.001	0.004	0.002	0.000	0.000	0.000	1.464	0.469	1.933	1.020	0.000	0.000	0	14.640	4.690	19.330	10.200	0.000	0.000
150	original	1.2	60x8 weeks	NOTEST	NA	VAC95	75	All	0.000	0.003	0.001	0.004	0.002	0.000	0.000	0.000	1.464	0.469	1.933	1.020	0.000	0.000	0	14.640	4.690	19.330	10.200	0.000	0.000
150	alpha	0.6	60x8 weeks	NOTEST	NA	VAC95	75	All	0.000	0.003	0.001	0.005	0.003	0.000	0.000	0.000	1.590	0.600	2.190	1.307	0.000	0.000	0	15.900	6.000	21.900	13.070	0.000	0.000
150	alpha	0.6	60x8 weeks	NOTEST	NA	VAC95	75	All	0.000	0.003	0.001	0.005	0.003	0.000	0.000	0.000	1.590	0.600	2.190	1.307	0.000	0.000	0	15.900	6.000	21.900	13.070	0.000	0.000
150	alpha	0.6	60x8 weeks	NOTEST	NA	VAC95	75	All	0.000	0.003	0.001	0.005	0.003	0.000	0.000	0.000	1.590	0.600	2.190	1.307	0.000	0.000	0	15.900	6.000	21.900	13.070	0.000	0.000
150	alpha	1	60x8 weeks	NOTEST	NA	VAC95	75	All	0.000	0.003	0.003	0.006	0.003	0.000	0.000	0.000	1.530	1.221	2.751	1.666	0.000	0.000	0	15.300	12.210	27.510	16.660	0.000	0.000
150	alpha	1	60x8 weeks	NOTEST	NA	VAC95	75	All	0.000	0.003	0.003	0.006	0.003	0.000	0.000	0.000	1.530	1.221	2.751	1.666	0.000	0.000	0	15.300	12.210	27.510	16.660	0.000	0.000
150	alpha	1	60x8 weeks	NOTEST	NA	VAC95	75	All	0.000	0.003	0.003	0.006	0.003	0.000	0.000	0.000	1.530	1.221	2.751	1.666	0.000	0.000	0	15.300	12.210	27.510	16.660	0.000	0.000
150	alpha	1.2	60x8 weeks	NOTEST	NA	VAC95	75	All	0.000	0.003	0.003	0.006	0.004	0.000	0.000	0.000	1.479	1.581	3.060	1.852	0.000	0.000	0	14.790	15.810	30.600	18.520	0.000	0.000
150	alpha	1.2	60x8 weeks	NOTEST	NA	VAC95	75	All	0.000	0.003	0.003	0.006	0.004	0.000	0.000	0.000	1.479	1.581	3.060	1.852	0.000	0.000	0	14.790	15.810	30.600	18.520	0.000	0.000
150	alpha	1.2	60x8 weeks	NOTEST	NA	VAC95	75	All	0.000	0.003	0.003	0.006	0.004	0.000	0.000	0.000	1.479	1.581	3.060	1.852	0.000	0.000	0	14.790	15.810	30.600	18.520	0.000	0.000
150	delta	0.6	60x8 weeks	NOTEST	NA	VAC95	75	All	0.000	0.004	0.005	0.008	0.005	0.000	0.000	0.000	1.769	2.267	4.036	2.456	0.000	0.000	0	17.690	22.670	40.360	24.560	0.000	0.000
150	delta	0.6	60x8 weeks	NOTEST	NA	VAC95	75	All	0.000	0.004	0.005	0.008	0.005	0.000	0.000	0.000	1.769	2.267	4.036	2.456	0.000	0.000	0	17.690	22.670	40.360	24.560	0.000	0.000
150	delta	0.6	60x8 weeks	NOTEST	NA	VAC95	75	All	0.000	0.004	0.005	0.008	0.005	0.000	0.000	0.000	1.769	2.267	4.036	2.456	0.000	0.000	0	17.690	22.670	40.360	24.560	0.000	0.000
150	delta	1	60x8 weeks	NOTEST	NA	VAC95	75	All	0.000	0.003	0.013	0.016	0.010	0.000	0.000	0.000	1.611	6.128	7.739	4.673	0.000	0.000	0	16.110	61.280	77.390	46.730	0.000	0.000

150	delta	1	60x8 weeks	NOTEST	NA	VAC95	75	All	0.000	0.003	0.013	0.016	0.010	0.000	0.000	0.000	1.611	6.128	7.739	4.673	0.000	0.000	0	16.110	61.280	77.390	46.730	0.000	0.000
150	delta	1	60x8 weeks	NOTEST	NA	VAC95	75	All	0.000	0.003	0.013	0.016	0.010	0.000	0.000	0.000	1.611	6.128	7.739	4.673	0.000	0.000	0	16.110	61.280	77.390	46.730	0.000	0.000
150	delta	1.2	60x8 weeks	NOTEST	NA	VAC95	75	All	0.000	0.003	0.019	0.022	0.013	0.000	0.000	0.000	1.610	9.052	10.662	6.391	0.000	0.000	0	16.100	90.520	106.620	63.910	0.000	0.000
150	delta	1.2	60x8 weeks	NOTEST	NA	VAC95	75	All	0.000	0.003	0.019	0.022	0.013	0.000	0.000	0.000	1.610	9.052	10.662	6.391	0.000	0.000	0	16.100	90.520	106.620	63.910	0.000	0.000
150	delta	1.2	60x8 weeks	NOTEST	NA	VAC95	75	All	0.000	0.003	0.019	0.022	0.013	0.000	0.000	0.000	1.610	9.052	10.662	6.391	0.000	0.000	0	16.100	90.520	106.620	63.910	0.000	0.000
150	original	0.6	60x8 weeks	NOTEST	NA	VAC95	100	All	0.000	0.001	0.000	0.001	0.000	0.000	0.000	0.000	0.365	0.000	0.365	0.141	0.000	0.000	0	3.650	0.000	3.650	1.410	0.000	0.000
150	original	0.6	60x8 weeks	NOTEST	NA	VAC95	100	All	0.000	0.001	0.000	0.001	0.000	0.000	0.000	0.000	0.365	0.000	0.365	0.141	0.000	0.000	0	3.650	0.000	3.650	1.410	0.000	0.000
150	original	0.6	60x8 weeks	NOTEST	NA	VAC95	100	All	0.000	0.001	0.000	0.001	0.000	0.000	0.000	0.000	0.365	0.000	0.365	0.141	0.000	0.000	0	3.650	0.000	3.650	1.410	0.000	0.000
150	original	1	60x8 weeks	NOTEST	NA	VAC95	100	All	0.000	0.001	0.000	0.001	0.000	0.000	0.000	0.000	0.377	0.000	0.377	0.147	0.000	0.000	0	3.770	0.000	3.770	1.470	0.000	0.000
150	original	1	60x8 weeks	NOTEST	NA	VAC95	100	All	0.000	0.001	0.000	0.001	0.000	0.000	0.000	0.000	0.377	0.000	0.377	0.147	0.000	0.000	0	3.770	0.000	3.770	1.470	0.000	0.000
150	original	1	60x8 weeks	NOTEST	NA	VAC95	100	All	0.000	0.001	0.000	0.001	0.000	0.000	0.000	0.000	0.377	0.000	0.377	0.147	0.000	0.000	0	3.770	0.000	3.770	1.470	0.000	0.000
150	original	1.2	60x8 weeks	NOTEST	NA	VAC95	100	All	0.000	0.001	0.000	0.001	0.000	0.000	0.000	0.000	0.357	0.001	0.358	0.127	0.000	0.000	0	3.570	0.010	3.580	1.270	0.000	0.000
150	original	1.2	60x8 weeks	NOTEST	NA	VAC95	100	All	0.000	0.001	0.000	0.001	0.000	0.000	0.000	0.000	0.357	0.001	0.358	0.127	0.000	0.000	0	3.570	0.010	3.580	1.270	0.000	0.000
150	original	1.2	60x8 weeks	NOTEST	NA	VAC95	100	All	0.000	0.001	0.000	0.001	0.000	0.000	0.000	0.000	0.357	0.001	0.358	0.127	0.000	0.000	0	3.570	0.010	3.580	1.270	0.000	0.000
150	alpha	0.6	60x8 weeks	NOTEST	NA	VAC95	100	All	0.000	0.001	0.000	0.001	0.001	0.000	0.000	0.000	0.469	0.034	0.503	0.252	0.000	0.000	0	4.690	0.340	5.030	2.520	0.000	0.000
150	alpha	0.6	60x8 weeks	NOTEST	NA	VAC95	100	All	0.000	0.001	0.000	0.001	0.001	0.000	0.000	0.000	0.469	0.034	0.503	0.252	0.000	0.000	0	4.690	0.340	5.030	2.520	0.000	0.000
150	alpha	0.6	60x8 weeks	NOTEST	NA	VAC95	100	All	0.000	0.001	0.000	0.001	0.001	0.000	0.000	0.000	0.469	0.034	0.503	0.252	0.000	0.000	0	4.690	0.340	5.030	2.520	0.000	0.000
150	alpha	1	60x8 weeks	NOTEST	NA	VAC95	100	All	0.000	0.001	0.000	0.001	0.000	0.000	0.000	0.000	0.441	0.037	0.478	0.239	0.000	0.000	0	4.410	0.370	4.780	2.390	0.000	0.000
150	alpha	1	60x8 weeks	NOTEST	NA	VAC95	100	All	0.000	0.001	0.000	0.001	0.000	0.000	0.000	0.000	0.441	0.037	0.478	0.239	0.000	0.000	0	4.410	0.370	4.780	2.390	0.000	0.000
150	alpha	1	60x8 weeks	NOTEST	NA	VAC95	100	All	0.000	0.001	0.000	0.001	0.000	0.000	0.000	0.000	0.441	0.037	0.478	0.239	0.000	0.000	0	4.410	0.370	4.780	2.390	0.000	0.000
150	alpha	1.2	60x8 weeks	NOTEST	NA	VAC95	100	All	0.000	0.001	0.000	0.001	0.000	0.000	0.000	0.000	0.463	0.043	0.506	0.236	0.000	0.000	0	4.630	0.430	5.060	2.360	0.000	0.000
150	alpha	1.2	60x8 weeks	NOTEST	NA	VAC95	100	All	0.000	0.001	0.000	0.001	0.000	0.000	0.000	0.000	0.463	0.043	0.506	0.236	0.000	0.000	0	4.630	0.430	5.060	2.360	0.000	0.000
150	alpha	1.2	60x8 weeks	NOTEST	NA	VAC95	100	All	0.000	0.001	0.000	0.001	0.000	0.000	0.000	0.000	0.463	0.043	0.506	0.236	0.000	0.000	0	4.630	0.430	5.060	2.360	0.000	0.000
150	delta	0.6	60x8 weeks	NOTEST	NA	VAC95	100	All	0.000	0.001	0.000	0.002	0.001	0.000	0.000	0.000	0.702	0.138	0.840	0.428	0.000	0.000	0	7.020	1.380	8.400	4.280	0.000	0.000
150	delta	0.6	60x8 weeks	NOTEST	NA	VAC95	100	All	0.000	0.001	0.000	0.002	0.001	0.000	0.000	0.000	0.702	0.138	0.840	0.428	0.000	0.000	0	7.020	1.380	8.400	4.280	0.000	0.000
150	delta	0.6	60x8 weeks	NOTEST	NA	VAC95	100	All	0.000	0.001	0.000	0.002	0.001	0.000	0.000	0.000	0.702	0.138	0.840	0.428	0.000	0.000	0	7.020	1.380	8.400	4.280	0.000	0.000
150	delta	1	60x8 weeks	NOTEST	NA	VAC95	100	All	0.000	0.001	0.001	0.002	0.001	0.000	0.000	0.000	0.712	0.275	0.987	0.550	0.000	0.000	0	7.120	2.750	9.870	5.500	0.000	0.000
150	delta	1	60x8 weeks	NOTEST	NA	VAC95	100	All	0.000	0.001	0.001	0.002	0.001	0.000	0.000	0.000	0.712	0.275	0.987	0.550	0.000	0.000	0	7.120	2.750	9.870	5.500	0.000	0.000
150	delta	1	60x8 weeks	NOTEST	NA	VAC95	100	All	0.000	0.001	0.001	0.002	0.001	0.000	0.000	0.000	0.712	0.275	0.987	0.550	0.000	0.000	0	7.120	2.750	9.870	5.500	0.000	0.000
150	delta	1.2	60x8 weeks	NOTEST	NA	VAC95	100	All	0.000	0.001	0.001	0.002	0.001	0.000	0.000	0.000	0.717	0.362	1.079	0.587	0.000	0.000	0	7.170	3.620	10.790	5.870	0.000	0.000
150	delta	1.2	60x8 weeks	NOTEST	NA	VAC95	100	All	0.000	0.001	0.001	0.002	0.001	0.000	0.000	0.000	0.717	0.362	1.079	0.587	0.000	0.000	0	7.170	3.620	10.790	5.870	0.000	0.000
150	delta	1.2	60x8 weeks	NOTEST	NA	VAC95	100	All	0.000	0.001	0.001	0.002	0.001	0.000	0.000	0.000	0.717	0.362	1.079	0.587	0.000	0.000	0	7.170	3.620	10.790	5.870	0.000	0.000
150	original	0.6	60x8 weeks	NOTEST	NA	VAC70	25	All	0.000	0.008	0.005	0.013	0.007	0.000	0.000	0.000	3.914	2.172	6.086	3.268	0.000	0.000	0	39.140	21.720	60.860	32.680	0.000	0.000
150	original	0.6	60x8 weeks	NOTEST	NA	VAC70	25	All	0.000	0.008	0.005	0.013	0.007	0.000	0.000	0.000	3.914	2.172	6.086	3.268	0.000	0.000	0	39.140	21.720	60.860	32.680	0.000	0.000
150	original	0.6	60x8 weeks	NOTEST	NA	VAC70	25	All	0.000	0.008	0.005	0.013	0.007	0.000	0.000	0.000	3.914	2.172	6.086	3.268	0.000	0.000	0	39.140	21.720	60.860	32.680	0.000	0.000
150	original	1	60x8 weeks	NOTEST	NA	VAC70	25	All	0.000	0.008	0.011	0.019	0.010	0.000	0.000	0.000	3.836	5.053	8.889	4.814	0.000	0.000	0	38.360	50.530	88.890	48.140	0.000	0.000
150	original	1	60x8 weeks	NOTEST	NA	VAC70	25	All	0.000	0.008	0.011	0.019	0.010	0.000	0.000	0.000	3.836	5.053	8.889	4.814	0.000	0.000	0	38.360	50.530	88.890	48.140	0.000	0.000
150	original	1	60x8 weeks	NOTEST	NA	VAC70	25	All	0.000	0.008	0.011	0.019	0.010	0.000	0.000	0.000	3.836	5.053	8.889	4.814	0.000	0.000	0	38.360	50.530	88.890	48.140	0.000	0.000
150	original	1.2	60x8 weeks	NOTEST	NA	VAC70	25	All	0.000	0.008	0.015	0.023	0.013	0.000	0.000	0.000	3.787	7.402	11.189	6.094	0.000	0.000	0	37.870	74.020	111.890	60.940	0.000	0.000
150	original	1.2	60x8 weeks	NOTEST	NA	VAC70	25	All	0.000	0.008	0.015	0.023	0.013	0.000	0.000	0.000	3.787	7.402	11.189	6.094	0.000	0.000	0	37.870	74.020	111.890	60.940	0.000	0.000
150	original	1.2	60x8 weeks	NOTEST	NA	VAC70	25	All	0.000	0.008	0.015	0.023	0.013	0.000	0.000	0.000	3.787	7.402	11.189	6.094	0.000	0.000	0	37.870	74.020	111.890	60.940	0.000	0.000
150	alpha	0.6	60x8 weeks	NOTEST	NA	VAC70	25	All	0.000	0.008	0.018	0.026	0.016	0.000	0.000	0.000	3.792	8.755	12.547	7.915	0.000	0.000	0	37.920	87.550	125.470	79.150	0.000	0.000
150	alpha	0.6	60x8 weeks	NOTEST	NA	VAC70	25	All	0.000	0.008	0.018	0.026	0.016	0.000	0.000	0.000	3.792	8.755	12.547	7.915	0.000	0.000	0	37.920	87.550	125.470	79.150	0.000	0.000
150	alpha	0.6	60x8 weeks	NOTEST	NA	VAC70	25	All	0.000	0.008	0.018	0.026	0.016	0.000	0.000	0.000	3.792	8.755	12.547	7.915	0.000	0.000	0	37.920	87.550	125.470	79.150	0.000	0.000
150	alpha	1	60x8 weeks	NOTEST	NA	VAC70	25	All	0.000	0.006	0.057	0.063	0.041	0.000	0.000	0.000	3.104	27.317	30.421	19.638	0.000	0.000	0	31.040	273.170	304.210	196.380	0.000	0.000
150	alpha	1	60x8 weeks	NOTEST	NA	VAC70	25	All	0.000	0.006	0.057	0.063	0.041	0.000	0.000	0.000	3.104	27.317	30.421	19.638	0.000	0.000	0	31.040	273.170	304.210	196.380	0.000	0.000
150	alpha	1	60x8 weeks	NOTEST	NA	VAC70	25	All	0.000	0.006	0.057	0.063	0.041	0.000	0.000	0.000	3.104	27.317	30.421	19.638	0.000	0.000	0	31.040	273.170	304.210	196.380	0.000	0.000
150	alpha	1.2	60x8 weeks	NOTEST	NA	VAC70	25	All	0.000	0.006	0.074	0.080	0.052	0.000	0.000	0.000	2.785	35.568	38.353	25.064	0.000	0.000	0	27.850	355.680	383.530	250.640	0.000	0.000
150	alpha	1.2	60x8 weeks	NOTEST	NA	VAC70	25	All	0.000	0.006	0.074	0.080	0.052	0.000	0.000	0.000	2.785	35.568	38.353	25.064	0.000	0.000	0	27.850	355.680	383.530	250.640	0.000	0.000
150	alpha	1.2	60x8 weeks	NOTEST	NA	VAC70	25	All	0.000	0.006	0.074	0.080	0.052	0.000	0.000	0.000	2.785	35.568	38.353	25.064	0.000	0.000	0	27.850	355.680	383.530	250.640	0.000	0.000
150	delta	0.6	60x8 weeks	NOTEST	NA	VAC70	25	All	0.000	0.005	0.087	0.092	0.061	0.000	0.000	0.000	2.591	41.595	44.186	29.421	0.000	0.000	0	25.910	415.950	441.860	294.210	0.000	0.000
150	delta	0.6	60x8 weeks	NOTEST	NA	VAC70	25	All	0.000	0.005	0.087	0.092	0.061	0.000	0.000	0.000	2.591	41.595	44.186	29.421	0.000	0.000	0	25.910	415.950	441.860	294.210	0.000	0.000
150	delta	0.6	60x8 weeks	NOTEST	NA	VAC70	25	All	0.000	0.005	0.087	0.092	0.061	0.000	0.000	0.000	2.591	41.595	44.186	29.421	0.000	0.000	0	25.910	415.950	441.860	294.210	0.000	0.000
150	delta	1	60x8 weeks	NOTEST	NA	VAC70	25	All	0.000	0.004	0.110	0.114	0.078	0.000	0.000	0.000	1.858	52.808	54.666	37.370	0.000	0.000	0	18.580	528.080	546.660	373.700	0.000	0.000
150	delta	1	60x8 weeks	NOTEST	NA	VAC70	25	All	0.000	0.004	0.110	0.114	0.078	0.000	0.000	0.000	1.858	52.808	54.666	37.370	0.000	0.000	0	18.580	528.080	546.660	373.700	0.000	0.000
150	delta	1	60x8 weeks	NOTEST	NA	VAC70	25	All	0.000	0.004	0.110	0.114	0.078	0.000	0.000	0.000	1.858	52.808	54.666	37.370	0.000	0.000	0	18.580	528.080	546.660	373.700	0.000	0.000
150	delta	1.2	60x8 weeks	NOTEST	NA	VAC70	25	All	0.000	0.004	0.115	0.119	0.081	0.000	0.000	0.000	1.739	55.313	57.052	38.813	0.000	0.000	0	17.390	553.130	570.520	388.130	0.000	0.000
150	delta	1.2	60x8 weeks	NOTEST	NA	VAC70	25	All	0.000	0.004	0.115	0.119	0.081	0.000	0.000	0.000	1.739	55.313	57.052	38.813	0.000	0.000	0	17.390	553.130	570.520	388.130	0.000	0.000
150	delta	1.2	60x8 weeks	NOTEST	NA	VAC70	25	All	0.000	0.004	0.115	0.119	0.081	0.000	0.000	0.000	1.739	55.313	57.052	38.813	0.000	0.000	0	17.390	553.130	570.520	388.130	0.000	0.000
150	original	0.6	60x8 weeks	NOTEST	NA	VAC70	50	All	0.000	0.007	0.002	0.009	0.005	0.000	0.000	0.000	3.188	0.970	4.158	2.180	0.000	0.000	0	31.880	9.700	41.580	21.800	0.000	0.000
150	original	0.6	60x8 weeks	NOTEST	NA	VAC70	50	All	0.000	0.007	0.002	0.009	0.005	0.000	0.000	0.000	3.188	0.970	4.158	2.180	0.000	0.000	0	31.880	9.700	41.580	21.800	0.000	0.000
150	original	0.6	60x8 weeks	NOTEST	NA	VAC70	50	All	0.000	0.007	0.002	0.009	0.005	0.000	0.000	0.000	3.188	0.970	4.158	2.180	0.000	0.000	0	31.880	9.700	41.580	21.800	0.000	0.000
150	original	1	60x8 weeks	NOTEST	NA	VAC70	50	All	0.000	0.007	0.004	0.011	0.006	0.000	0.000	0.000	3.146	2.028	5.174	2.799	0.000	0.000	0	31.460	20.280	51.740	27.990	0.000	0.000
150	original	1	60x8 weeks	NOTEST	NA	VAC70	50	All	0.000	0.007	0.004	0.011	0.006	0.000	0.000	0.000	3.146	2.028	5.174	2.799	0.000	0.000	0	31.460	20.280	51.740	27.990	0.000	0.000
150	original	1	60x8 weeks	NOTEST	NA	VAC70	50	All	0.000	0.007	0.004	0.011	0.006	0.000	0.000	0.000	3.146	2.028	5.174	2.799	0.000	0.000	0	31.460	20.280	51.740	27.990	0.000	0.000
150	original	1.2	60x8 weeks	NOTEST	NA	VAC70	50	All	0.000	0.007	0.005	0.012	0.006	0.000	0.000	0.000	3.121	2.588	5.709	3.056	0.000	0.000	0	31.210	25.880	57.090	30.560	0.000	0.000
150	original	1.2	60x8 weeks	NOTEST	NA	VAC70	50	All	0.000	0.007	0.005	0.012	0.006	0.000	0.000	0.000	3.121	2.588	5.709	3.056	0.000	0.000	0	31.210	25.880	57.090	30.560	0.000	0.000
150	original	1.2	60x8 weeks	NOTEST	NA	VAC70	50	All	0.000	0.007	0.005	0.012	0.006	0.000	0.000	0.000	3.121	2.588	5.709	3.056	0.000	0.000	0	31.210	25.880	57.090	30.560	0.000	0.000
150	alpha	0.6	60x8 weeks	NOTEST	NA	VAC70	50	All	0.000	0.006	0.008	0.014	0.009	0.000	0.000	0.000	3.045	3.870	6.915	4.298	0.000	0.000	0	30.450	38.700	69.150	42.980	0.000	0.000
150	alpha	0.6	60x8 weeks	NOTEST	NA	VAC70	50	All	0.000	0.006	0.008	0.014	0.009	0.000	0.000	0.000	3.045	3.870	6.915	4.298	0.000	0.000	0	30.450	38.700	69.150	42.980	0.000	0.000
150	alpha	0.6	60x8 weeks	NOTEST	NA	VAC70	50	All	0.000	0.006	0.008	0.014	0.009	0.000	0.000	0.000	3.045	3.870	6.915	4.298	0.000	0.000	0	30.450	38.700	69.150	42.980	0.000	0.000

150	alpha	1	60x8 weeks	NOTEST	NA	VAC70	50	All	0.000	0.006	0.023	0.029	0.018	0.000	0.000	0.000	2.894	10.878	13.772	8.575	0.000	0.000	0	28.940	108.780	137.720	85.750	0.000	0.000
150	alpha	1	60x8 weeks	NOTEST	NA	VAC70	50	All	0.000	0.006	0.023	0.029	0.018	0.000	0.000	0.000	2.894	10.878	13.772	8.575	0.000	0.000	0	28.940	108.780	137.720	85.750	0.000	0.000
150	alpha	1	60x8 weeks	NOTEST	NA	VAC70	50	All	0.000	0.006	0.023	0.029	0.018	0.000	0.000	0.000	2.894	10.878	13.772	8.575	0.000	0.000	0	28.940	108.780	137.720	85.750	0.000	0.000
150	alpha	1.2	60x8 weeks	NOTEST	NA	VAC70	50	All	0.000	0.006	0.035	0.040	0.025	0.000	0.000	0.000	2.727	16.704	19.431	12.236	0.000	0.000	0	27.270	167.040	194.310	122.360	0.000	0.000
150	alpha	1.2	60x8 weeks	NOTEST	NA	VAC70	50	All	0.000	0.006	0.035	0.040	0.025	0.000	0.000	0.000	2.727	16.704	19.431	12.236	0.000	0.000	0	27.270	167.040	194.310	122.360	0.000	0.000
150	alpha	1.2	60x8 weeks	NOTEST	NA	VAC70	50	All	0.000	0.006	0.035	0.040	0.025	0.000	0.000	0.000	2.727	16.704	19.431	12.236	0.000	0.000	0	27.270	167.040	194.310	122.360	0.000	0.000
150	delta	0.6	60x8 weeks	NOTEST	NA	VAC70	50	All	0.000	0.006	0.048	0.053	0.035	0.000	0.000	0.000	2.689	22.926	25.615	16.771	0.000	0.000	0	26.890	229.260	256.150	167.710	0.000	0.000
150	delta	0.6	60x8 weeks	NOTEST	NA	VAC70	50	All	0.000	0.006	0.048	0.053	0.035	0.000	0.000	0.000	2.689	22.926	25.615	16.771	0.000	0.000	0	26.890	229.260	256.150	167.710	0.000	0.000
150	delta	0.6	60x8 weeks	NOTEST	NA	VAC70	50	All	0.000	0.006	0.048	0.053	0.035	0.000	0.000	0.000	2.689	22.926	25.615	16.771	0.000	0.000	0	26.890	229.260	256.150	167.710	0.000	0.000
150	delta	1	60x8 weeks	NOTEST	NA	VAC70	50	All	0.000	0.004	0.092	0.096	0.064	0.000	0.000	0.000	2.010	44.089	46.099	30.638	0.000	0.000	0	20.100	440.890	460.990	306.380	0.000	0.000
150	delta	1	60x8 weeks	NOTEST	NA	VAC70	50	All	0.000	0.004	0.092	0.096	0.064	0.000	0.000	0.000	2.010	44.089	46.099	30.638	0.000	0.000	0	20.100	440.890	460.990	306.380	0.000	0.000
150	delta	1	60x8 weeks	NOTEST	NA	VAC70	50	All	0.000	0.004	0.092	0.096	0.064	0.000	0.000	0.000	2.010	44.089	46.099	30.638	0.000	0.000	0	20.100	440.890	460.990	306.380	0.000	0.000
150	delta	1.2	60x8 weeks	NOTEST	NA	VAC70	50	All	0.000	0.004	0.102	0.106	0.071	0.000	0.000	0.000	1.847	49.158	51.005	34.232	0.000	0.000	0	18.470	491.580	510.050	342.320	0.000	0.000
150	delta	1.2	60x8 weeks	NOTEST	NA	VAC70	50	All	0.000	0.004	0.102	0.106	0.071	0.000	0.000	0.000	1.847	49.158	51.005	34.232	0.000	0.000	0	18.470	491.580	510.050	342.320	0.000	0.000
150	delta	1.2	60x8 weeks	NOTEST	NA	VAC70	50	All	0.000	0.004	0.102	0.106	0.071	0.000	0.000	0.000	1.847	49.158	51.005	34.232	0.000	0.000	0	18.470	491.580	510.050	342.320	0.000	0.000
150	original	0.6	60x8 weeks	NOTEST	NA	VAC70	75	All	0.000	0.005	0.001	0.006	0.003	0.000	0.000	0.000	2.543	0.345	2.888	1.507	0.000	0.000	0	25.430	3.450	28.880	15.070	0.000	0.000
150	original	0.6	60x8 weeks	NOTEST	NA	VAC70	75	All	0.000	0.005	0.001	0.006	0.003	0.000	0.000	0.000	2.543	0.345	2.888	1.507	0.000	0.000	0	25.430	3.450	28.880	15.070	0.000	0.000
150	original	0.6	60x8 weeks	NOTEST	NA	VAC70	75	All	0.000	0.005	0.001	0.006	0.003	0.000	0.000	0.000	2.543	0.345	2.888	1.507	0.000	0.000	0	25.430	3.450	28.880	15.070	0.000	0.000
150	original	1	60x8 weeks	NOTEST	NA	VAC70	75	All	0.000	0.005	0.001	0.006	0.003	0.000	0.000	0.000	2.419	0.646	3.065	1.599	0.000	0.000	0	24.190	6.460	30.650	15.990	0.000	0.000
150	original	1	60x8 weeks	NOTEST	NA	VAC70	75	All	0.000	0.005	0.001	0.006	0.003	0.000	0.000	0.000	2.419	0.646	3.065	1.599	0.000	0.000	0	24.190	6.460	30.650	15.990	0.000	0.000
150	original	1	60x8 weeks	NOTEST	NA	VAC70	75	All	0.000	0.005	0.001	0.006	0.003	0.000	0.000	0.000	2.419	0.646	3.065	1.599	0.000	0.000	0	24.190	6.460	30.650	15.990	0.000	0.000
150	original	1.2	60x8 weeks	NOTEST	NA	VAC70	75	All	0.000	0.005	0.002	0.007	0.003	0.000	0.000	0.000	2.398	0.781	3.179	1.588	0.000	0.000	0	23.980	7.810	31.790	15.880	0.000	0.000
150	original	1.2	60x8 weeks	NOTEST	NA	VAC70	75	All	0.000	0.005	0.002	0.007	0.003	0.000	0.000	0.000	2.398	0.781	3.179	1.588	0.000	0.000	0	23.980	7.810	31.790	15.880	0.000	0.000
150	original	1.2	60x8 weeks	NOTEST	NA	VAC70	75	All	0.000	0.005	0.002	0.007	0.003	0.000	0.000	0.000	2.398	0.781	3.179	1.588	0.000	0.000	0	23.980	7.810	31.790	15.880	0.000	0.000
150	alpha	0.6	60x8 weeks	NOTEST	NA	VAC70	75	All	0.000	0.005	0.003	0.008	0.005	0.000	0.000	0.000	2.371	1.436	3.807	2.345	0.000	0.000	0	23.710	14.360	38.070	23.450	0.000	0.000
150	alpha	0.6	60x8 weeks	NOTEST	NA	VAC70	75	All	0.000	0.005	0.003	0.008	0.005	0.000	0.000	0.000	2.371	1.436	3.807	2.345	0.000	0.000	0	23.710	14.360	38.070	23.450	0.000	0.000
150	alpha	0.6	60x8 weeks	NOTEST	NA	VAC70	75	All	0.000	0.005	0.003	0.008	0.005	0.000	0.000	0.000	2.371	1.436	3.807	2.345	0.000	0.000	0	23.710	14.360	38.070	23.450	0.000	0.000
150	alpha	1	60x8 weeks	NOTEST	NA	VAC70	75	All	0.000	0.005	0.007	0.011	0.007	0.000	0.000	0.000	2.299	3.156	5.455	3.304	0.000	0.000	0	22.990	31.560	54.550	33.040	0.000	0.000
150	alpha	1	60x8 weeks	NOTEST	NA	VAC70	75	All	0.000	0.005	0.007	0.011	0.007	0.000	0.000	0.000	2.299	3.156	5.455	3.304	0.000	0.000	0	22.990	31.560	54.550	33.040	0.000	0.000
150	alpha	1	60x8 weeks	NOTEST	NA	VAC70	75	All	0.000	0.005	0.007	0.011	0.007	0.000	0.000	0.000	2.299	3.156	5.455	3.304	0.000	0.000	0	22.990	31.560	54.550	33.040	0.000	0.000
150	alpha	1.2	60x8 weeks	NOTEST	NA	VAC70	75	All	0.000	0.005	0.009	0.014	0.009	0.000	0.000	0.000	2.401	4.555	6.956	4.233	0.000	0.000	0	24.010	45.550	69.560	42.330	0.000	0.000
150	alpha	1.2	60x8 weeks	NOTEST	NA	VAC70	75	All	0.000	0.005	0.009	0.014	0.009	0.000	0.000	0.000	2.401	4.555	6.956	4.233	0.000	0.000	0	24.010	45.550	69.560	42.330	0.000	0.000
150	alpha	1.2	60x8 weeks	NOTEST	NA	VAC70	75	All	0.000	0.005	0.009	0.014	0.009	0.000	0.000	0.000	2.401	4.555	6.956	4.233	0.000	0.000	0	24.010	45.550	69.560	42.330	0.000	0.000
150	delta	0.6	60x8 weeks	NOTEST	NA	VAC70	75	All	0.000	0.005	0.015	0.020	0.013	0.000	0.000	0.000	2.601	7.130	9.731	6.162	0.000	0.000	0	26.010	71.300	97.310	61.620	0.000	0.000
150	delta	0.6	60x8 weeks	NOTEST	NA	VAC70	75	All	0.000	0.005	0.015	0.020	0.013	0.000	0.000	0.000	2.601	7.130	9.731	6.162	0.000	0.000	0	26.010	71.300	97.310	61.620	0.000	0.000
150	delta	0.6	60x8 weeks	NOTEST	NA	VAC70	75	All	0.000	0.005	0.015	0.020	0.013	0.000	0.000	0.000	2.601	7.130	9.731	6.162	0.000	0.000	0	26.010	71.300	97.310	61.620	0.000	0.000
150	delta	1	60x8 weeks	NOTEST	NA	VAC70	75	All	0.000	0.005	0.050	0.055	0.035	0.000	0.000	0.000	2.253	24.047	26.300	16.791	0.000	0.000	0	22.530	240.470	263.000	167.910	0.000	0.000
150	delta	1	60x8 weeks	NOTEST	NA	VAC70	75	All	0.000	0.005	0.050	0.055	0.035	0.000	0.000	0.000	2.253	24.047	26.300	16.791	0.000	0.000	0	22.530	240.470	263.000	167.910	0.000	0.000
150	delta	1	60x8 weeks	NOTEST	NA	VAC70	75	All	0.000	0.005	0.050	0.055	0.035	0.000	0.000	0.000	2.253	24.047	26.300	16.791	0.000	0.000	0	22.530	240.470	263.000	167.910	0.000	0.000
150	delta	1.2	60x8 weeks	NOTEST	NA	VAC70	75	All	0.000	0.004	0.069	0.073	0.047	0.000	0.000	0.000	1.988	33.027	35.015	22.519	0.000	0.000	0	19.880	330.270	350.150	225.190	0.000	0.000
150	delta	1.2	60x8 weeks	NOTEST	NA	VAC70	75	All	0.000	0.004	0.069	0.073	0.047	0.000	0.000	0.000	1.988	33.027	35.015	22.519	0.000	0.000	0	19.880	330.270	350.150	225.190	0.000	0.000
150	delta	1.2	60x8 weeks	NOTEST	NA	VAC70	75	All	0.000	0.004	0.069	0.073	0.047	0.000	0.000	0.000	1.988	33.027	35.015	22.519	0.000	0.000	0	19.880	330.270	350.150	225.190	0.000	0.000
150	original	0.6	60x8 weeks	NOTEST	NA	VAC70	100	All	0.000	0.003	0.000	0.004	0.002	0.000	0.000	0.000	1.661	0.021	1.682	0.816	0.000	0.000	0	16.610	0.210	16.820	8.160	0.000	0.000
150	original	0.6	60x8 weeks	NOTEST	NA	VAC70	100	All	0.000	0.003	0.000	0.004	0.002	0.000	0.000	0.000	1.661	0.021	1.682	0.816	0.000	0.000	0	16.610	0.210	16.820	8.160	0.000	0.000
150	original	0.6	60x8 weeks	NOTEST	NA	VAC70	100	All	0.000	0.003	0.000	0.004	0.002	0.000	0.000	0.000	1.661	0.021	1.682	0.816	0.000	0.000	0	16.610	0.210	16.820	8.160	0.000	0.000
150	original	1	60x8 weeks	NOTEST	NA	VAC70	100	All	0.000	0.004	0.000	0.004	0.002	0.000	0.000	0.000	1.694	0.024	1.718	0.846	0.000	0.000	0	16.940	0.240	17.180	8.460	0.000	0.000
150	original	1	60x8 weeks	NOTEST	NA	VAC70	100	All	0.000	0.004	0.000	0.004	0.002	0.000	0.000	0.000	1.694	0.024	1.718	0.846	0.000	0.000	0	16.940	0.240	17.180	8.460	0.000	0.000
150	original	1	60x8 weeks	NOTEST	NA	VAC70	100	All	0.000	0.004	0.000	0.004	0.002	0.000	0.000	0.000	1.694	0.024	1.718	0.846	0.000	0.000	0	16.940	0.240	17.180	8.460	0.000	0.000
150	original	1.2	60x8 weeks	NOTEST	NA	VAC70	100	All	0.000	0.003	0.000	0.003	0.002	0.000	0.000	0.000	1.625	0.025	1.650	0.763	0.000	0.000	0	16.250	0.250	16.500	7.630	0.000	0.000
150	original	1.2	60x8 weeks	NOTEST	NA	VAC70	100	All	0.000	0.003	0.000	0.003	0.002	0.000	0.000	0.000	1.625	0.025	1.650	0.763	0.000	0.000	0	16.250	0.250	16.500	7.630	0.000	0.000
150	original	1.2	60x8 weeks	NOTEST	NA	VAC70	100	All	0.000	0.003	0.000	0.003	0.002	0.000	0.000	0.000	1.625	0.025	1.650	0.763	0.000	0.000	0	16.250	0.250	16.500	7.630	0.000	0.000
150	alpha	0.6	60x8 weeks	NOTEST	NA	VAC70	100	All	0.000	0.003	0.001	0.004	0.002	0.000	0.000	0.000	1.588	0.309	1.897	1.105	0.000	0.000	0	15.880	3.090	18.970	11.050	0.000	0.000
150	alpha	0.6	60x8 weeks	NOTEST	NA	VAC70	100	All	0.000	0.003	0.001	0.004	0.002	0.000	0.000	0.000	1.588	0.309	1.897	1.105	0.000	0.000	0	15.880	3.090	18.970	11.050	0.000	0.000
150	alpha	0.6	60x8 weeks	NOTEST	NA	VAC70	100	All	0.000	0.003	0.001	0.004	0.002	0.000	0.000	0.000	1.588	0.309	1.897	1.105	0.000	0.000	0	15.880	3.090	18.970	11.050	0.000	0.000
150	alpha	1	60x8 weeks	NOTEST	NA	VAC70	100	All	0.000	0.003	0.001	0.005	0.003	0.000	0.000	0.000	1.598	0.652	2.250	1.341	0.000	0.000	0	15.980	6.520	22.500	13.410	0.000	0.000
150	alpha	1	60x8 weeks	NOTEST	NA	VAC70	100	All	0.000	0.003	0.001	0.005	0.003	0.000	0.000	0.000	1.598	0.652	2.250	1.341	0.000	0.000	0	15.980	6.520	22.500	13.410	0.000	0.000
150	alpha	1	60x8 weeks	NOTEST	NA	VAC70	100	All	0.000	0.003	0.001	0.005	0.003	0.000	0.000	0.000	1.598	0.652	2.250	1.341	0.000	0.000	0	15.980	6.520	22.500	13.410	0.000	0.000
150	alpha	1.2	60x8 weeks	NOTEST	NA	VAC70	100	All	0.000	0.003	0.002	0.005	0.003	0.000	0.000	0.000	1.527	0.745	2.272	1.364	0.000	0.000	0	15.270	7.450	22.720	13.640	0.000	0.000
150	alpha	1.2	60x8 weeks	NOTEST	NA	VAC70	100	All	0.000	0.003	0.002	0.005	0.003	0.000	0.000	0.000	1.527	0.745	2.272	1.364	0.000	0.000	0	15.270	7.450	22.720	13.640	0.000	0.000
150	alpha	1.2	60x8 weeks	NOTEST	NA	VAC70	100	All	0.000	0.003	0.002	0.005	0.003	0.000	0.000	0.000	1.527	0.745	2.272	1.364	0.000	0.000	0	15.270	7.450	22.720	13.640	0.000	0.000
150	delta	0.6	60x8 weeks	NOTEST	NA	VAC70	100	All	0.000	0.004	0.003	0.008	0.005	0.000	0.000	0.000	2.041	1.655	3.696	2.245	0.000	0.000	0	20.410	16.550	36.960	22.450	0.000	0.000
150	delta	0.6	60x8 weeks	NOTEST	NA	VAC70	100	All	0.000	0.004	0.003	0.008	0.005	0.000	0.000	0.000	2.041	1.655	3.696	2.245	0.000	0.000	0	20.410	16.550	36.960	22.450	0.000	0.000
150	delta	0.6	60x8 weeks	NOTEST	NA	VAC70	100	All	0.000	0.004	0.003	0.008	0.005	0.000	0.000	0.000	2.041	1.655	3.696	2.245	0.000	0.000	0	20.410	16.550	36.960	22.450	0.000	0.000
150	delta	1	60x8 weeks	NOTEST	NA	VAC70	100	All	0.000	0.004	0.009	0.013	0.008	0.000	0.000	0.000	1.982	4.392	6.374	3.969	0.000	0.000	0	19.820	43.920	63.740	39.690	0.000	0.000
150	delta	1	60x8 weeks	NOTEST	NA	VAC70	100	All	0.000	0.004	0.009	0.013	0.008	0.000	0.000	0.000	1.982	4.392	6.374	3.969	0.000	0.000	0	19.820	43.920	63.740	39.690	0.000	0.000
150	delta	1	60x8 weeks	NOTEST	NA	VAC70	100	All	0.000	0.004	0.009	0.013	0.008	0.000	0.000	0.000	1.982	4.392	6.374	3.969	0.000	0.000	0	19.820	43.920	63.740	39.690	0.000	0.000
150	delta	1.2	60x8 weeks	NOTEST	NA	VAC70	100	All	0.000	0.004	0.015	0.019	0.012	0.000	0.000	0.000	1.940	7.354	9.294	5.712	0.000	0.000	0	19.400	73.540	92.940	57.120	0.000	0.000
150	delta	1.2	60x8 weeks	NOTEST	NA	VAC70	100	All	0.000	0.004	0.015	0.019	0.012	0.000	0.000	0.000	1.940	7.354	9.294	5.712	0.000	0.000	0	19.400	73.540	92.940	57.120	0.000	0.000
150	delta	1.2	60x8 weeks	NOTEST	NA	VAC70	100	All	0.000	0.004	0.015	0.019	0.012	0.000	0.000	0.000	1.940	7.354	9.294	5.712	0.000	0.000	0	19.400	73.540	92.940	57.120	0.000	0.000
150	original	0.6	60x8 weeks	NOTEST	NA	VAC50	25	All	0.000	0.009	0.005	0.013	0.007	0.000	0.000	0.000	4.147	2.253	6.400	3.452	0.000	0.000	0	41.470	22.530	64.000	34.520	0.000	0.000
150	original	0.6	60x8 weeks	NOTEST	NA	VAC50	25	All	0.000	0.009	0.005	0.013	0.007	0.000	0.000	0.000	4.147	2.253	6.400	3.452	0.000	0.000	0	41.470	22.530	64.000	34.520	0.000	0.000

150	original	0.6	60x8 weeks	NOTEST	NA	VAC50	25	All	0.000	0.009	0.005	0.013	0.007	0.000	0.000	0.000	4.147	2.253	6.400	3.452	0.000	0.000	0	41.470	22.530	64.000	34.520	0.000	0.000
150	original	1	60x8 weeks	NOTEST	NA	VAC50	25	All	0.000	0.008	0.011	0.019	0.010	0.000	0.000	0.000	4.064	5.281	9.345	5.017	0.000	0.000	0	40.640	52.810	93.450	50.170	0.000	0.000
150	original	1	60x8 weeks	NOTEST	NA	VAC50	25	All	0.000	0.008	0.011	0.019	0.010	0.000	0.000	0.000	4.064	5.281	9.345	5.017	0.000	0.000	0	40.640	52.810	93.450	50.170	0.000	0.000
150	original	1	60x8 weeks	NOTEST	NA	VAC50	25	All	0.000	0.008	0.011	0.019	0.010	0.000	0.000	0.000	4.064	5.281	9.345	5.017	0.000	0.000	0	40.640	52.810	93.450	50.170	0.000	0.000
150	original	1.2	60x8 weeks	NOTEST	NA	VAC50	25	All	0.000	0.008	0.016	0.024	0.013	0.000	0.000	0.000	3.969	7.664	11.633	6.295	0.000	0.000	0	39.690	76.640	116.330	62.950	0.000	0.000
150	original	1.2	60x8 weeks	NOTEST	NA	VAC50	25	All	0.000	0.008	0.016	0.024	0.013	0.000	0.000	0.000	3.969	7.664	11.633	6.295	0.000	0.000	0	39.690	76.640	116.330	62.950	0.000	0.000
150	original	1.2	60x8 weeks	NOTEST	NA	VAC50	25	All	0.000	0.008	0.016	0.024	0.013	0.000	0.000	0.000	3.969	7.664	11.633	6.295	0.000	0.000	0	39.690	76.640	116.330	62.950	0.000	0.000
150	alpha	0.6	60x8 weeks	NOTEST	NA	VAC50	25	All	0.000	0.008	0.021	0.029	0.019	0.000	0.000	0.000	3.856	10.120	13.976	9.013	0.000	0.000	0	38.560	101.200	139.760	90.130	0.000	0.000
150	alpha	0.6	60x8 weeks	NOTEST	NA	VAC50	25	All	0.000	0.008	0.021	0.029	0.019	0.000	0.000	0.000	3.856	10.120	13.976	9.013	0.000	0.000	0	38.560	101.200	139.760	90.130	0.000	0.000
150	alpha	0.6	60x8 weeks	NOTEST	NA	VAC50	25	All	0.000	0.008	0.021	0.029	0.019	0.000	0.000	0.000	3.856	10.120	13.976	9.013	0.000	0.000	0	38.560	101.200	139.760	90.130	0.000	0.000
150	alpha	1	60x8 weeks	NOTEST	NA	VAC50	25	All	0.000	0.007	0.067	0.074	0.048	0.000	0.000	0.000	3.157	32.221	35.378	23.157	0.000	0.000	0	31.570	322.210	353.780	231.570	0.000	0.000
150	alpha	1	60x8 weeks	NOTEST	NA	VAC50	25	All	0.000	0.007	0.067	0.074	0.048	0.000	0.000	0.000	3.157	32.221	35.378	23.157	0.000	0.000	0	31.570	322.210	353.780	231.570	0.000	0.000
150	alpha	1	60x8 weeks	NOTEST	NA	VAC50	25	All	0.000	0.007	0.067	0.074	0.048	0.000	0.000	0.000	3.157	32.221	35.378	23.157	0.000	0.000	0	31.570	322.210	353.780	231.570	0.000	0.000
150	alpha	1.2	60x8 weeks	NOTEST	NA	VAC50	25	All	0.000	0.006	0.084	0.090	0.060	0.000	0.000	0.000	2.780	40.212	42.992	28.695	0.000	0.000	0	27.800	402.120	429.920	286.950	0.000	0.000
150	alpha	1.2	60x8 weeks	NOTEST	NA	VAC50	25	All	0.000	0.006	0.084	0.090	0.060	0.000	0.000	0.000	2.780	40.212	42.992	28.695	0.000	0.000	0	27.800	402.120	429.920	286.950	0.000	0.000
150	alpha	1.2	60x8 weeks	NOTEST	NA	VAC50	25	All	0.000	0.006	0.084	0.090	0.060	0.000	0.000	0.000	2.780	40.212	42.992	28.695	0.000	0.000	0	27.800	402.120	429.920	286.950	0.000	0.000
150	delta	0.6	60x8 weeks	NOTEST	NA	VAC50	25	All	0.000	0.005	0.095	0.101	0.068	0.000	0.000	0.000	2.631	45.774	48.405	32.759	0.000	0.000	0	26.310	457.740	484.050	327.590	0.000	0.000
150	delta	0.6	60x8 weeks	NOTEST	NA	VAC50	25	All	0.000	0.005	0.095	0.101	0.068	0.000	0.000	0.000	2.631	45.774	48.405	32.759	0.000	0.000	0	26.310	457.740	484.050	327.590	0.000	0.000
150	delta	0.6	60x8 weeks	NOTEST	NA	VAC50	25	All	0.000	0.005	0.095	0.101	0.068	0.000	0.000	0.000	2.631	45.774	48.405	32.759	0.000	0.000	0	26.310	457.740	484.050	327.590	0.000	0.000
150	delta	1	60x8 weeks	NOTEST	NA	VAC50	25	All	0.000	0.004	0.115	0.119	0.082	0.000	0.000	0.000	1.906	55.235	57.141	39.303	0.000	0.000	0	19.060	552.350	571.410	393.030	0.000	0.000
150	delta	1	60x8 weeks	NOTEST	NA	VAC50	25	All	0.000	0.004	0.115	0.119	0.082	0.000	0.000	0.000	1.906	55.235	57.141	39.303	0.000	0.000	0	19.060	552.350	571.410	393.030	0.000	0.000
150	delta	1	60x8 weeks	NOTEST	NA	VAC50	25	All	0.000	0.004	0.115	0.119	0.082	0.000	0.000	0.000	1.906	55.235	57.141	39.303	0.000	0.000	0	19.060	552.350	571.410	393.030	0.000	0.000
150	delta	1.2	60x8 weeks	NOTEST	NA	VAC50	25	All	0.000	0.004	0.119	0.122	0.084	0.000	0.000	0.000	1.786	56.894	58.680	40.223	0.000	0.000	0	17.860	568.940	586.800	402.230	0.000	0.000
150	delta	1.2	60x8 weeks	NOTEST	NA	VAC50	25	All	0.000	0.004	0.119	0.122	0.084	0.000	0.000	0.000	1.786	56.894	58.680	40.223	0.000	0.000	0	17.860	568.940	586.800	402.230	0.000	0.000
150	delta	1.2	60x8 weeks	NOTEST	NA	VAC50	25	All	0.000	0.004	0.119	0.122	0.084	0.000	0.000	0.000	1.786	56.894	58.680	40.223	0.000	0.000	0	17.860	568.940	586.800	402.230	0.000	0.000
150	original	0.6	60x8 weeks	NOTEST	NA	VAC50	50	All	0.000	0.008	0.002	0.010	0.005	0.000	0.000	0.000	3.689	1.140	4.829	2.541	0.000	0.000	0	36.890	11.400	48.290	25.410	0.000	0.000
150	original	0.6	60x8 weeks	NOTEST	NA	VAC50	50	All	0.000	0.008	0.002	0.010	0.005	0.000	0.000	0.000	3.689	1.140	4.829	2.541	0.000	0.000	0	36.890	11.400	48.290	25.410	0.000	0.000
150	original	0.6	60x8 weeks	NOTEST	NA	VAC50	50	All	0.000	0.008	0.002	0.010	0.005	0.000	0.000	0.000	3.689	1.140	4.829	2.541	0.000	0.000	0	36.890	11.400	48.290	25.410	0.000	0.000
150	original	1	60x8 weeks	NOTEST	NA	VAC50	50	All	0.000	0.008	0.005	0.013	0.007	0.000	0.000	0.000	3.608	2.394	6.002	3.243	0.000	0.000	0	36.080	23.940	60.020	32.430	0.000	0.000
150	original	1	60x8 weeks	NOTEST	NA	VAC50	50	All	0.000	0.008	0.005	0.013	0.007	0.000	0.000	0.000	3.608	2.394	6.002	3.243	0.000	0.000	0	36.080	23.940	60.020	32.430	0.000	0.000
150	original	1	60x8 weeks	NOTEST	NA	VAC50	50	All	0.000	0.008	0.005	0.013	0.007	0.000	0.000	0.000	3.608	2.394	6.002	3.243	0.000	0.000	0	36.080	23.940	60.020	32.430	0.000	0.000
150	original	1.2	60x8 weeks	NOTEST	NA	VAC50	50	All	0.000	0.008	0.006	0.014	0.007	0.000	0.000	0.000	3.642	3.105	6.747	3.490	0.000	0.000	0	36.420	31.050	67.470	34.900	0.000	0.000
150	original	1.2	60x8 weeks	NOTEST	NA	VAC50	50	All	0.000	0.008	0.006	0.014	0.007	0.000	0.000	0.000	3.642	3.105	6.747	3.490	0.000	0.000	0	36.420	31.050	67.470	34.900	0.000	0.000
150	original	1.2	60x8 weeks	NOTEST	NA	VAC50	50	All	0.000	0.008	0.006	0.014	0.007	0.000	0.000	0.000	3.642	3.105	6.747	3.490	0.000	0.000	0	36.420	31.050	67.470	34.900	0.000	0.000
150	alpha	0.6	60x8 weeks	NOTEST	NA	VAC50	50	All	0.000	0.007	0.011	0.019	0.012	0.000	0.000	0.000	3.533	5.406	8.939	5.653	0.000	0.000	0	35.330	54.060	89.390	56.530	0.000	0.000
150	alpha	0.6	60x8 weeks	NOTEST	NA	VAC50	50	All	0.000	0.007	0.011	0.019	0.012	0.000	0.000	0.000	3.533	5.406	8.939	5.653	0.000	0.000	0	35.330	54.060	89.390	56.530	0.000	0.000
150	alpha	0.6	60x8 weeks	NOTEST	NA	VAC50	50	All	0.000	0.007	0.011	0.019	0.012	0.000	0.000	0.000	3.533	5.406	8.939	5.653	0.000	0.000	0	35.330	54.060	89.390	56.530	0.000	0.000
150	alpha	1	60x8 weeks	NOTEST	NA	VAC50	50	All	0.000	0.007	0.033	0.040	0.025	0.000	0.000	0.000	3.190	15.859	19.049	12.034	0.000	0.000	0	31.900	158.590	190.490	120.340	0.000	0.000
150	alpha	1	60x8 weeks	NOTEST	NA	VAC50	50	All	0.000	0.007	0.033	0.040	0.025	0.000	0.000	0.000	3.190	15.859	19.049	12.034	0.000	0.000	0	31.900	158.590	190.490	120.340	0.000	0.000
150	alpha	1	60x8 weeks	NOTEST	NA	VAC50	50	All	0.000	0.007	0.033	0.040	0.025	0.000	0.000	0.000	3.190	15.859	19.049	12.034	0.000	0.000	0	31.900	158.590	190.490	120.340	0.000	0.000
150	alpha	1.2	60x8 weeks	NOTEST	NA	VAC50	50	All	0.000	0.006	0.047	0.053	0.034	0.000	0.000	0.000	2.916	22.628	25.544	16.139	0.000	0.000	0	29.160	226.280	255.440	161.390	0.000	0.000
150	alpha	1.2	60x8 weeks	NOTEST	NA	VAC50	50	All	0.000	0.006	0.047	0.053	0.034	0.000	0.000	0.000	2.916	22.628	25.544	16.139	0.000	0.000	0	29.160	226.280	255.440	161.390	0.000	0.000
150	alpha	1.2	60x8 weeks	NOTEST	NA	VAC50	50	All	0.000	0.006	0.047	0.053	0.034	0.000	0.000	0.000	2.916	22.628	25.544	16.139	0.000	0.000	0	29.160	226.280	255.440	161.390	0.000	0.000
150	delta	0.6	60x8 weeks	NOTEST	NA	VAC50	50	All	0.000	0.006	0.069	0.076	0.050	0.000	0.000	0.000	2.992	33.280	36.272	23.962	0.000	0.000	0	29.920	332.800	362.720	239.620	0.000	0.000
150	delta	0.6	60x8 weeks	NOTEST	NA	VAC50	50	All	0.000	0.006	0.069	0.076	0.050	0.000	0.000	0.000	2.992	33.280	36.272	23.962	0.000	0.000	0	29.920	332.800	362.720	239.620	0.000	0.000
150	delta	0.6	60x8 weeks	NOTEST	NA	VAC50	50	All	0.000	0.006	0.069	0.076	0.050	0.000	0.000	0.000	2.992	33.280	36.272	23.962	0.000	0.000	0	29.920	332.800	362.720	239.620	0.000	0.000
150	delta	1	60x8 weeks	NOTEST	NA	VAC50	50	All	0.000	0.004	0.108	0.112	0.076	0.000	0.000	0.000	2.064	51.855	53.919	36.633	0.000	0.000	0	20.640	518.550	539.190	366.330	0.000	0.000
150	delta	1	60x8 weeks	NOTEST	NA	VAC50	50	All	0.000	0.004	0.108	0.112	0.076	0.000	0.000	0.000	2.064	51.855	53.919	36.633	0.000	0.000	0	20.640	518.550	539.190	366.330	0.000	0.000
150	delta	1	60x8 weeks	NOTEST	NA	VAC50	50	All	0.000	0.004	0.108	0.112	0.076	0.000	0.000	0.000	2.064	51.855	53.919	36.633	0.000	0.000	0	20.640	518.550	539.190	366.330	0.000	0.000
150	delta	1.2	60x8 weeks	NOTEST	NA	VAC50	50	All	0.000	0.004	0.114	0.118	0.080	0.000	0.000	0.000	1.849	54.916	56.765	38.564	0.000	0.000	0	18.490	549.160	567.650	385.640	0.000	0.000
150	delta	1.2	60x8 weeks	NOTEST	NA	VAC50	50	All	0.000	0.004	0.114	0.118	0.080	0.000	0.000	0.000	1.849	54.916	56.765	38.564	0.000	0.000	0	18.490	549.160	567.650	385.640	0.000	0.000
150	delta	1.2	60x8 weeks	NOTEST	NA	VAC50	50	All	0.000	0.004	0.114	0.118	0.080	0.000	0.000	0.000	1.849	54.916	56.765	38.564	0.000	0.000	0	18.490	549.160	567.650	385.640	0.000	0.000
150	original	0.6	60x8 weeks	NOTEST	NA	VAC50	75	All	0.000	0.006	0.001	0.007	0.004	0.000	0.000	0.000	3.071	0.448	3.519	1.903	0.000	0.000	0	30.710	4.480	35.190	19.030	0.000	0.000
150	original	0.6	60x8 weeks	NOTEST	NA	VAC50	75	All	0.000	0.006	0.001	0.007	0.004	0.000	0.000	0.000	3.071	0.448	3.519	1.903	0.000	0.000	0	30.710	4.480	35.190	19.030	0.000	0.000
150	original	0.6	60x8 weeks	NOTEST	NA	VAC50	75	All	0.000	0.006	0.001	0.007	0.004	0.000	0.000	0.000	3.071	0.448	3.519	1.903	0.000	0.000	0	30.710	4.480	35.190	19.030	0.000	0.000
150	original	1	60x8 weeks	NOTEST	NA	VAC50	75	All	0.000	0.006	0.002	0.008	0.004	0.000	0.000	0.000	3.020	0.739	3.759	2.033	0.000	0.000	0	30.200	7.390	37.590	20.330	0.000	0.000
150	original	1	60x8 weeks	NOTEST	NA	VAC50	75	All	0.000	0.006	0.002	0.008	0.004	0.000	0.000	0.000	3.020	0.739	3.759	2.033	0.000	0.000	0	30.200	7.390	37.590	20.330	0.000	0.000
150	original	1	60x8 weeks	NOTEST	NA	VAC50	75	All	0.000	0.006	0.002	0.008	0.004	0.000	0.000	0.000	3.020	0.739	3.759	2.033	0.000	0.000	0	30.200	7.390	37.590	20.330	0.000	0.000
150	original	1.2	60x8 weeks	NOTEST	NA	VAC50	75	All	0.000	0.007	0.002	0.009	0.005	0.000	0.000	0.000	3.134	1.049	4.183	2.217	0.000	0.000	0	31.340	10.490	41.830	22.170	0.000	0.000
150	original	1.2	60x8 weeks	NOTEST	NA	VAC50	75	All	0.000	0.007	0.002	0.009	0.005	0.000	0.000	0.000	3.134	1.049	4.183	2.217	0.000	0.000	0	31.340	10.490	41.830	22.170	0.000	0.000
150	original	1.2	60x8 weeks	NOTEST	NA	VAC50	75	All	0.000	0.007	0.002	0.009	0.005	0.000	0.000	0.000	3.134	1.049	4.183	2.217	0.000	0.000	0	31.340	10.490	41.830	22.170	0.000	0.000
150	alpha	0.6	60x8 weeks	NOTEST	NA	VAC50	75	All	0.000	0.006	0.005	0.011	0.007	0.000	0.000	0.000	3.043	2.384	5.427	3.359	0.000	0.000	0	30.430	23.840	54.270	33.590	0.000	0.000
150	alpha	0.6	60x8 weeks	NOTEST	NA	VAC50	75	All	0.000	0.006	0.005	0.011	0.007	0.000	0.000	0.000	3.043	2.384	5.427	3.359	0.000	0.000	0	30.430	23.840	54.270	33.590	0.000	0.000
150	alpha	0.6	60x8 weeks	NOTEST	NA	VAC50	75	All	0.000	0.006	0.005	0.011	0.007	0.000	0.000	0.000	3.043	2.384	5.427	3.359	0.000	0.000	0	30.430	23.840	54.270	33.590	0.000	0.000
150	alpha	1	60x8 weeks	NOTEST	NA	VAC50	75	All	0.000	0.006	0.013	0.020	0.012	0.000	0.000	0.000	3.001	6.374	9.375	5.897	0.000	0.000	0	30.010	63.740	93.750	58.970	0.000	0.000
150	alpha	1	60x8 weeks	NOTEST	NA	VAC50	75	All	0.000	0.006	0.013	0.020	0.012	0.000	0.000	0.000	3.001	6.374	9.375	5.897	0.000	0.000	0	30.010	63.740	93.750	58.970	0.000	0.000
150	alpha	1	60x8 weeks	NOTEST	NA	VAC50	75	All	0.000	0.006	0.013	0.020	0.012	0.000	0.000	0.000	3.001	6.374	9.375	5.897	0.000	0.000	0	30.010	63.740	93.750	58.970	0.000	0.000
150	alpha	1.2	60x8 weeks	NOTEST	NA	VAC50	75	All	0.000	0.006	0.019	0.025	0.016	0.000	0.000	0.000	2.785	9.262	12.047	7.527	0.000	0.000	0	27.850	92.620	120.470	75.270	0.000	0.000
150	alpha	1.2	60x8 weeks	NOTEST	NA	VAC50	75	All	0.000	0.006	0.019	0.025	0.016	0.000	0.000	0.000	2.785	9.262	12.047	7.527	0.000	0.000	0	27.850	92.620	120.470	75.270	0.000	0.000
150	alpha	1.2	60x8 weeks	NOTEST	NA	VAC50	75	All	0.000	0.006	0.019	0.025	0.016	0.000	0.000	0.000	2.785	9.262	12.047	7.527	0.000	0.000	0	27.850	92.620	120.470	75.270	0.000	0.000
150	delta	0.6	60x8 weeks	NOTEST	NA	VAC50	75	All	0.000	0.007	0.034	0.041	0.027	0.000	0.000	0.000	3.297	16.547	19.844	12.813	0.000	0.000	0	32.970	165.470	198.440	128.130	0.000	0.000

150	delta	0.6	60x8 weeks	NOTEST	NA	VAC50	75	All	0.000	0.007	0.034	0.041	0.027	0.000	0.000	0.000	3.297	16.547	19.844	12.813	0.000	0.000	0	32.970	165.470	198.440	128.130	0.000	0.000
150	delta	0.6	60x8 weeks	NOTEST	NA	VAC50	75	All	0.000	0.007	0.034	0.041	0.027	0.000	0.000	0.000	3.297	16.547	19.844	12.813	0.000	0.000	0	32.970	165.470	198.440	128.130	0.000	0.000
150	delta	1	60x8 weeks	NOTEST	NA	VAC50	75	All	0.000	0.005	0.088	0.093	0.061	0.000	0.000	0.000	2.375	42.234	44.609	29.510	0.000	0.000	0	23.750	422.340	446.090	295.100	0.000	0.000
150	delta	1	60x8 weeks	NOTEST	NA	VAC50	75	All	0.000	0.005	0.088	0.093	0.061	0.000	0.000	0.000	2.375	42.234	44.609	29.510	0.000	0.000	0	23.750	422.340	446.090	295.100	0.000	0.000
150	delta	1	60x8 weeks	NOTEST	NA	VAC50	75	All	0.000	0.005	0.088	0.093	0.061	0.000	0.000	0.000	2.375	42.234	44.609	29.510	0.000	0.000	0	23.750	422.340	446.090	295.100	0.000	0.000
150	delta	1.2	60x8 weeks	NOTEST	NA	VAC50	75	All	0.000	0.004	0.102	0.106	0.071	0.000	0.000	0.000	2.085	48.812	50.897	34.067	0.000	0.000	0	20.850	488.120	508.970	340.670	0.000	0.000
150	delta	1.2	60x8 weeks	NOTEST	NA	VAC50	75	All	0.000	0.004	0.102	0.106	0.071	0.000	0.000	0.000	2.085	48.812	50.897	34.067	0.000	0.000	0	20.850	488.120	508.970	340.670	0.000	0.000
150	delta	1.2	60x8 weeks	NOTEST	NA	VAC50	75	All	0.000	0.004	0.102	0.106	0.071	0.000	0.000	0.000	2.085	48.812	50.897	34.067	0.000	0.000	0	20.850	488.120	508.970	340.670	0.000	0.000
150	original	0.6	60x8 weeks	NOTEST	NA	VAC50	100	All	0.000	0.005	0.000	0.005	0.003	0.000	0.000	0.000	2.429	0.030	2.459	1.270	0.000	0.000	0	24.290	0.300	24.590	12.700	0.000	0.000
150	original	0.6	60x8 weeks	NOTEST	NA	VAC50	100	All	0.000	0.005	0.000	0.005	0.003	0.000	0.000	0.000	2.429	0.030	2.459	1.270	0.000	0.000	0	24.290	0.300	24.590	12.700	0.000	0.000
150	original	0.6	60x8 weeks	NOTEST	NA	VAC50	100	All	0.000	0.005	0.000	0.005	0.003	0.000	0.000	0.000	2.429	0.030	2.459	1.270	0.000	0.000	0	24.290	0.300	24.590	12.700	0.000	0.000
150	original	1	60x8 weeks	NOTEST	NA	VAC50	100	All	0.000	0.005	0.000	0.005	0.003	0.000	0.000	0.000	2.532	0.042	2.574	1.327	0.000	0.000	0	25.320	0.420	25.740	13.270	0.000	0.000
150	original	1	60x8 weeks	NOTEST	NA	VAC50	100	All	0.000	0.005	0.000	0.005	0.003	0.000	0.000	0.000	2.532	0.042	2.574	1.327	0.000	0.000	0	25.320	0.420	25.740	13.270	0.000	0.000
150	original	1	60x8 weeks	NOTEST	NA	VAC50	100	All	0.000	0.005	0.000	0.005	0.003	0.000	0.000	0.000	2.532	0.042	2.574	1.327	0.000	0.000	0	25.320	0.420	25.740	13.270	0.000	0.000
150	original	1.2	60x8 weeks	NOTEST	NA	VAC50	100	All	0.000	0.005	0.000	0.006	0.003	0.000	0.000	0.000	2.601	0.065	2.666	1.359	0.000	0.000	0	26.010	0.650	26.660	13.590	0.000	0.000
150	original	1.2	60x8 weeks	NOTEST	NA	VAC50	100	All	0.000	0.005	0.000	0.006	0.003	0.000	0.000	0.000	2.601	0.065	2.666	1.359	0.000	0.000	0	26.010	0.650	26.660	13.590	0.000	0.000
150	original	1.2	60x8 weeks	NOTEST	NA	VAC50	100	All	0.000	0.005	0.000	0.006	0.003	0.000	0.000	0.000	2.601	0.065	2.666	1.359	0.000	0.000	0	26.010	0.650	26.660	13.590	0.000	0.000
150	alpha	0.6	60x8 weeks	NOTEST	NA	VAC50	100	All	0.000	0.005	0.002	0.007	0.004	0.000	0.000	0.000	2.406	0.849	3.255	1.986	0.000	0.000	0	24.060	8.490	32.550	19.860	0.000	0.000
150	alpha	0.6	60x8 weeks	NOTEST	NA	VAC50	100	All	0.000	0.005	0.002	0.007	0.004	0.000	0.000	0.000	2.406	0.849	3.255	1.986	0.000	0.000	0	24.060	8.490	32.550	19.860	0.000	0.000
150	alpha	0.6	60x8 weeks	NOTEST	NA	VAC50	100	All	0.000	0.005	0.002	0.007	0.004	0.000	0.000	0.000	2.406	0.849	3.255	1.986	0.000	0.000	0	24.060	8.490	32.550	19.860	0.000	0.000
150	alpha	1	60x8 weeks	NOTEST	NA	VAC50	100	All	0.000	0.005	0.004	0.009	0.005	0.000	0.000	0.000	2.417	1.838	4.255	2.602	0.000	0.000	0	24.170	18.380	42.550	26.020	0.000	0.000
150	alpha	1	60x8 weeks	NOTEST	NA	VAC50	100	All	0.000	0.005	0.004	0.009	0.005	0.000	0.000	0.000	2.417	1.838	4.255	2.602	0.000	0.000	0	24.170	18.380	42.550	26.020	0.000	0.000
150	alpha	1	60x8 weeks	NOTEST	NA	VAC50	100	All	0.000	0.005	0.004	0.009	0.005	0.000	0.000	0.000	2.417	1.838	4.255	2.602	0.000	0.000	0	24.170	18.380	42.550	26.020	0.000	0.000
150	alpha	1.2	60x8 weeks	NOTEST	NA	VAC50	100	All	0.000	0.005	0.006	0.011	0.007	0.000	0.000	0.000	2.510	2.665	5.175	3.244	0.000	0.000	0	25.100	26.650	51.750	32.440	0.000	0.000
150	alpha	1.2	60x8 weeks	NOTEST	NA	VAC50	100	All	0.000	0.005	0.006	0.011	0.007	0.000	0.000	0.000	2.510	2.665	5.175	3.244	0.000	0.000	0	25.100	26.650	51.750	32.440	0.000	0.000
150	alpha	1.2	60x8 weeks	NOTEST	NA	VAC50	100	All	0.000	0.005	0.006	0.011	0.007	0.000	0.000	0.000	2.510	2.665	5.175	3.244	0.000	0.000	0	25.100	26.650	51.750	32.440	0.000	0.000
150	delta	0.6	60x8 weeks	NOTEST	NA	VAC50	100	All	0.000	0.007	0.013	0.020	0.013	0.000	0.000	0.000	3.153	6.416	9.569	6.093	0.000	0.000	0	31.530	64.160	95.690	60.930	0.000	0.000
150	delta	0.6	60x8 weeks	NOTEST	NA	VAC50	100	All	0.000	0.007	0.013	0.020	0.013	0.000	0.000	0.000	3.153	6.416	9.569	6.093	0.000	0.000	0	31.530	64.160	95.690	60.930	0.000	0.000
150	delta	0.6	60x8 weeks	NOTEST	NA	VAC50	100	All	0.000	0.007	0.013	0.020	0.013	0.000	0.000	0.000	3.153	6.416	9.569	6.093	0.000	0.000	0	31.530	64.160	95.690	60.930	0.000	0.000
150	delta	1	60x8 weeks	NOTEST	NA	VAC50	100	All	0.000	0.006	0.049	0.055	0.035	0.000	0.000	0.000	2.744	23.744	26.488	17.028	0.000	0.000	0	27.440	237.440	264.880	170.280	0.000	0.000
150	delta	1	60x8 weeks	NOTEST	NA	VAC50	100	All	0.000	0.006	0.049	0.055	0.035	0.000	0.000	0.000	2.744	23.744	26.488	17.028	0.000	0.000	0	27.440	237.440	264.880	170.280	0.000	0.000
150	delta	1	60x8 weeks	NOTEST	NA	VAC50	100	All	0.000	0.006	0.049	0.055	0.035	0.000	0.000	0.000	2.744	23.744	26.488	17.028	0.000	0.000	0	27.440	237.440	264.880	170.280	0.000	0.000
150	delta	1.2	60x8 weeks	NOTEST	NA	VAC50	100	All	0.000	0.005	0.069	0.075	0.049	0.000	0.000	0.000	2.506	33.297	35.803	23.317	0.000	0.000	0	25.060	332.970	358.030	233.170	0.000	0.000
150	delta	1.2	60x8 weeks	NOTEST	NA	VAC50	100	All	0.000	0.005	0.069	0.075	0.049	0.000	0.000	0.000	2.506	33.297	35.803	23.317	0.000	0.000	0	25.060	332.970	358.030	233.170	0.000	0.000
150	delta	1.2	60x8 weeks	NOTEST	NA	VAC50	100	All	0.000	0.005	0.069	0.075	0.049	0.000	0.000	0.000	2.506	33.297	35.803	23.317	0.000	0.000	0	25.060	332.970	358.030	233.170	0.000	0.000
120	original	0.6	60x8 weeks	PCR90	Daily	NOVAC	0	All	4.928	0.008	0.002	0.010	0.001	0.010	0.005	2365.363	3.913	1.090	5.003	0.296	4.943	2.243	23654	39.130	10.900	50.030	2.960	49.430	22.430
120	original	0.6	60x8 weeks	PCR90	Daily	NOVAC	0	All	4.928	0.008	0.002	0.010	0.001	0.010	0.005	2365.363	3.913	1.090	5.003	0.296	4.943	2.243	23654	39.130	10.900	50.030	2.960	49.430	22.430
120	original	0.6	60x8 weeks	PCR90	Daily	NOVAC	0	All	4.928	0.008	0.002	0.010	0.001	0.010	0.005	2365.363	3.913	1.090	5.003	0.296	4.943	2.243	23654	39.130	10.900	50.030	2.960	49.430	22.430
120	original	1	60x8 weeks	PCR90	Daily	NOVAC	0	All	4.915	0.008	0.005	0.012	0.001	0.012	0.005	2359.202	3.792	2.170	5.962	0.378	5.859	2.275	23592	37.920	21.700	59.620	3.780	58.590	22.750
120	original	1	60x8 weeks	PCR90	Daily	NOVAC	0	All	4.915	0.008	0.005	0.012	0.001	0.012	0.005	2359.202	3.792	2.170	5.962	0.378	5.859	2.275	23592	37.920	21.700	59.620	3.780	58.590	22.750
120	original	1	60x8 weeks	PCR90	Daily	NOVAC	0	All	4.915	0.008	0.005	0.012	0.001	0.012	0.005	2359.202	3.792	2.170	5.962	0.378	5.859	2.275	23592	37.920	21.700	59.620	3.780	58.590	22.750
120	original	1.2	60x8 weeks	PCR90	Daily	NOVAC	0	All	4.905	0.008	0.006	0.014	0.001	0.014	0.005	2354.224	3.714	2.972	6.686	0.433	6.532	2.272	23542	37.140	29.720	66.860	4.330	65.320	22.720
120	original	1.2	60x8 weeks	PCR90	Daily	NOVAC	0	All	4.905	0.008	0.006	0.014	0.001	0.014	0.005	2354.224	3.714	2.972	6.686	0.433	6.532	2.272	23542	37.140	29.720	66.860	4.330	65.320	22.720
120	original	1.2	60x8 weeks	PCR90	Daily	NOVAC	0	All	4.905	0.008	0.006	0.014	0.001	0.014	0.005	2354.224	3.714	2.972	6.686	0.433	6.532	2.272	23542	37.140	29.720	66.860	4.330	65.320	22.720
120	alpha	0.6	60x8 weeks	PCR90	Daily	NOVAC	0	All	4.908	0.008	0.005	0.013	0.001	0.013	0.005	2356.022	3.789	2.545	6.334	0.399	6.336	2.327	23560	37.890	25.450	63.340	3.990	63.360	23.270
120	alpha	0.6	60x8 weeks	PCR90	Daily	NOVAC	0	All	4.908	0.008	0.005	0.013	0.001	0.013	0.005	2356.022	3.789	2.545	6.334	0.399	6.336	2.327	23560	37.890	25.450	63.340	3.990	63.360	23.270
120	alpha	0.6	60x8 weeks	PCR90	Daily	NOVAC	0	All	4.908	0.008	0.005	0.013	0.001	0.013	0.005	2356.022	3.789	2.545	6.334	0.399	6.336	2.327	23560	37.890	25.450	63.340	3.990	63.360	23.270
120	alpha	1	60x8 weeks	PCR90	Daily	NOVAC	0	All	4.839	0.008	0.015	0.023	0.002	0.023	0.005	2322.950	3.705	7.170	10.875	0.729	10.938	2.168	23230	37.050	71.700	108.750	7.290	109.380	21.680
120	alpha	1	60x8 weeks	PCR90	Daily	NOVAC	0	All	4.839	0.008	0.015	0.023	0.002	0.023	0.005	2322.950	3.705	7.170	10.875	0.729	10.938	2.168	23230	37.050	71.700	108.750	7.290	109.380	21.680
120	alpha	1	60x8 weeks	PCR90	Daily	NOVAC	0	All	4.839	0.008	0.015	0.023	0.002	0.023	0.005	2322.950	3.705	7.170	10.875	0.729	10.938	2.168	23230	37.050	71.700	108.750	7.290	109.380	21.680
120	alpha	1.2	60x8 weeks	PCR90	Daily	NOVAC	0	All	4.782	0.007	0.023	0.030	0.002	0.031	0.004	2295.441	3.473	11.089	14.562	1.006	14.784	2.058	22954	34.730	110.890	145.620	10.060	147.840	20.580
120	alpha	1.2	60x8 weeks	PCR90	Daily	NOVAC	0	All	4.782	0.007	0.023	0.030	0.002	0.031	0.004	2295.441	3.473	11.089	14.562	1.006	14.784	2.058	22954	34.730	110.890	145.620	10.060	147.840	20.580
120	alpha	1.2	60x8 weeks	PCR90	Daily	NOVAC	0	All	4.782	0.007	0.023	0.030	0.002	0.031	0.004	2295.441	3.473	11.089	14.562	1.006	14.784	2.058	22954	34.730	110.890	145.620	10.060	147.840	20.580
120	delta	0.6	60x8 weeks	PCR90	Daily	NOVAC	0	All	4.736	0.007	0.030	0.037	0.004	0.036	0.004	2273.499	3.368	14.205	17.573	1.858	17.060	2.056	22735	33.680	142.050	175.730	18.580	170.600	20.560
120	delta	0.6	60x8 weeks	PCR90	Daily	NOVAC	0	All	4.736	0.007	0.030	0.037	0.004	0.036	0.004	2273.499	3.368	14.205	17.573	1.858	17.060	2.056	22735	33.680	142.050	175.730	18.580	170.600	20.560
120	delta	0.6	60x8 weeks	PCR90	Daily	NOVAC	0	All	4.736	0.007	0.030	0.037	0.004	0.036	0.004	2273.499	3.368	14.205	17.573	1.858	17.060	2.056	22735	33.680	142.050	175.730	18.580	170.600	20.560
120	delta	1	60x8 weeks	PCR90	Daily	NOVAC	0	All	4.279	0.005	0.091	0.096	0.010	0.095	0.003	2054.059	2.426	43.599	46.025	4.863	45.608	1.370	20541	24.260	435.990	460.250	48.630	456.080	13.700
120	delta	1	60x8 weeks	PCR90	Daily	NOVAC	0	All	4.279	0.005	0.091	0.096	0.010	0.095	0.003	2054.059	2.426	43.599	46.025	4.863	45.608	1.370	20541	24.260	435.990	460.250	48.630	456.080	13.700
120	delta	1	60x8 weeks	PCR90	Daily	NOVAC	0	All	4.279	0.005	0.091	0.096	0.010	0.095	0.003	2054.059	2.426	43.599	46.025	4.863	45.608	1.370	20541	24.260	435.990	460.250	48.630	456.080	13.700
120	delta	1.2	60x8 weeks	PCR90	Daily	NOVAC	0	All	4.177	0.005	0.103	0.108	0.011	0.108	0.003	2005.127	2.182	49.638	51.820	5.503	51.627	1.250	20051	21.820	496.380	518.200	55.030	516.270	12.500
120	delta	1.2	60x8 weeks	PCR90	Daily	NOVAC	0	All	4.177	0.005	0.103	0.108	0.011	0.108	0.003	2005.127	2.182	49.638	51.820	5.503	51.627	1.250	20051	21.820	496.380	518.200	55.030	516.270	12.500
120	delta	1.2	60x8 weeks	PCR90	Daily	NOVAC	0	All	4.177	0.005	0.103	0.108	0.011	0.108	0.003	2005.127	2.182	49.638	51.820	5.503	51.627	1.250	20051	21.820	496.380	518.200	55.030	516.270	12.500
120	original	0.6	60x8 weeks	PCR90	Daily	VAC95	25	All	4.948	0.006	0.001	0.008	0.001	0.007	0.005	2374.910	3.074	0.572	3.646	0.242	3.538	2.380	23749	30.740	5.720	36.460	2.420	35.380	23.800
120	original	0.6	60x8 weeks	PCR90	Daily	VAC95	25	All	4.948	0.006	0.001	0.008	0.001	0.007	0.005	2374.910	3.074	0.572	3.646	0.242	3.538	2.380	23749	30.740	5.720	36.460	2.420	35.380	23.800
120	original	0.6	60x8 weeks	PCR90	Daily	VAC95	25	All	4.948	0.006	0.001	0.008	0.001	0.007	0.005	2374.910	3.074	0.572	3.646	0.242	3.538	2.380	23749	30.740	5.720	36.460	2.420	35.380	23.800
120	original	1	60x8 weeks	PCR90	Daily	VAC95	25	All	4.944	0.006	0.002	0.008	0.001	0.008	0.005	2372.976	2.895	1.029	3.924	0.285	3.825	2.390	23730	28.950	10.290	39.240	2.850	38.250	23.900
120	original	1	60x8 weeks	PCR90	Daily	VAC95	25	All	4.944	0.006	0.002	0.008	0.001	0.008	0.005	2372.976	2.895	1.029	3.924	0.285	3.825	2.390	23730	28.950	10.290	39.240	2.850	38.250	23.900
120	original	1	60x8 weeks	PCR90	Daily	VAC95	25	All	4.944	0.006	0.002	0.008	0.001	0.008	0.005	2372.976	2.895	1.029	3.924	0.285	3.825	2.390	23730	28.950	10.290	39.240	2.850	38.250	23.900
120	original	1.2	60x8 weeks	PCR90	Daily	VAC95	25	All	4.938	0.006	0.003	0.009	0.001	0.009	0.005	2370.052	2.908	1.485	4.393	0.303	4.252	2.356	23701	29.080	14.850	43.930	3.030	42.520	23.560
120	original	1.2	60x8 weeks	PCR90	Daily	VAC95	25	All	4.938	0.006	0.003	0.009	0.001	0.009	0.005	2370.052	2.908	1.485	4.393	0.303	4.252	2.356	23701	29.080	14.850	43.930	3.030	42.520	23.560
120	original	1.2	60x8 weeks	PCR90	Daily	VAC95	25	All	4.938	0.006	0.003	0.009	0.001	0.009	0.005	2370.052	2.908	1.485	4.393	0.303	4.252	2.356	23701	29.080	14.850	43.930	3.030	42.520	23.560

120	alpha	0.6	60x8 weeks	PCR90	Daily	VAC95	25	All	4.940	0.006	0.003	0.008	0.001	0.008	0.005	2371.372	2.845	1.225	4.070	0.304	4.042	2.302	23714	28.450	12.250	40.700	3.040	40.420	23.020
120	alpha	0.6	60x8 weeks	PCR90	Daily	VAC95	25	All	4.940	0.006	0.003	0.008	0.001	0.008	0.005	2371.372	2.845	1.225	4.070	0.304	4.042	2.302	23714	28.450	12.250	40.700	3.040	40.420	23.020
120	alpha	0.6	60x8 weeks	PCR90	Daily	VAC95	25	All	4.940	0.006	0.003	0.008	0.001	0.008	0.005	2371.372	2.845	1.225	4.070	0.304	4.042	2.302	23714	28.450	12.250	40.700	3.040	40.420	23.020
120	alpha	1	60x8 weeks	PCR90	Daily	VAC95	25	All	4.910	0.006	0.006	0.013	0.001	0.013	0.005	2357.031	2.954	3.091	6.045	0.391	6.134	2.253	23570	29.540	30.910	60.450	3.910	61.340	22.530
120	alpha	1	60x8 weeks	PCR90	Daily	VAC95	25	All	4.910	0.006	0.006	0.013	0.001	0.013	0.005	2357.031	2.954	3.091	6.045	0.391	6.134	2.253	23570	29.540	30.910	60.450	3.910	61.340	22.530
120	alpha	1	60x8 weeks	PCR90	Daily	VAC95	25	All	4.910	0.006	0.006	0.013	0.001	0.013	0.005	2357.031	2.954	3.091	6.045	0.391	6.134	2.253	23570	29.540	30.910	60.450	3.910	61.340	22.530
120	alpha	1.2	60x8 weeks	PCR90	Daily	VAC95	25	All	4.894	0.006	0.009	0.015	0.001	0.015	0.005	2349.172	2.868	4.369	7.237	0.477	7.273	2.252	23492	28.680	43.690	72.370	4.770	72.730	22.520
120	alpha	1.2	60x8 weeks	PCR90	Daily	VAC95	25	All	4.894	0.006	0.009	0.015	0.001	0.015	0.005	2349.172	2.868	4.369	7.237	0.477	7.273	2.252	23492	28.680	43.690	72.370	4.770	72.730	22.520
120	alpha	1.2	60x8 weeks	PCR90	Daily	VAC95	25	All	4.894	0.006	0.009	0.015	0.001	0.015	0.005	2349.172	2.868	4.369	7.237	0.477	7.273	2.252	23492	28.680	43.690	72.370	4.770	72.730	22.520
120	delta	0.6	60x8 weeks	PCR90	Daily	VAC95	25	All	4.875	0.006	0.011	0.018	0.002	0.017	0.005	2340.091	2.954	5.447	8.401	0.835	8.192	2.228	23401	29.540	54.470	84.010	8.350	81.920	22.280
120	delta	0.6	60x8 weeks	PCR90	Daily	VAC95	25	All	4.875	0.006	0.011	0.018	0.002	0.017	0.005	2340.091	2.954	5.447	8.401	0.835	8.192	2.228	23401	29.540	54.470	84.010	8.350	81.920	22.280
120	delta	0.6	60x8 weeks	PCR90	Daily	VAC95	25	All	4.875	0.006	0.011	0.018	0.002	0.017	0.005	2340.091	2.954	5.447	8.401	0.835	8.192	2.228	23401	29.540	54.470	84.010	8.350	81.920	22.280
120	delta	1	60x8 weeks	PCR90	Daily	VAC95	25	All	4.700	0.005	0.037	0.042	0.004	0.041	0.004	2256.201	2.435	17.591	20.026	2.030	19.592	2.018	22562	24.350	175.910	200.260	20.300	195.920	20.180
120	delta	1	60x8 weeks	PCR90	Daily	VAC95	25	All	4.700	0.005	0.037	0.042	0.004	0.041	0.004	2256.201	2.435	17.591	20.026	2.030	19.592	2.018	22562	24.350	175.910	200.260	20.300	195.920	20.180
120	delta	1	60x8 weeks	PCR90	Daily	VAC95	25	All	4.700	0.005	0.037	0.042	0.004	0.041	0.004	2256.201	2.435	17.591	20.026	2.030	19.592	2.018	22562	24.350	175.910	200.260	20.300	195.920	20.180
120	delta	1.2	60x8 weeks	PCR90	Daily	VAC95	25	All	4.577	0.005	0.053	0.058	0.006	0.057	0.004	2196.984	2.282	25.660	27.942	2.954	27.276	1.858	21970	22.820	256.600	279.420	29.540	272.760	18.580
120	delta	1.2	60x8 weeks	PCR90	Daily	VAC95	25	All	4.577	0.005	0.053	0.058	0.006	0.057	0.004	2196.984	2.282	25.660	27.942	2.954	27.276	1.858	21970	22.820	256.600	279.420	29.540	272.760	18.580
120	delta	1.2	60x8 weeks	PCR90	Daily	VAC95	25	All	4.577	0.005	0.053	0.058	0.006	0.057	0.004	2196.984	2.282	25.660	27.942	2.954	27.276	1.858	21970	22.820	256.600	279.420	29.540	272.760	18.580
120	original	0.6	60x8 weeks	PCR90	Daily	VAC95	50	All	4.968	0.004	0.001	0.005	0.000	0.005	0.005	2384.428	2.110	0.241	2.351	0.204	2.164	2.313	23844	21.100	2.410	23.510	2.040	21.640	23.130
120	original	0.6	60x8 weeks	PCR90	Daily	VAC95	50	All	4.968	0.004	0.001	0.005	0.000	0.005	0.005	2384.428	2.110	0.241	2.351	0.204	2.164	2.313	23844	21.100	2.410	23.510	2.040	21.640	23.130
120	original	0.6	60x8 weeks	PCR90	Daily	VAC95	50	All	4.968	0.004	0.001	0.005	0.000	0.005	0.005	2384.428	2.110	0.241	2.351	0.204	2.164	2.313	23844	21.100	2.410	23.510	2.040	21.640	23.130
120	original	1	60x8 weeks	PCR90	Daily	VAC95	50	All	4.965	0.004	0.001	0.005	0.000	0.005	0.005	2383.200	2.056	0.480	2.536	0.190	2.355	2.276	23832	20.560	4.800	25.360	1.900	23.550	22.760
120	original	1	60x8 weeks	PCR90	Daily	VAC95	50	All	4.965	0.004	0.001	0.005	0.000	0.005	0.005	2383.200	2.056	0.480	2.536	0.190	2.355	2.276	23832	20.560	4.800	25.360	1.900	23.550	22.760
120	original	1	60x8 weeks	PCR90	Daily	VAC95	50	All	4.965	0.004	0.001	0.005	0.000	0.005	0.005	2383.200	2.056	0.480	2.536	0.190	2.355	2.276	23832	20.560	4.800	25.360	1.900	23.550	22.760
120	original	1.2	60x8 weeks	PCR90	Daily	VAC95	50	All	4.965	0.004	0.001	0.005	0.000	0.005	0.005	2383.232	2.002	0.501	2.503	0.187	2.337	2.278	23832	20.020	5.010	25.030	1.870	23.370	22.780
120	original	1.2	60x8 weeks	PCR90	Daily	VAC95	50	All	4.965	0.004	0.001	0.005	0.000	0.005	0.005	2383.232	2.002	0.501	2.503	0.187	2.337	2.278	23832	20.020	5.010	25.030	1.870	23.370	22.780
120	original	1.2	60x8 weeks	PCR90	Daily	VAC95	50	All	4.965	0.004	0.001	0.005	0.000	0.005	0.005	2383.232	2.002	0.501	2.503	0.187	2.337	2.278	23832	20.020	5.010	25.030	1.870	23.370	22.780
120	alpha	0.6	60x8 weeks	PCR90	Daily	VAC95	50	All	4.963	0.004	0.001	0.005	0.000	0.005	0.005	2382.077	2.009	0.540	2.549	0.171	2.546	2.308	23821	20.090	5.400	25.490	1.710	25.460	23.080
120	alpha	0.6	60x8 weeks	PCR90	Daily	VAC95	50	All	4.963	0.004	0.001	0.005	0.000	0.005	0.005	2382.077	2.009	0.540	2.549	0.171	2.546	2.308	23821	20.090	5.400	25.490	1.710	25.460	23.080
120	alpha	0.6	60x8 weeks	PCR90	Daily	VAC95	50	All	4.963	0.004	0.001	0.005	0.000	0.005	0.005	2382.077	2.009	0.540	2.549	0.171	2.546	2.308	23821	20.090	5.400	25.490	1.710	25.460	23.080
120	alpha	1	60x8 weeks	PCR90	Daily	VAC95	50	All	4.955	0.004	0.002	0.006	0.000	0.006	0.005	2378.561	2.056	1.013	3.069	0.223	3.055	2.405	23786	20.560	10.130	30.690	2.230	30.550	24.050
120	alpha	1	60x8 weeks	PCR90	Daily	VAC95	50	All	4.955	0.004	0.002	0.006	0.000	0.006	0.005	2378.561	2.056	1.013	3.069	0.223	3.055	2.405	23786	20.560	10.130	30.690	2.230	30.550	24.050
120	alpha	1	60x8 weeks	PCR90	Daily	VAC95	50	All	4.955	0.004	0.002	0.006	0.000	0.006	0.005	2378.561	2.056	1.013	3.069	0.223	3.055	2.405	23786	20.560	10.130	30.690	2.230	30.550	24.050
120	alpha	1.2	60x8 weeks	PCR90	Daily	VAC95	50	All	4.948	0.004	0.003	0.008	0.001	0.007	0.005	2374.972	2.110	1.521	3.631	0.257	3.593	2.382	23750	21.100	15.210	36.310	2.570	35.930	23.820
120	alpha	1.2	60x8 weeks	PCR90	Daily	VAC95	50	All	4.948	0.004	0.003	0.008	0.001	0.007	0.005	2374.972	2.110	1.521	3.631	0.257	3.593	2.382	23750	21.100	15.210	36.310	2.570	35.930	23.820
120	alpha	1.2	60x8 weeks	PCR90	Daily	VAC95	50	All	4.948	0.004	0.003	0.008	0.001	0.007	0.005	2374.972	2.110	1.521	3.631	0.257	3.593	2.382	23750	21.100	15.210	36.310	2.570	35.930	23.820
120	delta	0.6	60x8 weeks	PCR90	Daily	VAC95	50	All	4.939	0.005	0.004	0.009	0.001	0.008	0.005	2370.675	2.254	1.949	4.203	0.413	4.023	2.268	23707	22.540	19.490	42.030	4.130	40.230	22.680
120	delta	0.6	60x8 weeks	PCR90	Daily	VAC95	50	All	4.939	0.005	0.004	0.009	0.001	0.008	0.005	2370.675	2.254	1.949	4.203	0.413	4.023	2.268	23707	22.540	19.490	42.030	4.130	40.230	22.680
120	delta	0.6	60x8 weeks	PCR90	Daily	VAC95	50	All	4.939	0.005	0.004	0.009	0.001	0.008	0.005	2370.675	2.254	1.949	4.203	0.413	4.023	2.268	23707	22.540	19.490	42.030	4.130	40.230	22.680
120	delta	1	60x8 weeks	PCR90	Daily	VAC95	50	All	4.893	0.004	0.011	0.015	0.002	0.015	0.005	2348.494	2.130	5.170	7.300	0.725	7.040	2.298	23485	21.300	51.700	73.000	7.250	70.400	22.980
120	delta	1	60x8 weeks	PCR90	Daily	VAC95	50	All	4.893	0.004	0.011	0.015	0.002	0.015	0.005	2348.494	2.130	5.170	7.300	0.725	7.040	2.298	23485	21.300	51.700	73.000	7.250	70.400	22.980
120	delta	1	60x8 weeks	PCR90	Daily	VAC95	50	All	4.893	0.004	0.011	0.015	0.002	0.015	0.005	2348.494	2.130	5.170	7.300	0.725	7.040	2.298	23485	21.300	51.700	73.000	7.250	70.400	22.980
120	delta	1.2	60x8 weeks	PCR90	Daily	VAC95	50	All	4.866	0.004	0.015	0.019	0.002	0.019	0.005	2335.451	2.093	7.211	9.304	0.933	8.975	2.202	23355	20.930	72.110	93.040	9.330	89.750	22.020
120	delta	1.2	60x8 weeks	PCR90	Daily	VAC95	50	All	4.866	0.004	0.015	0.019	0.002	0.019	0.005	2335.451	2.093	7.211	9.304	0.933	8.975	2.202	23355	20.930	72.110	93.040	9.330	89.750	22.020
120	delta	1.2	60x8 weeks	PCR90	Daily	VAC95	50	All	4.866	0.004	0.015	0.019	0.002	0.019	0.005	2335.451	2.093	7.211	9.304	0.933	8.975	2.202	23355	20.930	72.110	93.040	9.330	89.750	22.020
120	original	0.6	60x8 weeks	PCR90	Daily	VAC95	75	All	4.983	0.003	0.000	0.003	0.000	0.002	0.005	2391.766	1.230	0.083	1.313	0.140	1.104	2.319	23918	12.300	0.830	13.130	1.400	11.040	23.190
120	original	0.6	60x8 weeks	PCR90	Daily	VAC95	75	All	4.983	0.003	0.000	0.003	0.000	0.002	0.005	2391.766	1.230	0.083	1.313	0.140	1.104	2.319	23918	12.300	0.830	13.130	1.400	11.040	23.190
120	original	0.6	60x8 weeks	PCR90	Daily	VAC95	75	All	4.983	0.003	0.000	0.003	0.000	0.002	0.005	2391.766	1.230	0.083	1.313	0.140	1.104	2.319	23918	12.300	0.830	13.130	1.400	11.040	23.190
120	original	1	60x8 weeks	PCR90	Daily	VAC95	75	All	4.982	0.002	0.000	0.003	0.000	0.002	0.005	2391.423	1.195	0.146	1.341	0.147	1.143	2.406	23914	11.950	1.460	13.410	1.470	11.430	24.060
120	original	1	60x8 weeks	PCR90	Daily	VAC95	75	All	4.982	0.002	0.000	0.003	0.000	0.002	0.005	2391.423	1.195	0.146	1.341	0.147	1.143	2.406	23914	11.950	1.460	13.410	1.470	11.430	24.060
120	original	1	60x8 weeks	PCR90	Daily	VAC95	75	All	4.982	0.002	0.000	0.003	0.000	0.002	0.005	2391.423	1.195	0.146	1.341	0.147	1.143	2.406	23914	11.950	1.460	13.410	1.470	11.430	24.060
120	original	1.2	60x8 weeks	PCR90	Daily	VAC95	75	All	4.983	0.002	0.000	0.003	0.000	0.002	0.005	2391.672	1.190	0.127	1.317	0.125	1.127	2.487	23917	11.900	1.270	13.170	1.250	11.270	24.870
120	original	1.2	60x8 weeks	PCR90	Daily	VAC95	75	All	4.983	0.002	0.000	0.003	0.000	0.002	0.005	2391.672	1.190	0.127	1.317	0.125	1.127	2.487	23917	11.900	1.270	13.170	1.250	11.270	24.870
120	original	1.2	60x8 weeks	PCR90	Daily	VAC95	75	All	4.983	0.002	0.000	0.003	0.000	0.002	0.005	2391.672	1.190	0.127	1.317	0.125	1.127	2.487	23917	11.900	1.270	13.170	1.250	11.270	24.870
120	alpha	0.6	60x8 weeks	PCR90	Daily	VAC95	75	All	4.979	0.003	0.000	0.003	0.000	0.003	0.005	2389.971	1.282	0.149	1.431	0.091	1.410	2.386	23900	12.820	1.490	14.310	0.910	14.100	23.860
120	alpha	0.6	60x8 weeks	PCR90	Daily	VAC95	75	All	4.979	0.003	0.000	0.003	0.000	0.003	0.005	2389.971	1.282	0.149	1.431	0.091	1.410	2.386	23900	12.820	1.490	14.310	0.910	14.100	23.860
120	alpha	0.6	60x8 weeks	PCR90	Daily	VAC95	75	All	4.979	0.003	0.000	0.003	0.000	0.003	0.005	2389.971	1.282	0.149	1.431	0.091	1.410	2.386	23900	12.820	1.490	14.310	0.910	14.100	23.860
120	alpha	1	60x8 weeks	PCR90	Daily	VAC95	75	All	4.979	0.002	0.001	0.003	0.000	0.003	0.005	2389.981	1.157	0.288	1.445	0.120	1.400	2.375	23900	11.570	2.880	14.450	1.200	14.000	23.750
120	alpha	1	60x8 weeks	PCR90	Daily	VAC95	75	All	4.979	0.002	0.001	0.003	0.000	0.003	0.005	2389.981	1.157	0.288	1.445	0.120	1.400	2.375	23900	11.570	2.880	14.450	1.200	14.000	23.750
120	alpha	1	60x8 weeks	PCR90	Daily	VAC95	75	All	4.979	0.002	0.001	0.003	0.000	0.003	0.005	2389.981	1.157	0.288	1.445	0.120	1.400	2.375	23900	11.570	2.880	14.450	1.200	14.000	23.750
120	alpha	1.2	60x8 weeks	PCR90	Daily	VAC95	75	All	4.976	0.003	0.001	0.003	0.000	0.003	0.005	2388.646	1.283	0.360	1.643	0.109	1.621	2.311	23886	12.830	3.600	16.430	1.090	16.210	23.110
120	alpha	1.2	60x8 weeks	PCR90	Daily	VAC95	75	All	4.976	0.003	0.001	0.003	0.000	0.003	0.005	2388.646	1.283	0.360	1.643	0.109	1.621	2.311	23886	12.830	3.600	16.430	1.090	16.210	23.110
120	alpha	1.2	60x8 weeks	PCR90	Daily	VAC95	75	All	4.976	0.003	0.001	0.003	0.000	0.003	0.005	2388.646	1.283	0.360	1.643	0.109	1.621	2.311	23886	12.830	3.600	16.430	1.090	16.210	23.110
120	delta	0.6	60x8 weeks	PCR90	Daily	VAC95	75	All	4.972	0.003	0.001	0.004	0.000	0.004	0.005	2386.509	1.418	0.531	1.949	0.180	1.892	2.311	23865	14.180	5.310	19.490	1.800	18.920	23.110
120	delta	0.6	60x8 weeks	PCR90	Daily	VAC95	75	All	4.972	0.003	0.001	0.004	0.000	0.004	0.005	2386.509	1.418	0.531	1.949	0.180	1.892	2.311	23865	14.180	5.310	19.490	1.800	18.920	23.110
120	delta	0.6	60x8 weeks	PCR90	Daily	VAC95	75	All	4.972	0.003	0.001	0.004	0.000	0.004	0.005	2386.509	1.418	0.531	1.949	0.180	1.892	2.311	23865	14.180	5.310	19.490	1.800	18.920	23.110
120	delta	1	60x8 weeks	PCR90	Daily	VAC95	75	All	4.962	0.003	0.002	0.005	0.000	0.005	0.005	2381.809	1.431	1.160	2.591	0.236	2.538	2.368	23818	14.310	11.600	25.910	2.360	25.380	23.680
120	delta	1	60x8 weeks	PCR90	Daily	VAC95	75	All	4.962	0.003	0.002	0.005	0.000	0.005	0.005	2381.809	1.431	1.160	2.591	0.236	2.538	2.368	23818	14.310	11.600	25.910	2.360	25.380	23.680
120	delta	1	60x8 weeks	PCR90	Daily	VAC95	75	All	4.962	0.003	0.002	0.005	0.000	0.005	0.005	2381.809	1.431	1.160	2.591	0.236	2.538	2.368	23818	14.310	11.600	25.910	2.360	25.380	23.680
120	delta	1.2	60x8 weeks	PCR90	Daily	VAC95	75	All	4.959	0.003	0.003	0.006	0.001	0.006	0.005	2380.525	1.394	1.377	2.771	0.257	2.682	2.344	23805	13.940	13.770	27.710	2.570	26.820	23.440
120	delta	1.2	60x8 weeks	PCR90	Daily	VAC95	75	All	4.959	0.003	0.003	0.006	0.001	0.006	0.005	2380.525	1.394	1.377	2.771	0.257	2.682	2.344	23805	13.940	13.770	27.710	2.570	26.820	23.440

120	delta	1.2	60x8 weeks	PCR90	Daily	VAC95	75	All	4.959	0.003	0.003	0.006	0.001	0.006	0.005	2380.525	1.394	1.377	2.771	0.257	2.682	2.344	23805	13.940	13.770	27.710	2.570	26.820	23.440
120	original	0.6	60x8 weeks	PCR90	Daily	VAC95	100	All	4.998	0.001	0.000	0.001	0.000	0.000	0.005	2399.023	0.279	0.000	0.279	0.094	0.058	2.402	23990	2.790	0.000	2.790	0.940	0.580	24.020
120	original	0.6	60x8 weeks	PCR90	Daily	VAC95	100	All	4.998	0.001	0.000	0.001	0.000	0.000	0.005	2399.023	0.279	0.000	0.279	0.094	0.058	2.402	23990	2.790	0.000	2.790	0.940	0.580	24.020
120	original	0.6	60x8 weeks	PCR90	Daily	VAC95	100	All	4.998	0.001	0.000	0.001	0.000	0.000	0.005	2399.023	0.279	0.000	0.279	0.094	0.058	2.402	23990	2.790	0.000	2.790	0.940	0.580	24.020
120	original	1	60x8 weeks	PCR90	Daily	VAC95	100	All	4.998	0.001	0.000	0.001	0.000	0.000	0.005	2399.152	0.270	0.000	0.270	0.080	0.056	2.438	23992	2.700	0.000	2.700	0.800	0.560	24.380
120	original	1	60x8 weeks	PCR90	Daily	VAC95	100	All	4.998	0.001	0.000	0.001	0.000	0.000	0.005	2399.152	0.270	0.000	0.270	0.080	0.056	2.438	23992	2.700	0.000	2.700	0.800	0.560	24.380
120	original	1	60x8 weeks	PCR90	Daily	VAC95	100	All	4.998	0.001	0.000	0.001	0.000	0.000	0.005	2399.152	0.270	0.000	0.270	0.080	0.056	2.438	23992	2.700	0.000	2.700	0.800	0.560	24.380
120	original	1.2	60x8 weeks	PCR90	Daily	VAC95	100	All	4.998	0.001	0.000	0.001	0.000	0.000	0.005	2398.944	0.314	0.001	0.315	0.083	0.079	2.475	23989	3.140	0.010	3.150	0.830	0.790	24.750
120	original	1.2	60x8 weeks	PCR90	Daily	VAC95	100	All	4.998	0.001	0.000	0.001	0.000	0.000	0.005	2398.944	0.314	0.001	0.315	0.083	0.079	2.475	23989	3.140	0.010	3.150	0.830	0.790	24.750
120	original	1.2	60x8 weeks	PCR90	Daily	VAC95	100	All	4.998	0.001	0.000	0.001	0.000	0.000	0.005	2398.944	0.314	0.001	0.315	0.083	0.079	2.475	23989	3.140	0.010	3.150	0.830	0.790	24.750
120	alpha	0.6	60x8 weeks	PCR90	Daily	VAC95	100	All	4.995	0.001	0.000	0.001	0.000	0.001	0.005	2397.489	0.369	0.012	0.381	0.030	0.357	2.404	23975	3.690	0.120	3.810	0.300	3.570	24.040
120	alpha	0.6	60x8 weeks	PCR90	Daily	VAC95	100	All	4.995	0.001	0.000	0.001	0.000	0.001	0.005	2397.489	0.369	0.012	0.381	0.030	0.357	2.404	23975	3.690	0.120	3.810	0.300	3.570	24.040
120	alpha	0.6	60x8 weeks	PCR90	Daily	VAC95	100	All	4.995	0.001	0.000	0.001	0.000	0.001	0.005	2397.489	0.369	0.012	0.381	0.030	0.357	2.404	23975	3.690	0.120	3.810	0.300	3.570	24.040
120	alpha	1	60x8 weeks	PCR90	Daily	VAC95	100	All	4.994	0.001	0.000	0.001	0.000	0.001	0.005	2397.313	0.394	0.018	0.412	0.018	0.395	2.332	23973	3.940	0.180	4.120	0.180	3.950	23.320
120	alpha	1	60x8 weeks	PCR90	Daily	VAC95	100	All	4.994	0.001	0.000	0.001	0.000	0.001	0.005	2397.313	0.394	0.018	0.412	0.018	0.395	2.332	23973	3.940	0.180	4.120	0.180	3.950	23.320
120	alpha	1	60x8 weeks	PCR90	Daily	VAC95	100	All	4.994	0.001	0.000	0.001	0.000	0.001	0.005	2397.313	0.394	0.018	0.412	0.018	0.395	2.332	23973	3.940	0.180	4.120	0.180	3.950	23.320
120	alpha	1.2	60x8 weeks	PCR90	Daily	VAC95	100	All	4.994	0.001	0.000	0.001	0.000	0.001	0.005	2397.312	0.369	0.014	0.383	0.024	0.383	2.351	23973	3.690	0.140	3.830	0.240	3.830	23.510
120	alpha	1.2	60x8 weeks	PCR90	Daily	VAC95	100	All	4.994	0.001	0.000	0.001	0.000	0.001	0.005	2397.312	0.369	0.014	0.383	0.024	0.383	2.351	23973	3.690	0.140	3.830	0.240	3.830	23.510
120	alpha	1.2	60x8 weeks	PCR90	Daily	VAC95	100	All	4.994	0.001	0.000	0.001	0.000	0.001	0.005	2397.312	0.369	0.014	0.383	0.024	0.383	2.351	23973	3.690	0.140	3.830	0.240	3.830	23.510
120	delta	0.6	60x8 weeks	PCR90	Daily	VAC95	100	All	4.992	0.001	0.000	0.001	0.000	0.001	0.005	2395.930	0.542	0.040	0.582	0.053	0.562	2.400	23959	5.420	0.400	5.820	0.530	5.620	24.000
120	delta	0.6	60x8 weeks	PCR90	Daily	VAC95	100	All	4.992	0.001	0.000	0.001	0.000	0.001	0.005	2395.930	0.542	0.040	0.582	0.053	0.562	2.400	23959	5.420	0.400	5.820	0.530	5.620	24.000
120	delta	0.6	60x8 weeks	PCR90	Daily	VAC95	100	All	4.992	0.001	0.000	0.001	0.000	0.001	0.005	2395.930	0.542	0.040	0.582	0.053	0.562	2.400	23959	5.420	0.400	5.820	0.530	5.620	24.000
120	delta	1	60x8 weeks	PCR90	Daily	VAC95	100	All	4.990	0.001	0.000	0.001	0.000	0.001	0.005	2395.439	0.604	0.072	0.676	0.064	0.646	2.433	23954	6.040	0.720	6.760	0.640	6.460	24.330
120	delta	1	60x8 weeks	PCR90	Daily	VAC95	100	All	4.990	0.001	0.000	0.001	0.000	0.001	0.005	2395.439	0.604	0.072	0.676	0.064	0.646	2.433	23954	6.040	0.720	6.760	0.640	6.460	24.330
120	delta	1	60x8 weeks	PCR90	Daily	VAC95	100	All	4.990	0.001	0.000	0.001	0.000	0.001	0.005	2395.439	0.604	0.072	0.676	0.064	0.646	2.433	23954	6.040	0.720	6.760	0.640	6.460	24.330
120	delta	1.2	60x8 weeks	PCR90	Daily	VAC95	100	All	4.991	0.001	0.000	0.001	0.000	0.001	0.005	2395.778	0.526	0.086	0.612	0.054	0.586	2.332	23958	5.260	0.860	6.120	0.540	5.860	23.320
120	delta	1.2	60x8 weeks	PCR90	Daily	VAC95	100	All	4.991	0.001	0.000	0.001	0.000	0.001	0.005	2395.778	0.526	0.086	0.612	0.054	0.586	2.332	23958	5.260	0.860	6.120	0.540	5.860	23.320
120	delta	1.2	60x8 weeks	PCR90	Daily	VAC95	100	All	4.991	0.001	0.000	0.001	0.000	0.001	0.005	2395.778	0.526	0.086	0.612	0.054	0.586	2.332	23958	5.260	0.860	6.120	0.540	5.860	23.320
120	original	0.6	60x8 weeks	PCR90	Daily	VAC70	25	All	4.946	0.007	0.001	0.008	0.001	0.008	0.005	2373.864	3.290	0.646	3.936	0.354	3.608	2.307	23739	32.900	6.460	39.360	3.540	36.080	23.070
120	original	0.6	60x8 weeks	PCR90	Daily	VAC70	25	All	4.946	0.007	0.001	0.008	0.001	0.008	0.005	2373.864	3.290	0.646	3.936	0.354	3.608	2.307	23739	32.900	6.460	39.360	3.540	36.080	23.070
120	original	0.6	60x8 weeks	PCR90	Daily	VAC70	25	All	4.946	0.007	0.001	0.008	0.001	0.008	0.005	2373.864	3.290	0.646	3.936	0.354	3.608	2.307	23739	32.900	6.460	39.360	3.540	36.080	23.070
120	original	1	60x8 weeks	PCR90	Daily	VAC70	25	All	4.938	0.007	0.003	0.010	0.001	0.009	0.005	2370.006	3.266	1.324	4.590	0.461	4.137	2.221	23700	32.660	13.240	45.900	4.610	41.370	22.210
120	original	1	60x8 weeks	PCR90	Daily	VAC70	25	All	4.938	0.007	0.003	0.010	0.001	0.009	0.005	2370.006	3.266	1.324	4.590	0.461	4.137	2.221	23700	32.660	13.240	45.900	4.610	41.370	22.210
120	original	1	60x8 weeks	PCR90	Daily	VAC70	25	All	4.938	0.007	0.003	0.010	0.001	0.009	0.005	2370.006	3.266	1.324	4.590	0.461	4.137	2.221	23700	32.660	13.240	45.900	4.610	41.370	22.210
120	original	1.2	60x8 weeks	PCR90	Daily	VAC70	25	All	4.936	0.007	0.003	0.010	0.001	0.009	0.005	2369.122	3.222	1.509	4.731	0.435	4.262	2.297	23691	32.220	15.090	47.310	4.350	42.620	22.970
120	original	1.2	60x8 weeks	PCR90	Daily	VAC70	25	All	4.936	0.007	0.003	0.010	0.001	0.009	0.005	2369.122	3.222	1.509	4.731	0.435	4.262	2.297	23691	32.220	15.090	47.310	4.350	42.620	22.970
120	original	1.2	60x8 weeks	PCR90	Daily	VAC70	25	All	4.936	0.007	0.003	0.010	0.001	0.009	0.005	2369.122	3.222	1.509	4.731	0.435	4.262	2.297	23691	32.220	15.090	47.310	4.350	42.620	22.970
120	alpha	0.6	60x8 weeks	PCR90	Daily	VAC70	25	All	4.929	0.007	0.003	0.010	0.001	0.010	0.005	2366.004	3.256	1.575	4.831	0.311	4.825	2.330	23660	32.560	15.750	48.310	3.110	48.250	23.300
120	alpha	0.6	60x8 weeks	PCR90	Daily	VAC70	25	All	4.929	0.007	0.003	0.010	0.001	0.010	0.005	2366.004	3.256	1.575	4.831	0.311	4.825	2.330	23660	32.560	15.750	48.310	3.110	48.250	23.300
120	alpha	0.6	60x8 weeks	PCR90	Daily	VAC70	25	All	4.929	0.007	0.003	0.010	0.001	0.010	0.005	2366.004	3.256	1.575	4.831	0.311	4.825	2.330	23660	32.560	15.750	48.310	3.110	48.250	23.300
120	alpha	1	60x8 weeks	PCR90	Daily	VAC70	25	All	4.901	0.007	0.007	0.014	0.001	0.014	0.005	2352.478	3.136	3.581	6.717	0.490	6.720	2.210	23525	31.360	35.810	67.170	4.900	67.200	22.100
120	alpha	1	60x8 weeks	PCR90	Daily	VAC70	25	All	4.901	0.007	0.007	0.014	0.001	0.014	0.005	2352.478	3.136	3.581	6.717	0.490	6.720	2.210	23525	31.360	35.810	67.170	4.900	67.200	22.100
120	alpha	1	60x8 weeks	PCR90	Daily	VAC70	25	All	4.901	0.007	0.007	0.014	0.001	0.014	0.005	2352.478	3.136	3.581	6.717	0.490	6.720	2.210	23525	31.360	35.810	67.170	4.900	67.200	22.100
120	alpha	1.2	60x8 weeks	PCR90	Daily	VAC70	25	All	4.879	0.007	0.010	0.017	0.001	0.017	0.005	2341.962	3.162	5.021	8.183	0.596	8.236	2.262	23420	31.620	50.210	81.830	5.960	82.360	22.620
120	alpha	1.2	60x8 weeks	PCR90	Daily	VAC70	25	All	4.879	0.007	0.010	0.017	0.001	0.017	0.005	2341.962	3.162	5.021	8.183	0.596	8.236	2.262	23420	31.620	50.210	81.830	5.960	82.360	22.620
120	alpha	1.2	60x8 weeks	PCR90	Daily	VAC70	25	All	4.879	0.007	0.010	0.017	0.001	0.017	0.005	2341.962	3.162	5.021	8.183	0.596	8.236	2.262	23420	31.620	50.210	81.830	5.960	82.360	22.620
120	delta	0.6	60x8 weeks	PCR90	Daily	VAC70	25	All	4.856	0.007	0.014	0.020	0.002	0.020	0.004	2330.853	3.165	6.599	9.764	0.936	9.498	2.097	23309	31.650	65.990	97.640	9.360	94.980	20.970
120	delta	0.6	60x8 weeks	PCR90	Daily	VAC70	25	All	4.856	0.007	0.014	0.020	0.002	0.020	0.004	2330.853	3.165	6.599	9.764	0.936	9.498	2.097	23309	31.650	65.990	97.640	9.360	94.980	20.970
120	delta	0.6	60x8 weeks	PCR90	Daily	VAC70	25	All	4.856	0.007	0.014	0.020	0.002	0.020	0.004	2330.853	3.165	6.599	9.764	0.936	9.498	2.097	23309	31.650	65.990	97.640	9.360	94.980	20.970
120	delta	1	60x8 weeks	PCR90	Daily	VAC70	25	All	4.594	0.006	0.050	0.056	0.006	0.054	0.004	2205.066	2.656	24.002	26.658	2.782	26.147	1.860	22051	26.560	240.020	266.580	27.820	261.470	18.600
120	delta	1	60x8 weeks	PCR90	Daily	VAC70	25	All	4.594	0.006	0.050	0.056	0.006	0.054	0.004	2205.066	2.656	24.002	26.658	2.782	26.147	1.860	22051	26.560	240.020	266.580	27.820	261.470	18.600
120	delta	1	60x8 weeks	PCR90	Daily	VAC70	25	All	4.594	0.006	0.050	0.056	0.006	0.054	0.004	2205.066	2.656	24.002	26.658	2.782	26.147	1.860	22051	26.560	240.020	266.580	27.820	261.470	18.600
120	delta	1.2	60x8 weeks	PCR90	Daily	VAC70	25	All	4.446	0.005	0.070	0.075	0.008	0.074	0.003	2133.996	2.290	33.738	36.028	3.748	35.434	1.639	21340	22.900	337.380	360.280	37.480	354.340	16.390
120	delta	1.2	60x8 weeks	PCR90	Daily	VAC70	25	All	4.446	0.005	0.070	0.075	0.008	0.074	0.003	2133.996	2.290	33.738	36.028	3.748	35.434	1.639	21340	22.900	337.380	360.280	37.480	354.340	16.390
120	delta	1.2	60x8 weeks	PCR90	Daily	VAC70	25	All	4.446	0.005	0.070	0.075	0.008	0.074	0.003	2133.996	2.290	33.738	36.028	3.748	35.434	1.639	21340	22.900	337.380	360.280	37.480	354.340	16.390
120	original	0.6	60x8 weeks	PCR90	Daily	VAC70	50	All	4.963	0.005	0.001	0.006	0.001	0.005	0.005	2382.288	2.576	0.323	2.899	0.399	2.319	2.305	23823	25.760	3.230	28.990	3.990	23.190	23.050
120	original	0.6	60x8 weeks	PCR90	Daily	VAC70	50	All	4.963	0.005	0.001	0.006	0.001	0.005	0.005	2382.288	2.576	0.323	2.899	0.399	2.319	2.305	23823	25.760	3.230	28.990	3.990	23.190	23.050
120	original	0.6	60x8 weeks	PCR90	Daily	VAC70	50	All	4.963	0.005	0.001	0.006	0.001	0.005	0.005	2382.288	2.576	0.323	2.899	0.399	2.319	2.305	23823	25.760	3.230	28.990	3.990	23.190	23.050
120	original	1	60x8 weeks	PCR90	Daily	VAC70	50	All	4.957	0.006	0.001	0.007	0.001	0.006	0.005	2379.482	2.640	0.617	3.257	0.443	2.646	2.262	23795	26.400	6.170	32.570	4.430	26.460	22.620
120	original	1	60x8 weeks	PCR90	Daily	VAC70	50	All	4.957	0.006	0.001	0.007	0.001	0.006	0.005	2379.482	2.640	0.617	3.257	0.443	2.646	2.262	23795	26.400	6.170	32.570	4.430	26.460	22.620
120	original	1	60x8 weeks	PCR90	Daily	VAC70	50	All	4.957	0.006	0.001	0.007	0.001	0.006	0.005	2379.482	2.640	0.617	3.257	0.443	2.646	2.262	23795	26.400	6.170	32.570	4.430	26.460	22.620
120	original	1.2	60x8 weeks	PCR90	Daily	VAC70	50	All	4.959	0.005	0.001	0.007	0.001	0.005	0.005	2380.503	2.542	0.683	3.225	0.420	2.541	2.323	23805	25.420	6.830	32.250	4.200	25.410	23.230
120	original	1.2	60x8 weeks	PCR90	Daily	VAC70	50	All	4.959	0.005	0.001	0.007	0.001	0.005	0.005	2380.503	2.542	0.683	3.225	0.420	2.541	2.323	23805	25.420	6.830	32.250	4.200	25.410	23.230
120	original	1.2	60x8 weeks	PCR90	Daily	VAC70	50	All	4.959	0.005	0.001	0.007	0.001	0.005	0.005	2380.503	2.542	0.683	3.225	0.420	2.541	2.323	23805	25.420	6.830	32.250	4.200	25.410	23.230
120	alpha	0.6	60x8 weeks	PCR90	Daily	VAC70	50	All	4.949	0.005	0.002	0.007	0.001	0.007	0.005	2375.572	2.537	0.889	3.426	0.262	3.429	2.364	23756	25.370	8.890	34.260	2.620	34.290	23.640
120	alpha	0.6	60x8 weeks	PCR90	Daily	VAC70	50	All	4.949	0.005	0.002	0.007	0.001	0.007	0.005	2375.572	2.537	0.889	3.426	0.262	3.429	2.364	23756	25.370	8.890	34.260	2.620	34.290	23.640
120	alpha	0.6	60x8 weeks	PCR90	Daily	VAC70	50	All	4.949	0.005	0.002	0.007	0.001	0.007	0.005	2375.572	2.537	0.889	3.426	0.262	3.429	2.364	23756	25.370	8.890	34.260	2.620	34.290	23.640
120	alpha	1	60x8 weeks	PCR90	Daily	VAC70	50	All	4.942	0.005	0.003	0.008	0.001	0.008	0.005	2371.953	2.501	1.531	4.032	0.287	3.990	2.312	23720	25.010	15.310	40.320	2.870	39.900	23.120
120	alpha	1	60x8 weeks	PCR90	Daily	VAC70	50	All	4.942	0.005	0.003	0.008	0.001	0.008	0.005	2371.953	2.501	1.531	4.032	0.287	3.990	2.312	23720	25.010	15.310	40.320	2.870	39.900	23.120
120	alpha	1	60x8 weeks	PCR90	Daily	VAC70	50	All	4.942	0.005	0.003	0.008	0.001	0.008	0.005	2371.953	2.501	1.531	4.032	0.287	3.990	2.312	23720	25.010	15.310	40.320	2.870	39.900	23.120
120	alpha	1.2	60x8 weeks	PCR90	Daily	VAC70	50	All	4.929	0.005	0.005	0.010	0.001	0.010	0.005	2365.820	2.547	2.328	4.875	0.366	4.852	2.260	23658	25.470	23.280	48.750	3.660	48.520	22.600

120	alpha	1.2	60x8 weeks	PCR90	Daily	VAC70	50	All	4.929	0.005	0.005	0.010	0.001	0.010	0.005	2365.820	2.547	2.328	4.875	0.366	4.852	2.260	23658	25.470	23.280	48.750	3.660	48.520	22.600
120	alpha	1.2	60x8 weeks	PCR90	Daily	VAC70	50	All	4.929	0.005	0.005	0.010	0.001	0.010	0.005	2365.820	2.547	2.328	4.875	0.366	4.852	2.260	23658	25.470	23.280	48.750	3.660	48.520	22.600
120	delta	0.6	60x8 weeks	PCR90	Daily	VAC70	50	All	4.911	0.006	0.007	0.013	0.001	0.012	0.005	2357.455	2.808	3.209	6.017	0.612	5.804	2.297	23575	28.080	32.090	60.170	6.120	58.040	22.970
120	delta	0.6	60x8 weeks	PCR90	Daily	VAC70	50	All	4.911	0.006	0.007	0.013	0.001	0.012	0.005	2357.455	2.808	3.209	6.017	0.612	5.804	2.297	23575	28.080	32.090	60.170	6.120	58.040	22.970
120	delta	0.6	60x8 weeks	PCR90	Daily	VAC70	50	All	4.911	0.006	0.007	0.013	0.001	0.012	0.005	2357.455	2.808	3.209	6.017	0.612	5.804	2.297	23575	28.080	32.090	60.170	6.120	58.040	22.970
120	delta	1	60x8 weeks	PCR90	Daily	VAC70	50	All	4.825	0.005	0.019	0.025	0.002	0.024	0.005	2315.982	2.501	9.268	11.769	1.193	11.523	2.176	23160	25.010	92.680	117.690	11.930	115.230	21.760
120	delta	1	60x8 weeks	PCR90	Daily	VAC70	50	All	4.825	0.005	0.019	0.025	0.002	0.024	0.005	2315.982	2.501	9.268	11.769	1.193	11.523	2.176	23160	25.010	92.680	117.690	11.930	115.230	21.760
120	delta	1	60x8 weeks	PCR90	Daily	VAC70	50	All	4.825	0.005	0.019	0.025	0.002	0.024	0.005	2315.982	2.501	9.268	11.769	1.193	11.523	2.176	23160	25.010	92.680	117.690	11.930	115.230	21.760
120	delta	1.2	60x8 weeks	PCR90	Daily	VAC70	50	All	4.733	0.005	0.032	0.037	0.004	0.036	0.004	2271.742	2.397	15.324	17.721	1.789	17.370	2.005	22717	23.970	153.240	177.210	17.890	173.700	20.050
120	delta	1.2	60x8 weeks	PCR90	Daily	VAC70	50	All	4.733	0.005	0.032	0.037	0.004	0.036	0.004	2271.742	2.397	15.324	17.721	1.789	17.370	2.005	22717	23.970	153.240	177.210	17.890	173.700	20.050
120	delta	1.2	60x8 weeks	PCR90	Daily	VAC70	50	All	4.733	0.005	0.032	0.037	0.004	0.036	0.004	2271.742	2.397	15.324	17.721	1.789	17.370	2.005	22717	23.970	153.240	177.210	17.890	173.700	20.050
120	original	0.6	60x8 weeks	PCR90	Daily	VAC70	75	All	4.977	0.004	0.000	0.004	0.001	0.003	0.005	2389.058	1.923	0.118	2.041	0.468	1.205	2.336	23891	19.230	1.180	20.410	4.680	12.050	23.360
120	original	0.6	60x8 weeks	PCR90	Daily	VAC70	75	All	4.977	0.004	0.000	0.004	0.001	0.003	0.005	2389.058	1.923	0.118	2.041	0.468	1.205	2.336	23891	19.230	1.180	20.410	4.680	12.050	23.360
120	original	0.6	60x8 weeks	PCR90	Daily	VAC70	75	All	4.977	0.004	0.000	0.004	0.001	0.003	0.005	2389.058	1.923	0.118	2.041	0.468	1.205	2.336	23891	19.230	1.180	20.410	4.680	12.050	23.360
120	original	1	60x8 weeks	PCR90	Daily	VAC70	75	All	4.977	0.004	0.000	0.004	0.001	0.003	0.005	2388.749	1.860	0.223	2.083	0.435	1.274	2.353	23887	18.600	2.230	20.830	4.350	12.740	23.530
120	original	1	60x8 weeks	PCR90	Daily	VAC70	75	All	4.977	0.004	0.000	0.004	0.001	0.003	0.005	2388.749	1.860	0.223	2.083	0.435	1.274	2.353	23887	18.600	2.230	20.830	4.350	12.740	23.530
120	original	1	60x8 weeks	PCR90	Daily	VAC70	75	All	4.977	0.004	0.000	0.004	0.001	0.003	0.005	2388.749	1.860	0.223	2.083	0.435	1.274	2.353	23887	18.600	2.230	20.830	4.350	12.740	23.530
120	original	1.2	60x8 weeks	PCR90	Daily	VAC70	75	All	4.975	0.004	0.001	0.005	0.001	0.003	0.005	2388.046	1.986	0.292	2.278	0.505	1.341	2.459	23880	19.860	2.920	22.780	5.050	13.410	24.590
120	original	1.2	60x8 weeks	PCR90	Daily	VAC70	75	All	4.975	0.004	0.001	0.005	0.001	0.003	0.005	2388.046	1.986	0.292	2.278	0.505	1.341	2.459	23880	19.860	2.920	22.780	5.050	13.410	24.590
120	original	1.2	60x8 weeks	PCR90	Daily	VAC70	75	All	4.975	0.004	0.001	0.005	0.001	0.003	0.005	2388.046	1.986	0.292	2.278	0.505	1.341	2.459	23880	19.860	2.920	22.780	5.050	13.410	24.590
120	alpha	0.6	60x8 weeks	PCR90	Daily	VAC70	75	All	4.966	0.004	0.001	0.005	0.000	0.005	0.005	2383.852	1.918	0.356	2.274	0.163	2.261	2.304	23839	19.180	3.560	22.740	1.630	22.610	23.040
120	alpha	0.6	60x8 weeks	PCR90	Daily	VAC70	75	All	4.966	0.004	0.001	0.005	0.000	0.005	0.005	2383.852	1.918	0.356	2.274	0.163	2.261	2.304	23839	19.180	3.560	22.740	1.630	22.610	23.040
120	alpha	0.6	60x8 weeks	PCR90	Daily	VAC70	75	All	4.966	0.004	0.001	0.005	0.000	0.005	0.005	2383.852	1.918	0.356	2.274	0.163	2.261	2.304	23839	19.180	3.560	22.740	1.630	22.610	23.040
120	alpha	1	60x8 weeks	PCR90	Daily	VAC70	75	All	4.962	0.004	0.001	0.005	0.000	0.005	0.005	2381.945	1.922	0.663	2.585	0.193	2.531	2.320	23819	19.220	6.630	25.850	1.930	25.310	23.200
120	alpha	1	60x8 weeks	PCR90	Daily	VAC70	75	All	4.962	0.004	0.001	0.005	0.000	0.005	0.005	2381.945	1.922	0.663	2.585	0.193	2.531	2.320	23819	19.220	6.630	25.850	1.930	25.310	23.200
120	alpha	1	60x8 weeks	PCR90	Daily	VAC70	75	All	4.962	0.004	0.001	0.005	0.000	0.005	0.005	2381.945	1.922	0.663	2.585	0.193	2.531	2.320	23819	19.220	6.630	25.850	1.930	25.310	23.200
120	alpha	1.2	60x8 weeks	PCR90	Daily	VAC70	75	All	4.959	0.004	0.002	0.006	0.000	0.006	0.005	2380.177	1.927	0.974	2.901	0.204	2.845	2.285	23802	19.270	9.740	29.010	2.040	28.450	22.850
120	alpha	1.2	60x8 weeks	PCR90	Daily	VAC70	75	All	4.959	0.004	0.002	0.006	0.000	0.006	0.005	2380.177	1.927	0.974	2.901	0.204	2.845	2.285	23802	19.270	9.740	29.010	2.040	28.450	22.850
120	alpha	1.2	60x8 weeks	PCR90	Daily	VAC70	75	All	4.959	0.004	0.002	0.006	0.000	0.006	0.005	2380.177	1.927	0.974	2.901	0.204	2.845	2.285	23802	19.270	9.740	29.010	2.040	28.450	22.850
120	delta	0.6	60x8 weeks	PCR90	Daily	VAC70	75	All	4.950	0.005	0.003	0.007	0.001	0.007	0.005	2375.940	2.188	1.284	3.472	0.344	3.302	2.317	23759	21.880	12.840	34.720	3.440	33.020	23.170
120	delta	0.6	60x8 weeks	PCR90	Daily	VAC70	75	All	4.950	0.005	0.003	0.007	0.001	0.007	0.005	2375.940	2.188	1.284	3.472	0.344	3.302	2.317	23759	21.880	12.840	34.720	3.440	33.020	23.170
120	delta	0.6	60x8 weeks	PCR90	Daily	VAC70	75	All	4.950	0.005	0.003	0.007	0.001	0.007	0.005	2375.940	2.188	1.284	3.472	0.344	3.302	2.317	23759	21.880	12.840	34.720	3.440	33.020	23.170
120	delta	1	60x8 weeks	PCR90	Daily	VAC70	75	All	4.927	0.005	0.006	0.011	0.001	0.010	0.005	2364.760	2.168	2.919	5.087	0.491	4.919	2.383	23648	21.680	29.190	50.870	4.910	49.190	23.830
120	delta	1	60x8 weeks	PCR90	Daily	VAC70	75	All	4.927	0.005	0.006	0.011	0.001	0.010	0.005	2364.760	2.168	2.919	5.087	0.491	4.919	2.383	23648	21.680	29.190	50.870	4.910	49.190	23.830
120	delta	1	60x8 weeks	PCR90	Daily	VAC70	75	All	4.927	0.005	0.006	0.011	0.001	0.010	0.005	2364.760	2.168	2.919	5.087	0.491	4.919	2.383	23648	21.680	29.190	50.870	4.910	49.190	23.830
120	delta	1.2	60x8 weeks	PCR90	Daily	VAC70	75	All	4.900	0.005	0.010	0.014	0.001	0.014	0.005	2352.032	2.209	4.686	6.895	0.708	6.608	2.256	23520	22.090	46.860	68.950	7.080	66.080	22.560
120	delta	1.2	60x8 weeks	PCR90	Daily	VAC70	75	All	4.900	0.005	0.010	0.014	0.001	0.014	0.005	2352.032	2.209	4.686	6.895	0.708	6.608	2.256	23520	22.090	46.860	68.950	7.080	66.080	22.560
120	delta	1.2	60x8 weeks	PCR90	Daily	VAC70	75	All	4.900	0.005	0.010	0.014	0.001	0.014	0.005	2352.032	2.209	4.686	6.895	0.708	6.608	2.256	23520	22.090	46.860	68.950	7.080	66.080	22.560
120	original	0.6	60x8 weeks	PCR90	Daily	VAC70	100	All	4.989	0.003	0.000	0.003	0.001	0.001	0.005	2394.638	1.373	0.009	1.382	0.514	0.311	2.434	23946	13.730	0.090	13.820	5.140	3.110	24.340
120	original	0.6	60x8 weeks	PCR90	Daily	VAC70	100	All	4.989	0.003	0.000	0.003	0.001	0.001	0.005	2394.638	1.373	0.009	1.382	0.514	0.311	2.434	23946	13.730	0.090	13.820	5.140	3.110	24.340
120	original	0.6	60x8 weeks	PCR90	Daily	VAC70	100	All	4.989	0.003	0.000	0.003	0.001	0.001	0.005	2394.638	1.373	0.009	1.382	0.514	0.311	2.434	23946	13.730	0.090	13.820	5.140	3.110	24.340
120	original	1	60x8 weeks	PCR90	Daily	VAC70	100	All	4.990	0.003	0.000	0.003	0.001	0.001	0.005	2395.102	1.268	0.023	1.291	0.477	0.280	2.374	23951	12.680	0.230	12.910	4.770	2.800	23.740
120	original	1	60x8 weeks	PCR90	Daily	VAC70	100	All	4.990	0.003	0.000	0.003	0.001	0.001	0.005	2395.102	1.268	0.023	1.291	0.477	0.280	2.374	23951	12.680	0.230	12.910	4.770	2.800	23.740
120	original	1	60x8 weeks	PCR90	Daily	VAC70	100	All	4.990	0.003	0.000	0.003	0.001	0.001	0.005	2395.102	1.268	0.023	1.291	0.477	0.280	2.374	23951	12.680	0.230	12.910	4.770	2.800	23.740
120	original	1.2	60x8 weeks	PCR90	Daily	VAC70	100	All	4.989	0.003	0.000	0.003	0.001	0.001	0.005	2394.886	1.369	0.017	1.386	0.524	0.277	2.406	23949	13.690	0.170	13.860	5.240	2.770	24.060
120	original	1.2	60x8 weeks	PCR90	Daily	VAC70	100	All	4.989	0.003	0.000	0.003	0.001	0.001	0.005	2394.886	1.369	0.017	1.386	0.524	0.277	2.406	23949	13.690	0.170	13.860	5.240	2.770	24.060
120	original	1.2	60x8 weeks	PCR90	Daily	VAC70	100	All	4.989	0.003	0.000	0.003	0.001	0.001	0.005	2394.886	1.369	0.017	1.386	0.524	0.277	2.406	23949	13.690	0.170	13.860	5.240	2.770	24.060
120	alpha	0.6	60x8 weeks	PCR90	Daily	VAC70	100	All	4.979	0.003	0.000	0.003	0.000	0.003	0.005	2390.048	1.297	0.118	1.415	0.112	1.383	2.384	23900	12.970	1.180	14.150	1.120	13.830	23.840
120	alpha	0.6	60x8 weeks	PCR90	Daily	VAC70	100	All	4.979	0.003	0.000	0.003	0.000	0.003	0.005	2390.048	1.297	0.118	1.415	0.112	1.383	2.384	23900	12.970	1.180	14.150	1.120	13.830	23.840
120	alpha	0.6	60x8 weeks	PCR90	Daily	VAC70	100	All	4.979	0.003	0.000	0.003	0.000	0.003	0.005	2390.048	1.297	0.118	1.415	0.112	1.383	2.384	23900	12.970	1.180	14.150	1.120	13.830	23.840
120	alpha	1	60x8 weeks	PCR90	Daily	VAC70	100	All	4.979	0.003	0.000	0.003	0.000	0.003	0.005	2389.998	1.313	0.194	1.507	0.120	1.415	2.412	23900	13.130	1.940	15.070	1.200	14.150	24.120
120	alpha	1	60x8 weeks	PCR90	Daily	VAC70	100	All	4.979	0.003	0.000	0.003	0.000	0.003	0.005	2389.998	1.313	0.194	1.507	0.120	1.415	2.412	23900	13.130	1.940	15.070	1.200	14.150	24.120
120	alpha	1	60x8 weeks	PCR90	Daily	VAC70	100	All	4.979	0.003	0.000	0.003	0.000	0.003	0.005	2389.998	1.313	0.194	1.507	0.120	1.415	2.412	23900	13.130	1.940	15.070	1.200	14.150	24.120
120	alpha	1.2	60x8 weeks	PCR90	Daily	VAC70	100	All	4.979	0.003	0.000	0.003	0.000	0.003	0.005	2389.752	1.274	0.223	1.497	0.111	1.450	2.433	23898	12.740	2.230	14.970	1.110	14.500	24.330
120	alpha	1.2	60x8 weeks	PCR90	Daily	VAC70	100	All	4.979	0.003	0.000	0.003	0.000	0.003	0.005	2389.752	1.274	0.223	1.497	0.111	1.450	2.433	23898	12.740	2.230	14.970	1.110	14.500	24.330
120	alpha	1.2	60x8 weeks	PCR90	Daily	VAC70	100	All	4.979	0.003	0.000	0.003	0.000	0.003	0.005	2389.752	1.274	0.223	1.497	0.111	1.450	2.433	23898	12.740	2.230	14.970	1.110	14.500	24.330
120	delta	0.6	60x8 weeks	PCR90	Daily	VAC70	100	All	4.970	0.004	0.001	0.004	0.000	0.004	0.005	2385.559	1.685	0.438	2.123	0.200	1.992	2.428	23856	16.850	4.380	21.230	2.000	19.920	24.280
120	delta	0.6	60x8 weeks	PCR90	Daily	VAC70	100	All	4.970	0.004	0.001	0.004	0.000	0.004	0.005	2385.559	1.685	0.438	2.123	0.200	1.992	2.428	23856	16.850	4.380	21.230	2.000	19.920	24.280
120	delta	0.6	60x8 weeks	PCR90	Daily	VAC70	100	All	4.970	0.004	0.001	0.004	0.000	0.004	0.005	2385.559	1.685	0.438	2.123	0.200	1.992	2.428	23856	16.850	4.380	21.230	2.000	19.920	24.280
120	delta	1	60x8 weeks	PCR90	Daily	VAC70	100	All	4.965	0.003	0.002	0.005	0.000	0.005	0.005	2383.397	1.565	0.800	2.365	0.233	2.281	2.409	23834	15.650	8.000	23.650	2.330	22.810	24.090
120	delta	1	60x8 weeks	PCR90	Daily	VAC70	100	All	4.965	0.003	0.002	0.005	0.000	0.005	0.005	2383.397	1.565	0.800	2.365	0.233	2.281	2.409	23834	15.650	8.000	23.650	2.330	22.810	24.090
120	delta	1	60x8 weeks	PCR90	Daily	VAC70	100	All	4.965	0.003	0.002	0.005	0.000	0.005	0.005	2383.397	1.565	0.800	2.365	0.233	2.281	2.409	23834	15.650	8.000	23.650	2.330	22.810	24.090
120	delta	1.2	60x8 weeks	PCR90	Daily	VAC70	100	All	4.958	0.004	0.002	0.006	0.001	0.006	0.005	2380.054	1.708	1.185	2.893	0.269	2.807	2.353	23801	17.080	11.850	28.930	2.690	28.070	23.530
120	delta	1.2	60x8 weeks	PCR90	Daily	VAC70	100	All	4.958	0.004	0.002	0.006	0.001	0.006	0.005	2380.054	1.708	1.185	2.893	0.269	2.807	2.353	23801	17.080	11.850	28.930	2.690	28.070	23.530
120	delta	1.2	60x8 weeks	PCR90	Daily	VAC70	100	All	4.958	0.004	0.002	0.006	0.001	0.006	0.005	2380.054	1.708	1.185	2.893	0.269	2.807	2.353	23801	17.080	11.850	28.930	2.690	28.070	23.530
120	original	0.6	60x8 weeks	PCR90	Daily	VAC50	25	All	4.946	0.007	0.001	0.009	0.001	0.007	0.005	2374.056	3.454	0.629	4.083	0.470	3.503	2.285	23741	34.540	6.290	40.830	4.700	35.030	22.850
120	original	0.6	60x8 weeks	PCR90	Daily	VAC50	25	All	4.946	0.007	0.001	0.009	0.001	0.007	0.005	2374.056	3.454	0.629	4.083	0.470	3.503	2.285	23741	34.540	6.290	40.830	4.700	35.030	22.850
120	original	0.6	60x8 weeks	PCR90	Daily	VAC50	25	All	4.946	0.007	0.001	0.009	0.001	0.007	0.005	2374.056	3.454	0.629	4.083	0.470	3.503	2.285	23741	34.540	6.290	40.830	4.700	35.030	22.850
120	original	1	60x8 weeks	PCR90	Daily	VAC50	25	All	4.938	0.007	0.003	0.010	0.001	0.008	0.005	2370.307	3.362	1.300	4.662	0.552	3.996	2.349	23703	33.620	13.000	46.620	5.520	39.960	23.490
120	original	1	60x8 weeks	PCR90	Daily	VAC50	25	All	4.938	0.007	0.003	0.010	0.001	0.008	0.005	2370.307	3.362	1.300	4.662	0.552	3.996	2.349	23703	33.620	13.000	46.620	5.520	39.960	23.490
120	original	1	60x8 weeks	PCR90	Daily	VAC50	25	All	4.938	0.007	0.003	0.010	0.001	0.008	0.005	2370.307	3.362	1.300	4.662	0.552	3.996	2.349	23703	33.620	13.000	46.620	5.520	39.960	23.490

120	original	1.2	60x8 weeks	PCR90	Daily	VAC50	25	All	4.932	0.007	0.003	0.010	0.001	0.009	0.005	2367.553	3.429	1.562	4.991	0.568	4.355	2.328	23676	34.290	15.620	49.910	5.680	43.550	23.280
120	original	1.2	60x8 weeks	PCR90	Daily	VAC50	25	All	4.932	0.007	0.003	0.010	0.001	0.009	0.005	2367.553	3.429	1.562	4.991	0.568	4.355	2.328	23676	34.290	15.620	49.910	5.680	43.550	23.280
120	original	1.2	60x8 weeks	PCR90	Daily	VAC50	25	All	4.932	0.007	0.003	0.010	0.001	0.009	0.005	2367.553	3.429	1.562	4.991	0.568	4.355	2.328	23676	34.290	15.620	49.910	5.680	43.550	23.280
120	alpha	0.6	60x8 weeks	PCR90	Daily	VAC50	25	All	4.923	0.007	0.004	0.011	0.001	0.011	0.005	2363.273	3.421	1.822	5.243	0.354	5.229	2.301	23633	34.210	18.220	52.430	3.540	52.290	23.010
120	alpha	0.6	60x8 weeks	PCR90	Daily	VAC50	25	All	4.923	0.007	0.004	0.011	0.001	0.011	0.005	2363.273	3.421	1.822	5.243	0.354	5.229	2.301	23633	34.210	18.220	52.430	3.540	52.290	23.010
120	alpha	0.6	60x8 weeks	PCR90	Daily	VAC50	25	All	4.923	0.007	0.004	0.011	0.001	0.011	0.005	2363.273	3.421	1.822	5.243	0.354	5.229	2.301	23633	34.210	18.220	52.430	3.540	52.290	23.010
120	alpha	1	60x8 weeks	PCR90	Daily	VAC50	25	All	4.895	0.007	0.008	0.015	0.001	0.015	0.005	2349.507	3.162	3.998	7.160	0.511	7.136	2.298	23495	31.620	39.980	71.600	5.110	71.360	22.980
120	alpha	1	60x8 weeks	PCR90	Daily	VAC50	25	All	4.895	0.007	0.008	0.015	0.001	0.015	0.005	2349.507	3.162	3.998	7.160	0.511	7.136	2.298	23495	31.620	39.980	71.600	5.110	71.360	22.980
120	alpha	1	60x8 weeks	PCR90	Daily	VAC50	25	All	4.895	0.007	0.008	0.015	0.001	0.015	0.005	2349.507	3.162	3.998	7.160	0.511	7.136	2.298	23495	31.620	39.980	71.600	5.110	71.360	22.980
120	alpha	1.2	60x8 weeks	PCR90	Daily	VAC50	25	All	4.860	0.007	0.013	0.020	0.001	0.020	0.005	2332.978	3.340	6.025	9.365	0.656	9.448	2.249	23330	33.400	60.250	93.650	6.560	94.480	22.490
120	alpha	1.2	60x8 weeks	PCR90	Daily	VAC50	25	All	4.860	0.007	0.013	0.020	0.001	0.020	0.005	2332.978	3.340	6.025	9.365	0.656	9.448	2.249	23330	33.400	60.250	93.650	6.560	94.480	22.490
120	alpha	1.2	60x8 weeks	PCR90	Daily	VAC50	25	All	4.860	0.007	0.013	0.020	0.001	0.020	0.005	2332.978	3.340	6.025	9.365	0.656	9.448	2.249	23330	33.400	60.250	93.650	6.560	94.480	22.490
120	delta	0.6	60x8 weeks	PCR90	Daily	VAC50	25	All	4.834	0.007	0.016	0.023	0.002	0.023	0.005	2320.108	3.401	7.855	11.256	1.156	10.943	2.175	23201	34.010	78.550	112.560	11.560	109.430	21.750
120	delta	0.6	60x8 weeks	PCR90	Daily	VAC50	25	All	4.834	0.007	0.016	0.023	0.002	0.023	0.005	2320.108	3.401	7.855	11.256	1.156	10.943	2.175	23201	34.010	78.550	112.560	11.560	109.430	21.750
120	delta	0.6	60x8 weeks	PCR90	Daily	VAC50	25	All	4.834	0.007	0.016	0.023	0.002	0.023	0.005	2320.108	3.401	7.855	11.256	1.156	10.943	2.175	23201	34.010	78.550	112.560	11.560	109.430	21.750
120	delta	1	60x8 weeks	PCR90	Daily	VAC50	25	All	4.538	0.006	0.057	0.063	0.007	0.062	0.004	2178.376	2.706	27.589	30.295	3.121	29.781	1.714	21784	27.060	275.890	302.950	31.210	297.810	17.140
120	delta	1	60x8 weeks	PCR90	Daily	VAC50	25	All	4.538	0.006	0.057	0.063	0.007	0.062	0.004	2178.376	2.706	27.589	30.295	3.121	29.781	1.714	21784	27.060	275.890	302.950	31.210	297.810	17.140
120	delta	1	60x8 weeks	PCR90	Daily	VAC50	25	All	4.538	0.006	0.057	0.063	0.007	0.062	0.004	2178.376	2.706	27.589	30.295	3.121	29.781	1.714	21784	27.060	275.890	302.950	31.210	297.810	17.140
120	delta	1.2	60x8 weeks	PCR90	Daily	VAC50	25	All	4.358	0.005	0.082	0.087	0.009	0.086	0.003	2091.676	2.431	39.341	41.772	4.334	41.158	1.574	20917	24.310	393.410	417.720	43.340	411.580	15.740
120	delta	1.2	60x8 weeks	PCR90	Daily	VAC50	25	All	4.358	0.005	0.082	0.087	0.009	0.086	0.003	2091.676	2.431	39.341	41.772	4.334	41.158	1.574	20917	24.310	393.410	417.720	43.340	411.580	15.740
120	delta	1.2	60x8 weeks	PCR90	Daily	VAC50	25	All	4.358	0.005	0.082	0.087	0.009	0.086	0.003	2091.676	2.431	39.341	41.772	4.334	41.158	1.574	20917	24.310	393.410	417.720	43.340	411.580	15.740
120	original	0.6	60x8 weeks	PCR90	Daily	VAC50	50	All	4.958	0.006	0.001	0.007	0.001	0.005	0.005	2379.935	3.023	0.387	3.410	0.656	2.397	2.289	23799	30.230	3.870	34.100	6.560	23.970	22.890
120	original	0.6	60x8 weeks	PCR90	Daily	VAC50	50	All	4.958	0.006	0.001	0.007	0.001	0.005	0.005	2379.935	3.023	0.387	3.410	0.656	2.397	2.289	23799	30.230	3.870	34.100	6.560	23.970	22.890
120	original	0.6	60x8 weeks	PCR90	Daily	VAC50	50	All	4.958	0.006	0.001	0.007	0.001	0.005	0.005	2379.935	3.023	0.387	3.410	0.656	2.397	2.289	23799	30.230	3.870	34.100	6.560	23.970	22.890
120	original	1	60x8 weeks	PCR90	Daily	VAC50	50	All	4.956	0.006	0.001	0.008	0.001	0.005	0.005	2378.805	2.949	0.698	3.647	0.658	2.586	2.328	23788	29.490	6.980	36.470	6.580	25.860	23.280
120	original	1	60x8 weeks	PCR90	Daily	VAC50	50	All	4.956	0.006	0.001	0.008	0.001	0.005	0.005	2378.805	2.949	0.698	3.647	0.658	2.586	2.328	23788	29.490	6.980	36.470	6.580	25.860	23.280
120	original	1	60x8 weeks	PCR90	Daily	VAC50	50	All	4.956	0.006	0.001	0.008	0.001	0.005	0.005	2378.805	2.949	0.698	3.647	0.658	2.586	2.328	23788	29.490	6.980	36.470	6.580	25.860	23.280
120	original	1.2	60x8 weeks	PCR90	Daily	VAC50	50	All	4.953	0.006	0.002	0.008	0.001	0.006	0.005	2377.482	3.003	0.824	3.827	0.690	2.759	2.274	23775	30.030	8.240	38.270	6.900	27.590	22.740
120	original	1.2	60x8 weeks	PCR90	Daily	VAC50	50	All	4.953	0.006	0.002	0.008	0.001	0.006	0.005	2377.482	3.003	0.824	3.827	0.690	2.759	2.274	23775	30.030	8.240	38.270	6.900	27.590	22.740
120	original	1.2	60x8 weeks	PCR90	Daily	VAC50	50	All	4.953	0.006	0.002	0.008	0.001	0.006	0.005	2377.482	3.003	0.824	3.827	0.690	2.759	2.274	23775	30.030	8.240	38.270	6.900	27.590	22.740
120	alpha	0.6	60x8 weeks	PCR90	Daily	VAC50	50	All	4.940	0.006	0.002	0.009	0.001	0.008	0.005	2371.287	2.982	1.115	4.097	0.301	4.059	2.377	23713	29.820	11.150	40.970	3.010	40.590	23.770
120	alpha	0.6	60x8 weeks	PCR90	Daily	VAC50	50	All	4.940	0.006	0.002	0.009	0.001	0.008	0.005	2371.287	2.982	1.115	4.097	0.301	4.059	2.377	23713	29.820	11.150	40.970	3.010	40.590	23.770
120	alpha	0.6	60x8 weeks	PCR90	Daily	VAC50	50	All	4.940	0.006	0.002	0.009	0.001	0.008	0.005	2371.287	2.982	1.115	4.097	0.301	4.059	2.377	23713	29.820	11.150	40.970	3.010	40.590	23.770
120	alpha	1	60x8 weeks	PCR90	Daily	VAC50	50	All	4.926	0.006	0.005	0.010	0.001	0.010	0.005	2364.559	2.797	2.224	5.021	0.371	5.017	2.279	23646	27.970	22.240	50.210	3.710	50.170	22.790
120	alpha	1	60x8 weeks	PCR90	Daily	VAC50	50	All	4.926	0.006	0.005	0.010	0.001	0.010	0.005	2364.559	2.797	2.224	5.021	0.371	5.017	2.279	23646	27.970	22.240	50.210	3.710	50.170	22.790
120	alpha	1	60x8 weeks	PCR90	Daily	VAC50	50	All	4.926	0.006	0.005	0.010	0.001	0.010	0.005	2364.559	2.797	2.224	5.021	0.371	5.017	2.279	23646	27.970	22.240	50.210	3.710	50.170	22.790
120	alpha	1.2	60x8 weeks	PCR90	Daily	VAC50	50	All	4.912	0.006	0.006	0.012	0.001	0.013	0.005	2357.864	2.858	3.073	5.931	0.409	6.005	2.235	23579	28.580	30.730	59.310	4.090	60.050	22.350
120	alpha	1.2	60x8 weeks	PCR90	Daily	VAC50	50	All	4.912	0.006	0.006	0.012	0.001	0.013	0.005	2357.864	2.858	3.073	5.931	0.409	6.005	2.235	23579	28.580	30.730	59.310	4.090	60.050	22.350
120	alpha	1.2	60x8 weeks	PCR90	Daily	VAC50	50	All	4.912	0.006	0.006	0.012	0.001	0.013	0.005	2357.864	2.858	3.073	5.931	0.409	6.005	2.235	23579	28.580	30.730	59.310	4.090	60.050	22.350
120	delta	0.6	60x8 weeks	PCR90	Daily	VAC50	50	All	4.887	0.007	0.009	0.016	0.002	0.015	0.005	2345.943	3.164	4.496	7.660	0.793	7.364	2.247	23459	31.640	44.960	76.600	7.930	73.640	22.470
120	delta	0.6	60x8 weeks	PCR90	Daily	VAC50	50	All	4.887	0.007	0.009	0.016	0.002	0.015	0.005	2345.943	3.164	4.496	7.660	0.793	7.364	2.247	23459	31.640	44.960	76.600	7.930	73.640	22.470
120	delta	0.6	60x8 weeks	PCR90	Daily	VAC50	50	All	4.887	0.007	0.009	0.016	0.002	0.015	0.005	2345.943	3.164	4.496	7.660	0.793	7.364	2.247	23459	31.640	44.960	76.600	7.930	73.640	22.470
120	delta	1	60x8 weeks	PCR90	Daily	VAC50	50	All	4.710	0.006	0.035	0.041	0.004	0.039	0.004	2260.572	2.935	16.598	19.533	2.045	18.875	1.935	22606	29.350	165.980	195.330	20.450	188.750	19.350
120	delta	1	60x8 weeks	PCR90	Daily	VAC50	50	All	4.710	0.006	0.035	0.041	0.004	0.039	0.004	2260.572	2.935	16.598	19.533	2.045	18.875	1.935	22606	29.350	165.980	195.330	20.450	188.750	19.350
120	delta	1	60x8 weeks	PCR90	Daily	VAC50	50	All	4.710	0.006	0.035	0.041	0.004	0.039	0.004	2260.572	2.935	16.598	19.533	2.045	18.875	1.935	22606	29.350	165.980	195.330	20.450	188.750	19.350
120	delta	1.2	60x8 weeks	PCR90	Daily	VAC50	50	All	4.598	0.005	0.050	0.055	0.006	0.054	0.004	2206.834	2.637	23.795	26.432	2.787	25.942	1.930	22068	26.370	237.950	264.320	27.870	259.420	19.300
120	delta	1.2	60x8 weeks	PCR90	Daily	VAC50	50	All	4.598	0.005	0.050	0.055	0.006	0.054	0.004	2206.834	2.637	23.795	26.432	2.787	25.942	1.930	22068	26.370	237.950	264.320	27.870	259.420	19.300
120	delta	1.2	60x8 weeks	PCR90	Daily	VAC50	50	All	4.598	0.005	0.050	0.055	0.006	0.054	0.004	2206.834	2.637	23.795	26.432	2.787	25.942	1.930	22068	26.370	237.950	264.320	27.870	259.420	19.300
120	original	0.6	60x8 weeks	PCR90	Daily	VAC50	75	All	4.971	0.005	0.000	0.006	0.001	0.003	0.005	2386.177	2.492	0.181	2.673	0.714	1.417	2.398	23862	24.920	1.810	26.730	7.140	14.170	23.980
120	original	0.6	60x8 weeks	PCR90	Daily	VAC50	75	All	4.971	0.005	0.000	0.006	0.001	0.003	0.005	2386.177	2.492	0.181	2.673	0.714	1.417	2.398	23862	24.920	1.810	26.730	7.140	14.170	23.980
120	original	0.6	60x8 weeks	PCR90	Daily	VAC50	75	All	4.971	0.005	0.000	0.006	0.001	0.003	0.005	2386.177	2.492	0.181	2.673	0.714	1.417	2.398	23862	24.920	1.810	26.730	7.140	14.170	23.980
120	original	1	60x8 weeks	PCR90	Daily	VAC50	75	All	4.971	0.005	0.001	0.006	0.001	0.003	0.005	2386.275	2.453	0.264	2.717	0.678	1.418	2.329	23863	24.530	2.640	27.170	6.780	14.180	23.290
120	original	1	60x8 weeks	PCR90	Daily	VAC50	75	All	4.971	0.005	0.001	0.006	0.001	0.003	0.005	2386.275	2.453	0.264	2.717	0.678	1.418	2.329	23863	24.530	2.640	27.170	6.780	14.180	23.290
120	original	1	60x8 weeks	PCR90	Daily	VAC50	75	All	4.971	0.005	0.001	0.006	0.001	0.003	0.005	2386.275	2.453	0.264	2.717	0.678	1.418	2.329	23863	24.530	2.640	27.170	6.780	14.180	23.290
120	original	1.2	60x8 weeks	PCR90	Daily	VAC50	75	All	4.969	0.005	0.001	0.006	0.002	0.003	0.005	2385.288	2.464	0.365	2.829	0.782	1.471	2.328	23853	24.640	3.650	28.290	7.820	14.710	23.280
120	original	1.2	60x8 weeks	PCR90	Daily	VAC50	75	All	4.969	0.005	0.001	0.006	0.002	0.003	0.005	2385.288	2.464	0.365	2.829	0.782	1.471	2.328	23853	24.640	3.650	28.290	7.820	14.710	23.280
120	original	1.2	60x8 weeks	PCR90	Daily	VAC50	75	All	4.969	0.005	0.001	0.006	0.002	0.003	0.005	2385.288	2.464	0.365	2.829	0.782	1.471	2.328	23853	24.640	3.650	28.290	7.820	14.710	23.280
120	alpha	0.6	60x8 weeks	PCR90	Daily	VAC50	75	All	4.955	0.005	0.001	0.007	0.000	0.006	0.005	2378.209	2.553	0.631	3.184	0.236	3.088	2.267	23782	25.530	6.310	31.840	2.360	30.880	22.670
120	alpha	0.6	60x8 weeks	PCR90	Daily	VAC50	75	All	4.955	0.005	0.001	0.007	0.000	0.006	0.005	2378.209	2.553	0.631	3.184	0.236	3.088	2.267	23782	25.530	6.310	31.840	2.360	30.880	22.670
120	alpha	0.6	60x8 weeks	PCR90	Daily	VAC50	75	All	4.955	0.005	0.001	0.007	0.000	0.006	0.005	2378.209	2.553	0.631	3.184	0.236	3.088	2.267	23782	25.530	6.310	31.840	2.360	30.880	22.670
120	alpha	1	60x8 weeks	PCR90	Daily	VAC50	75	All	4.949	0.005	0.002	0.007	0.001	0.007	0.005	2375.309	2.466	1.086	3.552	0.281	3.457	2.396	23753	24.660	10.860	35.520	2.810	34.570	23.960
120	alpha	1	60x8 weeks	PCR90	Daily	VAC50	75	All	4.949	0.005	0.002	0.007	0.001	0.007	0.005	2375.309	2.466	1.086	3.552	0.281	3.457	2.396	23753	24.660	10.860	35.520	2.810	34.570	23.960
120	alpha	1	60x8 weeks	PCR90	Daily	VAC50	75	All	4.949	0.005	0.002	0.007	0.001	0.007	0.005	2375.309	2.466	1.086	3.552	0.281	3.457	2.396	23753	24.660	10.860	35.520	2.810	34.570	23.960
120	alpha	1.2	60x8 weeks	PCR90	Daily	VAC50	75	All	4.938	0.005	0.003	0.009	0.001	0.009	0.005	2370.325	2.601	1.652	4.253	0.357	4.167	2.309	23703	26.010	16.520	42.530	3.570	41.670	23.090
120	alpha	1.2	60x8 weeks	PCR90	Daily	VAC50	75	All	4.938	0.005	0.003	0.009	0.001	0.009	0.005	2370.325	2.601	1.652	4.253	0.357	4.167	2.309	23703	26.010	16.520	42.530	3.570	41.670	23.090
120	alpha	1.2	60x8 weeks	PCR90	Daily	VAC50	75	All	4.938	0.005	0.003	0.009	0.001	0.009	0.005	2370.325	2.601	1.652	4.253	0.357	4.167	2.309	23703	26.010	16.520	42.530	3.570	41.670	23.090
120	delta	0.6	60x8 weeks	PCR90	Daily	VAC50	75	All	4.922	0.006	0.005	0.011	0.001	0.011	0.005	2362.615	2.879	2.402	5.281	0.587	5.054	2.343	23626	28.790	24.020	52.810	5.870	50.540	23.430
120	delta	0.6	60x8 weeks	PCR90	Daily	VAC50	75	All	4.922	0.006	0.005	0.011	0.001	0.011	0.005	2362.615	2.879	2.402	5.281	0.587	5.054	2.343	23626	28.790	24.020	52.810	5.870	50.540	23.430
120	delta	0.6	60x8 weeks	PCR90	Daily	VAC50	75	All	4.922	0.006	0.005	0.011	0.001	0.011	0.005	2362.615	2.879	2.402	5.281	0.587	5.054	2.343	23626	28.790	24.020	52.810	5.870	50.540	23.430
120	delta	1	60x8 weeks	PCR90	Daily	VAC50	75	All	4.855	0.006	0.015	0.021	0.002	0.020	0.005	2330.480	2.830	7.061	9.891	1.030	9.537	2.278	23305	28.300	70.610	98.910	10.300	95.370	22.780
120	delta	1	60x8 weeks	PCR90	Daily	VAC50	75	All	4.855	0.006	0.015	0.021	0.002	0.020	0.005	2330.480	2.830	7.061	9.891	1.030	9.537	2.278	23305	28.300	70.610	98.910	10.300	95.370	22.780

120	delta	1	60x8 weeks	PCR90	Daily	VAC50	75	All	4.855	0.006	0.015	0.021	0.002	0.020	0.005	2330.480	2.830	7.061	9.891	1.030	9.537	2.278	23305	28.300	70.610	98.910	10.300	95.370	22.780
120	delta	1.2	60x8 weeks	PCR90	Daily	VAC50	75	All	4.795	0.006	0.024	0.029	0.003	0.028	0.004	2301.612	2.757	11.298	14.055	1.457	13.553	2.140	23016	27.570	112.980	140.550	14.570	135.530	21.400
120	delta	1.2	60x8 weeks	PCR90	Daily	VAC50	75	All	4.795	0.006	0.024	0.029	0.003	0.028	0.004	2301.612	2.757	11.298	14.055	1.457	13.553	2.140	23016	27.570	112.980	140.550	14.570	135.530	21.400
120	delta	1.2	60x8 weeks	PCR90	Daily	VAC50	75	All	4.795	0.006	0.024	0.029	0.003	0.028	0.004	2301.612	2.757	11.298	14.055	1.457	13.553	2.140	23016	27.570	112.980	140.550	14.570	135.530	21.400
120	original	0.6	60x8 weeks	PCR90	Daily	VAC50	100	All	4.982	0.005	0.000	0.005	0.002	0.001	0.005	2391.224	2.162	0.034	2.196	0.855	0.491	2.343	23912	21.620	0.340	21.960	8.550	4.910	23.430
120	original	0.6	60x8 weeks	PCR90	Daily	VAC50	100	All	4.982	0.005	0.000	0.005	0.002	0.001	0.005	2391.224	2.162	0.034	2.196	0.855	0.491	2.343	23912	21.620	0.340	21.960	8.550	4.910	23.430
120	original	0.6	60x8 weeks	PCR90	Daily	VAC50	100	All	4.982	0.005	0.000	0.005	0.002	0.001	0.005	2391.224	2.162	0.034	2.196	0.855	0.491	2.343	23912	21.620	0.340	21.960	8.550	4.910	23.430
120	original	1	60x8 weeks	PCR90	Daily	VAC50	100	All	4.983	0.004	0.000	0.004	0.002	0.001	0.005	2391.729	2.050	0.029	2.079	0.800	0.468	2.303	23917	20.500	0.290	20.790	8.000	4.680	23.030
120	original	1	60x8 weeks	PCR90	Daily	VAC50	100	All	4.983	0.004	0.000	0.004	0.002	0.001	0.005	2391.729	2.050	0.029	2.079	0.800	0.468	2.303	23917	20.500	0.290	20.790	8.000	4.680	23.030
120	original	1	60x8 weeks	PCR90	Daily	VAC50	100	All	4.983	0.004	0.000	0.004	0.002	0.001	0.005	2391.729	2.050	0.029	2.079	0.800	0.468	2.303	23917	20.500	0.290	20.790	8.000	4.680	23.030
120	original	1.2	60x8 weeks	PCR90	Daily	VAC50	100	All	4.983	0.004	0.000	0.004	0.002	0.001	0.005	2391.920	1.992	0.049	2.041	0.779	0.444	2.358	23919	19.920	0.490	20.410	7.790	4.440	23.580
120	original	1.2	60x8 weeks	PCR90	Daily	VAC50	100	All	4.983	0.004	0.000	0.004	0.002	0.001	0.005	2391.920	1.992	0.049	2.041	0.779	0.444	2.358	23919	19.920	0.490	20.410	7.790	4.440	23.580
120	original	1.2	60x8 weeks	PCR90	Daily	VAC50	100	All	4.983	0.004	0.000	0.004	0.002	0.001	0.005	2391.920	1.992	0.049	2.041	0.779	0.444	2.358	23919	19.920	0.490	20.410	7.790	4.440	23.580
120	alpha	0.6	60x8 weeks	PCR90	Daily	VAC50	100	All	4.966	0.004	0.001	0.005	0.000	0.005	0.005	2383.800	2.057	0.278	2.335	0.199	2.247	2.310	23838	20.570	2.780	23.350	1.990	22.470	23.100
120	alpha	0.6	60x8 weeks	PCR90	Daily	VAC50	100	All	4.966	0.004	0.001	0.005	0.000	0.005	0.005	2383.800	2.057	0.278	2.335	0.199	2.247	2.310	23838	20.570	2.780	23.350	1.990	22.470	23.100
120	alpha	0.6	60x8 weeks	PCR90	Daily	VAC50	100	All	4.966	0.004	0.001	0.005	0.000	0.005	0.005	2383.800	2.057	0.278	2.335	0.199	2.247	2.310	23838	20.570	2.780	23.350	1.990	22.470	23.100
120	alpha	1	60x8 weeks	PCR90	Daily	VAC50	100	All	4.963	0.004	0.001	0.005	0.000	0.005	0.005	2382.414	2.036	0.478	2.514	0.207	2.445	2.359	23824	20.360	4.780	25.140	2.070	24.450	23.590
120	alpha	1	60x8 weeks	PCR90	Daily	VAC50	100	All	4.963	0.004	0.001	0.005	0.000	0.005	0.005	2382.414	2.036	0.478	2.514	0.207	2.445	2.359	23824	20.360	4.780	25.140	2.070	24.450	23.590
120	alpha	1	60x8 weeks	PCR90	Daily	VAC50	100	All	4.963	0.004	0.001	0.005	0.000	0.005	0.005	2382.414	2.036	0.478	2.514	0.207	2.445	2.359	23824	20.360	4.780	25.140	2.070	24.450	23.590
120	alpha	1.2	60x8 weeks	PCR90	Daily	VAC50	100	All	4.961	0.004	0.001	0.006	0.000	0.005	0.005	2381.441	2.031	0.646	2.677	0.205	2.618	2.398	23814	20.310	6.460	26.770	2.050	26.180	23.980
120	alpha	1.2	60x8 weeks	PCR90	Daily	VAC50	100	All	4.961	0.004	0.001	0.006	0.000	0.005	0.005	2381.441	2.031	0.646	2.677	0.205	2.618	2.398	23814	20.310	6.460	26.770	2.050	26.180	23.980
120	alpha	1.2	60x8 weeks	PCR90	Daily	VAC50	100	All	4.961	0.004	0.001	0.006	0.000	0.005	0.005	2381.441	2.031	0.646	2.677	0.205	2.618	2.398	23814	20.310	6.460	26.770	2.050	26.180	23.980
120	delta	0.6	60x8 weeks	PCR90	Daily	VAC50	100	All	4.945	0.006	0.002	0.008	0.001	0.008	0.005	2373.401	2.643	1.162	3.805	0.393	3.637	2.297	23734	26.430	11.620	38.050	3.930	36.370	22.970
120	delta	0.6	60x8 weeks	PCR90	Daily	VAC50	100	All	4.945	0.006	0.002	0.008	0.001	0.008	0.005	2373.401	2.643	1.162	3.805	0.393	3.637	2.297	23734	26.430	11.620	38.050	3.930	36.370	22.970
120	delta	0.6	60x8 weeks	PCR90	Daily	VAC50	100	All	4.945	0.006	0.002	0.008	0.001	0.008	0.005	2373.401	2.643	1.162	3.805	0.393	3.637	2.297	23734	26.430	11.620	38.050	3.930	36.370	22.970
120	delta	1	60x8 weeks	PCR90	Daily	VAC50	100	All	4.920	0.005	0.006	0.011	0.001	0.011	0.005	2361.839	2.600	2.837	5.437	0.579	5.217	2.271	23618	26.000	28.370	54.370	5.790	52.170	22.710
120	delta	1	60x8 weeks	PCR90	Daily	VAC50	100	All	4.920	0.005	0.006	0.011	0.001	0.011	0.005	2361.839	2.600	2.837	5.437	0.579	5.217	2.271	23618	26.000	28.370	54.370	5.790	52.170	22.710
120	delta	1	60x8 weeks	PCR90	Daily	VAC50	100	All	4.920	0.005	0.006	0.011	0.001	0.011	0.005	2361.839	2.600	2.837	5.437	0.579	5.217	2.271	23618	26.000	28.370	54.370	5.790	52.170	22.710
120	delta	1.2	60x8 weeks	PCR90	Daily	VAC50	100	All	4.898	0.006	0.009	0.015	0.001	0.014	0.005	2351.128	2.683	4.364	7.047	0.709	6.753	2.244	23511	26.830	43.640	70.470	7.090	67.530	22.440
120	delta	1.2	60x8 weeks	PCR90	Daily	VAC50	100	All	4.898	0.006	0.009	0.015	0.001	0.014	0.005	2351.128	2.683	4.364	7.047	0.709	6.753	2.244	23511	26.830	43.640	70.470	7.090	67.530	22.440
120	delta	1.2	60x8 weeks	PCR90	Daily	VAC50	100	All	4.898	0.006	0.009	0.015	0.001	0.014	0.005	2351.128	2.683	4.364	7.047	0.709	6.753	2.244	23511	26.830	43.640	70.470	7.090	67.530	22.440
120	original	0.6	60x8 weeks	PCR90	MWF	NOVAC	0	All	2.960	0.008	0.003	0.011	0.001	0.010	0.003	1420.607	3.861	1.391	5.252	0.525	4.867	1.370	14206	38.610	13.910	52.520	5.250	48.670	13.700
120	original	0.6	60x8 weeks	PCR90	MWF	NOVAC	0	All	2.960	0.008	0.003	0.011	0.001	0.010	0.003	1420.607	3.861	1.391	5.252	0.525	4.867	1.370	14206	38.610	13.910	52.520	5.250	48.670	13.700
120	original	0.6	60x8 weeks	PCR90	MWF	NOVAC	0	All	2.960	0.008	0.003	0.011	0.001	0.010	0.003	1420.607	3.861	1.391	5.252	0.525	4.867	1.370	14206	38.610	13.910	52.520	5.250	48.670	13.700
120	original	1	60x8 weeks	PCR90	MWF	NOVAC	0	All	2.947	0.008	0.006	0.014	0.002	0.013	0.003	1414.633	3.824	3.022	6.846	0.738	6.290	1.366	14146	38.240	30.220	68.460	7.380	62.900	13.660
120	original	1	60x8 weeks	PCR90	MWF	NOVAC	0	All	2.947	0.008	0.006	0.014	0.002	0.013	0.003	1414.633	3.824	3.022	6.846	0.738	6.290	1.366	14146	38.240	30.220	68.460	7.380	62.900	13.660
120	original	1	60x8 weeks	PCR90	MWF	NOVAC	0	All	2.947	0.008	0.006	0.014	0.002	0.013	0.003	1414.633	3.824	3.022	6.846	0.738	6.290	1.366	14146	38.240	30.220	68.460	7.380	62.900	13.660
120	original	1.2	60x8 weeks	PCR90	MWF	NOVAC	0	All	2.939	0.008	0.008	0.016	0.002	0.015	0.003	1410.872	3.779	3.949	7.728	0.758	7.267	1.338	14109	37.790	39.490	77.280	7.580	72.670	13.380
120	original	1.2	60x8 weeks	PCR90	MWF	NOVAC	0	All	2.939	0.008	0.008	0.016	0.002	0.015	0.003	1410.872	3.779	3.949	7.728	0.758	7.267	1.338	14109	37.790	39.490	77.280	7.580	72.670	13.380
120	original	1.2	60x8 weeks	PCR90	MWF	NOVAC	0	All	2.939	0.008	0.008	0.016	0.002	0.015	0.003	1410.872	3.779	3.949	7.728	0.758	7.267	1.338	14109	37.790	39.490	77.280	7.580	72.670	13.380
120	alpha	0.6	60x8 weeks	PCR90	MWF	NOVAC	0	All	2.940	0.008	0.008	0.016	0.002	0.015	0.003	1410.994	3.752	4.001	7.753	0.813	7.343	1.379	14110	37.520	40.010	77.530	8.130	73.430	13.790
120	alpha	0.6	60x8 weeks	PCR90	MWF	NOVAC	0	All	2.940	0.008	0.008	0.016	0.002	0.015	0.003	1410.994	3.752	4.001	7.753	0.813	7.343	1.379	14110	37.520	40.010	77.530	8.130	73.430	13.790
120	alpha	0.6	60x8 weeks	PCR90	MWF	NOVAC	0	All	2.940	0.008	0.008	0.016	0.002	0.015	0.003	1410.994	3.752	4.001	7.753	0.813	7.343	1.379	14110	37.520	40.010	77.530	8.130	73.430	13.790
120	alpha	1	60x8 weeks	PCR90	MWF	NOVAC	0	All	2.877	0.007	0.025	0.032	0.004	0.031	0.003	1380.763	3.502	12.031	15.533	1.743	14.687	1.274	13808	35.020	120.310	155.330	17.430	146.870	12.740
120	alpha	1	60x8 weeks	PCR90	MWF	NOVAC	0	All	2.877	0.007	0.025	0.032	0.004	0.031	0.003	1380.763	3.502	12.031	15.533	1.743	14.687	1.274	13808	35.020	120.310	155.330	17.430	146.870	12.740
120	alpha	1	60x8 weeks	PCR90	MWF	NOVAC	0	All	2.877	0.007	0.025	0.032	0.004	0.031	0.003	1380.763	3.502	12.031	15.533	1.743	14.687	1.274	13808	35.020	120.310	155.330	17.430	146.870	12.740
120	alpha	1.2	60x8 weeks	PCR90	MWF	NOVAC	0	All	2.816	0.007	0.041	0.048	0.006	0.045	0.003	1351.564	3.290	19.608	22.898	2.719	21.643	1.210	13516	32.900	196.080	228.980	27.190	216.430	12.100
120	alpha	1.2	60x8 weeks	PCR90	MWF	NOVAC	0	All	2.816	0.007	0.041	0.048	0.006	0.045	0.003	1351.564	3.290	19.608	22.898	2.719	21.643	1.210	13516	32.900	196.080	228.980	27.190	216.430	12.100
120	alpha	1.2	60x8 weeks	PCR90	MWF	NOVAC	0	All	2.816	0.007	0.041	0.048	0.006	0.045	0.003	1351.564	3.290	19.608	22.898	2.719	21.643	1.210	13516	32.900	196.080	228.980	27.190	216.430	12.100
120	delta	0.6	60x8 weeks	PCR90	MWF	NOVAC	0	All	2.769	0.007	0.052	0.058	0.009	0.053	0.002	1328.968	3.171	24.740	27.911	4.542	25.320	1.094	13290	31.710	247.400	279.110	45.420	253.200	10.940
120	delta	0.6	60x8 weeks	PCR90	MWF	NOVAC	0	All	2.769	0.007	0.052	0.058	0.009	0.053	0.002	1328.968	3.171	24.740	27.911	4.542	25.320	1.094	13290	31.710	247.400	279.110	45.420	253.200	10.940
120	delta	0.6	60x8 weeks	PCR90	MWF	NOVAC	0	All	2.769	0.007	0.052	0.058	0.009	0.053	0.002	1328.968	3.171	24.740	27.911	4.542	25.320	1.094	13290	31.710	247.400	279.110	45.420	253.200	10.940
120	delta	1	60x8 weeks	PCR90	MWF	NOVAC	0	All	2.534	0.004	0.106	0.110	0.019	0.101	0.001	1216.488	2.029	50.930	52.959	9.297	48.649	0.696	12165	20.290	509.300	529.590	92.970	486.490	6.960
120	delta	1	60x8 weeks	PCR90	MWF	NOVAC	0	All	2.534	0.004	0.106	0.110	0.019	0.101	0.001	1216.488	2.029	50.930	52.959	9.297	48.649	0.696	12165	20.290	509.300	529.590	92.970	486.490	6.960
120	delta	1	60x8 weeks	PCR90	MWF	NOVAC	0	All	2.534	0.004	0.106	0.110	0.019	0.101	0.001	1216.488	2.029	50.930	52.959	9.297	48.649	0.696	12165	20.290	509.300	529.590	92.970	486.490	6.960
120	delta	1.2	60x8 weeks	PCR90	MWF	NOVAC	0	All	2.519	0.004	0.111	0.115	0.020	0.105	0.001	1208.906	1.908	53.274	55.182	9.587	50.492	0.655	12089	19.080	532.740	551.820	95.870	504.920	6.550
120	delta	1.2	60x8 weeks	PCR90	MWF	NOVAC	0	All	2.519	0.004	0.111	0.115	0.020	0.105	0.001	1208.906	1.908	53.274	55.182	9.587	50.492	0.655	12089	19.080	532.740	551.820	95.870	504.920	6.550
120	delta	1.2	60x8 weeks	PCR90	MWF	NOVAC	0	All	2.519	0.004	0.111	0.115	0.020	0.105	0.001	1208.906	1.908	53.274	55.182	9.587	50.492	0.655	12089	19.080	532.740	551.820	95.870	504.920	6.550
120	original	0.6	60x8 weeks	PCR90	MWF	VAC95	25	All	2.972	0.006	0.001	0.008	0.001	0.007	0.003	1426.734	2.910	0.715	3.625	0.388	3.313	1.366	14267	29.100	7.150	36.250	3.880	33.130	13.660
120	original	0.6	60x8 weeks	PCR90	MWF	VAC95	25	All	2.972	0.006	0.001	0.008	0.001	0.007	0.003	1426.734	2.910	0.715	3.625	0.388	3.313	1.366	14267	29.100	7.150	36.250	3.880	33.130	13.660
120	original	0.6	60x8 weeks	PCR90	MWF	VAC95	25	All	2.972	0.006	0.001	0.008	0.001	0.007	0.003	1426.734	2.910	0.715	3.625	0.388	3.313	1.366	14267	29.100	7.150	36.250	3.880	33.130	13.660
120	original	1	60x8 weeks	PCR90	MWF	VAC95	25	All	2.968	0.006	0.003	0.009	0.001	0.008	0.003	1424.474	2.890	1.392	4.282	0.437	3.871	1.417	14245	28.900	13.920	42.820	4.370	38.710	14.170
120	original	1	60x8 weeks	PCR90	MWF	VAC95	25	All	2.968	0.006	0.003	0.009	0.001	0.008	0.003	1424.474	2.890	1.392	4.282	0.437	3.871	1.417	14245	28.900	13.920	42.820	4.370	38.710	14.170
120	original	1	60x8 weeks	PCR90	MWF	VAC95	25	All	2.968	0.006	0.003	0.009	0.001	0.008	0.003	1424.474	2.890	1.392	4.282	0.437	3.871	1.417	14245	28.900	13.920	42.820	4.370	38.710	14.170
120	original	1.2	60x8 weeks	PCR90	MWF	VAC95	25	All	2.964	0.006	0.004	0.010	0.001	0.009	0.003	1422.815	2.936	1.813	4.749	0.525	4.262	1.415	14228	29.360	18.130	47.490	5.250	42.620	14.150
120	original	1.2	60x8 weeks	PCR90	MWF	VAC95	25	All	2.964	0.006	0.004	0.010	0.001	0.009	0.003	1422.815	2.936	1.813	4.749	0.525	4.262	1.415	14228	29.360	18.130	47.490	5.250	42.620	14.150
120	original	1.2	60x8 weeks	PCR90	MWF	VAC95	25	All	2.964	0.006	0.004	0.010	0.001	0.009	0.003	1422.815	2.936	1.813	4.749	0.525	4.262	1.415	14228	29.360	18.130	47.490	5.250	42.620	14.150
120	alpha	0.6	60x8 weeks	PCR90	MWF	VAC95	25	All	2.961	0.006	0.004	0.010	0.001	0.010	0.003	1421.053	3.007	1.922	4.929	0.558	4.686	1.462	14211	30.070	19.220	49.290	5.580	46.860	14.620
120	alpha	0.6	60x8 weeks	PCR90	MWF	VAC95	25	All	2.961	0.006	0.004	0.010	0.001	0.010	0.003	1421.053	3.007	1.922	4.929	0.558	4.686	1.462	14211	30.070	19.220	49.290	5.580	46.860	14.620
120	alpha	0.6	60x8 weeks	PCR90	MWF	VAC95	25	All	2.961	0.006	0.004	0.010	0.001	0.010	0.003	1421.053	3.007	1.922	4.929	0.558	4.686	1.462	14211	30.070	19.220	49.290	5.580	46.860	14.620
120	alpha	1	60x8 weeks	PCR90	MWF	VAC95	25	All	2.942	0.006	0.009	0.015	0.002	0.015	0.003	1412.051	2.869	4.458	7.327	0.753	6.977	1.297	14121	28.690	44.580	73.270	7.530	69.770	12.970

120	alpha	1	60x8 weeks	PCR90	MWF	VAC95	25	All	2.942	0.006	0.009	0.015	0.002	0.015	0.003	1412.051	2.869	4.458	7.327	0.753	6.977	1.297	14121	28.690	44.580	73.270	7.530	69.770	12.970
120	alpha	1	60x8 weeks	PCR90	MWF	VAC95	25	All	2.942	0.006	0.009	0.015	0.002	0.015	0.003	1412.051	2.869	4.458	7.327	0.753	6.977	1.297	14121	28.690	44.580	73.270	7.530	69.770	12.970
120	alpha	1.2	60x8 weeks	PCR90	MWF	VAC95	25	All	2.923	0.006	0.015	0.021	0.002	0.019	0.003	1402.999	2.862	7.000	9.862	1.098	9.276	1.355	14030	28.620	70.000	98.620	10.980	92.760	13.550
120	alpha	1.2	60x8 weeks	PCR90	MWF	VAC95	25	All	2.923	0.006	0.015	0.021	0.002	0.019	0.003	1402.999	2.862	7.000	9.862	1.098	9.276	1.355	14030	28.620	70.000	98.620	10.980	92.760	13.550
120	alpha	1.2	60x8 weeks	PCR90	MWF	VAC95	25	All	2.923	0.006	0.015	0.021	0.002	0.019	0.003	1402.999	2.862	7.000	9.862	1.098	9.276	1.355	14030	28.620	70.000	98.620	10.980	92.760	13.550
120	delta	0.6	60x8 weeks	PCR90	MWF	VAC95	25	All	2.905	0.006	0.019	0.024	0.004	0.022	0.003	1394.553	2.817	8.932	11.749	1.851	10.634	1.312	13946	28.170	89.320	117.490	18.510	106.340	13.120
120	delta	0.6	60x8 weeks	PCR90	MWF	VAC95	25	All	2.905	0.006	0.019	0.024	0.004	0.022	0.003	1394.553	2.817	8.932	11.749	1.851	10.634	1.312	13946	28.170	89.320	117.490	18.510	106.340	13.120
120	delta	0.6	60x8 weeks	PCR90	MWF	VAC95	25	All	2.905	0.006	0.019	0.024	0.004	0.022	0.003	1394.553	2.817	8.932	11.749	1.851	10.634	1.312	13946	28.170	89.320	117.490	18.510	106.340	13.120
120	delta	1	60x8 weeks	PCR90	MWF	VAC95	25	All	2.748	0.005	0.058	0.063	0.011	0.057	0.002	1319.271	2.189	27.855	30.044	5.070	27.178	1.086	13193	21.890	278.550	300.440	50.700	271.780	10.860
120	delta	1	60x8 weeks	PCR90	MWF	VAC95	25	All	2.748	0.005	0.058	0.063	0.011	0.057	0.002	1319.271	2.189	27.855	30.044	5.070	27.178	1.086	13193	21.890	278.550	300.440	50.700	271.780	10.860
120	delta	1	60x8 weeks	PCR90	MWF	VAC95	25	All	2.748	0.005	0.058	0.063	0.011	0.057	0.002	1319.271	2.189	27.855	30.044	5.070	27.178	1.086	13193	21.890	278.550	300.440	50.700	271.780	10.860
120	delta	1.2	60x8 weeks	PCR90	MWF	VAC95	25	All	2.689	0.004	0.073	0.077	0.013	0.070	0.002	1290.828	1.949	35.230	37.179	6.204	33.678	0.912	12908	19.490	352.300	371.790	62.040	336.780	9.120
120	delta	1.2	60x8 weeks	PCR90	MWF	VAC95	25	All	2.689	0.004	0.073	0.077	0.013	0.070	0.002	1290.828	1.949	35.230	37.179	6.204	33.678	0.912	12908	19.490	352.300	371.790	62.040	336.780	9.120
120	delta	1.2	60x8 weeks	PCR90	MWF	VAC95	25	All	2.689	0.004	0.073	0.077	0.013	0.070	0.002	1290.828	1.949	35.230	37.179	6.204	33.678	0.912	12908	19.490	352.300	371.790	62.040	336.780	9.120
120	original	0.6	60x8 weeks	PCR90	MWF	VAC95	50	All	2.982	0.004	0.001	0.005	0.001	0.004	0.003	1431.347	2.114	0.301	2.415	0.276	2.139	1.374	14313	21.140	3.010	24.150	2.760	21.390	13.740
120	original	0.6	60x8 weeks	PCR90	MWF	VAC95	50	All	2.982	0.004	0.001	0.005	0.001	0.004	0.003	1431.347	2.114	0.301	2.415	0.276	2.139	1.374	14313	21.140	3.010	24.150	2.760	21.390	13.740
120	original	0.6	60x8 weeks	PCR90	MWF	VAC95	50	All	2.982	0.004	0.001	0.005	0.001	0.004	0.003	1431.347	2.114	0.301	2.415	0.276	2.139	1.374	14313	21.140	3.010	24.150	2.760	21.390	13.740
120	original	1	60x8 weeks	PCR90	MWF	VAC95	50	All	2.980	0.004	0.001	0.006	0.001	0.005	0.003	1430.594	2.075	0.574	2.649	0.325	2.295	1.425	14306	20.750	5.740	26.490	3.250	22.950	14.250
120	original	1	60x8 weeks	PCR90	MWF	VAC95	50	All	2.980	0.004	0.001	0.006	0.001	0.005	0.003	1430.594	2.075	0.574	2.649	0.325	2.295	1.425	14306	20.750	5.740	26.490	3.250	22.950	14.250
120	original	1	60x8 weeks	PCR90	MWF	VAC95	50	All	2.980	0.004	0.001	0.006	0.001	0.005	0.003	1430.594	2.075	0.574	2.649	0.325	2.295	1.425	14306	20.750	5.740	26.490	3.250	22.950	14.250
120	original	1.2	60x8 weeks	PCR90	MWF	VAC95	50	All	2.980	0.004	0.001	0.006	0.001	0.005	0.003	1430.360	2.009	0.691	2.700	0.315	2.350	1.388	14304	20.090	6.910	27.000	3.150	23.500	13.880
120	original	1.2	60x8 weeks	PCR90	MWF	VAC95	50	All	2.980	0.004	0.001	0.006	0.001	0.005	0.003	1430.360	2.009	0.691	2.700	0.315	2.350	1.388	14304	20.090	6.910	27.000	3.150	23.500	13.880
120	original	1.2	60x8 weeks	PCR90	MWF	VAC95	50	All	2.980	0.004	0.001	0.006	0.001	0.005	0.003	1430.360	2.009	0.691	2.700	0.315	2.350	1.388	14304	20.090	6.910	27.000	3.150	23.500	13.880
120	alpha	0.6	60x8 weeks	PCR90	MWF	VAC95	50	All	2.977	0.004	0.002	0.006	0.001	0.006	0.003	1429.123	2.155	0.734	2.889	0.288	2.741	1.357	14291	21.550	7.340	28.890	2.880	27.410	13.570
120	alpha	0.6	60x8 weeks	PCR90	MWF	VAC95	50	All	2.977	0.004	0.002	0.006	0.001	0.006	0.003	1429.123	2.155	0.734	2.889	0.288	2.741	1.357	14291	21.550	7.340	28.890	2.880	27.410	13.570
120	alpha	0.6	60x8 weeks	PCR90	MWF	VAC95	50	All	2.977	0.004	0.002	0.006	0.001	0.006	0.003	1429.123	2.155	0.734	2.889	0.288	2.741	1.357	14291	21.550	7.340	28.890	2.880	27.410	13.570
120	alpha	1	60x8 weeks	PCR90	MWF	VAC95	50	All	2.970	0.004	0.003	0.008	0.001	0.007	0.003	1425.547	2.045	1.667	3.712	0.398	3.560	1.394	14255	20.450	16.670	37.120	3.980	35.600	13.940
120	alpha	1	60x8 weeks	PCR90	MWF	VAC95	50	All	2.970	0.004	0.003	0.008	0.001	0.007	0.003	1425.547	2.045	1.667	3.712	0.398	3.560	1.394	14255	20.450	16.670	37.120	3.980	35.600	13.940
120	alpha	1	60x8 weeks	PCR90	MWF	VAC95	50	All	2.970	0.004	0.003	0.008	0.001	0.007	0.003	1425.547	2.045	1.667	3.712	0.398	3.560	1.394	14255	20.450	16.670	37.120	3.980	35.600	13.940
120	alpha	1.2	60x8 weeks	PCR90	MWF	VAC95	50	All	2.968	0.004	0.004	0.008	0.001	0.008	0.003	1424.446	2.037	2.027	4.064	0.457	3.845	1.446	14244	20.370	20.270	40.640	4.570	38.450	14.460
120	alpha	1.2	60x8 weeks	PCR90	MWF	VAC95	50	All	2.968	0.004	0.004	0.008	0.001	0.008	0.003	1424.446	2.037	2.027	4.064	0.457	3.845	1.446	14244	20.370	20.270	40.640	4.570	38.450	14.460
120	alpha	1.2	60x8 weeks	PCR90	MWF	VAC95	50	All	2.968	0.004	0.004	0.008	0.001	0.008	0.003	1424.446	2.037	2.027	4.064	0.457	3.845	1.446	14244	20.370	20.270	40.640	4.570	38.450	14.460
120	delta	0.6	60x8 weeks	PCR90	MWF	VAC95	50	All	2.960	0.004	0.006	0.010	0.002	0.009	0.003	1420.936	2.122	2.882	5.004	0.780	4.489	1.363	14209	21.220	28.820	50.040	7.800	44.890	13.630
120	delta	0.6	60x8 weeks	PCR90	MWF	VAC95	50	All	2.960	0.004	0.006	0.010	0.002	0.009	0.003	1420.936	2.122	2.882	5.004	0.780	4.489	1.363	14209	21.220	28.820	50.040	7.800	44.890	13.630
120	delta	0.6	60x8 weeks	PCR90	MWF	VAC95	50	All	2.960	0.004	0.006	0.010	0.002	0.009	0.003	1420.936	2.122	2.882	5.004	0.780	4.489	1.363	14209	21.220	28.820	50.040	7.800	44.890	13.630
120	delta	1	60x8 weeks	PCR90	MWF	VAC95	50	All	2.917	0.004	0.018	0.022	0.003	0.020	0.003	1400.110	2.045	8.566	10.611	1.669	9.431	1.325	14001	20.450	85.660	106.110	16.690	94.310	13.250
120	delta	1	60x8 weeks	PCR90	MWF	VAC95	50	All	2.917	0.004	0.018	0.022	0.003	0.020	0.003	1400.110	2.045	8.566	10.611	1.669	9.431	1.325	14001	20.450	85.660	106.110	16.690	94.310	13.250
120	delta	1	60x8 weeks	PCR90	MWF	VAC95	50	All	2.917	0.004	0.018	0.022	0.003	0.020	0.003	1400.110	2.045	8.566	10.611	1.669	9.431	1.325	14001	20.450	85.660	106.110	16.690	94.310	13.250
120	delta	1.2	60x8 weeks	PCR90	MWF	VAC95	50	All	2.877	0.004	0.028	0.032	0.005	0.028	0.003	1380.927	1.861	13.386	15.247	2.519	13.644	1.316	13809	18.610	133.860	152.470	25.190	136.440	13.160
120	delta	1.2	60x8 weeks	PCR90	MWF	VAC95	50	All	2.877	0.004	0.028	0.032	0.005	0.028	0.003	1380.927	1.861	13.386	15.247	2.519	13.644	1.316	13809	18.610	133.860	152.470	25.190	136.440	13.160
120	delta	1.2	60x8 weeks	PCR90	MWF	VAC95	50	All	2.877	0.004	0.028	0.032	0.005	0.028	0.003	1380.927	1.861	13.386	15.247	2.519	13.644	1.316	13809	18.610	133.860	152.470	25.190	136.440	13.160
120	original	0.6	60x8 weeks	PCR90	MWF	VAC95	75	All	2.991	0.003	0.000	0.003	0.000	0.002	0.003	1435.527	1.208	0.089	1.297	0.189	1.048	1.412	14355	12.080	0.890	12.970	1.890	10.480	14.120
120	original	0.6	60x8 weeks	PCR90	MWF	VAC95	75	All	2.991	0.003	0.000	0.003	0.000	0.002	0.003	1435.527	1.208	0.089	1.297	0.189	1.048	1.412	14355	12.080	0.890	12.970	1.890	10.480	14.120
120	original	0.6	60x8 weeks	PCR90	MWF	VAC95	75	All	2.991	0.003	0.000	0.003	0.000	0.002	0.003	1435.527	1.208	0.089	1.297	0.189	1.048	1.412	14355	12.080	0.890	12.970	1.890	10.480	14.120
120	original	1	60x8 weeks	PCR90	MWF	VAC95	75	All	2.990	0.002	0.000	0.003	0.000	0.002	0.003	1435.415	1.180	0.149	1.329	0.192	1.062	1.416	14354	11.800	1.490	13.290	1.920	10.620	14.160
120	original	1	60x8 weeks	PCR90	MWF	VAC95	75	All	2.990	0.002	0.000	0.003	0.000	0.002	0.003	1435.415	1.180	0.149	1.329	0.192	1.062	1.416	14354	11.800	1.490	13.290	1.920	10.620	14.160
120	original	1	60x8 weeks	PCR90	MWF	VAC95	75	All	2.990	0.002	0.000	0.003	0.000	0.002	0.003	1435.415	1.180	0.149	1.329	0.192	1.062	1.416	14354	11.800	1.490	13.290	1.920	10.620	14.160
120	original	1.2	60x8 weeks	PCR90	MWF	VAC95	75	All	2.990	0.003	0.000	0.003	0.000	0.002	0.003	1435.433	1.205	0.173	1.378	0.211	1.061	1.423	14354	12.050	1.730	13.780	2.110	10.610	14.230
120	original	1.2	60x8 weeks	PCR90	MWF	VAC95	75	All	2.990	0.003	0.000	0.003	0.000	0.002	0.003	1435.433	1.205	0.173	1.378	0.211	1.061	1.423	14354	12.050	1.730	13.780	2.110	10.610	14.230
120	original	1.2	60x8 weeks	PCR90	MWF	VAC95	75	All	2.990	0.003	0.000	0.003	0.000	0.002	0.003	1435.433	1.205	0.173	1.378	0.211	1.061	1.423	14354	12.050	1.730	13.780	2.110	10.610	14.230
120	alpha	0.6	60x8 weeks	PCR90	MWF	VAC95	75	All	2.988	0.003	0.000	0.003	0.000	0.003	0.003	1434.241	1.316	0.219	1.535	0.167	1.436	1.435	14342	13.160	2.190	15.350	1.670	14.360	14.350
120	alpha	0.6	60x8 weeks	PCR90	MWF	VAC95	75	All	2.988	0.003	0.000	0.003	0.000	0.003	0.003	1434.241	1.316	0.219	1.535	0.167	1.436	1.435	14342	13.160	2.190	15.350	1.670	14.360	14.350
120	alpha	0.6	60x8 weeks	PCR90	MWF	VAC95	75	All	2.988	0.003	0.000	0.003	0.000	0.003	0.003	1434.241	1.316	0.219	1.535	0.167	1.436	1.435	14342	13.160	2.190	15.350	1.670	14.360	14.350
120	alpha	1	60x8 weeks	PCR90	MWF	VAC95	75	All	2.987	0.003	0.001	0.003	0.000	0.003	0.003	1433.891	1.219	0.418	1.637	0.158	1.547	1.377	14339	12.190	4.180	16.370	1.580	15.470	13.770
120	alpha	1	60x8 weeks	PCR90	MWF	VAC95	75	All	2.987	0.003	0.001	0.003	0.000	0.003	0.003	1433.891	1.219	0.418	1.637	0.158	1.547	1.377	14339	12.190	4.180	16.370	1.580	15.470	13.770
120	alpha	1	60x8 weeks	PCR90	MWF	VAC95	75	All	2.987	0.003	0.001	0.003	0.000	0.003	0.003	1433.891	1.219	0.418	1.637	0.158	1.547	1.377	14339	12.190	4.180	16.370	1.580	15.470	13.770
120	alpha	1.2	60x8 weeks	PCR90	MWF	VAC95	75	All	2.987	0.003	0.001	0.003	0.000	0.003	0.003	1433.900	1.202	0.450	1.652	0.200	1.521	1.465	14339	12.020	4.500	16.520	2.000	15.210	14.650
120	alpha	1.2	60x8 weeks	PCR90	MWF	VAC95	75	All	2.987	0.003	0.001	0.003	0.000	0.003	0.003	1433.900	1.202	0.450	1.652	0.200	1.521	1.465	14339	12.020	4.500	16.520	2.000	15.210	14.650
120	alpha	1.2	60x8 weeks	PCR90	MWF	VAC95	75	All	2.987	0.003	0.001	0.003	0.000	0.003	0.003	1433.900	1.202	0.450	1.652	0.200	1.521	1.465	14339	12.020	4.500	16.520	2.000	15.210	14.650
120	delta	0.6	60x8 weeks	PCR90	MWF	VAC95	75	All	2.984	0.003	0.001	0.004	0.001	0.004	0.003	1432.337	1.355	0.672	2.027	0.311	1.807	1.405	14323	13.550	6.720	20.270	3.110	18.070	14.050
120	delta	0.6	60x8 weeks	PCR90	MWF	VAC95	75	All	2.984	0.003	0.001	0.004	0.001	0.004	0.003	1432.337	1.355	0.672	2.027	0.311	1.807	1.405	14323	13.550	6.720	20.270	3.110	18.070	14.050
120	delta	0.6	60x8 weeks	PCR90	MWF	VAC95	75	All	2.984	0.003	0.001	0.004	0.001	0.004	0.003	1432.337	1.355	0.672	2.027	0.311	1.807	1.405	14323	13.550	6.720	20.270	3.110	18.070	14.050
120	delta	1	60x8 weeks	PCR90	MWF	VAC95	75	All	2.978	0.003	0.003	0.006	0.001	0.005	0.003	1429.461	1.302	1.479	2.781	0.396	2.526	1.383	14295	13.020	14.790	27.810	3.960	25.260	13.830
120	delta	1	60x8 weeks	PCR90	MWF	VAC95	75	All	2.978	0.003	0.003	0.006	0.001	0.005	0.003	1429.461	1.302	1.479	2.781	0.396	2.526	1.383	14295	13.020	14.790	27.810	3.960	25.260	13.830
120	delta	1	60x8 weeks	PCR90	MWF	VAC95	75	All	2.978	0.003	0.003	0.006	0.001	0.005	0.003	1429.461	1.302	1.479	2.781	0.396	2.526	1.383	14295	13.020	14.790	27.810	3.960	25.260	13.830
120	delta	1.2	60x8 weeks	PCR90	MWF	VAC95	75	All	2.974	0.003	0.004	0.007	0.001	0.006	0.003	1427.608	1.330	1.923	3.253	0.515	2.867	1.355	14276	13.300	19.230	32.530	5.150	28.670	13.550
120	delta	1.2	60x8 weeks	PCR90	MWF	VAC95	75	All	2.974	0.003	0.004	0.007	0.001	0.006	0.003	1427.608	1.330	1.923	3.253	0.515	2.867	1.355	14276	13.300	19.230	32.530	5.150	28.670	13.550
120	delta	1.2	60x8 weeks	PCR90	MWF	VAC95	75	All	2.974	0.003	0.004	0.007	0.001	0.006	0.003	1427.608	1.330	1.923	3.253	0.515	2.867	1.355	14276	13.300	19.230	32.530	5.150	28.670	13.550
120	original	0.6	60x8 weeks	PCR90	MWF	VAC95	100	All	2.999	0.001	0.000	0.001	0.000	0.000	0.003	1439.567	0.257	0.000	0.257	0.093	0.024	1.368	14396	2.570	0.000	2.570	0.930	0.240	13.680
120	original	0.6	60x8 weeks	PCR90	MWF	VAC95	100	All	2.999	0.001	0.000	0.001	0.000	0.000	0.003	1439.567	0.257	0.000	0.257	0.093	0.024	1.368	14396	2.570	0.000	2.570	0.930	0.240	13.680
120	original	0.6	60x8 weeks	PCR90	MWF	VAC95	100	All	2.999	0.001	0.000	0.001	0.000	0.000	0.003	1439.567	0.257	0.000	0.257	0.093	0.024	1.368	14396	2.570	0.000	2.570	0.930	0.240	13.680

120	original	1	60x8 weeks	PCR90	MWF	VAC95	100	All	2.999	0.001	0.000	0.001	0.000	0.000	0.003	1439.538	0.274	0.001	0.275	0.095	0.026	1.416	14395	2.740	0.010	2.750	0.950	0.260	14.160
120	original	1	60x8 weeks	PCR90	MWF	VAC95	100	All	2.999	0.001	0.000	0.001	0.000	0.000	0.003	1439.538	0.274	0.001	0.275	0.095	0.026	1.416	14395	2.740	0.010	2.750	0.950	0.260	14.160
120	original	1	60x8 weeks	PCR90	MWF	VAC95	100	All	2.999	0.001	0.000	0.001	0.000	0.000	0.003	1439.538	0.274	0.001	0.275	0.095	0.026	1.416	14395	2.740	0.010	2.750	0.950	0.260	14.160
120	original	1.2	60x8 weeks	PCR90	MWF	VAC95	100	All	2.999	0.001	0.000	0.001	0.000	0.000	0.003	1439.509	0.298	0.001	0.299	0.081	0.053	1.439	14395	2.980	0.010	2.990	0.810	0.530	14.390
120	original	1.2	60x8 weeks	PCR90	MWF	VAC95	100	All	2.999	0.001	0.000	0.001	0.000	0.000	0.003	1439.509	0.298	0.001	0.299	0.081	0.053	1.439	14395	2.980	0.010	2.990	0.810	0.530	14.390
120	original	1.2	60x8 weeks	PCR90	MWF	VAC95	100	All	2.999	0.001	0.000	0.001	0.000	0.000	0.003	1439.509	0.298	0.001	0.299	0.081	0.053	1.439	14395	2.980	0.010	2.990	0.810	0.530	14.390
120	alpha	0.6	60x8 weeks	PCR90	MWF	VAC95	100	All	2.997	0.001	0.000	0.001	0.000	0.001	0.003	1438.715	0.346	0.013	0.359	0.032	0.335	1.442	14387	3.460	0.130	3.590	0.320	3.350	14.420
120	alpha	0.6	60x8 weeks	PCR90	MWF	VAC95	100	All	2.997	0.001	0.000	0.001	0.000	0.001	0.003	1438.715	0.346	0.013	0.359	0.032	0.335	1.442	14387	3.460	0.130	3.590	0.320	3.350	14.420
120	alpha	0.6	60x8 weeks	PCR90	MWF	VAC95	100	All	2.997	0.001	0.000	0.001	0.000	0.001	0.003	1438.715	0.346	0.013	0.359	0.032	0.335	1.442	14387	3.460	0.130	3.590	0.320	3.350	14.420
120	alpha	1	60x8 weeks	PCR90	MWF	VAC95	100	All	2.997	0.001	0.000	0.001	0.000	0.001	0.003	1438.528	0.377	0.015	0.392	0.038	0.364	1.493	14385	3.770	0.150	3.920	0.380	3.640	14.930
120	alpha	1	60x8 weeks	PCR90	MWF	VAC95	100	All	2.997	0.001	0.000	0.001	0.000	0.001	0.003	1438.528	0.377	0.015	0.392	0.038	0.364	1.493	14385	3.770	0.150	3.920	0.380	3.640	14.930
120	alpha	1	60x8 weeks	PCR90	MWF	VAC95	100	All	2.997	0.001	0.000	0.001	0.000	0.001	0.003	1438.528	0.377	0.015	0.392	0.038	0.364	1.493	14385	3.770	0.150	3.920	0.380	3.640	14.930
120	alpha	1.2	60x8 weeks	PCR90	MWF	VAC95	100	All	2.997	0.001	0.000	0.001	0.000	0.001	0.003	1438.581	0.357	0.029	0.386	0.044	0.357	1.439	14386	3.570	0.290	3.860	0.440	3.570	14.390
120	alpha	1.2	60x8 weeks	PCR90	MWF	VAC95	100	All	2.997	0.001	0.000	0.001	0.000	0.001	0.003	1438.581	0.357	0.029	0.386	0.044	0.357	1.439	14386	3.570	0.290	3.860	0.440	3.570	14.390
120	alpha	1.2	60x8 weeks	PCR90	MWF	VAC95	100	All	2.997	0.001	0.000	0.001	0.000	0.001	0.003	1438.581	0.357	0.029	0.386	0.044	0.357	1.439	14386	3.570	0.290	3.860	0.440	3.570	14.390
120	delta	0.6	60x8 weeks	PCR90	MWF	VAC95	100	All	2.995	0.001	0.000	0.001	0.000	0.001	0.003	1437.631	0.580	0.049	0.629	0.098	0.553	1.361	14376	5.800	0.490	6.290	0.980	5.530	13.610
120	delta	0.6	60x8 weeks	PCR90	MWF	VAC95	100	All	2.995	0.001	0.000	0.001	0.000	0.001	0.003	1437.631	0.580	0.049	0.629	0.098	0.553	1.361	14376	5.800	0.490	6.290	0.980	5.530	13.610
120	delta	0.6	60x8 weeks	PCR90	MWF	VAC95	100	All	2.995	0.001	0.000	0.001	0.000	0.001	0.003	1437.631	0.580	0.049	0.629	0.098	0.553	1.361	14376	5.800	0.490	6.290	0.980	5.530	13.610
120	delta	1	60x8 weeks	PCR90	MWF	VAC95	100	All	2.995	0.001	0.000	0.001	0.000	0.001	0.003	1437.623	0.551	0.089	0.640	0.073	0.588	1.442	14376	5.510	0.890	6.400	0.730	5.880	14.420
120	delta	1	60x8 weeks	PCR90	MWF	VAC95	100	All	2.995	0.001	0.000	0.001	0.000	0.001	0.003	1437.623	0.551	0.089	0.640	0.073	0.588	1.442	14376	5.510	0.890	6.400	0.730	5.880	14.420
120	delta	1	60x8 weeks	PCR90	MWF	VAC95	100	All	2.995	0.001	0.000	0.001	0.000	0.001	0.003	1437.623	0.551	0.089	0.640	0.073	0.588	1.442	14376	5.510	0.890	6.400	0.730	5.880	14.420
120	delta	1.2	60x8 weeks	PCR90	MWF	VAC95	100	All	2.995	0.001	0.000	0.001	0.000	0.001	0.003	1437.407	0.561	0.139	0.700	0.086	0.630	1.391	14374	5.610	1.390	7.000	0.860	6.300	13.910
120	delta	1.2	60x8 weeks	PCR90	MWF	VAC95	100	All	2.995	0.001	0.000	0.001	0.000	0.001	0.003	1437.407	0.561	0.139	0.700	0.086	0.630	1.391	14374	5.610	1.390	7.000	0.860	6.300	13.910
120	delta	1.2	60x8 weeks	PCR90	MWF	VAC95	100	All	2.995	0.001	0.000	0.001	0.000	0.001	0.003	1437.407	0.561	0.139	0.700	0.086	0.630	1.391	14374	5.610	1.390	7.000	0.860	6.300	13.910
120	original	0.6	60x8 weeks	PCR90	MWF	VAC70	25	All	2.971	0.007	0.002	0.009	0.001	0.007	0.003	1425.867	3.281	0.802	4.083	0.545	3.390	1.412	14259	32.810	8.020	40.830	5.450	33.900	14.120
120	original	0.6	60x8 weeks	PCR90	MWF	VAC70	25	All	2.971	0.007	0.002	0.009	0.001	0.007	0.003	1425.867	3.281	0.802	4.083	0.545	3.390	1.412	14259	32.810	8.020	40.830	5.450	33.900	14.120
120	original	0.6	60x8 weeks	PCR90	MWF	VAC70	25	All	2.971	0.007	0.002	0.009	0.001	0.007	0.003	1425.867	3.281	0.802	4.083	0.545	3.390	1.412	14259	32.810	8.020	40.830	5.450	33.900	14.120
120	original	1	60x8 weeks	PCR90	MWF	VAC70	25	All	2.965	0.007	0.003	0.010	0.001	0.008	0.003	1423.171	3.237	1.584	4.821	0.628	4.078	1.358	14232	32.370	15.840	48.210	6.280	40.780	13.580
120	original	1	60x8 weeks	PCR90	MWF	VAC70	25	All	2.965	0.007	0.003	0.010	0.001	0.008	0.003	1423.171	3.237	1.584	4.821	0.628	4.078	1.358	14232	32.370	15.840	48.210	6.280	40.780	13.580
120	original	1	60x8 weeks	PCR90	MWF	VAC70	25	All	2.965	0.007	0.003	0.010	0.001	0.008	0.003	1423.171	3.237	1.584	4.821	0.628	4.078	1.358	14232	32.370	15.840	48.210	6.280	40.780	13.580
120	original	1.2	60x8 weeks	PCR90	MWF	VAC70	25	All	2.960	0.007	0.005	0.011	0.001	0.010	0.003	1420.933	3.259	2.169	5.428	0.699	4.584	1.388	14209	32.590	21.690	54.280	6.990	45.840	13.880
120	original	1.2	60x8 weeks	PCR90	MWF	VAC70	25	All	2.960	0.007	0.005	0.011	0.001	0.010	0.003	1420.933	3.259	2.169	5.428	0.699	4.584	1.388	14209	32.590	21.690	54.280	6.990	45.840	13.880
120	original	1.2	60x8 weeks	PCR90	MWF	VAC70	25	All	2.960	0.007	0.005	0.011	0.001	0.010	0.003	1420.933	3.259	2.169	5.428	0.699	4.584	1.388	14209	32.590	21.690	54.280	6.990	45.840	13.880
120	alpha	0.6	60x8 weeks	PCR90	MWF	VAC70	25	All	2.958	0.007	0.005	0.011	0.001	0.011	0.003	1419.687	3.204	2.174	5.378	0.592	5.054	1.388	14197	32.040	21.740	53.780	5.920	50.540	13.880
120	alpha	0.6	60x8 weeks	PCR90	MWF	VAC70	25	All	2.958	0.007	0.005	0.011	0.001	0.011	0.003	1419.687	3.204	2.174	5.378	0.592	5.054	1.388	14197	32.040	21.740	53.780	5.920	50.540	13.880
120	alpha	0.6	60x8 weeks	PCR90	MWF	VAC70	25	All	2.958	0.007	0.005	0.011	0.001	0.011	0.003	1419.687	3.204	2.174	5.378	0.592	5.054	1.388	14197	32.040	21.740	53.780	5.920	50.540	13.880
120	alpha	1	60x8 weeks	PCR90	MWF	VAC70	25	All	2.928	0.006	0.012	0.019	0.002	0.018	0.003	1405.663	3.056	5.908	8.964	1.035	8.488	1.302	14057	30.560	59.080	89.640	10.350	84.880	13.020
120	alpha	1	60x8 weeks	PCR90	MWF	VAC70	25	All	2.928	0.006	0.012	0.019	0.002	0.018	0.003	1405.663	3.056	5.908	8.964	1.035	8.488	1.302	14057	30.560	59.080	89.640	10.350	84.880	13.020
120	alpha	1	60x8 weeks	PCR90	MWF	VAC70	25	All	2.928	0.006	0.012	0.019	0.002	0.018	0.003	1405.663	3.056	5.908	8.964	1.035	8.488	1.302	14057	30.560	59.080	89.640	10.350	84.880	13.020
120	alpha	1.2	60x8 weeks	PCR90	MWF	VAC70	25	All	2.908	0.006	0.018	0.024	0.003	0.023	0.003	1395.667	3.044	8.589	11.633	1.337	10.901	1.323	13957	30.440	85.890	116.330	13.370	109.010	13.230
120	alpha	1.2	60x8 weeks	PCR90	MWF	VAC70	25	All	2.908	0.006	0.018	0.024	0.003	0.023	0.003	1395.667	3.044	8.589	11.633	1.337	10.901	1.323	13957	30.440	85.890	116.330	13.370	109.010	13.230
120	alpha	1.2	60x8 weeks	PCR90	MWF	VAC70	25	All	2.908	0.006	0.018	0.024	0.003	0.023	0.003	1395.667	3.044	8.589	11.633	1.337	10.901	1.323	13957	30.440	85.890	116.330	13.370	109.010	13.230
120	delta	0.6	60x8 weeks	PCR90	MWF	VAC70	25	All	2.888	0.006	0.023	0.029	0.005	0.026	0.003	1386.139	2.953	11.070	14.023	2.207	12.576	1.252	13861	29.530	110.700	140.230	22.070	125.760	12.520
120	delta	0.6	60x8 weeks	PCR90	MWF	VAC70	25	All	2.888	0.006	0.023	0.029	0.005	0.026	0.003	1386.139	2.953	11.070	14.023	2.207	12.576	1.252	13861	29.530	110.700	140.230	22.070	125.760	12.520
120	delta	0.6	60x8 weeks	PCR90	MWF	VAC70	25	All	2.888	0.006	0.023	0.029	0.005	0.026	0.003	1386.139	2.953	11.070	14.023	2.207	12.576	1.252	13861	29.530	110.700	140.230	22.070	125.760	12.520
120	delta	1	60x8 weeks	PCR90	MWF	VAC70	25	All	2.680	0.005	0.074	0.079	0.014	0.071	0.002	1286.620	2.274	35.528	37.802	6.524	34.070	1.042	12866	22.740	355.280	378.020	65.240	340.700	10.420
120	delta	1	60x8 weeks	PCR90	MWF	VAC70	25	All	2.680	0.005	0.074	0.079	0.014	0.071	0.002	1286.620	2.274	35.528	37.802	6.524	34.070	1.042	12866	22.740	355.280	378.020	65.240	340.700	10.420
120	delta	1	60x8 weeks	PCR90	MWF	VAC70	25	All	2.680	0.005	0.074	0.079	0.014	0.071	0.002	1286.620	2.274	35.528	37.802	6.524	34.070	1.042	12866	22.740	355.280	378.020	65.240	340.700	10.420
120	delta	1.2	60x8 weeks	PCR90	MWF	VAC70	25	All	2.615	0.004	0.090	0.094	0.016	0.086	0.002	1255.406	2.017	43.308	45.325	7.741	41.204	0.825	12554	20.170	433.080	453.250	77.410	412.040	8.250
120	delta	1.2	60x8 weeks	PCR90	MWF	VAC70	25	All	2.615	0.004	0.090	0.094	0.016	0.086	0.002	1255.406	2.017	43.308	45.325	7.741	41.204	0.825	12554	20.170	433.080	453.250	77.410	412.040	8.250
120	delta	1.2	60x8 weeks	PCR90	MWF	VAC70	25	All	2.615	0.004	0.090	0.094	0.016	0.086	0.002	1255.406	2.017	43.308	45.325	7.741	41.204	0.825	12554	20.170	433.080	453.250	77.410	412.040	8.250
120	original	0.6	60x8 weeks	PCR90	MWF	VAC70	50	All	2.979	0.005	0.001	0.006	0.001	0.005	0.003	1429.972	2.623	0.411	3.034	0.530	2.215	1.428	14300	26.230	4.110	30.340	5.300	22.150	14.280
120	original	0.6	60x8 weeks	PCR90	MWF	VAC70	50	All	2.979	0.005	0.001	0.006	0.001	0.005	0.003	1429.972	2.623	0.411	3.034	0.530	2.215	1.428	14300	26.230	4.110	30.340	5.300	22.150	14.280
120	original	0.6	60x8 weeks	PCR90	MWF	VAC70	50	All	2.979	0.005	0.001	0.006	0.001	0.005	0.003	1429.972	2.623	0.411	3.034	0.530	2.215	1.428	14300	26.230	4.110	30.340	5.300	22.150	14.280
120	original	1	60x8 weeks	PCR90	MWF	VAC70	50	All	2.977	0.005	0.002	0.007	0.001	0.005	0.003	1428.858	2.612	0.778	3.390	0.583	2.497	1.392	14289	26.120	7.780	33.900	5.830	24.970	13.920
120	original	1	60x8 weeks	PCR90	MWF	VAC70	50	All	2.977	0.005	0.002	0.007	0.001	0.005	0.003	1428.858	2.612	0.778	3.390	0.583	2.497	1.392	14289	26.120	7.780	33.900	5.830	24.970	13.920
120	original	1	60x8 weeks	PCR90	MWF	VAC70	50	All	2.977	0.005	0.002	0.007	0.001	0.005	0.003	1428.858	2.612	0.778	3.390	0.583	2.497	1.392	14289	26.120	7.780	33.900	5.830	24.970	13.920
120	original	1.2	60x8 weeks	PCR90	MWF	VAC70	50	All	2.976	0.005	0.002	0.007	0.001	0.005	0.003	1428.444	2.528	0.977	3.505	0.616	2.590	1.417	14284	25.280	9.770	35.050	6.160	25.900	14.170
120	original	1.2	60x8 weeks	PCR90	MWF	VAC70	50	All	2.976	0.005	0.002	0.007	0.001	0.005	0.003	1428.444	2.528	0.977	3.505	0.616	2.590	1.417	14284	25.280	9.770	35.050	6.160	25.900	14.170
120	original	1.2	60x8 weeks	PCR90	MWF	VAC70	50	All	2.976	0.005	0.002	0.007	0.001	0.005	0.003	1428.444	2.528	0.977	3.505	0.616	2.590	1.417	14284	25.280	9.770	35.050	6.160	25.900	14.170
120	alpha	0.6	60x8 weeks	PCR90	MWF	VAC70	50	All	2.971	0.005	0.002	0.008	0.001	0.007	0.003	1426.159	2.496	1.189	3.685	0.418	3.453	1.376	14262	24.960	11.890	36.850	4.180	34.530	13.760
120	alpha	0.6	60x8 weeks	PCR90	MWF	VAC70	50	All	2.971	0.005	0.002	0.008	0.001	0.007	0.003	1426.159	2.496	1.189	3.685	0.418	3.453	1.376	14262	24.960	11.890	36.850	4.180	34.530	13.760
120	alpha	0.6	60x8 weeks	PCR90	MWF	VAC70	50	All	2.971	0.005	0.002	0.008	0.001	0.007	0.003	1426.159	2.496	1.189	3.685	0.418	3.453	1.376	14262	24.960	11.890	36.850	4.180	34.530	13.760
120	alpha	1	60x8 weeks	PCR90	MWF	VAC70	50	All	2.961	0.005	0.005	0.010	0.001	0.010	0.003	1421.459	2.513	2.504	5.017	0.577	4.646	1.365	14215	25.130	25.040	50.170	5.770	46.460	13.650
120	alpha	1	60x8 weeks	PCR90	MWF	VAC70	50	All	2.961	0.005	0.005	0.010	0.001	0.010	0.003	1421.459	2.513	2.504	5.017	0.577	4.646	1.365	14215	25.130	25.040	50.170	5.770	46.460	13.650
120	alpha	1	60x8 weeks	PCR90	MWF	VAC70	50	All	2.961	0.005	0.005	0.010	0.001	0.010	0.003	1421.459	2.513	2.504	5.017	0.577	4.646	1.365	14215	25.130	25.040	50.170	5.770	46.460	13.650
120	alpha	1.2	60x8 weeks	PCR90	MWF	VAC70	50	All	2.951	0.005	0.008	0.013	0.001	0.012	0.003	1416.646	2.485	3.620	6.105	0.673	5.788	1.373	14166	24.850	36.200	61.050	6.730	57.880	13.730
120	alpha	1.2	60x8 weeks	PCR90	MWF	VAC70	50	All	2.951	0.005	0.008	0.013	0.001	0.012	0.003	1416.646	2.485	3.620	6.105	0.673	5.788	1.373	14166	24.850	36.200	61.050	6.730	57.880	13.730
120	alpha	1.2	60x8 weeks	PCR90	MWF	VAC70	50	All	2.951	0.005	0.008	0.013	0.001	0.012	0.003	1416.646	2.485	3.620	6.105	0.673	5.788	1.373	14166	24.850	36.200	61.050	6.730	57.880	13.730
120	delta	0.6	60x8 weeks	PCR90	MWF	VAC70	50	All	2.939	0.006	0.010	0.016	0.002	0.014	0.003	1410.827	2.743	4.953	7.696	1.192	6.885	1.368	14108	27.430	49.530	76.960	11.920	68.850	13.680
120	delta	0.6	60x8 weeks	PCR90	MWF	VAC70	50	All	2.939	0.006	0.010	0.016	0.002	0.014	0.003	1410.827	2.743	4.953	7.696	1.192	6.885	1.368	14108	27.430	49.530	76.960	11.920	68.850	13.680

120	delta	0.6	60x8 weeks	PCR90	MWF	VAC70	50	All	2.939	0.006	0.010	0.016	0.002	0.014	0.003	1410.827	2.743	4.953	7.696	1.192	6.885	1.368	14108	27.430	49.530	76.960	11.920	68.850	13.680
120	delta	1	60x8 weeks	PCR90	MWF	VAC70	50	All	2.842	0.005	0.035	0.040	0.007	0.036	0.003	1364.169	2.425	16.974	19.399	3.194	17.296	1.236	13642	24.250	169.740	193.990	31.940	172.960	12.360
120	delta	1	60x8 weeks	PCR90	MWF	VAC70	50	All	2.842	0.005	0.035	0.040	0.007	0.036	0.003	1364.169	2.425	16.974	19.399	3.194	17.296	1.236	13642	24.250	169.740	193.990	31.940	172.960	12.360
120	delta	1	60x8 weeks	PCR90	MWF	VAC70	50	All	2.842	0.005	0.035	0.040	0.007	0.036	0.003	1364.169	2.425	16.974	19.399	3.194	17.296	1.236	13642	24.250	169.740	193.990	31.940	172.960	12.360
120	delta	1.2	60x8 weeks	PCR90	MWF	VAC70	50	All	2.785	0.004	0.050	0.055	0.009	0.049	0.002	1336.994	2.144	24.126	26.270	4.421	23.461	1.189	13370	21.440	241.260	262.700	44.210	234.610	11.890
120	delta	1.2	60x8 weeks	PCR90	MWF	VAC70	50	All	2.785	0.004	0.050	0.055	0.009	0.049	0.002	1336.994	2.144	24.126	26.270	4.421	23.461	1.189	13370	21.440	241.260	262.700	44.210	234.610	11.890
120	delta	1.2	60x8 weeks	PCR90	MWF	VAC70	50	All	2.785	0.004	0.050	0.055	0.009	0.049	0.002	1336.994	2.144	24.126	26.270	4.421	23.461	1.189	13370	21.440	241.260	262.700	44.210	234.610	11.890
120	original	0.6	60x8 weeks	PCR90	MWF	VAC70	75	All	2.987	0.004	0.000	0.004	0.001	0.002	0.003	1433.906	1.886	0.181	2.067	0.557	1.104	1.445	14339	18.860	1.810	20.670	5.570	11.040	14.450
120	original	0.6	60x8 weeks	PCR90	MWF	VAC70	75	All	2.987	0.004	0.000	0.004	0.001	0.002	0.003	1433.906	1.886	0.181	2.067	0.557	1.104	1.445	14339	18.860	1.810	20.670	5.570	11.040	14.450
120	original	0.6	60x8 weeks	PCR90	MWF	VAC70	75	All	2.987	0.004	0.000	0.004	0.001	0.002	0.003	1433.906	1.886	0.181	2.067	0.557	1.104	1.445	14339	18.860	1.810	20.670	5.570	11.040	14.450
120	original	1	60x8 weeks	PCR90	MWF	VAC70	75	All	2.987	0.004	0.001	0.005	0.001	0.002	0.003	1433.586	1.952	0.257	2.209	0.584	1.168	1.400	14336	19.520	2.570	22.090	5.840	11.680	14.000
120	original	1	60x8 weeks	PCR90	MWF	VAC70	75	All	2.987	0.004	0.001	0.005	0.001	0.002	0.003	1433.586	1.952	0.257	2.209	0.584	1.168	1.400	14336	19.520	2.570	22.090	5.840	11.680	14.000
120	original	1	60x8 weeks	PCR90	MWF	VAC70	75	All	2.987	0.004	0.001	0.005	0.001	0.002	0.003	1433.586	1.952	0.257	2.209	0.584	1.168	1.400	14336	19.520	2.570	22.090	5.840	11.680	14.000
120	original	1.2	60x8 weeks	PCR90	MWF	VAC70	75	All	2.987	0.004	0.001	0.004	0.001	0.002	0.003	1433.731	1.857	0.289	2.146	0.564	1.129	1.372	14337	18.570	2.890	21.460	5.640	11.290	13.720
120	original	1.2	60x8 weeks	PCR90	MWF	VAC70	75	All	2.987	0.004	0.001	0.004	0.001	0.002	0.003	1433.731	1.857	0.289	2.146	0.564	1.129	1.372	14337	18.570	2.890	21.460	5.640	11.290	13.720
120	original	1.2	60x8 weeks	PCR90	MWF	VAC70	75	All	2.987	0.004	0.001	0.004	0.001	0.002	0.003	1433.731	1.857	0.289	2.146	0.564	1.129	1.372	14337	18.570	2.890	21.460	5.640	11.290	13.720
120	alpha	0.6	60x8 weeks	PCR90	MWF	VAC70	75	All	2.981	0.004	0.001	0.005	0.001	0.005	0.003	1431.081	1.899	0.473	2.372	0.276	2.210	1.372	14311	18.990	4.730	23.720	2.760	22.100	13.720
120	alpha	0.6	60x8 weeks	PCR90	MWF	VAC70	75	All	2.981	0.004	0.001	0.005	0.001	0.005	0.003	1431.081	1.899	0.473	2.372	0.276	2.210	1.372	14311	18.990	4.730	23.720	2.760	22.100	13.720
120	alpha	0.6	60x8 weeks	PCR90	MWF	VAC70	75	All	2.981	0.004	0.001	0.005	0.001	0.005	0.003	1431.081	1.899	0.473	2.372	0.276	2.210	1.372	14311	18.990	4.730	23.720	2.760	22.100	13.720
120	alpha	1	60x8 weeks	PCR90	MWF	VAC70	75	All	2.978	0.004	0.002	0.006	0.001	0.005	0.003	1429.474	1.933	0.918	2.851	0.324	2.634	1.391	14295	19.330	9.180	28.510	3.240	26.340	13.910
120	alpha	1	60x8 weeks	PCR90	MWF	VAC70	75	All	2.978	0.004	0.002	0.006	0.001	0.005	0.003	1429.474	1.933	0.918	2.851	0.324	2.634	1.391	14295	19.330	9.180	28.510	3.240	26.340	13.910
120	alpha	1	60x8 weeks	PCR90	MWF	VAC70	75	All	2.978	0.004	0.002	0.006	0.001	0.005	0.003	1429.474	1.933	0.918	2.851	0.324	2.634	1.391	14295	19.330	9.180	28.510	3.240	26.340	13.910
120	alpha	1.2	60x8 weeks	PCR90	MWF	VAC70	75	All	2.974	0.004	0.003	0.007	0.001	0.006	0.003	1427.473	1.972	1.320	3.292	0.382	3.071	1.380	14275	19.720	13.200	32.920	3.820	30.710	13.800
120	alpha	1.2	60x8 weeks	PCR90	MWF	VAC70	75	All	2.974	0.004	0.003	0.007	0.001	0.006	0.003	1427.473	1.972	1.320	3.292	0.382	3.071	1.380	14275	19.720	13.200	32.920	3.820	30.710	13.800
120	alpha	1.2	60x8 weeks	PCR90	MWF	VAC70	75	All	2.974	0.004	0.003	0.007	0.001	0.006	0.003	1427.473	1.972	1.320	3.292	0.382	3.071	1.380	14275	19.720	13.200	32.920	3.820	30.710	13.800
120	delta	0.6	60x8 weeks	PCR90	MWF	VAC70	75	All	2.968	0.005	0.004	0.008	0.001	0.007	0.003	1424.645	2.190	1.842	4.032	0.655	3.571	1.408	14246	21.900	18.420	40.320	6.550	35.710	14.080
120	delta	0.6	60x8 weeks	PCR90	MWF	VAC70	75	All	2.968	0.005	0.004	0.008	0.001	0.007	0.003	1424.645	2.190	1.842	4.032	0.655	3.571	1.408	14246	21.900	18.420	40.320	6.550	35.710	14.080
120	delta	0.6	60x8 weeks	PCR90	MWF	VAC70	75	All	2.968	0.005	0.004	0.008	0.001	0.007	0.003	1424.645	2.190	1.842	4.032	0.655	3.571	1.408	14246	21.900	18.420	40.320	6.550	35.710	14.080
120	delta	1	60x8 weeks	PCR90	MWF	VAC70	75	All	2.945	0.004	0.010	0.014	0.002	0.013	0.003	1413.434	2.155	4.709	6.864	1.141	6.124	1.331	14134	21.550	47.090	68.640	11.410	61.240	13.310
120	delta	1	60x8 weeks	PCR90	MWF	VAC70	75	All	2.945	0.004	0.010	0.014	0.002	0.013	0.003	1413.434	2.155	4.709	6.864	1.141	6.124	1.331	14134	21.550	47.090	68.640	11.410	61.240	13.310
120	delta	1	60x8 weeks	PCR90	MWF	VAC70	75	All	2.945	0.004	0.010	0.014	0.002	0.013	0.003	1413.434	2.155	4.709	6.864	1.141	6.124	1.331	14134	21.550	47.090	68.640	11.410	61.240	13.310
120	delta	1.2	60x8 weeks	PCR90	MWF	VAC70	75	All	2.924	0.004	0.016	0.020	0.003	0.018	0.003	1403.323	2.068	7.757	9.825	1.537	8.747	1.302	14033	20.680	77.570	98.250	15.370	87.470	13.020
120	delta	1.2	60x8 weeks	PCR90	MWF	VAC70	75	All	2.924	0.004	0.016	0.020	0.003	0.018	0.003	1403.323	2.068	7.757	9.825	1.537	8.747	1.302	14033	20.680	77.570	98.250	15.370	87.470	13.020
120	delta	1.2	60x8 weeks	PCR90	MWF	VAC70	75	All	2.924	0.004	0.016	0.020	0.003	0.018	0.003	1403.323	2.068	7.757	9.825	1.537	8.747	1.302	14033	20.680	77.570	98.250	15.370	87.470	13.020
120	original	0.6	60x8 weeks	PCR90	MWF	VAC70	100	All	2.995	0.003	0.000	0.003	0.001	0.000	0.003	1437.433	1.287	0.009	1.296	0.508	0.171	1.406	14374	12.870	0.090	12.960	5.080	1.710	14.060
120	original	0.6	60x8 weeks	PCR90	MWF	VAC70	100	All	2.995	0.003	0.000	0.003	0.001	0.000	0.003	1437.433	1.287	0.009	1.296	0.508	0.171	1.406	14374	12.870	0.090	12.960	5.080	1.710	14.060
120	original	0.6	60x8 weeks	PCR90	MWF	VAC70	100	All	2.995	0.003	0.000	0.003	0.001	0.000	0.003	1437.433	1.287	0.009	1.296	0.508	0.171	1.406	14374	12.870	0.090	12.960	5.080	1.710	14.060
120	original	1	60x8 weeks	PCR90	MWF	VAC70	100	All	2.994	0.003	0.000	0.003	0.001	0.000	0.003	1437.155	1.314	0.015	1.329	0.541	0.206	1.485	14372	13.140	0.150	13.290	5.410	2.060	14.850
120	original	1	60x8 weeks	PCR90	MWF	VAC70	100	All	2.994	0.003	0.000	0.003	0.001	0.000	0.003	1437.155	1.314	0.015	1.329	0.541	0.206	1.485	14372	13.140	0.150	13.290	5.410	2.060	14.850
120	original	1	60x8 weeks	PCR90	MWF	VAC70	100	All	2.994	0.003	0.000	0.003	0.001	0.000	0.003	1437.155	1.314	0.015	1.329	0.541	0.206	1.485	14372	13.140	0.150	13.290	5.410	2.060	14.850
120	original	1.2	60x8 weeks	PCR90	MWF	VAC70	100	All	2.994	0.003	0.000	0.003	0.001	0.000	0.003	1437.240	1.299	0.014	1.313	0.543	0.184	1.369	14372	12.990	0.140	13.130	5.430	1.840	13.690
120	original	1.2	60x8 weeks	PCR90	MWF	VAC70	100	All	2.994	0.003	0.000	0.003	0.001	0.000	0.003	1437.240	1.299	0.014	1.313	0.543	0.184	1.369	14372	12.990	0.140	13.130	5.430	1.840	13.690
120	original	1.2	60x8 weeks	PCR90	MWF	VAC70	100	All	2.994	0.003	0.000	0.003	0.001	0.000	0.003	1437.240	1.299	0.014	1.313	0.543	0.184	1.369	14372	12.990	0.140	13.130	5.430	1.840	13.690
120	alpha	0.6	60x8 weeks	PCR90	MWF	VAC70	100	All	2.989	0.003	0.000	0.003	0.000	0.003	0.003	1434.830	1.245	0.124	1.369	0.156	1.265	1.492	14348	12.450	1.240	13.690	1.560	12.650	14.920
120	alpha	0.6	60x8 weeks	PCR90	MWF	VAC70	100	All	2.989	0.003	0.000	0.003	0.000	0.003	0.003	1434.830	1.245	0.124	1.369	0.156	1.265	1.492	14348	12.450	1.240	13.690	1.560	12.650	14.920
120	alpha	0.6	60x8 weeks	PCR90	MWF	VAC70	100	All	2.989	0.003	0.000	0.003	0.000	0.003	0.003	1434.830	1.245	0.124	1.369	0.156	1.265	1.492	14348	12.450	1.240	13.690	1.560	12.650	14.920
120	alpha	1	60x8 weeks	PCR90	MWF	VAC70	100	All	2.988	0.003	0.001	0.003	0.000	0.003	0.003	1434.351	1.279	0.258	1.537	0.189	1.370	1.446	14344	12.790	2.580	15.370	1.890	13.700	14.460
120	alpha	1	60x8 weeks	PCR90	MWF	VAC70	100	All	2.988	0.003	0.001	0.003	0.000	0.003	0.003	1434.351	1.279	0.258	1.537	0.189	1.370	1.446	14344	12.790	2.580	15.370	1.890	13.700	14.460
120	alpha	1	60x8 weeks	PCR90	MWF	VAC70	100	All	2.988	0.003	0.001	0.003	0.000	0.003	0.003	1434.351	1.279	0.258	1.537	0.189	1.370	1.446	14344	12.790	2.580	15.370	1.890	13.700	14.460
120	alpha	1.2	60x8 weeks	PCR90	MWF	VAC70	100	All	2.988	0.003	0.001	0.003	0.000	0.003	0.003	1434.472	1.218	0.267	1.485	0.172	1.355	1.397	14345	12.180	2.670	14.850	1.720	13.550	13.970
120	alpha	1.2	60x8 weeks	PCR90	MWF	VAC70	100	All	2.988	0.003	0.001	0.003	0.000	0.003	0.003	1434.472	1.218	0.267	1.485	0.172	1.355	1.397	14345	12.180	2.670	14.850	1.720	13.550	13.970
120	alpha	1.2	60x8 weeks	PCR90	MWF	VAC70	100	All	2.988	0.003	0.001	0.003	0.000	0.003	0.003	1434.472	1.218	0.267	1.485	0.172	1.355	1.397	14345	12.180	2.670	14.850	1.720	13.550	13.970
120	delta	0.6	60x8 weeks	PCR90	MWF	VAC70	100	All	2.984	0.003	0.001	0.004	0.001	0.004	0.003	1432.275	1.577	0.497	2.074	0.302	1.826	1.424	14323	15.770	4.970	20.740	3.020	18.260	14.240
120	delta	0.6	60x8 weeks	PCR90	MWF	VAC70	100	All	2.984	0.003	0.001	0.004	0.001	0.004	0.003	1432.275	1.577	0.497	2.074	0.302	1.826	1.424	14323	15.770	4.970	20.740	3.020	18.260	14.240
120	delta	0.6	60x8 weeks	PCR90	MWF	VAC70	100	All	2.984	0.003	0.001	0.004	0.001	0.004	0.003	1432.275	1.577	0.497	2.074	0.302	1.826	1.424	14323	15.770	4.970	20.740	3.020	18.260	14.240
120	delta	1	60x8 weeks	PCR90	MWF	VAC70	100	All	2.979	0.003	0.002	0.006	0.001	0.005	0.003	1429.746	1.647	1.144	2.791	0.417	2.446	1.434	14297	16.470	11.440	27.910	4.170	24.460	14.340
120	delta	1	60x8 weeks	PCR90	MWF	VAC70	100	All	2.979	0.003	0.002	0.006	0.001	0.005	0.003	1429.746	1.647	1.144	2.791	0.417	2.446	1.434	14297	16.470	11.440	27.910	4.170	24.460	14.340
120	delta	1	60x8 weeks	PCR90	MWF	VAC70	100	All	2.979	0.003	0.002	0.006	0.001	0.005	0.003	1429.746	1.647	1.144	2.791	0.417	2.446	1.434	14297	16.470	11.440	27.910	4.170	24.460	14.340
120	delta	1.2	60x8 weeks	PCR90	MWF	VAC70	100	All	2.976	0.003	0.003	0.007	0.001	0.006	0.003	1428.297	1.589	1.570	3.159	0.480	2.813	1.399	14283	15.890	15.700	31.590	4.800	28.130	13.990
120	delta	1.2	60x8 weeks	PCR90	MWF	VAC70	100	All	2.976	0.003	0.003	0.007	0.001	0.006	0.003	1428.297	1.589	1.570	3.159	0.480	2.813	1.399	14283	15.890	15.700	31.590	4.800	28.130	13.990
120	delta	1.2	60x8 weeks	PCR90	MWF	VAC70	100	All	2.976	0.003	0.003	0.007	0.001	0.006	0.003	1428.297	1.589	1.570	3.159	0.480	2.813	1.399	14283	15.890	15.700	31.590	4.800	28.130	13.990
120	original	0.6	60x8 weeks	PCR90	MWF	VAC50	25	All	2.969	0.007	0.002	0.009	0.001	0.007	0.003	1425.246	3.417	0.839	4.256	0.583	3.499	1.467	14252	34.170	8.390	42.560	5.830	34.990	14.670
120	original	0.6	60x8 weeks	PCR90	MWF	VAC50	25	All	2.969	0.007	0.002	0.009	0.001	0.007	0.003	1425.246	3.417	0.839	4.256	0.583	3.499	1.467	14252	34.170	8.390	42.560	5.830	34.990	14.670
120	original	0.6	60x8 weeks	PCR90	MWF	VAC50	25	All	2.969	0.007	0.002	0.009	0.001	0.007	0.003	1425.246	3.417	0.839	4.256	0.583	3.499	1.467	14252	34.170	8.390	42.560	5.830	34.990	14.670
120	original	1	60x8 weeks	PCR90	MWF	VAC50	25	All	2.962	0.007	0.004	0.011	0.002	0.009	0.003	1421.953	3.376	1.853	5.229	0.796	4.220	1.371	14220	33.760	18.530	52.290	7.960	42.200	13.710
120	original	1	60x8 weeks	PCR90	MWF	VAC50	25	All	2.962	0.007	0.004	0.011	0.002	0.009	0.003	1421.953	3.376	1.853	5.229	0.796	4.220	1.371	14220	33.760	18.530	52.290	7.960	42.200	13.710
120	original	1	60x8 weeks	PCR90	MWF	VAC50	25	All	2.962	0.007	0.004	0.011	0.002	0.009	0.003	1421.953	3.376	1.853	5.229	0.796	4.220	1.371	14220	33.760	18.530	52.290	7.960	42.200	13.710
120	original	1.2	60x8 weeks	PCR90	MWF	VAC50	25	All	2.959	0.007	0.005	0.012	0.002	0.009	0.003	1420.474	3.425	2.236	5.661	0.895	4.517	1.351	14205	34.250	22.360	56.610	8.950	45.170	13.510
120	original	1.2	60x8 weeks	PCR90	MWF	VAC50	25	All	2.959	0.007	0.005	0.012	0.002	0.009	0.003	1420.474	3.425	2.236	5.661	0.895	4.517	1.351	14205	34.250	22.360	56.610	8.950	45.170	13.510
120	original	1.2	60x8 weeks	PCR90	MWF	VAC50	25	All	2.959	0.007	0.005	0.012	0.002	0.009	0.003	1420.474	3.425	2.236	5.661	0.895	4.517	1.351	14205	34.250	22.360	56.610	8.950	45.170	13.510
120	alpha	0.6	60x8 weeks	PCR90	MWF	VAC50	25	All	2.952	0.007	0.005	0.012	0.001	0.012	0.003	1417.155	3.448	2.547	5.995	0.650	5.686	1.346	14172	34.480	25.470	59.950	6.500	56.860	13.460

120	alpha	0.6	60x8 weeks	PCR90	MWF	VAC50	25	All	2.952	0.007	0.005	0.012	0.001	0.012	0.003	1417.155	3.448	2.547	5.995	0.650	5.686	1.346	14172	34.480	25.470	59.950	6.500	56.860	13.460
120	alpha	0.6	60x8 weeks	PCR90	MWF	VAC50	25	All	2.952	0.007	0.005	0.012	0.001	0.012	0.003	1417.155	3.448	2.547	5.995	0.650	5.686	1.346	14172	34.480	25.470	59.950	6.500	56.860	13.460
120	alpha	1	60x8 weeks	PCR90	MWF	VAC50	25	All	2.922	0.007	0.014	0.020	0.002	0.019	0.003	1402.796	3.261	6.500	9.761	1.155	9.137	1.363	14028	32.610	65.000	97.610	11.550	91.370	13.630
120	alpha	1	60x8 weeks	PCR90	MWF	VAC50	25	All	2.922	0.007	0.014	0.020	0.002	0.019	0.003	1402.796	3.261	6.500	9.761	1.155	9.137	1.363	14028	32.610	65.000	97.610	11.550	91.370	13.630
120	alpha	1	60x8 weeks	PCR90	MWF	VAC50	25	All	2.922	0.007	0.014	0.020	0.002	0.019	0.003	1402.796	3.261	6.500	9.761	1.155	9.137	1.363	14028	32.610	65.000	97.610	11.550	91.370	13.630
120	alpha	1.2	60x8 weeks	PCR90	MWF	VAC50	25	All	2.897	0.007	0.021	0.027	0.003	0.025	0.003	1390.421	3.168	9.906	13.074	1.578	12.151	1.251	13904	31.680	99.060	130.740	15.780	121.510	12.510
120	alpha	1.2	60x8 weeks	PCR90	MWF	VAC50	25	All	2.897	0.007	0.021	0.027	0.003	0.025	0.003	1390.421	3.168	9.906	13.074	1.578	12.151	1.251	13904	31.680	99.060	130.740	15.780	121.510	12.510
120	alpha	1.2	60x8 weeks	PCR90	MWF	VAC50	25	All	2.897	0.007	0.021	0.027	0.003	0.025	0.003	1390.421	3.168	9.906	13.074	1.578	12.151	1.251	13904	31.680	99.060	130.740	15.780	121.510	12.510
120	delta	0.6	60x8 weeks	PCR90	MWF	VAC50	25	All	2.857	0.007	0.030	0.037	0.006	0.033	0.003	1371.586	3.187	14.628	17.815	2.955	15.859	1.221	13716	31.870	146.280	178.150	29.550	158.590	12.210
120	delta	0.6	60x8 weeks	PCR90	MWF	VAC50	25	All	2.857	0.007	0.030	0.037	0.006	0.033	0.003	1371.586	3.187	14.628	17.815	2.955	15.859	1.221	13716	31.870	146.280	178.150	29.550	158.590	12.210
120	delta	0.6	60x8 weeks	PCR90	MWF	VAC50	25	All	2.857	0.007	0.030	0.037	0.006	0.033	0.003	1371.586	3.187	14.628	17.815	2.955	15.859	1.221	13716	31.870	146.280	178.150	29.550	158.590	12.210
120	delta	1	60x8 weeks	PCR90	MWF	VAC50	25	All	2.636	0.005	0.086	0.091	0.015	0.082	0.002	1265.447	2.270	41.290	43.560	7.419	39.330	0.866	12654	22.700	412.900	435.600	74.190	393.300	8.660
120	delta	1	60x8 weeks	PCR90	MWF	VAC50	25	All	2.636	0.005	0.086	0.091	0.015	0.082	0.002	1265.447	2.270	41.290	43.560	7.419	39.330	0.866	12654	22.700	412.900	435.600	74.190	393.300	8.660
120	delta	1	60x8 weeks	PCR90	MWF	VAC50	25	All	2.636	0.005	0.086	0.091	0.015	0.082	0.002	1265.447	2.270	41.290	43.560	7.419	39.330	0.866	12654	22.700	412.900	435.600	74.190	393.300	8.660
120	delta	1.2	60x8 weeks	PCR90	MWF	VAC50	25	All	2.557	0.004	0.102	0.106	0.019	0.097	0.002	1227.380	2.014	48.768	50.782	8.916	46.373	0.791	12274	20.140	487.680	507.820	89.160	463.730	7.910
120	delta	1.2	60x8 weeks	PCR90	MWF	VAC50	25	All	2.557	0.004	0.102	0.106	0.019	0.097	0.002	1227.380	2.014	48.768	50.782	8.916	46.373	0.791	12274	20.140	487.680	507.820	89.160	463.730	7.910
120	delta	1.2	60x8 weeks	PCR90	MWF	VAC50	25	All	2.557	0.004	0.102	0.106	0.019	0.097	0.002	1227.380	2.014	48.768	50.782	8.916	46.373	0.791	12274	20.140	487.680	507.820	89.160	463.730	7.910
120	original	0.6	60x8 weeks	PCR90	MWF	VAC50	50	All	2.977	0.006	0.001	0.007	0.002	0.005	0.003	1428.729	3.034	0.520	3.554	0.765	2.356	1.383	14287	30.340	5.200	35.540	7.650	23.560	13.830
120	original	0.6	60x8 weeks	PCR90	MWF	VAC50	50	All	2.977	0.006	0.001	0.007	0.002	0.005	0.003	1428.729	3.034	0.520	3.554	0.765	2.356	1.383	14287	30.340	5.200	35.540	7.650	23.560	13.830
120	original	0.6	60x8 weeks	PCR90	MWF	VAC50	50	All	2.977	0.006	0.001	0.007	0.002	0.005	0.003	1428.729	3.034	0.520	3.554	0.765	2.356	1.383	14287	30.340	5.200	35.540	7.650	23.560	13.830
120	original	1	60x8 weeks	PCR90	MWF	VAC50	50	All	2.974	0.006	0.002	0.008	0.002	0.005	0.003	1427.626	2.952	0.913	3.865	0.835	2.548	1.318	14276	29.520	9.130	38.650	8.350	25.480	13.180
120	original	1	60x8 weeks	PCR90	MWF	VAC50	50	All	2.974	0.006	0.002	0.008	0.002	0.005	0.003	1427.626	2.952	0.913	3.865	0.835	2.548	1.318	14276	29.520	9.130	38.650	8.350	25.480	13.180
120	original	1	60x8 weeks	PCR90	MWF	VAC50	50	All	2.974	0.006	0.002	0.008	0.002	0.005	0.003	1427.626	2.952	0.913	3.865	0.835	2.548	1.318	14276	29.520	9.130	38.650	8.350	25.480	13.180
120	original	1.2	60x8 weeks	PCR90	MWF	VAC50	50	All	2.974	0.006	0.002	0.008	0.002	0.005	0.003	1427.336	2.872	1.075	3.947	0.904	2.535	1.464	14273	28.720	10.750	39.470	9.040	25.350	14.640
120	original	1.2	60x8 weeks	PCR90	MWF	VAC50	50	All	2.974	0.006	0.002	0.008	0.002	0.005	0.003	1427.336	2.872	1.075	3.947	0.904	2.535	1.464	14273	28.720	10.750	39.470	9.040	25.350	14.640
120	original	1.2	60x8 weeks	PCR90	MWF	VAC50	50	All	2.974	0.006	0.002	0.008	0.002	0.005	0.003	1427.336	2.872	1.075	3.947	0.904	2.535	1.464	14273	28.720	10.750	39.470	9.040	25.350	14.640
120	alpha	0.6	60x8 weeks	PCR90	MWF	VAC50	50	All	2.967	0.006	0.003	0.009	0.001	0.008	0.003	1423.985	2.873	1.342	4.215	0.478	3.956	1.412	14240	28.730	13.420	42.150	4.780	39.560	14.120
120	alpha	0.6	60x8 weeks	PCR90	MWF	VAC50	50	All	2.967	0.006	0.003	0.009	0.001	0.008	0.003	1423.985	2.873	1.342	4.215	0.478	3.956	1.412	14240	28.730	13.420	42.150	4.780	39.560	14.120
120	alpha	0.6	60x8 weeks	PCR90	MWF	VAC50	50	All	2.967	0.006	0.003	0.009	0.001	0.008	0.003	1423.985	2.873	1.342	4.215	0.478	3.956	1.412	14240	28.730	13.420	42.150	4.780	39.560	14.120
120	alpha	1	60x8 weeks	PCR90	MWF	VAC50	50	All	2.950	0.006	0.007	0.013	0.002	0.012	0.003	1416.060	2.885	3.341	6.226	0.728	5.862	1.310	14161	28.850	33.410	62.260	7.280	58.620	13.100
120	alpha	1	60x8 weeks	PCR90	MWF	VAC50	50	All	2.950	0.006	0.007	0.013	0.002	0.012	0.003	1416.060	2.885	3.341	6.226	0.728	5.862	1.310	14161	28.850	33.410	62.260	7.280	58.620	13.100
120	alpha	1	60x8 weeks	PCR90	MWF	VAC50	50	All	2.950	0.006	0.007	0.013	0.002	0.012	0.003	1416.060	2.885	3.341	6.226	0.728	5.862	1.310	14161	28.850	33.410	62.260	7.280	58.620	13.100
120	alpha	1.2	60x8 weeks	PCR90	MWF	VAC50	50	All	2.937	0.006	0.011	0.017	0.002	0.016	0.003	1409.976	2.895	5.059	7.954	0.876	7.513	1.312	14100	28.950	50.590	79.540	8.760	75.130	13.120
120	alpha	1.2	60x8 weeks	PCR90	MWF	VAC50	50	All	2.937	0.006	0.011	0.017	0.002	0.016	0.003	1409.976	2.895	5.059	7.954	0.876	7.513	1.312	14100	28.950	50.590	79.540	8.760	75.130	13.120
120	alpha	1.2	60x8 weeks	PCR90	MWF	VAC50	50	All	2.937	0.006	0.011	0.017	0.002	0.016	0.003	1409.976	2.895	5.059	7.954	0.876	7.513	1.312	14100	28.950	50.590	79.540	8.760	75.130	13.120
120	delta	0.6	60x8 weeks	PCR90	MWF	VAC50	50	All	2.909	0.006	0.017	0.023	0.004	0.021	0.003	1396.484	3.075	8.185	11.260	1.920	9.992	1.375	13965	30.750	81.850	112.600	19.200	99.920	13.750
120	delta	0.6	60x8 weeks	PCR90	MWF	VAC50	50	All	2.909	0.006	0.017	0.023	0.004	0.021	0.003	1396.484	3.075	8.185	11.260	1.920	9.992	1.375	13965	30.750	81.850	112.600	19.200	99.920	13.750
120	delta	0.6	60x8 weeks	PCR90	MWF	VAC50	50	All	2.909	0.006	0.017	0.023	0.004	0.021	0.003	1396.484	3.075	8.185	11.260	1.920	9.992	1.375	13965	30.750	81.850	112.600	19.200	99.920	13.750
120	delta	1	60x8 weeks	PCR90	MWF	VAC50	50	All	2.754	0.005	0.057	0.063	0.011	0.056	0.002	1322.021	2.581	27.518	30.099	5.090	26.928	1.016	13220	25.810	275.180	300.990	50.900	269.280	10.160
120	delta	1	60x8 weeks	PCR90	MWF	VAC50	50	All	2.754	0.005	0.057	0.063	0.011	0.056	0.002	1322.021	2.581	27.518	30.099	5.090	26.928	1.016	13220	25.810	275.180	300.990	50.900	269.280	10.160
120	delta	1	60x8 weeks	PCR90	MWF	VAC50	50	All	2.754	0.005	0.057	0.063	0.011	0.056	0.002	1322.021	2.581	27.518	30.099	5.090	26.928	1.016	13220	25.810	275.180	300.990	50.900	269.280	10.160
120	delta	1.2	60x8 weeks	PCR90	MWF	VAC50	50	All	2.666	0.005	0.079	0.084	0.014	0.075	0.002	1279.485	2.205	37.960	40.165	6.881	35.990	0.956	12795	22.050	379.600	401.650	68.810	359.900	9.560
120	delta	1.2	60x8 weeks	PCR90	MWF	VAC50	50	All	2.666	0.005	0.079	0.084	0.014	0.075	0.002	1279.485	2.205	37.960	40.165	6.881	35.990	0.956	12795	22.050	379.600	401.650	68.810	359.900	9.560
120	delta	1.2	60x8 weeks	PCR90	MWF	VAC50	50	All	2.666	0.005	0.079	0.084	0.014	0.075	0.002	1279.485	2.205	37.960	40.165	6.881	35.990	0.956	12795	22.050	379.600	401.650	68.810	359.900	9.560
120	original	0.6	60x8 weeks	PCR90	MWF	VAC50	75	All	2.985	0.005	0.000	0.006	0.002	0.003	0.003	1432.619	2.447	0.232	2.679	0.774	1.228	1.380	14326	24.470	2.320	26.790	7.740	12.280	13.800
120	original	0.6	60x8 weeks	PCR90	MWF	VAC50	75	All	2.985	0.005	0.000	0.006	0.002	0.003	0.003	1432.619	2.447	0.232	2.679	0.774	1.228	1.380	14326	24.470	2.320	26.790	7.740	12.280	13.800
120	original	0.6	60x8 weeks	PCR90	MWF	VAC50	75	All	2.985	0.005	0.000	0.006	0.002	0.003	0.003	1432.619	2.447	0.232	2.679	0.774	1.228	1.380	14326	24.470	2.320	26.790	7.740	12.280	13.800
120	original	1	60x8 weeks	PCR90	MWF	VAC50	75	All	2.984	0.005	0.001	0.006	0.002	0.003	0.003	1432.482	2.427	0.310	2.737	0.804	1.233	1.476	14325	24.270	3.100	27.370	8.040	12.330	14.760
120	original	1	60x8 weeks	PCR90	MWF	VAC50	75	All	2.984	0.005	0.001	0.006	0.002	0.003	0.003	1432.482	2.427	0.310	2.737	0.804	1.233	1.476	14325	24.270	3.100	27.370	8.040	12.330	14.760
120	original	1	60x8 weeks	PCR90	MWF	VAC50	75	All	2.984	0.005	0.001	0.006	0.002	0.003	0.003	1432.482	2.427	0.310	2.737	0.804	1.233	1.476	14325	24.270	3.100	27.370	8.040	12.330	14.760
120	original	1.2	60x8 weeks	PCR90	MWF	VAC50	75	All	2.982	0.005	0.001	0.006	0.002	0.003	0.003	1431.537	2.568	0.475	3.043	0.944	1.366	1.392	14315	25.680	4.750	30.430	9.440	13.660	13.920
120	original	1.2	60x8 weeks	PCR90	MWF	VAC50	75	All	2.982	0.005	0.001	0.006	0.002	0.003	0.003	1431.537	2.568	0.475	3.043	0.944	1.366	1.392	14315	25.680	4.750	30.430	9.440	13.660	13.920
120	original	1.2	60x8 weeks	PCR90	MWF	VAC50	75	All	2.982	0.005	0.001	0.006	0.002	0.003	0.003	1431.537	2.568	0.475	3.043	0.944	1.366	1.392	14315	25.680	4.750	30.430	9.440	13.660	13.920
120	alpha	0.6	60x8 weeks	PCR90	MWF	VAC50	75	All	2.974	0.005	0.002	0.007	0.001	0.006	0.003	1427.742	2.527	0.809	3.336	0.422	3.029	1.433	14277	25.270	8.090	33.360	4.220	30.290	14.330
120	alpha	0.6	60x8 weeks	PCR90	MWF	VAC50	75	All	2.974	0.005	0.002	0.007	0.001	0.006	0.003	1427.742	2.527	0.809	3.336	0.422	3.029	1.433	14277	25.270	8.090	33.360	4.220	30.290	14.330
120	alpha	0.6	60x8 weeks	PCR90	MWF	VAC50	75	All	2.974	0.005	0.002	0.007	0.001	0.006	0.003	1427.742	2.527	0.809	3.336	0.422	3.029	1.433	14277	25.270	8.090	33.360	4.220	30.290	14.330
120	alpha	1	60x8 weeks	PCR90	MWF	VAC50	75	All	2.967	0.005	0.003	0.009	0.001	0.008	0.003	1424.352	2.466	1.658	4.124	0.462	3.887	1.404	14244	24.660	16.580	41.240	4.620	38.870	14.040
120	alpha	1	60x8 weeks	PCR90	MWF	VAC50	75	All	2.967	0.005	0.003	0.009	0.001	0.008	0.003	1424.352	2.466	1.658	4.124	0.462	3.887	1.404	14244	24.660	16.580	41.240	4.620	38.870	14.040
120	alpha	1	60x8 weeks	PCR90	MWF	VAC50	75	All	2.967	0.005	0.003	0.009	0.001	0.008	0.003	1424.352	2.466	1.658	4.124	0.462	3.887	1.404	14244	24.660	16.580	41.240	4.620	38.870	14.040
120	alpha	1.2	60x8 weeks	PCR90	MWF	VAC50	75	All	2.964	0.005	0.005	0.010	0.001	0.009	0.003	1422.754	2.425	2.210	4.635	0.565	4.259	1.344	14228	24.250	22.100	46.350	5.650	42.590	13.440
120	alpha	1.2	60x8 weeks	PCR90	MWF	VAC50	75	All	2.964	0.005	0.005	0.010	0.001	0.009	0.003	1422.754	2.425	2.210	4.635	0.565	4.259	1.344	14228	24.250	22.100	46.350	5.650	42.590	13.440
120	alpha	1.2	60x8 weeks	PCR90	MWF	VAC50	75	All	2.964	0.005	0.005	0.010	0.001	0.009	0.003	1422.754	2.425	2.210	4.635	0.565	4.259	1.344	14228	24.250	22.100	46.350	5.650	42.590	13.440
120	delta	0.6	60x8 weeks	PCR90	MWF	VAC50	75	All	2.948	0.006	0.008	0.014	0.002	0.012	0.003	1415.075	2.937	3.639	6.576	1.036	5.876	1.301	14151	29.370	36.390	65.760	10.360	58.760	13.010
120	delta	0.6	60x8 weeks	PCR90	MWF	VAC50	75	All	2.948	0.006	0.008	0.014	0.002	0.012	0.003	1415.075	2.937	3.639	6.576	1.036	5.876	1.301	14151	29.370	36.390	65.760	10.360	58.760	13.010
120	delta	0.6	60x8 weeks	PCR90	MWF	VAC50	75	All	2.948	0.006	0.008	0.014	0.002	0.012	0.003	1415.075	2.937	3.639	6.576	1.036	5.876	1.301	14151	29.370	36.390	65.760	10.360	58.760	13.010
120	delta	1	60x8 weeks	PCR90	MWF	VAC50	75	All	2.877	0.006	0.027	0.032	0.006	0.028	0.003	1381.196	2.688	12.820	15.508	2.642	13.606	1.254	13812	26.880	128.200	155.080	26.420	136.060	12.540
120	delta	1	60x8 weeks	PCR90	MWF	VAC50	75	All	2.877	0.006	0.027	0.032	0.006	0.028	0.003	1381.196	2.688	12.820	15.508	2.642	13.606	1.254	13812	26.880	128.200	155.080	26.420	136.060	12.540
120	delta	1	60x8 weeks	PCR90	MWF	VAC50	75	All	2.877	0.006	0.027	0.032	0.006	0.028	0.003	1381.196	2.688	12.820	15.508	2.642	13.606	1.254	13812	26.880	128.200	155.080	26.420	136.060	12.540
120	delta	1.2	60x8 weeks	PCR90	MWF	VAC50	75	All	2.814	0.005	0.043	0.049	0.008	0.043	0.002	1350.802	2.544	20.775	23.319	3.921	20.626	1.145	13508	25.440	207.750	233.190	39.210	206.260	11.450
120	delta	1.2	60x8 weeks	PCR90	MWF	VAC50	75	All	2.814	0.005	0.043	0.049	0.008	0.043	0.002	1350.802	2.544	20.775	23.319	3.921	20.626	1.145	13508	25.440	207.750	233.190	39.210	206.260	11.450
120	delta	1.2	60x8 weeks	PCR90	MWF	VAC50	75	All	2.814	0.005	0.043	0.049	0.008	0.043	0.002	1350.802	2.544	20.775	23.319	3.921	20.626	1.145	13508	25.440	207.750	233.190	39.210	206.260	11.450

120	original	0.6	60x8 weeks	PCR90	MWF	VAC50	100	All	2.991	0.004	0.000	0.004	0.002	0.001	0.003	1435.443	2.017	0.021	2.038	0.902	0.289	1.428	14354	20.170	0.210	20.380	9.020	2.890	14.280
120	original	0.6	60x8 weeks	PCR90	MWF	VAC50	100	All	2.991	0.004	0.000	0.004	0.002	0.001	0.003	1435.443	2.017	0.021	2.038	0.902	0.289	1.428	14354	20.170	0.210	20.380	9.020	2.890	14.280
120	original	0.6	60x8 weeks	PCR90	MWF	VAC50	100	All	2.991	0.004	0.000	0.004	0.002	0.001	0.003	1435.443	2.017	0.021	2.038	0.902	0.289	1.428	14354	20.170	0.210	20.380	9.020	2.890	14.280
120	original	1	60x8 weeks	PCR90	MWF	VAC50	100	All	2.990	0.004	0.000	0.004	0.002	0.001	0.003	1435.382	2.060	0.051	2.111	0.895	0.318	1.407	14354	20.600	0.510	21.110	8.950	3.180	14.070
120	original	1	60x8 weeks	PCR90	MWF	VAC50	100	All	2.990	0.004	0.000	0.004	0.002	0.001	0.003	1435.382	2.060	0.051	2.111	0.895	0.318	1.407	14354	20.600	0.510	21.110	8.950	3.180	14.070
120	original	1	60x8 weeks	PCR90	MWF	VAC50	100	All	2.990	0.004	0.000	0.004	0.002	0.001	0.003	1435.382	2.060	0.051	2.111	0.895	0.318	1.407	14354	20.600	0.510	21.110	8.950	3.180	14.070
120	original	1.2	60x8 weeks	PCR90	MWF	VAC50	100	All	2.990	0.004	0.000	0.004	0.002	0.001	0.003	1435.431	2.032	0.042	2.074	0.870	0.311	1.408	14354	20.320	0.420	20.740	8.700	3.110	14.080
120	original	1.2	60x8 weeks	PCR90	MWF	VAC50	100	All	2.990	0.004	0.000	0.004	0.002	0.001	0.003	1435.431	2.032	0.042	2.074	0.870	0.311	1.408	14354	20.320	0.420	20.740	8.700	3.110	14.080
120	original	1.2	60x8 weeks	PCR90	MWF	VAC50	100	All	2.990	0.004	0.000	0.004	0.002	0.001	0.003	1435.431	2.032	0.042	2.074	0.870	0.311	1.408	14354	20.320	0.420	20.740	8.700	3.110	14.080
120	alpha	0.6	60x8 weeks	PCR90	MWF	VAC50	100	All	2.982	0.004	0.001	0.005	0.001	0.004	0.003	1431.468	1.963	0.328	2.291	0.280	2.083	1.413	14315	19.630	3.280	22.910	2.800	20.830	14.130
120	alpha	0.6	60x8 weeks	PCR90	MWF	VAC50	100	All	2.982	0.004	0.001	0.005	0.001	0.004	0.003	1431.468	1.963	0.328	2.291	0.280	2.083	1.413	14315	19.630	3.280	22.910	2.800	20.830	14.130
120	alpha	0.6	60x8 weeks	PCR90	MWF	VAC50	100	All	2.982	0.004	0.001	0.005	0.001	0.004	0.003	1431.468	1.963	0.328	2.291	0.280	2.083	1.413	14315	19.630	3.280	22.910	2.800	20.830	14.130
120	alpha	1	60x8 weeks	PCR90	MWF	VAC50	100	All	2.979	0.004	0.001	0.006	0.001	0.005	0.003	1429.852	2.102	0.642	2.744	0.339	2.482	1.422	14299	21.020	6.420	27.440	3.390	24.820	14.220
120	alpha	1	60x8 weeks	PCR90	MWF	VAC50	100	All	2.979	0.004	0.001	0.006	0.001	0.005	0.003	1429.852	2.102	0.642	2.744	0.339	2.482	1.422	14299	21.020	6.420	27.440	3.390	24.820	14.220
120	alpha	1	60x8 weeks	PCR90	MWF	VAC50	100	All	2.979	0.004	0.001	0.006	0.001	0.005	0.003	1429.852	2.102	0.642	2.744	0.339	2.482	1.422	14299	21.020	6.420	27.440	3.390	24.820	14.220
120	alpha	1.2	60x8 weeks	PCR90	MWF	VAC50	100	All	2.978	0.004	0.002	0.006	0.001	0.005	0.003	1429.406	2.022	0.832	2.854	0.370	2.570	1.467	14294	20.220	8.320	28.540	3.700	25.700	14.670
120	alpha	1.2	60x8 weeks	PCR90	MWF	VAC50	100	All	2.978	0.004	0.002	0.006	0.001	0.005	0.003	1429.406	2.022	0.832	2.854	0.370	2.570	1.467	14294	20.220	8.320	28.540	3.700	25.700	14.670
120	alpha	1.2	60x8 weeks	PCR90	MWF	VAC50	100	All	2.978	0.004	0.002	0.006	0.001	0.005	0.003	1429.406	2.022	0.832	2.854	0.370	2.570	1.467	14294	20.220	8.320	28.540	3.700	25.700	14.670
120	delta	0.6	60x8 weeks	PCR90	MWF	VAC50	100	All	2.966	0.006	0.004	0.009	0.001	0.008	0.003	1423.548	2.677	1.713	4.390	0.652	3.921	1.369	14235	26.770	17.130	43.900	6.520	39.210	13.690
120	delta	0.6	60x8 weeks	PCR90	MWF	VAC50	100	All	2.966	0.006	0.004	0.009	0.001	0.008	0.003	1423.548	2.677	1.713	4.390	0.652	3.921	1.369	14235	26.770	17.130	43.900	6.520	39.210	13.690
120	delta	0.6	60x8 weeks	PCR90	MWF	VAC50	100	All	2.966	0.006	0.004	0.009	0.001	0.008	0.003	1423.548	2.677	1.713	4.390	0.652	3.921	1.369	14235	26.770	17.130	43.900	6.520	39.210	13.690
120	delta	1	60x8 weeks	PCR90	MWF	VAC50	100	All	2.946	0.005	0.009	0.014	0.002	0.013	0.003	1414.006	2.523	4.420	6.943	1.099	6.154	1.327	14140	25.230	44.200	69.430	10.990	61.540	13.270
120	delta	1	60x8 weeks	PCR90	MWF	VAC50	100	All	2.946	0.005	0.009	0.014	0.002	0.013	0.003	1414.006	2.523	4.420	6.943	1.099	6.154	1.327	14140	25.230	44.200	69.430	10.990	61.540	13.270
120	delta	1	60x8 weeks	PCR90	MWF	VAC50	100	All	2.946	0.005	0.009	0.014	0.002	0.013	0.003	1414.006	2.523	4.420	6.943	1.099	6.154	1.327	14140	25.230	44.200	69.430	10.990	61.540	13.270
120	delta	1.2	60x8 weeks	PCR90	MWF	VAC50	100	All	2.916	0.005	0.017	0.022	0.004	0.020	0.003	1399.520	2.516	8.075	10.591	1.768	9.416	1.402	13995	25.160	80.750	105.910	17.680	94.160	14.020
120	delta	1.2	60x8 weeks	PCR90	MWF	VAC50	100	All	2.916	0.005	0.017	0.022	0.004	0.020	0.003	1399.520	2.516	8.075	10.591	1.768	9.416	1.402	13995	25.160	80.750	105.910	17.680	94.160	14.020
120	delta	1.2	60x8 weeks	PCR90	MWF	VAC50	100	All	2.916	0.005	0.017	0.022	0.004	0.020	0.003	1399.520	2.516	8.075	10.591	1.768	9.416	1.402	13995	25.160	80.750	105.910	17.680	94.160	14.020
120	original	0.6	60x8 weeks	PCR90	M	NOVAC	0	All	0.989	0.008	0.005	0.013	0.004	0.006	0.001	474.565	3.774	2.447	6.221	2.096	2.883	0.472	4746	37.740	24.470	62.210	20.960	28.830	4.720
120	original	0.6	60x8 weeks	PCR90	M	NOVAC	0	All	0.989	0.008	0.005	0.013	0.004	0.006	0.001	474.565	3.774	2.447	6.221	2.096	2.883	0.472	4746	37.740	24.470	62.210	20.960	28.830	4.720
120	original	0.6	60x8 weeks	PCR90	M	NOVAC	0	All	0.989	0.008	0.005	0.013	0.004	0.006	0.001	474.565	3.774	2.447	6.221	2.096	2.883	0.472	4746	37.740	24.470	62.210	20.960	28.830	4.720
120	original	1	60x8 weeks	PCR90	M	NOVAC	0	All	0.983	0.008	0.012	0.020	0.006	0.009	0.001	471.869	3.711	5.773	9.484	3.088	4.451	0.401	4719	37.110	57.730	94.840	30.880	44.510	4.010
120	original	1	60x8 weeks	PCR90	M	NOVAC	0	All	0.983	0.008	0.012	0.020	0.006	0.009	0.001	471.869	3.711	5.773	9.484	3.088	4.451	0.401	4719	37.110	57.730	94.840	30.880	44.510	4.010
120	original	1	60x8 weeks	PCR90	M	NOVAC	0	All	0.983	0.008	0.012	0.020	0.006	0.009	0.001	471.869	3.711	5.773	9.484	3.088	4.451	0.401	4719	37.110	57.730	94.840	30.880	44.510	4.010
120	original	1.2	60x8 weeks	PCR90	M	NOVAC	0	All	0.978	0.008	0.019	0.026	0.009	0.012	0.001	469.206	3.670	9.006	12.676	4.223	5.892	0.412	4692	36.700	90.060	126.760	42.230	58.920	4.120
120	original	1.2	60x8 weeks	PCR90	M	NOVAC	0	All	0.978	0.008	0.019	0.026	0.009	0.012	0.001	469.206	3.670	9.006	12.676	4.223	5.892	0.412	4692	36.700	90.060	126.760	42.230	58.920	4.120
120	original	1.2	60x8 weeks	PCR90	M	NOVAC	0	All	0.978	0.008	0.019	0.026	0.009	0.012	0.001	469.206	3.670	9.006	12.676	4.223	5.892	0.412	4692	36.700	90.060	126.760	42.230	58.920	4.120
120	alpha	0.6	60x8 weeks	PCR90	M	NOVAC	0	All	0.973	0.008	0.021	0.028	0.011	0.014	0.001	467.165	3.602	9.876	13.478	5.233	6.559	0.427	4672	36.020	98.760	134.780	52.330	65.590	4.270
120	alpha	0.6	60x8 weeks	PCR90	M	NOVAC	0	All	0.973	0.008	0.021	0.028	0.011	0.014	0.001	467.165	3.602	9.876	13.478	5.233	6.559	0.427	4672	36.020	98.760	134.780	52.330	65.590	4.270
120	alpha	0.6	60x8 weeks	PCR90	M	NOVAC	0	All	0.973	0.008	0.021	0.028	0.011	0.014	0.001	467.165	3.602	9.876	13.478	5.233	6.559	0.427	4672	36.020	98.760	134.780	52.330	65.590	4.270
120	alpha	1	60x8 weeks	PCR90	M	NOVAC	0	All	0.926	0.006	0.068	0.074	0.029	0.038	0.001	444.545	2.886	32.727	35.613	13.780	18.052	0.346	4445	28.860	327.270	356.130	137.800	180.520	3.460
120	alpha	1	60x8 weeks	PCR90	M	NOVAC	0	All	0.926	0.006	0.068	0.074	0.029	0.038	0.001	444.545	2.886	32.727	35.613	13.780	18.052	0.346	4445	28.860	327.270	356.130	137.800	180.520	3.460
120	alpha	1	60x8 weeks	PCR90	M	NOVAC	0	All	0.926	0.006	0.068	0.074	0.029	0.038	0.001	444.545	2.886	32.727	35.613	13.780	18.052	0.346	4445	28.860	327.270	356.130	137.800	180.520	3.460
120	alpha	1.2	60x8 weeks	PCR90	M	NOVAC	0	All	0.902	0.005	0.088	0.094	0.038	0.048	0.001	433.124	2.526	42.404	44.930	18.078	22.943	0.307	4331	25.260	424.040	449.300	180.780	229.430	3.070
120	alpha	1.2	60x8 weeks	PCR90	M	NOVAC	0	All	0.902	0.005	0.088	0.094	0.038	0.048	0.001	433.124	2.526	42.404	44.930	18.078	22.943	0.307	4331	25.260	424.040	449.300	180.780	229.430	3.070
120	alpha	1.2	60x8 weeks	PCR90	M	NOVAC	0	All	0.902	0.005	0.088	0.094	0.038	0.048	0.001	433.124	2.526	42.404	44.930	18.078	22.943	0.307	4331	25.260	424.040	449.300	180.780	229.430	3.070
120	delta	0.6	60x8 weeks	PCR90	M	NOVAC	0	All	0.890	0.005	0.097	0.102	0.045	0.049	0.001	427.074	2.361	46.460	48.821	21.734	23.395	0.295	4271	23.610	464.600	488.210	217.340	233.950	2.950
120	delta	0.6	60x8 weeks	PCR90	M	NOVAC	0	All	0.890	0.005	0.097	0.102	0.045	0.049	0.001	427.074	2.361	46.460	48.821	21.734	23.395	0.295	4271	23.610	464.600	488.210	217.340	233.950	2.950
120	delta	0.6	60x8 weeks	PCR90	M	NOVAC	0	All	0.890	0.005	0.097	0.102	0.045	0.049	0.001	427.074	2.361	46.460	48.821	21.734	23.395	0.295	4271	23.610	464.600	488.210	217.340	233.950	2.950
120	delta	1	60x8 weeks	PCR90	M	NOVAC	0	All	0.864	0.004	0.115	0.118	0.053	0.059	0.000	414.951	1.696	55.164	56.860	25.568	28.173	0.235	4150	16.960	551.640	568.600	255.680	281.730	2.350
120	delta	1	60x8 weeks	PCR90	M	NOVAC	0	All	0.864	0.004	0.115	0.118	0.053	0.059	0.000	414.951	1.696	55.164	56.860	25.568	28.173	0.235	4150	16.960	551.640	568.600	255.680	281.730	2.350
120	delta	1	60x8 weeks	PCR90	M	NOVAC	0	All	0.864	0.004	0.115	0.118	0.053	0.059	0.000	414.951	1.696	55.164	56.860	25.568	28.173	0.235	4150	16.960	551.640	568.600	255.680	281.730	2.350
120	delta	1.2	60x8 weeks	PCR90	M	NOVAC	0	All	0.860	0.003	0.117	0.120	0.055	0.059	0.000	412.718	1.598	56.087	57.685	26.417	28.556	0.190	4127	15.980	560.870	576.850	264.170	285.560	1.900
120	delta	1.2	60x8 weeks	PCR90	M	NOVAC	0	All	0.860	0.003	0.117	0.120	0.055	0.059	0.000	412.718	1.598	56.087	57.685	26.417	28.556	0.190	4127	15.980	560.870	576.850	264.170	285.560	1.900
120	delta	1.2	60x8 weeks	PCR90	M	NOVAC	0	All	0.860	0.003	0.117	0.120	0.055	0.059	0.000	412.718	1.598	56.087	57.685	26.417	28.556	0.190	4127	15.980	560.870	576.850	264.170	285.560	1.900
120	original	0.6	60x8 weeks	PCR90	M	VAC95	25	All	0.992	0.006	0.003	0.009	0.003	0.004	0.001	476.230	3.020	1.322	4.342	1.488	1.992	0.465	4762	30.200	13.220	43.420	14.880	19.920	4.650
120	original	0.6	60x8 weeks	PCR90	M	VAC95	25	All	0.992	0.006	0.003	0.009	0.003	0.004	0.001	476.230	3.020	1.322	4.342	1.488	1.992	0.465	4762	30.200	13.220	43.420	14.880	19.920	4.650
120	original	0.6	60x8 weeks	PCR90	M	VAC95	25	All	0.992	0.006	0.003	0.009	0.003	0.004	0.001	476.230	3.020	1.322	4.342	1.488	1.992	0.465	4762	30.200	13.220	43.420	14.880	19.920	4.650
120	original	1	60x8 weeks	PCR90	M	VAC95	25	All	0.990	0.006	0.006	0.012	0.004	0.005	0.001	475.023	2.976	2.860	5.836	2.009	2.585	0.492	4750	29.760	28.600	58.360	20.090	25.850	4.920
120	original	1	60x8 weeks	PCR90	M	VAC95	25	All	0.990	0.006	0.006	0.012	0.004	0.005	0.001	475.023	2.976	2.860	5.836	2.009	2.585	0.492	4750	29.760	28.600	58.360	20.090	25.850	4.920
120	original	1	60x8 weeks	PCR90	M	VAC95	25	All	0.990	0.006	0.006	0.012	0.004	0.005	0.001	475.023	2.976	2.860	5.836	2.009	2.585	0.492	4750	29.760	28.600	58.360	20.090	25.850	4.920
120	original	1.2	60x8 weeks	PCR90	M	VAC95	25	All	0.988	0.006	0.008	0.014	0.005	0.006	0.001	474.384	2.960	3.644	6.604	2.202	3.037	0.445	4744	29.600	36.440	66.040	22.020	30.370	4.450
120	original	1.2	60x8 weeks	PCR90	M	VAC95	25	All	0.988	0.006	0.008	0.014	0.005	0.006	0.001	474.384	2.960	3.644	6.604	2.202	3.037	0.445	4744	29.600	36.440	66.040	22.020	30.370	4.450
120	original	1.2	60x8 weeks	PCR90	M	VAC95	25	All	0.988	0.006	0.008	0.014	0.005	0.006	0.001	474.384	2.960	3.644	6.604	2.202	3.037	0.445	4744	29.600	36.440	66.040	22.020	30.370	4.450
120	alpha	0.6	60x8 weeks	PCR90	M	VAC95	25	All	0.986	0.006	0.008	0.014	0.005	0.007	0.001	473.458	2.896	3.891	6.787	2.631	3.296	0.433	4735	28.960	38.910	67.870	26.310	32.960	4.330
120	alpha	0.6	60x8 weeks	PCR90	M	VAC95	25	All	0.986	0.006	0.008	0.014	0.005	0.007	0.001	473.458	2.896	3.891	6.787	2.631	3.296	0.433	4735	28.960	38.910	67.870	26.310	32.960	4.330
120	alpha	0.6	60x8 weeks	PCR90	M	VAC95	25	All	0.986	0.006	0.008	0.014	0.005	0.007	0.001	473.458	2.896	3.891	6.787	2.631	3.296	0.433	4735	28.960	38.910	67.870	26.310	32.960	4.330
120	alpha	1	60x8 weeks	PCR90	M	VAC95	25	All	0.970	0.006	0.026	0.031	0.012	0.016	0.001	465.416	2.673	12.446	15.119	5.782	7.486	0.428	4654	26.730	124.460	151.190	57.820	74.860	4.280
120	alpha	1	60x8 weeks	PCR90	M	VAC95	25	All	0.970	0.006	0.026	0.031	0.012	0.016	0.001	465.416	2.673	12.446	15.119	5.782	7.486	0.428	4654	26.730	124.460	151.190	57.820	74.860	4.280
120	alpha	1	60x8 weeks	PCR90	M	VAC95	25	All	0.970	0.006	0.026	0.031	0.012	0.016	0.001	465.416	2.673	12.446	15.119	5.782	7.486	0.428	4654	26.730	124.460	151.190	57.820	74.860	4.280
120	alpha	1.2	60x8 weeks	PCR90	M	VAC95	25	All	0.957	0.005	0.039	0.045	0.017	0.022	0.001	459.434	2.474	18.892	21.366	8.266	10.551	0.405	4594	24.740	188.920	213.660	82.660	105.510	4.050
120	alpha	1.2	60x8 weeks	PCR90	M	VAC95	25	All	0.957	0.005	0.039	0.045	0.017	0.022	0.001	459.434	2.474	18.892	21.366	8.266	10.551	0.405	4594	24.740	188.920	213.660	82.660	105.510	4.050

120	alpha	1.2	60x8 weeks	PCR90	M	VAC95	25	All	0.957	0.005	0.039	0.045	0.017	0.022	0.001	459.434	2.474	18.892	21.366	8.266	10.551	0.405	4594	24.740	188.920	213.660	82.660	105.510	4.050
120	delta	0.6	60x8 weeks	PCR90	M	VAC95	25	All	0.949	0.005	0.046	0.051	0.022	0.024	0.001	455.565	2.419	21.945	24.364	10.495	11.287	0.390	4556	24.190	219.450	243.640	104.950	112.870	3.900
120	delta	0.6	60x8 weeks	PCR90	M	VAC95	25	All	0.949	0.005	0.046	0.051	0.022	0.024	0.001	455.565	2.419	21.945	24.364	10.495	11.287	0.390	4556	24.190	219.450	243.640	104.950	112.870	3.900
120	delta	0.6	60x8 weeks	PCR90	M	VAC95	25	All	0.949	0.005	0.046	0.051	0.022	0.024	0.001	455.565	2.419	21.945	24.364	10.495	11.287	0.390	4556	24.190	219.450	243.640	104.950	112.870	3.900
120	delta	1	60x8 weeks	PCR90	M	VAC95	25	All	0.901	0.004	0.088	0.092	0.040	0.045	0.001	432.655	1.746	42.342	44.088	19.243	21.457	0.301	4327	17.460	423.420	440.880	192.430	214.570	3.010
120	delta	1	60x8 weeks	PCR90	M	VAC95	25	All	0.901	0.004	0.088	0.092	0.040	0.045	0.001	432.655	1.746	42.342	44.088	19.243	21.457	0.301	4327	17.460	423.420	440.880	192.430	214.570	3.010
120	delta	1	60x8 weeks	PCR90	M	VAC95	25	All	0.901	0.004	0.088	0.092	0.040	0.045	0.001	432.655	1.746	42.342	44.088	19.243	21.457	0.301	4327	17.460	423.420	440.880	192.430	214.570	3.010
120	delta	1.2	60x8 weeks	PCR90	M	VAC95	25	All	0.895	0.003	0.093	0.097	0.042	0.047	0.001	429.591	1.601	44.775	46.376	20.127	22.570	0.285	4296	16.010	447.750	463.760	201.270	225.700	2.850
120	delta	1.2	60x8 weeks	PCR90	M	VAC95	25	All	0.895	0.003	0.093	0.097	0.042	0.047	0.001	429.591	1.601	44.775	46.376	20.127	22.570	0.285	4296	16.010	447.750	463.760	201.270	225.700	2.850
120	delta	1.2	60x8 weeks	PCR90	M	VAC95	25	All	0.895	0.003	0.093	0.097	0.042	0.047	0.001	429.591	1.601	44.775	46.376	20.127	22.570	0.285	4296	16.010	447.750	463.760	201.270	225.700	2.850
120	original	0.6	60x8 weeks	PCR90	M	VAC95	50	All	0.995	0.004	0.001	0.005	0.002	0.002	0.001	477.784	2.080	0.501	2.581	0.890	1.120	0.481	4778	20.800	5.010	25.810	8.900	11.200	4.810
120	original	0.6	60x8 weeks	PCR90	M	VAC95	50	All	0.995	0.004	0.001	0.005	0.002	0.002	0.001	477.784	2.080	0.501	2.581	0.890	1.120	0.481	4778	20.800	5.010	25.810	8.900	11.200	4.810
120	original	0.6	60x8 weeks	PCR90	M	VAC95	50	All	0.995	0.004	0.001	0.005	0.002	0.002	0.001	477.784	2.080	0.501	2.581	0.890	1.120	0.481	4778	20.800	5.010	25.810	8.900	11.200	4.810
120	original	1	60x8 weeks	PCR90	M	VAC95	50	All	0.995	0.004	0.002	0.007	0.002	0.003	0.001	477.363	2.131	1.003	3.134	1.071	1.369	0.472	4774	21.310	10.030	31.340	10.710	13.690	4.720
120	original	1	60x8 weeks	PCR90	M	VAC95	50	All	0.995	0.004	0.002	0.007	0.002	0.003	0.001	477.363	2.131	1.003	3.134	1.071	1.369	0.472	4774	21.310	10.030	31.340	10.710	13.690	4.720
120	original	1	60x8 weeks	PCR90	M	VAC95	50	All	0.995	0.004	0.002	0.007	0.002	0.003	0.001	477.363	2.131	1.003	3.134	1.071	1.369	0.472	4774	21.310	10.030	31.340	10.710	13.690	4.720
120	original	1.2	60x8 weeks	PCR90	M	VAC95	50	All	0.995	0.004	0.002	0.006	0.002	0.003	0.001	477.392	1.971	1.139	3.110	1.047	1.379	0.425	4774	19.710	11.390	31.100	10.470	13.790	4.250
120	original	1.2	60x8 weeks	PCR90	M	VAC95	50	All	0.995	0.004	0.002	0.006	0.002	0.003	0.001	477.392	1.971	1.139	3.110	1.047	1.379	0.425	4774	19.710	11.390	31.100	10.470	13.790	4.250
120	original	1.2	60x8 weeks	PCR90	M	VAC95	50	All	0.995	0.004	0.002	0.006	0.002	0.003	0.001	477.392	1.971	1.139	3.110	1.047	1.379	0.425	4774	19.710	11.390	31.100	10.470	13.790	4.250
120	alpha	0.6	60x8 weeks	PCR90	M	VAC95	50	All	0.993	0.004	0.003	0.008	0.003	0.004	0.001	476.577	2.099	1.518	3.617	1.355	1.751	0.461	4766	20.990	15.180	36.170	13.550	17.510	4.610
120	alpha	0.6	60x8 weeks	PCR90	M	VAC95	50	All	0.993	0.004	0.003	0.008	0.003	0.004	0.001	476.577	2.099	1.518	3.617	1.355	1.751	0.461	4766	20.990	15.180	36.170	13.550	17.510	4.610
120	alpha	0.6	60x8 weeks	PCR90	M	VAC95	50	All	0.993	0.004	0.003	0.008	0.003	0.004	0.001	476.577	2.099	1.518	3.617	1.355	1.751	0.461	4766	20.990	15.180	36.170	13.550	17.510	4.610
120	alpha	1	60x8 weeks	PCR90	M	VAC95	50	All	0.989	0.004	0.008	0.012	0.004	0.006	0.001	474.797	2.045	3.643	5.688	2.104	2.784	0.468	4748	20.450	36.430	56.880	21.040	27.840	4.680
120	alpha	1	60x8 weeks	PCR90	M	VAC95	50	All	0.989	0.004	0.008	0.012	0.004	0.006	0.001	474.797	2.045	3.643	5.688	2.104	2.784	0.468	4748	20.450	36.430	56.880	21.040	27.840	4.680
120	alpha	1	60x8 weeks	PCR90	M	VAC95	50	All	0.989	0.004	0.008	0.012	0.004	0.006	0.001	474.797	2.045	3.643	5.688	2.104	2.784	0.468	4748	20.450	36.430	56.880	21.040	27.840	4.680
120	alpha	1.2	60x8 weeks	PCR90	M	VAC95	50	All	0.986	0.004	0.011	0.015	0.006	0.007	0.001	473.136	1.998	5.214	7.212	2.698	3.573	0.432	4731	19.980	52.140	72.120	26.980	35.730	4.320
120	alpha	1.2	60x8 weeks	PCR90	M	VAC95	50	All	0.986	0.004	0.011	0.015	0.006	0.007	0.001	473.136	1.998	5.214	7.212	2.698	3.573	0.432	4731	19.980	52.140	72.120	26.980	35.730	4.320
120	alpha	1.2	60x8 weeks	PCR90	M	VAC95	50	All	0.986	0.004	0.011	0.015	0.006	0.007	0.001	473.136	1.998	5.214	7.212	2.698	3.573	0.432	4731	19.980	52.140	72.120	26.980	35.730	4.320
120	delta	0.6	60x8 weeks	PCR90	M	VAC95	50	All	0.983	0.004	0.013	0.017	0.007	0.008	0.001	471.841	2.050	6.326	8.376	3.515	3.850	0.485	4718	20.500	63.260	83.760	35.150	38.500	4.850
120	delta	0.6	60x8 weeks	PCR90	M	VAC95	50	All	0.983	0.004	0.013	0.017	0.007	0.008	0.001	471.841	2.050	6.326	8.376	3.515	3.850	0.485	4718	20.500	63.260	83.760	35.150	38.500	4.850
120	delta	0.6	60x8 weeks	PCR90	M	VAC95	50	All	0.983	0.004	0.013	0.017	0.007	0.008	0.001	471.841	2.050	6.326	8.376	3.515	3.850	0.485	4718	20.500	63.260	83.760	35.150	38.500	4.850
120	delta	1	60x8 weeks	PCR90	M	VAC95	50	All	0.958	0.004	0.040	0.043	0.018	0.020	0.001	459.813	1.719	19.054	20.773	8.618	9.605	0.406	4598	17.190	190.540	207.730	86.180	96.050	4.060
120	delta	1	60x8 weeks	PCR90	M	VAC95	50	All	0.958	0.004	0.040	0.043	0.018	0.020	0.001	459.813	1.719	19.054	20.773	8.618	9.605	0.406	4598	17.190	190.540	207.730	86.180	96.050	4.060
120	delta	1	60x8 weeks	PCR90	M	VAC95	50	All	0.958	0.004	0.040	0.043	0.018	0.020	0.001	459.813	1.719	19.054	20.773	8.618	9.605	0.406	4598	17.190	190.540	207.730	86.180	96.050	4.060
120	delta	1.2	60x8 weeks	PCR90	M	VAC95	50	All	0.944	0.003	0.051	0.055	0.023	0.026	0.001	453.296	1.656	24.571	26.227	11.032	12.430	0.365	4533	16.560	245.710	262.270	110.320	124.300	3.650
120	delta	1.2	60x8 weeks	PCR90	M	VAC95	50	All	0.944	0.003	0.051	0.055	0.023	0.026	0.001	453.296	1.656	24.571	26.227	11.032	12.430	0.365	4533	16.560	245.710	262.270	110.320	124.300	3.650
120	delta	1.2	60x8 weeks	PCR90	M	VAC95	50	All	0.944	0.003	0.051	0.055	0.023	0.026	0.001	453.296	1.656	24.571	26.227	11.032	12.430	0.365	4533	16.560	245.710	262.270	110.320	124.300	3.650
120	original	0.6	60x8 weeks	PCR90	M	VAC95	75	All	0.998	0.002	0.000	0.003	0.001	0.001	0.001	479.017	1.148	0.094	1.242	0.402	0.494	0.470	4790	11.480	0.940	12.420	4.020	4.940	4.700
120	original	0.6	60x8 weeks	PCR90	M	VAC95	75	All	0.998	0.002	0.000	0.003	0.001	0.001	0.001	479.017	1.148	0.094	1.242	0.402	0.494	0.470	4790	11.480	0.940	12.420	4.020	4.940	4.700
120	original	0.6	60x8 weeks	PCR90	M	VAC95	75	All	0.998	0.002	0.000	0.003	0.001	0.001	0.001	479.017	1.148	0.094	1.242	0.402	0.494	0.470	4790	11.480	0.940	12.420	4.020	4.940	4.700
120	original	1	60x8 weeks	PCR90	M	VAC95	75	All	0.998	0.002	0.000	0.003	0.001	0.001	0.001	478.911	1.136	0.183	1.319	0.467	0.523	0.448	4789	11.360	1.830	13.190	4.670	5.230	4.480
120	original	1	60x8 weeks	PCR90	M	VAC95	75	All	0.998	0.002	0.000	0.003	0.001	0.001	0.001	478.911	1.136	0.183	1.319	0.467	0.523	0.448	4789	11.360	1.830	13.190	4.670	5.230	4.480
120	original	1	60x8 weeks	PCR90	M	VAC95	75	All	0.998	0.002	0.000	0.003	0.001	0.001	0.001	478.911	1.136	0.183	1.319	0.467	0.523	0.448	4789	11.360	1.830	13.190	4.670	5.230	4.480
120	original	1.2	60x8 weeks	PCR90	M	VAC95	75	All	0.998	0.003	0.001	0.003	0.001	0.001	0.001	478.821	1.207	0.255	1.462	0.502	0.554	0.474	4788	12.070	2.550	14.620	5.020	5.540	4.740
120	original	1.2	60x8 weeks	PCR90	M	VAC95	75	All	0.998	0.003	0.001	0.003	0.001	0.001	0.001	478.821	1.207	0.255	1.462	0.502	0.554	0.474	4788	12.070	2.550	14.620	5.020	5.540	4.740
120	original	1.2	60x8 weeks	PCR90	M	VAC95	75	All	0.998	0.003	0.001	0.003	0.001	0.001	0.001	478.821	1.207	0.255	1.462	0.502	0.554	0.474	4788	12.070	2.550	14.620	5.020	5.540	4.740
120	alpha	0.6	60x8 weeks	PCR90	M	VAC95	75	All	0.997	0.003	0.001	0.003	0.001	0.002	0.001	478.475	1.238	0.394	1.632	0.593	0.815	0.432	4785	12.380	3.940	16.320	5.930	8.150	4.320
120	alpha	0.6	60x8 weeks	PCR90	M	VAC95	75	All	0.997	0.003	0.001	0.003	0.001	0.002	0.001	478.475	1.238	0.394	1.632	0.593	0.815	0.432	4785	12.380	3.940	16.320	5.930	8.150	4.320
120	alpha	0.6	60x8 weeks	PCR90	M	VAC95	75	All	0.997	0.003	0.001	0.003	0.001	0.002	0.001	478.475	1.238	0.394	1.632	0.593	0.815	0.432	4785	12.380	3.940	16.320	5.930	8.150	4.320
120	alpha	1	60x8 weeks	PCR90	M	VAC95	75	All	0.996	0.003	0.002	0.004	0.002	0.002	0.001	478.114	1.236	0.780	2.016	0.742	0.946	0.496	4781	12.360	7.800	20.160	7.420	9.460	4.960
120	alpha	1	60x8 weeks	PCR90	M	VAC95	75	All	0.996	0.003	0.002	0.004	0.002	0.002	0.001	478.114	1.236	0.780	2.016	0.742	0.946	0.496	4781	12.360	7.800	20.160	7.420	9.460	4.960
120	alpha	1	60x8 weeks	PCR90	M	VAC95	75	All	0.996	0.003	0.002	0.004	0.002	0.002	0.001	478.114	1.236	0.780	2.016	0.742	0.946	0.496	4781	12.360	7.800	20.160	7.420	9.460	4.960
120	alpha	1.2	60x8 weeks	PCR90	M	VAC95	75	All	0.996	0.002	0.002	0.004	0.002	0.002	0.001	478.196	1.177	0.821	1.998	0.723	0.940	0.463	4782	11.770	8.210	19.980	7.230	9.400	4.630
120	alpha	1.2	60x8 weeks	PCR90	M	VAC95	75	All	0.996	0.002	0.002	0.004	0.002	0.002	0.001	478.196	1.177	0.821	1.998	0.723	0.940	0.463	4782	11.770	8.210	19.980	7.230	9.400	4.630
120	alpha	1.2	60x8 weeks	PCR90	M	VAC95	75	All	0.996	0.002	0.002	0.004	0.002	0.002	0.001	478.196	1.177	0.821	1.998	0.723	0.940	0.463	4782	11.770	8.210	19.980	7.230	9.400	4.630
120	delta	0.6	60x8 weeks	PCR90	M	VAC95	75	All	0.995	0.003	0.003	0.006	0.002	0.003	0.001	477.434	1.426	1.284	2.710	1.105	1.219	0.474	4774	14.260	12.840	27.100	11.050	12.190	4.740
120	delta	0.6	60x8 weeks	PCR90	M	VAC95	75	All	0.995	0.003	0.003	0.006	0.002	0.003	0.001	477.434	1.426	1.284	2.710	1.105	1.219	0.474	4774	14.260	12.840	27.100	11.050	12.190	4.740
120	delta	0.6	60x8 weeks	PCR90	M	VAC95	75	All	0.995	0.003	0.003	0.006	0.002	0.003	0.001	477.434	1.426	1.284	2.710	1.105	1.219	0.474	4774	14.260	12.840	27.100	11.050	12.190	4.740
120	delta	1	60x8 weeks	PCR90	M	VAC95	75	All	0.990	0.003	0.007	0.010	0.004	0.005	0.001	475.417	1.358	3.443	4.801	1.931	2.262	0.484	4754	13.580	34.430	48.010	19.310	22.620	4.840
120	delta	1	60x8 weeks	PCR90	M	VAC95	75	All	0.990	0.003	0.007	0.010	0.004	0.005	0.001	475.417	1.358	3.443	4.801	1.931	2.262	0.484	4754	13.580	34.430	48.010	19.310	22.620	4.840
120	delta	1	60x8 weeks	PCR90	M	VAC95	75	All	0.990	0.003	0.007	0.010	0.004	0.005	0.001	475.417	1.358	3.443	4.801	1.931	2.262	0.484	4754	13.580	34.430	48.010	19.310	22.620	4.840
120	delta	1.2	60x8 weeks	PCR90	M	VAC95	75	All	0.988	0.003	0.010	0.013	0.005	0.006	0.001	474.192	1.350	4.697	6.047	2.432	2.857	0.421	4742	13.500	46.970	60.470	24.320	28.570	4.210
120	delta	1.2	60x8 weeks	PCR90	M	VAC95	75	All	0.988	0.003	0.010	0.013	0.005	0.006	0.001	474.192	1.350	4.697	6.047	2.432	2.857	0.421	4742	13.500	46.970	60.470	24.320	28.570	4.210
120	delta	1.2	60x8 weeks	PCR90	M	VAC95	75	All	0.988	0.003	0.010	0.013	0.005	0.006	0.001	474.192	1.350	4.697	6.047	2.432	2.857	0.421	4742	13.500	46.970	60.470	24.320	28.570	4.210
120	original	0.6	60x8 weeks	PCR90	M	VAC95	100	All	1.000	0.001	0.000	0.001	0.000	0.000	0.001	479.836	0.310	0.001	0.311	0.111	0.021	0.482	4798	3.100	0.010	3.110	1.110	0.210	4.820
120	original	0.6	60x8 weeks	PCR90	M	VAC95	100	All	1.000	0.001	0.000	0.001	0.000	0.000	0.001	479.836	0.310	0.001	0.311	0.111	0.021	0.482	4798	3.100	0.010	3.110	1.110	0.210	4.820
120	original	0.6	60x8 weeks	PCR90	M	VAC95	100	All	1.000	0.001	0.000	0.001	0.000	0.000	0.001	479.836	0.310	0.001	0.311	0.111	0.021	0.482	4798	3.100	0.010	3.110	1.110	0.210	4.820
120	original	1	60x8 weeks	PCR90	M	VAC95	100	All	1.000	0.001	0.000	0.001	0.000	0.000	0.001	479.841	0.317	0.000	0.317	0.113	0.014	0.476	4798	3.170	0.000	3.170	1.130	0.140	4.760
120	original	1	60x8 weeks	PCR90	M	VAC95	100	All	1.000	0.001	0.000	0.001	0.000	0.000	0.001	479.841	0.317	0.000	0.317	0.113	0.014	0.476	4798	3.170	0.000	3.170	1.130	0.140	4.760
120	original	1	60x8 weeks	PCR90	M	VAC95	100	All	1.000	0.001	0.000	0.001	0.000	0.000	0.001	479.841	0.317	0.000	0.317	0.113	0.014	0.476	4798	3.170	0.000	3.170	1.130	0.140	4.760
120	original	1.2	60x8 weeks	PCR90	M	VAC95	100	All	1.000	0.001	0.000	0.001	0.000	0.000	0.001	479.851	0.277	0.000	0.277	0.105	0.014	0.476	4799	2.770	0.000	2.770	1.050	0.140	4.760

120	original	1.2	60x8 weeks	PCR90	M	VAC95	100	All	1.000	0.001	0.000	0.001	0.000	0.000	0.001	479.851	0.277	0.000	0.277	0.105	0.014	0.476	4799	2.770	0.000	2.770	1.050	0.140	4.760
120	original	1.2	60x8 weeks	PCR90	M	VAC95	100	All	1.000	0.001	0.000	0.001	0.000	0.000	0.001	479.851	0.277	0.000	0.277	0.105	0.014	0.476	4799	2.770	0.000	2.770	1.050	0.140	4.760
120	alpha	0.6	60x8 weeks	PCR90	M	VAC95	100	All	0.999	0.001	0.000	0.001	0.000	0.000	0.001	479.723	0.323	0.018	0.341	0.092	0.178	0.514	4797	3.230	0.180	3.410	0.920	1.780	5.140
120	alpha	0.6	60x8 weeks	PCR90	M	VAC95	100	All	0.999	0.001	0.000	0.001	0.000	0.000	0.001	479.723	0.323	0.018	0.341	0.092	0.178	0.514	4797	3.230	0.180	3.410	0.920	1.780	5.140
120	alpha	0.6	60x8 weeks	PCR90	M	VAC95	100	All	0.999	0.001	0.000	0.001	0.000	0.000	0.001	479.723	0.323	0.018	0.341	0.092	0.178	0.514	4797	3.230	0.180	3.410	0.920	1.780	5.140
120	alpha	1	60x8 weeks	PCR90	M	VAC95	100	All	0.999	0.001	0.000	0.001	0.000	0.000	0.001	479.645	0.391	0.023	0.414	0.134	0.190	0.490	4796	3.910	0.230	4.140	1.340	1.900	4.900
120	alpha	1	60x8 weeks	PCR90	M	VAC95	100	All	0.999	0.001	0.000	0.001	0.000	0.000	0.001	479.645	0.391	0.023	0.414	0.134	0.190	0.490	4796	3.910	0.230	4.140	1.340	1.900	4.900
120	alpha	1	60x8 weeks	PCR90	M	VAC95	100	All	0.999	0.001	0.000	0.001	0.000	0.000	0.001	479.645	0.391	0.023	0.414	0.134	0.190	0.490	4796	3.910	0.230	4.140	1.340	1.900	4.900
120	alpha	1.2	60x8 weeks	PCR90	M	VAC95	100	All	0.999	0.001	0.000	0.001	0.000	0.000	0.001	479.635	0.388	0.032	0.420	0.141	0.196	0.467	4796	3.880	0.320	4.200	1.410	1.960	4.670
120	alpha	1.2	60x8 weeks	PCR90	M	VAC95	100	All	0.999	0.001	0.000	0.001	0.000	0.000	0.001	479.635	0.388	0.032	0.420	0.141	0.196	0.467	4796	3.880	0.320	4.200	1.410	1.960	4.670
120	alpha	1.2	60x8 weeks	PCR90	M	VAC95	100	All	0.999	0.001	0.000	0.001	0.000	0.000	0.001	479.635	0.388	0.032	0.420	0.141	0.196	0.467	4796	3.880	0.320	4.200	1.410	1.960	4.670
120	delta	0.6	60x8 weeks	PCR90	M	VAC95	100	All	0.999	0.001	0.000	0.001	0.000	0.001	0.001	479.412	0.567	0.100	0.667	0.234	0.299	0.457	4794	5.670	1.000	6.670	2.340	2.990	4.570
120	delta	0.6	60x8 weeks	PCR90	M	VAC95	100	All	0.999	0.001	0.000	0.001	0.000	0.001	0.001	479.412	0.567	0.100	0.667	0.234	0.299	0.457	4794	5.670	1.000	6.670	2.340	2.990	4.570
120	delta	0.6	60x8 weeks	PCR90	M	VAC95	100	All	0.999	0.001	0.000	0.001	0.000	0.001	0.001	479.412	0.567	0.100	0.667	0.234	0.299	0.457	4794	5.670	1.000	6.670	2.340	2.990	4.570
120	delta	1	60x8 weeks	PCR90	M	VAC95	100	All	0.999	0.001	0.000	0.002	0.001	0.001	0.001	479.348	0.575	0.154	0.729	0.270	0.343	0.478	4793	5.750	1.540	7.290	2.700	3.430	4.780
120	delta	1	60x8 weeks	PCR90	M	VAC95	100	All	0.999	0.001	0.000	0.002	0.001	0.001	0.001	479.348	0.575	0.154	0.729	0.270	0.343	0.478	4793	5.750	1.540	7.290	2.700	3.430	4.780
120	delta	1	60x8 weeks	PCR90	M	VAC95	100	All	0.999	0.001	0.000	0.002	0.001	0.001	0.001	479.348	0.575	0.154	0.729	0.270	0.343	0.478	4793	5.750	1.540	7.290	2.700	3.430	4.780
120	delta	1.2	60x8 weeks	PCR90	M	VAC95	100	All	0.999	0.001	0.000	0.002	0.001	0.001	0.001	479.289	0.616	0.196	0.812	0.277	0.390	0.461	4793	6.160	1.960	8.120	2.770	3.900	4.610
120	delta	1.2	60x8 weeks	PCR90	M	VAC95	100	All	0.999	0.001	0.000	0.002	0.001	0.001	0.001	479.289	0.616	0.196	0.812	0.277	0.390	0.461	4793	6.160	1.960	8.120	2.770	3.900	4.610
120	delta	1.2	60x8 weeks	PCR90	M	VAC95	100	All	0.999	0.001	0.000	0.002	0.001	0.001	0.001	479.289	0.616	0.196	0.812	0.277	0.390	0.461	4793	6.160	1.960	8.120	2.770	3.900	4.610
120	original	0.6	60x8 weeks	PCR90	M	VAC70	25	All	0.992	0.007	0.003	0.010	0.003	0.004	0.001	476.048	3.250	1.408	4.658	1.613	1.990	0.481	4760	32.500	14.080	46.580	16.130	19.900	4.810
120	original	0.6	60x8 weeks	PCR90	M	VAC70	25	All	0.992	0.007	0.003	0.010	0.003	0.004	0.001	476.048	3.250	1.408	4.658	1.613	1.990	0.481	4760	32.500	14.080	46.580	16.130	19.900	4.810
120	original	0.6	60x8 weeks	PCR90	M	VAC70	25	All	0.992	0.007	0.003	0.010	0.003	0.004	0.001	476.048	3.250	1.408	4.658	1.613	1.990	0.481	4760	32.500	14.080	46.580	16.130	19.900	4.810
120	original	1	60x8 weeks	PCR90	M	VAC70	25	All	0.989	0.007	0.006	0.013	0.005	0.006	0.001	474.745	3.273	3.017	6.290	2.185	2.680	0.431	4747	32.730	30.170	62.900	21.850	26.800	4.310
120	original	1	60x8 weeks	PCR90	M	VAC70	25	All	0.989	0.007	0.006	0.013	0.005	0.006	0.001	474.745	3.273	3.017	6.290	2.185	2.680	0.431	4747	32.730	30.170	62.900	21.850	26.800	4.310
120	original	1	60x8 weeks	PCR90	M	VAC70	25	All	0.989	0.007	0.006	0.013	0.005	0.006	0.001	474.745	3.273	3.017	6.290	2.185	2.680	0.431	4747	32.730	30.170	62.900	21.850	26.800	4.310
120	original	1.2	60x8 weeks	PCR90	M	VAC70	25	All	0.987	0.007	0.008	0.015	0.005	0.006	0.001	473.949	3.197	4.012	7.209	2.520	3.049	0.428	4739	31.970	40.120	72.090	25.200	30.490	4.280
120	original	1.2	60x8 weeks	PCR90	M	VAC70	25	All	0.987	0.007	0.008	0.015	0.005	0.006	0.001	473.949	3.197	4.012	7.209	2.520	3.049	0.428	4739	31.970	40.120	72.090	25.200	30.490	4.280
120	original	1.2	60x8 weeks	PCR90	M	VAC70	25	All	0.987	0.007	0.008	0.015	0.005	0.006	0.001	473.949	3.197	4.012	7.209	2.520	3.049	0.428	4739	31.970	40.120	72.090	25.200	30.490	4.280
120	alpha	0.6	60x8 weeks	PCR90	M	VAC70	25	All	0.984	0.006	0.010	0.017	0.006	0.008	0.001	472.348	3.092	4.890	7.982	3.041	3.913	0.441	4723	30.920	48.900	79.820	30.410	39.130	4.410
120	alpha	0.6	60x8 weeks	PCR90	M	VAC70	25	All	0.984	0.006	0.010	0.017	0.006	0.008	0.001	472.348	3.092	4.890	7.982	3.041	3.913	0.441	4723	30.920	48.900	79.820	30.410	39.130	4.410
120	alpha	0.6	60x8 weeks	PCR90	M	VAC70	25	All	0.984	0.006	0.010	0.017	0.006	0.008	0.001	472.348	3.092	4.890	7.982	3.041	3.913	0.441	4723	30.920	48.900	79.820	30.410	39.130	4.410
120	alpha	1	60x8 weeks	PCR90	M	VAC70	25	All	0.964	0.006	0.033	0.039	0.015	0.019	0.001	462.573	2.896	15.717	18.613	7.077	9.089	0.433	4626	28.960	157.170	186.130	70.770	90.890	4.330
120	alpha	1	60x8 weeks	PCR90	M	VAC70	25	All	0.964	0.006	0.033	0.039	0.015	0.019	0.001	462.573	2.896	15.717	18.613	7.077	9.089	0.433	4626	28.960	157.170	186.130	70.770	90.890	4.330
120	alpha	1	60x8 weeks	PCR90	M	VAC70	25	All	0.964	0.006	0.033	0.039	0.015	0.019	0.001	462.573	2.896	15.717	18.613	7.077	9.089	0.433	4626	28.960	157.170	186.130	70.770	90.890	4.330
120	alpha	1.2	60x8 weeks	PCR90	M	VAC70	25	All	0.947	0.005	0.049	0.054	0.021	0.027	0.001	454.324	2.615	23.453	26.068	10.259	12.932	0.395	4543	26.150	234.530	260.680	102.590	129.320	3.950
120	alpha	1.2	60x8 weeks	PCR90	M	VAC70	25	All	0.947	0.005	0.049	0.054	0.021	0.027	0.001	454.324	2.615	23.453	26.068	10.259	12.932	0.395	4543	26.150	234.530	260.680	102.590	129.320	3.950
120	alpha	1.2	60x8 weeks	PCR90	M	VAC70	25	All	0.947	0.005	0.049	0.054	0.021	0.027	0.001	454.324	2.615	23.453	26.068	10.259	12.932	0.395	4543	26.150	234.530	260.680	102.590	129.320	3.950
120	delta	0.6	60x8 weeks	PCR90	M	VAC70	25	All	0.934	0.005	0.059	0.064	0.028	0.030	0.001	448.457	2.465	28.290	30.755	13.429	14.288	0.371	4485	24.650	282.900	307.550	134.290	142.880	3.710
120	delta	0.6	60x8 weeks	PCR90	M	VAC70	25	All	0.934	0.005	0.059	0.064	0.028	0.030	0.001	448.457	2.465	28.290	30.755	13.429	14.288	0.371	4485	24.650	282.900	307.550	134.290	142.880	3.710
120	delta	0.6	60x8 weeks	PCR90	M	VAC70	25	All	0.934	0.005	0.059	0.064	0.028	0.030	0.001	448.457	2.465	28.290	30.755	13.429	14.288	0.371	4485	24.650	282.900	307.550	134.290	142.880	3.710
120	delta	1	60x8 weeks	PCR90	M	VAC70	25	All	0.883	0.004	0.103	0.106	0.047	0.052	0.001	423.642	1.802	49.299	51.101	22.733	24.826	0.282	4236	18.020	492.990	511.010	227.330	248.260	2.820
120	delta	1	60x8 weeks	PCR90	M	VAC70	25	All	0.883	0.004	0.103	0.106	0.047	0.052	0.001	423.642	1.802	49.299	51.101	22.733	24.826	0.282	4236	18.020	492.990	511.010	227.330	248.260	2.820
120	delta	1	60x8 weeks	PCR90	M	VAC70	25	All	0.883	0.004	0.103	0.106	0.047	0.052	0.001	423.642	1.802	49.299	51.101	22.733	24.826	0.282	4236	18.020	492.990	511.010	227.330	248.260	2.820
120	delta	1.2	60x8 weeks	PCR90	M	VAC70	25	All	0.876	0.003	0.108	0.112	0.050	0.055	0.001	420.587	1.622	52.054	53.676	23.789	26.170	0.259	4206	16.220	520.540	536.760	237.890	261.700	2.590
120	delta	1.2	60x8 weeks	PCR90	M	VAC70	25	All	0.876	0.003	0.108	0.112	0.050	0.055	0.001	420.587	1.622	52.054	53.676	23.789	26.170	0.259	4206	16.220	520.540	536.760	237.890	261.700	2.590
120	delta	1.2	60x8 weeks	PCR90	M	VAC70	25	All	0.876	0.003	0.108	0.112	0.050	0.055	0.001	420.587	1.622	52.054	53.676	23.789	26.170	0.259	4206	16.220	520.540	536.760	237.890	261.700	2.590
120	original	0.6	60x8 weeks	PCR90	M	VAC70	50	All	0.994	0.005	0.001	0.007	0.003	0.003	0.001	477.313	2.625	0.684	3.309	1.208	1.217	0.488	4773	26.250	6.840	33.090	12.080	12.170	4.880
120	original	0.6	60x8 weeks	PCR90	M	VAC70	50	All	0.994	0.005	0.001	0.007	0.003	0.003	0.001	477.313	2.625	0.684	3.309	1.208	1.217	0.488	4773	26.250	6.840	33.090	12.080	12.170	4.880
120	original	0.6	60x8 weeks	PCR90	M	VAC70	50	All	0.994	0.005	0.001	0.007	0.003	0.003	0.001	477.313	2.625	0.684	3.309	1.208	1.217	0.488	4773	26.250	6.840	33.090	12.080	12.170	4.880
120	original	1	60x8 weeks	PCR90	M	VAC70	50	All	0.993	0.005	0.003	0.008	0.003	0.003	0.001	476.810	2.634	1.266	3.900	1.528	1.321	0.473	4768	26.340	12.660	39.000	15.280	13.210	4.730
120	original	1	60x8 weeks	PCR90	M	VAC70	50	All	0.993	0.005	0.003	0.008	0.003	0.003	0.001	476.810	2.634	1.266	3.900	1.528	1.321	0.473	4768	26.340	12.660	39.000	15.280	13.210	4.730
120	original	1	60x8 weeks	PCR90	M	VAC70	50	All	0.993	0.005	0.003	0.008	0.003	0.003	0.001	476.810	2.634	1.266	3.900	1.528	1.321	0.473	4768	26.340	12.660	39.000	15.280	13.210	4.730
120	original	1.2	60x8 weeks	PCR90	M	VAC70	50	All	0.993	0.005	0.003	0.009	0.003	0.003	0.001	476.608	2.546	1.626	4.172	1.585	1.466	0.478	4766	25.460	16.260	41.720	15.850	14.660	4.780
120	original	1.2	60x8 weeks	PCR90	M	VAC70	50	All	0.993	0.005	0.003	0.009	0.003	0.003	0.001	476.608	2.546	1.626	4.172	1.585	1.466	0.478	4766	25.460	16.260	41.720	15.850	14.660	4.780
120	original	1.2	60x8 weeks	PCR90	M	VAC70	50	All	0.993	0.005	0.003	0.009	0.003	0.003	0.001	476.608	2.546	1.626	4.172	1.585	1.466	0.478	4766	25.460	16.260	41.720	15.850	14.660	4.780
120	alpha	0.6	60x8 weeks	PCR90	M	VAC70	50	All	0.991	0.005	0.004	0.010	0.004	0.004	0.001	475.720	2.556	2.053	4.609	1.797	2.146	0.466	4757	25.560	20.530	46.090	17.970	21.460	4.660
120	alpha	0.6	60x8 weeks	PCR90	M	VAC70	50	All	0.991	0.005	0.004	0.010	0.004	0.004	0.001	475.720	2.556	2.053	4.609	1.797	2.146	0.466	4757	25.560	20.530	46.090	17.970	21.460	4.660
120	alpha	0.6	60x8 weeks	PCR90	M	VAC70	50	All	0.991	0.005	0.004	0.010	0.004	0.004	0.001	475.720	2.556	2.053	4.609	1.797	2.146	0.466	4757	25.560	20.530	46.090	17.970	21.460	4.660
120	alpha	1	60x8 weeks	PCR90	M	VAC70	50	All	0.985	0.005	0.011	0.017	0.006	0.008	0.001	472.607	2.553	5.440	7.993	2.973	3.918	0.476	4726	25.530	54.400	79.930	29.730	39.180	4.760
120	alpha	1	60x8 weeks	PCR90	M	VAC70	50	All	0.985	0.005	0.011	0.017	0.006	0.008	0.001	472.607	2.553	5.440	7.993	2.973	3.918	0.476	4726	25.530	54.400	79.930	29.730	39.180	4.760
120	alpha	1	60x8 weeks	PCR90	M	VAC70	50	All	0.985	0.005	0.011	0.017	0.006	0.008	0.001	472.607	2.553	5.440	7.993	2.973	3.918	0.476	4726	25.530	54.400	79.930	29.730	39.180	4.760
120	alpha	1.2	60x8 weeks	PCR90	M	VAC70	50	All	0.978	0.005	0.018	0.023	0.009	0.011	0.001	469.477	2.431	8.657	11.088	4.322	5.214	0.430	4695	24.310	86.570	110.880	43.220	52.140	4.300
120	alpha	1.2	60x8 weeks	PCR90	M	VAC70	50	All	0.978	0.005	0.018	0.023	0.009	0.011	0.001	469.477	2.431	8.657	11.088	4.322	5.214	0.430	4695	24.310	86.570	110.880	43.220	52.140	4.300
120	alpha	1.2	60x8 weeks	PCR90	M	VAC70	50	All	0.978	0.005	0.018	0.023	0.009	0.011	0.001	469.477	2.431	8.657	11.088	4.322	5.214	0.430	4695	24.310	86.570	110.880	43.220	52.140	4.300
120	delta	0.6	60x8 weeks	PCR90	M	VAC70	50	All	0.971	0.005	0.025	0.030	0.013	0.014	0.001	465.925	2.476	12.063	14.539	6.149	6.608	0.433	4659	24.760	120.630	145.390	61.490	66.080	4.330
120	delta	0.6	60x8 weeks	PCR90	M	VAC70	50	All	0.971	0.005	0.025	0.030	0.013	0.014	0.001	465.925	2.476	12.063	14.539	6.149	6.608	0.433	4659	24.760	120.630	145.390	61.490	66.080	4.330
120	delta	0.6	60x8 weeks	PCR90	M	VAC70	50	All	0.971	0.005	0.025	0.030	0.013	0.014	0.001	465.925	2.476	12.063	14.539	6.149	6.608	0.433	4659	24.760	120.630	145.390	61.490	66.080	4.330
120	delta	1	60x8 weeks	PCR90	M	VAC70	50	All	0.920	0.004	0.074	0.078	0.034	0.036	0.001	441.660	1.972	35.313	37.285	16.200	17.423	0.361	4417	19.720	353.130	372.850	162.000	174.230	3.610
120	delta	1	60x8 weeks	PCR90	M	VAC70	50	All	0.920	0.004	0.074	0.078	0.034	0.036	0.001	441.660	1.972	35.313	37.285	16.200	17.423	0.361	4417	19.720	353.130	372.850	162.000	174.230	3.610
120	delta	1	60x8 weeks	PCR90	M	VAC70	50	All	0.920	0.004	0.074	0.078	0.034	0.036	0.001	441.660	1.972	35.313	37.285	16.200	17.423	0.361	4417	19.720	353.130	372.850	162.000	174.230	3.610

120	delta	1.2	60x8 weeks	PCR90	M	VAC70	50	All	0.904	0.004	0.087	0.091	0.040	0.043	0.001	434.019	1.741	41.802	43.543	19.073	20.504	0.291	4340	17.410	418.020	435.430	190.730	205.040	2.910
120	delta	1.2	60x8 weeks	PCR90	M	VAC70	50	All	0.904	0.004	0.087	0.091	0.040	0.043	0.001	434.019	1.741	41.802	43.543	19.073	20.504	0.291	4340	17.410	418.020	435.430	190.730	205.040	2.910
120	delta	1.2	60x8 weeks	PCR90	M	VAC70	50	All	0.904	0.004	0.087	0.091	0.040	0.043	0.001	434.019	1.741	41.802	43.543	19.073	20.504	0.291	4340	17.410	418.020	435.430	190.730	205.040	2.910
120	original	0.6	60x8 weeks	PCR90	M	VAC70	75	All	0.997	0.004	0.000	0.004	0.002	0.001	0.001	478.346	1.908	0.209	2.117	0.852	0.542	0.455	4783	19.080	2.090	21.170	8.520	5.420	4.550
120	original	0.6	60x8 weeks	PCR90	M	VAC70	75	All	0.997	0.004	0.000	0.004	0.002	0.001	0.001	478.346	1.908	0.209	2.117	0.852	0.542	0.455	4783	19.080	2.090	21.170	8.520	5.420	4.550
120	original	0.6	60x8 weeks	PCR90	M	VAC70	75	All	0.997	0.004	0.000	0.004	0.002	0.001	0.001	478.346	1.908	0.209	2.117	0.852	0.542	0.455	4783	19.080	2.090	21.170	8.520	5.420	4.550
120	original	1	60x8 weeks	PCR90	M	VAC70	75	All	0.996	0.004	0.001	0.005	0.002	0.001	0.001	478.219	1.924	0.399	2.323	0.957	0.615	0.482	4782	19.240	3.990	23.230	9.570	6.150	4.820
120	original	1	60x8 weeks	PCR90	M	VAC70	75	All	0.996	0.004	0.001	0.005	0.002	0.001	0.001	478.219	1.924	0.399	2.323	0.957	0.615	0.482	4782	19.240	3.990	23.230	9.570	6.150	4.820
120	original	1	60x8 weeks	PCR90	M	VAC70	75	All	0.996	0.004	0.001	0.005	0.002	0.001	0.001	478.219	1.924	0.399	2.323	0.957	0.615	0.482	4782	19.240	3.990	23.230	9.570	6.150	4.820
120	original	1.2	60x8 weeks	PCR90	M	VAC70	75	All	0.996	0.004	0.001	0.005	0.002	0.001	0.001	478.178	1.940	0.512	2.452	0.967	0.616	0.449	4782	19.400	5.120	24.520	9.670	6.160	4.490
120	original	1.2	60x8 weeks	PCR90	M	VAC70	75	All	0.996	0.004	0.001	0.005	0.002	0.001	0.001	478.178	1.940	0.512	2.452	0.967	0.616	0.449	4782	19.400	5.120	24.520	9.670	6.160	4.490
120	original	1.2	60x8 weeks	PCR90	M	VAC70	75	All	0.996	0.004	0.001	0.005	0.002	0.001	0.001	478.178	1.940	0.512	2.452	0.967	0.616	0.449	4782	19.400	5.120	24.520	9.670	6.160	4.490
120	alpha	0.6	60x8 weeks	PCR90	M	VAC70	75	All	0.995	0.004	0.002	0.006	0.002	0.003	0.001	477.520	1.918	0.792	2.710	0.995	1.303	0.504	4775	19.180	7.920	27.100	9.950	13.030	5.040
120	alpha	0.6	60x8 weeks	PCR90	M	VAC70	75	All	0.995	0.004	0.002	0.006	0.002	0.003	0.001	477.520	1.918	0.792	2.710	0.995	1.303	0.504	4775	19.180	7.920	27.100	9.950	13.030	5.040
120	alpha	0.6	60x8 weeks	PCR90	M	VAC70	75	All	0.995	0.004	0.002	0.006	0.002	0.003	0.001	477.520	1.918	0.792	2.710	0.995	1.303	0.504	4775	19.180	7.920	27.100	9.950	13.030	5.040
120	alpha	1	60x8 weeks	PCR90	M	VAC70	75	All	0.993	0.004	0.004	0.008	0.003	0.004	0.001	476.562	1.936	1.775	3.711	1.377	1.769	0.483	4766	19.360	17.750	37.110	13.770	17.690	4.830
120	alpha	1	60x8 weeks	PCR90	M	VAC70	75	All	0.993	0.004	0.004	0.008	0.003	0.004	0.001	476.562	1.936	1.775	3.711	1.377	1.769	0.483	4766	19.360	17.750	37.110	13.770	17.690	4.830
120	alpha	1	60x8 weeks	PCR90	M	VAC70	75	All	0.993	0.004	0.004	0.008	0.003	0.004	0.001	476.562	1.936	1.775	3.711	1.377	1.769	0.483	4766	19.360	17.750	37.110	13.770	17.690	4.830
120	alpha	1.2	60x8 weeks	PCR90	M	VAC70	75	All	0.991	0.004	0.005	0.009	0.003	0.004	0.001	475.900	1.843	2.478	4.321	1.669	2.027	0.478	4759	18.430	24.780	43.210	16.690	20.270	4.780
120	alpha	1.2	60x8 weeks	PCR90	M	VAC70	75	All	0.991	0.004	0.005	0.009	0.003	0.004	0.001	475.900	1.843	2.478	4.321	1.669	2.027	0.478	4759	18.430	24.780	43.210	16.690	20.270	4.780
120	alpha	1.2	60x8 weeks	PCR90	M	VAC70	75	All	0.991	0.004	0.005	0.009	0.003	0.004	0.001	475.900	1.843	2.478	4.321	1.669	2.027	0.478	4759	18.430	24.780	43.210	16.690	20.270	4.780
120	delta	0.6	60x8 weeks	PCR90	M	VAC70	75	All	0.988	0.004	0.008	0.012	0.005	0.006	0.001	474.366	2.055	3.819	5.874	2.469	2.641	0.468	4744	20.550	38.190	58.740	24.690	26.410	4.680
120	delta	0.6	60x8 weeks	PCR90	M	VAC70	75	All	0.988	0.004	0.008	0.012	0.005	0.006	0.001	474.366	2.055	3.819	5.874	2.469	2.641	0.468	4744	20.550	38.190	58.740	24.690	26.410	4.680
120	delta	0.6	60x8 weeks	PCR90	M	VAC70	75	All	0.988	0.004	0.008	0.012	0.005	0.006	0.001	474.366	2.055	3.819	5.874	2.469	2.641	0.468	4744	20.550	38.190	58.740	24.690	26.410	4.680
120	delta	1	60x8 weeks	PCR90	M	VAC70	75	All	0.970	0.004	0.028	0.032	0.013	0.014	0.001	465.476	1.922	13.412	15.334	6.412	6.880	0.442	4655	19.220	134.120	153.340	64.120	68.800	4.420
120	delta	1	60x8 weeks	PCR90	M	VAC70	75	All	0.970	0.004	0.028	0.032	0.013	0.014	0.001	465.476	1.922	13.412	15.334	6.412	6.880	0.442	4655	19.220	134.120	153.340	64.120	68.800	4.420
120	delta	1	60x8 weeks	PCR90	M	VAC70	75	All	0.970	0.004	0.028	0.032	0.013	0.014	0.001	465.476	1.922	13.412	15.334	6.412	6.880	0.442	4655	19.220	134.120	153.340	64.120	68.800	4.420
120	delta	1.2	60x8 weeks	PCR90	M	VAC70	75	All	0.952	0.004	0.045	0.049	0.021	0.023	0.001	456.795	1.855	21.722	23.577	9.955	10.874	0.415	4568	18.550	217.220	235.770	99.550	108.740	4.150
120	delta	1.2	60x8 weeks	PCR90	M	VAC70	75	All	0.952	0.004	0.045	0.049	0.021	0.023	0.001	456.795	1.855	21.722	23.577	9.955	10.874	0.415	4568	18.550	217.220	235.770	99.550	108.740	4.150
120	delta	1.2	60x8 weeks	PCR90	M	VAC70	75	All	0.952	0.004	0.045	0.049	0.021	0.023	0.001	456.795	1.855	21.722	23.577	9.955	10.874	0.415	4568	18.550	217.220	235.770	99.550	108.740	4.150
120	original	0.6	60x8 weeks	PCR90	M	VAC70	100	All	0.998	0.003	0.000	0.003	0.001	0.000	0.001	479.168	1.298	0.012	1.310	0.609	0.070	0.442	4792	12.980	0.120	13.100	6.090	0.700	4.420
120	original	0.6	60x8 weeks	PCR90	M	VAC70	100	All	0.998	0.003	0.000	0.003	0.001	0.000	0.001	479.168	1.298	0.012	1.310	0.609	0.070	0.442	4792	12.980	0.120	13.100	6.090	0.700	4.420
120	original	0.6	60x8 weeks	PCR90	M	VAC70	100	All	0.998	0.003	0.000	0.003	0.001	0.000	0.001	479.168	1.298	0.012	1.310	0.609	0.070	0.442	4792	12.980	0.120	13.100	6.090	0.700	4.420
120	original	1	60x8 weeks	PCR90	M	VAC70	100	All	0.998	0.003	0.000	0.003	0.001	0.000	0.001	479.229	1.267	0.013	1.280	0.575	0.057	0.461	4792	12.670	0.130	12.800	5.750	0.570	4.610
120	original	1	60x8 weeks	PCR90	M	VAC70	100	All	0.998	0.003	0.000	0.003	0.001	0.000	0.001	479.229	1.267	0.013	1.280	0.575	0.057	0.461	4792	12.670	0.130	12.800	5.750	0.570	4.610
120	original	1	60x8 weeks	PCR90	M	VAC70	100	All	0.998	0.003	0.000	0.003	0.001	0.000	0.001	479.229	1.267	0.013	1.280	0.575	0.057	0.461	4792	12.670	0.130	12.800	5.750	0.570	4.610
120	original	1.2	60x8 weeks	PCR90	M	VAC70	100	All	0.998	0.003	0.000	0.003	0.001	0.000	0.001	479.174	1.282	0.027	1.309	0.612	0.063	0.453	4792	12.820	0.270	13.090	6.120	0.630	4.530
120	original	1.2	60x8 weeks	PCR90	M	VAC70	100	All	0.998	0.003	0.000	0.003	0.001	0.000	0.001	479.174	1.282	0.027	1.309	0.612	0.063	0.453	4792	12.820	0.270	13.090	6.120	0.630	4.530
120	original	1.2	60x8 weeks	PCR90	M	VAC70	100	All	0.998	0.003	0.000	0.003	0.001	0.000	0.001	479.174	1.282	0.027	1.309	0.612	0.063	0.453	4792	12.820	0.270	13.090	6.120	0.630	4.530
120	alpha	0.6	60x8 weeks	PCR90	M	VAC70	100	All	0.997	0.003	0.000	0.003	0.001	0.001	0.001	478.658	1.273	0.209	1.482	0.553	0.675	0.483	4787	12.730	2.090	14.820	5.530	6.750	4.830
120	alpha	0.6	60x8 weeks	PCR90	M	VAC70	100	All	0.997	0.003	0.000	0.003	0.001	0.001	0.001	478.658	1.273	0.209	1.482	0.553	0.675	0.483	4787	12.730	2.090	14.820	5.530	6.750	4.830
120	alpha	0.6	60x8 weeks	PCR90	M	VAC70	100	All	0.997	0.003	0.000	0.003	0.001	0.001	0.001	478.658	1.273	0.209	1.482	0.553	0.675	0.483	4787	12.730	2.090	14.820	5.530	6.750	4.830
120	alpha	1	60x8 weeks	PCR90	M	VAC70	100	All	0.997	0.003	0.001	0.004	0.001	0.002	0.001	478.514	1.296	0.398	1.694	0.611	0.758	0.470	4785	12.960	3.980	16.940	6.110	7.580	4.700
120	alpha	1	60x8 weeks	PCR90	M	VAC70	100	All	0.997	0.003	0.001	0.004	0.001	0.002	0.001	478.514	1.296	0.398	1.694	0.611	0.758	0.470	4785	12.960	3.980	16.940	6.110	7.580	4.700
120	alpha	1	60x8 weeks	PCR90	M	VAC70	100	All	0.997	0.003	0.001	0.004	0.001	0.002	0.001	478.514	1.296	0.398	1.694	0.611	0.758	0.470	4785	12.960	3.980	16.940	6.110	7.580	4.700
120	alpha	1.2	60x8 weeks	PCR90	M	VAC70	100	All	0.996	0.003	0.001	0.004	0.001	0.002	0.001	478.286	1.329	0.505	1.834	0.707	0.845	0.468	4783	13.290	5.050	18.340	7.070	8.450	4.680
120	alpha	1.2	60x8 weeks	PCR90	M	VAC70	100	All	0.996	0.003	0.001	0.004	0.001	0.002	0.001	478.286	1.329	0.505	1.834	0.707	0.845	0.468	4783	13.290	5.050	18.340	7.070	8.450	4.680
120	alpha	1.2	60x8 weeks	PCR90	M	VAC70	100	All	0.996	0.003	0.001	0.004	0.001	0.002	0.001	478.286	1.329	0.505	1.834	0.707	0.845	0.468	4783	13.290	5.050	18.340	7.070	8.450	4.680
120	delta	0.6	60x8 weeks	PCR90	M	VAC70	100	All	0.995	0.003	0.002	0.005	0.002	0.002	0.001	477.522	1.614	0.977	2.591	1.112	1.139	0.449	4775	16.140	9.770	25.910	11.120	11.390	4.490
120	delta	0.6	60x8 weeks	PCR90	M	VAC70	100	All	0.995	0.003	0.002	0.005	0.002	0.002	0.001	477.522	1.614	0.977	2.591	1.112	1.139	0.449	4775	16.140	9.770	25.910	11.120	11.390	4.490
120	delta	0.6	60x8 weeks	PCR90	M	VAC70	100	All	0.995	0.003	0.002	0.005	0.002	0.002	0.001	477.522	1.614	0.977	2.591	1.112	1.139	0.449	4775	16.140	9.770	25.910	11.120	11.390	4.490
120	delta	1	60x8 weeks	PCR90	M	VAC70	100	All	0.992	0.003	0.005	0.009	0.004	0.004	0.001	476.168	1.651	2.453	4.104	1.702	1.820	0.494	4762	16.510	24.530	41.040	17.020	18.200	4.940
120	delta	1	60x8 weeks	PCR90	M	VAC70	100	All	0.992	0.003	0.005	0.009	0.004	0.004	0.001	476.168	1.651	2.453	4.104	1.702	1.820	0.494	4762	16.510	24.530	41.040	17.020	18.200	4.940
120	delta	1	60x8 weeks	PCR90	M	VAC70	100	All	0.992	0.003	0.005	0.009	0.004	0.004	0.001	476.168	1.651	2.453	4.104	1.702	1.820	0.494	4762	16.510	24.530	41.040	17.020	18.200	4.940
120	delta	1.2	60x8 weeks	PCR90	M	VAC70	100	All	0.990	0.003	0.007	0.011	0.004	0.005	0.001	475.302	1.567	3.542	5.109	2.092	2.290	0.467	4753	15.670	35.420	51.090	20.920	22.900	4.670
120	delta	1.2	60x8 weeks	PCR90	M	VAC70	100	All	0.990	0.003	0.007	0.011	0.004	0.005	0.001	475.302	1.567	3.542	5.109	2.092	2.290	0.467	4753	15.670	35.420	51.090	20.920	22.900	4.670
120	delta	1.2	60x8 weeks	PCR90	M	VAC70	100	All	0.990	0.003	0.007	0.011	0.004	0.005	0.001	475.302	1.567	3.542	5.109	2.092	2.290	0.467	4753	15.670	35.420	51.090	20.920	22.900	4.670
120	original	0.6	60x8 weeks	PCR90	M	VAC50	25	All	0.991	0.007	0.003	0.011	0.004	0.004	0.001	475.807	3.529	1.562	5.091	1.800	2.055	0.515	4758	35.290	15.620	50.910	18.000	20.550	5.150
120	original	0.6	60x8 weeks	PCR90	M	VAC50	25	All	0.991	0.007	0.003	0.011	0.004	0.004	0.001	475.807	3.529	1.562	5.091	1.800	2.055	0.515	4758	35.290	15.620	50.910	18.000	20.550	5.150
120	original	0.6	60x8 weeks	PCR90	M	VAC50	25	All	0.991	0.007	0.003	0.011	0.004	0.004	0.001	475.807	3.529	1.562	5.091	1.800	2.055	0.515	4758	35.290	15.620	50.910	18.000	20.550	5.150
120	original	1	60x8 weeks	PCR90	M	VAC50	25	All	0.988	0.007	0.007	0.014	0.005	0.006	0.001	474.201	3.542	3.363	6.905	2.566	2.690	0.473	4742	35.420	33.630	69.050	25.660	26.900	4.730
120	original	1	60x8 weeks	PCR90	M	VAC50	25	All	0.988	0.007	0.007	0.014	0.005	0.006	0.001	474.201	3.542	3.363	6.905	2.566	2.690	0.473	4742	35.420	33.630	69.050	25.660	26.900	4.730
120	original	1	60x8 weeks	PCR90	M	VAC50	25	All	0.988	0.007	0.007	0.014	0.005	0.006	0.001	474.201	3.542	3.363	6.905	2.566	2.690	0.473	4742	35.420	33.630	69.050	25.660	26.900	4.730
120	original	1.2	60x8 weeks	PCR90	M	VAC50	25	All	0.987	0.007	0.009	0.016	0.006	0.006	0.001	473.534	3.361	4.423	7.784	2.867	3.109	0.450	4735	33.610	44.230	77.840	28.670	31.090	4.500
120	original	1.2	60x8 weeks	PCR90	M	VAC50	25	All	0.987	0.007	0.009	0.016	0.006	0.006	0.001	473.534	3.361	4.423	7.784	2.867	3.109	0.450	4735	33.610	44.230	77.840	28.670	31.090	4.500
120	original	1.2	60x8 weeks	PCR90	M	VAC50	25	All	0.987	0.007	0.009	0.016	0.006	0.006	0.001	473.534	3.361	4.423	7.784	2.867	3.109	0.450	4735	33.610	44.230	77.840	28.670	31.090	4.500
120	alpha	0.6	60x8 weeks	PCR90	M	VAC50	25	All	0.982	0.007	0.012	0.019	0.007	0.009	0.001	471.573	3.377	5.636	9.013	3.439	4.346	0.420	4716	33.770	56.360	90.130	34.390	43.460	4.200
120	alpha	0.6	60x8 weeks	PCR90	M	VAC50	25	All	0.982	0.007	0.012	0.019	0.007	0.009	0.001	471.573	3.377	5.636	9.013	3.439	4.346	0.420	4716	33.770	56.360	90.130	34.390	43.460	4.200
120	alpha	0.6	60x8 weeks	PCR90	M	VAC50	25	All	0.982	0.007	0.012	0.019	0.007	0.009	0.001	471.573	3.377	5.636	9.013	3.439	4.346	0.420	4716	33.770	56.360	90.130	34.390	43.460	4.200
120	alpha	1	60x8 weeks	PCR90	M	VAC50	25	All	0.959	0.006	0.037	0.043	0.017	0.021	0.001	460.442	2.882	17.775	20.657	8.011	9.915	0.423	4604	28.820	177.750	206.570	80.110	99.150	4.230
120	alpha	1	60x8 weeks	PCR90	M	VAC50	25	All	0.959	0.006	0.037	0.043	0.017	0.021	0.001	460.442	2.882	17.775	20.657	8.011	9.915	0.423	4604	28.820	177.750	206.570	80.110	99.150	4.230

120	alpha	1	60x8 weeks	PCR90	M	VAC50	25	All	0.959	0.006	0.037	0.043	0.017	0.021	0.001	460.442	2.882	17.775	20.657	8.011	9.915	0.423	4604	28.820	177.750	206.570	80.110	99.150	4.230
120	alpha	1.2	60x8 weeks	PCR90	M	VAC50	25	All	0.941	0.006	0.055	0.061	0.024	0.030	0.001	451.565	2.702	26.486	29.188	11.426	14.274	0.337	4516	27.020	264.860	291.880	114.260	142.740	3.370
120	alpha	1.2	60x8 weeks	PCR90	M	VAC50	25	All	0.941	0.006	0.055	0.061	0.024	0.030	0.001	451.565	2.702	26.486	29.188	11.426	14.274	0.337	4516	27.020	264.860	291.880	114.260	142.740	3.370
120	alpha	1.2	60x8 weeks	PCR90	M	VAC50	25	All	0.941	0.006	0.055	0.061	0.024	0.030	0.001	451.565	2.702	26.486	29.188	11.426	14.274	0.337	4516	27.020	264.860	291.880	114.260	142.740	3.370
120	delta	0.6	60x8 weeks	PCR90	M	VAC50	25	All	0.917	0.005	0.073	0.079	0.035	0.037	0.001	440.377	2.585	35.135	37.720	16.569	17.736	0.319	4404	25.850	351.350	377.200	165.690	177.360	3.190
120	delta	0.6	60x8 weeks	PCR90	M	VAC50	25	All	0.917	0.005	0.073	0.079	0.035	0.037	0.001	440.377	2.585	35.135	37.720	16.569	17.736	0.319	4404	25.850	351.350	377.200	165.690	177.360	3.190
120	delta	0.6	60x8 weeks	PCR90	M	VAC50	25	All	0.917	0.005	0.073	0.079	0.035	0.037	0.001	440.377	2.585	35.135	37.720	16.569	17.736	0.319	4404	25.850	351.350	377.200	165.690	177.360	3.190
120	delta	1	60x8 weeks	PCR90	M	VAC50	25	All	0.874	0.004	0.109	0.113	0.051	0.055	0.001	419.598	1.791	52.410	54.201	24.428	26.213	0.253	4196	17.910	524.100	542.010	244.280	262.130	2.530
120	delta	1	60x8 weeks	PCR90	M	VAC50	25	All	0.874	0.004	0.109	0.113	0.051	0.055	0.001	419.598	1.791	52.410	54.201	24.428	26.213	0.253	4196	17.910	524.100	542.010	244.280	262.130	2.530
120	delta	1	60x8 weeks	PCR90	M	VAC50	25	All	0.874	0.004	0.109	0.113	0.051	0.055	0.001	419.598	1.791	52.410	54.201	24.428	26.213	0.253	4196	17.910	524.100	542.010	244.280	262.130	2.530
120	delta	1.2	60x8 weeks	PCR90	M	VAC50	25	All	0.872	0.003	0.111	0.115	0.051	0.056	0.000	418.448	1.663	53.379	55.042	24.485	27.014	0.230	4184	16.630	533.790	550.420	244.850	270.140	2.300
120	delta	1.2	60x8 weeks	PCR90	M	VAC50	25	All	0.872	0.003	0.111	0.115	0.051	0.056	0.000	418.448	1.663	53.379	55.042	24.485	27.014	0.230	4184	16.630	533.790	550.420	244.850	270.140	2.300
120	delta	1.2	60x8 weeks	PCR90	M	VAC50	25	All	0.872	0.003	0.111	0.115	0.051	0.056	0.000	418.448	1.663	53.379	55.042	24.485	27.014	0.230	4184	16.630	533.790	550.420	244.850	270.140	2.300
120	original	0.6	60x8 weeks	PCR90	M	VAC50	50	All	0.994	0.006	0.002	0.007	0.003	0.002	0.001	477.083	2.877	0.721	3.598	1.404	1.155	0.491	4771	28.770	7.210	35.980	14.040	11.550	4.910
120	original	0.6	60x8 weeks	PCR90	M	VAC50	50	All	0.994	0.006	0.002	0.007	0.003	0.002	0.001	477.083	2.877	0.721	3.598	1.404	1.155	0.491	4771	28.770	7.210	35.980	14.040	11.550	4.910
120	original	0.6	60x8 weeks	PCR90	M	VAC50	50	All	0.994	0.006	0.002	0.007	0.003	0.002	0.001	477.083	2.877	0.721	3.598	1.404	1.155	0.491	4771	28.770	7.210	35.980	14.040	11.550	4.910
120	original	1	60x8 weeks	PCR90	M	VAC50	50	All	0.993	0.006	0.003	0.009	0.004	0.003	0.001	476.446	2.926	1.554	4.480	1.760	1.395	0.458	4764	29.260	15.540	44.800	17.600	13.950	4.580
120	original	1	60x8 weeks	PCR90	M	VAC50	50	All	0.993	0.006	0.003	0.009	0.004	0.003	0.001	476.446	2.926	1.554	4.480	1.760	1.395	0.458	4764	29.260	15.540	44.800	17.600	13.950	4.580
120	original	1	60x8 weeks	PCR90	M	VAC50	50	All	0.993	0.006	0.003	0.009	0.004	0.003	0.001	476.446	2.926	1.554	4.480	1.760	1.395	0.458	4764	29.260	15.540	44.800	17.600	13.950	4.580
120	original	1.2	60x8 weeks	PCR90	M	VAC50	50	All	0.992	0.006	0.004	0.010	0.004	0.003	0.001	476.176	2.925	1.816	4.741	1.864	1.538	0.473	4762	29.250	18.160	47.410	18.640	15.380	4.730
120	original	1.2	60x8 weeks	PCR90	M	VAC50	50	All	0.992	0.006	0.004	0.010	0.004	0.003	0.001	476.176	2.925	1.816	4.741	1.864	1.538	0.473	4762	29.250	18.160	47.410	18.640	15.380	4.730
120	original	1.2	60x8 weeks	PCR90	M	VAC50	50	All	0.992	0.006	0.004	0.010	0.004	0.003	0.001	476.176	2.925	1.816	4.741	1.864	1.538	0.473	4762	29.250	18.160	47.410	18.640	15.380	4.730
120	alpha	0.6	60x8 weeks	PCR90	M	VAC50	50	All	0.989	0.006	0.006	0.012	0.005	0.006	0.001	474.586	2.889	2.845	5.734	2.268	2.657	0.466	4746	28.890	28.450	57.340	22.680	26.570	4.660
120	alpha	0.6	60x8 weeks	PCR90	M	VAC50	50	All	0.989	0.006	0.006	0.012	0.005	0.006	0.001	474.586	2.889	2.845	5.734	2.268	2.657	0.466	4746	28.890	28.450	57.340	22.680	26.570	4.660
120	alpha	0.6	60x8 weeks	PCR90	M	VAC50	50	All	0.989	0.006	0.006	0.012	0.005	0.006	0.001	474.586	2.889	2.845	5.734	2.268	2.657	0.466	4746	28.890	28.450	57.340	22.680	26.570	4.660
120	alpha	1	60x8 weeks	PCR90	M	VAC50	50	All	0.979	0.006	0.017	0.023	0.009	0.011	0.001	469.814	2.732	8.277	11.009	4.243	5.204	0.453	4698	27.320	82.770	110.090	42.430	52.040	4.530
120	alpha	1	60x8 weeks	PCR90	M	VAC50	50	All	0.979	0.006	0.017	0.023	0.009	0.011	0.001	469.814	2.732	8.277	11.009	4.243	5.204	0.453	4698	27.320	82.770	110.090	42.430	52.040	4.530
120	alpha	1	60x8 weeks	PCR90	M	VAC50	50	All	0.979	0.006	0.017	0.023	0.009	0.011	0.001	469.814	2.732	8.277	11.009	4.243	5.204	0.453	4698	27.320	82.770	110.090	42.430	52.040	4.530
120	alpha	1.2	60x8 weeks	PCR90	M	VAC50	50	All	0.971	0.006	0.026	0.032	0.012	0.015	0.001	466.036	2.747	12.441	15.188	5.936	7.147	0.425	4660	27.470	124.410	151.880	59.360	71.470	4.250
120	alpha	1.2	60x8 weeks	PCR90	M	VAC50	50	All	0.971	0.006	0.026	0.032	0.012	0.015	0.001	466.036	2.747	12.441	15.188	5.936	7.147	0.425	4660	27.470	124.410	151.880	59.360	71.470	4.250
120	alpha	1.2	60x8 weeks	PCR90	M	VAC50	50	All	0.971	0.006	0.026	0.032	0.012	0.015	0.001	466.036	2.747	12.441	15.188	5.936	7.147	0.425	4660	27.470	124.410	151.880	59.360	71.470	4.250
120	delta	0.6	60x8 weeks	PCR90	M	VAC50	50	All	0.952	0.006	0.043	0.049	0.021	0.022	0.001	456.824	2.689	20.592	23.281	10.129	10.578	0.412	4568	26.890	205.920	232.810	101.290	105.780	4.120
120	delta	0.6	60x8 weeks	PCR90	M	VAC50	50	All	0.952	0.006	0.043	0.049	0.021	0.022	0.001	456.824	2.689	20.592	23.281	10.129	10.578	0.412	4568	26.890	205.920	232.810	101.290	105.780	4.120
120	delta	0.6	60x8 weeks	PCR90	M	VAC50	50	All	0.952	0.006	0.043	0.049	0.021	0.022	0.001	456.824	2.689	20.592	23.281	10.129	10.578	0.412	4568	26.890	205.920	232.810	101.290	105.780	4.120
120	delta	1	60x8 weeks	PCR90	M	VAC50	50	All	0.897	0.004	0.093	0.098	0.043	0.046	0.001	430.513	2.034	44.767	46.801	20.612	22.102	0.317	4305	20.340	447.670	468.010	206.120	221.020	3.170
120	delta	1	60x8 weeks	PCR90	M	VAC50	50	All	0.897	0.004	0.093	0.098	0.043	0.046	0.001	430.513	2.034	44.767	46.801	20.612	22.102	0.317	4305	20.340	447.670	468.010	206.120	221.020	3.170
120	delta	1	60x8 weeks	PCR90	M	VAC50	50	All	0.897	0.004	0.093	0.098	0.043	0.046	0.001	430.513	2.034	44.767	46.801	20.612	22.102	0.317	4305	20.340	447.670	468.010	206.120	221.020	3.170
120	delta	1.2	60x8 weeks	PCR90	M	VAC50	50	All	0.884	0.004	0.103	0.107	0.048	0.052	0.001	424.230	1.786	49.638	51.424	22.895	24.726	0.259	4242	17.860	496.380	514.240	228.950	247.260	2.590
120	delta	1.2	60x8 weeks	PCR90	M	VAC50	50	All	0.884	0.004	0.103	0.107	0.048	0.052	0.001	424.230	1.786	49.638	51.424	22.895	24.726	0.259	4242	17.860	496.380	514.240	228.950	247.260	2.590
120	delta	1.2	60x8 weeks	PCR90	M	VAC50	50	All	0.884	0.004	0.103	0.107	0.048	0.052	0.001	424.230	1.786	49.638	51.424	22.895	24.726	0.259	4242	17.860	496.380	514.240	228.950	247.260	2.590
120	original	0.6	60x8 weeks	PCR90	M	VAC50	75	All	0.996	0.005	0.001	0.006	0.003	0.001	0.001	477.954	2.519	0.303	2.822	1.234	0.580	0.458	4780	25.190	3.030	28.220	12.340	5.800	4.580
120	original	0.6	60x8 weeks	PCR90	M	VAC50	75	All	0.996	0.005	0.001	0.006	0.003	0.001	0.001	477.954	2.519	0.303	2.822	1.234	0.580	0.458	4780	25.190	3.030	28.220	12.340	5.800	4.580
120	original	0.6	60x8 weeks	PCR90	M	VAC50	75	All	0.996	0.005	0.001	0.006	0.003	0.001	0.001	477.954	2.519	0.303	2.822	1.234	0.580	0.458	4780	25.190	3.030	28.220	12.340	5.800	4.580
120	original	1	60x8 weeks	PCR90	M	VAC50	75	All	0.995	0.005	0.001	0.006	0.003	0.001	0.001	477.728	2.546	0.531	3.077	1.314	0.658	0.444	4777	25.460	5.310	30.770	13.140	6.580	4.440
120	original	1	60x8 weeks	PCR90	M	VAC50	75	All	0.995	0.005	0.001	0.006	0.003	0.001	0.001	477.728	2.546	0.531	3.077	1.314	0.658	0.444	4777	25.460	5.310	30.770	13.140	6.580	4.440
120	original	1	60x8 weeks	PCR90	M	VAC50	75	All	0.995	0.005	0.001	0.006	0.003	0.001	0.001	477.728	2.546	0.531	3.077	1.314	0.658	0.444	4777	25.460	5.310	30.770	13.140	6.580	4.440
120	original	1.2	60x8 weeks	PCR90	M	VAC50	75	All	0.995	0.005	0.002	0.007	0.003	0.001	0.001	477.586	2.531	0.724	3.255	1.423	0.661	0.460	4776	25.310	7.240	32.550	14.230	6.610	4.600
120	original	1.2	60x8 weeks	PCR90	M	VAC50	75	All	0.995	0.005	0.002	0.007	0.003	0.001	0.001	477.586	2.531	0.724	3.255	1.423	0.661	0.460	4776	25.310	7.240	32.550	14.230	6.610	4.600
120	original	1.2	60x8 weeks	PCR90	M	VAC50	75	All	0.995	0.005	0.002	0.007	0.003	0.001	0.001	477.586	2.531	0.724	3.255	1.423	0.661	0.460	4776	25.310	7.240	32.550	14.230	6.610	4.600
120	alpha	0.6	60x8 weeks	PCR90	M	VAC50	75	All	0.992	0.005	0.003	0.008	0.003	0.004	0.001	476.334	2.446	1.415	3.861	1.493	1.818	0.502	4763	24.460	14.150	38.610	14.930	18.180	5.020
120	alpha	0.6	60x8 weeks	PCR90	M	VAC50	75	All	0.992	0.005	0.003	0.008	0.003	0.004	0.001	476.334	2.446	1.415	3.861	1.493	1.818	0.502	4763	24.460	14.150	38.610	14.930	18.180	5.020
120	alpha	0.6	60x8 weeks	PCR90	M	VAC50	75	All	0.992	0.005	0.003	0.008	0.003	0.004	0.001	476.334	2.446	1.415	3.861	1.493	1.818	0.502	4763	24.460	14.150	38.610	14.930	18.180	5.020
120	alpha	1	60x8 weeks	PCR90	M	VAC50	75	All	0.989	0.005	0.007	0.012	0.005	0.006	0.001	474.787	2.438	3.214	5.652	2.169	2.640	0.441	4748	24.380	32.140	56.520	21.690	26.400	4.410
120	alpha	1	60x8 weeks	PCR90	M	VAC50	75	All	0.989	0.005	0.007	0.012	0.005	0.006	0.001	474.787	2.438	3.214	5.652	2.169	2.640	0.441	4748	24.380	32.140	56.520	21.690	26.400	4.410
120	alpha	1	60x8 weeks	PCR90	M	VAC50	75	All	0.989	0.005	0.007	0.012	0.005	0.006	0.001	474.787	2.438	3.214	5.652	2.169	2.640	0.441	4748	24.380	32.140	56.520	21.690	26.400	4.410
120	alpha	1.2	60x8 weeks	PCR90	M	VAC50	75	All	0.985	0.005	0.010	0.015	0.006	0.007	0.001	473.003	2.511	4.892	7.403	2.855	3.494	0.448	4730	25.110	48.920	74.030	28.550	34.940	4.480
120	alpha	1.2	60x8 weeks	PCR90	M	VAC50	75	All	0.985	0.005	0.010	0.015	0.006	0.007	0.001	473.003	2.511	4.892	7.403	2.855	3.494	0.448	4730	25.110	48.920	74.030	28.550	34.940	4.480
120	alpha	1.2	60x8 weeks	PCR90	M	VAC50	75	All	0.985	0.005	0.010	0.015	0.006	0.007	0.001	473.003	2.511	4.892	7.403	2.855	3.494	0.448	4730	25.110	48.920	74.030	28.550	34.940	4.480
120	delta	0.6	60x8 weeks	PCR90	M	VAC50	75	All	0.976	0.006	0.019	0.025	0.011	0.011	0.001	468.597	2.781	9.200	11.981	5.161	5.246	0.450	4686	27.810	92.000	119.810	51.610	52.460	4.500
120	delta	0.6	60x8 weeks	PCR90	M	VAC50	75	All	0.976	0.006	0.019	0.025	0.011	0.011	0.001	468.597	2.781	9.200	11.981	5.161	5.246	0.450	4686	27.810	92.000	119.810	51.610	52.460	4.500
120	delta	0.6	60x8 weeks	PCR90	M	VAC50	75	All	0.976	0.006	0.019	0.025	0.011	0.011	0.001	468.597	2.781	9.200	11.981	5.161	5.246	0.450	4686	27.810	92.000	119.810	51.610	52.460	4.500
120	delta	1	60x8 weeks	PCR90	M	VAC50	75	All	0.933	0.005	0.062	0.067	0.029	0.031	0.001	447.893	2.247	29.892	32.139	13.882	14.850	0.378	4479	22.470	298.920	321.390	138.820	148.500	3.780
120	delta	1	60x8 weeks	PCR90	M	VAC50	75	All	0.933	0.005	0.062	0.067	0.029	0.031	0.001	447.893	2.247	29.892	32.139	13.882	14.850	0.378	4479	22.470	298.920	321.390	138.820	148.500	3.780
120	delta	1	60x8 weeks	PCR90	M	VAC50	75	All	0.933	0.005	0.062	0.067	0.029	0.031	0.001	447.893	2.247	29.892	32.139	13.882	14.850	0.378	4479	22.470	298.920	321.390	138.820	148.500	3.780
120	delta	1.2	60x8 weeks	PCR90	M	VAC50	75	All	0.908	0.004	0.083	0.087	0.039	0.041	0.001	436.041	1.952	39.909	41.861	18.534	19.442	0.314	4360	19.520	399.090	418.610	185.340	194.420	3.140
120	delta	1.2	60x8 weeks	PCR90	M	VAC50	75	All	0.908	0.004	0.083	0.087	0.039	0.041	0.001	436.041	1.952	39.909	41.861	18.534	19.442	0.314	4360	19.520	399.090	418.610	185.340	194.420	3.140
120	delta	1.2	60x8 weeks	PCR90	M	VAC50	75	All	0.908	0.004	0.083	0.087	0.039	0.041	0.001	436.041	1.952	39.909	41.861	18.534	19.442	0.314	4360	19.520	399.090	418.610	185.340	194.420	3.140
120	original	0.6	60x8 weeks	PCR90	M	VAC50	100	All	0.997	0.004	0.000	0.004	0.002	0.000	0.001	478.557	2.061	0.038	2.099	1.050	0.086	0.458	4786	20.610	0.380	20.990	10.500	0.860	4.580
120	original	0.6	60x8 weeks	PCR90	M	VAC50	100	All	0.997	0.004	0.000	0.004	0.002	0.000	0.001	478.557	2.061	0.038	2.099	1.050	0.086	0.458	4786	20.610	0.380	20.990	10.500	0.860	4.580
120	original	0.6	60x8 weeks	PCR90	M	VAC50	100	All	0.997	0.004	0.000	0.004	0.002	0.000	0.001	478.557	2.061	0.038	2.099	1.050	0.086	0.458	4786	20.610	0.380	20.990	10.500	0.860	4.580
120	original	1	60x8 weeks	PCR90	M	VAC50	100	All	0.997	0.004	0.000	0.004	0.002	0.000	0.001	478.595	2.032	0.045	2.077	1.040	0.104	0.426	4786	20.320	0.450	20.770	10.400	1.040	4.260

120	original	1	60x8 weeks	PCR90	M	VAC50	100	All	0.997	0.004	0.000	0.004	0.002	0.000	0.001	478.595	2.032	0.045	2.077	1.040	0.104	0.426	4786	20.320	0.450	20.770	10.400	1.040	4.260
120	original	1	60x8 weeks	PCR90	M	VAC50	100	All	0.997	0.004	0.000	0.004	0.002	0.000	0.001	478.595	2.032	0.045	2.077	1.040	0.104	0.426	4786	20.320	0.450	20.770	10.400	1.040	4.260
120	original	1.2	60x8 weeks	PCR90	M	VAC50	100	All	0.997	0.004	0.000	0.004	0.002	0.000	0.001	478.646	2.065	0.046	2.111	1.051	0.089	0.507	4786	20.650	0.460	21.110	10.510	0.890	5.070
120	original	1.2	60x8 weeks	PCR90	M	VAC50	100	All	0.997	0.004	0.000	0.004	0.002	0.000	0.001	478.646	2.065	0.046	2.111	1.051	0.089	0.507	4786	20.650	0.460	21.110	10.510	0.890	5.070
120	original	1.2	60x8 weeks	PCR90	M	VAC50	100	All	0.997	0.004	0.000	0.004	0.002	0.000	0.001	478.646	2.065	0.046	2.111	1.051	0.089	0.507	4786	20.650	0.460	21.110	10.510	0.890	5.070
120	alpha	0.6	60x8 weeks	PCR90	M	VAC50	100	All	0.995	0.004	0.001	0.006	0.002	0.003	0.001	477.482	2.087	0.587	2.674	1.058	1.213	0.469	4775	20.870	5.870	26.740	10.580	12.130	4.690
120	alpha	0.6	60x8 weeks	PCR90	M	VAC50	100	All	0.995	0.004	0.001	0.006	0.002	0.003	0.001	477.482	2.087	0.587	2.674	1.058	1.213	0.469	4775	20.870	5.870	26.740	10.580	12.130	4.690
120	alpha	0.6	60x8 weeks	PCR90	M	VAC50	100	All	0.995	0.004	0.001	0.006	0.002	0.003	0.001	477.482	2.087	0.587	2.674	1.058	1.213	0.469	4775	20.870	5.870	26.740	10.580	12.130	4.690
120	alpha	1	60x8 weeks	PCR90	M	VAC50	100	All	0.994	0.004	0.003	0.007	0.003	0.003	0.001	477.063	2.033	1.222	3.255	1.274	1.407	0.485	4771	20.330	12.220	32.550	12.740	14.070	4.850
120	alpha	1	60x8 weeks	PCR90	M	VAC50	100	All	0.994	0.004	0.003	0.007	0.003	0.003	0.001	477.063	2.033	1.222	3.255	1.274	1.407	0.485	4771	20.330	12.220	32.550	12.740	14.070	4.850
120	alpha	1	60x8 weeks	PCR90	M	VAC50	100	All	0.994	0.004	0.003	0.007	0.003	0.003	0.001	477.063	2.033	1.222	3.255	1.274	1.407	0.485	4771	20.330	12.220	32.550	12.740	14.070	4.850
120	alpha	1.2	60x8 weeks	PCR90	M	VAC50	100	All	0.993	0.004	0.003	0.007	0.003	0.003	0.001	476.650	2.036	1.540	3.576	1.368	1.651	0.460	4767	20.360	15.400	35.760	13.680	16.510	4.600
120	alpha	1.2	60x8 weeks	PCR90	M	VAC50	100	All	0.993	0.004	0.003	0.007	0.003	0.003	0.001	476.650	2.036	1.540	3.576	1.368	1.651	0.460	4767	20.360	15.400	35.760	13.680	16.510	4.600
120	alpha	1.2	60x8 weeks	PCR90	M	VAC50	100	All	0.993	0.004	0.003	0.007	0.003	0.003	0.001	476.650	2.036	1.540	3.576	1.368	1.651	0.460	4767	20.360	15.400	35.760	13.680	16.510	4.600
120	delta	0.6	60x8 weeks	PCR90	M	VAC50	100	All	0.988	0.006	0.008	0.013	0.006	0.006	0.001	474.022	2.695	3.639	6.334	2.702	2.803	0.423	4740	26.950	36.390	63.340	27.020	28.030	4.230
120	delta	0.6	60x8 weeks	PCR90	M	VAC50	100	All	0.988	0.006	0.008	0.013	0.006	0.006	0.001	474.022	2.695	3.639	6.334	2.702	2.803	0.423	4740	26.950	36.390	63.340	27.020	28.030	4.230
120	delta	0.6	60x8 weeks	PCR90	M	VAC50	100	All	0.988	0.006	0.008	0.013	0.006	0.006	0.001	474.022	2.695	3.639	6.334	2.702	2.803	0.423	4740	26.950	36.390	63.340	27.020	28.030	4.230
120	delta	1	60x8 weeks	PCR90	M	VAC50	100	All	0.969	0.005	0.027	0.032	0.014	0.014	0.001	465.304	2.393	12.987	15.380	6.592	6.850	0.433	4653	23.930	129.870	153.800	65.920	68.500	4.330
120	delta	1	60x8 weeks	PCR90	M	VAC50	100	All	0.969	0.005	0.027	0.032	0.014	0.014	0.001	465.304	2.393	12.987	15.380	6.592	6.850	0.433	4653	23.930	129.870	153.800	65.920	68.500	4.330
120	delta	1	60x8 weeks	PCR90	M	VAC50	100	All	0.969	0.005	0.027	0.032	0.014	0.014	0.001	465.304	2.393	12.987	15.380	6.592	6.850	0.433	4653	23.930	129.870	153.800	65.920	68.500	4.330
120	delta	1.2	60x8 weeks	PCR90	M	VAC50	100	All	0.954	0.005	0.043	0.048	0.021	0.022	0.001	457.723	2.301	20.824	23.125	10.001	10.356	0.364	4577	23.010	208.240	231.250	100.010	103.560	3.640
120	delta	1.2	60x8 weeks	PCR90	M	VAC50	100	All	0.954	0.005	0.043	0.048	0.021	0.022	0.001	457.723	2.301	20.824	23.125	10.001	10.356	0.364	4577	23.010	208.240	231.250	100.010	103.560	3.640
120	delta	1.2	60x8 weeks	PCR90	M	VAC50	100	All	0.954	0.005	0.043	0.048	0.021	0.022	0.001	457.723	2.301	20.824	23.125	10.001	10.356	0.364	4577	23.010	208.240	231.250	100.010	103.560	3.640
120	original	0.6	60x8 weeks	Ag80	Daily	NOVAC	0	All	4.939	0.008	0.001	0.009	0.000	0.009	0.096	2370.558	3.841	0.543	4.384	0.105	4.359	46.179	23706	38.410	5.430	43.840	1.050	43.590	461.790
120	original	0.6	60x8 weeks	Ag80	Daily	NOVAC	0	All	4.939	0.008	0.001	0.009	0.000	0.009	0.096	2370.558	3.841	0.543	4.384	0.105	4.359	46.179	23706	38.410	5.430	43.840	1.050	43.590	461.790
120	original	0.6	60x8 weeks	Ag80	Daily	NOVAC	0	All	4.939	0.008	0.001	0.009	0.000	0.009	0.096	2370.558	3.841	0.543	4.384	0.105	4.359	46.179	23706	38.410	5.430	43.840	1.050	43.590	461.790
120	original	1	60x8 weeks	Ag80	Daily	NOVAC	0	All	4.933	0.008	0.002	0.010	0.000	0.010	0.096	2367.811	3.843	0.921	4.764	0.116	4.766	46.123	23678	38.430	9.210	47.640	1.160	47.660	461.230
120	original	1	60x8 weeks	Ag80	Daily	NOVAC	0	All	4.933	0.008	0.002	0.010	0.000	0.010	0.096	2367.811	3.843	0.921	4.764	0.116	4.766	46.123	23678	38.430	9.210	47.640	1.160	47.660	461.230
120	original	1	60x8 weeks	Ag80	Daily	NOVAC	0	All	4.933	0.008	0.002	0.010	0.000	0.010	0.096	2367.811	3.843	0.921	4.764	0.116	4.766	46.123	23678	38.430	9.210	47.640	1.160	47.660	461.230
120	original	1.2	60x8 weeks	Ag80	Daily	NOVAC	0	All	4.926	0.008	0.003	0.011	0.000	0.011	0.096	2364.565	3.893	1.227	5.120	0.140	5.196	46.053	23646	38.930	12.270	51.200	1.400	51.960	460.530
120	original	1.2	60x8 weeks	Ag80	Daily	NOVAC	0	All	4.926	0.008	0.003	0.011	0.000	0.011	0.096	2364.565	3.893	1.227	5.120	0.140	5.196	46.053	23646	38.930	12.270	51.200	1.400	51.960	460.530
120	original	1.2	60x8 weeks	Ag80	Daily	NOVAC	0	All	4.926	0.008	0.003	0.011	0.000	0.011	0.096	2364.565	3.893	1.227	5.120	0.140	5.196	46.053	23646	38.930	12.270	51.200	1.400	51.960	460.530
120	alpha	0.6	60x8 weeks	Ag80	Daily	NOVAC	0	All	4.930	0.008	0.002	0.010	0.000	0.010	0.096	2366.631	3.857	0.923	4.780	0.127	4.929	46.274	23666	38.570	9.230	47.800	1.270	49.290	462.740
120	alpha	0.6	60x8 weeks	Ag80	Daily	NOVAC	0	All	4.930	0.008	0.002	0.010	0.000	0.010	0.096	2366.631	3.857	0.923	4.780	0.127	4.929	46.274	23666	38.570	9.230	47.800	1.270	49.290	462.740
120	alpha	0.6	60x8 weeks	Ag80	Daily	NOVAC	0	All	4.930	0.008	0.002	0.010	0.000	0.010	0.096	2366.631	3.857	0.923	4.780	0.127	4.929	46.274	23666	38.570	9.230	47.800	1.270	49.290	462.740
120	alpha	1	60x8 weeks	Ag80	Daily	NOVAC	0	All	4.919	0.008	0.004	0.011	0.000	0.012	0.095	2360.915	3.735	1.738	5.473	0.111	5.739	45.773	23609	37.350	17.380	54.730	1.110	57.390	457.730
120	alpha	1	60x8 weeks	Ag80	Daily	NOVAC	0	All	4.919	0.008	0.004	0.011	0.000	0.012	0.095	2360.915	3.735	1.738	5.473	0.111	5.739	45.773	23609	37.350	17.380	54.730	1.110	57.390	457.730
120	alpha	1	60x8 weeks	Ag80	Daily	NOVAC	0	All	4.919	0.008	0.004	0.011	0.000	0.012	0.095	2360.915	3.735	1.738	5.473	0.111	5.739	45.773	23609	37.350	17.380	54.730	1.110	57.390	457.730
120	alpha	1.2	60x8 weeks	Ag80	Daily	NOVAC	0	All	4.912	0.008	0.005	0.012	0.000	0.013	0.096	2357.662	3.830	2.169	5.999	0.144	6.235	45.866	23577	38.300	21.690	59.990	1.440	62.350	458.660
120	alpha	1.2	60x8 weeks	Ag80	Daily	NOVAC	0	All	4.912	0.008	0.005	0.012	0.000	0.013	0.096	2357.662	3.830	2.169	5.999	0.144	6.235	45.866	23577	38.300	21.690	59.990	1.440	62.350	458.660
120	alpha	1.2	60x8 weeks	Ag80	Daily	NOVAC	0	All	4.912	0.008	0.005	0.012	0.000	0.013	0.096	2357.662	3.830	2.169	5.999	0.144	6.235	45.866	23577	38.300	21.690	59.990	1.440	62.350	458.660
120	delta	0.6	60x8 weeks	Ag80	Daily	NOVAC	0	All	4.911	0.008	0.004	0.012	0.000	0.013	0.096	2357.440	3.862	2.106	5.968	0.172	6.179	45.883	23574	38.620	21.060	59.680	1.720	61.790	458.830
120	delta	0.6	60x8 weeks	Ag80	Daily	NOVAC	0	All	4.911	0.008	0.004	0.012	0.000	0.013	0.096	2357.440	3.862	2.106	5.968	0.172	6.179	45.883	23574	38.620	21.060	59.680	1.720	61.790	458.830
120	delta	0.6	60x8 weeks	Ag80	Daily	NOVAC	0	All	4.911	0.008	0.004	0.012	0.000	0.013	0.096	2357.440	3.862	2.106	5.968	0.172	6.179	45.883	23574	38.620	21.060	59.680	1.720	61.790	458.830
120	delta	1	60x8 weeks	Ag80	Daily	NOVAC	0	All	4.875	0.008	0.010	0.017	0.001	0.018	0.092	2340.017	3.802	4.579	8.381	0.251	8.742	44.381	23400	38.020	45.790	83.810	2.510	87.420	443.810
120	delta	1	60x8 weeks	Ag80	Daily	NOVAC	0	All	4.875	0.008	0.010	0.017	0.001	0.018	0.092	2340.017	3.802	4.579	8.381	0.251	8.742	44.381	23400	38.020	45.790	83.810	2.510	87.420	443.810
120	delta	1	60x8 weeks	Ag80	Daily	NOVAC	0	All	4.875	0.008	0.010	0.017	0.001	0.018	0.092	2340.017	3.802	4.579	8.381	0.251	8.742	44.381	23400	38.020	45.790	83.810	2.510	87.420	443.810
120	delta	1.2	60x8 weeks	Ag80	Daily	NOVAC	0	All	4.843	0.008	0.014	0.022	0.001	0.023	0.092	2324.812	3.671	6.650	10.321	0.247	10.868	44.069	23248	36.710	66.500	103.210	2.470	108.680	440.690
120	delta	1.2	60x8 weeks	Ag80	Daily	NOVAC	0	All	4.843	0.008	0.014	0.022	0.001	0.023	0.092	2324.812	3.671	6.650	10.321	0.247	10.868	44.069	23248	36.710	66.500	103.210	2.470	108.680	440.690
120	delta	1.2	60x8 weeks	Ag80	Daily	NOVAC	0	All	4.843	0.008	0.014	0.022	0.001	0.023	0.092	2324.812	3.671	6.650	10.321	0.247	10.868	44.069	23248	36.710	66.500	103.210	2.470	108.680	440.690
120	original	0.6	60x8 weeks	Ag80	Daily	VAC95	25	All	4.955	0.006	0.001	0.007	0.000	0.007	0.097	2378.563	2.946	0.290	3.236	0.114	3.159	46.410	23786	29.460	2.900	32.360	1.140	31.590	464.100
120	original	0.6	60x8 weeks	Ag80	Daily	VAC95	25	All	4.955	0.006	0.001	0.007	0.000	0.007	0.097	2378.563	2.946	0.290	3.236	0.114	3.159	46.410	23786	29.460	2.900	32.360	1.140	31.590	464.100
120	original	0.6	60x8 weeks	Ag80	Daily	VAC95	25	All	4.955	0.006	0.001	0.007	0.000	0.007	0.097	2378.563	2.946	0.290	3.236	0.114	3.159	46.410	23786	29.460	2.900	32.360	1.140	31.590	464.100
120	original	1	60x8 weeks	Ag80	Daily	VAC95	25	All	4.952	0.006	0.001	0.007	0.000	0.007	0.097	2377.122	2.931	0.522	3.453	0.082	3.412	46.572	23771	29.310	5.220	34.530	0.820	34.120	465.720
120	original	1	60x8 weeks	Ag80	Daily	VAC95	25	All	4.952	0.006	0.001	0.007	0.000	0.007	0.097	2377.122	2.931	0.522	3.453	0.082	3.412	46.572	23771	29.310	5.220	34.530	0.820	34.120	465.720
120	original	1	60x8 weeks	Ag80	Daily	VAC95	25	All	4.952	0.006	0.001	0.007	0.000	0.007	0.097	2377.122	2.931	0.522	3.453	0.082	3.412	46.572	23771	29.310	5.220	34.530	0.820	34.120	465.720
120	original	1.2	60x8 weeks	Ag80	Daily	VAC95	25	All	4.950	0.006	0.001	0.007	0.000	0.007	0.098	2375.901	2.923	0.665	3.588	0.116	3.523	47.096	23759	29.230	6.650	35.880	1.160	35.230	470.960
120	original	1.2	60x8 weeks	Ag80	Daily	VAC95	25	All	4.950	0.006	0.001	0.007	0.000	0.007	0.098	2375.901	2.923	0.665	3.588	0.116	3.523	47.096	23759	29.230	6.650	35.880	1.160	35.230	470.960
120	original	1.2	60x8 weeks	Ag80	Daily	VAC95	25	All	4.950	0.006	0.001	0.007	0.000	0.007	0.098	2375.901	2.923	0.665	3.588	0.116	3.523	47.096	23759	29.230	6.650	35.880	1.160	35.230	470.960
120	alpha	0.6	60x8 weeks	Ag80	Daily	VAC95	25	All	4.947	0.006	0.001	0.008	0.000	0.008	0.096	2374.425	3.110	0.515	3.625	0.094	3.767	46.033	23744	31.100	5.150	36.250	0.940	37.670	460.330
120	alpha	0.6	60x8 weeks	Ag80	Daily	VAC95	25	All	4.947	0.006	0.001	0.008	0.000	0.008	0.096	2374.425	3.110	0.515	3.625	0.094	3.767	46.033	23744	31.100	5.150	36.250	0.940	37.670	460.330
120	alpha	0.6	60x8 weeks	Ag80	Daily	VAC95	25	All	4.947	0.006	0.001	0.008	0.000	0.008	0.096	2374.425	3.110	0.515	3.625	0.094	3.767	46.033	23744	31.100	5.150	36.250	0.940	37.670	460.330
120	alpha	1	60x8 weeks	Ag80	Daily	VAC95	25	All	4.941	0.006	0.002	0.008	0.000	0.009	0.097	2371.856	3.082	0.961	4.043	0.074	4.164	46.387	23719	30.820	9.610	40.430	0.740	41.640	463.870
120	alpha	1	60x8 weeks	Ag80	Daily	VAC95	25	All	4.941	0.006	0.002	0.008	0.000	0.009	0.097	2371.856	3.082	0.961	4.043	0.074	4.164	46.387	23719	30.820	9.610	40.430	0.740	41.640	463.870
120	alpha	1	60x8 weeks	Ag80	Daily	VAC95	25	All	4.941	0.006	0.002	0.008	0.000	0.009	0.097	2371.856	3.082	0.961	4.043	0.074	4.164	46.387	23719	30.820	9.610	40.430	0.740	41.640	463.870
120	alpha	1.2	60x8 weeks	Ag80	Daily	VAC95	25	All	4.940	0.006	0.002	0.008	0.000	0.009	0.096	2371.308	2.915	1.110	4.025	0.090	4.206	46.239	23713	29.150	11.100	40.250	0.900	42.060	462.390
120	alpha	1.2	60x8 weeks	Ag80	Daily	VAC95	25	All	4.940	0.006	0.002	0.008	0.000	0.009	0.096	2371.308	2.915	1.110	4.025	0.090	4.206	46.239	23713	29.150	11.100	40.250	0.900	42.060	462.390
120	alpha	1.2	60x8 weeks	Ag80	Daily	VAC95	25	All	4.940	0.006	0.002	0.008	0.000	0.009	0.096	2371.308	2.915	1.110	4.025	0.090	4.206	46.239	23713	29.150	11.100	40.250	0.900	42.060	462.390
120	delta	0.6	60x8 weeks	Ag80	Daily	VAC95	25	All	4.935	0.006	0.003	0.009	0.000	0.009	0.097	2368.973	3.091	1.265	4.356	0.119	4.483	46.321	23690	30.910	12.650	43.560	1.190	44.830	463.210
120	delta	0.6	60x8 weeks	Ag80	Daily	VAC95	25	All	4.935	0.006	0.003	0.009	0.000	0.009	0.097	2368.973	3.091	1.265	4.356	0.119	4.483	46.321	23690	30.910	12.650	43.560	1.190	44.830	463.210
120	delta	0.6	60x8 weeks	Ag80	Daily	VAC95	25	All	4.935	0.006	0.003	0.009	0.000	0.009	0.097	2368.973	3.091	1.265	4.356	0.119	4.483	46.321	23690	30.910	12.650	43.560	1.190	44.830	463.210

120	delta	1	60x8 weeks	Ag80	Daily	VAC95	25	All	4.919	0.006	0.005	0.011	0.000	0.012	0.096	2361.272	3.013	2.414	5.427	0.159	5.642	45.884	23613	30.130	24.140	54.270	1.590	56.420	458.840
120	delta	1	60x8 weeks	Ag80	Daily	VAC95	25	All	4.919	0.006	0.005	0.011	0.000	0.012	0.096	2361.272	3.013	2.414	5.427	0.159	5.642	45.884	23613	30.130	24.140	54.270	1.590	56.420	458.840
120	delta	1	60x8 weeks	Ag80	Daily	VAC95	25	All	4.919	0.006	0.005	0.011	0.000	0.012	0.096	2361.272	3.013	2.414	5.427	0.159	5.642	45.884	23613	30.130	24.140	54.270	1.590	56.420	458.840
120	delta	1.2	60x8 weeks	Ag80	Daily	VAC95	25	All	4.908	0.006	0.007	0.013	0.000	0.013	0.095	2355.835	2.892	3.146	6.038	0.153	6.336	45.620	23558	28.920	31.460	60.380	1.530	63.360	456.200
120	delta	1.2	60x8 weeks	Ag80	Daily	VAC95	25	All	4.908	0.006	0.007	0.013	0.000	0.013	0.095	2355.835	2.892	3.146	6.038	0.153	6.336	45.620	23558	28.920	31.460	60.380	1.530	63.360	456.200
120	delta	1.2	60x8 weeks	Ag80	Daily	VAC95	25	All	4.908	0.006	0.007	0.013	0.000	0.013	0.095	2355.835	2.892	3.146	6.038	0.153	6.336	45.620	23558	28.920	31.460	60.380	1.530	63.360	456.200
120	original	0.6	60x8 weeks	Ag80	Daily	VAC95	50	All	4.968	0.005	0.000	0.005	0.000	0.005	0.098	2384.769	2.165	0.161	2.326	0.101	2.195	46.978	23848	21.650	1.610	23.260	1.010	21.950	469.780
120	original	0.6	60x8 weeks	Ag80	Daily	VAC95	50	All	4.968	0.005	0.000	0.005	0.000	0.005	0.098	2384.769	2.165	0.161	2.326	0.101	2.195	46.978	23848	21.650	1.610	23.260	1.010	21.950	469.780
120	original	0.6	60x8 weeks	Ag80	Daily	VAC95	50	All	4.968	0.005	0.000	0.005	0.000	0.005	0.098	2384.769	2.165	0.161	2.326	0.101	2.195	46.978	23848	21.650	1.610	23.260	1.010	21.950	469.780
120	original	1	60x8 weeks	Ag80	Daily	VAC95	50	All	4.969	0.004	0.001	0.005	0.000	0.005	0.098	2384.886	2.073	0.248	2.321	0.094	2.238	47.009	23849	20.730	2.480	23.210	0.940	22.380	470.090
120	original	1	60x8 weeks	Ag80	Daily	VAC95	50	All	4.969	0.004	0.001	0.005	0.000	0.005	0.098	2384.886	2.073	0.248	2.321	0.094	2.238	47.009	23849	20.730	2.480	23.210	0.940	22.380	470.090
120	original	1	60x8 weeks	Ag80	Daily	VAC95	50	All	4.969	0.004	0.001	0.005	0.000	0.005	0.098	2384.886	2.073	0.248	2.321	0.094	2.238	47.009	23849	20.730	2.480	23.210	0.940	22.380	470.090
120	original	1.2	60x8 weeks	Ag80	Daily	VAC95	50	All	4.970	0.004	0.001	0.005	0.000	0.004	0.098	2385.538	1.970	0.244	2.214	0.105	2.093	46.947	23855	19.700	2.440	22.140	1.050	20.930	469.470
120	original	1.2	60x8 weeks	Ag80	Daily	VAC95	50	All	4.970	0.004	0.001	0.005	0.000	0.004	0.098	2385.538	1.970	0.244	2.214	0.105	2.093	46.947	23855	19.700	2.440	22.140	1.050	20.930	469.470
120	original	1.2	60x8 weeks	Ag80	Daily	VAC95	50	All	4.970	0.004	0.001	0.005	0.000	0.004	0.098	2385.538	1.970	0.244	2.214	0.105	2.093	46.947	23855	19.700	2.440	22.140	1.050	20.930	469.470
120	alpha	0.6	60x8 weeks	Ag80	Daily	VAC95	50	All	4.965	0.004	0.000	0.005	0.000	0.005	0.099	2383.423	2.136	0.223	2.359	0.060	2.427	47.436	23834	21.360	2.230	23.590	0.600	24.270	474.360
120	alpha	0.6	60x8 weeks	Ag80	Daily	VAC95	50	All	4.965	0.004	0.000	0.005	0.000	0.005	0.099	2383.423	2.136	0.223	2.359	0.060	2.427	47.436	23834	21.360	2.230	23.590	0.600	24.270	474.360
120	alpha	0.6	60x8 weeks	Ag80	Daily	VAC95	50	All	4.965	0.004	0.000	0.005	0.000	0.005	0.099	2383.423	2.136	0.223	2.359	0.060	2.427	47.436	23834	21.360	2.230	23.590	0.600	24.270	474.360
120	alpha	1	60x8 weeks	Ag80	Daily	VAC95	50	All	4.964	0.004	0.001	0.005	0.000	0.005	0.097	2382.679	2.073	0.430	2.503	0.055	2.560	46.538	23827	20.730	4.300	25.030	0.550	25.600	465.380
120	alpha	1	60x8 weeks	Ag80	Daily	VAC95	50	All	4.964	0.004	0.001	0.005	0.000	0.005	0.097	2382.679	2.073	0.430	2.503	0.055	2.560	46.538	23827	20.730	4.300	25.030	0.550	25.600	465.380
120	alpha	1	60x8 weeks	Ag80	Daily	VAC95	50	All	4.964	0.004	0.001	0.005	0.000	0.005	0.097	2382.679	2.073	0.430	2.503	0.055	2.560	46.538	23827	20.730	4.300	25.030	0.550	25.600	465.380
120	alpha	1.2	60x8 weeks	Ag80	Daily	VAC95	50	All	4.964	0.004	0.001	0.005	0.000	0.005	0.099	2382.525	2.066	0.474	2.540	0.064	2.581	47.573	23825	20.660	4.740	25.400	0.640	25.810	475.730
120	alpha	1.2	60x8 weeks	Ag80	Daily	VAC95	50	All	4.964	0.004	0.001	0.005	0.000	0.005	0.099	2382.525	2.066	0.474	2.540	0.064	2.581	47.573	23825	20.660	4.740	25.400	0.640	25.810	475.730
120	alpha	1.2	60x8 weeks	Ag80	Daily	VAC95	50	All	4.964	0.004	0.001	0.005	0.000	0.005	0.099	2382.525	2.066	0.474	2.540	0.064	2.581	47.573	23825	20.660	4.740	25.400	0.640	25.810	475.730
120	delta	0.6	60x8 weeks	Ag80	Daily	VAC95	50	All	4.959	0.005	0.001	0.006	0.000	0.006	0.098	2380.523	2.197	0.564	2.761	0.092	2.835	47.073	23805	21.970	5.640	27.610	0.920	28.350	470.730
120	delta	0.6	60x8 weeks	Ag80	Daily	VAC95	50	All	4.959	0.005	0.001	0.006	0.000	0.006	0.098	2380.523	2.197	0.564	2.761	0.092	2.835	47.073	23805	21.970	5.640	27.610	0.920	28.350	470.730
120	delta	0.6	60x8 weeks	Ag80	Daily	VAC95	50	All	4.959	0.005	0.001	0.006	0.000	0.006	0.098	2380.523	2.197	0.564	2.761	0.092	2.835	47.073	23805	21.970	5.640	27.610	0.920	28.350	470.730
120	delta	1	60x8 weeks	Ag80	Daily	VAC95	50	All	4.952	0.005	0.002	0.007	0.000	0.007	0.097	2376.842	2.224	1.040	3.264	0.096	3.369	46.740	23768	22.240	10.400	32.640	0.960	33.690	467.400
120	delta	1	60x8 weeks	Ag80	Daily	VAC95	50	All	4.952	0.005	0.002	0.007	0.000	0.007	0.097	2376.842	2.224	1.040	3.264	0.096	3.369	46.740	23768	22.240	10.400	32.640	0.960	33.690	467.400
120	delta	1	60x8 weeks	Ag80	Daily	VAC95	50	All	4.952	0.005	0.002	0.007	0.000	0.007	0.097	2376.842	2.224	1.040	3.264	0.096	3.369	46.740	23768	22.240	10.400	32.640	0.960	33.690	467.400
120	delta	1.2	60x8 weeks	Ag80	Daily	VAC95	50	All	4.947	0.005	0.003	0.007	0.000	0.008	0.098	2374.751	2.172	1.367	3.539	0.105	3.680	46.933	23748	21.720	13.670	35.390	1.050	36.800	469.330
120	delta	1.2	60x8 weeks	Ag80	Daily	VAC95	50	All	4.947	0.005	0.003	0.007	0.000	0.008	0.098	2374.751	2.172	1.367	3.539	0.105	3.680	46.933	23748	21.720	13.670	35.390	1.050	36.800	469.330
120	delta	1.2	60x8 weeks	Ag80	Daily	VAC95	50	All	4.947	0.005	0.003	0.007	0.000	0.008	0.098	2374.751	2.172	1.367	3.539	0.105	3.680	46.933	23748	21.720	13.670	35.390	1.050	36.800	469.330
120	original	0.6	60x8 weeks	Ag80	Daily	VAC95	75	All	4.984	0.003	0.000	0.003	0.000	0.002	0.098	2392.382	1.244	0.033	1.277	0.097	1.059	47.189	23924	12.440	0.330	12.770	0.970	10.590	471.890
120	original	0.6	60x8 weeks	Ag80	Daily	VAC95	75	All	4.984	0.003	0.000	0.003	0.000	0.002	0.098	2392.382	1.244	0.033	1.277	0.097	1.059	47.189	23924	12.440	0.330	12.770	0.970	10.590	471.890
120	original	0.6	60x8 weeks	Ag80	Daily	VAC95	75	All	4.984	0.003	0.000	0.003	0.000	0.002	0.098	2392.382	1.244	0.033	1.277	0.097	1.059	47.189	23924	12.440	0.330	12.770	0.970	10.590	471.890
120	original	1	60x8 weeks	Ag80	Daily	VAC95	75	All	4.985	0.002	0.000	0.003	0.000	0.002	0.098	2392.565	1.156	0.078	1.234	0.091	1.047	47.175	23926	11.560	0.780	12.340	0.910	10.470	471.750
120	original	1	60x8 weeks	Ag80	Daily	VAC95	75	All	4.985	0.002	0.000	0.003	0.000	0.002	0.098	2392.565	1.156	0.078	1.234	0.091	1.047	47.175	23926	11.560	0.780	12.340	0.910	10.470	471.750
120	original	1	60x8 weeks	Ag80	Daily	VAC95	75	All	4.985	0.002	0.000	0.003	0.000	0.002	0.098	2392.565	1.156	0.078	1.234	0.091	1.047	47.175	23926	11.560	0.780	12.340	0.910	10.470	471.750
120	original	1.2	60x8 weeks	Ag80	Daily	VAC95	75	All	4.984	0.002	0.000	0.003	0.000	0.002	0.099	2392.405	1.159	0.090	1.249	0.081	1.095	47.320	23924	11.590	0.900	12.490	0.810	10.950	473.200
120	original	1.2	60x8 weeks	Ag80	Daily	VAC95	75	All	4.984	0.002	0.000	0.003	0.000	0.002	0.099	2392.405	1.159	0.090	1.249	0.081	1.095	47.320	23924	11.590	0.900	12.490	0.810	10.950	473.200
120	original	1.2	60x8 weeks	Ag80	Daily	VAC95	75	All	4.984	0.002	0.000	0.003	0.000	0.002	0.099	2392.405	1.159	0.090	1.249	0.081	1.095	47.320	23924	11.590	0.900	12.490	0.810	10.950	473.200
120	alpha	0.6	60x8 weeks	Ag80	Daily	VAC95	75	All	4.980	0.003	0.000	0.003	0.000	0.003	0.099	2390.557	1.295	0.074	1.369	0.046	1.387	47.460	23906	12.950	0.740	13.690	0.460	13.870	474.600
120	alpha	0.6	60x8 weeks	Ag80	Daily	VAC95	75	All	4.980	0.003	0.000	0.003	0.000	0.003	0.099	2390.557	1.295	0.074	1.369	0.046	1.387	47.460	23906	12.950	0.740	13.690	0.460	13.870	474.600
120	alpha	0.6	60x8 weeks	Ag80	Daily	VAC95	75	All	4.980	0.003	0.000	0.003	0.000	0.003	0.099	2390.557	1.295	0.074	1.369	0.046	1.387	47.460	23906	12.950	0.740	13.690	0.460	13.870	474.600
120	alpha	1	60x8 weeks	Ag80	Daily	VAC95	75	All	4.981	0.002	0.000	0.003	0.000	0.003	0.099	2390.878	1.188	0.108	1.296	0.022	1.345	47.544	23909	11.880	1.080	12.960	0.220	13.450	475.440
120	alpha	1	60x8 weeks	Ag80	Daily	VAC95	75	All	4.981	0.002	0.000	0.003	0.000	0.003	0.099	2390.878	1.188	0.108	1.296	0.022	1.345	47.544	23909	11.880	1.080	12.960	0.220	13.450	475.440
120	alpha	1	60x8 weeks	Ag80	Daily	VAC95	75	All	4.981	0.002	0.000	0.003	0.000	0.003	0.099	2390.878	1.188	0.108	1.296	0.022	1.345	47.544	23909	11.880	1.080	12.960	0.220	13.450	475.440
120	alpha	1.2	60x8 weeks	Ag80	Daily	VAC95	75	All	4.979	0.003	0.000	0.003	0.000	0.003	0.098	2390.146	1.238	0.164	1.402	0.033	1.473	47.120	23901	12.380	1.640	14.020	0.330	14.730	471.200
120	alpha	1.2	60x8 weeks	Ag80	Daily	VAC95	75	All	4.979	0.003	0.000	0.003	0.000	0.003	0.098	2390.146	1.238	0.164	1.402	0.033	1.473	47.120	23901	12.380	1.640	14.020	0.330	14.730	471.200
120	alpha	1.2	60x8 weeks	Ag80	Daily	VAC95	75	All	4.979	0.003	0.000	0.003	0.000	0.003	0.098	2390.146	1.238	0.164	1.402	0.033	1.473	47.120	23901	12.380	1.640	14.020	0.330	14.730	471.200
120	delta	0.6	60x8 weeks	Ag80	Daily	VAC95	75	All	4.975	0.003	0.000	0.004	0.000	0.004	0.099	2388.125	1.491	0.210	1.701	0.051	1.736	47.597	23881	14.910	2.100	17.010	0.510	17.360	475.970
120	delta	0.6	60x8 weeks	Ag80	Daily	VAC95	75	All	4.975	0.003	0.000	0.004	0.000	0.004	0.099	2388.125	1.491	0.210	1.701	0.051	1.736	47.597	23881	14.910	2.100	17.010	0.510	17.360	475.970
120	delta	0.6	60x8 weeks	Ag80	Daily	VAC95	75	All	4.975	0.003	0.000	0.004	0.000	0.004	0.099	2388.125	1.491	0.210	1.701	0.051	1.736	47.597	23881	14.910	2.100	17.010	0.510	17.360	475.970
120	delta	1	60x8 weeks	Ag80	Daily	VAC95	75	All	4.974	0.003	0.001	0.004	0.000	0.004	0.100	2387.428	1.417	0.346	1.763	0.068	1.805	47.845	23874	14.170	3.460	17.630	0.680	18.050	478.450
120	delta	1	60x8 weeks	Ag80	Daily	VAC95	75	All	4.974	0.003	0.001	0.004	0.000	0.004	0.100	2387.428	1.417	0.346	1.763	0.068	1.805	47.845	23874	14.170	3.460	17.630	0.680	18.050	478.450
120	delta	1	60x8 weeks	Ag80	Daily	VAC95	75	All	4.974	0.003	0.001	0.004	0.000	0.004	0.100	2387.428	1.417	0.346	1.763	0.068	1.805	47.845	23874	14.170	3.460	17.630	0.680	18.050	478.450
120	delta	1.2	60x8 weeks	Ag80	Daily	VAC95	75	All	4.973	0.003	0.001	0.004	0.000	0.004	0.099	2387.131	1.429	0.400	1.829	0.057	1.878	47.510	23871	14.290	4.000	18.290	0.570	18.780	475.100
120	delta	1.2	60x8 weeks	Ag80	Daily	VAC95	75	All	4.973	0.003	0.001	0.004	0.000	0.004	0.099	2387.131	1.429	0.400	1.829	0.057	1.878	47.510	23871	14.290	4.000	18.290	0.570	18.780	475.100
120	delta	1.2	60x8 weeks	Ag80	Daily	VAC95	75	All	4.973	0.003	0.001	0.004	0.000	0.004	0.099	2387.131	1.429	0.400	1.829	0.057	1.878	47.510	23871	14.290	4.000	18.290	0.570	18.780	475.100
120	original	0.6	60x8 weeks	Ag80	Daily	VAC95	100	All	4.998	0.001	0.000	0.001	0.000	0.000	0.099	2399.149	0.280	0.000	0.280	0.086	0.042	47.580	23991	2.800	0.000	2.800	0.860	0.420	475.800
120	original	0.6	60x8 weeks	Ag80	Daily	VAC95	100	All	4.998	0.001	0.000	0.001	0.000	0.000	0.099	2399.149	0.280	0.000	0.280	0.086	0.042	47.580	23991	2.800	0.000	2.800	0.860	0.420	475.800
120	original	0.6	60x8 weeks	Ag80	Daily	VAC95	100	All	4.998	0.001	0.000	0.001	0.000	0.000	0.099	2399.149	0.280	0.000	0.280	0.086	0.042	47.580	23991	2.800	0.000	2.800	0.860	0.420	475.800
120	original	1	60x8 weeks	Ag80	Daily	VAC95	100	All	4.998	0.001	0.000	0.001	0.000	0.000	0.099	2399.119	0.276	0.000	0.276	0.086	0.054	47.696	23991	2.760	0.000	2.760	0.860	0.540	476.960
120	original	1	60x8 weeks	Ag80	Daily	VAC95	100	All	4.998	0.001	0.000	0.001	0.000	0.000	0.099	2399.119	0.276	0.000	0.276	0.086	0.054	47.696	23991	2.760	0.000	2.760	0.860	0.540	476.960
120	original	1	60x8 weeks	Ag80	Daily	VAC95	100	All	4.998	0.001	0.000	0.001	0.000	0.000	0.099	2399.119	0.276	0.000	0.276	0.086	0.054	47.696	23991	2.760	0.000	2.760	0.860	0.540	476.960
120	original	1.2	60x8 weeks	Ag80	Daily	VAC95	100	All	4.998	0.001	0.000	0.001	0.000	0.000	0.099	2399.052	0.293	0.000	0.293	0.098	0.057	47.713	23991	2.930	0.000	2.930	0.980	0.570	477.130
120	original	1.2	60x8 weeks	Ag80	Daily	VAC95	100	All	4.998	0.001	0.000	0.001	0.000	0.000	0.099	2399.052	0.293	0.000	0.293	0.098	0.057	47.713	23991	2.930	0.000	2.930	0.980	0.570	477.130
120	original	1.2	60x8 weeks	Ag80	Daily	VAC95	100	All	4.998	0.001	0.000	0.001	0.000	0.000	0.099	2399.052	0.293	0.000	0.293	0.098	0.057	47.713	23991	2.930	0.000	2.930	0.980	0.570	477.130
120	alpha	0.6	60x8 weeks	Ag80	Daily	VAC95	100	All	4.995	0.001	0.000	0.001	0.000	0.001	0.099	2397.759	0.344	0.003	0.347	0.009	0.334	47.558	23978	3.440	0.030	3.470	0.090	3.340	475.580
120	alpha	0.6	60x8 weeks	Ag80	Daily	VAC95	100	All	4.995	0.001	0.000	0.001	0.000	0.001	0.099	2397.759	0.344	0.003	0.347	0.009	0.334	47.558	23978	3.440	0.030	3.470	0.090	3.340	475.580

120	alpha	0.6	60x8 weeks	Ag80	Daily	VAC95	100	All	4.995	0.001	0.000	0.001	0.000	0.001	0.099	2397.759	0.344	0.003	0.347	0.009	0.334	47.558	23978	3.440	0.030	3.470	0.090	3.340	475.580
120	alpha	1	60x8 weeks	Ag80	Daily	VAC95	100	All	4.995	0.001	0.000	0.001	0.000	0.001	0.100	2397.526	0.366	0.006	0.372	0.011	0.365	47.764	23975	3.660	0.060	3.720	0.110	3.650	477.640
120	alpha	1	60x8 weeks	Ag80	Daily	VAC95	100	All	4.995	0.001	0.000	0.001	0.000	0.001	0.100	2397.526	0.366	0.006	0.372	0.011	0.365	47.764	23975	3.660	0.060	3.720	0.110	3.650	477.640
120	alpha	1	60x8 weeks	Ag80	Daily	VAC95	100	All	4.995	0.001	0.000	0.001	0.000	0.001	0.100	2397.526	0.366	0.006	0.372	0.011	0.365	47.764	23975	3.660	0.060	3.720	0.110	3.650	477.640
120	alpha	1.2	60x8 weeks	Ag80	Daily	VAC95	100	All	4.994	0.001	0.000	0.001	0.000	0.001	0.100	2397.336	0.401	0.008	0.409	0.009	0.399	47.992	23973	4.010	0.080	4.090	0.090	3.990	479.920
120	alpha	1.2	60x8 weeks	Ag80	Daily	VAC95	100	All	4.994	0.001	0.000	0.001	0.000	0.001	0.100	2397.336	0.401	0.008	0.409	0.009	0.399	47.992	23973	4.010	0.080	4.090	0.090	3.990	479.920
120	alpha	1.2	60x8 weeks	Ag80	Daily	VAC95	100	All	4.994	0.001	0.000	0.001	0.000	0.001	0.100	2397.336	0.401	0.008	0.409	0.009	0.399	47.992	23973	4.010	0.080	4.090	0.090	3.990	479.920
120	delta	0.6	60x8 weeks	Ag80	Daily	VAC95	100	All	4.992	0.001	0.000	0.001	0.000	0.001	0.099	2396.085	0.564	0.016	0.580	0.021	0.575	47.696	23961	5.640	0.160	5.800	0.210	5.750	476.960
120	delta	0.6	60x8 weeks	Ag80	Daily	VAC95	100	All	4.992	0.001	0.000	0.001	0.000	0.001	0.099	2396.085	0.564	0.016	0.580	0.021	0.575	47.696	23961	5.640	0.160	5.800	0.210	5.750	476.960
120	delta	0.6	60x8 weeks	Ag80	Daily	VAC95	100	All	4.992	0.001	0.000	0.001	0.000	0.001	0.099	2396.085	0.564	0.016	0.580	0.021	0.575	47.696	23961	5.640	0.160	5.800	0.210	5.750	476.960
120	delta	1	60x8 weeks	Ag80	Daily	VAC95	100	All	4.991	0.001	0.000	0.001	0.000	0.001	0.099	2395.823	0.580	0.032	0.612	0.019	0.612	47.730	23958	5.800	0.320	6.120	0.190	6.120	477.300
120	delta	1	60x8 weeks	Ag80	Daily	VAC95	100	All	4.991	0.001	0.000	0.001	0.000	0.001	0.099	2395.823	0.580	0.032	0.612	0.019	0.612	47.730	23958	5.800	0.320	6.120	0.190	6.120	477.300
120	delta	1	60x8 weeks	Ag80	Daily	VAC95	100	All	4.991	0.001	0.000	0.001	0.000	0.001	0.099	2395.823	0.580	0.032	0.612	0.019	0.612	47.730	23958	5.800	0.320	6.120	0.190	6.120	477.300
120	delta	1.2	60x8 weeks	Ag80	Daily	VAC95	100	All	4.991	0.001	0.000	0.001	0.000	0.001	0.100	2395.618	0.590	0.046	0.636	0.015	0.640	47.870	23956	5.900	0.460	6.360	0.150	6.400	478.700
120	delta	1.2	60x8 weeks	Ag80	Daily	VAC95	100	All	4.991	0.001	0.000	0.001	0.000	0.001	0.100	2395.618	0.590	0.046	0.636	0.015	0.640	47.870	23956	5.900	0.460	6.360	0.150	6.400	478.700
120	delta	1.2	60x8 weeks	Ag80	Daily	VAC95	100	All	4.991	0.001	0.000	0.001	0.000	0.001	0.100	2395.618	0.590	0.046	0.636	0.015	0.640	47.870	23956	5.900	0.460	6.360	0.150	6.400	478.700
120	original	0.6	60x8 weeks	Ag80	Daily	VAC70	25	All	4.950	0.007	0.001	0.008	0.000	0.007	0.097	2376.105	3.300	0.344	3.644	0.232	3.343	46.554	23761	33.000	3.440	36.440	2.320	33.430	465.540
120	original	0.6	60x8 weeks	Ag80	Daily	VAC70	25	All	4.950	0.007	0.001	0.008	0.000	0.007	0.097	2376.105	3.300	0.344	3.644	0.232	3.343	46.554	23761	33.000	3.440	36.440	2.320	33.430	465.540
120	original	0.6	60x8 weeks	Ag80	Daily	VAC70	25	All	4.950	0.007	0.001	0.008	0.000	0.007	0.097	2376.105	3.300	0.344	3.644	0.232	3.343	46.554	23761	33.000	3.440	36.440	2.320	33.430	465.540
120	original	1	60x8 weeks	Ag80	Daily	VAC70	25	All	4.949	0.007	0.001	0.008	0.000	0.007	0.097	2375.493	3.171	0.561	3.732	0.233	3.447	46.743	23755	31.710	5.610	37.320	2.330	34.470	467.430
120	original	1	60x8 weeks	Ag80	Daily	VAC70	25	All	4.949	0.007	0.001	0.008	0.000	0.007	0.097	2375.493	3.171	0.561	3.732	0.233	3.447	46.743	23755	31.710	5.610	37.320	2.330	34.470	467.430
120	original	1	60x8 weeks	Ag80	Daily	VAC70	25	All	4.949	0.007	0.001	0.008	0.000	0.007	0.097	2375.493	3.171	0.561	3.732	0.233	3.447	46.743	23755	31.710	5.610	37.320	2.330	34.470	467.430
120	original	1.2	60x8 weeks	Ag80	Daily	VAC70	25	All	4.948	0.007	0.001	0.008	0.000	0.007	0.097	2375.178	3.157	0.700	3.857	0.236	3.496	46.386	23752	31.570	7.000	38.570	2.360	34.960	463.860
120	original	1.2	60x8 weeks	Ag80	Daily	VAC70	25	All	4.948	0.007	0.001	0.008	0.000	0.007	0.097	2375.178	3.157	0.700	3.857	0.236	3.496	46.386	23752	31.570	7.000	38.570	2.360	34.960	463.860
120	original	1.2	60x8 weeks	Ag80	Daily	VAC70	25	All	4.948	0.007	0.001	0.008	0.000	0.007	0.097	2375.178	3.157	0.700	3.857	0.236	3.496	46.386	23752	31.570	7.000	38.570	2.360	34.960	463.860
120	alpha	0.6	60x8 weeks	Ag80	Daily	VAC70	25	All	4.944	0.007	0.001	0.008	0.000	0.008	0.097	2373.172	3.269	0.548	3.817	0.104	3.936	46.347	23732	32.690	5.480	38.170	1.040	39.360	463.470
120	alpha	0.6	60x8 weeks	Ag80	Daily	VAC70	25	All	4.944	0.007	0.001	0.008	0.000	0.008	0.097	2373.172	3.269	0.548	3.817	0.104	3.936	46.347	23732	32.690	5.480	38.170	1.040	39.360	463.470
120	alpha	0.6	60x8 weeks	Ag80	Daily	VAC70	25	All	4.944	0.007	0.001	0.008	0.000	0.008	0.097	2373.172	3.269	0.548	3.817	0.104	3.936	46.347	23732	32.690	5.480	38.170	1.040	39.360	463.470
120	alpha	1	60x8 weeks	Ag80	Daily	VAC70	25	All	4.939	0.007	0.002	0.009	0.000	0.009	0.097	2370.643	3.145	1.044	4.189	0.118	4.320	46.510	23706	31.450	10.440	41.890	1.180	43.200	465.100
120	alpha	1	60x8 weeks	Ag80	Daily	VAC70	25	All	4.939	0.007	0.002	0.009	0.000	0.009	0.097	2370.643	3.145	1.044	4.189	0.118	4.320	46.510	23706	31.450	10.440	41.890	1.180	43.200	465.100
120	alpha	1	60x8 weeks	Ag80	Daily	VAC70	25	All	4.939	0.007	0.002	0.009	0.000	0.009	0.097	2370.643	3.145	1.044	4.189	0.118	4.320	46.510	23706	31.450	10.440	41.890	1.180	43.200	465.100
120	alpha	1.2	60x8 weeks	Ag80	Daily	VAC70	25	All	4.932	0.007	0.003	0.010	0.000	0.010	0.096	2367.566	3.207	1.377	4.584	0.118	4.736	46.202	23676	32.070	13.770	45.840	1.180	47.360	462.020
120	alpha	1.2	60x8 weeks	Ag80	Daily	VAC70	25	All	4.932	0.007	0.003	0.010	0.000	0.010	0.096	2367.566	3.207	1.377	4.584	0.118	4.736	46.202	23676	32.070	13.770	45.840	1.180	47.360	462.020
120	alpha	1.2	60x8 weeks	Ag80	Daily	VAC70	25	All	4.932	0.007	0.003	0.010	0.000	0.010	0.096	2367.566	3.207	1.377	4.584	0.118	4.736	46.202	23676	32.070	13.770	45.840	1.180	47.360	462.020
120	delta	0.6	60x8 weeks	Ag80	Daily	VAC70	25	All	4.928	0.007	0.003	0.010	0.000	0.010	0.096	2365.430	3.373	1.524	4.897	0.147	5.039	46.212	23654	33.730	15.240	48.970	1.470	50.390	462.120
120	delta	0.6	60x8 weeks	Ag80	Daily	VAC70	25	All	4.928	0.007	0.003	0.010	0.000	0.010	0.096	2365.430	3.373	1.524	4.897	0.147	5.039	46.212	23654	33.730	15.240	48.970	1.470	50.390	462.120
120	delta	0.6	60x8 weeks	Ag80	Daily	VAC70	25	All	4.928	0.007	0.003	0.010	0.000	0.010	0.096	2365.430	3.373	1.524	4.897	0.147	5.039	46.212	23654	33.730	15.240	48.970	1.470	50.390	462.120
120	delta	1	60x8 weeks	Ag80	Daily	VAC70	25	All	4.908	0.007	0.006	0.013	0.000	0.013	0.094	2356.002	3.221	2.872	6.093	0.164	6.329	45.108	23560	32.210	28.720	60.930	1.640	63.290	451.080
120	delta	1	60x8 weeks	Ag80	Daily	VAC70	25	All	4.908	0.007	0.006	0.013	0.000	0.013	0.094	2356.002	3.221	2.872	6.093	0.164	6.329	45.108	23560	32.210	28.720	60.930	1.640	63.290	451.080
120	delta	1	60x8 weeks	Ag80	Daily	VAC70	25	All	4.908	0.007	0.006	0.013	0.000	0.013	0.094	2356.002	3.221	2.872	6.093	0.164	6.329	45.108	23560	32.210	28.720	60.930	1.640	63.290	451.080
120	delta	1.2	60x8 weeks	Ag80	Daily	VAC70	25	All	4.898	0.007	0.008	0.014	0.000	0.015	0.095	2350.974	3.226	3.603	6.829	0.187	7.114	45.452	23510	32.260	36.030	68.290	1.870	71.140	454.520
120	delta	1.2	60x8 weeks	Ag80	Daily	VAC70	25	All	4.898	0.007	0.008	0.014	0.000	0.015	0.095	2350.974	3.226	3.603	6.829	0.187	7.114	45.452	23510	32.260	36.030	68.290	1.870	71.140	454.520
120	delta	1.2	60x8 weeks	Ag80	Daily	VAC70	25	All	4.898	0.007	0.008	0.014	0.000	0.015	0.095	2350.974	3.226	3.603	6.829	0.187	7.114	45.452	23510	32.260	36.030	68.290	1.870	71.140	454.520
120	original	0.6	60x8 weeks	Ag80	Daily	VAC70	50	All	4.965	0.005	0.000	0.006	0.001	0.005	0.097	2383.354	2.558	0.196	2.754	0.330	2.198	46.615	23834	25.580	1.960	27.540	3.300	21.980	466.150
120	original	0.6	60x8 weeks	Ag80	Daily	VAC70	50	All	4.965	0.005	0.000	0.006	0.001	0.005	0.097	2383.354	2.558	0.196	2.754	0.330	2.198	46.615	23834	25.580	1.960	27.540	3.300	21.980	466.150
120	original	0.6	60x8 weeks	Ag80	Daily	VAC70	50	All	4.965	0.005	0.000	0.006	0.001	0.005	0.097	2383.354	2.558	0.196	2.754	0.330	2.198	46.615	23834	25.580	1.960	27.540	3.300	21.980	466.150
120	original	1	60x8 weeks	Ag80	Daily	VAC70	50	All	4.964	0.005	0.001	0.006	0.001	0.005	0.098	2382.749	2.597	0.315	2.912	0.320	2.294	46.817	23827	25.970	3.150	29.120	3.200	22.940	468.170
120	original	1	60x8 weeks	Ag80	Daily	VAC70	50	All	4.964	0.005	0.001	0.006	0.001	0.005	0.098	2382.749	2.597	0.315	2.912	0.320	2.294	46.817	23827	25.970	3.150	29.120	3.200	22.940	468.170
120	original	1	60x8 weeks	Ag80	Daily	VAC70	50	All	4.964	0.005	0.001	0.006	0.001	0.005	0.098	2382.749	2.597	0.315	2.912	0.320	2.294	46.817	23827	25.970	3.150	29.120	3.200	22.940	468.170
120	original	1.2	60x8 weeks	Ag80	Daily	VAC70	50	All	4.962	0.005	0.001	0.006	0.001	0.005	0.098	2381.930	2.597	0.394	2.991	0.350	2.407	47.043	23819	25.970	3.940	29.910	3.500	24.070	470.430
120	original	1.2	60x8 weeks	Ag80	Daily	VAC70	50	All	4.962	0.005	0.001	0.006	0.001	0.005	0.098	2381.930	2.597	0.394	2.991	0.350	2.407	47.043	23819	25.970	3.940	29.910	3.500	24.070	470.430
120	original	1.2	60x8 weeks	Ag80	Daily	VAC70	50	All	4.962	0.005	0.001	0.006	0.001	0.005	0.098	2381.930	2.597	0.394	2.991	0.350	2.407	47.043	23819	25.970	3.940	29.910	3.500	24.070	470.430
120	alpha	0.6	60x8 weeks	Ag80	Daily	VAC70	50	All	4.957	0.006	0.001	0.006	0.000	0.006	0.098	2379.256	2.662	0.326	2.988	0.083	3.081	46.804	23793	26.620	3.260	29.880	0.830	30.810	468.040
120	alpha	0.6	60x8 weeks	Ag80	Daily	VAC70	50	All	4.957	0.006	0.001	0.006	0.000	0.006	0.098	2379.256	2.662	0.326	2.988	0.083	3.081	46.804	23793	26.620	3.260	29.880	0.830	30.810	468.040
120	alpha	0.6	60x8 weeks	Ag80	Daily	VAC70	50	All	4.957	0.006	0.001	0.006	0.000	0.006	0.098	2379.256	2.662	0.326	2.988	0.083	3.081	46.804	23793	26.620	3.260	29.880	0.830	30.810	468.040
120	alpha	1	60x8 weeks	Ag80	Daily	VAC70	50	All	4.953	0.005	0.001	0.007	0.000	0.007	0.097	2377.429	2.555	0.650	3.205	0.069	3.324	46.715	23774	25.550	6.500	32.050	0.690	33.240	467.150
120	alpha	1	60x8 weeks	Ag80	Daily	VAC70	50	All	4.953	0.005	0.001	0.007	0.000	0.007	0.097	2377.429	2.555	0.650	3.205	0.069	3.324	46.715	23774	25.550	6.500	32.050	0.690	33.240	467.150
120	alpha	1	60x8 weeks	Ag80	Daily	VAC70	50	All	4.953	0.005	0.001	0.007	0.000	0.007	0.097	2377.429	2.555	0.650	3.205	0.069	3.324	46.715	23774	25.550	6.500	32.050	0.690	33.240	467.150
120	alpha	1.2	60x8 weeks	Ag80	Daily	VAC70	50	All	4.951	0.005	0.002	0.007	0.000	0.007	0.098	2376.286	2.612	0.768	3.380	0.086	3.463	46.808	23763	26.120	7.680	33.800	0.860	34.630	468.080
120	alpha	1.2	60x8 weeks	Ag80	Daily	VAC70	50	All	4.951	0.005	0.002	0.007	0.000	0.007	0.098	2376.286	2.612	0.768	3.380	0.086	3.463	46.808	23763	26.120	7.680	33.800	0.860	34.630	468.080
120	alpha	1.2	60x8 weeks	Ag80	Daily	VAC70	50	All	4.951	0.005	0.002	0.007	0.000	0.007	0.098	2376.286	2.612	0.768	3.380	0.086	3.463	46.808	23763	26.120	7.680	33.800	0.860	34.630	468.080
120	delta	0.6	60x8 weeks	Ag80	Daily	VAC70	50	All	4.945	0.006	0.002	0.008	0.000	0.008	0.097	2373.686	2.923	0.824	3.747	0.119	3.831	46.402	23737	29.230	8.240	37.470	1.190	38.310	464.020
120	delta	0.6	60x8 weeks	Ag80	Daily	VAC70	50	All	4.945	0.006	0.002	0.008	0.000	0.008	0.097	2373.686	2.923	0.824	3.747	0.119	3.831	46.402	23737	29.230	8.240	37.470	1.190	38.310	464.020
120	delta	0.6	60x8 weeks	Ag80	Daily	VAC70	50	All	4.945	0.006	0.002	0.008	0.000	0.008	0.097	2373.686	2.923	0.824	3.747	0.119	3.831	46.402	23737	29.230	8.240	37.470	1.190	38.310	464.020
120	delta	1	60x8 weeks	Ag80	Daily	VAC70	50	All	4.938	0.006	0.003	0.009	0.000	0.009	0.096	2370.185	2.722	1.495	4.217	0.126	4.358	46.225	23702	27.220	14.950	42.170	1.260	43.580	462.250
120	delta	1	60x8 weeks	Ag80	Daily	VAC70	50	All	4.938	0.006	0.003	0.009	0.000	0.009	0.096	2370.185	2.722	1.495	4.217	0.126	4.358	46.225	23702	27.220	14.950	42.170	1.260	43.580	462.250
120	delta	1	60x8 weeks	Ag80	Daily	VAC70	50	All	4.938	0.006	0.003	0.009	0.000	0.009	0.096	2370.185	2.722	1.495	4.217	0.126	4.358	46.225	23702	27.220	14.950	42.170	1.260	43.580	462.250
120	delta	1.2	60x8 weeks	Ag80	Daily	VAC70	50	All	4.929	0.006	0.004	0.010	0.000	0.010	0.097	2366.136	2.692	2.013	4.705	0.156	4.872	46.413	23661	26.920	20.130	47.050	1.560	48.720	464.130
120	delta	1.2	60x8 weeks	Ag80	Daily	VAC70	50	All	4.929	0.006	0.004	0.010	0.000	0.010	0.097	2366.136	2.692	2.013	4.705	0.156	4.872	46.413	23661	26.920	20.130	47.050	1.560	48.720	464.130
120	delta	1.2	60x8 weeks	Ag80	Daily	VAC70	50	All	4.929	0.006	0.004	0.010	0.000	0.010	0.097	2366.136	2.692	2.013	4.705	0.156	4.872	46.413	23661	26.920	20.130	47.050	1.560	48.720	464.130
120	original	0.6	60x8 weeks	Ag80	Daily	VAC70	75	All	4.978	0.004	0.000	0.004	0.001	0.002	0.099	2389.656	1.973	0.064	2.037	0.415	1.174	47.552	23897	19.730	0.640	20.370	4.150	11.740	475.520

120	original	0.6	60x8 weeks	Ag80	Daily	VAC70	75	All	4.978	0.004	0.000	0.004	0.001	0.002	0.099	2389.656	1.973	0.064	2.037	0.415	1.174	47.552	23897	19.730	0.640	20.370	4.150	11.740	475.520
120	original	0.6	60x8 weeks	Ag80	Daily	VAC70	75	All	4.978	0.004	0.000	0.004	0.001	0.002	0.099	2389.656	1.973	0.064	2.037	0.415	1.174	47.552	23897	19.730	0.640	20.370	4.150	11.740	475.520
120	original	1	60x8 weeks	Ag80	Daily	VAC70	75	All	4.977	0.004	0.000	0.004	0.001	0.003	0.097	2389.055	1.977	0.125	2.102	0.427	1.235	46.669	23891	19.770	1.250	21.020	4.270	12.350	466.690
120	original	1	60x8 weeks	Ag80	Daily	VAC70	75	All	4.977	0.004	0.000	0.004	0.001	0.003	0.097	2389.055	1.977	0.125	2.102	0.427	1.235	46.669	23891	19.770	1.250	21.020	4.270	12.350	466.690
120	original	1	60x8 weeks	Ag80	Daily	VAC70	75	All	4.977	0.004	0.000	0.004	0.001	0.003	0.097	2389.055	1.977	0.125	2.102	0.427	1.235	46.669	23891	19.770	1.250	21.020	4.270	12.350	466.690
120	original	1.2	60x8 weeks	Ag80	Daily	VAC70	75	All	4.977	0.004	0.000	0.004	0.001	0.003	0.098	2389.014	2.010	0.132	2.142	0.427	1.238	47.180	23890	20.100	1.320	21.420	4.270	12.380	471.800
120	original	1.2	60x8 weeks	Ag80	Daily	VAC70	75	All	4.977	0.004	0.000	0.004	0.001	0.003	0.098	2389.014	2.010	0.132	2.142	0.427	1.238	47.180	23890	20.100	1.320	21.420	4.270	12.380	471.800
120	original	1.2	60x8 weeks	Ag80	Daily	VAC70	75	All	4.977	0.004	0.000	0.004	0.001	0.003	0.098	2389.014	2.010	0.132	2.142	0.427	1.238	47.180	23890	20.100	1.320	21.420	4.270	12.380	471.800
120	alpha	0.6	60x8 weeks	Ag80	Daily	VAC70	75	All	4.969	0.004	0.000	0.004	0.000	0.005	0.098	2385.176	1.938	0.176	2.114	0.054	2.171	47.064	23852	19.380	1.760	21.140	0.540	21.710	470.640
120	alpha	0.6	60x8 weeks	Ag80	Daily	VAC70	75	All	4.969	0.004	0.000	0.004	0.000	0.005	0.098	2385.176	1.938	0.176	2.114	0.054	2.171	47.064	23852	19.380	1.760	21.140	0.540	21.710	470.640
120	alpha	0.6	60x8 weeks	Ag80	Daily	VAC70	75	All	4.969	0.004	0.000	0.004	0.000	0.005	0.098	2385.176	1.938	0.176	2.114	0.054	2.171	47.064	23852	19.380	1.760	21.140	0.540	21.710	470.640
120	alpha	1	60x8 weeks	Ag80	Daily	VAC70	75	All	4.968	0.004	0.001	0.005	0.000	0.005	0.098	2384.725	1.892	0.293	2.185	0.051	2.252	47.073	23847	18.920	2.930	21.850	0.510	22.520	470.730
120	alpha	1	60x8 weeks	Ag80	Daily	VAC70	75	All	4.968	0.004	0.001	0.005	0.000	0.005	0.098	2384.725	1.892	0.293	2.185	0.051	2.252	47.073	23847	18.920	2.930	21.850	0.510	22.520	470.730
120	alpha	1	60x8 weeks	Ag80	Daily	VAC70	75	All	4.968	0.004	0.001	0.005	0.000	0.005	0.098	2384.725	1.892	0.293	2.185	0.051	2.252	47.073	23847	18.920	2.930	21.850	0.510	22.520	470.730
120	alpha	1.2	60x8 weeks	Ag80	Daily	VAC70	75	All	4.966	0.004	0.001	0.005	0.000	0.005	0.098	2383.646	1.971	0.379	2.350	0.069	2.414	47.013	23836	19.710	3.790	23.500	0.690	24.140	470.130
120	alpha	1.2	60x8 weeks	Ag80	Daily	VAC70	75	All	4.966	0.004	0.001	0.005	0.000	0.005	0.098	2383.646	1.971	0.379	2.350	0.069	2.414	47.013	23836	19.710	3.790	23.500	0.690	24.140	470.130
120	alpha	1.2	60x8 weeks	Ag80	Daily	VAC70	75	All	4.966	0.004	0.001	0.005	0.000	0.005	0.098	2383.646	1.971	0.379	2.350	0.069	2.414	47.013	23836	19.710	3.790	23.500	0.690	24.140	470.130
120	delta	0.6	60x8 weeks	Ag80	Daily	VAC70	75	All	4.961	0.005	0.001	0.006	0.000	0.006	0.098	2381.224	2.238	0.411	2.649	0.078	2.741	46.986	23812	22.380	4.110	26.490	0.780	27.410	469.860
120	delta	0.6	60x8 weeks	Ag80	Daily	VAC70	75	All	4.961	0.005	0.001	0.006	0.000	0.006	0.098	2381.224	2.238	0.411	2.649	0.078	2.741	46.986	23812	22.380	4.110	26.490	0.780	27.410	469.860
120	delta	0.6	60x8 weeks	Ag80	Daily	VAC70	75	All	4.961	0.005	0.001	0.006	0.000	0.006	0.098	2381.224	2.238	0.411	2.649	0.078	2.741	46.986	23812	22.380	4.110	26.490	0.780	27.410	469.860
120	delta	1	60x8 weeks	Ag80	Daily	VAC70	75	All	4.957	0.005	0.002	0.006	0.000	0.006	0.097	2379.262	2.172	0.790	2.962	0.090	3.025	46.726	23793	21.720	7.900	29.620	0.900	30.250	467.260
120	delta	1	60x8 weeks	Ag80	Daily	VAC70	75	All	4.957	0.005	0.002	0.006	0.000	0.006	0.097	2379.262	2.172	0.790	2.962	0.090	3.025	46.726	23793	21.720	7.900	29.620	0.900	30.250	467.260
120	delta	1	60x8 weeks	Ag80	Daily	VAC70	75	All	4.957	0.005	0.002	0.006	0.000	0.006	0.097	2379.262	2.172	0.790	2.962	0.090	3.025	46.726	23793	21.720	7.900	29.620	0.900	30.250	467.260
120	delta	1.2	60x8 weeks	Ag80	Daily	VAC70	75	All	4.954	0.005	0.002	0.006	0.000	0.007	0.098	2377.887	2.181	0.887	3.068	0.094	3.180	46.927	23779	21.810	8.870	30.680	0.940	31.800	469.270
120	delta	1.2	60x8 weeks	Ag80	Daily	VAC70	75	All	4.954	0.005	0.002	0.006	0.000	0.007	0.098	2377.887	2.181	0.887	3.068	0.094	3.180	46.927	23779	21.810	8.870	30.680	0.940	31.800	469.270
120	delta	1.2	60x8 weeks	Ag80	Daily	VAC70	75	All	4.954	0.005	0.002	0.006	0.000	0.007	0.098	2377.887	2.181	0.887	3.068	0.094	3.180	46.927	23779	21.810	8.870	30.680	0.940	31.800	469.270
120	original	0.6	60x8 weeks	Ag80	Daily	VAC70	100	All	4.990	0.003	0.000	0.003	0.001	0.001	0.099	2395.236	1.268	0.006	1.274	0.482	0.248	47.439	23952	12.680	0.060	12.740	4.820	2.480	474.390
120	original	0.6	60x8 weeks	Ag80	Daily	VAC70	100	All	4.990	0.003	0.000	0.003	0.001	0.001	0.099	2395.236	1.268	0.006	1.274	0.482	0.248	47.439	23952	12.680	0.060	12.740	4.820	2.480	474.390
120	original	0.6	60x8 weeks	Ag80	Daily	VAC70	100	All	4.990	0.003	0.000	0.003	0.001	0.001	0.099	2395.236	1.268	0.006	1.274	0.482	0.248	47.439	23952	12.680	0.060	12.740	4.820	2.480	474.390
120	original	1	60x8 weeks	Ag80	Daily	VAC70	100	All	4.990	0.003	0.000	0.003	0.001	0.001	0.099	2395.278	1.277	0.011	1.288	0.478	0.257	47.594	23953	12.770	0.110	12.880	4.780	2.570	475.940
120	original	1	60x8 weeks	Ag80	Daily	VAC70	100	All	4.990	0.003	0.000	0.003	0.001	0.001	0.099	2395.278	1.277	0.011	1.288	0.478	0.257	47.594	23953	12.770	0.110	12.880	4.780	2.570	475.940
120	original	1	60x8 weeks	Ag80	Daily	VAC70	100	All	4.990	0.003	0.000	0.003	0.001	0.001	0.099	2395.278	1.277	0.011	1.288	0.478	0.257	47.594	23953	12.770	0.110	12.880	4.780	2.570	475.940
120	original	1.2	60x8 weeks	Ag80	Daily	VAC70	100	All	4.990	0.003	0.000	0.003	0.001	0.001	0.099	2395.321	1.255	0.021	1.276	0.469	0.252	47.589	23953	12.550	0.210	12.760	4.690	2.520	475.890
120	original	1.2	60x8 weeks	Ag80	Daily	VAC70	100	All	4.990	0.003	0.000	0.003	0.001	0.001	0.099	2395.321	1.255	0.021	1.276	0.469	0.252	47.589	23953	12.550	0.210	12.760	4.690	2.520	475.890
120	original	1.2	60x8 weeks	Ag80	Daily	VAC70	100	All	4.990	0.003	0.000	0.003	0.001	0.001	0.099	2395.321	1.255	0.021	1.276	0.469	0.252	47.589	23953	12.550	0.210	12.760	4.690	2.520	475.890
120	alpha	0.6	60x8 weeks	Ag80	Daily	VAC70	100	All	4.982	0.003	0.000	0.003	0.000	0.003	0.099	2391.386	1.234	0.048	1.282	0.043	1.267	47.407	23914	12.340	0.480	12.820	0.430	12.670	474.070
120	alpha	0.6	60x8 weeks	Ag80	Daily	VAC70	100	All	4.982	0.003	0.000	0.003	0.000	0.003	0.099	2391.386	1.234	0.048	1.282	0.043	1.267	47.407	23914	12.340	0.480	12.820	0.430	12.670	474.070
120	alpha	0.6	60x8 weeks	Ag80	Daily	VAC70	100	All	4.982	0.003	0.000	0.003	0.000	0.003	0.099	2391.386	1.234	0.048	1.282	0.043	1.267	47.407	23914	12.340	0.480	12.820	0.430	12.670	474.070
120	alpha	1	60x8 weeks	Ag80	Daily	VAC70	100	All	4.981	0.003	0.000	0.003	0.000	0.003	0.099	2391.042	1.251	0.080	1.331	0.049	1.321	47.332	23910	12.510	0.800	13.310	0.490	13.210	473.320
120	alpha	1	60x8 weeks	Ag80	Daily	VAC70	100	All	4.981	0.003	0.000	0.003	0.000	0.003	0.099	2391.042	1.251	0.080	1.331	0.049	1.321	47.332	23910	12.510	0.800	13.310	0.490	13.210	473.320
120	alpha	1	60x8 weeks	Ag80	Daily	VAC70	100	All	4.981	0.003	0.000	0.003	0.000	0.003	0.099	2391.042	1.251	0.080	1.331	0.049	1.321	47.332	23910	12.510	0.800	13.310	0.490	13.210	473.320
120	alpha	1.2	60x8 weeks	Ag80	Daily	VAC70	100	All	4.980	0.003	0.000	0.003	0.000	0.003	0.099	2390.569	1.263	0.114	1.377	0.053	1.374	47.575	23906	12.630	1.140	13.770	0.530	13.740	475.750
120	alpha	1.2	60x8 weeks	Ag80	Daily	VAC70	100	All	4.980	0.003	0.000	0.003	0.000	0.003	0.099	2390.569	1.263	0.114	1.377	0.053	1.374	47.575	23906	12.630	1.140	13.770	0.530	13.740	475.750
120	alpha	1.2	60x8 weeks	Ag80	Daily	VAC70	100	All	4.980	0.003	0.000	0.003	0.000	0.003	0.099	2390.569	1.263	0.114	1.377	0.053	1.374	47.575	23906	12.630	1.140	13.770	0.530	13.740	475.750
120	delta	0.6	60x8 weeks	Ag80	Daily	VAC70	100	All	4.973	0.003	0.000	0.004	0.000	0.004	0.098	2387.239	1.673	0.167	1.840	0.074	1.861	47.138	23872	16.730	1.670	18.400	0.740	18.610	471.380
120	delta	0.6	60x8 weeks	Ag80	Daily	VAC70	100	All	4.973	0.003	0.000	0.004	0.000	0.004	0.098	2387.239	1.673	0.167	1.840	0.074	1.861	47.138	23872	16.730	1.670	18.400	0.740	18.610	471.380
120	delta	0.6	60x8 weeks	Ag80	Daily	VAC70	100	All	4.973	0.003	0.000	0.004	0.000	0.004	0.098	2387.239	1.673	0.167	1.840	0.074	1.861	47.138	23872	16.730	1.670	18.400	0.740	18.610	471.380
120	delta	1	60x8 weeks	Ag80	Daily	VAC70	100	All	4.970	0.004	0.001	0.004	0.000	0.004	0.099	2385.555	1.776	0.302	2.078	0.075	2.086	47.446	23856	17.760	3.020	20.780	0.750	20.860	474.460
120	delta	1	60x8 weeks	Ag80	Daily	VAC70	100	All	4.970	0.004	0.001	0.004	0.000	0.004	0.099	2385.555	1.776	0.302	2.078	0.075	2.086	47.446	23856	17.760	3.020	20.780	0.750	20.860	474.460
120	delta	1	60x8 weeks	Ag80	Daily	VAC70	100	All	4.970	0.004	0.001	0.004	0.000	0.004	0.099	2385.555	1.776	0.302	2.078	0.075	2.086	47.446	23856	17.760	3.020	20.780	0.750	20.860	474.460
120	delta	1.2	60x8 weeks	Ag80	Daily	VAC70	100	All	4.971	0.003	0.001	0.004	0.000	0.004	0.098	2386.090	1.627	0.372	1.999	0.067	2.032	46.966	23861	16.270	3.720	19.990	0.670	20.320	469.660
120	delta	1.2	60x8 weeks	Ag80	Daily	VAC70	100	All	4.971	0.003	0.001	0.004	0.000	0.004	0.098	2386.090	1.627	0.372	1.999	0.067	2.032	46.966	23861	16.270	3.720	19.990	0.670	20.320	469.660
120	delta	1.2	60x8 weeks	Ag80	Daily	VAC70	100	All	4.971	0.003	0.001	0.004	0.000	0.004	0.098	2386.090	1.627	0.372	1.999	0.067	2.032	46.966	23861	16.270	3.720	19.990	0.670	20.320	469.660
120	original	0.6	60x8 weeks	Ag80	Daily	VAC50	25	All	4.950	0.007	0.001	0.008	0.001	0.007	0.097	2376.084	3.391	0.358	3.749	0.268	3.343	46.619	23761	33.910	3.580	37.490	2.680	33.430	466.190
120	original	0.6	60x8 weeks	Ag80	Daily	VAC50	25	All	4.950	0.007	0.001	0.008	0.001	0.007	0.097	2376.084	3.391	0.358	3.749	0.268	3.343	46.619	23761	33.910	3.580	37.490	2.680	33.430	466.190
120	original	0.6	60x8 weeks	Ag80	Daily	VAC50	25	All	4.950	0.007	0.001	0.008	0.001	0.007	0.097	2376.084	3.391	0.358	3.749	0.268	3.343	46.619	23761	33.910	3.580	37.490	2.680	33.430	466.190
120	original	1	60x8 weeks	Ag80	Daily	VAC50	25	All	4.947	0.007	0.001	0.009	0.001	0.007	0.097	2374.407	3.486	0.601	4.087	0.335	3.553	46.522	23744	34.860	6.010	40.870	3.350	35.530	465.220
120	original	1	60x8 weeks	Ag80	Daily	VAC50	25	All	4.947	0.007	0.001	0.009	0.001	0.007	0.097	2374.407	3.486	0.601	4.087	0.335	3.553	46.522	23744	34.860	6.010	40.870	3.350	35.530	465.220
120	original	1	60x8 weeks	Ag80	Daily	VAC50	25	All	4.947	0.007	0.001	0.009	0.001	0.007	0.097	2374.407	3.486	0.601	4.087	0.335	3.553	46.522	23744	34.860	6.010	40.870	3.350	35.530	465.220
120	original	1.2	60x8 weeks	Ag80	Daily	VAC50	25	All	4.945	0.007	0.002	0.009	0.001	0.008	0.096	2373.427	3.450	0.788	4.238	0.337	3.701	45.966	23734	34.500	7.880	42.380	3.370	37.010	459.660
120	original	1.2	60x8 weeks	Ag80	Daily	VAC50	25	All	4.945	0.007	0.002	0.009	0.001	0.008	0.096	2373.427	3.450	0.788	4.238	0.337	3.701	45.966	23734	34.500	7.880	42.380	3.370	37.010	459.660
120	original	1.2	60x8 weeks	Ag80	Daily	VAC50	25	All	4.945	0.007	0.002	0.009	0.001	0.008	0.096	2373.427	3.450	0.788	4.238	0.337	3.701	45.966	23734	34.500	7.880	42.380	3.370	37.010	459.660
120	alpha	0.6	60x8 weeks	Ag80	Daily	VAC50	25	All	4.942	0.007	0.001	0.008	0.000	0.009	0.097	2372.073	3.416	0.594	4.010	0.097	4.125	46.545	23721	34.160	5.940	40.100	0.970	41.250	465.450
120	alpha	0.6	60x8 weeks	Ag80	Daily	VAC50	25	All	4.942	0.007	0.001	0.008	0.000	0.009	0.097	2372.073	3.416	0.594	4.010	0.097	4.125	46.545	23721	34.160	5.940	40.100	0.970	41.250	465.450
120	alpha	0.6	60x8 weeks	Ag80	Daily	VAC50	25	All	4.942	0.007	0.001	0.008	0.000	0.009	0.097	2372.073	3.416	0.594	4.010	0.097	4.125	46.545	23721	34.160	5.940	40.100	0.970	41.250	465.450
120	alpha	1	60x8 weeks	Ag80	Daily	VAC50	25	All	4.932	0.007	0.002	0.010	0.000	0.010	0.096	2367.412	3.503	1.174	4.677	0.094	4.848	46.198	23674	35.030	11.740	46.770	0.940	48.480	461.980
120	alpha	1	60x8 weeks	Ag80	Daily	VAC50	25	All	4.932	0.007	0.002	0.010	0.000	0.010	0.096	2367.412	3.503	1.174	4.677	0.094	4.848	46.198	23674	35.030	11.740	46.770	0.940	48.480	461.980
120	alpha	1	60x8 weeks	Ag80	Daily	VAC50	25	All	4.932	0.007	0.002	0.010	0.000	0.010	0.096	2367.412	3.503	1.174	4.677	0.094	4.848	46.198	23674	35.030	11.740	46.770	0.940	48.480	461.980
120	alpha	1.2	60x8 weeks	Ag80	Daily	VAC50	25	All	4.928	0.007	0.003	0.010	0.000	0.011	0.095	2365.466	3.386	1.486	4.872	0.109	5.062	45.619	23655	33.860	14.860	48.720	1.090	50.620	456.190
120	alpha	1.2	60x8 weeks	Ag80	Daily	VAC50	25	All	4.928	0.007	0.003	0.010	0.000	0.011	0.095	2365.466	3.386	1.486	4.872	0.109	5.062	45.619	23655	33.860	14.860	48.720	1.090	50.620	456.190
120	alpha	1.2	60x8 weeks	Ag80	Daily	VAC50	25	All	4.928	0.007	0.003	0.010	0.000	0.011	0.095	2365.466	3.386	1.486	4.872	0.109	5.062	45.619	23655	33.860	14.860	48.720	1.090	50.620	456.190

120	delta	0.6	60x8 weeks	Ag80	Daily	VAC50	25	All	4.927	0.007	0.003	0.010	0.000	0.011	0.096	2364.884	3.485	1.533	5.018	0.140	5.135	46.064	23649	34.850	15.330	50.180	1.400	51.350	460.640
120	delta	0.6	60x8 weeks	Ag80	Daily	VAC50	25	All	4.927	0.007	0.003	0.010	0.000	0.011	0.096	2364.884	3.485	1.533	5.018	0.140	5.135	46.064	23649	34.850	15.330	50.180	1.400	51.350	460.640
120	delta	0.6	60x8 weeks	Ag80	Daily	VAC50	25	All	4.927	0.007	0.003	0.010	0.000	0.011	0.096	2364.884	3.485	1.533	5.018	0.140	5.135	46.064	23649	34.850	15.330	50.180	1.400	51.350	460.640
120	delta	1	60x8 weeks	Ag80	Daily	VAC50	25	All	4.902	0.007	0.007	0.014	0.000	0.014	0.095	2352.853	3.485	3.198	6.683	0.184	6.931	45.366	23529	34.850	31.980	66.830	1.840	69.310	453.660
120	delta	1	60x8 weeks	Ag80	Daily	VAC50	25	All	4.902	0.007	0.007	0.014	0.000	0.014	0.095	2352.853	3.485	3.198	6.683	0.184	6.931	45.366	23529	34.850	31.980	66.830	1.840	69.310	453.660
120	delta	1	60x8 weeks	Ag80	Daily	VAC50	25	All	4.902	0.007	0.007	0.014	0.000	0.014	0.095	2352.853	3.485	3.198	6.683	0.184	6.931	45.366	23529	34.850	31.980	66.830	1.840	69.310	453.660
120	delta	1.2	60x8 weeks	Ag80	Daily	VAC50	25	All	4.881	0.007	0.009	0.016	0.000	0.017	0.095	2343.034	3.408	4.471	7.879	0.202	8.276	45.523	23430	34.080	44.710	78.790	2.020	82.760	455.230
120	delta	1.2	60x8 weeks	Ag80	Daily	VAC50	25	All	4.881	0.007	0.009	0.016	0.000	0.017	0.095	2343.034	3.408	4.471	7.879	0.202	8.276	45.523	23430	34.080	44.710	78.790	2.020	82.760	455.230
120	delta	1.2	60x8 weeks	Ag80	Daily	VAC50	25	All	4.881	0.007	0.009	0.016	0.000	0.017	0.095	2343.034	3.408	4.471	7.879	0.202	8.276	45.523	23430	34.080	44.710	78.790	2.020	82.760	455.230
120	original	0.6	60x8 weeks	Ag80	Daily	VAC50	50	All	4.961	0.006	0.000	0.007	0.001	0.005	0.097	2381.404	2.991	0.206	3.197	0.480	2.321	46.715	23814	29.910	2.060	31.970	4.800	23.210	467.150
120	original	0.6	60x8 weeks	Ag80	Daily	VAC50	50	All	4.961	0.006	0.000	0.007	0.001	0.005	0.097	2381.404	2.991	0.206	3.197	0.480	2.321	46.715	23814	29.910	2.060	31.970	4.800	23.210	467.150
120	original	0.6	60x8 weeks	Ag80	Daily	VAC50	50	All	4.961	0.006	0.000	0.007	0.001	0.005	0.097	2381.404	2.991	0.206	3.197	0.480	2.321	46.715	23814	29.910	2.060	31.970	4.800	23.210	467.150
120	original	1	60x8 weeks	Ag80	Daily	VAC50	50	All	4.961	0.006	0.001	0.007	0.001	0.005	0.098	2381.308	2.904	0.360	3.264	0.489	2.353	46.894	23813	29.040	3.600	32.640	4.890	23.530	468.940
120	original	1	60x8 weeks	Ag80	Daily	VAC50	50	All	4.961	0.006	0.001	0.007	0.001	0.005	0.098	2381.308	2.904	0.360	3.264	0.489	2.353	46.894	23813	29.040	3.600	32.640	4.890	23.530	468.940
120	original	1	60x8 weeks	Ag80	Daily	VAC50	50	All	4.961	0.006	0.001	0.007	0.001	0.005	0.098	2381.308	2.904	0.360	3.264	0.489	2.353	46.894	23813	29.040	3.600	32.640	4.890	23.530	468.940
120	original	1.2	60x8 weeks	Ag80	Daily	VAC50	50	All	4.960	0.006	0.001	0.007	0.001	0.005	0.097	2380.571	2.954	0.476	3.430	0.510	2.458	46.712	23806	29.540	4.760	34.300	5.100	24.580	467.120
120	original	1.2	60x8 weeks	Ag80	Daily	VAC50	50	All	4.960	0.006	0.001	0.007	0.001	0.005	0.097	2380.571	2.954	0.476	3.430	0.510	2.458	46.712	23806	29.540	4.760	34.300	5.100	24.580	467.120
120	original	1.2	60x8 weeks	Ag80	Daily	VAC50	50	All	4.960	0.006	0.001	0.007	0.001	0.005	0.097	2380.571	2.954	0.476	3.430	0.510	2.458	46.712	23806	29.540	4.760	34.300	5.100	24.580	467.120
120	alpha	0.6	60x8 weeks	Ag80	Daily	VAC50	50	All	4.950	0.006	0.001	0.007	0.000	0.007	0.097	2375.993	3.017	0.445	3.462	0.100	3.535	46.636	23760	30.170	4.450	34.620	1.000	35.350	466.360
120	alpha	0.6	60x8 weeks	Ag80	Daily	VAC50	50	All	4.950	0.006	0.001	0.007	0.000	0.007	0.097	2375.993	3.017	0.445	3.462	0.100	3.535	46.636	23760	30.170	4.450	34.620	1.000	35.350	466.360
120	alpha	0.6	60x8 weeks	Ag80	Daily	VAC50	50	All	4.950	0.006	0.001	0.007	0.000	0.007	0.097	2375.993	3.017	0.445	3.462	0.100	3.535	46.636	23760	30.170	4.450	34.620	1.000	35.350	466.360
120	alpha	1	60x8 weeks	Ag80	Daily	VAC50	50	All	4.946	0.006	0.002	0.008	0.000	0.008	0.097	2373.860	2.982	0.750	3.732	0.093	3.811	46.634	23739	29.820	7.500	37.320	0.930	38.110	466.340
120	alpha	1	60x8 weeks	Ag80	Daily	VAC50	50	All	4.946	0.006	0.002	0.008	0.000	0.008	0.097	2373.860	2.982	0.750	3.732	0.093	3.811	46.634	23739	29.820	7.500	37.320	0.930	38.110	466.340
120	alpha	1	60x8 weeks	Ag80	Daily	VAC50	50	All	4.946	0.006	0.002	0.008	0.000	0.008	0.097	2373.860	2.982	0.750	3.732	0.093	3.811	46.634	23739	29.820	7.500	37.320	0.930	38.110	466.340
120	alpha	1.2	60x8 weeks	Ag80	Daily	VAC50	50	All	4.941	0.006	0.002	0.008	0.000	0.009	0.097	2371.917	2.980	1.009	3.989	0.115	4.130	46.514	23719	29.800	10.090	39.890	1.150	41.300	465.140
120	alpha	1.2	60x8 weeks	Ag80	Daily	VAC50	50	All	4.941	0.006	0.002	0.008	0.000	0.009	0.097	2371.917	2.980	1.009	3.989	0.115	4.130	46.514	23719	29.800	10.090	39.890	1.150	41.300	465.140
120	alpha	1.2	60x8 weeks	Ag80	Daily	VAC50	50	All	4.941	0.006	0.002	0.008	0.000	0.009	0.097	2371.917	2.980	1.009	3.989	0.115	4.130	46.514	23719	29.800	10.090	39.890	1.150	41.300	465.140
120	delta	0.6	60x8 weeks	Ag80	Daily	VAC50	50	All	4.936	0.007	0.002	0.009	0.000	0.009	0.096	2369.419	3.246	1.091	4.337	0.149	4.478	46.061	23694	32.460	10.910	43.370	1.490	44.780	460.610
120	delta	0.6	60x8 weeks	Ag80	Daily	VAC50	50	All	4.936	0.007	0.002	0.009	0.000	0.009	0.096	2369.419	3.246	1.091	4.337	0.149	4.478	46.061	23694	32.460	10.910	43.370	1.490	44.780	460.610
120	delta	0.6	60x8 weeks	Ag80	Daily	VAC50	50	All	4.936	0.007	0.002	0.009	0.000	0.009	0.096	2369.419	3.246	1.091	4.337	0.149	4.478	46.061	23694	32.460	10.910	43.370	1.490	44.780	460.610
120	delta	1	60x8 weeks	Ag80	Daily	VAC50	50	All	4.919	0.007	0.005	0.011	0.000	0.012	0.096	2360.920	3.217	2.222	5.439	0.166	5.645	45.877	23609	32.170	22.220	54.390	1.660	56.450	458.770
120	delta	1	60x8 weeks	Ag80	Daily	VAC50	50	All	4.919	0.007	0.005	0.011	0.000	0.012	0.096	2360.920	3.217	2.222	5.439	0.166	5.645	45.877	23609	32.170	22.220	54.390	1.660	56.450	458.770
120	delta	1	60x8 weeks	Ag80	Daily	VAC50	50	All	4.919	0.007	0.005	0.011	0.000	0.012	0.096	2360.920	3.217	2.222	5.439	0.166	5.645	45.877	23609	32.170	22.220	54.390	1.660	56.450	458.770
120	delta	1.2	60x8 weeks	Ag80	Daily	VAC50	50	All	4.909	0.007	0.006	0.013	0.000	0.013	0.095	2356.096	3.210	2.884	6.094	0.146	6.311	45.423	23561	32.100	28.840	60.940	1.460	63.110	454.230
120	delta	1.2	60x8 weeks	Ag80	Daily	VAC50	50	All	4.909	0.007	0.006	0.013	0.000	0.013	0.095	2356.096	3.210	2.884	6.094	0.146	6.311	45.423	23561	32.100	28.840	60.940	1.460	63.110	454.230
120	delta	1.2	60x8 weeks	Ag80	Daily	VAC50	50	All	4.909	0.007	0.006	0.013	0.000	0.013	0.095	2356.096	3.210	2.884	6.094	0.146	6.311	45.423	23561	32.100	28.840	60.940	1.460	63.110	454.230
120	original	0.6	60x8 weeks	Ag80	Daily	VAC50	75	All	4.973	0.005	0.000	0.005	0.001	0.003	0.097	2387.176	2.501	0.095	2.596	0.641	1.315	46.788	23872	25.010	0.950	25.960	6.410	13.150	467.880
120	original	0.6	60x8 weeks	Ag80	Daily	VAC50	75	All	4.973	0.005	0.000	0.005	0.001	0.003	0.097	2387.176	2.501	0.095	2.596	0.641	1.315	46.788	23872	25.010	0.950	25.960	6.410	13.150	467.880
120	original	0.6	60x8 weeks	Ag80	Daily	VAC50	75	All	4.973	0.005	0.000	0.005	0.001	0.003	0.097	2387.176	2.501	0.095	2.596	0.641	1.315	46.788	23872	25.010	0.950	25.960	6.410	13.150	467.880
120	original	1	60x8 weeks	Ag80	Daily	VAC50	75	All	4.973	0.005	0.000	0.006	0.001	0.003	0.098	2387.212	2.504	0.167	2.671	0.636	1.331	46.962	23872	25.040	1.670	26.710	6.360	13.310	469.620
120	original	1	60x8 weeks	Ag80	Daily	VAC50	75	All	4.973	0.005	0.000	0.006	0.001	0.003	0.098	2387.212	2.504	0.167	2.671	0.636	1.331	46.962	23872	25.040	1.670	26.710	6.360	13.310	469.620
120	original	1	60x8 weeks	Ag80	Daily	VAC50	75	All	4.973	0.005	0.000	0.006	0.001	0.003	0.098	2387.212	2.504	0.167	2.671	0.636	1.331	46.962	23872	25.040	1.670	26.710	6.360	13.310	469.620
120	original	1.2	60x8 weeks	Ag80	Daily	VAC50	75	All	4.972	0.005	0.000	0.006	0.001	0.003	0.098	2386.645	2.523	0.204	2.727	0.701	1.353	46.991	23866	25.230	2.040	27.270	7.010	13.530	469.910
120	original	1.2	60x8 weeks	Ag80	Daily	VAC50	75	All	4.972	0.005	0.000	0.006	0.001	0.003	0.098	2386.645	2.523	0.204	2.727	0.701	1.353	46.991	23866	25.230	2.040	27.270	7.010	13.530	469.910
120	original	1.2	60x8 weeks	Ag80	Daily	VAC50	75	All	4.972	0.005	0.000	0.006	0.001	0.003	0.098	2386.645	2.523	0.204	2.727	0.701	1.353	46.991	23866	25.230	2.040	27.270	7.010	13.530	469.910
120	alpha	0.6	60x8 weeks	Ag80	Daily	VAC50	75	All	4.960	0.005	0.001	0.006	0.000	0.006	0.097	2380.766	2.503	0.246	2.749	0.078	2.821	46.731	23808	25.030	2.460	27.490	0.780	28.210	467.310
120	alpha	0.6	60x8 weeks	Ag80	Daily	VAC50	75	All	4.960	0.005	0.001	0.006	0.000	0.006	0.097	2380.766	2.503	0.246	2.749	0.078	2.821	46.731	23808	25.030	2.460	27.490	0.780	28.210	467.310
120	alpha	0.6	60x8 weeks	Ag80	Daily	VAC50	75	All	4.960	0.005	0.001	0.006	0.000	0.006	0.097	2380.766	2.503	0.246	2.749	0.078	2.821	46.731	23808	25.030	2.460	27.490	0.780	28.210	467.310
120	alpha	1	60x8 weeks	Ag80	Daily	VAC50	75	All	4.958	0.005	0.001	0.006	0.000	0.006	0.098	2379.829	2.431	0.457	2.888	0.086	2.939	46.855	23798	24.310	4.570	28.880	0.860	29.390	468.550
120	alpha	1	60x8 weeks	Ag80	Daily	VAC50	75	All	4.958	0.005	0.001	0.006	0.000	0.006	0.098	2379.829	2.431	0.457	2.888	0.086	2.939	46.855	23798	24.310	4.570	28.880	0.860	29.390	468.550
120	alpha	1	60x8 weeks	Ag80	Daily	VAC50	75	All	4.958	0.005	0.001	0.006	0.000	0.006	0.098	2379.829	2.431	0.457	2.888	0.086	2.939	46.855	23798	24.310	4.570	28.880	0.860	29.390	468.550
120	alpha	1.2	60x8 weeks	Ag80	Daily	VAC50	75	All	4.957	0.005	0.001	0.006	0.000	0.006	0.097	2379.239	2.468	0.563	3.031	0.080	3.065	46.569	23792	24.680	5.630	30.310	0.800	30.650	465.690
120	alpha	1.2	60x8 weeks	Ag80	Daily	VAC50	75	All	4.957	0.005	0.001	0.006	0.000	0.006	0.097	2379.239	2.468	0.563	3.031	0.080	3.065	46.569	23792	24.680	5.630	30.310	0.800	30.650	465.690
120	alpha	1.2	60x8 weeks	Ag80	Daily	VAC50	75	All	4.957	0.005	0.001	0.006	0.000	0.006	0.097	2379.239	2.468	0.563	3.031	0.080	3.065	46.569	23792	24.680	5.630	30.310	0.800	30.650	465.690
120	delta	0.6	60x8 weeks	Ag80	Daily	VAC50	75	All	4.945	0.006	0.002	0.008	0.000	0.008	0.097	2373.496	3.006	0.763	3.769	0.132	3.860	46.752	23735	30.060	7.630	37.690	1.320	38.600	467.520
120	delta	0.6	60x8 weeks	Ag80	Daily	VAC50	75	All	4.945	0.006	0.002	0.008	0.000	0.008	0.097	2373.496	3.006	0.763	3.769	0.132	3.860	46.752	23735	30.060	7.630	37.690	1.320	38.600	467.520
120	delta	0.6	60x8 weeks	Ag80	Daily	VAC50	75	All	4.945	0.006	0.002	0.008	0.000	0.008	0.097	2373.496	3.006	0.763	3.769	0.132	3.860	46.752	23735	30.060	7.630	37.690	1.320	38.600	467.520
120	delta	1	60x8 weeks	Ag80	Daily	VAC50	75	All	4.933	0.006	0.003	0.009	0.000	0.010	0.096	2367.634	2.996	1.532	4.528	0.146	4.660	46.270	23676	29.960	15.320	45.280	1.460	46.600	462.700
120	delta	1	60x8 weeks	Ag80	Daily	VAC50	75	All	4.933	0.006	0.003	0.009	0.000	0.010	0.096	2367.634	2.996	1.532	4.528	0.146	4.660	46.270	23676	29.960	15.320	45.280	1.460	46.600	462.700
120	delta	1	60x8 weeks	Ag80	Daily	VAC50	75	All	4.933	0.006	0.003	0.009	0.000	0.010	0.096	2367.634	2.996	1.532	4.528	0.146	4.660	46.270	23676	29.960	15.320	45.280	1.460	46.600	462.700
120	delta	1.2	60x8 weeks	Ag80	Daily	VAC50	75	All	4.929	0.006	0.004	0.010	0.000	0.010	0.096	2366.103	2.867	1.865	4.732	0.121	4.944	46.314	23661	28.670	18.650	47.320	1.210	49.440	463.140
120	delta	1.2	60x8 weeks	Ag80	Daily	VAC50	75	All	4.929	0.006	0.004	0.010	0.000	0.010	0.096	2366.103	2.867	1.865	4.732	0.121	4.944	46.314	23661	28.670	18.650	47.320	1.210	49.440	463.140
120	delta	1.2	60x8 weeks	Ag80	Daily	VAC50	75	All	4.929	0.006	0.004	0.010	0.000	0.010	0.096	2366.103	2.867	1.865	4.732	0.121	4.944	46.314	23661	28.670	18.650	47.320	1.210	49.440	463.140
120	original	0.6	60x8 weeks	Ag80	Daily	VAC50	100	All	4.984	0.004	0.000	0.004	0.002	0.001	0.099	2392.272	2.036	0.029	2.065	0.774	0.424	47.382	23923	20.360	0.290	20.650	7.740	4.240	473.820
120	original	0.6	60x8 weeks	Ag80	Daily	VAC50	100	All	4.984	0.004	0.000	0.004	0.002	0.001	0.099	2392.272	2.036	0.029	2.065	0.774	0.424	47.382	23923	20.360	0.290	20.650	7.740	4.240	473.820
120	original	0.6	60x8 weeks	Ag80	Daily	VAC50	100	All	4.984	0.004	0.000	0.004	0.002	0.001	0.099	2392.272	2.036	0.029	2.065	0.774	0.424	47.382	23923	20.360	0.290	20.650	7.740	4.240	473.820
120	original	1	60x8 weeks	Ag80	Daily	VAC50	100	All	4.983	0.004	0.000	0.004	0.002	0.001	0.099	2391.706	2.105	0.035	2.140	0.834	0.442	47.649	23917	21.050	0.350	21.400	8.340	4.420	476.490
120	original	1	60x8 weeks	Ag80	Daily	VAC50	100	All	4.983	0.004	0.000	0.004	0.002	0.001	0.099	2391.706	2.105	0.035	2.140	0.834	0.442	47.649	23917	21.050	0.350	21.400	8.340	4.420	476.490
120	original	1	60x8 weeks	Ag80	Daily	VAC50	100	All	4.983	0.004	0.000	0.004	0.002	0.001	0.099	2391.706	2.105	0.035	2.140	0.834	0.442	47.649	23917	21.050	0.350	21.400	8.340	4.420	476.490
120	original	1.2	60x8 weeks	Ag80	Daily	VAC50	100	All	4.983	0.004	0.000	0.005	0.002	0.001	0.098	2391.699	2.151	0.052	2.203	0.868	0.413	47.115	23917	21.510	0.520	22.030	8.680	4.130	471.150
120	original	1.2	60x8 weeks	Ag80	Daily	VAC50	100	All	4.983	0.004	0.000	0.005	0.002	0.001	0.098	2391.699	2.151	0.052	2.203	0.868	0.413	47.115	23917	21.510	0.520	22.030	8.680	4.130	471.150

120	original	1.2	60x8 weeks	Ag80	Daily	VAC50	100	All	4.983	0.004	0.000	0.005	0.002	0.001	0.098	2391.699	2.151	0.052	2.203	0.868	0.413	47.115	23917	21.510	0.520	22.030	8.680	4.130	471.150
120	alpha	0.6	60x8 weeks	Ag80	Daily	VAC50	100	All	4.969	0.004	0.000	0.005	0.000	0.005	0.099	2384.983	2.034	0.154	2.188	0.079	2.186	47.430	23850	20.340	1.540	21.880	0.790	21.860	474.300
120	alpha	0.6	60x8 weeks	Ag80	Daily	VAC50	100	All	4.969	0.004	0.000	0.005	0.000	0.005	0.099	2384.983	2.034	0.154	2.188	0.079	2.186	47.430	23850	20.340	1.540	21.880	0.790	21.860	474.300
120	alpha	0.6	60x8 weeks	Ag80	Daily	VAC50	100	All	4.969	0.004	0.000	0.005	0.000	0.005	0.099	2384.983	2.034	0.154	2.188	0.079	2.186	47.430	23850	20.340	1.540	21.880	0.790	21.860	474.300
120	alpha	1	60x8 weeks	Ag80	Daily	VAC50	100	All	4.967	0.004	0.000	0.005	0.000	0.005	0.099	2384.185	2.097	0.231	2.328	0.082	2.331	47.399	23842	20.970	2.310	23.280	0.820	23.310	473.990
120	alpha	1	60x8 weeks	Ag80	Daily	VAC50	100	All	4.967	0.004	0.000	0.005	0.000	0.005	0.099	2384.185	2.097	0.231	2.328	0.082	2.331	47.399	23842	20.970	2.310	23.280	0.820	23.310	473.990
120	alpha	1	60x8 weeks	Ag80	Daily	VAC50	100	All	4.967	0.004	0.000	0.005	0.000	0.005	0.099	2384.185	2.097	0.231	2.328	0.082	2.331	47.399	23842	20.970	2.310	23.280	0.820	23.310	473.990
120	alpha	1.2	60x8 weeks	Ag80	Daily	VAC50	100	All	4.967	0.004	0.001	0.005	0.000	0.005	0.098	2384.215	2.013	0.293	2.306	0.100	2.261	47.031	23842	20.130	2.930	23.060	1.000	22.610	470.310
120	alpha	1.2	60x8 weeks	Ag80	Daily	VAC50	100	All	4.967	0.004	0.001	0.005	0.000	0.005	0.098	2384.215	2.013	0.293	2.306	0.100	2.261	47.031	23842	20.130	2.930	23.060	1.000	22.610	470.310
120	alpha	1.2	60x8 weeks	Ag80	Daily	VAC50	100	All	4.967	0.004	0.001	0.005	0.000	0.005	0.098	2384.215	2.013	0.293	2.306	0.100	2.261	47.031	23842	20.130	2.930	23.060	1.000	22.610	470.310
120	delta	0.6	60x8 weeks	Ag80	Daily	VAC50	100	All	4.955	0.006	0.001	0.006	0.000	0.007	0.097	2378.559	2.641	0.454	3.095	0.102	3.126	46.635	23786	26.410	4.540	30.950	1.020	31.260	466.350
120	delta	0.6	60x8 weeks	Ag80	Daily	VAC50	100	All	4.955	0.006	0.001	0.006	0.000	0.007	0.097	2378.559	2.641	0.454	3.095	0.102	3.126	46.635	23786	26.410	4.540	30.950	1.020	31.260	466.350
120	delta	0.6	60x8 weeks	Ag80	Daily	VAC50	100	All	4.955	0.006	0.001	0.006	0.000	0.007	0.097	2378.559	2.641	0.454	3.095	0.102	3.126	46.635	23786	26.410	4.540	30.950	1.020	31.260	466.350
120	delta	1	60x8 weeks	Ag80	Daily	VAC50	100	All	4.950	0.005	0.002	0.007	0.000	0.007	0.097	2375.962	2.639	0.778	3.417	0.099	3.537	46.569	23760	26.390	7.780	34.170	0.990	35.370	465.690
120	delta	1	60x8 weeks	Ag80	Daily	VAC50	100	All	4.950	0.005	0.002	0.007	0.000	0.007	0.097	2375.962	2.639	0.778	3.417	0.099	3.537	46.569	23760	26.390	7.780	34.170	0.990	35.370	465.690
120	delta	1	60x8 weeks	Ag80	Daily	VAC50	100	All	4.950	0.005	0.002	0.007	0.000	0.007	0.097	2375.962	2.639	0.778	3.417	0.099	3.537	46.569	23760	26.390	7.780	34.170	0.990	35.370	465.690
120	delta	1.2	60x8 weeks	Ag80	Daily	VAC50	100	All	4.946	0.006	0.002	0.008	0.000	0.008	0.097	2374.106	2.649	1.021	3.670	0.122	3.754	46.779	23741	26.490	10.210	36.700	1.220	37.540	467.790
120	delta	1.2	60x8 weeks	Ag80	Daily	VAC50	100	All	4.946	0.006	0.002	0.008	0.000	0.008	0.097	2374.106	2.649	1.021	3.670	0.122	3.754	46.779	23741	26.490	10.210	36.700	1.220	37.540	467.790
120	delta	1.2	60x8 weeks	Ag80	Daily	VAC50	100	All	4.946	0.006	0.002	0.008	0.000	0.008	0.097	2374.106	2.649	1.021	3.670	0.122	3.754	46.779	23741	26.490	10.210	36.700	1.220	37.540	467.790
120	original	0.6	60x8 weeks	Ag80	MWF	NOVAC	0	All	2.964	0.008	0.002	0.010	0.000	0.009	0.058	1422.835	3.893	0.856	4.749	0.194	4.545	27.651	14228	38.930	8.560	47.490	1.940	45.450	276.510
120	original	0.6	60x8 weeks	Ag80	MWF	NOVAC	0	All	2.964	0.008	0.002	0.010	0.000	0.009	0.058	1422.835	3.893	0.856	4.749	0.194	4.545	27.651	14228	38.930	8.560	47.490	1.940	45.450	276.510
120	original	0.6	60x8 weeks	Ag80	MWF	NOVAC	0	All	2.964	0.008	0.002	0.010	0.000	0.009	0.058	1422.835	3.893	0.856	4.749	0.194	4.545	27.651	14228	38.930	8.560	47.490	1.940	45.450	276.510
120	original	1	60x8 weeks	Ag80	MWF	NOVAC	0	All	2.960	0.008	0.003	0.011	0.000	0.011	0.057	1420.856	3.798	1.547	5.345	0.226	5.119	27.455	14209	37.980	15.470	53.450	2.260	51.190	274.550
120	original	1	60x8 weeks	Ag80	MWF	NOVAC	0	All	2.960	0.008	0.003	0.011	0.000	0.011	0.057	1420.856	3.798	1.547	5.345	0.226	5.119	27.455	14209	37.980	15.470	53.450	2.260	51.190	274.550
120	original	1	60x8 weeks	Ag80	MWF	NOVAC	0	All	2.960	0.008	0.003	0.011	0.000	0.011	0.057	1420.856	3.798	1.547	5.345	0.226	5.119	27.455	14209	37.980	15.470	53.450	2.260	51.190	274.550
120	original	1.2	60x8 weeks	Ag80	MWF	NOVAC	0	All	2.957	0.008	0.004	0.012	0.000	0.011	0.057	1419.549	3.785	1.853	5.638	0.239	5.417	27.537	14195	37.850	18.530	56.380	2.390	54.170	275.370
120	original	1.2	60x8 weeks	Ag80	MWF	NOVAC	0	All	2.957	0.008	0.004	0.012	0.000	0.011	0.057	1419.549	3.785	1.853	5.638	0.239	5.417	27.537	14195	37.850	18.530	56.380	2.390	54.170	275.370
120	original	1.2	60x8 weeks	Ag80	MWF	NOVAC	0	All	2.957	0.008	0.004	0.012	0.000	0.011	0.057	1419.549	3.785	1.853	5.638	0.239	5.417	27.537	14195	37.850	18.530	56.380	2.390	54.170	275.370
120	alpha	0.6	60x8 weeks	Ag80	MWF	NOVAC	0	All	2.956	0.008	0.003	0.011	0.000	0.012	0.057	1418.929	3.879	1.626	5.505	0.237	5.558	27.452	14189	38.790	16.260	55.050	2.370	55.580	274.520
120	alpha	0.6	60x8 weeks	Ag80	MWF	NOVAC	0	All	2.956	0.008	0.003	0.011	0.000	0.012	0.057	1418.929	3.879	1.626	5.505	0.237	5.558	27.452	14189	38.790	16.260	55.050	2.370	55.580	274.520
120	alpha	0.6	60x8 weeks	Ag80	MWF	NOVAC	0	All	2.956	0.008	0.003	0.011	0.000	0.012	0.057	1418.929	3.879	1.626	5.505	0.237	5.558	27.452	14189	38.790	16.260	55.050	2.370	55.580	274.520
120	alpha	1	60x8 weeks	Ag80	MWF	NOVAC	0	All	2.943	0.008	0.007	0.015	0.001	0.015	0.056	1412.552	3.772	3.512	7.284	0.318	7.349	27.052	14126	37.720	35.120	72.840	3.180	73.490	270.520
120	alpha	1	60x8 weeks	Ag80	MWF	NOVAC	0	All	2.943	0.008	0.007	0.015	0.001	0.015	0.056	1412.552	3.772	3.512	7.284	0.318	7.349	27.052	14126	37.720	35.120	72.840	3.180	73.490	270.520
120	alpha	1	60x8 weeks	Ag80	MWF	NOVAC	0	All	2.943	0.008	0.007	0.015	0.001	0.015	0.056	1412.552	3.772	3.512	7.284	0.318	7.349	27.052	14126	37.720	35.120	72.840	3.180	73.490	270.520
120	alpha	1.2	60x8 weeks	Ag80	MWF	NOVAC	0	All	2.931	0.008	0.011	0.018	0.001	0.018	0.056	1406.752	3.642	5.194	8.836	0.416	8.863	26.900	14068	36.420	51.940	88.360	4.160	88.630	269.000
120	alpha	1.2	60x8 weeks	Ag80	MWF	NOVAC	0	All	2.931	0.008	0.011	0.018	0.001	0.018	0.056	1406.752	3.642	5.194	8.836	0.416	8.863	26.900	14068	36.420	51.940	88.360	4.160	88.630	269.000
120	alpha	1.2	60x8 weeks	Ag80	MWF	NOVAC	0	All	2.931	0.008	0.011	0.018	0.001	0.018	0.056	1406.752	3.642	5.194	8.836	0.416	8.863	26.900	14068	36.420	51.940	88.360	4.160	88.630	269.000
120	delta	0.6	60x8 weeks	Ag80	MWF	NOVAC	0	All	2.924	0.008	0.012	0.020	0.001	0.020	0.055	1403.527	3.784	5.824	9.608	0.608	9.482	26.562	14035	37.840	58.240	96.080	6.080	94.820	265.620
120	delta	0.6	60x8 weeks	Ag80	MWF	NOVAC	0	All	2.924	0.008	0.012	0.020	0.001	0.020	0.055	1403.527	3.784	5.824	9.608	0.608	9.482	26.562	14035	37.840	58.240	96.080	6.080	94.820	265.620
120	delta	0.6	60x8 weeks	Ag80	MWF	NOVAC	0	All	2.924	0.008	0.012	0.020	0.001	0.020	0.055	1403.527	3.784	5.824	9.608	0.608	9.482	26.562	14035	37.840	58.240	96.080	6.080	94.820	265.620
120	delta	1	60x8 weeks	Ag80	MWF	NOVAC	0	All	2.795	0.007	0.045	0.052	0.003	0.052	0.048	1341.811	3.284	21.727	25.011	1.676	24.993	22.908	13418	32.840	217.270	250.110	16.760	249.930	229.080
120	delta	1	60x8 weeks	Ag80	MWF	NOVAC	0	All	2.795	0.007	0.045	0.052	0.003	0.052	0.048	1341.811	3.284	21.727	25.011	1.676	24.993	22.908	13418	32.840	217.270	250.110	16.760	249.930	229.080
120	delta	1	60x8 weeks	Ag80	MWF	NOVAC	0	All	2.795	0.007	0.045	0.052	0.003	0.052	0.048	1341.811	3.284	21.727	25.011	1.676	24.993	22.908	13418	32.840	217.270	250.110	16.760	249.930	229.080
120	delta	1.2	60x8 weeks	Ag80	MWF	NOVAC	0	All	2.715	0.006	0.065	0.071	0.005	0.072	0.042	1303.066	2.923	31.338	34.261	2.364	34.405	20.232	13031	29.230	313.380	342.610	23.640	344.050	202.320
120	delta	1.2	60x8 weeks	Ag80	MWF	NOVAC	0	All	2.715	0.006	0.065	0.071	0.005	0.072	0.042	1303.066	2.923	31.338	34.261	2.364	34.405	20.232	13031	29.230	313.380	342.610	23.640	344.050	202.320
120	delta	1.2	60x8 weeks	Ag80	MWF	NOVAC	0	All	2.715	0.006	0.065	0.071	0.005	0.072	0.042	1303.066	2.923	31.338	34.261	2.364	34.405	20.232	13031	29.230	313.380	342.610	23.640	344.050	202.320
120	original	0.6	60x8 weeks	Ag80	MWF	VAC95	25	All	2.974	0.006	0.001	0.007	0.000	0.007	0.058	1427.704	3.011	0.467	3.478	0.173	3.235	27.807	14277	30.110	4.670	34.780	1.730	32.350	278.070
120	original	0.6	60x8 weeks	Ag80	MWF	VAC95	25	All	2.974	0.006	0.001	0.007	0.000	0.007	0.058	1427.704	3.011	0.467	3.478	0.173	3.235	27.807	14277	30.110	4.670	34.780	1.730	32.350	278.070
120	original	0.6	60x8 weeks	Ag80	MWF	VAC95	25	All	2.974	0.006	0.001	0.007	0.000	0.007	0.058	1427.704	3.011	0.467	3.478	0.173	3.235	27.807	14277	30.110	4.670	34.780	1.730	32.350	278.070
120	original	1	60x8 weeks	Ag80	MWF	VAC95	25	All	2.972	0.006	0.002	0.008	0.000	0.007	0.059	1426.796	2.941	0.777	3.718	0.172	3.502	28.137	14268	29.410	7.770	37.180	1.720	35.020	281.370
120	original	1	60x8 weeks	Ag80	MWF	VAC95	25	All	2.972	0.006	0.002	0.008	0.000	0.007	0.059	1426.796	2.941	0.777	3.718	0.172	3.502	28.137	14268	29.410	7.770	37.180	1.720	35.020	281.370
120	original	1	60x8 weeks	Ag80	MWF	VAC95	25	All	2.972	0.006	0.002	0.008	0.000	0.007	0.059	1426.796	2.941	0.777	3.718	0.172	3.502	28.137	14268	29.410	7.770	37.180	1.720	35.020	281.370
120	original	1.2	60x8 weeks	Ag80	MWF	VAC95	25	All	2.970	0.006	0.002	0.008	0.000	0.008	0.058	1425.630	2.970	1.094	4.064	0.166	3.814	27.836	14256	29.700	10.940	40.640	1.660	38.140	278.360
120	original	1.2	60x8 weeks	Ag80	MWF	VAC95	25	All	2.970	0.006	0.002	0.008	0.000	0.008	0.058	1425.630	2.970	1.094	4.064	0.166	3.814	27.836	14256	29.700	10.940	40.640	1.660	38.140	278.360
120	original	1.2	60x8 weeks	Ag80	MWF	VAC95	25	All	2.970	0.006	0.002	0.008	0.000	0.008	0.058	1425.630	2.970	1.094	4.064	0.166	3.814	27.836	14256	29.700	10.940	40.640	1.660	38.140	278.360
120	alpha	0.6	60x8 weeks	Ag80	MWF	VAC95	25	All	2.970	0.006	0.002	0.008	0.000	0.008	0.059	1425.570	2.982	0.823	3.805	0.160	3.835	28.114	14256	29.820	8.230	38.050	1.600	38.350	281.140
120	alpha	0.6	60x8 weeks	Ag80	MWF	VAC95	25	All	2.970	0.006	0.002	0.008	0.000	0.008	0.059	1425.570	2.982	0.823	3.805	0.160	3.835	28.114	14256	29.820	8.230	38.050	1.600	38.350	281.140
120	alpha	0.6	60x8 weeks	Ag80	MWF	VAC95	25	All	2.970	0.006	0.002	0.008	0.000	0.008	0.059	1425.570	2.982	0.823	3.805	0.160	3.835	28.114	14256	29.820	8.230	38.050	1.600	38.350	281.140
120	alpha	1	60x8 weeks	Ag80	MWF	VAC95	25	All	2.963	0.006	0.004	0.010	0.000	0.010	0.057	1422.260	2.967	1.731	4.698	0.209	4.728	27.467	14223	29.670	17.310	46.980	2.090	47.280	274.670
120	alpha	1	60x8 weeks	Ag80	MWF	VAC95	25	All	2.963	0.006	0.004	0.010	0.000	0.010	0.057	1422.260	2.967	1.731	4.698	0.209	4.728	27.467	14223	29.670	17.310	46.980	2.090	47.280	274.670
120	alpha	1	60x8 weeks	Ag80	MWF	VAC95	25	All	2.963	0.006	0.004	0.010	0.000	0.010	0.057	1422.260	2.967	1.731	4.698	0.209	4.728	27.467	14223	29.670	17.310	46.980	2.090	47.280	274.670
120	alpha	1.2	60x8 weeks	Ag80	MWF	VAC95	25	All	2.958	0.006	0.005	0.011	0.000	0.011	0.057	1419.899	2.930	2.383	5.313	0.239	5.328	27.315	14199	29.300	23.830	53.130	2.390	53.280	273.150
120	alpha	1.2	60x8 weeks	Ag80	MWF	VAC95	25	All	2.958	0.006	0.005	0.011	0.000	0.011	0.057	1419.899	2.930	2.383	5.313	0.239	5.328	27.315	14199	29.300	23.830	53.130	2.390	53.280	273.150
120	alpha	1.2	60x8 weeks	Ag80	MWF	VAC95	25	All	2.958	0.006	0.005	0.011	0.000	0.011	0.057	1419.899	2.930	2.383	5.313	0.239	5.328	27.315	14199	29.300	23.830	53.130	2.390	53.280	273.150
120	delta	0.6	60x8 weeks	Ag80	MWF	VAC95	25	All	2.956	0.006	0.005	0.011	0.001	0.011	0.057	1419.053	2.953	2.560	5.513	0.291	5.481	27.422	14191	29.530	25.600	55.130	2.910	54.810	274.220
120	delta	0.6	60x8 weeks	Ag80	MWF	VAC95	25	All	2.956	0.006	0.005	0.011	0.001	0.011	0.057	1419.053	2.953	2.560	5.513	0.291	5.481	27.422	14191	29.530	25.600	55.130	2.910	54.810	274.220
120	delta	0.6	60x8 weeks	Ag80	MWF	VAC95	25	All	2.956	0.006	0.005	0.011	0.001	0.011	0.057	1419.053	2.953	2.560	5.513	0.291	5.481	27.422	14191	29.530	25.600	55.130	2.910	54.810	274.220
120	delta	1	60x8 weeks	Ag80	MWF	VAC95	25	All	2.918	0.006	0.016	0.022	0.001	0.021	0.055	1400.642	2.860	7.543	10.403	0.696	10.296	26.470	14006	28.600	75.430	104.030	6.960	102.960	264.700
120	delta	1	60x8 weeks	Ag80	MWF	VAC95	25	All	2.918	0.006	0.016	0.022	0.001	0.021	0.055	1400.642	2.860	7.543	10.403	0.696	10.296	26.470	14006	28.600	75.430	104.030	6.960	102.960	264.700
120	delta	1	60x8 weeks	Ag80	MWF	VAC95	25	All	2.918	0.006	0.016	0.022	0.001	0.021	0.055	1400.642	2.860	7.543	10.403	0.696	10.296	26.470	14006	28.600	75.430	104.030	6.960	102.960	264.700
120	delta	1.2	60x8 weeks	Ag80	MWF	VAC95	25	All	2.878	0.006	0.026	0.032	0.002	0.031	0.053	1381.677	2.728	12.416	15.144	1.015	15.003	25.339	13817	27.280	124.160	151.440	10.150	150.030	253.390

120	delta	1.2	60x8 weeks	Ag80	MWF	VAC95	25	All	2.878	0.006	0.026	0.032	0.002	0.031	0.053	1381.677	2.728	12.416	15.144	1.015	15.003	25.339	13817	27.280	124.160	151.440	10.150	150.030	253.390
120	delta	1.2	60x8 weeks	Ag80	MWF	VAC95	25	All	2.878	0.006	0.026	0.032	0.002	0.031	0.053	1381.677	2.728	12.416	15.144	1.015	15.003	25.339	13817	27.280	124.160	151.440	10.150	150.030	253.390
120	original	0.6	60x8 weeks	Ag80	MWF	VAC95	50	All	2.984	0.004	0.000	0.005	0.000	0.004	0.058	1432.262	2.090	0.165	2.255	0.133	1.989	28.065	14323	20.900	1.650	22.550	1.330	19.890	280.650
120	original	0.6	60x8 weeks	Ag80	MWF	VAC95	50	All	2.984	0.004	0.000	0.005	0.000	0.004	0.058	1432.262	2.090	0.165	2.255	0.133	1.989	28.065	14323	20.900	1.650	22.550	1.330	19.890	280.650
120	original	0.6	60x8 weeks	Ag80	MWF	VAC95	50	All	2.984	0.004	0.000	0.005	0.000	0.004	0.058	1432.262	2.090	0.165	2.255	0.133	1.989	28.065	14323	20.900	1.650	22.550	1.330	19.890	280.650
120	original	1	60x8 weeks	Ag80	MWF	VAC95	50	All	2.983	0.004	0.001	0.005	0.000	0.005	0.059	1431.679	2.073	0.312	2.385	0.132	2.162	28.310	14317	20.730	3.120	23.850	1.320	21.620	283.100
120	original	1	60x8 weeks	Ag80	MWF	VAC95	50	All	2.983	0.004	0.001	0.005	0.000	0.005	0.059	1431.679	2.073	0.312	2.385	0.132	2.162	28.310	14317	20.730	3.120	23.850	1.320	21.620	283.100
120	original	1	60x8 weeks	Ag80	MWF	VAC95	50	All	2.983	0.004	0.001	0.005	0.000	0.005	0.059	1431.679	2.073	0.312	2.385	0.132	2.162	28.310	14317	20.730	3.120	23.850	1.320	21.620	283.100
120	original	1.2	60x8 weeks	Ag80	MWF	VAC95	50	All	2.981	0.004	0.001	0.005	0.000	0.005	0.059	1431.057	2.094	0.450	2.544	0.181	2.264	28.305	14311	20.940	4.500	25.440	1.810	22.640	283.050
120	original	1.2	60x8 weeks	Ag80	MWF	VAC95	50	All	2.981	0.004	0.001	0.005	0.000	0.005	0.059	1431.057	2.094	0.450	2.544	0.181	2.264	28.305	14311	20.940	4.500	25.440	1.810	22.640	283.050
120	original	1.2	60x8 weeks	Ag80	MWF	VAC95	50	All	2.981	0.004	0.001	0.005	0.000	0.005	0.059	1431.057	2.094	0.450	2.544	0.181	2.264	28.305	14311	20.940	4.500	25.440	1.810	22.640	283.050
120	alpha	0.6	60x8 weeks	Ag80	MWF	VAC95	50	All	2.980	0.004	0.001	0.005	0.000	0.005	0.059	1430.390	2.123	0.432	2.555	0.118	2.556	28.303	14304	21.230	4.320	25.550	1.180	25.560	283.030
120	alpha	0.6	60x8 weeks	Ag80	MWF	VAC95	50	All	2.980	0.004	0.001	0.005	0.000	0.005	0.059	1430.390	2.123	0.432	2.555	0.118	2.556	28.303	14304	21.230	4.320	25.550	1.180	25.560	283.030
120	alpha	0.6	60x8 weeks	Ag80	MWF	VAC95	50	All	2.980	0.004	0.001	0.005	0.000	0.005	0.059	1430.390	2.123	0.432	2.555	0.118	2.556	28.303	14304	21.230	4.320	25.550	1.180	25.560	283.030
120	alpha	1	60x8 weeks	Ag80	MWF	VAC95	50	All	2.979	0.004	0.001	0.006	0.000	0.006	0.058	1429.693	2.142	0.623	2.765	0.140	2.708	28.059	14297	21.420	6.230	27.650	1.400	27.080	280.590
120	alpha	1	60x8 weeks	Ag80	MWF	VAC95	50	All	2.979	0.004	0.001	0.006	0.000	0.006	0.058	1429.693	2.142	0.623	2.765	0.140	2.708	28.059	14297	21.420	6.230	27.650	1.400	27.080	280.590
120	alpha	1	60x8 weeks	Ag80	MWF	VAC95	50	All	2.979	0.004	0.001	0.006	0.000	0.006	0.058	1429.693	2.142	0.623	2.765	0.140	2.708	28.059	14297	21.420	6.230	27.650	1.400	27.080	280.590
120	alpha	1.2	60x8 weeks	Ag80	MWF	VAC95	50	All	2.977	0.004	0.002	0.006	0.000	0.006	0.058	1428.756	2.113	0.876	2.989	0.135	2.957	27.944	14288	21.130	8.760	29.890	1.350	29.570	279.440
120	alpha	1.2	60x8 weeks	Ag80	MWF	VAC95	50	All	2.977	0.004	0.002	0.006	0.000	0.006	0.058	1428.756	2.113	0.876	2.989	0.135	2.957	27.944	14288	21.130	8.760	29.890	1.350	29.570	279.440
120	alpha	1.2	60x8 weeks	Ag80	MWF	VAC95	50	All	2.977	0.004	0.002	0.006	0.000	0.006	0.058	1428.756	2.113	0.876	2.989	0.135	2.957	27.944	14288	21.130	8.760	29.890	1.350	29.570	279.440
120	delta	0.6	60x8 weeks	Ag80	MWF	VAC95	50	All	2.975	0.004	0.002	0.007	0.000	0.007	0.058	1427.796	2.133	1.075	3.208	0.178	3.200	27.962	14278	21.330	10.750	32.080	1.780	32.000	279.620
120	delta	0.6	60x8 weeks	Ag80	MWF	VAC95	50	All	2.975	0.004	0.002	0.007	0.000	0.007	0.058	1427.796	2.133	1.075	3.208	0.178	3.200	27.962	14278	21.330	10.750	32.080	1.780	32.000	279.620
120	delta	0.6	60x8 weeks	Ag80	MWF	VAC95	50	All	2.975	0.004	0.002	0.007	0.000	0.007	0.058	1427.796	2.133	1.075	3.208	0.178	3.200	27.962	14278	21.330	10.750	32.080	1.780	32.000	279.620
120	delta	1	60x8 weeks	Ag80	MWF	VAC95	50	All	2.963	0.005	0.005	0.010	0.001	0.010	0.058	1422.232	2.174	2.488	4.662	0.271	4.608	28.072	14222	21.740	24.880	46.620	2.710	46.080	280.720
120	delta	1	60x8 weeks	Ag80	MWF	VAC95	50	All	2.963	0.005	0.005	0.010	0.001	0.010	0.058	1422.232	2.174	2.488	4.662	0.271	4.608	28.072	14222	21.740	24.880	46.620	2.710	46.080	280.720
120	delta	1	60x8 weeks	Ag80	MWF	VAC95	50	All	2.963	0.005	0.005	0.010	0.001	0.010	0.058	1422.232	2.174	2.488	4.662	0.271	4.608	28.072	14222	21.740	24.880	46.620	2.710	46.080	280.720
120	delta	1.2	60x8 weeks	Ag80	MWF	VAC95	50	All	2.955	0.004	0.007	0.012	0.001	0.012	0.057	1418.282	2.132	3.558	5.690	0.384	5.597	27.462	14183	21.320	35.580	56.900	3.840	55.970	274.620
120	delta	1.2	60x8 weeks	Ag80	MWF	VAC95	50	All	2.955	0.004	0.007	0.012	0.001	0.012	0.057	1418.282	2.132	3.558	5.690	0.384	5.597	27.462	14183	21.320	35.580	56.900	3.840	55.970	274.620
120	delta	1.2	60x8 weeks	Ag80	MWF	VAC95	50	All	2.955	0.004	0.007	0.012	0.001	0.012	0.057	1418.282	2.132	3.558	5.690	0.384	5.597	27.462	14183	21.320	35.580	56.900	3.840	55.970	274.620
120	original	0.6	60x8 weeks	Ag80	MWF	VAC95	75	All	2.992	0.002	0.000	0.003	0.000	0.002	0.059	1435.970	1.148	0.058	1.206	0.103	0.994	28.354	14360	11.480	0.580	12.060	1.030	9.940	283.540
120	original	0.6	60x8 weeks	Ag80	MWF	VAC95	75	All	2.992	0.002	0.000	0.003	0.000	0.002	0.059	1435.970	1.148	0.058	1.206	0.103	0.994	28.354	14360	11.480	0.580	12.060	1.030	9.940	283.540
120	original	0.6	60x8 weeks	Ag80	MWF	VAC95	75	All	2.992	0.002	0.000	0.003	0.000	0.002	0.059	1435.970	1.148	0.058	1.206	0.103	0.994	28.354	14360	11.480	0.580	12.060	1.030	9.940	283.540
120	original	1	60x8 weeks	Ag80	MWF	VAC95	75	All	2.991	0.002	0.000	0.003	0.000	0.002	0.060	1435.793	1.194	0.081	1.275	0.109	1.064	28.660	14358	11.940	0.810	12.750	1.090	10.640	286.600
120	original	1	60x8 weeks	Ag80	MWF	VAC95	75	All	2.991	0.002	0.000	0.003	0.000	0.002	0.060	1435.793	1.194	0.081	1.275	0.109	1.064	28.660	14358	11.940	0.810	12.750	1.090	10.640	286.600
120	original	1	60x8 weeks	Ag80	MWF	VAC95	75	All	2.991	0.002	0.000	0.003	0.000	0.002	0.060	1435.793	1.194	0.081	1.275	0.109	1.064	28.660	14358	11.940	0.810	12.750	1.090	10.640	286.600
120	original	1.2	60x8 weeks	Ag80	MWF	VAC95	75	All	2.991	0.002	0.000	0.003	0.000	0.002	0.060	1435.778	1.180	0.138	1.318	0.127	1.053	28.746	14358	11.800	1.380	13.180	1.270	10.530	287.460
120	original	1.2	60x8 weeks	Ag80	MWF	VAC95	75	All	2.991	0.002	0.000	0.003	0.000	0.002	0.060	1435.778	1.180	0.138	1.318	0.127	1.053	28.746	14358	11.800	1.380	13.180	1.270	10.530	287.460
120	original	1.2	60x8 weeks	Ag80	MWF	VAC95	75	All	2.991	0.002	0.000	0.003	0.000	0.002	0.060	1435.778	1.180	0.138	1.318	0.127	1.053	28.746	14358	11.800	1.380	13.180	1.270	10.530	287.460
120	alpha	0.6	60x8 weeks	Ag80	MWF	VAC95	75	All	2.989	0.003	0.000	0.003	0.000	0.003	0.059	1434.693	1.293	0.124	1.417	0.050	1.425	28.553	14347	12.930	1.240	14.170	0.500	14.250	285.530
120	alpha	0.6	60x8 weeks	Ag80	MWF	VAC95	75	All	2.989	0.003	0.000	0.003	0.000	0.003	0.059	1434.693	1.293	0.124	1.417	0.050	1.425	28.553	14347	12.930	1.240	14.170	0.500	14.250	285.530
120	alpha	0.6	60x8 weeks	Ag80	MWF	VAC95	75	All	2.989	0.003	0.000	0.003	0.000	0.003	0.059	1434.693	1.293	0.124	1.417	0.050	1.425	28.553	14347	12.930	1.240	14.170	0.500	14.250	285.530
120	alpha	1	60x8 weeks	Ag80	MWF	VAC95	75	All	2.988	0.003	0.000	0.003	0.000	0.003	0.059	1434.403	1.278	0.197	1.475	0.071	1.477	28.480	14344	12.780	1.970	14.750	0.710	14.770	284.800
120	alpha	1	60x8 weeks	Ag80	MWF	VAC95	75	All	2.988	0.003	0.000	0.003	0.000	0.003	0.059	1434.403	1.278	0.197	1.475	0.071	1.477	28.480	14344	12.780	1.970	14.750	0.710	14.770	284.800
120	alpha	1	60x8 weeks	Ag80	MWF	VAC95	75	All	2.988	0.003	0.000	0.003	0.000	0.003	0.059	1434.403	1.278	0.197	1.475	0.071	1.477	28.480	14344	12.780	1.970	14.750	0.710	14.770	284.800
120	alpha	1.2	60x8 weeks	Ag80	MWF	VAC95	75	All	2.989	0.003	0.000	0.003	0.000	0.003	0.059	1434.515	1.207	0.224	1.431	0.056	1.449	28.183	14345	12.070	2.240	14.310	0.560	14.490	281.830
120	alpha	1.2	60x8 weeks	Ag80	MWF	VAC95	75	All	2.989	0.003	0.000	0.003	0.000	0.003	0.059	1434.515	1.207	0.224	1.431	0.056	1.449	28.183	14345	12.070	2.240	14.310	0.560	14.490	281.830
120	alpha	1.2	60x8 weeks	Ag80	MWF	VAC95	75	All	2.989	0.003	0.000	0.003	0.000	0.003	0.059	1434.515	1.207	0.224	1.431	0.056	1.449	28.183	14345	12.070	2.240	14.310	0.560	14.490	281.830
120	delta	0.6	60x8 weeks	Ag80	MWF	VAC95	75	All	2.987	0.003	0.001	0.003	0.000	0.003	0.059	1433.685	1.371	0.298	1.669	0.104	1.634	28.210	14337	13.710	2.980	16.690	1.040	16.340	282.100
120	delta	0.6	60x8 weeks	Ag80	MWF	VAC95	75	All	2.987	0.003	0.001	0.003	0.000	0.003	0.059	1433.685	1.371	0.298	1.669	0.104	1.634	28.210	14337	13.710	2.980	16.690	1.040	16.340	282.100
120	delta	0.6	60x8 weeks	Ag80	MWF	VAC95	75	All	2.987	0.003	0.001	0.003	0.000	0.003	0.059	1433.685	1.371	0.298	1.669	0.104	1.634	28.210	14337	13.710	2.980	16.690	1.040	16.340	282.100
120	delta	1	60x8 weeks	Ag80	MWF	VAC95	75	All	2.984	0.003	0.001	0.004	0.000	0.004	0.059	1432.537	1.403	0.587	1.990	0.110	1.942	28.314	14325	14.030	5.870	19.900	1.100	19.420	283.140
120	delta	1	60x8 weeks	Ag80	MWF	VAC95	75	All	2.984	0.003	0.001	0.004	0.000	0.004	0.059	1432.537	1.403	0.587	1.990	0.110	1.942	28.314	14325	14.030	5.870	19.900	1.100	19.420	283.140
120	delta	1	60x8 weeks	Ag80	MWF	VAC95	75	All	2.984	0.003	0.001	0.004	0.000	0.004	0.059	1432.537	1.403	0.587	1.990	0.110	1.942	28.314	14325	14.030	5.870	19.900	1.100	19.420	283.140
120	delta	1.2	60x8 weeks	Ag80	MWF	VAC95	75	All	2.983	0.003	0.002	0.004	0.000	0.004	0.059	1431.867	1.417	0.734	2.151	0.118	2.132	28.251	14319	14.170	7.340	21.510	1.180	21.320	282.510
120	delta	1.2	60x8 weeks	Ag80	MWF	VAC95	75	All	2.983	0.003	0.002	0.004	0.000	0.004	0.059	1431.867	1.417	0.734	2.151	0.118	2.132	28.251	14319	14.170	7.340	21.510	1.180	21.320	282.510
120	delta	1.2	60x8 weeks	Ag80	MWF	VAC95	75	All	2.983	0.003	0.002	0.004	0.000	0.004	0.059	1431.867	1.417	0.734	2.151	0.118	2.132	28.251	14319	14.170	7.340	21.510	1.180	21.320	282.510
120	original	0.6	60x8 weeks	Ag80	MWF	VAC95	100	All	2.999	0.001	0.000	0.001	0.000	0.000	0.060	1439.519	0.292	0.000	0.292	0.093	0.034	28.911	14395	2.920	0.000	2.920	0.930	0.340	289.110
120	original	0.6	60x8 weeks	Ag80	MWF	VAC95	100	All	2.999	0.001	0.000	0.001	0.000	0.000	0.060	1439.519	0.292	0.000	0.292	0.093	0.034	28.911	14395	2.920	0.000	2.920	0.930	0.340	289.110
120	original	0.6	60x8 weeks	Ag80	MWF	VAC95	100	All	2.999	0.001	0.000	0.001	0.000	0.000	0.060	1439.519	0.292	0.000	0.292	0.093	0.034	28.911	14395	2.920	0.000	2.920	0.930	0.340	289.110
120	original	1	60x8 weeks	Ag80	MWF	VAC95	100	All	2.999	0.001	0.000	0.001	0.000	0.000	0.060	1439.452	0.256	0.001	0.257	0.097	0.042	28.798	14395	2.560	0.010	2.570	0.970	0.420	287.980
120	original	1	60x8 weeks	Ag80	MWF	VAC95	100	All	2.999	0.001	0.000	0.001	0.000	0.000	0.060	1439.452	0.256	0.001	0.257	0.097	0.042	28.798	14395	2.560	0.010	2.570	0.970	0.420	287.980
120	original	1	60x8 weeks	Ag80	MWF	VAC95	100	All	2.999	0.001	0.000	0.001	0.000	0.000	0.060	1439.452	0.256	0.001	0.257	0.097	0.042	28.798	14395	2.560	0.010	2.570	0.970	0.420	287.980
120	original	1.2	60x8 weeks	Ag80	MWF	VAC95	100	All	2.999	0.001	0.000	0.001	0.000	0.000	0.060	1439.536	0.287	0.001	0.288	0.082	0.036	28.751	14395	2.870	0.010	2.880	0.820	0.360	287.510
120	original	1.2	60x8 weeks	Ag80	MWF	VAC95	100	All	2.999	0.001	0.000	0.001	0.000	0.000	0.060	1439.536	0.287	0.001	0.288	0.082	0.036	28.751	14395	2.870	0.010	2.880	0.820	0.360	287.510
120	original	1.2	60x8 weeks	Ag80	MWF	VAC95	100	All	2.999	0.001	0.000	0.001	0.000	0.000	0.060	1439.536	0.287	0.001	0.288	0.082	0.036	28.751	14395	2.870	0.010	2.880	0.820	0.360	287.510
120	alpha	0.6	60x8 weeks	Ag80	MWF	VAC95	100	All	2.997	0.001	0.000	0.001	0.000	0.001	0.059	1438.745	0.352	0.009	0.361	0.021	0.332	28.342	14387	3.520	0.090	3.610	0.210	3.320	283.420
120	alpha	0.6	60x8 weeks	Ag80	MWF	VAC95	100	All	2.997	0.001	0.000	0.001	0.000	0.001	0.059	1438.745	0.352	0.009	0.361	0.021	0.332	28.342	14387	3.520	0.090	3.610	0.210	3.320	283.420
120	alpha	0.6	60x8 weeks	Ag80	MWF	VAC95	100	All	2.997	0.001	0.000	0.001	0.000	0.001	0.059	1438.745	0.352	0.009	0.361	0.021	0.332	28.342	14387	3.520	0.090	3.610	0.210	3.320	283.420
120	alpha	1	60x8 weeks	Ag80	MWF	VAC95	100	All	2.997	0.001	0.000	0.001	0.000	0.001	0.060	1438.669	0.363	0.010	0.373	0.014	0.353	28.643	14387	3.630	0.100	3.730	0.140	3.530	286.430
120	alpha	1	60x8 weeks	Ag80	MWF	VAC95	100	All	2.997	0.001	0.000	0.001	0.000	0.001	0.060	1438.669	0.363	0.010	0.373	0.014	0.353	28.643	14387	3.630	0.100	3.730	0.140	3.530	286.430
120	alpha	1	60x8 weeks	Ag80	MWF	VAC95	100	All	2.997	0.001	0.000	0.001	0.000	0.001	0.060	1438.669	0.363	0.010	0.373	0.014	0.353	28.643	14387	3.630	0.100	3.730	0.140	3.530	286.430

120	alpha	1.2	60x8 weeks	Ag80	MWF	VAC95	100	All	2.997	0.001	0.000	0.001	0.000	0.001	0.060	1438.694	0.355	0.008	0.363	0.030	0.338	28.739	14387	3.550	0.080	3.630	0.300	3.380	287.390
120	alpha	1.2	60x8 weeks	Ag80	MWF	VAC95	100	All	2.997	0.001	0.000	0.001	0.000	0.001	0.060	1438.694	0.355	0.008	0.363	0.030	0.338	28.739	14387	3.550	0.080	3.630	0.300	3.380	287.390
120	alpha	1.2	60x8 weeks	Ag80	MWF	VAC95	100	All	2.997	0.001	0.000	0.001	0.000	0.001	0.060	1438.694	0.355	0.008	0.363	0.030	0.338	28.739	14387	3.550	0.080	3.630	0.300	3.380	287.390
120	delta	0.6	60x8 weeks	Ag80	MWF	VAC95	100	All	2.996	0.001	0.000	0.001	0.000	0.001	0.060	1437.864	0.569	0.025	0.594	0.028	0.570	28.915	14379	5.690	0.250	5.940	0.280	5.700	289.150
120	delta	0.6	60x8 weeks	Ag80	MWF	VAC95	100	All	2.996	0.001	0.000	0.001	0.000	0.001	0.060	1437.864	0.569	0.025	0.594	0.028	0.570	28.915	14379	5.690	0.250	5.940	0.280	5.700	289.150
120	delta	0.6	60x8 weeks	Ag80	MWF	VAC95	100	All	2.996	0.001	0.000	0.001	0.000	0.001	0.060	1437.864	0.569	0.025	0.594	0.028	0.570	28.915	14379	5.690	0.250	5.940	0.280	5.700	289.150
120	delta	1	60x8 weeks	Ag80	MWF	VAC95	100	All	2.995	0.001	0.000	0.001	0.000	0.001	0.060	1437.566	0.616	0.055	0.671	0.031	0.652	28.668	14376	6.160	0.550	6.710	0.310	6.520	286.680
120	delta	1	60x8 weeks	Ag80	MWF	VAC95	100	All	2.995	0.001	0.000	0.001	0.000	0.001	0.060	1437.566	0.616	0.055	0.671	0.031	0.652	28.668	14376	6.160	0.550	6.710	0.310	6.520	286.680
120	delta	1	60x8 weeks	Ag80	MWF	VAC95	100	All	2.995	0.001	0.000	0.001	0.000	0.001	0.060	1437.566	0.616	0.055	0.671	0.031	0.652	28.668	14376	6.160	0.550	6.710	0.310	6.520	286.680
120	delta	1.2	60x8 weeks	Ag80	MWF	VAC95	100	All	2.995	0.001	0.000	0.001	0.000	0.001	0.060	1437.497	0.592	0.083	0.675	0.041	0.653	28.707	14375	5.920	0.830	6.750	0.410	6.530	287.070
120	delta	1.2	60x8 weeks	Ag80	MWF	VAC95	100	All	2.995	0.001	0.000	0.001	0.000	0.001	0.060	1437.497	0.592	0.083	0.675	0.041	0.653	28.707	14375	5.920	0.830	6.750	0.410	6.530	287.070
120	delta	1.2	60x8 weeks	Ag80	MWF	VAC95	100	All	2.995	0.001	0.000	0.001	0.000	0.001	0.060	1437.497	0.592	0.083	0.675	0.041	0.653	28.707	14375	5.920	0.830	6.750	0.410	6.530	287.070
120	original	0.6	60x8 weeks	Ag80	MWF	VAC70	25	All	2.973	0.007	0.001	0.008	0.001	0.007	0.059	1426.942	3.325	0.524	3.849	0.274	3.353	28.148	14269	33.250	5.240	38.490	2.740	33.530	281.480
120	original	0.6	60x8 weeks	Ag80	MWF	VAC70	25	All	2.973	0.007	0.001	0.008	0.001	0.007	0.059	1426.942	3.325	0.524	3.849	0.274	3.353	28.148	14269	33.250	5.240	38.490	2.740	33.530	281.480
120	original	0.6	60x8 weeks	Ag80	MWF	VAC70	25	All	2.973	0.007	0.001	0.008	0.001	0.007	0.059	1426.942	3.325	0.524	3.849	0.274	3.353	28.148	14269	33.250	5.240	38.490	2.740	33.530	281.480
120	original	1	60x8 weeks	Ag80	MWF	VAC70	25	All	2.968	0.007	0.002	0.009	0.001	0.008	0.058	1424.873	3.338	0.954	4.292	0.365	3.783	27.650	14249	33.380	9.540	42.920	3.650	37.830	276.500
120	original	1	60x8 weeks	Ag80	MWF	VAC70	25	All	2.968	0.007	0.002	0.009	0.001	0.008	0.058	1424.873	3.338	0.954	4.292	0.365	3.783	27.650	14249	33.380	9.540	42.920	3.650	37.830	276.500
120	original	1	60x8 weeks	Ag80	MWF	VAC70	25	All	2.968	0.007	0.002	0.009	0.001	0.008	0.058	1424.873	3.338	0.954	4.292	0.365	3.783	27.650	14249	33.380	9.540	42.920	3.650	37.830	276.500
120	original	1.2	60x8 weeks	Ag80	MWF	VAC70	25	All	2.969	0.007	0.002	0.009	0.001	0.008	0.058	1425.040	3.206	1.171	4.377	0.343	3.824	27.701	14250	32.060	11.710	43.770	3.430	38.240	277.010
120	original	1.2	60x8 weeks	Ag80	MWF	VAC70	25	All	2.969	0.007	0.002	0.009	0.001	0.008	0.058	1425.040	3.206	1.171	4.377	0.343	3.824	27.701	14250	32.060	11.710	43.770	3.430	38.240	277.010
120	original	1.2	60x8 weeks	Ag80	MWF	VAC70	25	All	2.969	0.007	0.002	0.009	0.001	0.008	0.058	1425.040	3.206	1.171	4.377	0.343	3.824	27.701	14250	32.060	11.710	43.770	3.430	38.240	277.010
120	alpha	0.6	60x8 weeks	Ag80	MWF	VAC70	25	All	2.968	0.007	0.002	0.009	0.000	0.009	0.058	1424.449	3.138	1.004	4.142	0.201	4.121	27.932	14244	31.380	10.040	41.420	2.010	41.210	279.320
120	alpha	0.6	60x8 weeks	Ag80	MWF	VAC70	25	All	2.968	0.007	0.002	0.009	0.000	0.009	0.058	1424.449	3.138	1.004	4.142	0.201	4.121	27.932	14244	31.380	10.040	41.420	2.010	41.210	279.320
120	alpha	0.6	60x8 weeks	Ag80	MWF	VAC70	25	All	2.968	0.007	0.002	0.009	0.000	0.009	0.058	1424.449	3.138	1.004	4.142	0.201	4.121	27.932	14244	31.380	10.040	41.420	2.010	41.210	279.320
120	alpha	1	60x8 weeks	Ag80	MWF	VAC70	25	All	2.959	0.007	0.004	0.011	0.000	0.011	0.058	1420.093	3.175	2.082	5.257	0.227	5.282	27.710	14201	31.750	20.820	52.570	2.270	52.820	277.100
120	alpha	1	60x8 weeks	Ag80	MWF	VAC70	25	All	2.959	0.007	0.004	0.011	0.000	0.011	0.058	1420.093	3.175	2.082	5.257	0.227	5.282	27.710	14201	31.750	20.820	52.570	2.270	52.820	277.100
120	alpha	1	60x8 weeks	Ag80	MWF	VAC70	25	All	2.959	0.007	0.004	0.011	0.000	0.011	0.058	1420.093	3.175	2.082	5.257	0.227	5.282	27.710	14201	31.750	20.820	52.570	2.270	52.820	277.100
120	alpha	1.2	60x8 weeks	Ag80	MWF	VAC70	25	All	2.955	0.007	0.005	0.012	0.001	0.012	0.057	1418.351	3.202	2.590	5.792	0.266	5.757	27.321	14184	32.020	25.900	57.920	2.660	57.570	273.210
120	alpha	1.2	60x8 weeks	Ag80	MWF	VAC70	25	All	2.955	0.007	0.005	0.012	0.001	0.012	0.057	1418.351	3.202	2.590	5.792	0.266	5.757	27.321	14184	32.020	25.900	57.920	2.660	57.570	273.210
120	alpha	1.2	60x8 weeks	Ag80	MWF	VAC70	25	All	2.955	0.007	0.005	0.012	0.001	0.012	0.057	1418.351	3.202	2.590	5.792	0.266	5.757	27.321	14184	32.020	25.900	57.920	2.660	57.570	273.210
120	delta	0.6	60x8 weeks	Ag80	MWF	VAC70	25	All	2.948	0.007	0.007	0.014	0.001	0.013	0.057	1415.161	3.243	3.302	6.545	0.454	6.421	27.128	14152	32.430	33.020	65.450	4.540	64.210	271.280
120	delta	0.6	60x8 weeks	Ag80	MWF	VAC70	25	All	2.948	0.007	0.007	0.014	0.001	0.013	0.057	1415.161	3.243	3.302	6.545	0.454	6.421	27.128	14152	32.430	33.020	65.450	4.540	64.210	271.280
120	delta	0.6	60x8 weeks	Ag80	MWF	VAC70	25	All	2.948	0.007	0.007	0.014	0.001	0.013	0.057	1415.161	3.243	3.302	6.545	0.454	6.421	27.128	14152	32.430	33.020	65.450	4.540	64.210	271.280
120	delta	1	60x8 weeks	Ag80	MWF	VAC70	25	All	2.899	0.006	0.020	0.026	0.002	0.026	0.054	1391.520	3.071	9.573	12.644	0.826	12.502	25.844	13915	30.710	95.730	126.440	8.260	125.020	258.440
120	delta	1	60x8 weeks	Ag80	MWF	VAC70	25	All	2.899	0.006	0.020	0.026	0.002	0.026	0.054	1391.520	3.071	9.573	12.644	0.826	12.502	25.844	13915	30.710	95.730	126.440	8.260	125.020	258.440
120	delta	1	60x8 weeks	Ag80	MWF	VAC70	25	All	2.899	0.006	0.020	0.026	0.002	0.026	0.054	1391.520	3.071	9.573	12.644	0.826	12.502	25.844	13915	30.710	95.730	126.440	8.260	125.020	258.440
120	delta	1.2	60x8 weeks	Ag80	MWF	VAC70	25	All	2.852	0.006	0.033	0.039	0.003	0.038	0.050	1368.743	2.864	15.626	18.490	1.261	18.308	24.227	13687	28.640	156.260	184.900	12.610	183.080	242.270
120	delta	1.2	60x8 weeks	Ag80	MWF	VAC70	25	All	2.852	0.006	0.033	0.039	0.003	0.038	0.050	1368.743	2.864	15.626	18.490	1.261	18.308	24.227	13687	28.640	156.260	184.900	12.610	183.080	242.270
120	delta	1.2	60x8 weeks	Ag80	MWF	VAC70	25	All	2.852	0.006	0.033	0.039	0.003	0.038	0.050	1368.743	2.864	15.626	18.490	1.261	18.308	24.227	13687	28.640	156.260	184.900	12.610	183.080	242.270
120	original	0.6	60x8 weeks	Ag80	MWF	VAC70	50	All	2.981	0.005	0.001	0.006	0.001	0.004	0.059	1431.081	2.528	0.248	2.776	0.383	2.071	28.245	14311	25.280	2.480	27.760	3.830	20.710	282.450
120	original	0.6	60x8 weeks	Ag80	MWF	VAC70	50	All	2.981	0.005	0.001	0.006	0.001	0.004	0.059	1431.081	2.528	0.248	2.776	0.383	2.071	28.245	14311	25.280	2.480	27.760	3.830	20.710	282.450
120	original	0.6	60x8 weeks	Ag80	MWF	VAC70	50	All	2.981	0.005	0.001	0.006	0.001	0.004	0.059	1431.081	2.528	0.248	2.776	0.383	2.071	28.245	14311	25.280	2.480	27.760	3.830	20.710	282.450
120	original	1	60x8 weeks	Ag80	MWF	VAC70	50	All	2.980	0.005	0.001	0.006	0.001	0.005	0.058	1430.172	2.581	0.449	3.030	0.406	2.273	27.993	14302	25.810	4.490	30.300	4.060	22.730	279.930
120	original	1	60x8 weeks	Ag80	MWF	VAC70	50	All	2.980	0.005	0.001	0.006	0.001	0.005	0.058	1430.172	2.581	0.449	3.030	0.406	2.273	27.993	14302	25.810	4.490	30.300	4.060	22.730	279.930
120	original	1	60x8 weeks	Ag80	MWF	VAC70	50	All	2.980	0.005	0.001	0.006	0.001	0.005	0.058	1430.172	2.581	0.449	3.030	0.406	2.273	27.993	14302	25.810	4.490	30.300	4.060	22.730	279.930
120	original	1.2	60x8 weeks	Ag80	MWF	VAC70	50	All	2.979	0.005	0.001	0.007	0.001	0.005	0.058	1429.765	2.610	0.557	3.167	0.425	2.372	27.970	14298	26.100	5.570	31.670	4.250	23.720	279.700
120	original	1.2	60x8 weeks	Ag80	MWF	VAC70	50	All	2.979	0.005	0.001	0.007	0.001	0.005	0.058	1429.765	2.610	0.557	3.167	0.425	2.372	27.970	14298	26.100	5.570	31.670	4.250	23.720	279.700
120	original	1.2	60x8 weeks	Ag80	MWF	VAC70	50	All	2.979	0.005	0.001	0.007	0.001	0.005	0.058	1429.765	2.610	0.557	3.167	0.425	2.372	27.970	14298	26.100	5.570	31.670	4.250	23.720	279.700
120	alpha	0.6	60x8 weeks	Ag80	MWF	VAC70	50	All	2.976	0.005	0.001	0.006	0.000	0.007	0.058	1428.287	2.601	0.492	3.093	0.116	3.132	27.963	14283	26.010	4.920	30.930	1.160	31.320	279.630
120	alpha	0.6	60x8 weeks	Ag80	MWF	VAC70	50	All	2.976	0.005	0.001	0.006	0.000	0.007	0.058	1428.287	2.601	0.492	3.093	0.116	3.132	27.963	14283	26.010	4.920	30.930	1.160	31.320	279.630
120	alpha	0.6	60x8 weeks	Ag80	MWF	VAC70	50	All	2.976	0.005	0.001	0.006	0.000	0.007	0.058	1428.287	2.601	0.492	3.093	0.116	3.132	27.963	14283	26.010	4.920	30.930	1.160	31.320	279.630
120	alpha	1	60x8 weeks	Ag80	MWF	VAC70	50	All	2.973	0.005	0.002	0.007	0.000	0.007	0.058	1426.802	2.591	0.998	3.589	0.162	3.535	27.845	14268	25.910	9.980	35.890	1.620	35.350	278.450
120	alpha	1	60x8 weeks	Ag80	MWF	VAC70	50	All	2.973	0.005	0.002	0.007	0.000	0.007	0.058	1426.802	2.591	0.998	3.589	0.162	3.535	27.845	14268	25.910	9.980	35.890	1.620	35.350	278.450
120	alpha	1	60x8 weeks	Ag80	MWF	VAC70	50	All	2.973	0.005	0.002	0.007	0.000	0.007	0.058	1426.802	2.591	0.998	3.589	0.162	3.535	27.845	14268	25.910	9.980	35.890	1.620	35.350	278.450
120	alpha	1.2	60x8 weeks	Ag80	MWF	VAC70	50	All	2.969	0.005	0.003	0.008	0.000	0.008	0.058	1424.991	2.597	1.335	3.932	0.192	3.940	27.883	14250	25.970	13.350	39.320	1.920	39.400	278.830
120	alpha	1.2	60x8 weeks	Ag80	MWF	VAC70	50	All	2.969	0.005	0.003	0.008	0.000	0.008	0.058	1424.991	2.597	1.335	3.932	0.192	3.940	27.883	14250	25.970	13.350	39.320	1.920	39.400	278.830
120	alpha	1.2	60x8 weeks	Ag80	MWF	VAC70	50	All	2.969	0.005	0.003	0.008	0.000	0.008	0.058	1424.991	2.597	1.335	3.932	0.192	3.940	27.883	14250	25.970	13.350	39.320	1.920	39.400	278.830
120	delta	0.6	60x8 weeks	Ag80	MWF	VAC70	50	All	2.964	0.006	0.004	0.010	0.001	0.009	0.058	1422.799	2.769	1.802	4.571	0.280	4.487	27.732	14228	27.690	18.020	45.710	2.800	44.870	277.320
120	delta	0.6	60x8 weeks	Ag80	MWF	VAC70	50	All	2.964	0.006	0.004	0.010	0.001	0.009	0.058	1422.799	2.769	1.802	4.571	0.280	4.487	27.732	14228	27.690	18.020	45.710	2.800	44.870	277.320
120	delta	0.6	60x8 weeks	Ag80	MWF	VAC70	50	All	2.964	0.006	0.004	0.010	0.001	0.009	0.058	1422.799	2.769	1.802	4.571	0.280	4.487	27.732	14228	27.690	18.020	45.710	2.800	44.870	277.320
120	delta	1	60x8 weeks	Ag80	MWF	VAC70	50	All	2.947	0.005	0.009	0.014	0.001	0.014	0.056	1414.332	2.594	4.136	6.730	0.447	6.572	27.076	14143	25.940	41.360	67.300	4.470	65.720	270.760
120	delta	1	60x8 weeks	Ag80	MWF	VAC70	50	All	2.947	0.005	0.009	0.014	0.001	0.014	0.056	1414.332	2.594	4.136	6.730	0.447	6.572	27.076	14143	25.940	41.360	67.300	4.470	65.720	270.760
120	delta	1	60x8 weeks	Ag80	MWF	VAC70	50	All	2.947	0.005	0.009	0.014	0.001	0.014	0.056	1414.332	2.594	4.136	6.730	0.447	6.572	27.076	14143	25.940	41.360	67.300	4.470	65.720	270.760
120	delta	1.2	60x8 weeks	Ag80	MWF	VAC70	50	All	2.930	0.005	0.013	0.018	0.001	0.018	0.056	1406.400	2.498	6.197	8.695	0.552	8.628	26.955	14064	24.980	61.970	86.950	5.520	86.280	269.550
120	delta	1.2	60x8 weeks	Ag80	MWF	VAC70	50	All	2.930	0.005	0.013	0.018	0.001	0.018	0.056	1406.400	2.498	6.197	8.695	0.552	8.628	26.955	14064	24.980	61.970	86.950	5.520	86.280	269.550
120	delta	1.2	60x8 weeks	Ag80	MWF	VAC70	50	All	2.930	0.005	0.013	0.018	0.001	0.018	0.056	1406.400	2.498	6.197	8.695	0.552	8.628	26.955	14064	24.980	61.970	86.950	5.520	86.280	269.550
120	original	0.6	60x8 weeks	Ag80	MWF	VAC70	75	All	2.988	0.004	0.000	0.004	0.001	0.002	0.059	1434.043	1.993	0.091	2.084	0.475	1.126	28.337	14340	19.930	0.910	20.840	4.750	11.260	283.370
120	original	0.6	60x8 weeks	Ag80	MWF	VAC70	75	All	2.988	0.004	0.000	0.004	0.001	0.002	0.059	1434.043	1.993	0.091	2.084	0.475	1.126	28.337	14340	19.930	0.910	20.840	4.750	11.260	283.370
120	original	0.6	60x8 weeks	Ag80	MWF	VAC70	75	All	2.988	0.004	0.000	0.004	0.001	0.002	0.059	1434.043	1.993	0.091	2.084	0.475	1.126	28.337	14340	19.930	0.910	20.840	4.750	11.260	283.370
120	original	1	60x8 weeks	Ag80	MWF	VAC70	75	All	2.987	0.004	0.000	0.004	0.001	0.002	0.059	1433.957	1.974	0.150	2.124	0.494	1.132	28.124	14340	19.740	1.500	21.240	4.940	11.320	281.240
120	original	1	60x8 weeks	Ag80	MWF	VAC70	75	All	2.987	0.004	0.000	0.004	0.001	0.002	0.059	1433.957	1.974	0.150	2.124	0.494	1.132	28.124	14340	19.740	1.500	21.240	4.940	11.320	281.240

120	original	1	60x8 weeks	Ag80	MWF	VAC70	75	All	2.987	0.004	0.000	0.004	0.001	0.002	0.059	1433.957	1.974	0.150	2.124	0.494	1.132	28.124	14340	19.740	1.500	21.240	4.940	11.320	281.240
120	original	1.2	60x8 weeks	Ag80	MWF	VAC70	75	All	2.988	0.004	0.000	0.004	0.001	0.002	0.059	1434.045	1.921	0.194	2.115	0.490	1.128	28.486	14340	19.210	1.940	21.150	4.900	11.280	284.860
120	original	1.2	60x8 weeks	Ag80	MWF	VAC70	75	All	2.988	0.004	0.000	0.004	0.001	0.002	0.059	1434.045	1.921	0.194	2.115	0.490	1.128	28.486	14340	19.210	1.940	21.150	4.900	11.280	284.860
120	original	1.2	60x8 weeks	Ag80	MWF	VAC70	75	All	2.988	0.004	0.000	0.004	0.001	0.002	0.059	1434.045	1.921	0.194	2.115	0.490	1.128	28.486	14340	19.210	1.940	21.150	4.900	11.280	284.860
120	alpha	0.6	60x8 weeks	Ag80	MWF	VAC70	75	All	2.983	0.004	0.001	0.005	0.000	0.004	0.059	1431.826	1.896	0.272	2.168	0.109	2.145	28.103	14318	18.960	2.720	21.680	1.090	21.450	281.030
120	alpha	0.6	60x8 weeks	Ag80	MWF	VAC70	75	All	2.983	0.004	0.001	0.005	0.000	0.004	0.059	1431.826	1.896	0.272	2.168	0.109	2.145	28.103	14318	18.960	2.720	21.680	1.090	21.450	281.030
120	alpha	0.6	60x8 weeks	Ag80	MWF	VAC70	75	All	2.983	0.004	0.001	0.005	0.000	0.004	0.059	1431.826	1.896	0.272	2.168	0.109	2.145	28.103	14318	18.960	2.720	21.680	1.090	21.450	281.030
120	alpha	1	60x8 weeks	Ag80	MWF	VAC70	75	All	2.982	0.004	0.001	0.005	0.000	0.005	0.059	1431.559	1.890	0.395	2.285	0.132	2.210	28.382	14316	18.900	3.950	22.850	1.320	22.100	283.820
120	alpha	1	60x8 weeks	Ag80	MWF	VAC70	75	All	2.982	0.004	0.001	0.005	0.000	0.005	0.059	1431.559	1.890	0.395	2.285	0.132	2.210	28.382	14316	18.900	3.950	22.850	1.320	22.100	283.820
120	alpha	1	60x8 weeks	Ag80	MWF	VAC70	75	All	2.982	0.004	0.001	0.005	0.000	0.005	0.059	1431.559	1.890	0.395	2.285	0.132	2.210	28.382	14316	18.900	3.950	22.850	1.320	22.100	283.820
120	alpha	1.2	60x8 weeks	Ag80	MWF	VAC70	75	All	2.980	0.004	0.001	0.005	0.000	0.005	0.059	1430.537	1.921	0.600	2.521	0.117	2.492	28.184	14305	19.210	6.000	25.210	1.170	24.920	281.840
120	alpha	1.2	60x8 weeks	Ag80	MWF	VAC70	75	All	2.980	0.004	0.001	0.005	0.000	0.005	0.059	1430.537	1.921	0.600	2.521	0.117	2.492	28.184	14305	19.210	6.000	25.210	1.170	24.920	281.840
120	alpha	1.2	60x8 weeks	Ag80	MWF	VAC70	75	All	2.980	0.004	0.001	0.005	0.000	0.005	0.059	1430.537	1.921	0.600	2.521	0.117	2.492	28.184	14305	19.210	6.000	25.210	1.170	24.920	281.840
120	delta	0.6	60x8 weeks	Ag80	MWF	VAC70	75	All	2.977	0.005	0.001	0.006	0.000	0.006	0.058	1428.939	2.223	0.715	2.938	0.185	2.866	27.988	14289	22.230	7.150	29.380	1.850	28.660	279.880
120	delta	0.6	60x8 weeks	Ag80	MWF	VAC70	75	All	2.977	0.005	0.001	0.006	0.000	0.006	0.058	1428.939	2.223	0.715	2.938	0.185	2.866	27.988	14289	22.230	7.150	29.380	1.850	28.660	279.880
120	delta	0.6	60x8 weeks	Ag80	MWF	VAC70	75	All	2.977	0.005	0.001	0.006	0.000	0.006	0.058	1428.939	2.223	0.715	2.938	0.185	2.866	27.988	14289	22.230	7.150	29.380	1.850	28.660	279.880
120	delta	1	60x8 weeks	Ag80	MWF	VAC70	75	All	2.971	0.005	0.003	0.008	0.000	0.008	0.058	1426.006	2.226	1.556	3.782	0.235	3.673	27.884	14260	22.260	15.560	37.820	2.350	36.730	278.840
120	delta	1	60x8 weeks	Ag80	MWF	VAC70	75	All	2.971	0.005	0.003	0.008	0.000	0.008	0.058	1426.006	2.226	1.556	3.782	0.235	3.673	27.884	14260	22.260	15.560	37.820	2.350	36.730	278.840
120	delta	1	60x8 weeks	Ag80	MWF	VAC70	75	All	2.971	0.005	0.003	0.008	0.000	0.008	0.058	1426.006	2.226	1.556	3.782	0.235	3.673	27.884	14260	22.260	15.560	37.820	2.350	36.730	278.840
120	delta	1.2	60x8 weeks	Ag80	MWF	VAC70	75	All	2.966	0.004	0.004	0.009	0.001	0.009	0.058	1423.710	2.128	2.158	4.286	0.262	4.218	28.067	14237	21.280	21.580	42.860	2.620	42.180	280.670
120	delta	1.2	60x8 weeks	Ag80	MWF	VAC70	75	All	2.966	0.004	0.004	0.009	0.001	0.009	0.058	1423.710	2.128	2.158	4.286	0.262	4.218	28.067	14237	21.280	21.580	42.860	2.620	42.180	280.670
120	delta	1.2	60x8 weeks	Ag80	MWF	VAC70	75	All	2.966	0.004	0.004	0.009	0.001	0.009	0.058	1423.710	2.128	2.158	4.286	0.262	4.218	28.067	14237	21.280	21.580	42.860	2.620	42.180	280.670
120	original	0.6	60x8 weeks	Ag80	MWF	VAC70	100	All	2.995	0.003	0.000	0.003	0.001	0.000	0.059	1437.424	1.251	0.003	1.254	0.523	0.147	28.557	14374	12.510	0.030	12.540	5.230	1.470	285.570
120	original	0.6	60x8 weeks	Ag80	MWF	VAC70	100	All	2.995	0.003	0.000	0.003	0.001	0.000	0.059	1437.424	1.251	0.003	1.254	0.523	0.147	28.557	14374	12.510	0.030	12.540	5.230	1.470	285.570
120	original	0.6	60x8 weeks	Ag80	MWF	VAC70	100	All	2.995	0.003	0.000	0.003	0.001	0.000	0.059	1437.424	1.251	0.003	1.254	0.523	0.147	28.557	14374	12.510	0.030	12.540	5.230	1.470	285.570
120	original	1	60x8 weeks	Ag80	MWF	VAC70	100	All	2.994	0.003	0.000	0.003	0.001	0.000	0.060	1437.357	1.329	0.020	1.349	0.546	0.154	28.805	14374	13.290	0.200	13.490	5.460	1.540	288.050
120	original	1	60x8 weeks	Ag80	MWF	VAC70	100	All	2.994	0.003	0.000	0.003	0.001	0.000	0.060	1437.357	1.329	0.020	1.349	0.546	0.154	28.805	14374	13.290	0.200	13.490	5.460	1.540	288.050
120	original	1	60x8 weeks	Ag80	MWF	VAC70	100	All	2.994	0.003	0.000	0.003	0.001	0.000	0.060	1437.357	1.329	0.020	1.349	0.546	0.154	28.805	14374	13.290	0.200	13.490	5.460	1.540	288.050
120	original	1.2	60x8 weeks	Ag80	MWF	VAC70	100	All	2.994	0.003	0.000	0.003	0.001	0.000	0.059	1437.201	1.289	0.025	1.314	0.566	0.157	28.456	14372	12.890	0.250	13.140	5.660	1.570	284.560
120	original	1.2	60x8 weeks	Ag80	MWF	VAC70	100	All	2.994	0.003	0.000	0.003	0.001	0.000	0.059	1437.201	1.289	0.025	1.314	0.566	0.157	28.456	14372	12.890	0.250	13.140	5.660	1.570	284.560
120	original	1.2	60x8 weeks	Ag80	MWF	VAC70	100	All	2.994	0.003	0.000	0.003	0.001	0.000	0.059	1437.201	1.289	0.025	1.314	0.566	0.157	28.456	14372	12.890	0.250	13.140	5.660	1.570	284.560
120	alpha	0.6	60x8 weeks	Ag80	MWF	VAC70	100	All	2.989	0.003	0.000	0.003	0.000	0.003	0.059	1434.916	1.311	0.082	1.393	0.073	1.332	28.331	14349	13.110	0.820	13.930	0.730	13.320	283.310
120	alpha	0.6	60x8 weeks	Ag80	MWF	VAC70	100	All	2.989	0.003	0.000	0.003	0.000	0.003	0.059	1434.916	1.311	0.082	1.393	0.073	1.332	28.331	14349	13.110	0.820	13.930	0.730	13.320	283.310
120	alpha	0.6	60x8 weeks	Ag80	MWF	VAC70	100	All	2.989	0.003	0.000	0.003	0.000	0.003	0.059	1434.916	1.311	0.082	1.393	0.073	1.332	28.331	14349	13.110	0.820	13.930	0.730	13.320	283.310
120	alpha	1	60x8 weeks	Ag80	MWF	VAC70	100	All	2.989	0.002	0.000	0.003	0.000	0.003	0.060	1434.944	1.198	0.156	1.354	0.073	1.305	28.590	14349	11.980	1.560	13.540	0.730	13.050	285.900
120	alpha	1	60x8 weeks	Ag80	MWF	VAC70	100	All	2.989	0.002	0.000	0.003	0.000	0.003	0.060	1434.944	1.198	0.156	1.354	0.073	1.305	28.590	14349	11.980	1.560	13.540	0.730	13.050	285.900
120	alpha	1	60x8 weeks	Ag80	MWF	VAC70	100	All	2.989	0.002	0.000	0.003	0.000	0.003	0.060	1434.944	1.198	0.156	1.354	0.073	1.305	28.590	14349	11.980	1.560	13.540	0.730	13.050	285.900
120	alpha	1.2	60x8 weeks	Ag80	MWF	VAC70	100	All	2.990	0.003	0.000	0.003	0.000	0.003	0.060	1434.987	1.240	0.129	1.369	0.071	1.298	28.561	14350	12.400	1.290	13.690	0.710	12.980	285.610
120	alpha	1.2	60x8 weeks	Ag80	MWF	VAC70	100	All	2.990	0.003	0.000	0.003	0.000	0.003	0.060	1434.987	1.240	0.129	1.369	0.071	1.298	28.561	14350	12.400	1.290	13.690	0.710	12.980	285.610
120	alpha	1.2	60x8 weeks	Ag80	MWF	VAC70	100	All	2.990	0.003	0.000	0.003	0.000	0.003	0.060	1434.987	1.240	0.129	1.369	0.071	1.298	28.561	14350	12.400	1.290	13.690	0.710	12.980	285.610
120	delta	0.6	60x8 weeks	Ag80	MWF	VAC70	100	All	2.985	0.003	0.001	0.004	0.000	0.004	0.059	1432.858	1.631	0.256	1.887	0.097	1.862	28.334	14329	16.310	2.560	18.870	0.970	18.620	283.340
120	delta	0.6	60x8 weeks	Ag80	MWF	VAC70	100	All	2.985	0.003	0.001	0.004	0.000	0.004	0.059	1432.858	1.631	0.256	1.887	0.097	1.862	28.334	14329	16.310	2.560	18.870	0.970	18.620	283.340
120	delta	0.6	60x8 weeks	Ag80	MWF	VAC70	100	All	2.985	0.003	0.001	0.004	0.000	0.004	0.059	1432.858	1.631	0.256	1.887	0.097	1.862	28.334	14329	16.310	2.560	18.870	0.970	18.620	283.340
120	delta	1	60x8 weeks	Ag80	MWF	VAC70	100	All	2.983	0.003	0.001	0.004	0.000	0.004	0.059	1431.981	1.642	0.483	2.125	0.118	2.122	28.272	14320	16.420	4.830	21.250	1.180	21.220	282.720
120	delta	1	60x8 weeks	Ag80	MWF	VAC70	100	All	2.983	0.003	0.001	0.004	0.000	0.004	0.059	1431.981	1.642	0.483	2.125	0.118	2.122	28.272	14320	16.420	4.830	21.250	1.180	21.220	282.720
120	delta	1	60x8 weeks	Ag80	MWF	VAC70	100	All	2.983	0.003	0.001	0.004	0.000	0.004	0.059	1431.981	1.642	0.483	2.125	0.118	2.122	28.272	14320	16.420	4.830	21.250	1.180	21.220	282.720
120	delta	1.2	60x8 weeks	Ag80	MWF	VAC70	100	All	2.982	0.003	0.001	0.005	0.000	0.005	0.058	1431.438	1.625	0.654	2.279	0.135	2.225	28.028	14314	16.250	6.540	22.790	1.350	22.250	280.280
120	delta	1.2	60x8 weeks	Ag80	MWF	VAC70	100	All	2.982	0.003	0.001	0.005	0.000	0.005	0.058	1431.438	1.625	0.654	2.279	0.135	2.225	28.028	14314	16.250	6.540	22.790	1.350	22.250	280.280
120	delta	1.2	60x8 weeks	Ag80	MWF	VAC70	100	All	2.982	0.003	0.001	0.005	0.000	0.005	0.058	1431.438	1.625	0.654	2.279	0.135	2.225	28.028	14314	16.250	6.540	22.790	1.350	22.250	280.280
120	original	0.6	60x8 weeks	Ag80	MWF	VAC50	25	All	2.972	0.007	0.001	0.008	0.001	0.007	0.058	1426.538	3.398	0.553	3.951	0.372	3.363	27.684	14265	33.980	5.530	39.510	3.720	33.630	276.840
120	original	0.6	60x8 weeks	Ag80	MWF	VAC50	25	All	2.972	0.007	0.001	0.008	0.001	0.007	0.058	1426.538	3.398	0.553	3.951	0.372	3.363	27.684	14265	33.980	5.530	39.510	3.720	33.630	276.840
120	original	0.6	60x8 weeks	Ag80	MWF	VAC50	25	All	2.972	0.007	0.001	0.008	0.001	0.007	0.058	1426.538	3.398	0.553	3.951	0.372	3.363	27.684	14265	33.980	5.530	39.510	3.720	33.630	276.840
120	original	1	60x8 weeks	Ag80	MWF	VAC50	25	All	2.970	0.007	0.002	0.009	0.001	0.007	0.057	1425.382	3.440	0.868	4.308	0.440	3.586	27.535	14254	34.400	8.680	43.080	4.400	35.860	275.350
120	original	1	60x8 weeks	Ag80	MWF	VAC50	25	All	2.970	0.007	0.002	0.009	0.001	0.007	0.057	1425.382	3.440	0.868	4.308	0.440	3.586	27.535	14254	34.400	8.680	43.080	4.400	35.860	275.350
120	original	1	60x8 weeks	Ag80	MWF	VAC50	25	All	2.970	0.007	0.002	0.009	0.001	0.007	0.057	1425.382	3.440	0.868	4.308	0.440	3.586	27.535	14254	34.400	8.680	43.080	4.400	35.860	275.350
120	original	1.2	60x8 weeks	Ag80	MWF	VAC50	25	All	2.967	0.007	0.002	0.010	0.001	0.008	0.058	1424.235	3.502	1.175	4.677	0.509	3.842	27.693	14242	35.020	11.750	46.770	5.090	38.420	276.930
120	original	1.2	60x8 weeks	Ag80	MWF	VAC50	25	All	2.967	0.007	0.002	0.010	0.001	0.008	0.058	1424.235	3.502	1.175	4.677	0.509	3.842	27.693	14242	35.020	11.750	46.770	5.090	38.420	276.930
120	original	1.2	60x8 weeks	Ag80	MWF	VAC50	25	All	2.967	0.007	0.002	0.010	0.001	0.008	0.058	1424.235	3.502	1.175	4.677	0.509	3.842	27.693	14242	35.020	11.750	46.770	5.090	38.420	276.930
120	alpha	0.6	60x8 weeks	Ag80	MWF	VAC50	25	All	2.965	0.007	0.002	0.009	0.000	0.009	0.058	1423.085	3.347	1.167	4.514	0.185	4.518	28.026	14231	33.470	11.670	45.140	1.850	45.180	280.260
120	alpha	0.6	60x8 weeks	Ag80	MWF	VAC50	25	All	2.965	0.007	0.002	0.009	0.000	0.009	0.058	1423.085	3.347	1.167	4.514	0.185	4.518	28.026	14231	33.470	11.670	45.140	1.850	45.180	280.260
120	alpha	0.6	60x8 weeks	Ag80	MWF	VAC50	25	All	2.965	0.007	0.002	0.009	0.000	0.009	0.058	1423.085	3.347	1.167	4.514	0.185	4.518	28.026	14231	33.470	11.670	45.140	1.850	45.180	280.260
120	alpha	1	60x8 weeks	Ag80	MWF	VAC50	25	All	2.957	0.007	0.005	0.011	0.001	0.011	0.057	1419.501	3.277	2.175	5.452	0.268	5.420	27.585	14195	32.770	21.750	54.520	2.680	54.200	275.850
120	alpha	1	60x8 weeks	Ag80	MWF	VAC50	25	All	2.957	0.007	0.005	0.011	0.001	0.011	0.057	1419.501	3.277	2.175	5.452	0.268	5.420	27.585	14195	32.770	21.750	54.520	2.680	54.200	275.850
120	alpha	1	60x8 weeks	Ag80	MWF	VAC50	25	All	2.957	0.007	0.005	0.011	0.001	0.011	0.057	1419.501	3.277	2.175	5.452	0.268	5.420	27.585	14195	32.770	21.750	54.520	2.680	54.200	275.850
120	alpha	1.2	60x8 weeks	Ag80	MWF	VAC50	25	All	2.949	0.007	0.007	0.014	0.001	0.014	0.057	1415.514	3.365	3.177	6.542	0.292	6.491	27.161	14155	33.650	31.770	65.420	2.920	64.910	271.610
120	alpha	1.2	60x8 weeks	Ag80	MWF	VAC50	25	All	2.949	0.007	0.007	0.014	0.001	0.014	0.057	1415.514	3.365	3.177	6.542	0.292	6.491	27.161	14155	33.650	31.770	65.420	2.920	64.910	271.610
120	alpha	1.2	60x8 weeks	Ag80	MWF	VAC50	25	All	2.949	0.007	0.007	0.014	0.001	0.014	0.057	1415.514	3.365	3.177	6.542	0.292	6.491	27.161	14155	33.650	31.770	65.420	2.920	64.910	271.610
120	delta	0.6	60x8 weeks	Ag80	MWF	VAC50	25	All	2.943	0.007	0.007	0.015	0.001	0.015	0.057	1412.869	3.472	3.583	7.055	0.445	7.036	27.126	14129	34.720	35.830	70.550	4.450	70.360	271.260
120	delta	0.6	60x8 weeks	Ag80	MWF	VAC50	25	All	2.943	0.007	0.007	0.015	0.001	0.015	0.057	1412.869	3.472	3.583	7.055	0.445	7.036	27.126	14129	34.720	35.830	70.550	4.450	70.360	271.260
120	delta	0.6	60x8 weeks	Ag80	MWF	VAC50	25	All	2.943	0.007	0.007	0.015	0.001	0.015	0.057	1412.869	3.472	3.583	7.055	0.445	7.036	27.126	14129	34.720	35.830	70.550	4.450	70.360	271.260
120	delta	1	60x8 weeks	Ag80	MWF	VAC50	25	All	2.873	0.007	0.026	0.033	0.002	0.033	0.053	1378.821	3.270	12.509	15.779	1.064	15.668	25.208	13788	32.700	125.090	157.790	10.640	156.680	252.080

120	delta	1	60x8 weeks	Ag80	MWF	VAC50	25	All	2.873	0.007	0.026	0.033	0.002	0.033	0.053	1378.821	3.270	12.509	15.779	1.064	15.668	25.208	13788	32.700	125.090	157.790	10.640	156.680	252.080
120	delta	1	60x8 weeks	Ag80	MWF	VAC50	25	All	2.873	0.007	0.026	0.033	0.002	0.033	0.053	1378.821	3.270	12.509	15.779	1.064	15.668	25.208	13788	32.700	125.090	157.790	10.640	156.680	252.080
120	delta	1.2	60x8 weeks	Ag80	MWF	VAC50	25	All	2.825	0.006	0.039	0.045	0.003	0.045	0.050	1355.984	2.931	18.655	21.586	1.514	21.383	23.894	13560	29.310	186.550	215.860	15.140	213.830	238.940
120	delta	1.2	60x8 weeks	Ag80	MWF	VAC50	25	All	2.825	0.006	0.039	0.045	0.003	0.045	0.050	1355.984	2.931	18.655	21.586	1.514	21.383	23.894	13560	29.310	186.550	215.860	15.140	213.830	238.940
120	delta	1.2	60x8 weeks	Ag80	MWF	VAC50	25	All	2.825	0.006	0.039	0.045	0.003	0.045	0.050	1355.984	2.931	18.655	21.586	1.514	21.383	23.894	13560	29.310	186.550	215.860	15.140	213.830	238.940
120	original	0.6	60x8 weeks	Ag80	MWF	VAC50	50	All	2.980	0.006	0.001	0.007	0.001	0.004	0.058	1430.240	2.966	0.257	3.223	0.584	2.128	28.024	14302	29.660	2.570	32.230	5.840	21.280	280.240
120	original	0.6	60x8 weeks	Ag80	MWF	VAC50	50	All	2.980	0.006	0.001	0.007	0.001	0.004	0.058	1430.240	2.966	0.257	3.223	0.584	2.128	28.024	14302	29.660	2.570	32.230	5.840	21.280	280.240
120	original	0.6	60x8 weeks	Ag80	MWF	VAC50	50	All	2.980	0.006	0.001	0.007	0.001	0.004	0.058	1430.240	2.966	0.257	3.223	0.584	2.128	28.024	14302	29.660	2.570	32.230	5.840	21.280	280.240
120	original	1	60x8 weeks	Ag80	MWF	VAC50	50	All	2.978	0.006	0.001	0.007	0.001	0.005	0.058	1429.301	2.946	0.512	3.458	0.624	2.310	28.014	14293	29.460	5.120	34.580	6.240	23.100	280.140
120	original	1	60x8 weeks	Ag80	MWF	VAC50	50	All	2.978	0.006	0.001	0.007	0.001	0.005	0.058	1429.301	2.946	0.512	3.458	0.624	2.310	28.014	14293	29.460	5.120	34.580	6.240	23.100	280.140
120	original	1	60x8 weeks	Ag80	MWF	VAC50	50	All	2.978	0.006	0.001	0.007	0.001	0.005	0.058	1429.301	2.946	0.512	3.458	0.624	2.310	28.014	14293	29.460	5.120	34.580	6.240	23.100	280.140
120	original	1.2	60x8 weeks	Ag80	MWF	VAC50	50	All	2.977	0.006	0.001	0.007	0.001	0.005	0.058	1428.829	2.914	0.671	3.585	0.614	2.452	27.758	14288	29.140	6.710	35.850	6.140	24.520	277.580
120	original	1.2	60x8 weeks	Ag80	MWF	VAC50	50	All	2.977	0.006	0.001	0.007	0.001	0.005	0.058	1428.829	2.914	0.671	3.585	0.614	2.452	27.758	14288	29.140	6.710	35.850	6.140	24.520	277.580
120	original	1.2	60x8 weeks	Ag80	MWF	VAC50	50	All	2.977	0.006	0.001	0.007	0.001	0.005	0.058	1428.829	2.914	0.671	3.585	0.614	2.452	27.758	14288	29.140	6.710	35.850	6.140	24.520	277.580
120	alpha	0.6	60x8 weeks	Ag80	MWF	VAC50	50	All	2.973	0.006	0.001	0.007	0.000	0.007	0.058	1427.077	2.842	0.657	3.499	0.187	3.401	27.812	14271	28.420	6.570	34.990	1.870	34.010	278.120
120	alpha	0.6	60x8 weeks	Ag80	MWF	VAC50	50	All	2.973	0.006	0.001	0.007	0.000	0.007	0.058	1427.077	2.842	0.657	3.499	0.187	3.401	27.812	14271	28.420	6.570	34.990	1.870	34.010	278.120
120	alpha	0.6	60x8 weeks	Ag80	MWF	VAC50	50	All	2.973	0.006	0.001	0.007	0.000	0.007	0.058	1427.077	2.842	0.657	3.499	0.187	3.401	27.812	14271	28.420	6.570	34.990	1.870	34.010	278.120
120	alpha	1	60x8 weeks	Ag80	MWF	VAC50	50	All	2.966	0.006	0.003	0.009	0.000	0.009	0.058	1423.830	2.954	1.409	4.363	0.196	4.280	27.660	14238	29.540	14.090	43.630	1.960	42.800	276.600
120	alpha	1	60x8 weeks	Ag80	MWF	VAC50	50	All	2.966	0.006	0.003	0.009	0.000	0.009	0.058	1423.830	2.954	1.409	4.363	0.196	4.280	27.660	14238	29.540	14.090	43.630	1.960	42.800	276.600
120	alpha	1	60x8 weeks	Ag80	MWF	VAC50	50	All	2.966	0.006	0.003	0.009	0.000	0.009	0.058	1423.830	2.954	1.409	4.363	0.196	4.280	27.660	14238	29.540	14.090	43.630	1.960	42.800	276.600
120	alpha	1.2	60x8 weeks	Ag80	MWF	VAC50	50	All	2.961	0.006	0.004	0.010	0.001	0.010	0.058	1421.305	3.007	1.996	5.003	0.269	4.909	27.863	14213	30.070	19.960	50.030	2.690	49.090	278.630
120	alpha	1.2	60x8 weeks	Ag80	MWF	VAC50	50	All	2.961	0.006	0.004	0.010	0.001	0.010	0.058	1421.305	3.007	1.996	5.003	0.269	4.909	27.863	14213	30.070	19.960	50.030	2.690	49.090	278.630
120	alpha	1.2	60x8 weeks	Ag80	MWF	VAC50	50	All	2.961	0.006	0.004	0.010	0.001	0.010	0.058	1421.305	3.007	1.996	5.003	0.269	4.909	27.863	14213	30.070	19.960	50.030	2.690	49.090	278.630
120	delta	0.6	60x8 weeks	Ag80	MWF	VAC50	50	All	2.956	0.007	0.005	0.012	0.001	0.011	0.058	1418.963	3.278	2.364	5.642	0.363	5.513	27.640	14190	32.780	23.640	56.420	3.630	55.130	276.400
120	delta	0.6	60x8 weeks	Ag80	MWF	VAC50	50	All	2.956	0.007	0.005	0.012	0.001	0.011	0.058	1418.963	3.278	2.364	5.642	0.363	5.513	27.640	14190	32.780	23.640	56.420	3.630	55.130	276.400
120	delta	0.6	60x8 weeks	Ag80	MWF	VAC50	50	All	2.956	0.007	0.005	0.012	0.001	0.011	0.058	1418.963	3.278	2.364	5.642	0.363	5.513	27.640	14190	32.780	23.640	56.420	3.630	55.130	276.400
120	delta	1	60x8 weeks	Ag80	MWF	VAC50	50	All	2.922	0.006	0.013	0.020	0.001	0.020	0.055	1402.794	3.051	6.476	9.527	0.649	9.451	26.486	14028	30.510	64.760	95.270	6.490	94.510	264.860
120	delta	1	60x8 weeks	Ag80	MWF	VAC50	50	All	2.922	0.006	0.013	0.020	0.001	0.020	0.055	1402.794	3.051	6.476	9.527	0.649	9.451	26.486	14028	30.510	64.760	95.270	6.490	94.510	264.860
120	delta	1	60x8 weeks	Ag80	MWF	VAC50	50	All	2.922	0.006	0.013	0.020	0.001	0.020	0.055	1402.794	3.051	6.476	9.527	0.649	9.451	26.486	14028	30.510	64.760	95.270	6.490	94.510	264.860
120	delta	1.2	60x8 weeks	Ag80	MWF	VAC50	50	All	2.897	0.006	0.021	0.028	0.002	0.027	0.053	1390.373	3.051	10.191	13.242	0.910	12.947	25.512	13904	30.510	101.910	132.420	9.100	129.470	255.120
120	delta	1.2	60x8 weeks	Ag80	MWF	VAC50	50	All	2.897	0.006	0.021	0.028	0.002	0.027	0.053	1390.373	3.051	10.191	13.242	0.910	12.947	25.512	13904	30.510	101.910	132.420	9.100	129.470	255.120
120	delta	1.2	60x8 weeks	Ag80	MWF	VAC50	50	All	2.897	0.006	0.021	0.028	0.002	0.027	0.053	1390.373	3.051	10.191	13.242	0.910	12.947	25.512	13904	30.510	101.910	132.420	9.100	129.470	255.120
120	original	0.6	60x8 weeks	Ag80	MWF	VAC50	75	All	2.985	0.005	0.000	0.005	0.002	0.002	0.059	1432.975	2.438	0.117	2.555	0.746	1.147	28.245	14330	24.380	1.170	25.550	7.460	11.470	282.450
120	original	0.6	60x8 weeks	Ag80	MWF	VAC50	75	All	2.985	0.005	0.000	0.005	0.002	0.002	0.059	1432.975	2.438	0.117	2.555	0.746	1.147	28.245	14330	24.380	1.170	25.550	7.460	11.470	282.450
120	original	0.6	60x8 weeks	Ag80	MWF	VAC50	75	All	2.985	0.005	0.000	0.005	0.002	0.002	0.059	1432.975	2.438	0.117	2.555	0.746	1.147	28.245	14330	24.380	1.170	25.550	7.460	11.470	282.450
120	original	1	60x8 weeks	Ag80	MWF	VAC50	75	All	2.985	0.005	0.000	0.006	0.002	0.002	0.059	1432.786	2.422	0.221	2.643	0.751	1.184	28.275	14328	24.220	2.210	26.430	7.510	11.840	282.750
120	original	1	60x8 weeks	Ag80	MWF	VAC50	75	All	2.985	0.005	0.000	0.006	0.002	0.002	0.059	1432.786	2.422	0.221	2.643	0.751	1.184	28.275	14328	24.220	2.210	26.430	7.510	11.840	282.750
120	original	1	60x8 weeks	Ag80	MWF	VAC50	75	All	2.985	0.005	0.000	0.006	0.002	0.002	0.059	1432.786	2.422	0.221	2.643	0.751	1.184	28.275	14328	24.220	2.210	26.430	7.510	11.840	282.750
120	original	1.2	60x8 weeks	Ag80	MWF	VAC50	75	All	2.985	0.005	0.001	0.006	0.002	0.003	0.059	1432.676	2.491	0.294	2.785	0.751	1.247	28.393	14327	24.910	2.940	27.850	7.510	12.470	283.930
120	original	1.2	60x8 weeks	Ag80	MWF	VAC50	75	All	2.985	0.005	0.001	0.006	0.002	0.003	0.059	1432.676	2.491	0.294	2.785	0.751	1.247	28.393	14327	24.910	2.940	27.850	7.510	12.470	283.930
120	original	1.2	60x8 weeks	Ag80	MWF	VAC50	75	All	2.985	0.005	0.001	0.006	0.002	0.003	0.059	1432.676	2.491	0.294	2.785	0.751	1.247	28.393	14327	24.910	2.940	27.850	7.510	12.470	283.930
120	alpha	0.6	60x8 weeks	Ag80	MWF	VAC50	75	All	2.978	0.005	0.001	0.006	0.000	0.006	0.059	1429.244	2.512	0.429	2.941	0.150	2.847	28.137	14292	25.120	4.290	29.410	1.500	28.470	281.370
120	alpha	0.6	60x8 weeks	Ag80	MWF	VAC50	75	All	2.978	0.005	0.001	0.006	0.000	0.006	0.059	1429.244	2.512	0.429	2.941	0.150	2.847	28.137	14292	25.120	4.290	29.410	1.500	28.470	281.370
120	alpha	0.6	60x8 weeks	Ag80	MWF	VAC50	75	All	2.978	0.005	0.001	0.006	0.000	0.006	0.059	1429.244	2.512	0.429	2.941	0.150	2.847	28.137	14292	25.120	4.290	29.410	1.500	28.470	281.370
120	alpha	1	60x8 weeks	Ag80	MWF	VAC50	75	All	2.975	0.005	0.002	0.007	0.000	0.007	0.059	1427.936	2.454	0.781	3.235	0.184	3.163	28.254	14279	24.540	7.810	32.350	1.840	31.630	282.540
120	alpha	1	60x8 weeks	Ag80	MWF	VAC50	75	All	2.975	0.005	0.002	0.007	0.000	0.007	0.059	1427.936	2.454	0.781	3.235	0.184	3.163	28.254	14279	24.540	7.810	32.350	1.840	31.630	282.540
120	alpha	1	60x8 weeks	Ag80	MWF	VAC50	75	All	2.975	0.005	0.002	0.007	0.000	0.007	0.059	1427.936	2.454	0.781	3.235	0.184	3.163	28.254	14279	24.540	7.810	32.350	1.840	31.630	282.540
120	alpha	1.2	60x8 weeks	Ag80	MWF	VAC50	75	All	2.974	0.005	0.002	0.007	0.000	0.007	0.059	1427.422	2.391	1.014	3.405	0.169	3.328	28.097	14274	23.910	10.140	34.050	1.690	33.280	280.970
120	alpha	1.2	60x8 weeks	Ag80	MWF	VAC50	75	All	2.974	0.005	0.002	0.007	0.000	0.007	0.059	1427.422	2.391	1.014	3.405	0.169	3.328	28.097	14274	23.910	10.140	34.050	1.690	33.280	280.970
120	alpha	1.2	60x8 weeks	Ag80	MWF	VAC50	75	All	2.974	0.005	0.002	0.007	0.000	0.007	0.059	1427.422	2.391	1.014	3.405	0.169	3.328	28.097	14274	23.910	10.140	34.050	1.690	33.280	280.970
120	delta	0.6	60x8 weeks	Ag80	MWF	VAC50	75	All	2.966	0.006	0.003	0.009	0.001	0.009	0.057	1423.880	2.950	1.365	4.315	0.266	4.217	27.591	14239	29.500	13.650	43.150	2.660	42.170	275.910
120	delta	0.6	60x8 weeks	Ag80	MWF	VAC50	75	All	2.966	0.006	0.003	0.009	0.001	0.009	0.057	1423.880	2.950	1.365	4.315	0.266	4.217	27.591	14239	29.500	13.650	43.150	2.660	42.170	275.910
120	delta	0.6	60x8 weeks	Ag80	MWF	VAC50	75	All	2.966	0.006	0.003	0.009	0.001	0.009	0.057	1423.880	2.950	1.365	4.315	0.266	4.217	27.591	14239	29.500	13.650	43.150	2.660	42.170	275.910
120	delta	1	60x8 weeks	Ag80	MWF	VAC50	75	All	2.954	0.006	0.006	0.012	0.001	0.012	0.057	1417.842	2.824	3.110	5.934	0.370	5.802	27.367	14178	28.240	31.100	59.340	3.700	58.020	273.670
120	delta	1	60x8 weeks	Ag80	MWF	VAC50	75	All	2.954	0.006	0.006	0.012	0.001	0.012	0.057	1417.842	2.824	3.110	5.934	0.370	5.802	27.367	14178	28.240	31.100	59.340	3.700	58.020	273.670
120	delta	1	60x8 weeks	Ag80	MWF	VAC50	75	All	2.954	0.006	0.006	0.012	0.001	0.012	0.057	1417.842	2.824	3.110	5.934	0.370	5.802	27.367	14178	28.240	31.100	59.340	3.700	58.020	273.670
120	delta	1.2	60x8 weeks	Ag80	MWF	VAC50	75	All	2.935	0.006	0.011	0.017	0.001	0.017	0.056	1408.590	2.964	5.274	8.238	0.600	8.040	26.644	14086	29.640	52.740	82.380	6.000	80.400	266.440
120	delta	1.2	60x8 weeks	Ag80	MWF	VAC50	75	All	2.935	0.006	0.011	0.017	0.001	0.017	0.056	1408.590	2.964	5.274	8.238	0.600	8.040	26.644	14086	29.640	52.740	82.380	6.000	80.400	266.440
120	delta	1.2	60x8 weeks	Ag80	MWF	VAC50	75	All	2.935	0.006	0.011	0.017	0.001	0.017	0.056	1408.590	2.964	5.274	8.238	0.600	8.040	26.644	14086	29.640	52.740	82.380	6.000	80.400	266.440
120	original	0.6	60x8 weeks	Ag80	MWF	VAC50	100	All	2.991	0.004	0.000	0.004	0.002	0.001	0.059	1435.603	2.016	0.018	2.034	0.885	0.254	28.348	14356	20.160	0.180	20.340	8.850	2.540	283.480
120	original	0.6	60x8 weeks	Ag80	MWF	VAC50	100	All	2.991	0.004	0.000	0.004	0.002	0.001	0.059	1435.603	2.016	0.018	2.034	0.885	0.254	28.348	14356	20.160	0.180	20.340	8.850	2.540	283.480
120	original	0.6	60x8 weeks	Ag80	MWF	VAC50	100	All	2.991	0.004	0.000	0.004	0.002	0.001	0.059	1435.603	2.016	0.018	2.034	0.885	0.254	28.348	14356	20.160	0.180	20.340	8.850	2.540	283.480
120	original	1	60x8 weeks	Ag80	MWF	VAC50	100	All	2.991	0.004	0.000	0.004	0.002	0.001	0.059	1435.577	2.058	0.050	2.108	0.887	0.257	28.176	14356	20.580	0.500	21.080	8.870	2.570	281.760
120	original	1	60x8 weeks	Ag80	MWF	VAC50	100	All	2.991	0.004	0.000	0.004	0.002	0.001	0.059	1435.577	2.058	0.050	2.108	0.887	0.257	28.176	14356	20.580	0.500	21.080	8.870	2.570	281.760
120	original	1	60x8 weeks	Ag80	MWF	VAC50	100	All	2.991	0.004	0.000	0.004	0.002	0.001	0.059	1435.577	2.058	0.050	2.108	0.887	0.257	28.176	14356	20.580	0.500	21.080	8.870	2.570	281.760
120	original	1.2	60x8 weeks	Ag80	MWF	VAC50	100	All	2.990	0.004	0.000	0.004	0.002	0.001	0.059	1435.280	2.120	0.036	2.156	0.960	0.279	28.089	14353	21.200	0.360	21.560	9.600	2.790	280.890
120	original	1.2	60x8 weeks	Ag80	MWF	VAC50	100	All	2.990	0.004	0.000	0.004	0.002	0.001	0.059	1435.280	2.120	0.036	2.156	0.960	0.279	28.089	14353	21.200	0.360	21.560	9.600	2.790	280.890
120	original	1.2	60x8 weeks	Ag80	MWF	VAC50	100	All	2.990	0.004	0.000	0.004	0.002	0.001	0.059	1435.280	2.120	0.036	2.156	0.960	0.279	28.089	14353	21.200	0.360	21.560	9.600	2.790	280.890
120	alpha	0.6	60x8 weeks	Ag80	MWF	VAC50	100	All	2.983	0.004	0.000	0.005	0.000	0.004	0.059	1431.806	2.008	0.220	2.228	0.122	2.130	28.247	14318	20.080	2.200	22.280	1.220	21.300	282.470
120	alpha	0.6	60x8 weeks	Ag80	MWF	VAC50	100	All	2.983	0.004	0.000	0.005	0.000	0.004	0.059	1431.806	2.008	0.220	2.228	0.122	2.130	28.247	14318	20.080	2.200	22.280	1.220	21.300	282.470
120	alpha	0.6	60x8 weeks	Ag80	MWF	VAC50	100	All	2.983	0.004	0.000	0.005	0.000	0.004	0.059	1431.806	2.008	0.220	2.228	0.122	2.130	28.247	14318	20.080	2.200	22.280	1.220	21.300	282.470

120	alpha	1	60x8 weeks	Ag80	MWF	VAC50	100	All	2.981	0.004	0.001	0.005	0.000	0.005	0.059	1430.831	2.079	0.384	2.463	0.144	2.382	28.214	14308	20.790	3.840	24.630	1.440	23.820	282.140
120	alpha	1	60x8 weeks	Ag80	MWF	VAC50	100	All	2.981	0.004	0.001	0.005	0.000	0.005	0.059	1430.831	2.079	0.384	2.463	0.144	2.382	28.214	14308	20.790	3.840	24.630	1.440	23.820	282.140
120	alpha	1	60x8 weeks	Ag80	MWF	VAC50	100	All	2.981	0.004	0.001	0.005	0.000	0.005	0.059	1430.831	2.079	0.384	2.463	0.144	2.382	28.214	14308	20.790	3.840	24.630	1.440	23.820	282.140
120	alpha	1.2	60x8 weeks	Ag80	MWF	VAC50	100	All	2.980	0.004	0.001	0.005	0.000	0.005	0.059	1430.625	2.052	0.511	2.563	0.150	2.457	28.428	14306	20.520	5.110	25.630	1.500	24.570	284.280
120	alpha	1.2	60x8 weeks	Ag80	MWF	VAC50	100	All	2.980	0.004	0.001	0.005	0.000	0.005	0.059	1430.625	2.052	0.511	2.563	0.150	2.457	28.428	14306	20.520	5.110	25.630	1.500	24.570	284.280
120	alpha	1.2	60x8 weeks	Ag80	MWF	VAC50	100	All	2.980	0.004	0.001	0.005	0.000	0.005	0.059	1430.625	2.052	0.511	2.563	0.150	2.457	28.428	14306	20.520	5.110	25.630	1.500	24.570	284.280
120	delta	0.6	60x8 weeks	Ag80	MWF	VAC50	100	All	2.973	0.006	0.002	0.007	0.000	0.007	0.058	1426.902	2.680	0.773	3.453	0.215	3.420	27.894	14269	26.800	7.730	34.530	2.150	34.200	278.940
120	delta	0.6	60x8 weeks	Ag80	MWF	VAC50	100	All	2.973	0.006	0.002	0.007	0.000	0.007	0.058	1426.902	2.680	0.773	3.453	0.215	3.420	27.894	14269	26.800	7.730	34.530	2.150	34.200	278.940
120	delta	0.6	60x8 weeks	Ag80	MWF	VAC50	100	All	2.973	0.006	0.002	0.007	0.000	0.007	0.058	1426.902	2.680	0.773	3.453	0.215	3.420	27.894	14269	26.800	7.730	34.530	2.150	34.200	278.940
120	delta	1	60x8 weeks	Ag80	MWF	VAC50	100	All	2.967	0.006	0.003	0.009	0.001	0.009	0.058	1424.055	2.720	1.553	4.273	0.246	4.143	27.825	14241	27.200	15.530	42.730	2.460	41.430	278.250
120	delta	1	60x8 weeks	Ag80	MWF	VAC50	100	All	2.967	0.006	0.003	0.009	0.001	0.009	0.058	1424.055	2.720	1.553	4.273	0.246	4.143	27.825	14241	27.200	15.530	42.730	2.460	41.430	278.250
120	delta	1	60x8 weeks	Ag80	MWF	VAC50	100	All	2.967	0.006	0.003	0.009	0.001	0.009	0.058	1424.055	2.720	1.553	4.273	0.246	4.143	27.825	14241	27.200	15.530	42.730	2.460	41.430	278.250
120	delta	1.2	60x8 weeks	Ag80	MWF	VAC50	100	All	2.962	0.005	0.005	0.010	0.001	0.010	0.058	1421.561	2.612	2.278	4.890	0.338	4.730	27.717	14216	26.120	22.780	48.900	3.380	47.300	277.170
120	delta	1.2	60x8 weeks	Ag80	MWF	VAC50	100	All	2.962	0.005	0.005	0.010	0.001	0.010	0.058	1421.561	2.612	2.278	4.890	0.338	4.730	27.717	14216	26.120	22.780	48.900	3.380	47.300	277.170
120	delta	1.2	60x8 weeks	Ag80	MWF	VAC50	100	All	2.962	0.005	0.005	0.010	0.001	0.010	0.058	1421.561	2.612	2.278	4.890	0.338	4.730	27.717	14216	26.120	22.780	48.900	3.380	47.300	277.170
120	original	0.6	60x8 weeks	Ag80	M	NOVAC	0	All	0.990	0.008	0.004	0.012	0.003	0.006	0.019	475.165	3.817	1.955	5.772	1.596	2.803	9.144	4752	38.170	19.550	57.720	15.960	28.030	91.440
120	original	0.6	60x8 weeks	Ag80	M	NOVAC	0	All	0.990	0.008	0.004	0.012	0.003	0.006	0.019	475.165	3.817	1.955	5.772	1.596	2.803	9.144	4752	38.170	19.550	57.720	15.960	28.030	91.440
120	original	0.6	60x8 weeks	Ag80	M	NOVAC	0	All	0.990	0.008	0.004	0.012	0.003	0.006	0.019	475.165	3.817	1.955	5.772	1.596	2.803	9.144	4752	38.170	19.550	57.720	15.960	28.030	91.440
120	original	1	60x8 weeks	Ag80	M	NOVAC	0	All	0.985	0.008	0.010	0.018	0.005	0.009	0.019	472.933	3.747	4.774	8.521	2.297	4.212	8.919	4729	37.470	47.740	85.210	22.970	42.120	89.190
120	original	1	60x8 weeks	Ag80	M	NOVAC	0	All	0.985	0.008	0.010	0.018	0.005	0.009	0.019	472.933	3.747	4.774	8.521	2.297	4.212	8.919	4729	37.470	47.740	85.210	22.970	42.120	89.190
120	original	1	60x8 weeks	Ag80	M	NOVAC	0	All	0.985	0.008	0.010	0.018	0.005	0.009	0.019	472.933	3.747	4.774	8.521	2.297	4.212	8.919	4729	37.470	47.740	85.210	22.970	42.120	89.190
120	original	1.2	60x8 weeks	Ag80	M	NOVAC	0	All	0.981	0.008	0.015	0.022	0.006	0.011	0.018	470.967	3.714	6.997	10.711	2.897	5.302	8.844	4710	37.140	69.970	107.110	28.970	53.020	88.440
120	original	1.2	60x8 weeks	Ag80	M	NOVAC	0	All	0.981	0.008	0.015	0.022	0.006	0.011	0.018	470.967	3.714	6.997	10.711	2.897	5.302	8.844	4710	37.140	69.970	107.110	28.970	53.020	88.440
120	original	1.2	60x8 weeks	Ag80	M	NOVAC	0	All	0.981	0.008	0.015	0.022	0.006	0.011	0.018	470.967	3.714	6.997	10.711	2.897	5.302	8.844	4710	37.140	69.970	107.110	28.970	53.020	88.440
120	alpha	0.6	60x8 weeks	Ag80	M	NOVAC	0	All	0.980	0.008	0.014	0.021	0.006	0.012	0.019	470.412	3.714	6.496	10.210	3.027	5.907	8.939	4704	37.140	64.960	102.100	30.270	59.070	89.390
120	alpha	0.6	60x8 weeks	Ag80	M	NOVAC	0	All	0.980	0.008	0.014	0.021	0.006	0.012	0.019	470.412	3.714	6.496	10.210	3.027	5.907	8.939	4704	37.140	64.960	102.100	30.270	59.070	89.390
120	alpha	0.6	60x8 weeks	Ag80	M	NOVAC	0	All	0.980	0.008	0.014	0.021	0.006	0.012	0.019	470.412	3.714	6.496	10.210	3.027	5.907	8.939	4704	37.140	64.960	102.100	30.270	59.070	89.390
120	alpha	1	60x8 weeks	Ag80	M	NOVAC	0	All	0.949	0.007	0.045	0.051	0.016	0.030	0.016	455.591	3.192	21.452	24.644	7.588	14.515	7.906	4556	31.920	214.520	246.440	75.880	145.150	79.060
120	alpha	1	60x8 weeks	Ag80	M	NOVAC	0	All	0.949	0.007	0.045	0.051	0.016	0.030	0.016	455.591	3.192	21.452	24.644	7.588	14.515	7.906	4556	31.920	214.520	246.440	75.880	145.150	79.060
120	alpha	1	60x8 weeks	Ag80	M	NOVAC	0	All	0.949	0.007	0.045	0.051	0.016	0.030	0.016	455.591	3.192	21.452	24.644	7.588	14.515	7.906	4556	31.920	214.520	246.440	75.880	145.150	79.060
120	alpha	1.2	60x8 weeks	Ag80	M	NOVAC	0	All	0.929	0.006	0.065	0.071	0.022	0.042	0.015	445.818	2.997	31.093	34.090	10.564	20.218	7.044	4458	29.970	310.930	340.900	105.640	202.180	70.440
120	alpha	1.2	60x8 weeks	Ag80	M	NOVAC	0	All	0.929	0.006	0.065	0.071	0.022	0.042	0.015	445.818	2.997	31.093	34.090	10.564	20.218	7.044	4458	29.970	310.930	340.900	105.640	202.180	70.440
120	alpha	1.2	60x8 weeks	Ag80	M	NOVAC	0	All	0.929	0.006	0.065	0.071	0.022	0.042	0.015	445.818	2.997	31.093	34.090	10.564	20.218	7.044	4458	29.970	310.930	340.900	105.640	202.180	70.440
120	delta	0.6	60x8 weeks	Ag80	M	NOVAC	0	All	0.918	0.006	0.074	0.080	0.026	0.047	0.014	440.540	2.780	35.382	38.162	12.597	22.534	6.515	4405	27.800	353.820	381.620	125.970	225.340	65.150
120	delta	0.6	60x8 weeks	Ag80	M	NOVAC	0	All	0.918	0.006	0.074	0.080	0.026	0.047	0.014	440.540	2.780	35.382	38.162	12.597	22.534	6.515	4405	27.800	353.820	381.620	125.970	225.340	65.150
120	delta	0.6	60x8 weeks	Ag80	M	NOVAC	0	All	0.918	0.006	0.074	0.080	0.026	0.047	0.014	440.540	2.780	35.382	38.162	12.597	22.534	6.515	4405	27.800	353.820	381.620	125.970	225.340	65.150
120	delta	1	60x8 weeks	Ag80	M	NOVAC	0	All	0.875	0.004	0.108	0.112	0.038	0.069	0.010	419.845	1.949	51.954	53.903	18.166	32.980	4.746	4198	19.490	519.540	539.030	181.660	329.800	47.460
120	delta	1	60x8 weeks	Ag80	M	NOVAC	0	All	0.875	0.004	0.108	0.112	0.038	0.069	0.010	419.845	1.949	51.954	53.903	18.166	32.980	4.746	4198	19.490	519.540	539.030	181.660	329.800	47.460
120	delta	1	60x8 weeks	Ag80	M	NOVAC	0	All	0.875	0.004	0.108	0.112	0.038	0.069	0.010	419.845	1.949	51.954	53.903	18.166	32.980	4.746	4198	19.490	519.540	539.030	181.660	329.800	47.460
120	delta	1.2	60x8 weeks	Ag80	M	NOVAC	0	All	0.867	0.004	0.114	0.117	0.040	0.071	0.009	416.359	1.828	54.553	56.381	19.359	34.309	4.465	4164	18.280	545.530	563.810	193.590	343.090	44.650
120	delta	1.2	60x8 weeks	Ag80	M	NOVAC	0	All	0.867	0.004	0.114	0.117	0.040	0.071	0.009	416.359	1.828	54.553	56.381	19.359	34.309	4.465	4164	18.280	545.530	563.810	193.590	343.090	44.650
120	delta	1.2	60x8 weeks	Ag80	M	NOVAC	0	All	0.867	0.004	0.114	0.117	0.040	0.071	0.009	416.359	1.828	54.553	56.381	19.359	34.309	4.465	4164	18.280	545.530	563.810	193.590	343.090	44.650
120	original	0.6	60x8 weeks	Ag80	M	VAC95	25	All	0.993	0.006	0.002	0.008	0.002	0.004	0.019	476.672	2.959	1.064	4.023	1.100	1.892	9.277	4767	29.590	10.640	40.230	11.000	18.920	92.770
120	original	0.6	60x8 weeks	Ag80	M	VAC95	25	All	0.993	0.006	0.002	0.008	0.002	0.004	0.019	476.672	2.959	1.064	4.023	1.100	1.892	9.277	4767	29.590	10.640	40.230	11.000	18.920	92.770
120	original	0.6	60x8 weeks	Ag80	M	VAC95	25	All	0.993	0.006	0.002	0.008	0.002	0.004	0.019	476.672	2.959	1.064	4.023	1.100	1.892	9.277	4767	29.590	10.640	40.230	11.000	18.920	92.770
120	original	1	60x8 weeks	Ag80	M	VAC95	25	All	0.992	0.006	0.004	0.010	0.003	0.005	0.019	476.003	2.882	2.083	4.965	1.322	2.388	9.294	4760	28.820	20.830	49.650	13.220	23.880	92.940
120	original	1	60x8 weeks	Ag80	M	VAC95	25	All	0.992	0.006	0.004	0.010	0.003	0.005	0.019	476.003	2.882	2.083	4.965	1.322	2.388	9.294	4760	28.820	20.830	49.650	13.220	23.880	92.940
120	original	1	60x8 weeks	Ag80	M	VAC95	25	All	0.992	0.006	0.004	0.010	0.003	0.005	0.019	476.003	2.882	2.083	4.965	1.322	2.388	9.294	4760	28.820	20.830	49.650	13.220	23.880	92.940
120	original	1.2	60x8 weeks	Ag80	M	VAC95	25	All	0.990	0.006	0.006	0.012	0.003	0.006	0.019	475.128	2.819	2.938	5.757	1.623	2.767	9.245	4751	28.190	29.380	57.570	16.230	27.670	92.450
120	original	1.2	60x8 weeks	Ag80	M	VAC95	25	All	0.990	0.006	0.006	0.012	0.003	0.006	0.019	475.128	2.819	2.938	5.757	1.623	2.767	9.245	4751	28.190	29.380	57.570	16.230	27.670	92.450
120	original	1.2	60x8 weeks	Ag80	M	VAC95	25	All	0.990	0.006	0.006	0.012	0.003	0.006	0.019	475.128	2.819	2.938	5.757	1.623	2.767	9.245	4751	28.190	29.380	57.570	16.230	27.670	92.450
120	alpha	0.6	60x8 weeks	Ag80	M	VAC95	25	All	0.988	0.006	0.006	0.012	0.004	0.007	0.019	474.215	2.910	3.010	5.920	1.832	3.394	9.214	4742	29.100	30.100	59.200	18.320	33.940	92.140
120	alpha	0.6	60x8 weeks	Ag80	M	VAC95	25	All	0.988	0.006	0.006	0.012	0.004	0.007	0.019	474.215	2.910	3.010	5.920	1.832	3.394	9.214	4742	29.100	30.100	59.200	18.320	33.940	92.140
120	alpha	0.6	60x8 weeks	Ag80	M	VAC95	25	All	0.988	0.006	0.006	0.012	0.004	0.007	0.019	474.215	2.910	3.010	5.920	1.832	3.394	9.214	4742	29.100	30.100	59.200	18.320	33.940	92.140
120	alpha	1	60x8 weeks	Ag80	M	VAC95	25	All	0.978	0.006	0.017	0.023	0.007	0.013	0.018	469.673	2.757	8.189	10.946	3.275	6.292	8.722	4697	27.570	81.890	109.460	32.750	62.920	87.220
120	alpha	1	60x8 weeks	Ag80	M	VAC95	25	All	0.978	0.006	0.017	0.023	0.007	0.013	0.018	469.673	2.757	8.189	10.946	3.275	6.292	8.722	4697	27.570	81.890	109.460	32.750	62.920	87.220
120	alpha	1	60x8 weeks	Ag80	M	VAC95	25	All	0.978	0.006	0.017	0.023	0.007	0.013	0.018	469.673	2.757	8.189	10.946	3.275	6.292	8.722	4697	27.570	81.890	109.460	32.750	62.920	87.220
120	alpha	1.2	60x8 weeks	Ag80	M	VAC95	25	All	0.973	0.006	0.024	0.029	0.009	0.017	0.018	466.883	2.646	11.426	14.072	4.134	8.142	8.715	4669	26.460	114.260	140.720	41.340	81.420	87.150
120	alpha	1.2	60x8 weeks	Ag80	M	VAC95	25	All	0.973	0.006	0.024	0.029	0.009	0.017	0.018	466.883	2.646	11.426	14.072	4.134	8.142	8.715	4669	26.460	114.260	140.720	41.340	81.420	87.150
120	alpha	1.2	60x8 weeks	Ag80	M	VAC95	25	All	0.973	0.006	0.024	0.029	0.009	0.017	0.018	466.883	2.646	11.426	14.072	4.134	8.142	8.715	4669	26.460	114.260	140.720	41.340	81.420	87.150
120	delta	0.6	60x8 weeks	Ag80	M	VAC95	25	All	0.966	0.006	0.030	0.035	0.012	0.020	0.017	463.482	2.654	14.299	16.953	5.522	9.609	8.213	4635	26.540	142.990	169.530	55.220	96.090	82.130
120	delta	0.6	60x8 weeks	Ag80	M	VAC95	25	All	0.966	0.006	0.030	0.035	0.012	0.020	0.017	463.482	2.654	14.299	16.953	5.522	9.609	8.213	4635	26.540	142.990	169.530	55.220	96.090	82.130
120	delta	0.6	60x8 weeks	Ag80	M	VAC95	25	All	0.966	0.006	0.030	0.035	0.012	0.020	0.017	463.482	2.654	14.299	16.953	5.522	9.609	8.213	4635	26.540	142.990	169.530	55.220	96.090	82.130
120	delta	1	60x8 weeks	Ag80	M	VAC95	25	All	0.921	0.004	0.072	0.076	0.025	0.045	0.014	441.910	1.973	34.422	36.395	12.011	21.484	6.861	4419	19.730	344.220	363.950	120.110	214.840	68.610
120	delta	1	60x8 weeks	Ag80	M	VAC95	25	All	0.921	0.004	0.072	0.076	0.025	0.045	0.014	441.910	1.973	34.422	36.395	12.011	21.484	6.861	4419	19.730	344.220	363.950	120.110	214.840	68.610
120	delta	1	60x8 weeks	Ag80	M	VAC95	25	All	0.921	0.004	0.072	0.076	0.025	0.045	0.014	441.910	1.973	34.422	36.395	12.011	21.484	6.861	4419	19.730	344.220	363.950	120.110	214.840	68.610
120	delta	1.2	60x8 weeks	Ag80	M	VAC95	25	All	0.907	0.004	0.082	0.086	0.029	0.052	0.013	435.232	1.810	39.460	41.270	13.807	24.846	6.020	4352	18.100	394.600	412.700	138.070	248.460	60.200
120	delta	1.2	60x8 weeks	Ag80	M	VAC95	25	All	0.907	0.004	0.082	0.086	0.029	0.052	0.013	435.232	1.810	39.460	41.270	13.807	24.846	6.020	4352	18.100	394.600	412.700	138.070	248.460	60.200
120	delta	1.2	60x8 weeks	Ag80	M	VAC95	25	All	0.907	0.004	0.082	0.086	0.029	0.052	0.013	435.232	1.810	39.460	41.270	13.807	24.846	6.020	4352	18.100	394.600	412.700	138.070	248.460	60.200
120	original	0.6	60x8 weeks	Ag80	M	VAC95	50	All	0.996	0.004	0.001	0.005	0.001	0.002	0.020	477.912	2.141	0.422	2.563	0.706	1.182	9.465	4779	21.410	4.220	25.630	7.060	11.820	94.650
120	original	0.6	60x8 weeks	Ag80	M	VAC95	50	All	0.996	0.004	0.001	0.005	0.001	0.002	0.020	477.912	2.141	0.422	2.563	0.706	1.182	9.465	4779	21.410	4.220	25.630	7.060	11.820	94.650

120	original	0.6	60x8 weeks	Ag80	M	VAC95	50	All	0.996	0.004	0.001	0.005	0.001	0.002	0.020	477.912	2.141	0.422	2.563	0.706	1.182	9.465	4779	21.410	4.220	25.630	7.060	11.820	94.650
120	original	1	60x8 weeks	Ag80	M	VAC95	50	All	0.995	0.004	0.002	0.006	0.002	0.003	0.019	477.689	2.056	0.849	2.905	0.811	1.329	9.343	4777	20.560	8.490	29.050	8.110	13.290	93.430
120	original	1	60x8 weeks	Ag80	M	VAC95	50	All	0.995	0.004	0.002	0.006	0.002	0.003	0.019	477.689	2.056	0.849	2.905	0.811	1.329	9.343	4777	20.560	8.490	29.050	8.110	13.290	93.430
120	original	1	60x8 weeks	Ag80	M	VAC95	50	All	0.995	0.004	0.002	0.006	0.002	0.003	0.019	477.689	2.056	0.849	2.905	0.811	1.329	9.343	4777	20.560	8.490	29.050	8.110	13.290	93.430
120	original	1.2	60x8 weeks	Ag80	M	VAC95	50	All	0.995	0.004	0.002	0.006	0.002	0.003	0.019	477.543	2.052	1.053	3.105	0.886	1.366	9.336	4775	20.520	10.530	31.050	8.860	13.660	93.360
120	original	1.2	60x8 weeks	Ag80	M	VAC95	50	All	0.995	0.004	0.002	0.006	0.002	0.003	0.019	477.543	2.052	1.053	3.105	0.886	1.366	9.336	4775	20.520	10.530	31.050	8.860	13.660	93.360
120	original	1.2	60x8 weeks	Ag80	M	VAC95	50	All	0.995	0.004	0.002	0.006	0.002	0.003	0.019	477.543	2.052	1.053	3.105	0.886	1.366	9.336	4775	20.520	10.530	31.050	8.860	13.660	93.360
120	alpha	0.6	60x8 weeks	Ag80	M	VAC95	50	All	0.994	0.004	0.002	0.007	0.002	0.004	0.020	476.980	2.119	1.119	3.238	1.044	1.718	9.563	4770	21.190	11.190	32.380	10.440	17.180	95.630
120	alpha	0.6	60x8 weeks	Ag80	M	VAC95	50	All	0.994	0.004	0.002	0.007	0.002	0.004	0.020	476.980	2.119	1.119	3.238	1.044	1.718	9.563	4770	21.190	11.190	32.380	10.440	17.180	95.630
120	alpha	0.6	60x8 weeks	Ag80	M	VAC95	50	All	0.994	0.004	0.002	0.007	0.002	0.004	0.020	476.980	2.119	1.119	3.238	1.044	1.718	9.563	4770	21.190	11.190	32.380	10.440	17.180	95.630
120	alpha	1	60x8 weeks	Ag80	M	VAC95	50	All	0.991	0.004	0.005	0.010	0.003	0.006	0.019	475.700	2.088	2.586	4.674	1.351	2.656	9.199	4757	20.880	25.860	46.740	13.510	26.560	91.990
120	alpha	1	60x8 weeks	Ag80	M	VAC95	50	All	0.991	0.004	0.005	0.010	0.003	0.006	0.019	475.700	2.088	2.586	4.674	1.351	2.656	9.199	4757	20.880	25.860	46.740	13.510	26.560	91.990
120	alpha	1	60x8 weeks	Ag80	M	VAC95	50	All	0.991	0.004	0.005	0.010	0.003	0.006	0.019	475.700	2.088	2.586	4.674	1.351	2.656	9.199	4757	20.880	25.860	46.740	13.510	26.560	91.990
120	alpha	1.2	60x8 weeks	Ag80	M	VAC95	50	All	0.989	0.004	0.007	0.012	0.004	0.007	0.019	474.714	2.080	3.581	5.661	1.683	3.215	9.206	4747	20.800	35.810	56.610	16.830	32.150	92.060
120	alpha	1.2	60x8 weeks	Ag80	M	VAC95	50	All	0.989	0.004	0.007	0.012	0.004	0.007	0.019	474.714	2.080	3.581	5.661	1.683	3.215	9.206	4747	20.800	35.810	56.610	16.830	32.150	92.060
120	alpha	1.2	60x8 weeks	Ag80	M	VAC95	50	All	0.989	0.004	0.007	0.012	0.004	0.007	0.019	474.714	2.080	3.581	5.661	1.683	3.215	9.206	4747	20.800	35.810	56.610	16.830	32.150	92.060
120	delta	0.6	60x8 weeks	Ag80	M	VAC95	50	All	0.988	0.004	0.008	0.013	0.004	0.007	0.019	474.063	2.068	4.014	6.082	1.940	3.494	9.138	4741	20.680	40.140	60.820	19.400	34.940	91.380
120	delta	0.6	60x8 weeks	Ag80	M	VAC95	50	All	0.988	0.004	0.008	0.013	0.004	0.007	0.019	474.063	2.068	4.014	6.082	1.940	3.494	9.138	4741	20.680	40.140	60.820	19.400	34.940	91.380
120	delta	0.6	60x8 weeks	Ag80	M	VAC95	50	All	0.988	0.004	0.008	0.013	0.004	0.007	0.019	474.063	2.068	4.014	6.082	1.940	3.494	9.138	4741	20.680	40.140	60.820	19.400	34.940	91.380
120	delta	1	60x8 weeks	Ag80	M	VAC95	50	All	0.971	0.004	0.025	0.029	0.009	0.017	0.018	466.294	1.885	12.172	14.057	4.533	8.033	8.663	4663	18.850	121.720	140.570	45.330	80.330	86.630
120	delta	1	60x8 weeks	Ag80	M	VAC95	50	All	0.971	0.004	0.025	0.029	0.009	0.017	0.018	466.294	1.885	12.172	14.057	4.533	8.033	8.663	4663	18.850	121.720	140.570	45.330	80.330	86.630
120	delta	1	60x8 weeks	Ag80	M	VAC95	50	All	0.971	0.004	0.025	0.029	0.009	0.017	0.018	466.294	1.885	12.172	14.057	4.533	8.033	8.663	4663	18.850	121.720	140.570	45.330	80.330	86.630
120	delta	1.2	60x8 weeks	Ag80	M	VAC95	50	All	0.960	0.004	0.037	0.040	0.013	0.023	0.017	460.823	1.769	17.648	19.417	6.302	11.261	8.324	4608	17.690	176.480	194.170	63.020	112.610	83.240
120	delta	1.2	60x8 weeks	Ag80	M	VAC95	50	All	0.960	0.004	0.037	0.040	0.013	0.023	0.017	460.823	1.769	17.648	19.417	6.302	11.261	8.324	4608	17.690	176.480	194.170	63.020	112.610	83.240
120	delta	1.2	60x8 weeks	Ag80	M	VAC95	50	All	0.960	0.004	0.037	0.040	0.013	0.023	0.017	460.823	1.769	17.648	19.417	6.302	11.261	8.324	4608	17.690	176.480	194.170	63.020	112.610	83.240
120	original	0.6	60x8 weeks	Ag80	M	VAC95	75	All	0.998	0.002	0.000	0.003	0.001	0.001	0.020	479.015	1.156	0.094	1.250	0.367	0.513	9.541	4790	11.560	0.940	12.500	3.670	5.130	95.410
120	original	0.6	60x8 weeks	Ag80	M	VAC95	75	All	0.998	0.002	0.000	0.003	0.001	0.001	0.020	479.015	1.156	0.094	1.250	0.367	0.513	9.541	4790	11.560	0.940	12.500	3.670	5.130	95.410
120	original	0.6	60x8 weeks	Ag80	M	VAC95	75	All	0.998	0.002	0.000	0.003	0.001	0.001	0.020	479.015	1.156	0.094	1.250	0.367	0.513	9.541	4790	11.560	0.940	12.500	3.670	5.130	95.410
120	original	1	60x8 weeks	Ag80	M	VAC95	75	All	0.998	0.003	0.000	0.003	0.001	0.001	0.019	478.895	1.225	0.193	1.418	0.428	0.545	9.345	4789	12.250	1.930	14.180	4.280	5.450	93.450
120	original	1	60x8 weeks	Ag80	M	VAC95	75	All	0.998	0.003	0.000	0.003	0.001	0.001	0.019	478.895	1.225	0.193	1.418	0.428	0.545	9.345	4789	12.250	1.930	14.180	4.280	5.450	93.450
120	original	1	60x8 weeks	Ag80	M	VAC95	75	All	0.998	0.003	0.000	0.003	0.001	0.001	0.019	478.895	1.225	0.193	1.418	0.428	0.545	9.345	4789	12.250	1.930	14.180	4.280	5.450	93.450
120	original	1.2	60x8 weeks	Ag80	M	VAC95	75	All	0.998	0.002	0.001	0.003	0.001	0.001	0.020	478.897	1.186	0.245	1.431	0.410	0.576	9.508	4789	11.860	2.450	14.310	4.100	5.760	95.080
120	original	1.2	60x8 weeks	Ag80	M	VAC95	75	All	0.998	0.002	0.001	0.003	0.001	0.001	0.020	478.897	1.186	0.245	1.431	0.410	0.576	9.508	4789	11.860	2.450	14.310	4.100	5.760	95.080
120	original	1.2	60x8 weeks	Ag80	M	VAC95	75	All	0.998	0.002	0.001	0.003	0.001	0.001	0.020	478.897	1.186	0.245	1.431	0.410	0.576	9.508	4789	11.860	2.450	14.310	4.100	5.760	95.080
120	alpha	0.6	60x8 weeks	Ag80	M	VAC95	75	All	0.997	0.003	0.001	0.003	0.001	0.002	0.020	478.521	1.273	0.279	1.552	0.477	0.848	9.384	4785	12.730	2.790	15.520	4.770	8.480	93.840
120	alpha	0.6	60x8 weeks	Ag80	M	VAC95	75	All	0.997	0.003	0.001	0.003	0.001	0.002	0.020	478.521	1.273	0.279	1.552	0.477	0.848	9.384	4785	12.730	2.790	15.520	4.770	8.480	93.840
120	alpha	0.6	60x8 weeks	Ag80	M	VAC95	75	All	0.997	0.003	0.001	0.003	0.001	0.002	0.020	478.521	1.273	0.279	1.552	0.477	0.848	9.384	4785	12.730	2.790	15.520	4.770	8.480	93.840
120	alpha	1	60x8 weeks	Ag80	M	VAC95	75	All	0.996	0.003	0.001	0.004	0.001	0.002	0.020	478.278	1.239	0.648	1.887	0.570	1.019	9.557	4783	12.390	6.480	18.870	5.700	10.190	95.570
120	alpha	1	60x8 weeks	Ag80	M	VAC95	75	All	0.996	0.003	0.001	0.004	0.001	0.002	0.020	478.278	1.239	0.648	1.887	0.570	1.019	9.557	4783	12.390	6.480	18.870	5.700	10.190	95.570
120	alpha	1	60x8 weeks	Ag80	M	VAC95	75	All	0.996	0.003	0.001	0.004	0.001	0.002	0.020	478.278	1.239	0.648	1.887	0.570	1.019	9.557	4783	12.390	6.480	18.870	5.700	10.190	95.570
120	alpha	1.2	60x8 weeks	Ag80	M	VAC95	75	All	0.996	0.002	0.001	0.004	0.001	0.002	0.020	478.285	1.189	0.696	1.885	0.557	1.023	9.389	4783	11.890	6.960	18.850	5.570	10.230	93.890
120	alpha	1.2	60x8 weeks	Ag80	M	VAC95	75	All	0.996	0.002	0.001	0.004	0.001	0.002	0.020	478.285	1.189	0.696	1.885	0.557	1.023	9.389	4783	11.890	6.960	18.850	5.570	10.230	93.890
120	alpha	1.2	60x8 weeks	Ag80	M	VAC95	75	All	0.996	0.002	0.001	0.004	0.001	0.002	0.020	478.285	1.189	0.696	1.885	0.557	1.023	9.389	4783	11.890	6.960	18.850	5.570	10.230	93.890
120	delta	0.6	60x8 weeks	Ag80	M	VAC95	75	All	0.996	0.003	0.002	0.005	0.002	0.003	0.020	477.870	1.370	0.925	2.295	0.742	1.240	9.639	4779	13.700	9.250	22.950	7.420	12.400	96.390
120	delta	0.6	60x8 weeks	Ag80	M	VAC95	75	All	0.996	0.003	0.002	0.005	0.002	0.003	0.020	477.870	1.370	0.925	2.295	0.742	1.240	9.639	4779	13.700	9.250	22.950	7.420	12.400	96.390
120	delta	0.6	60x8 weeks	Ag80	M	VAC95	75	All	0.996	0.003	0.002	0.005	0.002	0.003	0.020	477.870	1.370	0.925	2.295	0.742	1.240	9.639	4779	13.700	9.250	22.950	7.420	12.400	96.390
120	delta	1	60x8 weeks	Ag80	M	VAC95	75	All	0.993	0.003	0.005	0.008	0.002	0.004	0.019	476.468	1.444	2.257	3.701	1.166	2.099	9.236	4765	14.440	22.570	37.010	11.660	20.990	92.360
120	delta	1	60x8 weeks	Ag80	M	VAC95	75	All	0.993	0.003	0.005	0.008	0.002	0.004	0.019	476.468	1.444	2.257	3.701	1.166	2.099	9.236	4765	14.440	22.570	37.010	11.660	20.990	92.360
120	delta	1	60x8 weeks	Ag80	M	VAC95	75	All	0.993	0.003	0.005	0.008	0.002	0.004	0.019	476.468	1.444	2.257	3.701	1.166	2.099	9.236	4765	14.440	22.570	37.010	11.660	20.990	92.360
120	delta	1.2	60x8 weeks	Ag80	M	VAC95	75	All	0.991	0.003	0.007	0.009	0.003	0.005	0.019	475.683	1.281	3.259	4.540	1.412	2.534	9.301	4757	12.810	32.590	45.400	14.120	25.340	93.010
120	delta	1.2	60x8 weeks	Ag80	M	VAC95	75	All	0.991	0.003	0.007	0.009	0.003	0.005	0.019	475.683	1.281	3.259	4.540	1.412	2.534	9.301	4757	12.810	32.590	45.400	14.120	25.340	93.010
120	delta	1.2	60x8 weeks	Ag80	M	VAC95	75	All	0.991	0.003	0.007	0.009	0.003	0.005	0.019	475.683	1.281	3.259	4.540	1.412	2.534	9.301	4757	12.810	32.590	45.400	14.120	25.340	93.010
120	original	0.6	60x8 weeks	Ag80	M	VAC95	100	All	1.000	0.001	0.000	0.001	0.000	0.000	0.020	479.850	0.267	0.001	0.268	0.106	0.005	9.673	4799	2.670	0.010	2.680	1.060	0.050	96.730
120	original	0.6	60x8 weeks	Ag80	M	VAC95	100	All	1.000	0.001	0.000	0.001	0.000	0.000	0.020	479.850	0.267	0.001	0.268	0.106	0.005	9.673	4799	2.670	0.010	2.680	1.060	0.050	96.730
120	original	0.6	60x8 weeks	Ag80	M	VAC95	100	All	1.000	0.001	0.000	0.001	0.000	0.000	0.020	479.850	0.267	0.001	0.268	0.106	0.005	9.673	4799	2.670	0.010	2.680	1.060	0.050	96.730
120	original	1	60x8 weeks	Ag80	M	VAC95	100	All	1.000	0.001	0.000	0.001	0.000	0.000	0.020	479.863	0.274	0.000	0.274	0.097	0.012	9.531	4799	2.740	0.000	2.740	0.970	0.120	95.310
120	original	1	60x8 weeks	Ag80	M	VAC95	100	All	1.000	0.001	0.000	0.001	0.000	0.000	0.020	479.863	0.274	0.000	0.274	0.097	0.012	9.531	4799	2.740	0.000	2.740	0.970	0.120	95.310
120	original	1	60x8 weeks	Ag80	M	VAC95	100	All	1.000	0.001	0.000	0.001	0.000	0.000	0.020	479.863	0.274	0.000	0.274	0.097	0.012	9.531	4799	2.740	0.000	2.740	0.970	0.120	95.310
120	original	1.2	60x8 weeks	Ag80	M	VAC95	100	All	1.000	0.001	0.000	0.001	0.000	0.000	0.020	479.872	0.275	0.001	0.276	0.091	0.014	9.521	4799	2.750	0.010	2.760	0.910	0.140	95.210
120	original	1.2	60x8 weeks	Ag80	M	VAC95	100	All	1.000	0.001	0.000	0.001	0.000	0.000	0.020	479.872	0.275	0.001	0.276	0.091	0.014	9.521	4799	2.750	0.010	2.760	0.910	0.140	95.210
120	original	1.2	60x8 weeks	Ag80	M	VAC95	100	All	1.000	0.001	0.000	0.001	0.000	0.000	0.020	479.872	0.275	0.001	0.276	0.091	0.014	9.521	4799	2.750	0.010	2.760	0.910	0.140	95.210
120	alpha	0.6	60x8 weeks	Ag80	M	VAC95	100	All	0.999	0.001	0.000	0.001	0.000	0.000	0.020	479.703	0.329	0.013	0.342	0.087	0.179	9.568	4797	3.290	0.130	3.420	0.870	1.790	95.680
120	alpha	0.6	60x8 weeks	Ag80	M	VAC95	100	All	0.999	0.001	0.000	0.001	0.000	0.000	0.020	479.703	0.329	0.013	0.342	0.087	0.179	9.568	4797	3.290	0.130	3.420	0.870	1.790	95.680
120	alpha	0.6	60x8 weeks	Ag80	M	VAC95	100	All	0.999	0.001	0.000	0.001	0.000	0.000	0.020	479.703	0.329	0.013	0.342	0.087	0.179	9.568	4797	3.290	0.130	3.420	0.870	1.790	95.680
120	alpha	1	60x8 weeks	Ag80	M	VAC95	100	All	0.999	0.001	0.000	0.001	0.000	0.000	0.020	479.638	0.403	0.022	0.425	0.114	0.220	9.517	4796	4.030	0.220	4.250	1.140	2.200	95.170
120	alpha	1	60x8 weeks	Ag80	M	VAC95	100	All	0.999	0.001	0.000	0.001	0.000	0.000	0.020	479.638	0.403	0.022	0.425	0.114	0.220	9.517	4796	4.030	0.220	4.250	1.140	2.200	95.170
120	alpha	1	60x8 weeks	Ag80	M	VAC95	100	All	0.999	0.001	0.000	0.001	0.000	0.000	0.020	479.638	0.403	0.022	0.425	0.114	0.220	9.517	4796	4.030	0.220	4.250	1.140	2.200	95.170
120	alpha	1.2	60x8 weeks	Ag80	M	VAC95	100	All	0.999	0.001	0.000	0.001	0.000	0.000	0.020	479.688	0.378	0.032	0.410	0.114	0.200	9.514	4797	3.780	0.320	4.100	1.140	2.000	95.140
120	alpha	1.2	60x8 weeks	Ag80	M	VAC95	100	All	0.999	0.001	0.000	0.001	0.000	0.000	0.020	479.688	0.378	0.032	0.410	0.114	0.200	9.514	4797	3.780	0.320	4.100	1.140	2.000	95.140
120	alpha	1.2	60x8 weeks	Ag80	M	VAC95	100	All	0.999	0.001	0.000	0.001	0.000	0.000	0.020	479.688	0.378	0.032	0.410	0.114	0.200	9.514	4797	3.780	0.320	4.100	1.140	2.000	95.140
120	delta	0.6	60x8 weeks	Ag80	M	VAC95	100	All	0.999	0.001	0.000	0.001	0.000	0.001	0.020	479.378	0.583	0.083	0.666	0.215	0.353	9.695	4794	5.830	0.830	6.660	2.150	3.530	96.950

120	delta	0.6	60x8 weeks	Ag80	M	VAC95	100	All	0.999	0.001	0.000	0.001	0.000	0.001	0.020	479.378	0.583	0.083	0.666	0.215	0.353	9.695	4794	5.830	0.830	6.660	2.150	3.530	96.950
120	delta	0.6	60x8 weeks	Ag80	M	VAC95	100	All	0.999	0.001	0.000	0.001	0.000	0.001	0.020	479.378	0.583	0.083	0.666	0.215	0.353	9.695	4794	5.830	0.830	6.660	2.150	3.530	96.950
120	delta	1	60x8 weeks	Ag80	M	VAC95	100	All	0.999	0.001	0.000	0.001	0.000	0.001	0.020	479.387	0.548	0.150	0.698	0.208	0.367	9.618	4794	5.480	1.500	6.980	2.080	3.670	96.180
120	delta	1	60x8 weeks	Ag80	M	VAC95	100	All	0.999	0.001	0.000	0.001	0.000	0.001	0.020	479.387	0.548	0.150	0.698	0.208	0.367	9.618	4794	5.480	1.500	6.980	2.080	3.670	96.180
120	delta	1	60x8 weeks	Ag80	M	VAC95	100	All	0.999	0.001	0.000	0.001	0.000	0.001	0.020	479.387	0.548	0.150	0.698	0.208	0.367	9.618	4794	5.480	1.500	6.980	2.080	3.670	96.180
120	delta	1.2	60x8 weeks	Ag80	M	VAC95	100	All	0.998	0.001	0.000	0.002	0.001	0.001	0.020	479.234	0.612	0.213	0.825	0.248	0.447	9.482	4792	6.120	2.130	8.250	2.480	4.470	94.820
120	delta	1.2	60x8 weeks	Ag80	M	VAC95	100	All	0.998	0.001	0.000	0.002	0.001	0.001	0.020	479.234	0.612	0.213	0.825	0.248	0.447	9.482	4792	6.120	2.130	8.250	2.480	4.470	94.820
120	delta	1.2	60x8 weeks	Ag80	M	VAC95	100	All	0.998	0.001	0.000	0.002	0.001	0.001	0.020	479.234	0.612	0.213	0.825	0.248	0.447	9.482	4792	6.120	2.130	8.250	2.480	4.470	94.820
120	original	0.6	60x8 weeks	Ag80	M	VAC70	25	All	0.993	0.007	0.002	0.009	0.003	0.004	0.019	476.492	3.236	1.176	4.412	1.284	1.922	9.349	4765	32.360	11.760	44.120	12.840	19.220	93.490
120	original	0.6	60x8 weeks	Ag80	M	VAC70	25	All	0.993	0.007	0.002	0.009	0.003	0.004	0.019	476.492	3.236	1.176	4.412	1.284	1.922	9.349	4765	32.360	11.760	44.120	12.840	19.220	93.490
120	original	0.6	60x8 weeks	Ag80	M	VAC70	25	All	0.993	0.007	0.002	0.009	0.003	0.004	0.019	476.492	3.236	1.176	4.412	1.284	1.922	9.349	4765	32.360	11.760	44.120	12.840	19.220	93.490
120	original	1	60x8 weeks	Ag80	M	VAC70	25	All	0.991	0.006	0.005	0.011	0.003	0.005	0.019	475.667	3.097	2.310	5.407	1.561	2.396	9.160	4757	30.970	23.100	54.070	15.610	23.960	91.600
120	original	1	60x8 weeks	Ag80	M	VAC70	25	All	0.991	0.006	0.005	0.011	0.003	0.005	0.019	475.667	3.097	2.310	5.407	1.561	2.396	9.160	4757	30.970	23.100	54.070	15.610	23.960	91.600
120	original	1	60x8 weeks	Ag80	M	VAC70	25	All	0.991	0.006	0.005	0.011	0.003	0.005	0.019	475.667	3.097	2.310	5.407	1.561	2.396	9.160	4757	30.970	23.100	54.070	15.610	23.960	91.600
120	original	1.2	60x8 weeks	Ag80	M	VAC70	25	All	0.989	0.006	0.006	0.013	0.004	0.006	0.019	474.940	3.090	3.093	6.183	1.836	2.731	9.155	4749	30.900	30.930	61.830	18.360	27.310	91.550
120	original	1.2	60x8 weeks	Ag80	M	VAC70	25	All	0.989	0.006	0.006	0.013	0.004	0.006	0.019	474.940	3.090	3.093	6.183	1.836	2.731	9.155	4749	30.900	30.930	61.830	18.360	27.310	91.550
120	original	1.2	60x8 weeks	Ag80	M	VAC70	25	All	0.989	0.006	0.006	0.013	0.004	0.006	0.019	474.940	3.090	3.093	6.183	1.836	2.731	9.155	4749	30.900	30.930	61.830	18.360	27.310	91.550
120	alpha	0.6	60x8 weeks	Ag80	M	VAC70	25	All	0.987	0.007	0.007	0.014	0.004	0.008	0.019	473.525	3.236	3.588	6.824	2.074	3.865	9.045	4735	32.360	35.880	68.240	20.740	38.650	90.450
120	alpha	0.6	60x8 weeks	Ag80	M	VAC70	25	All	0.987	0.007	0.007	0.014	0.004	0.008	0.019	473.525	3.236	3.588	6.824	2.074	3.865	9.045	4735	32.360	35.880	68.240	20.740	38.650	90.450
120	alpha	0.6	60x8 weeks	Ag80	M	VAC70	25	All	0.987	0.007	0.007	0.014	0.004	0.008	0.019	473.525	3.236	3.588	6.824	2.074	3.865	9.045	4735	32.360	35.880	68.240	20.740	38.650	90.450
120	alpha	1	60x8 weeks	Ag80	M	VAC70	25	All	0.975	0.006	0.021	0.027	0.008	0.015	0.018	467.932	2.982	9.847	12.829	3.852	7.393	8.807	4679	29.820	98.470	128.290	38.520	73.930	88.070
120	alpha	1	60x8 weeks	Ag80	M	VAC70	25	All	0.975	0.006	0.021	0.027	0.008	0.015	0.018	467.932	2.982	9.847	12.829	3.852	7.393	8.807	4679	29.820	98.470	128.290	38.520	73.930	88.070
120	alpha	1	60x8 weeks	Ag80	M	VAC70	25	All	0.975	0.006	0.021	0.027	0.008	0.015	0.018	467.932	2.982	9.847	12.829	3.852	7.393	8.807	4679	29.820	98.470	128.290	38.520	73.930	88.070
120	alpha	1.2	60x8 weeks	Ag80	M	VAC70	25	All	0.964	0.006	0.031	0.037	0.011	0.022	0.017	462.698	2.847	14.920	17.767	5.476	10.323	8.396	4627	28.470	149.200	177.670	54.760	103.230	83.960
120	alpha	1.2	60x8 weeks	Ag80	M	VAC70	25	All	0.964	0.006	0.031	0.037	0.011	0.022	0.017	462.698	2.847	14.920	17.767	5.476	10.323	8.396	4627	28.470	149.200	177.670	54.760	103.230	83.960
120	alpha	1.2	60x8 weeks	Ag80	M	VAC70	25	All	0.964	0.006	0.031	0.037	0.011	0.022	0.017	462.698	2.847	14.920	17.767	5.476	10.323	8.396	4627	28.470	149.200	177.670	54.760	103.230	83.960
120	delta	0.6	60x8 weeks	Ag80	M	VAC70	25	All	0.957	0.006	0.037	0.043	0.014	0.025	0.017	459.580	2.829	17.854	20.683	6.720	11.963	8.066	4596	28.290	178.540	206.830	67.200	119.630	80.660
120	delta	0.6	60x8 weeks	Ag80	M	VAC70	25	All	0.957	0.006	0.037	0.043	0.014	0.025	0.017	459.580	2.829	17.854	20.683	6.720	11.963	8.066	4596	28.290	178.540	206.830	67.200	119.630	80.660
120	delta	0.6	60x8 weeks	Ag80	M	VAC70	25	All	0.957	0.006	0.037	0.043	0.014	0.025	0.017	459.580	2.829	17.854	20.683	6.720	11.963	8.066	4596	28.290	178.540	206.830	67.200	119.630	80.660
120	delta	1	60x8 weeks	Ag80	M	VAC70	25	All	0.901	0.004	0.088	0.092	0.031	0.055	0.013	432.596	2.038	42.101	44.139	14.803	26.382	6.026	4326	20.380	421.010	441.390	148.030	263.820	60.260
120	delta	1	60x8 weeks	Ag80	M	VAC70	25	All	0.901	0.004	0.088	0.092	0.031	0.055	0.013	432.596	2.038	42.101	44.139	14.803	26.382	6.026	4326	20.380	421.010	441.390	148.030	263.820	60.260
120	delta	1	60x8 weeks	Ag80	M	VAC70	25	All	0.901	0.004	0.088	0.092	0.031	0.055	0.013	432.596	2.038	42.101	44.139	14.803	26.382	6.026	4326	20.380	421.010	441.390	148.030	263.820	60.260
120	delta	1.2	60x8 weeks	Ag80	M	VAC70	25	All	0.888	0.004	0.098	0.102	0.034	0.062	0.011	426.103	1.853	47.233	49.086	16.553	29.623	5.330	4261	18.530	472.330	490.860	165.530	296.230	53.300
120	delta	1.2	60x8 weeks	Ag80	M	VAC70	25	All	0.888	0.004	0.098	0.102	0.034	0.062	0.011	426.103	1.853	47.233	49.086	16.553	29.623	5.330	4261	18.530	472.330	490.860	165.530	296.230	53.300
120	delta	1.2	60x8 weeks	Ag80	M	VAC70	25	All	0.888	0.004	0.098	0.102	0.034	0.062	0.011	426.103	1.853	47.233	49.086	16.553	29.623	5.330	4261	18.530	472.330	490.860	165.530	296.230	53.300
120	original	0.6	60x8 weeks	Ag80	M	VAC70	50	All	0.995	0.005	0.001	0.007	0.002	0.002	0.019	477.493	2.587	0.570	3.157	1.072	1.125	9.354	4775	25.870	5.700	31.570	10.720	11.250	93.540
120	original	0.6	60x8 weeks	Ag80	M	VAC70	50	All	0.995	0.005	0.001	0.007	0.002	0.002	0.019	477.493	2.587	0.570	3.157	1.072	1.125	9.354	4775	25.870	5.700	31.570	10.720	11.250	93.540
120	original	0.6	60x8 weeks	Ag80	M	VAC70	50	All	0.995	0.005	0.001	0.007	0.002	0.002	0.019	477.493	2.587	0.570	3.157	1.072	1.125	9.354	4775	25.870	5.700	31.570	10.720	11.250	93.540
120	original	1	60x8 weeks	Ag80	M	VAC70	50	All	0.994	0.005	0.002	0.007	0.002	0.003	0.019	477.304	2.485	1.047	3.532	1.122	1.293	9.338	4773	24.850	10.470	35.320	11.220	12.930	93.380
120	original	1	60x8 weeks	Ag80	M	VAC70	50	All	0.994	0.005	0.002	0.007	0.002	0.003	0.019	477.304	2.485	1.047	3.532	1.122	1.293	9.338	4773	24.850	10.470	35.320	11.220	12.930	93.380
120	original	1	60x8 weeks	Ag80	M	VAC70	50	All	0.994	0.005	0.002	0.007	0.002	0.003	0.019	477.304	2.485	1.047	3.532	1.122	1.293	9.338	4773	24.850	10.470	35.320	11.220	12.930	93.380
120	original	1.2	60x8 weeks	Ag80	M	VAC70	50	All	0.993	0.006	0.003	0.008	0.003	0.003	0.020	476.846	2.644	1.425	4.069	1.320	1.536	9.420	4768	26.440	14.250	40.690	13.200	15.360	94.200
120	original	1.2	60x8 weeks	Ag80	M	VAC70	50	All	0.993	0.006	0.003	0.008	0.003	0.003	0.020	476.846	2.644	1.425	4.069	1.320	1.536	9.420	4768	26.440	14.250	40.690	13.200	15.360	94.200
120	original	1.2	60x8 weeks	Ag80	M	VAC70	50	All	0.993	0.006	0.003	0.008	0.003	0.003	0.020	476.846	2.644	1.425	4.069	1.320	1.536	9.420	4768	26.440	14.250	40.690	13.200	15.360	94.200
120	alpha	0.6	60x8 weeks	Ag80	M	VAC70	50	All	0.992	0.005	0.003	0.009	0.003	0.005	0.019	476.058	2.543	1.674	4.217	1.290	2.357	9.285	4761	25.430	16.740	42.170	12.900	23.570	92.850
120	alpha	0.6	60x8 weeks	Ag80	M	VAC70	50	All	0.992	0.005	0.003	0.009	0.003	0.005	0.019	476.058	2.543	1.674	4.217	1.290	2.357	9.285	4761	25.430	16.740	42.170	12.900	23.570	92.850
120	alpha	0.6	60x8 weeks	Ag80	M	VAC70	50	All	0.992	0.005	0.003	0.009	0.003	0.005	0.019	476.058	2.543	1.674	4.217	1.290	2.357	9.285	4761	25.430	16.740	42.170	12.900	23.570	92.850
120	alpha	1	60x8 weeks	Ag80	M	VAC70	50	All	0.987	0.005	0.009	0.014	0.004	0.008	0.019	473.608	2.473	4.191	6.664	2.061	3.750	9.173	4736	24.730	41.910	66.640	20.610	37.500	91.730
120	alpha	1	60x8 weeks	Ag80	M	VAC70	50	All	0.987	0.005	0.009	0.014	0.004	0.008	0.019	473.608	2.473	4.191	6.664	2.061	3.750	9.173	4736	24.730	41.910	66.640	20.610	37.500	91.730
120	alpha	1	60x8 weeks	Ag80	M	VAC70	50	All	0.987	0.005	0.009	0.014	0.004	0.008	0.019	473.608	2.473	4.191	6.664	2.061	3.750	9.173	4736	24.730	41.910	66.640	20.610	37.500	91.730
120	alpha	1.2	60x8 weeks	Ag80	M	VAC70	50	All	0.985	0.005	0.012	0.017	0.005	0.009	0.019	472.602	2.410	5.653	8.063	2.443	4.496	8.996	4726	24.100	56.530	80.630	24.430	44.960	89.960
120	alpha	1.2	60x8 weeks	Ag80	M	VAC70	50	All	0.985	0.005	0.012	0.017	0.005	0.009	0.019	472.602	2.410	5.653	8.063	2.443	4.496	8.996	4726	24.100	56.530	80.630	24.430	44.960	89.960
120	alpha	1.2	60x8 weeks	Ag80	M	VAC70	50	All	0.985	0.005	0.012	0.017	0.005	0.009	0.019	472.602	2.410	5.653	8.063	2.443	4.496	8.996	4726	24.100	56.530	80.630	24.430	44.960	89.960
120	delta	0.6	60x8 weeks	Ag80	M	VAC70	50	All	0.981	0.005	0.014	0.020	0.006	0.011	0.019	470.978	2.514	6.902	9.416	3.038	5.272	8.978	4710	25.140	69.020	94.160	30.380	52.720	89.780
120	delta	0.6	60x8 weeks	Ag80	M	VAC70	50	All	0.981	0.005	0.014	0.020	0.006	0.011	0.019	470.978	2.514	6.902	9.416	3.038	5.272	8.978	4710	25.140	69.020	94.160	30.380	52.720	89.780
120	delta	0.6	60x8 weeks	Ag80	M	VAC70	50	All	0.981	0.005	0.014	0.020	0.006	0.011	0.019	470.978	2.514	6.902	9.416	3.038	5.272	8.978	4710	25.140	69.020	94.160	30.380	52.720	89.780
120	delta	1	60x8 weeks	Ag80	M	VAC70	50	All	0.947	0.005	0.048	0.052	0.017	0.030	0.016	454.534	2.202	22.904	25.106	8.320	14.400	7.744	4545	22.020	229.040	251.060	83.200	144.000	77.440
120	delta	1	60x8 weeks	Ag80	M	VAC70	50	All	0.947	0.005	0.048	0.052	0.017	0.030	0.016	454.534	2.202	22.904	25.106	8.320	14.400	7.744	4545	22.020	229.040	251.060	83.200	144.000	77.440
120	delta	1	60x8 weeks	Ag80	M	VAC70	50	All	0.947	0.005	0.048	0.052	0.017	0.030	0.016	454.534	2.202	22.904	25.106	8.320	14.400	7.744	4545	22.020	229.040	251.060	83.200	144.000	77.440
120	delta	1.2	60x8 weeks	Ag80	M	VAC70	50	All	0.926	0.004	0.068	0.073	0.024	0.042	0.014	444.356	2.001	32.836	34.837	11.479	20.362	6.947	4444	20.010	328.360	348.370	114.790	203.620	69.470
120	delta	1.2	60x8 weeks	Ag80	M	VAC70	50	All	0.926	0.004	0.068	0.073	0.024	0.042	0.014	444.356	2.001	32.836	34.837	11.479	20.362	6.947	4444	20.010	328.360	348.370	114.790	203.620	69.470
120	delta	1.2	60x8 weeks	Ag80	M	VAC70	50	All	0.926	0.004	0.068	0.073	0.024	0.042	0.014	444.356	2.001	32.836	34.837	11.479	20.362	6.947	4444	20.010	328.360	348.370	114.790	203.620	69.470
120	original	0.6	60x8 weeks	Ag80	M	VAC70	75	All	0.997	0.004	0.000	0.005	0.002	0.001	0.019	478.398	2.037	0.186	2.223	0.814	0.596	9.315	4784	20.370	1.860	22.230	8.140	5.960	93.150
120	original	0.6	60x8 weeks	Ag80	M	VAC70	75	All	0.997	0.004	0.000	0.005	0.002	0.001	0.019	478.398	2.037	0.186	2.223	0.814	0.596	9.315	4784	20.370	1.860	22.230	8.140	5.960	93.150
120	original	0.6	60x8 weeks	Ag80	M	VAC70	75	All	0.997	0.004	0.000	0.005	0.002	0.001	0.019	478.398	2.037	0.186	2.223	0.814	0.596	9.315	4784	20.370	1.860	22.230	8.140	5.960	93.150
120	original	1	60x8 weeks	Ag80	M	VAC70	75	All	0.996	0.004	0.001	0.005	0.002	0.001	0.020	478.294	1.931	0.385	2.316	0.901	0.550	9.380	4783	19.310	3.850	23.160	9.010	5.500	93.800
120	original	1	60x8 weeks	Ag80	M	VAC70	75	All	0.996	0.004	0.001	0.005	0.002	0.001	0.020	478.294	1.931	0.385	2.316	0.901	0.550	9.380	4783	19.310	3.850	23.160	9.010	5.500	93.800
120	original	1	60x8 weeks	Ag80	M	VAC70	75	All	0.996	0.004	0.001	0.005	0.002	0.001	0.020	478.294	1.931	0.385	2.316	0.901	0.550	9.380	4783	19.310	3.850	23.160	9.010	5.500	93.800
120	original	1.2	60x8 weeks	Ag80	M	VAC70	75	All	0.996	0.004	0.001	0.005	0.002	0.001	0.019	478.306	1.981	0.415	2.396	0.912	0.587	9.349	4783	19.810	4.150	23.960	9.120	5.870	93.490
120	original	1.2	60x8 weeks	Ag80	M	VAC70	75	All	0.996	0.004	0.001	0.005	0.002	0.001	0.019	478.306	1.981	0.415	2.396	0.912	0.587	9.349	4783	19.810	4.150	23.960	9.120	5.870	93.490
120	original	1.2	60x8 weeks	Ag80	M	VAC70	75	All	0.996	0.004	0.001	0.005	0.002	0.001	0.019	478.306	1.981	0.415	2.396	0.912	0.587	9.349	4783	19.810	4.150	23.960	9.120	5.870	93.490

120	alpha	0.6	60x8 weeks	Ag80	M	VAC70	75	All	0.995	0.004	0.001	0.005	0.002	0.003	0.019	477.679	1.884	0.672	2.556	0.755	1.385	9.288	4777	18.840	6.720	25.560	7.550	13.850	92.880
120	alpha	0.6	60x8 weeks	Ag80	M	VAC70	75	All	0.995	0.004	0.001	0.005	0.002	0.003	0.019	477.679	1.884	0.672	2.556	0.755	1.385	9.288	4777	18.840	6.720	25.560	7.550	13.850	92.880
120	alpha	0.6	60x8 weeks	Ag80	M	VAC70	75	All	0.995	0.004	0.001	0.005	0.002	0.003	0.019	477.679	1.884	0.672	2.556	0.755	1.385	9.288	4777	18.840	6.720	25.560	7.550	13.850	92.880
120	alpha	1	60x8 weeks	Ag80	M	VAC70	75	All	0.994	0.004	0.003	0.007	0.002	0.003	0.019	477.018	1.844	1.363	3.207	1.023	1.676	9.268	4770	18.440	13.630	32.070	10.230	16.760	92.680
120	alpha	1	60x8 weeks	Ag80	M	VAC70	75	All	0.994	0.004	0.003	0.007	0.002	0.003	0.019	477.018	1.844	1.363	3.207	1.023	1.676	9.268	4770	18.440	13.630	32.070	10.230	16.760	92.680
120	alpha	1	60x8 weeks	Ag80	M	VAC70	75	All	0.994	0.004	0.003	0.007	0.002	0.003	0.019	477.018	1.844	1.363	3.207	1.023	1.676	9.268	4770	18.440	13.630	32.070	10.230	16.760	92.680
120	alpha	1.2	60x8 weeks	Ag80	M	VAC70	75	All	0.993	0.004	0.004	0.008	0.002	0.004	0.020	476.498	1.886	1.952	3.838	1.140	2.141	9.445	4765	18.860	19.520	38.380	11.400	21.410	94.450
120	alpha	1.2	60x8 weeks	Ag80	M	VAC70	75	All	0.993	0.004	0.004	0.008	0.002	0.004	0.020	476.498	1.886	1.952	3.838	1.140	2.141	9.445	4765	18.860	19.520	38.380	11.400	21.410	94.450
120	alpha	1.2	60x8 weeks	Ag80	M	VAC70	75	All	0.993	0.004	0.004	0.008	0.002	0.004	0.020	476.498	1.886	1.952	3.838	1.140	2.141	9.445	4765	18.860	19.520	38.380	11.400	21.410	94.450
120	delta	0.6	60x8 weeks	Ag80	M	VAC70	75	All	0.990	0.004	0.005	0.010	0.003	0.005	0.019	475.400	2.137	2.593	4.730	1.584	2.589	9.153	4754	21.370	25.930	47.300	15.840	25.890	91.530
120	delta	0.6	60x8 weeks	Ag80	M	VAC70	75	All	0.990	0.004	0.005	0.010	0.003	0.005	0.019	475.400	2.137	2.593	4.730	1.584	2.589	9.153	4754	21.370	25.930	47.300	15.840	25.890	91.530
120	delta	0.6	60x8 weeks	Ag80	M	VAC70	75	All	0.990	0.004	0.005	0.010	0.003	0.005	0.019	475.400	2.137	2.593	4.730	1.584	2.589	9.153	4754	21.370	25.930	47.300	15.840	25.890	91.530
120	delta	1	60x8 weeks	Ag80	M	VAC70	75	All	0.979	0.004	0.018	0.023	0.007	0.012	0.019	469.779	2.121	8.718	10.839	3.550	5.890	8.951	4698	21.210	87.180	108.390	35.500	58.900	89.510
120	delta	1	60x8 weeks	Ag80	M	VAC70	75	All	0.979	0.004	0.018	0.023	0.007	0.012	0.019	469.779	2.121	8.718	10.839	3.550	5.890	8.951	4698	21.210	87.180	108.390	35.500	58.900	89.510
120	delta	1	60x8 weeks	Ag80	M	VAC70	75	All	0.979	0.004	0.018	0.023	0.007	0.012	0.019	469.779	2.121	8.718	10.839	3.550	5.890	8.951	4698	21.210	87.180	108.390	35.500	58.900	89.510
120	delta	1.2	60x8 weeks	Ag80	M	VAC70	75	All	0.971	0.004	0.027	0.031	0.010	0.017	0.018	466.138	1.916	12.848	14.764	4.688	8.262	8.747	4661	19.160	128.480	147.640	46.880	82.620	87.470
120	delta	1.2	60x8 weeks	Ag80	M	VAC70	75	All	0.971	0.004	0.027	0.031	0.010	0.017	0.018	466.138	1.916	12.848	14.764	4.688	8.262	8.747	4661	19.160	128.480	147.640	46.880	82.620	87.470
120	delta	1.2	60x8 weeks	Ag80	M	VAC70	75	All	0.971	0.004	0.027	0.031	0.010	0.017	0.018	466.138	1.916	12.848	14.764	4.688	8.262	8.747	4661	19.160	128.480	147.640	46.880	82.620	87.470
120	original	0.6	60x8 weeks	Ag80	M	VAC70	100	All	0.998	0.003	0.000	0.003	0.001	0.000	0.020	479.155	1.360	0.007	1.367	0.646	0.054	9.575	4792	13.600	0.070	13.670	6.460	0.540	95.750
120	original	0.6	60x8 weeks	Ag80	M	VAC70	100	All	0.998	0.003	0.000	0.003	0.001	0.000	0.020	479.155	1.360	0.007	1.367	0.646	0.054	9.575	4792	13.600	0.070	13.670	6.460	0.540	95.750
120	original	0.6	60x8 weeks	Ag80	M	VAC70	100	All	0.998	0.003	0.000	0.003	0.001	0.000	0.020	479.155	1.360	0.007	1.367	0.646	0.054	9.575	4792	13.600	0.070	13.670	6.460	0.540	95.750
120	original	1	60x8 weeks	Ag80	M	VAC70	100	All	0.998	0.003	0.000	0.003	0.001	0.000	0.020	479.251	1.320	0.018	1.338	0.590	0.032	9.572	4793	13.200	0.180	13.380	5.900	0.320	95.720
120	original	1	60x8 weeks	Ag80	M	VAC70	100	All	0.998	0.003	0.000	0.003	0.001	0.000	0.020	479.251	1.320	0.018	1.338	0.590	0.032	9.572	4793	13.200	0.180	13.380	5.900	0.320	95.720
120	original	1	60x8 weeks	Ag80	M	VAC70	100	All	0.998	0.003	0.000	0.003	0.001	0.000	0.020	479.251	1.320	0.018	1.338	0.590	0.032	9.572	4793	13.200	0.180	13.380	5.900	0.320	95.720
120	original	1.2	60x8 weeks	Ag80	M	VAC70	100	All	0.998	0.003	0.000	0.003	0.001	0.000	0.020	479.082	1.341	0.022	1.363	0.660	0.047	9.529	4791	13.410	0.220	13.630	6.600	0.470	95.290
120	original	1.2	60x8 weeks	Ag80	M	VAC70	100	All	0.998	0.003	0.000	0.003	0.001	0.000	0.020	479.082	1.341	0.022	1.363	0.660	0.047	9.529	4791	13.410	0.220	13.630	6.600	0.470	95.290
120	original	1.2	60x8 weeks	Ag80	M	VAC70	100	All	0.998	0.003	0.000	0.003	0.001	0.000	0.020	479.082	1.341	0.022	1.363	0.660	0.047	9.529	4791	13.410	0.220	13.630	6.600	0.470	95.290
120	alpha	0.6	60x8 weeks	Ag80	M	VAC70	100	All	0.997	0.003	0.000	0.003	0.001	0.001	0.020	478.720	1.257	0.159	1.416	0.469	0.690	9.485	4787	12.570	1.590	14.160	4.690	6.900	94.850
120	alpha	0.6	60x8 weeks	Ag80	M	VAC70	100	All	0.997	0.003	0.000	0.003	0.001	0.001	0.020	478.720	1.257	0.159	1.416	0.469	0.690	9.485	4787	12.570	1.590	14.160	4.690	6.900	94.850
120	alpha	0.6	60x8 weeks	Ag80	M	VAC70	100	All	0.997	0.003	0.000	0.003	0.001	0.001	0.020	478.720	1.257	0.159	1.416	0.469	0.690	9.485	4787	12.570	1.590	14.160	4.690	6.900	94.850
120	alpha	1	60x8 weeks	Ag80	M	VAC70	100	All	0.997	0.003	0.001	0.003	0.001	0.002	0.020	478.470	1.342	0.337	1.679	0.513	0.890	9.415	4785	13.420	3.370	16.790	5.130	8.900	94.150
120	alpha	1	60x8 weeks	Ag80	M	VAC70	100	All	0.997	0.003	0.001	0.003	0.001	0.002	0.020	478.470	1.342	0.337	1.679	0.513	0.890	9.415	4785	13.420	3.370	16.790	5.130	8.900	94.150
120	alpha	1	60x8 weeks	Ag80	M	VAC70	100	All	0.997	0.003	0.001	0.003	0.001	0.002	0.020	478.470	1.342	0.337	1.679	0.513	0.890	9.415	4785	13.420	3.370	16.790	5.130	8.900	94.150
120	alpha	1.2	60x8 weeks	Ag80	M	VAC70	100	All	0.997	0.003	0.001	0.004	0.001	0.002	0.020	478.371	1.342	0.461	1.803	0.557	0.947	9.496	4784	13.420	4.610	18.030	5.570	9.470	94.960
120	alpha	1.2	60x8 weeks	Ag80	M	VAC70	100	All	0.997	0.003	0.001	0.004	0.001	0.002	0.020	478.371	1.342	0.461	1.803	0.557	0.947	9.496	4784	13.420	4.610	18.030	5.570	9.470	94.960
120	alpha	1.2	60x8 weeks	Ag80	M	VAC70	100	All	0.997	0.003	0.001	0.004	0.001	0.002	0.020	478.371	1.342	0.461	1.803	0.557	0.947	9.496	4784	13.420	4.610	18.030	5.570	9.470	94.960
120	delta	0.6	60x8 weeks	Ag80	M	VAC70	100	All	0.995	0.003	0.001	0.005	0.002	0.003	0.020	477.824	1.617	0.684	2.301	0.757	1.231	9.418	4778	16.170	6.840	23.010	7.570	12.310	94.180
120	delta	0.6	60x8 weeks	Ag80	M	VAC70	100	All	0.995	0.003	0.001	0.005	0.002	0.003	0.020	477.824	1.617	0.684	2.301	0.757	1.231	9.418	4778	16.170	6.840	23.010	7.570	12.310	94.180
120	delta	0.6	60x8 weeks	Ag80	M	VAC70	100	All	0.995	0.003	0.001	0.005	0.002	0.003	0.020	477.824	1.617	0.684	2.301	0.757	1.231	9.418	4778	16.170	6.840	23.010	7.570	12.310	94.180
120	delta	1	60x8 weeks	Ag80	M	VAC70	100	All	0.994	0.003	0.004	0.007	0.002	0.004	0.019	476.932	1.568	1.688	3.256	1.056	1.774	9.336	4769	15.680	16.880	32.560	10.560	17.740	93.360
120	delta	1	60x8 weeks	Ag80	M	VAC70	100	All	0.994	0.003	0.004	0.007	0.002	0.004	0.019	476.932	1.568	1.688	3.256	1.056	1.774	9.336	4769	15.680	16.880	32.560	10.560	17.740	93.360
120	delta	1	60x8 weeks	Ag80	M	VAC70	100	All	0.994	0.003	0.004	0.007	0.002	0.004	0.019	476.932	1.568	1.688	3.256	1.056	1.774	9.336	4769	15.680	16.880	32.560	10.560	17.740	93.360
120	delta	1.2	60x8 weeks	Ag80	M	VAC70	100	All	0.992	0.003	0.005	0.009	0.003	0.005	0.019	476.056	1.652	2.500	4.152	1.324	2.263	9.203	4761	16.520	25.000	41.520	13.240	22.630	92.030
120	delta	1.2	60x8 weeks	Ag80	M	VAC70	100	All	0.992	0.003	0.005	0.009	0.003	0.005	0.019	476.056	1.652	2.500	4.152	1.324	2.263	9.203	4761	16.520	25.000	41.520	13.240	22.630	92.030
120	delta	1.2	60x8 weeks	Ag80	M	VAC70	100	All	0.992	0.003	0.005	0.009	0.003	0.005	0.019	476.056	1.652	2.500	4.152	1.324	2.263	9.203	4761	16.520	25.000	41.520	13.240	22.630	92.030
120	original	0.6	60x8 weeks	Ag80	M	VAC50	25	All	0.992	0.007	0.003	0.010	0.003	0.004	0.019	476.268	3.446	1.262	4.708	1.454	1.923	9.143	4763	34.460	12.620	47.080	14.540	19.230	91.430
120	original	0.6	60x8 weeks	Ag80	M	VAC50	25	All	0.992	0.007	0.003	0.010	0.003	0.004	0.019	476.268	3.446	1.262	4.708	1.454	1.923	9.143	4763	34.460	12.620	47.080	14.540	19.230	91.430
120	original	0.6	60x8 weeks	Ag80	M	VAC50	25	All	0.992	0.007	0.003	0.010	0.003	0.004	0.019	476.268	3.446	1.262	4.708	1.454	1.923	9.143	4763	34.460	12.620	47.080	14.540	19.230	91.430
120	original	1	60x8 weeks	Ag80	M	VAC50	25	All	0.990	0.007	0.005	0.012	0.004	0.005	0.019	475.283	3.276	2.629	5.905	1.847	2.449	9.129	4753	32.760	26.290	59.050	18.470	24.490	91.290
120	original	1	60x8 weeks	Ag80	M	VAC50	25	All	0.990	0.007	0.005	0.012	0.004	0.005	0.019	475.283	3.276	2.629	5.905	1.847	2.449	9.129	4753	32.760	26.290	59.050	18.470	24.490	91.290
120	original	1	60x8 weeks	Ag80	M	VAC50	25	All	0.990	0.007	0.005	0.012	0.004	0.005	0.019	475.283	3.276	2.629	5.905	1.847	2.449	9.129	4753	32.760	26.290	59.050	18.470	24.490	91.290
120	original	1.2	60x8 weeks	Ag80	M	VAC50	25	All	0.988	0.007	0.008	0.014	0.004	0.006	0.019	474.366	3.333	3.620	6.953	2.145	2.898	8.885	4744	33.330	36.200	69.530	21.450	28.980	88.850
120	original	1.2	60x8 weeks	Ag80	M	VAC50	25	All	0.988	0.007	0.008	0.014	0.004	0.006	0.019	474.366	3.333	3.620	6.953	2.145	2.898	8.885	4744	33.330	36.200	69.530	21.450	28.980	88.850
120	original	1.2	60x8 weeks	Ag80	M	VAC50	25	All	0.988	0.007	0.008	0.014	0.004	0.006	0.019	474.366	3.333	3.620	6.953	2.145	2.898	8.885	4744	33.330	36.200	69.530	21.450	28.980	88.850
120	alpha	0.6	60x8 weeks	Ag80	M	VAC50	25	All	0.985	0.007	0.008	0.015	0.005	0.009	0.019	472.895	3.378	4.060	7.438	2.302	4.194	9.100	4729	33.780	40.600	74.380	23.020	41.940	91.000
120	alpha	0.6	60x8 weeks	Ag80	M	VAC50	25	All	0.985	0.007	0.008	0.015	0.005	0.009	0.019	472.895	3.378	4.060	7.438	2.302	4.194	9.100	4729	33.780	40.600	74.380	23.020	41.940	91.000
120	alpha	0.6	60x8 weeks	Ag80	M	VAC50	25	All	0.985	0.007	0.008	0.015	0.005	0.009	0.019	472.895	3.378	4.060	7.438	2.302	4.194	9.100	4729	33.780	40.600	74.380	23.020	41.940	91.000
120	alpha	1	60x8 weeks	Ag80	M	VAC50	25	All	0.972	0.006	0.023	0.029	0.009	0.017	0.018	466.462	3.064	11.084	14.148	4.304	8.123	8.449	4665	30.640	110.840	141.480	43.040	81.230	84.490
120	alpha	1	60x8 weeks	Ag80	M	VAC50	25	All	0.972	0.006	0.023	0.029	0.009	0.017	0.018	466.462	3.064	11.084	14.148	4.304	8.123	8.449	4665	30.640	110.840	141.480	43.040	81.230	84.490
120	alpha	1	60x8 weeks	Ag80	M	VAC50	25	All	0.972	0.006	0.023	0.029	0.009	0.017	0.018	466.462	3.064	11.084	14.148	4.304	8.123	8.449	4665	30.640	110.840	141.480	43.040	81.230	84.490
120	alpha	1.2	60x8 weeks	Ag80	M	VAC50	25	All	0.959	0.006	0.036	0.042	0.013	0.025	0.017	460.105	2.883	17.443	20.326	6.291	11.954	8.175	4601	28.830	174.430	203.260	62.910	119.540	81.750
120	alpha	1.2	60x8 weeks	Ag80	M	VAC50	25	All	0.959	0.006	0.036	0.042	0.013	0.025	0.017	460.105	2.883	17.443	20.326	6.291	11.954	8.175	4601	28.830	174.430	203.260	62.910	119.540	81.750
120	alpha	1.2	60x8 weeks	Ag80	M	VAC50	25	All	0.959	0.006	0.036	0.042	0.013	0.025	0.017	460.105	2.883	17.443	20.326	6.291	11.954	8.175	4601	28.830	174.430	203.260	62.910	119.540	81.750
120	delta	0.6	60x8 weeks	Ag80	M	VAC50	25	All	0.947	0.006	0.046	0.052	0.017	0.030	0.016	454.661	2.867	21.982	24.849	8.374	14.292	7.853	4547	28.670	219.820	248.490	83.740	142.920	78.530
120	delta	0.6	60x8 weeks	Ag80	M	VAC50	25	All	0.947	0.006	0.046	0.052	0.017	0.030	0.016	454.661	2.867	21.982	24.849	8.374	14.292	7.853	4547	28.670	219.820	248.490	83.740	142.920	78.530
120	delta	0.6	60x8 weeks	Ag80	M	VAC50	25	All	0.947	0.006	0.046	0.052	0.017	0.030	0.016	454.661	2.867	21.982	24.849	8.374	14.292	7.853	4547	28.670	219.820	248.490	83.740	142.920	78.530
120	delta	1	60x8 weeks	Ag80	M	VAC50	25	All	0.890	0.004	0.097	0.101	0.035	0.060	0.012	427.410	2.134	46.498	48.632	16.564	28.870	5.622	4274	21.340	464.980	486.320	165.640	288.700	56.220
120	delta	1	60x8 weeks	Ag80	M	VAC50	25	All	0.890	0.004	0.097	0.101	0.035	0.060	0.012	427.410	2.134	46.498	48.632	16.564	28.870	5.622	4274	21.340	464.980	486.320	165.640	288.700	56.220
120	delta	1	60x8 weeks	Ag80	M	VAC50	25	All	0.890	0.004	0.097	0.101	0.035	0.060	0.012	427.410	2.134	46.498	48.632	16.564	28.870	5.622	4274	21.340	464.980	486.320	165.640	288.700	56.220
120	delta	1.2	60x8 weeks	Ag80	M	VAC50	25	All	0.879	0.004	0.105	0.109	0.038	0.066	0.011	421.722	1.923	50.420	52.343	18.002	31.465	5.066	4217	19.230	504.200	523.430	180.020	314.650	50.660
120	delta	1.2	60x8 weeks	Ag80	M	VAC50	25	All	0.879	0.004	0.105	0.109	0.038	0.066	0.011	421.722	1.923	50.420	52.343	18.002	31.465	5.066	4217	19.230	504.200	523.430	180.020	314.650	50.660

120	delta	1.2	60x8 weeks	Ag80	M	VAC50	25	All	0.879	0.004	0.105	0.109	0.038	0.066	0.011	421.722	1.923	50.420	52.343	18.002	31.465	5.066	4217	19.230	504.200	523.430	180.020	314.650	50.660
120	original	0.6	60x8 weeks	Ag80	M	VAC50	50	All	0.994	0.006	0.001	0.007	0.003	0.002	0.019	477.201	2.877	0.675	3.552	1.345	1.110	9.348	4772	28.770	6.750	35.520	13.450	11.100	93.480
120	original	0.6	60x8 weeks	Ag80	M	VAC50	50	All	0.994	0.006	0.001	0.007	0.003	0.002	0.019	477.201	2.877	0.675	3.552	1.345	1.110	9.348	4772	28.770	6.750	35.520	13.450	11.100	93.480
120	original	0.6	60x8 weeks	Ag80	M	VAC50	50	All	0.994	0.006	0.001	0.007	0.003	0.002	0.019	477.201	2.877	0.675	3.552	1.345	1.110	9.348	4772	28.770	6.750	35.520	13.450	11.100	93.480
120	original	1	60x8 weeks	Ag80	M	VAC50	50	All	0.993	0.006	0.003	0.009	0.003	0.003	0.019	476.682	2.972	1.250	4.222	1.520	1.370	9.275	4767	29.720	12.500	42.220	15.200	13.700	92.750
120	original	1	60x8 weeks	Ag80	M	VAC50	50	All	0.993	0.006	0.003	0.009	0.003	0.003	0.019	476.682	2.972	1.250	4.222	1.520	1.370	9.275	4767	29.720	12.500	42.220	15.200	13.700	92.750
120	original	1	60x8 weeks	Ag80	M	VAC50	50	All	0.993	0.006	0.003	0.009	0.003	0.003	0.019	476.682	2.972	1.250	4.222	1.520	1.370	9.275	4767	29.720	12.500	42.220	15.200	13.700	92.750
120	original	1.2	60x8 weeks	Ag80	M	VAC50	50	All	0.993	0.006	0.003	0.009	0.003	0.003	0.019	476.535	2.915	1.631	4.546	1.628	1.432	9.269	4765	29.150	16.310	45.460	16.280	14.320	92.690
120	original	1.2	60x8 weeks	Ag80	M	VAC50	50	All	0.993	0.006	0.003	0.009	0.003	0.003	0.019	476.535	2.915	1.631	4.546	1.628	1.432	9.269	4765	29.150	16.310	45.460	16.280	14.320	92.690
120	original	1.2	60x8 weeks	Ag80	M	VAC50	50	All	0.993	0.006	0.003	0.009	0.003	0.003	0.019	476.535	2.915	1.631	4.546	1.628	1.432	9.269	4765	29.150	16.310	45.460	16.280	14.320	92.690
120	alpha	0.6	60x8 weeks	Ag80	M	VAC50	50	All	0.990	0.006	0.005	0.011	0.003	0.006	0.019	475.213	2.911	2.203	5.114	1.601	2.811	9.075	4752	29.110	22.030	51.140	16.010	28.110	90.750
120	alpha	0.6	60x8 weeks	Ag80	M	VAC50	50	All	0.990	0.006	0.005	0.011	0.003	0.006	0.019	475.213	2.911	2.203	5.114	1.601	2.811	9.075	4752	29.110	22.030	51.140	16.010	28.110	90.750
120	alpha	0.6	60x8 weeks	Ag80	M	VAC50	50	All	0.990	0.006	0.005	0.011	0.003	0.006	0.019	475.213	2.911	2.203	5.114	1.601	2.811	9.075	4752	29.110	22.030	51.140	16.010	28.110	90.750
120	alpha	1	60x8 weeks	Ag80	M	VAC50	50	All	0.983	0.006	0.012	0.018	0.005	0.010	0.019	471.908	2.877	5.633	8.510	2.622	4.789	9.010	4719	28.770	56.330	85.100	26.220	47.890	90.100
120	alpha	1	60x8 weeks	Ag80	M	VAC50	50	All	0.983	0.006	0.012	0.018	0.005	0.010	0.019	471.908	2.877	5.633	8.510	2.622	4.789	9.010	4719	28.770	56.330	85.100	26.220	47.890	90.100
120	alpha	1	60x8 weeks	Ag80	M	VAC50	50	All	0.983	0.006	0.012	0.018	0.005	0.010	0.019	471.908	2.877	5.633	8.510	2.622	4.789	9.010	4719	28.770	56.330	85.100	26.220	47.890	90.100
120	alpha	1.2	60x8 weeks	Ag80	M	VAC50	50	All	0.978	0.006	0.017	0.023	0.007	0.013	0.019	469.436	2.748	8.393	11.141	3.484	6.177	8.931	4694	27.480	83.930	111.410	34.840	61.770	89.310
120	alpha	1.2	60x8 weeks	Ag80	M	VAC50	50	All	0.978	0.006	0.017	0.023	0.007	0.013	0.019	469.436	2.748	8.393	11.141	3.484	6.177	8.931	4694	27.480	83.930	111.410	34.840	61.770	89.310
120	alpha	1.2	60x8 weeks	Ag80	M	VAC50	50	All	0.978	0.006	0.017	0.023	0.007	0.013	0.019	469.436	2.748	8.393	11.141	3.484	6.177	8.931	4694	27.480	83.930	111.410	34.840	61.770	89.310
120	delta	0.6	60x8 weeks	Ag80	M	VAC50	50	All	0.969	0.006	0.025	0.031	0.010	0.017	0.018	465.228	2.951	11.989	14.940	5.021	8.380	8.548	4652	29.510	119.890	149.400	50.210	83.800	85.480
120	delta	0.6	60x8 weeks	Ag80	M	VAC50	50	All	0.969	0.006	0.025	0.031	0.010	0.017	0.018	465.228	2.951	11.989	14.940	5.021	8.380	8.548	4652	29.510	119.890	149.400	50.210	83.800	85.480
120	delta	0.6	60x8 weeks	Ag80	M	VAC50	50	All	0.969	0.006	0.025	0.031	0.010	0.017	0.018	465.228	2.951	11.989	14.940	5.021	8.380	8.548	4652	29.510	119.890	149.400	50.210	83.800	85.480
120	delta	1	60x8 weeks	Ag80	M	VAC50	50	All	0.919	0.005	0.074	0.079	0.026	0.046	0.014	440.883	2.350	35.686	38.036	12.619	22.101	6.643	4409	23.500	356.860	380.360	126.190	221.010	66.430
120	delta	1	60x8 weeks	Ag80	M	VAC50	50	All	0.919	0.005	0.074	0.079	0.026	0.046	0.014	440.883	2.350	35.686	38.036	12.619	22.101	6.643	4409	23.500	356.860	380.360	126.190	221.010	66.430
120	delta	1	60x8 weeks	Ag80	M	VAC50	50	All	0.919	0.005	0.074	0.079	0.026	0.046	0.014	440.883	2.350	35.686	38.036	12.619	22.101	6.643	4409	23.500	356.860	380.360	126.190	221.010	66.430
120	delta	1.2	60x8 weeks	Ag80	M	VAC50	50	All	0.900	0.004	0.091	0.096	0.032	0.056	0.013	432.237	2.130	43.809	45.939	15.451	26.896	6.101	4322	21.300	438.090	459.390	154.510	268.960	61.010
120	delta	1.2	60x8 weeks	Ag80	M	VAC50	50	All	0.900	0.004	0.091	0.096	0.032	0.056	0.013	432.237	2.130	43.809	45.939	15.451	26.896	6.101	4322	21.300	438.090	459.390	154.510	268.960	61.010
120	delta	1.2	60x8 weeks	Ag80	M	VAC50	50	All	0.900	0.004	0.091	0.096	0.032	0.056	0.013	432.237	2.130	43.809	45.939	15.451	26.896	6.101	4322	21.300	438.090	459.390	154.510	268.960	61.010
120	original	0.6	60x8 weeks	Ag80	M	VAC50	75	All	0.996	0.005	0.001	0.006	0.002	0.001	0.019	478.056	2.537	0.287	2.824	1.118	0.532	9.303	4781	25.370	2.870	28.240	11.180	5.320	93.030
120	original	0.6	60x8 weeks	Ag80	M	VAC50	75	All	0.996	0.005	0.001	0.006	0.002	0.001	0.019	478.056	2.537	0.287	2.824	1.118	0.532	9.303	4781	25.370	2.870	28.240	11.180	5.320	93.030
120	original	0.6	60x8 weeks	Ag80	M	VAC50	75	All	0.996	0.005	0.001	0.006	0.002	0.001	0.019	478.056	2.537	0.287	2.824	1.118	0.532	9.303	4781	25.370	2.870	28.240	11.180	5.320	93.030
120	original	1	60x8 weeks	Ag80	M	VAC50	75	All	0.996	0.005	0.001	0.006	0.003	0.001	0.020	477.928	2.438	0.446	2.884	1.204	0.589	9.396	4779	24.380	4.460	28.840	12.040	5.890	93.960
120	original	1	60x8 weeks	Ag80	M	VAC50	75	All	0.996	0.005	0.001	0.006	0.003	0.001	0.020	477.928	2.438	0.446	2.884	1.204	0.589	9.396	4779	24.380	4.460	28.840	12.040	5.890	93.960
120	original	1	60x8 weeks	Ag80	M	VAC50	75	All	0.996	0.005	0.001	0.006	0.003	0.001	0.020	477.928	2.438	0.446	2.884	1.204	0.589	9.396	4779	24.380	4.460	28.840	12.040	5.890	93.960
120	original	1.2	60x8 weeks	Ag80	M	VAC50	75	All	0.995	0.005	0.001	0.006	0.003	0.001	0.019	477.780	2.481	0.554	3.035	1.234	0.661	9.204	4778	24.810	5.540	30.350	12.340	6.610	92.040
120	original	1.2	60x8 weeks	Ag80	M	VAC50	75	All	0.995	0.005	0.001	0.006	0.003	0.001	0.019	477.780	2.481	0.554	3.035	1.234	0.661	9.204	4778	24.810	5.540	30.350	12.340	6.610	92.040
120	original	1.2	60x8 weeks	Ag80	M	VAC50	75	All	0.995	0.005	0.001	0.006	0.003	0.001	0.019	477.780	2.481	0.554	3.035	1.234	0.661	9.204	4778	24.810	5.540	30.350	12.340	6.610	92.040
120	alpha	0.6	60x8 weeks	Ag80	M	VAC50	75	All	0.993	0.005	0.002	0.007	0.002	0.004	0.019	476.837	2.446	0.999	3.445	1.096	1.778	9.330	4768	24.460	9.990	34.450	10.960	17.780	93.300
120	alpha	0.6	60x8 weeks	Ag80	M	VAC50	75	All	0.993	0.005	0.002	0.007	0.002	0.004	0.019	476.837	2.446	0.999	3.445	1.096	1.778	9.330	4768	24.460	9.990	34.450	10.960	17.780	93.300
120	alpha	0.6	60x8 weeks	Ag80	M	VAC50	75	All	0.993	0.005	0.002	0.007	0.002	0.004	0.019	476.837	2.446	0.999	3.445	1.096	1.778	9.330	4768	24.460	9.990	34.450	10.960	17.780	93.300
120	alpha	1	60x8 weeks	Ag80	M	VAC50	75	All	0.990	0.005	0.005	0.010	0.003	0.006	0.019	475.381	2.448	2.563	5.011	1.596	2.692	9.308	4754	24.480	25.630	50.110	15.960	26.920	93.080
120	alpha	1	60x8 weeks	Ag80	M	VAC50	75	All	0.990	0.005	0.005	0.010	0.003	0.006	0.019	475.381	2.448	2.563	5.011	1.596	2.692	9.308	4754	24.480	25.630	50.110	15.960	26.920	93.080
120	alpha	1	60x8 weeks	Ag80	M	VAC50	75	All	0.990	0.005	0.005	0.010	0.003	0.006	0.019	475.381	2.448	2.563	5.011	1.596	2.692	9.308	4754	24.480	25.630	50.110	15.960	26.920	93.080
120	alpha	1.2	60x8 weeks	Ag80	M	VAC50	75	All	0.988	0.005	0.007	0.012	0.004	0.007	0.019	474.457	2.390	3.564	5.954	1.907	3.140	9.359	4745	23.900	35.640	59.540	19.070	31.400	93.590
120	alpha	1.2	60x8 weeks	Ag80	M	VAC50	75	All	0.988	0.005	0.007	0.012	0.004	0.007	0.019	474.457	2.390	3.564	5.954	1.907	3.140	9.359	4745	23.900	35.640	59.540	19.070	31.400	93.590
120	alpha	1.2	60x8 weeks	Ag80	M	VAC50	75	All	0.988	0.005	0.007	0.012	0.004	0.007	0.019	474.457	2.390	3.564	5.954	1.907	3.140	9.359	4745	23.900	35.640	59.540	19.070	31.400	93.590
120	delta	0.6	60x8 weeks	Ag80	M	VAC50	75	All	0.982	0.006	0.012	0.018	0.006	0.010	0.019	471.449	2.915	5.959	8.874	2.983	4.834	8.923	4714	29.150	59.590	88.740	29.830	48.340	89.230
120	delta	0.6	60x8 weeks	Ag80	M	VAC50	75	All	0.982	0.006	0.012	0.018	0.006	0.010	0.019	471.449	2.915	5.959	8.874	2.983	4.834	8.923	4714	29.150	59.590	88.740	29.830	48.340	89.230
120	delta	0.6	60x8 weeks	Ag80	M	VAC50	75	All	0.982	0.006	0.012	0.018	0.006	0.010	0.019	471.449	2.915	5.959	8.874	2.983	4.834	8.923	4714	29.150	59.590	88.740	29.830	48.340	89.230
120	delta	1	60x8 weeks	Ag80	M	VAC50	75	All	0.955	0.005	0.040	0.045	0.015	0.026	0.017	458.171	2.598	19.173	21.771	7.339	12.361	8.285	4582	25.980	191.730	217.710	73.390	123.610	82.850
120	delta	1	60x8 weeks	Ag80	M	VAC50	75	All	0.955	0.005	0.040	0.045	0.015	0.026	0.017	458.171	2.598	19.173	21.771	7.339	12.361	8.285	4582	25.980	191.730	217.710	73.390	123.610	82.850
120	delta	1	60x8 weeks	Ag80	M	VAC50	75	All	0.955	0.005	0.040	0.045	0.015	0.026	0.017	458.171	2.598	19.173	21.771	7.339	12.361	8.285	4582	25.980	191.730	217.710	73.390	123.610	82.850
120	delta	1.2	60x8 weeks	Ag80	M	VAC50	75	All	0.936	0.005	0.059	0.064	0.021	0.036	0.015	449.180	2.378	28.201	30.579	10.241	17.365	7.352	4492	23.780	282.010	305.790	102.410	173.650	73.520
120	delta	1.2	60x8 weeks	Ag80	M	VAC50	75	All	0.936	0.005	0.059	0.064	0.021	0.036	0.015	449.180	2.378	28.201	30.579	10.241	17.365	7.352	4492	23.780	282.010	305.790	102.410	173.650	73.520
120	delta	1.2	60x8 weeks	Ag80	M	VAC50	75	All	0.936	0.005	0.059	0.064	0.021	0.036	0.015	449.180	2.378	28.201	30.579	10.241	17.365	7.352	4492	23.780	282.010	305.790	102.410	173.650	73.520
120	original	0.6	60x8 weeks	Ag80	M	VAC50	100	All	0.997	0.004	0.000	0.004	0.002	0.000	0.020	478.687	2.036	0.026	2.062	1.000	0.070	9.528	4787	20.360	0.260	20.620	10.000	0.700	95.280
120	original	0.6	60x8 weeks	Ag80	M	VAC50	100	All	0.997	0.004	0.000	0.004	0.002	0.000	0.020	478.687	2.036	0.026	2.062	1.000	0.070	9.528	4787	20.360	0.260	20.620	10.000	0.700	95.280
120	original	0.6	60x8 weeks	Ag80	M	VAC50	100	All	0.997	0.004	0.000	0.004	0.002	0.000	0.020	478.687	2.036	0.026	2.062	1.000	0.070	9.528	4787	20.360	0.260	20.620	10.000	0.700	95.280
120	original	1	60x8 weeks	Ag80	M	VAC50	100	All	0.997	0.004	0.000	0.005	0.002	0.000	0.020	478.630	2.134	0.037	2.171	1.030	0.079	9.418	4786	21.340	0.370	21.710	10.300	0.790	94.180
120	original	1	60x8 weeks	Ag80	M	VAC50	100	All	0.997	0.004	0.000	0.005	0.002	0.000	0.020	478.630	2.134	0.037	2.171	1.030	0.079	9.418	4786	21.340	0.370	21.710	10.300	0.790	94.180
120	original	1	60x8 weeks	Ag80	M	VAC50	100	All	0.997	0.004	0.000	0.005	0.002	0.000	0.020	478.630	2.134	0.037	2.171	1.030	0.079	9.418	4786	21.340	0.370	21.710	10.300	0.790	94.180
120	original	1.2	60x8 weeks	Ag80	M	VAC50	100	All	0.997	0.004	0.000	0.004	0.002	0.000	0.020	478.640	2.062	0.053	2.115	1.009	0.085	9.521	4786	20.620	0.530	21.150	10.090	0.850	95.210
120	original	1.2	60x8 weeks	Ag80	M	VAC50	100	All	0.997	0.004	0.000	0.004	0.002	0.000	0.020	478.640	2.062	0.053	2.115	1.009	0.085	9.521	4786	20.620	0.530	21.150	10.090	0.850	95.210
120	original	1.2	60x8 weeks	Ag80	M	VAC50	100	All	0.997	0.004	0.000	0.004	0.002	0.000	0.020	478.640	2.062	0.053	2.115	1.009	0.085	9.521	4786	20.620	0.530	21.150	10.090	0.850	95.210
120	alpha	0.6	60x8 weeks	Ag80	M	VAC50	100	All	0.995	0.004	0.001	0.005	0.002	0.003	0.020	477.554	2.101	0.522	2.623	0.879	1.316	9.454	4776	21.010	5.220	26.230	8.790	13.160	94.540
120	alpha	0.6	60x8 weeks	Ag80	M	VAC50	100	All	0.995	0.004	0.001	0.005	0.002	0.003	0.020	477.554	2.101	0.522	2.623	0.879	1.316	9.454	4776	21.010	5.220	26.230	8.790	13.160	94.540
120	alpha	0.6	60x8 weeks	Ag80	M	VAC50	100	All	0.995	0.004	0.001	0.005	0.002	0.003	0.020	477.554	2.101	0.522	2.623	0.879	1.316	9.454	4776	21.010	5.220	26.230	8.790	13.160	94.540
120	alpha	1	60x8 weeks	Ag80	M	VAC50	100	All	0.994	0.004	0.002	0.006	0.002	0.003	0.020	477.181	2.057	1.006	3.063	1.001	1.579	9.502	4772	20.570	10.060	30.630	10.010	15.790	95.020
120	alpha	1	60x8 weeks	Ag80	M	VAC50	100	All	0.994	0.004	0.002	0.006	0.002	0.003	0.020	477.181	2.057	1.006	3.063	1.001	1.579	9.502	4772	20.570	10.060	30.630	10.010	15.790	95.020
120	alpha	1	60x8 weeks	Ag80	M	VAC50	100	All	0.994	0.004	0.002	0.006	0.002	0.003	0.020	477.181	2.057	1.006	3.063	1.001	1.579	9.502	4772	20.570	10.060	30.630	10.010	15.790	95.020
120	alpha	1.2	60x8 weeks	Ag80	M	VAC50	100	All	0.994	0.004	0.003	0.007	0.002	0.004	0.019	476.892	2.006	1.318	3.324	1.080	1.732	9.306	4769	20.060	13.180	33.240	10.800	17.320	93.060

120	alpha	1.2	60x8 weeks	Ag80	M	VAC50	100	All	0.994	0.004	0.003	0.007	0.002	0.004	0.019	476.892	2.006	1.318	3.324	1.080	1.732	9.306	4769	20.060	13.180	33.240	10.800	17.320	93.060
120	alpha	1.2	60x8 weeks	Ag80	M	VAC50	100	All	0.994	0.004	0.003	0.007	0.002	0.004	0.019	476.892	2.006	1.318	3.324	1.080	1.732	9.306	4769	20.060	13.180	33.240	10.800	17.320	93.060
120	delta	0.6	60x8 weeks	Ag80	M	VAC50	100	All	0.990	0.005	0.006	0.011	0.004	0.006	0.019	475.000	2.633	2.687	5.320	1.778	2.877	9.223	4750	26.330	26.870	53.200	17.780	28.770	92.230
120	delta	0.6	60x8 weeks	Ag80	M	VAC50	100	All	0.990	0.005	0.006	0.011	0.004	0.006	0.019	475.000	2.633	2.687	5.320	1.778	2.877	9.223	4750	26.330	26.870	53.200	17.780	28.770	92.230
120	delta	0.6	60x8 weeks	Ag80	M	VAC50	100	All	0.990	0.005	0.006	0.011	0.004	0.006	0.019	475.000	2.633	2.687	5.320	1.778	2.877	9.223	4750	26.330	26.870	53.200	17.780	28.770	92.230
120	delta	1	60x8 weeks	Ag80	M	VAC50	100	All	0.979	0.005	0.016	0.022	0.007	0.012	0.019	469.901	2.550	7.844	10.394	3.565	5.531	8.973	4699	25.500	78.440	103.940	35.650	55.310	89.730
120	delta	1	60x8 weeks	Ag80	M	VAC50	100	All	0.979	0.005	0.016	0.022	0.007	0.012	0.019	469.901	2.550	7.844	10.394	3.565	5.531	8.973	4699	25.500	78.440	103.940	35.650	55.310	89.730
120	delta	1	60x8 weeks	Ag80	M	VAC50	100	All	0.979	0.005	0.016	0.022	0.007	0.012	0.019	469.901	2.550	7.844	10.394	3.565	5.531	8.973	4699	25.500	78.440	103.940	35.650	55.310	89.730
120	delta	1.2	60x8 weeks	Ag80	M	VAC50	100	All	0.970	0.005	0.026	0.031	0.010	0.017	0.018	465.581	2.458	12.470	14.928	4.963	8.259	8.682	4656	24.580	124.700	149.280	49.630	82.590	86.820
120	delta	1.2	60x8 weeks	Ag80	M	VAC50	100	All	0.970	0.005	0.026	0.031	0.010	0.017	0.018	465.581	2.458	12.470	14.928	4.963	8.259	8.682	4656	24.580	124.700	149.280	49.630	82.590	86.820
120	delta	1.2	60x8 weeks	Ag80	M	VAC50	100	All	0.970	0.005	0.026	0.031	0.010	0.017	0.018	465.581	2.458	12.470	14.928	4.963	8.259	8.682	4656	24.580	124.700	149.280	49.630	82.590	86.820
120	original	0.6	60x8 weeks	PCR90	Daily	NOVAC	0	Unvaccinate	4.927	0.008	0.002	0.010	0.001	0.010	0.005	2364.978	3.969	1.044	5.013	0.302	4.995	2.260	23650	39.690	10.440	50.130	3.020	49.950	22.600
120	original	0.6	60x8 weeks	PCR90	Daily	NOVAC	0	Unvaccinate	4.927	0.008	0.002	0.010	0.001	0.010	0.005	2364.978	3.969	1.044	5.013	0.302	4.995	2.260	23650	39.690	10.440	50.130	3.020	49.950	22.600
120	original	0.6	60x8 weeks	PCR90	Daily	NOVAC	0	Unvaccinate	4.927	0.008	0.002	0.010	0.001	0.010	0.005	2364.978	3.969	1.044	5.013	0.302	4.995	2.260	23650	39.690	10.440	50.130	3.020	49.950	22.600
120	original	1	60x8 weeks	PCR90	Daily	NOVAC	0	Unvaccinate	4.913	0.008	0.005	0.013	0.001	0.012	0.005	2358.106	3.893	2.222	6.115	0.411	5.969	2.245	23581	38.930	22.220	61.150	4.110	59.690	22.450
120	original	1	60x8 weeks	PCR90	Daily	NOVAC	0	Unvaccinate	4.913	0.008	0.005	0.013	0.001	0.012	0.005	2358.106	3.893	2.222	6.115	0.411	5.969	2.245	23581	38.930	22.220	61.150	4.110	59.690	22.450
120	original	1	60x8 weeks	PCR90	Daily	NOVAC	0	Unvaccinate	4.913	0.008	0.005	0.013	0.001	0.012	0.005	2358.106	3.893	2.222	6.115	0.411	5.969	2.245	23581	38.930	22.220	61.150	4.110	59.690	22.450
120	original	1.2	60x8 weeks	PCR90	Daily	NOVAC	0	Unvaccinate	4.902	0.008	0.006	0.014	0.001	0.014	0.005	2353.151	3.836	2.894	6.730	0.417	6.683	2.231	23532	38.360	28.940	67.300	4.170	66.830	22.310
120	original	1.2	60x8 weeks	PCR90	Daily	NOVAC	0	Unvaccinate	4.902	0.008	0.006	0.014	0.001	0.014	0.005	2353.151	3.836	2.894	6.730	0.417	6.683	2.231	23532	38.360	28.940	67.300	4.170	66.830	22.310
120	original	1.2	60x8 weeks	PCR90	Daily	NOVAC	0	Unvaccinate	4.902	0.008	0.006	0.014	0.001	0.014	0.005	2353.151	3.836	2.894	6.730	0.417	6.683	2.231	23532	38.360	28.940	67.300	4.170	66.830	22.310
120	alpha	0.6	60x8 weeks	PCR90	Daily	NOVAC	0	Unvaccinate	4.908	0.008	0.005	0.013	0.001	0.013	0.005	2356.028	3.756	2.586	6.342	0.399	6.335	2.181	23560	37.560	25.860	63.420	3.990	63.350	21.810
120	alpha	0.6	60x8 weeks	PCR90	Daily	NOVAC	0	Unvaccinate	4.908	0.008	0.005	0.013	0.001	0.013	0.005	2356.028	3.756	2.586	6.342	0.399	6.335	2.181	23560	37.560	25.860	63.420	3.990	63.350	21.810
120	alpha	0.6	60x8 weeks	PCR90	Daily	NOVAC	0	Unvaccinate	4.908	0.008	0.005	0.013	0.001	0.013	0.005	2356.028	3.756	2.586	6.342	0.399	6.335	2.181	23560	37.560	25.860	63.420	3.990	63.350	21.810
120	alpha	1	60x8 weeks	PCR90	Daily	NOVAC	0	Unvaccinate	4.844	0.008	0.014	0.022	0.002	0.022	0.005	2325.335	3.659	6.916	10.575	0.763	10.627	2.203	23253	36.590	69.160	105.750	7.630	106.270	22.030
120	alpha	1	60x8 weeks	PCR90	Daily	NOVAC	0	Unvaccinate	4.844	0.008	0.014	0.022	0.002	0.022	0.005	2325.335	3.659	6.916	10.575	0.763	10.627	2.203	23253	36.590	69.160	105.750	7.630	106.270	22.030
120	alpha	1	60x8 weeks	PCR90	Daily	NOVAC	0	Unvaccinate	4.844	0.008	0.014	0.022	0.002	0.022	0.005	2325.335	3.659	6.916	10.575	0.763	10.627	2.203	23253	36.590	69.160	105.750	7.630	106.270	22.030
120	alpha	1.2	60x8 weeks	PCR90	Daily	NOVAC	0	Unvaccinate	4.783	0.008	0.023	0.031	0.002	0.031	0.004	2295.869	3.629	11.106	14.735	1.022	14.831	2.107	22959	36.290	111.060	147.350	10.220	148.310	21.070
120	alpha	1.2	60x8 weeks	PCR90	Daily	NOVAC	0	Unvaccinate	4.783	0.008	0.023	0.031	0.002	0.031	0.004	2295.869	3.629	11.106	14.735	1.022	14.831	2.107	22959	36.290	111.060	147.350	10.220	148.310	21.070
120	alpha	1.2	60x8 weeks	PCR90	Daily	NOVAC	0	Unvaccinate	4.783	0.008	0.023	0.031	0.002	0.031	0.004	2295.869	3.629	11.106	14.735	1.022	14.831	2.107	22959	36.290	111.060	147.350	10.220	148.310	21.070
120	delta	0.6	60x8 weeks	PCR90	Daily	NOVAC	0	Unvaccinate	4.732	0.007	0.030	0.037	0.004	0.036	0.004	2271.594	3.395	14.472	17.867	1.826	17.474	1.997	22716	33.950	144.720	178.670	18.260	174.740	19.970
120	delta	0.6	60x8 weeks	PCR90	Daily	NOVAC	0	Unvaccinate	4.732	0.007	0.030	0.037	0.004	0.036	0.004	2271.594	3.395	14.472	17.867	1.826	17.474	1.997	22716	33.950	144.720	178.670	18.260	174.740	19.970
120	delta	0.6	60x8 weeks	PCR90	Daily	NOVAC	0	Unvaccinate	4.732	0.007	0.030	0.037	0.004	0.036	0.004	2271.594	3.395	14.472	17.867	1.826	17.474	1.997	22716	33.950	144.720	178.670	18.260	174.740	19.970
120	delta	1	60x8 weeks	PCR90	Daily	NOVAC	0	Unvaccinate	4.305	0.005	0.088	0.093	0.010	0.092	0.003	2066.584	2.509	42.226	44.735	4.697	44.155	1.425	20666	25.090	422.260	447.350	46.970	441.550	14.250
120	delta	1	60x8 weeks	PCR90	Daily	NOVAC	0	Unvaccinate	4.305	0.005	0.088	0.093	0.010	0.092	0.003	2066.584	2.509	42.226	44.735	4.697	44.155	1.425	20666	25.090	422.260	447.350	46.970	441.550	14.250
120	delta	1	60x8 weeks	PCR90	Daily	NOVAC	0	Unvaccinate	4.305	0.005	0.088	0.093	0.010	0.092	0.003	2066.584	2.509	42.226	44.735	4.697	44.155	1.425	20666	25.090	422.260	447.350	46.970	441.550	14.250
120	delta	1.2	60x8 weeks	PCR90	Daily	NOVAC	0	Unvaccinate	4.177	0.004	0.103	0.108	0.012	0.107	0.002	2005.060	2.058	49.649	51.707	5.649	51.278	1.122	20051	20.580	496.490	517.070	56.490	512.780	11.220
120	delta	1.2	60x8 weeks	PCR90	Daily	NOVAC	0	Unvaccinate	4.177	0.004	0.103	0.108	0.012	0.107	0.002	2005.060	2.058	49.649	51.707	5.649	51.278	1.122	20051	20.580	496.490	517.070	56.490	512.780	11.220
120	delta	1.2	60x8 weeks	PCR90	Daily	NOVAC	0	Unvaccinate	4.177	0.004	0.103	0.108	0.012	0.107	0.002	2005.060	2.058	49.649	51.707	5.649	51.278	1.122	20051	20.580	496.490	517.070	56.490	512.780	11.220
120	original	0.6	60x8 weeks	PCR90	Daily	VAC95	25	Unvaccinate	3.701	0.006	0.001	0.007	0.001	0.007	0.004	1776.493	2.971	0.559	3.530	0.254	3.365	1.711	17765	29.710	5.590	35.300	2.540	33.650	17.110
120	original	0.6	60x8 weeks	PCR90	Daily	VAC95	25	Unvaccinate	3.701	0.006	0.001	0.007	0.001	0.007	0.004	1776.493	2.971	0.559	3.530	0.254	3.365	1.711	17765	29.710	5.590	35.300	2.540	33.650	17.110
120	original	0.6	60x8 weeks	PCR90	Daily	VAC95	25	Unvaccinate	3.701	0.006	0.001	0.007	0.001	0.007	0.004	1776.493	2.971	0.559	3.530	0.254	3.365	1.711	17765	29.710	5.590	35.300	2.540	33.650	17.110
120	original	1	60x8 weeks	PCR90	Daily	VAC95	25	Unvaccinate	3.695	0.006	0.002	0.008	0.001	0.008	0.004	1773.365	2.877	1.138	4.015	0.273	3.848	1.747	17734	28.770	11.380	40.150	2.730	38.480	17.470
120	original	1	60x8 weeks	PCR90	Daily	VAC95	25	Unvaccinate	3.695	0.006	0.002	0.008	0.001	0.008	0.004	1773.365	2.877	1.138	4.015	0.273	3.848	1.747	17734	28.770	11.380	40.150	2.730	38.480	17.470
120	original	1	60x8 weeks	PCR90	Daily	VAC95	25	Unvaccinate	3.695	0.006	0.002	0.008	0.001	0.008	0.004	1773.365	2.877	1.138	4.015	0.273	3.848	1.747	17734	28.770	11.380	40.150	2.730	38.480	17.470
120	original	1.2	60x8 weeks	PCR90	Daily	VAC95	25	Unvaccinate	3.690	0.006	0.003	0.009	0.001	0.009	0.004	1771.145	2.998	1.356	4.354	0.299	4.148	1.771	17711	29.980	13.560	43.540	2.990	41.480	17.710
120	original	1.2	60x8 weeks	PCR90	Daily	VAC95	25	Unvaccinate	3.690	0.006	0.003	0.009	0.001	0.009	0.004	1771.145	2.998	1.356	4.354	0.299	4.148	1.771	17711	29.980	13.560	43.540	2.990	41.480	17.710
120	original	1.2	60x8 weeks	PCR90	Daily	VAC95	25	Unvaccinate	3.690	0.006	0.003	0.009	0.001	0.009	0.004	1771.145	2.998	1.356	4.354	0.299	4.148	1.771	17711	29.980	13.560	43.540	2.990	41.480	17.710
120	alpha	0.6	60x8 weeks	PCR90	Daily	VAC95	25	Unvaccinate	3.688	0.006	0.003	0.009	0.001	0.009	0.004	1770.042	3.000	1.393	4.393	0.354	4.263	1.728	17700	30.000	13.930	43.930	3.540	42.630	17.280
120	alpha	0.6	60x8 weeks	PCR90	Daily	VAC95	25	Unvaccinate	3.688	0.006	0.003	0.009	0.001	0.009	0.004	1770.042	3.000	1.393	4.393	0.354	4.263	1.728	17700	30.000	13.930	43.930	3.540	42.630	17.280
120	alpha	0.6	60x8 weeks	PCR90	Daily	VAC95	25	Unvaccinate	3.688	0.006	0.003	0.009	0.001	0.009	0.004	1770.042	3.000	1.393	4.393	0.354	4.263	1.728	17700	30.000	13.930	43.930	3.540	42.630	17.280
120	alpha	1	60x8 weeks	PCR90	Daily	VAC95	25	Unvaccinate	3.667	0.006	0.006	0.012	0.001	0.012	0.004	1760.102	2.923	2.995	5.918	0.485	5.708	1.688	17601	29.230	29.950	59.180	4.850	57.080	16.880
120	alpha	1	60x8 weeks	PCR90	Daily	VAC95	25	Unvaccinate	3.667	0.006	0.006	0.012	0.001	0.012	0.004	1760.102	2.923	2.995	5.918	0.485	5.708	1.688	17601	29.230	29.950	59.180	4.850	57.080	16.880
120	alpha	1	60x8 weeks	PCR90	Daily	VAC95	25	Unvaccinate	3.667	0.006	0.006	0.012	0.001	0.012	0.004	1760.102	2.923	2.995	5.918	0.485	5.708	1.688	17601	29.230	29.950	59.180	4.850	57.080	16.880
120	alpha	1.2	60x8 weeks	PCR90	Daily	VAC95	25	Unvaccinate	3.651	0.006	0.008	0.015	0.001	0.014	0.003	1752.675	2.936	4.071	7.007	0.567	6.787	1.646	17527	29.360	40.710	70.070	5.670	67.870	16.460
120	alpha	1.2	60x8 weeks	PCR90	Daily	VAC95	25	Unvaccinate	3.651	0.006	0.008	0.015	0.001	0.014	0.003	1752.675	2.936	4.071	7.007	0.567	6.787	1.646	17527	29.360	40.710	70.070	5.670	67.870	16.460
120	alpha	1.2	60x8 weeks	PCR90	Daily	VAC95	25	Unvaccinate	3.651	0.006	0.008	0.015	0.001	0.014	0.003	1752.675	2.936	4.071	7.007	0.567	6.787	1.646	17527	29.360	40.710	70.070	5.670	67.870	16.460
120	delta	0.6	60x8 weeks	PCR90	Daily	VAC95	25	Unvaccinate	3.631	0.006	0.012	0.018	0.002	0.016	0.004	1742.884	2.937	5.691	8.628	1.071	7.912	1.729	17429	29.370	56.910	86.280	10.710	79.120	17.290
120	delta	0.6	60x8 weeks	PCR90	Daily	VAC95	25	Unvaccinate	3.631	0.006	0.012	0.018	0.002	0.016	0.004	1742.884	2.937	5.691	8.628	1.071	7.912	1.729	17429	29.370	56.910	86.280	10.710	79.120	17.290
120	delta	0.6	60x8 weeks	PCR90	Daily	VAC95	25	Unvaccinate	3.631	0.006	0.012	0.018	0.002	0.016	0.004	1742.884	2.937	5.691	8.628	1.071	7.912	1.729	17429	29.370	56.910	86.280	10.710	79.120	17.290
120	delta	1	60x8 weeks	PCR90	Daily	VAC95	25	Unvaccinate	3.433	0.005	0.042	0.047	0.006	0.042	0.003	1648.028	2.500	19.931	22.431	3.111	20.383	1.421	16480	25.000	199.310	224.310	31.110	203.830	14.210
120	delta	1	60x8 weeks	PCR90	Daily	VAC95	25	Unvaccinate	3.433	0.005	0.042	0.047	0.006	0.042	0.003	1648.028	2.500	19.931	22.431	3.111	20.383	1.421	16480	25.000	199.310	224.310	31.110	203.830	14.210
120	delta	1	60x8 weeks	PCR90	Daily	VAC95	25	Unvaccinate	3.433	0.005	0.042	0.047	0.006	0.042	0.003	1648.028	2.500	19.931	22.431	3.111	20.383	1.421	16480	25.000	199.310	224.310	31.110	203.830	14.210
120	delta	1.2	60x8 weeks	PCR90	Daily	VAC95	25	Unvaccinate	3.337	0.005	0.058	0.062	0.009	0.056	0.003	1601.586	2.226	27.763	29.989	4.250	26.737	1.243	16016	22.260	277.630	299.890	42.500	267.370	12.430
120	delta	1.2	60x8 weeks	PCR90	Daily	VAC95	25	Unvaccinate	3.337	0.005	0.058	0.062	0.009	0.056	0.003	1601.586	2.226	27.763	29.989	4.250	26.737	1.243	16016	22.260	277.630	299.890	42.500	267.370	12.430
120	delta	1.2	60x8 weeks	PCR90	Daily	VAC95	25	Unvaccinate	3.337	0.005	0.058	0.062	0.009	0.056	0.003	1601.586	2.226	27.763	29.989	4.250	26.737	1.243	16016	22.260	277.630	299.890	42.500	267.370	12.430
120	original	0.6	60x8 weeks	PCR90	Daily	VAC95	50	Unvaccinate	2.467	0.004	0.001	0.005	0.000	0.005	0.002	1184.367	2.153	0.240	2.393	0.217	2.207	1.163	11844	21.530	2.400	23.930	2.170	22.070	11.630
120	original	0.6	60x8 weeks	PCR90	Daily	VAC95	50	Unvaccinate	2.467	0.004	0.001	0.005	0.000	0.005	0.002	1184.367	2.153	0.240	2.393	0.217	2.207	1.163	11844	21.530	2.400	23.930	2.170	22.070	11.630
120	original	0.6	60x8 weeks	PCR90	Daily	VAC95	50	Unvaccinate	2.467	0.004	0.001	0.005	0.000	0.005	0.002	1184.367	2.153	0.240	2.393	0.217	2.207	1.163	11844	21.530	2.400	23.930	2.170	22.070	11.630
120	original	1	60x8 weeks	PCR90	Daily	VAC95	50	Unvaccinate	2.466	0.004	0.001	0.005	0.000	0.005	0.002	1183.879	2.042	0.445	2.487	0.210	2.286	1.165	11839	20.420	4.450	24.870	2.100	22.860	11.650
120	original	1	60x8 weeks	PCR90	Daily	VAC95	50	Unvaccinate	2.466	0.004	0.001	0.005	0.000	0.005	0.002	1183.879	2.042	0.445	2.487	0.210	2.286	1.165	11839	20.420	4.450	24.870	2.100	22.860	11.650
120	original	1	60x8 weeks	PCR90	Daily	VAC95	50	Unvaccinate	2.466	0.004	0.001	0.005	0.000	0.005	0.002	1183.879	2.042	0.445	2.487	0.210	2.286	1.165	11839	20.420	4.450	24.870	2.100	22.860	11.650

120	original	1.2	60x8 weeks	PCR90	Daily	VAC95	50	Unvaccinate	2.464	0.004	0.001	0.006	0.000	0.005	0.002	1182.912	2.066	0.599	2.665	0.222	2.433	1.095	11829	20.660	5.990	26.650	2.220	24.330	10.950
120	original	1.2	60x8 weeks	PCR90	Daily	VAC95	50	Unvaccinate	2.464	0.004	0.001	0.006	0.000	0.005	0.002	1182.912	2.066	0.599	2.665	0.222	2.433	1.095	11829	20.660	5.990	26.650	2.220	24.330	10.950
120	original	1.2	60x8 weeks	PCR90	Daily	VAC95	50	Unvaccinate	2.464	0.004	0.001	0.006	0.000	0.005	0.002	1182.912	2.066	0.599	2.665	0.222	2.433	1.095	11829	20.660	5.990	26.650	2.220	24.330	10.950
120	alpha	0.6	60x8 weeks	PCR90	Daily	VAC95	50	Unvaccinate	2.464	0.004	0.001	0.005	0.001	0.005	0.002	1182.832	2.026	0.606	2.632	0.285	2.412	1.180	11828	20.260	6.060	26.320	2.850	24.120	11.800
120	alpha	0.6	60x8 weeks	PCR90	Daily	VAC95	50	Unvaccinate	2.464	0.004	0.001	0.005	0.001	0.005	0.002	1182.832	2.026	0.606	2.632	0.285	2.412	1.180	11828	20.260	6.060	26.320	2.850	24.120	11.800
120	alpha	0.6	60x8 weeks	PCR90	Daily	VAC95	50	Unvaccinate	2.464	0.004	0.001	0.005	0.001	0.005	0.002	1182.832	2.026	0.606	2.632	0.285	2.412	1.180	11828	20.260	6.060	26.320	2.850	24.120	11.800
120	alpha	1	60x8 weeks	PCR90	Daily	VAC95	50	Unvaccinate	2.455	0.004	0.003	0.007	0.001	0.006	0.002	1178.596	2.092	1.233	3.325	0.337	3.044	1.183	11786	20.920	12.330	33.250	3.370	30.440	11.830
120	alpha	1	60x8 weeks	PCR90	Daily	VAC95	50	Unvaccinate	2.455	0.004	0.003	0.007	0.001	0.006	0.002	1178.596	2.092	1.233	3.325	0.337	3.044	1.183	11786	20.920	12.330	33.250	3.370	30.440	11.830
120	alpha	1	60x8 weeks	PCR90	Daily	VAC95	50	Unvaccinate	2.455	0.004	0.003	0.007	0.001	0.006	0.002	1178.596	2.092	1.233	3.325	0.337	3.044	1.183	11786	20.920	12.330	33.250	3.370	30.440	11.830
120	alpha	1.2	60x8 weeks	PCR90	Daily	VAC95	50	Unvaccinate	2.452	0.004	0.003	0.007	0.001	0.007	0.002	1176.965	1.984	1.611	3.595	0.398	3.278	1.069	11770	19.840	16.110	35.950	3.980	32.780	10.690
120	alpha	1.2	60x8 weeks	PCR90	Daily	VAC95	50	Unvaccinate	2.452	0.004	0.003	0.007	0.001	0.007	0.002	1176.965	1.984	1.611	3.595	0.398	3.278	1.069	11770	19.840	16.110	35.950	3.980	32.780	10.690
120	alpha	1.2	60x8 weeks	PCR90	Daily	VAC95	50	Unvaccinate	2.452	0.004	0.003	0.007	0.001	0.007	0.002	1176.965	1.984	1.611	3.595	0.398	3.278	1.069	11770	19.840	16.110	35.950	3.980	32.780	10.690
120	delta	0.6	60x8 weeks	PCR90	Daily	VAC95	50	Unvaccinate	2.444	0.005	0.005	0.009	0.001	0.008	0.002	1173.311	2.166	2.255	4.421	0.701	3.667	1.199	11733	21.660	22.550	44.210	7.010	36.670	11.990
120	delta	0.6	60x8 weeks	PCR90	Daily	VAC95	50	Unvaccinate	2.444	0.005	0.005	0.009	0.001	0.008	0.002	1173.311	2.166	2.255	4.421	0.701	3.667	1.199	11733	21.660	22.550	44.210	7.010	36.670	11.990
120	delta	0.6	60x8 weeks	PCR90	Daily	VAC95	50	Unvaccinate	2.444	0.005	0.005	0.009	0.001	0.008	0.002	1173.311	2.166	2.255	4.421	0.701	3.667	1.199	11733	21.660	22.550	44.210	7.010	36.670	11.990
120	delta	1	60x8 weeks	PCR90	Daily	VAC95	50	Unvaccinate	2.391	0.004	0.014	0.018	0.003	0.015	0.002	1147.486	2.065	6.730	8.795	1.487	7.172	1.074	11475	20.650	67.300	87.950	14.870	71.720	10.740
120	delta	1	60x8 weeks	PCR90	Daily	VAC95	50	Unvaccinate	2.391	0.004	0.014	0.018	0.003	0.015	0.002	1147.486	2.065	6.730	8.795	1.487	7.172	1.074	11475	20.650	67.300	87.950	14.870	71.720	10.740
120	delta	1	60x8 weeks	PCR90	Daily	VAC95	50	Unvaccinate	2.391	0.004	0.014	0.018	0.003	0.015	0.002	1147.486	2.065	6.730	8.795	1.487	7.172	1.074	11475	20.650	67.300	87.950	14.870	71.720	10.740
120	delta	1.2	60x8 weeks	PCR90	Daily	VAC95	50	Unvaccinate	2.358	0.004	0.020	0.024	0.004	0.019	0.002	1131.698	1.956	9.765	11.721	2.087	9.302	1.049	11317	19.560	97.650	117.210	20.870	93.020	10.490
120	delta	1.2	60x8 weeks	PCR90	Daily	VAC95	50	Unvaccinate	2.358	0.004	0.020	0.024	0.004	0.019	0.002	1131.698	1.956	9.765	11.721	2.087	9.302	1.049	11317	19.560	97.650	117.210	20.870	93.020	10.490
120	delta	1.2	60x8 weeks	PCR90	Daily	VAC95	50	Unvaccinate	2.358	0.004	0.020	0.024	0.004	0.019	0.002	1131.698	1.956	9.765	11.721	2.087	9.302	1.049	11317	19.560	97.650	117.210	20.870	93.020	10.490
120	original	0.6	60x8 weeks	PCR90	Daily	VAC95	75	Unvaccinate	1.235	0.003	0.000	0.003	0.000	0.002	0.001	592.833	1.202	0.056	1.258	0.165	0.995	0.568	5928	12.020	0.560	12.580	1.650	9.950	5.680
120	original	0.6	60x8 weeks	PCR90	Daily	VAC95	75	Unvaccinate	1.235	0.003	0.000	0.003	0.000	0.002	0.001	592.833	1.202	0.056	1.258	0.165	0.995	0.568	5928	12.020	0.560	12.580	1.650	9.950	5.680
120	original	0.6	60x8 weeks	PCR90	Daily	VAC95	75	Unvaccinate	1.235	0.003	0.000	0.003	0.000	0.002	0.001	592.833	1.202	0.056	1.258	0.165	0.995	0.568	5928	12.020	0.560	12.580	1.650	9.950	5.680
120	original	1	60x8 weeks	PCR90	Daily	VAC95	75	Unvaccinate	1.235	0.003	0.000	0.003	0.000	0.002	0.001	592.624	1.205	0.109	1.314	0.160	1.064	0.580	5926	12.050	1.090	13.140	1.600	10.640	5.800
120	original	1	60x8 weeks	PCR90	Daily	VAC95	75	Unvaccinate	1.235	0.003	0.000	0.003	0.000	0.002	0.001	592.624	1.205	0.109	1.314	0.160	1.064	0.580	5926	12.050	1.090	13.140	1.600	10.640	5.800
120	original	1	60x8 weeks	PCR90	Daily	VAC95	75	Unvaccinate	1.235	0.003	0.000	0.003	0.000	0.002	0.001	592.624	1.205	0.109	1.314	0.160	1.064	0.580	5926	12.050	1.090	13.140	1.600	10.640	5.800
120	original	1.2	60x8 weeks	PCR90	Daily	VAC95	75	Unvaccinate	1.235	0.002	0.000	0.003	0.000	0.002	0.001	592.560	1.152	0.137	1.289	0.157	1.039	0.577	5926	11.520	1.370	12.890	1.570	10.390	5.770
120	original	1.2	60x8 weeks	PCR90	Daily	VAC95	75	Unvaccinate	1.235	0.002	0.000	0.003	0.000	0.002	0.001	592.560	1.152	0.137	1.289	0.157	1.039	0.577	5926	11.520	1.370	12.890	1.570	10.390	5.770
120	original	1.2	60x8 weeks	PCR90	Daily	VAC95	75	Unvaccinate	1.235	0.002	0.000	0.003	0.000	0.002	0.001	592.560	1.152	0.137	1.289	0.157	1.039	0.577	5926	11.520	1.370	12.890	1.570	10.390	5.770
120	alpha	0.6	60x8 weeks	PCR90	Daily	VAC95	75	Unvaccinate	1.234	0.003	0.000	0.003	0.000	0.002	0.001	592.552	1.224	0.199	1.423	0.235	1.083	0.557	5926	12.240	1.990	14.230	2.350	10.830	5.570
120	alpha	0.6	60x8 weeks	PCR90	Daily	VAC95	75	Unvaccinate	1.234	0.003	0.000	0.003	0.000	0.002	0.001	592.552	1.224	0.199	1.423	0.235	1.083	0.557	5926	12.240	1.990	14.230	2.350	10.830	5.570
120	alpha	0.6	60x8 weeks	PCR90	Daily	VAC95	75	Unvaccinate	1.234	0.003	0.000	0.003	0.000	0.002	0.001	592.552	1.224	0.199	1.423	0.235	1.083	0.557	5926	12.240	1.990	14.230	2.350	10.830	5.570
120	alpha	1	60x8 weeks	PCR90	Daily	VAC95	75	Unvaccinate	1.232	0.003	0.001	0.003	0.001	0.003	0.001	591.263	1.215	0.357	1.572	0.260	1.228	0.560	5913	12.150	3.570	15.720	2.600	12.280	5.600
120	alpha	1	60x8 weeks	PCR90	Daily	VAC95	75	Unvaccinate	1.232	0.003	0.001	0.003	0.001	0.003	0.001	591.263	1.215	0.357	1.572	0.260	1.228	0.560	5913	12.150	3.570	15.720	2.600	12.280	5.600
120	alpha	1	60x8 weeks	PCR90	Daily	VAC95	75	Unvaccinate	1.232	0.003	0.001	0.003	0.001	0.003	0.001	591.263	1.215	0.357	1.572	0.260	1.228	0.560	5913	12.150	3.570	15.720	2.600	12.280	5.600
120	alpha	1.2	60x8 weeks	PCR90	Daily	VAC95	75	Unvaccinate	1.232	0.003	0.001	0.003	0.001	0.003	0.001	591.156	1.210	0.381	1.591	0.265	1.242	0.593	5912	12.100	3.810	15.910	2.650	12.420	5.930
120	alpha	1.2	60x8 weeks	PCR90	Daily	VAC95	75	Unvaccinate	1.232	0.003	0.001	0.003	0.001	0.003	0.001	591.156	1.210	0.381	1.591	0.265	1.242	0.593	5912	12.100	3.810	15.910	2.650	12.420	5.930
120	alpha	1.2	60x8 weeks	PCR90	Daily	VAC95	75	Unvaccinate	1.232	0.003	0.001	0.003	0.001	0.003	0.001	591.156	1.210	0.381	1.591	0.265	1.242	0.593	5912	12.100	3.810	15.910	2.650	12.420	5.930
120	delta	0.6	60x8 weeks	PCR90	Daily	VAC95	75	Unvaccinate	1.228	0.003	0.002	0.005	0.001	0.003	0.001	589.558	1.412	0.751	2.163	0.501	1.433	0.561	5896	14.120	7.510	21.630	5.010	14.330	5.610
120	delta	0.6	60x8 weeks	PCR90	Daily	VAC95	75	Unvaccinate	1.228	0.003	0.002	0.005	0.001	0.003	0.001	589.558	1.412	0.751	2.163	0.501	1.433	0.561	5896	14.120	7.510	21.630	5.010	14.330	5.610
120	delta	0.6	60x8 weeks	PCR90	Daily	VAC95	75	Unvaccinate	1.228	0.003	0.002	0.005	0.001	0.003	0.001	589.558	1.412	0.751	2.163	0.501	1.433	0.561	5896	14.120	7.510	21.630	5.010	14.330	5.610
120	delta	1	60x8 weeks	PCR90	Daily	VAC95	75	Unvaccinate	1.221	0.003	0.003	0.006	0.002	0.004	0.001	585.894	1.353	1.632	2.985	0.731	1.919	0.547	5859	13.530	16.320	29.850	7.310	19.190	5.470
120	delta	1	60x8 weeks	PCR90	Daily	VAC95	75	Unvaccinate	1.221	0.003	0.003	0.006	0.002	0.004	0.001	585.894	1.353	1.632	2.985	0.731	1.919	0.547	5859	13.530	16.320	29.850	7.310	19.190	5.470
120	delta	1	60x8 weeks	PCR90	Daily	VAC95	75	Unvaccinate	1.221	0.003	0.003	0.006	0.002	0.004	0.001	585.894	1.353	1.632	2.985	0.731	1.919	0.547	5859	13.530	16.320	29.850	7.310	19.190	5.470
120	delta	1.2	60x8 weeks	PCR90	Daily	VAC95	75	Unvaccinate	1.215	0.003	0.004	0.007	0.002	0.005	0.001	583.306	1.427	2.156	3.583	0.880	2.270	0.522	5833	14.270	21.560	35.830	8.800	22.700	5.220
120	delta	1.2	60x8 weeks	PCR90	Daily	VAC95	75	Unvaccinate	1.215	0.003	0.004	0.007	0.002	0.005	0.001	583.306	1.427	2.156	3.583	0.880	2.270	0.522	5833	14.270	21.560	35.830	8.800	22.700	5.220
120	delta	1.2	60x8 weeks	PCR90	Daily	VAC95	75	Unvaccinate	1.215	0.003	0.004	0.007	0.002	0.005	0.001	583.306	1.427	2.156	3.583	0.880	2.270	0.522	5833	14.270	21.560	35.830	8.800	22.700	5.220
120	original	0.6	60x8 weeks	PCR90	Daily	VAC95	100	Unvaccinate	0.000	0.001	0.000	0.001	0.000	0.000	0.000	0.000	0.272	0.001	0.273	0.100	0.000	0.000	0	2.720	0.010	2.730	1.000	0.000	0.000
120	original	0.6	60x8 weeks	PCR90	Daily	VAC95	100	Unvaccinate	0.000	0.001	0.000	0.001	0.000	0.000	0.000	0.000	0.272	0.001	0.273	0.100	0.000	0.000	0	2.720	0.010	2.730	1.000	0.000	0.000
120	original	0.6	60x8 weeks	PCR90	Daily	VAC95	100	Unvaccinate	0.000	0.001	0.000	0.001	0.000	0.000	0.000	0.000	0.272	0.001	0.273	0.100	0.000	0.000	0	2.720	0.010	2.730	1.000	0.000	0.000
120	original	1	60x8 weeks	PCR90	Daily	VAC95	100	Unvaccinate	0.000	0.001	0.000	0.001	0.000	0.000	0.000	0.000	0.272	0.003	0.275	0.126	0.000	0.000	0	2.720	0.030	2.750	1.260	0.000	0.000
120	original	1	60x8 weeks	PCR90	Daily	VAC95	100	Unvaccinate	0.000	0.001	0.000	0.001	0.000	0.000	0.000	0.000	0.272	0.003	0.275	0.126	0.000	0.000	0	2.720	0.030	2.750	1.260	0.000	0.000
120	original	1	60x8 weeks	PCR90	Daily	VAC95	100	Unvaccinate	0.000	0.001	0.000	0.001	0.000	0.000	0.000	0.000	0.272	0.003	0.275	0.126	0.000	0.000	0	2.720	0.030	2.750	1.260	0.000	0.000
120	original	1.2	60x8 weeks	PCR90	Daily	VAC95	100	Unvaccinate	0.000	0.001	0.000	0.001	0.000	0.000	0.000	0.000	0.267	0.001	0.268	0.098	0.000	0.000	0	2.670	0.010	2.680	0.980	0.000	0.000
120	original	1.2	60x8 weeks	PCR90	Daily	VAC95	100	Unvaccinate	0.000	0.001	0.000	0.001	0.000	0.000	0.000	0.000	0.267	0.001	0.268	0.098	0.000	0.000	0	2.670	0.010	2.680	0.980	0.000	0.000
120	original	1.2	60x8 weeks	PCR90	Daily	VAC95	100	Unvaccinate	0.000	0.001	0.000	0.001	0.000	0.000	0.000	0.000	0.267	0.001	0.268	0.098	0.000	0.000	0	2.670	0.010	2.680	0.980	0.000	0.000
120	alpha	0.6	60x8 weeks	PCR90	Daily	VAC95	100	Unvaccinate	0.000	0.001	0.000	0.001	0.000	0.000	0.000	0.000	0.335	0.015	0.350	0.177	0.000	0.000	0	3.350	0.150	3.500	1.770	0.000	0.000
120	alpha	0.6	60x8 weeks	PCR90	Daily	VAC95	100	Unvaccinate	0.000	0.001	0.000	0.001	0.000	0.000	0.000	0.000	0.335	0.015	0.350	0.177	0.000	0.000	0	3.350	0.150	3.500	1.770	0.000	0.000
120	alpha	0.6	60x8 weeks	PCR90	Daily	VAC95	100	Unvaccinate	0.000	0.001	0.000	0.001	0.000	0.000	0.000	0.000	0.335	0.015	0.350	0.177	0.000	0.000	0	3.350	0.150	3.500	1.770	0.000	0.000
120	alpha	1	60x8 weeks	PCR90	Daily	VAC95	100	Unvaccinate	0.000	0.001	0.000	0.001	0.000	0.000	0.000	0.000	0.351	0.028	0.379	0.189	0.000	0.000	0	3.510	0.280	3.790	1.890	0.000	0.000
120	alpha	1	60x8 weeks	PCR90	Daily	VAC95	100	Unvaccinate	0.000	0.001	0.000	0.001	0.000	0.000	0.000	0.000	0.351	0.028	0.379	0.189	0.000	0.000	0	3.510	0.280	3.790	1.890	0.000	0.000
120	alpha	1	60x8 weeks	PCR90	Daily	VAC95	100	Unvaccinate	0.000	0.001	0.000	0.001	0.000	0.000	0.000	0.000	0.351	0.028	0.379	0.189	0.000	0.000	0	3.510	0.280	3.790	1.890	0.000	0.000
120	alpha	1.2	60x8 weeks	PCR90	Daily	VAC95	100	Unvaccinate	0.000	0.001	0.000	0.001	0.000	0.000	0.000	0.000	0.366	0.036	0.402	0.191	0.000	0.000	0	3.660	0.360	4.020	1.910	0.000	0.000
120	alpha	1.2	60x8 weeks	PCR90	Daily	VAC95	100	Unvaccinate	0.000	0.001	0.000	0.001	0.000	0.000	0.000	0.000	0.366	0.036	0.402	0.191	0.000	0.000	0	3.660	0.360	4.020	1.910	0.000	0.000
120	alpha	1.2	60x8 weeks	PCR90	Daily	VAC95	100	Unvaccinate	0.000	0.001	0.000	0.001	0.000	0.000	0.000	0.000	0.366	0.036	0.402	0.191	0.000	0.000	0	3.660	0.360	4.020	1.910	0.000	0.000
120	delta	0.6	60x8 weeks	PCR90	Daily	VAC95	100	Unvaccinate	0.000	0.001	0.000	0.001	0.001	0.000	0.000	0.000	0.571	0.117	0.688	0.350	0.000	0.000	0	5.710	1.170	6.880	3.500	0.000	0.000
120	delta	0.6	60x8 weeks	PCR90	Daily	VAC95	100	Unvaccinate	0.000	0.001	0.000	0.001	0.001	0.000	0.000	0.000	0.571	0.117	0.688	0.350	0.000	0.000	0	5.710	1.170	6.880	3.500	0.000	0.000
120	delta	0.6	60x8 weeks	PCR90	Daily	VAC95	100	Unvaccinate	0.000	0.001	0.000	0.001	0.001	0.000	0.000	0.000	0.571	0.117	0.688	0.350	0.000	0.000	0	5.710	1.170	6.880	3.500	0.000	0.000
120	delta	1	60x8 weeks	PCR90	Daily	VAC95	100	Unvaccinate	0.000	0.001	0.000	0.002	0.001	0.000	0.000	0.000	0.604	0.208	0.812	0.432	0.000	0.000	0	6.040	2.080	8.120	4.320	0.000	0.000
120	delta	1	60x8 weeks	PCR90	Daily	VAC95	100	Unvaccinate	0.000	0.001	0.000	0.002	0.001	0.000	0.000	0.000	0.604	0.208	0.812	0.432	0.000	0.000	0	6.040	2.080	8.120	4.320	0.000	0.000

120	delta	1	60x8 weeks	PCR90	Daily	VAC95	100	Invaccinate	0.000	0.001	0.000	0.002	0.001	0.000	0.000	0.000	0.604	0.208	0.812	0.432	0.000	0.000	0	6.040	2.080	8.120	4.320	0.000	0.000
120	delta	1.2	60x8 weeks	PCR90	Daily	VAC95	100	Invaccinate	0.000	0.001	0.001	0.002	0.001	0.000	0.000	0.000	0.607	0.326	0.933	0.473	0.000	0.000	0	6.070	3.260	9.330	4.730	0.000	0.000
120	delta	1.2	60x8 weeks	PCR90	Daily	VAC95	100	Invaccinate	0.000	0.001	0.001	0.002	0.001	0.000	0.000	0.000	0.607	0.326	0.933	0.473	0.000	0.000	0	6.070	3.260	9.330	4.730	0.000	0.000
120	delta	1.2	60x8 weeks	PCR90	Daily	VAC95	100	Invaccinate	0.000	0.001	0.001	0.002	0.001	0.000	0.000	0.000	0.607	0.326	0.933	0.473	0.000	0.000	0	6.070	3.260	9.330	4.730	0.000	0.000
120	original	0.6	60x8 weeks	PCR90	Daily	VAC70	25	Invaccinate	3.700	0.007	0.001	0.008	0.001	0.007	0.004	1776.169	3.148	0.656	3.804	0.426	3.362	1.711	17762	31.480	6.560	38.040	4.260	33.620	17.110
120	original	0.6	60x8 weeks	PCR90	Daily	VAC70	25	Invaccinate	3.700	0.007	0.001	0.008	0.001	0.007	0.004	1776.169	3.148	0.656	3.804	0.426	3.362	1.711	17762	31.480	6.560	38.040	4.260	33.620	17.110
120	original	0.6	60x8 weeks	PCR90	Daily	VAC70	25	Invaccinate	3.700	0.007	0.001	0.008	0.001	0.007	0.004	1776.169	3.148	0.656	3.804	0.426	3.362	1.711	17762	31.480	6.560	38.040	4.260	33.620	17.110
120	original	1	60x8 weeks	PCR90	Daily	VAC70	25	Invaccinate	3.692	0.007	0.003	0.009	0.001	0.008	0.004	1771.977	3.285	1.259	4.544	0.492	3.991	1.695	17720	32.850	12.590	45.440	4.920	39.910	16.950
120	original	1	60x8 weeks	PCR90	Daily	VAC70	25	Invaccinate	3.692	0.007	0.003	0.009	0.001	0.008	0.004	1771.977	3.285	1.259	4.544	0.492	3.991	1.695	17720	32.850	12.590	45.440	4.920	39.910	16.950
120	original	1	60x8 weeks	PCR90	Daily	VAC70	25	Invaccinate	3.692	0.007	0.003	0.009	0.001	0.008	0.004	1771.977	3.285	1.259	4.544	0.492	3.991	1.695	17720	32.850	12.590	45.440	4.920	39.910	16.950
120	original	1.2	60x8 weeks	PCR90	Daily	VAC70	25	Invaccinate	3.689	0.007	0.003	0.010	0.001	0.009	0.004	1770.698	3.252	1.562	4.814	0.512	4.224	1.744	17707	32.520	15.620	48.140	5.120	42.240	17.440
120	original	1.2	60x8 weeks	PCR90	Daily	VAC70	25	Invaccinate	3.689	0.007	0.003	0.010	0.001	0.009	0.004	1770.698	3.252	1.562	4.814	0.512	4.224	1.744	17707	32.520	15.620	48.140	5.120	42.240	17.440
120	original	1.2	60x8 weeks	PCR90	Daily	VAC70	25	Invaccinate	3.689	0.007	0.003	0.010	0.001	0.009	0.004	1770.698	3.252	1.562	4.814	0.512	4.224	1.744	17707	32.520	15.620	48.140	5.120	42.240	17.440
120	alpha	0.6	60x8 weeks	PCR90	Daily	VAC70	25	Invaccinate	3.686	0.007	0.004	0.010	0.001	0.009	0.003	1769.326	3.200	1.691	4.891	0.583	4.353	1.668	17693	32.000	16.910	48.910	5.830	43.530	16.680
120	alpha	0.6	60x8 weeks	PCR90	Daily	VAC70	25	Invaccinate	3.686	0.007	0.004	0.010	0.001	0.009	0.003	1769.326	3.200	1.691	4.891	0.583	4.353	1.668	17693	32.000	16.910	48.910	5.830	43.530	16.680
120	alpha	0.6	60x8 weeks	PCR90	Daily	VAC70	25	Invaccinate	3.686	0.007	0.004	0.010	0.001	0.009	0.003	1769.326	3.200	1.691	4.891	0.583	4.353	1.668	17693	32.000	16.910	48.910	5.830	43.530	16.680
120	alpha	1	60x8 weeks	PCR90	Daily	VAC70	25	Invaccinate	3.660	0.006	0.008	0.014	0.002	0.013	0.003	1756.569	3.094	3.782	6.876	0.875	6.184	1.594	17566	30.940	37.820	68.760	8.750	61.840	15.940
120	alpha	1	60x8 weeks	PCR90	Daily	VAC70	25	Invaccinate	3.660	0.006	0.008	0.014	0.002	0.013	0.003	1756.569	3.094	3.782	6.876	0.875	6.184	1.594	17566	30.940	37.820	68.760	8.750	61.840	15.940
120	alpha	1	60x8 weeks	PCR90	Daily	VAC70	25	Invaccinate	3.660	0.006	0.008	0.014	0.002	0.013	0.003	1756.569	3.094	3.782	6.876	0.875	6.184	1.594	17566	30.940	37.820	68.760	8.750	61.840	15.940
120	alpha	1.2	60x8 weeks	PCR90	Daily	VAC70	25	Invaccinate	3.637	0.006	0.011	0.018	0.002	0.016	0.003	1745.841	3.075	5.498	8.573	0.974	7.761	1.676	17458	30.750	54.980	85.730	9.740	77.610	16.760
120	alpha	1.2	60x8 weeks	PCR90	Daily	VAC70	25	Invaccinate	3.637	0.006	0.011	0.018	0.002	0.016	0.003	1745.841	3.075	5.498	8.573	0.974	7.761	1.676	17458	30.750	54.980	85.730	9.740	77.610	16.760
120	alpha	1.2	60x8 weeks	PCR90	Daily	VAC70	25	Invaccinate	3.637	0.006	0.011	0.018	0.002	0.016	0.003	1745.841	3.075	5.498	8.573	0.974	7.761	1.676	17458	30.750	54.980	85.730	9.740	77.610	16.760
120	delta	0.6	60x8 weeks	PCR90	Daily	VAC70	25	Invaccinate	3.604	0.006	0.017	0.024	0.004	0.020	0.003	1729.765	3.114	8.224	11.338	1.954	9.558	1.640	17298	31.140	82.240	113.380	19.540	95.580	16.400
120	delta	0.6	60x8 weeks	PCR90	Daily	VAC70	25	Invaccinate	3.604	0.006	0.017	0.024	0.004	0.020	0.003	1729.765	3.114	8.224	11.338	1.954	9.558	1.640	17298	31.140	82.240	113.380	19.540	95.580	16.400
120	delta	0.6	60x8 weeks	PCR90	Daily	VAC70	25	Invaccinate	3.604	0.006	0.017	0.024	0.004	0.020	0.003	1729.765	3.114	8.224	11.338	1.954	9.558	1.640	17298	31.140	82.240	113.380	19.540	95.580	16.400
120	delta	1	60x8 weeks	PCR90	Daily	VAC70	25	Invaccinate	3.359	0.005	0.059	0.065	0.013	0.052	0.003	1612.467	2.525	28.505	31.030	6.036	25.121	1.314	16125	25.250	285.050	310.300	60.360	251.210	13.140
120	delta	1	60x8 weeks	PCR90	Daily	VAC70	25	Invaccinate	3.359	0.005	0.059	0.065	0.013	0.052	0.003	1612.467	2.525	28.505	31.030	6.036	25.121	1.314	16125	25.250	285.050	310.300	60.360	251.210	13.140
120	delta	1	60x8 weeks	PCR90	Daily	VAC70	25	Invaccinate	3.359	0.005	0.059	0.065	0.013	0.052	0.003	1612.467	2.525	28.505	31.030	6.036	25.121	1.314	16125	25.250	285.050	310.300	60.360	251.210	13.140
120	delta	1.2	60x8 weeks	PCR90	Daily	VAC70	25	Invaccinate	3.232	0.005	0.081	0.086	0.019	0.068	0.002	1551.255	2.182	38.967	41.149	8.937	32.588	1.104	15513	21.820	389.670	411.490	89.370	325.880	11.040
120	delta	1.2	60x8 weeks	PCR90	Daily	VAC70	25	Invaccinate	3.232	0.005	0.081	0.086	0.019	0.068	0.002	1551.255	2.182	38.967	41.149	8.937	32.588	1.104	15513	21.820	389.670	411.490	89.370	325.880	11.040
120	delta	1.2	60x8 weeks	PCR90	Daily	VAC70	25	Invaccinate	3.232	0.005	0.081	0.086	0.019	0.068	0.002	1551.255	2.182	38.967	41.149	8.937	32.588	1.104	15513	21.820	389.670	411.490	89.370	325.880	11.040
120	original	0.6	60x8 weeks	PCR90	Daily	VAC70	50	Invaccinate	2.469	0.005	0.001	0.006	0.001	0.005	0.002	1184.927	2.619	0.337	2.956	0.530	2.164	1.133	11849	26.190	3.370	29.560	5.300	21.640	11.330
120	original	0.6	60x8 weeks	PCR90	Daily	VAC70	50	Invaccinate	2.469	0.005	0.001	0.006	0.001	0.005	0.002	1184.927	2.619	0.337	2.956	0.530	2.164	1.133	11849	26.190	3.370	29.560	5.300	21.640	11.330
120	original	0.6	60x8 weeks	PCR90	Daily	VAC70	50	Invaccinate	2.469	0.005	0.001	0.006	0.001	0.005	0.002	1184.927	2.619	0.337	2.956	0.530	2.164	1.133	11849	26.190	3.370	29.560	5.300	21.640	11.330
120	original	1	60x8 weeks	PCR90	Daily	VAC70	50	Invaccinate	2.465	0.005	0.001	0.007	0.001	0.005	0.002	1183.312	2.596	0.632	3.228	0.552	2.366	1.095	11833	25.960	6.320	32.280	5.520	23.660	10.950
120	original	1	60x8 weeks	PCR90	Daily	VAC70	50	Invaccinate	2.465	0.005	0.001	0.007	0.001	0.005	0.002	1183.312	2.596	0.632	3.228	0.552	2.366	1.095	11833	25.960	6.320	32.280	5.520	23.660	10.950
120	original	1	60x8 weeks	PCR90	Daily	VAC70	50	Invaccinate	2.465	0.005	0.001	0.007	0.001	0.005	0.002	1183.312	2.596	0.632	3.228	0.552	2.366	1.095	11833	25.960	6.320	32.280	5.520	23.660	10.950
120	original	1.2	60x8 weeks	PCR90	Daily	VAC70	50	Invaccinate	2.464	0.005	0.001	0.007	0.001	0.005	0.002	1182.804	2.561	0.715	3.276	0.544	2.462	1.088	11828	25.610	7.150	32.760	5.440	24.620	10.880
120	original	1.2	60x8 weeks	PCR90	Daily	VAC70	50	Invaccinate	2.464	0.005	0.001	0.007	0.001	0.005	0.002	1182.804	2.561	0.715	3.276	0.544	2.462	1.088	11828	25.610	7.150	32.760	5.440	24.620	10.880
120	original	1.2	60x8 weeks	PCR90	Daily	VAC70	50	Invaccinate	2.464	0.005	0.001	0.007	0.001	0.005	0.002	1182.804	2.561	0.715	3.276	0.544	2.462	1.088	11828	25.610	7.150	32.760	5.440	24.620	10.880
120	alpha	0.6	60x8 weeks	PCR90	Daily	VAC70	50	Invaccinate	2.460	0.005	0.002	0.007	0.001	0.006	0.002	1180.842	2.567	0.972	3.539	0.673	2.717	1.187	11808	25.670	9.720	35.390	6.730	27.170	11.870
120	alpha	0.6	60x8 weeks	PCR90	Daily	VAC70	50	Invaccinate	2.460	0.005	0.002	0.007	0.001	0.006	0.002	1180.842	2.567	0.972	3.539	0.673	2.717	1.187	11808	25.670	9.720	35.390	6.730	27.170	11.870
120	alpha	0.6	60x8 weeks	PCR90	Daily	VAC70	50	Invaccinate	2.460	0.005	0.002	0.007	0.001	0.006	0.002	1180.842	2.567	0.972	3.539	0.673	2.717	1.187	11808	25.670	9.720	35.390	6.730	27.170	11.870
120	alpha	1	60x8 weeks	PCR90	Daily	VAC70	50	Invaccinate	2.447	0.005	0.005	0.010	0.002	0.008	0.002	1174.349	2.574	2.333	4.907	0.999	3.629	1.174	11743	25.740	23.330	49.070	9.990	36.290	11.740
120	alpha	1	60x8 weeks	PCR90	Daily	VAC70	50	Invaccinate	2.447	0.005	0.005	0.010	0.002	0.008	0.002	1174.349	2.574	2.333	4.907	0.999	3.629	1.174	11743	25.740	23.330	49.070	9.990	36.290	11.740
120	alpha	1	60x8 weeks	PCR90	Daily	VAC70	50	Invaccinate	2.447	0.005	0.005	0.010	0.002	0.008	0.002	1174.349	2.574	2.333	4.907	0.999	3.629	1.174	11743	25.740	23.330	49.070	9.990	36.290	11.740
120	alpha	1.2	60x8 weeks	PCR90	Daily	VAC70	50	Invaccinate	2.440	0.005	0.006	0.011	0.002	0.009	0.002	1170.963	2.512	3.000	5.512	1.093	4.120	1.100	11710	25.120	30.000	55.120	10.930	41.200	11.000
120	alpha	1.2	60x8 weeks	PCR90	Daily	VAC70	50	Invaccinate	2.440	0.005	0.006	0.011	0.002	0.009	0.002	1170.963	2.512	3.000	5.512	1.093	4.120	1.100	11710	25.120	30.000	55.120	10.930	41.200	11.000
120	alpha	1.2	60x8 weeks	PCR90	Daily	VAC70	50	Invaccinate	2.440	0.005	0.006	0.011	0.002	0.009	0.002	1170.963	2.512	3.000	5.512	1.093	4.120	1.100	11710	25.120	30.000	55.120	10.930	41.200	11.000
120	delta	0.6	60x8 weeks	PCR90	Daily	VAC70	50	Invaccinate	2.423	0.006	0.010	0.016	0.004	0.010	0.002	1163.169	2.677	4.987	7.664	2.053	5.028	1.117	11632	26.770	49.870	76.640	20.530	50.280	11.170
120	delta	0.6	60x8 weeks	PCR90	Daily	VAC70	50	Invaccinate	2.423	0.006	0.010	0.016	0.004	0.010	0.002	1163.169	2.677	4.987	7.664	2.053	5.028	1.117	11632	26.770	49.870	76.640	20.530	50.280	11.170
120	delta	0.6	60x8 weeks	PCR90	Daily	VAC70	50	Invaccinate	2.423	0.006	0.010	0.016	0.004	0.010	0.002	1163.169	2.677	4.987	7.664	2.053	5.028	1.117	11632	26.770	49.870	76.640	20.530	50.280	11.170
120	delta	1	60x8 weeks	PCR90	Daily	VAC70	50	Invaccinate	2.314	0.005	0.036	0.041	0.012	0.025	0.002	1110.831	2.370	17.315	19.685	5.768	12.136	0.982	11108	23.700	173.150	196.850	57.680	121.360	9.820
120	delta	1	60x8 weeks	PCR90	Daily	VAC70	50	Invaccinate	2.314	0.005	0.036	0.041	0.012	0.025	0.002	1110.831	2.370	17.315	19.685	5.768	12.136	0.982	11108	23.700	173.150	196.850	57.680	121.360	9.820
120	delta	1	60x8 weeks	PCR90	Daily	VAC70	50	Invaccinate	2.314	0.005	0.036	0.041	0.012	0.025	0.002	1110.831	2.370	17.315	19.685	5.768	12.136	0.982	11108	23.700	173.150	196.850	57.680	121.360	9.820
120	delta	1.2	60x8 weeks	PCR90	Daily	VAC70	50	Invaccinate	2.240	0.004	0.054	0.059	0.019	0.035	0.002	1075.163	2.156	26.147	28.303	8.975	16.769	0.828	10752	21.560	261.470	283.030	89.750	167.690	8.280
120	delta	1.2	60x8 weeks	PCR90	Daily	VAC70	50	Invaccinate	2.240	0.004	0.054	0.059	0.019	0.035	0.002	1075.163	2.156	26.147	28.303	8.975	16.769	0.828	10752	21.560	261.470	283.030	89.750	167.690	8.280
120	delta	1.2	60x8 weeks	PCR90	Daily	VAC70	50	Invaccinate	2.240	0.004	0.054	0.059	0.019	0.035	0.002	1075.163	2.156	26.147	28.303	8.975	16.769	0.828	10752	21.560	261.470	283.030	89.750	167.690	8.280
120	original	0.6	60x8 weeks	PCR90	Daily	VAC70	75	Invaccinate	1.235	0.004	0.000	0.004	0.001	0.002	0.001	592.737	1.964	0.119	2.083	0.570	1.060	0.533	5927	19.640	1.190	20.830	5.700	10.600	5.330
120	original	0.6	60x8 weeks	PCR90	Daily	VAC70	75	Invaccinate	1.235	0.004	0.000	0.004	0.001	0.002	0.001	592.737	1.964	0.119	2.083	0.570	1.060	0.533	5927	19.640	1.190	20.830	5.700	10.600	5.330
120	original	0.6	60x8 weeks	PCR90	Daily	VAC70	75	Invaccinate	1.235	0.004	0.000	0.004	0.001	0.002	0.001	592.737	1.964	0.119	2.083	0.570	1.060	0.533	5927	19.640	1.190	20.830	5.700	10.600	5.330
120	original	1	60x8 weeks	PCR90	Daily	VAC70	75	Invaccinate	1.235	0.004	0.000	0.005	0.001	0.002	0.001	592.561	1.993	0.211	2.204	0.622	1.052	0.564	5926	19.930	2.110	22.040	6.220	10.520	5.640
120	original	1	60x8 weeks	PCR90	Daily	VAC70	75	Invaccinate	1.235	0.004	0.000	0.005	0.001	0.002	0.001	592.561	1.993	0.211	2.204	0.622	1.052	0.564	5926	19.930	2.110	22.040	6.220	10.520	5.640
120	original	1	60x8 weeks	PCR90	Daily	VAC70	75	Invaccinate	1.235	0.004	0.000	0.005	0.001	0.002	0.001	592.561	1.993	0.211	2.204	0.622	1.052	0.564	5926	19.930	2.110	22.040	6.220	10.520	5.640
120	original	1.2	60x8 weeks	PCR90	Daily	VAC70	75	Invaccinate	1.235	0.004	0.000	0.004	0.001	0.002	0.001	592.935	1.918	0.233	2.151	0.594	1.004	0.606	5929	19.180	2.330	21.510	5.940	10.040	6.060
120	original	1.2	60x8 weeks	PCR90	Daily	VAC70	75	Invaccinate	1.235	0.004	0.000	0.004	0.001	0.002	0.001	592.935	1.918	0.233	2.151	0.594	1.004	0.606	5929	19.180	2.330	21.510	5.940	10.040	6.060
120	original	1.2	60x8 weeks	PCR90	Daily	VAC70	75	Invaccinate	1.235	0.004	0.000	0.004	0.001	0.002	0.001	592.935	1.918	0.233	2.151	0.594	1.004	0.606	5929	19.180	2.330	21.510	5.940	10.040	6.060
120	alpha	0.6	60x8 weeks	PCR90	Daily	VAC70	75	Invaccinate	1.232	0.004	0.001	0.005	0.002	0.002	0.001	591.591	1.936	0.534	2.470	0.842	1.184	0.557	5916	19.360	5.340	24.700	8.420	11.840	5.570
120	alpha	0.6	60x8 weeks	PCR90	Daily	VAC70	75	Invaccinate	1.232	0.004	0.001	0.005	0.002	0.002	0.001	591.591	1.936	0.534	2.470	0.842	1.184	0.557	5916	19.360	5.340	24.700	8.420	11.840	5.570
120	alpha	0.6	60x8 weeks	PCR90	Daily	VAC70	75	Invaccinate	1.232	0.004	0.001	0.005	0.002	0.002	0.001	591.591	1.936	0.534	2.470	0.842	1.184	0.557	5916	19.360	5.340	24.700	8.420	11.840	5.570
120	alpha	1	60x8 weeks	PCR90	Daily	VAC70	75	Invaccinate	1.227	0.004	0.002	0.007	0.002	0.003	0.001	589.120	1.944	1.197	3.141	1.061	1.544	0.558	5891	19.440	11.970	31.410	10.610	15.440	5.580

120	alpha	1	60x8 weeks	PCR90	Daily	VAC70	75	Unvaccinate	1.227	0.004	0.002	0.007	0.002	0.003	0.001	589.120	1.944	1.197	3.141	1.061	1.544	0.558	5891	19.440	11.970	31.410	10.610	15.440	5.580
120	alpha	1	60x8 weeks	PCR90	Daily	VAC70	75	Unvaccinate	1.227	0.004	0.002	0.007	0.002	0.003	0.001	589.120	1.944	1.197	3.141	1.061	1.544	0.558	5891	19.440	11.970	31.410	10.610	15.440	5.580
120	alpha	1.2	60x8 weeks	PCR90	Daily	VAC70	75	Unvaccinate	1.226	0.004	0.003	0.007	0.002	0.003	0.001	588.254	1.966	1.476	3.442	1.173	1.653	0.548	5883	19.660	14.760	34.420	11.730	16.530	5.480
120	alpha	1.2	60x8 weeks	PCR90	Daily	VAC70	75	Unvaccinate	1.226	0.004	0.003	0.007	0.002	0.003	0.001	588.254	1.966	1.476	3.442	1.173	1.653	0.548	5883	19.660	14.760	34.420	11.730	16.530	5.480
120	alpha	1.2	60x8 weeks	PCR90	Daily	VAC70	75	Unvaccinate	1.226	0.004	0.003	0.007	0.002	0.003	0.001	588.254	1.966	1.476	3.442	1.173	1.653	0.548	5883	19.660	14.760	34.420	11.730	16.530	5.480
120	delta	0.6	60x8 weeks	PCR90	Daily	VAC70	75	Unvaccinate	1.217	0.005	0.006	0.011	0.004	0.004	0.001	584.352	2.207	2.948	5.155	2.081	2.106	0.578	5844	22.070	29.480	51.550	20.810	21.060	5.780
120	delta	0.6	60x8 weeks	PCR90	Daily	VAC70	75	Unvaccinate	1.217	0.005	0.006	0.011	0.004	0.004	0.001	584.352	2.207	2.948	5.155	2.081	2.106	0.578	5844	22.070	29.480	51.550	20.810	21.060	5.780
120	delta	0.6	60x8 weeks	PCR90	Daily	VAC70	75	Unvaccinate	1.217	0.005	0.006	0.011	0.004	0.004	0.001	584.352	2.207	2.948	5.155	2.081	2.106	0.578	5844	22.070	29.480	51.550	20.810	21.060	5.780
120	delta	1	60x8 weeks	PCR90	Daily	VAC70	75	Unvaccinate	1.186	0.004	0.019	0.023	0.010	0.009	0.001	569.062	2.073	9.142	11.215	4.721	4.205	0.501	5691	20.730	91.420	112.150	47.210	42.050	5.010
120	delta	1	60x8 weeks	PCR90	Daily	VAC70	75	Unvaccinate	1.186	0.004	0.019	0.023	0.010	0.009	0.001	569.062	2.073	9.142	11.215	4.721	4.205	0.501	5691	20.730	91.420	112.150	47.210	42.050	5.010
120	delta	1	60x8 weeks	PCR90	Daily	VAC70	75	Unvaccinate	1.186	0.004	0.019	0.023	0.010	0.009	0.001	569.062	2.073	9.142	11.215	4.721	4.205	0.501	5691	20.730	91.420	112.150	47.210	42.050	5.010
120	delta	1.2	60x8 weeks	PCR90	Daily	VAC70	75	Unvaccinate	1.162	0.004	0.030	0.035	0.015	0.012	0.001	557.731	1.958	14.633	16.591	7.177	5.769	0.493	5577	19.580	146.330	165.910	71.770	57.690	4.930
120	delta	1.2	60x8 weeks	PCR90	Daily	VAC70	75	Unvaccinate	1.162	0.004	0.030	0.035	0.015	0.012	0.001	557.731	1.958	14.633	16.591	7.177	5.769	0.493	5577	19.580	146.330	165.910	71.770	57.690	4.930
120	delta	1.2	60x8 weeks	PCR90	Daily	VAC70	75	Unvaccinate	1.162	0.004	0.030	0.035	0.015	0.012	0.001	557.731	1.958	14.633	16.591	7.177	5.769	0.493	5577	19.580	146.330	165.910	71.770	57.690	4.930
120	original	0.6	60x8 weeks	PCR90	Daily	VAC70	100	Unvaccinate	0.000	0.003	0.000	0.003	0.001	0.000	0.000	0.000	1.337	0.008	1.345	0.666	0.000	0.000	0	13.370	0.080	13.450	6.660	0.000	0.000
120	original	0.6	60x8 weeks	PCR90	Daily	VAC70	100	Unvaccinate	0.000	0.003	0.000	0.003	0.001	0.000	0.000	0.000	1.337	0.008	1.345	0.666	0.000	0.000	0	13.370	0.080	13.450	6.660	0.000	0.000
120	original	0.6	60x8 weeks	PCR90	Daily	VAC70	100	Unvaccinate	0.000	0.003	0.000	0.003	0.001	0.000	0.000	0.000	1.337	0.008	1.345	0.666	0.000	0.000	0	13.370	0.080	13.450	6.660	0.000	0.000
120	original	1	60x8 weeks	PCR90	Daily	VAC70	100	Unvaccinate	0.000	0.003	0.000	0.003	0.001	0.000	0.000	0.000	1.341	0.016	1.357	0.692	0.000	0.000	0	13.410	0.160	13.570	6.920	0.000	0.000
120	original	1	60x8 weeks	PCR90	Daily	VAC70	100	Unvaccinate	0.000	0.003	0.000	0.003	0.001	0.000	0.000	0.000	1.341	0.016	1.357	0.692	0.000	0.000	0	13.410	0.160	13.570	6.920	0.000	0.000
120	original	1	60x8 weeks	PCR90	Daily	VAC70	100	Unvaccinate	0.000	0.003	0.000	0.003	0.001	0.000	0.000	0.000	1.341	0.016	1.357	0.692	0.000	0.000	0	13.410	0.160	13.570	6.920	0.000	0.000
120	original	1.2	60x8 weeks	PCR90	Daily	VAC70	100	Unvaccinate	0.000	0.003	0.000	0.003	0.001	0.000	0.000	0.000	1.298	0.018	1.316	0.643	0.000	0.000	0	12.980	0.180	13.160	6.430	0.000	0.000
120	original	1.2	60x8 weeks	PCR90	Daily	VAC70	100	Unvaccinate	0.000	0.003	0.000	0.003	0.001	0.000	0.000	0.000	1.298	0.018	1.316	0.643	0.000	0.000	0	12.980	0.180	13.160	6.430	0.000	0.000
120	original	1.2	60x8 weeks	PCR90	Daily	VAC70	100	Unvaccinate	0.000	0.003	0.000	0.003	0.001	0.000	0.000	0.000	1.298	0.018	1.316	0.643	0.000	0.000	0	12.980	0.180	13.160	6.430	0.000	0.000
120	alpha	0.6	60x8 weeks	PCR90	Daily	VAC70	100	Unvaccinate	0.000	0.003	0.001	0.003	0.002	0.000	0.000	0.000	1.264	0.277	1.541	0.870	0.000	0.000	0	12.640	2.770	15.410	8.700	0.000	0.000
120	alpha	0.6	60x8 weeks	PCR90	Daily	VAC70	100	Unvaccinate	0.000	0.003	0.001	0.003	0.002	0.000	0.000	0.000	1.264	0.277	1.541	0.870	0.000	0.000	0	12.640	2.770	15.410	8.700	0.000	0.000
120	alpha	0.6	60x8 weeks	PCR90	Daily	VAC70	100	Unvaccinate	0.000	0.003	0.001	0.003	0.002	0.000	0.000	0.000	1.264	0.277	1.541	0.870	0.000	0.000	0	12.640	2.770	15.410	8.700	0.000	0.000
120	alpha	1	60x8 weeks	PCR90	Daily	VAC70	100	Unvaccinate	0.000	0.003	0.001	0.004	0.002	0.000	0.000	0.000	1.286	0.485	1.771	1.045	0.000	0.000	0	12.860	4.850	17.710	10.450	0.000	0.000
120	alpha	1	60x8 weeks	PCR90	Daily	VAC70	100	Unvaccinate	0.000	0.003	0.001	0.004	0.002	0.000	0.000	0.000	1.286	0.485	1.771	1.045	0.000	0.000	0	12.860	4.850	17.710	10.450	0.000	0.000
120	alpha	1	60x8 weeks	PCR90	Daily	VAC70	100	Unvaccinate	0.000	0.003	0.001	0.004	0.002	0.000	0.000	0.000	1.286	0.485	1.771	1.045	0.000	0.000	0	12.860	4.850	17.710	10.450	0.000	0.000
120	alpha	1.2	60x8 weeks	PCR90	Daily	VAC70	100	Unvaccinate	0.000	0.002	0.001	0.004	0.002	0.000	0.000	0.000	1.172	0.651	1.823	1.074	0.000	0.000	0	11.720	6.510	18.230	10.740	0.000	0.000
120	alpha	1.2	60x8 weeks	PCR90	Daily	VAC70	100	Unvaccinate	0.000	0.002	0.001	0.004	0.002	0.000	0.000	0.000	1.172	0.651	1.823	1.074	0.000	0.000	0	11.720	6.510	18.230	10.740	0.000	0.000
120	alpha	1.2	60x8 weeks	PCR90	Daily	VAC70	100	Unvaccinate	0.000	0.002	0.001	0.004	0.002	0.000	0.000	0.000	1.172	0.651	1.823	1.074	0.000	0.000	0	11.720	6.510	18.230	10.740	0.000	0.000
120	delta	0.6	60x8 weeks	PCR90	Daily	VAC70	100	Unvaccinate	0.000	0.003	0.003	0.006	0.004	0.000	0.000	0.000	1.646	1.362	3.008	1.837	0.000	0.000	0	16.460	13.620	30.080	18.370	0.000	0.000
120	delta	0.6	60x8 weeks	PCR90	Daily	VAC70	100	Unvaccinate	0.000	0.003	0.003	0.006	0.004	0.000	0.000	0.000	1.646	1.362	3.008	1.837	0.000	0.000	0	16.460	13.620	30.080	18.370	0.000	0.000
120	delta	0.6	60x8 weeks	PCR90	Daily	VAC70	100	Unvaccinate	0.000	0.003	0.003	0.006	0.004	0.000	0.000	0.000	1.646	1.362	3.008	1.837	0.000	0.000	0	16.460	13.620	30.080	18.370	0.000	0.000
120	delta	1	60x8 weeks	PCR90	Daily	VAC70	100	Unvaccinate	0.000	0.003	0.008	0.011	0.007	0.000	0.000	0.000	1.548	3.749	5.297	3.294	0.000	0.000	0	15.480	37.490	52.970	32.940	0.000	0.000
120	delta	1	60x8 weeks	PCR90	Daily	VAC70	100	Unvaccinate	0.000	0.003	0.008	0.011	0.007	0.000	0.000	0.000	1.548	3.749	5.297	3.294	0.000	0.000	0	15.480	37.490	52.970	32.940	0.000	0.000
120	delta	1	60x8 weeks	PCR90	Daily	VAC70	100	Unvaccinate	0.000	0.003	0.008	0.011	0.007	0.000	0.000	0.000	1.548	3.749	5.297	3.294	0.000	0.000	0	15.480	37.490	52.970	32.940	0.000	0.000
120	delta	1.2	60x8 weeks	PCR90	Daily	VAC70	100	Unvaccinate	0.000	0.003	0.012	0.016	0.010	0.000	0.000	0.000	1.558	5.917	7.475	4.588	0.000	0.000	0	15.580	59.170	74.750	45.880	0.000	0.000
120	delta	1.2	60x8 weeks	PCR90	Daily	VAC70	100	Unvaccinate	0.000	0.003	0.012	0.016	0.010	0.000	0.000	0.000	1.558	5.917	7.475	4.588	0.000	0.000	0	15.580	59.170	74.750	45.880	0.000	0.000
120	delta	1.2	60x8 weeks	PCR90	Daily	VAC70	100	Unvaccinate	0.000	0.003	0.012	0.016	0.010	0.000	0.000	0.000	1.558	5.917	7.475	4.588	0.000	0.000	0	15.580	59.170	74.750	45.880	0.000	0.000
120	original	0.6	60x8 weeks	PCR90	Daily	VAC50	25	Unvaccinate	3.700	0.007	0.002	0.009	0.001	0.007	0.003	1776.003	3.419	0.729	4.148	0.573	3.449	1.658	17760	34.190	7.290	41.480	5.730	34.490	16.580
120	original	0.6	60x8 weeks	PCR90	Daily	VAC50	25	Unvaccinate	3.700	0.007	0.002	0.009	0.001	0.007	0.003	1776.003	3.419	0.729	4.148	0.573	3.449	1.658	17760	34.190	7.290	41.480	5.730	34.490	16.580
120	original	0.6	60x8 weeks	PCR90	Daily	VAC50	25	Unvaccinate	3.700	0.007	0.002	0.009	0.001	0.007	0.003	1776.003	3.419	0.729	4.148	0.573	3.449	1.658	17760	34.190	7.290	41.480	5.730	34.490	16.580
120	original	1	60x8 weeks	PCR90	Daily	VAC50	25	Unvaccinate	3.691	0.007	0.003	0.010	0.001	0.008	0.004	1771.762	3.553	1.327	4.880	0.641	4.029	1.743	17718	35.530	13.270	48.800	6.410	40.290	17.430
120	original	1	60x8 weeks	PCR90	Daily	VAC50	25	Unvaccinate	3.691	0.007	0.003	0.010	0.001	0.008	0.004	1771.762	3.553	1.327	4.880	0.641	4.029	1.743	17718	35.530	13.270	48.800	6.410	40.290	17.430
120	original	1	60x8 weeks	PCR90	Daily	VAC50	25	Unvaccinate	3.691	0.007	0.003	0.010	0.001	0.008	0.004	1771.762	3.553	1.327	4.880	0.641	4.029	1.743	17718	35.530	13.270	48.800	6.410	40.290	17.430
120	original	1.2	60x8 weeks	PCR90	Daily	VAC50	25	Unvaccinate	3.688	0.007	0.004	0.011	0.001	0.009	0.004	1770.400	3.366	1.692	5.058	0.707	4.206	1.737	17704	33.660	16.920	50.580	7.070	42.060	17.370
120	original	1.2	60x8 weeks	PCR90	Daily	VAC50	25	Unvaccinate	3.688	0.007	0.004	0.011	0.001	0.009	0.004	1770.400	3.366	1.692	5.058	0.707	4.206	1.737	17704	33.660	16.920	50.580	7.070	42.060	17.370
120	original	1.2	60x8 weeks	PCR90	Daily	VAC50	25	Unvaccinate	3.688	0.007	0.004	0.011	0.001	0.009	0.004	1770.400	3.366	1.692	5.058	0.707	4.206	1.737	17704	33.660	16.920	50.580	7.070	42.060	17.370
120	alpha	0.6	60x8 weeks	PCR90	Daily	VAC50	25	Unvaccinate	3.684	0.007	0.004	0.011	0.002	0.009	0.004	1768.394	3.388	1.997	5.385	0.786	4.513	1.688	17684	33.880	19.970	53.850	7.860	45.130	16.880
120	alpha	0.6	60x8 weeks	PCR90	Daily	VAC50	25	Unvaccinate	3.684	0.007	0.004	0.011	0.002	0.009	0.004	1768.394	3.388	1.997	5.385	0.786	4.513	1.688	17684	33.880	19.970	53.850	7.860	45.130	16.880
120	alpha	0.6	60x8 weeks	PCR90	Daily	VAC50	25	Unvaccinate	3.684	0.007	0.004	0.011	0.002	0.009	0.004	1768.394	3.388	1.997	5.385	0.786	4.513	1.688	17684	33.880	19.970	53.850	7.860	45.130	16.880
120	alpha	1	60x8 weeks	PCR90	Daily	VAC50	25	Unvaccinate	3.648	0.007	0.010	0.017	0.003	0.015	0.004	1751.080	3.345	4.918	8.263	1.347	6.968	1.690	17511	33.450	49.180	82.630	13.470	69.680	16.900
120	alpha	1	60x8 weeks	PCR90	Daily	VAC50	25	Unvaccinate	3.648	0.007	0.010	0.017	0.003	0.015	0.004	1751.080	3.345	4.918	8.263	1.347	6.968	1.690	17511	33.450	49.180	82.630	13.470	69.680	16.900
120	alpha	1	60x8 weeks	PCR90	Daily	VAC50	25	Unvaccinate	3.648	0.007	0.010	0.017	0.003	0.015	0.004	1751.080	3.345	4.918	8.263	1.347	6.968	1.690	17511	33.450	49.180	82.630	13.470	69.680	16.900
120	alpha	1.2	60x8 weeks	PCR90	Daily	VAC50	25	Unvaccinate	3.617	0.007	0.016	0.022	0.004	0.019	0.003	1736.017	3.250	7.545	10.795	1.736	9.110	1.593	17360	32.500	75.450	107.950	17.360	91.100	15.930
120	alpha	1.2	60x8 weeks	PCR90	Daily	VAC50	25	Unvaccinate	3.617	0.007	0.016	0.022	0.004	0.019	0.003	1736.017	3.250	7.545	10.795	1.736	9.110	1.593	17360	32.500	75.450	107.950	17.360	91.100	15.930
120	alpha	1.2	60x8 weeks	PCR90	Daily	VAC50	25	Unvaccinate	3.617	0.007	0.016	0.022	0.004	0.019	0.003	1736.017	3.250	7.545	10.795	1.736	9.110	1.593	17360	32.500	75.450	107.950	17.360	91.100	15.930
120	delta	0.6	60x8 weeks	PCR90	Daily	VAC50	25	Unvaccinate	3.573	0.007	0.024	0.031	0.007	0.024	0.003	1715.098	3.247	11.541	14.788	3.205	11.456	1.602	17151	32.470	115.410	147.880	32.050	114.560	16.020
120	delta	0.6	60x8 weeks	PCR90	Daily	VAC50	25	Unvaccinate	3.573	0.007	0.024	0.031	0.007	0.024	0.003	1715.098	3.247	11.541	14.788	3.205	11.456	1.602	17151	32.470	115.410	147.880	32.050	114.560	16.020
120	delta	0.6	60x8 weeks	PCR90	Daily	VAC50	25	Unvaccinate	3.573	0.007	0.024	0.031	0.007	0.024	0.003	1715.098	3.247	11.541	14.788	3.205	11.456	1.602	17151	32.470	115.410	147.880	32.050	114.560	16.020
120	delta	1	60x8 weeks	PCR90	Daily	VAC50	25	Unvaccinate	3.284	0.005	0.076	0.081	0.018	0.062	0.002	1576.230	2.442	36.326	38.768	8.850	29.736	1.175	15762	24.420	363.260	387.680	88.500	297.360	11.750
120	delta	1	60x8 weeks	PCR90	Daily	VAC50	25	Unvaccinate	3.284	0.005	0.076	0.081	0.018	0.062	0.002	1576.230	2.442	36.326	38.768	8.850	29.736	1.175	15762	24.420	363.260	387.680	88.500	297.360	11.750
120	delta	1	60x8 weeks	PCR90	Daily	VAC50	25	Unvaccinate	3.284	0.005	0.076	0.081	0.018	0.062	0.002	1576.230	2.442	36.326	38.768	8.850	29.736	1.175	15762	24.420	363.260	387.680	88.500	297.360	11.750
120	delta	1.2	60x8 weeks	PCR90	Daily	VAC50	25	Unvaccinate	3.174	0.004	0.095	0.100	0.024	0.075	0.002	1523.489	2.106	45.783	47.889	11.500	36.233	1.028	15235	21.060	457.830	478.890	115.000	362.330	10.280
120	delta	1.2	60x8 weeks	PCR90	Daily	VAC50	25	Unvaccinate	3.174	0.004	0.095	0.100	0.024	0.075	0.002	1523.489	2.106	45.783	47.889	11.500	36.233	1.028	15235	21.060	457.830	478.890	115.000	362.330	10.280
120	delta	1.2	60x8 weeks	PCR90	Daily	VAC50	25	Unvaccinate	3.174	0.004	0.095	0.100	0.024	0.075	0.002	1523.489	2.106	45.783	47.889	11.500	36.233	1.028	15235	21.060	457.830	478.890	115.000	362.330	10.280
120	original	0.6	60x8 weeks	PCR90	Daily	VAC50	50	Unvaccinate	2.469	0.006	0.001	0.007	0.002	0.004	0.002	1185.241	2.966	0.378	3.344	0.732	2.102	1.142	11852	29.660	3.780	33.440	7.320	21.020	11.420
120	original	0.6	60x8 weeks	PCR90	Daily	VAC50	50	Unvaccinate	2.469	0.006	0.001	0.007	0.002	0.004	0.002	1185.241	2.966	0.378	3.344	0.732	2.102	1.142	11852	29.660	3.780	33.440	7.320	21.020	11.420
120	original	0.6	60x8 weeks	PCR90	Daily	VAC50	50	Unvaccinate	2.469	0.006	0.001	0.007	0.002	0.004	0.002	1185.241	2.966	0.378	3.344	0.732	2.102	1.142	11852	29.660	3.780	33.440	7.320	21.020	11.420

120	original	1	60x8 weeks	PCR90	Daily	VAC50	50	Unvaccinate	2.465	0.006	0.001	0.008	0.002	0.005	0.002	1183.192	3.036	0.703	3.739	0.788	2.411	1.147	11832	30.360	7.030	37.390	7.880	24.110	11.470
120	original	1	60x8 weeks	PCR90	Daily	VAC50	50	Unvaccinate	2.465	0.006	0.001	0.008	0.002	0.005	0.002	1183.192	3.036	0.703	3.739	0.788	2.411	1.147	11832	30.360	7.030	37.390	7.880	24.110	11.470
120	original	1	60x8 weeks	PCR90	Daily	VAC50	50	Unvaccinate	2.465	0.006	0.001	0.008	0.002	0.005	0.002	1183.192	3.036	0.703	3.739	0.788	2.411	1.147	11832	30.360	7.030	37.390	7.880	24.110	11.470
120	original	1.2	60x8 weeks	PCR90	Daily	VAC50	50	Unvaccinate	2.466	0.006	0.002	0.008	0.002	0.005	0.002	1183.578	2.785	0.885	3.670	0.842	2.335	1.123	11836	27.850	8.850	36.700	8.420	23.350	11.230
120	original	1.2	60x8 weeks	PCR90	Daily	VAC50	50	Unvaccinate	2.466	0.006	0.002	0.008	0.002	0.005	0.002	1183.578	2.785	0.885	3.670	0.842	2.335	1.123	11836	27.850	8.850	36.700	8.420	23.350	11.230
120	original	1.2	60x8 weeks	PCR90	Daily	VAC50	50	Unvaccinate	2.466	0.006	0.002	0.008	0.002	0.005	0.002	1183.578	2.785	0.885	3.670	0.842	2.335	1.123	11836	27.850	8.850	36.700	8.420	23.350	11.230
120	alpha	0.6	60x8 weeks	PCR90	Daily	VAC50	50	Unvaccinate	2.456	0.006	0.003	0.009	0.002	0.006	0.002	1178.710	2.964	1.501	4.465	1.100	3.005	1.084	11787	29.640	15.010	44.650	11.000	30.050	10.840
120	alpha	0.6	60x8 weeks	PCR90	Daily	VAC50	50	Unvaccinate	2.456	0.006	0.003	0.009	0.002	0.006	0.002	1178.710	2.964	1.501	4.465	1.100	3.005	1.084	11787	29.640	15.010	44.650	11.000	30.050	10.840
120	alpha	0.6	60x8 weeks	PCR90	Daily	VAC50	50	Unvaccinate	2.456	0.006	0.003	0.009	0.002	0.006	0.002	1178.710	2.964	1.501	4.465	1.100	3.005	1.084	11787	29.640	15.010	44.650	11.000	30.050	10.840
120	alpha	1	60x8 weeks	PCR90	Daily	VAC50	50	Unvaccinate	2.441	0.006	0.007	0.013	0.003	0.008	0.002	1171.636	2.813	3.481	6.294	1.645	4.040	1.071	11716	28.130	34.810	62.940	16.450	40.400	10.710
120	alpha	1	60x8 weeks	PCR90	Daily	VAC50	50	Unvaccinate	2.441	0.006	0.007	0.013	0.003	0.008	0.002	1171.636	2.813	3.481	6.294	1.645	4.040	1.071	11716	28.130	34.810	62.940	16.450	40.400	10.710
120	alpha	1	60x8 weeks	PCR90	Daily	VAC50	50	Unvaccinate	2.441	0.006	0.007	0.013	0.003	0.008	0.002	1171.636	2.813	3.481	6.294	1.645	4.040	1.071	11716	28.130	34.810	62.940	16.450	40.400	10.710
120	alpha	1.2	60x8 weeks	PCR90	Daily	VAC50	50	Unvaccinate	2.424	0.006	0.011	0.017	0.004	0.011	0.002	1163.584	2.811	5.236	8.047	2.097	5.207	1.157	11636	28.110	52.360	80.470	20.970	52.070	11.570
120	alpha	1.2	60x8 weeks	PCR90	Daily	VAC50	50	Unvaccinate	2.424	0.006	0.011	0.017	0.004	0.011	0.002	1163.584	2.811	5.236	8.047	2.097	5.207	1.157	11636	28.110	52.360	80.470	20.970	52.070	11.570
120	alpha	1.2	60x8 weeks	PCR90	Daily	VAC50	50	Unvaccinate	2.424	0.006	0.011	0.017	0.004	0.011	0.002	1163.584	2.811	5.236	8.047	2.097	5.207	1.157	11636	28.110	52.360	80.470	20.970	52.070	11.570
120	delta	0.6	60x8 weeks	PCR90	Daily	VAC50	50	Unvaccinate	2.400	0.006	0.018	0.025	0.008	0.014	0.002	1151.893	3.067	8.760	11.827	3.862	6.651	1.109	11519	30.670	87.600	118.270	38.620	66.510	11.090
120	delta	0.6	60x8 weeks	PCR90	Daily	VAC50	50	Unvaccinate	2.400	0.006	0.018	0.025	0.008	0.014	0.002	1151.893	3.067	8.760	11.827	3.862	6.651	1.109	11519	30.670	87.600	118.270	38.620	66.510	11.090
120	delta	0.6	60x8 weeks	PCR90	Daily	VAC50	50	Unvaccinate	2.400	0.006	0.018	0.025	0.008	0.014	0.002	1151.893	3.067	8.760	11.827	3.862	6.651	1.109	11519	30.670	87.600	118.270	38.620	66.510	11.090
120	delta	1	60x8 weeks	PCR90	Daily	VAC50	50	Unvaccinate	2.234	0.005	0.062	0.067	0.024	0.035	0.002	1072.528	2.460	29.772	32.232	11.480	17.008	0.822	10725	24.600	297.720	322.320	114.800	170.080	8.220
120	delta	1	60x8 weeks	PCR90	Daily	VAC50	50	Unvaccinate	2.234	0.005	0.062	0.067	0.024	0.035	0.002	1072.528	2.460	29.772	32.232	11.480	17.008	0.822	10725	24.600	297.720	322.320	114.800	170.080	8.220
120	delta	1	60x8 weeks	PCR90	Daily	VAC50	50	Unvaccinate	2.234	0.005	0.062	0.067	0.024	0.035	0.002	1072.528	2.460	29.772	32.232	11.480	17.008	0.822	10725	24.600	297.720	322.320	114.800	170.080	8.220
120	delta	1.2	60x8 weeks	PCR90	Daily	VAC50	50	Unvaccinate	2.145	0.004	0.086	0.091	0.034	0.047	0.002	1029.530	2.142	41.325	43.467	16.264	22.405	0.769	10295	21.420	413.250	434.670	162.640	224.050	7.690
120	delta	1.2	60x8 weeks	PCR90	Daily	VAC50	50	Unvaccinate	2.145	0.004	0.086	0.091	0.034	0.047	0.002	1029.530	2.142	41.325	43.467	16.264	22.405	0.769	10295	21.420	413.250	434.670	162.640	224.050	7.690
120	delta	1.2	60x8 weeks	PCR90	Daily	VAC50	50	Unvaccinate	2.145	0.004	0.086	0.091	0.034	0.047	0.002	1029.530	2.142	41.325	43.467	16.264	22.405	0.769	10295	21.420	413.250	434.670	162.640	224.050	7.690
120	original	0.6	60x8 weeks	PCR90	Daily	VAC50	75	Unvaccinate	1.235	0.005	0.000	0.006	0.002	0.002	0.001	592.825	2.554	0.160	2.714	0.935	1.028	0.595	5928	25.540	1.600	27.140	9.350	10.280	5.950
120	original	0.6	60x8 weeks	PCR90	Daily	VAC50	75	Unvaccinate	1.235	0.005	0.000	0.006	0.002	0.002	0.001	592.825	2.554	0.160	2.714	0.935	1.028	0.595	5928	25.540	1.600	27.140	9.350	10.280	5.950
120	original	0.6	60x8 weeks	PCR90	Daily	VAC50	75	Unvaccinate	1.235	0.005	0.000	0.006	0.002	0.002	0.001	592.825	2.554	0.160	2.714	0.935	1.028	0.595	5928	25.540	1.600	27.140	9.350	10.280	5.950
120	original	1	60x8 weeks	PCR90	Daily	VAC50	75	Unvaccinate	1.235	0.005	0.001	0.006	0.002	0.002	0.001	592.758	2.475	0.302	2.777	0.935	1.027	0.587	5928	24.750	3.020	27.770	9.350	10.270	5.870
120	original	1	60x8 weeks	PCR90	Daily	VAC50	75	Unvaccinate	1.235	0.005	0.001	0.006	0.002	0.002	0.001	592.758	2.475	0.302	2.777	0.935	1.027	0.587	5928	24.750	3.020	27.770	9.350	10.270	5.870
120	original	1	60x8 weeks	PCR90	Daily	VAC50	75	Unvaccinate	1.235	0.005	0.001	0.006	0.002	0.002	0.001	592.758	2.475	0.302	2.777	0.935	1.027	0.587	5928	24.750	3.020	27.770	9.350	10.270	5.870
120	original	1.2	60x8 weeks	PCR90	Daily	VAC50	75	Unvaccinate	1.234	0.005	0.001	0.006	0.002	0.002	0.001	592.533	2.471	0.330	2.801	0.945	1.057	0.602	5925	24.710	3.300	28.010	9.450	10.570	6.020
120	original	1.2	60x8 weeks	PCR90	Daily	VAC50	75	Unvaccinate	1.234	0.005	0.001	0.006	0.002	0.002	0.001	592.533	2.471	0.330	2.801	0.945	1.057	0.602	5925	24.710	3.300	28.010	9.450	10.570	6.020
120	original	1.2	60x8 weeks	PCR90	Daily	VAC50	75	Unvaccinate	1.234	0.005	0.001	0.006	0.002	0.002	0.001	592.533	2.471	0.330	2.801	0.945	1.057	0.602	5925	24.710	3.300	28.010	9.450	10.570	6.020
120	alpha	0.6	60x8 weeks	PCR90	Daily	VAC50	75	Unvaccinate	1.229	0.005	0.002	0.008	0.003	0.003	0.001	590.004	2.534	1.165	3.699	1.510	1.405	0.585	5900	25.340	11.650	36.990	15.100	14.050	5.850
120	alpha	0.6	60x8 weeks	PCR90	Daily	VAC50	75	Unvaccinate	1.229	0.005	0.002	0.008	0.003	0.003	0.001	590.004	2.534	1.165	3.699	1.510	1.405	0.585	5900	25.340	11.650	36.990	15.100	14.050	5.850
120	alpha	0.6	60x8 weeks	PCR90	Daily	VAC50	75	Unvaccinate	1.229	0.005	0.002	0.008	0.003	0.003	0.001	590.004	2.534	1.165	3.699	1.510	1.405	0.585	5900	25.340	11.650	36.990	15.100	14.050	5.850
120	alpha	1	60x8 weeks	PCR90	Daily	VAC50	75	Unvaccinate	1.222	0.005	0.005	0.011	0.004	0.004	0.001	586.565	2.554	2.492	5.046	2.083	1.903	0.540	5866	25.540	24.920	50.460	20.830	19.030	5.400
120	alpha	1	60x8 weeks	PCR90	Daily	VAC50	75	Unvaccinate	1.222	0.005	0.005	0.011	0.004	0.004	0.001	586.565	2.554	2.492	5.046	2.083	1.903	0.540	5866	25.540	24.920	50.460	20.830	19.030	5.400
120	alpha	1	60x8 weeks	PCR90	Daily	VAC50	75	Unvaccinate	1.222	0.005	0.005	0.011	0.004	0.004	0.001	586.565	2.554	2.492	5.046	2.083	1.903	0.540	5866	25.540	24.920	50.460	20.830	19.030	5.400
120	alpha	1.2	60x8 weeks	PCR90	Daily	VAC50	75	Unvaccinate	1.216	0.005	0.008	0.013	0.005	0.005	0.001	583.872	2.402	3.626	6.028	2.484	2.286	0.490	5839	24.020	36.260	60.280	24.840	22.860	4.900
120	alpha	1.2	60x8 weeks	PCR90	Daily	VAC50	75	Unvaccinate	1.216	0.005	0.008	0.013	0.005	0.005	0.001	583.872	2.402	3.626	6.028	2.484	2.286	0.490	5839	24.020	36.260	60.280	24.840	22.860	4.900
120	alpha	1.2	60x8 weeks	PCR90	Daily	VAC50	75	Unvaccinate	1.216	0.005	0.008	0.013	0.005	0.005	0.001	583.872	2.402	3.626	6.028	2.484	2.286	0.490	5839	24.020	36.260	60.280	24.840	22.860	4.900
120	delta	0.6	60x8 weeks	PCR90	Daily	VAC50	75	Unvaccinate	1.204	0.006	0.015	0.021	0.010	0.006	0.001	577.918	2.846	7.050	9.896	4.679	2.969	0.522	5779	28.460	70.500	98.960	46.790	29.690	5.220
120	delta	0.6	60x8 weeks	PCR90	Daily	VAC50	75	Unvaccinate	1.204	0.006	0.015	0.021	0.010	0.006	0.001	577.918	2.846	7.050	9.896	4.679	2.969	0.522	5779	28.460	70.500	98.960	46.790	29.690	5.220
120	delta	0.6	60x8 weeks	PCR90	Daily	VAC50	75	Unvaccinate	1.204	0.006	0.015	0.021	0.010	0.006	0.001	577.918	2.846	7.050	9.896	4.679	2.969	0.522	5779	28.460	70.500	98.960	46.790	29.690	5.220
120	delta	1	60x8 weeks	PCR90	Daily	VAC50	75	Unvaccinate	1.131	0.005	0.053	0.058	0.028	0.016	0.001	542.747	2.419	25.262	27.681	13.406	7.709	0.405	5427	24.190	252.620	276.810	134.060	77.090	4.050
120	delta	1	60x8 weeks	PCR90	Daily	VAC50	75	Unvaccinate	1.131	0.005	0.053	0.058	0.028	0.016	0.001	542.747	2.419	25.262	27.681	13.406	7.709	0.405	5427	24.190	252.620	276.810	134.060	77.090	4.050
120	delta	1	60x8 weeks	PCR90	Daily	VAC50	75	Unvaccinate	1.131	0.005	0.053	0.058	0.028	0.016	0.001	542.747	2.419	25.262	27.681	13.406	7.709	0.405	5427	24.190	252.620	276.810	134.060	77.090	4.050
120	delta	1.2	60x8 weeks	PCR90	Daily	VAC50	75	Unvaccinate	1.093	0.004	0.073	0.078	0.040	0.021	0.001	524.788	2.052	35.176	37.228	19.072	9.900	0.402	5248	20.520	351.760	372.280	190.720	99.000	4.020
120	delta	1.2	60x8 weeks	PCR90	Daily	VAC50	75	Unvaccinate	1.093	0.004	0.073	0.078	0.040	0.021	0.001	524.788	2.052	35.176	37.228	19.072	9.900	0.402	5248	20.520	351.760	372.280	190.720	99.000	4.020
120	delta	1.2	60x8 weeks	PCR90	Daily	VAC50	75	Unvaccinate	1.093	0.004	0.073	0.078	0.040	0.021	0.001	524.788	2.052	35.176	37.228	19.072	9.900	0.402	5248	20.520	351.760	372.280	190.720	99.000	4.020
120	original	0.6	60x8 weeks	PCR90	Daily	VAC50	100	Unvaccinate	0.000	0.004	0.000	0.004	0.002	0.000	0.000	0.000	2.051	0.032	2.083	1.092	0.000	0.000	0	20.510	0.320	20.830	10.920	0.000	0.000
120	original	0.6	60x8 weeks	PCR90	Daily	VAC50	100	Unvaccinate	0.000	0.004	0.000	0.004	0.002	0.000	0.000	0.000	2.051	0.032	2.083	1.092	0.000	0.000	0	20.510	0.320	20.830	10.920	0.000	0.000
120	original	0.6	60x8 weeks	PCR90	Daily	VAC50	100	Unvaccinate	0.000	0.004	0.000	0.004	0.002	0.000	0.000	0.000	2.051	0.032	2.083	1.092	0.000	0.000	0	20.510	0.320	20.830	10.920	0.000	0.000
120	original	1	60x8 weeks	PCR90	Daily	VAC50	100	Unvaccinate	0.000	0.004	0.000	0.004	0.002	0.000	0.000	0.000	2.035	0.036	2.071	1.043	0.000	0.000	0	20.350	0.360	20.710	10.430	0.000	0.000
120	original	1	60x8 weeks	PCR90	Daily	VAC50	100	Unvaccinate	0.000	0.004	0.000	0.004	0.002	0.000	0.000	0.000	2.035	0.036	2.071	1.043	0.000	0.000	0	20.350	0.360	20.710	10.430	0.000	0.000
120	original	1	60x8 weeks	PCR90	Daily	VAC50	100	Unvaccinate	0.000	0.004	0.000	0.004	0.002	0.000	0.000	0.000	2.035	0.036	2.071	1.043	0.000	0.000	0	20.350	0.360	20.710	10.430	0.000	0.000
120	original	1.2	60x8 weeks	PCR90	Daily	VAC50	100	Unvaccinate	0.000	0.004	0.000	0.004	0.002	0.000	0.000	0.000	2.027	0.046	2.073	1.092	0.000	0.000	0	20.270	0.460	20.730	10.920	0.000	0.000
120	original	1.2	60x8 weeks	PCR90	Daily	VAC50	100	Unvaccinate	0.000	0.004	0.000	0.004	0.002	0.000	0.000	0.000	2.027	0.046	2.073	1.092	0.000	0.000	0	20.270	0.460	20.730	10.920	0.000	0.000
120	original	1.2	60x8 weeks	PCR90	Daily	VAC50	100	Unvaccinate	0.000	0.004	0.000	0.004	0.002	0.000	0.000	0.000	2.027	0.046	2.073	1.092	0.000	0.000	0	20.270	0.460	20.730	10.920	0.000	0.000
120	alpha	0.6	60x8 weeks	PCR90	Daily	VAC50	100	Unvaccinate	0.000	0.004	0.001	0.006	0.003	0.000	0.000	0.000	1.990	0.714	2.704	1.635	0.000	0.000	0	19.900	7.140	27.040	16.350	0.000	0.000
120	alpha	0.6	60x8 weeks	PCR90	Daily	VAC50	100	Unvaccinate	0.000	0.004	0.001	0.006	0.003	0.000	0.000	0.000	1.990	0.714	2.704	1.635	0.000	0.000	0	19.900	7.140	27.040	16.350	0.000	0.000
120	alpha	0.6	60x8 weeks	PCR90	Daily	VAC50	100	Unvaccinate	0.000	0.004	0.001	0.006	0.003	0.000	0.000	0.000	1.990	0.714	2.704	1.635	0.000	0.000	0	19.900	7.140	27.040	16.350	0.000	0.000
120	alpha	1	60x8 weeks	PCR90	Daily	VAC50	100	Unvaccinate	0.000	0.004	0.003	0.008	0.005	0.000	0.000	0.000	2.050	1.646	3.696	2.217	0.000	0.000	0	20.500	16.460	36.960	22.170	0.000	0.000
120	alpha	1	60x8 weeks	PCR90	Daily	VAC50	100	Unvaccinate	0.000	0.004	0.003	0.008	0.005	0.000	0.000	0.000	2.050	1.646	3.696	2.217	0.000	0.000	0	20.500	16.460	36.960	22.170	0.000	0.000
120	alpha	1	60x8 weeks	PCR90	Daily	VAC50	100	Unvaccinate	0.000	0.004	0.003	0.008	0.005	0.000	0.000	0.000	2.050	1.646	3.696	2.217	0.000	0.000	0	20.500	16.460	36.960	22.170	0.000	0.000
120	alpha	1.2	60x8 weeks	PCR90	Daily	VAC50	100	Unvaccinate	0.000	0.004	0.005	0.009	0.006	0.000	0.000	0.000	2.020	2.319	4.339	2.658	0.000	0.000	0	20.200	23.190	43.390	26.580	0.000	0.000
120	alpha	1.2	60x8 weeks	PCR90	Daily	VAC50	100	Unvaccinate	0.000	0.004	0.005	0.009	0.006	0.000	0.000	0.000	2.020	2.319	4.339	2.658	0.000	0.000	0	20.200	23.190	43.390	26.580	0.000	0.000
120	alpha	1.2	60x8 weeks	PCR90	Daily	VAC50	100	Unvaccinate	0.000	0.004	0.005	0.009	0.006	0.000	0.000	0.000	2.020	2.319	4.339	2.658	0.000	0.000	0	20.200	23.190	43.390	26.580	0.000	0.000
120	delta	0.6	60x8 weeks	PCR90	Daily	VAC50	100	Unvaccinate	0.000	0.005	0.011	0.016	0.010	0.000	0.000	0.000	2.508	5.370	7.878	4.961	0.000	0.000	0	25.080	53.700	78.780	49.610	0.000	0.000
120	delta	0.6	60x8 weeks	PCR90	Daily	VAC50	100	Unvaccinate	0.000	0.005	0.011	0.016	0.010	0.000	0.000	0.000	2.508	5.370	7.878	4.961	0.000	0.000	0	25.080	53.700	78.780	49.610	0.000	0.000

120	delta	0.6	60x8 weeks	PCR90	Daily	VAC50	100	Unvaccinate	0.000	0.005	0.011	0.016	0.010	0.000	0.000	0.000	2.508	5.370	7.878	4.961	0.000	0.000	0	25.080	53.700	78.780	49.610	0.000	0.000
120	delta	1	60x8 weeks	PCR90	Daily	VAC50	100	Unvaccinate	0.000	0.005	0.041	0.046	0.029	0.000	0.000	0.000	2.284	19.621	21.905	13.990	0.000	0.000	0	22.840	196.210	219.050	139.900	0.000	0.000
120	delta	1	60x8 weeks	PCR90	Daily	VAC50	100	Unvaccinate	0.000	0.005	0.041	0.046	0.029	0.000	0.000	0.000	2.284	19.621	21.905	13.990	0.000	0.000	0	22.840	196.210	219.050	139.900	0.000	0.000
120	delta	1	60x8 weeks	PCR90	Daily	VAC50	100	Unvaccinate	0.000	0.005	0.041	0.046	0.029	0.000	0.000	0.000	2.284	19.621	21.905	13.990	0.000	0.000	0	22.840	196.210	219.050	139.900	0.000	0.000
120	delta	1.2	60x8 weeks	PCR90	Daily	VAC50	100	Unvaccinate	0.000	0.004	0.061	0.065	0.042	0.000	0.000	0.000	2.074	29.352	31.426	20.165	0.000	0.000	0	20.740	293.520	314.260	201.650	0.000	0.000
120	delta	1.2	60x8 weeks	PCR90	Daily	VAC50	100	Unvaccinate	0.000	0.004	0.061	0.065	0.042	0.000	0.000	0.000	2.074	29.352	31.426	20.165	0.000	0.000	0	20.740	293.520	314.260	201.650	0.000	0.000
120	delta	1.2	60x8 weeks	PCR90	Daily	VAC50	100	Unvaccinate	0.000	0.004	0.061	0.065	0.042	0.000	0.000	0.000	2.074	29.352	31.426	20.165	0.000	0.000	0	20.740	293.520	314.260	201.650	0.000	0.000
120	original	0.6	60x8 weeks	PCR90	MWF	NOVAC	0	Unvaccinate	2.959	0.008	0.003	0.011	0.001	0.010	0.003	1420.211	3.881	1.439	5.320	0.531	4.955	1.382	14202	38.810	14.390	53.200	5.310	49.550	13.820
120	original	0.6	60x8 weeks	PCR90	MWF	NOVAC	0	Unvaccinate	2.959	0.008	0.003	0.011	0.001	0.010	0.003	1420.211	3.881	1.439	5.320	0.531	4.955	1.382	14202	38.810	14.390	53.200	5.310	49.550	13.820
120	original	0.6	60x8 weeks	PCR90	MWF	NOVAC	0	Unvaccinate	2.959	0.008	0.003	0.011	0.001	0.010	0.003	1420.211	3.881	1.439	5.320	0.531	4.955	1.382	14202	38.810	14.390	53.200	5.310	49.550	13.820
120	original	1	60x8 weeks	PCR90	MWF	NOVAC	0	Unvaccinate	2.948	0.008	0.006	0.014	0.001	0.013	0.003	1415.231	3.766	2.993	6.759	0.678	6.269	1.339	14152	37.660	29.930	67.590	6.780	62.690	13.390
120	original	1	60x8 weeks	PCR90	MWF	NOVAC	0	Unvaccinate	2.948	0.008	0.006	0.014	0.001	0.013	0.003	1415.231	3.766	2.993	6.759	0.678	6.269	1.339	14152	37.660	29.930	67.590	6.780	62.690	13.390
120	original	1	60x8 weeks	PCR90	MWF	NOVAC	0	Unvaccinate	2.948	0.008	0.006	0.014	0.001	0.013	0.003	1415.231	3.766	2.993	6.759	0.678	6.269	1.339	14152	37.660	29.930	67.590	6.780	62.690	13.390
120	original	1.2	60x8 weeks	PCR90	MWF	NOVAC	0	Unvaccinate	2.939	0.008	0.009	0.016	0.002	0.015	0.003	1410.831	3.744	4.082	7.826	0.825	7.282	1.346	14108	37.440	40.820	78.260	8.250	72.820	13.460
120	original	1.2	60x8 weeks	PCR90	MWF	NOVAC	0	Unvaccinate	2.939	0.008	0.009	0.016	0.002	0.015	0.003	1410.831	3.744	4.082	7.826	0.825	7.282	1.346	14108	37.440	40.820	78.260	8.250	72.820	13.460
120	original	1.2	60x8 weeks	PCR90	MWF	NOVAC	0	Unvaccinate	2.939	0.008	0.009	0.016	0.002	0.015	0.003	1410.831	3.744	4.082	7.826	0.825	7.282	1.346	14108	37.440	40.820	78.260	8.250	72.820	13.460
120	alpha	0.6	60x8 weeks	PCR90	MWF	NOVAC	0	Unvaccinate	2.937	0.008	0.009	0.017	0.002	0.016	0.003	1409.917	3.849	4.178	8.027	0.837	7.607	1.316	14099	38.490	41.780	80.270	8.370	76.070	13.160
120	alpha	0.6	60x8 weeks	PCR90	MWF	NOVAC	0	Unvaccinate	2.937	0.008	0.009	0.017	0.002	0.016	0.003	1409.917	3.849	4.178	8.027	0.837	7.607	1.316	14099	38.490	41.780	80.270	8.370	76.070	13.160
120	alpha	0.6	60x8 weeks	PCR90	MWF	NOVAC	0	Unvaccinate	2.937	0.008	0.009	0.017	0.002	0.016	0.003	1409.917	3.849	4.178	8.027	0.837	7.607	1.316	14099	38.490	41.780	80.270	8.370	76.070	13.160
120	alpha	1	60x8 weeks	PCR90	MWF	NOVAC	0	Unvaccinate	2.873	0.007	0.026	0.033	0.004	0.031	0.003	1378.808	3.487	12.258	15.745	1.807	15.047	1.277	13788	34.870	122.580	157.450	18.070	150.470	12.770
120	alpha	1	60x8 weeks	PCR90	MWF	NOVAC	0	Unvaccinate	2.873	0.007	0.026	0.033	0.004	0.031	0.003	1378.808	3.487	12.258	15.745	1.807	15.047	1.277	13788	34.870	122.580	157.450	18.070	150.470	12.770
120	alpha	1	60x8 weeks	PCR90	MWF	NOVAC	0	Unvaccinate	2.873	0.007	0.026	0.033	0.004	0.031	0.003	1378.808	3.487	12.258	15.745	1.807	15.047	1.277	13788	34.870	122.580	157.450	18.070	150.470	12.770
120	alpha	1.2	60x8 weeks	PCR90	MWF	NOVAC	0	Unvaccinate	2.818	0.007	0.040	0.047	0.005	0.045	0.003	1352.553	3.351	19.397	22.748	2.533	21.621	1.248	13526	33.510	193.970	227.480	25.330	216.210	12.480
120	alpha	1.2	60x8 weeks	PCR90	MWF	NOVAC	0	Unvaccinate	2.818	0.007	0.040	0.047	0.005	0.045	0.003	1352.553	3.351	19.397	22.748	2.533	21.621	1.248	13526	33.510	193.970	227.480	25.330	216.210	12.480
120	alpha	1.2	60x8 weeks	PCR90	MWF	NOVAC	0	Unvaccinate	2.818	0.007	0.040	0.047	0.005	0.045	0.003	1352.553	3.351	19.397	22.748	2.533	21.621	1.248	13526	33.510	193.970	227.480	25.330	216.210	12.480
120	delta	0.6	60x8 weeks	PCR90	MWF	NOVAC	0	Unvaccinate	2.775	0.006	0.051	0.057	0.010	0.051	0.002	1332.119	3.091	24.447	27.538	4.631	24.657	1.051	13321	30.910	244.470	275.380	46.310	246.570	10.510
120	delta	0.6	60x8 weeks	PCR90	MWF	NOVAC	0	Unvaccinate	2.775	0.006	0.051	0.057	0.010	0.051	0.002	1332.119	3.091	24.447	27.538	4.631	24.657	1.051	13321	30.910	244.470	275.380	46.310	246.570	10.510
120	delta	0.6	60x8 weeks	PCR90	MWF	NOVAC	0	Unvaccinate	2.775	0.006	0.051	0.057	0.010	0.051	0.002	1332.119	3.091	24.447	27.538	4.631	24.657	1.051	13321	30.910	244.470	275.380	46.310	246.570	10.510
120	delta	1	60x8 weeks	PCR90	MWF	NOVAC	0	Unvaccinate	2.540	0.004	0.106	0.111	0.019	0.101	0.001	1219.294	1.999	51.042	53.041	9.141	48.589	0.708	12193	19.990	510.420	530.410	91.410	485.890	7.080
120	delta	1	60x8 weeks	PCR90	MWF	NOVAC	0	Unvaccinate	2.540	0.004	0.106	0.111	0.019	0.101	0.001	1219.294	1.999	51.042	53.041	9.141	48.589	0.708	12193	19.990	510.420	530.410	91.410	485.890	7.080
120	delta	1	60x8 weeks	PCR90	MWF	NOVAC	0	Unvaccinate	2.540	0.004	0.106	0.111	0.019	0.101	0.001	1219.294	1.999	51.042	53.041	9.141	48.589	0.708	12193	19.990	510.420	530.410	91.410	485.890	7.080
120	delta	1.2	60x8 weeks	PCR90	MWF	NOVAC	0	Unvaccinate	2.510	0.004	0.113	0.117	0.020	0.107	0.001	1204.968	1.851	54.153	56.004	9.767	51.306	0.643	12050	18.510	541.530	560.040	97.670	513.060	6.430
120	delta	1.2	60x8 weeks	PCR90	MWF	NOVAC	0	Unvaccinate	2.510	0.004	0.113	0.117	0.020	0.107	0.001	1204.968	1.851	54.153	56.004	9.767	51.306	0.643	12050	18.510	541.530	560.040	97.670	513.060	6.430
120	delta	1.2	60x8 weeks	PCR90	MWF	NOVAC	0	Unvaccinate	2.510	0.004	0.113	0.117	0.020	0.107	0.001	1204.968	1.851	54.153	56.004	9.767	51.306	0.643	12050	18.510	541.530	560.040	97.670	513.060	6.430
120	original	0.6	60x8 weeks	PCR90	MWF	VAC95	25	Unvaccinate	2.222	0.006	0.002	0.008	0.001	0.007	0.002	1066.576	2.968	0.779	3.747	0.380	3.410	1.038	10666	29.680	7.790	37.470	3.800	34.100	10.380
120	original	0.6	60x8 weeks	PCR90	MWF	VAC95	25	Unvaccinate	2.222	0.006	0.002	0.008	0.001	0.007	0.002	1066.576	2.968	0.779	3.747	0.380	3.410	1.038	10666	29.680	7.790	37.470	3.800	34.100	10.380
120	original	0.6	60x8 weeks	PCR90	MWF	VAC95	25	Unvaccinate	2.222	0.006	0.002	0.008	0.001	0.007	0.002	1066.576	2.968	0.779	3.747	0.380	3.410	1.038	10666	29.680	7.790	37.470	3.800	34.100	10.380
120	original	1	60x8 weeks	PCR90	MWF	VAC95	25	Unvaccinate	2.217	0.006	0.003	0.009	0.001	0.008	0.002	1064.288	2.951	1.374	4.325	0.453	3.968	1.120	10643	29.510	13.740	43.250	4.530	39.680	11.200
120	original	1	60x8 weeks	PCR90	MWF	VAC95	25	Unvaccinate	2.217	0.006	0.003	0.009	0.001	0.008	0.002	1064.288	2.951	1.374	4.325	0.453	3.968	1.120	10643	29.510	13.740	43.250	4.530	39.680	11.200
120	original	1	60x8 weeks	PCR90	MWF	VAC95	25	Unvaccinate	2.217	0.006	0.003	0.009	0.001	0.008	0.002	1064.288	2.951	1.374	4.325	0.453	3.968	1.120	10643	29.510	13.740	43.250	4.530	39.680	11.200
120	original	1.2	60x8 weeks	PCR90	MWF	VAC95	25	Unvaccinate	2.214	0.006	0.004	0.010	0.001	0.009	0.002	1062.670	2.940	1.838	4.778	0.477	4.348	1.064	10627	29.400	18.380	47.780	4.770	43.480	10.640
120	original	1.2	60x8 weeks	PCR90	MWF	VAC95	25	Unvaccinate	2.214	0.006	0.004	0.010	0.001	0.009	0.002	1062.670	2.940	1.838	4.778	0.477	4.348	1.064	10627	29.400	18.380	47.780	4.770	43.480	10.640
120	original	1.2	60x8 weeks	PCR90	MWF	VAC95	25	Unvaccinate	2.214	0.006	0.004	0.010	0.001	0.009	0.002	1062.670	2.940	1.838	4.778	0.477	4.348	1.064	10627	29.400	18.380	47.780	4.770	43.480	10.640
120	alpha	0.6	60x8 weeks	PCR90	MWF	VAC95	25	Unvaccinate	2.212	0.006	0.004	0.010	0.001	0.009	0.002	1061.944	2.929	1.945	4.874	0.615	4.463	1.033	10619	29.290	19.450	48.740	6.150	44.630	10.330
120	alpha	0.6	60x8 weeks	PCR90	MWF	VAC95	25	Unvaccinate	2.212	0.006	0.004	0.010	0.001	0.009	0.002	1061.944	2.929	1.945	4.874	0.615	4.463	1.033	10619	29.290	19.450	48.740	6.150	44.630	10.330
120	alpha	0.6	60x8 weeks	PCR90	MWF	VAC95	25	Unvaccinate	2.212	0.006	0.004	0.010	0.001	0.009	0.002	1061.944	2.929	1.945	4.874	0.615	4.463	1.033	10619	29.290	19.450	48.740	6.150	44.630	10.330
120	alpha	1	60x8 weeks	PCR90	MWF	VAC95	25	Unvaccinate	2.190	0.006	0.010	0.016	0.002	0.015	0.002	1051.320	2.872	4.884	7.756	0.983	7.130	1.037	10513	28.720	48.840	77.560	9.830	71.300	10.370
120	alpha	1	60x8 weeks	PCR90	MWF	VAC95	25	Unvaccinate	2.190	0.006	0.010	0.016	0.002	0.015	0.002	1051.320	2.872	4.884	7.756	0.983	7.130	1.037	10513	28.720	48.840	77.560	9.830	71.300	10.370
120	alpha	1	60x8 weeks	PCR90	MWF	VAC95	25	Unvaccinate	2.190	0.006	0.010	0.016	0.002	0.015	0.002	1051.320	2.872	4.884	7.756	0.983	7.130	1.037	10513	28.720	48.840	77.560	9.830	71.300	10.370
120	alpha	1.2	60x8 weeks	PCR90	MWF	VAC95	25	Unvaccinate	2.174	0.006	0.015	0.021	0.003	0.019	0.002	1043.548	2.919	7.057	9.976	1.257	9.093	0.978	10435	29.190	70.570	99.760	12.570	90.930	9.780
120	alpha	1.2	60x8 weeks	PCR90	MWF	VAC95	25	Unvaccinate	2.174	0.006	0.015	0.021	0.003	0.019	0.002	1043.548	2.919	7.057	9.976	1.257	9.093	0.978	10435	29.190	70.570	99.760	12.570	90.930	9.780
120	alpha	1.2	60x8 weeks	PCR90	MWF	VAC95	25	Unvaccinate	2.174	0.006	0.015	0.021	0.003	0.019	0.002	1043.548	2.919	7.057	9.976	1.257	9.093	0.978	10435	29.190	70.570	99.760	12.570	90.930	9.780
120	delta	0.6	60x8 weeks	PCR90	MWF	VAC95	25	Unvaccinate	2.158	0.006	0.020	0.025	0.005	0.021	0.002	1036.048	2.790	9.416	12.206	2.255	10.259	0.974	10360	27.900	94.160	122.060	22.550	102.590	9.740
120	delta	0.6	60x8 weeks	PCR90	MWF	VAC95	25	Unvaccinate	2.158	0.006	0.020	0.025	0.005	0.021	0.002	1036.048	2.790	9.416	12.206	2.255	10.259	0.974	10360	27.900	94.160	122.060	22.550	102.590	9.740
120	delta	0.6	60x8 weeks	PCR90	MWF	VAC95	25	Unvaccinate	2.158	0.006	0.020	0.025	0.005	0.021	0.002	1036.048	2.790	9.416	12.206	2.255	10.259	0.974	10360	27.900	94.160	122.060	22.550	102.590	9.740
120	delta	1	60x8 weeks	PCR90	MWF	VAC95	25	Unvaccinate	2.011	0.004	0.062	0.066	0.014	0.054	0.002	965.207	2.129	29.596	31.725	6.627	25.821	0.739	9652	21.290	295.960	317.250	66.270	258.210	7.390
120	delta	1	60x8 weeks	PCR90	MWF	VAC95	25	Unvaccinate	2.011	0.004	0.062	0.066	0.014	0.054	0.002	965.207	2.129	29.596	31.725	6.627	25.821	0.739	9652	21.290	295.960	317.250	66.270	258.210	7.390
120	delta	1	60x8 weeks	PCR90	MWF	VAC95	25	Unvaccinate	2.011	0.004	0.062	0.066	0.014	0.054	0.002	965.207	2.129	29.596	31.725	6.627	25.821	0.739	9652	21.290	295.960	317.250	66.270	258.210	7.390
120	delta	1.2	60x8 weeks	PCR90	MWF	VAC95	25	Unvaccinate	1.959	0.004	0.077	0.081	0.017	0.065	0.001	940.375	1.900	36.968	38.868	8.359	31.298	0.593	9404	19.000	369.680	388.680	83.590	312.980	5.930
120	delta	1.2	60x8 weeks	PCR90	MWF	VAC95	25	Unvaccinate	1.959	0.004	0.077	0.081	0.017	0.065	0.001	940.375	1.900	36.968	38.868	8.359	31.298	0.593	9404	19.000	369.680	388.680	83.590	312.980	5.930
120	delta	1.2	60x8 weeks	PCR90	MWF	VAC95	25	Unvaccinate	1.959	0.004	0.077	0.081	0.017	0.065	0.001	940.375	1.900	36.968	38.868	8.359	31.298	0.593	9404	19.000	369.680	388.680	83.590	312.980	5.930
120	original	0.6	60x8 weeks	PCR90	MWF	VAC95	50	Unvaccinate	1.484	0.004	0.001	0.005	0.001	0.004	0.002	712.105	1.997	0.304	2.301	0.301	1.968	0.727	7121	19.970	3.040	23.010	3.010	19.680	7.270
120	original	0.6	60x8 weeks	PCR90	MWF	VAC95	50	Unvaccinate	1.484	0.004	0.001	0.005	0.001	0.004	0.002	712.105	1.997	0.304	2.301	0.301	1.968	0.727	7121	19.970	3.040	23.010	3.010	19.680	7.270
120	original	0.6	60x8 weeks	PCR90	MWF	VAC95	50	Unvaccinate	1.484	0.004	0.001	0.005	0.001	0.004	0.002	712.105	1.997	0.304	2.301	0.301	1.968	0.727	7121	19.970	3.040	23.010	3.010	19.680	7.270
120	original	1	60x8 weeks	PCR90	MWF	VAC95	50	Unvaccinate	1.480	0.004	0.001	0.006	0.001	0.005	0.002	710.448	2.123	0.606	2.729	0.277	2.413	0.720	7104	21.230	6.060	27.290	2.770	24.130	7.200
120	original	1	60x8 weeks	PCR90	MWF	VAC95	50	Unvaccinate	1.480	0.004	0.001	0.006	0.001	0.005	0.002	710.448	2.123	0.606	2.729	0.277	2.413	0.720	7104	21.230	6.060	27.290	2.770	24.130	7.200
120	original	1	60x8 weeks	PCR90	MWF	VAC95	50	Unvaccinate	1.480	0.004	0.001	0.006	0.001	0.005	0.002	710.448	2.123	0.606	2.729	0.277	2.413	0.720	7104	21.230	6.060	27.290	2.770	24.130	7.200
120	original	1.2	60x8 weeks	PCR90	MWF	VAC95	50	Unvaccinate	1.479	0.004	0.002	0.006	0.001	0.005	0.001	710.086	2.033	0.799	2.832	0.309	2.502	0.696	7101	20.330	7.990	28.320	3.090	25.020	6.960
120	original	1.2	60x8 weeks	PCR90	MWF	VAC95	50	Unvaccinate	1.479	0.004	0.002	0.006	0.001	0.005	0.001	710.086	2.033	0.799	2.832	0.309	2.502	0.696	7101	20.330	7.990	28.320	3.090	25.020	6.960
120	original	1.2	60x8 weeks	PCR90	MWF	VAC95	50	Unvaccinate	1.479	0.004	0.002	0.006	0.001	0.005	0.001	710.086	2.033	0.799	2.832	0.309	2.502	0.696	7101	20.330	7.990	28.320	3.090	25.020	6.960
120	alpha	0.6	60x8 weeks	PCR90	MWF	VAC95	50	Unvaccinate	1.480	0.004	0.002	0.006	0.001	0.005	0.001	710.366	2.098	0.753	2.851	0.432	2.412	0.666	7104	20.980	7.530	28.510	4.320	24.120	6.660

120	alpha	0.6	60x8 weeks	PCR90	MWF	VAC95	50	Unvaccinate	1.480	0.004	0.002	0.006	0.001	0.005	0.001	710.366	2.098	0.753	2.851	0.432	2.412	0.666	7104	20.980	7.530	28.510	4.320	24.120	6.660
120	alpha	0.6	60x8 weeks	PCR90	MWF	VAC95	50	Unvaccinate	1.480	0.004	0.002	0.006	0.001	0.005	0.001	710.366	2.098	0.753	2.851	0.432	2.412	0.666	7104	20.980	7.530	28.510	4.320	24.120	6.660
120	alpha	1	60x8 weeks	PCR90	MWF	VAC95	50	Unvaccinate	1.471	0.004	0.004	0.008	0.001	0.007	0.001	706.131	2.139	1.836	3.975	0.596	3.410	0.662	7061	21.390	18.360	39.750	5.960	34.100	6.620
120	alpha	1	60x8 weeks	PCR90	MWF	VAC95	50	Unvaccinate	1.471	0.004	0.004	0.008	0.001	0.007	0.001	706.131	2.139	1.836	3.975	0.596	3.410	0.662	7061	21.390	18.360	39.750	5.960	34.100	6.620
120	alpha	1	60x8 weeks	PCR90	MWF	VAC95	50	Unvaccinate	1.471	0.004	0.004	0.008	0.001	0.007	0.001	706.131	2.139	1.836	3.975	0.596	3.410	0.662	7061	21.390	18.360	39.750	5.960	34.100	6.620
120	alpha	1.2	60x8 weeks	PCR90	MWF	VAC95	50	Unvaccinate	1.467	0.004	0.005	0.009	0.001	0.008	0.001	704.333	2.048	2.416	4.464	0.665	3.859	0.682	7043	20.480	24.160	44.640	6.650	38.590	6.820
120	alpha	1.2	60x8 weeks	PCR90	MWF	VAC95	50	Unvaccinate	1.467	0.004	0.005	0.009	0.001	0.008	0.001	704.333	2.048	2.416	4.464	0.665	3.859	0.682	7043	20.480	24.160	44.640	6.650	38.590	6.820
120	alpha	1.2	60x8 weeks	PCR90	MWF	VAC95	50	Unvaccinate	1.467	0.004	0.005	0.009	0.001	0.008	0.001	704.333	2.048	2.416	4.464	0.665	3.859	0.682	7043	20.480	24.160	44.640	6.650	38.590	6.820
120	delta	0.6	60x8 weeks	PCR90	MWF	VAC95	50	Unvaccinate	1.464	0.004	0.007	0.011	0.002	0.008	0.001	702.548	2.140	3.267	5.407	1.166	4.076	0.631	7025	21.400	32.670	54.070	11.660	40.760	6.310
120	delta	0.6	60x8 weeks	PCR90	MWF	VAC95	50	Unvaccinate	1.464	0.004	0.007	0.011	0.002	0.008	0.001	702.548	2.140	3.267	5.407	1.166	4.076	0.631	7025	21.400	32.670	54.070	11.660	40.760	6.310
120	delta	0.6	60x8 weeks	PCR90	MWF	VAC95	50	Unvaccinate	1.464	0.004	0.007	0.011	0.002	0.008	0.001	702.548	2.140	3.267	5.407	1.166	4.076	0.631	7025	21.400	32.670	54.070	11.660	40.760	6.310
120	delta	1	60x8 weeks	PCR90	MWF	VAC95	50	Unvaccinate	1.421	0.004	0.021	0.025	0.006	0.019	0.001	682.074	1.977	10.194	12.171	2.781	8.949	0.627	6821	19.770	101.940	121.710	27.810	89.490	6.270
120	delta	1	60x8 weeks	PCR90	MWF	VAC95	50	Unvaccinate	1.421	0.004	0.021	0.025	0.006	0.019	0.001	682.074	1.977	10.194	12.171	2.781	8.949	0.627	6821	19.770	101.940	121.710	27.810	89.490	6.270
120	delta	1	60x8 weeks	PCR90	MWF	VAC95	50	Unvaccinate	1.421	0.004	0.021	0.025	0.006	0.019	0.001	682.074	1.977	10.194	12.171	2.781	8.949	0.627	6821	19.770	101.940	121.710	27.810	89.490	6.270
120	delta	1.2	60x8 weeks	PCR90	MWF	VAC95	50	Unvaccinate	1.393	0.004	0.030	0.034	0.008	0.024	0.001	668.629	1.834	14.596	16.430	3.956	11.720	0.591	6686	18.340	145.960	164.300	39.560	117.200	5.910
120	delta	1.2	60x8 weeks	PCR90	MWF	VAC95	50	Unvaccinate	1.393	0.004	0.030	0.034	0.008	0.024	0.001	668.629	1.834	14.596	16.430	3.956	11.720	0.591	6686	18.340	145.960	164.300	39.560	117.200	5.910
120	delta	1.2	60x8 weeks	PCR90	MWF	VAC95	50	Unvaccinate	1.393	0.004	0.030	0.034	0.008	0.024	0.001	668.629	1.834	14.596	16.430	3.956	11.720	0.591	6686	18.340	145.960	164.300	39.560	117.200	5.910
120	original	0.6	60x8 weeks	PCR90	MWF	VAC95	75	Unvaccinate	0.742	0.002	0.000	0.003	0.000	0.002	0.001	356.114	1.186	0.074	1.260	0.198	0.962	0.312	3561	11.860	0.740	12.600	1.980	9.620	3.120
120	original	0.6	60x8 weeks	PCR90	MWF	VAC95	75	Unvaccinate	0.742	0.002	0.000	0.003	0.000	0.002	0.001	356.114	1.186	0.074	1.260	0.198	0.962	0.312	3561	11.860	0.740	12.600	1.980	9.620	3.120
120	original	0.6	60x8 weeks	PCR90	MWF	VAC95	75	Unvaccinate	0.742	0.002	0.000	0.003	0.000	0.002	0.001	356.114	1.186	0.074	1.260	0.198	0.962	0.312	3561	11.860	0.740	12.600	1.980	9.620	3.120
120	original	1	60x8 weeks	PCR90	MWF	VAC95	75	Unvaccinate	0.742	0.003	0.000	0.003	0.000	0.002	0.001	356.017	1.218	0.117	1.335	0.232	0.990	0.350	3560	12.180	1.170	13.350	2.320	9.900	3.500
120	original	1	60x8 weeks	PCR90	MWF	VAC95	75	Unvaccinate	0.742	0.003	0.000	0.003	0.000	0.002	0.001	356.017	1.218	0.117	1.335	0.232	0.990	0.350	3560	12.180	1.170	13.350	2.320	9.900	3.500
120	original	1	60x8 weeks	PCR90	MWF	VAC95	75	Unvaccinate	0.742	0.003	0.000	0.003	0.000	0.002	0.001	356.017	1.218	0.117	1.335	0.232	0.990	0.350	3560	12.180	1.170	13.350	2.320	9.900	3.500
120	original	1.2	60x8 weeks	PCR90	MWF	VAC95	75	Unvaccinate	0.741	0.002	0.000	0.003	0.000	0.002	0.001	355.793	1.184	0.189	1.373	0.196	1.053	0.355	3558	11.840	1.890	13.730	1.960	10.530	3.550
120	original	1.2	60x8 weeks	PCR90	MWF	VAC95	75	Unvaccinate	0.741	0.002	0.000	0.003	0.000	0.002	0.001	355.793	1.184	0.189	1.373	0.196	1.053	0.355	3558	11.840	1.890	13.730	1.960	10.530	3.550
120	original	1.2	60x8 weeks	PCR90	MWF	VAC95	75	Unvaccinate	0.741	0.002	0.000	0.003	0.000	0.002	0.001	355.793	1.184	0.189	1.373	0.196	1.053	0.355	3558	11.840	1.890	13.730	1.960	10.530	3.550
120	alpha	0.6	60x8 weeks	PCR90	MWF	VAC95	75	Unvaccinate	0.741	0.003	0.000	0.003	0.001	0.002	0.001	355.676	1.249	0.235	1.484	0.303	1.088	0.291	3557	12.490	2.350	14.840	3.030	10.880	2.910
120	alpha	0.6	60x8 weeks	PCR90	MWF	VAC95	75	Unvaccinate	0.741	0.003	0.000	0.003	0.001	0.002	0.001	355.676	1.249	0.235	1.484	0.303	1.088	0.291	3557	12.490	2.350	14.840	3.030	10.880	2.910
120	alpha	0.6	60x8 weeks	PCR90	MWF	VAC95	75	Unvaccinate	0.741	0.003	0.000	0.003	0.001	0.002	0.001	355.676	1.249	0.235	1.484	0.303	1.088	0.291	3557	12.490	2.350	14.840	3.030	10.880	2.910
120	alpha	1	60x8 weeks	PCR90	MWF	VAC95	75	Unvaccinate	0.740	0.003	0.001	0.003	0.001	0.003	0.001	355.223	1.206	0.451	1.657	0.324	1.225	0.383	3552	12.060	4.510	16.570	3.240	12.250	3.830
120	alpha	1	60x8 weeks	PCR90	MWF	VAC95	75	Unvaccinate	0.740	0.003	0.001	0.003	0.001	0.003	0.001	355.223	1.206	0.451	1.657	0.324	1.225	0.383	3552	12.060	4.510	16.570	3.240	12.250	3.830
120	alpha	1	60x8 weeks	PCR90	MWF	VAC95	75	Unvaccinate	0.740	0.003	0.001	0.003	0.001	0.003	0.001	355.223	1.206	0.451	1.657	0.324	1.225	0.383	3552	12.060	4.510	16.570	3.240	12.250	3.830
120	alpha	1.2	60x8 weeks	PCR90	MWF	VAC95	75	Unvaccinate	0.739	0.003	0.001	0.004	0.001	0.003	0.001	354.884	1.240	0.532	1.772	0.347	1.266	0.346	3549	12.400	5.320	17.720	3.470	12.660	3.460
120	alpha	1.2	60x8 weeks	PCR90	MWF	VAC95	75	Unvaccinate	0.739	0.003	0.001	0.004	0.001	0.003	0.001	354.884	1.240	0.532	1.772	0.347	1.266	0.346	3549	12.400	5.320	17.720	3.470	12.660	3.460
120	alpha	1.2	60x8 weeks	PCR90	MWF	VAC95	75	Unvaccinate	0.739	0.003	0.001	0.004	0.001	0.003	0.001	354.884	1.240	0.532	1.772	0.347	1.266	0.346	3549	12.400	5.320	17.720	3.470	12.660	3.460
120	delta	0.6	60x8 weeks	PCR90	MWF	VAC95	75	Unvaccinate	0.738	0.003	0.002	0.005	0.001	0.003	0.001	354.099	1.380	0.909	2.289	0.626	1.395	0.334	3541	13.800	9.090	22.890	6.260	13.950	3.340
120	delta	0.6	60x8 weeks	PCR90	MWF	VAC95	75	Unvaccinate	0.738	0.003	0.002	0.005	0.001	0.003	0.001	354.099	1.380	0.909	2.289	0.626	1.395	0.334	3541	13.800	9.090	22.890	6.260	13.950	3.340
120	delta	0.6	60x8 weeks	PCR90	MWF	VAC95	75	Unvaccinate	0.738	0.003	0.002	0.005	0.001	0.003	0.001	354.099	1.380	0.909	2.289	0.626	1.395	0.334	3541	13.800	9.090	22.890	6.260	13.950	3.340
120	delta	1	60x8 weeks	PCR90	MWF	VAC95	75	Unvaccinate	0.732	0.003	0.005	0.007	0.002	0.004	0.001	351.287	1.381	2.194	3.575	1.010	2.070	0.354	3513	13.810	21.940	35.750	10.100	20.700	3.540
120	delta	1	60x8 weeks	PCR90	MWF	VAC95	75	Unvaccinate	0.732	0.003	0.005	0.007	0.002	0.004	0.001	351.287	1.381	2.194	3.575	1.010	2.070	0.354	3513	13.810	21.940	35.750	10.100	20.700	3.540
120	delta	1	60x8 weeks	PCR90	MWF	VAC95	75	Unvaccinate	0.732	0.003	0.005	0.007	0.002	0.004	0.001	351.287	1.381	2.194	3.575	1.010	2.070	0.354	3513	13.810	21.940	35.750	10.100	20.700	3.540
120	delta	1.2	60x8 weeks	PCR90	MWF	VAC95	75	Unvaccinate	0.729	0.003	0.006	0.009	0.003	0.005	0.001	349.755	1.295	2.906	4.201	1.215	2.417	0.345	3498	12.950	29.060	42.010	12.150	24.170	3.450
120	delta	1.2	60x8 weeks	PCR90	MWF	VAC95	75	Unvaccinate	0.729	0.003	0.006	0.009	0.003	0.005	0.001	349.755	1.295	2.906	4.201	1.215	2.417	0.345	3498	12.950	29.060	42.010	12.150	24.170	3.450
120	delta	1.2	60x8 weeks	PCR90	MWF	VAC95	75	Unvaccinate	0.729	0.003	0.006	0.009	0.003	0.005	0.001	349.755	1.295	2.906	4.201	1.215	2.417	0.345	3498	12.950	29.060	42.010	12.150	24.170	3.450
120	original	0.6	60x8 weeks	PCR90	MWF	VAC95	100	Unvaccinate	0.000	0.001	0.000	0.001	0.000	0.000	0.000	0.000	0.274	0.000	0.274	0.093	0.000	0.000	0	2.740	0.000	2.740	0.930	0.000	0.000
120	original	0.6	60x8 weeks	PCR90	MWF	VAC95	100	Unvaccinate	0.000	0.001	0.000	0.001	0.000	0.000	0.000	0.000	0.274	0.000	0.274	0.093	0.000	0.000	0	2.740	0.000	2.740	0.930	0.000	0.000
120	original	0.6	60x8 weeks	PCR90	MWF	VAC95	100	Unvaccinate	0.000	0.001	0.000	0.001	0.000	0.000	0.000	0.000	0.274	0.000	0.274	0.093	0.000	0.000	0	2.740	0.000	2.740	0.930	0.000	0.000
120	original	1	60x8 weeks	PCR90	MWF	VAC95	100	Unvaccinate	0.000	0.001	0.000	0.001	0.000	0.000	0.000	0.000	0.265	0.000	0.265	0.106	0.000	0.000	0	2.650	0.000	2.650	1.060	0.000	0.000
120	original	1	60x8 weeks	PCR90	MWF	VAC95	100	Unvaccinate	0.000	0.001	0.000	0.001	0.000	0.000	0.000	0.000	0.265	0.000	0.265	0.106	0.000	0.000	0	2.650	0.000	2.650	1.060	0.000	0.000
120	original	1	60x8 weeks	PCR90	MWF	VAC95	100	Unvaccinate	0.000	0.001	0.000	0.001	0.000	0.000	0.000	0.000	0.265	0.000	0.265	0.106	0.000	0.000	0	2.650	0.000	2.650	1.060	0.000	0.000
120	original	1.2	60x8 weeks	PCR90	MWF	VAC95	100	Unvaccinate	0.000	0.001	0.000	0.001	0.000	0.000	0.000	0.000	0.292	0.002	0.294	0.114	0.000	0.000	0	2.920	0.020	2.940	1.140	0.000	0.000
120	original	1.2	60x8 weeks	PCR90	MWF	VAC95	100	Unvaccinate	0.000	0.001	0.000	0.001	0.000	0.000	0.000	0.000	0.292	0.002	0.294	0.114	0.000	0.000	0	2.920	0.020	2.940	1.140	0.000	0.000
120	original	1.2	60x8 weeks	PCR90	MWF	VAC95	100	Unvaccinate	0.000	0.001	0.000	0.001	0.000	0.000	0.000	0.000	0.292	0.002	0.294	0.114	0.000	0.000	0	2.920	0.020	2.940	1.140	0.000	0.000
120	alpha	0.6	60x8 weeks	PCR90	MWF	VAC95	100	Unvaccinate	0.000	0.001	0.000	0.001	0.000	0.000	0.000	0.000	0.369	0.021	0.390	0.193	0.000	0.000	0	3.690	0.210	3.900	1.930	0.000	0.000
120	alpha	0.6	60x8 weeks	PCR90	MWF	VAC95	100	Unvaccinate	0.000	0.001	0.000	0.001	0.000	0.000	0.000	0.000	0.369	0.021	0.390	0.193	0.000	0.000	0	3.690	0.210	3.900	1.930	0.000	0.000
120	alpha	0.6	60x8 weeks	PCR90	MWF	VAC95	100	Unvaccinate	0.000	0.001	0.000	0.001	0.000	0.000	0.000	0.000	0.369	0.021	0.390	0.193	0.000	0.000	0	3.690	0.210	3.900	1.930	0.000	0.000
120	alpha	1	60x8 weeks	PCR90	MWF	VAC95	100	Unvaccinate	0.000	0.001	0.000	0.001	0.000	0.000	0.000	0.000	0.361	0.032	0.393	0.191	0.000	0.000	0	3.610	0.320	3.930	1.910	0.000	0.000
120	alpha	1	60x8 weeks	PCR90	MWF	VAC95	100	Unvaccinate	0.000	0.001	0.000	0.001	0.000	0.000	0.000	0.000	0.361	0.032	0.393	0.191	0.000	0.000	0	3.610	0.320	3.930	1.910	0.000	0.000
120	alpha	1	60x8 weeks	PCR90	MWF	VAC95	100	Unvaccinate	0.000	0.001	0.000	0.001	0.000	0.000	0.000	0.000	0.361	0.032	0.393	0.191	0.000	0.000	0	3.610	0.320	3.930	1.910	0.000	0.000
120	alpha	1.2	60x8 weeks	PCR90	MWF	VAC95	100	Unvaccinate	0.000	0.001	0.000	0.001	0.000	0.000	0.000	0.000	0.369	0.042	0.411	0.194	0.000	0.000	0	3.690	0.420	4.110	1.940	0.000	0.000
120	alpha	1.2	60x8 weeks	PCR90	MWF	VAC95	100	Unvaccinate	0.000	0.001	0.000	0.001	0.000	0.000	0.000	0.000	0.369	0.042	0.411	0.194	0.000	0.000	0	3.690	0.420	4.110	1.940	0.000	0.000
120	alpha	1.2	60x8 weeks	PCR90	MWF	VAC95	100	Unvaccinate	0.000	0.001	0.000	0.001	0.000	0.000	0.000	0.000	0.369	0.042	0.411	0.194	0.000	0.000	0	3.690	0.420	4.110	1.940	0.000	0.000
120	delta	0.6	60x8 weeks	PCR90	MWF	VAC95	100	Unvaccinate	0.000	0.001	0.000	0.001	0.001	0.000	0.000	0.000	0.579	0.131	0.710	0.369	0.000	0.000	0	5.790	1.310	7.100	3.690	0.000	0.000
120	delta	0.6	60x8 weeks	PCR90	MWF	VAC95	100	Unvaccinate	0.000	0.001	0.000	0.001	0.001	0.000	0.000	0.000	0.579	0.131	0.710	0.369	0.000	0.000	0	5.790	1.310	7.100	3.690	0.000	0.000
120	delta	0.6	60x8 weeks	PCR90	MWF	VAC95	100	Unvaccinate	0.000	0.001	0.000	0.001	0.001	0.000	0.000	0.000	0.579	0.131	0.710	0.369	0.000	0.000	0	5.790	1.310	7.100	3.690	0.000	0.000
120	delta	1	60x8 weeks	PCR90	MWF	VAC95	100	Unvaccinate	0.000	0.001	0.000	0.002	0.001	0.000	0.000	0.000	0.553	0.218	0.771	0.400	0.000	0.000	0	5.530	2.180	7.710	4.000	0.000	0.000
120	delta	1	60x8 weeks	PCR90	MWF	VAC95	100	Unvaccinate	0.000	0.001	0.000	0.002	0.001	0.000	0.000	0.000	0.553	0.218	0.771	0.400	0.000	0.000	0	5.530	2.180	7.710	4.000	0.000	0.000
120	delta	1	60x8 weeks	PCR90	MWF	VAC95	100	Unvaccinate	0.000	0.001	0.000	0.002	0.001	0.000	0.000	0.000	0.553	0.218	0.771	0.400	0.000	0.000	0	5.530	2.180	7.710	4.000	0.000	0.000
120	delta	1.2	60x8 weeks	PCR90	MWF	VAC95	100	Unvaccinate	0.000	0.001	0.001	0.002	0.001	0.000	0.000	0.000	0.583	0.293	0.876	0.471	0.000	0.000	0	5.830	2.930	8.760	4.710	0.000	0.000
120	delta	1.2	60x8 weeks	PCR90	MWF	VAC95	100	Unvaccinate	0.000	0.001	0.001	0.002	0.001	0.000	0.000	0.000	0.583	0.293	0.876	0.471	0.000	0.000	0	5.830	2.930	8.760	4.710	0.000	0.000
120	delta	1.2	60x8 weeks	PCR90	MWF	VAC95	100	Unvaccinate	0.000	0.001	0.001	0.002	0.001	0.000	0.000	0.000	0.583	0.293	0.876	0.471	0.000	0.000	0	5.830	2.930	8.760	4.710	0.000	0.000

120	original	0.6	60x8 weeks	PCR90	MWF	VAC70	25	Jnvaccinate	2.223	0.007	0.002	0.008	0.001	0.007	0.002	1066.971	3.172	0.776	3.948	0.570	3.293	1.064	10670	31.720	7.760	39.480	5.700	32.930	10.640
120	original	0.6	60x8 weeks	PCR90	MWF	VAC70	25	Jnvaccinate	2.223	0.007	0.002	0.008	0.001	0.007	0.002	1066.971	3.172	0.776	3.948	0.570	3.293	1.064	10670	31.720	7.760	39.480	5.700	32.930	10.640
120	original	0.6	60x8 weeks	PCR90	MWF	VAC70	25	Jnvaccinate	2.223	0.007	0.002	0.008	0.001	0.007	0.002	1066.971	3.172	0.776	3.948	0.570	3.293	1.064	10670	31.720	7.760	39.480	5.700	32.930	10.640
120	original	1	60x8 weeks	PCR90	MWF	VAC70	25	Jnvaccinate	2.217	0.007	0.003	0.010	0.001	0.008	0.002	1064.365	3.172	1.567	4.739	0.679	3.929	1.021	10644	31.720	15.670	47.390	6.790	39.290	10.210
120	original	1	60x8 weeks	PCR90	MWF	VAC70	25	Jnvaccinate	2.217	0.007	0.003	0.010	0.001	0.008	0.002	1064.365	3.172	1.567	4.739	0.679	3.929	1.021	10644	31.720	15.670	47.390	6.790	39.290	10.210
120	original	1	60x8 weeks	PCR90	MWF	VAC70	25	Jnvaccinate	2.217	0.007	0.003	0.010	0.001	0.008	0.002	1064.365	3.172	1.567	4.739	0.679	3.929	1.021	10644	31.720	15.670	47.390	6.790	39.290	10.210
120	original	1.2	60x8 weeks	PCR90	MWF	VAC70	25	Jnvaccinate	2.213	0.007	0.005	0.011	0.002	0.009	0.002	1062.379	3.229	2.191	5.420	0.726	4.464	0.994	10624	32.290	21.910	54.200	7.260	44.640	9.940
120	original	1.2	60x8 weeks	PCR90	MWF	VAC70	25	Jnvaccinate	2.213	0.007	0.005	0.011	0.002	0.009	0.002	1062.379	3.229	2.191	5.420	0.726	4.464	0.994	10624	32.290	21.910	54.200	7.260	44.640	9.940
120	original	1.2	60x8 weeks	PCR90	MWF	VAC70	25	Jnvaccinate	2.213	0.007	0.005	0.011	0.002	0.009	0.002	1062.379	3.229	2.191	5.420	0.726	4.464	0.994	10624	32.290	21.910	54.200	7.260	44.640	9.940
120	alpha	0.6	60x8 weeks	PCR90	MWF	VAC70	25	Jnvaccinate	2.211	0.007	0.005	0.011	0.002	0.010	0.002	1061.453	3.199	2.278	5.477	0.849	4.654	1.061	10615	31.990	22.780	54.770	8.490	46.540	10.610
120	alpha	0.6	60x8 weeks	PCR90	MWF	VAC70	25	Jnvaccinate	2.211	0.007	0.005	0.011	0.002	0.010	0.002	1061.453	3.199	2.278	5.477	0.849	4.654	1.061	10615	31.990	22.780	54.770	8.490	46.540	10.610
120	alpha	0.6	60x8 weeks	PCR90	MWF	VAC70	25	Jnvaccinate	2.211	0.007	0.005	0.011	0.002	0.010	0.002	1061.453	3.199	2.278	5.477	0.849	4.654	1.061	10615	31.990	22.780	54.770	8.490	46.540	10.610
120	alpha	1	60x8 weeks	PCR90	MWF	VAC70	25	Jnvaccinate	2.183	0.007	0.013	0.020	0.003	0.017	0.002	1047.843	3.136	6.296	9.432	1.542	8.028	0.946	10478	31.360	62.960	94.320	15.420	80.280	9.460
120	alpha	1	60x8 weeks	PCR90	MWF	VAC70	25	Jnvaccinate	2.183	0.007	0.013	0.020	0.003	0.017	0.002	1047.843	3.136	6.296	9.432	1.542	8.028	0.946	10478	31.360	62.960	94.320	15.420	80.280	9.460
120	alpha	1	60x8 weeks	PCR90	MWF	VAC70	25	Jnvaccinate	2.183	0.007	0.013	0.020	0.003	0.017	0.002	1047.843	3.136	6.296	9.432	1.542	8.028	0.946	10478	31.360	62.960	94.320	15.420	80.280	9.460
120	alpha	1.2	60x8 weeks	PCR90	MWF	VAC70	25	Jnvaccinate	2.157	0.006	0.021	0.027	0.005	0.023	0.002	1035.577	2.911	10.046	12.957	2.195	10.926	0.923	10356	29.110	100.460	129.570	21.950	109.260	9.230
120	alpha	1.2	60x8 weeks	PCR90	MWF	VAC70	25	Jnvaccinate	2.157	0.006	0.021	0.027	0.005	0.023	0.002	1035.577	2.911	10.046	12.957	2.195	10.926	0.923	10356	29.110	100.460	129.570	21.950	109.260	9.230
120	alpha	1.2	60x8 weeks	PCR90	MWF	VAC70	25	Jnvaccinate	2.157	0.006	0.021	0.027	0.005	0.023	0.002	1035.577	2.911	10.046	12.957	2.195	10.926	0.923	10356	29.110	100.460	129.570	21.950	109.260	9.230
120	delta	0.6	60x8 weeks	PCR90	MWF	VAC70	25	Jnvaccinate	2.134	0.006	0.029	0.035	0.008	0.027	0.002	1024.283	2.955	14.039	16.994	4.037	12.869	0.931	10243	29.550	140.390	169.940	40.370	128.690	9.310
120	delta	0.6	60x8 weeks	PCR90	MWF	VAC70	25	Jnvaccinate	2.134	0.006	0.029	0.035	0.008	0.027	0.002	1024.283	2.955	14.039	16.994	4.037	12.869	0.931	10243	29.550	140.390	169.940	40.370	128.690	9.310
120	delta	0.6	60x8 weeks	PCR90	MWF	VAC70	25	Jnvaccinate	2.134	0.006	0.029	0.035	0.008	0.027	0.002	1024.283	2.955	14.039	16.994	4.037	12.869	0.931	10243	29.550	140.390	169.940	40.370	128.690	9.310
120	delta	1	60x8 weeks	PCR90	MWF	VAC70	25	Jnvaccinate	1.973	0.005	0.081	0.086	0.022	0.062	0.001	947.026	2.195	38.894	41.089	10.773	29.749	0.650	9470	21.950	388.940	410.890	107.730	297.490	6.500
120	delta	1	60x8 weeks	PCR90	MWF	VAC70	25	Jnvaccinate	1.973	0.005	0.081	0.086	0.022	0.062	0.001	947.026	2.195	38.894	41.089	10.773	29.749	0.650	9470	21.950	388.940	410.890	107.730	297.490	6.500
120	delta	1	60x8 weeks	PCR90	MWF	VAC70	25	Jnvaccinate	1.973	0.005	0.081	0.086	0.022	0.062	0.001	947.026	2.195	38.894	41.089	10.773	29.749	0.650	9470	21.950	388.940	410.890	107.730	297.490	6.500
120	delta	1.2	60x8 weeks	PCR90	MWF	VAC70	25	Jnvaccinate	1.927	0.004	0.097	0.101	0.028	0.072	0.001	925.031	1.910	46.446	48.356	13.211	34.440	0.610	9250	19.100	464.460	483.560	132.110	344.400	6.100
120	delta	1.2	60x8 weeks	PCR90	MWF	VAC70	25	Jnvaccinate	1.927	0.004	0.097	0.101	0.028	0.072	0.001	925.031	1.910	46.446	48.356	13.211	34.440	0.610	9250	19.100	464.460	483.560	132.110	344.400	6.100
120	delta	1.2	60x8 weeks	PCR90	MWF	VAC70	25	Jnvaccinate	1.927	0.004	0.097	0.101	0.028	0.072	0.001	925.031	1.910	46.446	48.356	13.211	34.440	0.610	9250	19.100	464.460	483.560	132.110	344.400	6.100
120	original	0.6	60x8 weeks	PCR90	MWF	VAC70	50	Jnvaccinate	1.482	0.005	0.001	0.006	0.001	0.005	0.001	711.231	2.639	0.446	3.085	0.614	2.173	0.706	7112	26.390	4.460	30.850	6.140	21.730	7.060
120	original	0.6	60x8 weeks	PCR90	MWF	VAC70	50	Jnvaccinate	1.482	0.005	0.001	0.006	0.001	0.005	0.001	711.231	2.639	0.446	3.085	0.614	2.173	0.706	7112	26.390	4.460	30.850	6.140	21.730	7.060
120	original	0.6	60x8 weeks	PCR90	MWF	VAC70	50	Jnvaccinate	1.482	0.005	0.001	0.006	0.001	0.005	0.001	711.231	2.639	0.446	3.085	0.614	2.173	0.706	7112	26.390	4.460	30.850	6.140	21.730	7.060
120	original	1	60x8 weeks	PCR90	MWF	VAC70	50	Jnvaccinate	1.482	0.005	0.001	0.007	0.001	0.005	0.001	711.194	2.512	0.697	3.209	0.622	2.237	0.670	7112	25.120	6.970	32.090	6.220	22.370	6.700
120	original	1	60x8 weeks	PCR90	MWF	VAC70	50	Jnvaccinate	1.482	0.005	0.001	0.007	0.001	0.005	0.001	711.194	2.512	0.697	3.209	0.622	2.237	0.670	7112	25.120	6.970	32.090	6.220	22.370	6.700
120	original	1	60x8 weeks	PCR90	MWF	VAC70	50	Jnvaccinate	1.482	0.005	0.001	0.007	0.001	0.005	0.001	711.194	2.512	0.697	3.209	0.622	2.237	0.670	7112	25.120	6.970	32.090	6.220	22.370	6.700
120	original	1.2	60x8 weeks	PCR90	MWF	VAC70	50	Jnvaccinate	1.481	0.005	0.002	0.007	0.001	0.005	0.001	710.642	2.504	0.914	3.418	0.683	2.358	0.684	7106	25.040	9.140	34.180	6.830	23.580	6.840
120	original	1.2	60x8 weeks	PCR90	MWF	VAC70	50	Jnvaccinate	1.481	0.005	0.002	0.007	0.001	0.005	0.001	710.642	2.504	0.914	3.418	0.683	2.358	0.684	7106	25.040	9.140	34.180	6.830	23.580	6.840
120	original	1.2	60x8 weeks	PCR90	MWF	VAC70	50	Jnvaccinate	1.481	0.005	0.002	0.007	0.001	0.005	0.001	710.642	2.504	0.914	3.418	0.683	2.358	0.684	7106	25.040	9.140	34.180	6.830	23.580	6.840
120	alpha	0.6	60x8 weeks	PCR90	MWF	VAC70	50	Jnvaccinate	1.477	0.006	0.003	0.008	0.002	0.006	0.001	708.892	2.641	1.307	3.948	0.926	2.765	0.688	7089	26.410	13.070	39.480	9.260	27.650	6.880
120	alpha	0.6	60x8 weeks	PCR90	MWF	VAC70	50	Jnvaccinate	1.477	0.006	0.003	0.008	0.002	0.006	0.001	708.892	2.641	1.307	3.948	0.926	2.765	0.688	7089	26.410	13.070	39.480	9.260	27.650	6.880
120	alpha	0.6	60x8 weeks	PCR90	MWF	VAC70	50	Jnvaccinate	1.477	0.006	0.003	0.008	0.002	0.006	0.001	708.892	2.641	1.307	3.948	0.926	2.765	0.688	7089	26.410	13.070	39.480	9.260	27.650	6.880
120	alpha	1	60x8 weeks	PCR90	MWF	VAC70	50	Jnvaccinate	1.467	0.005	0.007	0.012	0.003	0.008	0.001	704.050	2.511	3.162	5.673	1.365	3.943	0.711	7041	25.110	31.620	56.730	13.650	39.430	7.110
120	alpha	1	60x8 weeks	PCR90	MWF	VAC70	50	Jnvaccinate	1.467	0.005	0.007	0.012	0.003	0.008	0.001	704.050	2.511	3.162	5.673	1.365	3.943	0.711	7041	25.110	31.620	56.730	13.650	39.430	7.110
120	alpha	1	60x8 weeks	PCR90	MWF	VAC70	50	Jnvaccinate	1.467	0.005	0.007	0.012	0.003	0.008	0.001	704.050	2.511	3.162	5.673	1.365	3.943	0.711	7041	25.110	31.620	56.730	13.650	39.430	7.110
120	alpha	1.2	60x8 weeks	PCR90	MWF	VAC70	50	Jnvaccinate	1.460	0.005	0.009	0.014	0.003	0.010	0.001	700.642	2.581	4.348	6.929	1.679	4.804	0.625	7006	25.810	43.480	69.290	16.790	48.040	6.250
120	alpha	1.2	60x8 weeks	PCR90	MWF	VAC70	50	Jnvaccinate	1.460	0.005	0.009	0.014	0.003	0.010	0.001	700.642	2.581	4.348	6.929	1.679	4.804	0.625	7006	25.810	43.480	69.290	16.790	48.040	6.250
120	alpha	1.2	60x8 weeks	PCR90	MWF	VAC70	50	Jnvaccinate	1.460	0.005	0.009	0.014	0.003	0.010	0.001	700.642	2.581	4.348	6.929	1.679	4.804	0.625	7006	25.810	43.480	69.290	16.790	48.040	6.250
120	delta	0.6	60x8 weeks	PCR90	MWF	VAC70	50	Jnvaccinate	1.449	0.005	0.015	0.020	0.006	0.012	0.001	695.551	2.632	6.966	9.598	2.988	5.735	0.681	6956	26.320	69.660	95.980	29.880	57.350	6.810
120	delta	0.6	60x8 weeks	PCR90	MWF	VAC70	50	Jnvaccinate	1.449	0.005	0.015	0.020	0.006	0.012	0.001	695.551	2.632	6.966	9.598	2.988	5.735	0.681	6956	26.320	69.660	95.980	29.880	57.350	6.810
120	delta	0.6	60x8 weeks	PCR90	MWF	VAC70	50	Jnvaccinate	1.449	0.005	0.015	0.020	0.006	0.012	0.001	695.551	2.632	6.966	9.598	2.988	5.735	0.681	6956	26.320	69.660	95.980	29.880	57.350	6.810
120	delta	1	60x8 weeks	PCR90	MWF	VAC70	50	Jnvaccinate	1.373	0.005	0.048	0.053	0.018	0.029	0.001	659.092	2.207	23.192	25.399	8.576	14.099	0.540	6591	22.070	231.920	253.990	85.760	140.990	5.400
120	delta	1	60x8 weeks	PCR90	MWF	VAC70	50	Jnvaccinate	1.373	0.005	0.048	0.053	0.018	0.029	0.001	659.092	2.207	23.192	25.399	8.576	14.099	0.540	6591	22.070	231.920	253.990	85.760	140.990	5.400
120	delta	1	60x8 weeks	PCR90	MWF	VAC70	50	Jnvaccinate	1.373	0.005	0.048	0.053	0.018	0.029	0.001	659.092	2.207	23.192	25.399	8.576	14.099	0.540	6591	22.070	231.920	253.990	85.760	140.990	5.400
120	delta	1.2	60x8 weeks	PCR90	MWF	VAC70	50	Jnvaccinate	1.330	0.004	0.069	0.073	0.026	0.039	0.001	638.450	1.958	33.226	35.184	12.663	18.558	0.475	6385	19.580	332.260	351.840	126.630	185.580	4.750
120	delta	1.2	60x8 weeks	PCR90	MWF	VAC70	50	Jnvaccinate	1.330	0.004	0.069	0.073	0.026	0.039	0.001	638.450	1.958	33.226	35.184	12.663	18.558	0.475	6385	19.580	332.260	351.840	126.630	185.580	4.750
120	delta	1.2	60x8 weeks	PCR90	MWF	VAC70	50	Jnvaccinate	1.330	0.004	0.069	0.073	0.026	0.039	0.001	638.450	1.958	33.226	35.184	12.663	18.558	0.475	6385	19.580	332.260	351.840	126.630	185.580	4.750
120	original	0.6	60x8 weeks	PCR90	MWF	VAC70	75	Jnvaccinate	0.742	0.004	0.000	0.004	0.001	0.002	0.001	356.325	1.920	0.145	2.065	0.607	0.917	0.373	3563	19.200	1.450	20.650	6.070	9.170	3.730
120	original	0.6	60x8 weeks	PCR90	MWF	VAC70	75	Jnvaccinate	0.742	0.004	0.000	0.004	0.001	0.002	0.001	356.325	1.920	0.145	2.065	0.607	0.917	0.373	3563	19.200	1.450	20.650	6.070	9.170	3.730
120	original	0.6	60x8 weeks	PCR90	MWF	VAC70	75	Jnvaccinate	0.742	0.004	0.000	0.004	0.001	0.002	0.001	356.325	1.920	0.145	2.065	0.607	0.917	0.373	3563	19.200	1.450	20.650	6.070	9.170	3.730
120	original	1	60x8 weeks	PCR90	MWF	VAC70	75	Jnvaccinate	0.742	0.004	0.001	0.005	0.001	0.002	0.001	356.073	1.942	0.267	2.209	0.665	0.995	0.313	3561	19.420	2.670	22.090	6.650	9.950	3.130
120	original	1	60x8 weeks	PCR90	MWF	VAC70	75	Jnvaccinate	0.742	0.004	0.001	0.005	0.001	0.002	0.001	356.073	1.942	0.267	2.209	0.665	0.995	0.313	3561	19.420	2.670	22.090	6.650	9.950	3.130
120	original	1	60x8 weeks	PCR90	MWF	VAC70	75	Jnvaccinate	0.742	0.004	0.001	0.005	0.001	0.002	0.001	356.073	1.942	0.267	2.209	0.665	0.995	0.313	3561	19.420	2.670	22.090	6.650	9.950	3.130
120	original	1.2	60x8 weeks	PCR90	MWF	VAC70	75	Jnvaccinate	0.742	0.004	0.001	0.005	0.001	0.002	0.001	356.058	1.968	0.301	2.269	0.694	0.974	0.346	3561	19.680	3.010	22.690	6.940	9.740	3.460
120	original	1.2	60x8 weeks	PCR90	MWF	VAC70	75	Jnvaccinate	0.742	0.004	0.001	0.005	0.001	0.002	0.001	356.058	1.968	0.301	2.269	0.694	0.974	0.346	3561	19.680	3.010	22.690	6.940	9.740	3.460
120	original	1.2	60x8 weeks	PCR90	MWF	VAC70	75	Jnvaccinate	0.742	0.004	0.001	0.005	0.001	0.002	0.001	356.058	1.968	0.301	2.269	0.694	0.974	0.346	3561	19.680	3.010	22.690	6.940	9.740	3.460
120	alpha	0.6	60x8 weeks	PCR90	MWF	VAC70	75	Jnvaccinate	0.740	0.004	0.001	0.005	0.002	0.003	0.001	355.112	1.881	0.675	2.556	0.880	1.218	0.329	3551	18.810	6.750	25.560	8.800	12.180	3.290
120	alpha	0.6	60x8 weeks	PCR90	MWF	VAC70	75	Jnvaccinate	0.740	0.004	0.001	0.005	0.002	0.003	0.001	355.112	1.881	0.675	2.556	0.880	1.218	0.329	3551	18.810	6.750	25.560	8.800	12.180	3.290
120	alpha	0.6	60x8 weeks	PCR90	MWF	VAC70	75	Jnvaccinate	0.740	0.004	0.001	0.005	0.002	0.003	0.001	355.112	1.881	0.675	2.556	0.880	1.218	0.329	3551	18.810	6.750	25.560	8.800	12.180	3.290
120	alpha	1	60x8 weeks	PCR90	MWF	VAC70	75	Jnvaccinate	0.737	0.004	0.003	0.007	0.002	0.003	0.001	353.809	1.847	1.402	3.249	1.139	1.542	0.344	3538	18.470	14.020	32.490	11.390	15.420	3.440
120	alpha	1	60x8 weeks	PCR90	MWF	VAC70	75	Jnvaccinate	0.737	0.004	0.003	0.007	0.002	0.003	0.001	353.809	1.847	1.402	3.249	1.139	1.542	0.344	3538	18.470	14.020	32.490	11.390	15.420	3.440
120	alpha	1	60x8 weeks	PCR90	MWF	VAC70	75	Jnvaccinate	0.737	0.004	0.003	0.007	0.002	0.003	0.001	353.809	1.847	1.402	3.249	1.139	1.542	0.344	3538	18.470	14.020	32.490	11.390	15.420	3.440
120	alpha	1.2	60x8 weeks	PCR90	MWF	VAC70	75	Jnvaccinate	0.735	0.004	0.004	0.008	0.003	0.004	0.001	352.930	1.966	1.916	3.882	1.369	1.778	0.325	3529	19.660	19.160	38.820	13.690	17.780	3.250
120	alpha	1.2	60x8 weeks	PCR90	MWF	VAC70	75	Jnvaccinate	0.735	0.004	0.004	0.008	0.003	0.004	0.001	352.930	1.966	1.916	3.882	1.369	1.778	0.325	3529	19.660	19.160	38.820	13.690	17.780	3.250

120	alpha	1.2	60x8 weeks	PCR90	MWF	VAC70	75	Unvaccinate	0.735	0.004	0.004	0.008	0.003	0.004	0.001	352.930	1.966	1.916	3.882	1.369	1.778	0.325	3529	19.660	19.160	38.820	13.690	17.780	3.250
120	delta	0.6	60x8 weeks	PCR90	MWF	VAC70	75	Unvaccinate	0.732	0.004	0.007	0.011	0.005	0.004	0.001	351.599	2.123	3.241	5.364	2.276	1.958	0.331	3516	21.230	32.410	53.640	22.760	19.580	3.310
120	delta	0.6	60x8 weeks	PCR90	MWF	VAC70	75	Unvaccinate	0.732	0.004	0.007	0.011	0.005	0.004	0.001	351.599	2.123	3.241	5.364	2.276	1.958	0.331	3516	21.230	32.410	53.640	22.760	19.580	3.310
120	delta	0.6	60x8 weeks	PCR90	MWF	VAC70	75	Unvaccinate	0.732	0.004	0.007	0.011	0.005	0.004	0.001	351.599	2.123	3.241	5.364	2.276	1.958	0.331	3516	21.230	32.410	53.640	22.760	19.580	3.310
120	delta	1	60x8 weeks	PCR90	MWF	VAC70	75	Unvaccinate	0.709	0.004	0.024	0.028	0.013	0.010	0.001	340.176	2.046	11.476	13.522	6.102	4.587	0.293	3402	20.460	114.760	135.220	61.020	45.870	2.930
120	delta	1	60x8 weeks	PCR90	MWF	VAC70	75	Unvaccinate	0.709	0.004	0.024	0.028	0.013	0.010	0.001	340.176	2.046	11.476	13.522	6.102	4.587	0.293	3402	20.460	114.760	135.220	61.020	45.870	2.930
120	delta	1	60x8 weeks	PCR90	MWF	VAC70	75	Unvaccinate	0.709	0.004	0.024	0.028	0.013	0.010	0.001	340.176	2.046	11.476	13.522	6.102	4.587	0.293	3402	20.460	114.760	135.220	61.020	45.870	2.930
120	delta	1.2	60x8 weeks	PCR90	MWF	VAC70	75	Unvaccinate	0.696	0.004	0.036	0.040	0.018	0.012	0.001	334.298	1.817	17.197	19.014	8.774	5.934	0.268	3343	18.170	171.970	190.140	87.740	59.340	2.680
120	delta	1.2	60x8 weeks	PCR90	MWF	VAC70	75	Unvaccinate	0.696	0.004	0.036	0.040	0.018	0.012	0.001	334.298	1.817	17.197	19.014	8.774	5.934	0.268	3343	18.170	171.970	190.140	87.740	59.340	2.680
120	delta	1.2	60x8 weeks	PCR90	MWF	VAC70	75	Unvaccinate	0.696	0.004	0.036	0.040	0.018	0.012	0.001	334.298	1.817	17.197	19.014	8.774	5.934	0.268	3343	18.170	171.970	190.140	87.740	59.340	2.680
120	original	0.6	60x8 weeks	PCR90	MWF	VAC70	100	Unvaccinate	0.000	0.003	0.000	0.003	0.001	0.000	0.000	0.000	1.251	0.012	1.263	0.638	0.000	0.000	0	12.510	0.120	12.630	6.380	0.000	0.000
120	original	0.6	60x8 weeks	PCR90	MWF	VAC70	100	Unvaccinate	0.000	0.003	0.000	0.003	0.001	0.000	0.000	0.000	1.251	0.012	1.263	0.638	0.000	0.000	0	12.510	0.120	12.630	6.380	0.000	0.000
120	original	0.6	60x8 weeks	PCR90	MWF	VAC70	100	Unvaccinate	0.000	0.003	0.000	0.003	0.001	0.000	0.000	0.000	1.251	0.012	1.263	0.638	0.000	0.000	0	12.510	0.120	12.630	6.380	0.000	0.000
120	original	1	60x8 weeks	PCR90	MWF	VAC70	100	Unvaccinate	0.000	0.003	0.000	0.003	0.001	0.000	0.000	0.000	1.258	0.017	1.275	0.598	0.000	0.000	0	12.580	0.170	12.750	5.980	0.000	0.000
120	original	1	60x8 weeks	PCR90	MWF	VAC70	100	Unvaccinate	0.000	0.003	0.000	0.003	0.001	0.000	0.000	0.000	1.258	0.017	1.275	0.598	0.000	0.000	0	12.580	0.170	12.750	5.980	0.000	0.000
120	original	1	60x8 weeks	PCR90	MWF	VAC70	100	Unvaccinate	0.000	0.003	0.000	0.003	0.001	0.000	0.000	0.000	1.258	0.017	1.275	0.598	0.000	0.000	0	12.580	0.170	12.750	5.980	0.000	0.000
120	original	1.2	60x8 weeks	PCR90	MWF	VAC70	100	Unvaccinate	0.000	0.003	0.000	0.003	0.001	0.000	0.000	0.000	1.339	0.021	1.360	0.651	0.000	0.000	0	13.390	0.210	13.600	6.510	0.000	0.000
120	original	1.2	60x8 weeks	PCR90	MWF	VAC70	100	Unvaccinate	0.000	0.003	0.000	0.003	0.001	0.000	0.000	0.000	1.339	0.021	1.360	0.651	0.000	0.000	0	13.390	0.210	13.600	6.510	0.000	0.000
120	original	1.2	60x8 weeks	PCR90	MWF	VAC70	100	Unvaccinate	0.000	0.003	0.000	0.003	0.001	0.000	0.000	0.000	1.339	0.021	1.360	0.651	0.000	0.000	0	13.390	0.210	13.600	6.510	0.000	0.000
120	alpha	0.6	60x8 weeks	PCR90	MWF	VAC70	100	Unvaccinate	0.000	0.003	0.001	0.003	0.002	0.000	0.000	0.000	1.279	0.241	1.520	0.892	0.000	0.000	0	12.790	2.410	15.200	8.920	0.000	0.000
120	alpha	0.6	60x8 weeks	PCR90	MWF	VAC70	100	Unvaccinate	0.000	0.003	0.001	0.003	0.002	0.000	0.000	0.000	1.279	0.241	1.520	0.892	0.000	0.000	0	12.790	2.410	15.200	8.920	0.000	0.000
120	alpha	0.6	60x8 weeks	PCR90	MWF	VAC70	100	Unvaccinate	0.000	0.003	0.001	0.003	0.002	0.000	0.000	0.000	1.279	0.241	1.520	0.892	0.000	0.000	0	12.790	2.410	15.200	8.920	0.000	0.000
120	alpha	1	60x8 weeks	PCR90	MWF	VAC70	100	Unvaccinate	0.000	0.003	0.001	0.004	0.002	0.000	0.000	0.000	1.288	0.510	1.798	1.068	0.000	0.000	0	12.880	5.100	17.980	10.680	0.000	0.000
120	alpha	1	60x8 weeks	PCR90	MWF	VAC70	100	Unvaccinate	0.000	0.003	0.001	0.004	0.002	0.000	0.000	0.000	1.288	0.510	1.798	1.068	0.000	0.000	0	12.880	5.100	17.980	10.680	0.000	0.000
120	alpha	1	60x8 weeks	PCR90	MWF	VAC70	100	Unvaccinate	0.000	0.003	0.001	0.004	0.002	0.000	0.000	0.000	1.288	0.510	1.798	1.068	0.000	0.000	0	12.880	5.100	17.980	10.680	0.000	0.000
120	alpha	1.2	60x8 weeks	PCR90	MWF	VAC70	100	Unvaccinate	0.000	0.003	0.001	0.004	0.002	0.000	0.000	0.000	1.224	0.625	1.849	1.049	0.000	0.000	0	12.240	6.250	18.490	10.490	0.000	0.000
120	alpha	1.2	60x8 weeks	PCR90	MWF	VAC70	100	Unvaccinate	0.000	0.003	0.001	0.004	0.002	0.000	0.000	0.000	1.224	0.625	1.849	1.049	0.000	0.000	0	12.240	6.250	18.490	10.490	0.000	0.000
120	alpha	1.2	60x8 weeks	PCR90	MWF	VAC70	100	Unvaccinate	0.000	0.003	0.001	0.004	0.002	0.000	0.000	0.000	1.224	0.625	1.849	1.049	0.000	0.000	0	12.240	6.250	18.490	10.490	0.000	0.000
120	delta	0.6	60x8 weeks	PCR90	MWF	VAC70	100	Unvaccinate	0.000	0.004	0.003	0.006	0.004	0.000	0.000	0.000	1.700	1.400	3.100	1.907	0.000	0.000	0	17.000	14.000	31.000	19.070	0.000	0.000
120	delta	0.6	60x8 weeks	PCR90	MWF	VAC70	100	Unvaccinate	0.000	0.004	0.003	0.006	0.004	0.000	0.000	0.000	1.700	1.400	3.100	1.907	0.000	0.000	0	17.000	14.000	31.000	19.070	0.000	0.000
120	delta	0.6	60x8 weeks	PCR90	MWF	VAC70	100	Unvaccinate	0.000	0.004	0.003	0.006	0.004	0.000	0.000	0.000	1.700	1.400	3.100	1.907	0.000	0.000	0	17.000	14.000	31.000	19.070	0.000	0.000
120	delta	1	60x8 weeks	PCR90	MWF	VAC70	100	Unvaccinate	0.000	0.003	0.008	0.011	0.007	0.000	0.000	0.000	1.553	3.811	5.364	3.320	0.000	0.000	0	15.530	38.110	53.640	33.200	0.000	0.000
120	delta	1	60x8 weeks	PCR90	MWF	VAC70	100	Unvaccinate	0.000	0.003	0.008	0.011	0.007	0.000	0.000	0.000	1.553	3.811	5.364	3.320	0.000	0.000	0	15.530	38.110	53.640	33.200	0.000	0.000
120	delta	1	60x8 weeks	PCR90	MWF	VAC70	100	Unvaccinate	0.000	0.003	0.008	0.011	0.007	0.000	0.000	0.000	1.553	3.811	5.364	3.320	0.000	0.000	0	15.530	38.110	53.640	33.200	0.000	0.000
120	delta	1.2	60x8 weeks	PCR90	MWF	VAC70	100	Unvaccinate	0.000	0.003	0.013	0.016	0.010	0.000	0.000	0.000	1.641	6.121	7.762	4.704	0.000	0.000	0	16.410	61.210	77.620	47.040	0.000	0.000
120	delta	1.2	60x8 weeks	PCR90	MWF	VAC70	100	Unvaccinate	0.000	0.003	0.013	0.016	0.010	0.000	0.000	0.000	1.641	6.121	7.762	4.704	0.000	0.000	0	16.410	61.210	77.620	47.040	0.000	0.000
120	delta	1.2	60x8 weeks	PCR90	MWF	VAC70	100	Unvaccinate	0.000	0.003	0.013	0.016	0.010	0.000	0.000	0.000	1.641	6.121	7.762	4.704	0.000	0.000	0	16.410	61.210	77.620	47.040	0.000	0.000
120	original	0.6	60x8 weeks	PCR90	MWF	VAC50	25	Unvaccinate	2.222	0.007	0.002	0.009	0.001	0.007	0.002	1066.557	3.447	0.822	4.269	0.679	3.383	1.041	10666	34.470	8.220	42.690	6.790	33.830	10.410
120	original	0.6	60x8 weeks	PCR90	MWF	VAC50	25	Unvaccinate	2.222	0.007	0.002	0.009	0.001	0.007	0.002	1066.557	3.447	0.822	4.269	0.679	3.383	1.041	10666	34.470	8.220	42.690	6.790	33.830	10.410
120	original	0.6	60x8 weeks	PCR90	MWF	VAC50	25	Unvaccinate	2.222	0.007	0.002	0.009	0.001	0.007	0.002	1066.557	3.447	0.822	4.269	0.679	3.383	1.041	10666	34.470	8.220	42.690	6.790	33.830	10.410
120	original	1	60x8 weeks	PCR90	MWF	VAC50	25	Unvaccinate	2.217	0.007	0.004	0.011	0.002	0.008	0.002	1064.095	3.457	1.688	5.145	0.851	4.007	1.025	10641	34.570	16.880	51.450	8.510	40.070	10.250
120	original	1	60x8 weeks	PCR90	MWF	VAC50	25	Unvaccinate	2.217	0.007	0.004	0.011	0.002	0.008	0.002	1064.095	3.457	1.688	5.145	0.851	4.007	1.025	10641	34.570	16.880	51.450	8.510	40.070	10.250
120	original	1	60x8 weeks	PCR90	MWF	VAC50	25	Unvaccinate	2.217	0.007	0.004	0.011	0.002	0.008	0.002	1064.095	3.457	1.688	5.145	0.851	4.007	1.025	10641	34.570	16.880	51.450	8.510	40.070	10.250
120	original	1.2	60x8 weeks	PCR90	MWF	VAC50	25	Unvaccinate	2.214	0.007	0.005	0.012	0.002	0.009	0.002	1062.559	3.279	2.252	5.531	0.879	4.339	1.050	10626	32.790	22.520	55.310	8.790	43.390	10.500
120	original	1.2	60x8 weeks	PCR90	MWF	VAC50	25	Unvaccinate	2.214	0.007	0.005	0.012	0.002	0.009	0.002	1062.559	3.279	2.252	5.531	0.879	4.339	1.050	10626	32.790	22.520	55.310	8.790	43.390	10.500
120	original	1.2	60x8 weeks	PCR90	MWF	VAC50	25	Unvaccinate	2.214	0.007	0.005	0.012	0.002	0.009	0.002	1062.559	3.279	2.252	5.531	0.879	4.339	1.050	10626	32.790	22.520	55.310	8.790	43.390	10.500
120	alpha	0.6	60x8 weeks	PCR90	MWF	VAC50	25	Unvaccinate	2.209	0.007	0.006	0.013	0.002	0.010	0.002	1060.235	3.408	2.731	6.139	1.144	4.923	1.052	10602	34.080	27.310	61.390	11.440	49.230	10.520
120	alpha	0.6	60x8 weeks	PCR90	MWF	VAC50	25	Unvaccinate	2.209	0.007	0.006	0.013	0.002	0.010	0.002	1060.235	3.408	2.731	6.139	1.144	4.923	1.052	10602	34.080	27.310	61.390	11.440	49.230	10.520
120	alpha	0.6	60x8 weeks	PCR90	MWF	VAC50	25	Unvaccinate	2.209	0.007	0.006	0.013	0.002	0.010	0.002	1060.235	3.408	2.731	6.139	1.144	4.923	1.052	10602	34.080	27.310	61.390	11.440	49.230	10.520
120	alpha	1	60x8 weeks	PCR90	MWF	VAC50	25	Unvaccinate	2.177	0.007	0.016	0.023	0.004	0.018	0.002	1044.985	3.218	7.678	10.896	2.098	8.696	0.976	10450	32.180	76.780	108.960	20.980	86.960	9.760
120	alpha	1	60x8 weeks	PCR90	MWF	VAC50	25	Unvaccinate	2.177	0.007	0.016	0.023	0.004	0.018	0.002	1044.985	3.218	7.678	10.896	2.098	8.696	0.976	10450	32.180	76.780	108.960	20.980	86.960	9.760
120	alpha	1	60x8 weeks	PCR90	MWF	VAC50	25	Unvaccinate	2.177	0.007	0.016	0.023	0.004	0.018	0.002	1044.985	3.218	7.678	10.896	2.098	8.696	0.976	10450	32.180	76.780	108.960	20.980	86.960	9.760
120	alpha	1.2	60x8 weeks	PCR90	MWF	VAC50	25	Unvaccinate	2.148	0.006	0.025	0.032	0.006	0.025	0.002	1030.842	3.065	12.118	15.183	2.977	12.155	0.888	10308	30.650	121.180	151.830	29.770	121.550	8.880
120	alpha	1.2	60x8 weeks	PCR90	MWF	VAC50	25	Unvaccinate	2.148	0.006	0.025	0.032	0.006	0.025	0.002	1030.842	3.065	12.118	15.183	2.977	12.155	0.888	10308	30.650	121.180	151.830	29.770	121.550	8.880
120	alpha	1.2	60x8 weeks	PCR90	MWF	VAC50	25	Unvaccinate	2.148	0.006	0.025	0.032	0.006	0.025	0.002	1030.842	3.065	12.118	15.183	2.977	12.155	0.888	10308	30.650	121.180	151.830	29.770	121.550	8.880
120	delta	0.6	60x8 weeks	PCR90	MWF	VAC50	25	Unvaccinate	2.116	0.006	0.037	0.043	0.011	0.031	0.002	1015.467	3.102	17.758	20.860	5.515	14.946	0.940	10155	31.020	177.580	208.600	55.150	149.460	9.400
120	delta	0.6	60x8 weeks	PCR90	MWF	VAC50	25	Unvaccinate	2.116	0.006	0.037	0.043	0.011	0.031	0.002	1015.467	3.102	17.758	20.860	5.515	14.946	0.940	10155	31.020	177.580	208.600	55.150	149.460	9.400
120	delta	0.6	60x8 weeks	PCR90	MWF	VAC50	25	Unvaccinate	2.116	0.006	0.037	0.043	0.011	0.031	0.002	1015.467	3.102	17.758	20.860	5.515	14.946	0.940	10155	31.020	177.580	208.600	55.150	149.460	9.400
120	delta	1	60x8 weeks	PCR90	MWF	VAC50	25	Unvaccinate	1.946	0.004	0.092	0.097	0.028	0.068	0.001	934.164	2.107	44.385	46.492	13.421	32.406	0.614	9342	21.070	443.850	464.920	134.210	324.060	6.140
120	delta	1	60x8 weeks	PCR90	MWF	VAC50	25	Unvaccinate	1.946	0.004	0.092	0.097	0.028	0.068	0.001	934.164	2.107	44.385	46.492	13.421	32.406	0.614	9342	21.070	443.850	464.920	134.210	324.060	6.140
120	delta	1	60x8 weeks	PCR90	MWF	VAC50	25	Unvaccinate	1.946	0.004	0.092	0.097	0.028	0.068	0.001	934.164	2.107	44.385	46.492	13.421	32.406	0.614	9342	21.070	443.850	464.920	134.210	324.060	6.140
120	delta	1.2	60x8 weeks	PCR90	MWF	VAC50	25	Unvaccinate	1.904	0.004	0.105	0.109	0.032	0.076	0.001	913.928	1.979	50.571	52.550	15.347	36.605	0.536	9139	19.790	505.710	525.500	153.470	366.050	5.360
120	delta	1.2	60x8 weeks	PCR90	MWF	VAC50	25	Unvaccinate	1.904	0.004	0.105	0.109	0.032	0.076	0.001	913.928	1.979	50.571	52.550	15.347	36.605	0.536	9139	19.790	505.710	525.500	153.470	366.050	5.360
120	delta	1.2	60x8 weeks	PCR90	MWF	VAC50	25	Unvaccinate	1.904	0.004	0.105	0.109	0.032	0.076	0.001	913.928	1.979	50.571	52.550	15.347	36.605	0.536	9139	19.790	505.710	525.500	153.470	366.050	5.360
120	original	0.6	60x8 weeks	PCR90	MWF	VAC50	50	Unvaccinate	1.483	0.006	0.001	0.007	0.002	0.004	0.001	711.826	2.935	0.470	3.405	0.849	2.040	0.696	7118	29.350	4.700	34.050	8.490	20.400	6.960
120	original	0.6	60x8 weeks	PCR90	MWF	VAC50	50	Unvaccinate	1.483	0.006	0.001	0.007	0.002	0.004	0.001	711.826	2.935	0.470	3.405	0.849	2.040	0.696	7118	29.350	4.700	34.050	8.490	20.400	6.960
120	original	0.6	60x8 weeks	PCR90	MWF	VAC50	50	Unvaccinate	1.483	0.006	0.001	0.007	0.002	0.004	0.001	711.826	2.935	0.470	3.405	0.849	2.040	0.696	7118	29.350	4.700	34.050	8.490	20.400	6.960
120	original	1	60x8 weeks	PCR90	MWF	VAC50	50	Unvaccinate	1.480	0.006	0.002	0.008	0.002	0.005	0.001	710.277	3.038	0.940	3.978	1.026	2.400	0.682	7103	30.380	9.400	39.780	10.260	24.000	6.820
120	original	1	60x8 weeks	PCR90	MWF	VAC50	50	Unvaccinate	1.480	0.006	0.002	0.008	0.002	0.005	0.001	710.277	3.038	0.940	3.978	1.026	2.400	0.682	7103	30.380	9.400	39.780	10.260	24.000	6.820
120	original	1	60x8 weeks	PCR90	MWF	VAC50	50	Unvaccinate	1.480	0.006	0.002	0.008	0.002	0.005	0.001	710.277	3.038	0.940	3.978	1.026	2.400	0.682	7103	30.380	9.400	39.780	10.260	24.000	6.820
120	original	1.2	60x8 weeks	PCR90	MWF	VAC50	50	Unvaccinate	1.479	0.006	0.002	0.009	0.002	0.005	0.001	710.004	2.947	1.192	4.139	1.035	2.470	0.692	7100	29.470	11.920	41.390	10.350	24.700	6.920

120	original	1.2	60x8 weeks	PCR90	MWF	VAC50	50	Unvaccinate	1.479	0.006	0.002	0.009	0.002	0.005	0.001	710.004	2.947	1.192	4.139	1.035	2.470	0.692	7100	29.470	11.920	41.390	10.350	24.700	6.920
120	original	1.2	60x8 weeks	PCR90	MWF	VAC50	50	Unvaccinate	1.479	0.006	0.002	0.009	0.002	0.005	0.001	710.004	2.947	1.192	4.139	1.035	2.470	0.692	7100	29.470	11.920	41.390	10.350	24.700	6.920
120	alpha	0.6	60x8 weeks	PCR90	MWF	VAC50	50	Unvaccinate	1.475	0.006	0.004	0.010	0.003	0.006	0.001	708.072	2.952	1.961	4.913	1.450	2.943	0.614	7081	29.520	19.610	49.130	14.500	29.430	6.140
120	alpha	0.6	60x8 weeks	PCR90	MWF	VAC50	50	Unvaccinate	1.475	0.006	0.004	0.010	0.003	0.006	0.001	708.072	2.952	1.961	4.913	1.450	2.943	0.614	7081	29.520	19.610	49.130	14.500	29.430	6.140
120	alpha	0.6	60x8 weeks	PCR90	MWF	VAC50	50	Unvaccinate	1.475	0.006	0.004	0.010	0.003	0.006	0.001	708.072	2.952	1.961	4.913	1.450	2.943	0.614	7081	29.520	19.610	49.130	14.500	29.430	6.140
120	alpha	1	60x8 weeks	PCR90	MWF	VAC50	50	Unvaccinate	1.461	0.006	0.010	0.016	0.005	0.010	0.001	701.041	2.884	4.960	7.844	2.275	4.731	0.620	7010	28.840	49.600	78.440	22.750	47.310	6.200
120	alpha	1	60x8 weeks	PCR90	MWF	VAC50	50	Unvaccinate	1.461	0.006	0.010	0.016	0.005	0.010	0.001	701.041	2.884	4.960	7.844	2.275	4.731	0.620	7010	28.840	49.600	78.440	22.750	47.310	6.200
120	alpha	1	60x8 weeks	PCR90	MWF	VAC50	50	Unvaccinate	1.461	0.006	0.010	0.016	0.005	0.010	0.001	701.041	2.884	4.960	7.844	2.275	4.731	0.620	7010	28.840	49.600	78.440	22.750	47.310	6.200
120	alpha	1.2	60x8 weeks	PCR90	MWF	VAC50	50	Unvaccinate	1.450	0.006	0.015	0.021	0.006	0.012	0.001	695.840	2.837	7.210	10.047	2.989	5.974	0.639	6958	28.370	72.100	100.470	29.890	59.740	6.390
120	alpha	1.2	60x8 weeks	PCR90	MWF	VAC50	50	Unvaccinate	1.450	0.006	0.015	0.021	0.006	0.012	0.001	695.840	2.837	7.210	10.047	2.989	5.974	0.639	6958	28.370	72.100	100.470	29.890	59.740	6.390
120	alpha	1.2	60x8 weeks	PCR90	MWF	VAC50	50	Unvaccinate	1.450	0.006	0.015	0.021	0.006	0.012	0.001	695.840	2.837	7.210	10.047	2.989	5.974	0.639	6958	28.370	72.100	100.470	29.890	59.740	6.390
120	delta	0.6	60x8 weeks	PCR90	MWF	VAC50	50	Unvaccinate	1.431	0.006	0.025	0.031	0.012	0.016	0.001	687.022	2.955	12.064	15.019	5.557	7.617	0.629	6870	29.550	120.640	150.190	55.570	76.170	6.290
120	delta	0.6	60x8 weeks	PCR90	MWF	VAC50	50	Unvaccinate	1.431	0.006	0.025	0.031	0.012	0.016	0.001	687.022	2.955	12.064	15.019	5.557	7.617	0.629	6870	29.550	120.640	150.190	55.570	76.170	6.290
120	delta	0.6	60x8 weeks	PCR90	MWF	VAC50	50	Unvaccinate	1.431	0.006	0.025	0.031	0.012	0.016	0.001	687.022	2.955	12.064	15.019	5.557	7.617	0.629	6870	29.550	120.640	150.190	55.570	76.170	6.290
120	delta	1	60x8 weeks	PCR90	MWF	VAC50	50	Unvaccinate	1.325	0.005	0.078	0.083	0.033	0.040	0.001	636.090	2.217	37.465	39.682	15.767	18.965	0.469	6361	22.170	374.650	396.820	157.670	189.650	4.690
120	delta	1	60x8 weeks	PCR90	MWF	VAC50	50	Unvaccinate	1.325	0.005	0.078	0.083	0.033	0.040	0.001	636.090	2.217	37.465	39.682	15.767	18.965	0.469	6361	22.170	374.650	396.820	157.670	189.650	4.690
120	delta	1	60x8 weeks	PCR90	MWF	VAC50	50	Unvaccinate	1.325	0.005	0.078	0.083	0.033	0.040	0.001	636.090	2.217	37.465	39.682	15.767	18.965	0.469	6361	22.170	374.650	396.820	157.670	189.650	4.690
120	delta	1.2	60x8 weeks	PCR90	MWF	VAC50	50	Unvaccinate	1.286	0.004	0.097	0.101	0.042	0.047	0.001	617.084	1.992	46.396	48.388	19.992	22.768	0.418	6171	19.920	463.960	483.880	199.920	227.680	4.180
120	delta	1.2	60x8 weeks	PCR90	MWF	VAC50	50	Unvaccinate	1.286	0.004	0.097	0.101	0.042	0.047	0.001	617.084	1.992	46.396	48.388	19.992	22.768	0.418	6171	19.920	463.960	483.880	199.920	227.680	4.180
120	delta	1.2	60x8 weeks	PCR90	MWF	VAC50	50	Unvaccinate	1.286	0.004	0.097	0.101	0.042	0.047	0.001	617.084	1.992	46.396	48.388	19.992	22.768	0.418	6171	19.920	463.960	483.880	199.920	227.680	4.180
120	original	0.6	60x8 weeks	PCR90	MWF	VAC50	75	Unvaccinate	0.741	0.005	0.000	0.006	0.002	0.002	0.001	355.885	2.468	0.201	2.669	0.901	1.041	0.362	3559	24.680	2.010	26.690	9.010	10.410	3.620
120	original	0.6	60x8 weeks	PCR90	MWF	VAC50	75	Unvaccinate	0.741	0.005	0.000	0.006	0.002	0.002	0.001	355.885	2.468	0.201	2.669	0.901	1.041	0.362	3559	24.680	2.010	26.690	9.010	10.410	3.620
120	original	0.6	60x8 weeks	PCR90	MWF	VAC50	75	Unvaccinate	0.741	0.005	0.000	0.006	0.002	0.002	0.001	355.885	2.468	0.201	2.669	0.901	1.041	0.362	3559	24.680	2.010	26.690	9.010	10.410	3.620
120	original	1	60x8 weeks	PCR90	MWF	VAC50	75	Unvaccinate	0.742	0.005	0.001	0.006	0.002	0.002	0.001	356.082	2.433	0.331	2.764	0.993	0.995	0.366	3561	24.330	3.310	27.640	9.930	9.950	3.660
120	original	1	60x8 weeks	PCR90	MWF	VAC50	75	Unvaccinate	0.742	0.005	0.001	0.006	0.002	0.002	0.001	356.082	2.433	0.331	2.764	0.993	0.995	0.366	3561	24.330	3.310	27.640	9.930	9.950	3.660
120	original	1	60x8 weeks	PCR90	MWF	VAC50	75	Unvaccinate	0.742	0.005	0.001	0.006	0.002	0.002	0.001	356.082	2.433	0.331	2.764	0.993	0.995	0.366	3561	24.330	3.310	27.640	9.930	9.950	3.660
120	original	1.2	60x8 weeks	PCR90	MWF	VAC50	75	Unvaccinate	0.742	0.005	0.001	0.006	0.002	0.002	0.001	356.090	2.494	0.423	2.917	1.080	0.988	0.345	3561	24.940	4.230	29.170	10.800	9.880	3.450
120	original	1.2	60x8 weeks	PCR90	MWF	VAC50	75	Unvaccinate	0.742	0.005	0.001	0.006	0.002	0.002	0.001	356.090	2.494	0.423	2.917	1.080	0.988	0.345	3561	24.940	4.230	29.170	10.800	9.880	3.450
120	original	1.2	60x8 weeks	PCR90	MWF	VAC50	75	Unvaccinate	0.742	0.005	0.001	0.006	0.002	0.002	0.001	356.090	2.494	0.423	2.917	1.080	0.988	0.345	3561	24.940	4.230	29.170	10.800	9.880	3.450
120	alpha	0.6	60x8 weeks	PCR90	MWF	VAC50	75	Unvaccinate	0.739	0.005	0.002	0.008	0.003	0.003	0.001	354.797	2.486	1.181	3.667	1.540	1.309	0.356	3548	24.860	11.810	36.670	15.400	13.090	3.560
120	alpha	0.6	60x8 weeks	PCR90	MWF	VAC50	75	Unvaccinate	0.739	0.005	0.002	0.008	0.003	0.003	0.001	354.797	2.486	1.181	3.667	1.540	1.309	0.356	3548	24.860	11.810	36.670	15.400	13.090	3.560
120	alpha	0.6	60x8 weeks	PCR90	MWF	VAC50	75	Unvaccinate	0.739	0.005	0.002	0.008	0.003	0.003	0.001	354.797	2.486	1.181	3.667	1.540	1.309	0.356	3548	24.860	11.810	36.670	15.400	13.090	3.560
120	alpha	1	60x8 weeks	PCR90	MWF	VAC50	75	Unvaccinate	0.735	0.005	0.006	0.011	0.005	0.004	0.001	352.675	2.435	2.804	5.239	2.261	1.798	0.337	3527	24.350	28.040	52.390	22.610	17.980	3.370
120	alpha	1	60x8 weeks	PCR90	MWF	VAC50	75	Unvaccinate	0.735	0.005	0.006	0.011	0.005	0.004	0.001	352.675	2.435	2.804	5.239	2.261	1.798	0.337	3527	24.350	28.040	52.390	22.610	17.980	3.370
120	alpha	1	60x8 weeks	PCR90	MWF	VAC50	75	Unvaccinate	0.735	0.005	0.006	0.011	0.005	0.004	0.001	352.675	2.435	2.804	5.239	2.261	1.798	0.337	3527	24.350	28.040	52.390	22.610	17.980	3.370
120	alpha	1.2	60x8 weeks	PCR90	MWF	VAC50	75	Unvaccinate	0.731	0.005	0.008	0.013	0.006	0.005	0.001	350.838	2.363	4.010	6.373	2.688	2.260	0.349	3508	23.630	40.100	63.730	26.880	22.600	3.490
120	alpha	1.2	60x8 weeks	PCR90	MWF	VAC50	75	Unvaccinate	0.731	0.005	0.008	0.013	0.006	0.005	0.001	350.838	2.363	4.010	6.373	2.688	2.260	0.349	3508	23.630	40.100	63.730	26.880	22.600	3.490
120	alpha	1.2	60x8 weeks	PCR90	MWF	VAC50	75	Unvaccinate	0.731	0.005	0.008	0.013	0.006	0.005	0.001	350.838	2.363	4.010	6.373	2.688	2.260	0.349	3508	23.630	40.100	63.730	26.880	22.600	3.490
120	delta	0.6	60x8 weeks	PCR90	MWF	VAC50	75	Unvaccinate	0.722	0.006	0.017	0.023	0.011	0.006	0.001	346.754	2.717	8.377	11.094	5.391	3.077	0.325	3468	27.170	83.770	110.940	53.910	30.770	3.250
120	delta	0.6	60x8 weeks	PCR90	MWF	VAC50	75	Unvaccinate	0.722	0.006	0.017	0.023	0.011	0.006	0.001	346.754	2.717	8.377	11.094	5.391	3.077	0.325	3468	27.170	83.770	110.940	53.910	30.770	3.250
120	delta	0.6	60x8 weeks	PCR90	MWF	VAC50	75	Unvaccinate	0.722	0.006	0.017	0.023	0.011	0.006	0.001	346.754	2.717	8.377	11.094	5.391	3.077	0.325	3468	27.170	83.770	110.940	53.910	30.770	3.250
120	delta	1	60x8 weeks	PCR90	MWF	VAC50	75	Unvaccinate	0.680	0.005	0.059	0.063	0.032	0.016	0.001	326.298	2.276	28.103	30.379	15.424	7.712	0.240	3263	22.760	281.030	303.790	154.240	77.120	2.400
120	delta	1	60x8 weeks	PCR90	MWF	VAC50	75	Unvaccinate	0.680	0.005	0.059	0.063	0.032	0.016	0.001	326.298	2.276	28.103	30.379	15.424	7.712	0.240	3263	22.760	281.030	303.790	154.240	77.120	2.400
120	delta	1	60x8 weeks	PCR90	MWF	VAC50	75	Unvaccinate	0.680	0.005	0.059	0.063	0.032	0.016	0.001	326.298	2.276	28.103	30.379	15.424	7.712	0.240	3263	22.760	281.030	303.790	154.240	77.120	2.400
120	delta	1.2	60x8 weeks	PCR90	MWF	VAC50	75	Unvaccinate	0.657	0.004	0.080	0.084	0.045	0.021	0.000	315.579	2.000	38.362	40.362	21.402	9.886	0.199	3156	20.000	383.620	403.620	214.020	98.860	1.990
120	delta	1.2	60x8 weeks	PCR90	MWF	VAC50	75	Unvaccinate	0.657	0.004	0.080	0.084	0.045	0.021	0.000	315.579	2.000	38.362	40.362	21.402	9.886	0.199	3156	20.000	383.620	403.620	214.020	98.860	1.990
120	delta	1.2	60x8 weeks	PCR90	MWF	VAC50	75	Unvaccinate	0.657	0.004	0.080	0.084	0.045	0.021	0.000	315.579	2.000	38.362	40.362	21.402	9.886	0.199	3156	20.000	383.620	403.620	214.020	98.860	1.990
120	original	0.6	60x8 weeks	PCR90	MWF	VAC50	100	Unvaccinate	0.000	0.004	0.000	0.004	0.002	0.000	0.000	0.000	2.119	0.024	2.143	1.100	0.000	0.000	0	21.190	0.240	21.430	11.000	0.000	0.000
120	original	0.6	60x8 weeks	PCR90	MWF	VAC50	100	Unvaccinate	0.000	0.004	0.000	0.004	0.002	0.000	0.000	0.000	2.119	0.024	2.143	1.100	0.000	0.000	0	21.190	0.240	21.430	11.000	0.000	0.000
120	original	0.6	60x8 weeks	PCR90	MWF	VAC50	100	Unvaccinate	0.000	0.004	0.000	0.004	0.002	0.000	0.000	0.000	2.119	0.024	2.143	1.100	0.000	0.000	0	21.190	0.240	21.430	11.000	0.000	0.000
120	original	1	60x8 weeks	PCR90	MWF	VAC50	100	Unvaccinate	0.000	0.004	0.000	0.004	0.002	0.000	0.000	0.000	1.973	0.043	2.016	1.053	0.000	0.000	0	19.730	0.430	20.160	10.530	0.000	0.000
120	original	1	60x8 weeks	PCR90	MWF	VAC50	100	Unvaccinate	0.000	0.004	0.000	0.004	0.002	0.000	0.000	0.000	1.973	0.043	2.016	1.053	0.000	0.000	0	19.730	0.430	20.160	10.530	0.000	0.000
120	original	1	60x8 weeks	PCR90	MWF	VAC50	100	Unvaccinate	0.000	0.004	0.000	0.004	0.002	0.000	0.000	0.000	1.973	0.043	2.016	1.053	0.000	0.000	0	19.730	0.430	20.160	10.530	0.000	0.000
120	original	1.2	60x8 weeks	PCR90	MWF	VAC50	100	Unvaccinate	0.000	0.004	0.000	0.004	0.002	0.000	0.000	0.000	2.056	0.056	2.112	1.117	0.000	0.000	0	20.560	0.560	21.120	11.170	0.000	0.000
120	original	1.2	60x8 weeks	PCR90	MWF	VAC50	100	Unvaccinate	0.000	0.004	0.000	0.004	0.002	0.000	0.000	0.000	2.056	0.056	2.112	1.117	0.000	0.000	0	20.560	0.560	21.120	11.170	0.000	0.000
120	original	1.2	60x8 weeks	PCR90	MWF	VAC50	100	Unvaccinate	0.000	0.004	0.000	0.004	0.002	0.000	0.000	0.000	2.056	0.056	2.112	1.117	0.000	0.000	0	20.560	0.560	21.120	11.170	0.000	0.000
120	alpha	0.6	60x8 weeks	PCR90	MWF	VAC50	100	Unvaccinate	0.000	0.004	0.002	0.006	0.003	0.000	0.000	0.000	1.974	0.734	2.708	1.635	0.000	0.000	0	19.740	7.340	27.080	16.350	0.000	0.000
120	alpha	0.6	60x8 weeks	PCR90	MWF	VAC50	100	Unvaccinate	0.000	0.004	0.002	0.006	0.003	0.000	0.000	0.000	1.974	0.734	2.708	1.635	0.000	0.000	0	19.740	7.340	27.080	16.350	0.000	0.000
120	alpha	0.6	60x8 weeks	PCR90	MWF	VAC50	100	Unvaccinate	0.000	0.004	0.002	0.006	0.003	0.000	0.000	0.000	1.974	0.734	2.708	1.635	0.000	0.000	0	19.740	7.340	27.080	16.350	0.000	0.000
120	alpha	1	60x8 weeks	PCR90	MWF	VAC50	100	Unvaccinate	0.000	0.004	0.003	0.007	0.004	0.000	0.000	0.000	1.963	1.375	3.338	2.034	0.000	0.000	0	19.630	13.750	33.380	20.340	0.000	0.000
120	alpha	1	60x8 weeks	PCR90	MWF	VAC50	100	Unvaccinate	0.000	0.004	0.003	0.007	0.004	0.000	0.000	0.000	1.963	1.375	3.338	2.034	0.000	0.000	0	19.630	13.750	33.380	20.340	0.000	0.000
120	alpha	1	60x8 weeks	PCR90	MWF	VAC50	100	Unvaccinate	0.000	0.004	0.003	0.007	0.004	0.000	0.000	0.000	1.963	1.375	3.338	2.034	0.000	0.000	0	19.630	13.750	33.380	20.340	0.000	0.000
120	alpha	1.2	60x8 weeks	PCR90	MWF	VAC50	100	Unvaccinate	0.000	0.004	0.004	0.008	0.005	0.000	0.000	0.000	1.930	1.999	3.929	2.374	0.000	0.000	0	19.300	19.990	39.290	23.740	0.000	0.000
120	alpha	1.2	60x8 weeks	PCR90	MWF	VAC50	100	Unvaccinate	0.000	0.004	0.004	0.008	0.005	0.000	0.000	0.000	1.930	1.999	3.929	2.374	0.000	0.000	0	19.300	19.990	39.290	23.740	0.000	0.000
120	alpha	1.2	60x8 weeks	PCR90	MWF	VAC50	100	Unvaccinate	0.000	0.004	0.004	0.008	0.005	0.000	0.000	0.000	1.930	1.999	3.929	2.374	0.000	0.000	0	19.300	19.990	39.290	23.740	0.000	0.000
120	delta	0.6	60x8 weeks	PCR90	MWF	VAC50	100	Unvaccinate	0.000	0.005	0.011	0.016	0.010	0.000	0.000	0.000	2.530	5.093	7.623	4.895	0.000	0.000	0	25.300	50.930	76.230	48.950	0.000	0.000
120	delta	0.6	60x8 weeks	PCR90	MWF	VAC50	100	Unvaccinate	0.000	0.005	0.011	0.016	0.010	0.000	0.000	0.000	2.530	5.093	7.623	4.895	0.000	0.000	0	25.300	50.930	76.230	48.950	0.000	0.000
120	delta	0.6	60x8 weeks	PCR90	MWF	VAC50	100	Unvaccinate	0.000	0.005	0.011	0.016	0.010	0.000	0.000	0.000	2.530	5.093	7.623	4.895	0.000	0.000	0	25.300	50.930	76.230	48.950	0.000	0.000
120	delta	1	60x8 weeks	PCR90	MWF	VAC50	100	Unvaccinate	0.000	0.005	0.043	0.048	0.030	0.000	0.000	0.000	2.383	20.465	22.848	14.515	0.000	0.000	0	23.830	204.650	228.480	145.150	0.000	0.000
120	delta	1	60x8 weeks	PCR90	MWF	VAC50	100	Unvaccinate	0.000	0.005	0.043	0.048	0.030	0.000	0.000	0.000	2.383	20.465	22.848	14.515	0.000	0.000	0	23.830	204.650	228.480	145.150	0.000	0.000
120	delta	1	60x8 weeks	PCR90	MWF	VAC50	100	Unvaccinate	0.000	0.005	0.043	0.048	0.030	0.000	0.000	0.000	2.383	20.465	22.848	14.515	0.000	0.000	0	23.830	204.650	228.480	145.150	0.000	0.000

120	delta	1.2	60x8 weeks	PCR90	MWF	VAC50	100	Unvaccinate	0.000	0.004	0.062	0.066	0.043	0.000	0.000	0.000	2.096	29.561	31.657	20.471	0.000	0.000	0	20.960	295.610	316.570	204.710	0.000	0.000
120	delta	1.2	60x8 weeks	PCR90	MWF	VAC50	100	Unvaccinate	0.000	0.004	0.062	0.066	0.043	0.000	0.000	0.000	2.096	29.561	31.657	20.471	0.000	0.000	0	20.960	295.610	316.570	204.710	0.000	0.000
120	delta	1.2	60x8 weeks	PCR90	MWF	VAC50	100	Unvaccinate	0.000	0.004	0.062	0.066	0.043	0.000	0.000	0.000	2.096	29.561	31.657	20.471	0.000	0.000	0	20.960	295.610	316.570	204.710	0.000	0.000
120	original	0.6	60x8 weeks	PCR90	M	NOVAC	0	Unvaccinate	0.989	0.008	0.005	0.013	0.004	0.006	0.001	474.593	3.822	2.486	6.308	2.115	2.927	0.453	4746	38.220	24.860	63.080	21.150	29.270	4.530
120	original	0.6	60x8 weeks	PCR90	M	NOVAC	0	Unvaccinate	0.989	0.008	0.005	0.013	0.004	0.006	0.001	474.593	3.822	2.486	6.308	2.115	2.927	0.453	4746	38.220	24.860	63.080	21.150	29.270	4.530
120	original	0.6	60x8 weeks	PCR90	M	NOVAC	0	Unvaccinate	0.989	0.008	0.005	0.013	0.004	0.006	0.001	474.593	3.822	2.486	6.308	2.115	2.927	0.453	4746	38.220	24.860	63.080	21.150	29.270	4.530
120	original	1	60x8 weeks	PCR90	M	NOVAC	0	Unvaccinate	0.982	0.008	0.013	0.021	0.007	0.010	0.001	471.462	3.772	6.227	9.999	3.322	4.626	0.432	4715	37.720	62.270	99.990	33.220	46.260	4.320
120	original	1	60x8 weeks	PCR90	M	NOVAC	0	Unvaccinate	0.982	0.008	0.013	0.021	0.007	0.010	0.001	471.462	3.772	6.227	9.999	3.322	4.626	0.432	4715	37.720	62.270	99.990	33.220	46.260	4.320
120	original	1	60x8 weeks	PCR90	M	NOVAC	0	Unvaccinate	0.982	0.008	0.013	0.021	0.007	0.010	0.001	471.462	3.772	6.227	9.999	3.322	4.626	0.432	4715	37.720	62.270	99.990	33.220	46.260	4.320
120	original	1.2	60x8 weeks	PCR90	M	NOVAC	0	Unvaccinate	0.977	0.007	0.019	0.026	0.009	0.012	0.001	469.086	3.547	9.115	12.662	4.184	5.985	0.438	4691	35.470	91.150	126.620	41.840	59.850	4.380
120	original	1.2	60x8 weeks	PCR90	M	NOVAC	0	Unvaccinate	0.977	0.007	0.019	0.026	0.009	0.012	0.001	469.086	3.547	9.115	12.662	4.184	5.985	0.438	4691	35.470	91.150	126.620	41.840	59.850	4.380
120	original	1.2	60x8 weeks	PCR90	M	NOVAC	0	Unvaccinate	0.977	0.007	0.019	0.026	0.009	0.012	0.001	469.086	3.547	9.115	12.662	4.184	5.985	0.438	4691	35.470	91.150	126.620	41.840	59.850	4.380
120	alpha	0.6	60x8 weeks	PCR90	M	NOVAC	0	Unvaccinate	0.974	0.007	0.020	0.028	0.011	0.013	0.001	467.643	3.568	9.647	13.215	5.073	6.290	0.458	4676	35.680	96.470	132.150	50.730	62.900	4.580
120	alpha	0.6	60x8 weeks	PCR90	M	NOVAC	0	Unvaccinate	0.974	0.007	0.020	0.028	0.011	0.013	0.001	467.643	3.568	9.647	13.215	5.073	6.290	0.458	4676	35.680	96.470	132.150	50.730	62.900	4.580
120	alpha	0.6	60x8 weeks	PCR90	M	NOVAC	0	Unvaccinate	0.974	0.007	0.020	0.028	0.011	0.013	0.001	467.643	3.568	9.647	13.215	5.073	6.290	0.458	4676	35.680	96.470	132.150	50.730	62.900	4.580
120	alpha	1	60x8 weeks	PCR90	M	NOVAC	0	Unvaccinate	0.926	0.006	0.068	0.074	0.029	0.037	0.001	444.243	2.861	32.561	35.422	14.141	17.682	0.327	4442	28.610	325.610	354.220	141.410	176.820	3.270
120	alpha	1	60x8 weeks	PCR90	M	NOVAC	0	Unvaccinate	0.926	0.006	0.068	0.074	0.029	0.037	0.001	444.243	2.861	32.561	35.422	14.141	17.682	0.327	4442	28.610	325.610	354.220	141.410	176.820	3.270
120	alpha	1	60x8 weeks	PCR90	M	NOVAC	0	Unvaccinate	0.926	0.006	0.068	0.074	0.029	0.037	0.001	444.243	2.861	32.561	35.422	14.141	17.682	0.327	4442	28.610	325.610	354.220	141.410	176.820	3.270
120	alpha	1.2	60x8 weeks	PCR90	M	NOVAC	0	Unvaccinate	0.901	0.005	0.089	0.095	0.038	0.048	0.001	432.392	2.519	42.893	45.412	18.306	23.082	0.297	4324	25.190	428.930	454.120	183.060	230.820	2.970
120	alpha	1.2	60x8 weeks	PCR90	M	NOVAC	0	Unvaccinate	0.901	0.005	0.089	0.095	0.038	0.048	0.001	432.392	2.519	42.893	45.412	18.306	23.082	0.297	4324	25.190	428.930	454.120	183.060	230.820	2.970
120	alpha	1.2	60x8 weeks	PCR90	M	NOVAC	0	Unvaccinate	0.901	0.005	0.089	0.095	0.038	0.048	0.001	432.392	2.519	42.893	45.412	18.306	23.082	0.297	4324	25.190	428.930	454.120	183.060	230.820	2.970
120	delta	0.6	60x8 weeks	PCR90	M	NOVAC	0	Unvaccinate	0.891	0.005	0.095	0.100	0.044	0.048	0.001	427.627	2.329	45.659	47.988	21.343	23.054	0.282	4276	23.290	456.590	479.880	213.430	230.540	2.820
120	delta	0.6	60x8 weeks	PCR90	M	NOVAC	0	Unvaccinate	0.891	0.005	0.095	0.100	0.044	0.048	0.001	427.627	2.329	45.659	47.988	21.343	23.054	0.282	4276	23.290	456.590	479.880	213.430	230.540	2.820
120	delta	0.6	60x8 weeks	PCR90	M	NOVAC	0	Unvaccinate	0.891	0.005	0.095	0.100	0.044	0.048	0.001	427.627	2.329	45.659	47.988	21.343	23.054	0.282	4276	23.290	456.590	479.880	213.430	230.540	2.820
120	delta	1	60x8 weeks	PCR90	M	NOVAC	0	Unvaccinate	0.862	0.004	0.115	0.119	0.054	0.059	0.000	413.723	1.723	55.348	57.071	26.058	28.286	0.199	4137	17.230	553.480	570.710	260.580	282.860	1.990
120	delta	1	60x8 weeks	PCR90	M	NOVAC	0	Unvaccinate	0.862	0.004	0.115	0.119	0.054	0.059	0.000	413.723	1.723	55.348	57.071	26.058	28.286	0.199	4137	17.230	553.480	570.710	260.580	282.860	1.990
120	delta	1	60x8 weeks	PCR90	M	NOVAC	0	Unvaccinate	0.862	0.004	0.115	0.119	0.054	0.059	0.000	413.723	1.723	55.348	57.071	26.058	28.286	0.199	4137	17.230	553.480	570.710	260.580	282.860	1.990
120	delta	1.2	60x8 weeks	PCR90	M	NOVAC	0	Unvaccinate	0.862	0.003	0.116	0.119	0.054	0.059	0.000	413.665	1.595	55.559	57.154	25.990	28.227	0.217	4137	15.950	555.590	571.540	259.900	282.270	2.170
120	delta	1.2	60x8 weeks	PCR90	M	NOVAC	0	Unvaccinate	0.862	0.003	0.116	0.119	0.054	0.059	0.000	413.665	1.595	55.559	57.154	25.990	28.227	0.217	4137	15.950	555.590	571.540	259.900	282.270	2.170
120	delta	1.2	60x8 weeks	PCR90	M	NOVAC	0	Unvaccinate	0.862	0.003	0.116	0.119	0.054	0.059	0.000	413.665	1.595	55.559	57.154	25.990	28.227	0.217	4137	15.950	555.590	571.540	259.900	282.270	2.170
120	original	0.6	60x8 weeks	PCR90	M	VAC95	25	Unvaccinate	0.743	0.006	0.002	0.009	0.003	0.004	0.001	356.464	2.959	1.197	4.156	1.360	1.921	0.380	3565	29.590	11.970	41.560	13.600	19.210	3.800
120	original	0.6	60x8 weeks	PCR90	M	VAC95	25	Unvaccinate	0.743	0.006	0.002	0.009	0.003	0.004	0.001	356.464	2.959	1.197	4.156	1.360	1.921	0.380	3565	29.590	11.970	41.560	13.600	19.210	3.800
120	original	0.6	60x8 weeks	PCR90	M	VAC95	25	Unvaccinate	0.743	0.006	0.002	0.009	0.003	0.004	0.001	356.464	2.959	1.197	4.156	1.360	1.921	0.380	3565	29.590	11.970	41.560	13.600	19.210	3.800
120	original	1	60x8 weeks	PCR90	M	VAC95	25	Unvaccinate	0.741	0.006	0.005	0.011	0.004	0.005	0.001	355.455	2.870	2.501	5.371	1.818	2.448	0.346	3555	28.700	25.010	53.710	18.180	24.480	3.460
120	original	1	60x8 weeks	PCR90	M	VAC95	25	Unvaccinate	0.741	0.006	0.005	0.011	0.004	0.005	0.001	355.455	2.870	2.501	5.371	1.818	2.448	0.346	3555	28.700	25.010	53.710	18.180	24.480	3.460
120	original	1	60x8 weeks	PCR90	M	VAC95	25	Unvaccinate	0.741	0.006	0.005	0.011	0.004	0.005	0.001	355.455	2.870	2.501	5.371	1.818	2.448	0.346	3555	28.700	25.010	53.710	18.180	24.480	3.460
120	original	1.2	60x8 weeks	PCR90	M	VAC95	25	Unvaccinate	0.738	0.006	0.008	0.014	0.005	0.006	0.001	354.430	2.993	3.794	6.787	2.227	3.059	0.335	3544	29.930	37.940	67.870	22.270	30.590	3.350
120	original	1.2	60x8 weeks	PCR90	M	VAC95	25	Unvaccinate	0.738	0.006	0.008	0.014	0.005	0.006	0.001	354.430	2.993	3.794	6.787	2.227	3.059	0.335	3544	29.930	37.940	67.870	22.270	30.590	3.350
120	original	1.2	60x8 weeks	PCR90	M	VAC95	25	Unvaccinate	0.738	0.006	0.008	0.014	0.005	0.006	0.001	354.430	2.993	3.794	6.787	2.227	3.059	0.335	3544	29.930	37.940	67.870	22.270	30.590	3.350
120	alpha	0.6	60x8 weeks	PCR90	M	VAC95	25	Unvaccinate	0.737	0.006	0.008	0.014	0.006	0.007	0.001	353.644	2.908	4.022	6.930	2.691	3.196	0.323	3536	29.080	40.220	69.300	26.910	31.960	3.230
120	alpha	0.6	60x8 weeks	PCR90	M	VAC95	25	Unvaccinate	0.737	0.006	0.008	0.014	0.006	0.007	0.001	353.644	2.908	4.022	6.930	2.691	3.196	0.323	3536	29.080	40.220	69.300	26.910	31.960	3.230
120	alpha	0.6	60x8 weeks	PCR90	M	VAC95	25	Unvaccinate	0.737	0.006	0.008	0.014	0.006	0.007	0.001	353.644	2.908	4.022	6.930	2.691	3.196	0.323	3536	29.080	40.220	69.300	26.910	31.960	3.230
120	alpha	1	60x8 weeks	PCR90	M	VAC95	25	Unvaccinate	0.721	0.006	0.026	0.032	0.012	0.015	0.001	346.072	2.772	12.429	15.201	5.913	7.132	0.308	3461	27.720	124.290	152.010	59.130	71.320	3.080
120	alpha	1	60x8 weeks	PCR90	M	VAC95	25	Unvaccinate	0.721	0.006	0.026	0.032	0.012	0.015	0.001	346.072	2.772	12.429	15.201	5.913	7.132	0.308	3461	27.720	124.290	152.010	59.130	71.320	3.080
120	alpha	1	60x8 weeks	PCR90	M	VAC95	25	Unvaccinate	0.721	0.006	0.026	0.032	0.012	0.015	0.001	346.072	2.772	12.429	15.201	5.913	7.132	0.308	3461	27.720	124.290	152.010	59.130	71.320	3.080
120	alpha	1.2	60x8 weeks	PCR90	M	VAC95	25	Unvaccinate	0.709	0.005	0.039	0.044	0.017	0.021	0.001	340.300	2.470	18.488	20.958	8.288	9.995	0.262	3403	24.700	184.880	209.580	82.880	99.950	2.620
120	alpha	1.2	60x8 weeks	PCR90	M	VAC95	25	Unvaccinate	0.709	0.005	0.039	0.044	0.017	0.021	0.001	340.300	2.470	18.488	20.958	8.288	9.995	0.262	3403	24.700	184.880	209.580	82.880	99.950	2.620
120	alpha	1.2	60x8 weeks	PCR90	M	VAC95	25	Unvaccinate	0.709	0.005	0.039	0.044	0.017	0.021	0.001	340.300	2.470	18.488	20.958	8.288	9.995	0.262	3403	24.700	184.880	209.580	82.880	99.950	2.620
120	delta	0.6	60x8 weeks	PCR90	M	VAC95	25	Unvaccinate	0.701	0.005	0.048	0.053	0.024	0.023	0.001	336.262	2.394	22.937	25.331	11.302	10.801	0.279	3363	23.940	229.370	253.310	113.020	108.010	2.790
120	delta	0.6	60x8 weeks	PCR90	M	VAC95	25	Unvaccinate	0.701	0.005	0.048	0.053	0.024	0.023	0.001	336.262	2.394	22.937	25.331	11.302	10.801	0.279	3363	23.940	229.370	253.310	113.020	108.010	2.790
120	delta	0.6	60x8 weeks	PCR90	M	VAC95	25	Unvaccinate	0.701	0.005	0.048	0.053	0.024	0.023	0.001	336.262	2.394	22.937	25.331	11.302	10.801	0.279	3363	23.940	229.370	253.310	113.020	108.010	2.790
120	delta	1	60x8 weeks	PCR90	M	VAC95	25	Unvaccinate	0.662	0.004	0.087	0.091	0.042	0.039	0.000	317.780	1.712	41.843	43.555	20.050	18.556	0.214	3178	17.120	418.430	435.550	200.500	185.560	2.140
120	delta	1	60x8 weeks	PCR90	M	VAC95	25	Unvaccinate	0.662	0.004	0.087	0.091	0.042	0.039	0.000	317.780	1.712	41.843	43.555	20.050	18.556	0.214	3178	17.120	418.430	435.550	200.500	185.560	2.140
120	delta	1	60x8 weeks	PCR90	M	VAC95	25	Unvaccinate	0.662	0.004	0.087	0.091	0.042	0.039	0.000	317.780	1.712	41.843	43.555	20.050	18.556	0.214	3178	17.120	418.430	435.550	200.500	185.560	2.140
120	delta	1.2	60x8 weeks	PCR90	M	VAC95	25	Unvaccinate	0.659	0.003	0.092	0.095	0.044	0.040	0.000	316.393	1.588	44.082	45.670	21.253	19.123	0.183	3164	15.880	440.820	456.700	212.530	191.230	1.830
120	delta	1.2	60x8 weeks	PCR90	M	VAC95	25	Unvaccinate	0.659	0.003	0.092	0.095	0.044	0.040	0.000	316.393	1.588	44.082	45.670	21.253	19.123	0.183	3164	15.880	440.820	456.700	212.530	191.230	1.830
120	delta	1.2	60x8 weeks	PCR90	M	VAC95	25	Unvaccinate	0.659	0.003	0.092	0.095	0.044	0.040	0.000	316.393	1.588	44.082	45.670	21.253	19.123	0.183	3164	15.880	440.820	456.700	212.530	191.230	1.830
120	original	0.6	60x8 weeks	PCR90	M	VAC95	50	Unvaccinate	0.496	0.004	0.001	0.005	0.002	0.002	0.000	238.066	2.051	0.463	2.514	0.806	1.069	0.217	2381	20.510	4.630	25.140	8.060	10.690	2.170
120	original	0.6	60x8 weeks	PCR90	M	VAC95	50	Unvaccinate	0.496	0.004	0.001	0.005	0.002	0.002	0.000	238.066	2.051	0.463	2.514	0.806	1.069	0.217	2381	20.510	4.630	25.140	8.060	10.690	2.170
120	original	0.6	60x8 weeks	PCR90	M	VAC95	50	Unvaccinate	0.496	0.004	0.001	0.005	0.002	0.002	0.000	238.066	2.051	0.463	2.514	0.806	1.069	0.217	2381	20.510	4.630	25.140	8.060	10.690	2.170
120	original	1	60x8 weeks	PCR90	M	VAC95	50	Unvaccinate	0.495	0.004	0.002	0.006	0.002	0.003	0.000	237.646	2.047	0.916	2.963	0.976	1.284	0.207	2376	20.470	9.160	29.630	9.760	12.840	2.070
120	original	1	60x8 weeks	PCR90	M	VAC95	50	Unvaccinate	0.495	0.004	0.002	0.006	0.002	0.003	0.000	237.646	2.047	0.916	2.963	0.976	1.284	0.207	2376	20.470	9.160	29.630	9.760	12.840	2.070
120	original	1	60x8 weeks	PCR90	M	VAC95	50	Unvaccinate	0.495	0.004	0.002	0.006	0.002	0.003	0.000	237.646	2.047	0.916	2.963	0.976	1.284	0.207	2376	20.470	9.160	29.630	9.760	12.840	2.070
120	original	1.2	60x8 weeks	PCR90	M	VAC95	50	Unvaccinate	0.495	0.004	0.003	0.007	0.002	0.003	0.000	237.366	2.081	1.280	3.361	1.121	1.418	0.229	2374	20.810	12.800	33.610	11.210	14.180	2.290
120	original	1.2	60x8 weeks	PCR90	M	VAC95	50	Unvaccinate	0.495	0.004	0.003	0.007	0.002	0.003	0.000	237.366	2.081	1.280	3.361	1.121	1.418	0.229	2374	20.810	12.800	33.610	11.210	14.180	2.290
120	original	1.2	60x8 weeks	PCR90	M	VAC95	50	Unvaccinate	0.495	0.004	0.003	0.007	0.002	0.003	0.000	237.366	2.081	1.280	3.361	1.121	1.418	0.229	2374	20.810	12.800	33.610	11.210	14.180	2.290
120	alpha	0.6	60x8 weeks	PCR90	M	VAC95	50	Unvaccinate	0.493	0.005	0.003	0.008	0.003	0.003	0.000	236.788	2.180	1.460	3.640	1.436	1.588	0.233	2368	21.800	14.600	36.400	14.360	15.880	2.330
120	alpha	0.6	60x8 weeks	PCR90	M	VAC95	50	Unvaccinate	0.493	0.005	0.003	0.008	0.003	0.003	0.000	236.788	2.180	1.460	3.640	1.436	1.588	0.233	2368	21.800	14.600	36.400	14.360	15.880	2.330
120	alpha	0.6	60x8 weeks	PCR90	M	VAC95	50	Unvaccinate	0.493	0.005	0.003	0.008	0.003	0.003	0.000	236.788	2.180	1.460	3.640	1.436	1.588	0.233	2368	21.800	14.600	36.400	14.360	15.880	2.330
120	alpha	1	60x8 weeks	PCR90	M	VAC95	50	Unvaccinate	0.490	0.004	0.008	0.012	0.005	0.005	0.000	235.030	2.031	3.723	5.754	2.336	2.438	0.226	2350	20.310	37.230	57.540	23.360	24.380	2.260
120	alpha	1	60x8 weeks	PCR90	M	VAC95	50	Unvaccinate	0.490	0.004	0.008	0.012	0.005	0.005	0.000	235.030	2.031	3.723	5.754	2.336	2.438	0.226	2350	20.310	37.230	57.540	23.360	24.380	2.260

120	alpha	1	60x8 weeks	PCR90	M	VAC95	50	Unvaccinate	0.490	0.004	0.008	0.012	0.005	0.005	0.000	235.030	2.031	3.723	5.754	2.336	2.438	0.226	2350	20.310	37.230	57.540	23.360	24.380	2.260
120	alpha	1.2	60x8 weeks	PCR90	M	VAC95	50	Unvaccinate	0.487	0.004	0.011	0.015	0.006	0.007	0.000	233.735	2.054	5.292	7.346	2.886	3.262	0.223	2337	20.540	52.920	73.460	28.860	32.620	2.230
120	alpha	1.2	60x8 weeks	PCR90	M	VAC95	50	Unvaccinate	0.487	0.004	0.011	0.015	0.006	0.007	0.000	233.735	2.054	5.292	7.346	2.886	3.262	0.223	2337	20.540	52.920	73.460	28.860	32.620	2.230
120	alpha	1.2	60x8 weeks	PCR90	M	VAC95	50	Unvaccinate	0.487	0.004	0.011	0.015	0.006	0.007	0.000	233.735	2.054	5.292	7.346	2.886	3.262	0.223	2337	20.540	52.920	73.460	28.860	32.620	2.230
120	delta	0.6	60x8 weeks	PCR90	M	VAC95	50	Unvaccinate	0.485	0.004	0.014	0.018	0.008	0.007	0.000	232.774	2.016	6.736	8.752	3.915	3.363	0.211	2328	20.160	67.360	87.520	39.150	33.630	2.110
120	delta	0.6	60x8 weeks	PCR90	M	VAC95	50	Unvaccinate	0.485	0.004	0.014	0.018	0.008	0.007	0.000	232.774	2.016	6.736	8.752	3.915	3.363	0.211	2328	20.160	67.360	87.520	39.150	33.630	2.110
120	delta	0.6	60x8 weeks	PCR90	M	VAC95	50	Unvaccinate	0.485	0.004	0.014	0.018	0.008	0.007	0.000	232.774	2.016	6.736	8.752	3.915	3.363	0.211	2328	20.160	67.360	87.520	39.150	33.630	2.110
120	delta	1	60x8 weeks	PCR90	M	VAC95	50	Unvaccinate	0.466	0.004	0.040	0.044	0.020	0.016	0.000	223.619	1.724	19.421	21.145	9.709	7.581	0.188	2236	17.240	194.210	211.450	97.090	75.810	1.880
120	delta	1	60x8 weeks	PCR90	M	VAC95	50	Unvaccinate	0.466	0.004	0.040	0.044	0.020	0.016	0.000	223.619	1.724	19.421	21.145	9.709	7.581	0.188	2236	17.240	194.210	211.450	97.090	75.810	1.880
120	delta	1	60x8 weeks	PCR90	M	VAC95	50	Unvaccinate	0.466	0.004	0.040	0.044	0.020	0.016	0.000	223.619	1.724	19.421	21.145	9.709	7.581	0.188	2236	17.240	194.210	211.450	97.090	75.810	1.880
120	delta	1.2	60x8 weeks	PCR90	M	VAC95	50	Unvaccinate	0.455	0.003	0.055	0.058	0.027	0.020	0.000	218.532	1.591	26.387	27.978	12.924	9.818	0.162	2185	15.910	263.870	279.780	129.240	98.180	1.620
120	delta	1.2	60x8 weeks	PCR90	M	VAC95	50	Unvaccinate	0.455	0.003	0.055	0.058	0.027	0.020	0.000	218.532	1.591	26.387	27.978	12.924	9.818	0.162	2185	15.910	263.870	279.780	129.240	98.180	1.620
120	delta	1.2	60x8 weeks	PCR90	M	VAC95	50	Unvaccinate	0.455	0.003	0.055	0.058	0.027	0.020	0.000	218.532	1.591	26.387	27.978	12.924	9.818	0.162	2185	15.910	263.870	279.780	129.240	98.180	1.620
120	original	0.6	60x8 weeks	PCR90	M	VAC95	75	Unvaccinate	0.248	0.002	0.000	0.003	0.001	0.001	0.000	119.051	1.164	0.128	1.292	0.418	0.516	0.130	1191	11.640	1.280	12.920	4.180	5.160	1.300
120	original	0.6	60x8 weeks	PCR90	M	VAC95	75	Unvaccinate	0.248	0.002	0.000	0.003	0.001	0.001	0.000	119.051	1.164	0.128	1.292	0.418	0.516	0.130	1191	11.640	1.280	12.920	4.180	5.160	1.300
120	original	0.6	60x8 weeks	PCR90	M	VAC95	75	Unvaccinate	0.248	0.002	0.000	0.003	0.001	0.001	0.000	119.051	1.164	0.128	1.292	0.418	0.516	0.130	1191	11.640	1.280	12.920	4.180	5.160	1.300
120	original	1	60x8 weeks	PCR90	M	VAC95	75	Unvaccinate	0.248	0.003	0.000	0.003	0.001	0.001	0.000	119.004	1.221	0.212	1.433	0.490	0.516	0.123	1190	12.210	2.120	14.330	4.900	5.160	1.230
120	original	1	60x8 weeks	PCR90	M	VAC95	75	Unvaccinate	0.248	0.003	0.000	0.003	0.001	0.001	0.000	119.004	1.221	0.212	1.433	0.490	0.516	0.123	1190	12.210	2.120	14.330	4.900	5.160	1.230
120	original	1	60x8 weeks	PCR90	M	VAC95	75	Unvaccinate	0.248	0.003	0.000	0.003	0.001	0.001	0.000	119.004	1.221	0.212	1.433	0.490	0.516	0.123	1190	12.210	2.120	14.330	4.900	5.160	1.230
120	original	1.2	60x8 weeks	PCR90	M	VAC95	75	Unvaccinate	0.248	0.002	0.001	0.003	0.001	0.001	0.000	118.997	1.151	0.282	1.433	0.481	0.542	0.097	1190	11.510	2.820	14.330	4.810	5.420	0.970
120	original	1.2	60x8 weeks	PCR90	M	VAC95	75	Unvaccinate	0.248	0.002	0.001	0.003	0.001	0.001	0.000	118.997	1.151	0.282	1.433	0.481	0.542	0.097	1190	11.510	2.820	14.330	4.810	5.420	0.970
120	original	1.2	60x8 weeks	PCR90	M	VAC95	75	Unvaccinate	0.248	0.002	0.001	0.003	0.001	0.001	0.000	118.997	1.151	0.282	1.433	0.481	0.542	0.097	1190	11.510	2.820	14.330	4.810	5.420	0.970
120	alpha	0.6	60x8 weeks	PCR90	M	VAC95	75	Unvaccinate	0.247	0.003	0.001	0.003	0.001	0.001	0.000	118.775	1.253	0.370	1.623	0.663	0.612	0.123	1188	12.530	3.700	16.230	6.630	6.120	1.230
120	alpha	0.6	60x8 weeks	PCR90	M	VAC95	75	Unvaccinate	0.247	0.003	0.001	0.003	0.001	0.001	0.000	118.775	1.253	0.370	1.623	0.663	0.612	0.123	1188	12.530	3.700	16.230	6.630	6.120	1.230
120	alpha	0.6	60x8 weeks	PCR90	M	VAC95	75	Unvaccinate	0.247	0.003	0.001	0.003	0.001	0.001	0.000	118.775	1.253	0.370	1.623	0.663	0.612	0.123	1188	12.530	3.700	16.230	6.630	6.120	1.230
120	alpha	1	60x8 weeks	PCR90	M	VAC95	75	Unvaccinate	0.247	0.003	0.002	0.004	0.002	0.001	0.000	118.596	1.232	0.734	1.966	0.822	0.685	0.116	1186	12.320	7.340	19.660	8.220	6.850	1.160
120	alpha	1	60x8 weeks	PCR90	M	VAC95	75	Unvaccinate	0.247	0.003	0.002	0.004	0.002	0.001	0.000	118.596	1.232	0.734	1.966	0.822	0.685	0.116	1186	12.320	7.340	19.660	8.220	6.850	1.160
120	alpha	1	60x8 weeks	PCR90	M	VAC95	75	Unvaccinate	0.247	0.003	0.002	0.004	0.002	0.001	0.000	118.596	1.232	0.734	1.966	0.822	0.685	0.116	1186	12.320	7.340	19.660	8.220	6.850	1.160
120	alpha	1.2	60x8 weeks	PCR90	M	VAC95	75	Unvaccinate	0.247	0.002	0.002	0.005	0.002	0.002	0.000	118.414	1.196	0.982	2.178	0.913	0.796	0.103	1184	11.960	9.820	21.780	9.130	7.960	1.030
120	alpha	1.2	60x8 weeks	PCR90	M	VAC95	75	Unvaccinate	0.247	0.002	0.002	0.005	0.002	0.002	0.000	118.414	1.196	0.982	2.178	0.913	0.796	0.103	1184	11.960	9.820	21.780	9.130	7.960	1.030
120	alpha	1.2	60x8 weeks	PCR90	M	VAC95	75	Unvaccinate	0.247	0.002	0.002	0.005	0.002	0.002	0.000	118.414	1.196	0.982	2.178	0.913	0.796	0.103	1184	11.960	9.820	21.780	9.130	7.960	1.030
120	delta	0.6	60x8 weeks	PCR90	M	VAC95	75	Unvaccinate	0.246	0.003	0.003	0.006	0.003	0.002	0.000	118.129	1.415	1.375	2.790	1.337	0.834	0.121	1181	14.150	13.750	27.900	13.370	8.340	1.210
120	delta	0.6	60x8 weeks	PCR90	M	VAC95	75	Unvaccinate	0.246	0.003	0.003	0.006	0.003	0.002	0.000	118.129	1.415	1.375	2.790	1.337	0.834	0.121	1181	14.150	13.750	27.900	13.370	8.340	1.210
120	delta	0.6	60x8 weeks	PCR90	M	VAC95	75	Unvaccinate	0.246	0.003	0.003	0.006	0.003	0.002	0.000	118.129	1.415	1.375	2.790	1.337	0.834	0.121	1181	14.150	13.750	27.900	13.370	8.340	1.210
120	delta	1	60x8 weeks	PCR90	M	VAC95	75	Unvaccinate	0.243	0.003	0.008	0.011	0.005	0.003	0.000	116.793	1.307	3.857	5.164	2.495	1.451	0.117	1168	13.070	38.570	51.640	24.950	14.510	1.170
120	delta	1	60x8 weeks	PCR90	M	VAC95	75	Unvaccinate	0.243	0.003	0.008	0.011	0.005	0.003	0.000	116.793	1.307	3.857	5.164	2.495	1.451	0.117	1168	13.070	38.570	51.640	24.950	14.510	1.170
120	delta	1	60x8 weeks	PCR90	M	VAC95	75	Unvaccinate	0.243	0.003	0.008	0.011	0.005	0.003	0.000	116.793	1.307	3.857	5.164	2.495	1.451	0.117	1168	13.070	38.570	51.640	24.950	14.510	1.170
120	delta	1.2	60x8 weeks	PCR90	M	VAC95	75	Unvaccinate	0.241	0.003	0.012	0.015	0.007	0.004	0.000	115.859	1.305	5.891	7.196	3.403	1.945	0.095	1159	13.050	58.910	71.960	34.030	19.450	0.950
120	delta	1.2	60x8 weeks	PCR90	M	VAC95	75	Unvaccinate	0.241	0.003	0.012	0.015	0.007	0.004	0.000	115.859	1.305	5.891	7.196	3.403	1.945	0.095	1159	13.050	58.910	71.960	34.030	19.450	0.950
120	delta	1.2	60x8 weeks	PCR90	M	VAC95	75	Unvaccinate	0.241	0.003	0.012	0.015	0.007	0.004	0.000	115.859	1.305	5.891	7.196	3.403	1.945	0.095	1159	13.050	58.910	71.960	34.030	19.450	0.950
120	original	0.6	60x8 weeks	PCR90	M	VAC95	100	Unvaccinate	0.000	0.001	0.000	0.001	0.000	0.000	0.000	0.000	0.282	0.001	0.283	0.116	0.000	0.000	0	2.820	0.010	2.830	1.160	0.000	0.000
120	original	0.6	60x8 weeks	PCR90	M	VAC95	100	Unvaccinate	0.000	0.001	0.000	0.001	0.000	0.000	0.000	0.000	0.282	0.001	0.283	0.116	0.000	0.000	0	2.820	0.010	2.830	1.160	0.000	0.000
120	original	0.6	60x8 weeks	PCR90	M	VAC95	100	Unvaccinate	0.000	0.001	0.000	0.001	0.000	0.000	0.000	0.000	0.282	0.001	0.283	0.116	0.000	0.000	0	2.820	0.010	2.830	1.160	0.000	0.000
120	original	1	60x8 weeks	PCR90	M	VAC95	100	Unvaccinate	0.000	0.001	0.000	0.001	0.000	0.000	0.000	0.000	0.263	0.000	0.263	0.097	0.000	0.000	0	2.630	0.000	2.630	0.970	0.000	0.000
120	original	1	60x8 weeks	PCR90	M	VAC95	100	Unvaccinate	0.000	0.001	0.000	0.001	0.000	0.000	0.000	0.000	0.263	0.000	0.263	0.097	0.000	0.000	0	2.630	0.000	2.630	0.970	0.000	0.000
120	original	1	60x8 weeks	PCR90	M	VAC95	100	Unvaccinate	0.000	0.001	0.000	0.001	0.000	0.000	0.000	0.000	0.263	0.000	0.263	0.097	0.000	0.000	0	2.630	0.000	2.630	0.970	0.000	0.000
120	original	1.2	60x8 weeks	PCR90	M	VAC95	100	Unvaccinate	0.000	0.001	0.000	0.001	0.000	0.000	0.000	0.000	0.316	0.001	0.317	0.132	0.000	0.000	0	3.160	0.010	3.170	1.320	0.000	0.000
120	original	1.2	60x8 weeks	PCR90	M	VAC95	100	Unvaccinate	0.000	0.001	0.000	0.001	0.000	0.000	0.000	0.000	0.316	0.001	0.317	0.132	0.000	0.000	0	3.160	0.010	3.170	1.320	0.000	0.000
120	original	1.2	60x8 weeks	PCR90	M	VAC95	100	Unvaccinate	0.000	0.001	0.000	0.001	0.000	0.000	0.000	0.000	0.316	0.001	0.317	0.132	0.000	0.000	0	3.160	0.010	3.170	1.320	0.000	0.000
120	alpha	0.6	60x8 weeks	PCR90	M	VAC95	100	Unvaccinate	0.000	0.001	0.000	0.001	0.000	0.000	0.000	0.000	0.346	0.023	0.369	0.178	0.000	0.000	0	3.460	0.230	3.690	1.780	0.000	0.000
120	alpha	0.6	60x8 weeks	PCR90	M	VAC95	100	Unvaccinate	0.000	0.001	0.000	0.001	0.000	0.000	0.000	0.000	0.346	0.023	0.369	0.178	0.000	0.000	0	3.460	0.230	3.690	1.780	0.000	0.000
120	alpha	0.6	60x8 weeks	PCR90	M	VAC95	100	Unvaccinate	0.000	0.001	0.000	0.001	0.000	0.000	0.000	0.000	0.346	0.023	0.369	0.178	0.000	0.000	0	3.460	0.230	3.690	1.780	0.000	0.000
120	alpha	1	60x8 weeks	PCR90	M	VAC95	100	Unvaccinate	0.000	0.001	0.000	0.001	0.000	0.000	0.000	0.000	0.369	0.041	0.410	0.217	0.000	0.000	0	3.690	0.410	4.100	2.170	0.000	0.000
120	alpha	1	60x8 weeks	PCR90	M	VAC95	100	Unvaccinate	0.000	0.001	0.000	0.001	0.000	0.000	0.000	0.000	0.369	0.041	0.410	0.217	0.000	0.000	0	3.690	0.410	4.100	2.170	0.000	0.000
120	alpha	1	60x8 weeks	PCR90	M	VAC95	100	Unvaccinate	0.000	0.001	0.000	0.001	0.000	0.000	0.000	0.000	0.369	0.041	0.410	0.217	0.000	0.000	0	3.690	0.410	4.100	2.170	0.000	0.000
120	alpha	1.2	60x8 weeks	PCR90	M	VAC95	100	Unvaccinate	0.000	0.001	0.000	0.001	0.000	0.000	0.000	0.000	0.349	0.041	0.390	0.189	0.000	0.000	0	3.490	0.410	3.900	1.890	0.000	0.000
120	alpha	1.2	60x8 weeks	PCR90	M	VAC95	100	Unvaccinate	0.000	0.001	0.000	0.001	0.000	0.000	0.000	0.000	0.349	0.041	0.390	0.189	0.000	0.000	0	3.490	0.410	3.900	1.890	0.000	0.000
120	alpha	1.2	60x8 weeks	PCR90	M	VAC95	100	Unvaccinate	0.000	0.001	0.000	0.001	0.000	0.000	0.000	0.000	0.349	0.041	0.390	0.189	0.000	0.000	0	3.490	0.410	3.900	1.890	0.000	0.000
120	delta	0.6	60x8 weeks	PCR90	M	VAC95	100	Unvaccinate	0.000	0.001	0.000	0.001	0.001	0.000	0.000	0.000	0.559	0.128	0.687	0.378	0.000	0.000	0	5.590	1.280	6.870	3.780	0.000	0.000
120	delta	0.6	60x8 weeks	PCR90	M	VAC95	100	Unvaccinate	0.000	0.001	0.000	0.001	0.001	0.000	0.000	0.000	0.559	0.128	0.687	0.378	0.000	0.000	0	5.590	1.280	6.870	3.780	0.000	0.000
120	delta	0.6	60x8 weeks	PCR90	M	VAC95	100	Unvaccinate	0.000	0.001	0.000	0.001	0.001	0.000	0.000	0.000	0.559	0.128	0.687	0.378	0.000	0.000	0	5.590	1.280	6.870	3.780	0.000	0.000
120	delta	1	60x8 weeks	PCR90	M	VAC95	100	Unvaccinate	0.000	0.001	0.000	0.002	0.001	0.000	0.000	0.000	0.534	0.215	0.749	0.402	0.000	0.000	0	5.340	2.150	7.490	4.020	0.000	0.000
120	delta	1	60x8 weeks	PCR90	M	VAC95	100	Unvaccinate	0.000	0.001	0.000	0.002	0.001	0.000	0.000	0.000	0.534	0.215	0.749	0.402	0.000	0.000	0	5.340	2.150	7.490	4.020	0.000	0.000
120	delta	1	60x8 weeks	PCR90	M	VAC95	100	Unvaccinate	0.000	0.001	0.000	0.002	0.001	0.000	0.000	0.000	0.534	0.215	0.749	0.402	0.000	0.000	0	5.340	2.150	7.490	4.020	0.000	0.000
120	delta	1.2	60x8 weeks	PCR90	M	VAC95	100	Unvaccinate	0.000	0.001	0.001	0.002	0.001	0.000	0.000	0.000	0.572	0.304	0.876	0.458	0.000	0.000	0	5.720	3.040	8.760	4.580	0.000	0.000
120	delta	1.2	60x8 weeks	PCR90	M	VAC95	100	Unvaccinate	0.000	0.001	0.001	0.002	0.001	0.000	0.000	0.000	0.572	0.304	0.876	0.458	0.000	0.000	0	5.720	3.040	8.760	4.580	0.000	0.000
120	delta	1.2	60x8 weeks	PCR90	M	VAC95	100	Unvaccinate	0.000	0.001	0.001	0.002	0.001	0.000	0.000	0.000	0.572	0.304	0.876	0.458	0.000	0.000	0	5.720	3.040	8.760	4.580	0.000	0.000
120	original	0.6	60x8 weeks	PCR90	M	VAC70	25	Unvaccinate	0.743	0.007	0.003	0.009	0.003	0.004	0.001	356.480	3.165	1.359	4.524	1.554	1.884	0.352	3565	31.650	13.590	45.240	15.540	18.840	3.520
120	original	0.6	60x8 weeks	PCR90	M	VAC70	25	Unvaccinate	0.743	0.007	0.003	0.009	0.003	0.004	0.001	356.480	3.165	1.359	4.524	1.554	1.884	0.352	3565	31.650	13.590	45.240	15.540	18.840	3.520
120	original	0.6	60x8 weeks	PCR90	M	VAC70	25	Unvaccinate	0.743	0.007	0.003	0.009	0.003	0.004	0.001	356.480	3.165	1.359	4.524	1.554	1.884	0.352	3565	31.650	13.590	45.240	15.540	18.840	3.520
120	original	1	60x8 weeks	PCR90	M	VAC70	25	Unvaccinate	0.740	0.007	0.006	0.012	0.004	0.005	0.001	355.414	3.206	2.754	5.960	2.074	2.490	0.343	3554	32.060	27.540	59.600	20.740	24.900	3.430

120	original	1	60x8 weeks	PCR90	M	VAC70	25	Unvaccinate	0.740	0.007	0.006	0.012	0.004	0.005	0.001	355.414	3.206	2.754	5.960	2.074	2.490	0.343	3554	32.060	27.540	59.600	20.740	24.900	3.430
120	original	1	60x8 weeks	PCR90	M	VAC70	25	Unvaccinate	0.740	0.007	0.006	0.012	0.004	0.005	0.001	355.414	3.206	2.754	5.960	2.074	2.490	0.343	3554	32.060	27.540	59.600	20.740	24.900	3.430
120	original	1.2	60x8 weeks	PCR90	M	VAC70	25	Unvaccinate	0.738	0.007	0.008	0.015	0.005	0.006	0.001	354.472	3.169	3.950	7.119	2.429	3.035	0.351	3545	31.690	39.500	71.190	24.290	30.350	3.510
120	original	1.2	60x8 weeks	PCR90	M	VAC70	25	Unvaccinate	0.738	0.007	0.008	0.015	0.005	0.006	0.001	354.472	3.169	3.950	7.119	2.429	3.035	0.351	3545	31.690	39.500	71.190	24.290	30.350	3.510
120	original	1.2	60x8 weeks	PCR90	M	VAC70	25	Unvaccinate	0.738	0.007	0.008	0.015	0.005	0.006	0.001	354.472	3.169	3.950	7.119	2.429	3.035	0.351	3545	31.690	39.500	71.190	24.290	30.350	3.510
120	alpha	0.6	60x8 weeks	PCR90	M	VAC70	25	Unvaccinate	0.736	0.007	0.010	0.017	0.007	0.007	0.001	353.216	3.157	4.898	8.055	3.319	3.422	0.311	3532	31.570	48.980	80.550	33.190	34.220	3.110
120	alpha	0.6	60x8 weeks	PCR90	M	VAC70	25	Unvaccinate	0.736	0.007	0.010	0.017	0.007	0.007	0.001	353.216	3.157	4.898	8.055	3.319	3.422	0.311	3532	31.570	48.980	80.550	33.190	34.220	3.110
120	alpha	0.6	60x8 weeks	PCR90	M	VAC70	25	Unvaccinate	0.736	0.007	0.010	0.017	0.007	0.007	0.001	353.216	3.157	4.898	8.055	3.319	3.422	0.311	3532	31.570	48.980	80.550	33.190	34.220	3.110
120	alpha	1	60x8 weeks	PCR90	M	VAC70	25	Unvaccinate	0.717	0.006	0.033	0.039	0.016	0.017	0.001	344.018	2.880	15.935	18.815	7.854	8.046	0.315	3440	28.800	159.350	188.150	78.540	80.460	3.150
120	alpha	1	60x8 weeks	PCR90	M	VAC70	25	Unvaccinate	0.717	0.006	0.033	0.039	0.016	0.017	0.001	344.018	2.880	15.935	18.815	7.854	8.046	0.315	3440	28.800	159.350	188.150	78.540	80.460	3.150
120	alpha	1	60x8 weeks	PCR90	M	VAC70	25	Unvaccinate	0.717	0.006	0.033	0.039	0.016	0.017	0.001	344.018	2.880	15.935	18.815	7.854	8.046	0.315	3440	28.800	159.350	188.150	78.540	80.460	3.150
120	alpha	1.2	60x8 weeks	PCR90	M	VAC70	25	Unvaccinate	0.702	0.005	0.051	0.056	0.024	0.024	0.001	337.087	2.558	24.404	26.962	11.516	11.419	0.265	3371	25.580	244.040	269.620	115.160	114.190	2.650
120	alpha	1.2	60x8 weeks	PCR90	M	VAC70	25	Unvaccinate	0.702	0.005	0.051	0.056	0.024	0.024	0.001	337.087	2.558	24.404	26.962	11.516	11.419	0.265	3371	25.580	244.040	269.620	115.160	114.190	2.650
120	alpha	1.2	60x8 weeks	PCR90	M	VAC70	25	Unvaccinate	0.702	0.005	0.051	0.056	0.024	0.024	0.001	337.087	2.558	24.404	26.962	11.516	11.419	0.265	3371	25.580	244.040	269.620	115.160	114.190	2.650
120	delta	0.6	60x8 weeks	PCR90	M	VAC70	25	Unvaccinate	0.693	0.005	0.062	0.067	0.031	0.026	0.001	332.790	2.438	29.569	32.007	15.048	12.397	0.253	3328	24.380	295.690	320.070	150.480	123.970	2.530
120	delta	0.6	60x8 weeks	PCR90	M	VAC70	25	Unvaccinate	0.693	0.005	0.062	0.067	0.031	0.026	0.001	332.790	2.438	29.569	32.007	15.048	12.397	0.253	3328	24.380	295.690	320.070	150.480	123.970	2.530
120	delta	0.6	60x8 weeks	PCR90	M	VAC70	25	Unvaccinate	0.693	0.005	0.062	0.067	0.031	0.026	0.001	332.790	2.438	29.569	32.007	15.048	12.397	0.253	3328	24.380	295.690	320.070	150.480	123.970	2.530
120	delta	1	60x8 weeks	PCR90	M	VAC70	25	Unvaccinate	0.657	0.004	0.104	0.107	0.053	0.040	0.000	315.526	1.772	49.810	51.582	25.220	19.312	0.189	3155	17.720	498.100	515.820	252.200	193.120	1.890
120	delta	1	60x8 weeks	PCR90	M	VAC70	25	Unvaccinate	0.657	0.004	0.104	0.107	0.053	0.040	0.000	315.526	1.772	49.810	51.582	25.220	19.312	0.189	3155	17.720	498.100	515.820	252.200	193.120	1.890
120	delta	1	60x8 weeks	PCR90	M	VAC70	25	Unvaccinate	0.657	0.004	0.104	0.107	0.053	0.040	0.000	315.526	1.772	49.810	51.582	25.220	19.312	0.189	3155	17.720	498.100	515.820	252.200	193.120	1.890
120	delta	1.2	60x8 weeks	PCR90	M	VAC70	25	Unvaccinate	0.654	0.003	0.107	0.110	0.055	0.041	0.000	313.991	1.579	51.356	52.935	26.533	19.671	0.206	3140	15.790	513.560	529.350	265.330	196.710	2.060
120	delta	1.2	60x8 weeks	PCR90	M	VAC70	25	Unvaccinate	0.654	0.003	0.107	0.110	0.055	0.041	0.000	313.991	1.579	51.356	52.935	26.533	19.671	0.206	3140	15.790	513.560	529.350	265.330	196.710	2.060
120	delta	1.2	60x8 weeks	PCR90	M	VAC70	25	Unvaccinate	0.654	0.003	0.107	0.110	0.055	0.041	0.000	313.991	1.579	51.356	52.935	26.533	19.671	0.206	3140	15.790	513.560	529.350	265.330	196.710	2.060
120	original	0.6	60x8 weeks	PCR90	M	VAC70	50	Unvaccinate	0.496	0.005	0.001	0.007	0.002	0.002	0.000	237.952	2.573	0.610	3.183	1.167	1.106	0.230	2380	25.730	6.100	31.830	11.670	11.060	2.300
120	original	0.6	60x8 weeks	PCR90	M	VAC70	50	Unvaccinate	0.496	0.005	0.001	0.007	0.002	0.002	0.000	237.952	2.573	0.610	3.183	1.167	1.106	0.230	2380	25.730	6.100	31.830	11.670	11.060	2.300
120	original	0.6	60x8 weeks	PCR90	M	VAC70	50	Unvaccinate	0.496	0.005	0.001	0.007	0.002	0.002	0.000	237.952	2.573	0.610	3.183	1.167	1.106	0.230	2380	25.730	6.100	31.830	11.670	11.060	2.300
120	original	1	60x8 weeks	PCR90	M	VAC70	50	Unvaccinate	0.495	0.005	0.002	0.008	0.003	0.003	0.000	237.663	2.625	1.177	3.802	1.436	1.295	0.227	2377	26.250	11.770	38.020	14.360	12.950	2.270
120	original	1	60x8 weeks	PCR90	M	VAC70	50	Unvaccinate	0.495	0.005	0.002	0.008	0.003	0.003	0.000	237.663	2.625	1.177	3.802	1.436	1.295	0.227	2377	26.250	11.770	38.020	14.360	12.950	2.270
120	original	1	60x8 weeks	PCR90	M	VAC70	50	Unvaccinate	0.495	0.005	0.002	0.008	0.003	0.003	0.000	237.663	2.625	1.177	3.802	1.436	1.295	0.227	2377	26.250	11.770	38.020	14.360	12.950	2.270
120	original	1.2	60x8 weeks	PCR90	M	VAC70	50	Unvaccinate	0.495	0.005	0.003	0.008	0.003	0.003	0.000	237.384	2.525	1.516	4.041	1.527	1.402	0.224	2374	25.250	15.160	40.410	15.270	14.020	2.240
120	original	1.2	60x8 weeks	PCR90	M	VAC70	50	Unvaccinate	0.495	0.005	0.003	0.008	0.003	0.003	0.000	237.384	2.525	1.516	4.041	1.527	1.402	0.224	2374	25.250	15.160	40.410	15.270	14.020	2.240
120	original	1.2	60x8 weeks	PCR90	M	VAC70	50	Unvaccinate	0.495	0.005	0.003	0.008	0.003	0.003	0.000	237.384	2.525	1.516	4.041	1.527	1.402	0.224	2374	25.250	15.160	40.410	15.270	14.020	2.240
120	alpha	0.6	60x8 weeks	PCR90	M	VAC70	50	Unvaccinate	0.493	0.005	0.004	0.010	0.004	0.003	0.000	236.748	2.476	2.158	4.634	1.982	1.655	0.221	2367	24.760	21.580	46.340	19.820	16.550	2.210
120	alpha	0.6	60x8 weeks	PCR90	M	VAC70	50	Unvaccinate	0.493	0.005	0.004	0.010	0.004	0.003	0.000	236.748	2.476	2.158	4.634	1.982	1.655	0.221	2367	24.760	21.580	46.340	19.820	16.550	2.210
120	alpha	0.6	60x8 weeks	PCR90	M	VAC70	50	Unvaccinate	0.493	0.005	0.004	0.010	0.004	0.003	0.000	236.748	2.476	2.158	4.634	1.982	1.655	0.221	2367	24.760	21.580	46.340	19.820	16.550	2.210
120	alpha	1	60x8 weeks	PCR90	M	VAC70	50	Unvaccinate	0.487	0.005	0.014	0.019	0.009	0.007	0.000	233.643	2.503	6.694	9.197	4.155	3.227	0.187	2336	25.030	66.940	91.970	41.550	32.270	1.870
120	alpha	1	60x8 weeks	PCR90	M	VAC70	50	Unvaccinate	0.487	0.005	0.014	0.019	0.009	0.007	0.000	233.643	2.503	6.694	9.197	4.155	3.227	0.187	2336	25.030	66.940	91.970	41.550	32.270	1.870
120	alpha	1	60x8 weeks	PCR90	M	VAC70	50	Unvaccinate	0.487	0.005	0.014	0.019	0.009	0.007	0.000	233.643	2.503	6.694	9.197	4.155	3.227	0.187	2336	25.030	66.940	91.970	41.550	32.270	1.870
120	alpha	1.2	60x8 weeks	PCR90	M	VAC70	50	Unvaccinate	0.484	0.005	0.020	0.025	0.011	0.009	0.000	232.090	2.437	9.403	11.840	5.256	4.129	0.214	2321	24.370	94.030	118.400	52.560	41.290	2.140
120	alpha	1.2	60x8 weeks	PCR90	M	VAC70	50	Unvaccinate	0.484	0.005	0.020	0.025	0.011	0.009	0.000	232.090	2.437	9.403	11.840	5.256	4.129	0.214	2321	24.370	94.030	118.400	52.560	41.290	2.140
120	alpha	1.2	60x8 weeks	PCR90	M	VAC70	50	Unvaccinate	0.484	0.005	0.020	0.025	0.011	0.009	0.000	232.090	2.437	9.403	11.840	5.256	4.129	0.214	2321	24.370	94.030	118.400	52.560	41.290	2.140
120	delta	0.6	60x8 weeks	PCR90	M	VAC70	50	Unvaccinate	0.477	0.005	0.030	0.035	0.017	0.011	0.000	228.993	2.479	14.517	16.996	8.387	5.066	0.205	2290	24.790	145.170	169.960	83.870	50.660	2.050
120	delta	0.6	60x8 weeks	PCR90	M	VAC70	50	Unvaccinate	0.477	0.005	0.030	0.035	0.017	0.011	0.000	228.993	2.479	14.517	16.996	8.387	5.066	0.205	2290	24.790	145.170	169.960	83.870	50.660	2.050
120	delta	0.6	60x8 weeks	PCR90	M	VAC70	50	Unvaccinate	0.477	0.005	0.030	0.035	0.017	0.011	0.000	228.993	2.479	14.517	16.996	8.387	5.066	0.205	2290	24.790	145.170	169.960	83.870	50.660	2.050
120	delta	1	60x8 weeks	PCR90	M	VAC70	50	Unvaccinate	0.450	0.004	0.077	0.081	0.043	0.021	0.000	216.172	1.857	36.801	38.658	20.421	10.317	0.163	2162	18.570	368.010	386.580	204.210	103.170	1.630
120	delta	1	60x8 weeks	PCR90	M	VAC70	50	Unvaccinate	0.450	0.004	0.077	0.081	0.043	0.021	0.000	216.172	1.857	36.801	38.658	20.421	10.317	0.163	2162	18.570	368.010	386.580	204.210	103.170	1.630
120	delta	1	60x8 weeks	PCR90	M	VAC70	50	Unvaccinate	0.450	0.004	0.077	0.081	0.043	0.021	0.000	216.172	1.857	36.801	38.658	20.421	10.317	0.163	2162	18.570	368.010	386.580	204.210	103.170	1.630
120	delta	1.2	60x8 weeks	PCR90	M	VAC70	50	Unvaccinate	0.444	0.004	0.090	0.094	0.050	0.025	0.000	212.936	1.717	43.302	45.019	24.130	11.765	0.136	2129	17.170	433.020	450.190	241.300	117.650	1.360
120	delta	1.2	60x8 weeks	PCR90	M	VAC70	50	Unvaccinate	0.444	0.004	0.090	0.094	0.050	0.025	0.000	212.936	1.717	43.302	45.019	24.130	11.765	0.136	2129	17.170	433.020	450.190	241.300	117.650	1.360
120	delta	1.2	60x8 weeks	PCR90	M	VAC70	50	Unvaccinate	0.444	0.004	0.090	0.094	0.050	0.025	0.000	212.936	1.717	43.302	45.019	24.130	11.765	0.136	2129	17.170	433.020	450.190	241.300	117.650	1.360
120	original	0.6	60x8 weeks	PCR90	M	VAC70	75	Unvaccinate	0.248	0.004	0.000	0.005	0.002	0.001	0.000	119.072	2.012	0.228	2.240	0.894	0.517	0.118	1191	20.120	2.280	22.400	8.940	5.170	1.180
120	original	0.6	60x8 weeks	PCR90	M	VAC70	75	Unvaccinate	0.248	0.004	0.000	0.005	0.002	0.001	0.000	119.072	2.012	0.228	2.240	0.894	0.517	0.118	1191	20.120	2.280	22.400	8.940	5.170	1.180
120	original	0.6	60x8 weeks	PCR90	M	VAC70	75	Unvaccinate	0.248	0.004	0.000	0.005	0.002	0.001	0.000	119.072	2.012	0.228	2.240	0.894	0.517	0.118	1191	20.120	2.280	22.400	8.940	5.170	1.180
120	original	1	60x8 weeks	PCR90	M	VAC70	75	Unvaccinate	0.248	0.004	0.001	0.005	0.002	0.001	0.000	118.984	1.899	0.456	2.355	0.995	0.552	0.116	1190	18.990	4.560	23.550	9.950	5.520	1.160
120	original	1	60x8 weeks	PCR90	M	VAC70	75	Unvaccinate	0.248	0.004	0.001	0.005	0.002	0.001	0.000	118.984	1.899	0.456	2.355	0.995	0.552	0.116	1190	18.990	4.560	23.550	9.950	5.520	1.160
120	original	1	60x8 weeks	PCR90	M	VAC70	75	Unvaccinate	0.248	0.004	0.001	0.005	0.002	0.001	0.000	118.984	1.899	0.456	2.355	0.995	0.552	0.116	1190	18.990	4.560	23.550	9.950	5.520	1.160
120	original	1.2	60x8 weeks	PCR90	M	VAC70	75	Unvaccinate	0.248	0.004	0.001	0.005	0.002	0.001	0.000	118.993	1.937	0.463	2.400	1.002	0.529	0.092	1190	19.370	4.630	24.000	10.020	5.290	0.920
120	original	1.2	60x8 weeks	PCR90	M	VAC70	75	Unvaccinate	0.248	0.004	0.001	0.005	0.002	0.001	0.000	118.993	1.937	0.463	2.400	1.002	0.529	0.092	1190	19.370	4.630	24.000	10.020	5.290	0.920
120	original	1.2	60x8 weeks	PCR90	M	VAC70	75	Unvaccinate	0.248	0.004	0.001	0.005	0.002	0.001	0.000	118.993	1.937	0.463	2.400	1.002	0.529	0.092	1190	19.370	4.630	24.000	10.020	5.290	0.920
120	alpha	0.6	60x8 weeks	PCR90	M	VAC70	75	Unvaccinate	0.247	0.004	0.002	0.006	0.003	0.001	0.000	118.789	1.906	0.849	2.755	1.307	0.644	0.122	1188	19.060	8.490	27.550	13.070	6.440	1.220
120	alpha	0.6	60x8 weeks	PCR90	M	VAC70	75	Unvaccinate	0.247	0.004	0.002	0.006	0.003	0.001	0.000	118.789	1.906	0.849	2.755	1.307	0.644	0.122	1188	19.060	8.490	27.550	13.070	6.440	1.220
120	alpha	0.6	60x8 weeks	PCR90	M	VAC70	75	Unvaccinate	0.247	0.004	0.002	0.006	0.003	0.001	0.000	118.789	1.906	0.849	2.755	1.307	0.644	0.122	1188	19.060	8.490	27.550	13.070	6.440	1.220
120	alpha	1	60x8 weeks	PCR90	M	VAC70	75	Unvaccinate	0.246	0.004	0.004	0.008	0.004	0.002	0.000	118.214	1.938	1.925	3.863	1.882	0.928	0.132	1182	19.380	19.250	38.630	18.820	9.280	1.320
120	alpha	1	60x8 weeks	PCR90	M	VAC70	75	Unvaccinate	0.246	0.004	0.004	0.008	0.004	0.002	0.000	118.214	1.938	1.925	3.863	1.882	0.928	0.132	1182	19.380	19.250	38.630	18.820	9.280	1.320
120	alpha	1	60x8 weeks	PCR90	M	VAC70	75	Unvaccinate	0.246	0.004	0.004	0.008	0.004	0.002	0.000	118.214	1.938	1.925	3.863	1.882	0.928	0.132	1182	19.380	19.250	38.630	18.820	9.280	1.320
120	alpha	1.2	60x8 weeks	PCR90	M	VAC70	75	Unvaccinate	0.245	0.004	0.006	0.010	0.005	0.002	0.000	117.739	1.862	2.959	4.821	2.329	1.178	0.131	1177	18.620	29.590	48.210	23.290	11.780	1.310
120	alpha	1.2	60x8 weeks	PCR90	M	VAC70	75	Unvaccinate	0.245	0.004	0.006	0.010	0.005	0.002	0.000	117.739	1.862	2.959	4.821	2.329	1.178	0.131	1177	18.620	29.590	48.210	23.290	11.780	1.310
120	alpha	1.2	60x8 weeks	PCR90	M	VAC70	75	Unvaccinate	0.245	0.004	0.006	0.010	0.005	0.002	0.000	117.739	1.862	2.959	4.821	2.329	1.178	0.131	1177	18.620	29.590	48.210	23.290	11.780	1.310
120	delta	0.6	60x8 weeks	PCR90	M	VAC70	75	Unvaccinate	0.244	0.005	0.011	0.016	0.008	0.003	0.000	116.899	2.182	5.296	7.478	4.070	1.434	0.108	1169	21.820	52.960	74.780	40.700	14.340	1.080
120	delta	0.6	60x8 weeks	PCR90	M	VAC70	75	Unvaccinate	0.244	0.005	0.011	0.016	0.008	0.003	0.000	116.899	2.182	5.296	7.478	4.070	1.434	0.108	1169	21.820	52.960	74.780	40.700	14.340	1.080
120	delta	0.6	60x8 weeks	PCR90	M	VAC70	75	Unvaccinate	0.244	0.005	0.011	0.016	0.008	0.003	0.000	116.899	2.182	5.296	7.478	4.070	1.434	0.108	1169	21.820	52.960	74.780	40.700	14.340	1.080

120	delta	1	60x8 weeks	PCR90	M	VAC70	75	Unvaccinate	0.236	0.004	0.036	0.040	0.022	0.007	0.000	113.130	1.914	17.280	19.194	10.716	3.170	0.114	1131	19.140	172.800	191.940	107.160	31.700	1.140
120	delta	1	60x8 weeks	PCR90	M	VAC70	75	Unvaccinate	0.236	0.004	0.036	0.040	0.022	0.007	0.000	113.130	1.914	17.280	19.194	10.716	3.170	0.114	1131	19.140	172.800	191.940	107.160	31.700	1.140
120	delta	1	60x8 weeks	PCR90	M	VAC70	75	Unvaccinate	0.236	0.004	0.036	0.040	0.022	0.007	0.000	113.130	1.914	17.280	19.194	10.716	3.170	0.114	1131	19.140	172.800	191.940	107.160	31.700	1.140
120	delta	1.2	60x8 weeks	PCR90	M	VAC70	75	Unvaccinate	0.232	0.004	0.052	0.056	0.032	0.008	0.000	111.254	1.776	25.178	26.954	15.175	3.992	0.074	1113	17.760	251.780	269.540	151.750	39.920	0.740
120	delta	1.2	60x8 weeks	PCR90	M	VAC70	75	Unvaccinate	0.232	0.004	0.052	0.056	0.032	0.008	0.000	111.254	1.776	25.178	26.954	15.175	3.992	0.074	1113	17.760	251.780	269.540	151.750	39.920	0.740
120	delta	1.2	60x8 weeks	PCR90	M	VAC70	75	Unvaccinate	0.232	0.004	0.052	0.056	0.032	0.008	0.000	111.254	1.776	25.178	26.954	15.175	3.992	0.074	1113	17.760	251.780	269.540	151.750	39.920	0.740
120	original	0.6	60x8 weeks	PCR90	M	VAC70	100	Unvaccinate	0.000	0.003	0.000	0.003	0.001	0.000	0.000	0.000	1.279	0.007	1.286	0.613	0.000	0.000	0	12.790	0.070	12.860	6.130	0.000	0.000
120	original	0.6	60x8 weeks	PCR90	M	VAC70	100	Unvaccinate	0.000	0.003	0.000	0.003	0.001	0.000	0.000	0.000	1.279	0.007	1.286	0.613	0.000	0.000	0	12.790	0.070	12.860	6.130	0.000	0.000
120	original	0.6	60x8 weeks	PCR90	M	VAC70	100	Unvaccinate	0.000	0.003	0.000	0.003	0.001	0.000	0.000	0.000	1.279	0.007	1.286	0.613	0.000	0.000	0	12.790	0.070	12.860	6.130	0.000	0.000
120	original	1	60x8 weeks	PCR90	M	VAC70	100	Unvaccinate	0.000	0.003	0.000	0.003	0.001	0.000	0.000	0.000	1.256	0.015	1.271	0.643	0.000	0.000	0	12.560	0.150	12.710	6.430	0.000	0.000
120	original	1	60x8 weeks	PCR90	M	VAC70	100	Unvaccinate	0.000	0.003	0.000	0.003	0.001	0.000	0.000	0.000	1.256	0.015	1.271	0.643	0.000	0.000	0	12.560	0.150	12.710	6.430	0.000	0.000
120	original	1	60x8 weeks	PCR90	M	VAC70	100	Unvaccinate	0.000	0.003	0.000	0.003	0.001	0.000	0.000	0.000	1.256	0.015	1.271	0.643	0.000	0.000	0	12.560	0.150	12.710	6.430	0.000	0.000
120	original	1.2	60x8 weeks	PCR90	M	VAC70	100	Unvaccinate	0.000	0.003	0.000	0.003	0.001	0.000	0.000	0.000	1.274	0.012	1.286	0.621	0.000	0.000	0	12.740	0.120	12.860	6.210	0.000	0.000
120	original	1.2	60x8 weeks	PCR90	M	VAC70	100	Unvaccinate	0.000	0.003	0.000	0.003	0.001	0.000	0.000	0.000	1.274	0.012	1.286	0.621	0.000	0.000	0	12.740	0.120	12.860	6.210	0.000	0.000
120	original	1.2	60x8 weeks	PCR90	M	VAC70	100	Unvaccinate	0.000	0.003	0.000	0.003	0.001	0.000	0.000	0.000	1.274	0.012	1.286	0.621	0.000	0.000	0	12.740	0.120	12.860	6.210	0.000	0.000
120	alpha	0.6	60x8 weeks	PCR90	M	VAC70	100	Unvaccinate	0.000	0.003	0.001	0.003	0.002	0.000	0.000	0.000	1.282	0.254	1.536	0.886	0.000	0.000	0	12.820	2.540	15.360	8.860	0.000	0.000
120	alpha	0.6	60x8 weeks	PCR90	M	VAC70	100	Unvaccinate	0.000	0.003	0.001	0.003	0.002	0.000	0.000	0.000	1.282	0.254	1.536	0.886	0.000	0.000	0	12.820	2.540	15.360	8.860	0.000	0.000
120	alpha	0.6	60x8 weeks	PCR90	M	VAC70	100	Unvaccinate	0.000	0.003	0.001	0.003	0.002	0.000	0.000	0.000	1.282	0.254	1.536	0.886	0.000	0.000	0	12.820	2.540	15.360	8.860	0.000	0.000
120	alpha	1	60x8 weeks	PCR90	M	VAC70	100	Unvaccinate	0.000	0.003	0.001	0.004	0.002	0.000	0.000	0.000	1.268	0.501	1.769	1.027	0.000	0.000	0	12.680	5.010	17.690	10.270	0.000	0.000
120	alpha	1	60x8 weeks	PCR90	M	VAC70	100	Unvaccinate	0.000	0.003	0.001	0.004	0.002	0.000	0.000	0.000	1.268	0.501	1.769	1.027	0.000	0.000	0	12.680	5.010	17.690	10.270	0.000	0.000
120	alpha	1	60x8 weeks	PCR90	M	VAC70	100	Unvaccinate	0.000	0.003	0.001	0.004	0.002	0.000	0.000	0.000	1.268	0.501	1.769	1.027	0.000	0.000	0	12.680	5.010	17.690	10.270	0.000	0.000
120	alpha	1.2	60x8 weeks	PCR90	M	VAC70	100	Unvaccinate	0.000	0.003	0.001	0.004	0.002	0.000	0.000	0.000	1.247	0.674	1.921	1.144	0.000	0.000	0	12.470	6.740	19.210	11.440	0.000	0.000
120	alpha	1.2	60x8 weeks	PCR90	M	VAC70	100	Unvaccinate	0.000	0.003	0.001	0.004	0.002	0.000	0.000	0.000	1.247	0.674	1.921	1.144	0.000	0.000	0	12.470	6.740	19.210	11.440	0.000	0.000
120	alpha	1.2	60x8 weeks	PCR90	M	VAC70	100	Unvaccinate	0.000	0.003	0.001	0.004	0.002	0.000	0.000	0.000	1.247	0.674	1.921	1.144	0.000	0.000	0	12.470	6.740	19.210	11.440	0.000	0.000
120	delta	0.6	60x8 weeks	PCR90	M	VAC70	100	Unvaccinate	0.000	0.003	0.003	0.006	0.004	0.000	0.000	0.000	1.616	1.334	2.950	1.782	0.000	0.000	0	16.160	13.340	29.500	17.820	0.000	0.000
120	delta	0.6	60x8 weeks	PCR90	M	VAC70	100	Unvaccinate	0.000	0.003	0.003	0.006	0.004	0.000	0.000	0.000	1.616	1.334	2.950	1.782	0.000	0.000	0	16.160	13.340	29.500	17.820	0.000	0.000
120	delta	0.6	60x8 weeks	PCR90	M	VAC70	100	Unvaccinate	0.000	0.003	0.003	0.006	0.004	0.000	0.000	0.000	1.616	1.334	2.950	1.782	0.000	0.000	0	16.160	13.340	29.500	17.820	0.000	0.000
120	delta	1	60x8 weeks	PCR90	M	VAC70	100	Unvaccinate	0.000	0.003	0.008	0.011	0.007	0.000	0.000	0.000	1.557	3.677	5.234	3.217	0.000	0.000	0	15.570	36.770	52.340	32.170	0.000	0.000
120	delta	1	60x8 weeks	PCR90	M	VAC70	100	Unvaccinate	0.000	0.003	0.008	0.011	0.007	0.000	0.000	0.000	1.557	3.677	5.234	3.217	0.000	0.000	0	15.570	36.770	52.340	32.170	0.000	0.000
120	delta	1	60x8 weeks	PCR90	M	VAC70	100	Unvaccinate	0.000	0.003	0.008	0.011	0.007	0.000	0.000	0.000	1.557	3.677	5.234	3.217	0.000	0.000	0	15.570	36.770	52.340	32.170	0.000	0.000
120	delta	1.2	60x8 weeks	PCR90	M	VAC70	100	Unvaccinate	0.000	0.003	0.012	0.015	0.009	0.000	0.000	0.000	1.526	5.845	7.371	4.435	0.000	0.000	0	15.260	58.450	73.710	44.350	0.000	0.000
120	delta	1.2	60x8 weeks	PCR90	M	VAC70	100	Unvaccinate	0.000	0.003	0.012	0.015	0.009	0.000	0.000	0.000	1.526	5.845	7.371	4.435	0.000	0.000	0	15.260	58.450	73.710	44.350	0.000	0.000
120	delta	1.2	60x8 weeks	PCR90	M	VAC70	100	Unvaccinate	0.000	0.003	0.012	0.015	0.009	0.000	0.000	0.000	1.526	5.845	7.371	4.435	0.000	0.000	0	15.260	58.450	73.710	44.350	0.000	0.000
120	original	0.6	60x8 weeks	PCR90	M	VAC50	25	Unvaccinate	0.743	0.007	0.003	0.010	0.004	0.004	0.001	356.448	3.324	1.457	4.781	1.735	1.925	0.352	3564	33.240	14.570	47.810	17.350	19.250	3.520
120	original	0.6	60x8 weeks	PCR90	M	VAC50	25	Unvaccinate	0.743	0.007	0.003	0.010	0.004	0.004	0.001	356.448	3.324	1.457	4.781	1.735	1.925	0.352	3564	33.240	14.570	47.810	17.350	19.250	3.520
120	original	0.6	60x8 weeks	PCR90	M	VAC50	25	Unvaccinate	0.743	0.007	0.003	0.010	0.004	0.004	0.001	356.448	3.324	1.457	4.781	1.735	1.925	0.352	3564	33.240	14.570	47.810	17.350	19.250	3.520
120	original	1	60x8 weeks	PCR90	M	VAC50	25	Unvaccinate	0.740	0.007	0.007	0.014	0.005	0.005	0.001	355.140	3.370	3.251	6.621	2.420	2.587	0.309	3551	33.700	32.510	66.210	24.200	25.870	3.090
120	original	1	60x8 weeks	PCR90	M	VAC50	25	Unvaccinate	0.740	0.007	0.007	0.014	0.005	0.005	0.001	355.140	3.370	3.251	6.621	2.420	2.587	0.309	3551	33.700	32.510	66.210	24.200	25.870	3.090
120	original	1	60x8 weeks	PCR90	M	VAC50	25	Unvaccinate	0.740	0.007	0.007	0.014	0.005	0.005	0.001	355.140	3.370	3.251	6.621	2.420	2.587	0.309	3551	33.700	32.510	66.210	24.200	25.870	3.090
120	original	1.2	60x8 weeks	PCR90	M	VAC50	25	Unvaccinate	0.738	0.007	0.009	0.016	0.006	0.006	0.001	354.391	3.311	4.371	7.682	2.891	2.972	0.305	3544	33.110	43.710	76.820	28.910	29.720	3.050
120	original	1.2	60x8 weeks	PCR90	M	VAC50	25	Unvaccinate	0.738	0.007	0.009	0.016	0.006	0.006	0.001	354.391	3.311	4.371	7.682	2.891	2.972	0.305	3544	33.110	43.710	76.820	28.910	29.720	3.050
120	original	1.2	60x8 weeks	PCR90	M	VAC50	25	Unvaccinate	0.738	0.007	0.009	0.016	0.006	0.006	0.001	354.391	3.311	4.371	7.682	2.891	2.972	0.305	3544	33.110	43.710	76.820	28.910	29.720	3.050
120	alpha	0.6	60x8 weeks	PCR90	M	VAC50	25	Unvaccinate	0.735	0.007	0.012	0.018	0.008	0.008	0.001	352.890	3.241	5.612	8.853	3.730	3.641	0.350	3529	32.410	56.120	88.530	37.300	36.410	3.500
120	alpha	0.6	60x8 weeks	PCR90	M	VAC50	25	Unvaccinate	0.735	0.007	0.012	0.018	0.008	0.008	0.001	352.890	3.241	5.612	8.853	3.730	3.641	0.350	3529	32.410	56.120	88.530	37.300	36.410	3.500
120	alpha	0.6	60x8 weeks	PCR90	M	VAC50	25	Unvaccinate	0.735	0.007	0.012	0.018	0.008	0.008	0.001	352.890	3.241	5.612	8.853	3.730	3.641	0.350	3529	32.410	56.120	88.530	37.300	36.410	3.500
120	alpha	1	60x8 weeks	PCR90	M	VAC50	25	Unvaccinate	0.712	0.006	0.041	0.047	0.020	0.019	0.001	341.645	2.920	19.851	22.771	9.831	9.295	0.292	3416	29.200	198.510	227.710	98.310	92.950	2.920
120	alpha	1	60x8 weeks	PCR90	M	VAC50	25	Unvaccinate	0.712	0.006	0.041	0.047	0.020	0.019	0.001	341.645	2.920	19.851	22.771	9.831	9.295	0.292	3416	29.200	198.510	227.710	98.310	92.950	2.920
120	alpha	1	60x8 weeks	PCR90	M	VAC50	25	Unvaccinate	0.712	0.006	0.041	0.047	0.020	0.019	0.001	341.645	2.920	19.851	22.771	9.831	9.295	0.292	3416	29.200	198.510	227.710	98.310	92.950	2.920
120	alpha	1.2	60x8 weeks	PCR90	M	VAC50	25	Unvaccinate	0.702	0.006	0.055	0.061	0.026	0.024	0.001	337.062	2.691	26.451	29.142	12.689	11.557	0.273	3371	26.910	264.510	291.420	126.890	115.570	2.730
120	alpha	1.2	60x8 weeks	PCR90	M	VAC50	25	Unvaccinate	0.702	0.006	0.055	0.061	0.026	0.024	0.001	337.062	2.691	26.451	29.142	12.689	11.557	0.273	3371	26.910	264.510	291.420	126.890	115.570	2.730
120	alpha	1.2	60x8 weeks	PCR90	M	VAC50	25	Unvaccinate	0.702	0.006	0.055	0.061	0.026	0.024	0.001	337.062	2.691	26.451	29.142	12.689	11.557	0.273	3371	26.910	264.510	291.420	126.890	115.570	2.730
120	delta	0.6	60x8 weeks	PCR90	M	VAC50	25	Unvaccinate	0.684	0.005	0.076	0.081	0.039	0.030	0.000	328.333	2.551	36.461	39.012	18.958	14.265	0.226	3283	25.510	364.610	390.120	189.580	142.650	2.260
120	delta	0.6	60x8 weeks	PCR90	M	VAC50	25	Unvaccinate	0.684	0.005	0.076	0.081	0.039	0.030	0.000	328.333	2.551	36.461	39.012	18.958	14.265	0.226	3283	25.510	364.610	390.120	189.580	142.650	2.260
120	delta	0.6	60x8 weeks	PCR90	M	VAC50	25	Unvaccinate	0.684	0.005	0.076	0.081	0.039	0.030	0.000	328.333	2.551	36.461	39.012	18.958	14.265	0.226	3283	25.510	364.610	390.120	189.580	142.650	2.260
120	delta	1	60x8 weeks	PCR90	M	VAC50	25	Unvaccinate	0.652	0.004	0.111	0.114	0.058	0.042	0.000	312.983	1.727	53.204	54.931	27.754	20.304	0.197	3130	17.270	532.040	549.310	277.540	203.040	1.970
120	delta	1	60x8 weeks	PCR90	M	VAC50	25	Unvaccinate	0.652	0.004	0.111	0.114	0.058	0.042	0.000	312.983	1.727	53.204	54.931	27.754	20.304	0.197	3130	17.270	532.040	549.310	277.540	203.040	1.970
120	delta	1	60x8 weeks	PCR90	M	VAC50	25	Unvaccinate	0.652	0.004	0.111	0.114	0.058	0.042	0.000	312.983	1.727	53.204	54.931	27.754	20.304	0.197	3130	17.270	532.040	549.310	277.540	203.040	1.970
120	delta	1.2	60x8 weeks	PCR90	M	VAC50	25	Unvaccinate	0.650	0.003	0.113	0.116	0.059	0.043	0.000	311.954	1.615	54.280	55.895	28.494	20.648	0.156	3120	16.150	542.800	558.950	284.940	206.480	1.560
120	delta	1.2	60x8 weeks	PCR90	M	VAC50	25	Unvaccinate	0.650	0.003	0.113	0.116	0.059	0.043	0.000	311.954	1.615	54.280	55.895	28.494	20.648	0.156	3120	16.150	542.800	558.950	284.940	206.480	1.560
120	delta	1.2	60x8 weeks	PCR90	M	VAC50	25	Unvaccinate	0.650	0.003	0.113	0.116	0.059	0.043	0.000	311.954	1.615	54.280	55.895	28.494	20.648	0.156	3120	16.150	542.800	558.950	284.940	206.480	1.560
120	original	0.6	60x8 weeks	PCR90	M	VAC50	50	Unvaccinate	0.495	0.006	0.002	0.008	0.003	0.002	0.000	237.813	3.018	0.833	3.851	1.574	1.163	0.227	2378	30.180	8.330	38.510	15.740	11.630	2.270
120	original	0.6	60x8 weeks	PCR90	M	VAC50	50	Unvaccinate	0.495	0.006	0.002	0.008	0.003	0.002	0.000	237.813	3.018	0.833	3.851	1.574	1.163	0.227	2378	30.180	8.330	38.510	15.740	11.630	2.270
120	original	0.6	60x8 weeks	PCR90	M	VAC50	50	Unvaccinate	0.495	0.006	0.002	0.008	0.003	0.002	0.000	237.813	3.018	0.833	3.851	1.574	1.163	0.227	2378	30.180	8.330	38.510	15.740	11.630	2.270
120	original	1	60x8 weeks	PCR90	M	VAC50	50	Unvaccinate	0.495	0.006	0.003	0.010	0.004	0.003	0.001	237.455	2.983	1.613	4.596	1.835	1.337	0.256	2375	29.830	16.130	45.960	18.350	13.370	2.560
120	original	1	60x8 weeks	PCR90	M	VAC50	50	Unvaccinate	0.495	0.006	0.003	0.010	0.004	0.003	0.001	237.455	2.983	1.613	4.596	1.835	1.337	0.256	2375	29.830	16.130	45.960	18.350	13.370	2.560
120	original	1	60x8 weeks	PCR90	M	VAC50	50	Unvaccinate	0.495	0.006	0.003	0.010	0.004	0.003	0.001	237.455	2.983	1.613	4.596	1.835	1.337	0.256	2375	29.830	16.130	45.960	18.350	13.370	2.560
120	original	1.2	60x8 weeks	PCR90	M	VAC50	50	Unvaccinate	0.494	0.006	0.004	0.010	0.004	0.003	0.000	237.176	2.939	1.998	4.937	2.042	1.478	0.207	2372	29.390	19.980	49.370	20.420	14.780	2.070
120	original	1.2	60x8 weeks	PCR90	M	VAC50	50	Unvaccinate	0.494	0.006	0.004	0.010	0.004	0.003	0.000	237.176	2.939	1.998	4.937	2.042	1.478	0.207	2372	29.390	19.980	49.370	20.420	14.780	2.070
120	original	1.2	60x8 weeks	PCR90	M	VAC50	50	Unvaccinate	0.494	0.006	0.004	0.010	0.004	0.003	0.000	237.176	2.939	1.998	4.937	2.042	1.478	0.207	2372	29.390	19.980	49.370	20.420	14.780	2.070
120	alpha	0.6	60x8 weeks	PCR90	M	VAC50	50	Unvaccinate	0.493	0.006	0.006	0.012	0.006	0.004	0.000	236.519	2.934	2.809	5.743	2.712	1.792	0.218	2365	29.340	28.090	57.430	27.120	17.920	2.180
120	alpha	0.6	60x8 weeks	PCR90	M	VAC50	50	Unvaccinate	0.493	0.006	0.006	0.012	0.006	0.004	0.000	236.519	2.934	2.809	5.743	2.712	1.792	0.218	2365	29.340	28.090	57.430	27.120	17.920	2.180

120	alpha	0.6	60x8 weeks	PCR90	M	VAC50	50	Unvaccinate	0.493	0.006	0.006	0.012	0.006	0.004	0.000	236.519	2.934	2.809	5.743	2.712	1.792	0.218	2365	29.340	28.090	57.430	27.120	17.920	2.180
120	alpha	1	60x8 weeks	PCR90	M	VAC50	50	Unvaccinate	0.485	0.006	0.020	0.025	0.012	0.008	0.000	232.737	2.793	9.380	12.173	5.790	3.685	0.225	2327	27.930	93.800	121.730	57.900	36.850	2.250
120	alpha	1	60x8 weeks	PCR90	M	VAC50	50	Unvaccinate	0.485	0.006	0.020	0.025	0.012	0.008	0.000	232.737	2.793	9.380	12.173	5.790	3.685	0.225	2327	27.930	93.800	121.730	57.900	36.850	2.250
120	alpha	1	60x8 weeks	PCR90	M	VAC50	50	Unvaccinate	0.485	0.006	0.020	0.025	0.012	0.008	0.000	232.737	2.793	9.380	12.173	5.790	3.685	0.225	2327	27.930	93.800	121.730	57.900	36.850	2.250
120	alpha	1.2	60x8 weeks	PCR90	M	VAC50	50	Unvaccinate	0.479	0.005	0.031	0.036	0.017	0.011	0.000	229.859	2.624	14.760	17.384	8.379	5.180	0.218	2299	26.240	147.600	173.840	83.790	51.800	2.180
120	alpha	1.2	60x8 weeks	PCR90	M	VAC50	50	Unvaccinate	0.479	0.005	0.031	0.036	0.017	0.011	0.000	229.859	2.624	14.760	17.384	8.379	5.180	0.218	2299	26.240	147.600	173.840	83.790	51.800	2.180
120	alpha	1.2	60x8 weeks	PCR90	M	VAC50	50	Unvaccinate	0.479	0.005	0.031	0.036	0.017	0.011	0.000	229.859	2.624	14.760	17.384	8.379	5.180	0.218	2299	26.240	147.600	173.840	83.790	51.800	2.180
120	delta	0.6	60x8 weeks	PCR90	M	VAC50	50	Unvaccinate	0.470	0.006	0.048	0.054	0.028	0.013	0.000	225.556	2.665	23.065	25.730	13.657	6.453	0.179	2256	26.650	230.650	257.300	136.570	64.530	1.790
120	delta	0.6	60x8 weeks	PCR90	M	VAC50	50	Unvaccinate	0.470	0.006	0.048	0.054	0.028	0.013	0.000	225.556	2.665	23.065	25.730	13.657	6.453	0.179	2256	26.650	230.650	257.300	136.570	64.530	1.790
120	delta	0.6	60x8 weeks	PCR90	M	VAC50	50	Unvaccinate	0.470	0.006	0.048	0.054	0.028	0.013	0.000	225.556	2.665	23.065	25.730	13.657	6.453	0.179	2256	26.650	230.650	257.300	136.570	64.530	1.790
120	delta	1	60x8 weeks	PCR90	M	VAC50	50	Unvaccinate	0.442	0.004	0.098	0.103	0.057	0.025	0.000	212.300	1.957	47.260	49.217	27.162	12.150	0.130	2123	19.570	472.600	492.170	271.620	121.500	1.300
120	delta	1	60x8 weeks	PCR90	M	VAC50	50	Unvaccinate	0.442	0.004	0.098	0.103	0.057	0.025	0.000	212.300	1.957	47.260	49.217	27.162	12.150	0.130	2123	19.570	472.600	492.170	271.620	121.500	1.300
120	delta	1	60x8 weeks	PCR90	M	VAC50	50	Unvaccinate	0.442	0.004	0.098	0.103	0.057	0.025	0.000	212.300	1.957	47.260	49.217	27.162	12.150	0.130	2123	19.570	472.600	492.170	271.620	121.500	1.300
120	delta	1.2	60x8 weeks	PCR90	M	VAC50	50	Unvaccinate	0.438	0.004	0.106	0.110	0.061	0.027	0.000	210.300	1.737	51.096	52.833	29.373	12.899	0.126	2103	17.370	510.960	528.330	293.730	128.990	1.260
120	delta	1.2	60x8 weeks	PCR90	M	VAC50	50	Unvaccinate	0.438	0.004	0.106	0.110	0.061	0.027	0.000	210.300	1.737	51.096	52.833	29.373	12.899	0.126	2103	17.370	510.960	528.330	293.730	128.990	1.260
120	delta	1.2	60x8 weeks	PCR90	M	VAC50	50	Unvaccinate	0.438	0.004	0.106	0.110	0.061	0.027	0.000	210.300	1.737	51.096	52.833	29.373	12.899	0.126	2103	17.370	510.960	528.330	293.730	128.990	1.260
120	original	0.6	60x8 weeks	PCR90	M	VAC50	75	Unvaccinate	0.248	0.005	0.001	0.006	0.003	0.001	0.000	119.032	2.525	0.282	2.807	1.255	0.515	0.108	1190	25.250	2.820	28.070	12.550	5.150	1.080
120	original	0.6	60x8 weeks	PCR90	M	VAC50	75	Unvaccinate	0.248	0.005	0.001	0.006	0.003	0.001	0.000	119.032	2.525	0.282	2.807	1.255	0.515	0.108	1190	25.250	2.820	28.070	12.550	5.150	1.080
120	original	0.6	60x8 weeks	PCR90	M	VAC50	75	Unvaccinate	0.248	0.005	0.001	0.006	0.003	0.001	0.000	119.032	2.525	0.282	2.807	1.255	0.515	0.108	1190	25.250	2.820	28.070	12.550	5.150	1.080
120	original	1	60x8 weeks	PCR90	M	VAC50	75	Unvaccinate	0.248	0.005	0.001	0.006	0.003	0.001	0.000	118.940	2.552	0.566	3.118	1.363	0.580	0.113	1189	25.520	5.660	31.180	13.630	5.800	1.130
120	original	1	60x8 weeks	PCR90	M	VAC50	75	Unvaccinate	0.248	0.005	0.001	0.006	0.003	0.001	0.000	118.940	2.552	0.566	3.118	1.363	0.580	0.113	1189	25.520	5.660	31.180	13.630	5.800	1.130
120	original	1	60x8 weeks	PCR90	M	VAC50	75	Unvaccinate	0.248	0.005	0.001	0.006	0.003	0.001	0.000	118.940	2.552	0.566	3.118	1.363	0.580	0.113	1189	25.520	5.660	31.180	13.630	5.800	1.130
120	original	1.2	60x8 weeks	PCR90	M	VAC50	75	Unvaccinate	0.248	0.005	0.002	0.007	0.003	0.001	0.000	118.891	2.553	0.720	3.273	1.498	0.613	0.114	1189	25.530	7.200	32.730	14.980	6.130	1.140
120	original	1.2	60x8 weeks	PCR90	M	VAC50	75	Unvaccinate	0.248	0.005	0.002	0.007	0.003	0.001	0.000	118.891	2.553	0.720	3.273	1.498	0.613	0.114	1189	25.530	7.200	32.730	14.980	6.130	1.140
120	original	1.2	60x8 weeks	PCR90	M	VAC50	75	Unvaccinate	0.248	0.005	0.002	0.007	0.003	0.001	0.000	118.891	2.553	0.720	3.273	1.498	0.613	0.114	1189	25.530	7.200	32.730	14.980	6.130	1.140
120	alpha	0.6	60x8 weeks	PCR90	M	VAC50	75	Unvaccinate	0.247	0.005	0.004	0.009	0.005	0.002	0.000	118.449	2.491	1.691	4.182	2.240	0.804	0.123	1184	24.910	16.910	41.820	22.400	8.040	1.230
120	alpha	0.6	60x8 weeks	PCR90	M	VAC50	75	Unvaccinate	0.247	0.005	0.004	0.009	0.005	0.002	0.000	118.449	2.491	1.691	4.182	2.240	0.804	0.123	1184	24.910	16.910	41.820	22.400	8.040	1.230
120	alpha	0.6	60x8 weeks	PCR90	M	VAC50	75	Unvaccinate	0.247	0.005	0.004	0.009	0.005	0.002	0.000	118.449	2.491	1.691	4.182	2.240	0.804	0.123	1184	24.910	16.910	41.820	22.400	8.040	1.230
120	alpha	1	60x8 weeks	PCR90	M	VAC50	75	Unvaccinate	0.245	0.005	0.008	0.013	0.007	0.002	0.000	117.833	2.425	3.960	6.385	3.374	1.172	0.120	1178	24.250	39.600	63.850	33.740	11.720	1.200
120	alpha	1	60x8 weeks	PCR90	M	VAC50	75	Unvaccinate	0.245	0.005	0.008	0.013	0.007	0.002	0.000	117.833	2.425	3.960	6.385	3.374	1.172	0.120	1178	24.250	39.600	63.850	33.740	11.720	1.200
120	alpha	1	60x8 weeks	PCR90	M	VAC50	75	Unvaccinate	0.245	0.005	0.008	0.013	0.007	0.002	0.000	117.833	2.425	3.960	6.385	3.374	1.172	0.120	1178	24.250	39.600	63.850	33.740	11.720	1.200
120	alpha	1.2	60x8 weeks	PCR90	M	VAC50	75	Unvaccinate	0.244	0.005	0.013	0.018	0.009	0.003	0.000	117.164	2.355	6.104	8.459	4.523	1.456	0.101	1172	23.550	61.040	84.590	45.230	14.560	1.010
120	alpha	1.2	60x8 weeks	PCR90	M	VAC50	75	Unvaccinate	0.244	0.005	0.013	0.018	0.009	0.003	0.000	117.164	2.355	6.104	8.459	4.523	1.456	0.101	1172	23.550	61.040	84.590	45.230	14.560	1.010
120	alpha	1.2	60x8 weeks	PCR90	M	VAC50	75	Unvaccinate	0.244	0.005	0.013	0.018	0.009	0.003	0.000	117.164	2.355	6.104	8.459	4.523	1.456	0.101	1172	23.550	61.040	84.590	45.230	14.560	1.010
120	delta	0.6	60x8 weeks	PCR90	M	VAC50	75	Unvaccinate	0.241	0.006	0.025	0.031	0.018	0.004	0.000	115.501	2.809	11.957	14.766	8.569	2.032	0.127	1155	28.090	119.570	147.660	85.690	20.320	1.270
120	delta	0.6	60x8 weeks	PCR90	M	VAC50	75	Unvaccinate	0.241	0.006	0.025	0.031	0.018	0.004	0.000	115.501	2.809	11.957	14.766	8.569	2.032	0.127	1155	28.090	119.570	147.660	85.690	20.320	1.270
120	delta	0.6	60x8 weeks	PCR90	M	VAC50	75	Unvaccinate	0.241	0.006	0.025	0.031	0.018	0.004	0.000	115.501	2.809	11.957	14.766	8.569	2.032	0.127	1155	28.090	119.570	147.660	85.690	20.320	1.270
120	delta	1	60x8 weeks	PCR90	M	VAC50	75	Unvaccinate	0.226	0.005	0.076	0.080	0.048	0.010	0.000	108.710	2.201	36.342	38.543	23.259	4.893	0.087	1087	22.010	363.420	385.430	232.590	48.930	0.870
120	delta	1	60x8 weeks	PCR90	M	VAC50	75	Unvaccinate	0.226	0.005	0.076	0.080	0.048	0.010	0.000	108.710	2.201	36.342	38.543	23.259	4.893	0.087	1087	22.010	363.420	385.430	232.590	48.930	0.870
120	delta	1	60x8 weeks	PCR90	M	VAC50	75	Unvaccinate	0.226	0.005	0.076	0.080	0.048	0.010	0.000	108.710	2.201	36.342	38.543	23.259	4.893	0.087	1087	22.010	363.420	385.430	232.590	48.930	0.870
120	delta	1.2	60x8 weeks	PCR90	M	VAC50	75	Unvaccinate	0.223	0.004	0.092	0.096	0.058	0.012	0.000	106.808	1.828	44.118	45.946	27.798	5.775	0.067	1068	18.280	441.180	459.460	277.980	57.750	0.670
120	delta	1.2	60x8 weeks	PCR90	M	VAC50	75	Unvaccinate	0.223	0.004	0.092	0.096	0.058	0.012	0.000	106.808	1.828	44.118	45.946	27.798	5.775	0.067	1068	18.280	441.180	459.460	277.980	57.750	0.670
120	delta	1.2	60x8 weeks	PCR90	M	VAC50	75	Unvaccinate	0.223	0.004	0.092	0.096	0.058	0.012	0.000	106.808	1.828	44.118	45.946	27.798	5.775	0.067	1068	18.280	441.180	459.460	277.980	57.750	0.670
120	original	0.6	60x8 weeks	PCR90	M	VAC50	100	Unvaccinate	0.000	0.004	0.000	0.004	0.002	0.000	0.000	0.000	2.091	0.029	2.120	1.052	0.000	0.000	0	20.910	0.290	21.200	10.520	0.000	0.000
120	original	0.6	60x8 weeks	PCR90	M	VAC50	100	Unvaccinate	0.000	0.004	0.000	0.004	0.002	0.000	0.000	0.000	2.091	0.029	2.120	1.052	0.000	0.000	0	20.910	0.290	21.200	10.520	0.000	0.000
120	original	0.6	60x8 weeks	PCR90	M	VAC50	100	Unvaccinate	0.000	0.004	0.000	0.004	0.002	0.000	0.000	0.000	2.091	0.029	2.120	1.052	0.000	0.000	0	20.910	0.290	21.200	10.520	0.000	0.000
120	original	1	60x8 weeks	PCR90	M	VAC50	100	Unvaccinate	0.000	0.004	0.000	0.004	0.002	0.000	0.000	0.000	2.072	0.045	2.117	1.118	0.000	0.000	0	20.720	0.450	21.170	11.180	0.000	0.000
120	original	1	60x8 weeks	PCR90	M	VAC50	100	Unvaccinate	0.000	0.004	0.000	0.004	0.002	0.000	0.000	0.000	2.072	0.045	2.117	1.118	0.000	0.000	0	20.720	0.450	21.170	11.180	0.000	0.000
120	original	1	60x8 weeks	PCR90	M	VAC50	100	Unvaccinate	0.000	0.004	0.000	0.004	0.002	0.000	0.000	0.000	2.072	0.045	2.117	1.118	0.000	0.000	0	20.720	0.450	21.170	11.180	0.000	0.000
120	original	1.2	60x8 weeks	PCR90	M	VAC50	100	Unvaccinate	0.000	0.004	0.000	0.004	0.002	0.000	0.000	0.000	2.053	0.056	2.109	1.103	0.000	0.000	0	20.530	0.560	21.090	11.030	0.000	0.000
120	original	1.2	60x8 weeks	PCR90	M	VAC50	100	Unvaccinate	0.000	0.004	0.000	0.004	0.002	0.000	0.000	0.000	2.053	0.056	2.109	1.103	0.000	0.000	0	20.530	0.560	21.090	11.030	0.000	0.000
120	original	1.2	60x8 weeks	PCR90	M	VAC50	100	Unvaccinate	0.000	0.004	0.000	0.004	0.002	0.000	0.000	0.000	2.053	0.056	2.109	1.103	0.000	0.000	0	20.530	0.560	21.090	11.030	0.000	0.000
120	alpha	0.6	60x8 weeks	PCR90	M	VAC50	100	Unvaccinate	0.000	0.004	0.002	0.006	0.003	0.000	0.000	0.000	2.016	0.724	2.740	1.667	0.000	0.000	0	20.160	7.240	27.400	16.670	0.000	0.000
120	alpha	0.6	60x8 weeks	PCR90	M	VAC50	100	Unvaccinate	0.000	0.004	0.002	0.006	0.003	0.000	0.000	0.000	2.016	0.724	2.740	1.667	0.000	0.000	0	20.160	7.240	27.400	16.670	0.000	0.000
120	alpha	0.6	60x8 weeks	PCR90	M	VAC50	100	Unvaccinate	0.000	0.004	0.002	0.006	0.003	0.000	0.000	0.000	2.016	0.724	2.740	1.667	0.000	0.000	0	20.160	7.240	27.400	16.670	0.000	0.000
120	alpha	1	60x8 weeks	PCR90	M	VAC50	100	Unvaccinate	0.000	0.004	0.003	0.007	0.004	0.000	0.000	0.000	2.014	1.414	3.428	2.016	0.000	0.000	0	20.140	14.140	34.280	20.160	0.000	0.000
120	alpha	1	60x8 weeks	PCR90	M	VAC50	100	Unvaccinate	0.000	0.004	0.003	0.007	0.004	0.000	0.000	0.000	2.014	1.414	3.428	2.016	0.000	0.000	0	20.140	14.140	34.280	20.160	0.000	0.000
120	alpha	1	60x8 weeks	PCR90	M	VAC50	100	Unvaccinate	0.000	0.004	0.003	0.007	0.004	0.000	0.000	0.000	2.014	1.414	3.428	2.016	0.000	0.000	0	20.140	14.140	34.280	20.160	0.000	0.000
120	alpha	1.2	60x8 weeks	PCR90	M	VAC50	100	Unvaccinate	0.000	0.004	0.005	0.009	0.005	0.000	0.000	0.000	2.000	2.179	4.179	2.518	0.000	0.000	0	20.000	21.790	41.790	25.180	0.000	0.000
120	alpha	1.2	60x8 weeks	PCR90	M	VAC50	100	Unvaccinate	0.000	0.004	0.005	0.009	0.005	0.000	0.000	0.000	2.000	2.179	4.179	2.518	0.000	0.000	0	20.000	21.790	41.790	25.180	0.000	0.000
120	alpha	1.2	60x8 weeks	PCR90	M	VAC50	100	Unvaccinate	0.000	0.004	0.005	0.009	0.005	0.000	0.000	0.000	2.000	2.179	4.179	2.518	0.000	0.000	0	20.000	21.790	41.790	25.180	0.000	0.000
120	delta	0.6	60x8 weeks	PCR90	M	VAC50	100	Unvaccinate	0.000	0.005	0.012	0.017	0.011	0.000	0.000	0.000	2.549	5.608	8.157	5.193	0.000	0.000	0	25.490	56.080	81.570	51.930	0.000	0.000
120	delta	0.6	60x8 weeks	PCR90	M	VAC50	100	Unvaccinate	0.000	0.005	0.012	0.017	0.011	0.000	0.000	0.000	2.549	5.608	8.157	5.193	0.000	0.000	0	25.490	56.080	81.570	51.930	0.000	0.000
120	delta	0.6	60x8 weeks	PCR90	M	VAC50	100	Unvaccinate	0.000	0.005	0.012	0.017	0.011	0.000	0.000	0.000	2.549	5.608	8.157	5.193	0.000	0.000	0	25.490	56.080	81.570	51.930	0.000	0.000
120	delta	1	60x8 weeks	PCR90	M	VAC50	100	Unvaccinate	0.000	0.005	0.042	0.047	0.030	0.000	0.000	0.000	2.255	20.212	22.467	14.236	0.000	0.000	0	22.550	202.120	224.670	142.360	0.000	0.000
120	delta	1	60x8 weeks	PCR90	M	VAC50	100	Unvaccinate	0.000	0.005	0.042	0.047	0.030	0.000	0.000	0.000	2.255	20.212	22.467	14.236	0.000	0.000	0	22.550	202.120	224.670	142.360	0.000	0.000
120	delta	1	60x8 weeks	PCR90	M	VAC50	100	Unvaccinate	0.000	0.005	0.042	0.047	0.030	0.000	0.000	0.000	2.255	20.212	22.467	14.236	0.000	0.000	0	22.550	202.120	224.670	142.360	0.000	0.000
120	delta	1.2	60x8 weeks	PCR90	M	VAC50	100	Unvaccinate	0.000	0.004	0.065	0.069	0.044	0.000	0.000	0.000	2.153	31.028	33.181	21.322	0.000	0.000	0	21.530	310.280	331.810	213.220	0.000	0.000
120	delta	1.2	60x8 weeks	PCR90	M	VAC50	100	Unvaccinate	0.000	0.004	0.065	0.069	0.044	0.000	0.000	0.000	2.153	31.028	33.181	21.322	0.000	0.000	0	21.530	310.280	331.810	213.220	0.000	0.000
120	delta	1.2	60x8 weeks	PCR90	M	VAC50	100	Unvaccinate	0.000	0.004	0.065	0.069	0.044	0.000	0.000	0.000	2.153	31.028	33.181	21.322	0.000	0.000	0	21.530	310.280	331.810	213.220	0.000	0.000
120	original	0.6	60x8 weeks	Ag80	Daily	NOVAC	0	Unvaccinate	4.937	0.008	0.001	0.009	0.000	0.009	0.096	2369.991	3.961	0.496	4.457	0.097	4.471	46.261	23700	39.610	4.960	44.570	0.970	44.710	462.610

120	original	0.6	60x8 weeks	Ag80	Daily	NOVAC	0	Unvaccinate	4.937	0.008	0.001	0.009	0.000	0.009	0.096	2369.991	3.961	0.496	4.457	0.097	4.471	46.261	23700	39.610	4.960	44.570	0.970	44.710	462.610
120	original	0.6	60x8 weeks	Ag80	Daily	NOVAC	0	Unvaccinate	4.937	0.008	0.001	0.009	0.000	0.009	0.096	2369.991	3.961	0.496	4.457	0.097	4.471	46.261	23700	39.610	4.960	44.570	0.970	44.710	462.610
120	original	1	60x8 weeks	Ag80	Daily	NOVAC	0	Unvaccinate	4.931	0.008	0.002	0.010	0.000	0.010	0.096	2367.102	3.895	0.954	4.849	0.122	4.867	46.028	23671	38.950	9.540	48.490	1.220	48.670	460.280
120	original	1	60x8 weeks	Ag80	Daily	NOVAC	0	Unvaccinate	4.931	0.008	0.002	0.010	0.000	0.010	0.096	2367.102	3.895	0.954	4.849	0.122	4.867	46.028	23671	38.950	9.540	48.490	1.220	48.670	460.280
120	original	1	60x8 weeks	Ag80	Daily	NOVAC	0	Unvaccinate	4.931	0.008	0.002	0.010	0.000	0.010	0.096	2367.102	3.895	0.954	4.849	0.122	4.867	46.028	23671	38.950	9.540	48.490	1.220	48.670	460.280
120	original	1.2	60x8 weeks	Ag80	Daily	NOVAC	0	Unvaccinate	4.926	0.008	0.003	0.011	0.000	0.011	0.096	2364.349	3.925	1.261	5.186	0.106	5.291	46.228	23643	39.250	12.610	51.860	1.060	52.910	462.280
120	original	1.2	60x8 weeks	Ag80	Daily	NOVAC	0	Unvaccinate	4.926	0.008	0.003	0.011	0.000	0.011	0.096	2364.349	3.925	1.261	5.186	0.106	5.291	46.228	23643	39.250	12.610	51.860	1.060	52.910	462.280
120	original	1.2	60x8 weeks	Ag80	Daily	NOVAC	0	Unvaccinate	4.926	0.008	0.003	0.011	0.000	0.011	0.096	2364.349	3.925	1.261	5.186	0.106	5.291	46.228	23643	39.250	12.610	51.860	1.060	52.910	462.280
120	alpha	0.6	60x8 weeks	Ag80	Daily	NOVAC	0	Unvaccinate	4.928	0.008	0.002	0.010	0.000	0.011	0.097	2365.484	3.936	0.958	4.894	0.125	5.056	46.524	23655	39.360	9.580	48.940	1.250	50.560	465.240
120	alpha	0.6	60x8 weeks	Ag80	Daily	NOVAC	0	Unvaccinate	4.928	0.008	0.002	0.010	0.000	0.011	0.097	2365.484	3.936	0.958	4.894	0.125	5.056	46.524	23655	39.360	9.580	48.940	1.250	50.560	465.240
120	alpha	0.6	60x8 weeks	Ag80	Daily	NOVAC	0	Unvaccinate	4.928	0.008	0.002	0.010	0.000	0.011	0.097	2365.484	3.936	0.958	4.894	0.125	5.056	46.524	23655	39.360	9.580	48.940	1.250	50.560	465.240
120	alpha	1	60x8 weeks	Ag80	Daily	NOVAC	0	Unvaccinate	4.921	0.008	0.003	0.011	0.000	0.012	0.096	2362.000	3.757	1.614	5.371	0.118	5.599	45.878	23620	37.570	16.140	53.710	1.180	55.990	458.780
120	alpha	1	60x8 weeks	Ag80	Daily	NOVAC	0	Unvaccinate	4.921	0.008	0.003	0.011	0.000	0.012	0.096	2362.000	3.757	1.614	5.371	0.118	5.599	45.878	23620	37.570	16.140	53.710	1.180	55.990	458.780
120	alpha	1	60x8 weeks	Ag80	Daily	NOVAC	0	Unvaccinate	4.921	0.008	0.003	0.011	0.000	0.012	0.096	2362.000	3.757	1.614	5.371	0.118	5.599	45.878	23620	37.570	16.140	53.710	1.180	55.990	458.780
120	alpha	1.2	60x8 weeks	Ag80	Daily	NOVAC	0	Unvaccinate	4.909	0.008	0.005	0.013	0.000	0.013	0.095	2356.243	3.922	2.220	6.142	0.136	6.401	45.468	23562	39.220	22.200	61.420	1.360	64.010	454.680
120	alpha	1.2	60x8 weeks	Ag80	Daily	NOVAC	0	Unvaccinate	4.909	0.008	0.005	0.013	0.000	0.013	0.095	2356.243	3.922	2.220	6.142	0.136	6.401	45.468	23562	39.220	22.200	61.420	1.360	64.010	454.680
120	alpha	1.2	60x8 weeks	Ag80	Daily	NOVAC	0	Unvaccinate	4.909	0.008	0.005	0.013	0.000	0.013	0.095	2356.243	3.922	2.220	6.142	0.136	6.401	45.468	23562	39.220	22.200	61.420	1.360	64.010	454.680
120	delta	0.6	60x8 weeks	Ag80	Daily	NOVAC	0	Unvaccinate	4.908	0.008	0.005	0.013	0.000	0.013	0.095	2355.903	3.910	2.215	6.125	0.185	6.378	45.709	23559	39.100	22.150	61.250	1.850	63.780	457.090
120	delta	0.6	60x8 weeks	Ag80	Daily	NOVAC	0	Unvaccinate	4.908	0.008	0.005	0.013	0.000	0.013	0.095	2355.903	3.910	2.215	6.125	0.185	6.378	45.709	23559	39.100	22.150	61.250	1.850	63.780	457.090
120	delta	0.6	60x8 weeks	Ag80	Daily	NOVAC	0	Unvaccinate	4.908	0.008	0.005	0.013	0.000	0.013	0.095	2355.903	3.910	2.215	6.125	0.185	6.378	45.709	23559	39.100	22.150	61.250	1.850	63.780	457.090
120	delta	1	60x8 weeks	Ag80	Daily	NOVAC	0	Unvaccinate	4.872	0.008	0.010	0.018	0.000	0.019	0.092	2338.424	3.819	4.721	8.540	0.227	8.926	44.385	23384	38.190	47.210	85.400	2.270	89.260	443.850
120	delta	1	60x8 weeks	Ag80	Daily	NOVAC	0	Unvaccinate	4.872	0.008	0.010	0.018	0.000	0.019	0.092	2338.424	3.819	4.721	8.540	0.227	8.926	44.385	23384	38.190	47.210	85.400	2.270	89.260	443.850
120	delta	1	60x8 weeks	Ag80	Daily	NOVAC	0	Unvaccinate	4.872	0.008	0.010	0.018	0.000	0.019	0.092	2338.424	3.819	4.721	8.540	0.227	8.926	44.385	23384	38.190	47.210	85.400	2.270	89.260	443.850
120	delta	1.2	60x8 weeks	Ag80	Daily	NOVAC	0	Unvaccinate	4.851	0.008	0.013	0.020	0.001	0.021	0.092	2328.601	3.627	6.185	9.812	0.261	10.265	44.094	23286	36.270	61.850	98.120	2.610	102.650	440.940
120	delta	1.2	60x8 weeks	Ag80	Daily	NOVAC	0	Unvaccinate	4.851	0.008	0.013	0.020	0.001	0.021	0.092	2328.601	3.627	6.185	9.812	0.261	10.265	44.094	23286	36.270	61.850	98.120	2.610	102.650	440.940
120	delta	1.2	60x8 weeks	Ag80	Daily	NOVAC	0	Unvaccinate	4.851	0.008	0.013	0.020	0.001	0.021	0.092	2328.601	3.627	6.185	9.812	0.261	10.265	44.094	23286	36.270	61.850	98.120	2.610	102.650	440.940
120	original	0.6	60x8 weeks	Ag80	Daily	VAC95	25	Unvaccinate	3.705	0.006	0.001	0.007	0.000	0.007	0.073	1778.164	2.994	0.291	3.285	0.117	3.227	34.961	17782	29.940	2.910	32.850	1.170	32.270	349.610
120	original	0.6	60x8 weeks	Ag80	Daily	VAC95	25	Unvaccinate	3.705	0.006	0.001	0.007	0.000	0.007	0.073	1778.164	2.994	0.291	3.285	0.117	3.227	34.961	17782	29.940	2.910	32.850	1.170	32.270	349.610
120	original	0.6	60x8 weeks	Ag80	Daily	VAC95	25	Unvaccinate	3.705	0.006	0.001	0.007	0.000	0.007	0.073	1778.164	2.994	0.291	3.285	0.117	3.227	34.961	17782	29.940	2.910	32.850	1.170	32.270	349.610
120	original	1	60x8 weeks	Ag80	Daily	VAC95	25	Unvaccinate	3.701	0.006	0.001	0.007	0.000	0.007	0.072	1776.595	2.988	0.540	3.528	0.105	3.445	34.692	17766	29.880	5.400	35.280	1.050	34.450	346.920
120	original	1	60x8 weeks	Ag80	Daily	VAC95	25	Unvaccinate	3.701	0.006	0.001	0.007	0.000	0.007	0.072	1776.595	2.988	0.540	3.528	0.105	3.445	34.692	17766	29.880	5.400	35.280	1.050	34.450	346.920
120	original	1	60x8 weeks	Ag80	Daily	VAC95	25	Unvaccinate	3.701	0.006	0.001	0.007	0.000	0.007	0.072	1776.595	2.988	0.540	3.528	0.105	3.445	34.692	17766	29.880	5.400	35.280	1.050	34.450	346.920
120	original	1.2	60x8 weeks	Ag80	Daily	VAC95	25	Unvaccinate	3.699	0.006	0.001	0.008	0.000	0.008	0.073	1775.543	2.984	0.692	3.676	0.122	3.605	34.907	17755	29.840	6.920	36.760	1.220	36.050	349.070
120	original	1.2	60x8 weeks	Ag80	Daily	VAC95	25	Unvaccinate	3.699	0.006	0.001	0.008	0.000	0.008	0.073	1775.543	2.984	0.692	3.676	0.122	3.605	34.907	17755	29.840	6.920	36.760	1.220	36.050	349.070
120	original	1.2	60x8 weeks	Ag80	Daily	VAC95	25	Unvaccinate	3.699	0.006	0.001	0.008	0.000	0.008	0.073	1775.543	2.984	0.692	3.676	0.122	3.605	34.907	17755	29.840	6.920	36.760	1.220	36.050	349.070
120	alpha	0.6	60x8 weeks	Ag80	Daily	VAC95	25	Unvaccinate	3.700	0.006	0.001	0.007	0.000	0.007	0.072	1775.974	2.957	0.558	3.515	0.149	3.545	34.799	17760	29.570	5.580	35.150	1.490	35.450	347.990
120	alpha	0.6	60x8 weeks	Ag80	Daily	VAC95	25	Unvaccinate	3.700	0.006	0.001	0.007	0.000	0.007	0.072	1775.974	2.957	0.558	3.515	0.149	3.545	34.799	17760	29.570	5.580	35.150	1.490	35.450	347.990
120	alpha	0.6	60x8 weeks	Ag80	Daily	VAC95	25	Unvaccinate	3.700	0.006	0.001	0.007	0.000	0.007	0.072	1775.974	2.957	0.558	3.515	0.149	3.545	34.799	17760	29.570	5.580	35.150	1.490	35.450	347.990
120	alpha	1	60x8 weeks	Ag80	Daily	VAC95	25	Unvaccinate	3.695	0.006	0.002	0.008	0.000	0.008	0.072	1773.580	2.945	0.918	3.863	0.137	3.928	34.655	17736	29.450	9.180	38.630	1.370	39.280	346.550
120	alpha	1	60x8 weeks	Ag80	Daily	VAC95	25	Unvaccinate	3.695	0.006	0.002	0.008	0.000	0.008	0.072	1773.580	2.945	0.918	3.863	0.137	3.928	34.655	17736	29.450	9.180	38.630	1.370	39.280	346.550
120	alpha	1	60x8 weeks	Ag80	Daily	VAC95	25	Unvaccinate	3.695	0.006	0.002	0.008	0.000	0.008	0.072	1773.580	2.945	0.918	3.863	0.137	3.928	34.655	17736	29.450	9.180	38.630	1.370	39.280	346.550
120	alpha	1.2	60x8 weeks	Ag80	Daily	VAC95	25	Unvaccinate	3.689	0.006	0.003	0.009	0.000	0.009	0.072	1770.539	3.001	1.315	4.316	0.182	4.343	34.340	17705	30.010	13.150	43.160	1.820	43.430	343.400
120	alpha	1.2	60x8 weeks	Ag80	Daily	VAC95	25	Unvaccinate	3.689	0.006	0.003	0.009	0.000	0.009	0.072	1770.539	3.001	1.315	4.316	0.182	4.343	34.340	17705	30.010	13.150	43.160	1.820	43.430	343.400
120	alpha	1.2	60x8 weeks	Ag80	Daily	VAC95	25	Unvaccinate	3.689	0.006	0.003	0.009	0.000	0.009	0.072	1770.539	3.001	1.315	4.316	0.182	4.343	34.340	17705	30.010	13.150	43.160	1.820	43.430	343.400
120	delta	0.6	60x8 weeks	Ag80	Daily	VAC95	25	Unvaccinate	3.687	0.006	0.003	0.009	0.001	0.009	0.072	1769.844	3.004	1.511	4.515	0.267	4.393	34.359	17698	30.040	15.110	45.150	2.670	43.930	343.590
120	delta	0.6	60x8 weeks	Ag80	Daily	VAC95	25	Unvaccinate	3.687	0.006	0.003	0.009	0.001	0.009	0.072	1769.844	3.004	1.511	4.515	0.267	4.393	34.359	17698	30.040	15.110	45.150	2.670	43.930	343.590
120	delta	0.6	60x8 weeks	Ag80	Daily	VAC95	25	Unvaccinate	3.687	0.006	0.003	0.009	0.001	0.009	0.072	1769.844	3.004	1.511	4.515	0.267	4.393	34.359	17698	30.040	15.110	45.150	2.670	43.930	343.590
120	delta	1	60x8 weeks	Ag80	Daily	VAC95	25	Unvaccinate	3.665	0.006	0.006	0.013	0.001	0.012	0.070	1758.978	2.966	3.060	6.026	0.360	5.934	33.747	17590	29.660	30.600	60.260	3.600	59.340	337.470
120	delta	1	60x8 weeks	Ag80	Daily	VAC95	25	Unvaccinate	3.665	0.006	0.006	0.013	0.001	0.012	0.070	1758.978	2.966	3.060	6.026	0.360	5.934	33.747	17590	29.660	30.600	60.260	3.600	59.340	337.470
120	delta	1	60x8 weeks	Ag80	Daily	VAC95	25	Unvaccinate	3.665	0.006	0.006	0.013	0.001	0.012	0.070	1758.978	2.966	3.060	6.026	0.360	5.934	33.747	17590	29.660	30.600	60.260	3.600	59.340	337.470
120	delta	1.2	60x8 weeks	Ag80	Daily	VAC95	25	Unvaccinate	3.649	0.006	0.009	0.015	0.001	0.014	0.070	1751.538	2.894	4.111	7.005	0.398	6.934	33.679	17515	28.940	41.110	70.050	3.980	69.340	336.790
120	delta	1.2	60x8 weeks	Ag80	Daily	VAC95	25	Unvaccinate	3.649	0.006	0.009	0.015	0.001	0.014	0.070	1751.538	2.894	4.111	7.005	0.398	6.934	33.679	17515	28.940	41.110	70.050	3.980	69.340	336.790
120	delta	1.2	60x8 weeks	Ag80	Daily	VAC95	25	Unvaccinate	3.649	0.006	0.009	0.015	0.001	0.014	0.070	1751.538	2.894	4.111	7.005	0.398	6.934	33.679	17515	28.940	41.110	70.050	3.980	69.340	336.790
120	original	0.6	60x8 weeks	Ag80	Daily	VAC95	50	Unvaccinate	2.470	0.004	0.000	0.005	0.000	0.004	0.048	1185.556	2.154	0.130	2.284	0.107	2.145	23.247	11856	21.540	1.300	22.840	1.070	21.450	232.470
120	original	0.6	60x8 weeks	Ag80	Daily	VAC95	50	Unvaccinate	2.470	0.004	0.000	0.005	0.000	0.004	0.048	1185.556	2.154	0.130	2.284	0.107	2.145	23.247	11856	21.540	1.300	22.840	1.070	21.450	232.470
120	original	0.6	60x8 weeks	Ag80	Daily	VAC95	50	Unvaccinate	2.470	0.004	0.000	0.005	0.000	0.004	0.048	1185.556	2.154	0.130	2.284	0.107	2.145	23.247	11856	21.540	1.300	22.840	1.070	21.450	232.470
120	original	1	60x8 weeks	Ag80	Daily	VAC95	50	Unvaccinate	2.470	0.004	0.000	0.005	0.000	0.004	0.048	1185.799	2.047	0.227	2.274	0.106	2.122	23.003	11858	20.470	2.270	22.740	1.060	21.220	230.030
120	original	1	60x8 weeks	Ag80	Daily	VAC95	50	Unvaccinate	2.470	0.004	0.000	0.005	0.000	0.004	0.048	1185.799	2.047	0.227	2.274	0.106	2.122	23.003	11858	20.470	2.270	22.740	1.060	21.220	230.030
120	original	1	60x8 weeks	Ag80	Daily	VAC95	50	Unvaccinate	2.470	0.004	0.000	0.005	0.000	0.004	0.048	1185.799	2.047	0.227	2.274	0.106	2.122	23.003	11858	20.470	2.270	22.740	1.060	21.220	230.030
120	original	1.2	60x8 weeks	Ag80	Daily	VAC95	50	Unvaccinate	2.469	0.004	0.001	0.005	0.000	0.005	0.049	1185.326	2.080	0.274	2.354	0.125	2.174	23.395	11853	20.800	2.740	23.540	1.250	21.740	233.950
120	original	1.2	60x8 weeks	Ag80	Daily	VAC95	50	Unvaccinate	2.469	0.004	0.001	0.005	0.000	0.005	0.049	1185.326	2.080	0.274	2.354	0.125	2.174	23.395	11853	20.800	2.740	23.540	1.250	21.740	233.950
120	original	1.2	60x8 weeks	Ag80	Daily	VAC95	50	Unvaccinate	2.469	0.004	0.001	0.005	0.000	0.005	0.049	1185.326	2.080	0.274	2.354	0.125	2.174	23.395	11853	20.800	2.740	23.540	1.250	21.740	233.950
120	alpha	0.6	60x8 weeks	Ag80	Daily	VAC95	50	Unvaccinate	2.467	0.004	0.001	0.005	0.000	0.005	0.048	1184.337	2.120	0.283	2.403	0.148	2.319	22.942	11843	21.200	2.830	24.030	1.480	23.190	229.420
120	alpha	0.6	60x8 weeks	Ag80	Daily	VAC95	50	Unvaccinate	2.467	0.004	0.001	0.005	0.000	0.005	0.048	1184.337	2.120	0.283	2.403	0.148	2.319	22.942	11843	21.200	2.830	24.030	1.480	23.190	229.420
120	alpha	0.6	60x8 weeks	Ag80	Daily	VAC95	50	Unvaccinate	2.467	0.004	0.001	0.005	0.000	0.005	0.048	1184.337	2.120	0.283	2.403	0.148	2.319	22.942	11843	21.200	2.830	24.030	1.480	23.190	229.420
120	alpha	1	60x8 weeks	Ag80	Daily	VAC95	50	Unvaccinate	2.464	0.004	0.001	0.006	0.000	0.005	0.048	1182.796	2.143	0.524	2.667	0.174	2.519	22.944	11828	21.430	5.240	26.670	1.740	25.190	229.440
120	alpha	1	60x8 weeks	Ag80	Daily	VAC95	50	Unvaccinate	2.464	0.004	0.001	0.006	0.000	0.005	0.048	1182.796	2.143	0.524	2.667	0.174	2.519	22.944	11828	21.430	5.240	26.670	1.740	25.190	229.440
120	alpha	1	60x8 weeks	Ag80	Daily	VAC95	50	Unvaccinate	2.464	0.004	0.001	0.006	0.000	0.005	0.048	1182.796	2.143	0.524	2.667	0.174	2.519	22.944	11828	21.430	5.240	26.670	1.740	25.190	229.440
120	alpha	1.2	60x8 weeks	Ag80	Daily	VAC95	50	Unvaccinate	2.464	0.004	0.001	0.006	0.000	0.005	0.048	1182.600	2.068	0.668	2.736	0.167	2.577	23.213	11826	20.680	6.680	27.360	1.670	25.770	232.130
120	alpha	1.2	60x8 weeks	Ag80	Daily	VAC95	50	Unvaccinate	2.464	0.004	0.001	0.006	0.000	0.005	0.048	1182.600	2.068	0.668	2.736	0.167	2.577	23.213	11826	20.680	6.680	27.360	1.670	25.770	232.130
120	alpha	1.2	60x8 weeks	Ag80	Daily	VAC95	50	Unvaccinate	2.464	0.004	0.001	0.006	0.000	0.005	0.048	1182.600	2.068	0.668	2.736	0.167	2.577	23.213	11826	20.680	6.680	27.360	1.670	25.770	232.130

120	delta	0.6	60x8 weeks	Ag80	Daily	VAC95	50	Unvaccinate	2.462	0.004	0.002	0.006	0.001	0.006	0.048	1181.619	2.152	0.787	2.939	0.285	2.658	22.985	11816	21.520	7.870	29.390	2.850	26.580	229.850
120	delta	0.6	60x8 weeks	Ag80	Daily	VAC95	50	Unvaccinate	2.462	0.004	0.002	0.006	0.001	0.006	0.048	1181.619	2.152	0.787	2.939	0.285	2.658	22.985	11816	21.520	7.870	29.390	2.850	26.580	229.850
120	delta	0.6	60x8 weeks	Ag80	Daily	VAC95	50	Unvaccinate	2.462	0.004	0.002	0.006	0.001	0.006	0.048	1181.619	2.152	0.787	2.939	0.285	2.658	22.985	11816	21.520	7.870	29.390	2.850	26.580	229.850
120	delta	1	60x8 weeks	Ag80	Daily	VAC95	50	Unvaccinate	2.452	0.004	0.003	0.008	0.001	0.007	0.047	1176.734	2.145	1.616	3.761	0.379	3.394	22.576	11767	21.450	16.160	37.610	3.790	33.940	225.760
120	delta	1	60x8 weeks	Ag80	Daily	VAC95	50	Unvaccinate	2.452	0.004	0.003	0.008	0.001	0.007	0.047	1176.734	2.145	1.616	3.761	0.379	3.394	22.576	11767	21.450	16.160	37.610	3.790	33.940	225.760
120	delta	1	60x8 weeks	Ag80	Daily	VAC95	50	Unvaccinate	2.452	0.004	0.003	0.008	0.001	0.007	0.047	1176.734	2.145	1.616	3.761	0.379	3.394	22.576	11767	21.450	16.160	37.610	3.790	33.940	225.760
120	delta	1.2	60x8 weeks	Ag80	Daily	VAC95	50	Unvaccinate	2.444	0.004	0.005	0.009	0.001	0.008	0.048	1173.295	2.132	2.189	4.321	0.443	3.852	22.860	11733	21.320	21.890	43.210	4.430	38.520	228.600
120	delta	1.2	60x8 weeks	Ag80	Daily	VAC95	50	Unvaccinate	2.444	0.004	0.005	0.009	0.001	0.008	0.048	1173.295	2.132	2.189	4.321	0.443	3.852	22.860	11733	21.320	21.890	43.210	4.430	38.520	228.600
120	delta	1.2	60x8 weeks	Ag80	Daily	VAC95	50	Unvaccinate	2.444	0.004	0.005	0.009	0.001	0.008	0.048	1173.295	2.132	2.189	4.321	0.443	3.852	22.860	11733	21.320	21.890	43.210	4.430	38.520	228.600
120	original	0.6	60x8 weeks	Ag80	Daily	VAC95	75	Unvaccinate	1.236	0.003	0.000	0.003	0.000	0.002	0.024	593.455	1.207	0.033	1.240	0.119	0.978	11.522	5935	12.070	0.330	12.400	1.190	9.780	115.220
120	original	0.6	60x8 weeks	Ag80	Daily	VAC95	75	Unvaccinate	1.236	0.003	0.000	0.003	0.000	0.002	0.024	593.455	1.207	0.033	1.240	0.119	0.978	11.522	5935	12.070	0.330	12.400	1.190	9.780	115.220
120	original	0.6	60x8 weeks	Ag80	Daily	VAC95	75	Unvaccinate	1.236	0.003	0.000	0.003	0.000	0.002	0.024	593.455	1.207	0.033	1.240	0.119	0.978	11.522	5935	12.070	0.330	12.400	1.190	9.780	115.220
120	original	1	60x8 weeks	Ag80	Daily	VAC95	75	Unvaccinate	1.234	0.003	0.000	0.003	0.000	0.002	0.024	592.536	1.261	0.058	1.319	0.106	1.107	11.599	5925	12.610	0.580	13.190	1.060	11.070	115.990
120	original	1	60x8 weeks	Ag80	Daily	VAC95	75	Unvaccinate	1.234	0.003	0.000	0.003	0.000	0.002	0.024	592.536	1.261	0.058	1.319	0.106	1.107	11.599	5925	12.610	0.580	13.190	1.060	11.070	115.990
120	original	1	60x8 weeks	Ag80	Daily	VAC95	75	Unvaccinate	1.234	0.003	0.000	0.003	0.000	0.002	0.024	592.536	1.261	0.058	1.319	0.106	1.107	11.599	5925	12.610	0.580	13.190	1.060	11.070	115.990
120	original	1.2	60x8 weeks	Ag80	Daily	VAC95	75	Unvaccinate	1.236	0.002	0.000	0.003	0.000	0.002	0.024	593.225	1.177	0.064	1.241	0.108	0.996	11.647	5932	11.770	0.640	12.410	1.080	9.960	116.470
120	original	1.2	60x8 weeks	Ag80	Daily	VAC95	75	Unvaccinate	1.236	0.002	0.000	0.003	0.000	0.002	0.024	593.225	1.177	0.064	1.241	0.108	0.996	11.647	5932	11.770	0.640	12.410	1.080	9.960	116.470
120	original	1.2	60x8 weeks	Ag80	Daily	VAC95	75	Unvaccinate	1.236	0.002	0.000	0.003	0.000	0.002	0.024	593.225	1.177	0.064	1.241	0.108	0.996	11.647	5932	11.770	0.640	12.410	1.080	9.960	116.470
120	alpha	0.6	60x8 weeks	Ag80	Daily	VAC95	75	Unvaccinate	1.235	0.003	0.000	0.003	0.000	0.002	0.024	592.697	1.256	0.123	1.379	0.179	1.073	11.517	5927	12.560	1.230	13.790	1.790	10.730	115.170
120	alpha	0.6	60x8 weeks	Ag80	Daily	VAC95	75	Unvaccinate	1.235	0.003	0.000	0.003	0.000	0.002	0.024	592.697	1.256	0.123	1.379	0.179	1.073	11.517	5927	12.560	1.230	13.790	1.790	10.730	115.170
120	alpha	0.6	60x8 weeks	Ag80	Daily	VAC95	75	Unvaccinate	1.235	0.003	0.000	0.003	0.000	0.002	0.024	592.697	1.256	0.123	1.379	0.179	1.073	11.517	5927	12.560	1.230	13.790	1.790	10.730	115.170
120	alpha	1	60x8 weeks	Ag80	Daily	VAC95	75	Unvaccinate	1.233	0.003	0.000	0.003	0.000	0.003	0.024	591.636	1.266	0.228	1.494	0.186	1.232	11.380	5916	12.660	2.280	14.940	1.860	12.320	113.800
120	alpha	1	60x8 weeks	Ag80	Daily	VAC95	75	Unvaccinate	1.233	0.003	0.000	0.003	0.000	0.003	0.024	591.636	1.266	0.228	1.494	0.186	1.232	11.380	5916	12.660	2.280	14.940	1.860	12.320	113.800
120	alpha	1	60x8 weeks	Ag80	Daily	VAC95	75	Unvaccinate	1.233	0.003	0.000	0.003	0.000	0.003	0.024	591.636	1.266	0.228	1.494	0.186	1.232	11.380	5916	12.660	2.280	14.940	1.860	12.320	113.800
120	alpha	1.2	60x8 weeks	Ag80	Daily	VAC95	75	Unvaccinate	1.233	0.003	0.000	0.003	0.000	0.002	0.024	591.999	1.229	0.224	1.453	0.178	1.174	11.579	5920	12.290	2.240	14.530	1.780	11.740	115.790
120	alpha	1.2	60x8 weeks	Ag80	Daily	VAC95	75	Unvaccinate	1.233	0.003	0.000	0.003	0.000	0.002	0.024	591.999	1.229	0.224	1.453	0.178	1.174	11.579	5920	12.290	2.240	14.530	1.780	11.740	115.790
120	alpha	1.2	60x8 weeks	Ag80	Daily	VAC95	75	Unvaccinate	1.233	0.003	0.000	0.003	0.000	0.002	0.024	591.999	1.229	0.224	1.453	0.178	1.174	11.579	5920	12.290	2.240	14.530	1.780	11.740	115.790
120	delta	0.6	60x8 weeks	Ag80	Daily	VAC95	75	Unvaccinate	1.233	0.003	0.001	0.003	0.001	0.002	0.024	591.989	1.314	0.330	1.644	0.322	1.150	11.563	5920	13.140	3.300	16.440	3.220	11.500	115.630
120	delta	0.6	60x8 weeks	Ag80	Daily	VAC95	75	Unvaccinate	1.233	0.003	0.001	0.003	0.001	0.002	0.024	591.989	1.314	0.330	1.644	0.322	1.150	11.563	5920	13.140	3.300	16.440	3.220	11.500	115.630
120	delta	0.6	60x8 weeks	Ag80	Daily	VAC95	75	Unvaccinate	1.233	0.003	0.001	0.003	0.001	0.002	0.024	591.989	1.314	0.330	1.644	0.322	1.150	11.563	5920	13.140	3.300	16.440	3.220	11.500	115.630
120	delta	1	60x8 weeks	Ag80	Daily	VAC95	75	Unvaccinate	1.229	0.003	0.002	0.004	0.001	0.003	0.024	589.728	1.367	0.776	2.143	0.383	1.509	11.536	5897	13.670	7.760	21.430	3.830	15.090	115.360
120	delta	1	60x8 weeks	Ag80	Daily	VAC95	75	Unvaccinate	1.229	0.003	0.002	0.004	0.001	0.003	0.024	589.728	1.367	0.776	2.143	0.383	1.509	11.536	5897	13.670	7.760	21.430	3.830	15.090	115.360
120	delta	1	60x8 weeks	Ag80	Daily	VAC95	75	Unvaccinate	1.229	0.003	0.002	0.004	0.001	0.003	0.024	589.728	1.367	0.776	2.143	0.383	1.509	11.536	5897	13.670	7.760	21.430	3.830	15.090	115.360
120	delta	1.2	60x8 weeks	Ag80	Daily	VAC95	75	Unvaccinate	1.227	0.003	0.002	0.005	0.001	0.003	0.024	588.857	1.391	0.938	2.329	0.461	1.641	11.305	5889	13.910	9.380	23.290	4.610	16.410	113.050
120	delta	1.2	60x8 weeks	Ag80	Daily	VAC95	75	Unvaccinate	1.227	0.003	0.002	0.005	0.001	0.003	0.024	588.857	1.391	0.938	2.329	0.461	1.641	11.305	5889	13.910	9.380	23.290	4.610	16.410	113.050
120	delta	1.2	60x8 weeks	Ag80	Daily	VAC95	75	Unvaccinate	1.227	0.003	0.002	0.005	0.001	0.003	0.024	588.857	1.391	0.938	2.329	0.461	1.641	11.305	5889	13.910	9.380	23.290	4.610	16.410	113.050
120	original	0.6	60x8 weeks	Ag80	Daily	VAC95	100	Unvaccinate	0.000	0.001	0.000	0.001	0.000	0.000	0.000	0.000	0.282	0.000	0.282	0.112	0.000	0.000	0	2.820	0.000	2.820	1.120	0.000	0.000
120	original	0.6	60x8 weeks	Ag80	Daily	VAC95	100	Unvaccinate	0.000	0.001	0.000	0.001	0.000	0.000	0.000	0.000	0.282	0.000	0.282	0.112	0.000	0.000	0	2.820	0.000	2.820	1.120	0.000	0.000
120	original	0.6	60x8 weeks	Ag80	Daily	VAC95	100	Unvaccinate	0.000	0.001	0.000	0.001	0.000	0.000	0.000	0.000	0.282	0.000	0.282	0.112	0.000	0.000	0	2.820	0.000	2.820	1.120	0.000	0.000
120	original	1	60x8 weeks	Ag80	Daily	VAC95	100	Unvaccinate	0.000	0.001	0.000	0.001	0.000	0.000	0.000	0.000	0.292	0.000	0.292	0.109	0.000	0.000	0	2.920	0.000	2.920	1.090	0.000	0.000
120	original	1	60x8 weeks	Ag80	Daily	VAC95	100	Unvaccinate	0.000	0.001	0.000	0.001	0.000	0.000	0.000	0.000	0.292	0.000	0.292	0.109	0.000	0.000	0	2.920	0.000	2.920	1.090	0.000	0.000
120	original	1	60x8 weeks	Ag80	Daily	VAC95	100	Unvaccinate	0.000	0.001	0.000	0.001	0.000	0.000	0.000	0.000	0.292	0.000	0.292	0.109	0.000	0.000	0	2.920	0.000	2.920	1.090	0.000	0.000
120	original	1.2	60x8 weeks	Ag80	Daily	VAC95	100	Unvaccinate	0.000	0.001	0.000	0.001	0.000	0.000	0.000	0.000	0.279	0.000	0.279	0.105	0.000	0.000	0	2.790	0.000	2.790	1.050	0.000	0.000
120	original	1.2	60x8 weeks	Ag80	Daily	VAC95	100	Unvaccinate	0.000	0.001	0.000	0.001	0.000	0.000	0.000	0.000	0.279	0.000	0.279	0.105	0.000	0.000	0	2.790	0.000	2.790	1.050	0.000	0.000
120	original	1.2	60x8 weeks	Ag80	Daily	VAC95	100	Unvaccinate	0.000	0.001	0.000	0.001	0.000	0.000	0.000	0.000	0.279	0.000	0.279	0.105	0.000	0.000	0	2.790	0.000	2.790	1.050	0.000	0.000
120	alpha	0.6	60x8 weeks	Ag80	Daily	VAC95	100	Unvaccinate	0.000	0.001	0.000	0.001	0.000	0.000	0.000	0.000	0.360	0.024	0.384	0.178	0.000	0.000	0	3.600	0.240	3.840	1.780	0.000	0.000
120	alpha	0.6	60x8 weeks	Ag80	Daily	VAC95	100	Unvaccinate	0.000	0.001	0.000	0.001	0.000	0.000	0.000	0.000	0.360	0.024	0.384	0.178	0.000	0.000	0	3.600	0.240	3.840	1.780	0.000	0.000
120	alpha	0.6	60x8 weeks	Ag80	Daily	VAC95	100	Unvaccinate	0.000	0.001	0.000	0.001	0.000	0.000	0.000	0.000	0.360	0.024	0.384	0.178	0.000	0.000	0	3.600	0.240	3.840	1.780	0.000	0.000
120	alpha	1	60x8 weeks	Ag80	Daily	VAC95	100	Unvaccinate	0.000	0.001	0.000	0.001	0.000	0.000	0.000	0.000	0.391	0.028	0.419	0.203	0.000	0.000	0	3.910	0.280	4.190	2.030	0.000	0.000
120	alpha	1	60x8 weeks	Ag80	Daily	VAC95	100	Unvaccinate	0.000	0.001	0.000	0.001	0.000	0.000	0.000	0.000	0.391	0.028	0.419	0.203	0.000	0.000	0	3.910	0.280	4.190	2.030	0.000	0.000
120	alpha	1	60x8 weeks	Ag80	Daily	VAC95	100	Unvaccinate	0.000	0.001	0.000	0.001	0.000	0.000	0.000	0.000	0.391	0.028	0.419	0.203	0.000	0.000	0	3.910	0.280	4.190	2.030	0.000	0.000
120	alpha	1.2	60x8 weeks	Ag80	Daily	VAC95	100	Unvaccinate	0.000	0.001	0.000	0.001	0.000	0.000	0.000	0.000	0.380	0.048	0.428	0.191	0.000	0.000	0	3.800	0.480	4.280	1.910	0.000	0.000
120	alpha	1.2	60x8 weeks	Ag80	Daily	VAC95	100	Unvaccinate	0.000	0.001	0.000	0.001	0.000	0.000	0.000	0.000	0.380	0.048	0.428	0.191	0.000	0.000	0	3.800	0.480	4.280	1.910	0.000	0.000
120	alpha	1.2	60x8 weeks	Ag80	Daily	VAC95	100	Unvaccinate	0.000	0.001	0.000	0.001	0.000	0.000	0.000	0.000	0.380	0.048	0.428	0.191	0.000	0.000	0	3.800	0.480	4.280	1.910	0.000	0.000
120	delta	0.6	60x8 weeks	Ag80	Daily	VAC95	100	Unvaccinate	0.000	0.001	0.000	0.001	0.001	0.000	0.000	0.000	0.562	0.113	0.675	0.342	0.000	0.000	0	5.620	1.130	6.750	3.420	0.000	0.000
120	delta	0.6	60x8 weeks	Ag80	Daily	VAC95	100	Unvaccinate	0.000	0.001	0.000	0.001	0.001	0.000	0.000	0.000	0.562	0.113	0.675	0.342	0.000	0.000	0	5.620	1.130	6.750	3.420	0.000	0.000
120	delta	0.6	60x8 weeks	Ag80	Daily	VAC95	100	Unvaccinate	0.000	0.001	0.000	0.001	0.001	0.000	0.000	0.000	0.562	0.113	0.675	0.342	0.000	0.000	0	5.620	1.130	6.750	3.420	0.000	0.000
120	delta	1	60x8 weeks	Ag80	Daily	VAC95	100	Unvaccinate	0.000	0.001	0.000	0.002	0.001	0.000	0.000	0.000	0.574	0.229	0.803	0.449	0.000	0.000	0	5.740	2.290	8.030	4.490	0.000	0.000
120	delta	1	60x8 weeks	Ag80	Daily	VAC95	100	Unvaccinate	0.000	0.001	0.000	0.002	0.001	0.000	0.000	0.000	0.574	0.229	0.803	0.449	0.000	0.000	0	5.740	2.290	8.030	4.490	0.000	0.000
120	delta	1	60x8 weeks	Ag80	Daily	VAC95	100	Unvaccinate	0.000	0.001	0.000	0.002	0.001	0.000	0.000	0.000	0.574	0.229	0.803	0.449	0.000	0.000	0	5.740	2.290	8.030	4.490	0.000	0.000
120	delta	1.2	60x8 weeks	Ag80	Daily	VAC95	100	Unvaccinate	0.000	0.001	0.001	0.002	0.001	0.000	0.000	0.000	0.521	0.243	0.764	0.435	0.000	0.000	0	5.210	2.430	7.640	4.350	0.000	0.000
120	delta	1.2	60x8 weeks	Ag80	Daily	VAC95	100	Unvaccinate	0.000	0.001	0.001	0.002	0.001	0.000	0.000	0.000	0.521	0.243	0.764	0.435	0.000	0.000	0	5.210	2.430	7.640	4.350	0.000	0.000
120	delta	1.2	60x8 weeks	Ag80	Daily	VAC95	100	Unvaccinate	0.000	0.001	0.001	0.002	0.001	0.000	0.000	0.000	0.521	0.243	0.764	0.435	0.000	0.000	0	5.210	2.430	7.640	4.350	0.000	0.000
120	original	0.6	60x8 weeks	Ag80	Daily	VAC70	25	Unvaccinate	3.704	0.007	0.001	0.008	0.001	0.007	0.073	1777.926	3.322	0.313	3.635	0.254	3.258	34.815	17779	33.220	3.130	36.350	2.540	32.580	348.150
120	original	0.6	60x8 weeks	Ag80	Daily	VAC70	25	Unvaccinate	3.704	0.007	0.001	0.008	0.001	0.007	0.073	1777.926	3.322	0.313	3.635	0.254	3.258	34.815	17779	33.220	3.130	36.350	2.540	32.580	348.150
120	original	0.6	60x8 weeks	Ag80	Daily	VAC70	25	Unvaccinate	3.704	0.007	0.001	0.008	0.001	0.007	0.073	1777.926	3.322	0.313	3.635	0.254	3.258	34.815	17779	33.220	3.130	36.350	2.540	32.580	348.150
120	original	1	60x8 weeks	Ag80	Daily	VAC70	25	Unvaccinate	3.703	0.007	0.001	0.008	0.001	0.007	0.072	1777.367	3.165	0.618	3.783	0.294	3.352	34.502	17774	31.650	6.180	37.830	2.940	33.520	345.020
120	original	1	60x8 weeks	Ag80	Daily	VAC70	25	Unvaccinate	3.703	0.007	0.001	0.008	0.001	0.007	0.072	1777.367	3.165	0.618	3.783	0.294	3.352	34.502	17774	31.650	6.180	37.830	2.940	33.520	345.020
120	original	1	60x8 weeks	Ag80	Daily	VAC70	25	Unvaccinate	3.703	0.007	0.001	0.008	0.001	0.007	0.072	1777.367	3.165	0.618	3.783	0.294	3.352	34.502	17774	31.650	6.180	37.830	2.940	33.520	345.020
120	original	1.2	60x8 weeks	Ag80	Daily	VAC70	25	Unvaccinate	3.701	0.007	0.001	0.008	0.001	0.007	0.073	1776.388	3.192	0.699	3.891	0.249	3.523	34.995	17764	31.920	6.990	38.910	2.490	35.230	349.950
120	original	1.2	60x8 weeks	Ag80	Daily	VAC70	25	Unvaccinate	3.701	0.007	0.001	0.008	0.001	0.007	0.073	1776.388	3.192	0.699	3.891	0.249	3.523	34.995	17764	31.920	6.990	38.910	2.490	35.230	349.950

120	original	1.2	60x8 weeks	Ag80	Daily	VAC70	25	Invaccinate	3.701	0.007	0.001	0.008	0.001	0.007	0.073	1776.388	3.192	0.699	3.891	0.249	3.523	34.995	17764	31.920	6.990	38.910	2.490	35.230	349.950
120	alpha	0.6	60x8 weeks	Ag80	Daily	VAC70	25	Invaccinate	3.697	0.007	0.002	0.008	0.001	0.008	0.072	1774.724	3.241	0.782	4.023	0.307	3.757	34.506	17747	32.410	7.820	40.230	3.070	37.570	345.060
120	alpha	0.6	60x8 weeks	Ag80	Daily	VAC70	25	Invaccinate	3.697	0.007	0.002	0.008	0.001	0.008	0.072	1774.724	3.241	0.782	4.023	0.307	3.757	34.506	17747	32.410	7.820	40.230	3.070	37.570	345.060
120	alpha	0.6	60x8 weeks	Ag80	Daily	VAC70	25	Invaccinate	3.697	0.007	0.002	0.008	0.001	0.008	0.072	1774.724	3.241	0.782	4.023	0.307	3.757	34.506	17747	32.410	7.820	40.230	3.070	37.570	345.060
120	alpha	1	60x8 weeks	Ag80	Daily	VAC70	25	Invaccinate	3.690	0.007	0.003	0.009	0.001	0.009	0.072	1771.063	3.207	1.330	4.537	0.362	4.279	34.455	17711	32.070	13.300	45.370	3.620	42.790	344.550
120	alpha	1	60x8 weeks	Ag80	Daily	VAC70	25	Invaccinate	3.690	0.007	0.003	0.009	0.001	0.009	0.072	1771.063	3.207	1.330	4.537	0.362	4.279	34.455	17711	32.070	13.300	45.370	3.620	42.790	344.550
120	alpha	1	60x8 weeks	Ag80	Daily	VAC70	25	Invaccinate	3.690	0.007	0.003	0.009	0.001	0.009	0.072	1771.063	3.207	1.330	4.537	0.362	4.279	34.455	17711	32.070	13.300	45.370	3.620	42.790	344.550
120	alpha	1.2	60x8 weeks	Ag80	Daily	VAC70	25	Invaccinate	3.684	0.007	0.004	0.010	0.001	0.010	0.071	1768.262	3.151	1.797	4.948	0.385	4.677	34.032	17683	31.510	17.970	49.480	3.850	46.770	340.320
120	alpha	1.2	60x8 weeks	Ag80	Daily	VAC70	25	Invaccinate	3.684	0.007	0.004	0.010	0.001	0.010	0.071	1768.262	3.151	1.797	4.948	0.385	4.677	34.032	17683	31.510	17.970	49.480	3.850	46.770	340.320
120	alpha	1.2	60x8 weeks	Ag80	Daily	VAC70	25	Invaccinate	3.684	0.007	0.004	0.010	0.001	0.010	0.071	1768.262	3.151	1.797	4.948	0.385	4.677	34.032	17683	31.510	17.970	49.480	3.850	46.770	340.320
120	delta	0.6	60x8 weeks	Ag80	Daily	VAC70	25	Invaccinate	3.678	0.007	0.005	0.012	0.001	0.010	0.071	1765.363	3.316	2.258	5.574	0.618	5.014	34.250	17654	33.160	22.580	55.740	6.180	50.140	342.500
120	delta	0.6	60x8 weeks	Ag80	Daily	VAC70	25	Invaccinate	3.678	0.007	0.005	0.012	0.001	0.010	0.071	1765.363	3.316	2.258	5.574	0.618	5.014	34.250	17654	33.160	22.580	55.740	6.180	50.140	342.500
120	delta	0.6	60x8 weeks	Ag80	Daily	VAC70	25	Invaccinate	3.678	0.007	0.005	0.012	0.001	0.010	0.071	1765.363	3.316	2.258	5.574	0.618	5.014	34.250	17654	33.160	22.580	55.740	6.180	50.140	342.500
120	delta	1	60x8 weeks	Ag80	Daily	VAC70	25	Invaccinate	3.637	0.007	0.011	0.018	0.002	0.016	0.069	1745.762	3.175	5.518	8.693	0.915	7.856	32.921	17458	31.750	55.180	86.930	9.150	78.560	329.210
120	delta	1	60x8 weeks	Ag80	Daily	VAC70	25	Invaccinate	3.637	0.007	0.011	0.018	0.002	0.016	0.069	1745.762	3.175	5.518	8.693	0.915	7.856	32.921	17458	31.750	55.180	86.930	9.150	78.560	329.210
120	delta	1	60x8 weeks	Ag80	Daily	VAC70	25	Invaccinate	3.637	0.007	0.011	0.018	0.002	0.016	0.069	1745.762	3.175	5.518	8.693	0.915	7.856	32.921	17458	31.750	55.180	86.930	9.150	78.560	329.210
120	delta	1.2	60x8 weeks	Ag80	Daily	VAC70	25	Invaccinate	3.602	0.007	0.017	0.024	0.003	0.021	0.067	1728.810	3.242	8.095	11.337	1.261	10.251	31.933	17288	32.420	80.950	113.370	12.610	102.510	319.330
120	delta	1.2	60x8 weeks	Ag80	Daily	VAC70	25	Invaccinate	3.602	0.007	0.017	0.024	0.003	0.021	0.067	1728.810	3.242	8.095	11.337	1.261	10.251	31.933	17288	32.420	80.950	113.370	12.610	102.510	319.330
120	delta	1.2	60x8 weeks	Ag80	Daily	VAC70	25	Invaccinate	3.602	0.007	0.017	0.024	0.003	0.021	0.067	1728.810	3.242	8.095	11.337	1.261	10.251	31.933	17288	32.420	80.950	113.370	12.610	102.510	319.330
120	original	0.6	60x8 weeks	Ag80	Daily	VAC70	50	Invaccinate	2.470	0.006	0.000	0.006	0.001	0.004	0.048	1185.718	2.655	0.191	2.846	0.419	2.114	22.858	11857	26.550	1.910	28.460	4.190	21.140	228.580
120	original	0.6	60x8 weeks	Ag80	Daily	VAC70	50	Invaccinate	2.470	0.006	0.000	0.006	0.001	0.004	0.048	1185.718	2.655	0.191	2.846	0.419	2.114	22.858	11857	26.550	1.910	28.460	4.190	21.140	228.580
120	original	0.6	60x8 weeks	Ag80	Daily	VAC70	50	Invaccinate	2.470	0.006	0.000	0.006	0.001	0.004	0.048	1185.718	2.655	0.191	2.846	0.419	2.114	22.858	11857	26.550	1.910	28.460	4.190	21.140	228.580
120	original	1	60x8 weeks	Ag80	Daily	VAC70	50	Invaccinate	2.470	0.005	0.001	0.006	0.001	0.004	0.048	1185.814	2.541	0.309	2.850	0.422	2.118	23.109	11858	25.410	3.090	28.500	4.220	21.180	231.090
120	original	1	60x8 weeks	Ag80	Daily	VAC70	50	Invaccinate	2.470	0.005	0.001	0.006	0.001	0.004	0.048	1185.814	2.541	0.309	2.850	0.422	2.118	23.109	11858	25.410	3.090	28.500	4.220	21.180	231.090
120	original	1	60x8 weeks	Ag80	Daily	VAC70	50	Invaccinate	2.470	0.005	0.001	0.006	0.001	0.004	0.048	1185.814	2.541	0.309	2.850	0.422	2.118	23.109	11858	25.410	3.090	28.500	4.220	21.180	231.090
120	original	1.2	60x8 weeks	Ag80	Daily	VAC70	50	Invaccinate	2.468	0.006	0.001	0.006	0.001	0.005	0.048	1184.470	2.645	0.401	3.046	0.458	2.281	23.108	11845	26.450	4.010	30.460	4.580	22.810	231.080
120	original	1.2	60x8 weeks	Ag80	Daily	VAC70	50	Invaccinate	2.468	0.006	0.001	0.006	0.001	0.005	0.048	1184.470	2.645	0.401	3.046	0.458	2.281	23.108	11845	26.450	4.010	30.460	4.580	22.810	231.080
120	original	1.2	60x8 weeks	Ag80	Daily	VAC70	50	Invaccinate	2.468	0.006	0.001	0.006	0.001	0.005	0.048	1184.470	2.645	0.401	3.046	0.458	2.281	23.108	11845	26.450	4.010	30.460	4.580	22.810	231.080
120	alpha	0.6	60x8 weeks	Ag80	Daily	VAC70	50	Invaccinate	2.464	0.005	0.001	0.007	0.001	0.005	0.048	1182.924	2.580	0.622	3.202	0.550	2.515	23.161	11829	25.800	6.220	32.020	5.500	25.150	231.610
120	alpha	0.6	60x8 weeks	Ag80	Daily	VAC70	50	Invaccinate	2.464	0.005	0.001	0.007	0.001	0.005	0.048	1182.924	2.580	0.622	3.202	0.550	2.515	23.161	11829	25.800	6.220	32.020	5.500	25.150	231.610
120	alpha	0.6	60x8 weeks	Ag80	Daily	VAC70	50	Invaccinate	2.464	0.005	0.001	0.007	0.001	0.005	0.048	1182.924	2.580	0.622	3.202	0.550	2.515	23.161	11829	25.800	6.220	32.020	5.500	25.150	231.610
120	alpha	1	60x8 weeks	Ag80	Daily	VAC70	50	Invaccinate	2.460	0.005	0.002	0.008	0.001	0.006	0.048	1180.700	2.538	1.074	3.612	0.593	2.824	22.822	11807	25.380	10.740	36.120	5.930	28.240	228.220
120	alpha	1	60x8 weeks	Ag80	Daily	VAC70	50	Invaccinate	2.460	0.005	0.002	0.008	0.001	0.006	0.048	1180.700	2.538	1.074	3.612	0.593	2.824	22.822	11807	25.380	10.740	36.120	5.930	28.240	228.220
120	alpha	1	60x8 weeks	Ag80	Daily	VAC70	50	Invaccinate	2.460	0.005	0.002	0.008	0.001	0.006	0.048	1180.700	2.538	1.074	3.612	0.593	2.824	22.822	11807	25.380	10.740	36.120	5.930	28.240	228.220
120	alpha	1.2	60x8 weeks	Ag80	Daily	VAC70	50	Invaccinate	2.455	0.005	0.003	0.009	0.001	0.007	0.048	1178.359	2.602	1.503	4.105	0.705	3.189	22.853	11784	26.020	15.030	41.050	7.050	31.890	228.530
120	alpha	1.2	60x8 weeks	Ag80	Daily	VAC70	50	Invaccinate	2.455	0.005	0.003	0.009	0.001	0.007	0.048	1178.359	2.602	1.503	4.105	0.705	3.189	22.853	11784	26.020	15.030	41.050	7.050	31.890	228.530
120	alpha	1.2	60x8 weeks	Ag80	Daily	VAC70	50	Invaccinate	2.455	0.005	0.003	0.009	0.001	0.007	0.048	1178.359	2.602	1.503	4.105	0.705	3.189	22.853	11784	26.020	15.030	41.050	7.050	31.890	228.530
120	delta	0.6	60x8 weeks	Ag80	Daily	VAC70	50	Invaccinate	2.451	0.006	0.004	0.010	0.002	0.007	0.047	1176.396	2.758	2.010	4.768	0.972	3.435	22.672	11764	27.580	20.100	47.680	9.720	34.350	226.720
120	delta	0.6	60x8 weeks	Ag80	Daily	VAC70	50	Invaccinate	2.451	0.006	0.004	0.010	0.002	0.007	0.047	1176.396	2.758	2.010	4.768	0.972	3.435	22.672	11764	27.580	20.100	47.680	9.720	34.350	226.720
120	delta	0.6	60x8 weeks	Ag80	Daily	VAC70	50	Invaccinate	2.451	0.006	0.004	0.010	0.002	0.007	0.047	1176.396	2.758	2.010	4.768	0.972	3.435	22.672	11764	27.580	20.100	47.680	9.720	34.350	226.720
120	delta	1	60x8 weeks	Ag80	Daily	VAC70	50	Invaccinate	2.420	0.006	0.011	0.016	0.003	0.012	0.046	1161.731	2.675	5.081	7.756	1.674	5.571	21.909	11617	26.750	50.810	77.560	16.740	55.710	219.090
120	delta	1	60x8 weeks	Ag80	Daily	VAC70	50	Invaccinate	2.420	0.006	0.011	0.016	0.003	0.012	0.046	1161.731	2.675	5.081	7.756	1.674	5.571	21.909	11617	26.750	50.810	77.560	16.740	55.710	219.090
120	delta	1	60x8 weeks	Ag80	Daily	VAC70	50	Invaccinate	2.420	0.006	0.011	0.016	0.003	0.012	0.046	1161.731	2.675	5.081	7.756	1.674	5.571	21.909	11617	26.750	50.810	77.560	16.740	55.710	219.090
120	delta	1.2	60x8 weeks	Ag80	Daily	VAC70	50	Invaccinate	2.394	0.005	0.017	0.022	0.005	0.015	0.044	1149.222	2.504	8.182	10.686	2.391	7.394	21.312	11492	25.040	81.820	106.860	23.910	73.940	213.120
120	delta	1.2	60x8 weeks	Ag80	Daily	VAC70	50	Invaccinate	2.394	0.005	0.017	0.022	0.005	0.015	0.044	1149.222	2.504	8.182	10.686	2.391	7.394	21.312	11492	25.040	81.820	106.860	23.910	73.940	213.120
120	delta	1.2	60x8 weeks	Ag80	Daily	VAC70	50	Invaccinate	2.394	0.005	0.017	0.022	0.005	0.015	0.044	1149.222	2.504	8.182	10.686	2.391	7.394	21.312	11492	25.040	81.820	106.860	23.910	73.940	213.120
120	original	0.6	60x8 weeks	Ag80	Daily	VAC70	75	Invaccinate	1.236	0.004	0.000	0.004	0.001	0.002	0.025	593.280	1.943	0.094	2.037	0.526	0.985	11.762	5933	19.430	0.940	20.370	5.260	9.850	117.620
120	original	0.6	60x8 weeks	Ag80	Daily	VAC70	75	Invaccinate	1.236	0.004	0.000	0.004	0.001	0.002	0.025	593.280	1.943	0.094	2.037	0.526	0.985	11.762	5933	19.430	0.940	20.370	5.260	9.850	117.620
120	original	0.6	60x8 weeks	Ag80	Daily	VAC70	75	Invaccinate	1.236	0.004	0.000	0.004	0.001	0.002	0.025	593.280	1.943	0.094	2.037	0.526	0.985	11.762	5933	19.430	0.940	20.370	5.260	9.850	117.620
120	original	1	60x8 weeks	Ag80	Daily	VAC70	75	Invaccinate	1.236	0.004	0.000	0.004	0.001	0.002	0.024	593.180	1.904	0.127	2.031	0.520	1.018	11.469	5932	19.040	1.270	20.310	5.200	10.180	114.690
120	original	1	60x8 weeks	Ag80	Daily	VAC70	75	Invaccinate	1.236	0.004	0.000	0.004	0.001	0.002	0.024	593.180	1.904	0.127	2.031	0.520	1.018	11.469	5932	19.040	1.270	20.310	5.200	10.180	114.690
120	original	1	60x8 weeks	Ag80	Daily	VAC70	75	Invaccinate	1.236	0.004	0.000	0.004	0.001	0.002	0.024	593.180	1.904	0.127	2.031	0.520	1.018	11.469	5932	19.040	1.270	20.310	5.200	10.180	114.690
120	original	1.2	60x8 weeks	Ag80	Daily	VAC70	75	Invaccinate	1.235	0.004	0.000	0.005	0.001	0.002	0.024	592.974	2.046	0.161	2.207	0.595	1.052	11.646	5930	20.460	1.610	22.070	5.950	10.520	116.460
120	original	1.2	60x8 weeks	Ag80	Daily	VAC70	75	Invaccinate	1.235	0.004	0.000	0.005	0.001	0.002	0.024	592.974	2.046	0.161	2.207	0.595	1.052	11.646	5930	20.460	1.610	22.070	5.950	10.520	116.460
120	original	1.2	60x8 weeks	Ag80	Daily	VAC70	75	Invaccinate	1.235	0.004	0.000	0.005	0.001	0.002	0.024	592.974	2.046	0.161	2.207	0.595	1.052	11.646	5930	20.460	1.610	22.070	5.950	10.520	116.460
120	alpha	0.6	60x8 weeks	Ag80	Daily	VAC70	75	Invaccinate	1.233	0.004	0.001	0.005	0.001	0.003	0.024	591.824	1.954	0.401	2.355	0.692	1.215	11.549	5918	19.540	4.010	23.550	6.920	12.150	115.490
120	alpha	0.6	60x8 weeks	Ag80	Daily	VAC70	75	Invaccinate	1.233	0.004	0.001	0.005	0.001	0.003	0.024	591.824	1.954	0.401	2.355	0.692	1.215	11.549	5918	19.540	4.010	23.550	6.920	12.150	115.490
120	alpha	0.6	60x8 weeks	Ag80	Daily	VAC70	75	Invaccinate	1.233	0.004	0.001	0.005	0.001	0.003	0.024	591.824	1.954	0.401	2.355	0.692	1.215	11.549	5918	19.540	4.010	23.550	6.920	12.150	115.490
120	alpha	1	60x8 weeks	Ag80	Daily	VAC70	75	Invaccinate	1.229	0.004	0.002	0.006	0.002	0.003	0.024	590.044	1.976	0.837	2.813	0.877	1.477	11.436	5900	19.760	8.370	28.130	8.770	14.770	114.360
120	alpha	1	60x8 weeks	Ag80	Daily	VAC70	75	Invaccinate	1.229	0.004	0.002	0.006	0.002	0.003	0.024	590.044	1.976	0.837	2.813	0.877	1.477	11.436	5900	19.760	8.370	28.130	8.770	14.770	114.360
120	alpha	1	60x8 weeks	Ag80	Daily	VAC70	75	Invaccinate	1.229	0.004	0.002	0.006	0.002	0.003	0.024	590.044	1.976	0.837	2.813	0.877	1.477	11.436	5900	19.760	8.370	28.130	8.770	14.770	114.360
120	alpha	1.2	60x8 weeks	Ag80	Daily	VAC70	75	Invaccinate	1.229	0.004	0.002	0.006	0.002	0.003	0.024	589.738	1.938	1.038	2.976	0.930	1.514	11.527	5897	19.380	10.380	29.760	9.300	15.140	115.270
120	alpha	1.2	60x8 weeks	Ag80	Daily	VAC70	75	Invaccinate	1.229	0.004	0.002	0.006	0.002	0.003	0.024	589.738	1.938	1.038	2.976	0.930	1.514	11.527	5897	19.380	10.380	29.760	9.300	15.140	115.270
120	alpha	1.2	60x8 weeks	Ag80	Daily	VAC70	75	Invaccinate	1.229	0.004	0.002	0.006	0.002	0.003	0.024	589.738	1.938	1.038	2.976	0.930	1.514	11.527	5897	19.380	10.380	29.760	9.300	15.140	115.270
120	delta	0.6	60x8 weeks	Ag80	Daily	VAC70	75	Invaccinate	1.225	0.005	0.004	0.008	0.003	0.004	0.023	588.051	2.211	1.714	3.925	1.425	1.762	11.034	5881	22.110	17.140	39.250	14.250	17.620	110.340
120	delta	0.6	60x8 weeks	Ag80	Daily	VAC70	75	Invaccinate	1.225	0.005	0.004	0.008	0.003	0.004	0.023	588.051	2.211	1.714	3.925	1.425	1.762	11.034	5881	22.110	17.140	39.250	14.250	17.620	110.340
120	delta	0.6	60x8 weeks	Ag80	Daily	VAC70	75	Invaccinate	1.225	0.005	0.004	0.008	0.003	0.004	0.023	588.051	2.211	1.714	3.925	1.425	1.762	11.034	5881	22.110	17.140	39.250	14.250	17.620	110.340
120	delta	1	60x8 weeks	Ag80	Daily	VAC70	75	Invaccinate	1.207	0.005	0.010	0.015	0.006	0.006	0.023	579.291	2.162	4.935	7.097	2.702	2.996	11.054	5793	21.620	49.350	70.970	27.020	29.960	110.540
120	delta	1	60x8 weeks	Ag80	Daily	VAC70	75	Invaccinate	1.207	0.005	0.010	0.015	0.006	0.006	0.023	579.291	2.162	4.935	7.097	2.702	2.996	11.054	5793	21.620	49.350	70.970	27.020	29.960	110.540
120	delta	1	60x8 weeks	Ag80	Daily	VAC70	75	Invaccinate	1.207	0.005	0.010	0.015	0.006	0.006	0.023	579.291	2.162	4.935	7.097	2.702	2.996	11.054	5793	21.620	49.350	70.970	27.020	29.960	110.540
120	delta	1.2	60x8 weeks	Ag80	Daily	VAC70	75	Invaccinate	1.192	0.004	0.016	0.020	0.008	0.008	0.022	572.233	2.065	7.687	9.752	3.743	4.069	10.406	5722	20.650	76.870	97.520	37.430	40.690	104.060

120	delta	1.2	60x8 weeks	Ag80	Daily	VAC70	75	Unvaccinate	1.192	0.004	0.016	0.020	0.008	0.008	0.022	572.233	2.065	7.687	9.752	3.743	4.069	10.406	5722	20.650	76.870	97.520	37.430	40.690	104.060
120	delta	1.2	60x8 weeks	Ag80	Daily	VAC70	75	Unvaccinate	1.192	0.004	0.016	0.020	0.008	0.008	0.022	572.233	2.065	7.687	9.752	3.743	4.069	10.406	5722	20.650	76.870	97.520	37.430	40.690	104.060
120	original	0.6	60x8 weeks	Ag80	Daily	VAC70	100	Unvaccinate	0.000	0.003	0.000	0.003	0.001	0.000	0.000	0.000	1.253	0.012	1.265	0.624	0.000	0.000	0	12.530	0.120	12.650	6.240	0.000	0.000
120	original	0.6	60x8 weeks	Ag80	Daily	VAC70	100	Unvaccinate	0.000	0.003	0.000	0.003	0.001	0.000	0.000	0.000	1.253	0.012	1.265	0.624	0.000	0.000	0	12.530	0.120	12.650	6.240	0.000	0.000
120	original	0.6	60x8 weeks	Ag80	Daily	VAC70	100	Unvaccinate	0.000	0.003	0.000	0.003	0.001	0.000	0.000	0.000	1.253	0.012	1.265	0.624	0.000	0.000	0	12.530	0.120	12.650	6.240	0.000	0.000
120	original	1	60x8 weeks	Ag80	Daily	VAC70	100	Unvaccinate	0.000	0.003	0.000	0.003	0.001	0.000	0.000	0.000	1.340	0.019	1.359	0.686	0.000	0.000	0	13.400	0.190	13.590	6.860	0.000	0.000
120	original	1	60x8 weeks	Ag80	Daily	VAC70	100	Unvaccinate	0.000	0.003	0.000	0.003	0.001	0.000	0.000	0.000	1.340	0.019	1.359	0.686	0.000	0.000	0	13.400	0.190	13.590	6.860	0.000	0.000
120	original	1	60x8 weeks	Ag80	Daily	VAC70	100	Unvaccinate	0.000	0.003	0.000	0.003	0.001	0.000	0.000	0.000	1.340	0.019	1.359	0.686	0.000	0.000	0	13.400	0.190	13.590	6.860	0.000	0.000
120	original	1.2	60x8 weeks	Ag80	Daily	VAC70	100	Unvaccinate	0.000	0.003	0.000	0.003	0.001	0.000	0.000	0.000	1.357	0.025	1.382	0.701	0.000	0.000	0	13.570	0.250	13.820	7.010	0.000	0.000
120	original	1.2	60x8 weeks	Ag80	Daily	VAC70	100	Unvaccinate	0.000	0.003	0.000	0.003	0.001	0.000	0.000	0.000	1.357	0.025	1.382	0.701	0.000	0.000	0	13.570	0.250	13.820	7.010	0.000	0.000
120	original	1.2	60x8 weeks	Ag80	Daily	VAC70	100	Unvaccinate	0.000	0.003	0.000	0.003	0.001	0.000	0.000	0.000	1.357	0.025	1.382	0.701	0.000	0.000	0	13.570	0.250	13.820	7.010	0.000	0.000
120	alpha	0.6	60x8 weeks	Ag80	Daily	VAC70	100	Unvaccinate	0.000	0.003	0.000	0.003	0.002	0.000	0.000	0.000	1.244	0.226	1.470	0.881	0.000	0.000	0	12.440	2.260	14.700	8.810	0.000	0.000
120	alpha	0.6	60x8 weeks	Ag80	Daily	VAC70	100	Unvaccinate	0.000	0.003	0.000	0.003	0.002	0.000	0.000	0.000	1.244	0.226	1.470	0.881	0.000	0.000	0	12.440	2.260	14.700	8.810	0.000	0.000
120	alpha	0.6	60x8 weeks	Ag80	Daily	VAC70	100	Unvaccinate	0.000	0.003	0.000	0.003	0.002	0.000	0.000	0.000	1.244	0.226	1.470	0.881	0.000	0.000	0	12.440	2.260	14.700	8.810	0.000	0.000
120	alpha	1	60x8 weeks	Ag80	Daily	VAC70	100	Unvaccinate	0.000	0.003	0.001	0.004	0.002	0.000	0.000	0.000	1.327	0.515	1.842	1.091	0.000	0.000	0	13.270	5.150	18.420	10.910	0.000	0.000
120	alpha	1	60x8 weeks	Ag80	Daily	VAC70	100	Unvaccinate	0.000	0.003	0.001	0.004	0.002	0.000	0.000	0.000	1.327	0.515	1.842	1.091	0.000	0.000	0	13.270	5.150	18.420	10.910	0.000	0.000
120	alpha	1	60x8 weeks	Ag80	Daily	VAC70	100	Unvaccinate	0.000	0.003	0.001	0.004	0.002	0.000	0.000	0.000	1.327	0.515	1.842	1.091	0.000	0.000	0	13.270	5.150	18.420	10.910	0.000	0.000
120	alpha	1.2	60x8 weeks	Ag80	Daily	VAC70	100	Unvaccinate	0.000	0.003	0.001	0.004	0.002	0.000	0.000	0.000	1.253	0.575	1.828	1.096	0.000	0.000	0	12.530	5.750	18.280	10.960	0.000	0.000
120	alpha	1.2	60x8 weeks	Ag80	Daily	VAC70	100	Unvaccinate	0.000	0.003	0.001	0.004	0.002	0.000	0.000	0.000	1.253	0.575	1.828	1.096	0.000	0.000	0	12.530	5.750	18.280	10.960	0.000	0.000
120	alpha	1.2	60x8 weeks	Ag80	Daily	VAC70	100	Unvaccinate	0.000	0.003	0.001	0.004	0.002	0.000	0.000	0.000	1.253	0.575	1.828	1.096	0.000	0.000	0	12.530	5.750	18.280	10.960	0.000	0.000
120	delta	0.6	60x8 weeks	Ag80	Daily	VAC70	100	Unvaccinate	0.000	0.003	0.003	0.006	0.004	0.000	0.000	0.000	1.672	1.307	2.979	1.805	0.000	0.000	0	16.720	13.070	29.790	18.050	0.000	0.000
120	delta	0.6	60x8 weeks	Ag80	Daily	VAC70	100	Unvaccinate	0.000	0.003	0.003	0.006	0.004	0.000	0.000	0.000	1.672	1.307	2.979	1.805	0.000	0.000	0	16.720	13.070	29.790	18.050	0.000	0.000
120	delta	0.6	60x8 weeks	Ag80	Daily	VAC70	100	Unvaccinate	0.000	0.003	0.003	0.006	0.004	0.000	0.000	0.000	1.672	1.307	2.979	1.805	0.000	0.000	0	16.720	13.070	29.790	18.050	0.000	0.000
120	delta	1	60x8 weeks	Ag80	Daily	VAC70	100	Unvaccinate	0.000	0.003	0.008	0.011	0.007	0.000	0.000	0.000	1.631	3.876	5.507	3.357	0.000	0.000	0	16.310	38.760	55.070	33.570	0.000	0.000
120	delta	1	60x8 weeks	Ag80	Daily	VAC70	100	Unvaccinate	0.000	0.003	0.008	0.011	0.007	0.000	0.000	0.000	1.631	3.876	5.507	3.357	0.000	0.000	0	16.310	38.760	55.070	33.570	0.000	0.000
120	delta	1	60x8 weeks	Ag80	Daily	VAC70	100	Unvaccinate	0.000	0.003	0.008	0.011	0.007	0.000	0.000	0.000	1.631	3.876	5.507	3.357	0.000	0.000	0	16.310	38.760	55.070	33.570	0.000	0.000
120	delta	1.2	60x8 weeks	Ag80	Daily	VAC70	100	Unvaccinate	0.000	0.003	0.014	0.018	0.011	0.000	0.000	0.000	1.595	6.929	8.524	5.231	0.000	0.000	0	15.950	69.290	85.240	52.310	0.000	0.000
120	delta	1.2	60x8 weeks	Ag80	Daily	VAC70	100	Unvaccinate	0.000	0.003	0.014	0.018	0.011	0.000	0.000	0.000	1.595	6.929	8.524	5.231	0.000	0.000	0	15.950	69.290	85.240	52.310	0.000	0.000
120	delta	1.2	60x8 weeks	Ag80	Daily	VAC70	100	Unvaccinate	0.000	0.003	0.014	0.018	0.011	0.000	0.000	0.000	1.595	6.929	8.524	5.231	0.000	0.000	0	15.950	69.290	85.240	52.310	0.000	0.000
120	original	0.6	60x8 weeks	Ag80	Daily	VAC50	25	Unvaccinate	3.704	0.007	0.001	0.008	0.001	0.007	0.072	1777.810	3.453	0.379	3.832	0.408	3.273	34.754	17778	34.530	3.790	38.320	4.080	32.730	347.540
120	original	0.6	60x8 weeks	Ag80	Daily	VAC50	25	Unvaccinate	3.704	0.007	0.001	0.008	0.001	0.007	0.072	1777.810	3.453	0.379	3.832	0.408	3.273	34.754	17778	34.530	3.790	38.320	4.080	32.730	347.540
120	original	0.6	60x8 weeks	Ag80	Daily	VAC50	25	Unvaccinate	3.704	0.007	0.001	0.008	0.001	0.007	0.072	1777.810	3.453	0.379	3.832	0.408	3.273	34.754	17778	34.530	3.790	38.320	4.080	32.730	347.540
120	original	1	60x8 weeks	Ag80	Daily	VAC50	25	Unvaccinate	3.702	0.007	0.001	0.008	0.001	0.007	0.072	1776.873	3.402	0.607	4.009	0.384	3.431	34.555	17769	34.020	6.070	40.090	3.840	34.310	345.550
120	original	1	60x8 weeks	Ag80	Daily	VAC50	25	Unvaccinate	3.702	0.007	0.001	0.008	0.001	0.007	0.072	1776.873	3.402	0.607	4.009	0.384	3.431	34.555	17769	34.020	6.070	40.090	3.840	34.310	345.550
120	original	1	60x8 weeks	Ag80	Daily	VAC50	25	Unvaccinate	3.702	0.007	0.001	0.008	0.001	0.007	0.072	1776.873	3.402	0.607	4.009	0.384	3.431	34.555	17769	34.020	6.070	40.090	3.840	34.310	345.550
120	original	1.2	60x8 weeks	Ag80	Daily	VAC50	25	Unvaccinate	3.698	0.007	0.002	0.009	0.001	0.008	0.072	1775.214	3.446	0.835	4.281	0.395	3.665	34.545	17752	34.460	8.350	42.810	3.950	36.650	345.450
120	original	1.2	60x8 weeks	Ag80	Daily	VAC50	25	Unvaccinate	3.698	0.007	0.002	0.009	0.001	0.008	0.072	1775.214	3.446	0.835	4.281	0.395	3.665	34.545	17752	34.460	8.350	42.810	3.950	36.650	345.450
120	original	1.2	60x8 weeks	Ag80	Daily	VAC50	25	Unvaccinate	3.698	0.007	0.002	0.009	0.001	0.008	0.072	1775.214	3.446	0.835	4.281	0.395	3.665	34.545	17752	34.460	8.350	42.810	3.950	36.650	345.450
120	alpha	0.6	60x8 weeks	Ag80	Daily	VAC50	25	Unvaccinate	3.696	0.007	0.002	0.009	0.001	0.008	0.072	1774.022	3.364	0.974	4.338	0.494	3.812	34.651	17740	33.640	9.740	43.380	4.940	38.120	346.510
120	alpha	0.6	60x8 weeks	Ag80	Daily	VAC50	25	Unvaccinate	3.696	0.007	0.002	0.009	0.001	0.008	0.072	1774.022	3.364	0.974	4.338	0.494	3.812	34.651	17740	33.640	9.740	43.380	4.940	38.120	346.510
120	alpha	0.6	60x8 weeks	Ag80	Daily	VAC50	25	Unvaccinate	3.696	0.007	0.002	0.009	0.001	0.008	0.072	1774.022	3.364	0.974	4.338	0.494	3.812	34.651	17740	33.640	9.740	43.380	4.940	38.120	346.510
120	alpha	1	60x8 weeks	Ag80	Daily	VAC50	25	Unvaccinate	3.686	0.007	0.004	0.011	0.001	0.010	0.072	1769.257	3.392	1.764	5.156	0.550	4.569	34.624	17693	33.920	17.640	51.560	5.500	45.690	346.240
120	alpha	1	60x8 weeks	Ag80	Daily	VAC50	25	Unvaccinate	3.686	0.007	0.004	0.011	0.001	0.010	0.072	1769.257	3.392	1.764	5.156	0.550	4.569	34.624	17693	33.920	17.640	51.560	5.500	45.690	346.240
120	alpha	1	60x8 weeks	Ag80	Daily	VAC50	25	Unvaccinate	3.686	0.007	0.004	0.011	0.001	0.010	0.072	1769.257	3.392	1.764	5.156	0.550	4.569	34.624	17693	33.920	17.640	51.560	5.500	45.690	346.240
120	alpha	1.2	60x8 weeks	Ag80	Daily	VAC50	25	Unvaccinate	3.679	0.007	0.005	0.012	0.001	0.010	0.071	1765.768	3.367	2.353	5.720	0.641	5.026	33.840	17658	33.670	23.530	57.200	6.410	50.260	338.400
120	alpha	1.2	60x8 weeks	Ag80	Daily	VAC50	25	Unvaccinate	3.679	0.007	0.005	0.012	0.001	0.010	0.071	1765.768	3.367	2.353	5.720	0.641	5.026	33.840	17658	33.670	23.530	57.200	6.410	50.260	338.400
120	alpha	1.2	60x8 weeks	Ag80	Daily	VAC50	25	Unvaccinate	3.679	0.007	0.005	0.012	0.001	0.010	0.071	1765.768	3.367	2.353	5.720	0.641	5.026	33.840	17658	33.670	23.530	57.200	6.410	50.260	338.400
120	delta	0.6	60x8 weeks	Ag80	Daily	VAC50	25	Unvaccinate	3.675	0.007	0.006	0.013	0.002	0.011	0.071	1763.947	3.458	2.807	6.265	0.912	5.261	34.049	17639	34.580	28.070	62.650	9.120	52.610	340.490
120	delta	0.6	60x8 weeks	Ag80	Daily	VAC50	25	Unvaccinate	3.675	0.007	0.006	0.013	0.002	0.011	0.071	1763.947	3.458	2.807	6.265	0.912	5.261	34.049	17639	34.580	28.070	62.650	9.120	52.610	340.490
120	delta	0.6	60x8 weeks	Ag80	Daily	VAC50	25	Unvaccinate	3.675	0.007	0.006	0.013	0.002	0.011	0.071	1763.947	3.458	2.807	6.265	0.912	5.261	34.049	17639	34.580	28.070	62.650	9.120	52.610	340.490
120	delta	1	60x8 weeks	Ag80	Daily	VAC50	25	Unvaccinate	3.612	0.007	0.017	0.024	0.004	0.020	0.068	1733.646	3.408	8.112	11.520	1.686	9.610	32.681	17336	34.080	81.120	115.200	16.860	96.100	326.810
120	delta	1	60x8 weeks	Ag80	Daily	VAC50	25	Unvaccinate	3.612	0.007	0.017	0.024	0.004	0.020	0.068	1733.646	3.408	8.112	11.520	1.686	9.610	32.681	17336	34.080	81.120	115.200	16.860	96.100	326.810
120	delta	1	60x8 weeks	Ag80	Daily	VAC50	25	Unvaccinate	3.612	0.007	0.017	0.024	0.004	0.020	0.068	1733.646	3.408	8.112	11.520	1.686	9.610	32.681	17336	34.080	81.120	115.200	16.860	96.100	326.810
120	delta	1.2	60x8 weeks	Ag80	Daily	VAC50	25	Unvaccinate	3.550	0.007	0.028	0.035	0.005	0.029	0.065	1704.125	3.281	13.349	16.630	2.491	13.859	30.967	17041	32.810	133.490	166.300	24.910	138.590	309.670
120	delta	1.2	60x8 weeks	Ag80	Daily	VAC50	25	Unvaccinate	3.550	0.007	0.028	0.035	0.005	0.029	0.065	1704.125	3.281	13.349	16.630	2.491	13.859	30.967	17041	32.810	133.490	166.300	24.910	138.590	309.670
120	delta	1.2	60x8 weeks	Ag80	Daily	VAC50	25	Unvaccinate	3.550	0.007	0.028	0.035	0.005	0.029	0.065	1704.125	3.281	13.349	16.630	2.491	13.859	30.967	17041	32.810	133.490	166.300	24.910	138.590	309.670
120	original	0.6	60x8 weeks	Ag80	Daily	VAC50	50	Unvaccinate	2.471	0.006	0.000	0.007	0.001	0.004	0.048	1185.871	2.977	0.231	3.208	0.613	2.086	23.092	11859	29.770	2.310	32.080	6.130	20.860	230.920
120	original	0.6	60x8 weeks	Ag80	Daily	VAC50	50	Unvaccinate	2.471	0.006	0.000	0.007	0.001	0.004	0.048	1185.871	2.977	0.231	3.208	0.613	2.086	23.092	11859	29.770	2.310	32.080	6.130	20.860	230.920
120	original	0.6	60x8 weeks	Ag80	Daily	VAC50	50	Unvaccinate	2.471	0.006	0.000	0.007	0.001	0.004	0.048	1185.871	2.977	0.231	3.208	0.613	2.086	23.092	11859	29.770	2.310	32.080	6.130	20.860	230.920
120	original	1	60x8 weeks	Ag80	Daily	VAC50	50	Unvaccinate	2.468	0.006	0.001	0.007	0.001	0.005	0.048	1184.768	2.967	0.370	3.337	0.629	2.233	23.077	11848	29.670	3.700	33.370	6.290	22.330	230.770
120	original	1	60x8 weeks	Ag80	Daily	VAC50	50	Unvaccinate	2.468	0.006	0.001	0.007	0.001	0.005	0.048	1184.768	2.967	0.370	3.337	0.629	2.233	23.077	11848	29.670	3.700	33.370	6.290	22.330	230.770
120	original	1	60x8 weeks	Ag80	Daily	VAC50	50	Unvaccinate	2.468	0.006	0.001	0.007	0.001	0.005	0.048	1184.768	2.967	0.370	3.337	0.629	2.233	23.077	11848	29.670	3.700	33.370	6.290	22.330	230.770
120	original	1.2	60x8 weeks	Ag80	Daily	VAC50	50	Unvaccinate	2.469	0.006	0.001	0.007	0.001	0.005	0.048	1185.264	2.852	0.425	3.277	0.644	2.173	23.244	11853	28.520	4.250	32.770	6.440	21.730	232.440
120	original	1.2	60x8 weeks	Ag80	Daily	VAC50	50	Unvaccinate	2.469	0.006	0.001	0.007	0.001	0.005	0.048	1185.264	2.852	0.425	3.277	0.644	2.173	23.244	11853	28.520	4.250	32.770	6.440	21.730	232.440
120	original	1.2	60x8 weeks	Ag80	Daily	VAC50	50	Unvaccinate	2.469	0.006	0.001	0.007	0.001	0.005	0.048	1185.264	2.852	0.425	3.277	0.644	2.173	23.244	11853	28.520	4.250	32.770	6.440	21.730	232.440
120	alpha	0.6	60x8 weeks	Ag80	Daily	VAC50	50	Unvaccinate	2.465	0.006	0.002	0.008	0.002	0.005	0.048	1183.129	2.899	0.813	3.712	0.870	2.502	22.838	11831	28.990	8.130	37.120	8.700	25.020	228.380
120	alpha	0.6	60x8 weeks	Ag80	Daily	VAC50	50	Unvaccinate	2.465	0.006	0.002	0.008	0.002	0.005	0.048	1183.129	2.899	0.813	3.712	0.870	2.502	22.838	11831	28.990	8.130	37.120	8.700	25.020	228.380
120	alpha	0.6	60x8 weeks	Ag80	Daily	VAC50	50	Unvaccinate	2.465	0.006	0.002	0.008	0.002	0.005	0.048	1183.129	2.899	0.813	3.712	0.870	2.502	22.838	11831	28.990	8.130	37.120	8.700	25.020	228.380
120	alpha	1	60x8 weeks	Ag80	Daily	VAC50	50	Unvaccinate	2.457	0.006	0.003	0.009	0.002	0.006	0.047	1179.310	2.871	1.673	4.544	1.030	3.051	22.703	11793	28.710	16.730	45.440	10.300	30.510	227.030
120	alpha	1	60x8 weeks	Ag80	Daily	VAC50	50	Unvaccinate	2.457	0.006	0.003	0.009	0.002	0.006	0.047	1179.310	2.871	1.673	4.544	1.030	3.051	22.703	11793	28.710	16.730	45.440	10.300	30.510	227.030
[truncated: 1,530,920 more chars]
